# An Intronic Signal for Alternative Splicing in the Human Genome

**DOI:** 10.1371/journal.pone.0001246

**Published:** 2007-11-28

**Authors:** Necat Havlioglu, Jun Wang, Kazuo Fushimi, Maria D. Vibranovski, Zhengyan Kan, Warren Gish, Alexei Fedorov, Manyuan Long, Jane Y. Wu

**Affiliations:** 1 Department of Pathology, Saint Louis University, St. Louis, Missouri, United States of America; 2 Department of Ecology and Evolution, The University of Chicago, Chicago, Illinois, United States of America; 3 Department of Neurology, Lurie Comprehensive Cancer Center, Center for Genetic Medicine, Northwestern University Feinberg School of Medicine, Chicago, Illinois, United States of America; 4 Department of Genetics, Washington University in St. Louis, St. Louis, Missouri, United States of America; 5 Department of Medicine and Program in Bioinformatics and Proteomics/Genomics, Medical University of Ohio, Toledo, Ohio, United States of America; Lehigh University, United States of America

## Abstract

An important level at which the expression of programmed cell death (PCD) genes is regulated is alternative splicing. Our previous work identified an intronic splicing regulatory element in caspase-2 (casp-2) gene. This 100-nucleotide intronic element, In100, consists of an upstream region containing a decoy 3′ splice site and a downstream region containing binding sites for splicing repressor PTB. Based on the signal of In100 element in casp-2, we have detected the In100-like sequences as a family of sequence elements associated with alternative splicing in the human genome by using computational and experimental approaches. A survey of human genome reveals the presence of more than four thousand In100-like elements in 2757 genes. These In100-like elements tend to locate more frequent in intronic regions than exonic regions. EST analyses indicate that the presence of In100-like elements correlates with the skipping of their immediate upstream exons, with 526 genes showing exon skipping in such a manner. In addition, In100-like elements are found in several human caspase genes near exons encoding the caspase active domain. RT-PCR experiments show that these caspase genes indeed undergo alternative splicing in a pattern predicted to affect their functional activity. Together, these results suggest that the In100-like elements represent a family of intronic signals for alternative splicing in the human genome.

## Introduction

As a major mechanism of genetic diversity by generating transcript variability, alternative splicing affects every aspect of cell survival and function. It is estimated that more than 60% of human genes undergo alternative splicing [1]. A variety of splicing regulatory elements have been identified by using molecular, biochemical, and computational approaches. However, our understanding of alternative splicing at the genomic level remains limited.

A large number of genes critical for cell death utilize alternative splicing to generate functionally distinct or even antagonistic isoforms [2], [3]. These genes range from death signals, death receptors, intracellular adaptor molecules to caspases [4], [5]. To understand mechanisms regulating alternative splicing events important for cell death gene function, we previously established a model system using the murine caspase-2 (casp-2) gene. Exclusion or inclusion of exon 9 leads to the formation of two functionally antagonistic isoforms, casp-2S or casp-2L respectively [6], [7]. Casp-2L and casp-2S have antagonistic activities in cell death [6], [8]. Dissection of cis-elements reveals an intronic element, In100, which is important for regulating casp-2 alternative splicing. In100 has two domains, an upstream one containing a decoy 3′ splice site and a downstream one with U/C-rich repeats that interact with PTB [9], [10].

In this study, we searched human genome for sequence elements with features of In100 and identified 2757 genes containing In100-like elements. These In100-containing genes are subdivided in 1865 gene families. Gene ontology analysis revealed that the products of these In100-containing genes are involved in various biology processes. These elements are significantly enriched in intronic regions. EST database analyses revealed a correlation of such elements with the skipping of their upstream exons. In addition, by RT-PCR, analyses of several human caspase genes indicate that these In100-containing caspase genes indeed undergo alternative splicing, with the skipping of exons immediate upstream of their In100-like elements. Taken together, these results support that In100-like elements represent a family of sequence signals for alternative splicing.

## Results

### A conserved intronic regulatory element, In100, in the caspase-2 gene

**Figure 1:**
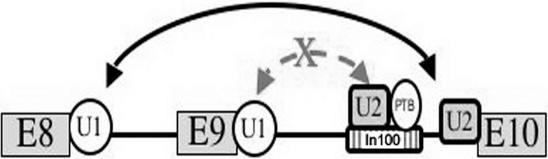
A diagram depicting In100 and its role in regulating casp-2. As demonstrated by our previous studies [9], [10], sequential deletion and biochemical experiments reveal that In100 in casp-2 gene represses exon 9 inclusion by engaging non-productive interaction with the 5′ splice site of exon 9. Interactions between U1 snRNP associated with exon 9 at the 5′ splice site and complexes associated with In100, including U2 snRNP and PTB, block rather than promote the selection of this 5′ splice site. The In100 element is diagramed as the hatched box. Exons 8, 9 and 10 are depicted as E8, E9 and E10 in gray boxes. U1 snRNP and U2 snRNPs are illustrated as U1 and U2.

In our previous studies, we established a casp-2 splicing model system and identified In100 as an important cis-element for casp-2 alternative splicing [9], [10]. This In100 element contains two domains, both of which are required for its activity in suppressing the use of the 5′ splice site of exon 9. The upstream domain contains a decoy 3′ splice site, highly similar to an authentic 3′ splice site, and a downstream domain with U/C-rich repeats interacting with polypyrimidine tract binding protein, PTB [9], [10]. Our biochemical experiments indicate that In100 engages in non-productive interactions with the 5′SS of exon 9, hence blocking its usage by the spliceosome (Figure 1).

**Figure 2:**
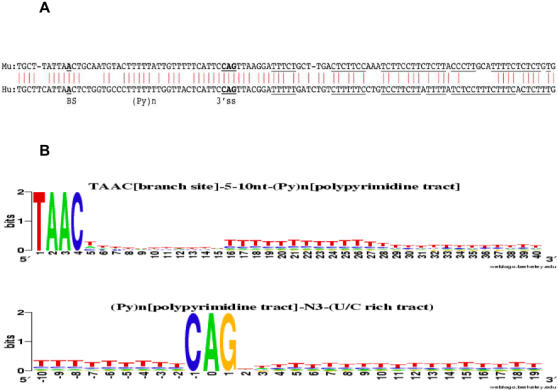
The evolutionary conservation of casp-2 In100 element and nucleotide composition of In100-like elements. (A) Alignment between human and mouse casp-2 In100 elements shows 74% sequence identity. Putative branch site A is in bold and underlined (BS). The polypyrimidine tract in the decoy 3′splice site (Py)n and CAG are indicated. (B) The nucleotide composition is displayed based on the identified 4342 In100 elements.

Comparative analyses of mouse and human casp-2 genomic sequences reveal that In100 element is conserved, with 74% nucleotide identity between mouse and human sequences (Figure 2A), although the overall nucleotide sequence of intron 9 is not conserved. This suggests a common mammalian ancestral In100-like element and the functional importance of In100-like elements in mammalian species. These observations raise a conjecture that the In100-like elements may represent a sequence motif regulating alternative splicing of many different genes. Testing this conjecture may help address several questions. First, are these In100-like elements wide spread in the human genome? Second, how are they distributed in the genome, e.g. among intronic and exonic regions? Third, is the presence of such elements associated with alternative splicing events in the human genome? To answer these questions, we designed the following bioinformatics and experimental approaches to search for and analyze In100-like elements in the human genome.

### In100-like sequence elements are widespread in the human genome

To test the hypothesis that a 3′ splice site-like sequence juxtaposed to U/C rich sequence may represent a general motif associated with alternative splicing, we screened the human genome for genes containing such sequences. First, an exon-intron sequence database was created containing all annotated human genes. An algorithm was developed to search for In100-like elements in this database. This algorithm used a 100-nucleotide sliding window to scan for the In100 signals from the 5′ to the 3′ end along each DNA sequence in the database. In100 signal is defined as: UAAC[branch site]-5∼10nt-(Py)n[polypyrimidine tract]-N_3_-(U/C rich, 20–60 nt), where n is bounded around 30∼40, “U/C rich” is defined as U/C content higher than 70%, and N_3_ = CAG. We also tested N_3_ = TAG, AAG and GAG (see below) and investigated the relative importance of these trinucleotides in defining 3′SS [11]. This search revealed 4342 candidate sequences with the features of In100 in 2757 genes (Table S1), suggesting that In100-like elements may represent a general sequence motif. The nucleotide composition of these In100-like elements was analyzed using PERL scripts and Web-Logo [12], as displayed in Figure 2B.

**Figure 3:**
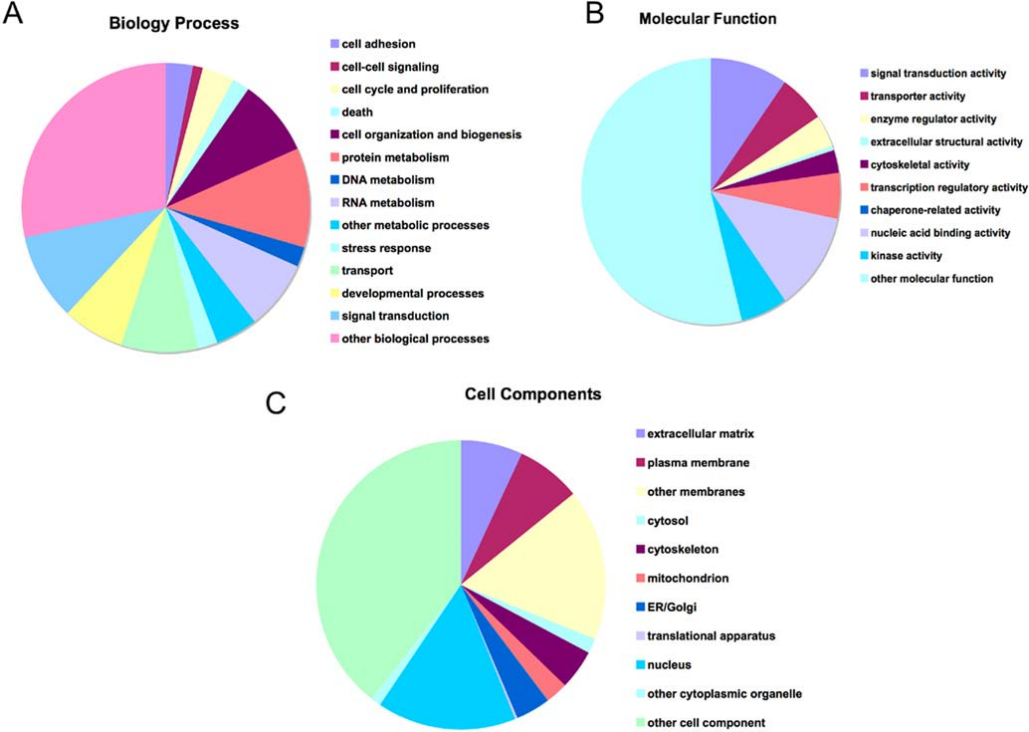
The distribution of gene ontology of In100-containing genes. The distribution of gene ontology terms of the In100-containing genes in (A) Biology Process, (B) Molecular Function and (C) Cell Component.

Gene family analysis showed that these In100-containing genes can be classified into 1865 gene families (Table S2). Gene ontology analysis showed that these genes are involved in various biological processes, molecular function and encoding different cell components (Figure 3). Among overrepresented In100-containing genes are those involved in biological processes of biopolymer modification, protein modification process, post-translation protein modification, with molecular functions of protein kinase activity, ATP binding and phosphotransferase activity and encoding cell components of basement membrane, cell membrane and synapses (Table S3).

### The significant enrichment of In100-like elements in intronic regions

To address whether these In100-like sequences are randomly distributed in intronic and exonic regions, we designed two tests. First, we compared the observed and expected frequencies of the candidate In100 elements in intronic and exonic sequences based on their total length distribution. The lengths of introns and exons for In100-containing genes were calculated, and frequencies of candidate In100 sequences were compared. If these In100-like elements were equally distributed, we would expect their frequencies in the intronic versus exonic regions to be 94.2% and 5.8% respectively, proportional to the relative lengths of introns and exons in the human exon-intron sequence database constructed (see Methods). As shown in Table 1, there is a positive bias towards intronic distribution (98.5%), and a negative bias towards exonic distribution (1.5%) (χ^2^ = 189.2, degree of freedom (df) = 1, P≈0). Thus, these In100-like elements are not randomly distributed and instead, significantly enriched in intronic regions.

**Table 1:** Intronic versus exonic distribution of In100-like elements identified in human genome

	Exons	Introns	Sum
Observed No.	65(1.5%)	4277 (98.5%)	4342 (100%)
Expected No.	251(5.8%)	4091 (94.2%)	4342(100%)
(O-E)^2^/E	137.8	8.5	146.3
Total lengths	65,661,947	1,068,751,168	1,134,413,115

χ^2^ = 146.3 for df = 1, P≅0, revealing a highly significant excess of In100 element candidates in intronic regions.

Additionally, we examined the distribution pattern of In100-elements in randomized human intron-exon databases. By simulation, we randomized the nucleotide sequences of human intron-exon database but maintaining the same exon-intron structure and nucleotide compositions (see Methods). We found that the frequency of In100-elements decreases by almost an order of magnitude in the randomized databases (Figure S3), as compared to the In100-element frequency observed in the real human intron-exon database. Thus, the significant decrease of In100-elements in the randomized human database indicates that there is a significant enrichment of In100-element in the human genome and that the detected genomic distribution of In100 in the human genome is not a statistical coincidence.

Next, we analyzed the trinucleotide sequences inside the decoy 3′ splice site of the In100 elements (i.e., in “N_3_” position). Genomic analyses have shown that in intronic regions, CAG are present more frequently than expected at the last three nucleotide positions as 3′ splicing signal [11], [13]. Thus, if the detected In100-like sequences are intronic elements; in intronic regions, the frequency of the defined elements with CAG-(U/C rich, 20-60nt) should be higher than its expected frequency, whereas the elements with TAG, GAG, or AAG should be observed in depletion, or at least not in excess. In addition, the excess of In100-elements with CAG should be exclusively observed in the intronic regions, not in exonic regions. We analyzed and compared the number of In100-like elements with CAG, AAG, GAG, and TAG as N_3_ in intronic and exonic regions respectively. Respectively, we identified 4277, 4643, 3814 and 5126 In100-like sequences in the intronic regions, and 65, 95, 57 and 80 in the exonic regions. As shown in Table 2, the frequency of CAG containing In100 elements are significantly higher in the intronic regions and lower than expected in the exonic regions, further supporting that the In100 elements are enriched in intronic regions.

**Table 2:** Frequency of defined trinucleotides of decoy 3′ splice sites in candidate In100 elements (numbers observed and expected for introns/exons)

	CAG		AAG		GAG		TAG		Total	
	Intronic	Exonic	Intronic	Exonic	Intronic	Exonic	Intronic	Exonic	Intronic	Exonic
**Obs No.**	4277	65	4643	95	3814	57	5126	80	17860	297
**Exp No.**	3572	73	5001	77	3750	74	5537	73	17860	297
**Excess (O-E/E)×100**	19.7	−11	−7.2	23	1.7	−23	−7.4	10		
**(O-E)^2^/E**	139.1	0.877	25.6	4.21	1.1	3.9	30.5	0.67	196.3	9.7

χ^2^ = 196.3 for df = 3 indicates a probability that is very low (P = 4.2×10^−42^), thus there is a significant excess (∼20%) of the In100 element candidate as defined by CAG at the last three positions of the decoy 3′ splice site inside the In100-like elements located in introns. The opposite pattern is observed for CAG inside the In100-like elements located in exons: depletion of 11% (X^2^ = 9.7 for df = 3; P = 0.021). The expected numbers were calculated separately by the nucleotide composition of intronic regions: 0.28 for A, 0.31 for T, 0.20 for C and 0.21 for G; and of exonic regions: 0.258 for A, 0.246 for T, 0.246 for C and 0.25 for G [43].

### The presence of In100 element correlates with skipping of its upstream exons

**Figure 4:**
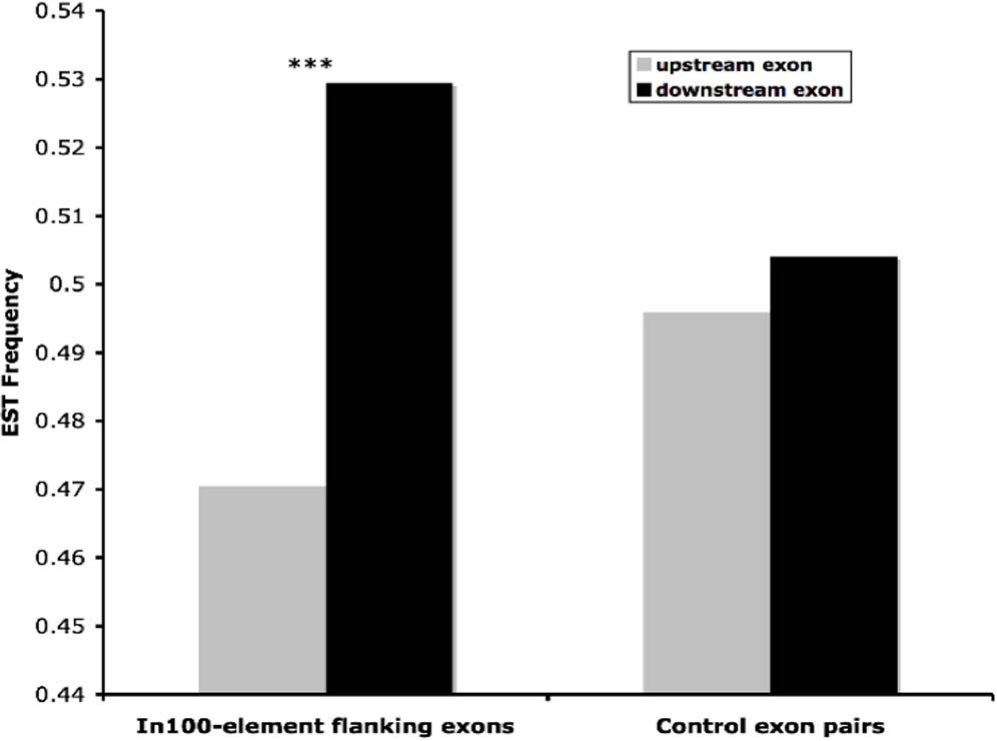
A biased distribution of transcripts favoring the downstream exons as revealed by EST analyses. ESTs frequency of upstream (5′) and downstream (3′) exons. The sample sizes for analyzed In100-element flanking exons and control exon pairs are: 2728 and 23624, respectively.

If In100 elements play a role in suppressing the inclusion of the immediate upstream exons, similar to that in caspase-2 gene (Figure 1; see the previous studies [9], [10]), it would be predicted that In100 immediate downstream exons would be more likely to appear in the human transcriptome than the immediate upstream exons. This prediction was tested by examining the relative abundance of the transcripts containing the upstream or downstream exons of In100-element in the human EST database. We isolated *in silico* the immediate upstream and downstream exons for 4277 In100-elements in the intronic regions and searched human EST databases for the transcripts that contain exons flanking In100-elements. We used equal or higher than 98% identity and 70% exon length coverage as alignment criteria to determine the match of an EST with a particular exon sequence. If an exon-EST alignment satisfied these criteria, we assumed the EST was transcribed from the exon. Among the 4277 sequences, we found 2728 In100-elements (in 2012 genes) that have both upstream and downstream exons annotated with ESTs. The frequency of ESTs representing the downstream exons of the In100-element is 12.5% higher than that of the upstream exons (Figure 4).

In order to examine whether this increase in the frequency of ESTs representing the exons immediately downstream of In100-elements is associated with the presence of In100-elements, we constructed and analyzed the negative control exon pairs of In100-elements. We selected exon pairs that do not immediately flank those elements from the same 2757 In100-element containing genes. In this set of 23624 pairs of control exons, the frequency of ESTs representing the downstream exons is only 1.7% higher than that of the upstream exons. The difference in the comparison of In100-element flanking exons and the control exon pairs is highly significant (Fisher's Exact Test, P<2.2×10^−16^, df = 1; Figure 4).

This control group of exon pairs also works as a control for over-representation of the downstream exons as a result of the biased cDNA cloning or EST distribution. Namely, the observed gradient of EST representation could merely be a reflection of an uneven EST distribution in the EST database, rather than the skipping of the In100- upstream exons. If this were true, we should expect to find a higher frequency of ESTs matched to the downstream than the upstream exon in the control group as well, since our control exon pairs show the same distribution of position along the mRNA sequences as the In100-element flanking exons (Figure S1). The highly significant difference (Fisher's Exact Test, P<2.2×10^−16^) between the differential EST frequency (downstream versus upstream exons) of the In100-containing exon pairs (12.5%) and the control group (1.7%) indicates that the observed gradient is not a result of biased EST distribution along the mRNA transcripts. This excess of downstream exons of In100-containing sequences in the human transcriptome supports the association between upstream exon skipping and the presence of In100-like elements.

**Figure 5:**
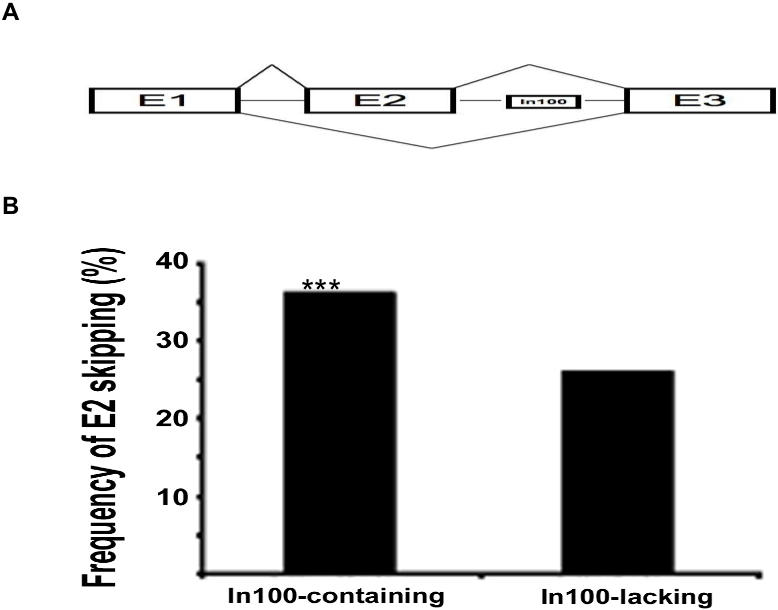
The alternative splicing of In100-containing genes. (A) A diagram depicting the alternative splicing of In100-containing genes. In100-containing genes were identified with three flanking exons (E1 to E3) from EST database, illustrated as E1-E2-In100-E3. 482 genes from EST database and an additional 44 genes from AltSplice [14] Database, with a total of 526 different genes show exon 2 skipping. (B) A higher frequency of skipping of exon2 in In100-containing exon triplets revealed by EST analyses. Frequency of skipping of exon2 in In100-containing and In100-lacking exon triplets by EST evidence is: 36% and 26%, respectively. The sample sizes for analyzed In100-containing and control exon triplets are: 1470 and 15943, respectively.

In order to verify and quantify this association, we examined three-exon cassette sequences and compared middle exon skipping (Figure 5A) in In100-element containing genes versus In100-lacking control group cassettes. The rationale is that for In100-containing exon triplets (e.g. exon1-exon2-In100-exon3; Figure 5A), the presence of ESTs representing both complete exon1-exon2-exon3 transcript and exon2-lacking transcript (i.e., exon1-exon3) indicates the alternative splicing event with exon2 skipping. However, due to the possible incompleteness of ESTs database, such exon-skipping evidence may not be found for every exon triplet. Therefore, from In100-containing genes, we only included the ones with sufficient EST information, namely the ones showing at least one EST simultaneously matching exon1 and exon3 (with/without exon2). Among them, 1470 triplets (from 1252 genes) are In100-containing exon triplets, and 15943 triplets (from 2036 genes) are In100-lacking exon triplets. These two groups of exon triplets have similar distribution of position along mRNA sequence (Figure S2). We treated the former group as the experimental set and the latter group as the control set. In the experimental set of 1470 In100-containing exon triplets, 524 triplets (36% of 1470) have at least one EST presenting exon 2 skipping; whereas in the control set of 15943 In100-lacking exon triplets, only 4116 triplets (26%) have such ESTs (Figure 5B and Table S6). Fisher's Exact Test showed that the frequency of the exon-skipping events is significantly higher in In100-containing exon triplets than In100-lacking ones (P<2.3×10^−15^). These 524 In100-containing exon triplets are from 482 genes (Table S4). Thus, 38% (482 out of 1252) of these In100-element containing genes show EST evidence for the skipping of exons immediate upstream of In100-residing introns.

Finally, we searched AltSplice [14] Database, a data source recording alternative-splicing events. We identified 69 In100-containing genes with clear evidence of skipping of exons upstream of the In100 element (Table S5). Twenty-five of these 69 genes were the same as the ones identified by our ESTs analysis. Thus, we have identified 526 In100-containing genes that have evidence of skipping of exons immediately upstream of In100-elements. These results provide strong evidence for the correlation of In100-elements with the skipping of their immediate upstream exons.

### The presence of In100-like elements in caspase genes and their alternative splicing

**Figure 6:**
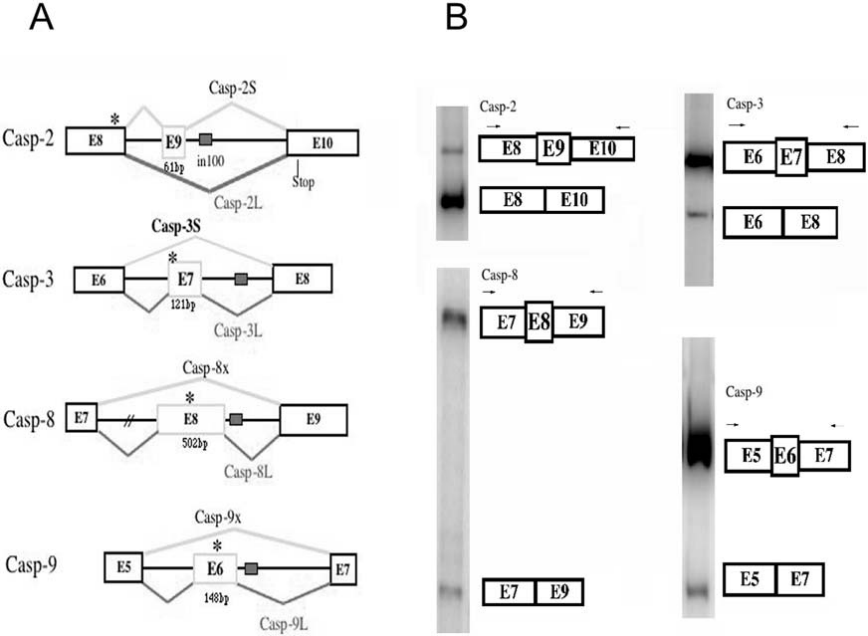
Alternative splicing of caspase genes. (A) A schematic illustration of In100-like elements in caspase genes and their alternative splicing patterns. Exons are depicted in rectangles, and In100 elements are illustrated as small gray boxes. The positions of regions encoding “QACRG” pentapeptide sequence are marked with “*”. (B) Alternative splicing of Caspase 2, 3, 8, 9 genes as detected by RT-PCR. Specific primers corresponding to the upstream and downstream exons were used in RT-PCR as described in Materials and Methods. The sizes of each exons are not drawn to scale. The positions of PCR primers are marked by arrows.

The analyses of human genomes described above have revealed a large number of In100-like elements and provide an opportunity to further investigate predicted alternative splicing patterns of the output candidate genes. Because In100 was first identified in casp-2 gene, we asked whether such elements are also found in other caspase genes. Indeed, the In100-like element was found in several human caspase gene sequences, including caspase 2, 3, 8 and 9 (Table 3). We systematically analyzed human caspase gene structures and examined the location of these In100-like elements. Interestingly, the In100-like elements in these caspase genes are located in introns adjacent to exons encoding caspase active sites with “QACXG” signature motif (Figure 6A). If these In100-like elements act to suppress the inclusion of the upstream exon, as predicted by our casp-2 study, such alternative splicing events will have significant functional impact because of the formation of isoforms that either contain or lack the caspase catalytically active domain.

**Table 3:** The Ensembl gene ID and transcript ID used to assign exon numbers

Gene	Ensembl gene ID	Transcript ID
Casp-2	ENSG00000106144	ENST00000350623
Casp-3	ENSG00000164305	ENST00000308394
Casp-8	ENSG00000064012	ENST00000303385
Casp-9	ENSG00000132906	ENST00000333868

We used two approaches to confirm these predicted alternative-splicing events in the corresponding caspase genes. First, we searched human EST and cDNA databases and aligned corresponding ESTs with the genomic sequences of these caspase genes. In each of the caspase genes examined, there are EST or cDNA sequences indicative of alternative splicing. The presence of these EST or cDNA sequences indicates that these caspase genes are alternatively spliced in this region. More importantly, caspase-2, 3, 8 and 9, all undergo alternative splicing in the exon encoding QACXG motif, the caspase active site.

We then used a RT-PCR assay to examine the alternative splicing of selected caspase genes in human cells. Using specific primers corresponding to flanking exonic sequences to perform RT-PCR assay, we confirmed that casp- 2, 3, 8 and 9 all undergo alternative splicing as predicted (Figure 6B). These experimental data suggest that the presence of In100-like elements has a predictive value for alternative splicing and strongly support the hypothesis that In100 elements represent intronic signals for alternative splicing.

## Discussion

In this study, we employed bioinformatics and experimental approaches to search for sequence signals associated with alternative splicing. Our data reveal that In100, originally identified in the mouse casp-2 gene, is present in thousands of unrelated human genes and possibly involved in regulating their alternative splicing. Of 1252 In100-containing genes for which sufficient EST information is available, 482 genes display skipping of exons immediate upstream of the In100-residing introns. An additional 44 In100-containing genes with such alternative splicing pattern are found in AltSplice Database [14]. The experimental analyses of elements found in human caspase genes, including casp-2, 3, 8 and 9, confirmed that these genes indeed undergo alternative splicing in the regions containing In100-elements. Our analyses support that In100 elements represent a family of intronic sequence motif involved in alternative splicing of many different genes.

Alternative splicing is a complex process involving interactions between multiple trans-acting factors with a large number of cis-acting regulatory elements. Identifying the In100-like elements in a large number of genes does not necessarily predict identical splicing patterns of these genes in different cell types, because In100-elements may interact with different cis- regulatory elements in different genes and trans-acting factors in different types of cells. How these unknown factors interact with In100-elements and define the alternative splicing patterns are interesting problems to be addressed by further experiments. The large number of In100 containing genes, each with its specifics in interaction with In100 elements, provides tremendous opportunities for the research community to further investigate detailed mechanisms of alternative splicing regulation in these genes (Table S1).

Intronic and exonic regulatory elements can enhance or suppress splicing, including exonic splicing enhancers (ESEs), intronic splicing enhancers (ISEs) or exonic splicing silencers (ESSs) and intronic splicing silencers (ISSs). Most of these cis-acting regulatory elements have been identified by biochemical and molecular studies of alternative splicing of individual genes of interest, (for recent reviews, see [15]–[20]). Programs predicting exonic splicing elements based on systematic evolution of ligands by exponential enrichment (SELEX) approach and computational analyses have been developed (for example, http://exon.cshl.org/ESE, also see [21]–[26]). Recently, a ternary combination of exonic UAGG and 5′-splice-site-proximal GGGG motifs have been reported that function cooperatively to silence the brain-region-specific CI exon 19 of the glutamate NMDA R1 receptor transcript [27].

A variety of intronic splicing regulatory elements have been identified using biochemical approaches. Only a few studies have reported systematic approaches to search for intronic regulatory elements. Analyses of small vertebrate introns suggest that GGG-containing sequences may act as intronic splicing enhancers in small introns [28]. A study of brain-enriched alternative exons shows that putative intronic splicing enhancers containing UGCAUG motif are often found in introns flanking alternatively spliced exons [29]. Analyses of 54 alternatively spliced exons revealed C-rich and G-rich sequences in the flanking introns [30].

In100 element has a complex structure. Most of the previously reported splicing regulatory elements, especially those identified by computational approaches, are in the range of 6-15mers [22]–[30], whereas In100-like elements described here are about 100 nucleotides in length, containing the decoy 3′ splice site domain and the pyrimidine-rich domain.

A role for pseudo-splice sites in regulating splicing has been suggested by previous studies. In *Drosophila* P-element intron 3 splicing, a pseudo 5′ splice site competes with the normal 5′ SS for interaction with U1 snRNP [31]. A sequence similar to a 5′ splice site [32] and a U-rich element adjacent to the regulated 5′ splice site [33] have been reported to act as intronic splicing repressors. In *C. elegans*, an alternative exon in U2AF65 pre-mRNA contains multiple copies of 3′ splice site consensus sequences [34]. These single case studies appeal for further studies at the genomic level. Our study of In100-elements suggests that decoy 3′ splice sites juxtaposed to pyrimidine-rich sequences may act as general signals for alternative splicing.

Mechanisms by which the decoy 3′ splice site of In100 is recognized as a signal for alternative splicing rather than an authentic 3′ splice site remain to be elucidated. PTB binding to the downstream domain of In100 plays an important role [10]. Our biochemical studies suggest the involvement of additional factors [9], (KF and JYW unpublished results). We noticed that some of In100-like elements contain polyU or polyC stretches that do not resemble typical PTB-binding sites. It is conceivable that such sequences may interact with other splicing regulatory factors.

In100 elements are distinct from other intronic splicing regulatory elements, including splicing suppressor elements containing hnRNP A1 or PTB binding sites, reviewed in [16], [17], [35], [36], UGCATG-containing elements [29], U/G-rich intronic elements that interact with CUG-repeat binding proteins, reviewed in [16] or (UCAUY)3-containing sequences binding to Nova proteins [37] or the C-rich and G-rich motifs [27], [30]. We examined these known splicing suppressor sequences and did not find sequence features of In100-elements.

Although a great number of human EST sequences have been generated, we were only able to obtain 2259 In100-element containing genes with flanking exons annotated with EST sequences. These ESTs clearly show a gradient distribution, with significantly higher frequency of EST representation of the exons immediate downstream of In100-containing introns. Analyses of In100-containing sequences annotated with further upstream and downstream exons in 1252 genes revealed 526 genes that have EST evidence of alternative splicing, showing that the presence of In100 elements correlates with the skipping of their immediate upstream exons. As we focused on In100-containing genes with sufficient EST data, these 526 genes only represent a small subset of possible alternative spliced genes. The observation that In100 elements exist in different gene families and their correlation with alternative splicing provides an opportunity for further study of In100 elements as a genomic signal for alternative splicing.

The finding that the exons upstream of In100 are often skipped corroborates with the alternative splicing patterns observed for several caspase genes, including casp-2, 3, 8 and 9. Some of these splicing isoforms have been reported as distinct cDNAs with different biological activities [8], [9], [38]–[40]. Our experiments also reveal previously unknown splicing isoforms, Casp-8x and Casp-9x, as a result of skipping of exons upstream of their In100-containing introns (Figure 6). These splicing isoforms encode products lacking the caspase active domain. It is obvious that the dosage of active caspases has to be tightly controlled in response to different cell death signals. By generating enzymatically active versus inactive isoforms, alternative splicing provides an excellent mechanism for fine-tuning the level of active caspases at the post-transcriptional level.

## Materials and Methods

### Construction of a human exon-intron sequence database

The sequence and intron/exon annotation of each gene in human genome were downloaded from BioMart, annotated by ENSEMBL 44 GENES (SANGER), (http://www.biomart.org). We constructed database where each human gene spans from the 5′ most exon to the 3′ most exon of all transcripts, including all coding and non-coding exonic sequence as well as intronic sequences. In addition, exon and intron positions as well as description of gene sequences were recorded.

### Creation of an algorithm to scan In100 candidate sequences

A program was written in C language to search In100 candidate sequences in the human exon-intron sequence database. In the program, we designed a sliding window along the DNA sequences to move from the 5′ to the 3′ end of each sequence. The sliding window is 100 nt in length based on the length of the In100 element defined by biochemical experiments on casp-2. The signal of In100 was defined as: UAAC[branch site]-5-10 nt-(Py)n[polypyrimidine tract = U/C rich]-CAG-(U/C rich, 20–60 nt). The program searched candidate sequences for In100 elements and recorded their locations in introns or exons, the locus names as well as the description of the gene. The nucleotide composition of In100-like elements in the regions with the features contributing to the alternative splicing was analyzed, using Web-Logo [12] (http://www.weblogo.berkeley.edu) and PERL scripts.

### Analysis of Gene ontology and Gene super-family

Both Gene family and Gene Ontology information were downloaded from BioMart, anotated by ENSEMBL 44 GENES (SANGER), (http://www.biomart.org). The classification of In100-containing genes into gene families was executed with PERL scripts. The GO terms were binned into MGI GO_Slim categories, using MGI GO_Slim Chart Tool (http://proto.informatics.jax.org/prototypes/GOTools/web-docs/MGI_GO_Slim_Chart.html). With GOstat [41] (http://gostat.wehi.edu.au/), the GO distribution of In100-containing genes and all Ensemble annotated human genes were compared and the over-presented GO terms in In100-containing genes were revealed.

### Analysis of the factors associated with the enrichment of In100-elements

To construct the control set for analyzing the factors associated with the enrichment of In100-elements, we generated 1000 intron-exon databases by simulation. In each simulated intron-exon database, we randomly generated a) 1500 genes; b) for each gene, the number of exons and introns are 7 and 6, since the average number of exon and intron in the real human intron-exon database per gene is 7.3 and 6.3; c) for each exon and intron sequence, the frequencies of their nucleotide composition are the same as the ones in the real human intron-exon database; d) the distribution of the length of intronic and exonic regions are the same as those in the real database. Then we searched In100-element sequences in those 1000 simulated intron-exon databases. We found that the total number of In100-elements found in the simulated databases is 8 times lower than the observed one in the real human intron-exon database. We also normalized these numbers by the total length of the corresponding databases (i.e. the frequency of In100-elements) and compared the frequency of In100-elements in the simulated database with the one observed in the human genome. The frequency of In100-elements in the human genome is significantly different from that detected in the simulated databases (Figure S3).

### Sequence analyses and statistical tests

To determine whether the detected In100 candidates are authentic intronic elements or random sequences, we used two approaches: (1) Comparing the frequencies of the candidates in exons and introns based on the total length distribution of exons and introns; (2) examining the frequency of trinucleotide (CAG, TAG, GAG and AAG) at the last three positions of the decoy 3′ splice sites in intronic and exonic regions, respectively.

First, a chi-square test was used to compare the observed frequency distribution of the elements in introns and exons with the expected frequency distribution based on the ratio of total exon length over total intron length with a degree of freedom of 1. Second, if the frequency differences of the In100 candidate in the four types (CAG, TAG, GAG, and AAG) are a consequence of random distribution of nucleotide composition, they are expected to be proportional to the frequency of four nucleotides A, T, G, C, in the human intronic and exonic regions respectively. We thus used human nucleotide frequencies in the intron and exon sequences as a theoretical expectation of the four types of candidate elements. Chi-square test was used to test the difference between observed and expected distribution with a degree of freedom of 3.

The Human EST database was downloaded from ncbi.nlm.nih.gov (dbEST release 041405). The pairs of immediate flanking exons of 4277 In100-elements (located within introns) were extracted according to the Ensembl 44 gene annotation. For In100-element lacking control group, we selected exon pairs located in the same In100-element containing genes by excluding exon pairs that immediately flank an In100-element. The first or the last exons of the genes were also excluded to avoid compounding effects due to potential influence of alternative promoter usage or alternative polyadenylation. In order to quantitatively analyze the splicing pattern of an exon pair, we separately aligned each exon against the human EST database using BLAT [42]. Alignments with sequence identity equal or higher than 98% that cover at least 70% exon length were used as the criteria to identify the match of an EST with a particular exon.

From 2757 In100-containing genes, we chose the triple exon cassettes with sufficient EST information, namely the ones with at least one EST simultaneously representing Exon1 and Exon3 (with/without Exon2). Alignments with at least 98% sequence identity and 70% exon length coverage were used as the criteria to identify the representation of an EST with particular exons. Next, In100-containing exon triplets (i.e. exon1-exon2-In100-exon3) and In100-lacking exon triplets (i.e., exon triplets not overlapped with In100-containing ones) were separated as experimental and negative control sets. The presence of ESTs with exon1-exon3 (lacking exon2) sequences is considered as the evidence of skipping of Exon2. Thus, the frequencies of exon2 skipping were computed for In100-containing or In100-lacking exon triplets respectively (Table S6). In addition, AltSplice [14] database (www.ebi.ac.uk/asd/altsplice) was searched for In100-element containing genes. All the intermediate steps were carried out with PERL scripts.

### RT-PCR detection of alternative splicing of caspases

RNA was extracted from HEK293 cells for detecting human caspase gene alternative splicing. Specific primers corresponding to the exonic regions of human caspase genes were used to detect splicing isoforms by RT-PCR with the previously described protocol [9]. The corresponding splicing isoforms were verified by sequencing.

## Supporting Information

Table S1 List of 4342 sequences containing In100-like elements.
(1.85 MB PDF)

Table S2 List of 1865 gene families involved in the 2491 In100-containing genes. These gene families are ordered by the number of In100-containing members in each family.
(0.35 MB XLS)

Table S3 The top over-presented GO terms of In100-containing genes revealed in Biology Process, Molecular Function and Cell Component.
(0.04 MB XLS)

Table S4 List of skipped In100-element upstream exon.Among total 524 cases, 395 show EST retaining and skipping the immediate upstream exons, and 129 present only EST skipping them.
(0.27 MB XLS)

Table S5 List of 75 In100-like sequences (in 69 genes) with evidence of skipping of In100 upstream exon, identified from AltSplice [14] database
(0.02 MB XLS)

Table S6 The frequencies of exon2 skipping of experimental and control exon triplets by EST evidence.
(0.07 MB PDF)

Figure S1 Upstream exon positional distribution along the mRNA sequence.(A) In100-element flanking exon pairs (2728). (B) Negative Control exon pairs (23624).
(0.02 MB PDF)

Figure S2 The 5′ end positional distribution along the mRNA sequence. (A) In100-containing exon triplets (524). (B) In100-lacking exon triplets (4116)
(0.03 MB PDF)

Figure S3 The frequency of In100-elements found in the randomized exon-intron databases and in the human genome In100 frequency: the number of In100 elements per million nt in the sequence database. The histogram of the frequency of In100-elements found in simulated databases is in black and the range of those frequencies is: 0 - 0.89 per million nt, the mean is 0.54 per million nt and the standard deviation is 0.10 per million nt; the frequency of In100-elements in the real human intron-exon database is in red and the value is 3.8 per million nt. The probability to observe such frequency of In100-elements in a randomized database is less than 0.001.
(0.02 MB PDF)
