# Supplementary material for: An Intronic Signal for Alternative Splicing in the Human Genome
Source: PLoS One. 2007 Nov 28;2(11):e1246. doi: 10.1371/journal.pone.0001246 (PMC2082412; doi:10.1371/journal.pone.0001246)
Supplement: Table S6 — The frequencies of exon2 skipping of experimental and control exon triplets by EST evidence. (0.07 MB PDF) [file pone.0001246.s006.pdf]

*Experimental group: In100_containing triplets: Exon1-In100-Exon2-Exon3

*Control group: In100_lacking triplets: Exon1-Exon2-Exon3. They are the exon triplets in the same gene sets as the In100_containing triplets and don't overlap with any exon in In100_containing triplets.

The num of exon triplets/genes in In100_containing triplets and In100_lacking triplets are summarized into 4 categories:

a)Only covering E1-E3 refers to: the triplets/ genes only have the ESTs which cover E1-E3

b)Covering E1-E3 and E1-E2-E3 refers to: the triplets/ genes have two types of ESTs: one covering E1-E3 and the other covering E1-E2-E3

c)Only covering E1-E2-E3 refers to: the triplets/ genes only have the ESTs which cover E1-E2-E3

d)= a) + b) = skipping events refers to: the union of the triplets/genes from group a) and group b)

	**In100_containing tri**		**In100_lacking tri**	
	**Triplets**	**Genes**	**Triplets**	**Genes**
a) Only convering E1-E3	129 (8.8%)	128 (10%)	1017 (6.4%)	742 (36%)
b) Covering E1-E3 and E1-E2-E3	395 (27%)	362 (28%)	3099 (19.4%)	1182 (58%)
c) Only covering E1-E2-E3	946 (64%)	831 (66%)	11827 (74%)	1866 (92%)
d) = a) + b) = skipping events	524 (36%)	482 (38%)	4116 (26%)	1490 (73%)
Total = a) + b) + c)	1470	1252	15943	2036
