# Supplementary figures and images for: An Intronic Signal for Alternative Splicing in the Human Genome
Source: PLoS One. 2007 Nov 28;2(11):e1246. doi: 10.1371/journal.pone.0001246 (PMC2082412; doi:10.1371/journal.pone.0001246)

**A**

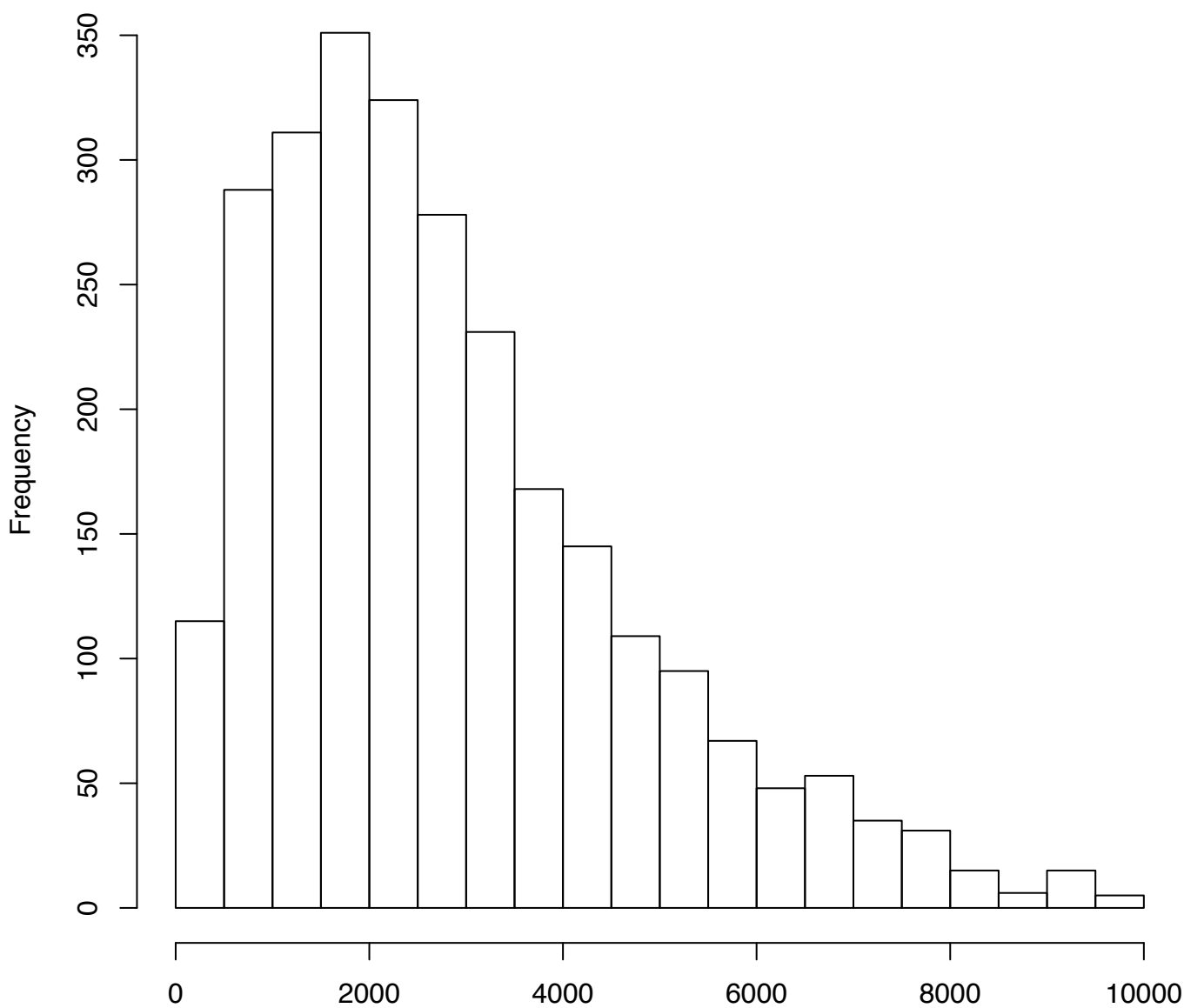

**B**

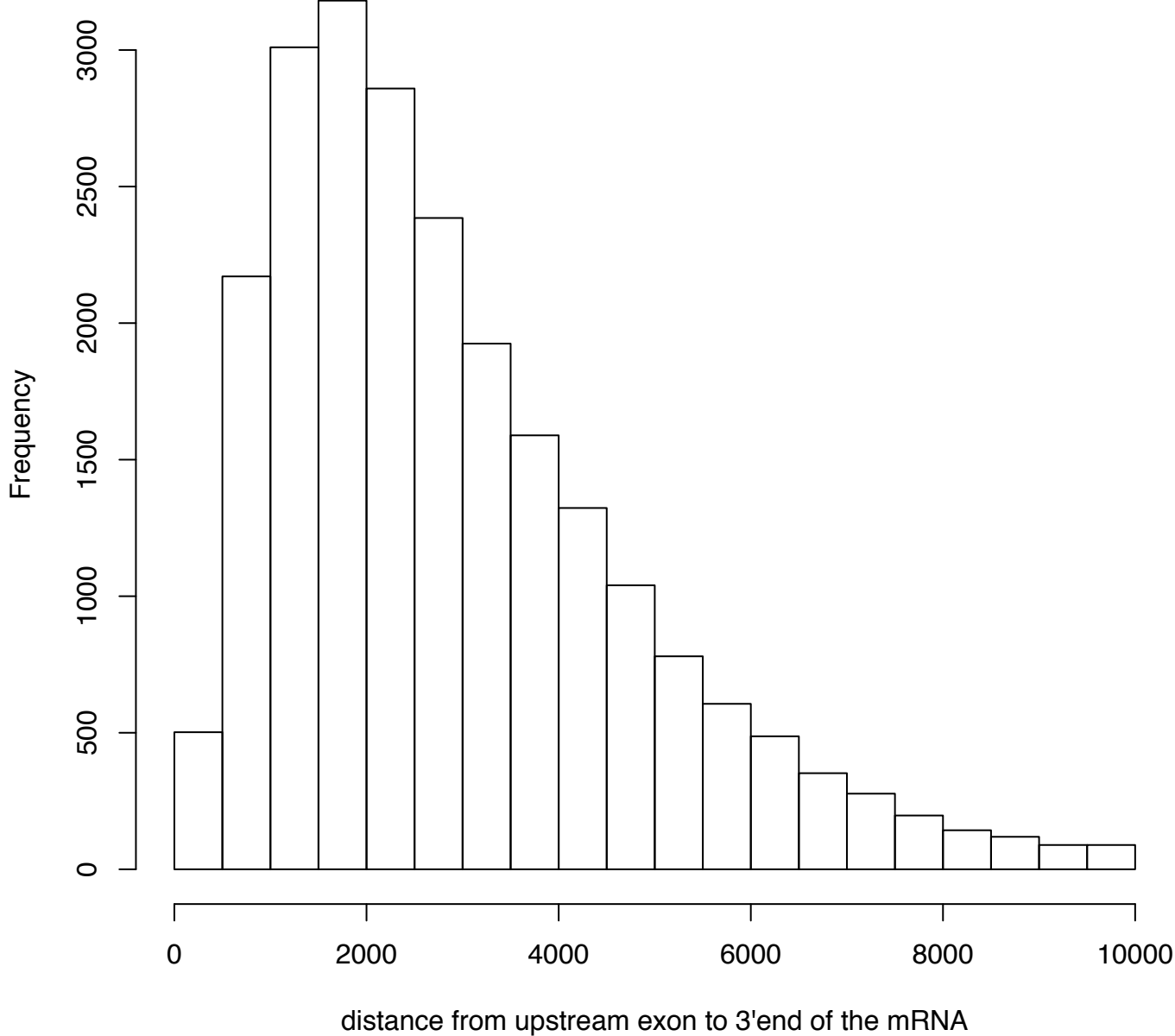

Supplement: Figure S1 — Upstream exon positional distribution along the mRNA sequence.(A) In100-element flanking exon pairs (2728). (B) Negative Control exon pairs (23624). (0.02 MB PDF) [file pone.0001246.s007.pdf]

**A**

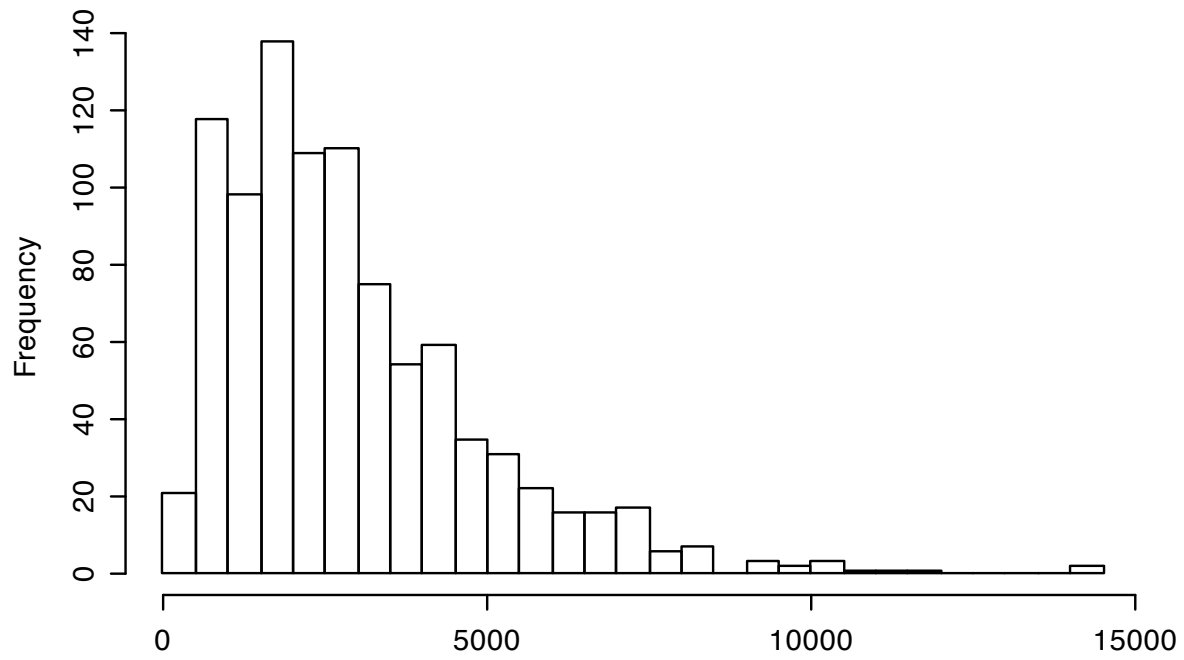

**B**

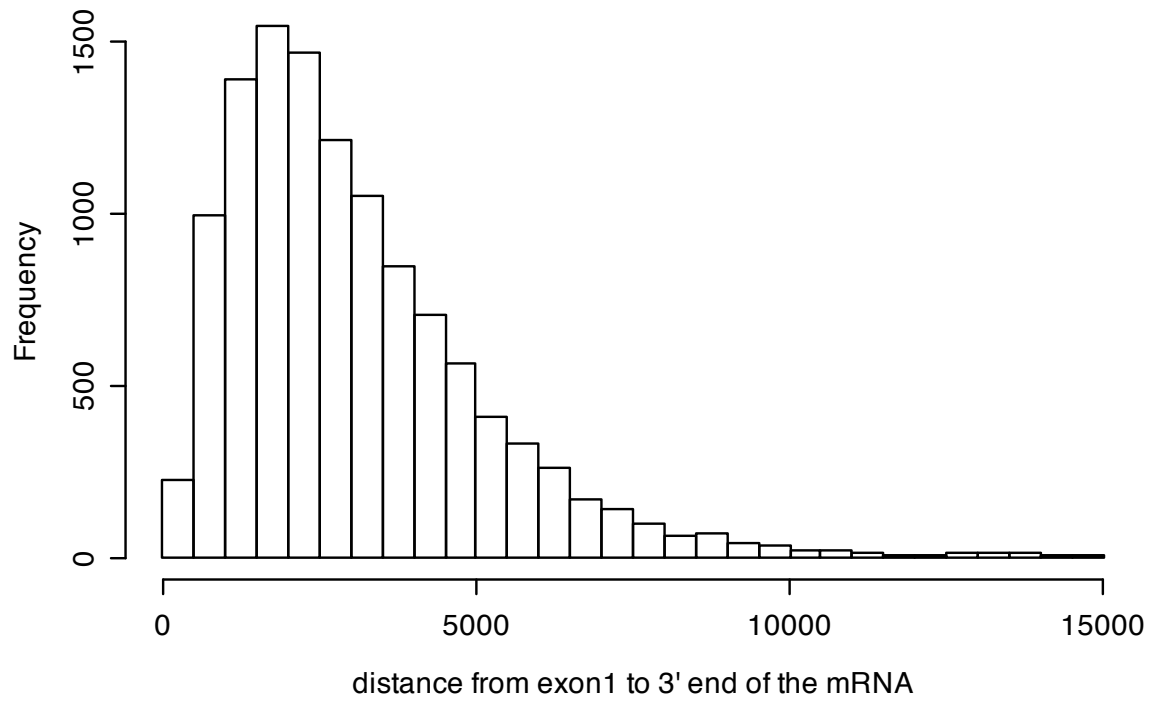

Supplement: Figure S2 — The 5′ end positional distribution along the mRNA sequence. (A) In100-containing exon triplets (524). (B) In100-lacking exon triplets (4116) (0.03 MB PDF) [file pone.0001246.s008.pdf]

Histogram of Frequency of In100-elements

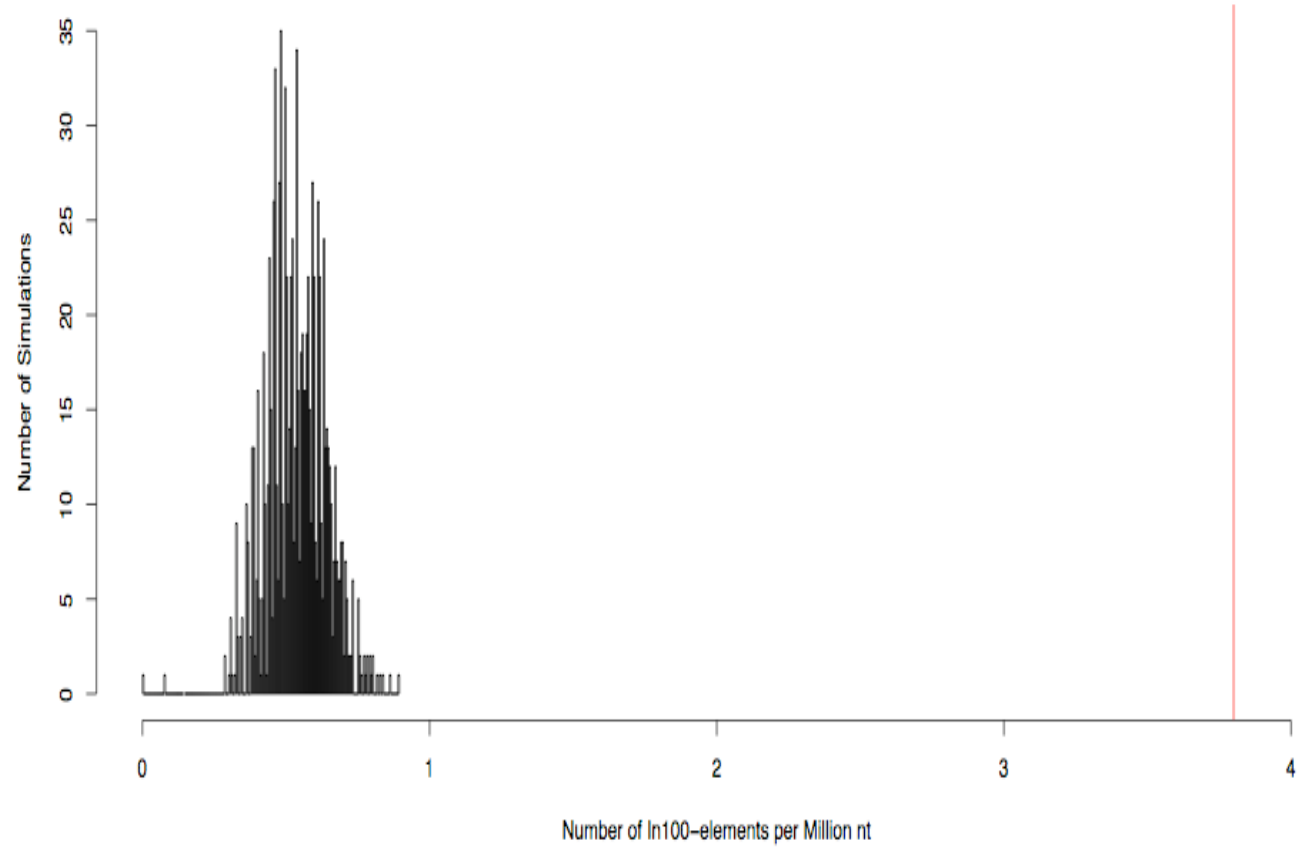

Supplement: Figure S3 — The frequency of In100-elements found in the randomized exon-intron databases and in the human genome In100 frequency: the number of In100 elements per million nt in the sequence database. The histogram of the frequency of In100-elements found in simulated databases is in black and the range of those frequencies is: 0 - 0.89 per million nt, the mean is 0.54 per million nt and the standard deviation is 0.10 per million nt; the frequency of In100-elements in the real human intron-exon database is in red and the value is 3.8 per million nt. The probability to observe such frequency of In100-elements in a randomized database is less than 0.001. (0.02 MB PDF) [file pone.0001246.s009.pdf]
