# Supplementary material for: An Intronic Signal for Alternative Splicing in the Human Genome
Source: PLoS One. 2007 Nov 28;2(11):e1246. doi: 10.1371/journal.pone.0001246 (PMC2082412; doi:10.1371/journal.pone.0001246)
Supplement: Table S1 — List of 4342 sequences containing In100-like elements. (1.85 MB PDF) [file pone.0001246.s001.pdf]

In100 candidate1 (in intron):

LOCUS ENSG00000099715

DESCRIPTION Protocadherin-11 Y-linked precursor (Protocadherin-11) (Protocaherin- 22) (Protocadherin on the Y chromosome) (PCDH-Y) (Protocadherin prosate cancer) (Protocadherin-PC). [Source:Uniprot/SWISSPROT;Acc:Q9BZA8]

TTATTAGATTTTTTTTCCTATAGAGTTGTTTAACCTCCTCATATGTTCTGGTTATTAATCCTTTATCAGATGTGTAGCTT
TTAAATATTTTCTCCCATTC

In100 candidate2 (in intron):

LOCUS ENSG00000099715

DESCRIPTION Protocadherin-11 Y-linked precursor (Protocadherin-11) (Protocaherin- 22) (Protocadherin on the Y chromosome) (PCDH-Y) (Protocadherin prosate cancer) (Protocadherin-PC). [Source:Uniprot/SWISSPROT;Acc:Q9BZA8]

ATATTCGTTCTTCCAATTCATTAACATGGAATGTCTTTCCATTTTTGTGTCCTCTACAGTTTTTTCCAGCACTGTTTTAT
AATGTTCATTGTCTTGATTT

In100 candidate3 (in intron):

LOCUS ENSG00000099715

DESCRIPTION Protocadherin-11 Y-linked precursor (Protocadherin-11) (Protocaherin- 22) (Protocadherin on the Y chromosome) (PCDH-Y) (Protocadherin prosate cancer) (Protocadherin-PC). [Source:Uniprot/SWISSPROT;Acc:Q9BZA8]

AGTAAGTTTACAAGTAACTTAAATGTGAGACTTTTTACTTTATTCTTCTTTTCAAGATTGTTTTCACTATTGCAGTTCTC
TTGAAATTCCATATGTTTTT

In100 candidate4 (in intron):

LOCUS ENSG00000099715

DESCRIPTION Protocadherin-11 Y-linked precursor (Protocadherin-11)

(Protocaherin- 22) (Protocadherin on the Y chromosome) (PCDH-Y) (Protocadherin

prosate cancer) (Protocadherin-PC). [Source:Uniprot/SWISSPROT;Acc:Q9BZA8]

ATGTATTTTAACCTAAATTTTATCTATCTATCTATCTATCTATCTATCTATCTATGTATCTATCTTCCTATTTAACAGAT

TTTCTATTTGTACATTCATG

In100 candidate5 (in intron):

LOCUS ENSG00000099715

DESCRIPTION Protocadherin-11 Y-linked precursor (Protocadherin-11)

(Protocaherin- 22) (Protocadherin on the Y chromosome) (PCDH-Y) (Protocadherin

prosate cancer) (Protocadherin-PC). [Source:Uniprot/SWISSPROT;Acc:Q9BZA8]

ATATTTTCTCCCATTTTGTGAGTTGTCTTTTAACTTTGTTTATTATTTTATTCATCCTGCAGAAGCTTTTTAACTTCACG

TGATCCCGTTTGTCCATTTT

In100 candidate6 (in intron):

LOCUS ENSG00000099715

DESCRIPTION Protocadherin-11 Y-linked precursor (Protocadherin-11)

(Protocaherin- 22) (Protocadherin on the Y chromosome) (PCDH-Y) (Protocadherin

prosate cancer) (Protocadherin-PC). [Source:Uniprot/SWISSPROT;Acc:Q9BZA8]

TGTCTTGATACCTATCTTGTCTTATATAACTGTTCTGCTCTTTTTTTTTTTATTTTCATTGGCAGGTATATTTTTGGCAG

ATATATTTTTCTATCCCCTT

In100 candidate7 (in intron):

LOCUS ENSG00000092377

DESCRIPTION F-box-like/WD repeat protein TBL1Y (Transducin beta-like 1Y proein) (Transducin-beta-like 1, Y-linked). [Source:Uniprot/SWISSPROT;Acc:Q9BQ7]

TCTAGTAACTTGCTTGCTTTTTTTTCCTGTTACCTTTTCTCCCTTAAATATTTTTTTTTTTTTTTTGAGACAGAGTCTC
GCTCTGTCTCTTGTGGGTCT

In100 candidate8 (in intron):

LOCUS ENSG00000092377

DESCRIPTION F-box-like/WD repeat protein TBL1Y (Transducin beta-like 1Y proein) (Transducin-beta-like 1, Y-linked). [Source:Uniprot/SWISSPROT;Acc:Q9BQ7]

AACCTACTGACATGTTGATTCTTTGAAAAGAAATTTAGAACTCATGTCTAACCACCCCTCCCCTTGTATTTCTTACAGCT
CTTTCTTCTCTAACTTTTTT

In100 candidate9 (in intron):

LOCUS ENSG00000099725

DESCRIPTION Serine/threonine-protein kinase PRKY (EC 2.7.11.1). [Source:Unirot/SWISSPROT;Acc:O43930]

TCCCACATTCCTCCATGGGAAGGTAACTTAATTTCTCTCCTTGTCTTGTTTCTTTCTTTCCTGACTTGTATCTCCCAGAG
CCTGCAGTACTTTTTTTCTT

In100 candidate10 (in intron):

LOCUS ENSG00000114374

DESCRIPTION Probable ubiquitin carboxyl-terminal hydrolase FAF-Y (EC 3.1.2.5) (Ubiquitin thioesterase FAF-Y) (Ubiquitin-specific-processing protease FF-Y) (Deubiquitinating enzyme FAF-Y) (Fat facets protein-related, Y- linked

(Ubiquitin-specific protease 9, Y chro [Source:Uniprot/SWISSPROT;Acc:O0050]

CTTTTATGTTTTAGAGATCGCTTCTTCCCTTAAACCTCATGAACTAACCTCTGCTAGTTTTAAGCTTTTCTTCTGCAGCT

TTCTTTTCTCTCAGCCTTTA

In100 candidate11 (in intron):

LOCUS ENSG00000067048

DESCRIPTION ATP-dependent RNA helicase DDX3Y (EC 3.6.1.-) (DEAD box protein3,

Y- chromosomal). [Source:Uniprot/SWISSPROT;Acc:O15523]

CTACTTCATAAGTTCTTTTACTTCTTAATCATAGACTGGCCTGTTGATTTAACTGAAAACACTTGATTTGTTTTCCAGAT

CATTTTCACTTTCCAACTTT

In100 candidate12 (in intron):

LOCUS ENSG00000183878

DESCRIPTION Ubiquitously transcribed Y chromosome tetratricopeptide repeat

rotein (Ubiquitously transcribed TPR protein on the Y chromosome).

[Source:niprot/SWISSPROT;Acc:O14607]

AGAATCCTCCGCATATAACAATATGTTCAATCTGCCACTTTTGGCATTCTGCTATTTTCAGTTAATTTTTTTTCATTTTT

TTCAATTTATTTTTGACTTT

In100 candidate13 (in intron):

LOCUS ENSG00000183878

DESCRIPTION Ubiquitously transcribed Y chromosome tetratricopeptide repeat

rotein (Ubiquitously transcribed TPR protein on the Y chromosome).

[Source:niprot/SWISSPROT;Acc:O14607]

AGATGAAGGTAATTAACCTGCTTTGGTTTCATGGAAAATTTTTTCTTTGCTTTTACCTTTTTCAGTCTCATCTACTGCCT

TCTGATTTAATGCTTCTAAT

In100 candidate14 (in intron):

LOCUS ENSG00000183878

DESCRIPTION Ubiquitously transcribed Y chromosome tetratricopeptide repeat

rotein (Ubiquitously transcribed TPR protein on the Y chromosome).

[Source:niprot/SWISSPROT;Acc:O14607]

AGTTGTATATGTTTTTTAACAAATTTTTTCCATTGGTTTTGTGTTTTCTATTTCAGTAATTTTTTTTCCTTCTTACCTGT

GTTATTTATTCCCTTGTATT

In100 candidate15 (in intron):

LOCUS ENSG00000183878

DESCRIPTION Ubiquitously transcribed Y chromosome tetratricopeptide repeat

rotein (Ubiquitously transcribed TPR protein on the Y chromosome).

[Source:niprot/SWISSPROT;Acc:O14607]

AAACACAAGCATAACCTCTACCCCAAGTTATTATTTTACCTATTTCCCAACTTTTTGTAATCAGATCTCTTCACCAAATC

AGTTGTTCTGCTTCTGTCTT

In100 candidate16 (in intron):

LOCUS ENSG00000165246

DESCRIPTION Neuroligin-4, Y-linked precursor (Neuroligin Y).

[Source:Unipro/SWISSPROT;Acc:Q8NFZ3]

GTTTTATGAGTTATGATGAATGCATCTTAGCTGTTTCTAACCTCCCTCCCATTCCCTATTTTTATTTGTCAGTTAAAGCA

CAGCATTTTTCCCTTTTTTT

In100 candidate17 (in intron):

LOCUS ENSG00000165246

DESCRIPTION Neuroligin-4, Y-linked precursor (Neuroligin Y).

[Source:Unipro/SWISSPROT;Acc:Q8NFZ3]

CCTATAACCATCAAAGATTAGTTTTTAATCTATTTGACTGATTTTTATTTTTTATACCTCTGCCTTCATTCTCACCAGAT

TTCTCTTTGACCACAGTTGT

In100 candidate18 (in intron):

LOCUS ENSG00000099749

DESCRIPTION Putative testis protein CYorf15A.

[Source:Uniprot/SWISSPROT;AccQ9BZA5]

ATTTTTGGTTATATAAAGTAAGTTTTAACCTAATGTCCAAATTAAATCCCTTTGTCTCTTTTCTCTGTCTCTTCCCAGTT

CTTTTTTCCTTGATCTTAAT

In100 candidate19 (in intron):

LOCUS ENSG00000180715

DESCRIPTION

AACTGATCCAAGCCTTAACTGTTGTACCTGTGTGATGAAGCAAATTTCTTTTTCCCCTCTTCTTTTTCATCTAATCAGAT

TCTAAATTCTACTCATTTCT

In100 candidate20 (in intron):

LOCUS ENSG00000101577

DESCRIPTION Lipin-2. [Source:Uniprot/SWISSPROT;Acc:Q92539]

AGTGTTCCACATAACGTGATGGCTGCCTCTGTATTTGTATTCACCAGTCGAAACCCGTCGCACTTTTTTTTTTTTTTTT

TTTTTTTTTAGCTGGAGTT

In100 candidate21 (in intron):

LOCUS ENSG00000170579

DESCRIPTION Disks large-associated protein 1 (DAP-1) (Guanylate kinaseassoiated protein) (hGKAP) (SAP90/PSD-95-associated protein 1) (SAPAP1) (PSD-9/SAP90-binding protein 1). [Source:Uniprot/SWISSPROT;Acc:O14490]

TCTAGTTCTGTCAATTGTTGGGAGGTAGTAACTGAGGTCTCCAACTACAATCGTGAATTTTTCTATTTCTCCTTTCAGTT

CTATCAGCTTTTTCATTCAA

In100 candidate22 (in intron):

LOCUS ENSG00000154655

DESCRIPTION l(3)mbt-like 4 [Source:RefSeq_peptide;Acc:NP_775735]

GTTTTATTTTAATCCTTTAAGAAGTTATGGAAACTGTTAACTTCCTGTCACTTTTCTCACCTAATTCGTCAGTTTCTTGT

GGGCATATCTTGGTTTTCTT

In100 candidate23 (in intron):

LOCUS ENSG00000154655

DESCRIPTION l(3)mbt-like 4 [Source:RefSeq_peptide;Acc:NP_775735]

TGTTGTGGGCCATTAACAATAATGTAACCCTTTTTGTGATTGCAGTTTCTCACATCCTTTCTGACCACCGCCATCTTTTT

TCTAGATAATTTCTTTGAAT

In100 candidate24 (in intron):

LOCUS ENSG00000088756

DESCRIPTION Rho GTPase activating protein 28 isoform b

[Source:RefSeq_peptie;Acc:NP_109597]

AATAACTAAATTAAACCTTAAATTTCAAGTTCTCTTCTTTTCCTTGAATTTTCTGCTCCCTTCAGAGTCATGTGTTTATT

TTAGCATCGTTGTTTTTCTC

In100 candidate25 (in intron):

LOCUS ENSG00000173482

DESCRIPTION Receptor-type tyrosine-protein phosphatase mu precursor (EC

3.13.48) (Protein-tyrosine phosphatase mu) (R-PTP-mu).

[Source:Uniprot/SWISSPRT;Acc:P28827]

GTTCTGGCACAAATAGCTAAAAACTCTCAACAAACGAGTTAACCTTGCTATTCCTAGTTTCTTAATCCTTCTGGTCAGTG

TTTTTTTGTTGTTGTTGTTG

In100 candidate26 (in intron):

LOCUS ENSG00000168502

DESCRIPTION Uncharacterized protein KIAA0802.

[Source:Uniprot/SWISSPROT;AccQ9Y4B5]

GTTTCCTAACTTCTTTTCCTTTTATTTCCCTTTCCTTTGCTTCACGCTTCTACATATCCTTTGGTTCAGTGGTCTCTCTC

AGCCTTATTGCTCATTTGAT

In100 candidate27 (in intron):

LOCUS ENSG00000188992

DESCRIPTION Lipase member I precursor (EC 3.1.1.-) (Membrane-associated phophatidic acid-selective phospholipase A1-beta) (mPA-PLA1 beta) (LPD lipase) [Source:Uniprot/SWISSPROT;Acc:Q6XZB0]

AAGTAGTAATATAACCCTTGTTACTGATTCCGTTTATTTGACTCTCCTCCTTTTCTTCTTTATTAGTCTAGCTAGCAGTC
TATTTATTGTATTTTTTTTT

In100 candidate28 (in intron):

LOCUS ENSG00000188992

DESCRIPTION Lipase member I precursor (EC 3.1.1.-) (Membrane-associated phophatidic acid-selective phospholipase A1-beta) (mPA-PLA1 beta) (LPD lipase) [Source:Uniprot/SWISSPROT;Acc:Q6XZB0]

TAGGAGTCTCTGCAAGGGGAGGCTCTAATGAGGGCATAACATAAGTCTCTTCTTTCTTTGTTATTTCTGTCATTGCAGCC
CTTTTCACTTCTTTGTCTGC

In100 candidate29 (in intron):

LOCUS ENSG00000155288

DESCRIPTION Putative ATP-binding cassette transporter C13. [Source:Uniprot/WISSPROT;Acc:Q9NSE7]

AGTATTTATTTTTCTGTGTCTAGCTTATTTCACTTAGCATAACGGCTTAAGGTCCATCCATGTTGTCTCATATTTCAGAG
TTGTCTTCTTTTTTAAGCCT

In100 candidate30 (in intron):

LOCUS ENSG00000155288

DESCRIPTION Putative ATP-binding cassette transporter C13.

[Source:Uniprot/WISSPROT;Acc:Q9NSE7]

GAAATTAACCTCTCATTTTACCTTTCATTTTCGTGGTGATTTTGCTGTCCTTTTTTCTATTGTTTCTTCTGTCTGCAGCA

CTTTATTGTTCATTCGTTTG

In100 candidate31 (in intron):

LOCUS ENSG00000155288

DESCRIPTION Putative ATP-binding cassette transporter C13.

[Source:Uniprot/WISSPROT;Acc:Q9NSE7]

AATCAACTCTAACTCCTTTTATTTTTCCTCATATATTTCTTTATACTCTTACATCTTTCCTGATTCCAGTCCTTTAGTGA

CTAAATTTTTGTTATTCTCT

In100 candidate32 (in intron):

LOCUS ENSG00000101745

DESCRIPTION Ankyrin repeat domain-containing protein 12 (Ankyrin repeatconaining cofactor 2) (GAC-1 protein). [Source:Uniprot/SWISSPROT;Acc:Q6UB98]

AGCATTCTTTCATAACTTTTTAATATTCCCTCTCTAGTTGTATTCAGGCTCAATCTCAGTGGTTTTTTTTTGTTTGTTTG

GTTGTTTTTATCTTTTCTCT

In100 candidate33 (in intron):

LOCUS ENSG00000155307

DESCRIPTION SAM domain-containing protein SAMSN-1 (SAM domain, SH3 domain ad nuclear localization signals protein 1) (SH3-SAM adaptor protein) (Hematooietic adaptor containing SH3 and SAM domains 1). [Source:Uniprot/SWISSPROTAcc:Q9NSI8]

GCATAGCCTTCTAGAGCTATCACTTTTCTATAGATTAATTTACACTTTCTAACTCCTACTATAGTTCTATGTACCCAGTC
TCCCTCAATTACTTTTTGAT

In100 candidate34 (in intron):

LOCUS ENSG00000155313

DESCRIPTION Ubiquitin carboxyl-terminal hydrolase 25 (EC 3.1.2.15) (Ubiquitn thioesterase 25) (Ubiquitin-specific-processing protease 25) (Deubiquitinting enzyme 25) (USP on chromosome 21). [Source:Uniprot/SWISSPROT;Acc:Q9UHP]

TTGTTTTTTAACGGTTTAAAGGTCTTTGCATATCTTTTGTTACAGTTGACTTTTTTGATGCTATTGTAAGTGGTATTTTT
TATTTTTATTTTTATTTTTT

In100 candidate35 (in intron):

LOCUS ENSG00000155313

DESCRIPTION Ubiquitin carboxyl-terminal hydrolase 25 (EC 3.1.2.15) (Ubiquitn thioesterase 25) (Ubiquitin-specific-processing protease 25) (Deubiquitinting enzyme 25) (USP on chromosome 21). [Source:Uniprot/SWISSPROT;Acc:Q9UHP]

TTTAGCTGAAGGAGCCAAGGACTGACTTAATGAGTCACCCACCGCTTAACACAAACTGTTTTTCTCCTTTGTTGTCAGTG
GTGCTTTAGTTTTCTTTTAC

In100 candidate36 (in intron):

LOCUS ENSG00000155313

DESCRIPTION Ubiquitin carboxyl-terminal hydrolase 25 (EC 3.1.2.15) (Ubiquitn thioesterase 25) (Ubiquitin-specific-processing protease 25) (Deubiquitinting

enzyme 25) (USP on chromosome 21). [Source:Uniprot/SWISSPROT;Acc:Q9UHP]

ATTTGGTTCATTTTCTACAAGTTAATTGTTTTTGCTAACTTGTAATTTTAACTTTTTTTTATATACTTCAGTGCTAAAAT

TTTCACCGTTTTCTCCCTTT

In100 candidate37 (in intron):

LOCUS ENSG00000155313

DESCRIPTION Ubiquitin carboxyl-terminal hydrolase 25 (EC 3.1.2.15) (Ubiquitn

thioesterase 25) (Ubiquitin-specific-processing protease 25) (Deubiquitinting

enzyme 25) (USP on chromosome 21). [Source:Uniprot/SWISSPROT;Acc:Q9UHP]

CCCTTCTCAAGGAAGAAATCATTTTTATAAAATATTGTTAACTTATATGTCTACCTCAACTAGTTTCTTTCTTTCCAGCC

ATCTCTGTTTCTTTATCCAT

In100 candidate38 (in intron):

LOCUS ENSG00000154845

DESCRIPTION Serine/threonine-protein phosphatase 4 regulatory subunit 1.

[Surce:Uniprot/SWISSPROT;Acc:Q8TF05]

AGATTTGAAAAATAAGAAATTTTTTATTTATCCCATATTAACCTTTTTTTTTTCTTTCTTTCTTTTTCTGGAGACAGGGT

CTTGCTTTGTCTTCCAGACT

In100 candidate39 (in intron):

LOCUS ENSG00000174496

DESCRIPTION

CTGGTATTAAAAAAATGGATTATTCTTTTTGATATAACTAAGATGAAACATTTTTTCATTTTTTCCTGTGATTTTCAGTT

TGTCATTTATTATTATATTT

In100 candidate40 (in intron):

LOCUS ENSG00000174496

DESCRIPTION

TGGGCGGACCATAACATAGAATTTGTGTTCTTGTGAAATTTCTCTCTCTGACCTGATTCTCTTTGATGTGCTCCCCAGGA

TTTTCTGTGTTTTAGATCTC

In100 candidate41 (in intron):

LOCUS ENSG00000174496

DESCRIPTION

TTGCTTATGGGCAAAGATGATTTTTTGGGGGAGATATTTGATTAACCTTCTAATTTCTTTTATCTTTGACACCTTCAGTC

TTCCTTCATTTACCTTTATA

In100 candidate42 (in intron):

LOCUS ENSG00000174496

DESCRIPTION

GAAATTGAAGCAAATGTGATTTCAAAGACCTAACTTTATCCAATTTTCTACTTTTTTCCAAGCTCTACATTTTCACAGTA

AAACTTCTGTTTTATCTTTT

In100 candidate43 (in intron):

LOCUS ENSG00000206249

DESCRIPTION

AGTTTGTGTCCTATATTGCTCTTCCATTATTTTTAACTCTTCTGTATCTTTTTCATACAACTCTTCAGCCTTTATTCTTT

CTTCTTCTTGTTGTTGTAAG

In100 candidate44 (in intron):

LOCUS ENSG00000154646

DESCRIPTION Enteropeptidase precursor (EC 3.4.21.9) (Enterokinase) (Serine rotease 7) [Contains: Enteropeptidase non-catalytic heavy chain; Enteropeptdase catalytic light chain]. [Source:Uniprot/SWISSPROT;Acc:P98073]

AGTTAACAAACCACTATTTTTCTTTCTTTCCTGTGATCTGTGCCGGATTGTTCAGAATTTAATCCTTGCTTCTTGACTTG
TTATCTTTTCTTTTCTTATT

In100 candidate45 (in intron):

LOCUS ENSG00000141404

DESCRIPTION Guanine nucleotide-binding protein G(olf) subunit alpha (Adenylte cyclase-stimulating G alpha protein, olfactory type). [Source:Uniprot/SWSSPROT;Acc:P38405]

TATTTTTATGTCTTGTAGATAAATATCATGAGTCAAATTTCATTAACATTGGGAAAAGTTTTTTACTTGCCTTGACAGAT
TTCCAATTGCTATATGTTTT

In100 candidate46 (in intron):

LOCUS ENSG00000154889

DESCRIPTION metallophosphoesterase 1 precursor [Source:RefSeq_peptide;Acc:N_075563]

AGGTATTTTATATTGTAATTCAAGATAGGTATTCTTGGCCACTAACCCAATGTCTTGTTTCTTTAGCTTCTCAGCTTCTT

TTTTACTTTCAAAGTTGGTC

In100 candidate47 (in intron):

LOCUS ENSG00000099968

DESCRIPTION Bcl-2-like 13 protein (Protein Mil1) (Bcl-rambo).

[Source:Uniprt/SWISSPROT;Acc:Q9BXK5]

TACACAAAAAAGAATAACCCGTTATGCCATTCTTTCCATTTCCAGAAGCAAAAAACAAAAGTATTTTTTTCATTTTATT

CTTTTTTTTTTTTTTTTTT

In100 candidate48 (in intron):

LOCUS ENSG00000154654

DESCRIPTION Neural cell adhesion molecule 2 precursor (N-CAM 2).

[Source:Unprot/SWISSPROT;Acc:O15394]

ATTCTACTACAAACACGTAACACGTGATTCATCTTCATTACTACTTGCTCCACTTGCCATTTCAATTTTATCAATCAGAA

TACTTCATCTTCTTCCTATT

In100 candidate49 (in intron):

LOCUS ENSG00000154727

DESCRIPTION GA-binding protein alpha chain (GABP-subunit alpha) (Transcripton factor E4TF1-60) (Nuclear respiratory factor 2 subunit alpha).

[Source:Uiprot/SWISSPROT;Acc:Q06546]

GTGAATATCCTTTTCCTATGCTTTAACTACTTTTCTAATTTGTCCCTTTTTTTGGTTATCAAATTCCAGGCCATTGTCTA

TTCCATCGTCACTTTTGGGT

In100 candidate50 (in intron):

LOCUS ENSG00000142192

DESCRIPTION Amyloid beta A4 protein precursor (APP) (ABPP) (Alzheimer diseae amyloid protein) (Cerebral vascular amyloid peptide) (CVAP) (Protease nexn-II) (PN-II) (APPI) (PreA4) [Contains: Soluble APP-alpha (S-APP- alpha); Sluble APPbeta

(S-APP-beta); C99; Beta- [Source:Uniprot/SWISSPROT;Acc:P0506]

CTCTGCTGTCTTATTTGTGTTGATATCTTTATCTTTATTTCTCTAAAAATAACATGTCTTTTTTTTCTGTTTTTACAGTT

TTCTCTATATCACTGGTTTT

In100 candidate51 (in intron):

LOCUS ENSG00000183785

DESCRIPTION Peroxisome assembly protein 26 (Peroxin-26).

[Source:Uniprot/SWSSPROT;Acc:Q7Z412]

GTTAACTTAGTCTTTAGATTCTCCCTGTTTCCCCATTTCTCTTTCTCTTATTCAGTGAGCTTTTTTAAAATATTTATTTA

TTTATTTTTATTTTTATTTT

In100 candidate52 (in intron):

LOCUS ENSG00000134278

DESCRIPTION spire homolog 1 [Source:RefSeq_peptide;Acc:NP_064533]

TGTATTTTAACTGTCATCTTTGATATTGCTAAATCTTTTTTGTTTTTTGTTTTTCTTAGTTTTTAGCAGAGTTTTACACT

GCAAGAACCTTTCTCTTTTT

In100 candidate53 (in intron):

LOCUS ENSG00000184979

DESCRIPTION Ubl carboxyl-terminal hydrolase 18 (EC 3.1.2.-) (Ubl thioesterae 18) (ISG15-specific-processing protease) (43 kDa ISG15-specific protease)(hUBP43). [Source:Uniprot/SWISSPROT;Acc:Q9UMW8]

CCCCTAACTCTCTTAAGATATCTTCACTCTTTTTCATTCTTTTTCATTTTTGCTCCTCTGAATGATTTCCAGTGACCTAT

CTTCTAGTTTGCTAATCCTT

In100 candidate54 (in intron):

LOCUS ENSG00000128789

DESCRIPTION tumor necrosis factor superfamily, member 5-induced protein 1 [ource:RefSeq_peptide;Acc:NP_064617]

ACTGAGAAAATTATTCTAACACTTAAGGCCATAACTACTTCTCAGAACTACAGTTTAATACTTGTTTTTCTTTTTTATAT

TTCCACAATTTGATTACTCT

In100 candidate55 (in intron):

LOCUS ENSG00000166265

DESCRIPTION Cysteine and tyrosine-rich protein 1 precursor (Proline-rich doain- containing protein). [Source:Uniprot/SWISSPROT;Acc:Q96J86]

GATACCTTCACGTCACTGACTCTTCTTTCATCCTTAACATCTTGTTAAGCCCATACAATTAATTTCCTTTTGTTTCAGAT

ATTTTATTCCTCATTTCTAG

In100 candidate56 (in intron):

LOCUS ENSG00000101639

DESCRIPTION centrosomal protein 192kDa [Source:RefSeq_peptide;Acc:NP_115518

CCTCTCTGGTCCGGAGTGTAATCTAACATCACATGATGCATTTGGTTATCCTATCTGCTTACTCTCTTTCAGAATAGAGT

TCCTTAGCCTTTCCTTATCT

In100 candidate57 (in intron):

LOCUS ENSG00000168675

DESCRIPTION Uncharacterized protein C18orf1.

[Source:Uniprot/SWISSPROT;Acc:15165]

GAATTTATCCTATTTGGAGTTTGTTGTAATTCCTGAGTGTATAGATTAACTTTTTCAATAACTTTAGTACATTTTCAGCC

ATTATTCTTTGGATTTTTGT

In100 candidate58 (in intron):

LOCUS ENSG00000067900

DESCRIPTION Rho-associated protein kinase 1 (EC 2.7.11.1) (Rho-associated,

oiled- coil-containing protein kinase 1) (p160 ROCK-1) (p160ROCK) (Renal

cacinoma antigen NY-REN-35). [Source:Uniprot/SWISSPROT;Acc:Q13464]

CAAAAGAGCCTGTTGATATGATCCATATGAGTCACAAACAAGACATAACCAACATTTATTTATTTATTTCGCTCTCAGTC

TTTTTTTTTTTTTTAAATTT

In100 candidate59 (in intron):

LOCUS ENSG00000067900

DESCRIPTION Rho-associated protein kinase 1 (EC 2.7.11.1) (Rho-associated,

oiled- coil-containing protein kinase 1) (p160 ROCK-1) (p160ROCK) (Renal

cacinoma antigen NY-REN-35). [Source:Uniprot/SWISSPROT;Acc:Q13464]

AGCTTAACCAAATATGTGCCAATTTTATCTTTTCAGAAGACCTACTTTGGGTCACATTGGCTTTCTCTGTTATTTTCCTA

TTCTGTGTTTTATTTATCTT

In100 candidate60 (in intron):

LOCUS ENSG00000093009

DESCRIPTION CDC45-related protein (PORC-PI-1) (Cdc45).

[Source:Uniprot/SWISPROT;Acc:O75419]

ATTCCTCTTTACTGTCTTAACCTAGAACCATACCTTACCTCTTTTTTTTCTTTTGTTTTTAATGACATTGAAGGCCAGTT

CTCTTTTCTAATATTTACTA

In100 candidate61 (in intron):

LOCUS ENSG00000101752

DESCRIPTION E3 ubiquitin-protein ligase MIB1 (EC 6.3.2.-) (Mind bomb homolo

1) (DAPK-interacting protein 1) (DIP-1) (Zinc finger ZZ type with ankyrin epeat

domain protein 2). [Source:Uniprot/SWISSPROT;Acc:Q86YT6]

TTTCCTCCACTCCTTTTCTGAGAGGTTCCTTAACTTAATTTTCCCACCCTTTTTCCAAGGTTTTTATTATTCCATCAGTT

TTAAATTTCAAGCTTTTCTT

In100 candidate62 (in intron):

LOCUS ENSG00000134490

DESCRIPTION CDNA FLJ41629 fis, clone DFNES2010502.

[Source:Uniprot/SPTREMBLAcc:Q6ZW41]

AGCTAGGTCTTTCTTAACAAAAAGTGGCAAAAGTGTTATTGCTTTTTTCCCCTCTTTTCAGATCTCATGTTCTTGTGTGG

CTTCCTGCTTCAGTGCTGTT

In100 candidate63 (in intron):

LOCUS ENSG00000053747

DESCRIPTION Laminin subunit alpha-3 precursor (Epiligrin 170 kDa subunit)
(170) (Nicein subunit alpha). [Source:Uniprot/SWISSPROT;Acc:Q16787]

ACTTGGGGAAGTTAATTAACCTTTCTGAGCATCTTTTTTTTTTTTTTCATCTATAAAATACTTACCTGTCTTACAGGG
CTCTTGTTTGACTTCATTAT

In100 candidate64 (in intron):

LOCUS ENSG00000040608

DESCRIPTION Reticulon-4 receptor precursor (Nogo receptor) (NgR) (Nogo-66
rceptor). [Source:Uniprot/SWISSPROT;Acc:Q9BZR6]

AAGATTTCTTTGACCTTGTTTTCTAACACTTGTATTGATTTTTTTTTTAATTTTAGTGATTCTATTTTTAATTTCCAGGC
ACTCTTTCTTTTTGTCTGAT

In100 candidate65 (in intron):

LOCUS ENSG00000142207

DESCRIPTION Nucleolar preribosomal-associated protein 1 (Fragment).
[SourceUniprot/SWISSPROT;Acc:O60287]

TGTGTTTGTCCTGTTTCTTTCTCATCCCTCTGTAACCAAAGGTCAACACTCTTCTGAGGTTCTGCTTTGTCTCGTCAGTA
TCTTATTTTGTATTTTCATA

In100 candidate66 (in intron):

LOCUS ENSG00000168234

DESCRIPTION TPR repeat-containing protein C18orf17.

[Source:Uniprot/SWISSPRT;Acc:Q8N584]

CTCCTAAACCCTAAATGAAGAAGGATAAATTTCTTTTAAATTATAACATACTCTTTTCTTTCTAGCCTATCAGAAGCCGG

TTTTTCCCCCTTTTTCTTGT

In100 candidate67 (in intron):

LOCUS ENSG00000161133

DESCRIPTION Ubiquitin carboxyl-terminal hydrolase 41 (EC 3.1.2.15) (Ubiquitn thioesterase 41) (Ubiquitin-specific-processing protease 41) (Deubiquitinting enzyme 41) (Fragment). [Source:Uniprot/SPTREMBL;Acc:Q3LFD5]

CCCCTAACTCTCTTAAGATATCTTCACTCTTTTTCATTCTTTTTCATTTTTGCTCCTCTGAATGATTTCCAGTGACCTAT

CTTCTAGTTTGCTAATCCTT

In100 candidate68 (in intron):

LOCUS ENSG00000198795

DESCRIPTION zinc finger protein 521 [Source:RefSeq_peptide;Acc:NP_056276]

CATCCAAAGGAAGTAACCCTCATCCCCGTTTTCTCACTTTTTCATATTTTTCAAGGGCTTACTTTTTATTTCCTACAGAG

CTTAATTTTTTTTTTTTTTT

In100 candidate69 (in intron):

LOCUS ENSG00000141380

DESCRIPTION SSXT protein (Synovial sarcoma, translocated to X chromosome) (YT protein). [Source:Uniprot/SWISSPROT;Acc:Q15532]

AGTTAAACTTAGACTAGACCTTGTAACTGTATCTTTATGTTTCTTAATCCCTTCAGACTTTTCCAACCATTTATCATTCT

AAGTATATTTTTTTCTGACC

In100 candidate70 (in intron):

LOCUS ENSG00000134504

DESCRIPTION BTB/POZ domain-containing protein KCTD1.

[Source:Uniprot/SWISSPOT;Acc:Q719H9]

TCTACTATTAAACATTACGGAAATATATAACTTAAAAGTGAAAATTCCCTGTAATGTTTTTTGTTTATATTCTTTCAGAT

TTTTCTATGTATACTTATTT

In100 candidate71 (in intron):

LOCUS ENSG00000170558

DESCRIPTION Cadherin-2 precursor (Neural-cadherin) (N-cadherin) (CD325

antien) (CDw325). [Source:Uniprot/SWISSPROT;Acc:P19022]

AGATTTTAATTGTACTAACTTTTAAATTGACTGCTACCTTATTCTGTGCTGTTTATATGCCTTTATTTTTCCAGTTCAAC

TTTTTTTTCTAGTGTTCTGT

In100 candidate72 (in intron):

LOCUS ENSG00000153339

DESCRIPTION Protein TRS85 homolog. [Source:Uniprot/SWISSPROT;Acc:Q9Y2L5]

AAGGAATAGAAGTGAAACCATCTGTCTTCATAATATTTAGAAATAACCACTTTAACATTTTGCTGTGTATCTTTCCAGTC

ATTTTGTTTTTATTTTTTTT

In100 candidate73 (in intron):

LOCUS ENSG00000153339

DESCRIPTION Protein TRS85 homolog. [Source:Uniprot/SWISSPROT;Acc:Q9Y2L5]

AATTGTGATTTCATTAGTTTACCTAACTGTAAGTCAAATTACCTATCTTGATCCAGTCTTTCCCATTTTTTAAATTCCCT

CCCACATCTTTTATACTTGT

In100 candidate74 (in intron):

LOCUS ENSG00000153339

DESCRIPTION Protein TRS85 homolog. [Source:Uniprot/SWISSPROT;Acc:Q9Y2L5]

AGGAAAATTGCTTTTAAGAAGAAAAAAATATATAACTTTTTTGTTTATTTTTCTTCCATAGCCTTTGTTTAGTACCAGTC

ACTCTTTTACTTTCCAATTG

In100 candidate75 (in intron):

LOCUS ENSG00000100814

DESCRIPTION E3 ubiquitin-protein ligase CCNB1IP1 (EC 6.3.2.-) (Cyclin-B1-

iteracting protein 1) (Human enhancer of invasion 10).

[Source:Uniprot/SWISSROT;Acc:Q9NPC3]

AACATTTTCTTTAGTTGGTTAATCTTGATTTTGGTACTAACTTGTTATCTAGTTCCTCATTTTCCCTTGCTGGATCAGTT

GATCCTTAATGATCTCTTCT

In100 candidate76 (in intron):

LOCUS ENSG00000141441

DESCRIPTION Protein FAM59A. [Source:Uniprot/SWISSPROT;Acc:Q9H706]

CTTAATATACTAGCACACAACTTATAGAATTAACTAAAATCACATATATACTTGCCTTTTCTTTGTTCTGTCAACCAGGG

TTTTTTTTTTTGTGCAACAT

In100 candidate77 (in intron):

LOCUS ENSG00000141441

DESCRIPTION Protein FAM59A. [Source:Uniprot/SWISSPROT;Acc:Q9H706]

GTGTCCTAAAATGTGTATTCACTCACCCCACTTACGTACATATTGTAACTTTTCAAAGAATTGTCCTGGCTTCATCAGAT

AGTTTCATCTGCTTTGTTTT

In100 candidate78 (in intron):

LOCUS ENSG00000185686

DESCRIPTION Melanoma antigen preferentially expressed in tumors

(Preferentilly expressed antigen of melanoma) (OPA-interacting protein 4)

(OIP4). [Souce:Uniprot/SWISSPROT;Acc:P78395]

ATATTCCATTTTAACTAAAAGGTTAAAAAATATTCCATAGATTTTTTTCTTTTTCTTTTCAGATGCTTTGGAATTAAAGA

CATTTTCTTTCTTTTTTTTC

In100 candidate79 (in intron):

LOCUS ENSG00000166960

DESCRIPTION Uncharacterized protein C18orf34.

[Source:Uniprot/SWISSPROT;AccQ5BJE1]

ATGTTAATTATTTTACTTTATTTTATTAACAATAACTCAAACTTTGATTTTCAAGATCTCATTTTTCAGAAATTTTCCCA

TTGTTGACAATCCTCATTTT

In100 candidate80 (in intron):

LOCUS ENSG00000166960

DESCRIPTION Uncharacterized protein C18orf34.

[Source:Uniprot/SWISSPROT;AccQ5BJE1]

AGATATGTGGTTTCCAATTATTTTTTTCCTAGTATGTAACTGGACTTTTCATTTTTTTTAGTTTGATGCAGTTCCATTT

CTGTATTTTTGTCTTTGAAG

In100 candidate81 (in intron):

LOCUS ENSG00000166960

DESCRIPTION Uncharacterized protein C18orf34.

[Source:Uniprot/SWISSPROT;AccQ5BJE1]

TAGCTTACACATGCCACTTAACCAAGTTGATTCTACCCTTAGCCTTGGAAACCTTTTTCTTTTGGCTTCCAGAACACATT

CTTCATGGTTTTTGCTATTT

In100 candidate82 (in intron):

LOCUS ENSG00000166960

DESCRIPTION Uncharacterized protein C18orf34.

[Source:Uniprot/SWISSPROT;AccQ5BJE1]

CTTCAAAAAAAATCAAAAGCTTATGGTTTAACTTAATTCTCCATCTCTACATTTGACTATATCCATTGTTTATCTCAGAA

AGCTCTGTTCTTCTTACTCT

In100 candidate83 (in intron):

LOCUS ENSG00000101746

DESCRIPTION Nucleolar protein 4 (Nucleolar-localized protein).

[Source:Unipot/SWISSPROT;Acc:O94818]

TGTCATTATATGCTTTGGGATGGGGTTCATTTGCATAGTTAACTTTATTTCATTCTTTTCATATCCTATCAGTTGCATTT

TTTTATCTTGAATACATTTT

In100 candidate84 (in intron):

LOCUS ENSG00000101746

DESCRIPTION Nucleolar protein 4 (Nucleolar-localized protein).

[Source:Unipot/SWISSPROT;Acc:O94818]

GTACAACTTTTGCCTTAAAGTATATTTGATCTGATATTTTTATAACCTCTCCACTTCTCTTTTGGTTTCCCTTTGCAGCG

TATGTGTTTTCATCCTTTTA

In100 candidate85 (in intron):

LOCUS ENSG00000101746

DESCRIPTION Nucleolar protein 4 (Nucleolar-localized protein).

[Source:Unipot/SWISSPROT;Acc:O94818]

CTTTTACATTTCTTTTAAAATATATATTATAAATAACATAAATATCAATTATCCTTACTAGATCCACTTGTTTTCAGACT

CATCTTCCAATTTACTAGTT

In100 candidate86 (in intron):

LOCUS ENSG00000101746

DESCRIPTION Nucleolar protein 4 (Nucleolar-localized protein).

[Source:Unipot/SWISSPROT;Acc:O94818]

TTGTCTTAGTTATATTTCAATTATTTTTTTAAAGACTTGTCATTAACTACTGTATTAAGTCACTGTCTTCAGCCTCTGCC

TGTTGGATTTTAACCTGTTT

In100 candidate87 (in intron):

LOCUS ENSG00000101746

DESCRIPTION Nucleolar protein 4 (Nucleolar-localized protein).

[Source:Unipot/SWISSPROT;Acc:O94818]

ATAATGGAAGATTTCTTTCTTTTTAAAAATAACATTAAAATAAGTTAATGTTTCATTTCCTATCCTTGGCAGATTTTTAT

TTTTCAATATGAATTGTTTT

In100 candidate88 (in intron):

LOCUS ENSG00000134769

DESCRIPTION Dystrobrevin alpha (Dystrobrevin-alpha) (Dystrophin-related

proein 3). [Source:Uniprot/SWISSPROT;Acc:Q9Y4J8]

TTGGTTGTAAATCATGTTATTTCATAAAGAAAAAGATAAGATCTTAGGTAACGTGAGTTCCTGGCACTTCTGTTCAGGGT

TTATCTCATTCCCTACAATT

In100 candidate89 (in intron):

LOCUS ENSG00000118267

DESCRIPTION zinc finger protein 271 [Source:RefSeq_peptide;Acc:NP_006620]

AAAAACCTTTCTTGGTTTTTATTTAACTGAAAATATCTTTATTTTAACTTTTCTTTCAGGCATTTTATCTTCTTATGCAA

GTCTATGTTGGCACTTCTTT

In100 candidate90 (in intron):

LOCUS ENSG00000186496

DESCRIPTION Zinc finger protein 396 (Zinc finger and SCAN

domaincontainingprotein

14). [Source:Uniprot/SWISSPROT;Acc:Q96N95]

ATCTTCCCTTTATTGGCCTAACTTATATTTTCTTACTTTATTTTCCACATTCCTTTTTGGCCCCTTGGCTTTCTTCAGCT

CATTTCTTTGCATAGTTACT

In100 candidate91 (in intron):

LOCUS ENSG00000205726

DESCRIPTION Intersectin-1 (SH3 domain-containing protein 1A) (SH3P17).

[Souce:Uniprot/SWISSPROT;Acc:Q15811]

CTAAATACACTTATACTTTCCTTTTCTTTTAACATACAATCTGATTTAGTTCTTTCTCTAGAATGTCTTGCTCAGATTAA

TTTGTCTTTTGTGGGTTTCT

In100 candidate92 (in intron):

LOCUS ENSG00000205726

DESCRIPTION Intersectin-1 (SH3 domain-containing protein 1A) (SH3P17).

[Souce:Uniprot/SWISSPROT;Acc:Q15811]

GTGTTACTCTTATAACAATTTTTAACTCATTCTTTTCCTTTTCTTCTCCTGACAGACATTTTTTTAAATGTAGCCAACAA

TACCTTTTTCCTCTACCTTC

In100 candidate93 (in intron):

LOCUS ENSG00000075643

DESCRIPTION Molybdenum cofactor sulfurase (EC 4.4.-.-) (MoCo sulfurase) (MO)

(HMCS). [Source:Uniprot/SWISSPROT;Acc:Q96EN8]

AGCTTTTGATACCTATACCCACCTAACTGGTGGTTTTAATTTTTTCCATCAGCAGACTCTGTCAAATTTAATTTTACCTT

TTATGGCTTTTTTTTTTTCT

In100 candidate94 (in intron):

LOCUS ENSG00000134775

DESCRIPTION formin homology 2 domain containing 3

[Source:RefSeq_peptide;Ac:NP_079411]

AGGCTGGCTTTTAACTGCTTTAGATCTATGTATTAATTTCTCCTACTCAGCAGCCCAAGCCTCTGCGGACTTTTTTTTTT

TTTTTGCTTTTCCAGTGCGC

In100 candidate95 (in intron):

LOCUS ENSG00000134775

DESCRIPTION formin homology 2 domain containing 3

[Source:RefSeq_peptide;Ac:NP_079411]

TATGAATTGCACTGCTTTTCTGTTTTGTAACTCAATGTTTTGCTATTATCTTTATAAATCCCTTTTACTTTCTTTCAGTT

TACCTTGTTATTTTTCTGGA

In100 candidate96 (in intron):

LOCUS ENSG00000150477

DESCRIPTION KIAA1328 (KIAA1328), mRNA [Source:RefSeq_dna;Acc:NM_020776]

TAAATATACGTGTGCATCATTCACCCACTTTCTAACATTCAAAGTTTTGTTGGTATCTTTTCCAGTTTCTCGGTCCATCC

CTGCATGTCTGTACTTTTTT

In100 candidate97 (in intron):

LOCUS ENSG00000092203

DESCRIPTION Epidermal Langerhans cell protein LCP1.

[Source:Uniprot/SWISSPRT;Acc:O94842]

AGTTAGAAGGTAACTGCATATCATGTTCCTGTTTTCTCTTTCTGTGTCCAAACCACTTCTTTTTAATATTTGCTTCAGTT

TTAGTTTTGTTTTATATTTA

In100 candidate98 (in intron):

LOCUS ENSG00000078142

DESCRIPTION Phosphatidylinositol 3-kinase catalytic subunit type 3 (EC 2.7..137) (PtdIns-3-kinase type 3) (PI3-kinase type 3) (PI3K type 3) (Phosphoinsitide-3-kinase class 3) (Phosphatidylinositol 3-kinase p100 subunit). [Souce:Uniprot/SWISSPROT;Acc:Q8NEB9]

AACTAACTCTTCTCATGGTTTAGGTGATACTGTATATCTCTTGTTTTTCTCTTCATCTGTCTTGCCATTTCTTCTCAGTT
TCATTTGTCAGCACTTTCCT

In100 candidate99 (in intron):

LOCUS ENSG00000180509

DESCRIPTION Potassium voltage-gated channel subfamily E member 1 (IKs produing slow voltage-gated potassium channel subunit beta Mink) (Minimal potassum channel) (Delayed rectifier potassium channel subunit IsK). [Source:Unipot/SWISSPROT;Acc:P15382]

TATCGATATCTTACATTTGTTACAATGAAACAAATAATGACCTGTTATTAACAAAAGTTCATAATTTTGTTCTTTCAGTC
TTTACTTAATGTCTTTTCCT

In100 candidate100 (in intron):

LOCUS ENSG00000152217

DESCRIPTION SET-binding protein (SEB). [Source:Uniprot/SWISSPROT;Acc:Q9Y6X0

ACTTTGATTATGTTCTGTTGTCCTGGTAACTTCATATGAATTTCCATGGTCTGTTAAACTGTTCTTTCTTTATTTCAGGC

ATTTGCTTTTTCTTCTGATC

In100 candidate101 (in intron):

LOCUS ENSG00000152217

DESCRIPTION SET-binding protein (SEB). [Source:Uniprot/SWISSPROT;Acc:Q9Y6X0

ATATCCTCATTTTTCCTCCTATGATATCTTTCTTCTTTGATAACCACCATTATTTGTGTTTCTGTTTATCTTCTCCAGTT

TGTCAATGTCCTTCTTACTC

In100 candidate102 (in intron):

LOCUS ENSG00000159216

DESCRIPTION Runt-related transcription factor 1 (Core-binding factor, alpha2 subunit) (CBF-alpha 2) (Acute myeloid leukemia 1 protein) (Oncogene AML-1 (Polyomavirus enhancer-binding protein 2 alpha B subunit) (PEBP2-alpha B) PEA2-alpha B) (SL3-3 enhancer factor 1 [Source:Uniprot/SWISSPROT;Acc:Q01196

AAAAGAATTATCATTCTTTAGAGTGATTATAAGGAAGAATTAATATTAACAAAATTCTCAATGTTTGGCCTTTCCCAGGA

TTCTCCTTATCAATCTATTA

In100 candidate103 (in intron):

LOCUS ENSG00000159256

DESCRIPTION MORC family CW-type zinc finger protein 3 (Zinc finger CW-type oiled- coil domain protein 3). [Source:Uniprot/SWISSPROT;Acc:Q14149]

AGCCATATTTTAGTGCTAACCGGGACAAAAGCCATAGTGTTTTTCCCAGTGTTGACTACTCTGCCTGGCCTCTCTCTTCT

GTCTTAATACTTACTGTGTT

In100 candidate104 (in intron):

LOCUS ENSG00000159267

DESCRIPTION Biotin--protein ligase (EC 6.3.4.-) (Biotin apo-protein ligase)[Includes: Biotin--[methylmalonyl-CoA-carboxytransferase] ligase (EC 6.3.4.); Biotin--[propionyl-CoA-carboxylase [ATP-hydrolyzing]] ligase (EC 6.3.4.1) (Holocarboxylase synthetase) (HCS); B

[Source:Uniprot/SWISSPROT;Acc:P5074]

TTTCCATGTATAACAAGAATGTTGTTATTGTCTTCACAGGGGATTTTATTATTGCCATTCTAGAATGGACCTTTTATTTC

TTCGCCATCTCCATTTTCTC

In100 candidate105 (in intron):

LOCUS ENSG00000134049

DESCRIPTION Immediate early response 3-interacting protein 1.

[Source:Uniprt/SWISSPROT;Acc:Q9Y5U9]

CTCTTAAGGAATATCTCCATGCAATTGCCCATAATCTCTCCCTGTAATAACACATTATCACATGTTGATTTTTTTCAGCT

TTCTTCTTTTTCTTTTTCAG

In100 candidate106 (in intron):

LOCUS ENSG00000141627

DESCRIPTION Dymeclin (Dyggve-Melchior-Clausen syndrome protein).

[Source:Unprot/SWISSPROT;Acc:Q7RTS9]

TTATTTGTCCTCTTGTTCTTTATTATGGGAGTTCCCTTAACTTTTATCTTTTCTCCGTTCTTTTGTTTTAAATTTCAGGT

GTCCTTTCTTGTTCTTTATT

In100 candidate107 (in intron):

LOCUS ENSG00000141627

DESCRIPTION Dymeclin (Dyggve-Melchior-Clausen syndrome protein).

[Source:Unprot/SWISSPROT;Acc:Q7RTS9]

TTGCACAATACCTTATAATAACAAAGCATTCCTAATTCTTTCTGCCTGTCTGTTGCATTTTCAGTTCACATATACATTGC

TGTCCTTCATTTCACTTCTT

In100 candidate108 (in intron):

LOCUS ENSG00000141627

DESCRIPTION Dymeclin (Dyggve-Melchior-Clausen syndrome protein).

[Source:Unprot/SWISSPROT;Acc:Q7RTS9]

TTATAACCAATACTGTTATTATTCTGTTGCTCACATTGTTCCAGCTTTGGCCATTGGGAGCTCTTTCATGTTGTGTCCTC

TGCCCTCTCAGTTTACTCTT

In100 candidate109 (in intron):

LOCUS ENSG00000141627

DESCRIPTION Dymeclin (Dyggve-Melchior-Clausen syndrome protein).

[Source:Unprot/SWISSPROT;Acc:Q7RTS9]

CATCTCATTCAATACATATTCGTGGGGTAAATTAACTTCACCCTGCCTCTGGATTCCTATTTCTCTCCTTCTTCTCAGCC

AAAATCTTGATTTCTCCTTT

In100 candidate110 (in intron):

LOCUS ENSG00000198412

DESCRIPTION

TAATAATAGTAACTGGGGAAATTATTCATTCTATTTGCCAAGGATTTCATTTCTCCCTCCTTCAGGCACATAGCAGAATT

TTTTTTTTTTACTTGCTTCT

In100 candidate111 (in intron):

LOCUS ENSG00000100122

DESCRIPTION Beta crystallin B1. [Source:Uniprot/SWISSPROT;Acc:P53674]

AGCCAAGAAAACCAATTTTTTGAAATAACACTTCCTCTCTTTCATTTGCTTCATTGTTAACCAGTCCCTAAGTCCTATTC

TTGCTTCCTTGGATTTGTTC

In100 candidate112 (in intron):

LOCUS ENSG00000157554

DESCRIPTION Transcriptional regulator ERG (Transforming protein ERG).

[Soure:Uniprot/SWISSPROT;Acc:P11308]

CACTGTTCACTTGGACATCTCATGCTAGCCTCCACACTCCTTAACATCTCTGTTTTTCCTTCTTTCTAGGTAGTTCAGTA

ATTTCTCTTACCATTTTTGA

In100 candidate113 (in intron):

LOCUS ENSG00000100154

DESCRIPTION Tetratricopeptide repeat protein 28 (TPR repeat protein 28).

[Surce:Uniprot/SWISSPROT;Acc:Q96AY4]

TAATCTAATATAACCACCTAGCCATCTTTAGTCACATCATTCTATTTCAATTTCCTCTTAAGCTCTTTTTACTATCAGAT

TGTTTTTGTTTTTTGGTCAT

In100 candidate114 (in intron):

LOCUS ENSG00000100154

DESCRIPTION Tetratricopeptide repeat protein 28 (TPR repeat protein 28).

[Surce:Uniprot/SWISSPROT;Acc:Q96AY4]

GCATAGTGCCTGAACTATTAATAATTTACATACCCATTAACTATGTTAATCATTCCCTTTTCTCTGCAGCCTCGTCAACG

TCTGTTATGTTTTGACTTTT

In100 candidate115 (in intron):

LOCUS ENSG00000100154

DESCRIPTION Tetratricopeptide repeat protein 28 (TPR repeat protein 28).

[Surce:Uniprot/SWISSPROT;Acc:Q96AY4]

ATATATTTAAAAACACTTGAACATAGTTACAATAACTTTTAATTTTCTTGTCTGCTAATTTTATCATCTCAGTCATTTTG

GCAATCTTTATTAATTTTTC

In100 candidate116 (in intron):

LOCUS ENSG00000100154

DESCRIPTION Tetratricopeptide repeat protein 28 (TPR repeat protein 28).

[Surce:Uniprot/SWISSPROT;Acc:Q96AY4]

ATAGAGACGCATTGAATCTTTACTTTCTTTAACTCTTACATTAATCTCTTTCTCTTTACCATCAGCCTGTACATATGTTC

AAGTCTCTCGTATTTTTTTT

In100 candidate117 (in intron):

LOCUS ENSG00000183765

DESCRIPTION Serine/threonine-protein kinase Chk2 (EC 2.7.11.1) (Cds1).

[Souce:Uniprot/SWISSPROT;Acc:O96017]

TTATGTATTGATAACATTTGTATGGTATCTATTTGTGTATTATTTTTTATTTTTATTTTTGTGTGTTATTTCACACAGTC

ATTTTAATTTATTTTTATTT

In100 candidate118 (in exon):

LOCUS ENSG00000185658

DESCRIPTION Bromodomain and WD repeat domain-containing protein 1 (WD repea

protein 9). [Source:Uniprot/SWISSPROT;Acc:Q9NSI6]

TAAAAACTGGTGATAGATGTCAAATCCATTAATTTATTTAAACTCTTCTTAACCTTCTCTTTTATAATCTGTTCTCAGGT

TTTAACTCCATGTTTACCAT

In100 candidate119 (in intron):

LOCUS ENSG00000157326

DESCRIPTION Dehydrogenase/reductase SDR family member 4 (EC 1.1.1.184)

(NADHdependent

carbonyl reductase/NADP-retinol dehydrogenase) (CR) (PHCR) (Peoxisomal

short-chain alcohol dehydrogenase) (NADPH-dependent retinol

dehydrgenase/reductase) (NDRD) (SCAD-SRL) (hu

[Source:Uniprot/SWISSPROT;Acc:Q9BTZ]

AGAGATTTGCTGCTAACCATTTAAGTTTGGCTTCTTTCTTTCTTTTTTTAGACAGACTCTTGCTCTGTCTTTTTTCCTTT

TTTTTTTTTTTCCCTCGCT

In100 candidate120 (in intron):

LOCUS ENSG00000187630

DESCRIPTION dehydrogenase/reductase (SDR family) member 4 like 2

[Source:ReSeq_peptide;Acc:NP_932349]

AGAGATTTGCTGCTAACCATTTAAGTTTGGCTTCTTTCTTTCTTTTTTTAGACAGACTCTTGCTCTGTCTTTTTTCCTTT

TTTTTTTTTTTCCCCCTCGC

In100 candidate121 (in intron):

LOCUS ENSG00000134042

DESCRIPTION Protein maestro (Male-specific transcription in the developing

eproductive organs) (Protein B29). [Source:Uniprot/SWISSPROT;Acc:Q9BYG7]

TTTAATTGAATGTTATCTATTAGCATAACAATAAGTGCCTCTGTCTTTGAAAATATAACTGTTCTTTTTTCCTTTCAGGA

CATATTTTCTCCCTTTATGA

In100 candidate122 (in intron):

LOCUS ENSG00000082212

DESCRIPTION NAD-dependent malic enzyme, mitochondrial precursor (EC 1.1.1.3)

(NAD-ME) (Malic enzyme 2). [Source:Uniprot/SWISSPROT;Acc:P23368]

AATTTTCTGATGGTAACAAATATCTCACTCATTTTTATTATCTATCTTTTGTTCTGTCTTGGTTATCAGGTTTGTTTGTA

AGGCTAATTTGTCCTTTTTT

In100 candidate123 (in intron):

LOCUS ENSG00000082212

DESCRIPTION NAD-dependent malic enzyme, mitochondrial precursor (EC 1.1.1.3)

(NAD-ME) (Malic enzyme 2). [Source:Uniprot/SWISSPROT;Acc:P23368]

CTGTTTTAAATTTTATAATGTGTATAATGTAACATTTAGTACTTTATCTTTCCCATCATTATGCTTTTTCAGTGTTTTCT

TGACTGTTCTAAATTGTTTT

In100 candidate124 (in intron):

LOCUS ENSG00000187323

DESCRIPTION Netrin receptor DCC precursor (Tumor suppressor protein DCC) (Clorectal cancer suppressor). [Source:Uniprot/SWISSPROT;Acc:P43146]

ATGACATTACCTGATATTTAGAATATAAACTATGATTTGTATGGCTAACATTTATTTATACTTTTTTTCTTTTCCAGGC
ACTGCTTTTTTTTTTCCTTT

In100 candidate125 (in intron):

LOCUS ENSG00000187323

DESCRIPTION Netrin receptor DCC precursor (Tumor suppressor protein DCC) (Clorectal cancer suppressor). [Source:Uniprot/SWISSPROT;Acc:P43146]

CATCGTGGCCTTTTATACCTTTATTTTAAGTGTTATTAATTAACCATCAAGGTTTTTCATAGCTTTCTGCAGGTTCATTT
CTAGTACATCATATTTATTT

In100 candidate126 (in intron):

LOCUS ENSG00000187323

DESCRIPTION Netrin receptor DCC precursor (Tumor suppressor protein DCC) (Clorectal cancer suppressor). [Source:Uniprot/SWISSPROT;Acc:P43146]

ATTGATTCTGCTGGGTAAATAAGGGTGTCCTTAAGTTTAACCCCATTGTCTGTACTCACTTCTTCTTTTGATTGTCAGTT
TTCTATATTCTTACTTTCCT

In100 candidate127 (in intron):

LOCUS ENSG00000187323

DESCRIPTION Netrin receptor DCC precursor (Tumor suppressor protein DCC) (Clorectal cancer suppressor). [Source:Uniprot/SWISSPROT;Acc:P43146]

GAGAAGATATTCATAAACATTTTATAACCTCTTACAATTTTTCCTATTTTCTTTCTTTCCCTTTTTATATTCATCCAGTT
TCATCTATTCTTTATTTCTT

In100 candidate128 (in intron):

LOCUS ENSG00000187323

DESCRIPTION Netrin receptor DCC precursor (Tumor suppressor protein DCC) (Clorectal cancer suppressor). [Source:Uniprot/SWISSPROT;Acc:P43146]

TGGTCCTTGATTCATTTCCTTAGGTCCACTAACTCCCTTTTCATTTAATTCTGTTGTTTTATCCTCCAGTCTTTGACCTC
TAGCTCTATTGAATTTATTT

In100 candidate129 (in intron):

LOCUS ENSG00000187323

DESCRIPTION Netrin receptor DCC precursor (Tumor suppressor protein DCC) (Clorectal cancer suppressor). [Source:Uniprot/SWISSPROT;Acc:P43146]

AGGACTCCTATTATCTGTACGTTAGATCTTCCTTCTCTAACTTCAATATTTATCACTTTCTCCTGAATCCTTTTTCAGTC
TTCTTAATTTCTTTTGTTAT

In100 candidate130 (in intron):

LOCUS ENSG00000187323

DESCRIPTION Netrin receptor DCC precursor (Tumor suppressor protein DCC) (Clorectal cancer suppressor). [Source:Uniprot/SWISSPROT;Acc:P43146]

CTGAAGACACATTTCACTTCTGCATAACTTTAATTCTATGTCTTGATCTTTTGTATCTTAAATCTCTATTCCCTTCAGAA
TTATCAGATTTTCTATTTCC

In100 candidate131 (in intron):

LOCUS ENSG00000166510

DESCRIPTION Coiled-coil domain-containing protein 68 (Cutaneous T-cell lympoma- associated antigen se57-1) (CTCL tumor antigen se57-1). [Source:Unipro/SWISSPROT;Acc:Q9H2F9]

GAAAATTTCTGCTTAACTCTACCGAAATTATTTACTTTCTTTCTGGTTTTGCATATCTTTCATCATCTTAATTTACAGCT
CTGACACTCATCTTATATTT

In100 candidate132 (in intron):

LOCUS ENSG00000196628

DESCRIPTION Transcription factor 4 (Immunoglobulin transcription factor 2) ITF-2) (SL3-3 enhancer factor 2) (SEF-2). [Source:Uniprot/SWISSPROT;Acc:P1584]

CTTCTTCTCCCTACTAAATCTTATCTAGAGTTTAACTATCTATTAATCATTCATTTTTGCACATTTTTACAGCTTTTGTG
GGGTATTTTACATTCTTTCT

In100 candidate133 (in intron):

LOCUS ENSG00000196628

DESCRIPTION Transcription factor 4 (Immunoglobulin transcription factor 2) ITF-2) (SL3-3 enhancer factor 2) (SEF-2). [Source:Uniprot/SWISSPROT;Acc:P1584]

TATGTTCTAATTTTTCTAGCTCTAATGTCATAGCTAACAAAGGCCATCTCTACTTAATTGCTTGTACTTTATTCTCAGGC
TTTTATGTTTTAACCTTTGC

In100 candidate134 (in intron):

LOCUS ENSG00000196628

DESCRIPTION Transcription factor 4 (Immunoglobulin transcription factor 2)

ITF-2) (SL3-3 enhancer factor 2) (SEF-2). [Source:Uniprot/SWISSPROT;Acc:P1584]

ACTTTTAAAATTTAATTGATTGAAATAGTTTTATTTGCTAACATAGCAAAATGTTCTCCTTTTTTCCAGATCACTGACAA

TTTCTGCATTTTCTATGTTT

In100 candidate135 (in intron):

LOCUS ENSG00000196628

DESCRIPTION Transcription factor 4 (Immunoglobulin transcription factor 2)

ITF-2) (SL3-3 enhancer factor 2) (SEF-2). [Source:Uniprot/SWISSPROT;Acc:P1584]

ATTATATTTTAAATATGCCTGAAGTCGTGAACTAACTAATGTTGATCATTTAGTCTTCTGGTCCTATCTCAGGAATATTA

TTGCCCTTCTTAATTCATCT

In100 candidate136 (in intron):

LOCUS ENSG00000091157

DESCRIPTION WD repeat protein 7 (TGF-beta resistance-associated protein TRA)

(Rabconnectin-3 beta). [Source:Uniprot/SWISSPROT;Acc:Q9Y4E6]

TGGACTTGTTAATTCAATGTTTGTCAATAATTAACATTTATTGTCACTTGTCTACTTTTTTTATACAGTTTATTTTTTT

TGACACTTGATTTTGCGGTT

In100 candidate137 (in intron):

LOCUS ENSG00000091157

DESCRIPTION WD repeat protein 7 (TGF-beta resistance-associated protein TRA)

(Rabconnectin-3 beta). [Source:Uniprot/SWISSPROT;Acc:Q9Y4E6]

TTCTATATGACTTTTCTTTTAACTGTAATAATTTTATTTTTTAAATTTTTATCTGTTTACAGTTTTTTTAAAAGTCCATT

TAATCTCTCAGTCTTGTTTT

In100 candidate138 (in intron):

LOCUS ENSG00000183067

DESCRIPTION IGSF5 protein (Fragment). [Source:Uniprot/SPTREMBL;Acc:Q9NSI5]

EXON

TTGTATGATTGAGAACAATTTTTATATGTTTTTATAACTTGTCATTCTATATCTTTTGCTCATTATTTTCTCTCTCAGAT

TTTTGGGCTTTTTACTCCTT

In100 candidate139 (in intron):

LOCUS ENSG00000171587

DESCRIPTION Down syndrome cell adhesion molecule precursor (CHD2).

[Source:niprot/SWISSPROT;Acc:O60469]

AGTTTGTTGGTGTATAACTGTTCATATTAGCTTTTTATAATCCTTCGTATTTCTGTTGTATCAGGTGTAATGTTTCTCCT

TTTATTTCTGATTTTGTCTT

In100 candidate140 (in intron):

LOCUS ENSG00000171587

DESCRIPTION Down syndrome cell adhesion molecule precursor (CHD2).

[Source:niprot/SWISSPROT;Acc:O60469]

CGTTTTTATTAACTTGTTCCTTTTGCTTTCCTCATGCATATTTTATTGGCTTTTTTCTAGTTCTTTCAGTTGGATGCTTA

TGTCATTTATTTTCATCCTT

In100 candidate141 (in intron):

LOCUS ENSG00000176635

DESCRIPTION HORMA domain containing 2

[Source:RefSeq_peptide;Acc:NP_689723]EXON

AATGAGATATGGCATAACTCTAATTTTCATTTTTGCCAACTAAATTCTCCCTCTTTTCGTGTTTTTATAGATTTTCAGAG

CTTTACCTCTTTACTTCTCT

In100 candidate142 (in intron):

LOCUS ENSG00000176635

DESCRIPTION HORMA domain containing 2

[Source:RefSeq_peptide;Acc:NP_689723]EXON

TCCTGAGCCCTCTATCCTTTTAATGTTTTCCCTCTAACTTCACTGCGGCTTTTGGTCTTGCTCAGATCATTCATTTTCTG

AAGTAGCCTCATTGCTTCTC

In100 candidate143 (in exon):

LOCUS ENSG00000170113

DESCRIPTION Non-imprinted in Prader-Willi/Angelman syndrome region protein .

[Source:Uniprot/SWISSPROT;Acc:Q7RTP0]

TAAAAATCTTAAATCCTCTCAATGGATGGTTAACAAATGCTCAAAGTCCATTACTCTTTTTATTGGCTCTTGCAGGTTTT

GTGTTTTATCATCAGTGCTT

In100 candidate144 (in intron):

LOCUS ENSG00000166562

DESCRIPTION Microsomal signal peptidase 21 kDa subunit (EC 3.4.-.-) (SPase 1

kDa subunit) (SPC21) (SEC11-like 3). [Source:Uniprot/SWISSPROT;Acc:Q9BY50

TATTTATTTTCCTATTTTGATTTATTGCACCAAAATACATTAACAATTATCTTAATTCATTTATGTCCTCACTTTCAGTT

GTATCCTTCATTATTGCCTC

In100 candidate145 (in intron):

LOCUS ENSG00000141956

DESCRIPTION PR domain zinc finger protein 15 (PR domain-containing protein 5) (Zinc finger protein 298). [Source:Uniprot/SWISSPROT;Acc:P57071]

TATTATTTGCTTTTTATACTTTTCATTCTGTGCCTTTCTAACAAGTTGGATGCTTAACTCATTACTTTCCAGCTGTTTTA

TTTTCTCATGTAAGCATTTC

In100 candidate146 (in intron):

LOCUS ENSG00000074695

DESCRIPTION ERGIC-53 protein precursor (ER-Golgi intermediate compartment 5 kDa protein) (Lectin, mannose-binding 1) (Gp58) (Intracellular mannose- spcific lectin MR60). [Source:Uniprot/SWISSPROT;Acc:P49257]

ACTAACTTAGGTTTGGATCTTGTTTCTGCCAGTTACTGCTATATGGTCTTGTGCAAGTCACTGAACCTCTGTAATGCTTC

ATTTCTTTTTTTTTTTTTT

In100 candidate147 (in intron):

LOCUS ENSG00000168952

DESCRIPTION Syntaxin-binding protein 6 (Amisyn).

[Source:Uniprot/SWISSPROT;cc:Q8NFX7]

TTGTCTTTTCAAGGCCACTTAGTGCTTAACTCAAGTACCCACTTTCCATCTCTTTAGTTGTCTGTTACCCTTCTACAGAA

TCCTCTTTAGTTTCTGCATT

In100 candidate148 (in intron):

LOCUS ENSG00000168952

DESCRIPTION Syntaxin-binding protein 6 (Amisyn).

[Source:Uniprot/SWISSPROT;cc:Q8NFX7]

AGAGAAATGAGGAAGAAAATGAGTAACAACTCTCAACATTTAGCTTTTTTTTACACATCCAGATATTGCTTTTAATTACT

TTTTTTTCTTATGCTTTAAG

In100 candidate149 (in intron):

LOCUS ENSG00000168952

DESCRIPTION Syntaxin-binding protein 6 (Amisyn).

[Source:Uniprot/SWISSPROT;cc:Q8NFX7]

CTTCTCCCATTTCACATAACATGCAAGTGACTTGTCTTTCTGTCTTTCTTCATTGGTCTTTGTTCCCTCTTCTACCAGTT

CTACTTTCCTGACTTGTTGC

In100 candidate150 (in intron):

LOCUS ENSG00000168952

DESCRIPTION Syntaxin-binding protein 6 (Amisyn).

[Source:Uniprot/SWISSPROT;cc:Q8NFX7]

CACTCTTATAGCTGCCTCTTTGATATATTGTCCTGACTTAACTTCCATTGTGCCAAATTCACCTTGCTTTTTCAGTTTCC

TTAGCAGGTTCCTTTACCTT

In100 candidate151 (in intron):

LOCUS ENSG00000101542

DESCRIPTION Cadherin-20 precursor. [Source:Uniprot/SWISSPROT;Acc:Q9HBT6]

CTCTTAGGGAGTCATAACTTAAATGATTCTGATTTCTCTAGCTCTTACTCATCAGCTTTCTCTTTAATTCCTAATTCCTA

AATAGCCATCCTGAATCTCT

In100 candidate152 (in intron):

LOCUS ENSG00000184304

DESCRIPTION Serine/threonine-protein kinase D1 (EC 2.7.11.13) (nPKC-D1)

(Prtein kinase D) (Protein kinase C mu type) (nPKC-mu).

[Source:Uniprot/SWISSPOT;Acc:Q15139]

TAAAAACATTTACCTTTTCCTCATTTCCTGTCAATTCATAACCTTAGTTACTTTAACTTTTTACATTTTACATCTCAGTT

ACTCTGCTCTGAATTTCTTA

In100 candidate153 (in intron):

LOCUS ENSG00000184304

DESCRIPTION Serine/threonine-protein kinase D1 (EC 2.7.11.13) (nPKC-D1)

(Prtein kinase D) (Protein kinase C mu type) (nPKC-mu).

[Source:Uniprot/SWISSPOT;Acc:Q15139]

TCTTCTCTAAAGTTAATGATTCTTAACTTTTGGTTAAACTTTTTTAGTTGCTGTCATAGCCTTCATTTTGTTCAGTACCT

GTGTCTATTATTTTTAATTT

In100 candidate154 (in intron):

LOCUS ENSG00000197563

DESCRIPTION GPI ethanolamine phosphate transferase 1 (EC 2.-.-.-) (Phosphatdylinositol-glycan biosynthesis class N protein) (PIG-N) (MCD4 homolog). [Surce:Uniprot/SWISSPROT;Acc:O95427]

TAGGATGTGCAAGTCATTGTTCTAAGCTAACTCTTGTTTATCTTTCATTTGCTTTTATTGTATTGGATTTTTCCTCAGTT
ATCTCTGTTTCTTCAGTTGA

In100 candidate155 (in intron):

LOCUS ENSG00000092108

DESCRIPTION Sec1 family domain-containing protein 1 (Syntaxin-binding proten 1- like 2) (Sly1p). [Source:Uniprot/SWISSPROT;Acc:Q8WVM8]

CATGACGTGTTAATTTGGATGGGAATATTTAATTGATATTTGATATAACATGACTTGTCTTCCATTGATTTTCAGTCATG
TAAATATTTTACTTTTTCTT

In100 candidate156 (in intron):

LOCUS ENSG00000114062

DESCRIPTION Ubiquitin-protein ligase E3A (EC 6.3.2.-) (E6AP ubiquitin-proten ligase) (Oncogenic protein-associated protein E6-AP) (Human papillomaviru E6-associated protein) (Renal carcinoma antigen NY-REN- 54). [Source:Uniprt/SWISSPROT;Acc:Q05086]

AGACTCTTCCCATAATAACTTTATTTAGACTTCCGAATTCTGTTTTTCTTGTTTATGTTTCTATAACAGATGTTACTTTT
TTCTGGACCTGTTATTACTA

In100 candidate157 (in intron):

LOCUS ENSG00000184792

DESCRIPTION Oxysterol-binding protein 2 (Oxysterol-binding protein-related rotein 4) (OSBP-related protein 4) (ORP-4). [Source:Uniprot/SWISSPROT;Acc:Q69R2]

GCTGTGTCCTGTTTTACACATGATGGATGTAACCCTACAATACACTTTGTTTTCTCTTTGTTTTCTTGCTTTATACAGTT

ATCTTTTTTTTTTTTTTTT

In100 candidate158 (in intron):

LOCUS ENSG00000196792

DESCRIPTION Striatin-3 (Cell-cycle autoantigen SG2NA) (S/G2 antigen). [Soure:Uniprot/SWISSPROT;Acc:Q13033]

TTGAACATACTGACATATTCTTTGAAAAGAAATTTAGAACTCATATCTAACAATCCCTCCCCTTGTGTTTCCTTACAGCT

TTCTTTTCAAACTTTTTTTT

In100 candidate159 (in intron):

LOCUS ENSG00000081913

DESCRIPTION PH domain leucine-rich repeat-containing protein phosphatase (E 3.1.3.16) (PH domain leucine-rich repeat protein phosphatase) (Pleckstrin omology domain-containing family E protein 1) (Suprachiasmatic nucleus circdian oscillatory protein) (hSCOP). [Source:Uniprot/SWISSPROT;Acc:O60346]

GAAAGTATGTATTTCTAGAGACTTTGAGATAACATTTAACATTCTGGCTATTTAACTTCTTCCTTTTCTTCCTTTCAGTT

TCTCTCTTTCCAGTTTCTGC

In100 candidate160 (in intron):

LOCUS ENSG00000206187

DESCRIPTION CDNA FLJ42943 fis, clone BRSTN2003835.

[Source:Uniprot/SPTREMBLAcc:Q6ZV66]

AGAAATACGATCTATTTCGGGATCGCTTGATGCCCGATAACATTCCCTAGATTTGCTGTTACCCAGGGTTTTTTATTTT

TATTTTTATTTTTCCCATAT

In100 candidate161 (in intron):

LOCUS ENSG00000166206

DESCRIPTION Gamma-aminobutyric-acid receptor subunit beta-3 precursor (GABAA)

receptor subunit beta-3). [Source:Uniprot/SWISSPROT;Acc:P28472]

TTAATGCCTAATTTAAGTAATACTTTTAATATTAATTTAATAACAAATTAAACATTATTTTTTTGTCAGGCATATACTTT

ATATCACATTTTTGTGTTTC

In100 candidate162 (in intron):

LOCUS ENSG00000092148

DESCRIPTION E3 ubiquitin-protein ligase HECTD1 (HECT domain-containing protin

1) (E3 ligase for inhibin receptor) (EULIR).

[Source:Uniprot/SWISSPROT;Ac:Q9ULT8]

TCTGTTTACCCTGTTGGTTATGATAACTTGGATCACTTGCTTAAGTTTTTCTCTTCCAGGTTTCTCTACTGTAAAGTTGT

AAAGTTGCTCTTTTCCTTTT

In100 candidate163 (in intron):

LOCUS ENSG00000182256

DESCRIPTION Gamma-aminobutyric-acid receptor subunit gamma-3 precursor

(GAB(A) receptor subunit gamma-3). [Source:Uniprot/SWISSPROT;Acc:Q99928]

CTTCCATTCTGTAGGCTGTGTCTTAACTCTGCTAATTGTTTCCTTTGTTGTACAGAATCTTTTTAGTTTGATGTAATCCA

ATTTGTCTATTTTTGCTTTT

In100 candidate164 (in intron):

LOCUS ENSG00000182256

DESCRIPTION Gamma-aminobutyric-acid receptor subunit gamma-3 precursor

(GAB(A) receptor subunit gamma-3). [Source:Uniprot/SWISSPROT;Acc:Q99928]

CATTAACATATTAAATAATTATTTAAATACCCTCTTTCCTATCTGGTTCTGTTCTTAGTAATTATTTTGCCTTTTCAGGC

TGCTATTTTTGGTTTTTGTA

In100 candidate165 (in intron):

LOCUS ENSG00000151413

DESCRIPTION Nucleotide-binding protein-like.

[Source:Uniprot/SWISSPROT;Acc:8TB37]

GGCATTTTGGTCACTATAACAAGTGTAATTTTGTGTTTAGCCCTTGTTATAGATATCTTTTTCTTATCTCAGTATGTCAT

TCATATTTTTCTAACTTGCT

In100 candidate166 (in intron):

LOCUS ENSG00000151413

DESCRIPTION Nucleotide-binding protein-like.

[Source:Uniprot/SWISSPROT;Acc:8TB37]

AGATAACAATTTTTTTCCTGATTTCTTTTTATTATCTGTCTTTATTCTTTTGTATTTCAGTAAATTTTCTTACAATCATT

ATTATTATTTTTTTTTTTTT

In100 candidate167 (in intron):

LOCUS ENSG00000151413

DESCRIPTION Nucleotide-binding protein-like.

[Source:Uniprot/SWISSPROT;Acc:8TB37]

CTTAGCAAGTTGCAAAGTGCTGAAGTTGATAACATGGGAACTAGGTTGTTTTATGTGTTCATTTCTTTCTCCAGTTCTTA

ATCCTATTTCTCACCGATAT

In100 candidate168 (in intron):

LOCUS ENSG00000104044

DESCRIPTION P protein (Melanocyte-specific transporter protein) (Pink-eyed

ilution protein homolog). [Source:Uniprot/SWISSPROT;Acc:Q04671]

AGTTTTGGTATCATTGCTTTTCTCTAACATTTTTTCCATTTTCTATTTCATTGATTTCTACTCCAGTCTTTATTATTTCT

TTTCTTCTACTTATTTTGGG

In100 candidate169 (in intron):

LOCUS ENSG00000104044

DESCRIPTION P protein (Melanocyte-specific transporter protein) (Pink-eyed

ilution protein homolog). [Source:Uniprot/SWISSPROT;Acc:Q04671]

AGCCTGGAATTCTTGTTTAAAAAATAGCACCTTTATAACTTTCATCACATTTCTTTTTCTCACAGAAAACCTTTTTTATC

CTTTGCCACTTTTTATTTTA

In100 candidate170 (in intron):

LOCUS ENSG00000100852

DESCRIPTION Rho GTPase-activating protein 5 (p190-B).

[Source:Uniprot/SWISSROT;Acc:Q13017]

TTTTTTATTTTTAACTTCTTTTTAAATTGTTTTCCTGTCTTATTCCAGGCCCTGAATATTTTCCTCCTCTTTATATTAGC

AATTACCAACTTGTATTCCT

In100 candidate171 (in intron):

LOCUS ENSG00000151320

DESCRIPTION A-kinase anchor protein 6 (Protein kinase A-anchoring protein 6 (PRKA6) (A-kinase anchor protein 100 kDa) (AKAP 100) (mAKAP). [Source:Unipot/SWISSPROT;Acc:Q13023]

GCATCTTTTCATATGTTTAACGATCATTTGTGTTACTTGTTCTATGAACTGTGCTCATATCCTTTCCCTATTTTTCAGTT

GTGTTGTTGGTCTTTTCTTT

In100 candidate172 (in intron):

LOCUS ENSG00000151320

DESCRIPTION A-kinase anchor protein 6 (Protein kinase A-anchoring protein 6 (PRKA6) (A-kinase anchor protein 100 kDa) (AKAP 100) (mAKAP). [Source:Unipot/SWISSPROT;Acc:Q13023]

AGGTTGTATTAACTATTGATAACCCCTTTATATATTTAGTTCCCCATACCAGTTCTTTTTGTTCGGCCGTCTACTTTTGG

CCACACATTTTTTCCTCTTT

In100 candidate173 (in intron):

LOCUS ENSG00000151320

DESCRIPTION A-kinase anchor protein 6 (Protein kinase A-anchoring protein 6 (PRKA6) (A-kinase anchor protein 100 kDa) (AKAP 100) (mAKAP). [Source:Unipot/SWISSPROT;Acc:Q13023]

ATGATATGGCCTCTTTGTAACCAATGAGCACTCTCTTTTTCAAATACAGTATTACAGGGTTTCTTACTTGGCATCCTTAT
GTTAGCCTCTACTTCCTTCT

In100 candidate174 (in intron):

LOCUS ENSG00000128731

DESCRIPTION HECT domain and RCC1-like domain-containing protein 2. [Source:niprot/SWISSPROT;Acc:O95714]

CTAACCCATGAACATAGTGTTTCTCTTTATTTTTAGGTGTTTAGTTTCTCTCAGCAATGTTTTCTAGTCCCAGTATTGT
GTAGGTTTTTTTTTTTTTT

In100 candidate175 (in intron):

LOCUS ENSG00000128731

DESCRIPTION HECT domain and RCC1-like domain-containing protein 2. [Source:niprot/SWISSPROT;Acc:O95714]

AGTCATTATTGGTTTTGCCCATGAACCATAACATTGCAATGTTGCTCTTCCCTCATATTTTTAGGCAGGGATTCCTTCTG
TCTTTCCTTAGTATTTATCT

In100 candidate176 (in intron):

LOCUS ENSG00000151322

DESCRIPTION Neuronal PAS domain-containing protein 3 (Neuronal PAS3) (Membe of PAS protein 6) (Basic-helix-loop-helix-PAS protein MOP6). [Source:Uniprt/SWISSPROT;Acc:Q8IXF0]

AGAGACCTAACATAATATGTCCCTTTTGATTCTTGCACAGTTCCATGGTTTTCTCTTACCTTTCTCAAAGAAGTGGCTTT

CCTTCTACTTTTTATTGCTA

In100 candidate177 (in intron):

LOCUS ENSG00000151322

DESCRIPTION Neuronal PAS domain-containing protein 3 (Neuronal PAS3) (Membe

of PAS protein 6) (Basic-helix-loop-helix-PAS protein MOP6).

[Source:Uniprt/SWISSPROT;Acc:Q8IXF0]

AGTATTATAACAAGAATAAAAACTTTTTTCTCTTTCTGTTATTCCTGGATTGGGTATTTTCTAGCCTTTCAGCCTAACTT

AACTGTATCCTTGCCATTTT

In100 candidate178 (in intron):

LOCUS ENSG00000151322

DESCRIPTION Neuronal PAS domain-containing protein 3 (Neuronal PAS3) (Membe

of PAS protein 6) (Basic-helix-loop-helix-PAS protein MOP6).

[Source:Uniprt/SWISSPROT;Acc:Q8IXF0]

AGCCTAACTTAACTGTATCCTTGCCATTTTGAGTAAACCTTTTATTTATTTTATGTCCTCCCCAGTCTGTCCTTCTATCC

ATATCTTATACTTTCTTCAA

In100 candidate179 (in intron):

LOCUS ENSG00000151322

DESCRIPTION Neuronal PAS domain-containing protein 3 (Neuronal PAS3) (Membe

of PAS protein 6) (Basic-helix-loop-helix-PAS protein MOP6).

[Source:Uniprt/SWISSPROT;Acc:Q8IXF0]

AATAACTGGGCCCAATGAGTCTCAATTAGTAGCTGCATTTTCTTCTTTATTTCCTCTTTGACTCTATTTCCCCGACAGTC

TGCTTTGTTCTTTTTCTTCT

In100 candidate180 (in intron):

LOCUS ENSG00000129518

DESCRIPTION E2F-associated phosphoprotein (EAPP).

[Source:Uniprot/SWISSPROTAcc:Q56P03]

TTTGTCATAACCACCTCACTGTTTTGTTTTTGTTTTCTTGATTACTTCCTTCTTTTCTGGCACTAAAAGATGCTCCAGGT

TCATCTTGTGTTCCTTATTC

In100 candidate181 (in intron):

LOCUS ENSG00000198604

DESCRIPTION Bromodomain adjacent to zinc finger domain protein 1A (ATPutilzing

chromatin assembly and remodeling factor 1) (hACF1) (ATP-dependent

chrmatin remodelling protein) (Williams syndrome transcription factor-related

hromatin-remodeling factor 180) (WCRF18 [Source:Uniprot/SWISSPROT;Acc:Q9NRL]

GCTTTTTCTTATTTTAAAAACTTGTCGTGAGGACCTTGATATTTGTAACCATTGGACTGATCATTTTCTAAACCTCAGGA

TCTCTGTTACTCCACTTTGT

In100 candidate182 (in intron):

LOCUS ENSG00000179676

DESCRIPTION CR020_HUMAN Isoform 2 of Q7Z4B0 - Homo sapiens (Human)

[Source:niprot/Varsplic;Acc:Q7Z4B0-2]

AATTCCAAATGTTCATGGGGAAAATTGAAATTTTTGTTTCTTTTCATAACATTTCCCATGTTTCATAAATTTTTCCAGAT

ACTCATTGATCTCTTTTATA

In100 candidate183 (in intron):

LOCUS ENSG00000081138

DESCRIPTION Cadherin-7 precursor. [Source:Uniprot/SWISSPROT;Acc:Q9ULB5]

GTGACCAAAACAATATTCGAATGATTAACATCTTATAATATATGTATATTTTTCTCAGTGCTGCTCTCTTCACACTGTCT

ATGGCTTCCAATTTATGTTT

In100 candidate184 (in intron):

LOCUS ENSG00000100883

DESCRIPTION Signal recognition particle 54 kDa protein (SRP54).

[Source:Unirot/SWISSPROT;Acc:P61011]

AAAGCAAGAATTCCATTTTATGGAAGGTAGGTTACTGTTTTTTATTTTAACACTTATATCCCTCTTCTTGTCTGTCAGCT

TTTTTTTTTTTTTTTTTTG

In100 candidate185 (in intron):

LOCUS ENSG00000166479

DESCRIPTION Protein disulfide-isomerase TXNDC10 precursor (EC 5.3.4.1)

(Thiredoxin domain-containing protein 10) (Thioredoxin-related transmembrane

prtein 3). [Source:Uniprot/SWISSPROT;Acc:Q96JJ7]

AATGTAACCAAATAATTCTGTATTATTTTCTCTCCCCTGAGATGTGCCTCCCTCCCTTTTTAATTTTTCCAGGATAACTG

TCTTTATTTTATTGTTATTT

In100 candidate186 (in intron):

LOCUS ENSG00000092020

DESCRIPTION Serine/threonine-protein phosphatase 2A regulatory subunit B''

ubunit gamma (Protein phosphatase subunit G5PR) (Rhabdomyosarcoma antigen MRMS-

40.6A) (Rhabdomyosarcoma antigen Mu-RMS-40.6C).

[Source:Uniprot/SWISSROT;Acc:Q969Q6]

AAAGGAAGGTACCTGTACTTGTACTTTGTGTGCTTTCTTGACCCCTTAACTCTTAAACCCACTGCATTCTTTTAGCAGAT

ATTTATTGCATGCCTGTTTT

In100 candidate187 (in intron):

LOCUS ENSG00000150636

DESCRIPTION Coiled-coil domain-containing protein 102B.

[Source:Uniprot/SWISPROT;Acc:Q68D86]

GATTATAGTTGTGAGATAACAAAATGTTCTCCCTACCAAATGTTTTGCCTTTCCTTATTCTGATATCAGGGGCTTTATTT

CTGTTCATTAGACTGTTTTT

In100 candidate188 (in intron):

LOCUS ENSG00000100890

DESCRIPTION Uncharacterized protein KIAA0391.

[Source:Uniprot/SWISSPROT;AccO15091]

AGGTCTTCTAACCCTGTCATTCATTCCTTTTTCTACTATTCCATGTTGCTACAGCCTTCCTGTTTCCCTTTTTATCAACA

TTTTATTTAAAAATTTTTCA

In100 candidate189 (in intron):

LOCUS ENSG00000100890

DESCRIPTION Uncharacterized protein KIAA0391.

[Source:Uniprot/SWISSPROT;AccO15091]

CGTTTTTTAGTGGGTAACACTATAGAGATACTTTTAAAGTTTTTTCGTATATTATCTGTTTTATTGTCTTCTCAGCACTT

CTTAAACATCTTATTTATTT

In100 candidate190 (in intron):

LOCUS ENSG00000100890

DESCRIPTION Uncharacterized protein KIAA0391.

[Source:Uniprot/SWISSPROT;AccO15091]

AGTGACTAAGTCTGCTCAATTCTGCTTTACTAACTCACAAAGAGCTCTGTTCTTTTCATTTTTACTGCCAGTTTTCTCAT

TATCTCTTGCCTGAATTACT

In100 candidate191 (in intron):

LOCUS ENSG00000198089

DESCRIPTION spindle assembly associated Sfi1 homolog isoform b

[Source:RefSq_peptide;Acc:NP_055590]

TTTTTCTTTTTTTGCCCTAACATTACAACAAATATTTACCCTTTCTGTTGCCTTTTTTCTTTCTTCTCACATCTCCAGGT

TTCTATCTGTGATAATTTTC

In100 candidate192 (in intron):

LOCUS ENSG00000206052

DESCRIPTION Docking protein 6 (Downstream of tyrosine kinase 6).

[Source:Unprot/SWISSPROT;Acc:Q6PKX4]

CATTCTACCCTGAGAAGTTGTAGTAGAGATCTGAAGGTTACTGAATCTTAACAAGTTATAAATATTTTTTATTTACAGCC

TGTTTTCCCTGTAAAACTTT

In100 candidate193 (in intron):

LOCUS ENSG00000206052

DESCRIPTION Docking protein 6 (Downstream of tyrosine kinase 6).

[Source:Unprot/SWISSPROT;Acc:Q6PKX4]

TTCACTTTGGAAATGATGAAAACCTTAACCCCTGAAATAGCTCGGGTTTTATTTTGTCTTGTGTCTTCATTTGTACAGGT

TCTCTTAATTCTATACCGTG

In100 candidate194 (in intron):

LOCUS ENSG00000206052

DESCRIPTION Docking protein 6 (Downstream of tyrosine kinase 6).

[Source:Unprot/SWISSPROT;Acc:Q6PKX4]

GATAACATTGCATAGAGCTCTTAGGTTGTAATATTTTCTTACTTCCTCACAGTGCTATCACTAAGCCTTATATTGTATTC

ATTTCTTCTTACTATTCATT

In100 candidate195 (in intron):

LOCUS ENSG00000160207

DESCRIPTION Heat shock factor 2-binding protein.

[Source:Uniprot/SWISSPROT;cc:O75031]

CTGATTACTGTAGCGACATAGTAAGCCTTAACACTGGATAAAATGATTCTTCCCATTTCATTCCTCTTTTTCAGAGTTGC

CCTAACTATTCTAGCTTCTT

In100 candidate196 (in intron):

LOCUS ENSG00000160207

DESCRIPTION Heat shock factor 2-binding protein.

[Source:Uniprot/SWISSPROT;cc:O75031]

TATTATTGTGGTTTGGTGGTGATCTGTAGTGGTAACATTTAAGTTTTTTCTTTTTTTCTGTGTTTGCTCTACCAGTGGTT

TTTATATTTTCATATGTTTT

In100 candidate197 (in intron):

LOCUS ENSG00000150637

DESCRIPTION CD226 antigen precursor (DNAX accessory molecule 1) (DNAM-1).

[ource:Uniprot/SWISSPROT;Acc:Q15762]

GGCTTTGTCGGGTTGTGTTTTTATCTCTTTATTGTAACTCACTCTTCCCACTTTATCTGTGCCTTTCAGTTAAGCGGTCT

TTTAGCTTATTTTTTATCTT

In100 candidate198 (in intron):

LOCUS ENSG00000150637

DESCRIPTION CD226 antigen precursor (DNAX accessory molecule 1) (DNAM-1).

[ource:Uniprot/SWISSPROT;Acc:Q15762]

CTCACACTTTCCTACTTTCTCCTTTTTCTAACTGACTAAGTTTCCATCACATTTGCTTTCTTTCCTATAGAGTTTCAGCC

GTCTCAATGTCTATGTTTTT

In100 candidate199 (in intron):

LOCUS ENSG00000176225

DESCRIPTION rotatin [Source:RefSeq_peptide;Acc:NP_775901]

GCCTCTCTTTAACTTTTTTCTCCTGTTTTATTTCTGCCAATATTTATTATCTCCTTCTACTTTTAGTTTAGTTTACAGCT

CTTTTTTCTAACATTTTAAG

In100 candidate200 (in intron):

LOCUS ENSG00000176225

DESCRIPTION rotatin [Source:RefSeq_peptide;Acc:NP_775901]

GTGAGGTCCCACTCCATGGAGTGCATGCTTGTTCAAGTTCCTAGCATGGTAACTGGTAGACCATTCTTTTGAGTCCAGAA

GTATGTTTGCTTCCTCCTTC

In100 candidate201 (in intron):

LOCUS ENSG00000170677

DESCRIPTION Suppressor of cytokine signaling 6 (SOCS-6) (Suppressor of

cytoine signaling 4) (SOCS-4) (Cytokine-inducible SH2 protein 4) (CIS-4).

[Soure:Uniprot/SWISSPROT;Acc:O14544]

TTTTGAAACTTTCTGAGATAATTTTAGTGTGTCCTTTCCATATATAACATATTATTGTTGTTTTTTTTTTTTAATCAGAT

CTTAAAATGTTTTTCTTCTA

In100 candidate202 (in intron):

LOCUS ENSG00000166342

DESCRIPTION Neuropilin and tolloid-like protein 1 precursor

(Brainspecifictransmembrane

protein containing 2 CUB and 1 LDL-receptor class A

domains potein 1). [Source:Uniprot/SWISSPROT;Acc:Q8TDF5]

ACTGGAGAGCTAATATCCAAACCCACTCTTTATAACTTTAAATTCTTCTTGCTTCTTTTTACTCATACTGGGATTCAGAT

TCTTCATTTCATACTCAGTA

In100 candidate203 (in intron):

LOCUS ENSG00000166912

DESCRIPTION myotubularin related protein 10

[Source:RefSeq_peptide;Acc:NP_00232]

AGTACATTTTGAAGTAAATGTGAACGGCTGAAGGATAACATTAAATGATCCCATTGTCTCTTTGTATTTGCCAGTCTCCT

TTATTTTTGCTATTCTTGGA

In100 candidate204 (in intron):

LOCUS ENSG00000160216

DESCRIPTION 1-acyl-sn-glycerol-3-phosphate acyltransferase gamma (EC 2.3.1.1)

(1- AGP acyltransferase 3) (1-AGPAT 3) (Lysophosphatidic acid acyltransfrase

gamma) (LPAAT-gamma) (1-acylglycerol-3-phosphate O- acyltransferase 3)

[Source:Uniprot/SWISSPROT;Acc:Q9NRZ7]

AGTTCTTGAACAAATAACTTTCAATTACAATTTTTTTTCTACTTATGATTGCCAGTTTTTCTTTTTTCTTTTTTTTTTTT

TTTGAGACAGAGTCTTGCTC

In100 candidate205 (in intron):

LOCUS ENSG00000100170

DESCRIPTION Sodium/glucose cotransporter 1 (Na(+)/glucose cotransporter 1)

High affinity sodium-glucose cotransporter).

[Source:Uniprot/SWISSPROT;Acc:13866]

CACTGATCACTTAGCTTTAACAATTGTCAACATTTTGCCATTTTTGGTTCATCTATCCTCTTCCAATTTTTTCCCCAGCA

GTATTTTATTTTATTTTATT

In100 candidate206 (in intron):

LOCUS ENSG00000169918

DESCRIPTION OTU domain-containing protein 7A (EC 3.-.-.-) (Zinc finger protin

Cezanne 2). [Source:Uniprot/SWISSPROT;Acc:Q8TE49]

CTTTAACCCTGTGTTTCTTTATTTCTGTTTTGTCTACTTTACTTTTCATTTTTATCATTCTTACTATGCTATCTGCAGTC

TGTTTTCTCTTTTCTCCCTT

In100 candidate207 (in intron):

LOCUS ENSG00000183032

DESCRIPTION Mitochondrial 2-oxodicarboxylate carrier (Solute carrier family25

member 21). [Source:Uniprot/SWISSPROT;Acc:Q9BQT8]

GTACTGAAGAGATTCTGGTGTGGTAATTGAATATAACAATTCTCGCTGAGCCTTTTTATGTTTATTCAGAGTTGATTTAA

TTCTTTCCATAGTTTTGTCT

In100 candidate208 (in intron):

LOCUS ENSG00000150656

DESCRIPTION Beta-Ala-His dipeptidase precursor (EC 3.4.13.20) (Carnosine

dieptidase 1) (CNDP dipeptidase 1) (Serum carnosinase) (Glutamate

carboxypeptdase-like protein 2). [Source:Uniprot/SWISSPROT;Acc:Q96KN2]

GAGGAGGAGGAGATTCCTGCTGAATCTGAACTATTCTCTCCTAACAACAATCATTGTTTTCATATTTTTTTCCTGCAGCT

TATTTCCTTCTTCCAACTGT

In100 candidate209 (in intron):

LOCUS ENSG00000175344

DESCRIPTION Neuronal acetylcholine receptor subunit alpha-7 precursor.

[Souce:Uniprot/SWISSPROT;Acc:P36544]

AGTGATTGTCGATATAGTTGGATCAATAACTATCATATTTGTTACTGATTTTTTTGTTGTCCTTGTTCCTTATTTCAGTT

TTTGTTTTCCACTCTGACTT

In100 candidate210 (in intron):

LOCUS ENSG00000175344

DESCRIPTION Neuronal acetylcholine receptor subunit alpha-7 precursor.

[Souce:Uniprot/SWISSPROT;Acc:P36544]

TTTTTTAGCTGTGTCTGTTTTGTTCCTTTCTGTTTCTCATCTAACTACGTAATAATTTGTTTTGGTTCTCAGTTTATTTG

TTTAGCTCTGCAATGTTCCT

In100 candidate211 (in intron):

LOCUS ENSG00000166540

DESCRIPTION zinc finger protein 407 [Source:RefSeq_peptide;Acc:NP_060227]

CCATTCTCCTGATTTCCTTTGCCTGTTCCGTATTTTTTTTTCTTGTTAACCTTCCTCTCCCTGTTCTGTTTCTTACAGTC

CTTTACTTTGTGGGTACTTT

In100 candidate212 (in intron):

LOCUS ENSG00000182090

DESCRIPTION Putative uncharacterized protein C14orf25.

[Source:Uniprot/SWISPROT;Acc:Q8IY25]

TATGTAACGGCCTTCTTTGTCTTTTTTGATCTTTGTTTATTTAAAGTCTGTTTTATCAGAGACTAAGATTGTAACCCCTG

CTTTTTTTTGCTTTCCATTT

In100 candidate213 (in intron):

LOCUS ENSG00000182090

DESCRIPTION Putative uncharacterized protein C14orf25.

[Source:Uniprot/SWISPROT;Acc:Q8IY25]

ATTAATTCTTTCAAAAACATAACTACTGGATTTGTTTATCTTTTGTATGGCTTTTCACATCTCAATTTCCTTCAGTTCAG

CTCTGATTTTGGTTATTTCT

In100 candidate214 (in intron):

LOCUS ENSG00000182090

DESCRIPTION Putative uncharacterized protein C14orf25.

[Source:Uniprot/SWISPROT;Acc:Q8IY25]

TTAAGTCATACTATTTATTTTCTGTGAATTAACATTTGGGTTATTTTCTCTCTTTTTTGTGCCCCCTCTTAAGAACAGGG

CTGCCCTAAATATTCTTTTT

In100 candidate215 (in intron):

LOCUS ENSG00000182090

DESCRIPTION Putative uncharacterized protein C14orf25.

[Source:Uniprot/SWISPROT;Acc:Q8IY25]

CCATGAAGTTCTTGACACCTGGACACATACTGGTCTCTGCTTAACACACCAACACATCTGCTTCTGATCTTCTAGCAGTT

GTTTTCATTTTAGTTTCCAT

In100 candidate216 (in intron):

LOCUS ENSG00000182090

DESCRIPTION Putative uncharacterized protein C14orf25.

[Souce:Uniprot/SWISPROT;Acc:Q8IY25]

AGATTTGTGGGCACTCATGAGGTTTTTTTGTACTTTGGATTATAACCTAATATTAAATTTTTGCTTGAATTGTTCCAGCT

TTGTTCCTGTTCTGTTGCTT

In100 candidate217 (in intron):

LOCUS ENSG00000139865

DESCRIPTION Tetratricopeptide repeat protein 6 (TPR repeat protein 6).

[Souce:Uniprot/SWISSPROT;Acc:Q86TZ1]

TTAAGTCATACTATTTATTTTCTGTGAATTAACATTTGGGTTATTTTCTCTCTTTTTTGTGCCCCCTCTTAAGAACAGGG

CTGCCCTAAATATTCTTTTT

In100 candidate218 (in intron):

LOCUS ENSG00000184459

DESCRIPTION Bactericidal/permeability-increasing protein-like 2 precursor.

Source:Uniprot/SWISSPROT;Acc:Q8NFQ6]

GAAGGTTTACAAATGGCTGATTATGGTCTCTGAGTAACCTCTTTTTTTTTTTTTTTTTTTTCATTTTTTTCCTCAGTG

TTCTGTATAACTCCTTTGCT

In100 candidate219 (in intron):

LOCUS ENSG00000101493

DESCRIPTION Zinc finger protein 516. [Source:Uniprot/SWISSPROT;Acc:Q92618]

EXON

TTCCTTTTTTGAGTGTTGCTGAATACTGGAGTAACTACACTTTCTTAGGGCTTTTTTCTTCCACTTGTTTCTGTTCAGCT

GTTTCTTTTTTGTGGTGTAC

In100 candidate220 (in intron):

LOCUS ENSG00000100934

DESCRIPTION Protein transport protein Sec23A (SEC23-related protein A).

[Sorce:Uniprot/SWISSPROT;Acc:Q15436]

AGTATAATCATGAAGTCTTTAAATTGCATTAAAACATAACTTTCAAAATTACCAATGTCTTCTTTTACACATTCTCAGTC

TTTTTTGCTTCTTACTGTTT

In100 candidate221 (in intron):

LOCUS ENSG00000186031

DESCRIPTION CDNA FLJ45135 fis, clone BRAWH3038252, highly similar to Formin1

isoform IV. [Source:Uniprot/SPTREMBL;Acc:Q6ZSY1]

GGTTGATAACAAATTAAAAAAAAAATCTTTGTCATGTTCCACATCTTTGTTATCACTTTTTCTTGTTCTCTTCATCAGTT

TTCTTCACTCTTGTCTTTTT

In100 candidate222 (in intron):

LOCUS ENSG00000206125

DESCRIPTION

CTAGAATAGAAGTGGAAATAACATGCATTGATTAGTTCATTCATTTTTCATTCATTTAAATTTTTTTACATTCAGTGCTG

TTACTTATTTTTGACACTTC

In100 candidate223 (in intron):

LOCUS ENSG00000198838

DESCRIPTION Ryanodine receptor 3 (Brain-type ryanodine receptor) (RyR3) (RY-3) (Brain ryanodine receptor-calcium release channel). [Source:Uniprot/SWISPROT;Acc:Q15413]

CAACTGAGAATAGTTTGCTTTGTGTCTTAGATCATTAACAAAGGTGAGATTTTTGTTCATTTAATGTTCTCCTCAGTCTG
GGTTCATTTCCCTGTTCCTT

In100 candidate224 (in intron):

LOCUS ENSG00000198838

DESCRIPTION Ryanodine receptor 3 (Brain-type ryanodine receptor) (RyR3) (RY-3) (Brain ryanodine receptor-calcium release channel). [Source:Uniprot/SWISPROT;Acc:Q15413]

GCTTTGCCTTGGGAATGGAGGTTTCTAACCAATTCTGTGATGCTTCATTTTATTCACAGACTGGTGTTATTTAAAGTGTT
TTTTGTTTTGTTTTGTTTTT

In100 candidate225 (in intron):

LOCUS ENSG00000133424

DESCRIPTION Glycosyltransferase-like protein LARGE1 (EC 2.4.-.-) (Acetylgluosaminyltransferase-like 1A). [Source:Uniprot/SWISSPROT;Acc:O95461]

TGAATTCTGTGAAAATTAACTCCCCTGCCTTTTGGTGCTCTTTTTTTCTCTTTCTCTCTGAAGTTGCAGTTAATTTCCCC

AATGGATTTCCTCAGTCTTT

In100 candidate226 (in intron):

LOCUS ENSG00000142185

DESCRIPTION Transient receptor potential cation channel subfamily M member

(EC 3.6.1.13) (Long transient receptor potential channel 2) (LTrpC2) (LTrp-2)

(Transient receptor potential channel 7) (TrpC7) (Estrogen- responsive

lementassociated

gene 1 protein). [Source:Uniprot/SWISSPROT;Acc:O94759]

TGTGATGTTTTTAACTTACTAATTCAATCTCTTTATGTGTTATAGGTCTATTTATATTTTCTATTTCTTCTCCAGTCACC

TTTGGTAGTTTGTGTCTTTC

In100 candidate227 (in intron):

LOCUS ENSG00000150527

DESCRIPTION Cutaneous T-cell lymphoma-associated antigen 5 (cTAGE-5 protein

(cTAGE family member 5) (Meningioma-expressed antigen 6/11).

[Source:Uniprt/SWISSPROT;Acc:O15320]

TTCTTTCTTTCTGGGACTATAAAAATGGGTATTTTAACTTTTTATTATATCTCATCTGTCTCTTACGTTCTTTTCCAGGG

TTTCCATCTTTTTGTTTGCC

In100 candidate228 (in intron):

LOCUS ENSG00000169857

DESCRIPTION Cell death regulator Aven. [Source:Uniprot/SWISSPROT;Acc:Q9NQS1

GTGATGTAAGCTATGCTAACAAGCCGTAATGTCCTTTTCTGCTAATTTCATATCTGTGTCAGTTCTGGCATTATTTTGAT

TGACTGATCATTCTCCTCTT

In100 candidate229 (in intron):

LOCUS ENSG00000180190

DESCRIPTION Uncharacterized protein C8orf42 (INM01).

[Source:Uniprot/SPTREML;Acc:Q86YL5]

TGTGAATGGCATTTCTTTTTCTATTGTCTAACTGTAATAAAGCCATTTATTTTTTATTTCTTTTATATTCAGCCATTTTT

TGTTAGGTCTAATCGTTCTT

In100 candidate230 (in intron):

LOCUS ENSG00000180184

DESCRIPTION

AGTCTTTTCAAAGAACTAACCATTGGTCTTGTTGATTTCTCTATTGTTTTTCTATTCTCTGTGTCAGTTATTTCTGCTCT

AATCTTGATTATTTCCTTTC

In100 candidate231 (in intron):

LOCUS ENSG00000166377

DESCRIPTION Probable phospholipid-transporting ATPase IIB (EC 3.6.3.1).

[Sorce:Uniprot/SWISSPROT;Acc:O43861]

AGTGTGCTGTACTGTGGCTTTATAGAAAATCTTAACATGGGTAGGCTAATTCTTCTTACTTTTTTCAGAAATCTTTAAGT

TATTCTAGTTCCTTTGCCTT

In100 candidate232 (in intron):

LOCUS ENSG00000166377

DESCRIPTION Probable phospholipid-transporting ATPase IIB (EC 3.6.3.1).

[Sorce:Uniprot/SWISSPROT;Acc:O43861]

AGTATCATAAGAATTATCAAAATGTTAAGTTCTTAGAGCTTTAAGTAACATATTTCTGTGTTTTTAATTTTCTACAGTCT

TACAGTTTTTTTTTTAAACT

In100 candidate233 (in intron):

LOCUS ENSG00000165379

DESCRIPTION Leucine-rich repeat and fibronectin type-III domain-containing

rotein 5 precursor. [Source:Uniprot/SWISSPROT;Acc:Q96NI6]

CCAATTTTGAGGCTGGGAAAAGGAAGCTACTTGAAACTTCCTAAAGGCTTAACTGGCTAACCTATTAGCTTATTTCAGTG

ATTTTTATTTTCTGTTTACT

In100 candidate234 (in intron):

LOCUS ENSG00000165379

DESCRIPTION Leucine-rich repeat and fibronectin type-III domain-containing

rotein 5 precursor. [Source:Uniprot/SWISSPROT;Acc:Q96NI6]

CCTGGATAAATACTTAACTGTATCTTCAACTCTTCTTTTAATTTCATCTATTGTAATTCATGGTACTATTTTTTCCAGTT

TGCTTTTTATATATATTTCA

In100 candidate235 (in intron):

LOCUS ENSG00000165379

DESCRIPTION Leucine-rich repeat and fibronectin type-III domain-containing

rotein 5 precursor. [Source:Uniprot/SWISSPROT;Acc:Q96NI6]

CTCTTCATTTATCCTTCTCCCACATTTTATGACTTAACCATTACTCATTTATTAGCTTAATTTTTATTTCCTCAGAAAAC

CTTCTTCTTACTTCTCTGAT

In100 candidate236 (in intron):

LOCUS ENSG00000036448

DESCRIPTION Myomesin-2 (M-protein) (165 kDa titin-associated protein) (165 Da connectin-associated protein). [Source:Uniprot/SWISSPROT;Acc:P54296]

TCAAAAATCTAATAACTAGGATTGATATTTCCCTACAATTTGCAGGTATTCTTCCTCACAAGGATTCATGCATTATTGAC

TTGCGATTTCTTTTCTTCCT

In100 candidate237 (in intron):

LOCUS ENSG00000100320

DESCRIPTION RNA-binding protein 9 (RNA-binding motif protein 9) (Hexaribonuleotide-binding protein 2) (Repressor of tamoxifen transcriptional activity. [Source:Uniprot/SWISSPROT;Acc:O43251]

CTCTTAACTGCATGTTATTTTTACTCCTTTCTCTTAACTAAATTTGTTTTCTTTCTCAGGACTTTACATCCTCTATCTCC

CTTCTAGTGGGTGGGTTTTT

In100 candidate238 (in intron):

LOCUS ENSG00000139915

DESCRIPTION MAM domain-containing protein 1 precursor (MAM domain-containin glycosylphosphatidylinositol anchor protein 2).

[Source:Uniprot/SWISSPROT;cc:Q7Z553]

CAAATTCAATGACATCATATGATATTTGATTAACAAATATATTCATTTCCTTAACAAATTTCTTATCAGTAAGCACTTAT

TTTGTCTCAATCTTTATTTT

In100 candidate239 (in intron):

LOCUS ENSG00000139915

DESCRIPTION MAM domain-containing protein 1 precursor (MAM domain-containin glycosylphosphatidylinositol anchor protein 2). [Source:Uniprot/SWISSPROT;cc:Q7Z553]

AGTTATAACCTTTCTTTTGTTTCTACTTAATCCTCAGACTATTTTGAAATCATTTCAAACCTTCTTTACTCTTTTTTTCC
TTCGTGTATCCATGTTTACA

In100 candidate240 (in intron):

LOCUS ENSG00000139915

DESCRIPTION MAM domain-containing protein 1 precursor (MAM domain-containin glycosylphosphatidylinositol anchor protein 2). [Source:Uniprot/SWISSPROT;cc:Q7Z553]

CTAAAACTTGCCTACTTCAAATCCAAAGAATTTAACCTTTTCTTTAACAAGTTCTTTATTTTTTCTATAGTCCACCAGCA
CTCTTATTGTTACTTTACAT

In100 candidate241 (in intron):

LOCUS ENSG00000139915

DESCRIPTION MAM domain-containing protein 1 precursor (MAM domain-containin glycosylphosphatidylinositol anchor protein 2). [Source:Uniprot/SWISSPROT;cc:Q7Z553]

GGTTTAGACAAATGTTTTTACTTTAGATTCTCATGGCACCAATTAACTTAATAAGAGTATCTTTTTTATCAGAGTATAAA
TTATTTATATTTCTTTTTCT

In100 candidate242 (in intron):

LOCUS ENSG00000139915

DESCRIPTION MAM domain-containing protein 1 precursor (MAM domain-containin

glycosylphosphatidylinositol anchor protein 2).

[Source:Uniprot/SWISSPROT;cc:Q7Z553]

CTTCCAATCCATTAACCTGTGATATCTTCCAATTTAATTGTATCCTCTTCAGTTTTTCTCATCGATGTTTGATGGTTTGA

ATTGTATCCATCTTTCCCCT

In100 candidate243 (in intron):

LOCUS ENSG00000139915

DESCRIPTION MAM domain-containing protein 1 precursor (MAM domain-containin

glycosylphosphatidylinositol anchor protein 2).

[Source:Uniprot/SWISSPROT;cc:Q7Z553]

AGTCCATAACACTGAATTACTTTTATTTTTACTTTATTACTCAGCATGTCAGTTTTTTTTTTTTTTTAATTTTTGCTGT

TTTTTAGCTTCTTGAAGTGG

In100 candidate244 (in intron):

LOCUS ENSG00000139915

DESCRIPTION MAM domain-containing protein 1 precursor (MAM domain-containin

glycosylphosphatidylinositol anchor protein 2).

[Source:Uniprot/SWISSPROT;cc:Q7Z553]

AATCAAGATGTTAGTTCTAAGCTATTCTAATTATGTTCCCACTAACTACACTTTCTTCATATTTCTTTACTTTAACAGAC

TTGTTTCTGACATTATGTTT

In100 candidate245 (in intron):

LOCUS ENSG00000139915

DESCRIPTION MAM domain-containing protein 1 precursor (MAM domain-containin

glycosylphosphatidylinositol anchor protein 2).

[Source:Uniprot/SWISSPROT;cc:Q7Z553]

AGACCATTAACATGGGATGTCTTTCCACTTCTTGGTGTCTTCCTCAATTTTTTTAACCAGTATTTTATAGTTTTCCTTA

TAAAGATCTTTTACTTCCTT

In100 candidate246 (in intron):

LOCUS ENSG00000139915

DESCRIPTION MAM domain-containing protein 1 precursor (MAM domain-containin

glycosylphosphatidylinositol anchor protein 2).

[Source:Uniprot/SWISSPROT;cc:Q7Z553]

ATAAGGTTCCAATTCAAGTGGTTTTTTTCCATATGCATAACCAATTGAATGGTATATATTTTTCTCTCTTCTGTGCAGAC

ACTCTATATATTTTATATTT

In100 candidate247 (in intron):

LOCUS ENSG00000134146

DESCRIPTION ATP binding domain 4 [Source:RefSeq_peptide;Acc:NP_542381]

AACATTGTTTTAAATGGATATAACTGACAAATTATTATGCTCTTCTGCAATTTCTTTGTCATTTTACTTTCAGCCTTAAC

AACTTTTTCCCCTATTTAAT

In100 candidate248 (in intron):

LOCUS ENSG00000134138

DESCRIPTION Homeobox protein Meis2 (Meis1-related protein 1).

[Source:Uniprt/SWISSPROT;Acc:O14770]

ATTATAACATCTTTCACACTGCGTTGTAATTATTTGATTCCTAGTCTCTCTCTCTCTCCCTGCTTTTCCAAGGTGCAGAT

TATCACTTCTTCGATTATCT

In100 candidate249 (in intron):

LOCUS ENSG00000134138

DESCRIPTION Homeobox protein Meis2 (Meis1-related protein 1).

[Source:Uniprt/SWISSPROT;Acc:O14770]

ATTAGTCTCTTTAACATTATTTCAAACTGGTTTGTTCCCTTTTGTGCAGTGTGACCTGTTCTTGTATTACAATAATCCCC

GATGTACCCTTTTTTCTTTC

In100 candidate250 (in intron):

LOCUS ENSG00000166068

DESCRIPTION Sprouty-related, EVH1 domain-containing protein 1 (Spred-1).

[Surce:Uniprot/SWISSPROT;Acc:Q7Z699]

AGTTTAACAACCACAAGAATCTCTTCTTTCTGTTGATTTTATCACCACATCTATTTCCGTTTTTGCCAGGCAACCACTAT

TTTGTTCTCTCAATTATTTC

In100 candidate251 (in intron):

LOCUS ENSG00000166068

DESCRIPTION Sprouty-related, EVH1 domain-containing protein 1 (Spred-1).

[Surce:Uniprot/SWISSPROT;Acc:Q7Z699]

AGAAGTTAGACAAGATTCTAACTTAGGTCTTTTCTCACCCATTTTTGTTTTGTTTTGTTTTTTTTCTCCCAGTATTCTAT

TCAGTTTGCTTATGATTTTT

In100 candidate252 (in intron):

LOCUS ENSG00000206194

DESCRIPTION ATP-binding cassette, sub-family A (ABC1), member 11 (pseudogen)

(ABCA11) on chromosome 4 [Source:RefSeq_dna;Acc:NR_002451]

TTATGATTTGTAAAGTATATGCATATGAATCTAGTAACTGGGATAATTTGTTATTTTTGTTTCTTTCAGCTATGTGTTCT

CATTTCACCCAAGACTTTTT

In100 candidate253 (in intron):

LOCUS ENSG00000172575

DESCRIPTION RAS guanyl releasing protein 1

[Source:RefSeq_peptide;Acc:NP_00730]

GGAACATCATGCTTATTACTCTTGACACTCATAGAACACTTATTTAACATTTATGTAGCTTTCTTTTCATTATCTCAGTC

TGATTCCTCAGCTATATTTT

In100 candidate254 (in intron):

LOCUS ENSG00000100485

DESCRIPTION Son of sevenless homolog 2 (SOS-2).

[Source:Uniprot/SWISSPROT;Ac:Q07890]

ACCTTTCTTCACTCTAGTAGAATAATTAACTAGAGTGAAGAAAGTTTTTTCATTTCCTCTCTCATTTTTTCTTTGCAGCC

AATTGTCTTTATATTATTTT

In100 candidate255 (in intron):

LOCUS ENSG00000100485

DESCRIPTION Son of sevenless homolog 2 (SOS-2).

[Source:Uniprot/SWISSPROT;Ac:Q07890]

AACATTGCTCTAAATTTCTATCTTGTTATCTTATAAAGGAAATAACTTTTGAAGTTAATATTTTTTTCTGTGCAGTGTTG

TATTCTTCCTCTCTTAGCTG

In100 candidate256 (in intron):

LOCUS ENSG00000155189

DESCRIPTION 1-acyl-sn-glycerol-3-phosphate acyltransferase epsilon (EC

2.3..51) (1-AGP acyltransferase 5) (1-AGPAT 5) (Lysophosphatidic acid

acyltranserase epsilon) (LPAAT-epsilon) (1-acylglycerol-3-phosphate

Oacyltransferas

5). [Source:Uniprot/SWISSPROT;Acc:Q9NUQ2]

CTAGTGTGTTGCTTTTAACCTGTAGCTTGAAAAAACTCTGCTTTCATTGTTTTTTTTTGTTTTCTGTCACTGGCTCAGCC

CTGCTTTCAATTGTTTATAT

In100 candidate257 (in intron):

LOCUS ENSG00000150667

DESCRIPTION fibrous sheath interacting protein 1

[Source:RefSeq_peptide;AccNP_689810]

ACTGATTGGTAAGAGCTCTATAAATATGTGAATATATTAACCTTTTGTATATCATCTTTGTTGCATTTATTTCCCCAGTT

TCTCAGGTTCCTTTTAATTT

In100 candidate258 (in intron):

LOCUS ENSG00000166073

DESCRIPTION Probable G-protein coupled receptor 176 (HB-954).

[Source:Uniprt/SWISSPROT;Acc:Q14439]

TTGTTGTGTTGTTAGGAATTTTTATATATTCTGGATATACATATAACATATATACATACATGTTACTTTTTCCTCAGAT

ATACCTTTTTTCAGTGTTCA

In100 candidate259 (in intron):

LOCUS ENSG00000012983

DESCRIPTION Mitogen-activated protein kinase kinase kinase kinase 5 (EC

2.711.1) (MAPK/ERK kinase kinase kinase 5) (MEK kinase kinase 5) (MEKKK 5)

(Kiase homologous to SPS1/STE20) (KHS). [Source:Uniprot/SWISSPROT;Acc:Q9Y4K4]

EXON

TCTCTATTCCCCATTTCCTTGTACTTAGGCCTTTTCTCCTAGATAATTATAACAATTTTGAACTGTAATTTCTTCCAGTT

TTTCCTTTTTTTCCTTCCAA

In100 candidate260 (in intron):

LOCUS ENSG00000198513

DESCRIPTION Atlastin (GTP-binding protein 3) (Guanine nucleotide-binding

prtein 3) (GBP-3) (Brain-specific GTP-binding protein).

[Source:Uniprot/SWISSROT;Acc:Q8WXF7]

TGGGAGCCACTGCACCCGGCCCCAATTTTTCCATATATTTGAAATAACAATTTAAGAAATTGTCCCTGTGTTCAGAAGAC

TTCTCTCGTGATCTGTTTTC

In100 candidate261 (in intron):

LOCUS ENSG00000156970

DESCRIPTION Mitotic checkpoint serine/threonine-protein kinase BUB1 beta (E

2.7.11.1) (hBUBR1) (MAD3/BUB1-related protein kinase) (Mitotic checkpoint inase MAD3L) (SSK1). [Source:Uniprot/SWISSPROT;Acc:O60566]

GAACCTCTAAAAATTTAACTTTTTTTCTCCTATATTCTCTCTTTGTCTTTTTACTTTACTTTCTGAGTATATTATCAGCT
TAATATTTTTGGTCTTTTTA

In100 candidate262 (in intron):

LOCUS ENSG00000100503

DESCRIPTION Ninein (hNinein) (Glycogen synthase kinase 3 beta-interacting potein) (GSK3B-interacting protein). [Source:Uniprot/SWISSPROT;Acc:Q8N4C6]

AACATACACACATGTTTGCATGATTCCGGAGGCTTGATGATCTAACAAGGAAAATTCCTTTTTTCTGTCAGTTTAATTTT
ACCTTGAGTCTTCTCTCGTG

In100 candidate263 (in exon):

LOCUS ENSG00000177306

DESCRIPTION similar to olfactory receptor 873 (PJCG6), mRNA [Source:RefSeq_na;Acc:NM_001040066]

TGTCATCAATAACATATTCATATATTTCGATAGTACTATGTTTGGTTTTCTTCCCATTTCAGGGATCTTTTTGTCTTACT
ATAAAATTGTCCCCTCCATT

In100 candidate264 (in intron):

LOCUS ENSG00000100505

DESCRIPTION Tripartite motif-containing protein 9 (RING finger protein 91).[Source:Uniprot/SWISSPROT;Acc:Q9C026]

GAGAAAAGCAAACTAACCAAAAGTAAGATCATAATTTTTATATTTTGTTTTTAACTCAGCCATTAGTCCTTTTTTATAGT

AAGTTGCTTTTTTAATTCTT

In100 candidate265 (in exon):

LOCUS ENSG00000197293

DESCRIPTION

CGTCATCAATAACATATTCATATATTTCGATAGTACTATGTTTGGTTTTCTTCCCATTTCAGGGATCTTTTTGTCTTACT

ATAAAATTGTCCCCTCCATT

In100 candidate266 (in exon):

LOCUS ENSG00000206014

DESCRIPTION

TGTCATCAATAACATATTCATATATTTCGATAGTACTATGTTTGGTTTTCTTCCCATTTCAGGGATCTTTTTGTCTTACT

ATAAAATTGTCCCCTCCATT

In100 candidate267 (in intron):

LOCUS ENSG00000139926

DESCRIPTION FERM domain-containing protein 6 (Willin). [Source:Uniprot/SWISPROT;Acc:Q96NE9]

AGTTCACCTGTTCATGCCTCCCTTGTGTGGTTCTTTCTTTTTAACTTTCAATATTTCCGTGATTTTTTTTTTCTTCAGGA

ACTTGGTTCCCTATTTTTCC

In100 candidate268 (in exon):

LOCUS ENSG00000197116

DESCRIPTION G protein-coupled receptor homolog clone G3 (Fragment).

[SourceUniprot/SPTREMBL;Acc:Q9UDD8]

AGCGTCATCAATAACATATTCATATATTTCGATAGTACTATGTTTGGTTTTCTTCCCATTTCAGGGATCTTTTTGTCTTA

CTATAAAATTTTCCCCTCCA

In100 candidate269 (in intron):

LOCUS ENSG00000180998

DESCRIPTION Protein GPR137C (Fragment). [Source:Uniprot/SPTREMBL;Acc:Q8N3F9

TTGCTTCTTTTCTAACTCCTCTCTTCCTTTCTTCCTTTTTTACTGTCTTCTTTTGTGGTTAAGTGATTTCTCTGGCAGTA

TGTTTTAATCTATTGCTTTT

In100 candidate270 (in intron):

LOCUS ENSG00000180998

DESCRIPTION Protein GPR137C (Fragment). [Source:Uniprot/SPTREMBL;Acc:Q8N3F9

TTTCAAGCTCACTGATTGTTCTGTTTAACCAATTCTGCTGTTGCTCCCTATTACATCTTTCATTTCATTCAGTGTATTTT

CAGCTCCTGAGTTTCTGTTT

In100 candidate271 (in intron):

LOCUS ENSG00000163950

DESCRIPTION Histone RNA hairpin-binding protein (Histone stem-loop-binding

rotein). [Source:Uniprot/SWISSPROT;Acc:Q14493]

GCAAACTAAGCTACTTCGTCTTCGTTGACTCATTACCTAATGCATCCTTAACCATGTTTAATCTCCCTTGATTATCAGTG

TGTTGATCCTTTCTCTTTTG

In100 candidate272 (in intron):

LOCUS ENSG00000147324

DESCRIPTION malignant fibrous histiocytoma amplified sequence 1

[Source:Refeq_peptide;Acc:NP_004216]

TCAAGGTGATTTTTCACCTTTGTTTATTTAACCTTGTGTTCTTTTTGTTTATCCAACCATTTGTGCTCTTGTTCCCAGTT

TTTTTTGTGAGTGATTCTCT

In100 candidate273 (in intron):

LOCUS ENSG00000147324

DESCRIPTION malignant fibrous histiocytoma amplified sequence 1

[Source:Refeq_peptide;Acc:NP_004216]

GGTAGTAACTCCTAGTCTCCTTCCTCTCCATCCTCTGTACCTTGTGTCTCTTTTCCCATTTCTGATTTTTGTATCCAGGA

GTTTCTGATTTTTGCCTTTT

In100 candidate274 (in intron):

LOCUS ENSG00000173273

DESCRIPTION Tankyrase-1 (EC 2.4.2.30) (TANK1) (Tankyrase I) (TNKS-1) (TRF1-

interacting ankyrin-related ADP-ribose polymerase).

[Source:Uniprot/SWISSPRT;Acc:O95271]

AAAAATCACATTTTCTGTCATTATCCTTTGCTACTTGAGCATCCACTAACATGACCTTTGGATATCCTGTCCTTTCAGTT

TTTTGACATCTTCCAGTTTC

In100 candidate275 (in intron):

LOCUS ENSG00000173273

DESCRIPTION Tankyrase-1 (EC 2.4.2.30) (TANK1) (Tankyrase I) (TNKS-1) (TRF1-interacting ankyrin-related ADP-ribose polymerase). [Source:Uniprot/SWISSPRT;Acc:O95271]

AGTTGTTGAGTAGTGAAGTTTCTAACTATAATTGTAGATTTAATTATTTCTCCTTTAACTTCTATCAGTTTTTGTTTCATATATTTTGTAGCTTTGTTGT

In100 candidate276 (in intron):

LOCUS ENSG00000173273

DESCRIPTION Tankyrase-1 (EC 2.4.2.30) (TANK1) (Tankyrase I) (TNKS-1) (TRF1-interacting ankyrin-related ADP-ribose polymerase). [Source:Uniprot/SWISSPRT;Acc:O95271]

AGATTTCACTTTATAATCATAACCATAAATCTCAAGTGTGTTTTTATCCTTGCCTCAGCCATTTCTTTTTACACATTTAATTTTTTCCTCTCAGCTTATT

In100 candidate277 (in intron):

LOCUS ENSG00000173273

DESCRIPTION Tankyrase-1 (EC 2.4.2.30) (TANK1) (Tankyrase I) (TNKS-1) (TRF1-interacting ankyrin-related ADP-ribose polymerase). [Source:Uniprot/SWISSPRT;Acc:O95271]

AAACGAATAATGCCTTACCTGCAATATATTAAATATAACTTTTTTATTCACCTTTTTAAATTCGACCTTCAGTGATCTGTAGTTATTATTCTTCATTTTC

In100 candidate278 (in intron):

LOCUS ENSG00000103148

DESCRIPTION UPF0171 protein C16orf35 (Alpha globin regulatory elementcontaning gene protein) (Protein CGTHBA) (-14 gene protein). [Source:Uniprot/SWISPROT;Acc:Q12980]

AGTTTCCATCATAGGGGTAACTACTGTTCCCATTTTTCTGTGTCTTTCCAGGACCTTCTGTGTCAATACAGGCGTATGTA

TTTCTTTTTTCTTTTTTTTT

In100 candidate279 (in intron):

LOCUS ENSG00000187446

DESCRIPTION Calcium-binding protein p22 (Calcium-binding protein CHP) (Calcneurin homologous protein) (Calcineurin B homolog). [Source:Uniprot/SWISSPRT;Acc:Q99653]

AGTAAACTTAACATTGATACAATCCTCTTATCTAATCTATTACTCTGTTTTATTAACATATTATCCAGTTTATTCTAATT

TATATTCATATTCCAATTTT

In100 candidate280 (in intron):

LOCUS ENSG00000175806

DESCRIPTION Peptide methionine sulfoxide reductase (EC 1.8.4.11) (Proteinethionine-

S-oxide reductase) (PMSR) (Peptide-methionine (S)-S-oxide reductae) (Peptide Met(O) reductase). [Source:Uniprot/SWISSPROT;Acc:Q9UJ68]

GAAACAAAAGAATTTAACTGATATTTTTATTAATTGTTCTTTCAGTCACACTTTCCCTTCTATCACATTATCATACGTGT

TCATAGTATCTTTGCTGTTT

In100 candidate281 (in intron):

LOCUS ENSG00000109685

DESCRIPTION Probable histone-lysine N-methyltransferase NSD2 (EC 2.1.1.43)

Nuclear SET domain-containing protein 2) (Wolf-Hirschhorn syndrome candidat 1

protein) (Multiple myeloma SET domain-containing protein) (Protein trithrax-5).

[Source:Uniprot/SWISSPROT;Acc:O96028]

AGAGATCTGACCTTTTAACCTGCTTACCTGTATTCACCTTCTCATTTGACTTCAGTGGAATCTGGTACCTCCTTTCTTTT

ATTTTGGGCCCGTCTCTTTC

In100 candidate282 (in exon):

LOCUS ENSG00000109685

DESCRIPTION Probable histone-lysine N-methyltransferase NSD2 (EC 2.1.1.43)

Nuclear SET domain-containing protein 2) (Wolf-Hirschhorn syndrome candidat 1

protein) (Multiple myeloma SET domain-containing protein) (Protein trithrax-5).

[Source:Uniprot/SWISSPROT;Acc:O96028]

CTTCTGAACTCGCTAGGTTCTTATCAACATTTGGGGGATAACTTTGTATATTTTTTTCATTTGGCTTTTCTTTACCAGTT

TCTGATTTTTATTCTCAATA

In100 candidate283 (in intron):

LOCUS ENSG00000020577

DESCRIPTION Sterile alpha motif domain-containing protein 4A.

[Source:Uniprt/SWISSPROT;Acc:Q9UPU9]

TGATGAGGTTATATATGCCGTGTTTTTAACTTTCATCTGCTTAGTGAATTCCTTTTGTCTTTTGATCTCTCTCAGCAACC

TAGTTTTTGTTTTTAATTTT

In100 candidate284 (in intron):

LOCUS ENSG00000131979

DESCRIPTION GTP cyclohydrolase I (EC 3.5.4.16) (GTP-CH-I).

[Source:Uniprot/WISSPROT;Acc:P30793]

AAGTGAGAATTCTGACTTTCTTTTGATCTAGAAGTTAAGTAACTCTGTAGACCTTTTATATTACTTTCACCCCAGAAGAA

AATTCCATTTTTCCCCTTTT

In100 candidate285 (in intron):

LOCUS ENSG00000146555

DESCRIPTION Protein sidekick-1 precursor.

[Source:Uniprot/SWISSPROT;Acc:Q7ZN4]

GGGTGATAGAGTGAGACCCTGTCTCTAAAAATACAAAGGTATTAAAGTTAACACTTGAAATGCCTTTCATTCTGTCAGTG

GCTCTTTTTTTGTTTGTTTT

In100 candidate286 (in intron):

LOCUS ENSG00000146555

DESCRIPTION Protein sidekick-1 precursor.

[Source:Uniprot/SWISSPROT;Acc:Q7ZN4]

AGTTTTATAGTATTTTAATTGCTTTACTTCCAAAAGGTGTTTTAATAACTGATAAAACAACTCTCTTCAACTTTTCAGAA

TTTTGTTATTACTTGTCATC

In100 candidate287 (in intron):

LOCUS ENSG00000146555

DESCRIPTION Protein sidekick-1 precursor.

[Source:Uniprot/SWISSPROT;Acc:Q7ZN4]

AAGTAACTATTCTTTTTGCGTACATTTTTGGTTTTCTTACAGTTGAATTTTGAAAATTCTTTTTTCTGCATAGTAGGTTT

TTGTTCTTTTTTTTTGTTTT

In100 candidate288 (in intron):

LOCUS ENSG00000146555

DESCRIPTION Protein sidekick-1 precursor.

[Source:Uniprot/SWISSPROT;Acc:Q7ZN4]

TCTGCCACATAGATAACCATATTTATTAGACTTTTGGTTTTTCCTTTTTCTTTTTCTTTCTGGCAACATAAACAGCTCTG

CGTATATGTGTACTTCCTCT

In100 candidate289 (in intron):

LOCUS ENSG00000104643

DESCRIPTION Myotubularin-related protein 9.

[Source:Uniprot/SWISSPROT;Acc:Q6QG7]

AAGCATAGATGATATGGAAATTTACAAGAGAATAACCGTATGTCTTGTACATTTTTTTTGTTTTTTTACTGACAGTTTGG

TTGCTGTGTGTCTTTCACTT

In100 candidate290 (in intron):

LOCUS ENSG00000167930

DESCRIPTION Protein ITFG3. [Source:Uniprot/SWISSPROT;Acc:Q9H0X4]

AGGGGAATAATCCGTTTGAATAACCATATATGAGGTATTGTGTTTATCTTTCTTTCTTCAAGTTATTCTTGGTGTCAGCT

CCTTTTTTTTTTTTTTTTCT

In100 candidate291 (in intron):

LOCUS ENSG00000128298

DESCRIPTION Brain-specific angiogenesis inhibitor 1-associated protein 2-lie protein 2 (BAI1-associated protein 2-like protein 2). [Source:Uniprot/SWISPROT;Acc:Q6UXY1]

ACTTGAGCTAACTGTATTTACTTTGTCATTTCCTCTTTTTCTCCCACACTATTTCTTGTCAGTTTGGTTGAGTTATTATA

CTATACATTCCCCTTTTCCC

In100 candidate292 (in intron):

LOCUS ENSG00000136573

DESCRIPTION Tyrosine-protein kinase BLK (EC 2.7.10.2) (B lymphocyte kinase)(p55- BLK). [Source:Uniprot/SWISSPROT;Acc:P51451]

GATTATATCATACAAAACATTCTGCAATTTACTCTTTTCATGTAACAATAATGTATCCTGGGTATTTTTCTTTGCCAGTT

CAGATCTCTTTTATCCTTTT

In100 candidate293 (in intron):

LOCUS ENSG00000126777

DESCRIPTION Kinectin (Kinesin receptor) (CG-1 antigen). [Source:Uniprot/SWISPROT;Acc:Q86UP2]

CCTTTTTATTTCTTTATGTCCCTTCCCACACTCTAACCACTACCTTTCTTACCTTTGCTTTTCTTTTAGTTTTGCCAGAT

CTAGTTGTATCCCTATTTTG

In100 candidate294 (in intron):

LOCUS ENSG00000126777

DESCRIPTION Kinectin (Kinesin receptor) (CG-1 antigen).

[Source:Uniprot/SWISPROT;Acc:Q86UP2]

AGAGCTAAATTAACTTTGTCTCAACCTCTTTACTTTTATTTTTCAGTTTTGCCTCCCTGCTTTTAAAACTTTGTCTTTGT

TCTTCCCTGAATTTTCCTGT

In100 candidate295 (in exon):

LOCUS ENSG00000157954

DESCRIPTION WD repeat domain phosphoinositide-interacting protein 2 (WIPI-2

(WIPI49-like protein 2). [Source:Uniprot/SWISSPROT;Acc:Q9Y4P8]

AACTCCATAACGCTGAGGAAATACATCATTTTCACTTCAGTGGCTTTTAAATCCTGCTTATGAATTTTAGCTTTTTGTTT

GTTTGTTTTCTCTTTTTGCC

In100 candidate296 (in intron):

LOCUS ENSG00000125386

DESCRIPTION Uncharacterized protein C4orf8 (Protein IT14).

[Source:Uniprot/WISSPROT;Acc:P78312]

TGATGAGTTTGAGTAAAATAAAGAAATACCTGTTAACATATTTGTAGTATTGTATCTTTATTCTGTTTGCAGATGATGTT

TCTCTCATATATATCATTTT

In100 candidate297 (in intron):

LOCUS ENSG00000100206

DESCRIPTION Meiotic recombination protein DMC1/LIM15 homolog.

[Source:Uniprt/SWISSPROT;Acc:Q14565]

AGTTATTTAGATTTAACTATAAAGATTTTTGTTTTCTTTGTTCATTATGGTTTCTTGCATCCCAGATGTTATTTTTTCCT

GTATTTTTGTTTTGCTGTAA

In100 candidate298 (in intron):

LOCUS ENSG00000103978

DESCRIPTION Transmembrane protein 87A precursor.

[Source:Uniprot/SWISSPROT;cc:Q8NBN3]

TGAGCCACCGTGCCCGGCCTGAGACACTTTAACATTATCTTTTTCTTTGTTATTTCTGTGCTTTCTCATATTTACCAGTT

GTCTTTGATTTTGTACTTTT

In100 candidate299 (in intron):

LOCUS ENSG00000090565

DESCRIPTION Rab11 family-interacting protein 3 (Rab11-FIP3) (EF handscontaning

Rab-interacting protein) (Eferin).

[Source:Uniprot/SWISSPROT;Acc:O7515]

AGAAAATGACCAATAATCCCACTATCCTGAGAAAAATAACATTCCCATTATTTACTACATTTCTTTTCAGACTTTTTTT

TTTTTTTTGAAATAGAGTCT

In100 candidate300 (in intron):

LOCUS ENSG00000092529

DESCRIPTION Neutral alpha-glucosidase C (EC 3.2.1.20).

[Source:Uniprot/SWISPROT;Acc:Q8TET4]

TTCCTCATCTTGTCCCGAATAACCGAATTTCTTCATTTCTTATTTATCCTTCCAGTATTTTTTTGTAAATACATGTATTT

TTATATTATTGCTTTTCTCT

In100 candidate301 (in intron):

LOCUS ENSG00000092529

DESCRIPTION Neutral alpha-glucosidase C (EC 3.2.1.20).

[Source:Uniprot/SWISPROT;Acc:Q8TET4]

AGTCATCATTAATAACCCTTCTTAATCTCTTGCCTTTCTGTTTTAAAACTTATCACAGAATGCTCTGTGTTAGCTTTGCA

TCTCTTTTATCTCCTCCAAT

In100 candidate302 (in intron):

LOCUS ENSG00000139971

DESCRIPTION Uncharacterized protein C14orf37 precursor.

[Source:Uniprot/SWISPROT;Acc:Q86TY3]

AGGTAGTTTTAATGGTTCCTGATAGTTTTCCTAATAACCTAAATTTCCATCATGTTTCCTCTATCCAGCCTCTTTTTCAA

CTTCTCTTTAATTAGAGTCC

In100 candidate303 (in intron):

LOCUS ENSG00000139971

DESCRIPTION Uncharacterized protein C14orf37 precursor.

[Source:Uniprot/SWISPROT;Acc:Q86TY3]

TTTCCTGGGAACCCTAACTCACGCTGAGCCCTTCCTTTTGAAATTCTTCCAGTCATTTGTCAGTATTATTTAATTTCTAT

TCACTGCACTATCTCATTTT

In100 candidate304 (in intron):

LOCUS ENSG00000011275

DESCRIPTION E3 ubiquitin-protein ligase TRIAD3 (EC 6.3.2.-) (Ubiquitinconjgating enzyme 7-interacting protein 1) (Zinc finger protein inhibiting NFappa-B) (Triad domain-containing protein 3). [Source:Uniprot/SWISSPROT;Acc:9NWF9]

CAAATGTGTGTGTGGTTGTGTAACCACCCATCTTTTCCCAATGTCACTTGTATTTATTTATCTTTTCTCACCTGCCAGCT
TTCTTCCCTGCTTGTTTTTC

In100 candidate305 (in intron):

LOCUS ENSG00000032219

DESCRIPTION AT-rich interactive domain-containing protein 4A (ARID domainontaining protein 4A) (Retinoblastoma-binding protein 1) (RBBP-1). [Source:niprot/SWISSPROT;Acc:P29374]

GGATAGTTTTCTGTCACTTGTAGTAAAAGTTATTGAGTAACAATGCTCCTTGCTTGTATTTTGTTCCACTTACTCCAGGT
ATCTTCCTTGTGGTGTTTTC

In100 candidate306 (in intron):

LOCUS ENSG00000205880

DESCRIPTION

TTTACTAATTATCCTGTTACCTATATCTAATATATCATTAATAACTTGCATTTATTTTTAAATTTTTTTTTAATTCAGAC
CTGCCTTTGTGTTTTGTTTT

In100 candidate307 (in intron):

LOCUS ENSG00000205877

DESCRIPTION

TTTACTAATTATCCTGTTACCTATATCTAATATATCATTAATAACTTGCATTTATTTTTAAATTTTTTTTTAATTCAGAC

CTGCCTTTGTGTTTTGTTTT

In100 candidate308 (in intron):

LOCUS ENSG00000197386

DESCRIPTION Huntingtin (Huntington disease protein) (HD protein).

[Source:Uiprot/SWISSPROT;Acc:P42858]

GGAAATGGGATGAAAAATTATGGGATGAAAAATTGCCTATGGATAGTTTAACTTGAAAGAATCTGCCTTTGTTTACAGAT

AGTTATCTTTTTTTCTTTTTT

In100 candidate309 (in intron):

LOCUS ENSG00000197386

DESCRIPTION Huntingtin (Huntington disease protein) (HD protein).

[Source:Uiprot/SWISSPROT;Acc:P42858]

ACATTTGGCATAAGAATAACTGGATAATTAAGCATCTTATTCTCTGGCCTGTGTCTTTACAGTTAAAGGTAGATTTACTC

ACCTCTCCTTTTTTGTTTTT

In100 candidate310 (in intron):

LOCUS ENSG00000164741

DESCRIPTION Rho GTPase-activating protein 7 (Rho-type GTPase-activating

proein 7) (Deleted in liver cancer 1 protein) (Dlc-1) (HP protein) (StAR-relatd

lipid transfer protein 12) (StARD12) (START domain-containing protein 12)

[Source:Uniprot/SWISSPROT;Acc:Q96QB1]

TCTTGAGTCTTTCCTAACTCTGGGAAAACTTTTCACTGTGCTCAAATTTCATCTTTTTCTCTCTTCTTTTTATTTCAGTT

TATCTCCTTCACTTATTTCA

In100 candidate311 (in intron):

LOCUS ENSG00000164741

DESCRIPTION Rho GTPase-activating protein 7 (Rho-type GTPase-activating

proein 7) (Deleted in liver cancer 1 protein) (Dlc-1) (HP protein) (StAR-relatd

lipid transfer protein 12) (StARD12) (START domain-containing protein 12)

[Source:Uniprot/SWISSPROT;Acc:Q96QB1]

GTAAAATTCTGTGATTTATAAACATTTTGCTTTTAGATTTAGAATTTTAACCAAGAAATAAATGTTGATTCTTTTCAGCC

TTCCCATTGTCTAGATTTTT

In100 candidate312 (in intron):

LOCUS ENSG00000151838

DESCRIPTION Putative uncharacterized protein ENST00000281581.

[Source:Uniprt/SWISSPROT;Acc:P0C221]

GTTAACATGGCATATGTCTTTTGTTTTAGATCTTCTTTGTTCCCTTTGCCTTAGCTGTTCAGTTGCACTATGATGTGTCC

AAATTTTCTTTTCATTTTTC

In100 candidate313 (in intron):

LOCUS ENSG00000128881

DESCRIPTION Tau-tubulin kinase 2 (EC 2.7.11.1).

[Source:Uniprot/SWISSPROT;Ac:Q6IQ55]

ATTTAACCCAATCTACTTCTAATTTTTTTATCCAGTGCCTCTCTCCCTCTTCCCTCTCATATTCCTTTCCCTCTGCTTTT

TACATTTATTATTATTATTT

In100 candidate314 (in intron):

LOCUS ENSG00000185053

DESCRIPTION Zeta-sarcoglycan (Zeta-SG) (ZSG1).

[Source:Uniprot/SWISSPROT;Ac:Q96LD1]

GAGTATTTTATCCTATGCTTATTTGCCATCTGTGTCTTCTTTGGTAACATCTTTTCATGTTTTTTGCCTATTTCTCAGTT

AGGTTGTTTGTTTTCTTGTT

In100 candidate315 (in intron):

LOCUS ENSG00000185053

DESCRIPTION Zeta-sarcoglycan (Zeta-SG) (ZSG1).

[Source:Uniprot/SWISSPROT;Ac:Q96LD1]

CTTAATATTGAGTCTTCCTATTCATGAACATGGATTAACTCTCTATTTATTTAGATATTCTTTGATTTCTTTCATCAGTA

TTTTGCGGTTTTCCTCATAT

In100 candidate316 (in intron):

LOCUS ENSG00000185053

DESCRIPTION Zeta-sarcoglycan (Zeta-SG) (ZSG1).

[Source:Uniprot/SWISSPROT;Ac:Q96LD1]

ATGTAAAACTGTCCTTTGTTAACCTCTTCAATGTGTCTTTTTTTAATTTCTTTTTTATTTCTTTGCTACACCCAGGTGCT

GTGATCTCTCACTTGGTTTC

In100 candidate317 (in intron):

LOCUS ENSG00000185053

DESCRIPTION Zeta-sarcoglycan (Zeta-SG) (ZSG1).

[Source:Uniprot/SWISSPROT;Ac:Q96LD1]

ATTTATAACATAGCTGGAACTTAATCCAATTATTTTTCTTCTTGCCCTACTACTGTATTCTTTCCCCCGTATCACAGCT

CATTCACTTTATTACTAGTT

In100 candidate318 (in intron):

LOCUS ENSG00000185053

DESCRIPTION Zeta-sarcoglycan (Zeta-SG) (ZSG1).

[Source:Uniprot/SWISSPROT;Ac:Q96LD1]

GTTTTAACATTTCTTTTTTCATCTCTGATTTTATTTGAGTTTTCTTTTATTTTTGTTGTCTATCTTAAGGTTTGTCAGTT

TTGTTTATCTTTTACAAACC

In100 candidate319 (in intron):

LOCUS ENSG00000185053

DESCRIPTION Zeta-sarcoglycan (Zeta-SG) (ZSG1).

[Source:Uniprot/SWISSPROT;Ac:Q96LD1]

CTACTAACCTTCCCCCTTTCCTGCTTCTTGCTAACTATTTTTGGAATTTTTTCAGTCCTCCTTTAATAATGCTTTAATAA

GCTTTGTTTTCTATCACCTC

In100 candidate320 (in intron):

LOCUS ENSG00000185053

DESCRIPTION Zeta-sarcoglycan (Zeta-SG) (ZSG1).

[Source:Uniprot/SWISSPROT;Ac:Q96LD1]

ACATAGTTTAAATAACTTTATTGATGTATTTTATTTACCTTTATCCATTCATTTATTTCTATTTCCTCTATGCAGAATGC

TGTATTAACTTTCTCCCCTT

In100 candidate321 (in intron):

LOCUS ENSG00000185053

DESCRIPTION Zeta-sarcoglycan (Zeta-SG) (ZSG1).

[Source:Uniprot/SWISSPROT;Ac:Q96LD1]

AAGGTATTTTGAAAATTGTTTGCAAGATTTTTCATAACCATTGGTGTCTTTCTCTTTATTATCAGTGTTATGGAAGCCAT

CCTCTATTATCTTTTTTCTC

In100 candidate322 (in intron):

LOCUS ENSG00000185053

DESCRIPTION Zeta-sarcoglycan (Zeta-SG) (ZSG1).

[Source:Uniprot/SWISSPROT;Ac:Q96LD1]

TTTAATGCTCAATAAATTTGGAAGAATGAATTCTGAATGAACTAACACCTAGGGAAGCCTTGCTGTTATTGTCAGAATCA

TTTACTTGTCTTTGATATTC

In100 candidate323 (in intron):

LOCUS ENSG00000185053

DESCRIPTION Zeta-sarcoglycan (Zeta-SG) (ZSG1).

[Source:Uniprot/SWISSPROT;Ac:Q96LD1]

AGACCCTCATCATTACTCTAGCTCTTGGCCCTTCCTCTATCTTCAAAGTTAACAACATTAGCCAACTGTCTGTCTCAGAC

TATTCTTGTGCCTCTCTCTT

In100 candidate324 (in intron):

LOCUS ENSG00000131951

DESCRIPTION Leucine-rich repeat-containing protein 9.

[Source:Uniprot/SPTREBL;Acc:Q6ZRR7]

AGAGTTTGGAAATACATTAACCGCCTCAACCTGTATTACTCTTGCTTCCAGCAACCTGACTTCACTATTCCCTTTTCCAT

TCCATTTTTACTGTAGCTTT

In100 candidate325 (in intron):

LOCUS ENSG00000104723

DESCRIPTION Tumor suppressor candidate 3 (Protein N33).

[Source:Uniprot/SWISPROT;Acc:Q13454]

TAGCTTTGAGGGTGTTCTTTGAATTAACTGCAACAAATATCTGAGTAGCTGTTTTATTCTTACTCTTTTCAGGATTATTG

TCTAGTGTCACTTTTGTCCT

In100 candidate326 (in intron):

LOCUS ENSG00000104723

DESCRIPTION Tumor suppressor candidate 3 (Protein N33).

[Source:Uniprot/SWISPROT;Acc:Q13454]

TTACGTACTTATCTCAATATAATCTACATTAAAATATTCTGTTTATAGTTAACCATCTTTGGAGTCTTTCCATGTCAGCC

TACTGTAATTTCAACTTTTT

In100 candidate327 (in intron):

LOCUS ENSG00000104723

DESCRIPTION Tumor suppressor candidate 3 (Protein N33).

[Source:Uniprot/SWISPROT;Acc:Q13454]

TTTTAATGTTATATTTACATGTGCTAATTTAGAATGGAATAACAAACATATTGTATTTTCCTTATGTTCTATGTTCAGCA

CATAATAATTTTCTTTCTTT

In100 candidate328 (in exon):

LOCUS ENSG00000136240

DESCRIPTION ER lumen protein retaining receptor 2 (KDEL receptor 2) (KDEL edoplasmic reticulum protein retention receptor 2) (ERD2-like protein 1) (EL-1). [Source:Uniprot/SWISSPROT;Acc:P33947]

AGCACCACTAACCATTCCTTACATTCTTTTTTGTATGTGTGGTTTTCTTTTTATTTAACCCGCAGCCGACATCGTAGTTT

CTTGTTTTGTTTTGTTTTAC

In100 candidate329 (in intron):

LOCUS ENSG00000137842

DESCRIPTION Transmembrane protein 62. [Source:Uniprot/SWISSPROT;Acc:Q0P6H9]EXON

CATTCTATTTAACCGACTTGTCTCTTTCATCTCTTTTACTGTACAGATTGCATCCCTTACCTCCCCCCTCTTTTTTGTAC

CCTTAAAATTTATTTGTTTT

In100 candidate330 (in intron):

LOCUS ENSG00000137842

DESCRIPTION Transmembrane protein 62.

[Source:Uniprot/SWISSPROT;Acc:Q0P6H9]EXON

AGCCACTTTGCTTTATATAACCACTTCGAATGTATATACATTTTCTCCTTAACCAGATAAGTTCCTCAATGCAGGAACTT

TTTTTTTTTTTTTTTTTTTT

In100 candidate331 (in intron):

LOCUS ENSG00000164631

DESCRIPTION Zinc finger protein 12 (Zinc finger protein KOX3) (Zinc finger

rotein 325) (Gonadotropin-inducible transcription repressor 3) (GIOT-3).

[Surce:Uniprot/SWISSPROT;Acc:P17014]

TTCTTGTGTTCTTCGTGTAACTTCTGGTTTTTTTTTTTAATCTTTACCGTGCAGTGCCTGAGACTGTTTCTGGACCCTG

TTCTACTTTGTACTTTTCTT

In100 candidate332 (in intron):

LOCUS ENSG00000163982

DESCRIPTION otopetrin 1 [Source:RefSeq_peptide;Acc:NP_819056]

AGAGATAACCATTGTTGACATTTTAGTTTGTGTCCTTCCAGATTTTTTCCGTCTGTCTGTGTGTGTGTGTATCCATCTAT

CTATCTATCATCTACATATC

In100 candidate333 (in intron):

LOCUS ENSG00000020426

DESCRIPTION CDK-activating kinase assembly factor MAT1 (RING finger

proteinMAT1) (Menage a trois) (CDK7/cyclin H assembly factor) (p36) (p35)

(Cyclin 1-interacting protein) (RING finger protein 66).

[Source:Uniprot/SWISSPROT;cc:P51948]

AGTACCCTGTTAACATTTTCTTTTTTGTTGTATAGTTCTGATTACTTTTTTGTTTTTACTACCTCAGCTCCTTCCCTAAT

ATTTTTGTAAGCTTTCTTTT

In100 candidate334 (in intron):

LOCUS ENSG00000020426

DESCRIPTION CDK-activating kinase assembly factor MAT1 (RING finger proteinMAT1) (Menage a trois) (CDK7/cyclin H assembly factor) (p36) (p35) (Cyclin 1-interacting protein) (RING finger protein 66). [Source:Uniprot/SWISSPROT;cc:P51948]

TGGTTTTTATTTTTCATTCTATTGATGAGTTGTATAACATTGATTTGCATTTTTTTTGTTGTTGTTGTTACATATCAGAG
CTGTTTTTTTTTCTTGTTTT

In100 candidate335 (in intron):

LOCUS ENSG00000103227

DESCRIPTION Transmembrane protein 112. [Source:Uniprot/SWISSPROT;Acc:Q96S06

CTTGATGTGGTCTTGATTAGCTTTTTAGTAAGTCTTGAAATTGGGTAACATGAGTCCTCCACCTCTGTTCTTTTCCAGAT
GATTTTGGCTTTTTAATCCC

In100 candidate336 (in intron):

LOCUS ENSG00000027075

DESCRIPTION Protein kinase C eta type (EC 2.7.11.13) (nPKC-eta) (PKC-L). [Surce:Uniprot/SWISSPROT;Acc:P24723]

GCCGTATGTTTAACTACATGATTTGTCTTTATTTCCAACATCTTGCCAGATATTTCTATTTGAGTACTTTTGTGTCCAAA

TTTAAACTCATTTCTTTCCC

In100 candidate337 (in intron):

LOCUS ENSG00000155970

DESCRIPTION EF-hand domain-containing family member A2.

[Source:Uniprot/SWISPROT;Acc:Q86XE3]

AACTTTGTTTTGCCTTCTAAGGATATAAATTATAACTTTGGCCTTCTCTGTGCATTGCTTTGTTTCTTCCAGGTTCTTTT

TTATTTCTGTTTGATTTTAT

In100 candidate338 (in intron):

LOCUS ENSG00000155970

DESCRIPTION EF-hand domain-containing family member A2.

[Source:Uniprot/SWISPROT;Acc:Q86XE3]

TTCTATTTGCTTGCATTTAACCCATTCCGTTTCTCTGTTTGTGCCTTTTGTCTCTCCAGCCAGCATTCTCTCTTTCCATA

TAGATTGATATTCATTCCTT

In100 candidate339 (in intron):

LOCUS ENSG00000168818

DESCRIPTION Syntaxin-18 (Cell growth-inhibiting gene 9 protein).

[Source:Unprot/SWISSPROT;Acc:Q9P2W9]

TTAACTTAGGGAGAACCAACATCTTTATATTTCTTTTTCTATCCAAGAATGATGTGTCCTTTCCTTTGTGTCTTTCAGGA

ATCTTTTTTGGTTTTTTACG

In100 candidate340 (in intron):

LOCUS ENSG00000106415

DESCRIPTION Glucocorticoid-induced transcript 1 protein.

[Source:Uniprot/SWSSPROT;Acc:Q86VQ1]

CTATTCTAAAAGATAATGCAAATATAATGTTAACTCCATGTAATATATGTTTGCCTGTGTTCATTCATTTGCTTTCAGTT

TTTCTCTTTTCCTTAAATTT

In100 candidate341 (in intron):

LOCUS ENSG00000176177

DESCRIPTION ENTH domain containing 1 [Source:RefSeq_peptide;Acc:NP_689725]

EXON

ACTTTGGTTTGTAGTCTGTTTTATCTGATATAAGTATAACTACTCCCACTCTTTTTTGGTTTCCAGTTACATGGAGTATC

TTTTTCTACCCCTTCACTTT

In100 candidate342 (in intron):

LOCUS ENSG00000003147

DESCRIPTION Islet cell autoantigen 1 (69 kDa islet cell autoantigen) (ICA69

(p69) (Islet cell autoantigen p69) (ICAp69).

[Source:Uniprot/SWISSPROT;AccQ05084]

TGTTTAACCACCCATTCCTCTCTTTCTCTTAGCAGTGGAACGTGTATTTCTCCCTGGCCTTGGTTTAAAGTCTTTCTCAC

TCATTCTTCGGTTTCTTTCC

In100 candidate343 (in intron):

LOCUS ENSG00000106443

DESCRIPTION PHD finger protein 14. [Source:Uniprot/SWISSPROT;Acc:O94880]

GTCTATCCTAAATTAGCATAACAAATTTATAAATTTTCTTCCCTCACATTTTCAATGTCTATTTTGCATTATTTGCAGAT

AAGCTTAATTTTCCCTTGTT

In100 candidate344 (in exon):

LOCUS ENSG00000100354

DESCRIPTION Trinucleotide repeat-containing 6B protein.

[Source:Uniprot/SWISPROT;Acc:Q9UPQ9]

TTTTCTTTGCTGTGAAAGTAACCCTAAAATATGTTTTCTTCTTTTGTATTTTTTTGGTTTTTGTTTTTAAGCCATCAGAA

GTTTCAGATCTTTACTCTTT

In100 candidate345 (in intron):

LOCUS ENSG00000140015

DESCRIPTION Potassium voltage-gated channel subfamily H member 5 (Voltagegted

potassium channel subunit Kv10.2) (Ether-a-go-go potassium channel 2)

(EAG2). [Source:Uniprot/SWISSPROT;Acc:Q8NCM2]

GTGTTCTGTCCTAGAAATATGAAATTCTGTGCTCATAAGCATGTAACATATGCACTTTTCTTACATTTTTATCAGTTATC

TTTTTTCCTGCATGTCAAAT

In100 candidate346 (in intron):

LOCUS ENSG00000005108

DESCRIPTION Thrombospondin type-1 domain-containing protein 7A precursor.

[ource:Uniprot/SWISSPROT;Acc:Q9UPZ6]

TATGTATACATGTGCCATGCTGGTGCGCTGCACCCACTAACGTGTCATCTAGCATTTTTTTATTCCTCTTTGAGACAGGT

CCACTTTATTCATTTTACTT

In100 candidate347 (in intron):

LOCUS ENSG00000168781

DESCRIPTION Histidine acid phosphatase domain containing 2A isoform 2

[Soure:RefSeq_peptide;Acc:NP_055474]

TCTCTCTGTTCCTTCCCTTAACTTTCTTATCTGCTTCTCTCCTTGTCTCTTTTTTGTCTCCAGTCTCCCTATCAGTCCTC

TTTACATCTGTTAAAATTTT

In100 candidate348 (in intron):

LOCUS ENSG00000003989

DESCRIPTION Low-affinity cationic amino acid transporter 2 (CAT-2) (CAT2).

Source:Uniprot/SWISSPROT;Acc:P52569]

TTTGCCACCACTAAATAACAATGAGTATTTTTGAGTCCTGCATCCTTTAGTTCTTCCTTCAGGACCACCATTCAGTTTTA

TTGTATTCTCTTAATGCTTT

In100 candidate349 (in intron):

LOCUS ENSG00000100357

DESCRIPTION Adenylosuccinate lyase (EC 4.3.2.2) (Adenylosuccinase) (ASL)

(AASE). [Source:Uniprot/SWISSPROT;Acc:P30566]

CTATTTGTAAATGACATTGAATCTCCGTAACGATTATTTGGTTAAACATTTTATCTCTTGTTTTATCTTTACCAGTTACT

CCCTCTCTTACTTAAATTGT

In100 candidate350 (in intron):

LOCUS ENSG00000154001

DESCRIPTION Serine/threonine-protein phosphatase 2A 56 kDa regulatory subunt epsilon isoform (PP2A, B subunit, B' epsilon isoform) (PP2A, B subunit, B6 epsilon isoform) (PP2A, B subunit, PR61 epsilon isoform) (PP2A, B subunit R5 epsilon isoform). [Source:Uniprot/SWISSPROT;Acc:Q16537]

CTGTCTATATTGGTTAATACATTTTTCTGGTTACAATGAAGTCTTGCTAACCTGAAAGCTTTTTTTTGTCTTTTTCAGAT

GACTTTCCATATCTTTTGTC

In100 candidate351 (in intron):

LOCUS ENSG00000006747

DESCRIPTION Adseverin (Scinderin). [Source:Uniprot/SWISSPROT;Acc:Q9Y6U3]

ATTAATAACTGTAACCATCATACTATACTTTGTCTCCAGAACTTATTTATCTTATACCTGAAAGTCTGAATTCTTTCACC

AACATTTCTCCATTTCCTCT

In100 candidate352 (in intron):

LOCUS ENSG00000196588

DESCRIPTION MKL/myocardin-like protein 1 (Myocardin-related transcription fctor A) (MRTF-A) (Megakaryoblastic leukemia 1 protein) (Megakaryocytic acut leukemia protein). [Source:Uniprot/SWISSPROT;Acc:Q969V6]

GCTTTCTCTCTCTCACTCTTCTTGAAGCCTTTCCTAACTCATATTTAGTGTTAGATGCCCCTCTTTTGGTTTTCCCAGTT

CCCAATTTTTTTTTTTTTTT

In100 candidate353 (in intron):

LOCUS ENSG00000136267

DESCRIPTION Diacylglycerol kinase beta (EC 2.7.1.107) (Diglyceride kinase bta) (DGK-beta) (DAG kinase beta) (90 kDa diacylglycerol kinase).

[Source:Unprot/SWISSPROT;Acc:Q9Y6T7]

GAAGACTGTGTAACTTCTGTTTTAATTGACACTCTTTATCGTATTTTTCCCTTCATTTTCAACTTACTACATCAGAATTG

TCTGTCAATTCTCTATGTTT

In100 candidate354 (in intron):

LOCUS ENSG00000136267

DESCRIPTION Diacylglycerol kinase beta (EC 2.7.1.107) (Diglyceride kinase

bta) (DGK-beta) (DAG kinase beta) (90 kDa diacylglycerol kinase).

[Source:Unprot/SWISSPROT;Acc:Q9Y6T7]

AACAACTTTTCTTTAACTGTATATGAGCCCTCTATACTTTTATTCTCCTCCACGTGTAGTTTGTTATTGTTATCAGAGTT

TACTTATTATTTTTCTATTG

In100 candidate355 (in intron):

LOCUS ENSG00000136267

DESCRIPTION Diacylglycerol kinase beta (EC 2.7.1.107) (Diglyceride kinase

bta) (DGK-beta) (DAG kinase beta) (90 kDa diacylglycerol kinase).

[Source:Unprot/SWISSPROT;Acc:Q9Y6T7]

AACTAACATACAAGGCTTGATTCTTTGCACTTAGCTTTTTTTCTCTCTCTCCCACATGTATTCAGTAACTTTGTCGTAGT

GTTTTCTGTGTTACCTCTTT

In100 candidate356 (in intron):

LOCUS ENSG00000136267

DESCRIPTION Diacylglycerol kinase beta (EC 2.7.1.107) (Diglyceride kinase bta) (DGK-beta) (DAG kinase beta) (90 kDa diacylglycerol kinase). [Source:Unprot/SWISSPROT;Acc:Q9Y6T7]

GAACCAAGTACTAACCCACGATCACATTTTCTTTCCTGTAAAGCTTCAGAATTTCTTTCTTCTCTCTGCAAAGTGATCCT
TACAATAGATCTCATTTTTC

In100 candidate357 (in intron):

LOCUS ENSG00000187546

DESCRIPTION CDNA FLJ16237 fis, clone HCASM2002754 (Hypothetical protein FLJ6237). [Source:Uniprot/SPTREMBL;Acc:Q6ZNB7]

GTGATAACCCCTTTATCATTTTTTTATTGTGTCTGTTTGATTCTTGTCACTTTTCTCCTTTATTAATCTGGCTACCAGTC
TATTTTGTTAATCTTTTCAA

In100 candidate358 (in intron):

LOCUS ENSG00000187546

DESCRIPTION CDNA FLJ16237 fis, clone HCASM2002754 (Hypothetical protein FLJ6237). [Source:Uniprot/SPTREMBL;Acc:Q6ZNB7]

CATCAAACAAGTATGTTTTTCTGAATCAAAATAATTTTAAATTAACTGTGACGTTTAATTCTTTCTGTGATTTTACAGCA
CGTCTGTGTGTTCTTTCTCA

In100 candidate359 (in intron):

LOCUS ENSG00000187546

DESCRIPTION CDNA FLJ16237 fis, clone HCASM2002754 (Hypothetical protein FLJ6237). [Source:Uniprot/SPTREMBL;Acc:Q6ZNB7]

TTTGGGAAACAAGACCTAAAGTGTTAACTTTCCATTTTGTTACAAGTTTTTCCCTTCAGCTTGTGTAATCACTGCTCTTC
AATGTTCCAGTTTCTGTTTC

In100 candidate360 (in intron):

LOCUS ENSG00000078674

DESCRIPTION Pericentriolar material 1 protein (PCM-1) (hPCM-1).

[Source:Unirot/SWISSPROT;Acc:Q15154]

TAACTGTTTGGTTTCTTATCACATTCTACTTGATACACTTTTCCCCTTTTGGCAGGCTCTCCTGGTTTCCCTCCTGTCTT

TCTGTACCTTATTAGTGTAT

In100 candidate361 (in intron):

LOCUS ENSG00000078674

DESCRIPTION Pericentriolar material 1 protein (PCM-1) (hPCM-1).

[Source:Unirot/SWISSPROT;Acc:Q15154]

AGCTAATTTGGATTAGCATTCTAACCTCCTGACCTCTGTTGCTATCCTTGCTTTTATTTTACAGATGGTAACTTTTTTTT

CCGATATGTTTATTCTTCCA

In100 candidate362 (in intron):

LOCUS ENSG00000078674

DESCRIPTION Pericentriolar material 1 protein (PCM-1) (hPCM-1).

[Source:Unirot/SWISSPROT;Acc:Q15154]

TATCTTTTGGTTTTACTTTGAGAGATTTTCTTTGACTTTAACACTTGTATGAAATTATTTTTATTTCAGCTGTCATATTT

TTAACTTCTGAACACTTTCT

In100 candidate363 (in intron):

LOCUS ENSG00000100380

DESCRIPTION Hsc70-interacting protein (Hip) (Suppression of tumorigenicity

rotein 13) (Putative tumor suppressor ST13) (Protein FAM10A1)

(Progesteronereceptor-associated p48 protein) (Renal carcinoma antigen NY-REN-

33). [Soure:Uniprot/SWISSPROT;Acc:P50502]

TTTTTTTATATATATTTATTTTTTTAACTCTCCAAGGTAACAATAGGTTATTATTTTTTTCTTTTTTCTGTTTTTCAGTT

GTATTTTATTTTATTTTTTA

In100 candidate364 (in intron):

LOCUS ENSG00000136261

DESCRIPTION Basic leucine zipper and W2 domain-containing protein 2.

[Sourc:Uniprot/SWISSPROT;Acc:Q9Y6E2]

TTAAATTGTTATTGCATAGAAAACCATATATTCTATACACCCTCCTTTTAACCAAGTACTTTACATTCTTTTATTCAGCT

TGCTTTTTTCCACTATCAAT

In100 candidate365 (in intron):

LOCUS ENSG00000136261

DESCRIPTION Basic leucine zipper and W2 domain-containing protein 2.

[Sourc:Uniprot/SWISSPROT;Acc:Q9Y6E2]

GAGGAATGATTGTTCCATACCACCGTTAAGAGCGTTACTGTGTTAACTATGTTTACCATCTTGTCTCTTTTTTGCCAGAT

TTTTACTTTCACCTTATTTT

In100 candidate366 (in intron):

LOCUS ENSG00000106541

DESCRIPTION Anterior gradient protein 2 homolog precursor (Secreted cement

land protein XAG-2 homolog) (AG-2 protein) (hAG-2) (HPC8).

[Source:Uniprot/WISSPROT;Acc:O95994]

AGTAACTCCTTCCAAATTCTCCCTTAATTTTTTTGCTCTTTATCACATCCATTCAGCACTTTATCTCATGTTCTGCTCTA

TCTTATTTATTATTTCATGC

In100 candidate367 (in intron):

LOCUS ENSG00000140009

DESCRIPTION Estrogen receptor beta (ER-beta).

[Source:Uniprot/SWISSPROT;AccQ92731]

TGAGGAAGTTAAAAAATGCTTGACATCTCATTTATCTAGAGGTAACTACTATTAGCATTTTAGTGTTCTTCTCTTCAGTC

TTTTTGTTATGCATGTTTTT

In100 candidate368 (in intron):

LOCUS ENSG00000140009

DESCRIPTION Estrogen receptor beta (ER-beta).

[Source:Uniprot/SWISSPROT;AccQ92731]

TGAATTTTAAAAATCAAAGGATTTACTATAGTCTTATACTACATAATAACACACTTTCCTTTTGATTCTAGTTCTCAGGA

AAGCCTTTTCTTCTTTTTTT

In100 candidate369 (in intron):

LOCUS ENSG00000048052

DESCRIPTION Histone deacetylase 9 (HD9) (HD7B) (HD7).

[Source:Uniprot/SWISSROT;Acc:Q9UKV0]

GATTAACCATAAATGTAATGGTTTCTTTCTGGATTCTAATTTCTATACCATTCATCTGTACATCTGTCCTTCTGCCAGTA

CTTCTTTGTTGTATTATTAT

In100 candidate370 (in intron):

LOCUS ENSG00000048052

DESCRIPTION Histone deacetylase 9 (HD9) (HD7B) (HD7).

[Source:Uniprot/SWISSROT;Acc:Q9UKV0]

TCTCATAGATTAACTAAAATGTGTTTGATATTTATCTTTGAAGTTTTATTTTTTATTTAGTATTCCTTCCCTCACCAGCA

TACTTAATCTCCATTCTTTG

In100 candidate371 (in intron):

LOCUS ENSG00000048052

DESCRIPTION Histone deacetylase 9 (HD9) (HD7B) (HD7).

[Source:Uniprot/SWISSROT;Acc:Q9UKV0]

AGATCGTTATGGAAAGAGCTGCTTGTGGGCTTAACTCCTTTTCCTAGTTATCTCTGTTTTTCCAGATGTTCTTTTTCTTT

CTCTAATCATTTTGAAATTG

In100 candidate372 (in intron):

LOCUS ENSG00000165807

DESCRIPTION Uncharacterized protein C14orf50.

[Source:Uniprot/SWISSPROT;AccQ96LQ0]

GTTTTTGCCGGTTATAAGTGATTAACTCATTTTAACATTCCACTTCTTTCCGATTTCAGTAAGCTAATATTTTCATGTGT

ATTTTCTGACTTTTCTCTTT

In100 candidate373 (in intron):

LOCUS ENSG00000004846

DESCRIPTION ATP-binding cassette sub-family B member 5 (P-glycoprotein ABCB) (ABCB5 P-gp). [Source:Uniprot/SWISSPROT;Acc:Q2M3G0]

GTAGCTAACATTTCTGGTCTCTTCCTTCCTTATTCTAGGTACCTATTTCCATTGGTATCATTTTCCTTCTGCATCCAGGT
CTTTTTTTCACCTTTCTTAA

In100 candidate374 (in intron):

LOCUS ENSG00000105866

DESCRIPTION Transcription factor Sp4 (SPR-1). [Source:Uniprot/SWISSPROT;AccQ02446]

CCCTCTGTTGCCTTCCCTAACCCTCACAAGGGGATTTAATTTTTCTTTCCTCCTCCTCTGTCAGGAGGCCCATCATTACT
TTTATTTGTCCTTCCTCTAT

In100 candidate375 (in intron):

LOCUS ENSG00000105877

DESCRIPTION Ciliary dynein heavy chain 11 (Axonemal beta dynein heavy chain11). [Source:Uniprot/SWISSPROT;Acc:Q96DT5]

AACTAGGATTAACATTCTGGCTATCTAAACTTTTTTTTTTTTTTAATCAGAAATTTTAACCACTATACCCTTTCTCTTG
TGATTCTTTTGCCCGTAACT

In100 candidate376 (in intron):

LOCUS ENSG00000105877

DESCRIPTION Ciliary dynein heavy chain 11 (Axonemal beta dynein heavy chain11). [Source:Uniprot/SWISSPROT;Acc:Q96DT5]

ACCATTTCAATTTGTTGACATATAACTGTTCATAGTAGTCTCTTATGATCCTTTGTATTTCTGTCGTATCAGTTGTAATA
ACTCTTCTTTCATTTCTGAT

In100 candidate377 (in intron):

LOCUS ENSG00000105877

DESCRIPTION Ciliary dynein heavy chain 11 (Axonemal beta dynein heavy chain11). [Source:Uniprot/SWISSPROT;Acc:Q96DT5]

CCCTAGAACCTACAAAAATTGTAGAGCCCTTCCTGTCACCACAACTAACTGTTGGTAGTTTTATTGATCCCTTTGCAGTT
GTGATCCTTCCTCTGATTGT

In100 candidate378 (in intron):

LOCUS ENSG00000136237

DESCRIPTION Rap guanine nucleotide exchange factor 5 (Guanine nucleotide exhange factor for Rap1) (Related to Epac) (Repac) (M-Ras-regulated Rap GEF) MRGEF).

[Source:Uniprot/SWISSPROT;Acc:Q92565]

AGTGCTTGTAACCTAAGTGACTATTAACATGCCTTCTTTTCTTATGCAGCAGCATCTCCCCACAATTTCTTATTTTCAGA
TCATAACTCTCTTCTTATTT

In100 candidate379 (in intron):

LOCUS ENSG00000136237

DESCRIPTION Rap guanine nucleotide exchange factor 5 (Guanine nucleotide

exhange factor for Rap1) (Related to Epac) (Repac) (M-Ras-regulated Rap GEF) MRGEF).

[Source:Uniprot/SWISSPROT;Acc:Q92565]

GTCGGGTGAGTCTAAGTGGCTCACCTTGACATACATCTGCTAACTTAATCTCTCTCTTAATCATTTCTATCTTGCCAGCA
ATATTCTTTTGACCTTTTTA

In100 candidate380 (in intron):

LOCUS ENSG00000136237

DESCRIPTION Rap guanine nucleotide exchange factor 5 (Guanine nucleotide exhange factor for Rap1) (Related to Epac) (Repac) (M-Ras-regulated Rap GEF) MRGEF).

[Source:Uniprot/SWISSPROT;Acc:Q92565]

TCCATACACATACTTTCTGAAGCTATTTCGTCTGTTAACCACATCTTAGAGTCTGTTCTGTTGTATCTGTTCATTCAGTT
GCTCCATTAAATTTTTTTTT

In100 candidate381 (in intron):

LOCUS ENSG00000156011

DESCRIPTION PH and SEC7 domain-containing protein 3 (Pleckstrin homology an SEC7 domain-containing protein 3) (Exchange factor for ADP-ribosylation fator guanine nucleotide factor 6) (Hepatocellular carcinoma- associated antien 67).

[Source:Uniprot/SWISSPROT;Acc:Q9NYI0]

CTTTTGTAAACTTTGGTCTGAGGGTTGCTTCTTATTCTTTTAACTGTGTGTTGGGTTAACTTCCTCTATATGCTTCAGGT
TGCCTAAACTTTCTCTATAT

In100 candidate382 (in intron):

LOCUS ENSG00000197195

DESCRIPTION Churchill protein. [Source:Uniprot/SWISSPROT;Acc:Q8WUH1]

AAATCATTTTAACTGCTCCTTTGTGTTTTCTCTTAGTTTTCTTTCCTGTTTTCTCGAAGTGATACTCCTCAGCTTTTGTT

CTGTGGTCTGGAACCTTCTT

In100 candidate383 (in intron):

LOCUS ENSG00000105889

DESCRIPTION Similar to Six transmembrane epithelial antigen of prostate.

[Surce:Uniprot/SPTREMBL;Acc:Q6NZ63]

TTTATCCTTTCTCTGTAATCCTTTAACTGTACTTTTCTCTTGACGTTTGTTCCAGTTGTTAATTTATTGCCTTGCAGCTC

CAAACCGTCCTTTTTGTCTT

In100 candidate384 (in intron):

LOCUS ENSG00000104611

DESCRIPTION SH2 domain-containing protein 4A (Protein SH(2)A).

[Source:Unipot/SWISSPROT;Acc:Q9H788]

ACATATATATTTATAACTGTTCTTGCTGTATTGAACTCTTTATTATTGTATACTGGCCTTCTTTGTCTCTTCTTACAGTT

TTCAGCTTCAGGTCTGCTTT

In100 candidate385 (in intron):

LOCUS ENSG00000104611

DESCRIPTION SH2 domain-containing protein 4A (Protein SH(2)A).

[Source:Unipot/SWISSPROT;Acc:Q9H788]

AACCCCTTTGTCTTCCTGAATTCCCTCTTTAATAACTCCAATATTTACTCTTTTGATGTTGTCCCACAGACCCCATAAGC

TTTCTTCATGTCATTGTTTT

In100 candidate386 (in intron):

LOCUS ENSG00000125954

DESCRIPTION Protein farnesyltransferase subunit beta (EC 2.5.1.58) (CAAX fanesyltransferase subunit beta) (RAS proteins prenyltransferase beta) (FTasebeta). [Source:Uniprot/SWISSPROT;Acc:P49356]

TAAAGTAACACTAATTTAGAAATGCTGGTTTTCCTCCTCTTCTTATTTATAAAATGTTTCTTTTCAGACTCTTCACATGC
ATCTTTGACTTCACTATTTT

In100 candidate387 (in intron):

LOCUS ENSG00000125952

DESCRIPTION Protein max (Myc-associated factor X).
[Source:Uniprot/SWISSPRO;Acc:P61244]

AAAGAAAAATAACCTTCCTTCAATGCTGCTATCCTGCTATTACAAACTACCATTTTTCACTTTTTTTTTTTTATACAGGG
TCTCACTCTTTTTTTTCAGG

In100 candidate388 (in intron):

LOCUS ENSG00000147408

DESCRIPTION Chondroitin beta-1,4-N-acetylgalactosaminyltransferase 1 (EC 2..1.174) (beta4GalNAcT-1). [Source:Uniprot/SWISSPROT;Acc:Q8TDX6]

CAAGTGATGAATAACTGATGTAGCCTGTTTTTTTATTTTTATTTTTTGAGTGCAGTATTTTCTTGTTCACTAACTTTTCT
ACAAATCATTTTCAGGCTTT

In100 candidate389 (in intron):

LOCUS ENSG00000147408

DESCRIPTION Chondroitin beta-1,4-N-acetylgalactosaminyltransferase 1 (EC 2..1.174) (beta4GalNAcT-1). [Source:Uniprot/SWISSPROT;Acc:Q8TDX6]

TCTCTGTTTTTTTAACACCCTTAGTCTTTTATCTGCTTTTACTTAAACCTTTATTGTGTCAGTAATTTGATTTTCTGACA

TATCTATTCTGTACCTTCCT

In100 candidate390 (in intron):

LOCUS ENSG00000122550

DESCRIPTION Kelch-like protein 7. [Source:Uniprot/SWISSPROT;Acc:Q8IXQ5]

AGCTGACTTGTTAACCTGCTTAAGTTGGATCTTTTCCTTTCTACATCAGGTAGCTGACCTTTTTAGATTAGAATACCTTT

CTTTTTCTTTTCCTTATGAT

In100 candidate391 (in intron):

LOCUS ENSG00000122550

DESCRIPTION Kelch-like protein 7. [Source:Uniprot/SWISSPROT;Acc:Q8IXQ5]

AGGTTTGTCCATTTTGTTTTAACTTTTCAAAAAAACAACTTTTTGTTTCATTGATCTTTTGTATTTTTTTCATTTCAGTT

TTATTTTTTCTCTGATCTTT

In100 candidate392 (in intron):

LOCUS ENSG00000122550

DESCRIPTION Kelch-like protein 7. [Source:Uniprot/SWISSPROT;Acc:Q8IXQ5]

AACATTTGTATGTGTAGTCTAATAAATTAACATATTCTTATATGTTTTTTGACTGTATCTTTGCATTGTCCTTTTCAGTT

ATATTTTTTTCCTTACTGAA

In100 candidate393 (in intron):

LOCUS ENSG00000183066

DESCRIPTION WBP2 N-terminal like [Source:RefSeq_peptide;Acc:NP_689826]

TAATTTTTCTTATTTCTGTCCTTCTGCTAACTTTGGGTCGAGTTTGTTCCTCTTTTTCTAGTTTCTTGATTTGTACAGTT

AGGTTTTTTTTTTTTTTTC

In100 candidate394 (in intron):

LOCUS ENSG00000136243

DESCRIPTION Nucleoporin-like 2 (NLP-1) (hCG1) (NUP42 homolog).

[Source:Unipot/SWISSPROT;Acc:O15504]

GCATTTTCCATGTTAAGCATTTAACTTTGCCTTTTTTAATGCACTTTTTTCTTCTCTTTTCTTTTTTTGAGTCAGAGTCT

TACTTTGTCATCCAGGCTTT

In100 candidate395 (in intron):

LOCUS ENSG00000033170

DESCRIPTION Alpha-(1,6)-fucosyltransferase (EC 2.4.1.68) (Glycoprotein 6-

alha-L- fucosyltransferase) (GDP-fucose--glycoprotein fucosyltransferase) (GD-

LFuc:

N-acetyl-beta-D-glucosaminide alpha1,6-fucosyltransferase) (alpha1-6ucT)

(Fucosyltransferase 8). [Source:Uniprot/SWISSPROT;Acc:Q9BYC5]

TCTTGAATTTCTCCAAGATTTGTATCTTGAAACCCTTTAACCCACCCCCCCCTTTTTTTTTTTTGCCATATCCACAGTC

TTTTTCGTGATTTCCTTGAT

In100 candidate396 (in intron):

LOCUS ENSG00000033170

DESCRIPTION Alpha-(1,6)-fucosyltransferase (EC 2.4.1.68) (Glycoprotein 6-alha-L- fucosyltransferase) (GDP-fucose--glycoprotein fucosyltransferase) (GD-LFuc:

N-acetyl-beta-D-glucosaminide alpha1,6-fucosyltransferase) (alpha1-6ucT) (Fucosyltransferase 8). [Source:Uniprot/SWISSPROT;Acc:Q9BYC5]

AGAAACCCTGACCCAACGGCTACCTTTGGGTAAGTGTTGGGATCCTGTAACAAAACTGCGAATTCTATCACTACAGTGTT

TATCTCATTCATCTGCTTTT

In100 candidate397 (in intron):

LOCUS ENSG00000033170

DESCRIPTION Alpha-(1,6)-fucosyltransferase (EC 2.4.1.68) (Glycoprotein 6-alha-L- fucosyltransferase) (GDP-fucose--glycoprotein fucosyltransferase) (GD-LFuc:

N-acetyl-beta-D-glucosaminide alpha1,6-fucosyltransferase) (alpha1-6ucT) (Fucosyltransferase 8). [Source:Uniprot/SWISSPROT;Acc:Q9BYC5]

GGAAGATAAGACTTTTTTAAATATATTGAAATAACTTGGTTAATAAATACTTTTTTTCTCTTTTTAGAAGCCAGGAACCT

GTTCTCTTTCCATAGTGTCT

In100 candidate398 (in intron):

LOCUS ENSG00000033170

DESCRIPTION Alpha-(1,6)-fucosyltransferase (EC 2.4.1.68) (Glycoprotein 6-alha-L- fucosyltransferase) (GDP-fucose--glycoprotein fucosyltransferase) (GD-LFuc:

N-acetyl-beta-D-glucosaminide alpha1,6-fucosyltransferase) (alpha1-6ucT) (Fucosyltransferase 8). [Source:Uniprot/SWISSPROT;Acc:Q9BYC5]

TGGTCCTGGACTTTTTTTTTTTGTTGGTAACTTTTTAATTACCATTTCAATCTTGTTGCTTGTTATTGTTCTGTTCAGGG

TATCTAATTCTTCCTGATTT

In100 candidate399 (in intron):

LOCUS ENSG00000033170

DESCRIPTION Alpha-(1,6)-fucosyltransferase (EC 2.4.1.68) (Glycoprotein 6-

alha-L- fucosyltransferase) (GDP-fucose--glycoprotein fucosyltransferase) (GD-

LFuc:

N-acetyl-beta-D-glucosaminide alpha1,6-fucosyltransferase) (alpha1-6ucT)

(Fucosyltransferase 8). [Source:Uniprot/SWISSPROT;Acc:Q9BYC5]

ATCTTCACATTTAAAGTGGATTTTTGTACCTAACATATAGTTGGTCTTCTTTTTTTTTTAAATCCACTTGGACAGTCTCT

GCCTCTTAACTGGTGTATTT

In100 candidate400 (in intron):

LOCUS ENSG00000033170

DESCRIPTION Alpha-(1,6)-fucosyltransferase (EC 2.4.1.68) (Glycoprotein 6-

alha-L- fucosyltransferase) (GDP-fucose--glycoprotein fucosyltransferase) (GD-

LFuc:

N-acetyl-beta-D-glucosaminide alpha1,6-fucosyltransferase) (alpha1-6ucT)

(Fucosyltransferase 8). [Source:Uniprot/SWISSPROT;Acc:Q9BYC5]

CTTAAGTGTTTTGTTTTTGCTTCCTTTAACATGACTCTGCGTTTTCATTTTCTTCTCTTACGATGTGGCCAGAATTTCTA

CTTAATAATTCTTATATCTT

In100 candidate401 (in intron):

LOCUS ENSG00000171877

DESCRIPTION FERM domain-containing protein 5.

[Source:Uniprot/SWISSPROT;AccQ7Z6J6]

CTATTTCTTCTATAACCCTATTTACATTCCTGTTACCACTTTTTGTTACTTTGCTGCATTTTCATCTTCGTTAGCCAGTT

CCCTCTTTCAGTTATCATTT

In100 candidate402 (in intron):

LOCUS ENSG00000136231

DESCRIPTION insulin-like growth factor 2 mRNA binding protein 3

[Source:Refeq_peptide;Acc:NP_006538]

GGAGTTTAAGGGTCACCTCTACTATATATTGTTAGAATATTTAACTTCACCACTAACTTCATACCAATTTATTTTCAGTC

TTAATTTTTCCTGGCTTTAT

In100 candidate403 (in intron):

LOCUS ENSG00000171723

DESCRIPTION Gephyrin. [Source:Uniprot/SWISSPROT;Acc:Q9NQX3]

AGTAACACTACTCTTTCCTTGTTTATCACTGATCTTTGTAGCCACTCATTCTCTACAGAATTTTTCTTCCTCTGTGTGGT

CTTAAATATCTGTGTTTTTT

In100 candidate404 (in intron):

LOCUS ENSG00000171723

DESCRIPTION Gephyrin. [Source:Uniprot/SWISSPROT;Acc:Q9NQX3]

TGTTTTCTTCTAACTTCTTTTTAATTTTTTTGTATTTATTTATTTATTGCCCTCAAACATTCTCCTATCCAGTTATTGTT

CTTTTATAAAGCTCATCTCT

In100 candidate405 (in intron):

LOCUS ENSG00000171723

DESCRIPTION Gephyrin. [Source:Uniprot/SWISSPROT;Acc:Q9NQX3]

TAACCTCTGTTTCTTATTTTCTTTCTTTTTATCTCTCTTTGCTGAATTCTATATCACTTCTTGAGACCTGTCTTCCAGTT

TATGAATTTTCCTTTCATCT

In100 candidate406 (in intron):

LOCUS ENSG00000171723

DESCRIPTION Gephyrin. [Source:Uniprot/SWISSPROT;Acc:Q9NQX3]

CATATGTTCTTTGGCTCTTTGAGATGTAGACTCTGTAGAGCTATTTAACTACCATTATTTTCTGGTGATTTTTTTCAGGT

ACCTTTCTTTATGATTTATG

In100 candidate407 (in intron):

LOCUS ENSG00000171723

DESCRIPTION Gephyrin. [Source:Uniprot/SWISSPROT;Acc:Q9NQX3]

CCACTGGCTCCAATGGTCACACCCTAGCTCCTTTCATTACTAATAACTATCACTCCTCTATAACTTTCATTTCAGTTATC

ACTCTCTTCAGTTGTCACTT

In100 candidate408 (in intron):

LOCUS ENSG00000171723

DESCRIPTION Gephyrin. [Source:Uniprot/SWISSPROT;Acc:Q9NQX3]

TGTTTTCTGGTTGTTTTATAACTCCTTTCTCCCTTTTTTACTTTCTTCATATGTGGTTTCAGTGATTTTCTCTGGTAGTA

TCTTTTAATTTGTTGCTTTT

In100 candidate409 (in intron):

LOCUS ENSG00000171723

DESCRIPTION Gephyrin. [Source:Uniprot/SWISSPROT;Acc:Q9NQX3]

TTTCTGATATAACCCATTTAGGTATTATATGTAATCAATCTTTTTTGTTCTTGTTTTTGTTTTTCTTTCTAAGACAGAGT

TTTTCTCTCTGGCTCAGGCT

In100 candidate410 (in intron):

LOCUS ENSG00000171723

DESCRIPTION Gephyrin. [Source:Uniprot/SWISSPROT;Acc:Q9NQX3]

AGATGCCATACCTTTTATTTGTAATCCCTGACAAATTTAACGTTAGGCCAAAAACTGTTTTATTTTGCTTTCCAGACTTT

CCTTTTTTTTCCCATAGAGTA

In100 candidate411 (in intron):

LOCUS ENSG00000171723

DESCRIPTION Gephyrin. [Source:Uniprot/SWISSPROT;Acc:Q9NQX3]

AACAAGTCTTTCAAGTAAACCAAATCTATAACTCCAATCCATATCTTCTTCCTAAACTTCAGTTTTTTTTCCAATTGTCT

GTTGTAGATCTTTTCTTAAA

In100 candidate412 (in intron):

LOCUS ENSG00000171723

DESCRIPTION Gephyrin. [Source:Uniprot/SWISSPROT;Acc:Q9NQX3]

TGTGATAACTTTTATACTCTTTTTTTCAATCTGTTATTTACCACTCACTTCAACTTTAGCAGTTCATCAATTCCTAACAT

TTTATTTCCTCCCTTAAAAT

In100 candidate413 (in intron):

LOCUS ENSG00000171723

DESCRIPTION Gephyrin. [Source:Uniprot/SWISSPROT;Acc:Q9NQX3]

CTTGATAAATTTTAAAATAATTTATTGTGCATCTTTTTTTTAACTTTAAAATTATCTTCTGATCATTCTGTTCAGTTCTC

TATTTTTTGGCACAGATTCC

In100 candidate414 (in intron):

LOCUS ENSG00000169193

DESCRIPTION coiled-coil domain containing 126

[Source:RefSeq_peptide;Acc:NP620126]

CTTTTTGCCTTAACATTTTCTGTTTTTATCCCTGCCTCACTTTACCATTTTTCTTTTATCTGTTTTTGTAATATTCAGTG

CTATTGTCTTCTATTCTGTC

In100 candidate415 (in intron):

LOCUS ENSG00000137770

DESCRIPTION CTD (carboxy-terminal domain, RNA polymerase II, polypeptide

A)small phosphatase like 2 [Source:RefSeq_peptide;Acc:NP_057480]

TATCAATTTCATTATTCGTTCAAAGGGATTTTTAATATTAACTGTTCATTCTGTTTAATTGATTTCCACTATTTTCAGTA

TTTTCTGTTTTCAGTTTGAT

In100 candidate416 (in intron):

LOCUS ENSG00000196335

DESCRIPTION Serine/threonine-protein kinase 31 (EC 2.7.11.1)

(Serine/threonne- protein kinase NYD-SPK) (Sugen kinase 396).

[Source:Uniprot/SWISSPROT;Ac:Q9BXU1]

TTAATGATACCTATTTGGTAAATTTCTCATTTATAACCTGAATTGTTTTTCTGATTTCTTTATATTATTTTTCAGAATTA

TCTTTTATCTCACTGAACTT

In100 candidate417 (in intron):

LOCUS ENSG00000196335

DESCRIPTION Serine/threonine-protein kinase 31 (EC 2.7.11.1)

(Serine/threonne- protein kinase NYD-SPK) (Sugen kinase 396).

[Source:Uniprot/SWISSPROT;Ac:Q9BXU1]

TATTTCTATGCGTCAATAACTTTTTTAAAGGCATTACATTTTATTTCTTTAACAGAGTGTGACCAAACATATTTGTTTTT

ATTTTTTATTTTTTTATTTT

In100 candidate418 (in intron):

LOCUS ENSG00000072415

DESCRIPTION MAGUK p55 subfamily member 5.

[Source:Uniprot/SWISSPROT;Acc:Q8NR9]

TATATGTATTTCGTAAATGTATTTGTCTTAAGTTTCTTCTACTTTAACTTTCTGTTTATTATTTCTTCATTCTACCAGAA

TTTATAGTTTTTCCAGTCTT

In100 candidate419 (in intron):

LOCUS ENSG00000072415

DESCRIPTION MAGUK p55 subfamily member 5.

[Source:Uniprot/SWISSPROT;Acc:Q8NR9]

AGAGTTCTAGGCATTGGAAAGTAGGATTTTCTGATAAAGTAACTCTTGGTGATTGCTTTCTGTTGCCTGTTTCAGAGTCC

ATTCTTTTACGTTTTAGACT

In100 candidate420 (in intron):

LOCUS ENSG00000134001

DESCRIPTION Eukaryotic translation initiation factor 2 subunit 1 (Eukaryoti translation initiation factor 2 subunit alpha) (eIF-2-alpha) (EIF- 2alpha)(EIF-2A). [Source:Uniprot/SWISSPROT;Acc:P05198]

TCATTTTAACGACTTTGTTGCCATTATTGTGAGCTTTTGCCTTGTTTTTCCTTCCATCCATCCAGCCTTGGATCAATGTT
CCTGCCTTCTCTGTACTTGT

In100 candidate421 (in intron):

LOCUS ENSG00000134001

DESCRIPTION Eukaryotic translation initiation factor 2 subunit 1 (Eukaryoti translation initiation factor 2 subunit alpha) (eIF-2-alpha) (EIF- 2alpha)(EIF-2A). [Source:Uniprot/SWISSPROT;Acc:P05198]

AGCCTTATGTATCATAACTTTTTTACAATAGTTCTACCTTTACTTTTTACAGTGCTACTCCTCTTTTAGTCTTTATTTCA
TATTTTCATTTGCTCACTCA

In100 candidate422 (in intron):

LOCUS ENSG00000105926

DESCRIPTION MAGUK p55 subfamily member 6 (Veli-associated MAGUK 1) (VAM-1).[Source:Uniprot/SWISSPROT;Acc:Q9NZW5]

TGCTAACAAAAATAAAATGTTAAAATTCTCTTCTATTCTCAGCACCATCTGTTTTTCCCCCAGTTTAGTTTGCTTAGTTT
ACCCGCTGTCACTGATGTTT

In100 candidate423 (in exon):

LOCUS ENSG00000070882

DESCRIPTION Oxysterol-binding protein-related protein 3 (OSBP-related proten 3) (ORP-3). [Source:Uniprot/SWISSPROT;Acc:Q9H4L5]

TGTATTGGCATAATTTGTTTGAATGTCTTTATTTTGATATTAACACCGGCATAAATCTATTTTTGCTACCAGATGATAGC

TATTTTCATTTGCTCTATTT

In100 candidate424 (in intron):

LOCUS ENSG00000109684

DESCRIPTION mast cell immunoreceptor signal transducer [Source:RefSeq_peptie;Acc:NP_443196]

AGTTTTTTCTTATTTAACCTGCTTTGGATACGTTGGATATCCTGATTCTCTATTTTGTTGTTTTTCACTATTTCTCAGTA

CTCTGTCATTATCTCTTTGA

In100 candidate425 (in intron):

LOCUS ENSG00000182185

DESCRIPTION DNA repair protein RAD51 homolog 2 (R51H2) (RAD51-like protein) (Rad51B). [Source:Uniprot/SWISSPROT;Acc:O15315]

ATAAGAAAACATAAAGCATAATAAAAGTAGGACTCCTTTCTTTTAACCTGCCTAACTCATGTTTATCCTTTATCTCAGGT

GTCATCTTCTTCATTTGGCC

In100 candidate426 (in intron):

LOCUS ENSG00000182185

DESCRIPTION DNA repair protein RAD51 homolog 2 (R51H2) (RAD51-like protein) (Rad51B). [Source:Uniprot/SWISSPROT;Acc:O15315]

TAGACACCAATGGCGTCTACTCTCCCTTATCATATAACTATCTTAATATTTGAAGGTTGTTTTCATCTTCCTCTTCAGTT

TTTCTCTTTTCTCTTTTACC

In100 candidate427 (in intron):

LOCUS ENSG00000182185

DESCRIPTION DNA repair protein RAD51 homolog 2 (R51H2) (RAD51-like protein)

(Rad51B). [Source:Uniprot/SWISSPROT;Acc:O15315]

TGCCTTTACAACTGGACTTGGAGATAAATAATAACATTATACCCATTTTTTTTTATCATGAAGCCTTTGACTTTCAGGG

GATATCTATTCTTTATTCCT

In100 candidate428 (in intron):

LOCUS ENSG00000182185

DESCRIPTION DNA repair protein RAD51 homolog 2 (R51H2) (RAD51-like protein)

(Rad51B). [Source:Uniprot/SWISSPROT;Acc:O15315]

GTTTTAACGTGCCTGTCTGTTCATTCTGTCTTTTCAACTCTGTCATTTTCAGGCATGTTTCTATTAATTGCCTTTTTTCC

TGATATGGGTCACATTTTTC

In100 candidate429 (in intron):

LOCUS ENSG00000139990

DESCRIPTION WD repeat protein 22 (Breakpoint cluster region protein 2)

(BCR2). [Source:Uniprot/SWISSPROT;Acc:Q96JK2]

CCCTTTAATCTTAATAACAATGAAAATTATTATTTAACTTTTTTTTTTTATCTTGTAACGTATGCCAGGCCTTCTGCTA

AGCACTTTACATGTCTTATT

In100 candidate430 (in intron):

LOCUS ENSG00000171763

DESCRIPTION spermatogenesis associated 5-like 1

[Source:RefSeq_peptide;Acc:P_076968]

AATATGGTTTTTAACTCTTACATAATACTCCATTGTTTCTTGTTTTATTTACTCATTCCCCAATTCATAGACATTCAGAA

TCTTTTTTATATCTATTTTA

In100 candidate431 (in intron):

LOCUS ENSG00000100647

DESCRIPTION Uncharacterized protein KIAA0247 precursor.

[Source:Uniprot/SWISPROT;Acc:Q92537]

TGATTCTACCACCCTAACATAATGTGTATTCTGTTTTTTCCATCTTCAGATGGTAGTACCTGTACATTCAGCTTTGTATT

TTTTTCACTTAGCTTTTTTT

In100 candidate432 (in intron):

LOCUS ENSG00000118564

DESCRIPTION F-box/LRR-repeat protein 5 (F-box and leucine-rich repeat proten

5) (F-box protein FBL4/FBL5) (p45SKP2-like protein).

[Source:Uniprot/SWISPROT;Acc:Q9UKA1]

TCCATCTTCCTAGAGCTTGTTTCTTATGGATCTTCCTCATTAACCGTCGGTTATTCTCTTTCTTTCATGTGTTTGCAGCC

TTACTTCTGCTGACTACTTT

In100 candidate433 (in intron):

LOCUS ENSG00000137767

DESCRIPTION Sulfide:quinone oxidoreductase, mitochondrial precursor (EC 1.--

.-). [Source:Uniprot/SWISSPROT;Acc:Q9Y6N5]

GGTCCCTGACATAACTTCCCTTTTTCTGCTCACATAATTCCTTTTTCTTCAGCTGTCTAGATGTCAACTTTGACTTTTTT

TTTTCTTCCAGAAGCCTTTC

In100 candidate434 (in intron):

LOCUS ENSG00000004468

DESCRIPTION ADP-ribosyl cyclase 1 (EC 3.2.2.5) (Cyclic ADP-ribose hydrolase1) (cADPr hydrolase 1) (Lymphocyte differentiation antigen CD38) (T10) (Acue lymphoblastic leukemia cells antigen CD38). [Source:Uniprot/SWISSPROT;AccP28907]

TTTTATTTTTTAATTCCCTGATCATTAACATAAGTAATTATCTTTTTATGTGTTTATTTGTCATTCAGGTTTCTTCTGT

GAAAAGCCTATTTCTGTCTT

In100 candidate435 (in intron):

LOCUS ENSG00000186976

DESCRIPTION DJ-1-binding protein (CAP-binding protein complex-interacting potein 1). [Source:Uniprot/SWISSPROT;Acc:Q5THR3]

AGCTGGGAACATGGGAGGAAAGCCCCATGAAACTCATAACTTTGTCATTCTTCGAATTTTGATTTCTCCCTCCATCAGCC

TTCTATTATTTACCTCAGAT

In100 candidate436 (in intron):

LOCUS ENSG00000106052

DESCRIPTION Tax1-binding protein 1 (TRAF6-binding protein).

[Source:UniprotSWISSPROT;Acc:Q86VP1]

CTTCTTGAACATATGGAATACACATGTTTAAATGTCCGTTCATTAACTTGATCATCTGGCTCATTTCTGTATCAGTTTTG

ATTGATTGGCTTTTCTACTC

In100 candidate437 (in intron):

LOCUS ENSG00000106052

DESCRIPTION Tax1-binding protein 1 (TRAF6-binding protein).

[Source:UniprotSWISSPROT;Acc:Q86VP1]

TTCTCCCATGAATTATGAGTTTTTCCACTCTGGCTGGTAGGAATAACCACTATTCCTGGCCCTTTTTGAGTTCCAGGCAC

TGTTCCCTGTAGTCCTTTTT

In100 candidate438 (in intron):

LOCUS ENSG00000106052

DESCRIPTION Tax1-binding protein 1 (TRAF6-binding protein).

[Source:UniprotSWISSPROT;Acc:Q86VP1]

GCTGTGTGATCTATTACTAATAGTGAAATGCCTAACATTGGATATTTCAAGTTCTTGTCTTGCTTTTCTTTTTCCCAGTA

GCTTTTTTTTTTTTTAACT

In100 candidate439 (in intron):

LOCUS ENSG00000146592

DESCRIPTION cAMP response element-binding protein 5 (CRE-BPa).

[Source:Unipot/SWISSPROT;Acc:Q02930]

ATTGCGCTTGTCTTTGTATTTATAAATAACATTCTTTCGGGTGCTTTCCCATGCATTATTTGCAGTTAAATGGTTTCCTT

GCTAATACCTCTTACTTTCC

In100 candidate440 (in intron):

LOCUS ENSG00000007062

DESCRIPTION Prominin-1 precursor (Prominin-like protein 1) (Antigen AC133)

CD133 antigen). [Source:Uniprot/SWISSPROT;Acc:O43490]

AACTGAATTTTAGTACTTTTTAGGTAAAAGATAACTCAAAGATTCTCTTTTTCTCTTCAGTGCCTCGTGTGTTATTATTA

AAGTTCTTTTTTTTTGTTTT

In100 candidate441 (in intron):

LOCUS ENSG00000007062

DESCRIPTION Prominin-1 precursor (Prominin-like protein 1) (Antigen AC133)

CD133 antigen). [Source:Uniprot/SWISSPROT;Acc:O43490]

GTGAATTTTTTTTTCTTGGTACTGATTTTTCTATACTTGTTTTAACTACTTTTTTCCTCTTGCTTAATCCTCTCAGCTTC

CCTAACCAGCCTTTTTATTT

In100 candidate442 (in intron):

LOCUS ENSG00000169762

DESCRIPTION transmembrane anterior posterior transformation 1

[Source:RefSe_peptide;Acc:NP_699196]

GTGTGCTACATCAAAATAACTTTTGTAAGGAATTAATCTCTACTTCTGTACCCTTTTAGTTCCCTTCAGTTCTCTTTTAC

TAGAAACAGGTTTTTTTTCC

In100 candidate443 (in intron):

LOCUS ENSG00000100731

DESCRIPTION Pecanex-like protein 1 (Pecanex homolog).

[Source:Uniprot/SWISSROT;Acc:Q96RV3]

TCTACTGAGAAATTAGATGTTAGTCTAATGGGGGTTTCCTTGTATGTAACTTAACCCTTTTTCTCTTGCTGTTTTCAGAA

TTCTCTTTTTGTCTTTGACT

In100 candidate444 (in intron):

LOCUS ENSG00000136193

DESCRIPTION Secernin-1. [Source:Uniprot/SWISSPROT;Acc:Q12765]

CTAGTTTTCTCTTGATTAGCAAAATGAAATGTATAGAATAACAAGAAGTTTTTTTTTTTTAGCTTATGTTTTGACAGCT

CTCCAGTATTTTTTACTGTA

In100 candidate445 (in intron):

LOCUS ENSG00000106080

DESCRIPTION FK506-binding protein 14 precursor (EC 5.2.1.8) (Peptidyl-proly

cis- trans isomerase) (PPIase) (Rotamase) (22 kDa FK506-binding protein) (KBP-

22). [Source:Uniprot/SWISSPROT;Acc:Q9NWM8]

AGGTTCCTGGGATTTTAACAAAGCTCTTTTCACTTTTAAAGATTTTTAATTTCCCTTCTCAGAAATTCATTTTAATTTCT

CCCCTCCCATGAAATGTCTT

In100 candidate446 (in intron):

LOCUS ENSG00000197555

DESCRIPTION Signal-induced proliferation-associated 1-like protein 1 (Highisk

human papilloma viruses E6 oncoproteins targeted protein 1) (E6- targetd

protein 1). [Source:Uniprot/SWISSPROT;Acc:O43166]

TATTGTTATCTGTGCCCCAACACCATCCACATAAAAAGATCAAAGTAACTACCTTGCTTGCTGATTTCCATATTCCAGTG

AGTTTCTGTGCTCTTTGTTT

In100 candidate447 (in intron):

LOCUS ENSG00000197555

DESCRIPTION Signal-induced proliferation-associated 1-like protein 1 (Highisk human papilloma viruses E6 oncoproteins targeted protein 1) (E6- targetd protein 1). [Source:Uniprot/SWISSPROT;Acc:O43166]

CTTTTTTTTTTCCCCCTAACCTTTTTTTTTCCTTCTGTGCTTCAGTTTGGATAATTTCTATTAATATATCTTAAAGCTCC

TTGTGTTTTTCTTTTCTTCC

In100 candidate448 (in intron):

LOCUS ENSG00000138944

DESCRIPTION OTTHUMP00000028979 (KIAA1644 protein). [Source:Uniprot/SPTREMBLAcc:Q5JZ71]

AACCTGTTGATTCTGTTAAATTTTCTATGTAAGTTATCCACAATTAACTGTAATTTTGGTTCTTCTATTCCAGTCCTATT

CTATGCTTATTTTTCTTGTC

In100 candidate449 (in intron):

LOCUS ENSG00000106086

DESCRIPTION pleckstrin homology domain containing, family A (phosphoinositie binding specific) member 8 [Source:RefSeq_peptide;Acc:NP_116028]

TAGTTCTGCCTATTCTAAAACTTGATATAACTGGAATATTATACTTTTTTGCCTCTGGCTTTTCTCATACAGTATGTTTT

TGAGATCCGTCCTTGTTTTC

In100 candidate450 (in intron):

LOCUS ENSG00000106086

DESCRIPTION pleckstrin homology domain containing, family A (phosphoinositie

binding specific) member 8 [Source:RefSeq_peptide;Acc:NP_116028]

CCTCCATCTTGGTCACTAACTAGCCTTCATTTGACCTTTTTGGTTTCCTCTTCAATCAGACTAGACAGTTGAACTCTATT

ACTGGTTCTCCCCTTTTTTT

In100 candidate451 (in intron):

LOCUS ENSG00000166147

DESCRIPTION Fibrillin-1 precursor. [Source:Uniprot/SWISSPROT;Acc:P35555]

GGAGACACATTTCCTTTTTCCTAACATTGGACTCTTTCCTTCCTGCTAAGTCCTGCTTGTTTTTTTTTTCCTTCACAGTA

TCTCTTTTATCTGTCCATTT

In100 candidate452 (in intron):

LOCUS ENSG00000182732

DESCRIPTION Regulator of G-protein signaling 6 (RGS6) (S914).

[Source:Uniprt/SWISSPROT;Acc:P49758]

GGGAGCCTCTTCAACTTGGCTCTTGATTCCTTTTGGTATAACTCTAGCACTATTTGATTCCTTCCTTTTTTATTACAGGC

TCATCTTGTGTATTTCCTGC

In100 candidate453 (in intron):

LOCUS ENSG00000182732

DESCRIPTION Regulator of G-protein signaling 6 (RGS6) (S914).

[Source:Uniprt/SWISSPROT;Acc:P49758]

GCCCCTGCCCCATGCTTTTGTTTTTTATTTGTCTAACTTTTTTTTTTTTTTTTGGAGATTTTCCATTTCAGGCTTTTGT

TGCTTTTCTCAAATGTCAAC

In100 candidate454 (in intron):

LOCUS ENSG00000182732

DESCRIPTION Regulator of G-protein signaling 6 (RGS6) (S914).

[Source:Uniprt/SWISSPROT;Acc:P49758]

GAGGAGAGGAAGGAAGGAGTGGCCAATTCATTTTCGTAACAAACACCCAATTTTGTTTTTCTCCGTGGACTATCCAGAT

GCATGCTTCTTTTACAGTTC

In100 candidate455 (in intron):

LOCUS ENSG00000182732

DESCRIPTION Regulator of G-protein signaling 6 (RGS6) (S914).

[Source:Uniprt/SWISSPROT;Acc:P49758]

AAAATAGGTGAATATAGCACAATAAGATATTGTAAGAGAGAGACTATATAACTTTTATTTACATAACTTTTCTTACAGTA

TATTGCTATGATTTTTCTCT

In100 candidate456 (in intron):

LOCUS ENSG00000186654

DESCRIPTION Rho GTPase-activating protein 8.

[Source:Uniprot/SWISSPROT;Acc:9NSG0]

CCATCTGATCTTAGGCTTTACTTTGTGGGAAGTTTTAAAATTACTAATGTAACCTCTTTGCTTGTTATATTTATTCAGAT

TTTATATTTCTTCTTGAGTC

In100 candidate457 (in intron):

LOCUS ENSG00000163257

DESCRIPTION CDNA FLJ20280 fis, clone HEP03525 (Chromosome 4 open reading frme 30). [Source:Uniprot/SPTREMBL;Acc:Q9NXF7]

TATGTATTAAATGGTTTTACTTTTCATTTTTTTCTTAACCTTGTTCTTAATTTTTTCTTAATTCTTATTCCAATACAGTT

TTACTTTTTTTCATATAATG

In100 candidate458 (in intron):

LOCUS ENSG00000205683

DESCRIPTION Zinc-finger protein DPF3 (cer-d4). [Source:Uniprot/SWISSPROT;Ac:Q92784]

CCCTGTGTACCTCATTTCTTCTCTTTCATGGTTTCCTAACTGGGGCTTTCACTGTTCTTTCCTTACTACTGGGCTCAGTA

TTTTGTTCTTTTTCCCTTAA

In100 candidate459 (in intron):

LOCUS ENSG00000205683

DESCRIPTION Zinc-finger protein DPF3 (cer-d4). [Source:Uniprot/SWISSPROT;Ac:Q92784]

GCTAACCTGTCTTGCAATGTGGGCAAAACCATCATATCTATTTTTTAGTTTTCTTTTTCTTTTTCCTTTTTCCCCCAGCT

TGCTTTTTTGTTTTCCTGGG

In100 candidate460 (in intron):

LOCUS ENSG00000106105

DESCRIPTION Glycyl-tRNA synthetase (EC 6.1.1.14) (Glycine--tRNA ligase)

(GlRS). [Source:Uniprot/SWISSPROT;Acc:P41250]

TCTTTTGGTTTGGGATATTAGCATTTAGATATTTACAAATTAACATATTTGGATTTTTTTCTTTTTAAATCATCCAGTTC

TTCAGTTTCCCTGCTGTAGT

In100 candidate461 (in intron):

LOCUS ENSG00000120645

DESCRIPTION IQ motif and Sec7 domain-containing protein 3.

[Source:Uniprot/WISSPROT;Acc:Q9UPP2]

AGTCTTAACTTTGGTGTCTCTCTTTGATCTGTCTTTTCCAGTCTCTTGCTCTCGCTCGTTTTCTTTTTCTATCTATCTCT

ATCATCCTGTTTCCTTTTTC

In100 candidate462 (in intron):

LOCUS ENSG00000165861

DESCRIPTION Zinc finger FYVE domain-containing protein 1 (Double FYVEcontaning

protein 1) (Tandem FYVE fingers-1) (SR3).

[Source:Uniprot/SWISSPROT;Ac:Q9HBF4]

TCTAAAAGAGGAGTTTCTCCCTACTCTTGCATCTAAAACATGCTAACGTTGGATTTTTTTTTTTTTTTTAACAGTTAC

TGTTATTTTCCCACACTCTT

In100 candidate463 (in intron):

LOCUS ENSG00000178177

DESCRIPTION transcription factor MLR1

[Source:RefSeq_peptide;Acc:NP_710153]EXON

CTTGATAACCATGATCATTTTTCATGTAAGATCTTAACTCCCTTTCCTCTCTTTGCCTTTGTCATATTTAACACTCAGAA

TCTTATCTGTGTTTTAATTC

In100 candidate464 (in intron):

LOCUS ENSG00000119707

DESCRIPTION Probable RNA-binding protein 25 (RNA-binding motif protein 25)

RNA- binding region-containing protein 7) (Protein S164).

[Source:Uniprot/SISSPROT;Acc:P49756]

GTTCTTTTAGAAGACTGGATCTCCTGGGCACTCTGTAAGTTTTAATTAACTGGTTTGAGGATGTTTTGGGTTTCTCAGTT

TTGTTTTCTTCTATTACTTC

In100 candidate465 (in intron):

LOCUS ENSG00000080815

DESCRIPTION Presenilin-1 (EC 3.4.23.-) (PS-1) (Protein S182) [Contains:

Preenilin-1 NTF subunit; Presenilin-1 CTF subunit; Presenilin-1 CTF12 (PS1-

CTF2)]. [Source:Uniprot/SWISSPROT;Acc:P49768]

GTTTAAATTTTCATCCTTAAATATAACTTTTAATGTAGTATTTTTAGCCCAGAAGGGCCCATGGTTGGTTTGTTTTCTCC

TTTGCATATCTCTTCTACTC

In100 candidate466 (in intron):

LOCUS ENSG00000145147

DESCRIPTION Slit homolog 2 protein precursor (Slit-2) [Contains: Slit homolg

2 protein N-product; Slit homolog 2 protein C-product].

[Source:Uniprot/SISSPROT;Acc:O94813]

TTTGCTTCTCTCTATTCATTTCTTAACTCGATTAAATTTTTTTACATTTTATTTTGCTACCTTATTTTCTCCAGCCACAA

TTTAAACTACTTTTTCCCTT

In100 candidate467 (in intron):

LOCUS ENSG00000145147

DESCRIPTION Slit homolog 2 protein precursor (Slit-2) [Contains: Slit homolg 2 protein N-product; Slit homolog 2 protein C-product].

[Source:Uniprot/SISSPROT;Acc:O94813]

ATTGTGATAATCCCCAACAACTATGACAAAGTTTGCTACCAATAACCTCTCCATCTTTGCATTTTTCTTTGTCTTCAGAG

TGCTTTCAGCAGTTTTTCTT

In100 candidate468 (in intron):

LOCUS ENSG00000106121

DESCRIPTION CDNA: FLJ22374 fis, clone HRC06766. (Fragment).

[Source:UniprotSPTREMBL;Acc:Q9H6D2]

TTATTCTAGTTGGAATAATTATGGCATTTATATTTTCTTTTTACTCGAATAACTTCCTTTAGTATTTTTTTTTAGCAGGT

ATGTTAAATTTTTTTTTCTA

In100 candidate469 (in intron):

LOCUS ENSG00000010379

DESCRIPTION Sodium- and chloride-dependent GABA transporter 2.

[Source:Unipot/SWISSPROT;Acc:Q9NSD5]

ATCCATGTAATTAGGTTTAACTCTATCATCCTATTATTTGTTTTCTCTTTGTCCTATCAGTTATTTGTTTCCCCCTTCCC

TCTCCTCCTGCTTTTTTTTT

In100 candidate470 (in intron):

LOCUS ENSG00000138593

DESCRIPTION Uncharacterized protein KIAA0256.

[Source:Uniprot/SWISSPROT;AccQ93073]

GTTTGTTAAACACTGAAAATATTTGTCAAGCCTGTTACATGTAACCACATAGGGGCTATTGTTTTTTGTTTTTAACAGAA

TTTGTCCTTCCTTTTTAAAA

In100 candidate471 (in intron):

LOCUS ENSG00000120889

DESCRIPTION Tumor necrosis factor receptor superfamily member 10B

precursor(Death receptor 5) (TNF-related apoptosis-inducing ligand receptor 2)

(TRAI receptor 2) (TRAIL-R2) (CD262 antigen).

[Source:Uniprot/SWISSPROT;Acc:O1473]

AGACTCTAGTAACCACCATTCTATTCTCTGCTTCTGAGTTCAGTTGTTTTTTTTTTTTTTTTTGACACAAAAATTTCA

CTTTTGTTGCCCAGGCTGTC

In100 candidate472 (in intron):

LOCUS ENSG00000156958

DESCRIPTION N-acetylgalactosamine kinase (EC 2.7.1.157) (GalNAc kinase)

(Gaactokinase 2). [Source:Uniprot/SWISSPROT;Acc:Q01415]

TTTGTTTTTGTTTGTTTGTTTAATAATATCCTTGTATATGCCTGTACTATAACTTAAAAAAAATCATTGCTTTGTCAGTT

TTTTTGGTATCTTTTAACTT

In100 candidate473 (in intron):

LOCUS ENSG00000156958

DESCRIPTION N-acetylgalactosamine kinase (EC 2.7.1.157) (GalNAc kinase)

(Gaactokinase 2). [Source:Uniprot/SWISSPROT;Acc:Q01415]

TGGTTTGGTGGTGTTCTGTAGTGGTAACATTTGAGTCCTTTCTCTTTCTTATTCATGAGTTTGCTCTACCAGTGGGCTTT

ATACTTTTATGTGTTTTCCT

In100 candidate474 (in intron):

LOCUS ENSG00000156958

DESCRIPTION N-acetylgalactosamine kinase (EC 2.7.1.157) (GalNAc kinase)

(Gaactokinase 2). [Source:Uniprot/SWISSPROT;Acc:Q01415]

TGCAATTGCTCACTGTCTAGTAGTGTCATAGTTTGTCTTATCTGTGTAACTAAGTTATAAGTTCCCTTAATGCAGAGGTT

CTACTTCATGTGGCTTTCCT

In100 candidate475 (in intron):

LOCUS ENSG00000156958

DESCRIPTION N-acetylgalactosamine kinase (EC 2.7.1.157) (GalNAc kinase)

(Gaactokinase 2). [Source:Uniprot/SWISSPROT;Acc:Q01415]

AGATTGCCTAAAAGAAAAACTAATTTTAACTGTTTACAATTATCCATTTTTATATTCTTTCTCTTTTATTTGAAACAGAT

TTTGTTTTAGAAATCTCTTT

In100 candidate476 (in intron):

LOCUS ENSG00000139044

DESCRIPTION N-acetyl-beta-glucosaminyl-glycoprotein 4-beta-

Nacetylgalactoaminyltransferase

2 (EC 2.4.1.244) (NGalNAc-T2) (Beta- 1,4-Nacetylgalactosminyltransferase

III) (Beta4GalNAc-T3) (Beta4GalNAcT3).

[Source:Uniprot/SWISPROT;Acc:Q6L9W6]

ACATTTGTCATGATTAATGAACCAATATCGGATGTTATTATTAACTCAAGTTCATACTGTATCCTGAATTCCTTTCAGAT

ATCCCTTTTCTGTTCCTAGA

In100 candidate477 (in intron):

LOCUS ENSG00000077935

DESCRIPTION Structural maintenance of chromosomes protein 1B (SMC1beta

protin). [Source:Uniprot/SWISSPROT;Acc:Q8NDV3]

ACATGAAGATATGATTGCTTATTCCTTCATATAACAAATATTTATGGTTTCTATCTTGTTCACAGTAACATCTTTACCCC

TTCAGATTGTATCTTATTTT

In100 candidate478 (in intron):

LOCUS ENSG00000166262

DESCRIPTION Uncharacterized protein C15orf33.

[Source:Uniprot/SWISSPROT;AccQ96M60]

TGTTCCGATTTTTCTTTAACTTCCCTTTTGATTTCTTCTTTGACCCATTGGCCAGGATTACATTGATTTCCAGATGTTTT

TTAATTTTCCCACATTCCTC

In100 candidate479 (in intron):

LOCUS ENSG00000145536

DESCRIPTION ADAMTS-16 precursor (EC 3.4.24.-) (A disintegrin and

metalloproeinase with thrombospondin motifs 16) (ADAM-TS 16) (ADAM-TS16). [Source:Unirot/SWISSPROT;Acc:Q8TE57]

CAATCGATTTTGTCTCTCTACGATTTTTAACGTTGTATTTAGTCTGTTTTGCCCTACAGACATTTTTGGTATTTTTATAA
AATCACTGCTTTCCTATTTC

In100 candidate480 (in intron):

LOCUS ENSG00000185774

DESCRIPTION Kv channel-interacting protein 4 (KChIP4) (A-type potassium chanel modulatory protein 4) (Potassium channel-interacting protein 4) (Calsenlin-like protein). [Source:Uniprot/SWISSPROT;Acc:Q6PIL6]

AGTAACTTTGTCAAAATACATTTATATTGGCTTTCCTCCATCCTTGTATCAGTCTCTATGCTTTTCTCACTCCTCCTTCC
TGAGATTATGTCTTTAGTAA

In100 candidate481 (in intron):

LOCUS ENSG00000185774

DESCRIPTION Kv channel-interacting protein 4 (KChIP4) (A-type potassium chanel modulatory protein 4) (Potassium channel-interacting protein 4) (Calsenlin-like protein). [Source:Uniprot/SWISSPROT;Acc:Q6PIL6]

TTGGTTTTATCCATCCTAAGAAGGCTGAATTAGCTGATGGGACTGATAACATGCGAATGCCTTTCTCTACTTATGCAGTA
GTTTCTCCTCTCTTTTCTTT

In100 candidate482 (in intron):

LOCUS ENSG00000185774

DESCRIPTION Kv channel-interacting protein 4 (KChIP4) (A-type potassium chanel modulatory protein 4) (Potassium channel-interacting protein 4) (Calsenlin-like protein). [Source:Uniprot/SWISSPROT;Acc:Q6PIL6]

TTCATTTTGCAACCTGCACTCTGTCTTTAATAACTGTGGTTTTATTGCTAGCCATTTTTCAGGTACCAGCCTTTGGCTAA
ATTTTATTCTATTTTACTTT

In100 candidate483 (in intron):

LOCUS ENSG00000060237

DESCRIPTION Serine/threonine-protein kinase WNK1 (EC 2.7.11.1) (Protein kinse with no lysine 1) (Protein kinase, lysine-deficient 1) (Kinase deficientprotein) (Erythrocyte 65 kDa protein) (p65). [Source:Uniprot/SWISSPROT;Acc:9H4A3]

TTGATAATAATAACTAATTGTATTTCCTCTTTTGTCTTTTGTTATATTTTTGCCTTCAGGTGCTTTTCTCCCCTAACCAG
TTTTCATAAGTTCCTTATAT

In100 candidate484 (in intron):

LOCUS ENSG00000187105

DESCRIPTION HEAT repeat-containing protein 4.
[Source:Uniprot/SWISSPROT;AccQ86WZ0]

ACAATTTGTCCATCACCACTCTTGGTGATAACCATGTGGAAGATCTATCAATTACTCTGGTTTCTCAGTTCCTTAATCTC
ATTATCTCCCATGATCTTTT

In100 candidate485 (in intron):

LOCUS ENSG00000104043

DESCRIPTION Probable phospholipid-transporting ATPase IM (EC 3.6.3.1) (ATPae class I type 8B member 4). [Source:Uniprot/SWISSPROT;Acc:Q8TF62]

TGATAAACCTTTAACCATATATCTTATCTTTTTATGCTCTTTTCTGAAATTTTAAACTTTTCATCTTTCCATATGCAGTT

TTCATATTTTCTACTGACCA

In100 candidate486 (in intron):

LOCUS ENSG00000104043

DESCRIPTION Probable phospholipid-transporting ATPase IM (EC 3.6.3.1) (ATPae class I type 8B member 4). [Source:Uniprot/SWISSPROT;Acc:Q8TF62]

ACTTTTTTCTTTCCATCTTTATCTCTTTCTTAACTTCCTTGCCCCTCTTATTTTATTTATTTTTTCAGTTTCCTCCAT

CTATTTCCCTTCTGTCTTTT

In100 candidate487 (in intron):

LOCUS ENSG00000104043

DESCRIPTION Probable phospholipid-transporting ATPase IM (EC 3.6.3.1) (ATPae class I type 8B member 4). [Source:Uniprot/SWISSPROT;Acc:Q8TF62]

CCAAAAAAAAAAAAAAACCAAAAAAATTATTGACAAGGCTCAAGGCTCTAACATTTGGTAAGACTGGTTGTTTTTCAGTG

CTCTTTTTTTCCCCTAGATT

In100 candidate488 (in intron):

LOCUS ENSG00000104043

DESCRIPTION Probable phospholipid-transporting ATPase IM (EC 3.6.3.1) (ATPae class I type 8B member 4). [Source:Uniprot/SWISSPROT;Acc:Q8TF62]

TTGGATTTACTTATTTTTTTCCTGCATTTTAATCAATAACTTCTGCTTCTTTATTAATGCCTTCTTCGTTCCTTACAGTT

TTTTGTTTTGCTTTTCCCTA

In100 candidate489 (in intron):

LOCUS ENSG00000154678

DESCRIPTION Calcium/calmodulin-dependent 3',5'-cyclic nucleotide phosphodieterase 1C (EC 3.1.4.17) (Cam-PDE 1C) (hCam-3). [Source:Uniprot/SWISSPROT;Ac:Q14123]

GGCTGAAGGTTTATGGATGGGGAGCACAAAGCGGTCTTTATATAACATGTTTAGTGTCTGAATTCTCCTTTGTCTCAGAA

TATCCTTTGCCTGATTTGCT

In100 candidate490 (in intron):

LOCUS ENSG00000154678

DESCRIPTION Calcium/calmodulin-dependent 3',5'-cyclic nucleotide phosphodieterase 1C (EC 3.1.4.17) (Cam-PDE 1C) (hCam-3). [Source:Uniprot/SWISSPROT;Ac:Q14123]

TCTGGCTATGGTTTTTGTTTAATAATACTGGAAACAAAAACCACACAACTAACATTTGTTAAGTGCTTTTTATGTCAGGC

GCTGTTCTAATTACTTTTTC

In100 candidate491 (in intron):

LOCUS ENSG00000154678

DESCRIPTION Calcium/calmodulin-dependent 3',5'-cyclic nucleotide phosphodieterase 1C (EC 3.1.4.17) (Cam-PDE 1C) (hCam-3). [Source:Uniprot/SWISSPROT;Ac:Q14123]

ATAAAACTGTGAGATTGTAAGGTTAACTTGATTTGAAGTAGAATTCTCTCTTCTTATCTATTGTTTCAGAACTAATAGGT

TTCATTGACTTTTCTCCTCC

In100 candidate492 (in intron):

LOCUS ENSG00000154678

DESCRIPTION Calcium/calmodulin-dependent 3',5'-cyclic nucleotide

phosphodieterase 1C (EC 3.1.4.17) (Cam-PDE 1C) (hCam-3).

[Source:Uniprot/SWISSPROT;Ac:Q14123]

TAATTGTGGTTTGGTGGTTTTCTGTAGTGGTAACATTTGGGTCTTTTCTCTTCCTCATTTGTGTATTTGCTTTACCAGTG

AGTTTTTTCCTTTTGCATTT

In100 candidate493 (in intron):

LOCUS ENSG00000002016

DESCRIPTION DNA repair protein RAD52 homolog.

[Source:Uniprot/SWISSPROT;AccP43351]

CCGATTTAACGGAATTCTTTGTTGGTGTTTTCATACTTACCGTGAGTTTTCTTTTTCAGGGTGATATTTTAGTTCCATCT

TAGGCTCTCCATTGTCCTCT

In100 candidate494 (in intron):

LOCUS ENSG00000105778

DESCRIPTION KIAA0241 (KIAA0241), mRNA [Source:RefSeq_dna;Acc:NM_015060]

GAGGCACAAAGAGAGCCCATGGAACTCATAACCATGTTATTCTTTGGGTCTCAAGTCTGTCTTCTCTCTACTTTTCAGTC

TTCTTATGTTTGATTTGTAT

In100 candidate495 (in intron):

LOCUS ENSG00000105778

DESCRIPTION KIAA0241 (KIAA0241), mRNA [Source:RefSeq_dna;Acc:NM_015060]

AGACACAAACCTGCATAACAAACCTTACCTTTATTTATCATACTTTTCTTGGTCACTATCCCATAGCTCAGTTCCCCCAT

ATCTTTCTTTCTTTGATTTA

In100 candidate496 (in intron):

LOCUS ENSG00000130638

DESCRIPTION Ataxin-10 (Spinocerebellar ataxia type 10 protein) (Brain proten

E46 homolog). [Source:Uniprot/SWISSPROT;Acc:Q9UBB4]

ATGTTTTTAATAGTTGAATAATATTTAACTATATAGACTCATCATAATTTTTTCATTTGTTCCCTATAGACTTTCCAGCT

GCTTTCAGGTTGCTTTTTTT

In100 candidate497 (in intron):

LOCUS ENSG00000140043

DESCRIPTION Zinc-binding alcohol dehydrogenase domain-containing protein 1 EC

1.-.-.-). [Source:Uniprot/SWISSPROT;Acc:Q8N8N7]

TCTAGTGGTAACCTTTCCTTCTTACAAATTTTTCTCATACCTCAAATTTTTCTAATTTCCTACTTCCTATTTTTTCAGAC

TTCATTATTATTACTTTTAT

In100 candidate498 (in intron):

LOCUS ENSG00000082805

DESCRIPTION ELKS/RAB6-interacting/CAST family member 1 (RAB6-interacting

prtein 2) (ERC protein 1). [Source:Uniprot/SWISSPROT;Acc:Q8IUD2]

AGGAATCTGTATTTGTGAAGGTTAACTGAACCTTTTTTGTTTGCTTACCATCTATTCAGTGTTTTCCCTATTCTTTGTTC

TATTTATTTAGTAATTTAAA

In100 candidate499 (in intron):

LOCUS ENSG00000082805

DESCRIPTION ELKS/RAB6-interacting/CAST family member 1 (RAB6-interacting prtein 2) (ERC protein 1). [Source:Uniprot/SWISSPROT;Acc:Q8IUD2]

TTAACATCCTTTCTAATCTTCATTATTTGTGGTATCTCTGCTCAAAGGTTCTTCCATTATCTTCCTCACCAGCCATCTTC

TGCTCATCTTTACATGCTTT

In100 candidate500 (in intron):

LOCUS ENSG00000082805

DESCRIPTION ELKS/RAB6-interacting/CAST family member 1 (RAB6-interacting prtein 2) (ERC protein 1). [Source:Uniprot/SWISSPROT;Acc:Q8IUD2]

AGATGCTATCAACATTCCCTTCCATGCTTCTGTAACTTCTGTTTATTTCGTTTTGCTTGCATCTCCAGTTTCCTAGGTTT

GTTTTTTTTTTTGTCCTAG

In100 candidate501 (in intron):

LOCUS ENSG00000082805

DESCRIPTION ELKS/RAB6-interacting/CAST family member 1 (RAB6-interacting prtein 2) (ERC protein 1). [Source:Uniprot/SWISSPROT;Acc:Q8IUD2]

ATCTTGACCTGCCTGTGAATCTTTAACATGTTTTATCTCTTCTTATTTCTGGAAACTTTCTTTATCTTTGGCTTCCAGGG

GACCACCTTCTTTTTGTTTT

In100 candidate502 (in intron):

LOCUS ENSG00000119725

DESCRIPTION Zinc finger protein 410 (Zinc finger protein APA-1) (Another patner for ARF 1). [Source:Uniprot/SWISSPROT;Acc:Q86VK4]

AGTTTAACAATTAGCTATGATGGTTTTAATGCTTTTTCTTTTCAGTTCAGCATGCCTATACTACTTACTCTGTGTCACTT
TTCTCTGTTCTAGTAATTTT

In100 candidate503 (in intron):

LOCUS ENSG00000104689

DESCRIPTION Tumor necrosis factor receptor superfamily member 10A precursor(Death receptor 4) (TNF-related apoptosis-inducing ligand receptor 1) (TRAI receptor 1) (TRAIL-R1) (CD261 antigen). [Source:Uniprot/SWISSPROT;Acc:O00020]

TACTATATACAAAAATTATCTCAAAATTCATCAATAACATTCCTTTTCCATGTTCTTCAAGTTTTCTGTTTCCTACAGTT
TCTGGTTCTTCTATTCAACA

In100 candidate504 (in intron):

LOCUS ENSG00000104689

DESCRIPTION Tumor necrosis factor receptor superfamily member 10A precursor(Death receptor 4) (TNF-related apoptosis-inducing ligand receptor 1) (TRAI receptor 1) (TRAIL-R1) (CD261 antigen). [Source:Uniprot/SWISSPROT;Acc:O00020]

AGGAGTAGTTTTGATAACTAAGTCAAAATCTTTACTTGCTATATGTCTATTCACATTGTCTCTTTCATCTTGATTCAGTT
TCAGTAGTTTGTGTCTTTCT

In100 candidate505 (in intron):

LOCUS ENSG00000186951

DESCRIPTION Peroxisome proliferator-activated receptor alpha (PPAR-alpha).

Source:Uniprot/SWISSPROT;Acc:Q07869]

TGCCTCATTTTATTGTTTTTCACATTAAATGTAACGACATTTAAAGTTCTGTATTTGTCCTAATCATTCCAGACTTCTTA

GAAGAACTATTTCTTTCTTT

In100 candidate506 (in intron):

LOCUS ENSG00000078295

DESCRIPTION Adenylate cyclase type 2 (EC 4.6.1.1) (Adenylate cyclase type I)

(ATP pyrophosphate-lyase 2) (Adenylyl cyclase 2).

[Source:Uniprot/SWISSPRT;Acc:Q08462]

AGCATCTAGTAAAGCTTGTTCCACACGTAACATTACCTCGCTCTTGTTCTTATAGTTATATTTATCTCCAGTTTCTTCTC

TGTTTCCTCTAATGATAATG

In100 candidate507 (in intron):

LOCUS ENSG00000081665

DESCRIPTION Zinc finger protein 93 (Zinc finger protein 505) (Zinc finger

potein HTF34). [Source:Uniprot/SWISSPROT;Acc:P35789]

TTGAAGTTTGTTTATAAAATTATGTTTATTGAGATGAAATTCACATAACATCAAATTCAATATCTTAATTATTTTCAGGT

TTACAGTTCAGTGTCTTTTT

In100 candidate508 (in intron):

LOCUS ENSG00000109606

DESCRIPTION Putative pre-mRNA-splicing factor ATP-dependent RNA helicase DH15 (EC 3.6.1.-) (DEAH box protein 15) (ATP-dependent RNA helicase #46). [Sorce:Uniprot/SWISSPROT;Acc:O43143]

CTTTCTAGAATATTAAATGCCGGTTATGCTTATTCAAGTTTCACCTGTTAACCTTATGTATGTCGTTGTTTGCTTCAGTT
CTAGTTGCTGATTATTTTTC

In100 candidate509 (in intron):

LOCUS ENSG00000122507

DESCRIPTION Protein PTHB1 (Parathyroid hormone-responsive B1 gene protein) Bardet-Biedl syndrome 9 protein). [Source:Uniprot/SWISSPROT;Acc:Q3SYG4]

TTTTTTTTTCCACTTCCTTCGAGATAACTCACTTCTGGCTTTGTTTTGATCTCAGTGAATGCCCCTGTTAAGCTTCTGGT
AGATTCCTCTTCTTTTTCTT

In100 candidate510 (in intron):

LOCUS ENSG00000122507

DESCRIPTION Protein PTHB1 (Parathyroid hormone-responsive B1 gene protein) Bardet-Biedl syndrome 9 protein). [Source:Uniprot/SWISSPROT;Acc:Q3SYG4]

CTCATAAATAAATTTATCTAATCTGTCTTCTCTTAACCACACAAGATTTCCATAAACCTTTCATTTTGCATTTTCCAGCT
TTCTATATTCATCTTGTTTT

In100 candidate511 (in intron):

LOCUS ENSG00000122507

DESCRIPTION Protein PTHB1 (Parathyroid hormone-responsive B1 gene protein) Bardet-Biedl syndrome 9 protein). [Source:Uniprot/SWISSPROT;Acc:Q3SYG4]

AAGTTAAGTGCATATCTTTGATTCTTCATAGATTATGGGAAATATGTTAACTTCTGGGTCGTATGTTTGCTTTTTCAGTT
TTATTTTTCTGGTTGGTGCT

In100 candidate512 (in intron):

LOCUS ENSG00000122507

DESCRIPTION Protein PTHB1 (Parathyroid hormone-responsive B1 gene protein)

Bardet-Biedl syndrome 9 protein). [Source:Uniprot/SWISSPROT;Acc:Q3SYG4]

AGTTGGCTTAAGCAAGAATAGGAAGTTGACTGGCTAACGTCACTTGAGCCTTCTCTGTGATCAGATCTGGTTTCTCTCTT

TTCATCTCTTGCTTCCATTT

In100 candidate513 (in intron):

LOCUS ENSG00000122507

DESCRIPTION Protein PTHB1 (Parathyroid hormone-responsive B1 gene protein)

Bardet-Biedl syndrome 9 protein). [Source:Uniprot/SWISSPROT;Acc:Q3SYG4]

TAGCACTGTGTTATAATGTATATTTCCCTATTGGTAACCCTGAATGCTATTTCCTCTTAAGTAGGTGTCTCTTTCCAGCT

TCATTTTCCCTGTGTTACTA

In100 candidate514 (in intron):

LOCUS ENSG00000122507

DESCRIPTION Protein PTHB1 (Parathyroid hormone-responsive B1 gene protein)

Bardet-Biedl syndrome 9 protein). [Source:Uniprot/SWISSPROT;Acc:Q3SYG4]

TGGCTACTGTGTAACACTCCTCATCCATTTTCTCCTTCAGTCTATGTTCTACCCATGATAAATTGCTATTTTCCTCCCAA

ATTCTCTTGCCCTTTTGTGT

In100 candidate515 (in intron):

LOCUS ENSG00000122507

DESCRIPTION Protein PTHB1 (Parathyroid hormone-responsive B1 gene protein) Bardet-Biedl syndrome 9 protein). [Source:Uniprot/SWISSPROT;Acc:Q3SYG4]

CTCAAGTCCTGGGCTTTTCTTTGGTAGGAGACTTTGTATAACTGATTCAATCTTCTTACTCGTTATTGCTCTGTTCAGAT
TTTCTATTTCTTCCTAGTTC

In100 candidate516 (in intron):

LOCUS ENSG00000122507

DESCRIPTION Protein PTHB1 (Parathyroid hormone-responsive B1 gene protein) Bardet-Biedl syndrome 9 protein). [Source:Uniprot/SWISSPROT;Acc:Q3SYG4]

TCCCAAAGAAAATTATAACATTATACGTAATGCTAAATTTCCCATCTTGGTTCCTTATCATCTGCTCAGAAATGTAATCA
TTCTTACTGTTTTTGTTTCT

In100 candidate517 (in intron):

LOCUS ENSG00000081014

DESCRIPTION AP-4 complex subunit epsilon-1 (Adapter-related protein complex4 epsilon-1 subunit) (Epsilon subunit of AP-4) (AP-4 adapter complex epsilo subunit). [Source:Uniprot/SWISSPROT;Acc:Q9UPM8]

ATGTCCTTTAACATTCTCTTTCTCTCTTTCCATATTCCAAATCAAATTTTATTTCTCCTGTGATCACTCAGGTGGCTCCT
ACCATCTGTTACCTTTTGTT

In100 candidate518 (in intron):

LOCUS ENSG00000081014

DESCRIPTION AP-4 complex subunit epsilon-1 (Adapter-related protein complex4 epsilon-1 subunit) (Epsilon subunit of AP-4) (AP-4 adapter complex epsilo

subunit). [Source:Uniprot/SWISSPROT;Acc:Q9UPM8]

AGGATTGGGACTAACTGAATGTGCTCCTTCTGTGTCATGCCTCATTTTGGATGGCTGTTTTTCTTCAGAAATCCTGTTCC

CTACTGATTTTTCTTACGTT

In100 candidate519 (in exon):

LOCUS ENSG00000197217

DESCRIPTION Ectonucleoside triphosphate diphosphohydrolase 4 (EC 3.6.1.6)

(TPDase 4) (Uridine-diphosphatase) (UDPase) (Lysosomal apyrase-like protein f 70

kDa). [Source:Uniprot/SWISSPROT;Acc:Q9Y227]

AAATGATAATCCTCTTTTTTCTCTATCTTGAAAGCTCTAACCTGAAGAGGGTGATTTTTTTTTTATTTATTGTTCAGGT

CTGTTACTGTATATGTTTTC

In100 candidate520 (in intron):

LOCUS ENSG00000181982

DESCRIPTION CDNA FLJ43363 fis, clone NT2RP7017474.

[Source:Uniprot/SPTREMBLAcc:Q6ZUS6]

TTATTCTTATTGTTTTGCTTTATAATACTTCTAATATCTTTTTGGGCTAACCTTCTTCACTTTGCTCTTCCTTTTCAGAG

GTTTCTTATATGTTACTTTT

In100 candidate521 (in intron):

LOCUS ENSG00000183578

DESCRIPTION tumor necrosis factor, alpha-induced protein 8-like 3

[Source:RfSeq_peptide;Acc:NP_997264]

ACAATTAATGTTGCTGCTGTTATTATCACATTAATATAACTGCCTAAAGAGATTTGTTTTGAATTTTCTCCTTTTCAGTC

TCTTGTTCATTATCTCTGGT

In100 candidate522 (in intron):

LOCUS ENSG00000167981

DESCRIPTION Zinc finger protein 597. [Source:Uniprot/SWISSPROT;Acc:Q96LX8]

EXON

GAATAACCCTTCTAAGGTTTCCTGGTCCTGTTTGTAACTACCTTAGCTTACTTTTTCCCCAGTATCCCTTGAAGATATTG

TCTATGTTTTGCTTTCTTTT

In100 candidate523 (in intron):

LOCUS ENSG00000197085

DESCRIPTION AAA1 protein isoform IV [Source:RefSeq_peptide;Acc:NP_997169]

TTTTGCTGAGAAATTTTCTGCTAGCCTTGTTATAACTCTCTTATATGTGATTTGCCTCTTCTCTCTTGCTGTTATCAGGA

TTTTCTCTGTTTTTGATTTT

In100 candidate524 (in intron):

LOCUS ENSG00000197085

DESCRIPTION AAA1 protein isoform IV [Source:RefSeq_peptide;Acc:NP_997169]

AGTAACTCTAAGCTTAAATTTGACTTTTTTCCTCTGCAGTTATGATTTGATATTTTCCATGCCCTTTCTATTTCATTTCT

TATTTTTCATTGTGTTGGGT

In100 candidate525 (in intron):

LOCUS ENSG00000197085

DESCRIPTION AAA1 protein isoform IV [Source:RefSeq_peptide;Acc:NP_997169]

AGCTCATTTTTAACATGATCAAAATAGAATCTTTATTTCTATCATTACTATCTTTTCTCCCAGTCTTTCCTACCTGAATA

ACTAGTTCCAGTTTCTTTTT

In100 candidate526 (in intron):

LOCUS ENSG00000104093

DESCRIPTION Protein DmX-like 2 (Rabconnectin-3).

[Source:Uniprot/SWISSPROT;cc:Q8TDJ6]

CTATGGTAAGCCTATTTTTATAACCCTGGAAGACATTATTATTTTACAGAGTCAATGCTTACTTAGATTTACCCATATGT

TACTTTGCTCTTCGTTTCTT

In100 candidate527 (in intron):

LOCUS ENSG00000189212

DESCRIPTION DPY19L2P1 protein. [Source:Uniprot/SPTREMBL;Acc:Q6NXN4]

ATGGCTATATACCTACAAAATATGCATTATGCCAATATATGTAATAACCATAAAATGTATTTTTTTCTTTTTTTTCAGAT

TTCAGTTACCCTTAGTTTCA

In100 candidate528 (in intron):

LOCUS ENSG00000054611

DESCRIPTION TBC1 domain family member 22A.

[Source:Uniprot/SWISSPROT;Acc:Q8UA7]

AGTTCTAACAAGACGGTGCCCATTTCTCTTTCTAATTTATTCACTTGTTTGCTCACTCATGAATTCAGTCATTTGCTCAT

TGATTTGTTCACTCATTTAC

In100 candidate529 (in intron):

LOCUS ENSG00000138594

DESCRIPTION Tropomodulin-3 (Ubiquitous tropomodulin) (U-Tmod).

[Source:Unipot/SWISSPROT;Acc:Q9NYL9]

AACATTTCCTGGATAACTACACCCACTCTGGATAGTTCCTTTCCTCCAGCATTTCTCCATATGATTTACATACCGTAAAG

TACTGCCTTTTTTTTTTTTT

In100 candidate530 (in intron):

LOCUS ENSG00000053900

DESCRIPTION Anaphase-promoting complex subunit 4 (APC4) (Cyclosome subunit).

[Source:Uniprot/SWISSPROT;Acc:Q9UJX5]

AAATGAAGGCTGAATTTATTGATTTTGTTGTAAATTGATGGGAATATAACTTTCCTATAAAACCATTCTTTGATTCAGAC

TTCTGATTTCTAATTCTTTA

In100 candidate531 (in intron):

LOCUS ENSG00000119682

DESCRIPTION K0317_HUMAN Isoform 2 of O15033 - Homo sapiens (Human)

[Source:niprot/Varsplic;Acc:O15033-2]

CTCTGTGAAACATCCCATTGCATCTCTTTAATCTCATAACCAAAACTGAGTTCTAATTCTGTTCTACTTCAGAACACTTT

GCTGTGTACCATTCTTCATT

In100 candidate532 (in intron):

LOCUS ENSG00000042980

DESCRIPTION ADAM 28 precursor (EC 3.4.24.-) (A disintegrin and

metalloprotenase domain 28) (Metalloproteinase-like, disintegrin-like, and cysteine- rih protein-L) (MDC-L) (eMDC II) (ADAM23). [Source:Uniprot/SWISSPROT;Acc:Q9UK2]

TTTCCATTCCTTGGTAACTACGTGGAAAGAGCTTTGCTTTTGAGACATTTTATCGTTTTGGCTTTCCTCTACCTCAGGAG
TGCTACTCCTCATTTCATTT

In100 candidate533 (in intron):

LOCUS ENSG00000119596

DESCRIPTION YLP motif-containing protein 1 (Nuclear protein ZAP3) (ZAP113).[Source:Uniprot/SWISSPROT;Acc:P49750]

TTAAGAATCTCTCTGAGGATAACAATTTGAGTTGAAAGTTCTTTTCTGCTTTTTGCATTCTGTTTTCTCCAAATTCAGAT
CCTATTTATTTATCTTTAGT

In100 candidate534 (in intron):

LOCUS ENSG00000069206

DESCRIPTION ADAM 7 precursor (A disintegrin and metalloproteinase domain 7)(Sperm maturation-related glycoprotein GP-83). [Source:Uniprot/SWISSPROT;Ac:Q9H2U9]

TACACTTCTTATTGACTAGTTACATTGCCTAGTAACATTGTATGAATGATCTTTCCATTTCTGTTCATTCAGTGTTGATA
GTTTATCATTCACTTCCTTT

In100 candidate535 (in intron):

LOCUS ENSG00000091490

DESCRIPTION

GCTGAGATTAACTGTCATCATTATGGGTATTTACTTTCCCTCCATCTGTGTCTTTTTTGGTTCTCTTCTTTGTCAGACCC

CTTTCTTTCTCTTTCTTGGT

In100 candidate536 (in intron):

LOCUS ENSG00000164236

DESCRIPTION

CGCGCTGAACACAATGAATGGACGCGTGTCAATTCACCATTATTAACTGAAGTCCGTTCTTTATTCGGATTTCCTCAGCG

TTTCCCGGATTTCCTTTTTC

In100 candidate537 (in intron):

LOCUS ENSG00000112977

DESCRIPTION Death-associated protein 1 (DAP-1).

[Source:Uniprot/SWISSPROT;Ac:P51397]

AGCCTTCCCTAGCCATTTTTGGTTATCTGTGACTTCTAACGTCTTTGTGAGTGTCCTTTTCATTTCGGCCAGTGCCCTGT

GCATTCTTACTTGGTTTTGT

In100 candidate538 (in intron):

LOCUS ENSG00000164542

DESCRIPTION

TGCCTCTCCGAATAACCTAGTATTCTCCTAACATCACAATACTTTCCTTTTTTTTTTTTTCTTTTCACTATGCTCCAGCT

TCAGGCCTTTTTTCTGGTCA

In100 candidate539 (in intron):

LOCUS ENSG00000197535

DESCRIPTION Myosin-Va (Dilute myosin heavy chain, non-muscle) (Myosin-12) (yosin heavy chain 12) (Myoxin). [Source:Uniprot/SWISSPROT;Acc:Q9Y4I1]

TGGAATGCTCTGCTCGTCCTCTTAACTTGCTTATCTCCCATTCATTTTGGCTTCTTTCAGTTTCAACTCCCACTACTAAT

TGCCTCATTATTTTCTTGTT

In100 candidate540 (in intron):

LOCUS ENSG00000197535

DESCRIPTION Myosin-Va (Dilute myosin heavy chain, non-muscle) (Myosin-12) (yosin heavy chain 12) (Myoxin). [Source:Uniprot/SWISSPROT;Acc:Q9Y4I1]

AGAACTTGTATAACAAGTGAGGAAATTGTCTTTTTCCTTGTCAGATTCTAAACCCTGGCATGGAGTCTGACTGTTTTCTT

TTCTTTTTTTTGCCTTGTTC

In100 candidate541 (in intron):

LOCUS ENSG00000121390

DESCRIPTION paraspeckle protein 1 [Source:RefSeq_peptide;Acc:NP_001035879]

EXON

CTCTGGAAAGATGTGGTGATGGGGCATTCTTGCTAACAACGTAAGTTTTAAATATCTTGTCCTTCTTCTATCCAGTTACA

TATGGGCTTCTCTCTGTTTT

In100 candidate542 (in intron):

LOCUS ENSG00000121390

DESCRIPTION paraspeckle protein 1 [Source:RefSeq_peptide;Acc:NP_001035879]

EXON

TTCCGATAACTTCTGATGTTGAGTACCCTTTATATACTGCTTTTTTTTTTTTTTTTTTTTGCCATTCAGCCACCTGCTTT

GTAAAGTACTCCTGCTTTTT

In100 candidate543 (in intron):

LOCUS ENSG00000169862

DESCRIPTION Catenin delta-2 (Delta-catenin) (Neural plakophilin-related

ARMrepeat protein) (NPRAP) (Neurojungin) (GT24).

[Source:Uniprot/SWISSPROT;AccQ9UQB3]

AAAGAAGGTGCTGTCTAACTCCGAAGAGATTTTGTCTTTTTTTTGGCAGTTTCTCTCAAGATCTTTGATGCTTACACAAC

TGTCCATTGCCTTTCCTTTT

In100 candidate544 (in intron):

LOCUS ENSG00000169862

DESCRIPTION Catenin delta-2 (Delta-catenin) (Neural plakophilin-related

ARMrepeat protein) (NPRAP) (Neurojungin) (GT24).

[Source:Uniprot/SWISSPROT;AccQ9UQB3]

AAAATATTTGTCATTTTATTTATCTTTTGAAAATAACTCAACTTTTTGTTTCATTGATATTTTATATTTTTGTCTCAGTT

TCATTTATTTCTGCTCCGAC

In100 candidate545 (in intron):

LOCUS ENSG00000169862

DESCRIPTION Catenin delta-2 (Delta-catenin) (Neural plakophilin-related

ARMrepeat protein) (NPRAP) (Neurojungin) (GT24).

[Source:Uniprot/SWISSPROT;AccQ9UQB3]

AAATATTCTCCTGCTTTCAAGCTTCTCTTATGTGATCCGCTCTTTAACCCTCCTACTTTCCTTTTTTTCATTATTCAGTT

TTTTTGCCTTTTTTGCCCTT

In100 candidate546 (in intron):

LOCUS ENSG00000169862

DESCRIPTION Catenin delta-2 (Delta-catenin) (Neural plakophilin-related

ARMrepeat protein) (NPRAP) (Neurojungin) (GT24).

[Source:Uniprot/SWISSPROT;AccQ9UQB3]

ATTCTCTCCCCTGTTCATCCTCCTCCCCTCCCTAGTCTCTGGTAACCACTATTCTACTCTCTGCTTCTATGATATCAGCT

TCTTTTTTAGATTCCATGTT

In100 candidate547 (in intron):

LOCUS ENSG00000155849

DESCRIPTION Engulfment and cell motility protein 1 (CED-12 homolog).

[Sourc:Uniprot/SWISSPROT;Acc:Q92556]

CTTCCAACTAACAAGACTAGATTTCTAGTAGAGTGAATGTCTGTGCCTTTTTCTTCCTCTTTCTCTTCTCTTCTTCAGTG

TCATCATCTTCTTGTTCTTC

In100 candidate548 (in intron):

LOCUS ENSG00000147459

DESCRIPTION Dedicator of cytokinesis protein 5.

[Source:Uniprot/SWISSPROT;Ac:Q9H7D0]

AGTCTGTCCCTAACTGGGACCAAGCTACCTATATCACTTTATTTCTACAGTTCATTTTTCAGTCCCCCCCATTGTTGGGT

ATTTAGCTTGTTTGCTGCTT

In100 candidate549 (in intron):

LOCUS ENSG00000147459

DESCRIPTION Dedicator of cytokinesis protein 5.

[Source:Uniprot/SWISSPROT;Ac:Q9H7D0]

GCAAGTCTCCCTTTAATTTTTCATTTTCAAGCTATTTTAAACTATTTTTAACCTTTTTTCACATGTTTATTCTTCCAGAT

ATATTTTATTACCATTTTTG

In100 candidate550 (in intron):

LOCUS ENSG00000147459

DESCRIPTION Dedicator of cytokinesis protein 5.

[Source:Uniprot/SWISSPROT;Ac:Q9H7D0]

AGATAACCATTATTAAGAGTTGGCACATTTTCTTTCTTGTTTCTTTTTTTAGATGTGTCACAGATCAATGTTTGTTTTTT

TGTTTTTTGTTTTTTTTTTT

In100 candidate551 (in exon):

LOCUS ENSG00000128989

DESCRIPTION cAMP-regulated phosphoprotein 19 (ARPP-19).

[Source:Uniprot/SWISPROT;Acc:P56211]

TGTATATGGAAGCATAGGTGTCTATATCCTTACTCTTTTATATAACACAATAATGGCGTTTTCCTTCTAATTTCTCAGTT

GTTAACTTCTTTCTCTTTTT

In100 candidate552 (in intron):

LOCUS ENSG00000073146

DESCRIPTION Putative helicase Mov10l1 (EC 3.6.1.-) (Moloney leukemia virus 0-

like protein 1) (MOV10-like 1). [Source:Uniprot/SWISSPROT;Acc:Q9BXT6]

AGGCTATGAACATGGAATGTGTTAACATTTATTTATGTCTTCTTTAATTTCTTACAGCAGTGTTTTGTCATTTTCTCTGT

ATATGTTTTTCACTTCTTTG

In100 candidate553 (in intron):

LOCUS ENSG00000109680

DESCRIPTION TBC1 domain family member 19.

[Source:Uniprot/SWISSPROT;Acc:Q8NT2]

TTTGACTACTTCAAGAGAAAATCATGTAATTTCTGTGTTAACCATTATCTAGTCTCAATTCATTTGTTTAACTTCCAGTG

GATTCTTTTTGCTGTCTGCC

In100 candidate554 (in intron):

LOCUS ENSG00000109680

DESCRIPTION TBC1 domain family member 19.

[Source:Uniprot/SWISSPROT;Acc:Q8NT2]

TTTAACCTCATTGACTATTCCTTTATTGACATATTCCTTTGCTTTTGTGACACAGTGCTCTTTCTGGCATTCTTCATTTG

TGGAGGGTCTTCTTTGTCTT

In100 candidate555 (in intron):

LOCUS ENSG00000109680

DESCRIPTION TBC1 domain family member 19.

[Source:Uniprot/SWISSPROT;Acc:Q8NT2]

ATCGGTTGGCTTGATGGTGTCCCATAAGTTCCTATGGCTCTGTTAACTTCAATTTTTTTTCCTTTCTGTTTCACAGCCTC

AATTATTTCCATTTCCCTAT

In100 candidate556 (in intron):

LOCUS ENSG00000166415

DESCRIPTION WD repeat protein 72. [Source:Uniprot/SWISSPROT;Acc:Q3MJ13]

TAGAAGGATGTTTAACTACTAATTCAATTTCTTTACTTGGTATAGGTCTGTTCCACTTTCTATTTCTTCTTATGCCAGTT

TTGGTCGTTTATGTCTTTTT

In100 candidate557 (in intron):

LOCUS ENSG00000166415

DESCRIPTION WD repeat protein 72. [Source:Uniprot/SWISSPROT;Acc:Q3MJ13]

TTCAACTCTTGGCTACCATATTGAGCTAGTAACTTTGAATTCAAATCTTTGAGTTCTTTTTCACTGCTTATTTCACAGGC

CTGAGCTATCTTTTAGTCTT

In100 candidate558 (in intron):

LOCUS ENSG00000130038

DESCRIPTION EF-hand calcium binding domain 4B

[Source:RefSeq_peptide;Acc:NP116069]

TAAAAATCACCCATAAACCCAATAAACAAGATAACCACCATTGATATTGTGGTACATTTCCTTCTAGTCTTTTTTCAGTG

TACATGTCTTTTCTTATTGC

In100 candidate559 (in intron):

LOCUS ENSG00000130038

DESCRIPTION EF-hand calcium binding domain 4B

[Source:RefSeq_peptide;Acc:NP116069]

AGTCACTCTAAAAATTATTAACGAGATCTTTTACATTCCTTTCTTTTGAACTCAGTCTTTGAAATCTCTTTTGCTCTTAC

ATCTCACTTTAGACTGTCCA

In100 candidate560 (in intron):

LOCUS ENSG00000137766

DESCRIPTION Unc-13 homolog C (Munc13-3) (Fragment).

[Source:Uniprot/SWISSPRT;Acc:Q8NB66]

TATAGATTGGGAATTTTAGTAGATACCAACAAATTAACCTCCAAAGGGGTTTATTAATTTTCAGTCCCTCCGGCAATCT

CTAAAAGTGTTTTTTTTTTC

In100 candidate561 (in intron):

LOCUS ENSG00000137766

DESCRIPTION Unc-13 homolog C (Munc13-3) (Fragment).

[Source:Uniprot/SWISSPRT;Acc:Q8NB66]

TGTGGTTTGGGATTTTCTATAGTCATAACATTTACGTCCTTTCTCTTCCTAATTTTTTTGTGTGTGCTCTACCAGTGGGT

TTTATCCTTTTGTGCATTTT

In100 candidate562 (in intron):

LOCUS ENSG00000169851

DESCRIPTION Protocadherin-7 precursor (Brain-heart protocadherin) (BH-Pcdh)

[Source:Uniprot/SWISSPROT;Acc:O60245]

ACCCACCTCCCTCCCACCACACTGCCTTAGGATGTTTTAACTTCCCTTTCCATAAACTTTTTTTTTCTTTTCTGCAGCA

ACTCTTTTTTTGCTGCCATT

In100 candidate563 (in intron):

LOCUS ENSG00000169851

DESCRIPTION Protocadherin-7 precursor (Brain-heart protocadherin) (BH-Pcdh)

[Source:Uniprot/SWISSPROT;Acc:O60245]

TCCCTTCAAAAAGTAATTTAAAGGAGAAAGTTCCCCTTAAGCTAACATAGTTCTTATTTCTTCTATTTTCAACTTCAGTT

CTTTTTTTTCCACTGTGTTC

In100 candidate564 (in intron):

LOCUS ENSG00000047621

DESCRIPTION Uncharacterized protein C12orf4.

[Source:Uniprot/SWISSPROT;Acc:9NQ89]

TTGCCTTCCTAGAAAATTATAACTACACTATTCCTCACGAATTTTCTTTTTTACTGTCACTTTATCCAGTTTATTGTGAT

ATATCTGTTGCTGTCCTTTC

In100 candidate565 (in intron):

LOCUS ENSG00000047365

DESCRIPTION Centaurin-delta 1 (Cnt-d1) (Arf-GAP, Rho-GAP, ankyrin repeat an

pleckstrin homology domain-containing protein 2) (PARX protein).

[Source:Uiprot/SWISSPROT;Acc:Q8WZ64]

GTGATCCTCTGACTGCTATTAAGCCCATTTTCTCACAAAGTTTCTAACTTTGCTAAGGATGTGTTTCTGATTTTTCAGTG

GTATGCTCTCTTTTTGTTTT

In100 candidate566 (in intron):

LOCUS ENSG00000047365

DESCRIPTION Centaurin-delta 1 (Cnt-d1) (Arf-GAP, Rho-GAP, ankyrin repeat an pleckstrin homology domain-containing protein 2) (PARX protein). [Source:Uiprot/SWISSPROT;Acc:Q8WZ64]

AAAAACATGTGCACAAGAGACTGGCTGCTAACTTTTTAGAGGCTTTTCTTCTTCTTTTTGTAACTTATTAAAATTCAGAT ATTCAACTTTTTTTTTTTTT

In100 candidate567 (in intron):

LOCUS ENSG00000006715

DESCRIPTION Vacuolar protein sorting-associated protein 41 homolog (S53). [ource:Uniprot/SWISSPROT;Acc:P49754]

TACTTTTTCAATCCCACTCTCTTTCCCTGTGGAACTGTAATAACATGAATGTTGGCCCTTTTATTATTTTTTTCACAGGT CCCTGAGACTCTTCATTTTT

In100 candidate568 (in intron):

LOCUS ENSG00000119685

DESCRIPTION Tubulin--tyrosine ligase-like protein 5 (SRC1 and TIF2-associatd modulatory protein). [Source:Uniprot/SWISSPROT;Acc:Q6EMB2]

GCTGCTAGTTTACACACAACCATTTATATGTCTGCTTTTAACATTGTCTGAGTGTGTTCCTGGGTTTCTTCTTATCAGGT TTGATTGCTTTAATCCCATC

In100 candidate569 (in intron):

LOCUS ENSG00000119685

DESCRIPTION Tubulin--tyrosine ligase-like protein 5 (SRC1 and TIF2-associatd modulatory protein). [Source:Uniprot/SWISSPROT;Acc:Q6EMB2]

AACTTTGGTTATATGTTCATAACATTTGAGGTTTGAGCCTTCTTTATTTGTCCCATTTTCCAGATAGGAATTTTTTCTTT
TTACTGTATTCTTTTTTTCT

In100 candidate570 (in intron):

LOCUS ENSG00000106536

DESCRIPTION POU domain, class 6, transcription factor 2 (Retina-derived POUdomain factor 1) (RPF-1). [Source:Uniprot/SWISSPROT;Acc:P78424]

CATTTTAAGGTTCCATGTGAATGTGAATATGTAACAAAACACCTACTCTATATTTCTATCCATCAGTATTTTATTTACCC
ACCAGCCTTCATTTCTCTTT

In100 candidate571 (in intron):

LOCUS ENSG00000106536

DESCRIPTION POU domain, class 6, transcription factor 2 (Retina-derived POUdomain factor 1) (RPF-1). [Source:Uniprot/SWISSPROT;Acc:P78424]

AACCATATCTTTTTTCTTAACAAACTTCCATTTTTTTTCACTCTCCCATTAAATCTCAGCTTTCTTTAAAACCTTCAAAA
CTTATTTATTTATTTATTTT

In100 candidate572 (in intron):

LOCUS ENSG00000106536

DESCRIPTION POU domain, class 6, transcription factor 2 (Retina-derived POUdomain factor 1) (RPF-1). [Source:Uniprot/SWISSPROT;Acc:P78424]

AAAACTTCTAAGTTGGAGTAGAATAGTAAATCCTTTAACCACTCTGAAATATGACTCAATTTCTTTTCCTTCCAGAATAG

CCCTTCTTACAATTTCCTTT

In100 candidate573 (in intron):

LOCUS ENSG00000166450

DESCRIPTION protogenin [Source:RefSeq_peptide;Acc:NP_776175]

ACTTTAACTGTATTAGGTATGTTCCTTTCATTCTGCAATCAGGGGAATCTTACATTTTAACATTTGCCGTTTTATCTCAT

TCATAGCTTACTTCTTTCTT

In100 candidate574 (in intron):

LOCUS ENSG00000154274

DESCRIPTION C4orf19 protein. [Source:Uniprot/SPTREMBL;Acc:Q8IY42]

AGTCTTGGTTAAATAGTAACTAGTACCTATGTCCTGCTTTTCTTTCTTGGTCATTAGGCAGATTTTCTTTTTGGTCATTC

TTTTTGGTCATTAGATTACA

In100 candidate575 (in intron):

LOCUS ENSG00000154274

DESCRIPTION C4orf19 protein. [Source:Uniprot/SPTREMBL;Acc:Q8IY42]

AGGTGTGCGCCACCACTGAATTTTTTAACAATCATCTTTAAATTTTTCTTTTACCTCCATAGTTTCTGGTCCCTCCAGAT

TGTTTTTGGCTTTCCCCGGC

In100 candidate576 (in intron):

LOCUS ENSG00000069869

DESCRIPTION E3 ubiquitin-protein ligase NEDD4 (EC 6.3.2.-).

[Source:UniprotSWISSPROT;Acc:P46934]

TGATCTTATTACTTTAATAACTTTATTTTTGCATATTTCCTTCTAATCTTTCTGTGTGTGCTTTTACAGTTGTCATAGAT

TTCAGCATTCTTATTTTTTC

In100 candidate577 (in intron):

LOCUS ENSG00000065883

DESCRIPTION Cell division cycle 2-like protein kinase 5 (EC 2.7.11.22)

(CDCrelated

protein kinase 5) (Cholinesterase-related cell division controlle).

[Source:Uniprot/SWISSPROT;Acc:Q14004]

TTTATTGAGGCATTTAGAAGTCGCCTAACATCTTTCTTGGCCTGTACTCTTTTTTTTTTTTTTTTAATTAAAAGCAGTT

CTTTTACCAACTTTTAATTT

In100 candidate578 (in intron):

LOCUS ENSG00000120907

DESCRIPTION Alpha-1A adrenergic receptor (Alpha 1A-adrenoceptor) (Alpha

1Aadrenoreceptor)

(Alpha-1C adrenergic receptor) (Alpha adrenergic receptor 1).

[Source:Uniprot/SWISSPROT;Acc:P35348]

TTCTTCACTTTTTTTATTTTAATTTTTTTACTTTTTCTTCTCTGGATAACTTCAAATTACTGGCCTTTTTATTCACAGAT

TCTTTCTTCTCCTTGATCAA

In100 candidate579 (in intron):

LOCUS ENSG00000134115

DESCRIPTION Contactin-6 precursor (Neural recognition molecule NB-3) (hNB-3.

[Source:Uniprot/SWISSPROT;Acc:Q9UQ52]

AGTATTTAGTCTGTATCTCAAATAACTCTTCAATCTGCCTCGTCCTTTTGATCCTTTTAGGTCAGTCCTTCATTATCTCC

TGTTAGAAGTCTCCTAATTT

In100 candidate580 (in intron):

LOCUS ENSG00000134115

DESCRIPTION Contactin-6 precursor (Neural recognition molecule NB-3) (hNB-3.

[Source:Uniprot/SWISSPROT;Acc:Q9UQ52]

CATTTCTTGTCAACTTTTTCCTTAAGAAGAATAACTTGACTTCTCTAAATACTCCTTTATTTCTCTCAGGGCATATGCAT

TTACTTACCCTATACTCTTT

In100 candidate581 (in intron):

LOCUS ENSG00000175600

DESCRIPTION Uncharacterized protein C7orf10 (Dermal papilla-derived

protein13). [Source:Uniprot/SWISSPROT;Acc:Q9HAC7]

AATAGCTGTAACAATTTACCACATTAAACATTGTTTTAGCTACTTCCCTTTTTCTGATGCTTTGCCTATCAGTTTACCTT

CTGATTATAATTTGGTTTTC

In100 candidate582 (in intron):

LOCUS ENSG00000175600

DESCRIPTION Uncharacterized protein C7orf10 (Dermal papilla-derived

protein13). [Source:Uniprot/SWISSPROT;Acc:Q9HAC7]

TGTAAGCCTTCTTAAGAACTCTTTGTAGGTTGATAACATTATGCCCAACTCATATTTGTTTTGACTAGTCTTTCTCAGTT

TGTGTTTTAATTTTATTTCT

In100 candidate583 (in intron):

LOCUS ENSG00000175600

DESCRIPTION Uncharacterized protein C7orf10 (Dermal papilla-derived

protein13). [Source:Uniprot/SWISSPROT;Acc:Q9HAC7]

GCAAATCTGCTATTGAGTCCTGGTGATTTTAACATTTCTGTTATTGTACTTTTCAACTCCAGATACTCTGTTCAGTGAGG

CACCTTTCTCATACCTTTTT

In100 candidate584 (in intron):

LOCUS ENSG00000176788

DESCRIPTION Brain acid soluble protein 1 (BASP1 protein) (Neuronal axonal

mmbrane protein NAP-22) (22 kDa neuronal tissue-enriched acidic protein).

[Surce:Uniprot/SWISSPROT;Acc:P80723]

AGAATGAAATAGTGTGTTTTTCTTCTGCACCCTAAGGATTTAAATAACATAAAAAATTATTTCTTAATGTTTTACAGACT

TTCCATACTCAGTACATTTT

In100 candidate585 (in intron):

LOCUS ENSG00000110799

DESCRIPTION von Willebrand factor precursor (vWF) [Contains: von

Willebrandantigen 2 (von Willebrand antigen II)].

[Source:Uniprot/SWISSPROT;Acc:P0427]

AGCAATGCCTGGCAAAACATTTCCTTCTGCATGAGAAGTAACTCCCCTTGGCATGTGCCAATGCTTCTCTTTCAGCCCCA

GTCTTAGGATTTGTTCTCTT

In100 candidate586 (in intron):

LOCUS ENSG00000106571

DESCRIPTION Zinc finger protein GLI3.

[Source:Uniprot/SWISSPROT;Acc:P10071]EXON

CTTTGTGCGTTTTCAAAGCCCTGCTGCTTCTTGCATGTCTAACTGGCATTTTTATAACTCTGTTCCTAGTATTCTCAGTG

TATTCTTCTTTTGTTTCTCG

In100 candidate587 (in intron):

LOCUS ENSG00000106571

DESCRIPTION Zinc finger protein GLI3.

[Source:Uniprot/SWISSPROT;Acc:P10071]EXON

TAACACACAAAATCACTTCATGAACATTCACTTCTCTTGTTTCTCTTTACTTTTTCTTTCCTCTTGCTCTCTGCCCAGGT

CTCTTGACTTTTTACAGCAT

In100 candidate588 (in intron):

LOCUS ENSG00000145526

DESCRIPTION Cadherin-18 precursor (Cadherin-14).

[Source:Uniprot/SWISSPROT;cc:Q13634]

TGGAATATACAATTATGTGGATAGCTGATATAACATTGAATGTGATAAACCTTTCTCTTTCTTATCCAGATTTATGAGCT

CTTAATCTTCTCTCAGGCCT

In100 candidate589 (in intron):

LOCUS ENSG00000145526

DESCRIPTION Cadherin-18 precursor (Cadherin-14).

[Source:Uniprot/SWISSPROT;cc:Q13634]

AGTATTTAATAACTTTTCTATATTTCTAATTTATTTCAGGACTGTTTAGTTCTCCTTTTATTGTTCTCATTCTGTTCCAT

TAATAGGTCTTAAGTTTACC

In100 candidate590 (in intron):

LOCUS ENSG00000145526

DESCRIPTION Cadherin-18 precursor (Cadherin-14).

[Source:Uniprot/SWISSPROT;cc:Q13634]

GTATTAATGTATAAAGTATATACTTACATTTCTAACATATTTAACACTTTGTTTGCTTCTCTCTGCTTCTATATCCAGTT

TCTTCTTCCTGGCTTCAATT

In100 candidate591 (in intron):

LOCUS ENSG00000145526

DESCRIPTION Cadherin-18 precursor (Cadherin-14).

[Source:Uniprot/SWISSPROT;cc:Q13634]

TCATTAAGATGAAAGGCTTTCAATTTTTCTTCTTGTGACCCTAACAAAGTGTTTTTATTCACTTATTCCAGAAATATTA

AACTTTCCTTCTCATATTTT

In100 candidate592 (in intron):

LOCUS ENSG00000145526

DESCRIPTION Cadherin-18 precursor (Cadherin-14).

[Source:Uniprot/SWISSPROT;cc:Q13634]

TGGTCCTGGACTTTTTTTTGTTGTTGGTAACTTTTTAATTACCCTGTCAATCTCACTGCTTGTTATTTGTCTGTTCAGAG

TTTATATTTTTTTCCTTGTT

In100 candidate593 (in intron):

LOCUS ENSG00000154162

DESCRIPTION Cadherin-12 precursor (Brain-cadherin) (BR-cadherin) (N-cadheri

2) (Neural type cadherin 2). [Source:Uniprot/SWISSPROT;Acc:P55289]

TTTCAAACCTAGAACATAATTAATTTTTCTTCTAATGCTATAACCCACTTAATTTCTTCTTTCTCTTTCAAGTAGCAGTT

TACATGTATTTTCCCCTTAG

In100 candidate594 (in intron):

LOCUS ENSG00000154162

DESCRIPTION Cadherin-12 precursor (Brain-cadherin) (BR-cadherin) (N-cadheri

2) (Neural type cadherin 2). [Source:Uniprot/SWISSPROT;Acc:P55289]

CCTTGGACCATTTCTTTCCTTGCCTTCTGAGTAACCACACTTGCCTATCTTTTACCTCACTTCTTATTGTTTCTCAGTTT

TCATTACTGCCACGTCTTAT

In100 candidate595 (in intron):

LOCUS ENSG00000154162

DESCRIPTION Cadherin-12 precursor (Brain-cadherin) (BR-cadherin) (N-cadheri

2) (Neural type cadherin 2). [Source:Uniprot/SWISSPROT;Acc:P55289]

AAAAATATTCTAAAATAAATGTTTATTAGAAAAGATAGTTAACATTCTGACTGTATATGTTTGTCTTTCCAGCTTTGTTA

CCTAACAGCTTGGCTCTTTT

In100 candidate596 (in intron):

LOCUS ENSG00000154162

DESCRIPTION Cadherin-12 precursor (Brain-cadherin) (BR-cadherin) (N-cadheri

2) (Neural type cadherin 2). [Source:Uniprot/SWISSPROT;Acc:P55289]

TTTGGAAAGTTCTCCGTTATTATCTTTTGAATAAACTTTCTAACCCTGTCTTTTTCTCTATCTCCTTTTTAAGGTCAGTA

ATTCTTAGATTTGCCCTTTT

In100 candidate597 (in intron):

LOCUS ENSG00000154162

DESCRIPTION Cadherin-12 precursor (Brain-cadherin) (BR-cadherin) (N-cadheri

2) (Neural type cadherin 2). [Source:Uniprot/SWISSPROT;Acc:P55289]

TAACCATAACAAAGTTTGTTGTGTCTTATGTATGTTGCTGTCTGTCCTCTACTTCTCTATTCTCTTACGGTCAACCAGCT

TCATTTGTTTCTGTGATCTG

In100 candidate598 (in intron):

LOCUS ENSG00000154162

DESCRIPTION Cadherin-12 precursor (Brain-cadherin) (BR-cadherin) (N-cadheri

2) (Neural type cadherin 2). [Source:Uniprot/SWISSPROT;Acc:P55289]

TAGGTTTACTGTTTTTAAAATGTAATGTTATTCAAGGAATAACAAATTTTTCTTTTTTTTTCAACTTTCATGTCCCAGAT

CATCCTTTCTTCCTTTTTTT

In100 candidate599 (in intron):

LOCUS ENSG00000154162

DESCRIPTION Cadherin-12 precursor (Brain-cadherin) (BR-cadherin) (N-cadheri

2) (Neural type cadherin 2). [Source:Uniprot/SWISSPROT;Acc:P55289]

CATATTTCTTTCAAATCTGCAATATAACTTACCTATCCATTATACTTATGACTTATTTGCTACTTTCTTCTTCAGGGTCA

GATTTTCTTCTGTTTAGTTT

In100 candidate600 (in intron):

LOCUS ENSG00000104228

DESCRIPTION Tripartite motif-containing protein 35 (Hemopoietic lineage swich protein 5). [Source:Uniprot/SWISSPROT;Acc:Q9UPQ4]

GTCATGGAATTGTATTTTTGTCTTTAACTTTATGATAGTCTCTTGCCATACACACCTCTTATTTTTCTGTGTATTCAGTT

ATCTCATTCTTTCTTCAAGG

In100 candidate601 (in intron):

LOCUS ENSG00000072756

DESCRIPTION tRNA-nucleotidyltransferase 1, mitochondrial precursor (EC 2.7..25) (Mitochondrial tRNA nucleotidyl transferase, CCA-adding) (mt tRNA adenlyltransferase) (mt tRNA CCA-pyrophosphorylase) (mt tRNA CCAdiphosphorylae) (mt CCA-adding enzyme). [Source:Uniprot/SWISSPROT;Acc:Q96Q11]

TGTTTTTAATTTCAAGATGATATAACTTGTTTTGGATTTTTCACTTACTGTGCAGGGCACTTAGTGGGCCCTCATGTTTC

TTTCATAATTTCATCCCTTT

In100 candidate602 (in intron):

LOCUS ENSG00000140650

DESCRIPTION Phosphomannomutase 2 (EC 5.4.2.8) (PMM 2). [Source:Uniprot/SWISPROT;Acc:O15305]

TACTGAAAATCAACCTTGGCAACCCACTAACTGACAAAAGAGTAAATTGTTTTTTTCAGTGACATATCATTAGCCCCTTT

TTCACCTTTTGCCTTTGTGT

In100 candidate603 (in intron):

LOCUS ENSG00000150457

DESCRIPTION Serine/threonine-protein kinase LATS2 (EC 2.7.11.1) (Large tumo suppressor homolog 2) (Serine/threonine-protein kinase kpm) (Kinase phosphrylated during mitosis protein) (Warts-like kinase). [Source:Uniprot/SWISSPOT;Acc:Q9NRM7]

GTGATGGGGGAGAGGACATCCCTGCATCCTGGCTTTAACATGGAACTTTCTCATGGTTCTTTTTCTTCAGTCTGCAGTGT

CTCTTGTGGCTCTTGTCCTT

In100 candidate604 (in intron):

LOCUS ENSG00000144455

DESCRIPTION Sulfatase-modifying factor 1 precursor (C-alpha-formyglycinegnerating

enzyme 1). [Source:Uniprot/SWISSPROT;Acc:Q8NBK3]

TGAATTTATTTAACTGAATTTATTTACTCTTCTTATTTCTACTACCAAACTTTGATATCGTTCCCTCTTTGTATACAGTT

ATTTCCCCTTCTATTTATAA

In100 candidate605 (in intron):

LOCUS ENSG00000035928

DESCRIPTION Replication factor C subunit 1 (Replication factor C large subuit) (RF-C 140 kDa subunit) (Activator 1 140 kDa subunit) (Activator 1 largesubunit) (A1 140 kDa subunit) (DNA-binding protein PO-GA). [Source:Uniprot/WISSPROT;Acc:P35251]

CCGTATTGTTAACTAACCTGTACATATTTTTCGAACCTTACATTTTTCCCTGTAGTTCTTCTGTTCCAGGGTCCCACACT

GCATTTAGTTGTGTCTTCTT

In100 candidate606 (in exon):

LOCUS ENSG00000113361

DESCRIPTION Cadherin-6 precursor (Kidney-cadherin) (K-cadherin).

[Source:Unprot/SWISSPROT;Acc:P55285]

TGAATGGTTTCCGATTTTATAATGGACTGCCCTATATAGTAACAAGTATTTCATGCTTGAGCTATTTCCTGCTTTCAGGG

TTTCTTTTTTCTAGTTCTTC

In100 candidate607 (in intron):

LOCUS ENSG00000137845

DESCRIPTION ADAM 10 precursor (EC 3.4.24.81) (A disintegrin and

metalloprotinase domain 10) (Mammalian disintegrin-metalloprotease) (Kuzbanian

proteinhomolog) (CD156c antigen) (CDw156). [Source:Uniprot/SWISSPROT;Acc:O14672]

ATCATTGTGGTTTGGTGGTTTTCTTTAGTGGTAACATTTGACTTCTTTTTCTCATTTGTGTATCTGCTCTACCAGTTAGC

TTTATATTTCTGTGTGTTTT

In100 candidate608 (in intron):

LOCUS ENSG00000150995

DESCRIPTION Inositol 1,4,5-trisphosphate receptor type 1 (Type 1 inositol

14,5- trisphosphate receptor) (Type 1 InsP3 receptor) (IP3 receptor isoform)

(InsP3R1) (IP3R). [Source:Uniprot/SWISSPROT;Acc:Q14643]

TTGTGTGTAGAGTAATAACCTTTTAATGCAATGGGCTACTCTTTTATTTTCCCAGGCTATGGTGATTTTTTTTCAACTTT

GTTTATGATGGTTCTTTTTT

In100 candidate609 (in intron):

LOCUS ENSG00000166669

DESCRIPTION activating transcription factor 7 interacting protein 2

[SourceRefSeq_peptide;Acc:NP_079273]

CATGGTTGTTTTAATGCTCTTGTTTTAACTCAAGTGTTTGGATCACCTGTGCATCTGTTTCTATTGTTTTTCTGTCAGTC

TCTGGTTTTTTTTTTGGCAT

In100 candidate610 (in intron):

LOCUS ENSG00000021645

DESCRIPTION neurexin 3 isoform alpha precursor

[Source:RefSeq_peptide;Acc:N_004787]

CTGGCTTGTGCAATAGGCTCTAACCATATTGACTTTTTTTTTTTTTAATCAGGCCTCATATGAACCCATTTCTTTCAGTC

ATAGAGTCTTTTTTGTTTTT

In100 candidate611 (in intron):

LOCUS ENSG00000021645

DESCRIPTION neurexin 3 isoform alpha precursor

[Source:RefSeq_peptide;Acc:N_004787]

TTTCTCCTCCCTATTTTCTTCTTCTAAATATGTCTTTTTAACCTCTGCATCTTTGTTCTTGCAATTTCCTTCATCAGAAT

TTTCTTCCCTTCCTTTTCCT

In100 candidate612 (in intron):

LOCUS ENSG00000021645

DESCRIPTION neurexin 3 isoform alpha precursor

[Source:RefSeq_peptide;Acc:N_004787]

ACTATCATCCTCATTTCTTTAACATGTATTTCTAGATCCTTTTTTGCTCAGAACATTGTTATGGCATTCCTCAGAACGCT

TTTAGTCTCTCTCTTTCTCT

In100 candidate613 (in intron):

LOCUS ENSG00000021645

DESCRIPTION neurexin 3 isoform alpha precursor

[Source:RefSeq_peptide;Acc:N_004787]

TTTAAGGTTTACTCTGTACTTTCATCCCTTTCTATGGGGAAGTAACTCTCTTATTTCATCCTTTTTATCTTTGTTCAGCA

CACACGTGCTCTTTTTATTT

In100 candidate614 (in intron):

LOCUS ENSG00000021645

DESCRIPTION neurexin 3 isoform alpha precursor

[Source:RefSeq_peptide;Acc:N_004787]

AGCTGTTTAACGATGCCATTTGGGAGCCACTCTCTTCTATCTTTCTGTTTCATCATCCTTGATGTATATGCTTTCAGCCT

CCAGTTTGTTGCCTCTTAGT

In100 candidate615 (in intron):

LOCUS ENSG00000021645

DESCRIPTION neurexin 3 isoform alpha precursor

[Source:RefSeq_peptide;Acc:N_004787]

GTAAAAGGAATAAATTATATATTTATCATAAATTTTAACTCTACAAAATGGCCATATTTATACTTTCTTCAGATTTCTCT

CAGTTCTAGTTAAGGTTTTT

In100 candidate616 (in intron):

LOCUS ENSG00000021645

DESCRIPTION neurexin 3 isoform alpha precursor

[Source:RefSeq_peptide;Acc:N_004787]

AGTTTTCACATCTATTAAATGTGCTGATTTAACTAAACTATCTTTATTTCCTTTAGGCTCAGACATTTCCTATTTCTTTT

ATTTCTATAACTGTACTTAT

In100 candidate617 (in intron):

LOCUS ENSG00000021645

DESCRIPTION neurexin 3 isoform alpha precursor

[Source:RefSeq_peptide;Acc:N_004787]

AGTTAGAAATTCTAAGTGGTCCTAAGGAATGACCAAATTGAATAACTCAAGTTTTCTTTCTTTTGTCATTACAATCAGTG

TGCTATCACTTTTTATTTTA

In100 candidate618 (in intron):

LOCUS ENSG00000021645

DESCRIPTION neurexin 3 isoform alpha precursor

[Source:RefSeq_peptide;Acc:N_004787]

TAACCTTGCCCTAAAGTCCTGCTCTATACTGGTAACCCTTTTATATCCTTTTCAGACATTTCCCTTCATAGTTCTAGCAA

TATGCTTGCATTTCTATTTC

In100 candidate619 (in intron):

LOCUS ENSG00000021645

DESCRIPTION neurexin 3 isoform alpha precursor

[Source:RefSeq_peptide;Acc:N_004787]

CAATTCCCTCTCTAACCCAAGGCAAAATGTTCAACTCTTTTTTGTATTCCCTTAAGAGCCCTTATTTTTTTTTTCAGTTT

CACCAATCATGTTCATTTCT

In100 candidate620 (in intron):

LOCUS ENSG00000021645

DESCRIPTION neurexin 3 isoform alpha precursor

[Source:RefSeq_peptide;Acc:N_004787]

CTAAATTTGGCTAACTTCCTTGGCCTTGTTCTATCATACTTACTACTTTATCTTCTTTCACTTTGCCAGCGAGTTTATTA

TTTTCTAGCTGTCTCCGTTT

In100 candidate621 (in intron):

LOCUS ENSG00000113360

DESCRIPTION Ribonuclease 3 (EC 3.1.26.3) (Ribonuclease III) (RNase III)

(Drsha) (p241). [Source:Uniprot/SWISSPROT;Acc:Q9NRR4]

GAACCCATCACTTTTAACCCCCAAATGTGTTCCTCCTTCCTTATTTTTATCTTCTAAACCAGAAACCTTAGGATTTATTT

TTGTCCCTAAGCTTTCTTCT

In100 candidate622 (in intron):

LOCUS ENSG00000128923

DESCRIPTION family with sequence similarity 63, member B (FAM63B), transcrit variant 1, mRNA [Source:RefSeq_dna;Acc:NM_001040450]

ACCTGCACCTGGACAAAGTATTCTCTGTTTATTAACTTCATGATTTCATCTTTATCTATTTATTTATTTTTACCACAGCT

GATTTTATCTTTTTTTTTTT

In100 candidate623 (in intron):

LOCUS ENSG00000100629

DESCRIPTION Uncharacterized protein C14orf145.

[Source:Uniprot/SWISSPROT;Ac:Q6ZU80]

AGATAAAGTTGAAAATTTAGAATGAAGAATTTCTGTATAACATTTACCTAGATTCCCTTATTATTCATATTTTACCAGAC

TTGCTTCATCATTTATTCTA

In100 candidate624 (in intron):

LOCUS ENSG00000100629

DESCRIPTION Uncharacterized protein C14orf145.

[Source:Uniprot/SWISSPROT;Ac:Q6ZU80]

ACTCTTGGGCCCATTATAAATTTGAGATTATCTAACTAGAACCTTATTTCTGTTTGTTATCTTTTAGACATCTATCAGTT

GTTCATTTCCTCTTTTCCTA

In100 candidate625 (in intron):

LOCUS ENSG00000100629

DESCRIPTION Uncharacterized protein C14orf145.

[Source:Uniprot/SWISSPROT;Ac:Q6ZU80]

AGTAACTTTGCTTAAATTGCATTCGTTTTTATTTCAGCCTTTAATATTATTTGATTTCTCTCTTTCTCTACCTTTTATCC

TATCTCCCCCTACCCTTTGT

In100 candidate626 (in intron):

LOCUS ENSG00000180776

DESCRIPTION Probable palmitoyltransferase ZDHHC20 (EC 2.3.1.-) (Zinc fingerDHHC domain-containing protein 20) (DHHC-20). [Source:Uniprot/SWISSPROT;AccQ5W0Z9]

GAAGTTCTTATAAAAGAACTTTTAACTTAAATCATTATCTCTTGGGGCTATTTTTACCGTGTCTCTCAGAGTCTTTTTTT
TTTTTTTGAGACAGAGTCTC

In100 candidate627 (in intron):

LOCUS ENSG00000165409

DESCRIPTION Thyrotropin receptor precursor (TSH-R) (Thyroid-stimulating horone receptor). [Source:Uniprot/SWISSPROT;Acc:P16473]

GATGTGAGACATGGAGTCAAAGGAGATCGTTTTGGAGCTTTAACATTTGACTGTCCTGCTGTATTTCAGACTTGCTGACT
TTTTGGACTTGTAGCCCCTT

In100 candidate628 (in intron):

LOCUS ENSG00000165409

DESCRIPTION Thyrotropin receptor precursor (TSH-R) (Thyroid-stimulating horone receptor). [Source:Uniprot/SWISSPROT;Acc:P16473]

TTCTATAACTCCATTTACATTTTGTTTGAATTTCCCTTTCAGTAATATTACATTTCATTTCTTGGCTTCATTTTCATCTT
TATTGGTAAATTCACTCTTT

In100 candidate629 (in intron):

LOCUS ENSG00000147419

DESCRIPTION Coiled-coil domain-containing protein 25.

[Source:Uniprot/SWISSROT;Acc:Q86WR0]

TGAATGGACCTTCTTGTCTTAACCACCTCTCTAATCTTGTTTGCTACAGATTATTCTTTTCAAAACATTGGTATTATGTA

GTCTCACTTTCCTTTGTCTC

In100 candidate630 (in intron):

LOCUS ENSG00000157483

DESCRIPTION Myosin-Ie (Myosin-Ic). [Source:Uniprot/SWISSPROT;Acc:Q12965]

AGGCGTGAGCCACCGCGCCTGGCTATATATAATTAATATAACAACCTAGATACCTTTTCAACTACAGTCTAGGTTTTTAT

TCTCCTTTGTTCCCTATTAG

In100 candidate631 (in intron):

LOCUS ENSG00000150712

DESCRIPTION myotubularin related protein 12

[Source:RefSeq_peptide;Acc:NP_01035536]

GTCTGAGGTACACAAAGGAAGAGGAAGTCAAAAGGGTAACTAGTTGCATGTGTTTTTAGGTTTATTTCCCATCAGGTTT

TCCCCACTAGCTCTAGTTTT

In100 candidate632 (in intron):

LOCUS ENSG00000134109

DESCRIPTION ER degradation-enhancing alpha-mannosidase-like 1.

[Source:Unipot/SWISSPROT;Acc:Q92611]

AGGCTCCTCTTGTTTCCGTGACACCATCTAACTGTTTGAAGATGATTTTCCTTTATTTGCTCAGTTCTTCTTTTGATTCC

TTTCCTTTGAGCTGCGATTC

In100 candidate633 (in intron):

LOCUS ENSG00000165487

DESCRIPTION EF-hand domain-containing family member A1.

[Source:Uniprot/SWISPROT;Acc:Q8IYU8]

CATACTGACATATTGATTCTTTGAAGAGAAATTTAGAACTCATATCTAACAACCCCTACCCTTGTGTTTCCTTAACAGCT

TTCTTTTCAAAGTTTTTTTA

In100 candidate634 (in intron):

LOCUS ENSG00000196277

DESCRIPTION Metabotropic glutamate receptor 7 precursor (mGluR7).

[Source:Uiprot/SWISSPROT;Acc:Q14831]

CTATCTCTACTTCCATAATTAATTATATACTAACATTTGATTGCATGACCTCTTTACAGTGTTACTGTGTACTAATGCTT

AGTCTTATTTTGTTTTCTCT

In100 candidate635 (in intron):

LOCUS ENSG00000196277

DESCRIPTION Metabotropic glutamate receptor 7 precursor (mGluR7).

[Source:Uiprot/SWISSPROT;Acc:Q14831]

GAGGTCGTAAATCTGGGAGAGTGCCTAACTGTGGCTCAACTTTTTTTCTCCTGGGACAGCCTGTTTGATTGTTCACCTGC

TTTTAATGTTCTTGTGATTT

In100 candidate636 (in intron):

LOCUS ENSG00000056097

DESCRIPTION zinc finger RNA binding protein

[Source:RefSeq_peptide;Acc:NP_07191]

TAAATCTGCTGGCAATTACTAGTATTCCGTCTAGAAAAGATTGTGTTTAACCATTAAAATTTTACTTGTTTTACTCAGGT

TTTTTCTGTTTTCCTCATAT

In100 candidate637 (in intron):

LOCUS ENSG00000056097

DESCRIPTION zinc finger RNA binding protein

[Source:RefSeq_peptide;Acc:NP_07191]

AGATTTCTGAAGTGGAAAATCCACTAACATTATTTCTTTGTTTTTATTGCCTTCTCCAGCTGTTTACCTAGTCTTGTTTC

TATCTCTATAATATTTCTAC

In100 candidate638 (in intron):

LOCUS ENSG00000108953

DESCRIPTION 14-3-3 protein epsilon (14-3-3E).

[Source:Uniprot/SWISSPROT;AccP62258]

AACTGCACATCATTAACATGGTCTTAATGCCTTTATCTTATTGCTGCAGATTTTTTCTTAACCTTTAACTTTTCTGGTTT

CAGAATGCTGCCAACATTTC

In100 candidate639 (in intron):

LOCUS ENSG00000121892

DESCRIPTION SCC-112 protein [Source:RefSeq_peptide;Acc:NP_056015]

ATTCTGAGTTAATTCTTATATGTATTAACAATCTAGTTTCTTTTTTTGTTTGTGTGTTTGCTTCTTGTGGATATCCAGGA

GTTTTTTTGTTTTGCTTTTG

In100 candidate640 (in intron):

LOCUS ENSG00000188897

DESCRIPTION CDNA FLJ44575 fis, clone UTERU3018154.

[Source:Uniprot/SPTREMBLAcc:Q6ZTK2]

GTCACTTCCTCCTAGAAGCCTTTGCTGACCTAACCAATTTTTATCAAATTCCTTCTTTATTGTTTTTCTTCACAGCCCTC

AGCATTTCTACCTGTGTTTT

In100 candidate641 (in intron):

LOCUS ENSG00000151388

DESCRIPTION ADAMTS-12 precursor (EC 3.4.24.-) (A disintegrin and

metalloproeinase with thrombospondin motifs 12) (ADAM-TS 12) (ADAM-TS12).

[Source:Unirot/SWISSPROT;Acc:P58397]

AGAAACCTTCCCTCTAAATACTATTTTCATTGTAACCCATAAATTCACATGTTTTGTTTCCATCTTTATGTAATTCAGTA

TTTTTTGTAGTTGCTCTTTT

In100 candidate642 (in intron):

LOCUS ENSG00000151388

DESCRIPTION ADAMTS-12 precursor (EC 3.4.24.-) (A disintegrin and

metalloproeinase with thrombospondin motifs 12) (ADAM-TS 12) (ADAM-TS12).

[Source:Unirot/SWISSPROT;Acc:P58397]

TAATTTGCATTTCCCTGATAGCTAATGATGTTGACTAACTTTTCATGTGCTTTTTTGACATCTGGATATCCTCTTCAGTT

CTCTTTGTGTAGTTTGCTTG

In100 candidate643 (in intron):

LOCUS ENSG00000042317

DESCRIPTION Spermatogenesis-associated protein 7 (Spermatogenesis-associate

protein HSD3) (HSD-3.1). [Source:Uniprot/SWISSPROT;Acc:Q9P0W8]

GTGTTTCATTTTGTTTATTAAATAACCATTACCGATAACATTTCTTTACAGGCAATCATAATGGCTTATGCATTTCTTTC

TCCTTCCTCTGATCTCAGTT

In100 candidate644 (in intron):

LOCUS ENSG00000163694

DESCRIPTION RNA-binding protein (FLJ20273), mRNA

[Source:RefSeq_dna;Acc:NM_19027]

TTTTAGGGGAATTGACCCAAACTGATTATCTTCGTAACCTATCTGATGTTCTCTTCATTTTTTGTTCTTTTCCTCCAGTT

TTGCCAATTCTGATGCTTTT

In100 candidate645 (in intron):

LOCUS ENSG00000082196

DESCRIPTION Complement C1q tumor necrosis factor-related protein 3 precurso

(Secretory protein CORS26). [Source:Uniprot/SWISSPROT;Acc:Q9BXJ4]

CTGAGGGAGGGTCTCCATGATAACCTCATTGAATTAGATTATCTTTGTCTTTCCCTCTCTAATTTCCAGCAGTTGACTTT

CGTCCTCATTCATGCTGTCT

In100 candidate646 (in intron):

LOCUS ENSG00000082196

DESCRIPTION Complement C1q tumor necrosis factor-related protein 3 precurso

(Secretory protein CORS26). [Source:Uniprot/SWISSPROT;Acc:Q9BXJ4]

AAGCAAAGACATGAGAATCCTCTTAATCTGGATACGCTGTGTTTGAAATAACGTGTTTGGTGCTTTCCACTTCTTCAGTT

TTGTTATTTATTTATTTTTT

In100 candidate647 (in intron):

LOCUS ENSG00000129003

DESCRIPTION Vacuolar protein sorting-associated protein 13C.

[Source:Unipro/SWISSPROT;Acc:Q709C8]

AGTCATTTTTCTTCTTTTGCCTTGCAAAACAAACACTTTATAACTTATGTCACCGTGATTACTTTTTCCAGTTCATGCGT

TGTTTTGTTTCTATCTTCTT

In100 candidate648 (in intron):

LOCUS ENSG00000105953

DESCRIPTION 2-oxoglutarate dehydrogenase E1 component, mitochondrial precuror

(EC 1.2.4.2) (Alpha-ketoglutarate dehydrogenase).

[Source:Uniprot/SWISSPOT;Acc:Q02218]

ACTGATTTCCTCTGTCTTTATTCTGCCCTTTAACCCATGCTAAAGCTTTTTTCTTTTGCTTAGTGCATTTTTCAGTTCTG

AAATGTCTGTTTTGTTCTCT

In100 candidate649 (in intron):

LOCUS ENSG00000196220

DESCRIPTION SLIT-ROBO Rho GTPase-activating protein 3 (srGAP3) (srGAP2)

(WAEassociated

Rac GTPase-activating protein) (WRP) (Mental disorder- associted GAP)

(Rho GTPase-activating protein 14). [Source:Uniprot/SWISSPROT;Acc:43295]

GGAAAATATAAGAATCTAGAAGCCCCTACCTGAAATAACCACTATAAACATTCAATGTATTTCCTTCCAGACTTCACGTC

TCTATCAATCACATTTGTTT

In100 candidate650 (in intron):

LOCUS ENSG00000196220

DESCRIPTION SLIT-ROBO Rho GTPase-activating protein 3 (srGAP3) (srGAP2)

(WAEassociated

Rac GTPase-activating protein) (WRP) (Mental disorder- associted GAP)

(Rho GTPase-activating protein 14). [Source:Uniprot/SWISSPROT;Acc:43295]

CACCCTAGCTTGGACCTCACGGGCACTTGGATGAGCTGACGGAAAGTAACACACGGGCAACTTCTGCTTTCTCTTCAGCT

TGTTCAACATTCCCTTTTGC

In100 candidate651 (in intron):

LOCUS ENSG00000048471

DESCRIPTION

TTCCTGTAAAAGTAACATGTTCACATGTTAAAGTAAGCCTTTTTGCTATACTCTTGCCTTCCTCTTCAGTCATGCTTCTT

ACCTTGCTCTGAGCATGTTT

In100 candidate652 (in intron):

LOCUS ENSG00000163697

DESCRIPTION Amyloid beta A4 precursor protein-binding family B member 2 (Fe5-

like protein). [Source:Uniprot/SWISSPROT;Acc:Q92870]

GTCATAAAATGAATGGCTTTCCGACTTAATTATACGTTTAAGTGATAACGACAATGAGTTTTATCTCTTTGGCTACAGTT

GGTTTTCTGATTCTCAATTC

In100 candidate653 (in intron):

LOCUS ENSG00000165521

DESCRIPTION echinoderm microtubule associated protein like 5

[Source:RefSeqpeptide;Acc:NP_899243]

GGATATCTTTTTCTGTTGTTATCACTTTGAATAACCTTTCTACTCCTGTCTCTTTATCTAGCTCCTCTTTAAGGCCAGTA

ACTCCTTTATTTGCCCTTTT

In100 candidate654 (in intron):

LOCUS ENSG00000165521

DESCRIPTION echinoderm microtubule associated protein like 5

[Source:RefSeqpeptide;Acc:NP_899243]

AATCTTGTATTTAACTCTTCCATTGAATTTTTAACTTATTTTATTTTTTGTTTCTGGAAGTTCCGTTTGCTTCTTCAGGT

CTCCTTGGATACTTTTTATA

In100 candidate655 (in intron):

LOCUS ENSG00000165521

DESCRIPTION echinoderm microtubule associated protein like 5

[Source:RefSeqpeptide;Acc:NP_899243]

ATAGTGCCTAACACATGGTCTGCTTTCTGAAAGTGTTGCTTTTCCTTTTTCTATATTTGCTCCCTGATTTGACCTCAGCC

TTTAAATTTTTTTTTGACA

In100 candidate656 (in intron):

LOCUS ENSG00000165521

DESCRIPTION echinoderm microtubule associated protein like 5

[Source:RefSeqpeptide;Acc:NP_899243]

CCATTTAACCTGCTGCCAATCTGTTGATCACTTCCTGGTTCTCAGTGTATTCACCTCACAGCAGTTTGTGACACAATTTA

TCATTCCCTTTTTTTTTTCT

In100 candidate657 (in intron):

LOCUS ENSG00000171914

DESCRIPTION Talin-2. [Source:Uniprot/SWISSPROT;Acc:Q9Y4G6]

AGTTATGGGTAACTGACATTGGATTTCTTACTCTATTCTTCTGATTCTTGAGATCATATCTCAGAGCTTTTCTTGATTTC

TTAATGTACTGCATGTTCCC

In100 candidate658 (in intron):

LOCUS ENSG00000140694

DESCRIPTION Poly(A)-specific ribonuclease PARN (EC 3.1.13.4) (Polyadenylate

specific ribonuclease) (Deadenylating nuclease) (Deadenylation nuclease).

Source:Uniprot/SWISSPROT;Acc:O95453]

AATCTGTAGTGATAACTGGTTTTTATTCGTGATATTACTCATTTGTGTCTCATTTTTCCTTGTTCATTCCAGTCAGCTGT

TACCATTTTGTTGATCTCTC

In100 candidate659 (in intron):

LOCUS ENSG00000064042

DESCRIPTION

GAAAACACTGTGTTTGAATAAATGTATTCCAATAGGCCTATTTATTAACTGGGAAACACTTACCTAGTCTTCAGAATTAA

TATTGCCCTTGCACTCCTTT

In100 candidate660 (in intron):

LOCUS ENSG00000147421

DESCRIPTION Homeobox-containing protein 1.

[Source:Uniprot/SWISSPROT;Acc:Q6T76]

AAAAAACCCATTTAATTCTTTTCCCCCACAAAAGTACATTCTTTTATTTAACCTGAATAAATATTCTCTTAAATTCAGCT

TCATTTTCGTAGGTCATTCT

In100 candidate661 (in intron):

LOCUS ENSG00000147421

DESCRIPTION Homeobox-containing protein 1.

[Source:Uniprot/SWISSPROT;Acc:Q6T76]

CCTTATGAATTTCTGTTTATCAAGATTATCTCTGTTGTTACCCTCATTTTAACTTCTTGACCAATTTAGTCCTTGCAGAC

CTCTCTTTTTTTCTGAAATT

In100 candidate662 (in intron):

LOCUS ENSG00000197892

DESCRIPTION Kinesin-like protein KIF13B (Kinesin-like protein GAKIN).

[Soure:Uniprot/SWISSPROT;Acc:Q9NQT8]

AGCATTTGGAATCTTTTTTTTTTTTTCCTTTGTTTTCTTTTCCTAACCTCCTGCAAGACTTTGTTTCTTTCTGTGCAGTT

TCATCTGTTAGTCATCGTTC

In100 candidate663 (in intron):

LOCUS ENSG00000053254

DESCRIPTION Checkpoint suppressor 1 (Forkhead box protein N3).

[Source:Unipot/SWISSPROT;Acc:O00409]

CAACTTCCTTTCTTCTAACTCATGGCTTATTCTGTTTCCTTCCTAGCAGGTATTACAGTCTGTCATAATCTTGCTTATTT

ATTTCTTTACTTACTGTGTT

In100 candidate664 (in intron):

LOCUS ENSG00000140025

DESCRIPTION Full-length cDNA clone CS0DG007YH10 of B cells (Ramos cell line

of Homo sapiens (human). [Source:Uniprot/SPTREMBL;Acc:Q86T09]

GATTTGAGGCTGCAACATTAACTCTTCCCTTGACTCTCTGTTATGTTGCATTGGTCTGTTTGTCTATCTTTGTGCCAGTA

TCACACTCTCTTTATTATAT

In100 candidate665 (in intron):

LOCUS ENSG00000109133

DESCRIPTION Transmembrane protein 33 (DB83 protein).

[Source:Uniprot/SWISSPOT;Acc:P57088]

GTAGCAAATTAACATTCATTTTTACTTGATTTCTTGAGTTTTAGCCTTTTTTCTTTCTGGTCCCTATATTCATTTCAGAC

ATTATCTTATATTCTCTTGC

In100 candidate666 (in intron):

LOCUS ENSG00000042088

DESCRIPTION Tyrosyl-DNA phosphodiesterase 1 (EC 3.1.4.-) (Tyr-DNA

phosphodisterase 1). [Source:Uniprot/SWISSPROT;Acc:Q9NUW8]

AGCACTTGCTATGCTAATATAAGAACATTTCTATTTTAATTTAACATCCTAAGTCTTTATGTTGCCACCACTTTCAGTGT

TTCTTTTTTTGTTTTTGGAG

In100 candidate667 (in intron):

LOCUS ENSG00000164742

DESCRIPTION Adenylate cyclase type 1 (EC 4.6.1.1) (Adenylate cyclase type I (ATP pyrophosphate-lyase 1) (Adenylyl cyclase 1) (Ca(2+)/calmodulin- activted adenylyl cyclase). [Source:Uniprot/SWISSPROT;Acc:Q08828]

TAAAAATATCTGATCTGTTTATTCATGTAGTAACTATTTCCTTTGCTTTTCATTCATTTATGCAGTCTATTTCTTTGTGT
TTTATGTATCTGCCTAAATT

In100 candidate668 (in intron):

LOCUS ENSG00000014824

DESCRIPTION solute carrier family 30 (zinc transporter), member 9 [Source:RfSeq_peptide;Acc:NP_006336]

AGGCTGTCATTAGATACATACATATTTAGAATTATTACCTAACACTGATGAATTATTCCCTCTATCATTGTGCAGCTACT
GTTTTTCTTTAGTGAGCTCT

In100 candidate669 (in exon):

LOCUS ENSG00000166128

DESCRIPTION Ras-related protein Rab-8B. [Source:Uniprot/SWISSPROT;Acc:Q9293]

AGGACCTAACTCGTTTTTTCCTTGTTTTATTACCTGTTGCAGAAGCGGTTATCTTTCTTTTTTACTTTGCACATCAGTGT
TAGCCTTTCCCTATTTCAGC

In100 candidate670 (in intron):

LOCUS ENSG00000152315

DESCRIPTION Potassium channel subfamily K member 13 (Tandem pore domain halthane- inhibited potassium channel 1) (THIK-1).

[Source:Uniprot/SWISSPROT;Ac:Q9HB14]

TCATCACCTCGAAATAACCTGATTGAAAATTTCGTGTATTTTTCCCCCCTTTTCTATGTATGTCCGTGGCAGACATTGTC

ATTTCTGAGGTCCATCTCTT

In100 candidate671 (in intron):

LOCUS ENSG00000132383

DESCRIPTION Replication protein A 70 kDa DNA-binding subunit (RP-A) (RF-A) Replication factor-A protein 1) (Single-stranded DNA-binding protein) (p70)

[Source:Uniprot/SWISSPROT;Acc:P27694]

TACTTGTTCTGTCATTTACTGAGAGGGATATTGAAATCTCTAACTGTAATAATTGTAAGTTTCTCTGTCTCTCTGCAGTT

CTATTAGTTTTTGCTTCATG

In100 candidate672 (in intron):

LOCUS ENSG00000113494

DESCRIPTION Prolactin receptor precursor (PRL-R).

[Source:Uniprot/SWISSPROTAcc:P16471]

TTTTATTTTCTCTACTTGCTCATTTCTAAATGATATTTTAACATCTATATTTTTTATTTGCTCTAATTCTCCAGTTTATT

CTCCATTCCATGCAAATCTT

In100 candidate673 (in intron):

LOCUS ENSG00000182326

DESCRIPTION Complement C1s subcomponent precursor (EC 3.4.21.42) (C1 esterae)

[Contains: Complement C1s subcomponent heavy chain; Complement C1s subcoponent light chain]. [Source:Uniprot/SWISSPROT;Acc:P09871]

CCTTTCCTACTTTTTCTTACCCTTATGGTTTTGGATTTAACCTCATTCTCCCTTCTTGGTGTTTGTTCTTGACCTCAGCC

TCTTTCTACTCTTTGTAGAC

In100 candidate674 (in intron):

LOCUS ENSG00000140455

DESCRIPTION Ubiquitin carboxyl-terminal hydrolase 3 (EC 3.1.2.15) (Ubiquiti thioesterase 3) (Ubiquitin-specific-processing protease 3) (Deubiquitinatig enzyme 3). [Source:Uniprot/SWISSPROT;Acc:Q9Y6I4]

TCATATTAAATTGAAATGCATTTGCTGAAAATGTCTCTTAACTGTTGTTTATTATCTTAAAACTCTTTTTTTACCCAGCA

GCTTTTTAACTTTGCTCTGC

In100 candidate675 (in intron):

LOCUS ENSG00000152582

DESCRIPTION KPL2 protein isoform 2 [Source:RefSeq_peptide;Acc:NP_653323]

GTGAACTAGTAAAGCTCTTTAGCTCATTGTAGTTAAACTAATAACCATCATTCTAGTCTGAATTTCTTCTTTCTCCAGTT

CTTCTGATCTCTTTTCACTC

In100 candidate676 (in intron):

LOCUS ENSG00000166780

DESCRIPTION Uncharacterized protein C16orf45.

[Source:Uniprot/SWISSPROT;AccQ96MC5]

AAATGTGTGAACAACCTTTTGTAATCTAACCTACTGACCTGCATGTTTTTTCTTTACCCCAGCTCATTCCTTACATGTAG

CCTCAATCTTCAGTTTGCTT

In100 candidate677 (in intron):

LOCUS ENSG00000070366

DESCRIPTION Telomerase-binding protein EST1A (EC 3.1.-.-) (Ever shorter

telmeres 1A) (Telomerase subunit EST1A) (EST1-like protein A) (hSmg5/7a).

[Souce:Uniprot/SWISSPROT;Acc:Q86US8]

TGTTTGTCTTTATTATTACGTCTTAAGAGTTCTCTCTATAACCTGGACATAAGCCCTTATCCGATTTATATTTTGCAGAA

ATTTTCTCCGGGTGCTTTCT

In100 candidate678 (in intron):

LOCUS ENSG00000070366

DESCRIPTION Telomerase-binding protein EST1A (EC 3.1.-.-) (Ever shorter

telmeres 1A) (Telomerase subunit EST1A) (EST1-like protein A) (hSmg5/7a).

[Souce:Uniprot/SWISSPROT;Acc:Q86US8]

TTATGATTGTTAACTTCTTTTTTTCTTTCACCTTTGAACTGTTGTCCAGTCTAAGACATCCTGTTATAGCATGTTCTATG

TATCTTTTTTCTATTTTTTT

In100 candidate679 (in intron):

LOCUS ENSG00000070366

DESCRIPTION Telomerase-binding protein EST1A (EC 3.1.-.-) (Ever shorter

telmeres 1A) (Telomerase subunit EST1A) (EST1-like protein A) (hSmg5/7a).

[Souce:Uniprot/SWISSPROT;Acc:Q86US8]

TTAAGTCTTCTATTGCATTATTTGTTCTCTTACATTATATCTTATAACCTTTATTTTTTTTGCCTGCCTTTTAGTCAGTC

CCTTTATATCTTTTTTTTTT

In100 candidate680 (in intron):

LOCUS ENSG00000183783

DESCRIPTION BTB/POZ domain-containing protein KCTD8.

[Source:Uniprot/SWISSPOT;Acc:Q6ZWB6]

AATGTGCCTAGAGGAGAAATTCTATTTTCTAACATCTCCCCTCTCTTTCTTGGTCCTTCTTACAGCATCTCATTTTACTC

AAGTCTGTGATCCTTGTCTT

In100 candidate681 (in intron):

LOCUS ENSG00000136273

DESCRIPTION Checkpoint protein HUS1 (hHUS1).

[Source:Uniprot/SWISSPROT;Acc:60921]

AGATCCTTTTAACTCTTTGTTTCTGTTTGGTAAATTTTATCTTTCTCCTTTCCATCCCAGTTTCTCTTACTATTCTCTCT

GTGGTGCCTATTTGCTCTAG

In100 candidate682 (in intron):

LOCUS ENSG00000100784

DESCRIPTION Ribosomal protein S6 kinase alpha-5 (EC 2.7.11.1) (Nuclear

mitoen- and stress-activated protein kinase 1) (90 kDa ribosomal protein S6

kinse 5) (RSK-like protein kinase) (RSKL). [Source:Uniprot/SWISSPROT;Acc:O7558]

CCATTTAACTATTCAAGCCCAAATTCTTGCCTTGCTTTCAGTTTCCCCTGTCTTTTTTCGCCCAGCTTACCCTGATATTC

TGCCTACTTTCTATTTTTTT

In100 candidate683 (in intron):

LOCUS ENSG00000100784

DESCRIPTION Ribosomal protein S6 kinase alpha-5 (EC 2.7.11.1) (Nuclear

mitoen- and stress-activated protein kinase 1) (90 kDa ribosomal protein S6

kinse 5) (RSK-like protein kinase) (RSKL). [Source:Uniprot/SWISSPROT;Acc:O7558]

CATAACATATGAACACCATATAATTCATAAAGCTTTTCCTTGTTCTTTTTTTATTCCTGCACAGTCCTTCGTTCCATTC

CATTGTATACTTAACTGGTT

In100 candidate684 (in intron):

LOCUS ENSG00000165392

DESCRIPTION Werner syndrome ATP-dependent helicase (EC 3.6.1.-).

[Source:Unprot/SWISSPROT;Acc:Q14191]

AGCAACACTATTAACTGAAGACTTCTATAATTTTTTTCTTTTATTTCTGATTCTTTCTTCGGTTTTCCCCCTCAGTTTTG

AACTTTTCTAATTTTGATTT

In100 candidate685 (in intron):

LOCUS ENSG00000132972

DESCRIPTION RING finger protein 17. [Source:Uniprot/SWISSPROT;Acc:Q9BXT8]

TCTTTTTTCCTAAGTTGTAAGAGTACTTTATGTGATAGCAATATTAACATTTCATCCTGTGTAACTGTTTTCTCCCAGCC

TCTCACTTTTGATTTTATTG

In100 candidate686 (in intron):

LOCUS ENSG00000132972

DESCRIPTION RING finger protein 17. [Source:Uniprot/SWISSPROT;Acc:Q9BXT8]

ATCTCCTATGTCCATAACCTATCTTCATTTCTACTGCCTTATTTAATTCTCATGTTATTTTGTGTAGCTCCTTGTCAGAC

TCTTCCTTAATGCTATAGTT

In100 candidate687 (in intron):

LOCUS ENSG00000168685

DESCRIPTION Interleukin-7 receptor alpha chain precursor (IL-7R-alpha) (CD17

antigen) (CDw127). [Source:Uniprot/SWISSPROT;Acc:P16871]

CCACTTCCTTGCCCACTCAAACATACTTACCCTTTAACATATCTCTTGACTTTTCCTGTCATATTGTAATCTGTCCAGAG

CCTCCTCTATTTGGGTTTTC

In100 candidate688 (in intron):

LOCUS ENSG00000168685

DESCRIPTION Interleukin-7 receptor alpha chain precursor (IL-7R-alpha) (CD17

antigen) (CDw127). [Source:Uniprot/SWISSPROT;Acc:P16871]

ATGTGATATTATGTATATAACCCTTTATACTATGTTATCATTTTCTTTAACACTTTTTTTAATCAATATTTTTTACAGCT

CTTATTTCTTACATATATTC

In100 candidate689 (in intron):

LOCUS ENSG00000157168

DESCRIPTION Pro-neuregulin-1, membrane-bound isoform precursor (Pro-NRG1)

[ontains: Neuregulin-1 (Neu differentiation factor) (Heregulin) (HRG) (Breas

cancer cell differentiation factor p45) (Acetylcholine receptor-inducing

ativity) (ARIA) (Sensory and motor neuro [Source:Uniprot/SWISSPROT;Acc:Q0229]

TCCCTAACTCCATAAATATTAACTACCCTCTTCTCCTTTTCCATTGATTTTTTGTTCTTTTCTATAAATTTCATTCAGAA

ACTCAATTTCCTTATTTTCT

In100 candidate690 (in intron):

LOCUS ENSG00000157168

DESCRIPTION Pro-neuregulin-1, membrane-bound isoform precursor (Pro-NRG1)

[ontains: Neuregulin-1 (Neu differentiation factor) (Heregulin) (HRG) (Breas

cancer cell differentiation factor p45) (Acetylcholine receptor-inducing

ativity) (ARIA) (Sensory and motor neuro [Source:Uniprot/SWISSPROT;Acc:Q0229]

ACACTGTTAACTGTCTGCTTGGCTTTTTGAGGCTTCCAGAGTTCAGAATTGTCTTCTCTTGAATAGGTCAGTGCATTTTT

TTCCTTCAGTTTTTCTCCTT

In100 candidate691 (in intron):

LOCUS ENSG00000157168

DESCRIPTION Pro-neuregulin-1, membrane-bound isoform precursor (Pro-NRG1)

[ontains: Neuregulin-1 (Neu differentiation factor) (Heregulin) (HRG) (Breas

cancer cell differentiation factor p45) (Acetylcholine receptor-inducing

ativity) (ARIA) (Sensory and motor neuro [Source:Uniprot/SWISSPROT;Acc:Q0229]

AAAAAAAAAAGAATGGATATAATTTAATATGTAACCTTCTCTTCCAATCTTTAGCTATGCTGTTGATTTTCTTCCCAGTC

TTCAGCTATGTTGTTTTTGT

In100 candidate692 (in intron):

LOCUS ENSG00000157168

DESCRIPTION Pro-neuregulin-1, membrane-bound isoform precursor (Pro-NRG1)

[ontains: Neuregulin-1 (Neu differentiation factor) (Heregulin) (HRG) (Breas cancer cell differentiation factor p45) (Acetylcholine receptor-inducing ativity) (ARIA) (Sensory and motor neuro [Source:Uniprot/SWISSPROT;Acc:Q0229]

AATAAGATTCTTTATCTATATGGCATAGTGGGTGAAAGGACACACTAACCTTACCTACTGCTCCTTTTATGTCAGAAAAT

CCATTTTCTAATCACTTTTT

In100 candidate693 (in intron):

LOCUS ENSG00000157168

DESCRIPTION Pro-neuregulin-1, membrane-bound isoform precursor (Pro-NRG1) [ontains: Neuregulin-1 (Neu differentiation factor) (Heregulin) (HRG) (Breas cancer cell differentiation factor p45) (Acetylcholine receptor-inducing ativity) (ARIA) (Sensory and motor neuro [Source:Uniprot/SWISSPROT;Acc:Q0229]

ATATGGAAGGTTATTTGTTTTAACAATCCCTATGTTAAGTTTTCTTTTTTCTATGTTTCAATTGACTTATTTTGTCAGAT

TTTAGGTTTTTTTTTTTTTT

In100 candidate694 (in intron):

LOCUS ENSG00000157168

DESCRIPTION Pro-neuregulin-1, membrane-bound isoform precursor (Pro-NRG1) [ontains: Neuregulin-1 (Neu differentiation factor) (Heregulin) (HRG) (Breas cancer cell differentiation factor p45) (Acetylcholine receptor-inducing ativity) (ARIA) (Sensory and motor neuro [Source:Uniprot/SWISSPROT;Acc:Q0229]

ACATCATTATTTTTGACATTGAGGATGTCATTTTGTAAATTAACCTATGACATTTTTACTTTTTGGTTAATCCTTCAGAT

TTACTCTTCATTGCTTTTAA

In100 candidate695 (in intron):

LOCUS ENSG00000177675

DESCRIPTION Scavenger receptor cysteine-rich type 1 protein M160 precursor

CD163 antigen-like 1) (CD163b antigen). [Source:Uniprot/SWISSPROT;Acc:Q9NR1]

TTTTTTAAAGTCTTGGATACATATTAACAAATTATCATCTATAAATTGTGTTTTTTTCTTCTATTCAATTTCAGTTCCCT

ATTATTCCACTATATCATAT

In100 candidate696 (in intron):

LOCUS ENSG00000177675

DESCRIPTION Scavenger receptor cysteine-rich type 1 protein M160 precursor

CD163 antigen-like 1) (CD163b antigen). [Source:Uniprot/SWISSPROT;Acc:Q9NR1]

TTAACAATATTAATTCTTTCCATCTATGAGCATGAAATATACTTCTTTTTTTTTGTCCTCTTCTATTTCTTTCATCAGTG

TTTTATAGTTTTCTTTGTAT

In100 candidate697 (in intron):

LOCUS ENSG00000179869

DESCRIPTION ATP-binding cassette sub-family A member 13.

[Source:Uniprot/SWSSPROT;Acc:Q86UQ4]

TTGTAATCTTTAACTGATTTATTTTTTTTATAATTTTTATCCCCTTCTTCAGAATCCCCACTTACTAATGTGGTTGTCAG

ACTTTTCTTTAATTCTTTTT

In100 candidate698 (in intron):

LOCUS ENSG00000179869

DESCRIPTION ATP-binding cassette sub-family A member 13.

[Source:Uniprot/SWSSPROT;Acc:Q86UQ4]

TCTCATTCTGTGGGTTGTCTTTTTATTTTTAACTCTGTTGATAGTTTCTTTTGCTTTGCAAAAGCCCTTCAGTTTACTTA

GGTCTCACTTGTCAGTTTTT

In100 candidate699 (in intron):

LOCUS ENSG00000133392

DESCRIPTION Myosin-11 (Myosin heavy chain 11) (Myosin heavy chain, smooth

mscle isoform) (SMMHC). [Source:Uniprot/SWISSPROT;Acc:P35749]

GTAACTGTCATATAGGATCTTTTTCACCATCCTTGAAATTCCCCTTAGTGCTTTCATAGTGGATTCCTTGTTTCCCAGTT

TTCATGTCTTCCTCTTTCTT

In100 candidate700 (in intron):

LOCUS ENSG00000042813

DESCRIPTION Zona pellucida-binding protein 1 precursor (Sp38).

[Source:Unipot/SWISSPROT;Acc:Q9BS86]

CACACATTTTTACATGCAAATTTGTATGTAAAAAGTCATTAACAAACCCAAGGTCACTTTAATTTTCTTTGTTTCCAGCT

TTCCAGTTTTGTGTATTTTA

In100 candidate701 (in intron):

LOCUS ENSG00000168671

DESCRIPTION UDP glycosyltransferase 3 family, polypeptide A2

[Source:RefSeqpeptide;Acc:NP_777574]

AGATTCTTCAAGATCACGGTCATAATGTCACCATGCTTAACCACAAAAGAGGTCCTTTTATGCCAGGTATCTTTTCTCAC

TTTTCTTGATTTCTCCTACT

In100 candidate702 (in exon):

LOCUS ENSG00000168671

DESCRIPTION UDP glycosyltransferase 3 family, polypeptide A2

[Source:RefSeqpeptide;Acc:NP_777574]

AGCCTCTCTCACTCCTGCCCCTACTATCTATCATGGAATAACATCCAAGAAAGACACCTTGCATATTCTTTCAGTTTCTG

TTTTGTTCTCCCACATATTC

In100 candidate703 (in intron):

LOCUS ENSG00000151834

DESCRIPTION Gamma-aminobutyric-acid receptor subunit alpha-2 precursor

(GAB(A) receptor subunit alpha-2). [Source:Uniprot/SWISSPROT;Acc:P47869]

TTTCATGACCTTAAATATTAACCTATATGTAGTTTATTCACAAATTTTAATTTCTAGCCCTGATGCTTTTTTTTTCAGCT

TTGCTTATTATTGCTCACAT

In100 candidate704 (in intron):

LOCUS ENSG00000164187

DESCRIPTION LMBR1 domain containing 2 [Source:RefSeq_peptide;Acc:NP_00100758]

CTTTAAATCATTTTGATTTGATTTTTATATATGGGGAGATAACTGGTCTAGTTTCATTCTTCTGCATATATCCAGTTTTC

CAGTTTTCCTGGCACCATTT

In100 candidate705 (in intron):

LOCUS ENSG00000127804

DESCRIPTION methyltransferase 10 domain containing

[Source:RefSeq_peptide;Ac:NP_076991]

AATGGCATGTTTAACTATTACTAGCTTTTCTTGTCTTCTAAATATTTCTTTTCCTTCATCTCCTGAAGAACCTTACAGTA

TCTTTTTTTTTTTTTTTTTT

In100 candidate706 (in intron):

LOCUS ENSG00000164500

DESCRIPTION

ACTTTCCTCAAATGGGGAGTGGAATTCTTTGAACATGCGACTAACCTGGAAATCCATTTCCCGGCTTGTATGTTTCAGTA

TCTTTAATAGCTTTGTTCCC

In100 candidate707 (in intron):

LOCUS ENSG00000007168

DESCRIPTION Platelet-activating factor acetylhydrolase IB subunit alpha (PA

acetylhydrolase 45 kDa subunit) (PAF-AH 45 kDa subunit) (PAF-AH alpha) (PAAH

alpha) (Lissencephaly-1 protein) (LIS-1). [Source:Uniprot/SWISSPROT;Acc:43034]

TAGTCTTGGGGATTAACATTAGGCTCCTTGCTTCTTATGCAGATTTCTGCAGCCAGCTTGAATTTCTCCCCAGAAAATGG

GTTTTTCCTTTCTTTTTTTT

In100 candidate708 (in intron):

LOCUS ENSG00000133393

DESCRIPTION LisH domain-containing protein C16orf63.

[Source:Uniprot/SWISSPOT;Acc:Q96NB1]

CCTTTCTCCCTCTTCTATTGGAGCATCTTAACTTCTATTGGAGCATCTTTCCTTTCTGTTGGCCCTGTTCAGATAGTAAC

TCCTTTATGGCATTTTCCTC

In100 candidate709 (in intron):

LOCUS ENSG00000103222

DESCRIPTION Multidrug resistance-associated protein 1 (ATP-binding

cassettesub- family C member 1) (Leukotriene C(4) transporter) (LTC4

transporter). Source:Uniprot/SWISSPROT;Acc:P33527]

AGCTGTTTTTGACTTGAGTAGGTCATCCTCCCTTAACATTCCTTGTTCCTGGTCTTTTTTTTTTTTCCTTTGAGGCAGAG

TTTCGCTCTTTTTTCCCAGG

In100 candidate710 (in intron):

LOCUS ENSG00000169118

DESCRIPTION Casein kinase I isoform gamma-1 (EC 2.7.11.1) (CKI-gamma 1).

[Surce:Uniprot/SWISSPROT;Acc:Q9HCP0]

ATCCTATTGCTGGATTGTTCTCTTTAACATTGTATAATTACCTTCTTTGTTGCTTTTTACAGCTTTGACTTAAGATCTTT

TGATATATCTACTCCTCCTT

In100 candidate711 (in intron):

LOCUS ENSG00000172728

DESCRIPTION fucosyltransferase 10 [Source:RefSeq_peptide;Acc:NP_116053]

CCTAACATATTACATAATGTATCAACTTTATTTTTCCCCCAGAAAACATTGTTCTGCAGTCCCCATCCTCCTTCTTTTCT

TTTTATCGATACATAATATT

In100 candidate712 (in intron):

LOCUS ENSG00000170516

DESCRIPTION Cytochrome c oxidase polypeptide VIIb2, mitochondrial precursor(EC 1.9.3.1). [Source:Uniprot/SWISSPROT;Acc:Q8TF08]

AGCTCTACTGCTCTCTAAGCCTTTGTTCCCCGGTAACTTGCATTACCTCTTTCTTTATGCCTCTGTCACAGTCTGTCACT

GCTTCATGTTCTTGGTATTT

In100 candidate713 (in intron):

LOCUS ENSG00000111704

DESCRIPTION Homeobox protein NANOG (Homeobox transcription factor Nanog) (hanog). [Source:Uniprot/SWISSPROT;Acc:Q9H9S0]

AGGGTAGGAGAAACCCTAACTCACACTGGTTCTCATTAAAATAAAAACTTTTTTTTTCCTTTTTTTCTTTCTGCCAGCAA

CTCCAGCTTGTTTTGTTTTT

In100 candidate714 (in intron):

LOCUS ENSG00000171970

DESCRIPTION Zinc finger protein 57. [Source:Uniprot/SWISSPROT;Acc:Q68EA5]

AGGGTAACCACTGTCCTGATTTTTTTTTTCATTCTCTCTACCTTTACCATTTATTATTTATCACCATACAGAGTTTTAAT

TTTGCCTCCTTTGAATTTTT

In100 candidate715 (in intron):

LOCUS ENSG00000173262

DESCRIPTION Solute carrier family 2, facilitated glucose transporter member14 (Glucose transporter type 14). [Source:Uniprot/SWISSPROT;Acc:Q8TDB8]

AGCAAATCCTTTCTGTTGTAAATTAACCTGTCATCTGTTTTCCCTTCCTTGTGATTCAGATGAACTATTCTTCCTTTCTT

TGTTTCTAGGTCTCATGATT

In100 candidate716 (in intron):

LOCUS ENSG00000185101

DESCRIPTION tumor protein p53 inducible protein 5 [Source:RefSeq_peptide;Ac:NP_001012302]

ATTTCTTTGTTTTACGTTTAACAATTAAAGTCTTAATTTCTAAGAGTGTTTTGTTTCCTCTTTATTTTTATTCACAGCA

TCCCTTCCGTGTTTTTATGT

In100 candidate717 (in intron):

LOCUS ENSG00000176654

DESCRIPTION Homeobox protein NANOG2. [Source:Uniprot/SWISSPROT;Acc:Q8N7R0]

EXON

TAGGAGAATTCCTAACTTACACTGGTTCTCATTAAAATAAAAACTACCTCTTCTTTTTCCTTTTTTTCTTTCTGCCAGCA

GCCTCCAGCTTCTTTTGTTT

In100 candidate718 (in intron):

LOCUS ENSG00000079215

DESCRIPTION Excitatory amino acid transporter 1 (Sodium-dependent glutamateaspartate transporter 1) (Glial glutamate transporter) (GLAST-1). [Source:Uiprot/SWISSPROT;Acc:P43003]

ATGGACAAGTTTAGATAACAAGGGCTGGTATTTTTCACTAACACTTGGTTTCCTCTGTGTGTTTTGCTTTTTCAGATTTA

TCTTTTCTGTGTTGAAATCC

In100 candidate719 (in intron):

LOCUS ENSG00000059804

DESCRIPTION Solute carrier family 2, facilitated glucose transporter member3

(Glucose transporter type 3, brain). [Source:Uniprot/SWISSPROT;Acc:P11169

TTAACTTCTTCCTTCCCCAATTTTCTTTCATTTTTACCAAGTTAACTTCACATTCTTATCTCCAGATAAGTTCTTTAGTA

TAATCTATTCCTTTGTCTTC

In100 candidate720 (in intron):

LOCUS ENSG00000163288

DESCRIPTION Gamma-aminobutyric-acid receptor subunit beta-1 precursor (GABAA)

receptor subunit beta-1). [Source:Uniprot/SWISSPROT;Acc:P18505]

TCATTCACTCTCTAACAATAAACTCTGCCCTTATGGCTACTTTCTGCATTTTTCTCCATTTTCTTTCTTTCTCTACAGGC

TAGCTTCCTCTTTTGATTCA

In100 candidate721 (in intron):

LOCUS ENSG00000163288

DESCRIPTION Gamma-aminobutyric-acid receptor subunit beta-1 precursor (GABAA)

receptor subunit beta-1). [Source:Uniprot/SWISSPROT;Acc:P18505]

GTAACCCCTGATTATATAATGGCCTTCTTTGTATTTTTTTTACAGTTTTTTGTCTTAAATAAATCTATTTTGCTTATATA

ATTATAGTTTCCCTCCTCCC

In100 candidate722 (in intron):

LOCUS ENSG00000180357

DESCRIPTION zinc finger protein 609 [Source:RefSeq_peptide;Acc:NP_055857]

GGAAAGAAAGAAGATGGGAAAGAGACCTGAGCTCACAATATCATGTGTAACAAAATCACTTTTTCCTATTTGCCTCAGAA

TTACTCTTTTTCCTAATTTG

In100 candidate723 (in intron):

LOCUS ENSG00000180357

DESCRIPTION zinc finger protein 609 [Source:RefSeq_peptide;Acc:NP_055857]

CTTTTGGCTTCTACTATTTACTTAACTGAAACTTAACGAGTCCATTTTTTCCTTTCTCTTCCTTCCCTTTCTCTTCAGTG

GATCTGTTTCTTCCCTTTAA

In100 candidate724 (in intron):

LOCUS ENSG00000180357

DESCRIPTION zinc finger protein 609 [Source:RefSeq_peptide;Acc:NP_055857]

AGTGACTTGTTATCTAACCTATTACTTTGTTTTGTTTTATTTTTATAACAGCACTTACTACTTTCTGATATTGTCATCTT

GTTTGTCAGTTTTTCTCTTC

In100 candidate725 (in intron):

LOCUS ENSG00000180357

DESCRIPTION zinc finger protein 609 [Source:RefSeq_peptide;Acc:NP_055857]

ACTTCTTGCTAAGTTTATTTAACACTAGCCCATTTTCTTCTTTAAACTTCATTTCCCCTTGACTTCTTATGACAGTGTTC

AGTTTTGGTTCTTAATTCTT

In100 candidate726 (in intron):

LOCUS ENSG00000180357

DESCRIPTION zinc finger protein 609 [Source:RefSeq_peptide;Acc:NP_055857]

CTTGAAACTCTCCTCTCTTGCCTTGGTCATAAATCTCATTTCTCTTTATAACTGGTTATTCTCTCTATTTTGTGCCAGTC

TCTCTTCCTTCTTGTATTCA

In100 candidate727 (in intron):

LOCUS ENSG00000156687

DESCRIPTION Netrin receptor UNC5D precursor (Unc-5 homolog D) (Unc-5 homolo

4). [Source:Uniprot/SWISSPROT;Acc:Q6UXZ4]

AGCATTAAACCAAGGAGGGTGATCCTTAACATTGAATTGTGTTTTCTCTGCTGTGTTTCAGACTTGTTTTATATGATCAT

ATTCTGCTTTCCTGTTTGGC

In100 candidate728 (in intron):

LOCUS ENSG00000164190

DESCRIPTION Nipped-B-like protein (Delangin) (SCC2 homolog).

[Source:Unipro/SWISSPROT;Acc:Q6KC79]

AGTTGATTTAGAAAACAAATACTAATATTTTTATTATGCTAACTTAGTTAAAATTTTCCTTTTATCTTCAGATTTACATT

TTTTTTTTAAATTTACCTTT

In100 candidate729 (in intron):

LOCUS ENSG00000205813

DESCRIPTION Olfactory receptor 3A2 (Olfactory receptor 17-228) (OR17-228).

Source:Uniprot/SWISSPROT;Acc:P47893]

AGCTCATAACTGTATTTATTTGTTATTTGTATTTCTTCTTTGCAGATTTCTTTTTCTTTTTTTGTCTTTTTTTTTTTTTT

TTTTTGCCCTTTTGGTGATA

In100 candidate730 (in intron):

LOCUS ENSG00000145244

DESCRIPTION Atrial natriuteric peptide-converting enzyme (EC 3.4.21.-)

(proANP- converting enzyme) (Corin) (Heart-specific serine proteinase ATC2)

(Trnsmembrane protease, serine 10). [Source:Uniprot/SWISSPROT;Acc:Q9Y5Q5]

TATCACTACATCCTTCTTGAAATACTTTCATTTTCTTCTAAAATAACACAAAAACTCCTTGTTTTCTTCATATGTCAGAG

ACTAACTCTTTTTCTCTTTT

In100 candidate731 (in intron):

LOCUS ENSG00000146648

DESCRIPTION Epidermal growth factor receptor precursor (EC 2.7.10.1) (Recepor

tyrosine-protein kinase ErbB-1). [Source:Uniprot/SWISSPROT;Acc:P00533]

AAACAAATATAAAGAAAACTTAACTAAAAATACTGCATATCTCTTTCAGATTATATATCCCAGAAAGGATATATTTTTCT

CCTTTCTGGTCTTCCTTTTT

In100 candidate732 (in intron):

LOCUS ENSG00000113569

DESCRIPTION Nuclear pore complex protein Nup155 (Nucleoporin Nup155) (155 ka

nucleoporin). [Source:Uniprot/SWISSPROT;Acc:O75694]

AGTTTATGCATTATTACTTTAACTCTGGATTCTGTTCTGTAACTTCTTGTCTACTTTCTACACTTTGTTCTACCTCAGTT

ACTGTCTCATCTATCTTTTT

In100 candidate733 (in intron):

LOCUS ENSG00000113569

DESCRIPTION Nuclear pore complex protein Nup155 (Nucleoporin Nup155) (155 ka nucleoporin). [Source:Uniprot/SWISSPROT;Acc:O75694]

GCTTCTTGCCTTGGAAAAACTAACTTATCTTTGCTTAACTCTCTAAACCTCTTTCTCTCTCTGTAATTTTCTGAGCAGTT
CCTTCCTCTTCATTTATTCT

In100 candidate734 (in intron):

LOCUS ENSG00000108381

DESCRIPTION Aspartoacylase (EC 3.5.1.15) (Aminoacylase-2) (ACY-2). [Source:niprot/SWISSPROT;Acc:P45381]

AGGAAATTTAAAGAAAAAAGACCACAAGAGTTTCCTATTTCTAACACCACTGATTTTTTCCATGTTCTTTCCCAGTCTTT
ATTCACTATGCATCTAACTT

In100 candidate735 (in intron):

LOCUS ENSG00000132932

DESCRIPTION Probable phospholipid-transporting ATPase IB (EC 3.6.3.1) (ATPae class I type 8A member 2) (ML-1). [Source:Uniprot/SWISSPROT;Acc:Q9NTI2]

TTGAGAGTGGACCTAACTCTCATGTAAGTTCTCTGTCTATATCAGTGGTCTCCAAAACTTTTTCATTGGATGTCCCATTT
GTAACACTTTTTTTTTTCTT

In100 candidate736 (in intron):

LOCUS ENSG00000167723

DESCRIPTION Transient receptor potential cation channel subfamily V member (TrpV3) (Vanilloid receptor-like 3) (VRL-3). [Source:Uniprot/SWISSPROT;AccQ8NET8]

GGTGAAAAGGGTTAACTCTTTAGGTTCTAATTTTCATTTTCCTGACCACCAGTGATTCCAAACATCTTTCACTTTGTTTA
TTCACTGTGTGTATTTTCTC

In100 candidate737 (in intron):

LOCUS ENSG00000082068

DESCRIPTION WD repeat domain 70 [Source:RefSeq_peptide;Acc:NP_060504]

TATTTCTCCTTCATTATAACTGCTGAGTGTACTAGTCTTGGGTGGCTTTTTTTTTTTTTTTTTCCTTTCAGCACTTTGA
CAATATCATTTCATTCTCTT

In100 candidate738 (in intron):

LOCUS ENSG00000082068

DESCRIPTION WD repeat domain 70 [Source:RefSeq_peptide;Acc:NP_060504]

AAGCTGGAAGTTATGTAAGCTAACCTGTGGGTTTTTTCTCCTGTAATTTTTTTCTTCTGCTTTGAAAGTTCAGGTTTCC
CGTCTTGCCTTAAAGGCTTT

In100 candidate739 (in intron):

LOCUS ENSG00000066455

DESCRIPTION Golgin subfamily A member 5 (Golgin-84) (RET-fused gene 5 proten) (Ret-II protein). [Source:Uniprot/SWISSPROT;Acc:Q8TBA6]

GCACTTGATTTTTAACTAGTTTTTTTTTTTGTTTGTTTGTTTGTTTTGGCTACAGATCTTATTGATATTTTAGCAATCAA
TCAATTTTTTTTTGTTTTTT

In100 candidate740 (in intron):

LOCUS ENSG00000176826

DESCRIPTION FK506 binding protein 9-like [Source:RefSeq_peptide;Acc:NP_87827]

CTGTCTGCCTAACCTCCCTGTCTCCCCTACCCCTTAGAGTTAACCATCTTTATTGGTTTCTATTTTTAATTTTTCCAGTG

TCTGGATTTTTTTTTTTTTT

In100 candidate741 (in intron):

LOCUS ENSG00000166532

DESCRIPTION family with sequence similarity 80, member B (FAM80B), mRNA

[Sorce:RefSeq_dna;Acc:NM_020734]

GGTTTGTAGAAAAGTACTGACTTTCTGGCTTCATGGTAACTAATGCCATGGTGTTATTTTTAATGTTTTCTTTTTCAGTG

TTTTTTTTTTCCCCTCTCAA

In100 candidate742 (in intron):

LOCUS ENSG00000166532

DESCRIPTION family with sequence similarity 80, member B (FAM80B), mRNA

[Sorce:RefSeq_dna;Acc:NM_020734]

CCATTAACCTAGCCTTACCATTAACATGTCTTTTTTTTTTTTTTTTTTTTTTTAATTCACAAGAGCCAGTATGCTACAAA

ATTTTTGTTGCTTTTTCCTT

In100 candidate743 (in intron):

LOCUS ENSG00000109171

DESCRIPTION SLAIN2 protein. [Source:Uniprot/SPTREMBL;Acc:Q8N5R3]

AACCAATAACTTCCCATTCCCTTTGCTTCCTCTCTGCCTGTTTTAGGTTTAGGTTGCCCCAGATCTCTCTTTTGCCTCAT

GTCTCTCCCTGTTTTGGTGT

In100 candidate744 (in intron):

LOCUS ENSG00000113594

DESCRIPTION Leukemia inhibitory factor receptor precursor (LIF receptor) (LFR)

(CD118 antigen). [Source:Uniprot/SWISSPROT;Acc:P42702]

TTTTAAACTATGGTAGGTAACATTTCTTTGCCCATCTTTTCCTTCACTTTTTTCATATAGTTCAGGTCATACTGGATATT

CAGTTTTGTTCCTTATCTTT

In100 candidate745 (in intron):

LOCUS ENSG00000133958

DESCRIPTION KIAA1409 (KIAA1409), mRNA [Source:RefSeq_dna;Acc:NM_020818]

TCATTGTTTGTGCTCTGTGGCTACTGTAACCACTAATTTCATATATTTATCTTTCCCTCTATATTCTTACCTTCTCAGGT

TTGCTTACCCTTTGTGTGTA

In100 candidate746 (in intron):

LOCUS ENSG00000133958

DESCRIPTION KIAA1409 (KIAA1409), mRNA [Source:RefSeq_dna;Acc:NM_020818]

GTATATCGGCTTTGAAGTAACTTAGATGTTTATATCTGACTTTCTTCTTTTCTTTTTTTTTTCCTCCTCTTTCCCAGGG

TCTATGTCTTTGTCTTGACT

In100 candidate747 (in intron):

LOCUS ENSG00000133958

DESCRIPTION KIAA1409 (KIAA1409), mRNA [Source:RefSeq_dna;Acc:NM_020818]

CTGTTAAAAATTTTAACCACTGGGATGGCTCAAATTTTTCCTTTTGACCTCTGAGTGTTCTTTGTAGTTTTCCATCAGTT

ATTAATATTTTTTCTATTTC

In100 candidate748 (in intron):

LOCUS ENSG00000133958

DESCRIPTION KIAA1409 (KIAA1409), mRNA [Source:RefSeq_dna;Acc:NM_020818]

CTTTGTCTAATTAAGATAAACTGAGATGGCAACTAAGATAATTAACTTAAGAACTTCCTTCTTTTCAAACATCAGCATTT

AGTGCTCTAAATTTCCTTTC

In100 candidate749 (in intron):

LOCUS ENSG00000145623

DESCRIPTION Oncostatin-M specific receptor subunit beta precursor.

[Source:niprot/SWISSPROT;Acc:Q99650]

AATTGAGCCCTTTATTACATATAACAACTTTCTTTTTCTCTTTTTCTGGTTCTTAACATCTGTTTTATCTAAATACAGCT

ACTCTCACTTGCTTTTGATT

In100 candidate750 (in intron):

LOCUS ENSG00000138614

DESCRIPTION Uncharacterized protein C15orf44.

[Source:Uniprot/SPTREMBL;Acc:96HX5]

GATGGATGCAAAGTGCTGACTACCACATGTGGCATAAATGCTCATTAACTGTGATGATGACGTTGTTTCATTCAGCTGTC

TTCCTTAGCCTGCTTCTGGT

In100 candidate751 (in intron):

LOCUS ENSG00000103534

DESCRIPTION transmembrane channel-like 5 [Source:RefSeq_peptide;Acc:NP_07906]

AGCCCTAACAACCATCAATGTTTTTATATTCTTATTTCATCTACACTTTCTCTTCCCAGCAAATCCCTTATTTTTATTCT

TAGTATTTATTTATTTTAAT

In100 candidate752 (in intron):

LOCUS ENSG00000132964

DESCRIPTION Cell division protein kinase 8 (EC 2.7.11.22) (EC 2.7.11.23)

(Potein kinase K35). [Source:Uniprot/SWISSPROT;Acc:P49336]

TTTTATCTCCTTTTAGTTTAATTTTTTTCTCTTTACTAACTATAGCTTTAGTTGCTTTCCATACATTTTTATTTGCAGTA

ATTTCATTATCATTTTTTCC

In100 candidate753 (in intron):

LOCUS ENSG00000132964

DESCRIPTION Cell division protein kinase 8 (EC 2.7.11.22) (EC 2.7.11.23)

(Potein kinase K35). [Source:Uniprot/SWISSPROT;Acc:P49336]

ATCAAGCCTAACAAACATTATATTTATTTTACCTAGTATTTCATTTAAATCTTTCATGCTTTCTATTTATCTCTCCAGCT

TTATGTTTTGTCTTCTACTT

In100 candidate754 (in intron):

LOCUS ENSG00000164327

DESCRIPTION rapamycin-insensitive companion of mTOR

[Source:RefSeq_peptide;cc:NP_689969]

ATGTATTGCTTATTGTCAAGAATAAACTCGTGTTAAGCATCTCCAAATAACATTTGTGTCACTTACTGTCTTGCTCAGTA

TTTCTTTTAACCACTGTTTA

In100 candidate755 (in exon):

LOCUS ENSG00000174485

DESCRIPTION C-myc promoter-binding protein (DENN domain-containing protein A). [Source:Uniprot/SWISSPROT;Acc:Q7Z401]

TTCCAAAACAATTCATACTAACAAATGCATCTGTCTTCTTTCTCACTGGCCTTTTTTTGATGCATTCAGGAAGTTTCTGA
CTTTTCTGTATCGTTATTCC

In100 candidate756 (in intron):

LOCUS ENSG00000109182

DESCRIPTION CDNA: FLJ21511 fis, clone COL05748. [Source:Uniprot/SPTREMBL;Ac:Q9H720]

AGTAGCAAAGGGGTTTAACAAATGGCCGTCCTTTTCACTCTTTTATTTCTTGAGACATCTTTTAATAGCCCTCAGCTATT
TTTATCCAATTCAGTCCTGT

In100 candidate757 (in intron):

LOCUS ENSG00000082074

DESCRIPTION FYN-binding protein (FYN-T-binding protein) (FYB-120/130) (p120p130) (SLP-76-associated phosphoprotein) (SLAP-130) (Adhesion and degranulaion promoting adaptor protein) (ADAP). [Source:Uniprot/SWISSPROT;Acc:O15117

TTAACTCCATGCCCTATTTAAGTTTACTTTTTCTCATGTTTTTGCACCCTCATTCCTTTCAACTCAAGTCCACAGTGCCT

CTTAGCCCTCATTTTGTTAT

In100 candidate758 (in intron):

LOCUS ENSG00000077782

DESCRIPTION Basic fibroblast growth factor receptor 1 precursor (EC 2.7.10.)

(FGFR-1) (bFGF-R) (Fms-like tyrosine kinase 2) (c-fgr) (CD331 antigen).

[ource:Uniprot/SWISSPROT;Acc:P11362]

GGACTAGAGTTTGGCTGGTTAACAAGGGGTACTACCTAATTGCTTTTCTCTGATATTCTCAGCCTCTTTTTCTGGAGGAG

TATCTCTGTCAGTTTCTTTT

In100 candidate759 (in intron):

LOCUS ENSG00000113600

DESCRIPTION Complement component C9 precursor [Contains: Complement componet

C9a; Complement component C9b]. [Source:Uniprot/SWISSPROT;Acc:P02748]

TAACTAATTCACTAATTTGTATTCTAATATTTGTTTCTTTTTTTCAAATCTATTTTTAATAACTTCCAGTTCTTTGCTAA

AATTTTCAGACTTGTCTCTT

In100 candidate760 (in intron):

LOCUS ENSG00000109184

DESCRIPTION DCN1-like protein 4 (Defective in cullin neddylation protein 1-

ike protein 4) (DCUN1 domain-containing protein 4).

[Source:Uniprot/SWISSPRT;Acc:Q92564]

TGAGGAGAATGAAGTTAGTTAACACCTTCCTCACCTGCTACTTTCAATTCTTTTGGTTTTTACCCTGCTTTTCAGCTCTG

CAATCTTTTTCCTGGACCTT

In100 candidate761 (in intron):

LOCUS ENSG00000153071

DESCRIPTION Disabled homolog 2 (Differentially expressed protein 2) (DOC-2)

[Source:Uniprot/SWISSPROT;Acc:P98082]

CCTAAAGCTAGAACCTCACCCATAACACATCTACCTCTTTTTCATTTCTTTTTTCCCTGCCTTCTCACCTCACAGATGTA

ACCTTGTTTTTGTTGGTTCC

In100 candidate762 (in intron):

LOCUS ENSG00000119698

DESCRIPTION HEAT-like repeat-containing protein isoform 1

[Source:RefSeq_petide;Acc:NP_478144]

CCTCTGCATTGAATATTATATAAATGTATAATATATAACATGTTATATATATATTTTCTCTCTGCTTTTATGTGTCAGGT

TTGTTTCTTTAGTTCTTTTA

In100 candidate763 (in intron):

LOCUS ENSG00000188993

DESCRIPTION

TGCTTTTAAAGAGATTTAAATATAAGAAAAAATATTTAACCTACATAAATTTCTGGAGCTCTTTGTTCCTTACTCCAGAC

TTCTATCTGGTTTCATTTTT

In100 candidate764 (in intron):

LOCUS ENSG00000163071

DESCRIPTION Spermatogenesis-associated protein 18.

[Source:Uniprot/SWISSPRO;Acc:Q8TC71]

CACCTTTTAAAGAAAGTGAACTGACTGGCCTAGAGGGACTAACACCCTTTTTATTCTTATTCTCCATTAATTCAGCTAAC

TTACAGTAGTTTTTCTTTCT

In100 candidate765 (in intron):

LOCUS ENSG00000165682

DESCRIPTION C-type lectin domain family 1 member B (C-type lectin-like

recetor 2) (CLEC-2). [Source:Uniprot/SWISSPROT;Acc:Q9P126]

AGTAAAGCTTTATTGGAAAAGTGTCATTTGTACTATAACCTCCACACATTTTCTTTTCTCTCTGTTACTTTGTCACAGTT

ATTTATTCGATTATTTTTTA

In100 candidate766 (in intron):

LOCUS ENSG00000168615

DESCRIPTION ADAM 9 precursor (EC 3.4.24.-) (A disintegrin and

metalloproteiase domain 9) (Metalloprotease/disintegrin/cysteine-rich protein 9)

(Myelom cell metalloproteinase) (Meltrin gamma) (Cellular disintegrin-related

protin). [Source:Uniprot/SWISSPROT;Acc:Q13443]

CAACTTTATCTTCTAACTCTTTTATTGCGGTTTTCATTTCAGGCACTATGATTTTAGTTTTCAGAAACTCTTTTATATTC

TCTGAGTTTTTTTCCCACTT

In100 candidate767 (in intron):

LOCUS ENSG00000184178

DESCRIPTION Sec1 family domain-containing protein 2 (Syntaxin-binding proten

1- like 1). [Source:Uniprot/SWISSPROT;Acc:Q8WU76]

TTTCTCTTGTTTTGTAACCAAGATTTTATATCTTTCTTGAAGGCTTTTTTTTTTTTTTTTGCCATTTTCCTTCCCAGAT

TGAGTTTTAACTTCTTCCCA

In100 candidate768 (in intron):

LOCUS ENSG00000184178

DESCRIPTION Sec1 family domain-containing protein 2 (Syntaxin-binding proten

1- like 1). [Source:Uniprot/SWISSPROT;Acc:Q8WU76]

AAAGCTTTGCTGGGTGTAGTATTCTTCACTAACGGTTTTGTTTTGTTTTTTTCATATTTTTCCTTTCAGCACTTTGAATA

TATCATCCCATTATCTCCTT

In100 candidate769 (in intron):

LOCUS ENSG00000184178

DESCRIPTION Sec1 family domain-containing protein 2 (Syntaxin-binding proten

1- like 1). [Source:Uniprot/SWISSPROT;Acc:Q8WU76]

TGTGGTTTGTTGTTGCTTTTACTGTTTGTTTGCTTTAACCTCTGTTCTATTCTCTTTTTATCTTAGGAATTCCAGTTCTC

ATTGTAGGCATCATCTTTCT

In100 candidate770 (in intron):

LOCUS ENSG00000197140

DESCRIPTION ADAM 32 precursor (A disintegrin and metalloproteinase domain 3).

[Source:Uniprot/SWISSPROT;Acc:Q8TC27]

GTTTCTTAACTGGGTTCTTCATGTATTACATCTTTCTACCTCTCTATCTGACATTGTTTCACTTCCTTATATCAGTTTCC

AAATCCTCCGTTTTCTTAAT

In100 candidate771 (in intron):

LOCUS ENSG00000132357

DESCRIPTION Caspase recruitment domain-containing protein 6.

[Source:Unipro/SWISSPROT;Acc:Q9BX69]

CAACTTTTGCCTTTAACATGTTATGGATGCCATTCCTCTGTCTTTTAGCTTCCATTGTTTCTGATGCTAAATCTTCAGTC

ATTTTATCATTGATCCTCTG

In100 candidate772 (in intron):

LOCUS ENSG00000103599

DESCRIPTION IQ motif containing H isoform 1

[Source:RefSeq_peptide;Acc:NP_01026885]

TTGTTGCTTATAGACTTAACTAATTTCAAAAATAATCTTTATGGTGCTTTTCACTTGTGTCACTTCCCTCAGTCATTTAA

ATTTTTTAGAATCTTTTTTT

In100 candidate773 (in intron):

LOCUS ENSG00000074755

DESCRIPTION zinc finger, ZZ type with EF hand domain 1

[Source:RefSeq_peptie;Acc:NP_055928]

CTTTTTTTTTCTTATACATCTTTTTATTTTCTTTGGAGTTAACAATTGAATACATACTTTAATTTTCCTAATTTTCAGTC

TTTTATCATTAATTTAGTCC

In100 candidate774 (in intron):

LOCUS ENSG00000197475

DESCRIPTION

TAGATGCCTTGAGACTTCCTACATAGACACGTCTTTAACAAATATAGACAATTTTCCTTCTTCATTTTCAACCAGTATGT

AAGTCTGCTTTTCATTTCTT

In100 candidate775 (in intron):

LOCUS ENSG00000169902

DESCRIPTION Protein-tyrosine sulfotransferase 1 (EC 2.8.2.20) (Tyrosylproten sulfotransferase-1) (TPST-1). [Source:Uniprot/SWISSPROT;Acc:O60507]

ATTGATTCTTACCTGTGTTATATTCATTCTGCTGTTGAGCTAACCCACCGAGTTATTATTTGGTTGTTTTTCAGTTCTA
TAATTTTTATTGGGTTTTTT

In100 candidate776 (in intron):

LOCUS ENSG00000169902

DESCRIPTION Protein-tyrosine sulfotransferase 1 (EC 2.8.2.20) (Tyrosylproten sulfotransferase-1) (TPST-1). [Source:Uniprot/SWISSPROT;Acc:O60507]

ATAGCTAACTGTGGCTTTTCTCATTTATTTGTTTTGTTTTGTATATTTATATGATTTCCTGTATTTTATCAGTTCTATAA
AATTCTCGACCATTTTTCTT

In100 candidate777 (in intron):

LOCUS ENSG00000104755

DESCRIPTION ADAM 2 precursor (A disintegrin and metalloproteinase domain 2)(Fertilin subunit beta) (PH-30) (PH30). [Source:Uniprot/SWISSPROT;Acc:Q9996]

ATTAACAAGTTGCATTGCCTTAGCTTTTTACTTTTAAGGAATTTTATTTATTTTTATTATTTTTTGTTTTTTCTTCAGTT
TTTCTGTTTTAGTTTTTTTA

In100 candidate778 (in intron):

LOCUS ENSG00000185722

DESCRIPTION Ankyrin repeat and FYVE domain-containing protein 1 (Ankyrin reeats hooked to a zinc finger motif). [Source:Uniprot/SWISSPROT;Acc:Q9P2R3]

EXON

GTGAACTACTTCAAAAGGTTCCAATTAAAAGTTTGCTTTTAACTTTTCCTCCTAGATTTTGCTCTCTCACAGCCTAATTT

CACAGTTTCTAGTGTTCCCT

In100 candidate779 (in intron):

LOCUS ENSG00000182836

DESCRIPTION phosphatidylinositol-specific phospholipase C, X domain containng 3 [Source:RefSeq_peptide;Acc:NP_001005473]

TTCTGAAGGCATGTTTCTGCACTTCACTTTATATGGCTTACACCTAACTTTATACTTCCCATTTTCTCATTCCATCAGAG

TTTATGTACTCCCTTTCTCT

In100 candidate780 (in intron):

LOCUS ENSG00000154710

DESCRIPTION BTB/POZ domain-containing protein KCTD7.

[Source:Uniprot/SWISSPOT;Acc:Q96MP8]

TGGTAATATGGTCCATGTTCTTAAGTTTACTTCGTCTAACATTGGTACAACCACTTAAGCTCTTTTTCATTGTTTCAGAG

TATGCTTTTCCTTTCATTAG

In100 candidate781 (in intron):

LOCUS ENSG00000083720

DESCRIPTION Succinyl-CoA:3-ketoacid-coenzyme A transferase 1,

mitochondrialprecursor (EC 2.8.3.5) (Somatic-type succinyl CoA:3-oxoacid

CoAtransferas)

(Scot-S). [Source:Uniprot/SWISSPROT;Acc:P55809]

AGAGCACCTCTGCATTTAACTTGTAACTTCTCTTATTGCTTATTGTCTCCCCCTTTTATTTTTCTAGACTTCCACAGCTG

CTTTTTTTTTTTTTCCCTCT

In100 candidate782 (in intron):

LOCUS ENSG00000083720

DESCRIPTION Succinyl-CoA:3-ketoacid-coenzyme A transferase 1,

mitochondrialprecursor (EC 2.8.3.5) (Somatic-type succinyl CoA:3-oxoacid

CoAtransferas)

(Scot-S). [Source:Uniprot/SWISSPROT;Acc:P55809]

CTTAGATCACTGATTTGAAACCTTTTTTCTCTAATATAAGCAAGTAACATCCTACAAATTTTTATGTTGTTTCAGTCTCT

TCAAAATACTTTCTAATCTT

In100 candidate783 (in intron):

LOCUS ENSG00000144560

DESCRIPTION Transcription cofactor vestigial-like protein 4 (Vgl-4).

[Sourc:Uniprot/SWISSPROT;Acc:Q14135]

CACTTTAGAAAAGTTAAGTCTAAAAATAATGCTCAATATAACATGGCCTTTGGATCGTTTTTCTGCCAGCTCTTCTGTAG

TGTTCATTAAGTACTTCCTT

In100 candidate784 (in intron):

LOCUS ENSG00000165061

DESCRIPTION zinc finger, matrin type 4 [Source:RefSeq_peptide;Acc:NP_078921

TTATTTTGGCCTCTTCACCTTCCAAATCCTTCCTTCACTGTCCACCTAACTGTTCATGTCTTTTGTTTCCTTCCAGCTCT

TCCCTGATGCTGGTCTTCTT

In100 candidate785 (in intron):

LOCUS ENSG00000165061

DESCRIPTION zinc finger, matrin type 4 [Source:RefSeq_peptide;Acc:NP_078921

AGCTCCTGGATTCATTAACTTTTTGAAGGGTTTTTTGTGTCTCTATTTCCTTCAGTTCTGCTCTGATTTTAGTTATTTCT

TGCCTTCTTCTAGCTTTTGA

In100 candidate786 (in intron):

LOCUS ENSG00000165061

DESCRIPTION zinc finger, matrin type 4 [Source:RefSeq_peptide;Acc:NP_078921

TCACCCTACTTAAAATTGCAAATTCCTGCTTCCGCATCCTTCATAACACTTCTCCTGCTTTGTTTTTCTTCACAGCCCTT

ATCATTTTCCAATGTCCTAT

In100 candidate787 (in intron):

LOCUS ENSG00000033800

DESCRIPTION E3 SUMO-protein ligase PIAS1 (Protein inhibitor of activated STT protein 1) (Gu-binding protein) (GBP) (RNA helicase II-binding protein) (EAD/H box-binding protein 1). [Source:Uniprot/SWISSPROT;Acc:O75925]

AGCTTCACTGCTAGTTGAGGATGCGTTTTTCTTTGGTAACTTTGTTACCACTCATATATTTGCTTTCCAGTTTCTACACT

TTTTGTTGCCCTCCTATTTC

In100 candidate788 (in exon):

LOCUS ENSG00000106609

DESCRIPTION CDNA FLJ10099 fis, clone HEMBA1002462 (C7orf42 protein)

(Chromoome 7 open reading frame 42). [Source:Uniprot/SPTREMBL;Acc:Q9NWD8]

GTGTTTGTGGCTTAATAATCATAGTAACAACAATAATACCTTTTTCTCCATTTTGCTTGCAGGAAACATACCTTAAGTTT

TTTTTGTTTTGTTTTTGTTT

In100 candidate789 (in intron):

LOCUS ENSG00000157152

DESCRIPTION Synapsin-2 (Synapsin II).

[Source:Uniprot/SWISSPROT;Acc:Q92777]EXON

TTTAACAAACTGTCAATTGGCTACATCCTTAGTATTCTCTCCTGAATACACTTTTTTATTCTTTGCCTTTCCAGGCTGAG

AGTTTTCCACATCTTTCCTT

In100 candidate790 (in intron):

LOCUS ENSG00000158486

DESCRIPTION dynein, axonemal, heavy polypeptide 3

[Source:RefSeq_peptide;Ac:NP_060009]

ATCTGTGGGTTAATAATCCATCATGAATCAATTGCCATTAACTTGCCAAGAACTTTTTTTCAATTTCAGTTTGAAGTTTC

ACTCTTCTGTGTGCTTATTT

In100 candidate791 (in intron):

LOCUS ENSG00000158486

DESCRIPTION dynein, axonemal, heavy polypeptide 3

[Source:RefSeq_peptide;Ac:NP_060009]

TAACAATATTGAGTCTTCCTATCCATGATTATGGAATATCTCTCCATTATTTATATCTTCTTTGATTTCTTTCATCAGAG

TTTTTTGTAGTTTTCCTCAT

In100 candidate792 (in intron):

LOCUS ENSG00000167670

DESCRIPTION Chromatin assembly factor 1 subunit A (CAF-1 subunit A) (Chromain

assembly factor I p150 subunit) (CAF-I 150 kDa subunit) (CAF-Ip150).

[Sorce:Uniprot/SWISSPROT;Acc:Q13111]

CTTTTTTTTTCTTTGCCTGATCTGTAAGTTTTTTTAACATAGTCTAGTATTTCATTCTCCGTGATATTTCGTCTCAGCC

TGTTACCTCTTTGTTTACAG

In100 candidate793 (in intron):

LOCUS ENSG00000198874

DESCRIPTION radical S-adenosyl methionine and flavodoxin domains 1

[Source:efSeq_peptide;Acc:NP_060734]

AGCTGTAACAATCAATTTTATATTTGCCCATATATTTACCTTTACTATGATCTTTACTTCTTCATGCAGCTTCAAATTAC

TGTCTAGTGCCCTTTCATTT

In100 candidate794 (in intron):

LOCUS ENSG00000132170

DESCRIPTION Peroxisome proliferator-activated receptor gamma (PPAR-gamma).

Source:Uniprot/SWISSPROT;Acc:P37231]

AAATTGGCACAAGTATCAAGGAAAGCTGGTACAACTTAATCTTAACCTGGAATCAACTCCTTTAATTTTTACATACAGAT

TTGGTTCCGTTTTTTAGATT

In100 candidate795 (in intron):

LOCUS ENSG00000158321

DESCRIPTION Autism susceptibility gene 2 protein.

[Source:Uniprot/SWISSPROTAcc:Q8WXX7]

CTCATTGTGGTTTTAAGTTGTATTTCCCTAATGACTACTGATGTTAACTATTGTTTTATGTATTCATTCACTTTCCAGAC

AGATATCTTTTTTTTTTTT

In100 candidate796 (in intron):

LOCUS ENSG00000158321

DESCRIPTION Autism susceptibility gene 2 protein.

[Source:Uniprot/SWISSPROTAcc:Q8WXX7]

TAACACCAAGCTGCGGATCCTCATCCTTGAATCTTTTTCTTCTAGACGTGATCTGACTTTTCTATTCTGTGTCCTCAGAA

TTATTCTTTGTATTTCTTTC

In100 candidate797 (in intron):

LOCUS ENSG00000158321

DESCRIPTION Autism susceptibility gene 2 protein.

[Source:Uniprot/SWISSPROTAcc:Q8WXX7]

TGCCATAGCTGAAGACCATCTAGTACCCTCTTTAACTTTTGCCACTTTGGAGTTTCCTCTCTACCGTTACTTATTCAGCA

GGTTTTTGTTTTTGTTTTTT

In100 candidate798 (in intron):

LOCUS ENSG00000066739

DESCRIPTION Uncharacterized protein C14orf103.

[Source:Uniprot/SWISSPROT;Ac:Q96BY7]

TTAAACGTTCTTTTACTAATAGTTATAACCTTTTACCCAAATTACCCAATTTTTCTCAGGTGACATGTTTCATCTACCCA

TTTATTATTGCATTTCATTT

In100 candidate799 (in intron):

LOCUS ENSG00000066739

DESCRIPTION Uncharacterized protein C14orf103.

[Source:Uniprot/SWISSPROT;Ac:Q96BY7]

TCCCCATCTAATTGCACCCCCCTTATTTGCACATAGTAACCTGGTTCCAAATTCGTCTCTCTTTCAATTCTCTGGCAGGA

ATTTCCTTTTCTTCTAGCTT

In100 candidate800 (in intron):

LOCUS ENSG00000078902

DESCRIPTION Toll-interacting protein.

[Source:Uniprot/SWISSPROT;Acc:Q9H0E2]EXON

TGCTGCACCCATTGACTCGTCATTTAACATTAGGTATTTCTCCTAAACAATTTCCTATTTTTTTCCTTGGGTCAGAATCC

CTTATGAATGTTATTTTTTC

In100 candidate801 (in intron):

LOCUS ENSG00000183166

DESCRIPTION Calneuron-1 (Calcium-binding protein CaBP8).

[Source:Uniprot/SWSSPROT;Acc:Q9BXU9]

AATTCTGTAAATATATCATCTTTGAGATCTCTAACTTTATATATTTTTTTATCTCTTTTTTCGTAACCTGACTTTCAGAT

GATTTTCCTTATACATCTAT

In100 candidate802 (in intron):

LOCUS ENSG00000183166

DESCRIPTION Calneuron-1 (Calcium-binding protein CaBP8).

[Source:Uniprot/SWSSPROT;Acc:Q9BXU9]

TACAAAGTAATGAGACTTAACCACATTTTATGCTTCGTCATTTGCCATTTTATCAGCATCATCCTTGATCCATTTTCCTC

CTCAGTACGTTTCTATTGAT

In100 candidate803 (in intron):

LOCUS ENSG00000188463

DESCRIPTION MGC87315 protein (Fragment). [Source:Uniprot/SPTREMBL;Acc:Q3KQX]

TTCCATTATTTCTTTCTTAACCTGTATGTAATTTTTATCACACATTTTACTTTTACTTTTGTTTTTTGGTTGAGTCAGGT

CTTGCTCTTTTGCCTAGGCT

In100 candidate804 (in intron):

LOCUS ENSG00000188463

DESCRIPTION MGC87315 protein (Fragment). [Source:Uniprot/SPTREMBL;Acc:Q3KQX]

AGCTATAACAATCAATTTTATATTTGCCCATATATTTACCTTTACTATGATCTTTACTTCTTCATGCAGCTTCAAATTAC

TGTCTAGTGCCCTTTCATTT

In100 candidate805 (in intron):

LOCUS ENSG00000060140

DESCRIPTION Tyrosine protein-kinase STYK1 (EC 2.7.10.2)

(Serine/threonine/trosine kinase 1) (Novel oncogene with kinase domain) (Protein

PK-unique). [ource:Uniprot/SWISSPROT;Acc:Q6J9G0]

AACCATCATCACTGTTGCTGTAACATTCTAATATATTTTTCTTTGGGCTTCCCCTACACAGGTGTTTTTCCCAGTCAATT

TCATTGTACTCTTGCCATAT

In100 candidate806 (in intron):

LOCUS ENSG00000196313

DESCRIPTION nuclear pore membrane protein 121

[Source:RefSeq_peptide;Acc:NP742017]

AGAAGTCTGACATAAAAATTAACTTGAGACTTTTATGTTCATTCTTTTTATCTTCCAGTTTCTTTCCTTCCTTGCTTTAA

AAATACCTTGGACTTATTTT

In100 candidate807 (in intron):

LOCUS ENSG00000132155

DESCRIPTION RAF proto-oncogene serine/threonine-protein kinase (EC 2.7.11.1

(Raf- 1) (C-RAF) (cRaf). [Source:Uniprot/SWISSPROT;Acc:P04049]

GTTGAAGCTGATAGTGATTGAACATATTTAGTTTAACTTAGTTAATGACTTTTAAATTTTTTTTATTTTTTCAGAACAA

TGCAAACTTTTTTTTTTTT

In100 candidate808 (in intron):

LOCUS ENSG00000100749

DESCRIPTION Serine/threonine-protein kinase VRK1 (EC 2.7.11.1) (Vacciniareated

kinase 1). [Source:Uniprot/SWISSPROT;Acc:Q99986]

GTTAATCATGAGTCTAACGTATTTTGTGTTTTTTGTTTGTTTGTTATCCATGTCCTTTCTCTTGACTTTATGTTTCAGAG

TATATGCTCAATCTTTTTTT

In100 candidate809 (in intron):

LOCUS ENSG00000197006

DESCRIPTION methyltransferase like 9 isoform 1

[Source:RefSeq_peptide;Acc:N_057109]

CATGTCTGCCTTAACACTTTCAATTCTGCCTTTCACTTTCACTTCACTTTATATAAGCCTTGCAGCTTTGAGACCTTCAT

CTTTTTACAAGCCATTTTTC

In100 candidate810 (in intron):

LOCUS ENSG00000134851

DESCRIPTION Transmembrane protein 165 (Transmembrane protein TPARL)

(Transmmbrane protein PT27). [Source:Uniprot/SWISSPROT;Acc:Q9HC07]

AGTTTTATAAACCAAACTTCTAACTAGGGGATTTGCTGCTTTTTCACTTTACTTTAGTATGTTGTTATCTGGCTTCAGTC

CTTGCCTTTTCCTTGAAATT

In100 candidate811 (in intron):

LOCUS ENSG00000155719

DESCRIPTION Otoancorin precursor. [Source:Uniprot/SWISSPROT;Acc:Q7RTW8]

ACTCCTTTCCCTAAGATAACACCAATGGGTACTTTTGGATATATTCTTTCAGTCTAAGTATTTTATTTGTTTATTAATTT

ATCCATCCATCCATCCATCC

In100 candidate812 (in intron):

LOCUS ENSG00000134852

DESCRIPTION Circadian locomoter output cycles protein kaput (hCLOCK).

[Soure:Uniprot/SWISSPROT;Acc:O15516]

AAAAGTTAACTTTTAGTTTAATTGACTGCCTGTGGTTTTCTTATTTCCGCCCTGGTCTTTATTTCCTTTCCTCAGCTTAC

TTTTGGGTTTAATTTGTTTT

In100 candidate813 (in intron):

LOCUS ENSG00000134852

DESCRIPTION Circadian locomoter output cycles protein kaput (hCLOCK).

[Soure:Uniprot/SWISSPROT;Acc:O15516]

GATTTTGGGCTGTGCTTCGCTTAGTTCTGTTTAAACTTAACACACTTGGGTCTGCTTTCTGTTCTCCAGAAATCTATTGT

AAACACTTTCTCTCTGTTCT

In100 candidate814 (in intron):

LOCUS ENSG00000090061

DESCRIPTION Cyclin-K. [Source:Uniprot/SWISSPROT;Acc:O75909]

AAGAGCTTTCTTTCTAAGGTTAACTTTCTTGCTGTGCTTTTTATTATCCCTTGACCAGTTAGCCTCCCTTGTTCTGACTC

CTCTAGTGATTCCTTTTTGT

In100 candidate815 (in intron):

LOCUS ENSG00000132182

DESCRIPTION Nuclear pore membrane glycoprotein 210 precursor (POM210) (Nuclar

pore protein gp210). [Source:Uniprot/SWISSPROT;Acc:Q8TEM1]

AGATTAACTCTTTATATTTTCTTTTCTGTTGGGTTTCCTTCCTTACAGTAATTTTTTCTTCTTTGGTATTTTTATTTCCT

TTATGTCTGCCTCTCTTCTT

In100 candidate816 (in intron):

LOCUS ENSG00000090989

DESCRIPTION Exocyst complex component 1 (Exocyst complex component Sec3).

[ource:Uniprot/SWISSPROT;Acc:Q9NV70]

TTGTTTTGGTTGTTTTTTGAAAGATTTATTTTGATAACTACTCTCTGCCTTTTGTTTCCCCCTTTGGTTCTACAGAGACC

TCATGATTCTATCTACTTTT

In100 candidate817 (in intron):

LOCUS ENSG00000137821

DESCRIPTION Leucine-rich repeat-containing protein 49 (Tubulin polyglutamylse

complex subunit 4) (PGs4). [Source:Uniprot/SWISSPROT;Acc:Q8IUZ0]

AGATTCATAACTTTCATTTATTTATTTATTTATTTATTTAATTTTCTTTTATTTATTTATTTATTTTTGAAACAGAGTTT

CACTCTTGTTGCCTATGCTG

In100 candidate818 (in intron):

LOCUS ENSG00000187720

DESCRIPTION thrombospondin, type I, domain containing 4

[Source:RefSeq_peptde;Acc:NP_079093]

TATGATACAATTTTTATAAACTTTTATAACCTCTTAAAGTTTTATTTTATTCTTTTTCAACTTTTTATATTTATTCAGTT

TTATCTATACCATCTTTTTA

In100 candidate819 (in intron):

LOCUS ENSG00000187720

DESCRIPTION thrombospondin, type I, domain containing 4

[Source:RefSeq_peptde;Acc:NP_079093]

GGGTACATTTATGAAAATTAAGAAACCAACCTTGGTACGTTACTATTAACTAAATTCTAGACTTTCTTTGGTTTTCAGCA

GTTTTTCCATTGCCATCCTT

In100 candidate820 (in intron):

LOCUS ENSG00000187720

DESCRIPTION thrombospondin, type I, domain containing 4

[Source:RefSeq_peptde;Acc:NP_079093]

GCTTCTGTTATGGTAACTACTTCTACAATTTTTATTGATTTCTTCTTGCCACTTTTTGCTTGTAGTTGCAGCCTTCAAAA

CTATCTTCTTCTAATCCTTT

In100 candidate821 (in intron):

LOCUS ENSG00000174799

DESCRIPTION Centrosomal protein of 135 kDa (Cep135 protein) (Centrosomal

prtein 4). [Source:Uniprot/SWISSPROT;Acc:Q66GS9]

CCCACAAAGACATAACTAAAAAAAAATTCTACTCTTTATCTCCCTCGCATTTTGACTTGTGGTTGTCTTAATTTACAGAT

TTGTATATTGCCTATCTCTT

In100 candidate822 (in intron):

LOCUS ENSG00000106635

DESCRIPTION B-cell CLL/lymphoma 7 protein family member B.

[Source:Uniprot/WISSPROT;Acc:Q9BQE9]

ACTGCTTTATTTCTTAACTTTTCTCTCATTCATTTCATTTCTTGTCTTTCCTGGTGCATTCTGAGTTTCTCAACGCAGTT

TTTGCTCATTAATCTTTTAT

In100 candidate823 (in intron):

LOCUS ENSG00000106635

DESCRIPTION B-cell CLL/lymphoma 7 protein family member B.

[Source:Uniprot/WISSPROT;Acc:Q9BQE9]

AGTCTAGTAACTGTTGGAATGAATGTCACTTTAAGTCCTTCTATTTTCAGCATTTATGCTGTCTGCATTACATGCTAAAT

TTTTTTCTTTTCTTTTTTTT

In100 candidate824 (in intron):

LOCUS ENSG00000066933

DESCRIPTION myosin IXA [Source:RefSeq_peptide;Acc:NP_008832]

GGGTATCGCTGGGTCATATTGTTAAGAGTGTGTTTAACTTTCTGTGAATGTTTTACTTTTTGTTAGATTGTCTTCCAGAG

TTGTTCTACCATTTTGCATT

In100 candidate825 (in intron):

LOCUS ENSG00000066933

DESCRIPTION myosin IXA [Source:RefSeq_peptide;Acc:NP_008832]

GAATTTGCTTTTTGTAACGCTTTTAAGTCTTTTTTTTGCCTTAGGCCTTTAGTTTTTAACAGTAGTTTGTCTAGGTATAG

ACTTCTTTTTATTTCTTTCT

In100 candidate826 (in intron):

LOCUS ENSG00000152520

DESCRIPTION PABP1-dependent poly A-specific ribonuclease subunit PAN3

[Soure:RefSeq_peptide;Acc:NP_787050]

TAGTTTTCCCCTAACCTATACTACATACTTCCATCTCATATTTTCCTATCTAGGCACATTTATTCTTCCTTCAGAACTTT

ACTCAGGCATTCACTTTTCT

In100 candidate827 (in intron):

LOCUS ENSG00000152520

DESCRIPTION PABP1-dependent poly A-specific ribonuclease subunit PAN3

[Soure:RefSeq_peptide;Acc:NP_787050]

TTTTAGGGTAGAATTGACATCTTTATAACAAATGAGTGTTTATTCCCCTTTGTTTAAGTAATCTGTTATTTCTGTCAGTA

GGTTTTTATGTTTTCTTCAT

In100 candidate828 (in intron):

LOCUS ENSG00000128059

DESCRIPTION Amidophosphoribosyltransferase precursor (EC 2.4.2.14) (Glutamie

phosphoribosylpyrophosphate amidotransferase) (ATASE) (GPAT).

[Source:Unirot/SWISSPROT;Acc:Q06203]

AGTTACCTAACTCCGGGCAAGTTTTCATTTACTTTCAGTGTTACTCTCACTCTTTTTTCTTTCTTCTTCTTTTAAAATAT

AAACTCTAGCTCCTGCAGGA

In100 candidate829 (in intron):

LOCUS ENSG00000128059

DESCRIPTION Amidophosphoribosyltransferase precursor (EC 2.4.2.14) (Glutamie

phosphoribosylpyrophosphate amidotransferase) (ATASE) (GPAT).

[Source:Unirot/SWISSPROT;Acc:Q06203]

GTAATCTTTGTCCTCTTAACCCCATTCACATTTGGTACTTTTATTATACTATTTACTGTTTTCTCTCAGGTGTTAAATTT

ATTTGCAGTTCTGTCCTTTT

In100 candidate830 (in intron):

LOCUS ENSG00000121380

DESCRIPTION Apoptosis facilitator Bcl-2-like 14 protein (Apoptosis regulato

Bcl- G). [Source:Uniprot/SWISSPROT;Acc:Q9BZR8]

ACTCAAGCTTTTTCTAACTGTAATTCTGTATTTTTTAGCAAATCTCTTCTTATCTCTTCCTTCCCAGCCTCTAGTATCCC

CTATTCTACTCTTTACTTTT

In100 candidate831 (in intron):

LOCUS ENSG00000164588

DESCRIPTION Potassium/sodium hyperpolarization-activated cyclic nucleotideated

channel 1 (Brain cyclic nucleotide-gated channel 1) (BCNG-1).

[Source:niprot/SWISSPROT;Acc:O60741]

TAATACCTACTCCTAATCATATTTTTTTAACTTTCTTTGAACTTACTCTTCTGTTCCTTTTCTAACATCTCAAATCAGAT

GCTTAGTTTATTAATTTCTC

In100 candidate832 (in intron):

LOCUS ENSG00000070018

DESCRIPTION Low-density lipoprotein receptor-related protein 6 precursor.

[ource:Uniprot/SWISSPROT;Acc:O75581]

TTAAGATGGTGAAATGCCAAGTAACTTAGTTTTAACTACTCTTTATTTTTCATTTGTGTTACTTGTCTTTTTTGGCAGCT

TTCTCAGTTCTCTCTCTGTT

In100 candidate833 (in intron):

LOCUS ENSG00000151883

DESCRIPTION Poly [ADP-ribose] polymerase 8 (EC 2.4.2.30) (PARP-8).

[Source:niprot/SWISSPROT;Acc:Q8N3A8]

TTCCAACTAACCCTATTAACTTCCTCTCAATTTTACCTCTTCCTTTTTTAGCTGTGTTCCATCATTTGCCAATGGCAGCT

CAGCTTCTTTGTTCTTTTTAT

In100 candidate834 (in intron):

LOCUS ENSG00000151883

DESCRIPTION Poly [ADP-ribose] polymerase 8 (EC 2.4.2.30) (PARP-8).

[Source:niprot/SWISSPROT;Acc:Q8N3A8]

ATAAACATATTGAAAGAGTGATAAAAAGGCTTTTAACATTGAGCTCCATCTTCCTTTTTTACGGCGGTTTTATTTCAGAT

CCCATTTGGATGTTTATCTT

In100 candidate835 (in intron):

LOCUS ENSG00000165714

DESCRIPTION LOH1CR12 [Source:RefSeq_peptide;Acc:NP_477517]

TTTTATCAATTGAGAGTGAGGTACTGAATTATTAACCTGTTTTGTTGAATTGTGTATTTTTCCTTTTGATTCTGTCAGTT

TTTCCTTCATTTGTTTTGAG

In100 candidate836 (in intron):

LOCUS ENSG00000165714

DESCRIPTION LOH1CR12 [Source:RefSeq_peptide;Acc:NP_477517]

CATCTTAATATTAAATCTTCCAATCAATGACTATGGGGTGTCTTAACATTTGTATGTGTCCTCTTTAATTCCTTTCAGCT

GTATTTTATGGTTTTTTTTA

In100 candidate837 (in intron):

LOCUS ENSG00000111266

DESCRIPTION Dual specificity protein phosphatase 16 (EC 3.1.3.48) (EC

3.1.316) (Mitogen-activated protein kinase phosphatase 7) (MAP kinase phosphatas

7) (MKP-7). [Source:Uniprot/SWISSPROT;Acc:Q9BY84]

TCTTAGTAGATACAACACACTTGTGGGGTTGGAAAAAATGTATAATATTAACATTAATAGTAATCTTTCTTGTTACAGGA

TTTCTTTTCATTTCTTTGCC

In100 candidate838 (in intron):

LOCUS ENSG00000160633

DESCRIPTION Scaffold attachment factor B (Scaffold attachment factor B1) (SFB)

(HSP27 estrogen response element-TATA box-binding protein) (HSP27 ERE-

TATAbinding

protein). [Source:Uniprot/SWISSPROT;Acc:Q15424]

ATTAGTGTAAAAACGTCCGGGTCCAAAGAGAGAGTGAGTATTAATTTTCTAACTAGGGTATTTTGCTCTTCTTTTCAGTT

TGTATTTCTGTGTGCGTCTT

In100 candidate839 (in intron):

LOCUS ENSG00000168575

DESCRIPTION solute carrier family 20, member 2

[Source:RefSeq_peptide;Acc:N_006740]

TATCATTGTGTTCTATGAATAGGACCATAATTATGAATGAATTTTAACATTCATTAGATTTTTTTTTAATTATTCAGTT

GCTTCTTGGTTTATATCTCC

In100 candidate840 (in intron):

LOCUS ENSG00000164171

DESCRIPTION Integrin alpha-2 precursor (Platelet membrane glycoprotein Ia)

GPIa) (Collagen receptor) (VLA-2 alpha chain) (CD49b antigen).

[Source:Unipot/SWISSPROT;Acc:P17301]

ACATCTAACACAAAAGGATCTCTGGTCACCTTTACTCACTTCTTTTAACTTTATTTTGCTCAGATAGTCAATGTTAATCA

CTCTGTTGCTCCTTCCCTTT

In100 candidate841 (in intron):

LOCUS ENSG00000131379

DESCRIPTION Uncharacterized protein C3orf20.

[Source:Uniprot/SWISSPROT;Acc:8ND61]

TAACATGAAGTATGTCTGTATTTCTTGTTCTCTGTTCTGTATATTTCATCTTTTTTTTTCCCTTTTGGTCAGCCTAGGTA

TTTTCTACTGACCTGTCTTC

In100 candidate842 (in intron):

LOCUS ENSG00000137817

DESCRIPTION Poly [ADP-ribose] polymerase 6 (EC 2.4.2.30) (PARP-6).

[Source:niprot/SWISSPROT;Acc:Q2NL67]

TACATCATTTCTCACCACTCCTCTGCAATTCACTATTCTCTATGCAACTAACACACACTACCTTATCTTTCCTTCAGGCC

TTCTGTATCCTATTACTTCT

In100 candidate843 (in intron):

LOCUS ENSG00000120925

DESCRIPTION ring finger protein 170 [Source:RefSeq_peptide;Acc:NP_112216]

CTTAACACGTGGAGTATTGAGCTGATTCCCTCCTTGACATAGTCTGTTCTTTTCTCAGTTTTGATTTCCTCCTCTTCTTC

ATTCCTCTCATCTGGTCTAT

In100 candidate844 (in intron):

LOCUS ENSG00000177463

DESCRIPTION Orphan nuclear receptor TR4 (Orphan nuclear receptor TAK1).

[Sorce:Uniprot/SWISSPROT;Acc:P49116]

AAAGATTTATTGTTATCTCAACAAGCTGAATCAAAAAGCCAATTATAACGGTTGATTTCTCTGTTTTTCTTCTTGCAGTT

TTATCAGTTTTTTTGGCTCA

In100 candidate845 (in intron):

LOCUS ENSG00000132938

DESCRIPTION Uncharacterized protein KIAA0774.

[Source:Uniprot/SWISSPROT;AccQ5JR59]

TAAAATAAAAAGTAAAAGCTCCTAAATCCTACTGATTAGAAGTAACCATTATTAAAAGTTTGGTACCTTATCTTTCAGAT

ATTTTTACTTATTTCTAAGC

In100 candidate846 (in intron):

LOCUS ENSG00000132938

DESCRIPTION Uncharacterized protein KIAA0774.

[Source:Uniprot/SWISSPROT;AccQ5JR59]

AATATACGAAATACATTTACAATAACTGTTTAACTGTCCCTGTCTAGTAATTCTGTTATCTGTGTCATTTTTTGTCAGTT

TCAATTGATTGCATCTTCTC

In100 candidate847 (in intron):

LOCUS ENSG00000164258

DESCRIPTION NADH dehydrogenase [ubiquinone] iron-sulfur protein 4,

mitochonrial precursor (EC 1.6.5.3) (EC 1.6.99.3) (NADH-ubiquinone

oxidoreductase 1 kDa subunit) (Complex I-18 kDa) (CI-18 kDa) (Complex I-AQDQ)

(CI- AQDQ). [ource:Uniprot/SWISSPROT;Acc:O43181]

AAAAAGATAACCTTAAGTTAAAGGCTTACTTTTGCTTTGACTGTTTACTTTTTTTTTCCCCCTCTAAGGGGTCAGTTTTC

TTTTGGTGAGATTACTTTCT

In100 candidate848 (in intron):

LOCUS ENSG00000164258

DESCRIPTION NADH dehydrogenase [ubiquinone] iron-sulfur protein 4,

mitochonrial precursor (EC 1.6.5.3) (EC 1.6.99.3) (NADH-ubiquinone

oxidoreductase 1 kDa subunit) (Complex I-18 kDa) (CI-18 kDa) (Complex I-AQDQ)

(CI- AQDQ). [ource:Uniprot/SWISSPROT;Acc:O43181]

TTGCACCTCCCATCTACTACTGGATGAACTCCTCTTTCTTCCTTTTAACTTTCACAATAATTTGTTCCTTTCTTGCAGCT

CTTATAGTATGGTTTTCTCT

In100 candidate849 (in intron):

LOCUS ENSG00000139514

DESCRIPTION High-affinity cationic amino acid transporter 1 (CAT-1) (CAT1)

System Y+ basic amino acid transporter) (Ecotropic retroviral leukemia recetor

homolog) (ERR) (Ecotropic retrovirus receptor homolog).

[Source:UniprotSWISSPROT;Acc:P30825]

CTGTTAAGTTCCTTTCTTTACTTATCTCTTTAACCATAACCCCCATTTCTTTTTGTTTTGTTTTTGTACTGCATGCAGGC

ATCTTTATTTGTTCCTGTCT

In100 candidate850 (in intron):

LOCUS ENSG00000185305

DESCRIPTION ADP-ribosylation factor-like 15 (ARL15), mRNA

[Source:RefSeq_dn;Acc:NM_019087]

AGTTTGTTAGCCAAGGACCTTTATTTATTTATTGCTAACCATAAATATGATTGCTCCATTTTTTGTTGCATGTTCCAGAT

GAGTAGTTTTTTTTATTCTT

In100 candidate851 (in intron):

LOCUS ENSG00000185305

DESCRIPTION ADP-ribosylation factor-like 15 (ARL15), mRNA

[Source:RefSeq_dn;Acc:NM_019087]

AGTAACTACCAACAAGAATCTTAACCTTTTTGCTATTTGTTTTCTGTATGTCTTACACATTTTTGTCATTCCCTCCAGTA

CTGCCTTCTTTTGTGTTTAG

In100 candidate852 (in intron):

LOCUS ENSG00000168522

DESCRIPTION Protein farnesyltransferase/geranylgeranyltransferase type I alha subunit (EC 2.5.1.58) (EC 2.5.1.59) (CAAX farnesyltransferase alpha subuit) (Ras proteins prenyltransferase alpha) (FTase-alpha) (Type I protein geanylgeranyltransferase

alpha subunit) [Source:Uniprot/SWISSPROT;Acc:P49354

AGCGTCATTCATTCAAAATAACTCTCCTTTTCTTGTTTGTGTTTCAGGGTTTATTTTCTCTTGATCTTTCTACTGCTTTT

GTTCTTCCTTCACAAAATCA

In100 candidate853 (in intron):

LOCUS ENSG00000013583

DESCRIPTION Heme-binding protein 1 (p22HBP).

[Source:Uniprot/SWISSPROT;Acc:9NRV9]

CTGTATTTTTTTCTGAATCTCCTGTTTAACTTTATCTTCCAACCTGTTTACTGAATTTTTCTTCTCTGTTATCAGATTTT

TAATTTCTGAGAGCTCTTTT

In100 candidate854 (in intron):

LOCUS ENSG00000166847

DESCRIPTION Dynactin subunit 5 (Dynactin subunit p25).

[Source:Uniprot/SWISPROT;Acc:Q9BTE1]

TCCATGGACTAGGTAAGGTCTCCTTGTTATATATGCTCATAACTTGATATTGTGTTTCTGTAGCCCTTATCACAGTTGTC

ACTACTTACTATTTAATCTC

In100 candidate855 (in intron):

LOCUS ENSG00000150471

DESCRIPTION Latrophilin-3 precursor (Calcium-independent alpha-latrotoxin

rceptor 3) (Lectomedin-3). [Source:Uniprot/SWISSPROT;Acc:Q9HAR2]

AATTAAAAAGCAATCTTAAAAAGTTGGTTCTTTTGTTCTTTAACAAAATGAAATGTTTCCCTTTAGCTTTATTCACAGCC

ATGTGTTTGACTCTTTTACT

In100 candidate856 (in intron):

LOCUS ENSG00000150471

DESCRIPTION Latrophilin-3 precursor (Calcium-independent alpha-latrotoxin

rceptor 3) (Lectomedin-3). [Source:Uniprot/SWISSPROT;Acc:Q9HAR2]

ATTTTGATAGCATCCTGTCCCTTGTGTCCACATCCTGATAGTTAACAAAATTCATAGTAATTTCCTCAAATCTTTCAGGG

TATTACTCTCTTTCTATTTT

In100 candidate857 (in intron):

LOCUS ENSG00000150471

DESCRIPTION Latrophilin-3 precursor (Calcium-independent alpha-latrotoxin

rceptor 3) (Lectomedin-3). [Source:Uniprot/SWISSPROT;Acc:Q9HAR2]

TGTAACCTTTCACCTTTTCTTCTTTCCTTATTCCTTCTTTCCTTTCTTTTTCTTCCTTTCTCTTTTCCTTCCAGTATCCA

TCCATCACTCATTTTTAATT

In100 candidate858 (in intron):

LOCUS ENSG00000150471

DESCRIPTION Latrophilin-3 precursor (Calcium-independent alpha-latrotoxin

rceptor 3) (Lectomedin-3). [Source:Uniprot/SWISSPROT;Acc:Q9HAR2]

GTAACTGCAAGTCTTCTTGTCTTGTTTCAGGAGCTCTTCTTCCACAGATTATCTGAATAAACCCATAGAATTTTCTTAGT

CTCTCTCTTTCTCCTTCTTT

In100 candidate859 (in intron):

LOCUS ENSG00000150471

DESCRIPTION Latrophilin-3 precursor (Calcium-independent alpha-latrotoxin

rceptor 3) (Lectomedin-3). [Source:Uniprot/SWISSPROT;Acc:Q9HAR2]

ACTTTTATACTTCACTGTTTTATAGCAAAGTTAACTCCGTTTAACTTTTAACTTTTCCTTGGTCACTACAGATTATCAT

TATGTTGGGGTTTCTTTTTT

In100 candidate860 (in intron):

LOCUS ENSG00000150471

DESCRIPTION Latrophilin-3 precursor (Calcium-independent alpha-latrotoxin

rceptor 3) (Lectomedin-3). [Source:Uniprot/SWISSPROT;Acc:Q9HAR2]

TTCATTTCACACTGTACATTTTACCTTCTAACACTATATGGTTTATTATTTGCTTCCCTTGCCAGAATGTTCAGTTCTAA

GATCATTTTTTCTCTGCTTT

In100 candidate861 (in intron):

LOCUS ENSG00000166233

DESCRIPTION Protein ariadne-1 homolog (ARI-1) (Ubiquitin-conjugating

enzymeE2- binding protein 1) (UbcH7-binding protein) (UbcM4-interacting protein)

HHARI) (H7-AP2) (Monocyte protein 6) (MOP-6).

[Source:Uniprot/SWISSPROT;AccQ9Y4X5]

TTTGGGCAAAGGTCGGAGCTGGTGGAGAAAGGACACCTTTAACTTTATGGTCTTTTCTTTTATCCATTATAGCAGTTTTC

CAGACCACTGTCATTTTCTT

In100 candidate862 (in intron):

LOCUS ENSG00000122042

DESCRIPTION Ubiquitin-like protein 3 precursor (Membrane-anchored

ubiquitinfold protein) (MUB) (HsMUB) (Protein HCG-1).

[Source:Uniprot/SWISSPROT;Acc:95164]

TAGCTAGTAGTCTAATTTAATCTTCAAATAACCAACTCCTGGATTTGTTGATCTTTTGTATGGTTTTTCACATCTCAGTT

TCCTTCATTTCAGCTCTAAT

In100 candidate863 (in intron):

LOCUS ENSG00000134531

DESCRIPTION Epithelial membrane protein 1 (EMP-1) (Tumor-associated membran

protein) (CL-20) (B4B protein). [Source:Uniprot/SWISSPROT;Acc:P54849]

TTAGATGAAAGTGGCTTATTAACCACTAGATTCATTGTTATTTTCCCAGTAGGTCTTATGTTTCTTAATTGTTTCCAGTT

GGACATTTCACTTTTTTCCT

In100 candidate864 (in intron):

LOCUS ENSG00000150086

DESCRIPTION Glutamate [NMDA] receptor subunit epsilon 2 precursor (N-

methylDaspartate

receptor subtype 2B) (NR2B) (NMDAR2B) (N-methyl-D-aspartate reeptor

subunit 3) (NR3) (hNR3). [Source:Uniprot/SWISSPROT;Acc:Q13224]

GTGTTATCTTCTGTAAATTATAGAAAGAATGATAACATAACCTTTTATATTTAGTCATTTATTTTCCATTTTCAGTACAT

CTTATTTCTTCTTGTAGATC

In100 candidate865 (in intron):

LOCUS ENSG00000150086

DESCRIPTION Glutamate [NMDA] receptor subunit epsilon 2 precursor (N-

methylDaspartate

receptor subtype 2B) (NR2B) (NMDAR2B) (N-methyl-D-aspartate reeptor

subunit 3) (NR3) (hNR3). [Source:Uniprot/SWISSPROT;Acc:Q13224]

CCCTGAAACCCTAATGGCGGTTTAGTGTAGGTCATGTTCAATACGTAACTTCAATAGGAGTATTAATTCCTTTCAGCGTT

GTTCACCCATCATTTCCTTT

In100 candidate866 (in intron):

LOCUS ENSG00000145242

DESCRIPTION Ephrin type-A receptor 5 precursor (EC 2.7.10.1) (Tyrosine-protin

kinase receptor EHK-1) (EPH homology kinase 1) (Receptor protein- tyrosie kinase

HEK7). [Source:Uniprot/SWISSPROT;Acc:P54756]

CTCTTTAGTTTTTGTTTTCTTTCAAGCCCTAATAACCTCTTATGTCTTCTCATAATGATTCTTCTCCAGCTTATGGCCTC

TTGCTAGATTGCCTTTATTC

In100 candidate867 (in intron):

LOCUS ENSG00000171681

DESCRIPTION activating transcription factor 7 interacting protein

[Source:RfSeq_peptide;Acc:NP_060649]

TTGGGTTAACAATAGTTGGAGGTCTTTGAGCTTCTTGAATTTGCATCTCCATTTTCTTCTTCCAGTTTCAGACATTTTCA

GCAATTATTTATCTTTCTCT

In100 candidate868 (in intron):

LOCUS ENSG00000121031

DESCRIPTION DNA-dependent protein kinase catalytic subunit (EC 2.7.11.1) (DAPK catalytic subunit) (DNA-PKcs) (DNPK1) (p460).

[Source:Uniprot/SWISSPRO;Acc:P78527]

TGTATAACATTAAGGAGCCTTCCTTGATTTATCTCTTCTGTGGCCTTGCAGGGTGTAGCCACCTTTCTCCTTTGGTATCT

TTATCCTCCAGTGGCTATTT

In100 candidate869 (in intron):

LOCUS ENSG00000121031

DESCRIPTION DNA-dependent protein kinase catalytic subunit (EC 2.7.11.1) (DAPK catalytic subunit) (DNA-PKcs) (DNPK1) (p460).

[Source:Uniprot/SWISSPRO;Acc:P78527]

TTTTTTTCTAACTGTATTAAGTACATTCTCATACTTTTGTGATATTTTTGCATTCCTTGCAGAGCATTGTGAACATAGTT

TAGCTTTTTTTTTTTTTTTT

In100 candidate870 (in intron):

LOCUS ENSG00000121031

DESCRIPTION DNA-dependent protein kinase catalytic subunit (EC 2.7.11.1) (DAPK catalytic subunit) (DNA-PKcs) (DNPK1) (p460).

[Source:Uniprot/SWISSPRO;Acc:P78527]

AGATTCACTGATTTTTTAAATAACATTTTAAACATTTTTCCATGTTAGTATTTGTATTTTTTTCTTTTCTACCAGACCCT

ACATATTATTTATTTTCTGA

In100 candidate871 (in intron):

LOCUS ENSG00000121031

DESCRIPTION DNA-dependent protein kinase catalytic subunit (EC 2.7.11.1) (DAPK catalytic subunit) (DNA-PKcs) (DNPK1) (p460). [Source:Uniprot/SWISSPRO;Acc:P78527]

CCCATCCATGAACATGAAGTAACTTTGCATTTCTTTGAGTCCTCTTCAATTTCTTTCATCCATGTTTTACAGTTTTCATT

GTAGAGATCTTTCATTTCTT

In100 candidate872 (in intron):

LOCUS ENSG00000067141

DESCRIPTION Neogenin precursor. [Source:Uniprot/SWISSPROT;Acc:Q92859]

AGTGGCTTAGTTACTATAGCTTTATACATACTTGTAACTAACAAGTGGAGTCCCTTTACTTTGTTTTGCAGAATTACTTT

GGCTCTTACTGGCTTTTTTT

In100 candidate873 (in intron):

LOCUS ENSG00000102781

DESCRIPTION Katanin p60 ATPase-containing subunit A-like 1 (EC 3.6.4.3) (Kaanin p60 subunit A-like 1) (p60 katanin-like 1). [Source:Uniprot/SWISSPROT;cc:Q9BW62]

ATACAAACGTTAATGAAATGTAGCTCCTGGCCTGAATAAATTAACTTTTAAGCTATATTTTTTCTGTTTTATTTCAGTGT

GTCTGGTCTCTTCCTACTAT

In100 candidate874 (in intron):

LOCUS ENSG00000006116

DESCRIPTION Voltage-dependent calcium channel gamma-3 subunit (Neuronal volage- gated calcium channel gamma-3 subunit).

[Source:Uniprot/SWISSPROT;Acc:60359]

GATTTTCATTCATTAGAGGAGATAACCCCAAATGTTGTGGATTTATTCTCTCCTCTCCTGAATATTATTCAGTTTTATAT

TACTTTAGTAATCTTATTTC

In100 candidate875 (in intron):

LOCUS ENSG00000140153

DESCRIPTION WD repeat protein 20 (DMR protein).

[Source:Uniprot/SWISSPROT;Ac:Q8TBZ3]

TTAACCTCAACTTTTTTTATACATTTTTGACCCACTCAGTACTTCACCTCCTTTGCTAGTTCTTTCTACTCTACCTAGA

ATTTAACTATCGGATTTCTT

In100 candidate876 (in intron):

LOCUS ENSG00000140153

DESCRIPTION WD repeat protein 20 (DMR protein).

[Source:Uniprot/SWISSPROT;Ac:Q8TBZ3]

CCTTTTAATTCCTCTCACAAACCTTAACCTTTTTTGACATAGATTCTTCTTCTTATTTATTTATTTATTTGACACAGGGT

CTTGCTTTGTCTTCCAGGCT

In100 candidate877 (in intron):

LOCUS ENSG00000131373

DESCRIPTION 2-hydroxyacyl-CoA lyase 1 (EC 4.1.-.-) (2-hydroxyphytanoyl-CoA

yase) (2-HPCL). [Source:Uniprot/SWISSPROT;Acc:Q9UJ83]

AGGGTATGGGTGTGTCTATTTATGCCTCACTAACTTTATTTGTTGTTTTCTTATTATTTGTTGTTTTGCAGTAATCTTTT

TTTTTCATGTTTTTCTTCTG

In100 candidate878 (in intron):

LOCUS ENSG00000033178

DESCRIPTION Ubiquitin-activating enzyme E1-like protein 2 (Monocyte protein4)

(MOP-4). [Source:Uniprot/SWISSPROT;Acc:A0AVT1]

AGTTTTTTTTAACCATATATATGTAGGTCTATTTCTGTACTCTATTGTCTTCATGTTGTTTCTGTCCTTTTGCCAGCTCC

ATCCTGTCTTGCTTATTGTG

In100 candidate879 (in intron):

LOCUS ENSG00000033178

DESCRIPTION Ubiquitin-activating enzyme E1-like protein 2 (Monocyte protein4)

(MOP-4). [Source:Uniprot/SWISSPROT;Acc:A0AVT1]

TCTTGCTTTAACATTCCTGATCTCTAATCTACCTATCTACTCTTCATCTAGTTTAGCTTTTTTTCTTCATCCTTTCAGTT

TCAGCCTAAATTTCTTTTTA

In100 candidate880 (in intron):

LOCUS ENSG00000169814

DESCRIPTION Biotinidase precursor (EC 3.5.1.12).

[Source:Uniprot/SWISSPROT;cc:P43251]

TTCTCCTTCTGGTATTCCCATTGTACATATTTATAACTTTTGTAGTCCACTGTTTGTTGATTTTTTTCTTTTTTTCAGTC

TTTTTTCTCTTTGCTTTTCA

In100 candidate881 (in intron):

LOCUS ENSG00000039123

DESCRIPTION Superkiller viralicidic activity 2-like 2 (EC 3.6.1.-) (ATPdepndent

helicase SKIV2L2). [Source:Uniprot/SWISSPROT;Acc:P42285]

TCTATTATTGAAAGTGGAGTATTGAAGTCTCGTAACTATTGTTGTTGTATTATCTATTTTTCCCTTCATTTCTATCAGTT

TTTGCTTCATGTGTTTTCAA

In100 candidate882 (in intron):

LOCUS ENSG00000039123

DESCRIPTION Superkiller viralicidic activity 2-like 2 (EC 3.6.1.-) (ATPdepndent

helicase SKIV2L2). [Source:Uniprot/SWISSPROT;Acc:P42285]

ACTATTAATTATCGTGTGTTCTCCTGCCTTGCCTCTTAACAAAAATTCTGCTGTTCCTGATTATTTCCATTTTACCAGTG

TTTTGTTCCTTTTCTATCCA

In100 candidate883 (in intron):

LOCUS ENSG00000189403

DESCRIPTION High mobility group protein B1 (High mobility group protein 1)

HMG- 1). [Source:Uniprot/SWISSPROT;Acc:P09429]

TGTTTCTGAATGCTTGCTGTGCTGAATATTGGCACTGAATGTTCATGTTTAACCTTTTGTCTAATTATTTCATTTCAGGT

CCTCCTTTTAGGTCATTATT

In100 candidate884 (in intron):

LOCUS ENSG00000156642

DESCRIPTION Neuroplastin precursor (Stromal cell-derived receptor 1) (SDR-1.

[Source:Uniprot/SWISSPROT;Acc:Q9Y639]

AGAGTAGCATTGTTTTATTTTGATAACCTCACATTTCTATATGTGTTTTCTTCAGTTCAGCTACCTTATATCTGACAGTA

ATTTTTTTCATTTATTCTCT

In100 candidate885 (in intron):

LOCUS ENSG00000130377

DESCRIPTION bubblegum related protein

[Source:RefSeq_peptide;Acc:NP_112186]EXON

TTATTCTTAGTTAACTGAGCCAAGTAGTTTATCATATGATTCCACTTCCTGCTTGGTACAACTTTTTGTTTTTCCCAGAA

TTAAGTTGCCTTTCTTTTCC

In100 candidate886 (in intron):

LOCUS ENSG00000067113

DESCRIPTION Lipid phosphate phosphohydrolase 1 (EC 3.1.3.4) (Phosphatidic aid phosphatase 2a) (Phosphatidate phosphohydrolase type 2a) (PAP2a) (PAP- 2) (PAP2-alpha). [Source:Uniprot/SWISSPROT;Acc:O14494]

AGTGAACATAATAACTTATAATTCTCCATCTGCATTTCAGTTTCTTTTCTTCTTTTCTTTTCTTTTTTCTTTTTTTTTTT

TTTTAAGACGGAATCTTGCT

In100 candidate887 (in intron):

LOCUS ENSG00000067113

DESCRIPTION Lipid phosphate phosphohydrolase 1 (EC 3.1.3.4) (Phosphatidic aid phosphatase 2a) (Phosphatidate phosphohydrolase type 2a) (PAP2a) (PAP- 2) (PAP2-alpha). [Source:Uniprot/SWISSPROT;Acc:O14494]

AGCAAAATGCATTGATATTAACAAGTTAATTTTGTTTTAATTTTATTTATTTATCTATTTTTTTTTGAGACAGGATCTT

TTTTTTTTTTTTTTTTTTTT

In100 candidate888 (in intron):

LOCUS ENSG00000067113

DESCRIPTION Lipid phosphate phosphohydrolase 1 (EC 3.1.3.4) (Phosphatidic aid phosphatase 2a) (Phosphatidate phosphohydrolase type 2a) (PAP2a) (PAP- 2) (PAP2-alpha). [Source:Uniprot/SWISSPROT;Acc:O14494]

CAAAAAAGCCACTTGTTTTGCCTCCTTTATGCTTTAACCATTGCTCAATCTTCAAAGTTTATTCTTCCTCAGCACCCTTT

TCTATTGACTCAAATTGTTT

In100 candidate889 (in intron):

LOCUS ENSG00000067113

DESCRIPTION Lipid phosphate phosphohydrolase 1 (EC 3.1.3.4) (Phosphatidic aid phosphatase 2a) (Phosphatidate phosphohydrolase type 2a) (PAP2a) (PAP- 2) (PAP2-alpha). [Source:Uniprot/SWISSPROT;Acc:O14494]

ATCAATTTTATTAACCTTTTGGTTAGTTTCAAGGGTTTCTTTTGATTTCATTGATTTTTATCTATTTTTTGTGTTCAGTT

TCATTTATTTTTGCTTTTAT

In100 candidate890 (in intron):

LOCUS ENSG00000034239

DESCRIPTION EF-hand calcium-binding domain-containing protein 1. [Source:Unprot/SWISSPROT;Acc:Q9HAE3]

AGCTGTTTTTGAAATCATAACCATTATATTGTTTCCTACTATTTATTTGCTGAATCTTTTTTCCCAGACTTTTTAGTGTT

TCTCTTTAAATGTGGTTTTC

In100 candidate891 (in intron):

LOCUS ENSG00000131386

DESCRIPTION Polypeptide N-acetylgalactosaminyltransferase-like protein 2 (E

2.4.1.41) (Protein-UDP acetylgalactosaminyltransferase-like protein 2)

(UDGalNAc:

polypeptide N-acetylgalactosaminyltransferase- like protein 2)

(Polpeptide GalNAc transferase-like protein [Source:Uniprot/SWISSPROT;Acc:Q8N3T]

ATACCTAGGCCCTTATAACCTATATATTTATGTTCTTTCTCCAAATGCTGACTTTCTAAATTTACTTATATTCCTCAGCT

CCTGACTTTTCTTCCTTATT

In100 candidate892 (in intron):

LOCUS ENSG00000089902

DESCRIPTION REST corepressor 1 (Protein CoREST).

[Source:Uniprot/SWISSPROT;cc:Q9UKL0]

GCTCATTGACATTCCTTCCTCTCAATACCTTAACTATGGCATGTTTTATAATTTGCTTTTGTTTTTGCTTTATCACAGTA

TTTTCCTATGTTTTTTATAG

In100 candidate893 (in intron):

LOCUS ENSG00000198092

DESCRIPTION transmembrane protease, serine 11F

[Source:RefSeq_peptide;Acc:N_997290]

GTGGGCAAGACGTAGGGGACAAAACAACTTATTACCTTTAACCATGAGACTTTTGTTTGTTTTACCTTTCAGATGATAAG

TCTTTCTATTACCTTGCCTC

In100 candidate894 (in intron):

LOCUS ENSG00000147481

DESCRIPTION Gamma-1-syntrophin (G1SYN) (Syntrophin 4) (SYN4).

[Source:Uniprt/SWISSPROT;Acc:Q9NSN8]

GAAATCCTTTTTAACCTCTGGGGAGAATGTCCCTTCCTGGTTGTGCTACTTTCTCTGCTTTCTTGTCTCATTCTACAGTT

ATTCTTTCATTGCTGTTTAG

In100 candidate895 (in intron):

LOCUS ENSG00000147481

DESCRIPTION Gamma-1-syntrophin (G1SYN) (Syntrophin 4) (SYN4).

[Source:Uniprt/SWISSPROT;Acc:Q9NSN8]

AATCTCTTAACTTGTATGTATACTTTATGAGTCTCTGCTTTTTCCCTGATTGTTTCCATTTCTTAAGTTTTTCAGCTTGA

ATTTTTGTTCTTATAGCTTT

In100 candidate896 (in intron):

LOCUS ENSG00000147481

DESCRIPTION Gamma-1-syntrophin (G1SYN) (Syntrophin 4) (SYN4).

[Source:Uniprt/SWISSPROT;Acc:Q9NSN8]

AGTAGTGATATCCCCTTTAACCTTTTTTTGTTGTGTTTATTTGATTCTACTCTCTTTTCTTCTTAGTTTAGCTAGCAGTC

TATCTATTTTGTTTTTTCAA

In100 candidate897 (in intron):

LOCUS ENSG00000147481

DESCRIPTION Gamma-1-syntrophin (G1SYN) (Syntrophin 4) (SYN4).

[Source:Uniprt/SWISSPROT;Acc:Q9NSN8]

TGAATAGTCTTTCCTTTTTTCAATCTTTCTTACAACTTTTATTCTAACTGGTTAATTCTCACAATTCTTTTATCCAGTTC

ACTGACTCTTCTGTTCTCTT

In100 candidate898 (in intron):

LOCUS ENSG00000152670

DESCRIPTION Probable ATP-dependent RNA helicase DDX4 (EC 3.6.1.-) (DEAD

boxprotein 4) (VASA homolog). [Source:Uniprot/SWISSPROT;Acc:Q9NQI0]

GTATAACTTATTTTCCATATGATTCTTCTAACATTTTATATAGTTTCCTTTTTTTTTTCTTTTTCTTTTTTGAGACAGTT

TTGCTCTTGTTGCCTAGGCT

In100 candidate899 (in intron):

LOCUS ENSG00000147485

DESCRIPTION peroxidasin homolog (Drosophila)-like (PXDNL), mRNA

[Source:Refeq_dna;Acc:NM_144651]

GAGCTTCGCCTTTCTCTGGTCCCTCTCTGATTAGCTTAATAAATAACCTCCTGAATTCTTTTTCTCGTAAGTCAGGGATT

TCTTCTTGGTTTGGATCCTT

In100 candidate900 (in intron):

LOCUS ENSG00000154814

DESCRIPTION oxidoreductase NAD-binding domain containing 1

[Source:RefSeq_pptide;Acc:NP_612390]

CACTGGCCTTGGTTCCTTTAACATGCCGGGCTTATTTCTGTGTTGGACCTTTGCTCTTGCTAGTCCTTCTGCCAGAGTTG

TCTGCCTTTAACATTTGCTT

In100 candidate901 (in intron):

LOCUS ENSG00000168300

DESCRIPTION Protein-L-isoaspartate O-methyltransferase domain-containing prtein 1. [Source:Uniprot/SWISSPROT;Acc:Q96MG8]

TTGCTAACTAAGATGCTGCTTTTCCTTTTTATTCATTACTTTTGGACAGATTTACTTTCTGTCATCATAGATAAGGTTTT

AGCTCCCATGCCCCTCTTTT

In100 candidate902 (in intron):

LOCUS ENSG00000147488

DESCRIPTION Suppression of tumorigenicity protein 18 (Zinc finger protein 37). [Source:Uniprot/SWISSPROT;Acc:O60284]

TGTGCTGTGAGGCACATGCCAAAACAAATCATATTATCATTTTAACTAAAATCCTCCTGTGTTTCTTTTTTTAAACAGTC

TTTCTGGTTGTCCAATTGCT

In100 candidate903 (in intron):

LOCUS ENSG00000147488

DESCRIPTION Suppression of tumorigenicity protein 18 (Zinc finger protein 37). [Source:Uniprot/SWISSPROT;Acc:O60284]

ATGGGTTTCTAATATTTATTTTTTATAACATATGTATTTTTCTTTTTCTTTTCTTTTTTTTTTTTTTTTTGAGACAGAG

TTTTACTCTTGTTGCCCAGT

In100 candidate904 (in intron):

LOCUS ENSG00000087128

DESCRIPTION Transmembrane protease, serine 11E precursor (EC 3.4.21.-) (Serne protease DESC1) [Contains: Transmembrane protease, serine 11E non- cataltic chain; Transmembrane protease, serine 11E catalytic chain]. [Source:Unirot/SWISSPROT;Acc:Q9UL52]

TTTATGAATAACTAAGTTCCACTAATTCTATTTTCCCAATAACTTTCCAATTTGCTCACCTCTTTATTCCAGTAATCTTG
CTACTGCTACTCATTATTCT

In100 candidate905 (in intron):

LOCUS ENSG00000131374

DESCRIPTION TBC1 domain family member 5. [Source:Uniprot/SWISSPROT;Acc:Q9269]

GGACCTAAAAAAAAAAAAAAATACTTCTTCGCTCCTAAGTAGTTATAACAACTGGCCTTCTCTCTCTTGGATTTGCAGTT
CATATTTTACTTTGCCTGGT

In100 candidate906 (in intron):

LOCUS ENSG00000131374

DESCRIPTION TBC1 domain family member 5. [Source:Uniprot/SWISSPROT;Acc:Q9269]

AGCATTTTATAGGCATTATAACATAGAATCCTCCATATCATTTCACTTTCTTTTTACAGGCAACTTTTTTGAAGGACTTA
TCTCTACTTTGCTACCTTTT

In100 candidate907 (in intron):

LOCUS ENSG00000131374

DESCRIPTION TBC1 domain family member 5. [Source:Uniprot/SWISSPROT;Acc:Q9269]

CTTTTAATACATCTTCTTTGCTGTAACATTCTAGTTTTCATTATGTGGTCTGCATTTTCTTTGTGTTCTACCTCCCAGGA
GATGTTTCTTCTCTTCTCAT

In100 candidate908 (in intron):

LOCUS ENSG00000131374

DESCRIPTION TBC1 domain family member 5. [Source:Uniprot/SWISSPROT;Acc:Q9269]

TTTATTTGCTGATTTCTTACTATTGATTTGATAGTCTATAATTAACATGTATCTTTTTAATAATTTTTTTTTCAGTTCTT

GGTACTTAATTCTAGTCCTT

In100 candidate909 (in intron):

LOCUS ENSG00000023287

DESCRIPTION RB1-inducible coiled-coil protein 1.

[Source:Uniprot/SWISSPROT;cc:Q8TDY2]

ACTTTTTCTCTTTTTTTAGTGGCTAACTGTAGCTAACCACTTGCTCATTTCTTAATTTTGTTCATTTTTCAGCTCTACAG

TTTCAGTTTGGTTCTTTTGT

In100 candidate910 (in intron):

LOCUS ENSG00000134352

DESCRIPTION Interleukin-6 receptor subunit beta precursor (IL-6R-beta)

(Intrleukin-6 signal transducer) (Membrane glycoprotein 130) (gp130) (Oncostati-

M receptor alpha subunit) (CD130 antigen) (CDw130).

[Source:Uniprot/SWISSPOT;Acc:P40189]

TATATTTAAACATATTTGATATGTTTTAACTCATTATGGATTGTCCCATCTTTGATCAGTGGCAGTTTACTCAAATTGGC

TCATTCATCCTTTTTTTTTT

In100 candidate911 (in intron):

LOCUS ENSG00000075413

DESCRIPTION MAP/microtubule affinity-regulating kinase 3 (EC 2.7.11.1) (Cdc5C- associated protein kinase 1) (cTAK1) (C-TAK1) (Serine/threonine proteinkinase p78) (Ser/Thr protein kinase PAR-1) (Protein kinase STK10). [Source:niprot/SWISSPROT;Acc:P27448]

AGTTCTCTCCCAACAATTGAAGATACCTGCGTTAACACATAATAAATTCTCATATGTGTCCTGTTATTTCTCCAGTCTGT
TTATTTAGTCCTATACCCAT

In100 candidate912 (in intron):

LOCUS ENSG00000173487

DESCRIPTION

TTTATGAATAACTAAGTTCCACTAATTCTATTTTCCCAATAACTTTCCAATTTGCTCACCTCTTTATTCCAGTAATCTTG
CTACTGCTACTCATTATTCT

In100 candidate913 (in intron):

LOCUS ENSG00000182568

DESCRIPTION DNA-binding protein SATB1 (Special AT-rich sequence-binding proein 1). [Source:Uniprot/SWISSPROT;Acc:Q01826]

AGTATCCATATTGGAAATTTCAAATTTAACCTACCATATTTCTATTTCTTATACTTTAGCTAACATATCTTCCTTCAGAT
TTCCCCACATTTTAACTTTT

In100 candidate914 (in intron):

LOCUS ENSG00000151491

DESCRIPTION Epidermal growth factor receptor kinase substrate 8. [Source:Unprot/SWISSPROT;Acc:Q12929]

CTTGAGAGGGAACCATGGCTCTTAACCTTTCATTTATACTTTCTTTCATTTTATTTCCTCTTCCTTCTGTTAAATCAGGT

ATTTTTATATTCCTCTACTC

In100 candidate915 (in intron):

LOCUS ENSG00000183960

DESCRIPTION Potassium voltage-gated channel subfamily H member 8 (Voltagegted potassium channel subunit Kv12.1) (Ether-a-go-go-like potassium channel3) (ELK channel 3) (ELK3) (ELK1) (hElk1). [Source:Uniprot/SWISSPROT;Acc:Q9642]

AGATTTTCTCCCACTGTGTGGGTTGTCTGTTAACTCTGCTGTTTATTTCTTTTGCTCTGCAGAAGCTTTTTAGTTTCATT

AAGTCCCATCTATTTATCTT

In100 candidate916 (in intron):

LOCUS ENSG00000182601

DESCRIPTION Heparan sulfate glucosamine 3-O-sulfotransferase 4 (EC 2.8.2.23 (Heparan sulfate D-glucosaminyl 3-O-sulfotransferase 4) (Heparan sulfate 3Osulfotransferase

4) (h3-OST-4). [Source:Uniprot/SWISSPROT;Acc:Q9Y661]

GCTCTCTTCACCTTTGTCCAACCCACTGCTTAACTTACATACTGAATTCTTAATTTCAGTTATTGTAGTTTTCAGTTCTA

GGATTTCTATTTTATTCCTT

In100 candidate917 (in intron):

LOCUS ENSG00000182601

DESCRIPTION Heparan sulfate glucosamine 3-O-sulfotransferase 4 (EC 2.8.2.23

(Heparan sulfate D-glucosaminyl 3-O-sulfotransferase 4) (Heparan sulfate

3Osulfotransferase

4) (h3-OST-4). [Source:Uniprot/SWISSPROT;Acc:Q9Y661]

AGTCCATAACATCCCCATTGCTCTTAGTACCTTATTTTATTTAACTCTCTCAGCTTTATAATACGTTTTTCTTTTCCTTA

CTATTTGCTTCTCACACTTT

In100 candidate918 (in intron):

LOCUS ENSG00000198277

DESCRIPTION UDP-glucuronosyltransferase 2B11 precursor (EC 2.4.1.17) (UDPGT.

[Source:Uniprot/SWISSPROT;Acc:O75310]

TGTTTAGTCTAAATTAACTTAGAATTGGTATAGATGGCCTTTTTTTTCTCTCTGCTGGTCTTTCCTTGCCTCTGCCAGCT

GCTTATGCTGCTGTTCTCTT

In100 candidate919 (in intron):

LOCUS ENSG00000171234

DESCRIPTION UDP-glucuronosyltransferase 2B7 precursor (EC 2.4.1.17)

(UDPGT)(3,4- catechol estrogen specific) (UDPGTh-2).

[Source:Uniprot/SWISSPROT;AccP16662]

GAGTATTCTATTTACATTAGTCTTTGAGTAGTTCTTATTTACTAACATCCCTTGATCTCATTCCTACTCTTTATACAGTT

CTCACATTCTATAACTTTTG

In100 candidate920 (in intron):

LOCUS ENSG00000135226

DESCRIPTION UDP-glucuronosyltransferase 2B28 precursor (EC 2.4.1.17) (UDPGT.

[Source:Uniprot/SWISSPROT;Acc:Q9BY64]

TACAATTTTCTTAGGTTCTGTTTATTTGTTTTCCTTTTTTTTTAACTGACTGAATAATTTCAATGACTCTTTCTTCAGGT

TTGCCAATTCTTTCTTCTAT

In100 candidate921 (in intron):

LOCUS ENSG00000048540

DESCRIPTION LIM domain only protein 3 (Neuronal-specific transcription factr

DAT1) (Rhombotin-3). [Source:Uniprot/SWISSPROT;Acc:Q8TAP4]

AACACTCCTAGAATAAGGAAATATTAAATATTTAACTTGTTTGAGGTCTTTTTATTCTGTCTGGTAGTCTCAGCTCTCTT

TTTTGCTTTTGTCTTCCTTT

In100 candidate922 (in intron):

LOCUS ENSG00000129810

DESCRIPTION Shugoshin-like 1 (hSgo1) (Serologically defined breast cancer

atigen NY-BR-85). [Source:Uniprot/SWISSPROT;Acc:Q5FBB7]

TCTCTTAACTCCTTTTTCATATTTTCCATCTTTTTAGCTACATTCTTTCCATATCATTTCGTCAGTTCTATCTTCCAGTT

CACTCATTCTATCCTTGGTT

In100 candidate923 (in intron):

LOCUS ENSG00000164512

DESCRIPTION Ankyrin repeat domain-containing protein 55.

[Source:Uniprot/SWSSPROT;Acc:Q3KP44]

AACAAACTATTATCTCCTAGGTTAATTAACAATAAAAAATAAAAGTTTTTATTTTACCTTCACTTATTCCTTCTTCAGCG

TTCTTCTTTTCTTTACGTAA

In100 candidate924 (in intron):

LOCUS ENSG00000182247

DESCRIPTION Ubiquitin-conjugating enzyme E2 E2 (EC 6.3.2.19) (Ubiquitinproein ligase E2) (Ubiquitin carrier protein E2) (UbcH8). [Source:Uniprot/SWISPROT;Acc:Q96LR5]

AATTATTTTTACTCCGCTTTTTCTTTCCGTAGGAGTTAACACCTCCTAACATTTACTTCTATCAGTTTCTTATGATTAAC TGTGTTGTGTATTCCCTCCC

In100 candidate925 (in intron):

LOCUS ENSG00000182247

DESCRIPTION Ubiquitin-conjugating enzyme E2 E2 (EC 6.3.2.19) (Ubiquitinproein ligase E2) (Ubiquitin carrier protein E2) (UbcH8). [Source:Uniprot/SWISPROT;Acc:Q96LR5]

AGTAGTTTGTTCAACAAGGAAATAACATATAAAGCAATATGTTTATTTCAGGTTATTTCCAGTTTATTTTCTTCATTTCT TTTTTCTTTTCTCTCTTCCA

In100 candidate926 (in intron):

LOCUS ENSG00000133105

DESCRIPTION Relaxin receptor 2 (Relaxin family peptide receptor 2) (Leucinerich repeat-containing G-protein coupled receptor 8) (G-protein coupled recptor affecting testicular descent) (G-protein coupled receptor 106). [Sourc:Uniprot/SWISSPROT;Acc:Q8WXD0]

AGTGTTAATAGAATGTGTCTGATGAAGGAAAGATAATTTTAACTAATTCTCCTTTTCTCTGATTCCAGTTTATTTTCTAT TCAATTGCTAGGTTTGTTTT

In100 candidate927 (in intron):

LOCUS ENSG00000170142

DESCRIPTION Ubiquitin-conjugating enzyme E2 E1 (EC 6.3.2.19) (Ubiquitinproein ligase E1) (Ubiquitin carrier protein E1) (UbcH6). [Source:Uniprot/SWISPROT;Acc:P51965]

AGTTAATTTTAACTATATAACACCATACCTTTTGGTCAATTTTTCAATTTTTGTCCCTTTTCTCCCATTTTTCCTCAGTC
TCCATATATTTGTCTCTTAG

In100 candidate928 (in intron):

LOCUS ENSG00000170142

DESCRIPTION Ubiquitin-conjugating enzyme E2 E1 (EC 6.3.2.19) (Ubiquitinproein ligase E1) (Ubiquitin carrier protein E1) (UbcH6). [Source:Uniprot/SWISPROT;Acc:P51965]

TACCTCCTTTTTTAAATAAACACAAATGGTAACTTGCTGTAAACACTGTTCTGTACCTTGGTTTTTTAACTTCAGATATC
TTGGACTTTCCCCAGTGTTT

In100 candidate929 (in intron):

LOCUS ENSG00000141968

DESCRIPTION Proto-oncogene vav. [Source:Uniprot/SWISSPROT;Acc:P15498]

GCCATCGTGCCTGGCCTGAAGGCTTTTATTTTTAACCAATTTTCATATATATGTTTTTTCTTTTTTTTTAACTTCCAGGA
ATTTCTAATTTTTTTAATTT

In100 candidate930 (in intron):

LOCUS ENSG00000206579

DESCRIPTION XK-related protein 4. [Source:Uniprot/SWISSPROT;Acc:Q5GH76]

GCTATCCTGATTGTCCCTCATATTATCTAACTAATTGTTTCCATCATTTACTTTAGTCAACTAGTTCTCTCTTTCAGTTG

ACTCCCTTTTTCTAGGTGCT

In100 candidate931 (in intron):

LOCUS ENSG00000206579

DESCRIPTION XK-related protein 4. [Source:Uniprot/SWISSPROT;Acc:Q5GH76]

ATTTTTGGTCATTGCCTCCTCCTGCCTTCTTATAACACCTAAACCACTGACTATCCCTCTTCTATTTTGCAGATTCGAGT

TCTCTCTTCCAATAAATTCT

In100 candidate932 (in intron):

LOCUS ENSG00000052126

DESCRIPTION Pleckstrin homology domain-containing family A member 5

(Phosphinositol 3-phosphate-binding protein 2) (PEPP-2).

[Source:Uniprot/SWISSPROTAcc:Q9HAU0]

ATTGTTACATATGTGAATACTTTTGTCAAAAGTCGTTGATGTATAACTAAAATTTGTCAATTTATTCTTCAGTGAAGTTT

TTTTTTTAATATCCTCACTT

In100 candidate933 (in intron):

LOCUS ENSG00000171427

DESCRIPTION

TCCGTCATATATATTGTGAATATGTTTCTTATTTTGTCGTTTGTCTTTTAACATTGTTGATGGTGTTTTTATTGCCAGAC

TTTTTTTTTATGTACATGGT

In100 candidate934 (in intron):

LOCUS ENSG00000137574

DESCRIPTION Putative RNA methyltransferase NCOA6IP (EC 2.1.1.-) (Nuclear reeptor coactivator 6-interacting protein) (PRIP-interacting protein with metyltransferase motif) (PIPMT) (PIMT) (CLL-associated antigen KW-2) (Hepatocelular carcinoma-associated antigen 137) [Source:Uniprot/SWISSPROT;Acc:Q96RS]

TATATATTTATATTTCCCTAACAAGGGGATAGGGTTTTCGTTTGTTTGTTTGTTTGTTTTACTATTTATTTATTGCAGTC

TACTATGTAATTTCTACTTT

In100 candidate935 (in intron):

LOCUS ENSG00000137574

DESCRIPTION Putative RNA methyltransferase NCOA6IP (EC 2.1.1.-) (Nuclear reeptor coactivator 6-interacting protein) (PRIP-interacting protein with metyltransferase motif) (PIPMT) (PIMT) (CLL-associated antigen KW-2) (Hepatocelular carcinoma-associated antigen 137) [Source:Uniprot/SWISSPROT;Acc:Q96RS]

AATTTCACTGTAATGTTGCTAACTGGGTTTTTCCTTTCTCTCTTATTTGATACTATCAGAACTAGCCCTAGTATTCTGAT

GTTTTTTGTGTTTTTTATTC

In100 candidate936 (in intron):

LOCUS ENSG00000137574

DESCRIPTION Putative RNA methyltransferase NCOA6IP (EC 2.1.1.-) (Nuclear reeptor coactivator 6-interacting protein) (PRIP-interacting protein with metyltransferase motif) (PIPMT) (PIMT) (CLL-associated antigen KW-2) (Hepatocelular carcinoma-associated antigen 137) [Source:Uniprot/SWISSPROT;Acc:Q96RS]

GAGACTCCATCTCAAAAAATAATAATAATAATAACTATATTTATAGTACCTTTATTTTTTTTTCAAGTAAACTTCAGAA
ATGGTTTTTCTTTTCTTTAA

In100 candidate937 (in intron):

LOCUS ENSG00000088808

DESCRIPTION Apoptosis-stimulating of p53 protein 1 (Protein phosphatase 1 rgulatory subunit 13B). [Source:Uniprot/SWISSPROT;Acc:Q96KQ4]

AGTCACCTAGGAGCATGGAGTTGGGGAGTAGATAGGGATTTAACTGCCTCTTAAATATTGTTCACTCAGTCTGCCTTATT
TTCAGTCCCTACATTTTATT

In100 candidate938 (in intron):

LOCUS ENSG00000156414

DESCRIPTION tudor domain containing 9 [Source:RefSeq_peptide;Acc:NP_694591]EXON

CTCCTCCTCTTTCCCTCTGAAGCACTGGTTATGTGAATCGGCTAACTGATTTAGTTATGTTATTATTTCTAATCTCAGGC
TTGTTTCTTTTCAGCGTTGT

In100 candidate939 (in intron):

LOCUS ENSG00000156414

DESCRIPTION tudor domain containing 9 [Source:RefSeq_peptide;Acc:NP_694591]EXON

AGCTCTTTTAGATAACGGGCTTAAAAGAAATGTAATCTGTTACCCTCCATTTTATTGTTTTTTTTTTTTGGTCAGGCTTT

GCTGTTCTTCTAAACTTATT

In100 candidate940 (in intron):

LOCUS ENSG00000151090

DESCRIPTION Thyroid hormone receptor beta-1.

[Source:Uniprot/SWISSPROT;Acc:10828]

AGATAATACTTTTATAACTTTTAAAGATTTTTGCTGTTGTTGTTTCTATTTGTTGTTGTCAGATTGTTTTCTCTAATCAA

TTCATCTATTCAGTGTTTCT

In100 candidate941 (in intron):

LOCUS ENSG00000151090

DESCRIPTION Thyroid hormone receptor beta-1.

[Source:Uniprot/SWISSPROT;Acc:10828]

AGCTCCTGGATTCGTTAACTTTCTGAAGGGTTTTTGTGTCTCTATTTCCTTCAGTTCTGCTCTGATTTTAGTTATTTCTT

GCCTTCTGCTAGCTTTTGAA

In100 candidate942 (in intron):

LOCUS ENSG00000151090

DESCRIPTION Thyroid hormone receptor beta-1.

[Source:Uniprot/SWISSPROT;Acc:10828]

ATGTATTTTCCTATAGATCTTAAAAAGATCTATTTCATATTCTATAACCTAAAATTAAGATATTTAGTTTCTTCTCAGAG

TTTTGCTTTTGTTTTGTTTT

In100 candidate943 (in intron):

LOCUS ENSG00000198920

DESCRIPTION Uncharacterized protein KIAA0753.

[Source:Uniprot/SWISSPROT;AccQ2KHM9]

AGTTCCCTGTAGATAACTAATCTCATTAGTCTCTGATTTTTGTTTTCTGTTTCTTTTTGCACAAGTGATTCAGATGTATA

TATATTTTCTCATGTCTTCT

In100 candidate944 (in intron):

LOCUS ENSG00000108590

DESCRIPTION Mediator of RNA polymerase II transcription subunit 31 (Mediato

complex subunit SOH1) (hSOH1). [Source:Uniprot/SWISSPROT;Acc:Q9Y3C7]

TCTCCGTTTGTGTTGCTGTTTTCTTTGATGATTTCTCTTTAACTTTAGAGAAACTTCTAACTTACTCCTTTTAATCAGGG

CTCCGCTTTTCTTAGATTTC

In100 candidate945 (in intron):

LOCUS ENSG00000077092

DESCRIPTION Retinoic acid receptor beta (RAR-beta) (RAR-epsilon) (HBVactivted

protein). [Source:Uniprot/SWISSPROT;Acc:P10826]

TATCATTGTGTTTTGTTTTCTGTAGTTGTAACCATTGGACCCTTTCCTTTCCTTATGGTGTGTTTGCTCTACCAGTGTTT

TTTATACTTTTGTGAGTTTT

In100 candidate946 (in intron):

LOCUS ENSG00000077092

DESCRIPTION Retinoic acid receptor beta (RAR-beta) (RAR-epsilon) (HBVactivted

protein). [Source:Uniprot/SWISSPROT;Acc:P10826]

TTTCTGAGTTTAAATTGTTTTCCTAACTCTACCACTTACTTCTTTGATCTGTCTTTATCACCTCTTGTTCAGTGTCCTCT

TGTTTTTAGAATAACTTTCT

In100 candidate947 (in intron):

LOCUS ENSG00000077092

DESCRIPTION Retinoic acid receptor beta (RAR-beta) (RAR-epsilon) (HBVactivted

protein). [Source:Uniprot/SWISSPROT;Acc:P10826]

ACATCGTGAACTCCTCTGTTTGTGGGTAACTGCCATCAACCTGTCTTTTTCAAGATACACTTCTTTCATCAGGAACTTCT

CTTTGTAAATTGTTATTTCC

In100 candidate948 (in intron):

LOCUS ENSG00000171105

DESCRIPTION Insulin receptor precursor (EC 2.7.10.1) (IR) (CD220 antigen)

[ontains: Insulin receptor subunit alpha; Insulin receptor subunit beta].

[Surce:Uniprot/SWISSPROT;Acc:P06213]

ATTTCATGCTCTCAACCCTGTATCTAGAACCCAAAATATAACTGGTGTATATAATGAGATTTTTTTTTTTCTCTCAGGC

CTCTCTTGTGTTAATTTCTG

In100 candidate949 (in intron):

LOCUS ENSG00000205754

DESCRIPTION liver-specific organic anion transporter 3TM12

[Source:RefSeq_pptide;Acc:NP_001009562]

ATAAAGAGGATAATGAAGATACAAAAATGGTACAATAGTAACATTGTCTATGTTGGGCTATGTTTCTTCTATCCCCAGTT

TTTTAAAGTTTTTTTTTAT

In100 candidate950 (in intron):

LOCUS ENSG00000134538

DESCRIPTION Solute carrier organic anion transporter family member 1B1 (Solte carrier family 21 member 6) (Sodium-independent organic anion- transportng polypeptide 2) (OATP 2) (Liver-specific organic anion transporter 1) (LS-1) (OATP-C). [Source:Uniprot/SWISSPROT;Acc:Q9Y6L6]

GCCTATTGACATTATATAGTCCTTCGATTAACCATTTTCCCCCTTTCCTTCTGATTTTTCTTAGATGTTCTTGGCAGCTC

TGTCACTCAGCTTTATTGCT

In100 candidate951 (in intron):

LOCUS ENSG00000134538

DESCRIPTION Solute carrier organic anion transporter family member 1B1 (Solte carrier family 21 member 6) (Sodium-independent organic anion- transportng polypeptide 2) (OATP 2) (Liver-specific organic anion transporter 1) (LS-1) (OATP-C). [Source:Uniprot/SWISSPROT;Acc:Q9Y6L6]

AGTATGTAGCATTTTTAGACTAGCTTATTTAACTTAATCACATGCATTTAAATTTCCTCCAGGTGTTTCAATTGCTTGAT

AGCTCATTTCTTTTTATTGC

In100 candidate952 (in intron):

LOCUS ENSG00000152932

DESCRIPTION Ras-related protein Rab-3C. [Source:Uniprot/SWISSPROT;Acc:Q96E1]

CCATGTAACCTGATGGGAAATGTCTGCTCATTTCTCACGGCCTCCGTTCAAAGTTTCTCTTACTGTTTTAGTTTCCAGTC

AGGGTTCTCTTGTTTTTTTC

In100 candidate953 (in intron):

LOCUS ENSG00000084453

DESCRIPTION Solute carrier organic anion transporter family member 1A2 (Solte carrier family 21 member 3) (Sodium-independent organic anion transporte) (Organic anion-transporting polypeptide 1) (OATP1) (OATP- A). [Source:Unirot/SWISSPROT;Acc:P46721]

TATTTATAACTTTTATGTCCTCTTGCTTAATTAATCCTTTTATCCTTATATAATGATCTTCTTCGTCTCTTCTTACAGTT

TTTTTCTTAAAATCTATTTT

In100 candidate954 (in intron):

LOCUS ENSG00000113448

DESCRIPTION cAMP-specific 3',5'-cyclic phosphodiesterase 4D (EC 3.1.4.17) (PDE3) (PDE43). [Source:Uniprot/SWISSPROT;Acc:Q08499]

ATACACTTGCTGTGATAACATTCTTTTTACTTTTGTAAATGCTTGAAATTTTCATATCTTTTTTTCCCCAGAACTTTTTC

TTTCCCCCAGAGCTTTTAAT

In100 candidate955 (in intron):

LOCUS ENSG00000113448

DESCRIPTION cAMP-specific 3',5'-cyclic phosphodiesterase 4D (EC 3.1.4.17)

(PDE3) (PDE43). [Source:Uniprot/SWISSPROT;Acc:Q08499]

GAAAAAGAAAAGGAAAATTTTCTAAAATTCCAAACCCCAAAATAACTGCTACTGACATTTTATTTTATATCCCTTCAGAT

TTATTTTTCTGTGCATATTT

In100 candidate956 (in intron):

LOCUS ENSG00000113448

DESCRIPTION cAMP-specific 3',5'-cyclic phosphodiesterase 4D (EC 3.1.4.17)

(PDE3) (PDE43). [Source:Uniprot/SWISSPROT;Acc:Q08499]

CTATGCATTCGTTAGAAAAAAGATCAAATACCTGTGCTTTTAACCTTTTTCTTTTCTTTTCTTTTCTTTTTGAAACAGAG

TCTTGCTCTGTTGCCTAGTT

In100 candidate957 (in intron):

LOCUS ENSG00000151092

DESCRIPTION Peptide-N(4)-(N-acetyl-beta-glucosaminyl)asparagine amidase

(EC3.5.1.52) (PNGase) (hPNGase) (Peptide:N-glycanase) (N-glycanase 1).

[SourceUniprot/SWISSPROT;Acc:Q96IV0]

GTTAATAGAATTATAGTATGTAATCTTTTGTTTCTAGCTCTTTGTGTAACATTTTTGAAGTTCTTCGTATTGCAGGATAT

ATCAGTATTTTGTTCCTTTT

In100 candidate958 (in intron):

LOCUS ENSG00000139618

DESCRIPTION Breast cancer type 2 susceptibility protein (Fanconi anemia grop

D1 protein). [Source:Uniprot/SWISSPROT;Acc:P51587]

CTGGGAATTAGTCACAATGTATCCTGTAACATCCTGGAACCTTTCCTTATGTCAGTCAGTCCTGGTCACCTGTTTGGTCC

CTGGCAGTTTTTTCTCATTT

In100 candidate959 (in intron):

LOCUS ENSG00000132463

DESCRIPTION G-rich sequence factor 1 (GRSF-1).

[Source:Uniprot/SWISSPROT;Ac:Q12849]

TGTAGTGAGCCATCAAATGCCCATAATTTAGATTAACAATTTTTTTTCGACTGCTCCTTTTTTTTTTTTTTGAGACAGAG

TTTCACTCTTTTTGCCTAGG

In100 candidate960 (in intron):

LOCUS ENSG00000198846

DESCRIPTION Thymus high mobility group box protein TOX.

[Source:Uniprot/SWISPROT;Acc:O94900]

TGCTGGATAATTAAAAAGACTGTATATCATGGCATTAACCACTAGTTTGATTTATTTGTCACCAGTGTTTCCTGACTCAG

TTTATTTTAGGGCTTTTTTT

In100 candidate961 (in intron):

LOCUS ENSG00000198846

DESCRIPTION Thymus high mobility group box protein TOX.

[Source:Uniprot/SWISPROT;Acc:O94900]

AACATTATTACTTTTTCACTTCCAACGTGTAACTTTAACCATATATCTTTTTTCCTCACAGTTTTCTTGTTTTTCTCTCT

TGTTTTGTGTGCCCTCTACC

In100 candidate962 (in intron):

LOCUS ENSG00000080493

DESCRIPTION Electrogenic sodium bicarbonate cotransporter 1 (Sodium bicarboate cotransporter) (Na(+)/HCO3(-) cotransporter) (Solute carrier family 4 mmber 4) (kNBC1). [Source:Uniprot/SWISSPROT;Acc:Q9Y6R1]

TTGTCTCAACTGGTTATGGTTAATTTTATTTGTGATTATCTATTAGAGGTAACACTGGTATTTATGCTCTAATTTCAGTC

ACTTGTTTGATGACCTTTCT

In100 candidate963 (in intron):

LOCUS ENSG00000080493

DESCRIPTION Electrogenic sodium bicarbonate cotransporter 1 (Sodium bicarboate cotransporter) (Na(+)/HCO3(-) cotransporter) (Solute carrier family 4 mmber 4) (kNBC1). [Source:Uniprot/SWISSPROT;Acc:Q9Y6R1]

GTGTCTGATAACCATAGTATATAATTCTCCTTTAAGGTTATTTCTAATTTCTGTTTTTACCCCCTCTTGGGATTCCAGTC

ATTTGATCTTGTCTTCTGTT

In100 candidate964 (in intron):

LOCUS ENSG00000080493

DESCRIPTION Electrogenic sodium bicarbonate cotransporter 1 (Sodium bicarboate cotransporter) (Na(+)/HCO3(-) cotransporter) (Solute carrier family 4 mmber 4) (kNBC1). [Source:Uniprot/SWISSPROT;Acc:Q9Y6R1]

GATAACTCCCAAATTCCTATTTTTGCCCTACTTTCATTTTTCCTTTTTATTTCTACTCTCATAATCCTATAGACTCAGTC

CTTTTATTTTATGCCTAGAC

In100 candidate965 (in intron):

LOCUS ENSG00000164181

DESCRIPTION ELOVL family member 7, elongation of long chain fatty acids

[Sorce:RefSeq_peptide;Acc:NP_079206]

AGGGTTTTTACAAACCACTGGATAACGTTCACTCTGAGTGTTACCTTCCTTCGCCTTCTCATTTTTTTTCTATGCAGTTT

TGCTTTCTTTTCCCTCATAT

In100 candidate966 (in intron):

LOCUS ENSG00000178741

DESCRIPTION Cytochrome c oxidase subunit 5A, mitochondrial precursor (EC

1..3.1) (Cytochrome c oxidase polypeptide Va).

[Source:Uniprot/SWISSPROT;Acc:20674]

ATAATATCTCCTTAATGTCCTCATTTAGGCTTCATTAACTCTACGCATTTCTCTGGCTGTTTTTAGGCTGTTTTCCAGTT

GAATCTCTTTTATTTTGGTT

In100 candidate967 (in intron):

LOCUS ENSG00000171316

DESCRIPTION Chromodomain-helicase-DNA-binding protein 7 (EC 3.6.1.-)

(ATPependent

helicase CHD7) (CHD-7) (Fragment). [Source:Uniprot/SWISSPROT;Acc:QP2D1]

TGAAAAGGGATTCATACCCACGCCTTATTGATAACTTTATTGAGGTGCTTTTTCATTCTTTTGTGTATTATTTGACAGAG

TGTTCTGTTTTTTTGCTCTG

In100 candidate968 (in intron):

LOCUS ENSG00000164182

DESCRIPTION Mimitin, mitochondrial precursor (Myc-induced mitochondrial proein) (MMTN) (B17.2-like) (B17.2L) (NDUFA12-like protein). [Source:Uniprot/SISSPROT;Acc:Q8N183]

GGCTATTTTAATCTGAAGTTTTCATATTTAATTTGTAACAAATTGAAAACACATTTTTTTCATTCATTTTGTTCTCAGCT
ACTTATAATCTTCCAATCTT

In100 candidate969 (in intron):

LOCUS ENSG00000164182

DESCRIPTION Mimitin, mitochondrial precursor (Myc-induced mitochondrial proein) (MMTN) (B17.2-like) (B17.2L) (NDUFA12-like protein). [Source:Uniprot/SISSPROT;Acc:Q8N183]

TATTTAACTTTCTAATGTGTTTACCAATATCTTTTCTCGTAACATATTCTTGCATTCCATTCTCCTTTCTATGCTCAGTT
TTCCTTCTTTACACATATAT

In100 candidate970 (in intron):

LOCUS ENSG00000164182

DESCRIPTION Mimitin, mitochondrial precursor (Myc-induced mitochondrial proein) (MMTN) (B17.2-like) (B17.2L) (NDUFA12-like protein). [Source:Uniprot/SISSPROT;Acc:Q8N183]

AACTCCATAACATTTGAGGTCATGTTTCTTAATTGATGCTCTCTTTGCAGCTATAACTTTTTTTTTCATTTCATTTTAT
ATGCTTGCTGTCATTTGCCC

In100 candidate971 (in intron):

LOCUS ENSG00000198363

DESCRIPTION Aspartyl/asparaginyl beta-hydroxylase (EC 1.14.11.16) (Aspartat beta- hydroxylase) (ASP beta-hydroxylase) (Peptide-aspartate beta- dioxygease).

[Source:Uniprot/SWISSPROT;Acc:Q12797]

ACTTTTATTTATGATACCTTTATAGTCTTAGAATAACTATAGAAAGGTTTTAATTATTTCAGATTAGAATGTACTTTCAT

TTTATATCACACTTTTTTTC

In100 candidate972 (in intron):

LOCUS ENSG00000139617

DESCRIPTION phosphonoformate immuno-associated protein 5

[Source:RefSeq_pepide;Acc:NP_055702]

TGTTTTCATTCTGTTGAACTTAGTGGTTTGAATTCTAACTCCTGAGAAAATTTCCACATCTTTGCATTTTGCTTCAGGCT

TCTTTTCCCCATAGTGTAAT

In100 candidate973 (in intron):

LOCUS ENSG00000139617

DESCRIPTION phosphonoformate immuno-associated protein 5

[Source:RefSeq_pepide;Acc:NP_055702]

TTCTGCTGATAAATCCACTGTTAATCTGATAGGAGTTCCCTTATATGTAACTAGATGTTTTTTTCTTGCCATTTTCAGAA

TTGTCTCTTTGACTTTTGAC

In100 candidate974 (in intron):

LOCUS ENSG00000163512

DESCRIPTION 5-azacytidine induced 2 [Source:RefSeq_peptide;Acc:NP_071906]

GTTTTTAAACATTGTCCTAGTATTTACATTATTCTTAACTATAACTTTTTTTCCTTTTCACTTATTCAGTGAGACATTTG

TCACTCATATTTTTTTTCTT

In100 candidate975 (in intron):

LOCUS ENSG00000163512

DESCRIPTION 5-azacytidine induced 2 [Source:RefSeq_peptide;Acc:NP_071906]

GGTAAATATTTGTAGGAGAGGATTTAACAACTTGGAGTTATATTCCAAATATTTTTCTTTCATTTTCAGGTTTACTTACC

TCCATAGCTGTATGTATTTT

In100 candidate976 (in intron):

LOCUS ENSG00000163512

DESCRIPTION 5-azacytidine induced 2 [Source:RefSeq_peptide;Acc:NP_071906]

TTTTTATGTTAACCTCATTCTCCACTGCATTTTGTTTTGTTTTCTATTTTTCGCCTCATCATTTCTGCCTGTTTGCAGTT

TATGTTCTTGTGTTTTGTTA

In100 candidate977 (in intron):

LOCUS ENSG00000156140

DESCRIPTION ADAMTS-3 precursor (EC 3.4.24.-) (A disintegrin and

metalloprotinase with thrombospondin motifs 3) (ADAM-TS 3) (ADAM-TS3)

(Procollagen II mino propeptide-processing enzyme) (Procollagen II N-proteinase)

(PC II-NP) [Source:Uniprot/SWISSPROT;Acc:O15072]

GTAATTAACTACCAAATATTCTTCTCATGATTCCACTCTATTTCTTTCTACTCTATGTTGACTTTTTATATAACTCAGAT

CATAATATTTCATATTTTTT

In100 candidate978 (in intron):

LOCUS ENSG00000083642

DESCRIPTION androgen-induced prostate proliferative shutoff associated protin

[Source:RefSeq_peptide;Acc:NP_055847]

AATTAAAAATAGGAGTAAGATACTGAGATAATAACCCCACTTCTCTCTTCTGTTTTTCTTTTTCATTCACACATACAGAC

ACATTCTTTTTTTTTTTAT

In100 candidate979 (in intron):

LOCUS ENSG00000083642

DESCRIPTION androgen-induced prostate proliferative shutoff associated protin

[Source:RefSeq_peptide;Acc:NP_055847]

ATTCTCTCATAACTAATTCTTCTTTTTATGTTTTTGATTCCATTTCATCCTTCCTGCTACAACACCTTACGCTGTCAGTT

ATTCCTTTATTTCTCAAATT

In100 candidate980 (in intron):

LOCUS ENSG00000205625

DESCRIPTION

ATAGTTGATTAACAAAACTTGAAAATTTTCTAGCTGTTCCTTGCCTGCTATTTGTTTTTCCTTTTCTTGTGCTATCAGCC

AGACCTTGTGGTTCTTTTTT

In100 candidate981 (in intron):

LOCUS ENSG00000206559

DESCRIPTION zinc finger, CW type with PWWP domain 2

[Source:RefSeq_peptide;cc:NP_001035522]

GTTATACCCCCTTTTAACATTTTTTATTGTTTCTATTTGATTCTTCTCTCTTTTCTTCTTTATTAGTCTCGCTAGCAGTC

TATCAATTTTGTTGATCTTT

In100 candidate982 (in intron):

LOCUS ENSG00000144642

DESCRIPTION RNA-binding motif, single-stranded-interacting protein 3.

[Soure:Uniprot/SWISSPROT;Acc:Q6XE24]

ACTCTTTCTCTACCTCATGTTTTAAGGCAAATAACTCTTATATTTGCCCTTTTGATACTACTTTCTAGATCTTGTCAGCA

TGCTTCATTGCTTTCTACTC

In100 candidate983 (in intron):

LOCUS ENSG00000144642

DESCRIPTION RNA-binding motif, single-stranded-interacting protein 3.

[Soure:Uniprot/SWISSPROT;Acc:Q6XE24]

TTTTTTAACGGACTTTATTCTCCTTTAATTTTTTATGTTCTCCTTGAAAACCTTAAGCTTTTTACCCAGAGTGCTTAATT

ATTCATTCACTCACTCATTC

In100 candidate984 (in intron):

LOCUS ENSG00000144642

DESCRIPTION RNA-binding motif, single-stranded-interacting protein 3.

[Soure:Uniprot/SWISSPROT;Acc:Q6XE24]

AGTAACTCACATGGCATGCTCTTAGCATCATCTAGCACAAGCTTTGTTTTCTTCTGTTCAACTTCCTTTCAGAGGTGCAT

TTTTACAACTTCCCTCTATT

In100 candidate985 (in intron):

LOCUS ENSG00000132466

DESCRIPTION ankyrin repeat domain protein 17 isoform b

[Source:RefSeq_peptie;Acc:NP_942592]

AGTTACACGTTTTTAGATATTTTGATATTGTCCTAAAAATAACATTGCCTCTGTACATCTTTTTTCAGCTGTTTTTCTCT

TTATTGTTTAGTTTTGCCAT

In100 candidate986 (in intron):

LOCUS ENSG00000086200

DESCRIPTION Importin-11 (Imp11) (Ran-binding protein 11) (RanBP11).

[SourceUniprot/SWISSPROT;Acc:Q9UI26]

CGATTATGAAGAAAAGAAACAAACTAACACCAAGCACTCTTCATCATTCCCCCACATTTTGATTTTTTGATGTCTCAGTT

TATATCTTTTTATATTGCCA

In100 candidate987 (in intron):

LOCUS ENSG00000111728

DESCRIPTION Alpha-N-acetylneuraminide alpha-2,8-sialyltransferase (EC

2.4.9.8) (Ganglioside GD3 synthase) (Ganglioside GT3 synthase) (Alpha-2,8-

sialytransferase 8A) (ST8Sia I). [Source:Uniprot/SWISSPROT;Acc:Q92185]

CAAAATGCACATTGCAATTCTCTGCTTTCTCTAACACTGTCTTTTTTCTTCCTATTTGTTATTTATTCATTCATTCAGAT

TTATTCATTTATTTTAATTA

In100 candidate988 (in intron):

LOCUS ENSG00000205485

DESCRIPTION

GCAAACCTACACATGTATCCCTGAACCTAAAATATAACTTAAAAATAAATATTTTCTTTTCATTTTTTGTATTACCAGTT

TAGTTTTCTTTTTTACCTCT

In100 candidate989 (in intron):

LOCUS ENSG00000172817

DESCRIPTION Cytochrome P450 7B1 (EC 1.14.13.100) (25-hydroxycholesterol 7-

apha- hydroxylase) (Oxysterol 7-alpha-hydroxylase).

[Source:Uniprot/SWISSPRO;Acc:O75881]

TATATAAGGATCCCTTTTACTCCATTTTCCAATAACACATTCCTTAGTTCTTCATTTCATGTGAGCCTTCACCAGCAGTG

CCTTTAACATTTCCTATTTC

In100 candidate990 (in intron):

LOCUS ENSG00000172817

DESCRIPTION Cytochrome P450 7B1 (EC 1.14.13.100) (25-hydroxycholesterol 7-

apha- hydroxylase) (Oxysterol 7-alpha-hydroxylase).

[Source:Uniprot/SWISSPRO;Acc:O75881]

TTGGTGATTTTCTGGACTCTATGTTATATCATGGCTTGTGGAAGCTTAACCCTGAACTCTGCTTTTATCTTCACACAGAA

TTCTCCCTGTGTTTGTGTCT

In100 candidate991 (in intron):

LOCUS ENSG00000172817

DESCRIPTION Cytochrome P450 7B1 (EC 1.14.13.100) (25-hydroxycholesterol 7-

apha- hydroxylase) (Oxysterol 7-alpha-hydroxylase).

[Source:Uniprot/SWISSPRO;Acc:O75881]

GGTAACATTCTCTTCATCATTTCTCATTGTTTTTATTTGAATCTTCTGTCTTTTCTTCTTTATTAGTCTAGGTAGCAGTA

TATCTATCTTATTAATTTTT

In100 candidate992 (in intron):

LOCUS ENSG00000172817

DESCRIPTION Cytochrome P450 7B1 (EC 1.14.13.100) (25-hydroxycholesterol 7-apha- hydroxylase) (Oxysterol 7-alpha-hydroxylase). [Source:Uniprot/SWISSPRO;Acc:O75881]

CCTCTTCTTAACTAAAGAGAAATGTCTTAAACCTCTTTACACTTTACATCCATTTTATGTATTTAGATTCTTCTCCAGTATTTCATTTTATTTATTTCAT

In100 candidate993 (in intron):

LOCUS ENSG00000134532

DESCRIPTION Transcription factor SOX-5. [Source:Uniprot/SWISSPROT;Acc:P3571]

TATTATATACTTACAAATTAGTGATTCTTTAACTTTTAGATATGCTTAAGTTCCTATTTGTTCAACTTCCAGAAAGTCTTTCTTTTAAGATTTTATCCTC

In100 candidate994 (in intron):

LOCUS ENSG00000134532

DESCRIPTION Transcription factor SOX-5. [Source:Uniprot/SWISSPROT;Acc:P3571]

AGTAGATAACATTTATTGAGCATATGTTTTTTTTTCAGGCATTGCGCTTTACATGTTTTATCTCTTTTAGCACTTTTAATCCTTGCAACTACTCTATTTA

In100 candidate995 (in intron):

LOCUS ENSG00000066855

DESCRIPTION Mitochondrial fission regulator 1 (Chondrocyte protein with a ply- proline region). [Source:Uniprot/SWISSPROT;Acc:Q15390]

AGAGTAACTTTGCTTCTTAACATTCTTTGTATACTATTCATATTTTAATATTTTTATCTTCCTTTCTACTTACCAGTTTT
CTAATCTTTGCCATCCTTTT

In100 candidate996 (in intron):

LOCUS ENSG00000172915

DESCRIPTION Protein neurobeachin (Lysosomal-trafficking regulator 2) (Proten BCL8B). [Source:Uniprot/SWISSPROT;Acc:Q8NFP9]

ATTTTTAAATGCTAGTAAAATGTTAGTAATGTCCCCTTACATTAACCTTAACTCTGCTTTCCTATAACTGATCTTCAGTG
GTCCCAGTTCTTTTTCTCTA

In100 candidate997 (in intron):

LOCUS ENSG00000172915

DESCRIPTION Protein neurobeachin (Lysosomal-trafficking regulator 2) (Proten BCL8B). [Source:Uniprot/SWISSPROT;Acc:Q8NFP9]

ATCCTGTTTTGGTACTTCCATTAACAATTATTTAGATAATTTTCATTGCCTCTGTCTTGTTTCAGTCTACCATTTCTTAG
ATATGGGTCTATCTCTTTTT

In100 candidate998 (in intron):

LOCUS ENSG00000172915

DESCRIPTION Protein neurobeachin (Lysosomal-trafficking regulator 2) (Proten BCL8B). [Source:Uniprot/SWISSPROT;Acc:Q8NFP9]

GGAGACCTGTTGTGTGCACCACTAACCCCATCCCATCTCTTTTGTTGTTGGCTCTGCTTCCTCAGAACTTCTGCTGCCTT

ATGATTTCTGTTGACTGCTT

In100 candidate999 (in intron):

LOCUS ENSG00000172915

DESCRIPTION Protein neurobeachin (Lysosomal-trafficking regulator 2) (Proten BCL8B). [Source:Uniprot/SWISSPROT;Acc:Q8NFP9]

CTTGAAGATGACTTGTAAAATTGATGGCTAACTTATCCTGCGGTTTTCTAAACTCTGTTTTTTTCTTCTTACCACAGCC

TCAGTTATGTTTAATTTCCC

In100 candidate1000 (in intron):

LOCUS ENSG00000205268

DESCRIPTION High-affinity cAMP-specific 3',5'-cyclic phosphodiesterase 7A (C 3.1.4.17) (HCP1) (TM22). [Source:Uniprot/SWISSPROT;Acc:Q13946]

TTAATGAAGTCAAGCATTTTTTTATATTTTTTTTAACCTTGTGAATAGTATATTTATGTCCTACCTGTTTTTCTACAGCT

TTTTAGTCTTTTTCTAATTG

In100 candidate1001 (in intron):

LOCUS ENSG00000135205

DESCRIPTION CDNA FLJ31980 fis, clone NT2RP7008487, weakly similar to TRICHOYALIN. [Source:Uniprot/SPTREMBL;Acc:Q96MS1]

CTTTTAGGATTACTCTAAAATTTATGGGTCTCACAAGTTTTTAACATACTGTGTATAACCTACTTTATATTCCTTCAGTT

GTCTTTGTCTTTTAACCTAC

In100 candidate1002 (in intron):

LOCUS ENSG00000135205

DESCRIPTION CDNA FLJ31980 fis, clone NT2RP7008487, weakly similar to

TRICHOYALIN. [Source:Uniprot/SPTREMBL;Acc:Q96MS1]

TCTCTCTTTTTATGCCTTTTGTGGTTTTAACTAAGCATTTAATATAATTCCATTTTCTCTCCTTTCTTAGTATATCAGTT

GTACATCTTTTTTTACTTTA

In100 candidate1003 (in intron):

LOCUS ENSG00000186479

DESCRIPTION R7 binding protein [Source:RefSeq_peptide;Acc:NP_001025046]

TTAAAGATTGGGGAAATTCACATATCTCAATAAACCTAACATGATTTTGTCCCCCAAATTTTTATTTATTTTCATCAGAT

TTATCGTTTTTCTAGCCTGT

In100 candidate1004 (in intron):

LOCUS ENSG00000153006

DESCRIPTION P18SRP protein [Source:RefSeq_peptide;Acc:NP_776190]

GTATTCTCTTATATAGTTATATATTTATATAACACCCCTTTATATTTCTGTGACATCACTTATACTCTCTTTTTTCAGTT

CTAATTTTGGCCCTGTCTTT

In100 candidate1005 (in intron):

LOCUS ENSG00000186088

DESCRIPTION

TAATGACCTCTGACTCTTTCTGCTTCTGCTTAACCTTCACATGTCTTGTTTCTCATTATTTCTTAGTGATCCAGACAAGG

AACCCCTTTCCTTATTTTTT

In100 candidate1006 (in intron):

LOCUS ENSG00000140367

DESCRIPTION Ubiquitin-conjugating enzyme E2 Q2 (EC 6.3.2.19) (Ubiquitinproein ligase Q2) (Ubiquitin carrier protein Q2).

[Source:Uniprot/SWISSPROT;Ac:Q8WVN8]

AACTAAGATTAACTGAAATTAACATTTCAATGTAAGTTCTTCTGGTCTTTCTTTTTCTGTGCTTATTTCAGCGTAACTGA

AATTCTTTCTATTCTTTTTT

In100 candidate1007 (in intron):

LOCUS ENSG00000187257

DESCRIPTION round spermatid basic protein 1-like

[Source:RefSeq_peptide;AccNP_940869]

TTGACATTTAATATGTCCTTAGATATTTTTTAAACTGTAAAATTAACGTGTTCAAAACTTTTAGTCTTCTTTATCCAGAT

TTGTCTTGCTATGTATGTTC

In100 candidate1008 (in intron):

LOCUS ENSG00000187257

DESCRIPTION round spermatid basic protein 1-like

[Source:RefSeq_peptide;AccNP_940869]

AAATAAGTTGGTTTTGTTTTTTTTTTTTTCTAAAGGAATCTCTTAACTGATTTCTTATTGGTTTGCATTTTTCAGTTCCT

GTTATAGTACCTTTTGGTTT

In100 candidate1009 (in intron):

LOCUS ENSG00000182973

DESCRIPTION CCR4-NOT transcription complex, subunit 10

[Source:RefSeq_peptie;Acc:NP_056257]

ACTGCCCTAAGAATTCTGTGTTGTGCCTGTTCATTCCCTTTCTACCTATAACCACTGATCTTTTTACTGTCTCTACAGTT

TTGCCTTTTCTACAGTGTCA

In100 candidate1010 (in intron):

LOCUS ENSG00000049192

DESCRIPTION ADAMTS-6 precursor (EC 3.4.24.-) (A disintegrin and

metalloprotinase with thrombospondin motifs 6) (ADAM-TS 6) (ADAM-TS6).

[Source:UniprotSWISSPROT;Acc:Q9UKP5]

CTGATTCATAACCATGGGATATCTTTCCATTGGTTTGTGTCCTCTTCAGTTTCTTTCATCAGTGTTTTGTAGTTGTCCTT

GTAGAGGTTTTTCACCTCCT

In100 candidate1011 (in intron):

LOCUS ENSG00000049192

DESCRIPTION ADAMTS-6 precursor (EC 3.4.24.-) (A disintegrin and

metalloprotinase with thrombospondin motifs 6) (ADAM-TS 6) (ADAM-TS6).

[Source:UniprotSWISSPROT;Acc:Q9UKP5]

CGAGACTCCGTCTCCAAAAAGGAAAAAAAAAATGTTTCTAACTATAATAGTGGATTTGTGTATTTCTCTTTGCAGTTCTA

TAAGTTTTTGACTCACTTTT

In100 candidate1012 (in intron):

LOCUS ENSG00000049192

DESCRIPTION ADAMTS-6 precursor (EC 3.4.24.-) (A disintegrin and

metalloprotinase with thrombospondin motifs 6) (ADAM-TS 6) (ADAM-TS6).

[Source:UniprotSWISSPROT;Acc:Q9UKP5]

AGGGAGGGTAGTAAAAAGCCTCAAAACAATCTCTGAGTGTGCCTGTAACATACTTGTTATTTATTCCCAATTCAGAATTT

TCTCTAGTGACATATTCTTT

In100 candidate1013 (in intron):

LOCUS ENSG00000187391

DESCRIPTION Membrane-associated guanylate kinase, WW and PDZ domain-containng protein 2 (Membrane-associated guanylate kinase inverted 2) (MAGI-2) (Atophin-1-interacting protein 1) (Atrophin-1-interacting protein A).

[Source:niprot/SWISSPROT;Acc:Q86UL8]

CTATTTTTAACTTCATTGATTTCTGCTCTTATCTTTACTATTTCCTTGTTTCTACTTGCTTTGGACTTATTTTGCCAGAT

TTTTTCCTAGAAATTTTACT

In100 candidate1014 (in intron):

LOCUS ENSG00000187391

DESCRIPTION Membrane-associated guanylate kinase, WW and PDZ domain-containng protein 2 (Membrane-associated guanylate kinase inverted 2) (MAGI-2) (Atophin-1-interacting protein 1) (Atrophin-1-interacting protein A).

[Source:niprot/SWISSPROT;Acc:Q86UL8]

ATATTTACTTCAAAATAATATTGGAATATCTCTTCAATAACTTGATCCATTTCCCTGATACTGTTTTTTTTCCTACAGTT

TTTACTATCTCACTCAACTT

In100 candidate1015 (in intron):

LOCUS ENSG00000187391

DESCRIPTION Membrane-associated guanylate kinase, WW and PDZ domain-containng protein 2 (Membrane-associated guanylate kinase inverted 2) (MAGI-2) (Atophin-1-interacting protein 1) (Atrophin-1-interacting protein A).

[Source:niprot/SWISSPROT;Acc:Q86UL8]

CATAGAGACTTTCATAACTTCATTTTAAAATCACTCCTCTCTTTTTTAATTTATGACTATGTCTCTCCGTTCCTTCAGGT
TTGTTCATTTTAAATGTTCT

In100 candidate1016 (in intron):

LOCUS ENSG00000187391

DESCRIPTION Membrane-associated guanylate kinase, WW and PDZ domain-containng protein 2 (Membrane-associated guanylate kinase inverted 2) (MAGI-2) (Atophin-1-interacting protein 1) (Atrophin-1-interacting protein A).

[Source:niprot/SWISSPROT;Acc:Q86UL8]

GCCAACAAAATTAACCCTTGACATTTTTTACTGATTTCCCAGTATTTGTGTTCATTAAGAAATATTTTACTTTCTCCCGC
ATTATCTTTTCAACTTTTTC

In100 candidate1017 (in intron):

LOCUS ENSG00000187391

DESCRIPTION Membrane-associated guanylate kinase, WW and PDZ domain-containng protein 2 (Membrane-associated guanylate kinase inverted 2) (MAGI-2) (Atophin-1-interacting protein 1) (Atrophin-1-interacting protein A).

[Source:niprot/SWISSPROT;Acc:Q86UL8]

GCCTTTGTAACCCAAATGCTATTGTGGTGCCTCTTTATTCTCATTTCTGTCTTTTCTTTTTGCCTTGCACAGTCCTCTTT

ATTCCCTCTTCATTTAAAAT

In100 candidate1018 (in intron):

LOCUS ENSG00000187391

DESCRIPTION Membrane-associated guanylate kinase, WW and PDZ domain-containng protein 2 (Membrane-associated guanylate kinase inverted 2) (MAGI-2) (Atophin-1-interacting protein 1) (Atrophin-1-interacting protein A). [Source:niprot/SWISSPROT;Acc:Q86UL8]

CTGAAGGCACTAACTGTGTCTTGTTCTTCTTTACTTCTCCAGTGCCTTGCACAAGCCTGTATAAAAATTAGTAGTTTCTT
TTTTCTTTTTTTTTTTTTTT

In100 candidate1019 (in intron):

LOCUS ENSG00000110619

DESCRIPTION Cysteinyl-tRNA synthetase, cytoplasmic (EC 6.1.1.16) (CysteinetRNA ligase) (CysRS). [Source:Uniprot/SWISSPROT;Acc:P49589]

CCTTCTCCGTGTCTCCTAACCTGTCTTTCATAAAATTTTCCTTCTTGTCTCTGTTTCATTTTGGATGACTTCTTCAGTTC
TGTCTTCCTGTTCACAAATT

In100 candidate1020 (in intron):

LOCUS ENSG00000140374

DESCRIPTION Electron transfer flavoprotein subunit alpha, mitochondrial preursor (Alpha-ETF). [Source:Uniprot/SWISSPROT;Acc:P13804]

GATAGAGTAAGGTAAATTATTAGCATCTGTAAACCTAAGAGAAAGATAACTTTTTCATATATTTATTTCACACAGTATTT

TTTAGAGTATATTTCTCTTT

In100 candidate1021 (in intron):

LOCUS ENSG00000123219

DESCRIPTION Centromere protein K (CENP-K) (Interphase centromere complex prtein 37) (Protein AF-5alpha) (p33). [Source:Uniprot/SWISSPROT;Acc:Q9BS16]

TAAAAGATAAGAAAATTAACATTCTTTTTCCTATACCCTTATTTCTATTTTGTGTTATTTTTACTTTGGCAGGTTATATT

TTATTCAGAACTTCTATTTT

In100 candidate1022 (in intron):

LOCUS ENSG00000104205

DESCRIPTION Serine/threonine-protein kinase Sgk3 (EC 2.7.11.1) (Serum/glucoorticoid-regulated kinase 3) (Serum/glucocorticoid- regulated kinase-like).[Source:Uniprot/SWISSPROT;Acc:Q96BR1]

GTGCTTTTAATGTAACTTAAAACGAAACCTATTCATAGGGTCTATTCTCTTCTCAGAAATTGTCCCCATCCCAATTCCTT

TTCTTATTTACTAGGTCATT

In100 candidate1023 (in intron):

LOCUS ENSG00000152936

DESCRIPTION intermediate filament tail domain containing 1 [Source:RefSeq_pptide;Acc:NP_689803]

AATAACACTGTTCCTGTTGTTATTGGTACGTAATCATTCTTTTGCTCTCTTCTTGCTCTTCCCTTTCAGTGTGATTTGGC

TTTTCTTAAACTTTTTACTC

In100 candidate1024 (in intron):

LOCUS ENSG00000140386

DESCRIPTION Zinc finger protein 291. [Source:Uniprot/SWISSPROT;Acc:Q9BY12]

EXON

GCCATGAATATGTGAGTTTTCTTTCTAACTTGATTCTGTTTACTTTATCTCTTGTGCCTGTTTTTATACCAGTACCACTC

TATCTTGATTGTTGTACCTT

In100 candidate1025 (in intron):

LOCUS ENSG00000140386

DESCRIPTION Zinc finger protein 291. [Source:Uniprot/SWISSPROT;Acc:Q9BY12]

EXON

AGTTAACTATTAGAAATGTTCAAATCTCTCTTTTGTGTTCCCTGAACAGAAATTGCTTTGTTATTTTTTTTCTGCTATCT

TTTACTGAGCCCTTACTATG

In100 candidate1026 (in intron):

LOCUS ENSG00000133083

DESCRIPTION Serine/threonine-protein kinase DCAMKL1 (EC 2.7.11.1)

(Doublecotin- like and CAM kinase-like 1). [Source:Uniprot/SWISSPROT;Acc:O15075]

CTTCTTAAGATAACATGAATTTATTAATTTTCTTGTTGTATTTTCACTTTATATTCTATTTCTTTTCCCCCACCCCAGCC

CTTTACCCTTTCATTTATTC

In100 candidate1027 (in intron):

LOCUS ENSG00000133083

DESCRIPTION Serine/threonine-protein kinase DCAMKL1 (EC 2.7.11.1)

(Doublecotin- like and CAM kinase-like 1). [Source:Uniprot/SWISSPROT;Acc:O15075]

AATTTTCTACTTAGAAAATCAAGGCATTGTATAACATCTGCAATCTTTTCTTTACATTTTCTCTTCCGGTCCTCACAGGC

CTTCCTTTTTATTGTATTCA

In100 candidate1028 (in intron):

LOCUS ENSG00000123104

DESCRIPTION Inositol 1,4,5-trisphosphate receptor type 2 (Type 2 inositol

14,5- trisphosphate receptor) (Type 2 InsP3 receptor) (IP3 receptor isoform)

(InsP3R2). [Source:Uniprot/SWISSPROT;Acc:Q14571]

AGTAACCTCTGCATACACAATTCTTTCTTCAGCTGAGGAAAATTTTATCTTCTATTATCTTTGATTATTGTTTGTGTTCC

ACTTATTCTCTCTTCCATTT

In100 candidate1029 (in intron):

LOCUS ENSG00000123104

DESCRIPTION Inositol 1,4,5-trisphosphate receptor type 2 (Type 2 inositol

14,5- trisphosphate receptor) (Type 2 InsP3 receptor) (IP3 receptor isoform)

(InsP3R2). [Source:Uniprot/SWISSPROT;Acc:Q14571]

AGTAACATTCATGAAGAGAATTTTGTTACACTTCCTTGCTTCATTTTTTTTTCCTTGTGGAAATTCTCTCTCCAGAAGTG

AATATCTTTTTTTTTTCATT

In100 candidate1030 (in intron):

LOCUS ENSG00000123104

DESCRIPTION Inositol 1,4,5-trisphosphate receptor type 2 (Type 2 inositol

14,5- trisphosphate receptor) (Type 2 InsP3 receptor) (IP3 receptor isoform)

(InsP3R2). [Source:Uniprot/SWISSPROT;Acc:Q14571]

AGTCTTTTCTAACATTCTCTGCCCACCACTCCCACTTCTTTTTCTTTTTATCTCTATGTTTTTCTGTCTTTTCACCAGCC

AACTTTTTTTCATGTGTTTT

In100 candidate1031 (in intron):

LOCUS ENSG00000123104

DESCRIPTION Inositol 1,4,5-trisphosphate receptor type 2 (Type 2 inositol

14,5- trisphosphate receptor) (Type 2 InsP3 receptor) (IP3 receptor isoform)

(InsP3R2). [Source:Uniprot/SWISSPROT;Acc:Q14571]

TAATCCATGGATGATAACATCAAAATTATTGGGAAGGGTTTGATTTATCTTCTCCTACCTTTCTGTTTTCCTCTCCAGAT

GCTTTCTGTGTTTGTGTCCT

In100 candidate1032 (in intron):

LOCUS ENSG00000123104

DESCRIPTION Inositol 1,4,5-trisphosphate receptor type 2 (Type 2 inositol

14,5- trisphosphate receptor) (Type 2 InsP3 receptor) (IP3 receptor isoform)

(InsP3R2). [Source:Uniprot/SWISSPROT;Acc:Q14571]

ATTATATATTTTAAAACTATTTGTTAACTTCCATTTCTTTTGTTTCCTTCTTCAGATCTCCAATTATGTATATGCTGAGT

CTTCCATTCACTGTCTTTTT

In100 candidate1033 (in intron):

LOCUS ENSG00000123104

DESCRIPTION Inositol 1,4,5-trisphosphate receptor type 2 (Type 2 inositol 14,5- trisphosphate receptor) (Type 2 InsP3 receptor) (IP3 receptor isoform) (InsP3R2). [Source:Uniprot/SWISSPROT;Acc:Q14571]

TAACCCACACCCTGTATTTTTTTTTCTATTTTTCTCTTTATGTGACAGCTATCACCACCTCCATTTCTTCACAATAGATG

TATGTGTGTCTCCTTTCTTT

In100 candidate1034 (in intron):

LOCUS ENSG00000163539

DESCRIPTION CLIP-associating protein 2 (Cytoplasmic linker-associated proten 2) (hOrbit2). [Source:Uniprot/SWISSPROT;Acc:O75122]

TGATGTTATTTATTTCTGTAGCTCTTTTATTTTCTGTAAGTTTATTAACCTATATATTCACTTCCTTTTAGTCAGCTTAC

CTACGTATTTAAGTTTTCTC

In100 candidate1035 (in intron):

LOCUS ENSG00000163539

DESCRIPTION CLIP-associating protein 2 (Cytoplasmic linker-associated proten 2) (hOrbit2). [Source:Uniprot/SWISSPROT;Acc:O75122]

AGTATTATTTCCATGACTTAAAAATACACATAAATGTTATATTGTACATAACATTCTACAACCTGACTTTTTACTCAGTG

CTTTTTTTTTATATTTTTTC

In100 candidate1036 (in intron):

LOCUS ENSG00000163539

DESCRIPTION CLIP-associating protein 2 (Cytoplasmic linker-associated proten 2) (hOrbit2). [Source:Uniprot/SWISSPROT;Acc:O75122]

ATTTATTCTGTGCCTGGTATTTGGCCCATGAGCCTCTGAAGTTTTCATCTAACTAATTCACTTCTTTTTGTTTTCCAGAC

ATTATTTTAAATTCTCTACT

In100 candidate1037 (in intron):

LOCUS ENSG00000163539

DESCRIPTION CLIP-associating protein 2 (Cytoplasmic linker-associated proten 2) (hOrbit2). [Source:Uniprot/SWISSPROT;Acc:O75122]

TCCTATCCATGAGCTGGGATGTTTTAACATTTGTTTGTGTCTTCTCTGATTTCTTTGAGCAGTGTTTTGTAATTCTCATT

GTAGAGCTCTTTCACTTCTC

In100 candidate1038 (in intron):

LOCUS ENSG00000104218

DESCRIPTION centrosome spindle pole associated protein 1 isoform a [Source:efSeq_peptide;Acc:NP_001070672]

AAATGCCATAAAAATTGTAGGGATATTTTAATAGAGAATAAGGTGGCTAACTTACCAAACTATAGTTTGTCTTTTCAGCT

TAATTTCCCTTCAAGGTTTC

In100 candidate1039 (in intron):

LOCUS ENSG00000104218

DESCRIPTION centrosome spindle pole associated protein 1 isoform a [Source:efSeq_peptide;Acc:NP_001070672]

GATCTTTGCTCCATAACTTCTAGCCATTTTAGCCTTCCTGAATTTTGCTCTTTATCTACTCAATTTAGCTCAGTTTCCTG

GCCCTCTTTGGGTTCTCTTT

In100 candidate1040 (in intron):

LOCUS ENSG00000104218

DESCRIPTION centrosome spindle pole associated protein 1 isoform a

[Source:efSeq_peptide;Acc:NP_001070672]

TCTGAAATTAACATTGCTATCATTTTCTTTCATTTATGTATGTCTGATATATATATATATTTTTGTCTATTCCTTCAGTT

TTACCTTTGTGTCACCATAT

In100 candidate1041 (in intron):

LOCUS ENSG00000153956

DESCRIPTION Dihydropyridine-sensitive L-type calcium channel subunits alpha

2/delta precursor [Contains: L-type calcium channel subunit alpha-2; L-typ

calcium channel subunit delta]. [Source:Uniprot/SWISSPROT;Acc:P54289]

TCAAGCCTCTGTGTTTTCCTCTAAGGCATTTCATCCTTTTTTATAACTGGCTTTCCATTTTTTTCTTTCTCTTATCAGCT

CATCATTCTTTTAAATGCTC

In100 candidate1042 (in intron):

LOCUS ENSG00000153956

DESCRIPTION Dihydropyridine-sensitive L-type calcium channel subunits alpha

2/delta precursor [Contains: L-type calcium channel subunit alpha-2; L-typ

calcium channel subunit delta]. [Source:Uniprot/SWISSPROT;Acc:P54289]

TTGTTTTTGTTTTCCTGGAAGTGCTAGATGATTGTTATTTAACCAAATAGTTTTTTTTTCTTTAACTCTTAGGCCAGTC

ATTCTATATCATTTCTATTT

In100 candidate1043 (in intron):

LOCUS ENSG00000153956

DESCRIPTION Dihydropyridine-sensitive L-type calcium channel subunits alpha

2/delta precursor [Contains: L-type calcium channel subunit alpha-2; L-typ
calcium channel subunit delta]. [Source:Uniprot/SWISSPROT;Acc:P54289]

CTGTCTCACTGGCATGTCAAGACTAACATTCGCAAGTGAACTATTATTTCTATTACTTTTCTCCATCAGCATCCTGATCT
TCTGTTAAGTTTTGCATCTC

In100 candidate1044 (in intron):

LOCUS ENSG00000153956

DESCRIPTION Dihydropyridine-sensitive L-type calcium channel subunits alpha
2/delta precursor [Contains: L-type calcium channel subunit alpha-2; L-typ
calcium channel subunit delta]. [Source:Uniprot/SWISSPROT;Acc:P54289]

TTTATTTTTTATTGAAATTCAAAGTTAACTGATTGTCTTGTATTTCTTTTTGCTAAATTCATCAACCTTGTCTTTCAGGC
TTTCAGTTTCTATATTTGTA

In100 candidate1045 (in intron):

LOCUS ENSG00000153956

DESCRIPTION Dihydropyridine-sensitive L-type calcium channel subunits alpha
2/delta precursor [Contains: L-type calcium channel subunit alpha-2; L-typ
calcium channel subunit delta]. [Source:Uniprot/SWISSPROT;Acc:P54289]

TCAATAATTTTGATTCTATATTCATTTTTAACTCTAGAATATTTTTTCTCTGTAATTTTTATTTCAACACAGCATTCCTA
GTTTTAACCACGACTTTTTT

In100 candidate1046 (in intron):

LOCUS ENSG00000153956

DESCRIPTION Dihydropyridine-sensitive L-type calcium channel subunits alpha 2/delta precursor [Contains: L-type calcium channel subunit alpha-2; L-typ calcium channel subunit delta]. [Source:Uniprot/SWISSPROT;Acc:P54289]

TGAAGATGCTCTAAGAATGATACTAGATAAATAATTCTAAAAATTGAATAACAATGATGAAACAATATTTTCTTCCAGTC

CTCTTTTGTGTGTCTGTATA

In100 candidate1047 (in intron):

LOCUS ENSG00000163738

DESCRIPTION methylenetetrahydrofolate dehydrogenase (NADP+ dependent) 2-lik [Source:RefSeq_peptide;Acc:NP_001004346]

GATTTATCAAGTACATTTTAACTTTTATGGTTTTTTTTCTAGTTTCATTTGTTTATTGTTCAATTTCTACAGTTAAATGA

TTCATTTTCTTATTTCTGTT

In100 candidate1048 (in intron):

LOCUS ENSG00000163738

DESCRIPTION methylenetetrahydrofolate dehydrogenase (NADP+ dependent) 2-lik [Source:RefSeq_peptide;Acc:NP_001004346]

TCTTTGCCATTAGCATAGTAGCTGGACTGTAATAACTATTTTATGAATATTTTTATCTTGAACTTTTTTCTGCAGCTATA

CTCAATGTATGTCTTCTTTT

In100 candidate1049 (in intron):

LOCUS ENSG00000123213

DESCRIPTION Neurolysin, mitochondrial precursor (EC 3.4.24.16) (Neurotensinendopeptidase) (Mitochondrial oligopeptidase M) (Microsomal endopeptidase) MEP). [Source:Uniprot/SWISSPROT;Acc:Q9BYT8]

CTTATCTCCTGGCCTTAAATTAACATAAGCTGATCTCTTATTTGGAACATTCTTTTCTTTCTCTCCCATTTCCAGAGCCT

AATACTTATTCATTTATCTT

In100 candidate1050 (in intron):

LOCUS ENSG00000066777

DESCRIPTION Brefeldin A-inhibited guanine nucleotide-exchange protein 1 (Brfeldin A-inhibited GEP 1) (p200 ARF-GEP1) (p200 ARF guanine nucleotide exchnge factor). [Source:Uniprot/SWISSPROT;Acc:Q9Y6D6]

TCTACCTCTATCTCTTTCTCTGCCTCTTTGAAGACCAATAACTTTTAGAATTGCCCTTTTAAAACTATTTTCCAGATTTT

ATAGGTGTGCTTCATTCTTT

In100 candidate1051 (in intron):

LOCUS ENSG00000120669

DESCRIPTION OTTHUMP00000018257. [Source:Uniprot/SPTREMBL;Acc:Q9H1T5]

TGAAAAGTACTATATATATAGGAGCTGTGCTTAACTACATTGTATTCTCCTGTTTTTTCTTTTGTAAGCGTTCAGTGGAG

TGATTTTTTTTTTACTTTTT

In100 candidate1052 (in intron):

LOCUS ENSG00000064115

DESCRIPTION Transmembrane 7 superfamily member 3 precursor (Seven span tranmembrane protein). [Source:Uniprot/SWISSPROT;Acc:Q9NS93]

TTAGGGAAAATATATAGAGGTTAAGATTATTCTAGTCTTAACACTATTTGTGTCTTTTTTTTGGCTGTTCTTCCACAGGC

TACTCATTCTTGATTTTTTT

In100 candidate1053 (in intron):

LOCUS ENSG00000186472

DESCRIPTION PCLO_HUMAN Isoform 3 of Q9Y6V0 - Homo sapiens (Human)

[Source:Uiprot/Varsplic;Acc:Q9Y6V0-3]

TTTGCTAGAGGATAGAAGAAAATACTATGGCTCTTAACCTGTAAATTTCTTCTCCTCCTGTATTTTATATCTCAGGTTTG

GTTTTAGTTACATTATTCTT

In100 candidate1054 (in intron):

LOCUS ENSG00000165078

DESCRIPTION Carboxypeptidase A6 precursor (EC 3.4.17.1) (Carboxypeptidase B.

[Source:Uniprot/SWISSPROT;Acc:Q8N4T0]

TGAGAATAAACAAACCATATTCAAACTATAACAATGGGAGACTTTTTATTGCTGATTCAGTCTCCTCACTCCATTATCAG

TCTGTTCAGATTTTCTATTT

In100 candidate1055 (in intron):

LOCUS ENSG00000165078

DESCRIPTION Carboxypeptidase A6 precursor (EC 3.4.17.1) (Carboxypeptidase B.

[Source:Uniprot/SWISSPROT;Acc:Q8N4T0]

TTGATGAATCTACATTTATGTCACTTTCTTATTGCTAACATCGGTCATTTCAATCTTCATTCTTTTGTCCTTAAGCAGAT

TTGCTAACAATTTTTCTATT

In100 candidate1056 (in intron):

LOCUS ENSG00000172995

DESCRIPTION cAMP-regulated phosphoprotein 21 (ARPP-21).

[Source:Uniprot/SWISPROT;Acc:Q9UBL0]

CCAATATAACCATAACTTTTAATTTTGTCTGGCCTCAGACTCAGCTTTCAACAGGTCCATTTCTAACTTAGGACCTCTTG

CCTGTTCTTTCTTTGCTTTT

In100 candidate1057 (in intron):

LOCUS ENSG00000172995

DESCRIPTION cAMP-regulated phosphoprotein 21 (ARPP-21).

[Source:Uniprot/SWISPROT;Acc:Q9UBL0]

TTATTGAGGTCACCGGTTTGTTTGTTTTTTTAACCTAGGGTCTGCTGTCTTTTCTTCCTTGGCAGTTCTGCAACTGTGGT

CATTGTCACATTTTTCTTTC

In100 candidate1058 (in intron):

LOCUS ENSG00000075213

DESCRIPTION Semaphorin-3A precursor (Semaphorin III) (Sema III).

[Source:Unprot/SWISSPROT;Acc:Q14563]

TTTTATGTAGGTTTTCTGTTAAGAACAAATCCTTACTAACAATGTCTCTGTCAAATTTTTGCTTCTTCTCTGTCCAGTTC

TTTTAATGCACTTTCCTCCT

In100 candidate1059 (in intron):

LOCUS ENSG00000075213

DESCRIPTION Semaphorin-3A precursor (Semaphorin III) (Sema III).

[Source:Unprot/SWISSPROT;Acc:Q14563]

AAACATGAAAAATAACACTAAATCCCATTGCCTGTTCACCTGTTTGGTACTTTTTCATCTTCCCTTTCATTCTCCAGCCT

TCATAATCCTTCTTTTACCT

In100 candidate1060 (in intron):

LOCUS ENSG00000165935

DESCRIPTION

TCTATAGATGATCAATAGAGGAGTGTTGAAATCTCCAACTGTAACTGTAGATATGTCTATTTCTGCTTGCAGAGCTATCA

GTTTTTGCTTCATGTACTTT

In100 candidate1061 (in intron):

LOCUS ENSG00000165935

DESCRIPTION

TAGGTTGTCTCATAACCCATAACTCACTGATGTTCTGGGCTTTTTTTTTCTCTCTTTTCTTTCTGTGTTTCATTTCAGAC

TTTCTATCACTATATCTTAA

In100 candidate1062 (in intron):

LOCUS ENSG00000165084

DESCRIPTION Vest-1 protein. [Source:Uniprot/SPTREMBL;Acc:Q96Q28]

AGACACCTTTTTCTCTGCTTTATATCATGTCTAACCCCTTTCTACTTCGTTCTTTTTTGCCCAGATTGGCATCATTGCTC

CTGTCTTTTTGGGTTTGTCT

In100 candidate1063 (in intron):

LOCUS ENSG00000165084

DESCRIPTION Vest-1 protein. [Source:Uniprot/SPTREMBL;Acc:Q96Q28]

TAACTTTTTTGTGATTGTTCTACATGTCCCTAAGACTGTTTTTTTTGTCTTATTTTTCTCTTTGTTGTTCAGATTAGTTA

AATTTTATTGCTCTGTCCTC

In100 candidate1064 (in intron):

LOCUS ENSG00000165084

DESCRIPTION Vest-1 protein. [Source:Uniprot/SPTREMBL;Acc:Q96Q28]

ATTTTCCTAGTATTAATATTAACTATGGCTAAACTTTACTTTGCATTGTCTTTTGTTTCAACTCTACTCCTATGACAGAT

TAATTACTTTGCATTTTCAT

In100 candidate1065 (in intron):

LOCUS ENSG00000165084

DESCRIPTION Vest-1 protein. [Source:Uniprot/SPTREMBL;Acc:Q96Q28]

TTTAAGAAATGTTCTGCCATTTATAAACTTTAGTATTTCTCCATAACCTCATGATGTTATGTTGTCCTTCAGGTTGTAAT

AATCTGTCATTCTTCACTTT

In100 candidate1066 (in intron):

LOCUS ENSG00000120693

DESCRIPTION Mothers against decapentaplegic homolog 9 (SMAD 9) (Mothers aganst DPP homolog 9) (Smad9) (Madh6). [Source:Uniprot/SWISSPROT;Acc:O15198]

TTTTAAAAAAATATAACAAAGGATATAAGTTTTTTTTTTTTTTTTTTTTTTTTTTTTTTTTTTTTGAGACAGGGT

CTTGCCCTTTTATCTAGGCT

In100 candidate1067 (in intron):

LOCUS ENSG00000137573

DESCRIPTION Extracellular sulfatase Sulf-1 precursor (EC 3.1.6.-) (HSulf-1)

[Source:Uniprot/SWISSPROT;Acc:Q8IWU6]

AAATCTGACTCCATACCCGCTACCATTAACTAGTCCTTATTCCACATTTTCCTTTTTTTCCTTTAAGCTCAGTTGACTTT

TTAACGAATGTCTTTTCCTC

In100 candidate1068 (in intron):

LOCUS ENSG00000167323

DESCRIPTION Stromal interaction molecule 1 precursor.

[Source:Uniprot/SWISSROT;Acc:Q13586]

AGGGAATGGTGGAAGAGGAAGTGTAACATGTTTTTGTGTCTTCAATAGCTTTTCAGCACTCTTTTTTTTTTTCCTCCTCT

TTTCTTCTGGAGACTTTGCA

In100 candidate1069 (in intron):

LOCUS ENSG00000167323

DESCRIPTION Stromal interaction molecule 1 precursor.

[Source:Uniprot/SWISSROT;Acc:Q13586]

CATTCTCTCCTCTCCTTCTGAGAATCTAATATTTATGTTAGATCTTTTAACTGTATTCCACATGTCTCTTATGCTCAGTT

CTGTTCTTTTTTCTCTCTGT

In100 candidate1070 (in intron):

LOCUS ENSG00000137571

DESCRIPTION Solute carrier organic anion transporter family member 5A1 (Solte carrier family 21 member 15) (Organic anion transporter polypeptide- relted protein 4) (OATP-RP4) (OATPRP4). [Source:Uniprot/SWISSPROT;Acc:Q9H2Y9]

EXON

CTCATTCATAGAAAAGTCCACTCCTTTATGACCTATGATTTAACCCCTGGTTACCTCTTCGACTTTATCTCCTTTCAGTC

CCACTTGTTTATTGTGTGTT

In100 candidate1071 (in intron):

LOCUS ENSG00000140396

DESCRIPTION Nuclear receptor coactivator 2 (NCoA-2) (Transcriptional

intermdiary factor 2). [Source:Uniprot/SWISSPROT;Acc:Q15596]

CTCACTGTATTCTGTGCTATATAACCCTTTTTTCCCATGTTTATTTTCAGCACTCTAATTTAAGACATCTTTTCTCACCT

TAACTCATCTCTGCTTCTTT

In100 candidate1072 (in intron):

LOCUS ENSG00000144674

DESCRIPTION Golgin subfamily A member 4 (Trans-Golgi p230) (256 kDa

golgin)(Golgin-245) (Protein 72.1). [Source:Uniprot/SWISSPROT;Acc:Q13439]

AGGTTGCTTCCTTGTTTTAGTGGTAAAGACATATCTAACCTTCTATGCTTTCCTTCTGCTTTTTAAATACCTGCAGACAT

CTGTTTCTTATCATTATATC

In100 candidate1073 (in intron):

LOCUS ENSG00000196567

DESCRIPTION similar to microtubule associated testis specific serine/threonne

protein kinase (LOC375449), mRNA [Source:RefSeq_dna;Acc:NM_198828]

CTTTAGCCATTACTACACTATTTTTAACAAGGGTAGACATATGTTTCTATATTTGCTCTTCTTTTACAGGTTTTTATGTA

GTACGTTCATCTACTCATTT

In100 candidate1074 (in intron):

LOCUS ENSG00000069020

DESCRIPTION Microtubule-associated serine/threonine-protein kinase 4 (EC 2..11.1). [Source:Uniprot/SWISSPROT;Acc:O15021]

TTTAGTTTAAAACATTAAAACCATACAAACATTGTATATTTGGAACACCTAACCTCTTCTAAGTGTTCTGTACCTCAGCA

CTTTTTTTATCTTGGGTGTT

In100 candidate1075 (in intron):

LOCUS ENSG00000133110

DESCRIPTION Periostin precursor (PN) (Osteoblast-specific factor 2) (OSF-2) [Source:Uniprot/SWISSPROT;Acc:Q15063]

TAGAAATAAAATTACGTTCATACTGTTTGAATAGTCCTAGTTGGTTAACTTTTTGAAAAGCTTGTATTTTACTCTCAGAA

ATGTATAATTTTTCTCTCTC

In100 candidate1076 (in intron):

LOCUS ENSG00000123106

DESCRIPTION Coiled-coil domain-containing protein 91 (GGA-binding partner) p56 accessory protein). [Source:Uniprot/SWISSPROT;Acc:Q7Z6B0]

TCTTCAAAGGTTAACATTTTACCACATTTGCTTTATCATTTTCTTCCCTTTCCTTCTCATTTTTCTTATTCTCCTCAGTA

TTTATTTAGTGTTCTTTTTG

In100 candidate1077 (in intron):

LOCUS ENSG00000123106

DESCRIPTION Coiled-coil domain-containing protein 91 (GGA-binding partner)

p56 accessory protein). [Source:Uniprot/SWISSPROT;Acc:Q7Z6B0]

TATCTGTAGGGTTACCACTTATTTGATTCTGTGCACTGAAACATATAACTTGTATTTGTACCTTTTTTTAATCAGTATTT

GCACCTTTTTATCAGTATTT

In100 candidate1078 (in intron):

LOCUS ENSG00000123106

DESCRIPTION Coiled-coil domain-containing protein 91 (GGA-binding partner)

p56 accessory protein). [Source:Uniprot/SWISSPROT;Acc:Q7Z6B0]

TCTGCTACCCCATGGGAATTATTAACCATTGTCCTATCTTTTTATATTTGTTGGTCAATGTATTCTATTTTCTTTCAGCC

ATTATTTTTCTAATTTTTGT

In100 candidate1079 (in intron):

LOCUS ENSG00000035115

DESCRIPTION SH3 domain containing, Ysc84-like 1

[Source:RefSeq_peptide;Acc:P_056492]

TTTACTCATTGAACTTGCTATATTAACCTTTTTTATTTCCCATTTTTTGTTCTGTTCCATTGTTTCTGTTCATTCAGGTT

ACCATCTGGTGTCATTTCCT

In100 candidate1080 (in intron):

LOCUS ENSG00000085563

DESCRIPTION Multidrug resistance protein 1 (EC 3.6.3.44) (ATP-binding cassete sub-family B member 1) (P-glycoprotein 1) (CD243 antigen). [Source:Uniprt/SWISSPROT;Acc:P08183]

TTTTTTAACAAGTCTAATTTCTCTCTGTTGTTCAGATTAGGAAATTTCTATTGTTTTGTCTTTCAGTTCACTGGTTCTTTCCACTGTAACTCCCATTTTC

In100 candidate1081 (in exon):

LOCUS ENSG00000085563

DESCRIPTION Multidrug resistance protein 1 (EC 3.6.3.44) (ATP-binding cassete sub-family B member 1) (P-glycoprotein 1) (CD243 antigen). [Source:Uniprt/SWISSPROT;Acc:P08183]

AATAGTAACTTGTTTTCACTATTGTTTCTAGCCCTTGGAATTATTTCTTTTATTACATTTTTCCTTCAGGTAAATGTTTCCATTTTCACTATATTCATTT

In100 candidate1082 (in intron):

LOCUS ENSG00000085563

DESCRIPTION Multidrug resistance protein 1 (EC 3.6.3.44) (ATP-binding cassete sub-family B member 1) (P-glycoprotein 1) (CD243 antigen). [Source:Uniprt/SWISSPROT;Acc:P08183]

TAATGACAAACGTTAGGCTTAAATTACAAATAATAATATACCTAACATTGGAAATTTCCATCATTCCTAGTTTGTCAGACTCCTTTATCTTGCTAATTTG

In100 candidate1083 (in intron):

LOCUS ENSG00000087502

DESCRIPTION Endoplasmic reticulum-Golgi intermediate compartment protein 2.[Source:Uniprot/SWISSPROT;Acc:Q96RQ1]

AACATTTTGGATGTATTTTGGATGTGTCTAAAGTCTGTTTTATAACCTTTTTTAATATTTTTGTTATTACTTTCCAGTAG

TTTCCTTTTCACTTTTCTTC

In100 candidate1084 (in intron):

LOCUS ENSG00000149932

DESCRIPTION

AATTAACCATATATGTGAGGATTTATTTCTGGGTTCTCTATTCTACTCCATTTGTTTCTCTATCTACCCTTAGGTCAGTA

TCACACTGGGTTTTTTCTTT

In100 candidate1085 (in intron):

LOCUS ENSG00000144677

DESCRIPTION CTD small phosphatase-like protein (CTDSP-like) (Small C-terminl

domain phosphatase 3) (Small CTD phosphatase 3) (SCP3) (Nuclear LIM

interctorinteracting

factor 1) (NLI-interacting factor 1) (NIF-like protein) (RSP3) (YA22

protein) (HYA22). [Source:Uniprot/SWISSPROT;Acc:O15194]

AGTATTTTGATTGATATACCCTCTATAACATTTTAATATCTTTATCTCAATTATTCTTTCCTAATGTTGCAGTTAACTGT

ATTTTTTCATTTTGCTACGT

In100 candidate1086 (in intron):

LOCUS ENSG00000187950

DESCRIPTION Ovochymase-1 precursor (EC 3.4.21.-).

[Source:Uniprot/SWISSPROTAcc:Q7RTY7]

AGCTCTGTGTGATTGGGCCTCCCCACTTAACCTCTTCCACATAATTTCCCTCTATTTTTCTTGTGGTTAACCTTCAGATA

CCGTGGCCTTTCTTCTTTCT

In100 candidate1087 (in intron):

LOCUS ENSG00000185787

DESCRIPTION Mortality factor 4-like protein 1 (MORF-related gene 15 protein

(Transcription factor-like protein MRG15) (MSL3-1 protein).

[Source:Unipro/SWISSPROT;Acc:Q9UBU8]

AGTATTCTTAAGTATTTGTAATAATAAAAACAAAAATTATAACTTTTATTAAGAGGTCATTTTATTTCCTACCAGAATTA

CTGATCTGATTTTTCTACTT

In100 candidate1088 (in intron):

LOCUS ENSG00000105784

DESCRIPTION Rap2-binding protein 9 [Source:RefSeq_peptide;Acc:NP_612147]

GTGCCCACCCTTCTCTGCTTAACCTAACTCTAGTTTTCTAGTTTTTCTGTCTCTGTTTTCTCTCCTCCAAAACAGGGCTT

TTTAAATACTCTTTGGTTTT

In100 candidate1089 (in intron):

LOCUS ENSG00000105784

DESCRIPTION Rap2-binding protein 9 [Source:RefSeq_peptide;Acc:NP_612147]

TCTATTAATTTTTTTAACCTTTCAAAAAACCAACTTTTCATTTAACTGATCCTTTGGGTTGATTTTTTTTTGTCTCAGTT

TCATTCAGTTCTGCTCTGAT

In100 candidate1090 (in intron):

LOCUS ENSG00000167346

DESCRIPTION Matrix metalloproteinase-26 precursor (EC 3.4.24.-) (MMP-26)

(Mtrilysin-2) (Endometase). [Source:Uniprot/SWISSPROT;Acc:Q9NRE1]

CATCTGATTTCATTTTTTCCTGTAAAATATTAGTAAACGCTGGTCAATTAACTGGAATCTTCTATTTCTTGCTCTCAGAT

CTATGTTCTGACCTTCAGTT

In100 candidate1091 (in intron):

LOCUS ENSG00000167346

DESCRIPTION Matrix metalloproteinase-26 precursor (EC 3.4.24.-) (MMP-26)

(Mtrilysin-2) (Endometase). [Source:Uniprot/SWISSPROT;Acc:Q9NRE1]

CTGTAACATTTGCCCTTTCCTCGTTTTCTGTTCTCTCCTTTTTCTTTGTCCTATTTCACACACTTTCAGCATTCCCTATG

CATAGTTAATTTTATCTTTT

In100 candidate1092 (in intron):

LOCUS ENSG00000133107

DESCRIPTION Short transient receptor potential channel 4 (TrpC4) (Trp-relatd

protein 4) (hTrp-4) (hTrp4). [Source:Uniprot/SWISSPROT;Acc:Q9UBN4]

GTGGGAACATTCATAACATTGTTGCAATATTTCCCCTTCTAGGCCTTTATATCAGGCATCTCTTGACGTTGGCCCTATTC

CTTTGTTCATCTATTGCAAT

In100 candidate1093 (in intron):

LOCUS ENSG00000133107

DESCRIPTION Short transient receptor potential channel 4 (TrpC4) (Trp-relatd

protein 4) (hTrp-4) (hTrp4). [Source:Uniprot/SWISSPROT;Acc:Q9UBN4]

GGTGTGAGCTGCCAATACTAACTTTTTAGTTATCTACATACTCACTTTTGCTGTGATCTTTATTTTCTCATACAGCTTTG

TGCTACTGTCTAATGTCCTT

In100 candidate1094 (in intron):

LOCUS ENSG00000133107

DESCRIPTION Short transient receptor potential channel 4 (TrpC4) (Trp-relatd protein 4) (hTrp-4) (hTrp4). [Source:Uniprot/SWISSPROT;Acc:Q9UBN4]

TAACTTTAAACAAAGTATTTAACCTTCCTCAGTCTCTAGTTCCTTACCTGTGAAATAGACATTTTATTCTTACCTTTTAC

ATATCTCCATATACTCCTTC

In100 candidate1095 (in intron):

LOCUS ENSG00000182674

DESCRIPTION Potassium voltage-gated channel subfamily B member 2 (Voltagegted potassium channel subunit Kv2.2). [Source:Uniprot/SWISSPROT;Acc:Q92953]EXON

TATCTGTCTTTCATAAAATTGTATCTAGATATACTTCCCTGTATCTAACCTATGACTTTTTTTTTGCTCTGTGACAGTT

CTCCTTTAAATTTTTCATTT

In100 candidate1096 (in intron):

LOCUS ENSG00000182674

DESCRIPTION Potassium voltage-gated channel subfamily B member 2 (Voltagegted potassium channel subunit Kv2.2). [Source:Uniprot/SWISSPROT;Acc:Q92953]EXON

TTATATTCATTAGTGAGAATAGTCTGTAACTTTATTGTACTTTCTTGTTCACTTTTAGCTACTAGCCAGATGAGTTAGGT

AGCTTTCTCTTTTTCTATCC

In100 candidate1097 (in intron):

LOCUS ENSG00000008277

DESCRIPTION ADAM 22 precursor (A disintegrin and metalloproteinase domain 2)

(Metalloproteinase-like, disintegrin-like, and cysteine-rich protein 2)

(etalloproteinase-disintegrin ADAM22-3). [Source:Uniprot/SWISSPROT;Acc:Q9P0K]

CCCTGTCTTTTCCAACAAAAATAAATTCAAGCTATTTAACCTTTTTGTGTGACTTTATTCACCAGACTTTGTCATTCGCT

GCCTGGAGCTGCATTTTCTT

In100 candidate1098 (in intron):

LOCUS ENSG00000008277

DESCRIPTION ADAM 22 precursor (A disintegrin and metalloproteinase domain 2)

(Metalloproteinase-like, disintegrin-like, and cysteine-rich protein 2)

(etalloproteinase-disintegrin ADAM22-3). [Source:Uniprot/SWISSPROT;Acc:Q9P0K]

GGTGAAAACCTGTCGTTTGCATATCCTTAACATTAGGGACTTAACTGCTTTTATTTTTCTCTTCTTTTTTAGCAGGTACT

TTGCTCTTAGAGTATTTCTT

In100 candidate1099 (in intron):

LOCUS ENSG00000008277

DESCRIPTION ADAM 22 precursor (A disintegrin and metalloproteinase domain 2)

(Metalloproteinase-like, disintegrin-like, and cysteine-rich protein 2)

(etalloproteinase-disintegrin ADAM22-3). [Source:Uniprot/SWISSPROT;Acc:Q9P0K]

AGTGTTTTTGACTGCTAACTTTTCCTTTTGATTCTTTCTTAGAATTTTTCTCTCTCTGCTTACATTATTCATCAGCTCTT

GGATGTTGTCTACTTTTTCC

In100 candidate1100 (in intron):

LOCUS ENSG00000008277

DESCRIPTION ADAM 22 precursor (A disintegrin and metalloproteinase domain 2)

(Metalloproteinase-like, disintegrin-like, and cysteine-rich protein 2)

(etalloproteinase-disintegrin ADAM22-3). [Source:Uniprot/SWISSPROT;Acc:Q9P0K]

TATGGCCCCTATATTTTATGGTGTTCACCATCTTTTAATAAGATTAACTCCTTCCTCATCACTTTTATTCCTATCCAGTG

GTTTTCTACTGTCTTTCTTT

In100 candidate1101 (in intron):

LOCUS ENSG00000008277

DESCRIPTION ADAM 22 precursor (A disintegrin and metalloproteinase domain 2)

(Metalloproteinase-like, disintegrin-like, and cysteine-rich protein 2)

(etalloproteinase-disintegrin ADAM22-3). [Source:Uniprot/SWISSPROT;Acc:Q9P0K]

TTTTTTTCTTTCAATTACTGAGGGAGGATTGTTGAAATCCTTAACTATAACTTTGAATTTGTCTATTTCTCCTTTCAGGT

CTGTCACTTTTTACTTCATG

In100 candidate1102 (in intron):

LOCUS ENSG00000008277

DESCRIPTION ADAM 22 precursor (A disintegrin and metalloproteinase domain 2)

(Metalloproteinase-like, disintegrin-like, and cysteine-rich protein 2)

(etalloproteinase-disintegrin ADAM22-3). [Source:Uniprot/SWISSPROT;Acc:Q9P0K]

ACATGCACCATAAAGGCATGCTCTGCTAATTAACAAAATAAAATGTCCTCATATATTGTATTGTTTTTCCAGTCAAAACT

TTTGCATCTTCCTCATTGTT

In100 candidate1103 (in intron):

LOCUS ENSG00000189292

DESCRIPTION RGPG542. [Source:Uniprot/SPTREMBL;Acc:Q6UX46]

TCTGTTTGCTTATTTATAACGATTTCAATCTATTTCTTAACTTCTCATGTTGCTTATGTATTTCCTGATTTCATTCAGTT

GTCTATCTGTGTCCCCTTGT

In100 candidate1104 (in intron):

LOCUS ENSG00000189292

DESCRIPTION RGPG542. [Source:Uniprot/SPTREMBL;Acc:Q6UX46]

ATAGAATTACATTTTTAAACATGTTCAAGATTATTTTTAACCTTTTATACTCCTTTCTGGTTTTCACTGTTCAGAGGCTA

TTTTAAAGCTCTTCCTCCTT

In100 candidate1105 (in intron):

LOCUS ENSG00000138760

DESCRIPTION Lysosome membrane protein 2 (Lysosome membrane protein II) (LIM

II) (Scavenger receptor class B member 2) (85 kDa lysosomal membrane

sialolycoprotein) (LGP85) (CD36 antigen-like 2).

[Source:Uniprot/SWISSPROT;Acc:Q4108]

TCTGTAGAGGGCTGTGGTCCTGATTACTATAGATGTAACCAAGAAAGAAATTATACTTTCCACATACCATTTTTGCAGTG

TTTCATTTATTTATTCATTT

In100 candidate1106 (in intron):

LOCUS ENSG00000172554

DESCRIPTION Gamma-2-syntrophin (G2SYN) (Syntrophin 5) (SYN5).

[Source:Uniprt/SWISSPROT;Acc:Q9NY99]

AAAATTTTCATAAATATTTTATAACCTCTTACACATTTTTCCATTCTCTTTCTTTTCTCAACTTTGTATATCCTTCAGTT

TATCTTTTTCTTTCTTCTTT

In100 candidate1107 (in intron):

LOCUS ENSG00000138771

DESCRIPTION shroom family member 3 protein

[Source:RefSeq_peptide;Acc:NP_06910]

TAGTAATCCTTTTTTCCAAAAGGTGATCATACTGTTTTGTAACATGCTTTTTCTTTTTCTTTTTTTTTTTTTTGGCAGAG

TTTTTGCTCTTGTTGCCCAG

In100 candidate1108 (in intron):

LOCUS ENSG00000182348

DESCRIPTION zinc finger 804B [Source:RefSeq_peptide;Acc:NP_857597]

TTGTTGTAAGGGCTACATGAAAGTATTCTGCTACTTTAACTTACTCTGCTTACAAATCTCCTCAATTTTTTTTTCCAGGT

CAACTTACCATTTTTCTTTT

In100 candidate1109 (in intron):

LOCUS ENSG00000182348

DESCRIPTION zinc finger 804B [Source:RefSeq_peptide;Acc:NP_857597]

ATCCAATTCTGCTAATTAAGTAATTATTGAAGACAAAATAACTCATAGAGAAGCCCTATATATTCCATATTTTTCCAGTC

TCTCCTCATTTAATATTCTT

In100 candidate1110 (in intron):

LOCUS ENSG00000182348

DESCRIPTION zinc finger 804B [Source:RefSeq_peptide;Acc:NP_857597]

AATAATTATTTAATGTAAGTATATTCATTCTGTGTAACTGGATGCTAACTTTGACTCTTTCCTCTTTGTTTGTGCCAGAC

TTCTGAATAGCCCTTTTTTC

In100 candidate1111 (in intron):

LOCUS ENSG00000182348

DESCRIPTION zinc finger 804B [Source:RefSeq_peptide;Acc:NP_857597]

GAATGTCTTTGTATCTGAGTGATTTAATTCTTTCATCATATGCTAACTCTTCTTCATTAATTTTTTTATTCAGCTGCCT

CTTGCCTAATGCTTTCAGTT

In100 candidate1112 (in intron):

LOCUS ENSG00000182348

DESCRIPTION zinc finger 804B [Source:RefSeq_peptide;Acc:NP_857597]

ATGGTTTATTGAATTTGTTTATCTTTCAAAAAAGCTAACTTTTTGTTTCATTGATCTTTTCTATTTTGTTAGTCTCAGTT

TTGTTTAGTTCTTTTCTGAG

In100 candidate1113 (in intron):

LOCUS ENSG00000183323

DESCRIPTION coiled-coil domain containing 125

[Source:RefSeq_peptide;Acc:NP789786]

ACCATGTCCAAACTGATGTTAACTTTTTACATATTTTATTTATTTATTTGTTTATTTATTTATTTATTTTCACACAGGGT

CTTGCTCTGTTGCCCAGTTT

In100 candidate1114 (in intron):

LOCUS ENSG00000086666

DESCRIPTION AN1-type zinc finger protein 6 (Zinc finger A20 domain-containig protein 3) (Associated with PRK1 protein). [Source:Uniprot/SWISSPROT;Acc:6FIF0]

AAATTGATGCGAGAGCAAAGGTTTATTTGGTTTCCATAACTATTTACTAATTTTATGCTTGCCAGATTTTAAGATTAAAC

TGTTTGCCATCTCTTTTTTT

In100 candidate1115 (in intron):

LOCUS ENSG00000040341

DESCRIPTION Double-stranded RNA-binding protein Staufen homolog 2. [Source:niprot/SWISSPROT;Acc:Q9NUL3]

GTTGCCTTTCACCTTTCTCTAGTGCCTCCTTGAGCTGCTTAATAACCGACCTACTGAATTCTTTTTCTGGTAATTCAGAT

ATTTCTTCCTGGTTTGGATC

In100 candidate1116 (in intron):

LOCUS ENSG00000188321

DESCRIPTION Zinc finger protein 559. [Source:Uniprot/SWISSPROT;Acc:Q9BR84]

EXON

TATATAGGCTTTCTACTCATTTCTCTCTCTGCTCCCTCTGTAACTTCTTTAATGTTTCTGTTCACTTGGCAGAGTTCCAC

AGGTTCTTTAGGCTTTCTTC

In100 candidate1117 (in intron):

LOCUS ENSG00000172939

DESCRIPTION Serine/threonine-protein kinase OSR1 (EC 2.7.11.1) (Oxidative sress- responsive 1 protein). [Source:Uniprot/SWISSPROT;Acc:O95747]

TCCTGATTCCTAACTTAGGGCTCTGTTCCTTCTGAATCTTATCTGTCTGTTTCACATATATATTCCACTCAGCTTCTTAG

CACTGAGTTCTTTAATTTTT

In100 candidate1118 (in intron):

LOCUS ENSG00000136379

DESCRIPTION

TTGTTGACTTTCTGCAAAGAAGTGAGAATTTTTCTTGGTTAACCAATTTTGCGGACTTCCTTTTACTATTTCCAGTGTTT

TATAGTTATTTTTGGTTCTT

In100 candidate1119 (in intron):

LOCUS ENSG00000198028

DESCRIPTION Zinc finger protein 560. [Source:Uniprot/SWISSPROT;Acc:Q96MR9]

EXON

TGTTTACTTGTGATTTTCTTTTTTCATATAGGCATTTATTGCTATTAACTGCCCTCTTTAAATCCTTTGCTTGTTCAGGT

GTGTGTTACTTCATTTCCAC

In100 candidate1120 (in intron):

LOCUS ENSG00000198028

DESCRIPTION Zinc finger protein 560. [Source:Uniprot/SWISSPROT;Acc:Q96MR9]

EXON

CTGGACTTGGATGTTTTGGGATATCTTAACTTTTCATCCTCCATTGCTTTGCATCTATCCAGACTTCCCTATTACCTAAC

AAATGACCTTCTTTATGTTT

In100 candidate1121 (in intron):

LOCUS ENSG00000138759

DESCRIPTION Extracellular matrix protein FRAS1 precursor.

[Source:Uniprot/SISSPROT;Acc:Q86XX4]

GGTGAAAAATTTGAGTCTCTTGAAACACTCGTTCTCAATTGAGGTCTAACAAAGTGACATTGTTTCTTCTTGTTTCAGCT

CTTCTCTCTCTCTCTATTTT

In100 candidate1122 (in intron):

LOCUS ENSG00000138759

DESCRIPTION Extracellular matrix protein FRAS1 precursor.

[Source:Uniprot/SISSPROT;Acc:Q86XX4]

CTTTTGACTTATATTTCCCCATATCCCCCTCCTCGTCGCTCCTGGTAACCACTGTTCTCTTTGTTTCTGTGTATCCAGCT

TTTGGAGTTTTTTGTTCCCA

In100 candidate1123 (in intron):

LOCUS ENSG00000150893

DESCRIPTION FRAS1-related extracellular matrix protein 2 precursor (ECM3

hoolog). [Source:Uniprot/SWISSPROT;Acc:Q5SZK8]

TAGGAATACTGATCTGTATAACATATTCTAATTTTCTCTTACGTAGAATATCATGTTCCTCCCTTTTTCTTTAATCAGAT

TCTCATTACACATCTTTTAA

In100 candidate1124 (in intron):

LOCUS ENSG00000150893

DESCRIPTION FRAS1-related extracellular matrix protein 2 precursor (ECM3

hoolog). [Source:Uniprot/SWISSPROT;Acc:Q5SZK8]

TGATAAGTATATTATTCTAGAGTAAAAATAACATTTACTTGGTTTAACTACACTTACTTTTATCCTACTGACTTTCAGTC

ATTTTTACTGTATCTGTTCA

In100 candidate1125 (in intron):

LOCUS ENSG00000150893

DESCRIPTION FRAS1-related extracellular matrix protein 2 precursor (ECM3 hoolog). [Source:Uniprot/SWISSPROT;Acc:Q5SZK8]

AAAAATAAAGATTCAATCCCTAGAAGAAAAATAAAGGCTCATATTAACAAATCTACCTCTTTTTCTTTATAGATCAGGTA

TCTCTACTTCCTTCATGTCT

In100 candidate1126 (in intron):

LOCUS ENSG00000127914

DESCRIPTION A-kinase anchor protein 9 (Protein kinase A-anchoring protein 9 (PRKA9) (A-kinase anchor protein 450 kDa) (AKAP 450) (A-kinase anchor protin 350 kDa) (AKAP 350) (hgAKAP 350) (AKAP 120-like protein) (Protein hyperin) (Protein yotiao) (Centrosome- and Go [Source:Uniprot/SWISSPROT;Acc:Q9999]

AATTTTAACTTGAGCCTATCATTTATTTATCATTTAAGTACTTTTTTTTTTAAACCATTGTTGCCTTTCTTTCAGTTAAT

CTATCTTGTATATTCTGCTC

In100 candidate1127 (in intron):

LOCUS ENSG00000170456

DESCRIPTION CDNA FLJ43333 fis, clone NT2RI3006376.

[Source:Uniprot/SPTREMBLAcc:Q6ZUT9]

GCCTTATCTCTGAACTTACTATATATAACATTGATATAACCTTTTCTATTGTTTTTGACGCCTATTTTATTCAGTTAGCA

TTATTTCCCTTGGCATCCTT

In100 candidate1128 (in exon):

LOCUS ENSG00000139160

DESCRIPTION

TATTTTAAATTTATGATCTGGTAATTCTAAAATGTCTGCCATAACTGAGTCTGGTTCTGATGCTTGTTTTCTTTTCAGAC

TGTTTTTTTGTCTTTTAGCA

In100 candidate1129 (in intron):

LOCUS ENSG00000174718

DESCRIPTION CDNA FLJ10652 fis, clone NT2RP2005886.

[Source:Uniprot/SPTREMBLAcc:Q9NVL6]

GTTTTCAAGGCTCATTTATGTTAACACATGTATGTTACTTCATTCGTTTTTATTACCAGATATTCCGTTGTATGGATATG

CCACTTTTGCTTATTCTTCC

In100 candidate1130 (in intron):

LOCUS ENSG00000185313

DESCRIPTION Sodium channel protein type 10 subunit alpha (Sodium channel

prtein type X subunit alpha) (Voltage-gated sodium channel subunit alpha Nav18)

(Peripheral nerve sodium channel 3) (hPN3). [Source:Uniprot/SWISSPROT;Ac:Q9Y5Y9]

TATTTCTCTAACTCTTCTTCTGCCCTCTTTATTGTCTCTTTTTCTTTTCTTTTCTTTTCTTTTTTTTTTTTTTTCAGTT

GTTTGGTTGCTTTGGTTTCT

In100 candidate1131 (in intron):

LOCUS ENSG00000140598

DESCRIPTION elongation factor Tu GTP binding domain containing 1 isoform 2

Source:RefSeq_peptide;Acc:NP_001035700]

GACTAGAATGCAAGGCTTGGGTGTAGCCTAACAAATCTCTGGCTCCGTTTTTTAAAATGTATTTTCCTTTTTACAGGAAC

TTGTTCTCCAATTTCCCTTT

In100 candidate1132 (in intron):

LOCUS ENSG00000140598

DESCRIPTION elongation factor Tu GTP binding domain containing 1 isoform 2

Source:RefSeq_peptide;Acc:NP_001035700]

AGACCATCACATTCCATACTATGGAAATCATCAACTTGAAATAACTTTCACATTATTTTCCTAACCTACATTTTGCAGCA

CTTTCTCTCTGGTTTTCTCA

In100 candidate1133 (in intron):

LOCUS ENSG00000151746

DESCRIPTION Protein bicaudal D homolog 1 (Bic-D 1).

[Source:Uniprot/SWISSPRT;Acc:Q96G01]

AGTTCTAACATGTTTAGCTAGGTTCTTCCTATTTTTTGTGCTTCATCAGATCATTTGGGCTCAGTGTTTTCTTTCTTTTT

ATTTTTTTCTTTTTTTTTGA

In100 candidate1134 (in intron):

LOCUS ENSG00000183722

DESCRIPTION Lipoma HMGIC fusion partner precursor.

[Source:Uniprot/SWISSPRO;Acc:Q9Y693]

CCTGTTTTTCCCGTAACCTCTTTTCTTAGCCTGTACTTTTCTACTATTTTGCTTTGTTTTGTTTTGTTTTTTGGTCAGCT

GTTCTTTCTCCTGGTTGGCA

In100 candidate1135 (in intron):

LOCUS ENSG00000183722

DESCRIPTION Lipoma HMGIC fusion partner precursor.

[Source:Uniprot/SWISSPRO;Acc:Q9Y693]

CACATTTCCCTAATAACTAGTGATGTTGAACTTCTTTTCATGTACTTATTGCCCATTTGTATACCTTCTTTTGTGCAGTG

TCTGTTCAAATCTTTTGCTC

In100 candidate1136 (in intron):

LOCUS ENSG00000183722

DESCRIPTION Lipoma HMGIC fusion partner precursor.

[Source:Uniprot/SWISSPRO;Acc:Q9Y693]

AGCTTTATTCAATAACCTATTCATACCTACCTGGTGTTTCTCATTTTTCACAGTTTTCATTTATTTAAGCTTTCTTACAA

CTTTATTCCTTTATTGTGTG

In100 candidate1137 (in intron):

LOCUS ENSG00000183722

DESCRIPTION Lipoma HMGIC fusion partner precursor.

[Source:Uniprot/SWISSPRO;Acc:Q9Y693]

ATTGCATAATACAAGTATGTTAAATTGGATTAAAATGAATAACAAATGCTATTTTTCCTTCGTTAAATATTCCAGATTTC

TTTGATTAGATATCATCTCT

In100 candidate1138 (in intron):

LOCUS ENSG00000188659

DESCRIPTION

TTTACTAACTGGCCACCTCTGCCTAGTTATCATTTCTGTTTCTTTACAGGTCTATTTTGAATGTAACTTCAGCTTTCCAT

AGCTCTTAGCTTTACCTCCT

In100 candidate1139 (in intron):

LOCUS ENSG00000164683

DESCRIPTION Hairy/enhancer-of-split related with YRPW motif 1 (Hairy and

enancer of split-related protein 1) (HESR-1) (Cardiovascular helix-

loophelixfactor

2) (HES-related repressor protein 2) (HERP2).

[Source:Uniprot/SWISSPOT;Acc:Q9Y5J3]

TTTTATTTTTGTTATTGATTAACGGAAATAACCACCTTTTTTTTTGTCTTTGAAATTTACAGTCGTAATGGTAATGCCTT

CCTTTGTTTTGCTTGCCTTC

In100 candidate1140 (in intron):

LOCUS ENSG00000147586

DESCRIPTION Mitochondrial 28S ribosomal protein S28 (S28mt) (MRP-S28) (MRP-

35). [Source:Uniprot/SWISSPROT;Acc:Q9Y2Q9]

TTTTCTTAGTCTGGCTAGAAGCTTACCTCAAATAACCAACTTTTGCTTTCGTTGATTCTATTGATTTCCTGTTTTCAGTT

TCATTGCTTTCTGTGCTAAT

In100 candidate1141 (in intron):

LOCUS ENSG00000001631

DESCRIPTION Krev interaction trapped protein 1 (Krev interaction trapped 1)(Cerebral cavernous malformations 1 protein). [Source:Uniprot/SWISSPROT;AccO00522]

CACACTCTGCTTTCTCTTTTTCACTCTTCATAAACCTTAACTTTTTCTGAGTTTTTGATCTGTTTCCCTGCTCTGCAGCA
TTTAATTCTTGACTACTTTT

In100 candidate1142 (in intron):

LOCUS ENSG00000205574

DESCRIPTION

AGTTGTATAAACATTGAGAATTATAATGCATTGCTAATGAGCTTAACCTTTTATCATTCCATCTCTGATATTTCAGCTTA
TTTTGAATCCTACTTTCTCT

In100 candidate1143 (in intron):

LOCUS ENSG00000076554

DESCRIPTION Tumor protein D52 (N8 protein). [Source:Uniprot/SWISSPROT;Acc:P5327]

TTAACCATTGAAGCTATCTACTTTTACACTTTTCTTTGGGAGATTTTTGATACTTGTTCAATCTCTTTACTTGTTCAGAT
TTCTTCTATTTTGTTGGGTC

In100 candidate1144 (in intron):

LOCUS ENSG00000130812

DESCRIPTION Angiopoietin-related protein 6 precursor (Angiopoietin-like 6) Angiopoietin-related growth factor) (Angiopoietin-related protein 5). [Soure:Uniprot/SWISSPROT;Acc:Q8NI99]

GAGCCACCGCGCCTGGCCCTACTGTTATTTTTAGCATTTAACATTGTCTAATATGCTCTATTTATTCATCCTATACAGTA

CATATTTATTTTTTTGCCCA

In100 candidate1145 (in intron):

LOCUS ENSG00000001629

DESCRIPTION Ankyrin repeat and IBR domain-containing protein 1 (Fragment).

Source:Uniprot/SWISSPROT;Acc:Q9P2G1]

AGCCAAATAACTTCATTGTGCATTTCTTTTCTTTTGAAGCCACTAAACTTTCTCCTGTTACAGTTTTCATGTCATCCCTT

GAACTGTCCTCTGCTAATTT

In100 candidate1146 (in intron):

LOCUS ENSG00000179978

DESCRIPTION Baculoviral IAP repeat-containing 1 variant (Fragment).

[SourceUniprot/SPTREMBL;Acc:Q59GI6]

TTTTAAAGATGTTACACTTTGCTGTGAGAGATGTAGCTGACATAACTAAATTTTTATTTTCCCTTTCATAGTTGCCAGTT

TGCCAAATTTTATTTCTCTG

In100 candidate1147 (in intron):

LOCUS ENSG00000197826

DESCRIPTION Uncharacterized protein C4orf22.

[Source:Uniprot/SPTREMBL;Acc:QV702]

TCATTCACCACTGTCCTTGAGCTAAAATGTTCTCTTAACCATCAAGTCTATAATTCTTCTTCCAGAGTATCTGTAGGGTT

TACCATTTCCTTTCTATTTT

In100 candidate1148 (in intron):

LOCUS ENSG00000205570

DESCRIPTION Beta-glucuronidase-like protein SMA3.

[Source:Uniprot/SWISSPROTAcc:Q15486]

AGTTGTATAAACATTGAGAATTATAATGCATTGCTAATGAGCTTAACCTTTTATCATTCCATCTCTGATATTTCAGCTTA

TTTTGAATCCTACTTTCTCT

In100 candidate1149 (in intron):

LOCUS ENSG00000138669

DESCRIPTION cGMP-dependent protein kinase 2 (EC 2.7.11.12) (CGK 2) (cGKII)

Type II cGMP-dependent protein kinase). [Source:Uniprot/SWISSPROT;Acc:Q1323]

AGGCGTGAGGCACTGCACTCTGCCTGATTTAAGCTAACTCTTAAATATGATTCCTTCATATATTGTTTCAGCTCAATTAT

TTCTCTTTCTTCAGGGTCCC

In100 candidate1150 (in intron):

LOCUS ENSG00000080603

DESCRIPTION Snf2-related CBP activator protein

[Source:RefSeq_peptide;Acc:N_006653]

TCTTATACATAGTAAGTGCTCAAGAAATGTAACCTGTAATTTCTGTGTTTCTCTTTATTCAGTGTTATTTCTGGTTTTTT

AAAAATACTTTTTCATCCCC

In100 candidate1151 (in intron):

LOCUS ENSG00000139117

DESCRIPTION Copine-8 (Copine VIII). [Source:Uniprot/SWISSPROT;Acc:Q86YQ8]

GATTTTCCAATCCATTAACATGGAGGTTTTTCCATTTTTTTGTCTTTCTTATTTATTTCATTCATGCTTTGTGTTCAGTG

TCTTTTACCTTCTTAGTTAA

In100 candidate1152 (in intron):

LOCUS ENSG00000139117

DESCRIPTION Copine-8 (Copine VIII). [Source:Uniprot/SWISSPROT;Acc:Q86YQ8]

CATGTCAACTGTGGTTTTTCCCATTGGTTAAGGGCTCCCTTAACAATGTCGAAGTCTAATTTCATGCCTTTCCAGTCACT

TTTGTTTCTTAGAACCATCT

In100 candidate1153 (in intron):

LOCUS ENSG00000139117

DESCRIPTION Copine-8 (Copine VIII). [Source:Uniprot/SWISSPROT;Acc:Q86YQ8]

GTCCTATTCCTTCTAACAAAAAAATGCTTCTCTTTCTGCCTTCTTCTTTCCCTTCTTGCTGTTATTGCCTTAGTTCAGAT

TCTCATTACTTTTCTACCGA

In100 candidate1154 (in intron):

LOCUS ENSG00000139116

DESCRIPTION Kinesin family member 21A (Kinesin-like protein KIF2) (Renal

cacinoma antigen NY-REN-62). [Source:Uniprot/SWISSPROT;Acc:Q7Z4S6]

TCTGGCACATAGTAATCCTGAAATGTTAGTTGGGACTAACTTGCAAAAATCTATTTCTTTAACAGAGATTCTTTCTAGTC

TTCACTTGGGCCCCCTTTTT

In100 candidate1155 (in intron):

LOCUS ENSG00000139116

DESCRIPTION Kinesin family member 21A (Kinesin-like protein KIF2) (Renal cacinoma antigen NY-REN-62). [Source:Uniprot/SWISSPROT;Acc:Q7Z4S6]

TGGTTATTAACTTTGCCAATATCACTGACATTCTTGCTTTCTCATACCTTGCATCATTCTGATATGTTTTTCAGCTTTT
TTTTTTATGGTAATGTATTC

In100 candidate1156 (in intron):

LOCUS ENSG00000064726

DESCRIPTION BTB/POZ domain-containing protein 1 (Hepatitis C virus NS5Atrnsactivated
protein 8) (HCV NS5A-transactivated protein 8). [Source:UniprotSWISSPROT;Acc:Q9H0C5]

ATTCTTTTCAAGTGCTTAACGTCTTCTTTTCACAAATTTCCTCTTTTCTCTATCCCTGTTAGATTGCAGTTTATCATTTC
ATTCTAGTATATTAGCCTCT

In100 candidate1157 (in intron):

LOCUS ENSG00000081770

DESCRIPTION Baculoviral IAP repeat-containing protein 1 (Neuronal apoptosisinhibitory protein). [Source:Uniprot/SWISSPROT;Acc:Q13075]

TTTTAAAGATGTTACACTTTGCTGTGAGAGATGTAGCTGACATAACTAAATTTTTATTTTCCCTTTCATAGTTGCCAGTT
TGCCAAATTTTATTTCTCTG

In100 candidate1158 (in intron):

LOCUS ENSG00000184672

DESCRIPTION RALY-like protein isoform 1 (RALY-like protein isoform 2).

[Souce:Uniprot/SPTREMBL;Acc:Q86SE5]

ATAATGATTATTTGTGTTATTTTTAACTCAATAAAACCCAAACAATATCTATCTTTTCTTTTCCTGTGTCTTTTCCAGAT

TTTCTTCTTCCTTCTCTTTT

In100 candidate1159 (in intron):

LOCUS ENSG00000151692

DESCRIPTION Ubiquitin-conjugating enzyme 7-interacting protein 4 (UbcM4-

ineracting protein 4) (RING finger protein 144).

[Source:Uniprot/SWISSPROT;Ac:P50876]

AACACTCTTGACTTTGTTGACCCTCTGTATTGTAACTTTACTTTGTGTTTTATTTATTTTTGTTTTTGTTTGTTACAGGT

CTTCTTTTGCTCTCTTTGAT

In100 candidate1160 (in intron):

LOCUS ENSG00000173208

DESCRIPTION ATP-binding cassette sub-family D member 2

(Adrenoleukodystrophrelated

protein) (hALDR) (Adrenoleukodystrophy-like 1).

[Source:Uniprot/SISSPROT;Acc:Q9UBJ2]

AGGAACAATTTATCTTTTTCCTTTATTTAACTTTTTCCTTTATTTATCTTTTTCCTTTCAAATTCAGATGCCTTTTATTT

CTTTTTCTTGCCTAATTGCT

In100 candidate1161 (in intron):

LOCUS ENSG00000120688

DESCRIPTION WW domain-binding protein 4 (WBP-4) (Formin-binding protein 21)

[Source:Uniprot/SWISSPROT;Acc:O75554]

AACCCATAGTTTTAGAATAAGTTTTAACACCTGGTAGAACTAAATTCTCCTTTTTCTTTTTATTACAGTTTTCCTTCCCT

TACATGTTTATTTTTCCGAA

In100 candidate1162 (in intron):

LOCUS ENSG00000136404

DESCRIPTION Transmembrane 6 superfamily member 1.

[Source:Uniprot/SWISSPROTAcc:Q9BZW5]

AGGCAAGGCCGGCTTCCCTTCTCTGTGAATTAACCATCCCCACACTCCTCTCCATTTCTTTTTTCACAGCTTTTCTTATA

TTCACCATCAGATTTTTCTT

In100 candidate1163 (in intron):

LOCUS ENSG00000133739

DESCRIPTION sodium channel associated protein 2 isoform a

[Source:RefSeq_petide;Acc:NP_208325]

GGTAACATAAATTTTTCACTCACTTTACTTGTCTTCCTTTACATCTTCATTATGTAATTTCTTTGTCTTGTTCTGCAGTA

CTTTACATTACTTCTATTAC

In100 candidate1164 (in intron):

LOCUS ENSG00000180116

DESCRIPTION CDNA FLJ40126 fis, clone TESTI2011028.

[Source:Uniprot/SPTREMBLAcc:Q8N818]

TATTAATGGTTTGATTTTCTGGTGTGGTAACATTTGAGTCGTTTTTCTTCCTTATTTGTATCTTTGCTCTACCAGTTAGT

TTTATACTTTCATGCATTTT

In100 candidate1165 (in intron):

LOCUS ENSG00000180116

DESCRIPTION CDNA FLJ40126 fis, clone TESTI2011028.

[Source:Uniprot/SPTREMBLAcc:Q8N818]

TTTATATTTATCTATAGGAAAACATAAATTGTTTAACAAGTTGGACTTACTTGTCTTAATTCTTTTCAGTTTTTTAGGAT

AATTGCTCTCTGTCATCTCT

In100 candidate1166 (in intron):

LOCUS ENSG00000180116

DESCRIPTION CDNA FLJ40126 fis, clone TESTI2011028.

[Source:Uniprot/SPTREMBLAcc:Q8N818]

TAGGAAATTAAATAAAGAATAACAAATTTCCAAATTGCCTTAATATGTTTGTCTTTCTTCAGCTGCATTTACTATGATTA

CCTGAAGCTTTTCCTTTTTC

In100 candidate1167 (in intron):

LOCUS ENSG00000180116

DESCRIPTION CDNA FLJ40126 fis, clone TESTI2011028.

[Source:Uniprot/SPTREMBLAcc:Q8N818]

TTCATTTCTTGGCAAGTGTCTATTCTGTTGCTTGTTGTTTTTTAACGAATTTAAATTTCATTTTATTGTTTACCACAGGC

TAATTTTTCTTTGTGGTTTA

In100 candidate1168 (in intron):

LOCUS ENSG00000004948

DESCRIPTION Calcitonin receptor precursor (CT-R).

[Source:Uniprot/SWISSPROTAcc:P30988]

TTTTGTTTTCATAGAATTTCAAGCTCACAAAAAAGTCGCAAGAATAGTAACAAAAAATCCTGTATGTTCTTTATCCAGAT

TTACCAATCCATGTTCTTTA

In100 candidate1169 (in intron):

LOCUS ENSG00000145293

DESCRIPTION Enolase-phosphatase E1 (EC 3.1.3.77) (2,3-diketo-5-methylthio-1

phosphopentane phosphatase) (MASA homolog). [Source:Uniprot/SWISSPROT;Acc:9UHY7]

GGTAAGATTGTGGAAGAACTGGATCTTTTACAACTGATAAAAGCTAACTCTTGTATTTTCTTTTACTCTTTTATCCAGGA

CATTTTATTTCCTTACATCG

In100 candidate1170 (in intron):

LOCUS ENSG00000151229

DESCRIPTION Proton myo-inositol cotransporter (H(+)-myo-inositol

cotransporer) (Hmit). [Source:Uniprot/SWISSPROT;Acc:Q96QE2]

AGATTTGGCCCATGAGCTATAGGTTGCTGATTCCTTCTTTAACCATTAAGTATGTCTGTTTTTATCAGTTTCCTCAGTTT

TTATTCTTTGGACTAATTTG

In100 candidate1171 (in intron):

LOCUS ENSG00000166503

DESCRIPTION Hepatoma-derived growth factor-related protein 3 (HRP-3)

(Hepatma- derived growth factor 2). [Source:Uniprot/SWISSPROT;Acc:Q9Y3E1]

TAGTACTTCGTTGGATATTAGTTTTGTAACCTGTTTTCATTTTTGCTTTTATTTCACTAATACATCTTTATCCAGAATTT

TACTCAAAACTTTTCTGTTT

In100 candidate1172 (in intron):

LOCUS ENSG00000166503

DESCRIPTION Hepatoma-derived growth factor-related protein 3 (HRP-3)

(Hepatma- derived growth factor 2). [Source:Uniprot/SWISSPROT;Acc:Q9Y3E1]

TAGAACATCACAATTAACTTGTATCTATTTACGTATTCTTTCCAGTCCCTCTTCCTACCCAGAGATGAGCACTAATGTTG

TATTATTTCCTTTTCTTTTT

In100 candidate1173 (in intron):

LOCUS ENSG00000145284

DESCRIPTION stearoyl-CoA desaturase 4 isoform a

[Source:RefSeq_peptide;Acc:P_001032671]

GACAATATTGAGTCTTCCTATTTATGAGCATGGGATAACTCTTCATCTATTTAGTTCTTCTTTGATTTCTTTCATCAGTG

TTTTATGATCTTCCTCATAC

In100 candidate1174 (in intron):

LOCUS ENSG00000140600

DESCRIPTION SH3-containing GRB2-like protein 3 (Endophilin-3) (Endophilin-A)

(SH3 domain protein 2C) (EEN-B2). [Source:Uniprot/SWISSPROT;Acc:Q99963]

TCGTCTACTATGATTCCTACCACACCACTGCTGGTTAAACTTTTAACTCTCTGTTCACTATGTTTTATTTTCCAGCCTCT

CTTTCTCTGTCCAACTTTTT

In100 candidate1175 (in intron):

LOCUS ENSG00000140600

DESCRIPTION SH3-containing GRB2-like protein 3 (Endophilin-3) (Endophilin-A) (SH3 domain protein 2C) (EEN-B2). [Source:Uniprot/SWISSPROT;Acc:Q99963]

TTCATTGCCGTATCTTGGGAACACTATATCACCCATAGGCTTGTTAACACACTGTCACCTCTTCTGTCTTTGCAGAGAAG

TTTATTCCTCATTATTCCTC

In100 candidate1176 (in exon):

LOCUS ENSG00000179859

DESCRIPTION

TGGGGGTATAAACCTCAATTTTTGTAACCCTATTTTGGCATTTTCCTGTCCTTACATCTCAGCTTAAATGCTCCCTCTTT

TGGGAGGCCTTTTTCTGCCT

In100 candidate1177 (in intron):

LOCUS ENSG00000120662

DESCRIPTION Peptide chain release factor 1, mitochondrial precursor (MRF-1) [Source:Uniprot/SWISSPROT;Acc:O75570]

TAGTTCTTTTTCCTACCCTTTTTTCTGATTGTAAAAAAATTATTTTTTTAACTTTTGAGAGCTCTTTCTTGTTCTCAGAA

TTGTTTTCTCATAGCATCCT

In100 candidate1178 (in intron):

LOCUS ENSG00000145734

DESCRIPTION transcription factor-like nuclear regulator [Source:RefSeq_peptde;Acc:NP_060899]

CCACATCTGTTGATTACTGAGAAAGGAGTGTTGAAGTCTCCATCTGTAACTGTGGATTTGCCTGTTTCTTTTTTTCAGTT

CTATTTGTTTTTGCTCCATA

In100 candidate1179 (in intron):

LOCUS ENSG00000143797

DESCRIPTION Membrane-bound O-acyltransferase domain-containing protein 2 (E 2.3.-.-) (O-acyltransferase domain-containing protein 2). [Source:Uniprot/WISSPROT;Acc:Q6ZWT7]

TCTTATGAACCAAAATATGGGATGTAAAGAACTGTAACCCACATAGTCTTGTTGCCCTTGCTTTTAAGTGCTTTTCAGAA

TTATTTGAATTACTTTTTTT

In100 candidate1180 (in intron):

LOCUS ENSG00000156218

DESCRIPTION ADAMTS-like protein 3 precursor (ADAMTSL-3) (Punctin-2). [Sourc:Uniprot/SWISSPROT;Acc:P82987]

CTTCATTTATTCAAGTATATTTTAGACCCCTTAGTAAAGTTTTGTAACTTAATAATGAGCTTTATTTCTTTTCAGGTCTA

TACCTACATCTATGTACTTT

In100 candidate1181 (in intron):

LOCUS ENSG00000156218

DESCRIPTION ADAMTS-like protein 3 precursor (ADAMTSL-3) (Punctin-2). [Sourc:Uniprot/SWISSPROT;Acc:P82987]

GTAGGCATGCTTCATTATTTTTTATTCTTTTTTCTTTTGTCTCCTCTAACTGTATTTTAAAATACCCTGTTTTCAGGCTC

ACCAGTTCTTTCTTCTGGTT

In100 candidate1182 (in intron):

LOCUS ENSG00000065989

DESCRIPTION cAMP-specific 3',5'-cyclic phosphodiesterase 4A (EC 3.1.4.17)

(PDE2) (PDE46). [Source:Uniprot/SWISSPROT;Acc:P27815]

CTACAAAATAAATAAATTTTAAAAGAAATCCATGCTCTGCTACTAATAACCATAGTATTTATCCTTTATGTGCCTCAGTT

TCTTTCTTTCTTCCTTCCTT

In100 candidate1183 (in intron):

LOCUS ENSG00000121236

DESCRIPTION Tripartite motif-containing protein 6 (RING finger protein

89).[Source:Uniprot/SWISSPROT;Acc:Q9C030]

AGAAATTCCCAACTTATGTGGTTTCCTCCATTTCTACACTCTCATAACCCTGGATCCCAATCTACTTCTTTCAGGCTTTA

CCTCGGCTTTTCTTTGTTTA

In100 candidate1184 (in intron):

LOCUS ENSG00000121236

DESCRIPTION Tripartite motif-containing protein 6 (RING finger protein

89).[Source:Uniprot/SWISSPROT;Acc:Q9C030]

TTTTCATATAACTCACTTCTGATTTTTCCATTTTTTTTCCTACTGTTCTTCTTTCTTTCATTAGTCTTACACCAGTCTTT

ATACACTTTGACTCATGTTT

In100 candidate1185 (in intron):

LOCUS ENSG00000018236

DESCRIPTION Contactin-1 precursor (Neural cell surface protein F3)

(Glycoprtein gp135). [Source:Uniprot/SWISSPROT;Acc:Q12860]

CTTAAAATTCACATTCTTTTTATTCTTCCATCTAACCTATAAACTTATTTTTCTGGTACTGTCAACTATGTTTCTCAGGC

CAGTCTATTACTTTCTATTT

In100 candidate1186 (in intron):

LOCUS ENSG00000189136

DESCRIPTION CDNA FLJ43276 fis, clone KIDNE2011532, moderately similar to Hoo sapiens melanoma-associated chondroitin sulfate proteoglycan 4. [Source:Uiprot/SPTREMBL;Acc:Q6ZUW4]

ACAACTAAAATTAATTGAAATTAACATTTCAATGTAAGTTGTTCTGGTCTTTTTCTGTGCTTATTTCAGCATAATTTAAA
TTCTTTCTATTCCTTTGTGT

In100 candidate1187 (in intron):

LOCUS ENSG00000168038

DESCRIPTION

ATTCTAACATAATCATGCAAACTTTGTTTTGATTAATATTTGCCTGCTATATGTTTTTTCATTCTTTTCCTTCAGACATC
TACTTTTATGTTTTAGATCT

In100 candidate1188 (in intron):

LOCUS ENSG00000168038

DESCRIPTION

ATTCTTCTAACCCATGAGCATAGGATATTTTTCTATTTATTTTTGTCCTCTTCAGTTTCTTATGTTTTGAAGTTTTCCTT
TTAGAGGTATTTTACCTCCT

In100 candidate1189 (in intron):

LOCUS ENSG00000168038

DESCRIPTION

CATCAAGTTAACTAATGAACAAGGTATAGCTTTCCATTTATTTAAGTCCTTTTTACTTTCAGCAATATTTTGTATTTTTC

ATGCACATGTCCTTCATCCT

In100 candidate1190 (in intron):

LOCUS ENSG00000168038

DESCRIPTION

TTATCTAAATGATAACCTTCCTTGCCTTATTTTTATTCATTTCTACCTTATTTATATTCTTTAGGTTACCAGTCCTATTC

TTTTTTAGATGGAGCCTCTC

In100 candidate1191 (in intron):

LOCUS ENSG00000176371

DESCRIPTION Zinc finger and SCAN domain-containing protein 2 (Zinc finger

potein 29 homolog) (Zfp-29). [Source:Uniprot/SWISSPROT;Acc:Q7Z7L9]

TCTTACTGTTAGCCACCCTGGGTTAGTTTTTGTTGGCCCATGGTGTAACTTTCACATGTTCTTATATCCTTACAGGTTTT

ATCTTTAAGTATTAGTTCTT

In100 candidate1192 (in intron):

LOCUS ENSG00000189308

DESCRIPTION lin-54 homolog [Source:RefSeq_peptide;Acc:NP_919258]

GTTCCTGTTTTAAGTGAATTAACATCAAGTTGCCTTTTTCTGATACCAGTTATCCTCAGGAATTGCGGATTTTCTGTATG

AATTTTTTTTTTTTTTTTTT

In100 candidate1193 (in intron):

LOCUS ENSG00000138663

DESCRIPTION COP9 signalosome complex subunit 4 (Signalosome subunit 4) (SGN)

(JAB1-containing signalosome subunit 4). [Source:Uniprot/SWISSPROT;Acc:Q9T78]

TAACCTCTAGGCCTCTTACTCTGTCTTTTCCATTTCAATGCTTTATTCTGGATTAGTTCTTCAGGCCTACCTTCTAGTTT

ATCACTTCTTCAACTGTGTT

In100 candidate1194 (in intron):

LOCUS ENSG00000138663

DESCRIPTION COP9 signalosome complex subunit 4 (Signalosome subunit 4) (SGN)

(JAB1-containing signalosome subunit 4). [Source:Uniprot/SWISSPROT;Acc:Q9T78]

TATTTGAAATATCTCACTCTGGCCATAACCTTTTTCTTGTTTCTCTAGTGGTCTGTGGTCTGTCTCCGTTACTCTCAGTT

CAATTCTTCCTTGACCTTAT

In100 candidate1195 (in intron):

LOCUS ENSG00000182606

DESCRIPTION Trafficking kinesin-binding protein 1 (106 kDa O-GlcNAc

transfease- interacting protein). [Source:Uniprot/SWISSPROT;Acc:Q9UPV9]

CCTCTGCTGCTTCCTAAAATGGATTAAAAATAACTTCCTAATATATTCTCCCTTCCTTTATATATCAGTTGCCCTGTTCA

CTTGGTTGCCCTTTTCATTT

In100 candidate1196 (in intron):

LOCUS ENSG00000165966

DESCRIPTION PDZ domain-containing RING finger protein 4 (Ligand of Numbproein X 4) (SEMACAP3-like protein). [Source:Uniprot/SWISSPROT;Acc:Q6ZMN7]

TAAGTATGTAACTTGTTTCTTCACTATTTCTTCTACTAACAATTTTTCTTTCTCAGCCCCTCCATTTCTGTCTGCTAAAT
ATATCCCATCTTATTATATT

In100 candidate1197 (in intron):

LOCUS ENSG00000165966

DESCRIPTION PDZ domain-containing RING finger protein 4 (Ligand of Numbproein X 4) (SEMACAP3-like protein). [Source:Uniprot/SWISSPROT;Acc:Q6ZMN7]

CCTTCTTTGCTTATAGATAGGTTAATATAATAACATCATTTATAGTCTCTTTGGTGTTCCAGCAGTTGTTAGTCTTTAAC
ATAGCTTTTGTTCTTCCCCT

In100 candidate1198 (in intron):

LOCUS ENSG00000165966

DESCRIPTION PDZ domain-containing RING finger protein 4 (Ligand of Numbproein X 4) (SEMACAP3-like protein). [Source:Uniprot/SWISSPROT;Acc:Q6ZMN7]

GAATAAGTAAAAACTGAACATGCTAGAGGAATTCTTAGAATGAGTAACTTCATAGATAATTTTGTTTTAATTTTACAGTT
CATTTTTTGATTACTTTTCT

In100 candidate1199 (in intron):

LOCUS ENSG00000158528

DESCRIPTION Neurabin-1 (Neurabin-I) (Neural tissue-specific F-
actinbindingprotein
I) (Protein phosphatase 1 regulatory subunit 9A).
[Source:Uniprot/SISSPROT;Acc:Q9ULJ8]

CTATTGCACAAGAAGAATAAGTTAGGATTTTATGGAAAGTAACTGTCTCTACTGTTTTGCCTGTTTACATATCTTCAGTA

ATGTATACCTTTTTCCTCTC

In100 candidate1200 (in intron):

LOCUS ENSG00000158528

DESCRIPTION Neurabin-1 (Neurabin-I) (Neural tissue-specific F-

actinbindingprotein

I) (Protein phosphatase 1 regulatory subunit 9A).

[Source:Uniprot/SISSPROT;Acc:Q9ULJ8]

CACTTTGTCCCAAATTATATTTTGTCTCATAATAAAAAATTAACTCTGGCTTTCTTTTGCTTTATATTTGTTTCACAGCA

TCTATTTTTTCCATCTTATG

In100 candidate1201 (in intron):

LOCUS ENSG00000173085

DESCRIPTION Para-hydroxybenzoate--polyprenyltransferase, mitochondrial

precrsor (EC 2.5.1.-) (PHB:polyprenyltransferase) (hCOQ2).

[Source:Uniprot/SWISPROT;Acc:Q96H96]

AAGACCCTCTGATTCCTCTCTCCCTCCTCCTAAAACTAGCTAACTAACTTAGATAGTTTTGTTTTATGCCTTTGCAGGAC

CCTGTGCTTGTCCTGTTGTT

In100 candidate1202 (in intron):

LOCUS ENSG00000134283

DESCRIPTION Periphilin-1 (Gastric cancer antigen Ga50).

[Source:Uniprot/SWISPROT;Acc:Q8NEY8]

CTAAGAATCTGATAGTTTGCAAGACATAGCCCCTAACTTTATCATCTCTTATGATTATGCTGTTTTTTTTCCCTGCAGTG

TCCTTTTTTTCCTACCTGGG

In100 candidate1203 (in intron):

LOCUS ENSG00000170289

DESCRIPTION Cyclic nucleotide-gated cation channel beta 3 (CNG channel beta3) (Cyclic nucleotide-gated channel beta 3) (Cone photoreceptor cGMP- gatedchannel subunit beta) (Cyclic nucleotide-gated cation channel modulatory suunit). [Source:Uniprot/SWISSPROT;Acc:Q9NQW8]

AGGTGTTGTAACTTCTAGATCTATTCTCCATATGCTTTTCTTACCTGATGTAACTTCAGTCCTGTCTTACTTTCCTTGTG

TACTTTTACACCTGTTGTCC

In100 candidate1204 (in intron):

LOCUS ENSG00000136383

DESCRIPTION Alpha-protein kinase 3 (EC 2.7.11.-) (Muscle alpha-protein kinae). [Source:Uniprot/SWISSPROT;Acc:Q96L96]

CCACATACCTAGGTGTGATACCTTATTGTCTATCTTCTTCTAACCATGGTAACTGCCTTTTAAAATTTTTTTCTGCAGTC

ATTTAAGTTCTCCTTTTACC

In100 candidate1205 (in intron):

LOCUS ENSG00000151694

DESCRIPTION ADAM 17 precursor (EC 3.4.24.86) (A disintegrin and metalloprotinase domain 17) (TNF-alpha-converting enzyme) (TNF-alpha convertase) (Snak venom-like protease) (CD156b antigen). [Source:Uniprot/SWISSPROT;Acc:P7853]

AGGCTGGTCTCAAACCCCTAACCTCGTGGTCCACCTGCCTCATCTTTTTTTTTTTTTTTTTTTTTTGAGACAGAATTT

CTCTCTTGTTGCCTAGGCTG

In100 candidate1206 (in intron):

LOCUS ENSG00000102763

DESCRIPTION OTTHUMP00000018323. [Source:Uniprot/SPTREMBL;Acc:Q5JUE1]

TTGTTGTATCCTACCTCTCCATTCTTGTTGCCATAACCTTACTGTAGGACTTTTTCCTTTTATAGGCAGATTATTGAAGT

AGCTTTCTGTTTCCTGTCTC

In100 candidate1207 (in intron):

LOCUS ENSG00000176571

DESCRIPTION cyclic nucleotide binding domain containing 1

[Source:RefSeq_petide;Acc:NP_775809]

TACCTTAGTGCAAAACACTATCATCTGTCACCTAGCTCACTGTAATAACCTCCTACTGATTTCTCTGCTTCTACTCAGGC

TTTCCTCAATCATTTATTTT

In100 candidate1208 (in intron):

LOCUS ENSG00000176571

DESCRIPTION cyclic nucleotide binding domain containing 1

[Source:RefSeq_petide;Acc:NP_775809]

TTTTTCTTAGCTTGCTTATGATTATTTTGAATTTTTTGTTGGGTAACTCCTATACCGCCATTTCTTTAGTGTCAGTTTCT

GAAGTTTTATTTATTTATTT

In100 candidate1209 (in intron):

LOCUS ENSG00000139174

DESCRIPTION Prickle-like protein 1 (REST/NRSF-interacting LIM domain protei

1). [Source:Uniprot/SWISSPROT;Acc:Q96MT3]

AGTGAATTTCTTCAAACTTCTTGGTTTTAACCAAATGATATTGCTTCTTCTTTTCCTCATTTTAAAGTTCAGTCATTTAC

TCAACAGATTTTTTCTTTCT

In100 candidate1210 (in intron):

LOCUS ENSG00000156103

DESCRIPTION Matrix metalloproteinase-16 precursor (EC 3.4.24.-) (MMP-16)

(Mmbrane-type matrix metalloproteinase 3) (MT-MMP 3) (MTMMP3) (Membrane-

typematrix

metalloproteinase) (MT3-MMP) (MT3MMP) (MMP- X2).

[Source:Uniprot/SWSSPROT;Acc:P51512]

AACATACAAAGATGATAACAAAAATGTCTGTGTTTCTGCAACCTATGATATTTTCCTTTTAATCTTTTTACTCTGCAGTT

TTTTGCTGCTCTGATTCATC

In100 candidate1211 (in intron):

LOCUS ENSG00000156103

DESCRIPTION Matrix metalloproteinase-16 precursor (EC 3.4.24.-) (MMP-16)

(Mmbrane-type matrix metalloproteinase 3) (MT-MMP 3) (MTMMP3) (Membrane-

typematrix

metalloproteinase) (MT3-MMP) (MT3MMP) (MMP- X2).

[Source:Uniprot/SWSSPROT;Acc:P51512]

AAAATATGTTGGATCTCTTAACTACATTCTAAATTTTCCATTTTCTCCATCCCGTGTCTCAATATTCTTTAAATACAGCA

TTACTTATTCTCTCGGTCAT

In100 candidate1212 (in intron):

LOCUS ENSG00000156103

DESCRIPTION Matrix metalloproteinase-16 precursor (EC 3.4.24.-) (MMP-16) (Mmbrane-type matrix metalloproteinase 3) (MT-MMP 3) (MTMMP3) (Membrane-typematrix metalloproteinase) (MT3-MMP) (MT3MMP) (MMP- X2). [Source:Uniprot/SWSSPROT;Acc:P51512]

ACCTACAACATATATAACTATTTGCTGTTGTTATTGGTTTTTTGTACTTGTTTTTGTCTTCCCCTCTTCCAGTGCCTTCT

CTGGTTTTAGTTGACCATTT

In100 candidate1213 (in intron):

LOCUS ENSG00000114857

DESCRIPTION NK-tumor recognition protein (Natural-killer cells cyclophilinelated protein) (NK-TR protein). [Source:Uniprot/SWISSPROT;Acc:P30414]

TGTTTCTTTCTCCCATTTAAATGTGATATATTTAACTTGCTTACTATTTTTTTCCTATGTATTTTATTTCAGTTGGTCGC

ATTATGTTTCAGCTCTTCTC

In100 candidate1214 (in intron):

LOCUS ENSG00000073417

DESCRIPTION High-affinity cAMP-specific and IBMX-insensitive 3',5'-cyclic posphodiesterase 8A (EC 3.1.4.17). [Source:Uniprot/SWISSPROT;Acc:O60658]

TTTTAAGGGTTTTTTTCTGGTAACCTAGTATATCATGTTTTTCTAATATTTATAAATATTTTCTTTCTTTCCATTCAGAC

TTTATTTTTTATCTATTTGA

In100 candidate1215 (in intron):

LOCUS ENSG00000158560

DESCRIPTION Cytoplasmic dynein 1 intermediate chain 1 (Dynein intermediate hain 1, cytosolic) (DH IC-1) (Cytoplasmic dynein intermediate chain 1). [Sorce:Uniprot/SWISSPROT;Acc:O14576]

ATGCTATGTGGTTTGCACACTTGATCCTTCCATCTCATGACAATAACTTCCATAGCCATCTCTCTGGATTTTTTTCAGCT

ATTTGTTTTCATGATTCTGC

In100 candidate1216 (in intron):

LOCUS ENSG00000163625

DESCRIPTION WD repeat and FYVE domain-containing protein 3 (Autophagy-linke FYVE protein) (Alfy). [Source:Uniprot/SWISSPROT;Acc:Q8IZQ1]

CTATTTTAAATCACCTTAACTAGTCCTTGAGCTGCCTCTAAATCATTTATCTTCTAAACTCTTATTCAGCACCGTTTACT

CATTTATGAATCATTTTTTT

In100 candidate1217 (in intron):

LOCUS ENSG00000163625

DESCRIPTION WD repeat and FYVE domain-containing protein 3 (Autophagy-linke FYVE protein) (Alfy). [Source:Uniprot/SWISSPROT;Acc:Q8IZQ1]

CATGTTTGTAACTGTGTTCTATTTGTTGCATTTGTTCTTTGTTCTTCCTCCCTTCTTTTCTTGCCTTCTCAGGTTTTAAC

AGCATTTTCTATGATTTCTT

In100 candidate1218 (in intron):

LOCUS ENSG00000163625

DESCRIPTION WD repeat and FYVE domain-containing protein 3 (Autophagy-linke FYVE protein) (Alfy). [Source:Uniprot/SWISSPROT;Acc:Q8IZQ1]

CAAACAAACCTCTGGTCTAGGGCCCACATAGTAGTAGGGACCTTGGGTCTAACTGCTCTTGAACATATTTTCCACCAGTC

CTTCTCTGTGTAGTTGCTTA

In100 candidate1219 (in intron):

LOCUS ENSG00000163625

DESCRIPTION WD repeat and FYVE domain-containing protein 3 (Autophagy-linke

FYVE protein) (Alfy). [Source:Uniprot/SWISSPROT;Acc:Q8IZQ1]

AGTAACATTGGCTATTGTCTGTTTCCCTCATATTCTTTCAAGTTATACTTCTTTTGCTAGCTTTCAGGTGTTAATATCCT

GGTTCTTACATCTTTCTTAC

In100 candidate1220 (in intron):

LOCUS ENSG00000163625

DESCRIPTION WD repeat and FYVE domain-containing protein 3 (Autophagy-linke

FYVE protein) (Alfy). [Source:Uniprot/SWISSPROT;Acc:Q8IZQ1]

CTGGATAGAAATGTTAACACATACTGAATTACTTCCTTCTTTGTTAATGCAGCATGTTTATCAGTTGCTGATTTTGCACT

TACTGTTATATTCCTCTTCT

In100 candidate1221 (in intron):

LOCUS ENSG00000198001

DESCRIPTION Interleukin-1 receptor-associated kinase 4 (EC 2.7.11.1) (IRAK-)

(NY- REN-64 antigen). [Source:Uniprot/SWISSPROT;Acc:Q9NWZ3]

AGTTTAGAAAGTAAACCTCTCCACTATGGAGGAGACATTGGTAGTAGGTAACCTCTTTTTGATTGTTCCTTTTGCAGTTT

TGTCTTCTTTGTCCCATATG

In100 candidate1222 (in intron):

LOCUS ENSG00000115750

DESCRIPTION TATA box-binding protein-associated factor RNA polymerase I

subnit B (TATA box-binding protein-associated factor 1B) (TBP-associated facto

1B) (TBP-associated factor RNA polymerase I 63 kDa) (TAFI63) (Transcriptio

factor SL1). [Source:Uniprot/SWISSPROT;Acc:Q53T94]

AGCTTTATTACACTTCTAATTATGACCTCTTTAACATGTGTTTTTTTTTCCTTTAGACAGACATTACTTACATTCCTTT

TTTCTTTTTTTTTTCTCCT

In100 candidate1223 (in intron):

LOCUS ENSG00000102780

DESCRIPTION Diacylglycerol kinase eta (EC 2.7.1.107) (Diglyceride kinase et)

(DGK-eta) (DAG kinase eta). [Source:Uniprot/SWISSPROT;Acc:Q86XP1]

GCCGTGATGCTTCCTCTAGCGCTATCTGGGCTTTAACCAATCCATTCCTTTCACATTTTGATTTTCTTTTCTTATCAGAT

TTTTCTTTGCTTTGTTCTTT

In100 candidate1224 (in intron):

LOCUS ENSG00000102780

DESCRIPTION Diacylglycerol kinase eta (EC 2.7.1.107) (Diglyceride kinase et)

(DGK-eta) (DAG kinase eta). [Source:Uniprot/SWISSPROT;Acc:Q86XP1]

AACTTTTTTTGAAGAAGTTATCACTGTTCTATAATGTTTAGTGTTTATAACTTATTCCTGTGTTATCCCTATTATCAGAG

TAGGTTTTTCTCCTTTTCAT

In100 candidate1225 (in intron):

LOCUS ENSG00000173811

DESCRIPTION Coiled-coil domain-containing protein 13.

[Source:Uniprot/SWISSROT;Acc:Q8IYE1]

TTCTTCCTATTCATGTGCATGAGATAACTATTTGTTTAGTTCTTGATTTTATCAGAGTTTTATAGTTTATAGTCTTTTCC

CAGAGTTTTATATTTCTCCT

In100 candidate1226 (in intron):

LOCUS ENSG00000170776

DESCRIPTION A-kinase anchor protein 13 (AKAP 13) (Protein kinase A-anchorin

protein 13) (Breast cancer nuclear receptor-binding auxiliary protein) (Huan

thyroid-anchoring protein 31) (Guanine nucleotide exchange factor Lbc) (KAP-Lbc)

(LBC oncogene) (P47) (Lymphoid [Source:Uniprot/SWISSPROT;Acc:Q1280]

AGAGGAAAAAAGGTGTAACTCTTTTTAAGGTTTTTCGTCATGTTTAACTTATGTGTTATTCCCAGATTTTTCTCCGATGT

TCATACTGTCTCTTGAAACC

In100 candidate1227 (in intron):

LOCUS ENSG00000170776

DESCRIPTION A-kinase anchor protein 13 (AKAP 13) (Protein kinase A-anchorin

protein 13) (Breast cancer nuclear receptor-binding auxiliary protein) (Huan

thyroid-anchoring protein 31) (Guanine nucleotide exchange factor Lbc) (KAP-Lbc)

(LBC oncogene) (P47) (Lymphoid [Source:Uniprot/SWISSPROT;Acc:Q1280]

TGAGAGAAATCTTTCTGGAATATTTGCTGCCTTTAACACCCCCTCACCCCCCATTTTTCTTTCAGTATATACCACATAAG

ACTGTCTTTTTTTTTTTCTT

In100 candidate1228 (in intron):

LOCUS ENSG00000164331

DESCRIPTION Ankyrin repeat family A protein 2 (RFXANK-like 2).

[Source:Unipot/SWISSPROT;Acc:Q9H9E1]

TACCCTGAATAATAAAACTATTCTCTTAGGATAACATCACCCAAGATTAATATATTTTCTTCCAGAACTTTCACTATGGT

TATGTAGACTTTTTTTTTTC

In100 candidate1229 (in intron):

LOCUS ENSG00000196636

DESCRIPTION ACN9 protein homolog, mitochondrial precursor.

[Source:Uniprot/WISSPROT;Acc:Q9NRP4]

ACAACTGAAGTTTTACATAGGGAGTAGAATTAACTGAAAGAGAGCTTCTGATTGTTTTGTTAAGCCCCTGTTTTTCAGTT

GGTGCACTTTTTTTTTCCTT

In100 candidate1230 (in intron):

LOCUS ENSG00000139173

DESCRIPTION Transmembrane protein 117. [Source:Uniprot/SWISSPROT;Acc:Q9H0C3

CCTGCTAAATAATAAAAGTTCTCTAGAACGTTTTAACTGTAATCAACCTCTTCTTACTTCCATGCGATTGCTGTTCAGGG

TTCTAGTTCTTTTTTTGGTT

In100 candidate1231 (in intron):

LOCUS ENSG00000139173

DESCRIPTION Transmembrane protein 117. [Source:Uniprot/SWISSPROT;Acc:Q9H0C3

CTTTCTTTTGATGATGTTGGTGGCAATCTGTCTCTCATTAACTTTGCTTCCTATTTCTCCTCTATTACTTTTTTTCAGTT

CTTCTCATTCCTTTTTAATG

In100 candidate1232 (in intron):

LOCUS ENSG00000139173

DESCRIPTION Transmembrane protein 117. [Source:Uniprot/SWISSPROT;Acc:Q9H0C3

AGATGGACTCTCACTAGGGAGGCTTCTTATAACTATTTAATATATATGTGCTTTTTCATTATATCCAGACTTATATTTGC

TTATTTATTTCCTCCATATC

In100 candidate1233 (in intron):

LOCUS ENSG00000139173

DESCRIPTION Transmembrane protein 117. [Source:Uniprot/SWISSPROT;Acc:Q9H0C3

TGCTTTAATATAAGAATTTTTAAAAATGTATCCTTAACTTATTAAAGTCTTACATTCACTTATTTTCCCTTCTTCCAGTT

TGCTTTCTTTTATTTTAATT

In100 candidate1234 (in intron):

LOCUS ENSG00000139173

DESCRIPTION Transmembrane protein 117. [Source:Uniprot/SWISSPROT;Acc:Q9H0C3

AGAAATCTCTTTGTGAATATTTAACATTCTCCTCAAGTCTCATCATGTTTTCCAGTCAACTCCCAGATAATTTGTCTTTT

TTTTTTTTACTTTTATTTTA

In100 candidate1235 (in intron):

LOCUS ENSG00000180694

DESCRIPTION transmembrane protein 64 [Source:RefSeq_peptide;Acc:NP_00100849]

CCTTTGACTAACATTTTTAAATAATCATCATGTTTTCCTCTGATTTTGTTTTTTCCTAAGTACAGGCAATTACTTGTTCA

CAATCTCTTGTTAATTCTTT

In100 candidate1236 (in intron):

LOCUS ENSG00000079805

DESCRIPTION Dynamin-2 (EC 3.6.5.5). [Source:Uniprot/SWISSPROT;Acc:P50570]

CCCTGAAAAATTTGCAACTCATTTCATGGCTAACTTAGATCTCTCATTTTTTATCCCAGTTGTTACAAGTATTCACATAT

TTTTATTTTTATTTATTTTT

In100 candidate1237 (in intron):

LOCUS ENSG00000184613

DESCRIPTION Protein kinase C-binding protein NELL2 precursor (NEL-like protin

2) (Nel-related protein 2). [Source:Uniprot/SWISSPROT;Acc:Q99435]

TGCAATTCCCACAAAGTGGAGAAATAATAACTTTGCTTTTTCTTGGTCTCTTCCTCTCTCTTTGCTAGTCATTAACAGTC

CCTCTTTGGTATTTGATTTT

In100 candidate1238 (in intron):

LOCUS ENSG00000038102

DESCRIPTION Rho-guanine nucleotide exchange factor

[Source:RefSeq_peptide;Ac:NP_001073948]

TGAATATTTTTAAAGTATAGAAATTATGTATTAGGCATGCAATTAACAAATGTTTCCATTTTGCCATATTTATTTCAGAT

CTTCGCCTTTTTGCTTTTTT

In100 candidate1239 (in intron):

LOCUS ENSG00000138639

DESCRIPTION Rho GTPase activating protein 24 isoform 1

[Source:RefSeq_peptie;Acc:NP_001020787]

TGCCTGGAGCAACATGAGCAAAGCTAACTTTTAAAATAAGCTCTTTCATTTTTTGCTTTTTAAATCTCCTATCATCAGTT

TACGTTTCACTTATGTTTTA

In100 candidate1240 (in intron):

LOCUS ENSG00000138639

DESCRIPTION Rho GTPase activating protein 24 isoform 1

[Source:RefSeq_peptie;Acc:NP_001020787]

ATTTGATAAATATCTCTAATTTTCTAAAATGCTTAAACTCCCTAACACATTGTGTCCTTGCTTTCTTCCTTCTTTCAGCT

TTACACTTTTCTTCTAGACT

In100 candidate1241 (in intron):

LOCUS ENSG00000147606

DESCRIPTION solute carrier family 26, member 7 isoform a

[Source:RefSeq_pepide;Acc:NP_439897]

TGGATTATCTCTCTTTAACATTTAAACTTTTGACCATCTCTTTAATTTTGAAATTTTCTTTTCTCACACCTTCAGAGATT

CCTTGCTAATCTGGTTCTTC

In100 candidate1242 (in intron):

LOCUS ENSG00000147606

DESCRIPTION solute carrier family 26, member 7 isoform a

[Source:RefSeq_pepide;Acc:NP_439897]

ATATACTCATCACCTAGATTCTAAAATTAACATTTACTATATTTGCCTTATTAAATATCTATTTATTCCTCTATTCAGTC

ATCAGTCTATCTTATTTTTT

In100 candidate1243 (in intron):

LOCUS ENSG00000147606

DESCRIPTION solute carrier family 26, member 7 isoform a

[Source:RefSeq_pepide;Acc:NP_439897]

TATTACAATATTTATAAATTTTAACAATATCACCTTTATGGTTTTTGTTCCTTTTTATCTTTTTCTTTAAGTCTTCAGGT

GTTTTGCTTTTTAAGCCTAT

In100 candidate1244 (in intron):

LOCUS ENSG00000109339

DESCRIPTION Mitogen-activated protein kinase 10 (EC 2.7.11.24) (Stressactiated

protein kinase JNK3) (c-Jun N-terminal kinase 3) (MAP kinase p49 3F12)

[Source:Uniprot/SWISSPROT;Acc:P53779]

TCTTCGTCTCTTTTGATCTTTGTTGGTTTAACAAATCCAACAAGTCTCTGTTTTATCAGAGACTAGGATTGTAACCTCTG

CCTTTTTTTGTTTTCCATTT

In100 candidate1245 (in intron):

LOCUS ENSG00000109339

DESCRIPTION Mitogen-activated protein kinase 10 (EC 2.7.11.24) (Stressactiated

protein kinase JNK3) (c-Jun N-terminal kinase 3) (MAP kinase p49 3F12)

[Source:Uniprot/SWISSPROT;Acc:P53779]

TATATGTACATAACAATTTTTAACACCTTTAATACTTTTTTCACTTTCCTTCCAGATGACTCCTTTATACATGTGCTAAT

TCATCATATGTCTTTTTTCT

In100 candidate1246 (in intron):

LOCUS ENSG00000189079

DESCRIPTION AT-rich interactive domain-containing protein 2 (ARID

domaincntaining

protein 2) (BRG1-associated factor 200) (BAF200).

[Source:Uniprot/WISSPROT;Acc:Q68CP9]

CCCTACCACTTTCCCCCTTGGTAATCACCATTCTGTTCTGTGTTTCTAACCATTTCTTAATTTTGTTTCATCTATCAGAC

TGTTTGTTTGTTTGTTTTTG

In100 candidate1247 (in intron):

LOCUS ENSG00000160746

DESCRIPTION transmembrane protein 16K (TMEM16K), mRNA

[Source:RefSeq_dna;Ac:NM_018075]

CTCCCACTCTGTGGGTTGTCTGTTAACTCTGCTGATTGTTTCTTTTGCTGTGCAGAAGCTTTTTAGTTTAATTAAGTCCC

ATCTATTTATCTTTATTTTT

In100 candidate1248 (in intron):

LOCUS ENSG00000164715

DESCRIPTION Serine/threonine-protein kinase LMTK2 precursor (EC 2.7.11.1)

(emur tyrosine kinase 2) (Serine/threonine protein kinase KPI-2)

(Kinase/phophatase/inhibitor 2) (Apoptosis-associated tyrosine kinase 2) (Brain

enrichd kinase) (hBREK) (CDK5/p35-regulated k

[Source:Uniprot/SWISSPROT;Acc:Q8IWU]

TTCCCTTCTAATTAAAGAAATTTAACTAGCTTTTCTTTTCTCTCTTTTTTTTAAAAACCCATTTTTTTGCAGGATTCCTT

TTATTTTTAAATAAATTCTC

In100 candidate1249 (in intron):

LOCUS ENSG00000139218

DESCRIPTION SFRS2-interacting protein (Splicing factor, arginine/serine-ric

2- interacting protein) (SC35-interacting protein 1) (CTD-associated SR prtein

11) (Splicing regulatory protein 129) (SRrp129) (Renal carcinoma antign NY-REN-

40). [Source:Uniprot/SWISSPROT;Acc:Q99590]

TATTAACATCCTTTCCCACTTTTCTCATTTTTACCTTTTTATAAGCACCATTTTCCTAGTCTTTTAGGCCAGGATATTGT

CTCCTTCACTGTAGTTGCTC

In100 candidate1250 (in intron):

LOCUS ENSG00000163629

DESCRIPTION Tyrosine-protein phosphatase non-receptor type 13 (EC

3.1.3.48)(Protein-tyrosine phosphatase 1E) (PTP-E1) (hPTPE1) (PTP-BAS)

(Proteintyroine

phosphatase PTPL1) (Fas-associated protein-tyrosine phosphatase 1)

(FAP1). [Source:Uniprot/SWISSPROT;Acc:Q12923]

TAAATAGCATAACTTATTTTTTCTGCTTTTCTTTTTTTTCCTCAGATTACCTCATTAGTCAAACATGTTTCACACTTTTA

ACCTGTTAATCCCTTTCTTT

In100 candidate1251 (in intron):

LOCUS ENSG00000111371

DESCRIPTION amino acid transporter system A1

[Source:RefSeq_peptide;Acc:NP_01070952]

ATCCCTCTTCTTCTATGTTTTCTATACCTTAGGGAAGAAGATTCCATAACTTCTTTCTGTATTCAATTTTCATGTCAGAA

AATTCTTCTCTTGCTTTCAT

In100 candidate1252 (in intron):

LOCUS ENSG00000120658

DESCRIPTION proliferation-inducing protein 38 (PIG38), mRNA

[Source:RefSeq_na;Acc:NM_017993]

TCTCATCTGTAAAATTATGGTATGGGATCTCTAAAAACATAACTAGCTAACAACAAGTTCTCTGATTCTGTCTTCCAGAG

CTGTCCTCTATTTTTGTGAC

In100 candidate1253 (in intron):

LOCUS ENSG00000120658

DESCRIPTION proliferation-inducing protein 38 (PIG38), mRNA

[Source:RefSeq_na;Acc:NM_017993]

TGTTTTTCTCAATTAATCTAGGTAAAGGTTTGTCAATTTTGTTTAACTTTTCGAAAAACCAACTTTTCATTTCATCAGTC

ATTTGTATTGTCTTCATTTT

In100 candidate1254 (in intron):

LOCUS ENSG00000120658

DESCRIPTION proliferation-inducing protein 38 (PIG38), mRNA

[Source:RefSeq_na;Acc:NM_017993]

ATAAGATACTCTTTAAAAATATATTTTTGAAAACAAAACATATAACAAGAAGGTTAGTATCCTTTCACATTTTTGCAGAT

CTTTTTAGTGTCTTACTCTG

In100 candidate1255 (in intron):

LOCUS ENSG00000120658

DESCRIPTION proliferation-inducing protein 38 (PIG38), mRNA

[Source:RefSeq_na;Acc:NM_017993]

TCTGTGTTGTTTCTGTTAACCTGCGTTTTAATGCATTTACCCATTTATCTGCTTTATACTGACCTATTTTTCCAGGTCTC

CCTTCTTTGGAGTGAGTTTC

In100 candidate1256 (in intron):

LOCUS ENSG00000196367

DESCRIPTION Transformation/transcription domain-associated protein

(350/400kDa PCAF-associated factor) (PAF350/400) (STAF40) (Tra1 homolog).

[Source:Uiprot/SWISSPROT;Acc:Q9Y4A5]

GTAAAGTAAGTCTTGGGCCTTCACAACCAAAACATAACCAAGTTACTATTTACTGCTTCTATCAGGACAAAGCTCTCCAC

TTGTTTTCTGTTTGTCTGTC

In100 candidate1257 (in intron):

LOCUS ENSG00000197302

DESCRIPTION Zinc finger protein 720. [Source:Uniprot/SPTREMBL;Acc:Q7Z2F6]

TCATTTGGTCATGGGCTTTTTCTCATTGGATTTTTTTATAACTGCTTCAATCTCCTTTCTTATTATTGGTCTGTTCAGTG

TTTCTGTTTCTTTGTGATTT

In100 candidate1258 (in intron):

LOCUS ENSG00000197302

DESCRIPTION Zinc finger protein 720. [Source:Uniprot/SPTREMBL;Acc:Q7Z2F6]

AGTCTCTGGTAACCACTGTTATACTCTGCTTATATCAGCTTTTTTTTCCTTTTCTTTTTCTTTTTTTTTTTTAAGACAGA

GTCTTGCTCTGTTGCCCAGG

In100 candidate1259 (in intron):

LOCUS ENSG00000145700

DESCRIPTION Ankyrin repeat domain-containing protein 31 (Fragment).

[SourceUniprot/SPTREMBL;Acc:Q8N7Z5]

ACTTTATGGATTTCTCAAGCCTCTCTTAACATGTTTCTTTGTATTAGCCTTTTTTTTTTAATGTGCCAGTGTTAACATTC

TCAGTGGCTGTTCCCTTCTT

In100 candidate1260 (in intron):

LOCUS ENSG00000198742

DESCRIPTION Smad ubiquitination regulatory factor 1 (EC 6.3.2.-)

(Ubiquitinprotein

ligase SMURF1) (Smad-specific E3 ubiquitin ligase 1) (hSMURF1).

[ource:Uniprot/SWISSPROT;Acc:Q9HCE7]

CAAGCCAAAAATAACATTACTATTCCTTTTTTAAATTTGCCTATATATTTTCCTTTATTAGTGTTCTTTATTTCTCAGGT

TACTGTCTAATGTTCTTTTT

In100 candidate1261 (in intron):

LOCUS ENSG00000102804

DESCRIPTION TSC22 domain family protein 1 (Transforming growth factor beta--induced transcript 4 protein) (Regulatory protein TSC-22) (TGFB- stimulatd clone 22 homolog) (Cerebral protein 2). [Source:Uniprot/SWISSPROT;Acc:Q1514]

AGTCCCTGTAACTTTAAATATGTATCTGCCTATCTTATTCTCCTTTCAGCTTTTCCTTGATCAGATGCTATGTGCTTTGA
ACTTCCTTCTCTTATTTATT

In100 candidate1262 (in intron):

LOCUS ENSG00000102804

DESCRIPTION TSC22 domain family protein 1 (Transforming growth factor beta--induced transcript 4 protein) (Regulatory protein TSC-22) (TGFB- stimulatd clone 22 homolog) (Cerebral protein 2). [Source:Uniprot/SWISSPROT;Acc:Q1514]

GTCAATTCCTGATTGAGGGGTGTTTAAGTCTTTAACTATGATAGTGGATTTGACTAGTCCTCCTTACATTTCTATCAGTT
TTTGTCTCATATTTTGATGC

In100 candidate1263 (in intron):

LOCUS ENSG00000197275

DESCRIPTION DNA repair and recombination protein RAD54B (EC 3.6.1.-) (RAD54homolog B). [Source:Uniprot/SWISSPROT;Acc:Q9Y620]

AGAAACTGTTAACTAGACTCATATTTCCTCCCAAATCTGCTTTTTCTTTCGAAGGTCTTATCTCAGTTGGTCTGTGAATC
ATCTTTGATTTCTTCTCTCT

In100 candidate1264 (in intron):

LOCUS ENSG00000083635

DESCRIPTION Nuclear fragile X mental retardation-interacting protein 1 (Nucear FMRP-interacting protein 1). [Source:Uniprot/SWISSPROT;Acc:Q9UHK0]

TGTGTATTCTGCTGACTGGCCTAACTTAAGGATTGTTTTTCTTTGTTTATTTTGGTATCTCATTAGTTAGCATCCAGTT

CATTTTGTTGCCATTGTTTA

In100 candidate1265 (in intron):

LOCUS ENSG00000130158

DESCRIPTION Dedicator of cytokinesis protein 6.

[Source:Uniprot/SWISSPROT;Ac:Q96HP0]

AAATTCTCCATCTTTTGTCTCATTTTTGCTCATCTTTTCTTCTAACTTTAAAAGTTTCTGTTTGCTGACTTCCAGATTTG

ATCATCCATGTGTCTGCTTC

In100 candidate1266 (in intron):

LOCUS ENSG00000113163

DESCRIPTION Goodpasture antigen-binding protein (EC 2.7.11.9) (GPBP) (Collaen

type IV alpha-3-binding protein) (StAR-related lipid transfer protein 11

(StARD11) (START domain-containing protein 11).

[Source:Uniprot/SWISSPROT;cc:Q9Y5P4]

CCTGGCTTTTTGTTGTTGTTTCCCACGTTTTTAACTCTGTTTTTTGTTTTTTTAACTCTGTCAGCCCTCTTTTCATCACT

TGTCACCTTAATTCTTCTTT

In100 candidate1267 (in intron):

LOCUS ENSG00000166579

DESCRIPTION Nuclear distribution protein nudE-like 1 (Protein Nudel)

(Mitosnassociated

protein 1). [Source:Uniprot/SWISSPROT;Acc:Q9GZM8]

TGTGTACCATACCTACTCTTTAACCTCATCAAGAGTATTACCCCATTTTTTAGTGTGTTTGTTTTTATCAGCCATCTCTT

GCTTTCACTTCCTTTCTATT

In100 candidate1268 (in intron):

LOCUS ENSG00000164941

DESCRIPTION Integrator complex subunit 8 (Int8) (KAONASHI protein 1)

(KAONAHI1). [Source:Uniprot/SWISSPROT;Acc:Q75QN2]

TTAAGTTTGCCCTCTTTCTATAACTCTGGGCCCCTTGTTCCTTTCACAGTTTTCAGTTGATGCTTATTGTCAGGTATTAT

TTGTTTTTGTTTTTGTTTTT

In100 candidate1269 (in intron):

LOCUS ENSG00000133026

DESCRIPTION Myosin-10 (Myosin heavy chain 10) (Myosin heavy chain, nonmuscl

IIb) (Nonmuscle myosin heavy chain IIb) (NMMHC II-b) (NMMHC-IIB) (Cellularmyosin

heavy chain, type B) (Nonmuscle myosin heavy chain-B) (NMMHC- B).

[Surce:Uniprot/SWISSPROT;Acc:P35580]

TAACACATGAATTCTTAATTGTAGTTACTGGTTTTCTTTTTTTGTTTTGTTTTGTTTTGTTTTGTTTTTCAGTTTTAGAA

TTTTCATTTGGGTTTTTCCT

In100 candidate1270 (in intron):

LOCUS ENSG00000171241

DESCRIPTION SHC SH2 domain-binding protein 1.

[Source:Uniprot/SWISSPROT;AccQ8NEM2]

TTTCTGCCAATTGTTGAGAGAGGGGTGTTGAAGTCTCTAACTATAACTGAATTTGTTTGTTTGCCTTTTCAGTACTATTA

GTTTTTGCTTCACATATTTT

In100 candidate1271 (in intron):

LOCUS ENSG00000163810

DESCRIPTION Protein-glutamine gamma-glutamyltransferase 4 (EC 2.3.2.13)

(Trnsglutaminase-4) (TGase-4) (Prostate transglutaminase) (TGP) (TG(P))

(Prostte-specific transglutaminase) (Fibrinoligase).

[Source:Uniprot/SWISSPROT;Ac:P49221]

AATAAATAAATAAATAAAAATAAATAATTAAAAAAATAAGTGTTAGCATAACACTTTCATCTACTATCTATATTCCAGTT

TGGTCACTTTTTTTTTAATT

In100 candidate1272 (in intron):

LOCUS ENSG00000156471

DESCRIPTION Phosphatidylserine synthase 1 (EC 2.7.8.-) (PtdSer synthase 1)

PSS-1) (Serine-exchange enzyme I). [Source:Uniprot/SWISSPROT;Acc:P48651]

GAATTAACCTCTTTGCTGTATTAAATATTCTGATTCTTGATCACGCTGCATCTCTCTTTATTCCCATTTTCTTCTCAGTC

TTTCTATGCACTTTGCTATT

In100 candidate1273 (in intron):

LOCUS ENSG00000145063

DESCRIPTION CDNA FLJ33534 fis, clone BRAMY2007411.

[Source:Uniprot/SPTREMBLAcc:Q8N2B8]

AGTGGTTTTTTTTTTTAACTTTATTGGTATATTTTTACCCCTTTCTTTGCTCATCTCAGGCCTTCCTTCTGCGTTCAA

TTTTCTTCTTTTTGAAGCAC

In100 candidate1274 (in intron):

LOCUS ENSG00000118777

DESCRIPTION ATP-binding cassette sub-family G member 2 (Placenta-specific

APbinding

cassette transporter) (Breast cancer resistance protein) (Mitoxatrone

resistance-associated protein) (CD338 antigen) (CDw338).

[Source:Unipot/SWISSPROT;Acc:Q9UNQ0]

ATAACACTGAACATTTTGTAGTTTTCTGAGTAGACCTTTTTATTTCACACCTTTTTCATCTTATTCTTCCACCTTCAGTA

CTTTTTCTTTTTCTTCTTAG

In100 candidate1275 (in intron):

LOCUS ENSG00000104324

DESCRIPTION plasma glutamate carboxypeptidase

[Source:RefSeq_peptide;Acc:NP057218]

GTAGATTTGTCACCAATTGCAAAGCCTTTTCTGACCCTCATAGAAATAACTATCAACTCTCTCCTCTATATTTTCCAGAT

TTCTTGGTATGTTCATCTCT

In100 candidate1276 (in intron):

LOCUS ENSG00000104324

DESCRIPTION plasma glutamate carboxypeptidase

[Source:RefSeq_peptide;Acc:NP057218]

ACCTGGTCATTCATTTGTTTAATCGCTTGACTGTACTAACTCTGTGATGTCTATTTCTCCTACACTGTTCAGCTTCTGAT

GTTCCTGTTTAGATTGTTTT

In100 candidate1277 (in intron):

LOCUS ENSG00000104324

DESCRIPTION plasma glutamate carboxypeptidase

[Source:RefSeq_peptide;Acc:NP057218]

TTCTAACATTTTCTATTAAAGTTGCTATTTTGCTCCTTTTTTTCTCCTTGTTTGCTTTCTATTGGATTCAGTGGTTTTTC

TTACCCTCTCTCCTCATTTT

In100 candidate1278 (in intron):

LOCUS ENSG00000160868

DESCRIPTION Cytochrome P450 3A4 (EC 1.14.13.67) (Quinine 3-monooxygenase)

(YPIIIA4) (Nifedipine oxidase) (Taurochenodeoxycholate 6-alpha- hydroxylase)(EC

1.14.13.97) (NF-25) (P450-PCN1). [Source:Uniprot/SWISSPROT;Acc:P08684]

EXON

AACTCTTAACCTCGTGTAGAGTGATTCTTCCCACTTTTATTGACTTATTTTTTCAGATTTGTTTTAACTCTTCCTGGTCC

TCTGCTTTTCCATAAAAATT

In100 candidate1279 (in intron):

LOCUS ENSG00000102893

DESCRIPTION Phosphorylase b kinase regulatory subunit beta (Phosphorylase

knase subunit beta). [Source:Uniprot/SWISSPROT;Acc:Q93100]

CACATGATTATTAGTCCATTAAAAAAGAATTATTCTTTATGTTTAACTTACTGGTTTTATAGCTTTCTTCTCTTTCAGTT

CATATTTCACAAATTTTCTT

In100 candidate1280 (in intron):

LOCUS ENSG00000147649

DESCRIPTION Protein LYRIC (Lysine-rich CEACAM1 co-isolated protein) (3D3/lyic) (Metastasis adhesion protein) (Metadherin) (Astrocyte elevated gene-1 potein) (AEG-1). [Source:Uniprot/SWISSPROT;Acc:Q86UE4]

CACTGCCCTACTTTAGGACTTTATTACCTTTTGTTTAGACTGTTCTAACATCATCCTAACTAGTTTTCCTGCTTCCAGTT TCTTTTTTGCCAATCTTTTC

In100 candidate1281 (in intron):

LOCUS ENSG00000134318

DESCRIPTION Rho-associated protein kinase 2 (EC 2.7.11.1) (Rho-associated, oiled- coil-containing protein kinase 2) (p164 ROCK-2) (Rho kinase 2). [Souce:Uniprot/SWISSPROT;Acc:O75116]

TGATAACATATCTACAACTACATTCTTTTTTCCCCTATTGCTCCAAATCTGCTTCATTTCCTTTGGTTTTAGATTCAGTG ATTGTTTCCACTATCCATTT

In100 candidate1282 (in intron):

LOCUS ENSG00000134318

DESCRIPTION Rho-associated protein kinase 2 (EC 2.7.11.1) (Rho-associated, oiled- coil-containing protein kinase 2) (p164 ROCK-2) (Rho kinase 2). [Souce:Uniprot/SWISSPROT;Acc:O75116]

GTCTTCCATTCTTTTTCTGTCTTTTTTGGTTTTAACTGATCATTTAATGTGATTTCATTTTCTCTCCTTTCTTAGCAGAT TGTTTATACTTTTTTTTTTG

In100 candidate1283 (in intron):

LOCUS ENSG00000134318

DESCRIPTION Rho-associated protein kinase 2 (EC 2.7.11.1) (Rho-associated,

oiled- coil-containing protein kinase 2) (p164 ROCK-2) (Rho kinase 2).

[Souce:Uniprot/SWISSPROT;Acc:O75116]

AGTTATGTTAATCTTGTAACTAATGATGACAAACCTCTCTTCATATATTTGTCAGTCATTTCTGTTTTGTGAATTACCTG

TTTATATCTTTTAACTCTTT

In100 candidate1284 (in intron):

LOCUS ENSG00000170310

DESCRIPTION Syntaxin-8. [Source:Uniprot/SWISSPROT;Acc:Q9UNK0]

AACAATATTAATTCTTCCAATCCATGAGCATGAAATAACTTTGCCTTTGTCTGTATCCTCTTCAATTTCTTTCATCAGTG

TTTTGTAGTTTTTCTTTTAG

In100 candidate1285 (in intron):

LOCUS ENSG00000170310

DESCRIPTION Syntaxin-8. [Source:Uniprot/SWISSPROT;Acc:Q9UNK0]

TAGTTTGAGGAGAATTGATATTTTAACACTATTGAGTCTCAAATTCTCTCAGCAATGCCTTTTAGTTTTTGCTATACGTG

TCTTATATATCTTTTATCCT

In100 candidate1286 (in intron):

LOCUS ENSG00000170310

DESCRIPTION Syntaxin-8. [Source:Uniprot/SWISSPROT;Acc:Q9UNK0]

AGTGTTGTCTAGAGCATTCTGGAGTAACGCAATCTCCCTTCCTTTCTCTCTTCTCTTCATTTTTTCTCCAGTGCTGTGTG

CGTATGTCTTTTTCTCTTTC

In100 candidate1287 (in intron):

LOCUS ENSG00000136152

DESCRIPTION Conserved oligomeric Golgi complex component 3 (Vesicle dockingprotein SEC34 homolog) (p94). [Source:Uniprot/SWISSPROT;Acc:Q96JB2]

AGATACTATTTAATAACCTTTTCTTTATTTTTCTCAAAATGTCTTTCAGATCCACTGTTAGTCACTTTTGGTTTCTTAAT

GTTCACTCTTTTGACTCTTA

In100 candidate1288 (in exon):

LOCUS ENSG00000166596

DESCRIPTION WD repeat protein 16 (WD40-repeat protein up-regulated in HCC).[Source:Uniprot/SWISSPROT;Acc:Q8N1V2]

AGTCCTGTTGAAGACTGAGTTTAGATAACTCCAACACTAGTCTTCATTTCTCACAGCTCTGTTTTTGTTCTTGAGTCAAT

TTTTCTCTTTTTCTTTATAG

In100 candidate1289 (in intron):

LOCUS ENSG00000163818

DESCRIPTION leucine zipper transcription factor-like 1 [Source:RefSeq_peptie;Acc:NP_065080]

CCCGTAATCTTCCTACCCTAAAATAACCACTTTTAACTTTCTGTTGTTTTCCCTCCCAGCCGTGTTTCTATGCATTCGTA

CACTTTGAACTTTTATTTTC

In100 candidate1290 (in intron):

LOCUS ENSG00000171295

DESCRIPTION Zinc finger protein 440. [Source:Uniprot/SWISSPROT;Acc:Q8IYI8]

EXON

AGGCGTGAGCCACTGCACCTGGCTAACAACCTAATTCATTATGGTTTTTTTTCCCCCAGAGCTTGTTTTCTTTAAAGCTC

GTTTTTATTTCCTTTTCAAT

In100 candidate1291 (in intron):

LOCUS ENSG00000138640

DESCRIPTION Protein FAM13A1. [Source:Uniprot/SWISSPROT;Acc:O94988]

AGTGCTATTGAAGGCAAATAACTATAGTCATGTTTATTTGTGTTTCCAGTTTGCTTTGTTTTGATTAATTTTTTGTTTTG

TGTTTTGTTTTTGAATTTTA

In100 candidate1292 (in intron):

LOCUS ENSG00000196757

DESCRIPTION Zinc finger protein 700. [Source:Uniprot/SWISSPROT;Acc:Q9H0M5]

EXON

AGCCACTGCACCTGGCTAACAACTTAATTCGTAATGGTTTACTTTTTTCCCCCAGAGCTTGTTTTCTTTTCTTTCTAGCT

GGTTTTTATTTCCTTTTCAA

In100 candidate1293 (in intron):

LOCUS ENSG00000104361

DESCRIPTION NIPA-like protein 2. [Source:Uniprot/SWISSPROT;Acc:Q9H841]

TAGCTATTATAAATAACCTTACTTCAAAATCAATTTTGTCTTCTTTTCCTGTGTTACAGGTATCATATTTTATCAGGAAT

TCCTTGGTGCTCCTTTTCTC

In100 candidate1294 (in intron):

LOCUS ENSG00000123200

DESCRIPTION Zinc finger CCCH domain-containing protein 13.

[Source:Uniprot/WISSPROT;Acc:Q5T200]

TCATTGTATCTTCTTTCATTCTTAACTTTCAAATTTTTCATCTCTTTATCTGTTTCGCCTCTAGGTCATATTCTCAGGCT

TACCTTCTAGTTTACCAATT

In100 candidate1295 (in intron):

LOCUS ENSG00000197054

DESCRIPTION zinc finger protein 440 like

[Source:RefSeq_peptide;Acc:NP_00102771]

AAAGTGCTGGGATTATAGACATGAGCAAATGCATGTGGATAACAACTTAATTTGTAGTGGTTTGCTTTTTTCCCCCAGAG

CTTGTTTTCTTTTCTTTCTA

In100 candidate1296 (in intron):

LOCUS ENSG00000007237

DESCRIPTION Growth-arrest-specific protein 7 (GAS-7).

[Source:Uniprot/SWISSROT;Acc:O60861]

CTCGAAGTAACCCTCCTCCCCCATCCTCTTCTTTACCCTTACTCCCTTTTCTTTCTGTTTGACAGCTCTATTGAGATATA

TCCCCATTCTATTCTATTCT

In100 candidate1297 (in intron):

LOCUS ENSG00000007237

DESCRIPTION Growth-arrest-specific protein 7 (GAS-7).

[Source:Uniprot/SWISSROT;Acc:O60861]

GAAATACCACACCTTGGATTTAACTTGCATTTGCATTTCCTTCATTGCTAGTCAGGTCGTGTCTGTTTTCAAATGTTTAT

TTACCATTTGAATTTCCTCT

In100 candidate1298 (in intron):

LOCUS ENSG00000007237

DESCRIPTION Growth-arrest-specific protein 7 (GAS-7).

[Source:Uniprot/SWISSROT;Acc:O60861]

TTAGCACCAAGACTTTCTTTTGCTTCCCATTCCCTAGCATAACCCTCTTGCTCCTCTGTTTCTCTTGATCTTTCTCAGAT

TTCTGTTTTTATTCGTGTCC

In100 candidate1299 (in intron):

LOCUS ENSG00000113231

DESCRIPTION High-affinity cAMP-specific and IBMX-insensitive 3',5'-cyclic

posphodiesterase 8B (EC 3.1.4.17) (HSPDE8B).

[Source:Uniprot/SWISSPROT;Acc:O5263]

GCTGTGCTCTGTGGGAGTAACTGAGGTTCTGGGAGAATCTGCTTGTTTCCCCTTTTCTTCTCTTTTTCCTTCTTCCAGCT

CTTATGTGTACTTCTCTGCC

In100 candidate1300 (in intron):

LOCUS ENSG00000102910

DESCRIPTION peroxisomal LON protease-like

[Source:RefSeq_peptide;Acc:NP_11378]

CAAACTCACCTGCCAAGCACCCGCCTCCTATTCCTAACTCTACTGGAGGTGTTGTGTTTTCAGTTTAGAGCTTCTCCTTT

CCTGGCAGTTATCCCTTATT

In100 candidate1301 (in intron):

LOCUS ENSG00000102910

DESCRIPTION peroxisomal LON protease-like

[Source:RefSeq_peptide;Acc:NP_11378]

AACCTCTGTGCTTTGTGTAACTTTTTGCTAAATTCCTGTCTTTGTCTTCTTGGAACAGTCTTCTACTTGTTACAGGATCT

TCCTATCTTTTGGATTTTAT

In100 candidate1302 (in intron):

LOCUS ENSG00000183801

DESCRIPTION Olfactomedin-like protein 1 precursor.

[Source:Uniprot/SWISSPRO;Acc:Q6UWY5]

TAGTAGCCTGAAGTGGCACTAAGGAGCTAGAAAACTGTAACTCCTTCGTTTTACTTCATTTTTCTCCTTTCTTCCAGTTT

AGATCCTTTATCATCCCATT

In100 candidate1303 (in intron):

LOCUS ENSG00000166387

DESCRIPTION Liprin-beta-2 (Protein tyrosine phosphatase receptor type f

polpeptide-interacting protein-binding protein 2) (PTPRF-interacting

proteinbnding

protein 2). [Source:Uniprot/SWISSPROT;Acc:Q8ND30]

TTGGACCTTGTCCTCCTTTTTTAATTCCATGCTTCCTAACTCCTCTTTTATATTTTTCACTTGTGTCTCTGTTTGCAGCA

TTCTTCAAATCTATCTTCCT

In100 candidate1304 (in intron):

LOCUS ENSG00000166387

DESCRIPTION Liprin-beta-2 (Protein tyrosine phosphatase receptor type f

polpeptide-interacting protein-binding protein 2) (PTPRF-interacting

proteinbnding

protein 2). [Source:Uniprot/SWISSPROT;Acc:Q8ND30]

TCATCTGCTATTTAACCTGTTCACTTAATTAATTTGCTTGGCTGCATTCTTTATTTTGGTTTGTATTTTTTTTTTCAGAT

TTTCCTATTTTTTAAAATCA

In100 candidate1305 (in intron):

LOCUS ENSG00000132549

DESCRIPTION Vacuolar protein sorting-associated protein 13B (Cohen

syndromeprotein 1). [Source:Uniprot/SWISSPROT;Acc:Q7Z7G8]

AGTTTAACTTCCTCCTTTCCATTTGGATTTTCTTTCCTTTTTTTTTTTTTTTTTTTTGGTGTGGAAACCCAGGACTTTCT

TTTCTTTCTTTTATTTTTTG

In100 candidate1306 (in intron):

LOCUS ENSG00000132549

DESCRIPTION Vacuolar protein sorting-associated protein 13B (Cohen

syndromeprotein 1). [Source:Uniprot/SWISSPROT;Acc:Q7Z7G8]

AGCTTGATATTTCACTGCTCTCTATTTAACCTAAATTGGGCTATCCATTATCATTTCAGTAAGGCATGTTACCTTGCCTT

GTTCAACTTTTCCTTTTTCA

In100 candidate1307 (in intron):

LOCUS ENSG00000132549

DESCRIPTION Vacuolar protein sorting-associated protein 13B (Cohen syndromeprotein 1). [Source:Uniprot/SWISSPROT;Acc:Q7Z7G8]

AATCCTTTGTGCTCTGCCTATTCATCCCTTCCTCTCCCTAACCACTGGCAACCACTGATCTTTTTACTGTCCTCACAGTT

TTGCCTTTTCTAGACTGTCA

In100 candidate1308 (in intron):

LOCUS ENSG00000132549

DESCRIPTION Vacuolar protein sorting-associated protein 13B (Cohen syndromeprotein 1). [Source:Uniprot/SWISSPROT;Acc:Q7Z7G8]

CTGTTGAAGTAAATAATTGTTGGGTACCTATTTACTCTAACCAAATATTAACCTTTTTATTCTTTTCAGCTTTTAAGTAC

TGCCCATTCCACTTCTATAT

In100 candidate1309 (in intron):

LOCUS ENSG00000132549

DESCRIPTION Vacuolar protein sorting-associated protein 13B (Cohen syndromeprotein 1). [Source:Uniprot/SWISSPROT;Acc:Q7Z7G8]

ATAAATAGACGTAGCACTTGTGATCTCCATTACAAATATATTAACCTTTGTTTTGTTCTTTTGTTTTGGCAGGAAAGTTT

TTGTTGGTGTTTTTTTTTTT

In100 candidate1310 (in intron):

LOCUS ENSG00000132549

DESCRIPTION Vacuolar protein sorting-associated protein 13B (Cohen syndromeprotein 1). [Source:Uniprot/SWISSPROT;Acc:Q7Z7G8]

GTCTACCCTATTATAACATTTTAAAAATATTAACATGTTTCTTCTCTTACACTTTCACTTCTTAGCCTTCAGCAGTTTTA

TCCTTTTGGAGTTTACTTTT

In100 candidate1311 (in intron):

LOCUS ENSG00000102921

DESCRIPTION NEDD4-binding protein 1 (N4BP1) (Fragment).

[Source:Uniprot/SWISPROT;Acc:O75113]

CATTTCCATAAAACATTCAAGTAAAGGAGATACAACTTTTTAACTTAAAGTGTGTTTATTACCATTCCAGTTTATATAAT

CCTGTCAACCCTTACCTTTT

In100 candidate1312 (in intron):

LOCUS ENSG00000140575

DESCRIPTION Ras GTPase-activating-like protein IQGAP1 (p195).

[Source:Uniprt/SWISSPROT;Acc:P46940]

AACACTGCTAATATAATACCTGGTAACGTTTTGAGTACTTACTCTGTGCCAGGCACTGTTCTTAGTATTTTATATGTACT

ATCATCTCTTTAATCCTCAT

In100 candidate1313 (in intron):

LOCUS ENSG00000197857

DESCRIPTION Zinc finger protein 44 (Zinc finger protein KOX7) (Gonadotropin

inducible transcription repressor 2) (GIOT-2).

[Source:Uniprot/SWISSPROT;Ac:P15621]

AATTTCTAACCCATATCATTTTTTTCTTTCTGATGAACTTCTTTTTTTTTTTAACTCAGACCAGGTAATTTCAATTGTTT

TACCTTCATTTTAGTTTTTT

In100 candidate1314 (in intron):

LOCUS ENSG00000184305

DESCRIPTION

GAATGATGATTTCCAATTTCATCCGTGTCCCTGGATTACCTGATGTAACATTTTTCCTTCTTTTATTTTTCACAGAAATT

ATTTTATTAACATCTTTTTC

In100 candidate1315 (in intron):

LOCUS ENSG00000184305

DESCRIPTION

AGTGGTGATATCCCCTTTAACATTTTTAATACATCTATTTGATTCTTCTCTCTTTTCTTCTTTATTAGTCTTAGCAGTCT

GTCGATTTTGTTGATCTTTT

In100 candidate1316 (in intron):

LOCUS ENSG00000184305

DESCRIPTION

AGTTGAATAACTCAATTTTTTATTGCTATTTCTTTAATATCCTTGTCAGATATATTTTCCTCATGTGTTACATCCCACTA

TGGACACTTTAGTTTTCTTT

In100 candidate1317 (in intron):

LOCUS ENSG00000184305

DESCRIPTION

AAATTAACTTACAATTGGTTTAGATGGCCTTTTTTTCTCTCTGCTGGTCTTTCCTTTTCTCTCTGCTGCCTCTGCCAGCT

GCTTATACTGCTGTTCTTTT

In100 candidate1318 (in intron):

LOCUS ENSG00000184305

DESCRIPTION

TATGAAATTCATTATTTGTAATTTTAACATTTCTGCCTTATCTGACTCTGGTTCTGATGCTCATTCATTCTCTTCCAGCT

GTGTTTATTTCCTTTTAGTG

In100 candidate1319 (in intron):

LOCUS ENSG00000152208

DESCRIPTION Glutamate receptor delta-2 subunit precursor (GluR delta-2).

[Surce:Uniprot/SWISSPROT;Acc:O43424]

TGAAGAGCTGCCCACTCTGGAGATTTGTTTCACTGTGATTAACATTGTACCTCTCATTTACTTACTTGTCAGAGGTTTTA

TTCAGTTAGTATCTTTTTCT

In100 candidate1320 (in intron):

LOCUS ENSG00000152208

DESCRIPTION Glutamate receptor delta-2 subunit precursor (GluR delta-2).

[Surce:Uniprot/SWISSPROT;Acc:O43424]

CACTATGATTTTTATCACCATGGACTGCTCCTTAACTTGACATGTATTCTTTTGCATCTGACTTCTTTCTTCCCTCAGGC

TTTTGAGACTTTCCCATGTT

In100 candidate1321 (in intron):

LOCUS ENSG00000152208

DESCRIPTION Glutamate receptor delta-2 subunit precursor (GluR delta-2).

[Surce:Uniprot/SWISSPROT;Acc:O43424]

AATCCCTAACACACCATATTTTCTATCTTCCTTCCTGCTCTATATTTGTCCATAGTACTTCTGTATAACATGTTTCAGTT

TTTAATTAATTATCTCATTT

In100 candidate1322 (in intron):

LOCUS ENSG00000152208

DESCRIPTION Glutamate receptor delta-2 subunit precursor (GluR delta-2).

[Surce:Uniprot/SWISSPROT;Acc:O43424]

GGAAATTAAAATGAATATATGCCTGAATGGATGAATAAATAAAGTTAACCATCTGCTCCTTCTTGTTCGTTCCTTCAGAA

CTCTTTATTCCATATTGGTT

In100 candidate1323 (in intron):

LOCUS ENSG00000152208

DESCRIPTION Glutamate receptor delta-2 subunit precursor (GluR delta-2).

[Surce:Uniprot/SWISSPROT;Acc:O43424]

ATCCCAAAAGTATGCTTCTATATTATAACATAGTAGCCCATGTGTAACTTTTTTTTTCCTCTTAATCTTTACAGTTTGTC

AAATCTCTGCTCCATGCTTT

In100 candidate1324 (in intron):

LOCUS ENSG00000152208

DESCRIPTION Glutamate receptor delta-2 subunit precursor (GluR delta-2).

[Surce:Uniprot/SWISSPROT;Acc:O43424]

TCCCATTTTAACCACGCTGGTCTCTTTGCTTATTTTTAGTCTCAGCATATTCTAACTTAGTTTATTTATGATTGCTTTTC

TATTTGCTTGGATTTCTCCT

In100 candidate1325 (in intron):

LOCUS ENSG00000152208

DESCRIPTION Glutamate receptor delta-2 subunit precursor (GluR delta-2).

[Surce:Uniprot/SWISSPROT;Acc:O43424]

AGGCCTCCTAAATGCTATTTATTTTCTAGCTGTTGGTGTTAGATAACCTAATTATACAATAGTCTCTTCCAGATATTTTC

TTTTTCTTATAGCTTATAGC

In100 candidate1326 (in intron):

LOCUS ENSG00000152208

DESCRIPTION Glutamate receptor delta-2 subunit precursor (GluR delta-2).

[Surce:Uniprot/SWISSPROT;Acc:O43424]

TGTGTAACTTATTCATTAGTCCTGATATATCATTTGCATTCTTTACTACTCCCAACTGTTATTTTTATTCTGCAGTAGAA

GTTTGTCTTTATTCTTTTTT

In100 candidate1327 (in intron):

LOCUS ENSG00000152208

DESCRIPTION Glutamate receptor delta-2 subunit precursor (GluR delta-2).

[Surce:Uniprot/SWISSPROT;Acc:O43424]

AGGTCCCTACCTCCGTCTTTACTGACATCTGCTAACAAATCCTAGCTTTGCCCATTTTCCTTCAGCCGCACTGGTCTTTG

TTTTTTCTTCCATGTGTATT

In100 candidate1328 (in intron):

LOCUS ENSG00000152208

DESCRIPTION Glutamate receptor delta-2 subunit precursor (GluR delta-2). [Surce:Uniprot/SWISSPROT;Acc:O43424]

TGATTGCAAAAACAAACAAGCAAAGAAGAAACTAATGAAAACTACACTTTAACTTCATCCCTTCTTTTAAGCTTTCAGTA
GTTTCCATTTATATCTTACT

In100 candidate1329 (in intron):

LOCUS ENSG00000163104

DESCRIPTION SWI/SNF-related matrix-associated actin-dependent regulator of hromatin subfamily A containing DEAD/H box 1 (EC 3.6.1.-) (ATP- dependent hlicase 1) (hHEL1). [Source:Uniprot/SWISSPROT;Acc:Q9H4L7]

TGAACATTTCATTTAGGTGGAATTATATAATATGTAACCTTTTATGTCTAGCCTCTTATTTTTTAATTTCAGAAAAATGT
GTTTTATTTGTTTTTTCTTT

In100 candidate1330 (in intron):

LOCUS ENSG00000163104

DESCRIPTION SWI/SNF-related matrix-associated actin-dependent regulator of hromatin subfamily A containing DEAD/H box 1 (EC 3.6.1.-) (ATP- dependent hlicase 1) (hHEL1). [Source:Uniprot/SWISSPROT;Acc:Q9H4L7]

GGGAATTTTTTTTTTCCCCTAACAAAACAATAACTTCAAAATAGCCTGTTGCCTCATTTTCTTTCATTATCTCAGAAAAT
CTTTAAATCACTTTTTTCCT

In100 candidate1331 (in exon):

LOCUS ENSG00000163104

DESCRIPTION SWI/SNF-related matrix-associated actin-dependent regulator of hromatin subfamily A containing DEAD/H box 1 (EC 3.6.1.-) (ATP- dependent

hlicase 1) (hHEL1). [Source:Uniprot/SWISSPROT;Acc:Q9H4L7]

AAGTGAAATTTAGAAGTGAAGTTGTCTATTTGATATTTAACTCTTTATTAAATCTTTCTTTAAATTTCTGCCTGTCAGTC

TATATTGCTGTTTTTATTAT

In100 candidate1332 (in intron):

LOCUS ENSG00000197299

DESCRIPTION Bloom syndrome protein (EC 3.6.1.-) (RecQ protein-like 3) (DNA

elicase, RecQ-like type 2). [Source:Uniprot/SWISSPROT;Acc:P54132]

TACAATACTATAAATCATACACTAGCTGAATAAATAACAAAGGGTTTTGTGTCCTCGTGGCTTTTACTATTCTGTCAGTA

CTTGCTCCGCTGCTTTTATT

In100 candidate1333 (in intron):

LOCUS ENSG00000132842

DESCRIPTION AP-3 complex subunit beta-1 (Adapter-related protein complex 3

eta-1 subunit) (Beta3A-adaptin) (Adaptor protein complex AP-3 beta-1 subuni)

(Clathrin assembly protein complex 3 beta-1 large chain).

[Source:UniprotSWISSPROT;Acc:O00203]

GTAGATGTATTTGTTTTAATGGAGTTTCTTTAATTGTAACTTAAATGTGCTGAGTTTTCTTTATTTCAGCCTACTAGTTA

CTGTTATCCTGGCTTCATTT

In100 candidate1334 (in intron):

LOCUS ENSG00000132842

DESCRIPTION AP-3 complex subunit beta-1 (Adapter-related protein complex 3 eta-1 subunit) (Beta3A-adaptin) (Adaptor protein complex AP-3 beta-1 subuni) (Clathrin assembly protein complex 3 beta-1 large chain). [Source:UniprotSWISSPROT;Acc:O00203]

TTGTAGCTCTTGACTGGCTTAGTAACAAATGAGATGCCATTTCCTTTGTGTTCCTATTCTCCCTATCTGTATATCCAGCT
ATTATTTTTCTCTTTTGCTA

In100 candidate1335 (in intron):

LOCUS ENSG00000132842

DESCRIPTION AP-3 complex subunit beta-1 (Adapter-related protein complex 3 eta-1 subunit) (Beta3A-adaptin) (Adaptor protein complex AP-3 beta-1 subuni) (Clathrin assembly protein complex 3 beta-1 large chain). [Source:UniprotSWISSPROT;Acc:O00203]

CTCCCACTCTGTGGGTTGTCTGTTAACTCTGCTGATTATTTCTTATGCTGTGCAGAAGCTTTCTAGTTTAATTAAGTCCC
ATCTATTTATCTTTGTTTTT

In100 candidate1336 (in exon):

LOCUS ENSG00000132842

DESCRIPTION AP-3 complex subunit beta-1 (Adapter-related protein complex 3 eta-1 subunit) (Beta3A-adaptin) (Adaptor protein complex AP-3 beta-1 subuni) (Clathrin assembly protein complex 3 beta-1 large chain). [Source:UniprotSWISSPROT;Acc:O00203]

TTTCCCCCTTTTTGCTTTGCTAACCAAAGAGCATATATTTTACTGTCAGTTGTCTCAACTCTTGAATCCATGTGGCGTTT
TCTCTGTCCTGCTGCTTCTT

In100 candidate1337 (in intron):

LOCUS ENSG00000163110

DESCRIPTION PDZ and LIM domain protein 5 (Enigma homolog) (Enigma-like PDZ nd LIM domains protein). [Source:Uniprot/SWISSPROT;Acc:Q96HC4]

AGTGTAACATGACTGACCATTCTATTTTTTTATCCACAGACTTTTTTTTTTTAATGGCTAATTTTGTATTTTTCCTGTTT TATTTTTCTTCCCAAAGTTT

In100 candidate1338 (in intron):

LOCUS ENSG00000132554

DESCRIPTION Regulator of G-protein signaling 22 (RGS22). [Source:Uniprot/SWSSPROT;Acc:Q8NE09]

AGCCTAATTCCTACCAAGATCTGAGTCTGATTCCAAGAGCTCTAACCACTTCTATTATTTATTTATGTCTTCCAGCACTT ACTTCTCTTGTTGGCTAATT

In100 candidate1339 (in intron):

LOCUS ENSG00000132554

DESCRIPTION Regulator of G-protein signaling 22 (RGS22). [Source:Uniprot/SWSSPROT;Acc:Q8NE09]

AAGCTAACTCTTCATTTGGCTTCTGTAGCATTACTCTGACTTGGTTTACCTCTTATCTCTCTGACTTCTATTTCTCAGGT TCTTTTACTATTTCTTCTGA

In100 candidate1340 (in intron):

LOCUS ENSG00000151779

DESCRIPTION neuroblastoma-amplified protein

[Source:RefSeq_peptide;Acc:NP_06993]

ACCGAAGCAAACGAAACAATAAAAAATCTCAAACAAATCTAACATAGAGTTTCACATCCTCTTTTTCTTCTTTTGCAGCT

GATAGCCTTTCTCTCTATTT

In100 candidate1341 (in intron):

LOCUS ENSG00000151779

DESCRIPTION neuroblastoma-amplified protein

[Source:RefSeq_peptide;Acc:NP_06993]

CTGTTTGGCACATCTTTATAAAAAGTTTCCATTAACAACAATAATAATACTCATTTTATGCTTTAATACTTATCTCAGTT

CCTTCTTTGTCCTTTATAGG

In100 candidate1342 (in intron):

LOCUS ENSG00000151779

DESCRIPTION neuroblastoma-amplified protein

[Source:RefSeq_peptide;Acc:NP_06993]

ATTTTATTAAGGCACCTTATATAGCAAAAGCTGGAAATAACCTTCAACCCCATGCATTTATCCCTTTTATAGTTCAGACC

TTCATTTTAACATCCTTAAT

In100 candidate1343 (in intron):

LOCUS ENSG00000163827

DESCRIPTION Leucine-rich repeat-containing protein 2.

[Source:Uniprot/SWISSROT;Acc:Q9BYS8]

GAAACGCCATCTCAAAAAAAAAAAAAAAAATCTAACTATAATTGTTGAATTGTCTCTTTGTCCTTTTAATTCCATCAGTT

TTTACTTCATATATTTTACG

In100 candidate1344 (in intron):

LOCUS ENSG00000166405

DESCRIPTION resistance to inhibitors of cholinesterase 3 homolog

[Source:ReSeq_peptide;Acc:NP_078833]

AGGTCATAACTATCTCATGTGATTTCTCTGATTTTGCCTCCTTTCGGTTTTCTTTCTGTTTTCCAAACAGATTATCTCTT

TTGTTTTAATCCTTCAACAG

In100 candidate1345 (in intron):

LOCUS ENSG00000085365

DESCRIPTION Secretory carrier-associated membrane protein 1 (Secretory carrer

membrane protein 1). [Source:Uniprot/SWISSPROT;Acc:O15126]

CTCTGCTCCCTTTTCTGGCCACCTTCCTATAACTTAGCTTCTGTTTGTCACTCTTTCTACCATGGCTGTTGTCAGCTTCT

ACTGCTGCTGTTATAGTTCC

In100 candidate1346 (in intron):

LOCUS ENSG00000085365

DESCRIPTION Secretory carrier-associated membrane protein 1 (Secretory carrer

membrane protein 1). [Source:Uniprot/SWISSPROT;Acc:O15126]

TGCTTAATTTTTAACTCACTTTCAAGTCTGGATTTGCTGTCTTGTCACCCTTCAAATACTCATTCCTCTTTTTGTCAGCT

TATACCATTTGCTTTATTCC

In100 candidate1347 (in intron):

LOCUS ENSG00000104450

DESCRIPTION Sperm-associated antigen 1 (Infertility-related sperm protein Sag-1) (HSD-3.8). [Source:Uniprot/SWISSPROT;Acc:Q07617]

AACAATTTTAAAACATCTAAAAACAATGTTGAGGTATATGTATTCTGTAACTTAGAATGCTGCTTTTTAAACTTTCAGCC

TTCTCATTTTATTGGCTTGA

In100 candidate1348 (in intron):

LOCUS ENSG00000130413

DESCRIPTION Serine/threonine-protein kinase 33 (EC 2.7.11.1). [Source:Uniprt/SWISSPROT;Acc:Q9BYT3]

CTAGGTGAGAATCTTAATTTTTACTTCGTTTTAAATTTTTGTTTTTTAACAAAGTTATCTACAATTTTGTTTTTTCAGAA

AGTCATTATTGTTTTTTTTT

In100 candidate1349 (in intron):

LOCUS ENSG00000138696

DESCRIPTION Bone morphogenetic protein receptor type IB precursor (EC 2.7.1.30) (CDw293 antigen). [Source:Uniprot/SWISSPROT;Acc:O00238]

CAATAATGTACATGTTCTTCTGTATGTTGCTTTGTACATGTAATAGTATAACATAAGGGTTTTTTTGTTTTGGTCCAGAT

TTTTTGTAAACATTATTTTT

In100 candidate1350 (in intron):

LOCUS ENSG00000138696

DESCRIPTION Bone morphogenetic protein receptor type IB precursor (EC 2.7.1.30) (CDw293 antigen). [Source:Uniprot/SWISSPROT;Acc:O00238]

CTGATGGGGAAGATGTATCTTTTGAAATGTGTCTTGATATTAACTAAGTTTTTGTCTTCTTACCCAATTTTACAGATTAA

ATATTTCTCATAATTTCTTT

In100 candidate1351 (in intron):

LOCUS ENSG00000113273

DESCRIPTION Arylsulfatase B precursor (EC 3.1.6.12) (ASB) (Nacetylgalactosmine-4-sulfatase) (G4S). [Source:Uniprot/SWISSPROT;Acc:P15848]

TGCTTTGAGGAGACTTTTTATTCATTTTTGAAGGCTTTGTTCTGAAGTAACCTTGGAAGTGCTTTGGTCTCTGTTCAGAT
AGTACCCTTCTCTTTTCCTT

In100 candidate1352 (in intron):

LOCUS ENSG00000113273

DESCRIPTION Arylsulfatase B precursor (EC 3.1.6.12) (ASB) (Nacetylgalactosmine-4-sulfatase) (G4S). [Source:Uniprot/SWISSPROT;Acc:P15848]

TGTCCTGTTTAACTGGTTCACTTCCTCATATTATTTTCCTCTCTACTCCGTTTGCTGTTTTCCTTTGGCCCTCAGTGCTC
TGTTTCTGGGCGTCTGCTTT

In100 candidate1353 (in intron):

LOCUS ENSG00000186106

DESCRIPTION Ankyrin repeat domain-containing protein 46 (Ankyrin repeat smal protein) (ANK-S). [Source:Uniprot/SWISSPROT;Acc:Q86W74]

CCTTACTGCTTATAATTTGGATTTTAAATTTCTATTGTGTTTTTATTTAACTTATAGTCTTTTTTTTTTTAGTCCCAGTC
CTCTTTAATACTGTTTTTTC

In100 candidate1354 (in intron):

LOCUS ENSG00000188803

DESCRIPTION FLJ45455-like protein. [Source:Uniprot/SPTREMBL;Acc:Q4PL63]

CTTAACTTACACTTCCTGCCTATACAACAATCAATGTGATTATTCTACTTTTTTTTCCTGCCTCCTTTCTCTTTCCAGTT

TTTAGTTGCATTATTTCTAC

In100 candidate1355 (in intron):

LOCUS ENSG00000007174

DESCRIPTION Ciliary dynein heavy chain 9 (Axonemal beta dynein heavy chain).

[Source:Uniprot/SWISSPROT;Acc:Q9NYC9]

AGCTGGTTAGAGGTTCTAGGCTTTTGGACACCATGGTAACTTAAGTCCTGCCCTTTTCTTTGTTCACCAGATAAGGCCTC

TTTGTTTTGTTTTCTGTTTC

In100 candidate1356 (in intron):

LOCUS ENSG00000007174

DESCRIPTION Ciliary dynein heavy chain 9 (Axonemal beta dynein heavy chain).

[Source:Uniprot/SWISSPROT;Acc:Q9NYC9]

GTGGTGTTGAAATATTTATTATTAATGCATTAGCATTCTGTATTATAACTGTATTTTTATTCATTCTGTGTTCAGATCAC

TGCCAGATTTTTTTATATCT

In100 candidate1357 (in intron):

LOCUS ENSG00000174226

DESCRIPTION CDNA FLJ36587 fis, clone TRACH2013928.

[Source:Uniprot/SPTREMBLAcc:Q8N9S9]

TTTTACTTTTTCTAACACAATTTTCACTTTTGTAATGGCTTTATTTTCCTCTTCCATTTCTTCCGTGAACTTTACCAGCT

CACTTTTATATCCTTTTGTT

In100 candidate1358 (in intron):

LOCUS ENSG00000163116

DESCRIPTION

TTATTCCAAGCAAGACAAGGTCTGTAGAATACTCTAACACATATCATAGTCTTCTTGACATATGCTTTTGTTCTTCAGTA

TTTTAATCCTATCCTTTTCT

In100 candidate1359 (in intron):

LOCUS ENSG00000163116

DESCRIPTION

AGGTACACCTAACAAACGTAGATTTGTTCTTTTTACACAGTTCCATATTTCTTGGAGGCTTTGTTCATTCATTTTCATTT

TTTTCTCTCATTTTCTCTTC

In100 candidate1360 (in intron):

LOCUS ENSG00000163116

DESCRIPTION

AGTGGCTGAAACAATTTACATTCCTACTAACAATGTATGAGTGTTCCCTTTTCTCTGCAGCTTTGCCAGCATCTATTTTT

TTTTTTTTTACTTTTTAAT

In100 candidate1361 (in intron):

LOCUS ENSG00000163116

DESCRIPTION

TCCTTTTCTTTGAGACTTCCCTCACTAATGACATCATCTCTGTAATAACTTGAATAAAACATCTCATTATTTGTTCAGTG

CATTTTGTCTTTCTCATCAG

In100 candidate1362 (in intron):

LOCUS ENSG00000197093

DESCRIPTION Galactose-3-O-sulfotransferase 4 (EC 2.8.2.-) (Galbeta1-

3GalNAc3'- sulfotransferase) (Beta-galactose-3-O-sulfotransferase 4).

[Source:Unipot/SWISSPROT;Acc:Q96RP7]

GAATCCCCTCCTCCTAATTAACACCCCCTATGTGTTTCTTTCTTCACAGCCATGTTCTCCTCGCTGTTTCTATTAGCCCC

TTCTATGAGAATACTTTATT

In100 candidate1363 (in intron):

LOCUS ENSG00000177200

DESCRIPTION Chromodomain-helicase-DNA-binding protein 9 (EC 3.6.1.-)

(ATPependent

helicase CHD9) (CHD-9) (Chromatin-related mesenchymal modulator) (ReMM)

(Chromatin remodeling factor CHROM1) (Peroxisomal proliferator-activaed receptor

A-interacting complex 320 k [Source:Uniprot/SWISSPROT;Acc:Q3L8U]

AAAGAGGTTTCCTAATCCTATTTTCTTTTCTATTGCTCTTGTTATTACATAACTATTTCTTAATCCATTTTTATGCAGTC

TTACTCCTAATTTTTGTTGG

In100 candidate1364 (in intron):

LOCUS ENSG00000177200

DESCRIPTION Chromodomain-helicase-DNA-binding protein 9 (EC 3.6.1.-)

(ATPependent

helicase CHD9) (CHD-9) (Chromatin-related mesenchymal modulator) (ReMM)

(Chromatin remodeling factor CHROM1) (Peroxisomal proliferator-activaed receptor

A-interacting complex 320 k [Source:Uniprot/SWISSPROT;Acc:Q3L8U]

TAACACCGTTGATAATCTAGTCTGTTATATTTAGTCCTTGTTGCTTATCATTCTCCCACAGGCATCTCCTTTGCTTGTC

ATGCCAAACTACTTGTTATT

In100 candidate1365 (in intron):

LOCUS ENSG00000177200

DESCRIPTION Chromodomain-helicase-DNA-binding protein 9 (EC 3.6.1.-)

(ATPependent

helicase CHD9) (CHD-9) (Chromatin-related mesenchymal modulator) (ReMM)

(Chromatin remodeling factor CHROM1) (Peroxisomal proliferator-activaed receptor

A-interacting complex 320 k [Source:Uniprot/SWISSPROT;Acc:Q3L8U]

AGTGAAAGTGGTGAGAGGGAATGAAGTGTAACTATGGTTGGTTCCTTAATGTTGCTTCAGAATATCTTTGTGTTTCTTCT

GATTTCCTGCTTCCAACATA

In100 candidate1366 (in intron):

LOCUS ENSG00000177200

DESCRIPTION Chromodomain-helicase-DNA-binding protein 9 (EC 3.6.1.-)

(ATPependent

helicase CHD9) (CHD-9) (Chromatin-related mesenchymal modulator) (ReMM)

(Chromatin remodeling factor CHROM1) (Peroxisomal proliferator-activaed receptor

A-interacting complex 320 k [Source:Uniprot/SWISSPROT;Acc:Q3L8U]

AAAGCCATTAACTAAACTAAATACCTATTTTAATTTTACCAGTTTTTCAACTAATGTCCCTTTTTGTTCCAAGATCCTAC

AATGTATTTGGTTTTTCTTT

In100 candidate1367 (in intron):

LOCUS ENSG00000066923

DESCRIPTION Cohesin subunit SA-3 (Stromal antigen 3) (Stromalin 3) (SCC3 hoolog 3). [Source:Uniprot/SWISSPROT;Acc:Q9UJ98]

GTTTCTGGCTATCTGTCTCTCCCTCTCCTAACCCAAACCTCATTTTTCAAACCCTTTGTTGTTACAGGTTATCCTGCCAG
CCTTGACTCTTGTCTATTTT

In100 candidate1368 (in intron):

LOCUS ENSG00000168785

DESCRIPTION Tetraspanin-5 (Tspan-5) (Transmembrane 4 superfamily member 9) Tetraspan NET-4). [Source:Uniprot/SWISSPROT;Acc:P62079]

ATTTTAGCACATAGAAAGGCTTAAACCCTCTTGTGAACTCCTACTTAACCATTTCTTTATTATACTCTTCTTACTCAGAT
TTATAAATTTTCCTTCCTTT

In100 candidate1369 (in intron):

LOCUS ENSG00000151247

DESCRIPTION Eukaryotic translation initiation factor 4E (eIF4E) (eIF-4E) (mNA cap-binding protein) (eIF-4F 25 kDa subunit). [Source:Uniprot/SWISSPROT;cc:P06730]

TTAACATAGATCTCTGTTGTTTGCTGCTTCAGTCTGATATCCAAACACTTGTTGCATTGCCCTTTTTTTGGTATCAGTCT
TTGTCCTAGTGCCTTTCTTT

In100 candidate1370 (in intron):

LOCUS ENSG00000197872

DESCRIPTION Protein FAM49A. [Source:Uniprot/SWISSPROT;Acc:Q9H0Q0]

TATACATTTAGCACCTATTAATTCTAACTATTTGGATAGTATTTTTGTATACATTTTTTCATCCCACAGACACCTTCTAA
GGTGATATTTTCATTTCTTT

In100 candidate1371 (in intron):

LOCUS ENSG00000065559

DESCRIPTION Dual specificity mitogen-activated protein kinase kinase 4 (EC .7.12.2) (MAP kinase kinase 4) (JNK-activating kinase 1) (c-Jun N-terminal inase kinase 1) (JNKK) (SAPK/ERK kinase 1) (SEK1). [Source:Uniprot/SWISSPRO;Acc:P45985]

TTATAGATGGCATCTTCTTAGAATCCTAAGAATTTTGATAACTGGATAGGAAAGACTACTTAACTATCCTTTCTTCAGTC

CTGGCTCTTTTTTCTGCGTG

In100 candidate1372 (in intron):

LOCUS ENSG00000065559

DESCRIPTION Dual specificity mitogen-activated protein kinase kinase 4 (EC .7.12.2) (MAP kinase kinase 4) (JNK-activating kinase 1) (c-Jun N-terminal inase kinase 1) (JNKK) (SAPK/ERK kinase 1) (SEK1). [Source:Uniprot/SWISSPRO;Acc:P45985]

GTTAATTAGTTAATTAGGGACTGAACTAGATTTTCTCTCCTAACTAGATTCTGTGGTCAACTTTTTCAGTGCTGTATTCG

TTTTCATTATAGACTTTCCC

In100 candidate1373 (in intron):

LOCUS ENSG00000104490

DESCRIPTION Neurocalcin-delta. [Source:Uniprot/SWISSPROT;Acc:P61601]

GGCAAGAATGTTCCTAAAACATTTATGTTGTTTATTAACATACAAAAGCTTTTATTTTCATCCAGTCAAACCTATCAATT

TACCCTTTTTGATCTATTTC

In100 candidate1374 (in intron):

LOCUS ENSG00000104490

DESCRIPTION Neurocalcin-delta. [Source:Uniprot/SWISSPROT;Acc:P61601]

CTCATTTTATAAAAGTTCAAATAACATGATCTCATTTTCAACTTTTCATGACTTAATTTTTTTTTCATTTTTCAGGCTTA

GTTTTTTTAATGCTTGTGTT

In100 candidate1375 (in intron):

LOCUS ENSG00000104490

DESCRIPTION Neurocalcin-delta. [Source:Uniprot/SWISSPROT;Acc:P61601]

TAACTACCTTTTTATCACCCTTCTTGTTTTCAGTTCCACCTCTCACCTTTATTTTCAGAGGTATCTGGTGCCTCTAGTTC

CTGAGCTTTTTCTGGCTTTT

In100 candidate1376 (in intron):

LOCUS ENSG00000104490

DESCRIPTION Neurocalcin-delta. [Source:Uniprot/SWISSPROT;Acc:P61601]

GTTAGGTCTCTTGGATCTCTCCTCTATGGTCTTAACTTTTTTCCCATATTTTCTTTCTTCCCTTTTATGTGCATTCAGAG

GATTTTCTCAATTTTGTCTT

In100 candidate1377 (in intron):

LOCUS ENSG00000179136

DESCRIPTION CDNA FLJ34690 fis, clone MESAN2000894.

[Source:Uniprot/SPTREMBLAcc:Q8NAV9]

TTTTCTCCCATTCTGTGTGTTGTCTGTTAACTCTGCTGATTATTTATTTTGCTGTGCAGCTTTTTAGTTTAATTAAGTCC

CATCTATTTATATTTGTTTT

In100 candidate1378 (in intron):

LOCUS ENSG00000085514

DESCRIPTION Paired immunoglobulin-like type 2 receptor alpha precursor (Inhbitory receptor PILR-alpha) (Cell surface receptor FDF03). [Source:Uniprot/WISSPROT;Acc:Q9UKJ1]

TGCCTGTGGCTATCTGTTCCCTAATTTATTCCTACGTAACTTTTCATTACTTTCTTCCTCTGAGTTTCTTTACAGCTGGC

TCCTCCTGTTTGGGGTTTTT

In100 candidate1379 (in intron):

LOCUS ENSG00000175356

DESCRIPTION Signal peptide, CUB and EGF-like domain-containing protein 2 prcursor (Protein CEGP1). [Source:Uniprot/SWISSPROT;Acc:Q9NQ36]

TTTCTTGTCCCCCTGAGGAAATTAATTACGAGATTTTAACTCCTCTTCTGTTCTCTAGATTATCTTTGTTTCCTCCAGGT

TTCTTTTTCCCCGTTGGTTT

In100 candidate1380 (in intron):

LOCUS ENSG00000102468

DESCRIPTION 5-hydroxytryptamine 2A receptor (5-HT-2A) (Serotonin receptor 2) (5- HT-2). [Source:Uniprot/SWISSPROT;Acc:P28223]

AGTTGACAAAGGAAAATGCTCTAAGAAAAGATAACTTTTGTTCTTATTCCCTTTTTATTTTTTAAGCATCAGAGCTATCA

AACTGCATTCTCTTATTTTT

In100 candidate1381 (in exon):

LOCUS ENSG00000102468

DESCRIPTION 5-hydroxytryptamine 2A receptor (5-HT-2A) (Serotonin receptor 2)

(5- HT-2). [Source:Uniprot/SWISSPROT;Acc:P28223]

AGACTTCAAATGTTATTCAATAACTATTGCTGCTTTCTCTTCTACTTCTTGTGCTTTACTCTGAATTTCCAGTGTGGTCT

TGTTTAATATTTGTTCCTCT

In100 candidate1382 (in intron):

LOCUS ENSG00000140718

DESCRIPTION fatso [Source:RefSeq_peptide;Acc:NP_001073901]

ATGCTCTGTTAACCTTTTTCTCCTGTCTTTTTCCTCTCTGTATTTCATTTTGGATAATTTGCCTTGCTGTGTCTTCAGGT

TCATTATTCTTTTCTTCTAT

In100 candidate1383 (in intron):

LOCUS ENSG00000172955

DESCRIPTION Alcohol dehydrogenase 6 (EC 1.1.1.1).

[Source:Uniprot/SWISSPROTAcc:P28332]

AACTACATCTTACCTTAGCTAACTCCATCTTTGCTTGTTCAATTCTTACTTCCAGAAAGCACATTTTATTCTTATATTGC

TTTGTACACATTCTTTTCCC

In100 candidate1384 (in intron):

LOCUS ENSG00000181555

DESCRIPTION Histone-lysine N-methyltransferase SETD2 (EC 2.1.1.43) (SET

domin- containing protein 2) (hSET2) (Huntingtin-interacting protein HYPB)

(Hutingtin yeast partner B) (Huntingtin-interacting protein 1) (HIF- 1) (p231HP). [Source:Uniprot/SWISSPROT;Acc:Q9BYW2]

CCTTCATTATGCCCCCTTCCCTCCCAACTCCACAAATTTACAATATAACCTGAGCCTATTTTCATCATATTTTGTCAGGA

TTATTCTTTTCATCTGCCTG

In100 candidate1385 (in intron):

LOCUS ENSG00000184014

DESCRIPTION Rab6-interacting protein 1 (Rab6IP1).

[Source:Uniprot/SWISSPROTAcc:Q6IQ26]

TTTTGTAACCTTTGACCTTCTAACTTTCTTGTGCCTTTTTTGACCACTAGATTTTTCAGATTTGTATCCCTCTAGGTGGT

GGGTTCTTTGGTTCTTTCTC

In100 candidate1386 (in intron):

LOCUS ENSG00000006744

DESCRIPTION Zinc phosphodiesterase ELAC protein 2 (EC 3.1.26.11) (Ribonuclese Z 2) (RNase Z 2) (tRNase Z 2) (tRNA 3 endonuclease 2) (ElaC homolog protin 2) (Heredity prostate cancer protein 2). [Source:Uniprot/SWISSPROT;Acc:QBQ52]

AGAGTTTAGATGCTCTGTTTCTCAAAATAACCCCATACTTTTATTTCCTTTTAAATTTTTTTCCAGTGCCCTGTCAGCCT

CCGTACATTTTTTTTTTTTT

In100 candidate1387 (in intron):

LOCUS ENSG00000006695

DESCRIPTION Protoheme IX farnesyltransferase, mitochondrial precursor (EC

25.1.-) (Heme O synthase). [Source:Uniprot/SWISSPROT;Acc:Q12887]

AGAGGTCAAAGTAACGCTCTTTTCTAATTATCTCTTTCTTTTCTTTTTTTAATCTTCAGTTATATTTTGCCATTCCTGAT

GGTTTTTGTTCTTCTGAATC

In100 candidate1388 (in intron):

LOCUS ENSG00000166478

DESCRIPTION Zinc finger protein 143 (SPH-binding factor).

[Source:Uniprot/SISSPROT;Acc:P52747]

AGTATTTGTCTTTTTGTAACTGAAAATTAGCCTATGCTAATTTTATTCTCAGCAGGGGTCTTTTTTTTTTTTTTTTTTT

TTTACCACCCCCCCATACGG

In100 candidate1389 (in intron):

LOCUS ENSG00000039319

DESCRIPTION Zinc finger FYVE domain-containing protein 16 (Endofin)

(Endosoeassociated

FYVE domain protein). [Source:Uniprot/SWISSPROT;Acc:Q7Z3T8]

CTTTTAACCTAGTTTTTGTATTTTTCTCTTCCTCGTCCCTCTGGGCATTACTTTAGGTAATTTCTTCAGATAGGTCTTCG

ATGTCATTCAGTTCTCTCTT

In100 candidate1390 (in intron):

LOCUS ENSG00000133789

DESCRIPTION Switch-associated protein 70 (SWAP-70).

[Source:Uniprot/SWISSPRT;Acc:Q9UH65]

AGGTTTTCTGTAAAATATTTTTAACCATGTTGGCGACTCCTATTTTTGCTATTTAATTTTTTCCTGAATCTAATCCAGGC

CCTTGATTTCTTTCTCTTTT

In100 candidate1391 (in intron):

LOCUS ENSG00000113318

DESCRIPTION DNA mismatch repair protein Msh3 (Divergent upstream protein)

(UP) (Mismatch repair protein 1) (MRP1). [Source:Uniprot/SWISSPROT;Acc:P2058]

TGGGAAAGTCTTACCAATTTTATCTTTTCAAAAAACTAACTTTTGGTTTGTTGATCTTTTGTATTTTCTTTGTTTCAGTT

TCATTTATTTCTGCTCTGAT

In100 candidate1392 (in intron):

LOCUS ENSG00000113318

DESCRIPTION DNA mismatch repair protein Msh3 (Divergent upstream protein)

(UP) (Mismatch repair protein 1) (MRP1). [Source:Uniprot/SWISSPROT;Acc:P2058]

AGGTTTTCTTTTTATCCCCCATTAGTAACATGTTATAAGGCTTTACTTTGATACCCAGTTGTTTGTGTGAAGTTTCTCAC

TTCCTTTTGGCTTTTGTCTA

In100 candidate1393 (in intron):

LOCUS ENSG00000113318

DESCRIPTION DNA mismatch repair protein Msh3 (Divergent upstream protein)

(UP) (Mismatch repair protein 1) (MRP1). [Source:Uniprot/SWISSPROT;Acc:P2058]

GACATGTTATACTTGAGCACAAATCTTGCTTTTCCCTTTTAACAACTATGTGACCTTTTTCAAATTACTACTTCTCAGTG

TCTCAGTTCTTTTCCTGTAA

In100 candidate1394 (in intron):

LOCUS ENSG00000113318

DESCRIPTION DNA mismatch repair protein Msh3 (Divergent upstream protein) (UP) (Mismatch repair protein 1) (MRP1). [Source:Uniprot/SWISSPROT;Acc:P2058]

AGATGGAGGAATTTTGATATCTCTAACTATAGTAGTATGTTCATCTGTTTCTCTTTGTAGTTCTATCAGCTTTTGCCTCA

TGTAGTTTGACATTCTCTTG

In100 candidate1395 (in intron):

LOCUS ENSG00000183571

DESCRIPTION

TTGCAAAGAAAAGTATCAAAAACAAACTACAATTTAACACCAACCTTCACCCACATTTTTGACTTTTTAGTGTTTCAGTT

TTATCTTTTTATGTTGCCTA

In100 candidate1396 (in intron):

LOCUS ENSG00000133812

DESCRIPTION Myotubularin-related protein 13 (SET-binding factor 2). [SourceUniprot/SWISSPROT;Acc:Q86WG5]

CTGGGTGATGAAACAAGACCCTATCTCAAAAAAAAAAGAAAGTGTTGTTTAACCTGCAAGTATTTCTGGATTTTCCAGCT

ATCTGCCTGTTATTGATTTC

In100 candidate1397 (in intron):

LOCUS ENSG00000113319

DESCRIPTION Ras protein-specific guanine nucleotide-releasing factor 2 [Souce:RefSeq_peptide;Acc:NP_008840]

AGAATTTTTATAACAATTGCTTTTTTTATGCTCTATTATTATTTTTCTCCCTTAAGTTCTCCCACAGCATATTCCTATCA

AGACTCTTCTATCTTTTCTT

In100 candidate1398 (in intron):

LOCUS ENSG00000113319

DESCRIPTION Ras protein-specific guanine nucleotide-releasing factor 2

[Souce:RefSeq_peptide;Acc:NP_008840]

AGAAAATTTAAAATTGCCTTATAACCATGGCCATCTTTGATTTTACCTTGTTTTTTTCTCTTGCTTTTATTCAGTAGTTC

ATCAATTTTTATTATTCCTT

In100 candidate1399 (in intron):

LOCUS ENSG00000113319

DESCRIPTION Ras protein-specific guanine nucleotide-releasing factor 2

[Souce:RefSeq_peptide;Acc:NP_008840]

GCTCACCCTGTGGCTTCACCTCACACTAACCCCTGTCTCCATTCTCCTTTGGCAGAAGTCACCTTCCGCCTTTTTCCTGG

CAGTATATTCTTGTTGCATT

In100 candidate1400 (in intron):

LOCUS ENSG00000185013

DESCRIPTION Retinol dehydrogenase 14 (EC 1.1.1.-) (Alcohol dehydrogenase

PA2). [Source:Uniprot/SWISSPROT;Acc:Q9HBH5]

TTGTTCTTTCAAATAACAAACTTTTGGTTTTTTGATCTTTTGTATATTTTTTCCCATCTCAATTTCATTCAGTTCAGCTC

TGATTATGGTTATTTCTTCT

In100 candidate1401 (in intron):

LOCUS ENSG00000068305

DESCRIPTION Myocyte-specific enhancer factor 2A (Serum response

factorlikeprotein

1). [Source:Uniprot/SWISSPROT;Acc:Q02078]

CTCTTCATGTTCTTATTTTTCTTTGTCTTCTGTAACATACTAAGTTTATTTATAATCTTTTTTTAATGCCCTTGTCAGTT

CTGTTAACTTTATCATTTTT

In100 candidate1402 (in intron):

LOCUS ENSG00000164035

DESCRIPTION Endomucin precursor (Endomucin-2) (Mucin-14) (Gastric cancer

anigen Ga34). [Source:Uniprot/SWISSPROT;Acc:Q9ULC0]

TGTCACAATTAGTGAACTAATACTGATAAATTATTGTTAACTAAATTCTGATTTCTTTTAGTCTTTACTCAGTCTTTTTT

CTGTTCCAAGAAATTTGCCT

In100 candidate1403 (in intron):

LOCUS ENSG00000177112

DESCRIPTION

TAAAACACTATAACTTGTTCCTCCTAATTTTATGTCCTTTAACAAATCTGTCTTTTCCTCCTCTCTTCCCTTTCCAGCCA

CTAGTATCCTCTATTCTGTT

In100 candidate1404 (in intron):

LOCUS ENSG00000177112

DESCRIPTION

AATTAAACAAGCTCTCTATTCTTTTCTTGAATAAATCTTAAAATAATAACCCTCCCTCTTAATTATATTGCTTTCCAGGA

TTTTTATTTCACCTTGTGTT

In100 candidate1405 (in intron):

LOCUS ENSG00000133800

DESCRIPTION Lymphatic vessel endothelial hyaluronic acid receptor 1 precursr (LYVE-1) (Cell surface retention sequence-binding protein 1) (CRSBP-1) (Haluronic acid receptor) (Extracellular link domain-containing protein 1). [ource:Uniprot/SWISSPROT;Acc:Q9Y5Y7]

GATTTGTCTATTTCCTCATGGTTTCATTTAACTTGTACTCCCATCCCATGTATTTCTTTTCTTTTTTTCCAGAGATAATT

TTATTTTATTATTTTATTTT

In100 candidate1406 (in intron):

LOCUS ENSG00000138814

DESCRIPTION Serine/threonine-protein phosphatase 2B catalytic subunit alphaisoform (EC 3.1.3.16) (Calmodulin-dependent calcineurin A subunit alpha isoorm) (CAM-PRP catalytic subunit). [Source:Uniprot/SWISSPROT;Acc:Q08209]

ATTCTTCATAGCTCCATTTAATAACTCTCTCTTCCTCTCTTTTATCTTTCAGCTTTCCCTTCACACTCATTCTTTGACAG

TTTCTCCTCTTTTCTCTGTT

In100 candidate1407 (in intron):

LOCUS ENSG00000138814

DESCRIPTION Serine/threonine-protein phosphatase 2B catalytic subunit alphaisoform (EC 3.1.3.16) (Calmodulin-dependent calcineurin A subunit alpha isoorm) (CAM-PRP catalytic subunit). [Source:Uniprot/SWISSPROT;Acc:Q08209]

AAAATAGAATAACCCCCAAATGCCTCAAATTTAATGACCTAATTTCCTCTTTTTCTCCCCTCCTTGTTTAATCTTCAGAT

TTCTTTTATTACTTACTATT

In100 candidate1408 (in intron):

LOCUS ENSG00000072952

DESCRIPTION JAW1-related protein isoform b

[Source:RefSeq_peptide;Acc:NP_56056]

GTCCACTCCTCTATAGAGTCTTCTTTAACTCCCCTCACTAAAGAGTTTTCTTTTCCTTCCTCTGAGCTTTCACAGAATGT

GGTCCATCCCTTGTTTCCTT

In100 candidate1409 (in intron):

LOCUS ENSG00000172497

DESCRIPTION Acyl-coenzyme A thioesterase 12 (EC 3.1.2.1) (Acyl-CoA

thioestease 12) (Acyl-CoA thioester hydrolase 12) (Cytoplasmic acetyl-CoA

hydrolase1) (CACH-1) (hCACH-1) (START domain-containing protein 12) (StARD12).

[Soure:Uniprot/SWISSPROT;Acc:Q8WYK0]

GCATTAACTAAACTGCTGTTAATCTTATCTGGTTTGTTTTTATTTCAGATGTTGTATTTTTTTACCTTAGAAAGTCCAC

TTTGATTTTTTTATATTTTC

In100 candidate1410 (in intron):

LOCUS ENSG00000173473

DESCRIPTION SWI/SNF-related matrix-associated actin-dependent regulator of

hromatin subfamily C member 1 (SWI/SNF complex 155 kDa subunit) (BRG1-assocated

factor 155). [Source:Uniprot/SWISSPROT;Acc:Q92922]

TGATGGGATGGAGATGTTATTGAAAAGAAGTGGGTTTATTAACCCTCCTTATGCTTATGATTTTTATACTACTTTCAGAG

ATCTGGATTTTCATTTTCCT

In100 candidate1411 (in intron):

LOCUS ENSG00000118965

DESCRIPTION WD repeat protein 35. [Source:Uniprot/SWISSPROT;Acc:Q9P2L0]

TTTAAAGAATTTAGAAAGTGAGAGCTTATTAATAACTAAGCTTAATACGTTTTTTGAATGTCTTATTTTCTAACCCAGTA

CTATATTTTGTCCCTTTTGA

In100 candidate1412 (in intron):

LOCUS ENSG00000118965

DESCRIPTION WD repeat protein 35. [Source:Uniprot/SWISSPROT;Acc:Q9P2L0]

AGAGTTTAGCCTTTTTCTAGTTCTAAGATACTTGAGTAACTTTTTTTAGAGATCTATCTTTCCATTTTTTCCAGGTTTT

GTAAAATAATCCTTTTTTCT

In100 candidate1413 (in intron):

LOCUS ENSG00000176406

DESCRIPTION Regulating synaptic membrane exocytosis protein 2 (Rab3-

interacing molecule 2) (RIM 2). [Source:Uniprot/SWISSPROT;Acc:Q9UQ26]

TTAACTTAATTTAATAAGTTCGGCCATTGTTGTAGCATTTATTCTACCCTATTTTCTATTTTTCTTTTGATTTCTCAGTT

ATTTGTATTTCAGACTTTTT

In100 candidate1414 (in intron):

LOCUS ENSG00000176406

DESCRIPTION Regulating synaptic membrane exocytosis protein 2 (Rab3-interacing molecule 2) (RIM 2). [Source:Uniprot/SWISSPROT;Acc:Q9UQ26]

AAAATAAATAATACCCTGCTTTTACATTTTAGTTAAGTATTAACTCTCTAGAATATTCACTTTTTTCAGGTTAACTTTTT

CCATAAGTATGCCTGCTTTT

In100 candidate1415 (in intron):

LOCUS ENSG00000153064

DESCRIPTION B-cell scaffold protein with ankyrin repeats. [Source:Uniprot/SISSPROT;Acc:Q8NDB2]

CAAAATGTAGAGTCAAGTCTTCTTTTATTTCATGAAGAATTTCTTAACTTATATCTAAGTTTACGGTTTATTTTTCAGGG

ACTCCTTTTTTATGTATTTT

In100 candidate1416 (in intron):

LOCUS ENSG00000153064

DESCRIPTION B-cell scaffold protein with ankyrin repeats. [Source:Uniprot/SISSPROT;Acc:Q8NDB2]

CTGTAACACGACTGCTCCTCCTATATGTACCATCTTATCCTGCTGTCTTGTCTGTTTCCATGTTTTCAGCACTTTACCTC

TTTCACACGTATGATCTTAT

In100 candidate1417 (in intron):

LOCUS ENSG00000145687

DESCRIPTION Single-stranded DNA-binding protein 2 (Sequence-specific single stranded-DNA-binding protein 2). [Source:Uniprot/SWISSPROT;Acc:P81877]

GCTGTTTGCTATTGTCCTGTAACTCTTGGATGATCTGTTCTCTTTTTTAATTCCCACTTTTTTTCCTCTGTGTTTCAGTT

TGAGTACTTTTTTTCAATCT

In100 candidate1418 (in intron):

LOCUS ENSG00000145687

DESCRIPTION Single-stranded DNA-binding protein 2 (Sequence-specific single stranded-DNA-binding protein 2). [Source:Uniprot/SWISSPROT;Acc:P81877]

TGCTACCTCCTTCTTAAAAATCTAACTTGCTTGACTTTTTATATTTTTCCTGATTTTCATCCAGTCACATATCCACCATT

CTTTCTCTATCTCTTTTTTT

In100 candidate1419 (in intron):

LOCUS ENSG00000145687

DESCRIPTION Single-stranded DNA-binding protein 2 (Sequence-specific single stranded-DNA-binding protein 2). [Source:Uniprot/SWISSPROT;Acc:P81877]

AGCTATATAACATAGCATAGCATTTCCCAAATAAACCATGATTTCTTTTCTTTCTTTCTTTGTTTTTTATAGCAGTTCTT

CTCTTTGTCTTTCTGATGGG

In100 candidate1420 (in intron):

LOCUS ENSG00000140470

DESCRIPTION ADAMTS-17 precursor (EC 3.4.24.-) (A disintegrin and metalloproeinase with thrombospondin motifs 17) (ADAM-TS 17) (ADAM-TS17). [Source:Unirot/SWISSPROT;Acc:Q8TE56]

TGCCACCCTCCCCTTCCCTAACCACTGGCACCCATTTATCTGTATTCCATCTTTATGATTTTCTCATCCCAATATCAGTT

TATTTTTCAAATTTTGTTTT

In100 candidate1421 (in intron):

LOCUS ENSG00000139644

DESCRIPTION Bax inhibitor 1 (BI-1) (Testis-enhanced gene transcript)

(Transembrane BAX inhibitor motif-containing protein 6).

[Source:Uniprot/SWISSPRO;Acc:P55061]

CACCTACTCATTAAAATTTGTAACCCCAAAATCAATACTCATGGCACTTTTTTTTTTTTTTTGAGACAGAGTCTCGCTC

TGTTACTCATGGCACTTTTT

In100 candidate1422 (in intron):

LOCUS ENSG00000154227

DESCRIPTION LAG1 longevity assurance homolog 3.

[Source:Uniprot/SWISSPROT;Ac:Q8IU89]

GTCTAGCTAGCAATCTATTTTGTCTATCTTTTCAAAAACTAACTTCTAATTTTGTTGATTCTTTGTAATTATTTTCAGTC

TCTTTCATTTAGATTTGCTC

In100 candidate1423 (in intron):

LOCUS ENSG00000152348

DESCRIPTION Autophagy-related protein 10 (APG10-like).

[Source:Uniprot/SWISPROT;Acc:Q9H0Y0]

TATTCAAAAATATATGTGTATTTAACTTTACACCCATTTATCAACTCTTTCTCAATTCCATTATACTCTTGAATCCAGTT

CTTACCATTTTCCTTTTCTG

In100 candidate1424 (in intron):

LOCUS ENSG00000047849

DESCRIPTION Microtubule-associated protein 4 (MAP 4).

[Source:Uniprot/SWISSROT;Acc:P27816]

CCATTCTTGCCCATGGCCCTAACTTCTATTTTGCTTGCTGTGTGTCTTCCCCACTTTGTCTCTTTCTCTGTTTCCCAGCT

TCATTTAATTACATCCTTCT

In100 candidate1425 (in intron):

LOCUS ENSG00000147650

DESCRIPTION Low-density lipoprotein receptor-related protein 12 precursor

(uppressor of tumorigenicity protein 7). [Source:Uniprot/SWISSPROT;Acc:Q9Y56]

CGACTTGTGGCTGATTTGGACACGCTTTTAGGGGAATGCTATTTTTAACAAGCTGTGTTCTCTTTTTTCTTGTAGCAGTA

CTGACTTCCCTTTTGATTAT

In100 candidate1426 (in intron):

LOCUS ENSG00000139687

DESCRIPTION Retinoblastoma-associated protein (PP110) (P105-RB) (RB).

[Soure:Uniprot/SWISSPROT;Acc:P06400]

AGTTCTGTATTTAAATGGATTCTAAGGTTATCATAACTCCTATATCATAGTTTCTCCATTTCTCAGTTCTTTATTTTTAT

TCATCTCTTAGCTGGCTGTA

In100 candidate1427 (in intron):

LOCUS ENSG00000169946

DESCRIPTION Zinc finger protein ZFPM2 (Zinc finger protein multitype 2)

(Frend of GATA protein 2) (FOG-2) (hFOG-2). [Source:Uniprot/SWISSPROT;Acc:Q8WW8]

AGCTGTAATAACTGGTCCCTTGAGATTTTTTATCTACTTATTATTTACTTTACAGCTTGGGTATAGCTTTCAGCAAGTCT

TTGCTCCATTTTTTTTCTTC

In100 candidate1428 (in intron):

LOCUS ENSG00000169946

DESCRIPTION Zinc finger protein ZFPM2 (Zinc finger protein multitype 2)

(Frend of GATA protein 2) (FOG-2) (hFOG-2). [Source:Uniprot/SWISSPROT;Acc:Q8WW8]

AGTAACCTCAAAATGTGATTTTCTTGCTCTCTGCAGAATCTTTGTCTTACTTTTCATGTTTAATGTTGAGGCCTAGATTA

TATTTTTCCCACTTTCTTTC

In100 candidate1429 (in intron):

LOCUS ENSG00000164830

DESCRIPTION Oxidation resistance protein 1.

[Source:Uniprot/SWISSPROT;Acc:QN573]

CACATATACTATCTCTCTCTCTCTTAGGTTTTTCAATTTTTAACTTTGTGTGCTATTTTCATTTTAGCCATTGTTCAGCC

AGCACCTTTCTTGTTTTTTG

In100 candidate1430 (in intron):

LOCUS ENSG00000161800

DESCRIPTION Rac GTPase-activating protein 1 (MgcRacGAP).

[Source:Uniprot/SWSSPROT;Acc:Q9H0H5]

TTGACTCCTTACTCTTGCCTTTCATCATTAACTTCATTCCTCTTTCTCTTTTTTGTATTCATTTTTTTTTTGAGACAGAA

TTTTTCGCTCTTTGTTGCCC

In100 candidate1431 (in intron):

LOCUS ENSG00000152422

DESCRIPTION DNA-repair protein XRCC4 (X-ray repair cross-complementing protin

4). [Source:Uniprot/SWISSPROT;Acc:Q13426]

AGTGGGCAAAATATATATGAGAAAAAGTATGTGGTCGCTTATTTAACTTAAACATTTAGTTTCTTGAACTTCTCAGTCTT

TACCTTTTTTATATATATAT

In100 candidate1432 (in intron):

LOCUS ENSG00000055917

DESCRIPTION Pumilio homolog 2 (Pumilio-2).

[Source:Uniprot/SWISSPROT;Acc:Q8B72]

AGTCTCGTAACAAATTCGAATTTATTTGCCGAATCTTTCCGTATTTCCTTTTTCAGCCTTAGTGTTGTTTTGTTTTGTTT

TGTTTTGTTTTTTTTGAGAT

In100 candidate1433 (in intron):

LOCUS ENSG00000055917

DESCRIPTION Pumilio homolog 2 (Pumilio-2).

[Source:Uniprot/SWISSPROT;Acc:Q8B72]

TTGTGATTAACTATCCTTCTCATCTTTTAATATGGTCCCTTTCATGAAGCTTTCCTCAATCTTTCCCAGTGCTCATTCAT

TGATAATTACTCCCTTGTCT

In100 candidate1434 (in intron):

LOCUS ENSG00000147655

DESCRIPTION R-spondin-2 precursor (Roof plate-specific spondin-2) (hRspo2).[Source:Uniprot/SWISSPROT;Acc:Q6UXX9]

TTTGTTTAGACTTGGCCGGAGTTAATAACAAGCAAGAATTATCTTATTTTTGCTCATAAACATGTTTCCTTTCTTCAGAA

TCCCTTTCCTCATCTTTTGT

In100 candidate1435 (in intron):

LOCUS ENSG00000164049

DESCRIPTION F-box/WD repeat protein 12 (F-box and WD-40 domain protein 12) Fbox only protein 35). [Source:Uniprot/SWISSPROT;Acc:Q6X9E4]

AAAGAATGTGTGTTCTTCTCTTGTTGGGCACAATGTTTTATAAGTAACAATTAGATCACCTTGGTTGATCTTCTTCAGTT

CTTTTCTATCCTTTCTGATT

In100 candidate1436 (in intron):

LOCUS ENSG00000145681

DESCRIPTION Hyaluronan and proteoglycan link protein 1 precursor (Proteoglyan link protein) (Cartilage link protein) (LP). [Source:Uniprot/SWISSPROT;Ac:P10915]

GGTAACATCCTCTTTGTCATTTCTAATCATGTTTATTTGGATATTCTCTCTTTTCTTCTTTATTACCCTAGTTAGCAGTC

TTTCGATCTTATTAATTTTT

In100 candidate1437 (in intron):

LOCUS ENSG00000138778

DESCRIPTION centromere protein E [Source:RefSeq_peptide;Acc:NP_001804]

TATTTGATTTTCTAACTTTTGATGTTACAACTGTTTTCCTTCAGTACTTTTACTATATCATTGCACTCCCTCCTGGCCTG

TGAGATTCCTCTTTGTTGTT

In100 candidate1438 (in intron):

LOCUS ENSG00000205038

DESCRIPTION fibrocystin L [Source:RefSeq_peptide;Acc:NP_803875]

TGCTTTGAGACTTGTCAAGGTTTTAGGTTGTTTGTCTTAACACTGGCTCTGCATCTTCTCGTTATGTTTTTGCATCAGGT

TTCTGTAGTCCTTGTCTCTG

In100 candidate1439 (in intron):

LOCUS ENSG00000133030

DESCRIPTION Myosin phosphatase Rho-interacting protein (Rho-interacting

proein 3) (M-RIP) (RIP3) (p116Rip). [Source:Uniprot/SWISSPROT;Acc:Q6WCQ1]

CTGTGATGCTATCCCTGTTTTATTTCTGATAACAATTTGTGCCATTATGTCTTTTTTTTTCTTTTTTGGTCTTGTCAGCA

GTGTTTTTTTGTTTTGTTTT

In100 candidate1440 (in intron):

LOCUS ENSG00000118961

DESCRIPTION CDNA: FLJ21820 fis, clone HEP01232 (Chromosome 2 open reading

fame 43). [Source:Uniprot/SPTREMBL;Acc:Q9H6V9]

TGAGTACTTTTTCATAATTTGAGTAACTTCTGAAATTTCTGCTCTTGTCTATTGCCCATCTTGATGTTTGGTTTCCAGCC

TTTTTCTTACTGGTTTGTAG

In100 candidate1441 (in intron):

LOCUS ENSG00000147654

DESCRIPTION Receptor-binding cancer antigen expressed on SiSo cells (Cancer associated surface antigen RCAS1) (Estrogen receptor-binding fragment- assciated gene 9 protein). [Source:Uniprot/SWISSPROT;Acc:O00559]

ATTTAAATAACTCGAGTACAATTTTTAATATTCTGGTTCTTTTTTTTTTTTTTTTTTTTTTTTTTTTAACAGTCACT
GTTCTTTTCATCCTGTCAAT

In100 candidate1442 (in intron):

LOCUS ENSG00000147654

DESCRIPTION Receptor-binding cancer antigen expressed on SiSo cells (Cancer associated surface antigen RCAS1) (Estrogen receptor-binding fragment- assciated gene 9 protein). [Source:Uniprot/SWISSPROT;Acc:O00559]

ACTTTTTTTTTCTTAATAACTATTCTTTTCTCTCTGAATTACCTTTGCATCTTTATCAGATTTCAGTTTTTGTATATACG
TGGGTCTATTCCTTTACTTT

In100 candidate1443 (in intron):

LOCUS ENSG00000164053

DESCRIPTION ATR-interacting protein (ATM and Rad3-related-interacting proten). [Source:Uniprot/SWISSPROT;Acc:Q8WXE1]

TGGTTTGGCCCTTTCTCACTCTCTGGGTCCTGGCTGCACTAACATTCTTTGAATTCTCTCTGCTTCTTTACAATACAGGA
ACTTTATACATTGTTCTTTC

In100 candidate1444 (in intron):

LOCUS ENSG00000066084

DESCRIPTION DIP2 disco-interacting protein 2 homolog B [Source:RefSeq_peptie;Acc:NP_775873]

TCTGGCTTCCTAACCTTCCTCGTGCTCTCTCTGGGGTTTCTTCTACTCTTTCTTTCTCCTGATGAGGGTATTCCTCAGAT

TTGTGTCTGTTCTTCACCTC

In100 candidate1445 (in intron):

LOCUS ENSG00000066084

DESCRIPTION DIP2 disco-interacting protein 2 homolog B

[Source:RefSeq_peptie;Acc:NP_775873]

AGTTTTAACTCTTAATTTGCCACTGTTTGTTTCATTTATGTTATTTTTACCTTCTCAGTTAGACTTCAGGTTTCTTTCTT

TTTTTTTTTTTTTTTTTTGA

In100 candidate1446 (in intron):

LOCUS ENSG00000066084

DESCRIPTION DIP2 disco-interacting protein 2 homolog B

[Source:RefSeq_peptie;Acc:NP_775873]

TCTCATTTTGGCCAAAGAATATGACACAATAACTAAAACAAATTTCTAGTTGTTTCTTGTGTTGTTTATTCTGTTCAGAG

GTTTCTTTTCTTTTTCTTTT

In100 candidate1447 (in intron):

LOCUS ENSG00000066084

DESCRIPTION DIP2 disco-interacting protein 2 homolog B

[Source:RefSeq_peptie;Acc:NP_775873]

AGTTTTCTGTTGTCCTTTAACATTTTAAAATATGCCTACATCTCTTTCATTTTTAGTTTTCTTCACAGACTCCTTTTGTT

TACCTCCCTCTCTGGTGTTT

In100 candidate1448 (in intron):

LOCUS ENSG00000066084

DESCRIPTION DIP2 disco-interacting protein 2 homolog B

[Source:RefSeq_peptie;Acc:NP_775873]

AAATTACACCAAGTAATGTGTGTTAACATTATGTGTCTGTGATATCTTTCCCTTTTTTTTCTTGACAATCAGTGCATTTT

GGTGTCTTAATTTTCATCTC

In100 candidate1449 (in intron):

LOCUS ENSG00000084674

DESCRIPTION Apolipoprotein B-100 precursor (Apo B-100) [Contains:

Apolipoprtein B-48 (Apo B-48)]. [Source:Uniprot/SWISSPROT;Acc:P04114]

AGAGAGAATTTCTCTGACCACCAAGTATGAAATAACACTTCTTCTATCCCTTTCTTTTATCCTTGTATCCAGTTTTACTC

TTCTTCATAACATTCATTAC

In100 candidate1450 (in intron):

LOCUS ENSG00000138777

DESCRIPTION Inorganic pyrophosphatase 2, mitochondrial precursor (EC 3.6.1.)

(PPase 2) (Pyrophosphatase SID6-306). [Source:Uniprot/SWISSPROT;Acc:Q9H2U]

TAGCCATGCACACTGTTTATCCAAAGTTCTTAACACACTGAGCTTTTCACCCTTTCTTATATATACCAGGTAGTTTTGCA

CCTTAGTGGTTTTATCTTTT

In100 candidate1451 (in intron):

LOCUS ENSG00000138777

DESCRIPTION Inorganic pyrophosphatase 2, mitochondrial precursor (EC 3.6.1.)

(PPase 2) (Pyrophosphatase SID6-306). [Source:Uniprot/SWISSPROT;Acc:Q9H2U]

AGCCTATATGCATAACTGTAGATTTTTATGGTTTTTTTCCTCCTAATGTTTAACTGTTTTCTTACCCAGGCCTAATTTTT

TTTTTCTTTTTTGTTTTTAT

In100 candidate1452 (in intron):

LOCUS ENSG00000148925

DESCRIPTION BTB/POZ domain-containing protein 10.

[Source:Uniprot/SWISSPROTAcc:Q9BSF8]

AGTATTTTCTTTCAACCCTTCTAGTAACTTTGTAAAAATTTCTGCTATTATTTTGCATTTCTCAGAACTTTTTCTTATTC

TTTAATTTTTTTAAAATTGC

In100 candidate1453 (in intron):

LOCUS ENSG00000148925

DESCRIPTION BTB/POZ domain-containing protein 10.

[Source:Uniprot/SWISSPROTAcc:Q9BSF8]

AGGAAACTGACGTGAGTACAATACTAATAACTAAATTATTGACCTTTCTTGTATTTTATCAGTTTTTACATGCACTTTAT

CTTTTTTTTATTACATGGTT

In100 candidate1454 (in intron):

LOCUS ENSG00000102531

DESCRIPTION Fibronectin type-III domain-containing protein 3a.

[Source:Unipot/SWISSPROT;Acc:Q9Y2H6]

TGCCGTCTTTATTTTGGTACAAAAACCCTACTTAACTGAAGTGGAGTATTATCTTCCATTTTGTTTCTTTAACAACAGAA

ATTGCCTGTTTCATCTTTTA

In100 candidate1455 (in intron):

LOCUS ENSG00000102531

DESCRIPTION Fibronectin type-III domain-containing protein 3a.

[Source:Unipot/SWISSPROT;Acc:Q9Y2H6]

TCACTCTTGCTTTTTTATTTTGTCCCTGTAACTTTTGAAAATTTTGCCTTTTGTATTTTTCATTGATTTTATATGCAGAG

TTTAGTTTGATTTTTCTCTA

In100 candidate1456 (in intron):

LOCUS ENSG00000164796

DESCRIPTION CUB and sushi domain-containing protein 3 precursor (CUB and suhi

multiple domains protein 3). [Source:Uniprot/SWISSPROT;Acc:Q7Z407]

AACTATCTGGACGTTTTTACTTAAAAGTAAATATTTAACAAATTTAATTTTTGTTCCTTTGACTATAAATCTCTTCAGAT

CTCTTCTTCAATTAATGCTG

In100 candidate1457 (in intron):

LOCUS ENSG00000164796

DESCRIPTION CUB and sushi domain-containing protein 3 precursor (CUB and suhi

multiple domains protein 3). [Source:Uniprot/SWISSPROT;Acc:Q7Z407]

CTTGTCAAAAAGCACACTCAATGTAAATCCATATATTATTTAACTATGGGTTTTAATTTCTATTTACAGTACTATTTTCA

AATTCTACACTTACTATTTC

In100 candidate1458 (in intron):

LOCUS ENSG00000152268

DESCRIPTION Spondin-1 precursor (F-spondin) (Vascular smooth muscle cell

grwth- promoting factor). [Source:Uniprot/SWISSPROT;Acc:Q9HCB6]

TTAAGTCCAAGTCTGGTAACTATAATATCTAGATCATTTGTAGGTCTATTTCTATTATTTGTTTTTCCCTTGCTTCAGTT

TCTTGGTATGTCTCATCATT

In100 candidate1459 (in intron):

LOCUS ENSG00000133027

DESCRIPTION Phosphatidylethanolamine N-methyltransferase (EC 2.1.1.17) (PEAT)

(PEMT) (PEMT2). [Source:Uniprot/SWISSPROT;Acc:Q9UBM1]

CTGAGAGGACCATGGAGGGCCCTAACAAGAACTGTGGATTTGTCTTTTCCACCCTTTAGTTCTGTCAGCTTTTGCTTCAT

ACCTCACCTCACTTCTTTTT

In100 candidate1460 (in intron):

LOCUS ENSG00000119778

DESCRIPTION ATPase family AAA domain-containing protein 2B.

[Source:UniprotSWISSPROT;Acc:Q9ULI0]

CCAAAAGGGATTTGTGTGGTAGGTAAAAGAATTTATAACTGCTTAAGACTGGTTTTTCTTAACTTTTACTTGCTGCAGTG

TATTTTTTTGTCTCAATTAT

In100 candidate1461 (in intron):

LOCUS ENSG00000138780

DESCRIPTION glutathione S-transferase, C-terminal domain containing isoform1

[Source:RefSeq_peptide;Acc:NP_001026890]

ACTTTGTTTCCTACTTAACCCATTTATTCTTTGTTCTTTTTTCCTCTTTTTCTGCCTGCTTTGAGGCAGTTTGAGTATTT

TTTATGATTCTGTCTTATCT

In100 candidate1462 (in intron):

LOCUS ENSG00000081189

DESCRIPTION Myocyte-specific enhancer factor 2C.

[Source:Uniprot/SWISSPROT;cc:Q06413]

ACCAAATTATTTTTAACTACTCCTCACCATTTTTATCACTACCAGCTTCTAGCTTATGCTAACACTACAGTTTCAGTCTC

TCCGTCGGCATTTTTTTTTT

In100 candidate1463 (in intron):

LOCUS ENSG00000129083

DESCRIPTION Coatomer subunit beta (Beta-coat protein) (Beta-COP).

[Source:Uiprot/SWISSPROT;Acc:P53618]

AGAAAATATATTTAACTTTATGTCTTATATTTTATATGTTAATTTTATTCTTGATTTTTTTCCCAGTTAATTTCTCTTTG

CCCTGATCTCCTCATTTGTT

In100 candidate1464 (in intron):

LOCUS ENSG00000159579

DESCRIPTION RING finger and SPRY domain-containing protein 1 precursor.

[Sorce:Uniprot/SWISSPROT;Acc:Q96DX4]

AGGATGCATGTTGCCCTAACCTACTATTTAATGTCTTTCTATGTTATGCTTTTTTCTGTACTCAGTGACTTTTTTTTTTT

TTTTTTTTTGAGACCGAGAT

In100 candidate1465 (in intron):

LOCUS ENSG00000173960

DESCRIPTION UBX domain-containing protein 4.

[Source:Uniprot/SWISSPROT;Acc:68543]

GCCAATAACTTAAGAATTTGTTACGGTTTTCTGGTCACGTTTCTTTATGGGTCCCTCTGTTTCAGGATTTTGGCTTATAA

ATCCCAGCTTCTTGTCTCTC

In100 candidate1466 (in intron):

LOCUS ENSG00000135457

DESCRIPTION Alpha-globin transcription factor CP2 (Transcription factor LSF

(SAA3 enhancer factor). [Source:Uniprot/SWISSPROT;Acc:Q12800]

TATACCTCCATGCTGAGGTGTGGATTTCCATTTAGGGGTATACATTTTTAACATACACTCAACTTATCCTTTTAACAGGT

ATGATTTCTATATCTTTTCT

In100 candidate1467 (in intron):

LOCUS ENSG00000145348

DESCRIPTION TBC domain-containing protein kinase-like protein.

[Source:Unipot/SWISSPROT;Acc:Q8TEA7]

AAACTTAAGAGCCATTATTCACTCATCCTTGACCTTTAACTCCTGTATATTTTCTCTTTCTGTATTTTCCTATACAGGCT

CTCATACATCTATCTTTCTT

In100 candidate1468 (in intron):

LOCUS ENSG00000113356

DESCRIPTION DNA-directed RNA polymerase III subunit G (EC 2.7.7.6) (DNAdircted RNA polymerase III 32 kDa polypeptide) (RNA polymerase III C32 subunit. [Source:Uniprot/SWISSPROT;Acc:O15318]

AACAAGTTTTAAAAAGAGTTACATATGTAACTCTTTTAAAAACCTCTCATTTTTCCTCCTGTTGGTTAATATGTACAGTT

TTAATGCTTTTTTTTTGGTC

In100 candidate1469 (in intron):

LOCUS ENSG00000152270

DESCRIPTION cGMP-inhibited 3',5'-cyclic phosphodiesterase B (EC 3.1.4.17) (yclic GMP-inhibited phosphodiesterase B) (CGI-PDE B) (CGIPDE1) (CGIP1). [Sorce:Uniprot/SWISSPROT;Acc:Q13370]

TTACGTTGAAGTATTCTTTTATCCCTGCCACAACTTCATTAACCGCATACTCCTTATTATCTGTGTTTCCACCAGATTTC

TTGTAATTTGCGTAATCCTC

In100 candidate1470 (in intron):

LOCUS ENSG00000152270

DESCRIPTION cGMP-inhibited 3',5'-cyclic phosphodiesterase B (EC 3.1.4.17) (yclic GMP-inhibited phosphodiesterase B) (CGI-PDE B) (CGIPDE1) (CGIP1). [Sorce:Uniprot/SWISSPROT;Acc:Q13370]

TTTAAATTACCTTACCATCTATTTAACTTAAAAATAAGCCACTTTTATTATTCAGTTAACAAACTGAATATCATTCTTTG

TTTTTATTTTTATTTTTTTT

In100 candidate1471 (in intron):

LOCUS ENSG00000164199

DESCRIPTION G-protein coupled receptor 98 precursor (Monogenic audiogenic

sizure susceptibility protein 1 homolog) (Very large G-protein coupled recepor 1) (Usher syndrome type-2C protein). [Source:Uniprot/SWISSPROT;Acc:Q8WXG]

CTTACACTCAATATTCTATAATCATGAATTATTTTCTAATCTAACTCCTAAATGTTTGTTTTCCTAATCCAGTTTCTCAT

TTCAACCATCATCTCTTATT

In100 candidate1472 (in intron):

LOCUS ENSG00000164199

DESCRIPTION G-protein coupled receptor 98 precursor (Monogenic audiogenic sizure susceptibility protein 1 homolog) (Very large G-protein coupled recepor 1) (Usher syndrome type-2C protein). [Source:Uniprot/SWISSPROT;Acc:Q8WXG]

AACTCACCTCTACTTGAATATGCTCCACTTAACTCAAATTCAAAATTTCTTTTTTTTTCTTTTCCAGATAATTCTTTTTT

TATTTTTAATTATACTTTAA

In100 candidate1473 (in intron):

LOCUS ENSG00000104447

DESCRIPTION Zinc finger transcription factor Trps1 (Zinc finger protein GC7) (Tricho-rhino-phalangeal syndrome type I protein).

[Source:Uniprot/SWISSPOT;Acc:Q9UHF7]

AATTTTATTGTATATTCAATTTGGAGATTAGGCTCTAACCTACCCTATATTTGGCTCTGTTTCAGGATATTTAAATTTCT

TTCATTGTAATTTTTGTTCC

In100 candidate1474 (in intron):

LOCUS ENSG00000175662

DESCRIPTION TOM1-like protein 2 (Target of Myb-like protein 2).

[Source:Unirot/SWISSPROT;Acc:Q6ZVM7]

AGTGTCTAGTTGCAATAACTGTCTTATCTCTCTGGTTCTTGGTAAAACATTTCTTCTTTTATTCCATTTGGTTTTCAGCC

TTCTTGTTTTATTTGTCTTG

In100 candidate1475 (in intron):

LOCUS ENSG00000160967

DESCRIPTION Protein CASP. [Source:Uniprot/SWISSPROT;Acc:Q13948]

TTTTTAACCTATATACAATTAATCTTTTTCTCTTCACCATTTAAGATTTTCCTTTTCTAATTTTGATTTCAGAAACTCTC

TTACGCTTACTGGTGTTCTT

In100 candidate1476 (in intron):

LOCUS ENSG00000160967

DESCRIPTION Protein CASP. [Source:Uniprot/SWISSPROT;Acc:Q13948]

TACTGATATAACTCTTAACTTAAATTTCTCTTTATTGTTGAATTGTTGATCCTTTTCTTTTTTCTAATTCACAGTATCTC

TGTCCTAGGCTTTCTGATCT

In100 candidate1477 (in intron):

LOCUS ENSG00000164023

DESCRIPTION Phosphatidylcholine:ceramide cholinephosphotransferase 2 (EC

2..8.27) (Sphingomyelin synthase 2). [Source:Uniprot/SWISSPROT;Acc:Q8NHU3]

AGTGTTATCTCATAAGCCTGGTGAGCCTAATTGAATAGCCACCCTTTAACGTCCCATCCTGCTTTGTCTTCGTAGCAGTT

TTAATCTTTCATTATTATTG

In100 candidate1478 (in intron):

LOCUS ENSG00000113391

DESCRIPTION

TAAATAACCATAACAATTATTTGTCATTTTAATATTTCACCTCTCACTGCCAATCTTTTTATGCTGTCACTTCAGGCATA

TATGCTTAATTTTACTTCCT

In100 candidate1479 (in intron):

LOCUS ENSG00000139629

DESCRIPTION Polypeptide N-acetylgalactosaminyltransferase 6 (EC 2.4.1.41)

(rotein-UDP acetylgalactosaminyltransferase 6) (UDP- GalNAc:polypeptide

Nactylgalactosaminyltransferase

6) (Polypeptide GalNAc transferase 6) (GalNAc-6)

(pp-GaNTase 6). [Source:Uniprot/SWISSPROT;Acc:Q8NCL4]

TTGAGAAAAAAACACTTTGAAGTCCCCCATTAACCATCTCTAATCCTGTACCGTTTTCTTCATCTTCAGAAACTGGTTAT

GTCATTTCTTTAGCTTTCTT

In100 candidate1480 (in intron):

LOCUS ENSG00000185261

DESCRIPTION

TCATCAAATATTTTTCATTTTAAAAATGCAAGTTTTGTGTATAATCACATAACAATGTATTCCTTTAAATTTTTTCAGAT

ATATAATTCTTTCCTTCTCT

In100 candidate1481 (in intron):

LOCUS ENSG00000141034

DESCRIPTION Uncharacterized protein C17orf39.

[Source:Uniprot/SWISSPROT;AccQ8IVV7]

TATGTCACGGTTTACCTATGTAACTGTTGTGGAGATTATTTTGTCACCCAGGTATTAAGCCTAGTACTGAATAGTTATTT

CTTCTGCTCCTCTCCCTTCT

In100 candidate1482 (in intron):

LOCUS ENSG00000110693

DESCRIPTION Transcription factor SOX-6. [Source:Uniprot/SWISSPROT;Acc:P3571]

CAAACTTCATAAAAATGATGTCATACTGTAACCTCTTGTGACTTGCTTTTTATTCTTATATTCTATCATCAGTTTTGCTG

TGTGAACTTTCATTCATTTT

In100 candidate1483 (in intron):

LOCUS ENSG00000110693

DESCRIPTION Transcription factor SOX-6. [Source:Uniprot/SWISSPROT;Acc:P3571]

CTCCCACTCTGTGGGTTGTCTGTTAACTCTGCTGATTATTTCTTTTGTTGTGCAGAAGCTTTTTAGTTTAATTAAGTCCC

ATCTATTTATCTTTGCTTTT

In100 candidate1484 (in intron):

LOCUS ENSG00000182197

DESCRIPTION Exostosin-1 (EC 2.4.1.224) (EC 2.4.1.225) (Glucuronosyl-

Nacetlglucosaminyl-

proteoglycan/N-acetylglucosaminyl-proteoglycan 4- alpha-

Naceylglucosaminyltransferase)

(Putative tumor suppressor protein EXT1) (Multipe

exostoses protein 1). [Source:Uniprot/SWISSPROT;Acc:Q16394]

GCATCGATTTTACATGAATATTTGGTATTCAAACTAAAGGCTATTTAACTGGTTTCAACTGCCTTTTATTTTATCCAGCT

TAATTTTATCTTCCTTCTGA

In100 candidate1485 (in intron):

LOCUS ENSG00000198677

DESCRIPTION Tetratricopeptide repeat protein KIAA0372 (TPR repeat protein KAA0372). [Source:Uniprot/SWISSPROT;Acc:Q6PGP7]

AAATAAAATAAAATAGATGGGGTTCTATGCTTTTGCTATTTTTATATATTAACAAATTAAAAATGTTTTTATTTGCAGAT

TTTACATTTTTATTGGCATT

In100 candidate1486 (in intron):

LOCUS ENSG00000177570

DESCRIPTION Sterile alpha motif domain-containing protein 12. [Source:Uniprt/SWISSPROT;Acc:Q8N8I0]

TAACCTATGCTCGTCTATCTTTATCACAGCTTACTGATTATCCACATGCATTACTTTTACTTGATTATTTTCATGACATT

TTATTTATTTATTTTTATTT

In100 candidate1487 (in intron):

LOCUS ENSG00000188517

DESCRIPTION Collagen alpha-1(XXV) chain (CLAC-P) (Alzheimer disease amyloid associated protein) (AMY) [Contains: Collagen-like Alzheimer amyloid plaqu component (CLAC)]. [Source:Uniprot/SWISSPROT;Acc:Q9BXS0]

GTGTTTTGGGTTTTTTCTTTCACATTAACTATTTTTGCCCTCATGTTTTCATCAGAATTTTTTATACATTAGTTATACAT

ATATATTTATTTTTTCTCCT

In100 candidate1488 (in intron):

LOCUS ENSG00000188517

DESCRIPTION Collagen alpha-1(XXV) chain (CLAC-P) (Alzheimer disease amyloid associated protein) (AMY) [Contains: Collagen-like Alzheimer amyloid plaqu component (CLAC)]. [Source:Uniprot/SWISSPROT;Acc:Q9BXS0]

TTCTTGAAATTCTCTCCAATCTTGATTTCTGTAACTCTAATTTTGTTGTCATTGTTTTTAGGTTTTTTTTATCCTCAGTT

TCCATTTTGGAACACTTTTC

In100 candidate1489 (in intron):

LOCUS ENSG00000188517

DESCRIPTION Collagen alpha-1(XXV) chain (CLAC-P) (Alzheimer disease amyloid associated protein) (AMY) [Contains: Collagen-like Alzheimer amyloid plaqu component (CLAC)]. [Source:Uniprot/SWISSPROT;Acc:Q9BXS0]

ACAATAAATGTGTCCACTTATCTCTCTCTTCTACTAACCACCAAATTCAACTTACTTTCATGTTCTTGATTACTTCAGTG

GCTTTCCAATTAATCTTTCT

In100 candidate1490 (in intron):

LOCUS ENSG00000188517

DESCRIPTION Collagen alpha-1(XXV) chain (CLAC-P) (Alzheimer disease amyloid associated protein) (AMY) [Contains: Collagen-like Alzheimer amyloid plaqu component (CLAC)]. [Source:Uniprot/SWISSPROT;Acc:Q9BXS0]

GTATAGGTTCTTTGCTCCAACTATCATTTTTAACTAGTAGAGCACTAAGTCGTTTTTTTTTTTTAAATCTATTTTCAGCT

CAGCCTATTTTTTGGCCTTT

In100 candidate1491 (in intron):

LOCUS ENSG00000188517

DESCRIPTION Collagen alpha-1(XXV) chain (CLAC-P) (Alzheimer disease amyloid associated protein) (AMY) [Contains: Collagen-like Alzheimer amyloid plaqu component (CLAC)]. [Source:Uniprot/SWISSPROT;Acc:Q9BXS0]

GCACATCTCATATATTTCTGTTATGTATATCTCTTTTAACCTTTCAATATTCATGTTGCTTTGCTTCAGACATTATTCAG

TTTTATTGTAACTCCCATCC

In100 candidate1492 (in intron):

LOCUS ENSG00000188211

DESCRIPTION

AGTTTGGGAAGTTTCTATTAACATCTTCAAGCTCACTGATTCTTTCCTTGTCAGTTTCTTTTTTTTTTTGAGACGGAGTT

TTGCTCTTGTTGCCCAGTCT

In100 candidate1493 (in intron):

LOCUS ENSG00000177302

DESCRIPTION DNA topoisomerase 3-alpha (EC 5.99.1.2) (DNA topoisomerase III lpha). [Source:Uniprot/SWISSPROT;Acc:Q13472]

TAACTTCTTATCCTTCAAGGCTCTGCTGAATTCATTCTCTCTCTCTCCCTTTTTTTTTTTTTTTTTAAGCAATCCAGAG

GTCTTGTATTTATCTATTTT

In100 candidate1494 (in intron):

LOCUS ENSG00000102753

DESCRIPTION Importin alpha-3 subunit (Karyopherin alpha-3 subunit) (SRP1-gama). [Source:Uniprot/SWISSPROT;Acc:O00505]

AGTTCTTAATTTTGGTTATGTGTCTAACTTAATAATTATTTTTTCATATCTCAGAGTTCTGTTTGGTTCTTTTCATTTTT

CAGATCTGGTCATTTCTGAT

In100 candidate1495 (in intron):

LOCUS ENSG00000102753

DESCRIPTION Importin alpha-3 subunit (Karyopherin alpha-3 subunit) (SRP1-gama). [Source:Uniprot/SWISSPROT;Acc:O00505]

GTCTGCAAAGTCTGTAGTGATAGTACCTTTTTCATTTCTAATATTATAACTCTGCATTTTCTCTTTTTTTTTGGCCAGTC

TGCTGATTTTTTTTTTTTTT

In100 candidate1496 (in intron):

LOCUS ENSG00000102753

DESCRIPTION Importin alpha-3 subunit (Karyopherin alpha-3 subunit) (SRP1-gama). [Source:Uniprot/SWISSPROT;Acc:O00505]

TTAACACACATATGCACATATTTTAATTCTTTTGGGAGCTTTTTTCTGTCCCTCTGATTATATCTCCTCTTGATACAGAT

CTTAACATTTACTTTCCTAT

In100 candidate1497 (in intron):

LOCUS ENSG00000102753

DESCRIPTION Importin alpha-3 subunit (Karyopherin alpha-3 subunit) (SRP1-gama). [Source:Uniprot/SWISSPROT;Acc:O00505]

GGGATTTAACTTTGTAAAGTATCCTTTTTTCTTTGTAATTCTTTTTAGATTAAATTCTACTGGTCGTCTTTTAACAGGGT

TACAACCTCAGCTTTCTTTT

In100 candidate1498 (in intron):

LOCUS ENSG00000064313

DESCRIPTION Transcription initiation factor TFIID subunit 2 (Transcription nitiation factor TFIID 150 kDa subunit) (TBP-associated factor 150 kDa) (TAII-150) (TAFII150) (150 kDa cofactor of initiator function). [Source:Unipro/SWISSPROT;Acc:Q6P1X5]

AGTTGAGGTGTTTAGGAAATTTCCAATATTAAATTTAACTCTTTCATTTAGATGATCTTCCTTGCTTCTTCTTTTCAGGT

TACTCATTTTTGTTTGCCCC

In100 candidate1499 (in intron):

LOCUS ENSG00000161040

DESCRIPTION F-box/LRR-repeat protein 13 (F-box and leucine-rich repeat protin 13). [Source:Uniprot/SWISSPROT;Acc:Q8NEE6]

CACCCACTGAGTTTTAAATTTCAATAACTATATTTTTAATTTTCATGATTTTATTTGGTTCTTTTACAGATCTTCCCAGT

CCTGTTTTGACAACATTTGT

In100 candidate1500 (in intron):

LOCUS ENSG00000161040

DESCRIPTION F-box/LRR-repeat protein 13 (F-box and leucine-rich repeat protin 13). [Source:Uniprot/SWISSPROT;Acc:Q8NEE6]

ATAACTTGATAAGTATTGTTTTAACTTTCTAAACCAACTATTCTATATTTGCTTTCCTCTTTCACTCAGTAATGTTTATT

TATTTATTTATTTAATTTTT

In100 candidate1501 (in intron):

LOCUS ENSG00000161040

DESCRIPTION F-box/LRR-repeat protein 13 (F-box and leucine-rich repeat protin 13). [Source:Uniprot/SWISSPROT;Acc:Q8NEE6]

TCAATATTGTAATTATATTTATAATGAGTATGTAGTCTTAACTCTAGGCCATTCATTGATGGTCTTTTTTTCTCACAGCT

TACTTATTCTTTAGCCTTTT

In100 candidate1502 (in intron):

LOCUS ENSG00000164292

DESCRIPTION Rho-related BTB domain-containing protein 3. [Source:Uniprot/SWSSPROT;Acc:O94955]

TTCTTTAAGATCCTAGTGTTTGCTGCATCTGGAGCTAACCCCCTTAAAATTATTTTATCTCTAGCTTATTTTATCCAGCT

CTCTTCCTTTTTATTTGAGC

In100 candidate1503 (in intron):

LOCUS ENSG00000138092

DESCRIPTION Centromere protein O (CENP-O) (Interphase centromere complex prtein 36). [Source:Uniprot/SWISSPROT;Acc:Q9BU64]

TTAAACTTTATTCTTATTGCCCTTGTTTCTTGTTTAATAAATCTTTGTCTAACTTAAGATCATGCTGATCTTTTCCAGGT

ATTTTATTTTTTTTATTTTT

In100 candidate1504 (in intron):

LOCUS ENSG00000155792

DESCRIPTION DEP domain containing 6 [Source:RefSeq_peptide;Acc:NP_073620]

GCCTGGGTTCTTATTCTGCCTCCACCATTTATTCTGTAACTTTAGGACAATATGTTTAGCCTCTCTGTGTCTCAGTTTCC

TCTTCTACGAAATGTTATTC

In100 candidate1505 (in intron):

LOCUS ENSG00000187955

DESCRIPTION Collagen alpha-1(XIV) chain precursor (Undulin).

[Source:Unipro/SWISSPROT;Acc:Q05707]

AATTTTTTCAAAAAACTAACTAACTACTGGATTCGTTGTCTTTTGAATATTTTTTCATGTCTTGATTTCCTTCAGTTCAG

CCCTGATCTTGGTTATTTCT

In100 candidate1506 (in intron):

LOCUS ENSG00000118985

DESCRIPTION RNA polymerase II elongation factor ELL2.

[Source:Uniprot/SWISSROT;Acc:O00472]

TCATATCATGTATTATTCTAAGCATATTATTGGCTTTTAACAAGAGCTGAGTGTATTTTATTTCTTCAGGATCAACTGTC

TGATTTCTTGTGCTATTTTC

In100 candidate1507 (in intron):

LOCUS ENSG00000198218

DESCRIPTION Glutamine-rich protein 1.

[Source:Uniprot/SWISSPROT;Acc:Q2TAL8]EXON

AAGGCTTAAAGAATGGATTTTGATGGTTTATAGCAAGTAACTTTTTGAAGCTTTTATTTTTTCTGACAGCTGAATATCTA

GCTTATTTGCCTTTTTGCTC

In100 candidate1508 (in intron):

LOCUS ENSG00000198218

DESCRIPTION Glutamine-rich protein 1.

[Source:Uniprot/SWISSPROT;Acc:Q2TAL8]EXON

TTATATAAATTAAAAAAATTGAAGGGAAGTAATATAAATAACATTTAATTTCTTTTTTGTTTTTTTTTCGAGACAGAGT

TTTGCTCTTTCACCTAGGCT

In100 candidate1509 (in intron):

LOCUS ENSG00000172167

DESCRIPTION Mdm2, transformed 3T3 cell double minute 2, p53 binding

proteinbinding protein [Source:RefSeq_peptide;Acc:NP_071328]

AAGATGAGTAATTAACACCCTTATTTTTCTTCCCTTTTTATCTTCTATCTCTAAACCTTATGCTATTTGCAGTATACAAG

TCTTAATTTTATTTTTCTTC

In100 candidate1510 (in intron):

LOCUS ENSG00000172164

DESCRIPTION Beta-1-syntrophin (59 kDa dystrophin-associated protein A1 basi

component 1) (DAPA1B) (Tax interaction protein 43) (TIP-43) (Syntrophin

2)(BSYN2). [Source:Uniprot/SWISSPROT;Acc:Q13884]

TTAAAATTATTTTTAAAGAATTTTCCCATTCCTTTAAGAGTTACAAGACTAACCATGCTCTCTATTTTCTTAGGTCAGTT

TTGTTAAGTTTATTTTTTTC

In100 candidate1511 (in intron):

LOCUS ENSG00000172164

DESCRIPTION Beta-1-syntrophin (59 kDa dystrophin-associated protein A1 basi

component 1) (DAPA1B) (Tax interaction protein 43) (TIP-43) (Syntrophin

2)(BSYN2). [Source:Uniprot/SWISSPROT;Acc:Q13884]

AAATATGGGTTTAAATCCCAAGTCTGTTACTTATTCCCTTTAACTTTAAGTAAATTATCCAATCTCTCTGATTTTCAGGT

TCCTTCTTTGCTATAGTCTT

In100 candidate1512 (in intron):

LOCUS ENSG00000153113

DESCRIPTION Calpastatin (Calpain inhibitor) (Sperm BS-17 component).

[Sourc:Uniprot/SWISSPROT;Acc:P20810]

AAGTGGCCTGGCTCCAAAGCCATTCTTCTAACCATGACACAACTTTTTTTCTTTTCTTTTCTTTTTTGAAACAGGCTCTT

GGGGTCTTGCTCTGTCACTT

In100 candidate1513 (in intron):

LOCUS ENSG00000141127

DESCRIPTION Phosphoribosyl pyrophosphate synthetase-associated protein 2 (PPP

synthetase-associated protein 2) (41 kDa phosphoribosypyrophosphate

syntetaseassociated

protein) (PAP41). [Source:Uniprot/SWISSPROT;Acc:O60256]

ATTAGTATATCTTACCAATTTAGAATTTAGAAAACTAAGTTTTCTGACATAACTGCTGACTTTTACATGTTCTTTCAGTG

TTTTTCCTTCTTTCTTTCTT

In100 candidate1514 (in intron):

LOCUS ENSG00000129158

DESCRIPTION Secretion-regulating guanine nucleotide exchange factor (Guanin nucleotide exchange factor-related protein) (Deafness locus-associated puttive guanine nucleotide exchange factor) (DelGEF).

[Source:Uniprot/SWISSPRO;Acc:Q9UGK8]

ACCTGTGTATACTCTCTAGTTGTCTAATCTCAAAGTAACCTGTTTTATCCATCTATATCTTTCTAATCCATTTATCAGTC

TCCATTTGTGTATCTATCTA

In100 candidate1515 (in intron):

LOCUS ENSG00000129158

DESCRIPTION Secretion-regulating guanine nucleotide exchange factor (Guanin nucleotide exchange factor-related protein) (Deafness locus-associated puttive guanine nucleotide exchange factor) (DelGEF).

[Source:Uniprot/SWISSPRO;Acc:Q9UGK8]

GAGCTTATTACTGAAAGATGTCCTGCTGCAAGTCCTTCTGTTAAGGGGATAACCAATCCTTTGCTTTTCTGAGTTCAGCA

TCCTGGCTTTTTTTTTCCTA

In100 candidate1516 (in intron):

LOCUS ENSG00000138660

DESCRIPTION Uncharacterized protein C4orf16.

[Source:Uniprot/SWISSPROT;Acc:63HQ0]

ATTCTTCTGTTTCTTCATTTTACACATCTTTTTTAAAAATATGATTAACTTCCTTCTCTTTCTTTTAAATCTTACAGGAG

CCCTTCTGTTTCCTTAAGTT

In100 candidate1517 (in intron):

LOCUS ENSG00000125107

DESCRIPTION CCR4-NOT transcription complex, subunit 1 isoform a

[Source:Refeq_peptide;Acc:NP_057368]

CTGGCCGAAATATGTATTCTTAACCTGTGTTTTGTTTTTGCTGTTTGTTTTACAGTTTCTTAAAATAGGTTCCATCCTGA

TTCTTTGTGTTTTTACTTTT

In100 candidate1518 (in intron):

LOCUS ENSG00000125107

DESCRIPTION CCR4-NOT transcription complex, subunit 1 isoform a

[Source:Refeq_peptide;Acc:NP_057368]

CTAATAACTAACTTGGGCCCTGATTTTCTCCCTTTTCAGTACTTTAAAAAATTTATTATATGCTTTTACTCTACTTTCTT

TGGTTTTCATTAGTTCTTTT

In100 candidate1519 (in intron):

LOCUS ENSG00000073331

DESCRIPTION Alpha-protein kinase 1 (EC 2.7.11.-) (Lymphocyte alpha-protein

inase) (Chromosome 4 kinase). [Source:Uniprot/SWISSPROT;Acc:Q96QP1]

ATTATATACTAATTTATTAATTATTGACATTTGGTATAACACTGTGCTCTCCTCATTATTTTTCATCAGGAATGTCTTTA

TTATTCTTGAACTGTTTTTC

In100 candidate1520 (in intron):

LOCUS ENSG00000166788

DESCRIPTION Protein SAAL1. [Source:Uniprot/SWISSPROT;Acc:Q96ER3]

ATCAAGTGGCATGGCTGGATCATACGGTAGGTGTATGCTTAACTTTTTAAGAAACTTTCAATTTGTTTTCCTAAGCAGTT

TTTTACCATTTTACATAACC

In100 candidate1521 (in intron):

LOCUS ENSG00000166788

DESCRIPTION Protein SAAL1. [Source:Uniprot/SWISSPROT;Acc:Q96ER3]

AAAAACAAAAAATTTCTGACATTTTTGTTGACATCGTGTTAACTTGATAGGATATGTTATGCCCTTTTATTTGTTCAGGT

CTTTTTTGTGTCCCTCAGTA

In100 candidate1522 (in intron):

LOCUS ENSG00000189056

DESCRIPTION Reelin precursor (EC 3.4.21.-).

[Source:Uniprot/SWISSPROT;Acc:P8509]

GATTTGCTTAATGTGACTTAAGTTTTATAACTGTGTTGTGACTCTTTATTACTGGATTTTGCTTTCATTCCTGATCAGTT

GTCTTTCTTCTTTCCTCTCT

In100 candidate1523 (in intron):

LOCUS ENSG00000072134

DESCRIPTION Epsin-2 (EPS-15-interacting protein 2).

[Source:Uniprot/SWISSPRT;Acc:O95208]

AGAGCACATTTTTATGGAATACTTCTTGCCTCCTGGGGCATAACCCTAGCCTTGGAAATTTTTTTTCCAGTTTTGTTCT

GTTTGCTGTTTTGTTTAGTC

In100 candidate1524 (in intron):

LOCUS ENSG00000102786

DESCRIPTION Integrator complex subunit 6 (Int6) (Protein deleted in cancer)

(Protein DDX26) (DBI-1) (Deleted in cancer 1) (DICE1).

[Source:Uniprot/SWSSPROT;Acc:Q9UL03]

TGCTAATGATAGGAATTCTTGCAACTAACTAGTTTACTATATTCTTCTTCTTCTTCTTCTTTTTTTTAATCTCAGGC

CGTTCATATTCTGTGTGTTC

In100 candidate1525 (in intron):

LOCUS ENSG00000139668

DESCRIPTION WD repeat and FYVE domain-containing protein 2 (WD40- and FYVE

omain- containing protein 2) (Zinc finger FYVE domain-containing protein 22.

[Source:Uniprot/SWISSPROT;Acc:Q96P53]

AACATTTTTTAATATCTGGTAGGGCTAACCTCCCTATCACATACCATATTGTTCTTATTTTCAGAGTATTCTTGGCTCTT

TTTGCTTATTTATTTTTCCA

In100 candidate1526 (in intron):

LOCUS ENSG00000138101

DESCRIPTION Dystrobrevin beta (Beta-dystrobrevin) (DTN-B).

[Source:Uniprot/WISSPROT;Acc:O60941]

AGAGAGCATGGTTCATATGATTTTAATCCTTTAACATTTATTGAGATTTGCTTTATGGCTCAGGATATGGTCAATTTCTT

TTTTTTTTTTTTTTTTTTG

In100 candidate1527 (in intron):

LOCUS ENSG00000145362

DESCRIPTION Ankyrin-2 (Brain ankyrin) (Ankyrin-B) (Ankyrin, nonerythroid).

Source:Uniprot/SWISSPROT;Acc:Q01484]

TAAAATGTAAAATATAATAGTGATTCCTATGTCATTAACATTTCTTATTCTTTCATTCATCAAATATTTATTTCTCAGCA

CTCATAACCTCTTCATTTTT

In100 candidate1528 (in intron):

LOCUS ENSG00000125166

DESCRIPTION Aspartate aminotransferase, mitochondrial precursor (EC 2.6.1.1

(Transaminase A) (Glutamate oxaloacetate transaminase 2).

[Source:Uniprot/WISSPROT;Acc:P00505]

AGAAAGTTTGTGAAAGGGAAAAAATTAACCATAACTCTAGCTATTCTAACTCAGCTTTTTTTTCCCTTACCCATTGTTT

GTTTGTTTTTTCTTTATTTG

In100 candidate1529 (in intron):

LOCUS ENSG00000187416

DESCRIPTION Lipoma HMGIC fusion partner-like 3 protein.

[Source:Uniprot/SWISPROT;Acc:Q86UP9]

ATTTCTTCCTTTGCTTTCTTTAAGTTTATTTAGTTGTTTTTCCCTAACTTCATCTGGGATGCTTTATTTATTTTTCAGTC

TTTCTTATTCATTGCTATAA

In100 candidate1530 (in intron):

LOCUS ENSG00000187416

DESCRIPTION Lipoma HMGIC fusion partner-like 3 protein.

[Source:Uniprot/SWISPROT;Acc:Q86UP9]

TTATTTTATTTTAGGTTTACTGTTTAACATACTTCATTTTTTTCTTCTTTCTTCTCCTTAATTTTCTGGCTTGGTCAGCT

CTCTCTTCTTTGCATTTTTG

In100 candidate1531 (in intron):

LOCUS ENSG00000150394

DESCRIPTION Cadherin-8 precursor. [Source:Uniprot/SWISSPROT;Acc:P55286]

TTCACTGAGGCTCTGCTCATTTTTCTTTATTTTTTTTTAACTGCTGTTCTTTGATTCTCTACTGATCTTCACTTTCAGTA

ATTCTTTCTTCTGCCAATTT

In100 candidate1532 (in intron):

LOCUS ENSG00000173402

DESCRIPTION Dystroglycan precursor (Dystrophin-associated glycoprotein 1)

[ontains: Alpha-dystroglycan (Alpha-DG); Beta-dystroglycan (Beta- DG)].

[Souce:Uniprot/SWISSPROT;Acc:Q14118]

TTCTTGAGATCAATTTTATTAATCTTTTCAAATAACTAGTTATTTGTTTCAATGGTTTTTCTCTGTTTTTGTTTTCAGTT

TCATTGGGGTTTTTTGCTTT

In100 candidate1533 (in intron):

LOCUS ENSG00000181171

DESCRIPTION Putative uncharacterized protein C8orf54.

[Source:Uniprot/SWISSROT;Acc:Q8NA97]

AGCACTATGCTGGTACACTAACACTACTAGCATTTTTCTTGTTTCCTACAGAGTCTCTGTAGTAAGTCTTCCTTTGCTTT

TTCCTACCTTTGATTTATGT

In100 candidate1534 (in intron):

LOCUS ENSG00000084733

DESCRIPTION Ras-related protein Rab-10. [Source:Uniprot/SWISSPROT;Acc:P6102]

ACTGTGCCCCGGCTAAATCTGATACCTCTTTGCTTAGAGCTTCAACTCCTAACTTAGATTGTTTTGTTTACCCTGCAGCA

ATTCATCTTTGCTCTCTTTT

In100 candidate1535 (in intron):

LOCUS ENSG00000145349

DESCRIPTION Calcium/calmodulin-dependent protein kinase type II delta

chain(EC 2.7.11.17) (CaM-kinase II delta chain) (CaM kinase II subunit delta)

(CMK-II subunit delta). [Source:Uniprot/SWISSPROT;Acc:Q13557]

CATTCCTCAAGTTTTTATGAAATAAATTAACTGTCATTTTTTGTTTGTTCTTTTGTCTTTAGCAGATGATTTTAACCATA

AATTTTGCCTTTTTATCCTT

In100 candidate1536 (in intron):

LOCUS ENSG00000145349

DESCRIPTION Calcium/calmodulin-dependent protein kinase type II delta

chain(EC 2.7.11.17) (CaM-kinase II delta chain) (CaM kinase II subunit delta)

(CMK-II subunit delta). [Source:Uniprot/SWISSPROT;Acc:Q13557]

AATTGATATGACTATGTTATATTCTAGTGCTTTTAACACACCTTCATCATCATCCTTAGTCTTTCTTTGTCTCCTCAGTT

TCTTTTAATTTCTTTGTCTC

In100 candidate1537 (in intron):

LOCUS ENSG00000074319

DESCRIPTION Tumor susceptibility gene 101 protein.

[Source:Uniprot/SWISSPRO;Acc:Q99816]

GATGTCTTCATTCTTCTCTGAATAACTCCTTGAAGTAACTCTCTTTGTTATCTTCAGTACCTCTTCTCCGTTTTTCTCTT

AAACCTCCTTTTGGACTTTT

In100 candidate1538 (in intron):

LOCUS ENSG00000136098

DESCRIPTION Serine/threonine-protein kinase Nek3 (EC 2.7.11.1) (NimA-relate

protein kinase 3) (HSPK 36). [Source:Uniprot/SWISSPROT;Acc:P51956]

CATTCTTGTATAATATAGAATATGTAACTATATACATATTATTTTTTTAACTCAGTCACTGTAATGGTTCGTTTTAATTT

TTCATTTTGGATTTTTTTTT

In100 candidate1539 (in intron):

LOCUS ENSG00000083290

DESCRIPTION Serine/threonine-protein kinase ULK2 (EC 2.7.11.1) (Unc-51-

likekinase 2). [Source:Uniprot/SWISSPROT;Acc:Q8IYT8]

TAATAACTCTTTTCCACTAATCTTTTTATCAGTGTAGTACCCTTGCTTTTAATTTTTCCTGCTGTTCTGCAGTTCAGAGA

CCCTCTGTTTTACTCTGTCC

In100 candidate1540 (in intron):

LOCUS ENSG00000083290

DESCRIPTION Serine/threonine-protein kinase ULK2 (EC 2.7.11.1) (Unc-51-

likekinase 2). [Source:Uniprot/SWISSPROT;Acc:Q8IYT8]

GGTCATTGTTTAGTCTTTTCTACTTAAGGCCAATAACCCTTAGTTTTGTTTTTTTTTTTTTTGGCAGCTTTTTTTAGA

TTTGTAGGCTTGCTTCATTC

In100 candidate1541 (in intron):

LOCUS ENSG00000146776

DESCRIPTION Ataxin-7-like protein 4. [Source:Uniprot/SWISSPROT;Acc:Q8N2T0]

EXON

AGGTAATAACATCCTAATATCTTTGAAGATAAGTTTTTAGTTTTTCTCTTTCCATCATCGTCTTAGTTTTTCCAGGT

TTGTTTCCTTCCTCTGTTCA

In100 candidate1542 (in intron):

LOCUS ENSG00000180801

DESCRIPTION arylsulfatase family, member J (ARSJ), mRNA

[Source:RefSeq_dna;cc:NM_024590]

TCTCCCTTCCCACCTAATTTAATGTAACTGCCTTCTGTCACTCTCTTGATTAGCTTATTTGCTTTTTCCCATAGCAGTT

ATCACTACCTGAATTTTCTT

In100 candidate1543 (in intron):

LOCUS ENSG00000197585

DESCRIPTION

TTTTTTGTAAAGTAACTGTTAAGTTTTCTTACCCATTTTTCTCCTGGATTATTTGTCTTTTTTTCTCTTGATTTGCAGTT

TTTTATATATTCTGATTTCC

In100 candidate1544 (in intron):

LOCUS ENSG00000128487

DESCRIPTION Sperm antigen with calponin homology and coiled-coil domains 1

Nuclear structure protein 5) (NSP5) (Sperm antigen HCMOGT-1).

[Source:Uniprt/SWISSPROT;Acc:Q5M775]

CTTCCCGTAGTGCTGCTATTTGGTTGTGCATTCTGTCTGCTGAGACTGTAACCTCAATACACATCACATATTTTCCAGAT

CATCCTGTTCTTTATCTCAG

In100 candidate1545 (in intron):

LOCUS ENSG00000128487

DESCRIPTION Sperm antigen with calponin homology and coiled-coil domains 1

Nuclear structure protein 5) (NSP5) (Sperm antigen HCMOGT-1).

[Source:Uniprt/SWISSPROT;Acc:Q5M775]

AGGAAATGAATTAACAATTTGTACAAGAACTTTTCTGTTCCTTCCTTCTCTAGGCATCTCATTATTTATACAGTTTCTTT

TTGCTCAGCTATCCTTCATC

In100 candidate1546 (in intron):

LOCUS ENSG00000008282

DESCRIPTION Synaptophysin-like protein 1 (Pantophysin).

[Source:Uniprot/SWISPROT;Acc:Q16563]

GTATTACCTGTAACCAAGATCATGATTTTATTCATCTCTGAGACTTTCTTTCATCTAGTCAGATTTATATCTGGGAAGAT

TAATTTCTTTTTTTTTTTTT

In100 candidate1547 (in intron):

LOCUS ENSG00000138653

DESCRIPTION Bifunctional heparan sulfate N-deacetylase/N-sulfotransferase 4(EC 2.8.2.8) (Glucosaminyl N-deacetylase/N-sulfotransferase 4) (NDST- 4) (Nheparan sulfate sulfotransferase 4) (N-HSST 4) [Includes: Heparan sulfate Ndeacetylase 4 (EC 3.-.-.-); Heparan sul [Source:Uniprot/SWISSPROT;Acc:Q9H3R]

GTTTTGTATTCCTATTAACTCTTTCAATTCCTTTTCTTTTTCTTCAAACTATTCTCAGCTTCTTCACCACCACTTTGTTT
TCAACTCACTTTAACATTTT

In100 candidate1548 (in intron):

LOCUS ENSG00000138653

DESCRIPTION Bifunctional heparan sulfate N-deacetylase/N-sulfotransferase 4(EC 2.8.2.8) (Glucosaminyl N-deacetylase/N-sulfotransferase 4) (NDST- 4) (Nheparan sulfate sulfotransferase 4) (N-HSST 4) [Includes: Heparan sulfate Ndeacetylase 4 (EC 3.-.-.-); Heparan sul [Source:Uniprot/SWISSPROT;Acc:Q9H3R]

CATGTAATTGTACTGAAGATTAAAATATTTAATAACTTCATTAATAATTTTCTTTTATTTTTTTAACATCTGAAGCAGAT
GTAGTTTACTTTCTCTTTTT

In100 candidate1549 (in intron):

LOCUS ENSG00000138653

DESCRIPTION Bifunctional heparan sulfate N-deacetylase/N-sulfotransferase 4(EC 2.8.2.8) (Glucosaminyl N-deacetylase/N-sulfotransferase 4) (NDST- 4) (Nheparan sulfate sulfotransferase 4) (N-HSST 4) [Includes: Heparan sulfate Ndeacetylase 4 (EC 3.-.-.-); Heparan sul [Source:Uniprot/SWISSPROT;Acc:Q9H3R]

AGCAAAAATATGCTAAAATAATATTTTGTCTCTTATTGCCATATAACCTTTTACTTTCTTTTTTTGTAACAGGAACAACT
GCACTTTATTTATTTCTTCT

In100 candidate1550 (in intron):

LOCUS ENSG00000164961

DESCRIPTION Uncharacterized protein KIAA0196.

[Source:Uniprot/SWISSPROT;AccQ12768]

TGAGGCTTGAAAAGATTACATCACTGCCCTATATTAACTCTTGTATAAATTCATTTACTCACTAATGTTTATCTCCAGGG

TTTTTCTTCCTTTAGGGCTC

In100 candidate1551 (in intron):

LOCUS ENSG00000113532

DESCRIPTION CMP-N-acetylneuraminate-poly-alpha-2,8-sialyltransferase (EC

2..99.-) (Alpha-2,8-sialyltransferase 8D) (ST8Sia IV) (Polysialyltransferase-1).

[Source:Uniprot/SWISSPROT;Acc:Q92187]

AGTCCCATGTACCGCTTTGTCTCATACATCCTGATAACAAGCCTGATTCATTCATCCTACTTTCTGGTTTTATCCAGCTC

TCTAACCATTTGTTTTACTA

In100 candidate1552 (in intron):

LOCUS ENSG00000164100

DESCRIPTION Bifunctional heparan sulfate N-deacetylase/N-sulfotransferase

3(EC 2.8.2.8) (Glucosaminyl N-deacetylase/N-sulfotransferase 3) (NDST- 3) (hDST-

3) (N-heparan sulfate sulfotransferase 3) (N-HSST 3) [Includes: Heparansulfate

N-deacetylase 3 (EC 3.-.-.-); H [Source:Uniprot/SWISSPROT;Acc:O9580]

CTTATTTATTTTTTCAAAAAACTAACTTCTGGTTTTGTTGATCTCTTACATGCTTTTTTATGTCTCAATTTTATTCAGTT

CATTTTTTCACATCTCAGTT

In100 candidate1553 (in intron):

LOCUS ENSG00000164100

DESCRIPTION Bifunctional heparan sulfate N-deacetylase/N-sulfotransferase 3(EC 2.8.2.8) (Glucosaminyl N-deacetylase/N-sulfotransferase 3) (NDST- 3) (hDST-3) (N-heparan sulfate sulfotransferase 3) (N-HSST 3) [Includes: Heparansulfate N-deacetylase 3 (EC 3.-.-.-); H [Source:Uniprot/SWISSPROT;Acc:O9580]

GAGTGCTAACCTTGGCTCCGCTTTGGCATTTTTGTGTCTCCCACTCCATGTTCCCATGCATTTAACAATTTCTTTCAGTT
TTTTCTTTCTTTTGTACCAG

In100 candidate1554 (in intron):

LOCUS ENSG00000164100

DESCRIPTION Bifunctional heparan sulfate N-deacetylase/N-sulfotransferase 3(EC 2.8.2.8) (Glucosaminyl N-deacetylase/N-sulfotransferase 3) (NDST- 3) (hDST-3) (N-heparan sulfate sulfotransferase 3) (N-HSST 3) [Includes: Heparansulfate N-deacetylase 3 (EC 3.-.-.-); H [Source:Uniprot/SWISSPROT;Acc:O9580]

AAACTCCCTTTAACTGTAAAGCCATTAATTTTTTCTTCTGATTTCTTCTTTTCTCTTTCCCACCTTTGTCTTGCCCAGTT
TTTACTTATTTTCATTCCTC

In100 candidate1555 (in intron):

LOCUS ENSG00000156831

DESCRIPTION E3 SUMO-protein ligase NSE2 (EC 6.-.-.-) (Non-structural mainteance of chromosomes element 2 homolog) (Non-SMC element 2 homolog) (hMMS21) [Source:Uniprot/SWISSPROT;Acc:Q96MF7]

AGAGGTAACTTTTCTCCTTCTATTCTGCCTTTCTGAATTCTTTTTCTGCATCACCTGTTCTTCAATCTTCCTGCAGCCCT
TTAACTCTTCTTATTTTTGT

In100 candidate1556 (in intron):

LOCUS ENSG00000164099

DESCRIPTION Neurotrypsin precursor (EC 3.4.21.-) (Serine protease 12)

(Motosin) (Leydin). [Source:Uniprot/SWISSPROT;Acc:P56730]

AGGATTCTAAATTTTAAAATGTTAACTCCATGAAAATATTTTGCTTAAGCCATCTTCTTTTCCTCAGCTTCCTCTATACC

TCCTATAATATTCTTTTATC

In100 candidate1557 (in intron):

LOCUS ENSG00000205359

DESCRIPTION solute carrier organic anion transporter family, member 6A1

[Sorce:RefSeq_peptide;Acc:NP_775759]

TGTTTTTTATAACGACTGCACCATTTTACATTCCTGCAGTGCACAGGGTTCCAATTTCTCCACATCCTCACCAATACTGT

TTTGTTTGTTTTTTCTGTTT

In100 candidate1558 (in intron):

LOCUS ENSG00000164597

DESCRIPTION Conserved oligomeric Golgi complex component 5 (13S Golgi

transort complex 90 kDa subunit) (GTC-90) (Golgi transport complex 1).

[Source:Uiprot/SWISSPROT;Acc:Q9UP83]

TATAACATATTGTCCTCTTCCTCATATGGTTGTTTTCATCTCCTAATCCTTTTGTGTCTCCCATCTCTAGGACTTCAGCA

GTACTGTTTTCTCTCCATTC

In100 candidate1559 (in intron):

LOCUS ENSG00000164597

DESCRIPTION Conserved oligomeric Golgi complex component 5 (13S Golgi transort complex 90 kDa subunit) (GTC-90) (Golgi transport complex 1). [Source:Uiprot/SWISSPROT;Acc:Q9UP83]

ATCTTTTTGCCTGTAGACTTGATAACACCCCGCACACTATTCATCTTCCACATTATTATCAGAGTGATTTTCTTTTTCTT
TTTTTTTGAGATGGAGTCTT

In100 candidate1560 (in intron):

LOCUS ENSG00000164597

DESCRIPTION Conserved oligomeric Golgi complex component 5 (13S Golgi transort complex 90 kDa subunit) (GTC-90) (Golgi transport complex 1). [Source:Uiprot/SWISSPROT;Acc:Q9UP83]

TGGAAATGCCCTTGTCCGCTCATTCTAACATCTATGTTAATTCCGGGTTTTTGTTTGGTTGAATTTTCTTTCCATCAGAC
CTGATATTTTTCTACTTATT

In100 candidate1561 (in intron):

LOCUS ENSG00000127527

DESCRIPTION Epidermal growth factor receptor substrate 15-like 1 (Eps15-relted protein) (Eps15R). [Source:Uniprot/SWISSPROT;Acc:Q9UBC2]

TAATTCCTCCTTTTCATTTGCAAGTAACTCTCCCCTCTGCCTTTTTTTTTTTTTACTGTTAGAAGCTTGCAGTGTATTTT
TCTGCTCAGCCATGATTTTT

In100 candidate1562 (in intron):

LOCUS ENSG00000145730

DESCRIPTION Peptidyl-glycine alpha-amidating monooxygenase precursor (PAM)

Includes: Peptidylglycine alpha-hydroxylating monooxygenase (EC 1.14.17.3) PHM);

Peptidyl-alpha-hydroxyglycine alpha-amidating lyase (EC 4.3.2.5)

(Pepidylamidoglycolate lyase) (PAL)]. [Source:Uniprot/SWISSPROT;Acc:P19021]

GCGGAGTATTGAAATGTTAACAAGACTGAAATGATTTTCCACCCCTTCTTTTTTTTAAATACCAGTCTGCCACAACATTT

TTCCTCTTAGGCTTTTATGT

In100 candidate1563 (in intron):

LOCUS ENSG00000172403

DESCRIPTION Synaptopodin-2 (Myopodin) (Genethonin 2).

[Source:Uniprot/SWISSROT;Acc:Q9UMS6]

AGTTAGAATTTTAACATAAATTTGATTCTGACTTTTTGTCAGTTGTTCTAGTTATCATATTTTAGTACTTTTTATCATCT

CCTTCATTGCTTCCTTCCCA

In100 candidate1564 (in intron):

LOCUS ENSG00000172403

DESCRIPTION Synaptopodin-2 (Myopodin) (Genethonin 2).

[Source:Uniprot/SWISSROT;Acc:Q9UMS6]

TGTTTTTAAATTTGGCTTTTACTCCCTAACTTTGGCATTTGTATTAACTTTGCCATTTTCCTTTATACAGGATACATGAT

TTTCTCCCCACTTCTAATTT

In100 candidate1565 (in intron):

LOCUS ENSG00000153310

DESCRIPTION Protein FAM49B (L1). [Source:Uniprot/SWISSPROT;Acc:Q9NUQ9]

TGGGACCTACTCTAACTTATTTTTTCATTTATCTCTTGCATAGTTTCAGTTGGTTTTGCCCTGTACTTTGAAATATCTTT

ATTCTGGTGTCATCTTTCTT

In100 candidate1566 (in intron):

LOCUS ENSG00000178636

DESCRIPTION CDNA FLJ40807 fis, clone TRACH2009268.

[Source:Uniprot/SPTREMBLAcc:Q8N7N2]

CAATAAGAGCACACCATTAACTCTTCCCTCCGTTTCTCTAATGTCATTTTCTCCTCATTCTGCAGCTACTTTTCTGGTGC

ACTTTAAATACTGTTTTTCC

In100 candidate1567 (in intron):

LOCUS ENSG00000132294

DESCRIPTION Protein EFR3-like. [Source:Uniprot/SWISSPROT;Acc:Q14156]

AGTATTTGAAAAATTATTCTTTGCATTTTCTAACACAATCCTTTTGTAATTTTCTTTCTGTTTATTCAGAGGTCTTCTCT

TTCTGCTCTTATTGTGCTGA

In100 candidate1568 (in intron):

LOCUS ENSG00000185238

DESCRIPTION Protein arginine N-methyltransferase 3 (EC 2.1.1.-) (Heterogeneus

nuclear ribonucleoprotein methyltransferase-like protein 3).

[Source:Unirot/SWISSPROT;Acc:O60678]

TTAACTCATGTAACACTTACCTTCTTGGTTCTCTTACTTTTGTGGCTCCAGCTTCTCGGTCAAGTCCCTTGAATTCTAGT

ATTCATTATCTATTCTCCCT

In100 candidate1569 (in intron):

LOCUS ENSG00000185238

DESCRIPTION Protein arginine N-methyltransferase 3 (EC 2.1.1.-) (Heterogeneus nuclear ribonucleoprotein methyltransferase-like protein 3). [Source:Unirot/SWISSPROT;Acc:O60678]

AGTGCTTACAACTGAAGTTAACTATATCTGTCATTTTTCTTTGTCTTAAAACTTATTTTTTGGTTACCCAGAACTTCCCA
GTATTTCTTTCACTATATTA

In100 candidate1570 (in intron):

LOCUS ENSG00000184156

DESCRIPTION Potassium voltage-gated channel subfamily KQT member 3 (Voltagegated potassium channel subunit Kv7.3) (Potassium channel subunit alpha KvLT3) (KQT-like 3). [Source:Uniprot/SWISSPROT;Acc:O43525]

ATGAAATCACATTCGTCCTTGAACATGATTAACTCATTTCCACCTTCTGATTTTTGCACCAGCTGCTCCCTCTGCCTGGA
ATATGCTTTCTGTATATCTT

In100 candidate1571 (in intron):

LOCUS ENSG00000184156

DESCRIPTION Potassium voltage-gated channel subfamily KQT member 3 (Voltagegated potassium channel subunit Kv7.3) (Potassium channel subunit alpha KvLT3) (KQT-like 3). [Source:Uniprot/SWISSPROT;Acc:O43525]

GGCACCTGGTAAGTGATACGTAAGTGTTTGTTACTGCTAACTTCATTTCTCCCTTTTTGTTCCGATCAGGCTGAATGAAA

TATTCTGTCTTTTTTTTTTT

In100 candidate1572 (in intron):

LOCUS ENSG00000184156

DESCRIPTION Potassium voltage-gated channel subfamily KQT member 3

(Voltagegated potassium channel subunit Kv7.3) (Potassium channel subunit alpha

KvLT3) (KQT-like 3). [Source:Uniprot/SWISSPROT;Acc:O43525]

AACTTTTAAGTAACAATCTCCCTGCCACCCACTTCCTCTTCCATTCTTTCATTGGTGATGTTCTTTCTCTATTTGCAGCC

CTACCTTGTCGGTTTTTTTC

In100 candidate1573 (in intron):

LOCUS ENSG00000145725

DESCRIPTION Histidine acid phosphatase domain containing 1

[Source:RefSeq_pptide;Acc:NP_056031]

AGTGCAATCACATTTAACAAATTGAAAATTCTTACTATGGCTTCTCAGTCCTTTGTGTGCATCTTAGCTACTTATCTGAC

ATGATTTTTTACCATTTTTC

In100 candidate1574 (in intron):

LOCUS ENSG00000145725

DESCRIPTION Histidine acid phosphatase domain containing 1

[Source:RefSeq_pptide;Acc:NP_056031]

ACCTTTCTAACCTCCTCATATATTTGTTTTTCCCCTCTTTCTTTTAAGTGTTCTTTTTGTAGCTGTTACGAATTTCAGGT

ACCTGTTTTTCTCTTTCTTA

In100 candidate1575 (in intron):

LOCUS ENSG00000165970

DESCRIPTION Sodium- and chloride-dependent glycine transporter 2 (GlyT2) (GyT-2) (Solute carrier family 6 member 5). [Source:Uniprot/SWISSPROT;Acc:Q9Y45]

AACGGGGGTTTAATAGTGTTGAGTGCTTGAATAATTCACGCCACCACCCTAACACGTGTGTCACTTTTCTCTTTCCAGTT
TATCCTTTGCTTCAGCTTTT

In100 candidate1576 (in intron):

LOCUS ENSG00000157884

DESCRIPTION calcium and integrin binding family member 4 [Source:RefSeq_pepide;Acc:NP_001025052]

GCTTATTAGATATAACTAATATGCTTATTCTTATTTATATCTAACCTTCTATACTGAATATCTTCTATATTCCAGGTGAT
GTCCATGTATTTTCTTATTT

In100 candidate1577 (in intron):

LOCUS ENSG00000138738

DESCRIPTION PR domain zinc finger protein 5 (PR domain-containing protein 5. [Source:Uniprot/SWISSPROT;Acc:Q9NQX1]

CATAACCTCCTATATTATTTTCTACTTTTCTTCTTACGTTTATTTACAGGACATCATATTGAGAATTTTCATGTTCAATA
CCTTTCCCTTTTATTTCTTC

In100 candidate1578 (in intron):

LOCUS ENSG00000165973

DESCRIPTION Protein kinase C-binding protein NELL1 precursor (NEL-like protin 1) (Nel-related protein 1). [Source:Uniprot/SWISSPROT;Acc:Q92832]

AGATTTAACTTTTTTAAAAGGGCTTCAACCTGCTTTTTCAGTTCCTTCTCTGAACTGACATGTTTTCTGGTTCTGAGTTT

TCTCCTCTCCTTTACTTCTT

In100 candidate1579 (in intron):

LOCUS ENSG00000165973

DESCRIPTION Protein kinase C-binding protein NELL1 precursor (NEL-like protin 1) (Nel-related protein 1). [Source:Uniprot/SWISSPROT;Acc:Q92832]

AGTCTCTAACTCTTGGGTTTATTCTTTACCCATTAGTTTTCTTCAAAACTTTGTCTCACTACTATGCAGTTTTTTTCCTT

CTTCGAATTCTCTCACCTTT

In100 candidate1580 (in intron):

LOCUS ENSG00000165973

DESCRIPTION Protein kinase C-binding protein NELL1 precursor (NEL-like protin 1) (Nel-related protein 1). [Source:Uniprot/SWISSPROT;Acc:Q92832]

AATTAAATGTAAATACCTACATTTAATTGCCTGCTTTTTCTCTAGATGATAACATTTTTAATAAATATTCTTCACCAGTA

TCTTCTTTATTTTCTTTTTA

In100 candidate1581 (in intron):

LOCUS ENSG00000165973

DESCRIPTION Protein kinase C-binding protein NELL1 precursor (NEL-like protin 1) (Nel-related protein 1). [Source:Uniprot/SWISSPROT;Acc:Q92832]

TTGAATTTTTATGATATTAGAGATGGGAAATGTTCACACTTAATTTTGTTAACATTTCTTTTCTCTCTATTATCGCAGGC

TAAATTTTTCCACTTTCTTA

In100 candidate1582 (in intron):

LOCUS ENSG00000165973

DESCRIPTION Protein kinase C-binding protein NELL1 precursor (NEL-like protin 1) (Nel-related protein 1). [Source:Uniprot/SWISSPROT;Acc:Q92832]

TCCTTATGCAATCAATTTGACTTTAACTATTTCCTCTCTCTCTCTATTTTTCTCACTTTCCTATTACTTGTGTCAGTA

CCTGAGTTTTTTCTTATCCT

In100 candidate1583 (in intron):

LOCUS ENSG00000165973

DESCRIPTION Protein kinase C-binding protein NELL1 precursor (NEL-like protin 1) (Nel-related protein 1). [Source:Uniprot/SWISSPROT;Acc:Q92832]

ATAATATTAACTGTCCTATTCCATTTTTTTGTGTCCTCTTCAATTTATTTCATCAACATTTTGTAGTTTTTATTGCAGAA

GTCTTTTACTTCCTTGGTTA

In100 candidate1584 (in intron):

LOCUS ENSG00000091129

DESCRIPTION Neuronal cell adhesion molecule precursor (Nr-CAM) (NgCAM-relatd cell adhesion molecule) (Ng-CAM-related) (hBravo). [Source:Uniprot/SWISSPOT;Acc:Q92823]

AATAGAATTAACCCAAAACAATTTACATTGTCCTGTGTTACTTTTTTTCTTTTTCAGGAATCTTTGTGACATTCTCTCTC

AGCATTCTTTATCCCCTGTT

In100 candidate1585 (in intron):

LOCUS ENSG00000091129

DESCRIPTION Neuronal cell adhesion molecule precursor (Nr-CAM) (NgCAM-relatd cell adhesion molecule) (Ng-CAM-related) (hBravo). [Source:Uniprot/SWISSPOT;Acc:Q92823]

CTATTAATCTTTTTACCTGTGTCTTATTAACTTGTTTACTTGTCCAATACATTTTTTCATTTAAATCATTTTTTTCAGGA

AGTTCTATTTGGTTCTTTTT

In100 candidate1586 (in intron):

LOCUS ENSG00000091129

DESCRIPTION Neuronal cell adhesion molecule precursor (Nr-CAM) (NgCAM-relatd cell adhesion molecule) (Ng-CAM-related) (hBravo). [Source:Uniprot/SWISSPOT;Acc:Q92823]

GATACTTTCTCCTATTCCATAGGTTGCCTTTAACTCTATTGATAATTTCCTTTCCTGTACAGATTTTTTGTTGTTGTTGT

TGTTTGATATAGTCCCACTT

In100 candidate1587 (in intron):

LOCUS ENSG00000091129

DESCRIPTION Neuronal cell adhesion molecule precursor (Nr-CAM) (NgCAM-relatd cell adhesion molecule) (Ng-CAM-related) (hBravo). [Source:Uniprot/SWISSPOT;Acc:Q92823]

GGGATAACAAGTATTCTCAATTTCACATCTGTTCTTTCAGACCTTTTCTTAATCTTTCCATCATATTTGCACATTTAGAG

ATCCATGGTCCCTTTGCTCT

In100 candidate1588 (in intron):

LOCUS ENSG00000184349

DESCRIPTION Ephrin-A5 precursor (EPH-related receptor tyrosine kinase ligan 7) (LERK-7) (AL-1). [Source:Uniprot/SWISSPROT;Acc:P52803]

AACTAATAGGAGTTTTTTGGTAGTAAGAAAAGTTTTAACTTATTTTAAAATCCTTTCATTTCAGGTATTTTTAAAGACCA

CCTTTCCATTCTATTTCTCC

In100 candidate1589 (in intron):

LOCUS ENSG00000102837

DESCRIPTION olfactomedin 4 precursor [Source:RefSeq_peptide;Acc:NP_006409]

EXON

GTGATTATATTGAGATATTTACCATTTACTGAGTAACATTTACATGTCTTCTCTGTATTTTTCGTAACTATTTTGCAGAG

TTGGTATTATTTTTCTTCTG

In100 candidate1590 (in intron):

LOCUS ENSG00000145743

DESCRIPTION F-box/LRR-repeat protein 17 (F-box and leucine-rich repeat protin 17) (F-box only protein 13) (Fragment). [Source:Uniprot/SWISSPROT;Acc:Q9F56]

AAGAGCTCTTTCTTCTTCAACTAACAAGACACAAAACAATGTTAATGTTTTTTTCTCTTTTCCTGTCATCAGAGCAAATC

TATCTTTTTTTTAGATTCTC

In100 candidate1591 (in intron):

LOCUS ENSG00000145743

DESCRIPTION F-box/LRR-repeat protein 17 (F-box and leucine-rich repeat protin 17) (F-box only protein 13) (Fragment). [Source:Uniprot/SWISSPROT;Acc:Q9F56]

CATGTGCTAATTTCATGTCTCTGTGTCATATTTTGGTAACTCTCACAACTTTTTTTTTTTCTAAATGTTACCTTCAGCTTTTTTTGTTGTTGAACTTTT

In100 candidate1592 (in intron):

LOCUS ENSG00000145743

DESCRIPTION F-box/LRR-repeat protein 17 (F-box and leucine-rich repeat protin 17) (F-box only protein 13) (Fragment). [Source:Uniprot/SWISSPROT;Acc:Q9F56]

GACATTTTTTATGGATTGTGGTATATATATTTTTAACCAACATAGTTTCTTTGTACCTGATTTCCTCTTATATCAGATCTCTTCAATTCCTGATTCTCAT

In100 candidate1593 (in intron):

LOCUS ENSG00000145743

DESCRIPTION F-box/LRR-repeat protein 17 (F-box and leucine-rich repeat protin 17) (F-box only protein 13) (Fragment). [Source:Uniprot/SWISSPROT;Acc:Q9F56]

TATATACCCATGTAGATTTGTTAATTAACTAATACTTTGATTTGTTCATTTGAAACTTGTCATTACCTGCTCTTCCAGTCTTTTTGTATATTTCCCCCTT

In100 candidate1594 (in intron):

LOCUS ENSG00000157851

DESCRIPTION Dihydropyrimidinase-related protein 5 (DRP-5) (ULIP6 protein) (ollapsin response mediator protein 5) (CRMP-5) (CRMP3-associated molecule) CRAM). [Source:Uniprot/SWISSPROT;Acc:Q9BPU6]

ACATTATTTTAATAACTGGCTTCATATTTTTTTCTTTTACCAACTGCTATACTAACCAGGATTTTTTTTCTTCATATAACACTATACCCTGAGTGTCTCC

In100 candidate1595 (in intron):

LOCUS ENSG00000157851

DESCRIPTION Dihydropyrimidinase-related protein 5 (DRP-5) (ULIP6 protein) (ollapsin response mediator protein 5) (CRMP-5) (CRMP3-associated molecule) CRAM). [Source:Uniprot/SWISSPROT;Acc:Q9BPU6]

AGGAATTGTATTGTATATATTTTATTAACTTTAAGTAAAATATTTTCTCTCGACTTCTGATGCCCAGTTTTTTCTCTTAT
CTTTCTGTCCTTTGGGTCCT

In100 candidate1596 (in intron):

LOCUS ENSG00000151422

DESCRIPTION Proto-oncogene tyrosine-protein kinase FER (EC 2.7.10.2) (p94-FR) (c- FER) (Tyrosine kinase 3). [Source:Uniprot/SWISSPROT;Acc:P16591]

AATTACTACTTTAGGCTGTAATAATATTTTAACAAGCATAATCACTGGCTTCTTCTGATGGCCTTTCAGACTTTTTCGGT
TTTTGAGACAGTCTTGCTCT

In100 candidate1597 (in intron):

LOCUS ENSG00000151422

DESCRIPTION Proto-oncogene tyrosine-protein kinase FER (EC 2.7.10.2) (p94-FR) (c- FER) (Tyrosine kinase 3). [Source:Uniprot/SWISSPROT;Acc:P16591]

TTCCCCTAACCTCCCATGGTTATCCTTCTCTCACAAGGCATTGTTTTCTCCTTTTTTGCATGTTTATGTTTTTAGCAGGG
CTTTATCTTGCTTTTATTTT

In100 candidate1598 (in intron):

LOCUS ENSG00000198961

DESCRIPTION E3 ubiquitin-protein ligase Praja2 (EC 6.3.2.-) (Praja-2)

(RINGfinger protein 131). [Source:Uniprot/SWISSPROT;Acc:O43164]

CATAAAGTTGTTTCTTAATTATTTTAATGCCTATAACGTCTGTAGTGGTGTCTTTCCTTTCATTCTTGTTATTAGCAGTG

TGACCTCTCATTTTTATTTG

In100 candidate1599 (in intron):

LOCUS ENSG00000134343

DESCRIPTION Transmembrane protein 16C. [Source:Uniprot/SWISSPROT;Acc:Q9BYT9

AAGCAACAAGTAAGTTTCCAAAAAAGATGATTGAATATAACAAATCTGAAGTTTATAGTTTTTATACTTTGTTTTCAGGA

CTTATTGCTATTTTAATTTT

In100 candidate1600 (in intron):

LOCUS ENSG00000134343

DESCRIPTION Transmembrane protein 16C. [Source:Uniprot/SWISSPROT;Acc:Q9BYT9

ATGCTTAACATAATTGGATGCTCGTAACTTTATGATTCTTTTCTCATCACTTTTGTGTGTCTCGGTTATTTGTTCCAGCT

GACCTGTGTTTATATCTCTC

In100 candidate1601 (in intron):

LOCUS ENSG00000134343

DESCRIPTION Transmembrane protein 16C. [Source:Uniprot/SWISSPROT;Acc:Q9BYT9

TTGCATGACTACAAAACAAGTGACATTAACTGTTTGACCACAATTTAGTTTTCACTTTCCAGTTGATGGACTATTTTATT

TGTTTTCAGCCTTCTGCTTT

In100 candidate1602 (in intron):

LOCUS ENSG00000112893

DESCRIPTION Alpha-mannosidase 2 (EC 3.2.1.114) (Alpha-mannosidase II)

(Mannsyl- oligosaccharide 1,3-1,6-alpha-mannosidase) (MAN II) (Golgi

alphamannsidase

II) (Mannosidase alpha class 2A member 1).

[Source:Uniprot/SWISSPROTAcc:Q16706]

AGAAGCACTGGTAACCTAATAATTTGAAGGTTTTTTTCTTTGCTTCCAGCATTCATTTATCTTCTACTTACATTTTTTCC

CCATTAGTTTATTGGCAGAT

In100 candidate1603 (in intron):

LOCUS ENSG00000112893

DESCRIPTION Alpha-mannosidase 2 (EC 3.2.1.114) (Alpha-mannosidase II)

(Mannsyl- oligosaccharide 1,3-1,6-alpha-mannosidase) (MAN II) (Golgi

alphamannsidase

II) (Mannosidase alpha class 2A member 1).

[Source:Uniprot/SWISSPROTAcc:Q16706]

TATTTTCATCTTTTAGTGTTTGATATACAAAATTTTAACATATTTATCTACTCAAATCTGTTATTCTGTTTTCTTCAGAT

ATTGCTTTTGCTGTCATGTT

In100 candidate1604 (in intron):

LOCUS ENSG00000112893

DESCRIPTION Alpha-mannosidase 2 (EC 3.2.1.114) (Alpha-mannosidase II)

(Mannsyl- oligosaccharide 1,3-1,6-alpha-mannosidase) (MAN II) (Golgi alpha

mannsidase II) (Mannosidase alpha class 2A member 1).

[Source:Uniprot/SWISSPROTAcc:Q16706]

AGATTTATTATTTTTGTCATTTAACATTTCTTATCTCCATTTCTCTTATATTTTCCTTCAGCTTGATCTTTGACTCTCCT

TTTGCTTGTTTTTTGTTAAC

In100 candidate1605 (in intron):

LOCUS ENSG00000139734

DESCRIPTION Protein diaphanous homolog 3 (Diaphanous-related formin-3) (DRF).

[Source:Uniprot/SWISSPROT;Acc:Q9NSV4]

TGTCCGTAGCTATCATCCCTTAACAAAATTATAAAGTTGTTACATTTCATTGTTGTTATCTCACACCTTTTGTCTCAGAA

TTTATCTTCCATTTTCTCTG

In100 candidate1606 (in intron):

LOCUS ENSG00000139734

DESCRIPTION Protein diaphanous homolog 3 (Diaphanous-related formin-3) (DRF).

[Source:Uniprot/SWISSPROT;Acc:Q9NSV4]

TCTAACTTGTGTATCTAGCTATTTTCTCCTTGTTACTTTTCTGCTCTACACATCGTTTACTCCAGTAACATTGGTCTCCT

TTTTCTCTAAATTTGGCTTT

In100 candidate1607 (in intron):

LOCUS ENSG00000139734

DESCRIPTION Protein diaphanous homolog 3 (Diaphanous-related formin-3) (DRF).

[Source:Uniprot/SWISSPROT;Acc:Q9NSV4]

CCCTCTTCTTTCTCAATGATTTTATAAAACACATATAATTTTTTTAATAACTCTTTTTGAGCAATTTTTACCTTCCAGAC

TTTCTATACTTTGGCTTTCA

In100 candidate1608 (in intron):

LOCUS ENSG00000139734

DESCRIPTION Protein diaphanous homolog 3 (Diaphanous-related formin-3) (DRF). [Source:Uniprot/SWISSPROT;Acc:Q9NSV4]

TTTGGCTAATATTTACATGCTTTTCTTGACTTTGTTGTTCTAACATATTGTCTTCTCTCTGGTCCTTGATTTCAGTGGTC

CTATTCTATTTGTAACCTTC

In100 candidate1609 (in intron):

LOCUS ENSG00000184903

DESCRIPTION Mitochondrial inner membrane protease subunit 2 (EC 3.4.99.-) (MP2- like protein). [Source:Uniprot/SWISSPROT;Acc:Q96T52]

CATTTCACTAAATAATAACTAATAAAAGTTGTGCCTTGTCACTGTTTTGGTGCCTGTTTATACTATTCCTTCTCACAGTG

ATGTTTTATTCCATTTTATT

In100 candidate1610 (in intron):

LOCUS ENSG00000184903

DESCRIPTION Mitochondrial inner membrane protease subunit 2 (EC 3.4.99.-) (MP2- like protein). [Source:Uniprot/SWISSPROT;Acc:Q96T52]

AACTAAGAATTTTACTTTGTTGGCTCTTAACATAATGAAATATATTCTGGTTTTCAGGCCCTTTTTTCGACTTTTGTTTC

CTGCATGTTCTTTCTTTGTC

In100 candidate1611 (in intron):

LOCUS ENSG00000184903

DESCRIPTION Mitochondrial inner membrane protease subunit 2 (EC 3.4.99.-)

(MP2- like protein). [Source:Uniprot/SWISSPROT;Acc:Q96T52]

CCCTCCCTCCCCTTCTCCCTCCCCCTCTCCTACTTTCCTTCCCTTTTTATAACTATTGTCATACTATGCCTTTACCAGTG

TTCTGTTTTTAATCCTCTAA

In100 candidate1612 (in intron):

LOCUS ENSG00000184903

DESCRIPTION Mitochondrial inner membrane protease subunit 2 (EC 3.4.99.-)

(MP2- like protein). [Source:Uniprot/SWISSPROT;Acc:Q96T52]

TATATGACTTATTTTTAACCCATGAATTTAAATTTCCTTATTAAATGTTTCATTTGCTTTTGCCTTTCATTTTTCAGGAA

TCATTTCTCCTAGTCTGTCT

In100 candidate1613 (in intron):

LOCUS ENSG00000184903

DESCRIPTION Mitochondrial inner membrane protease subunit 2 (EC 3.4.99.-)

(MP2- like protein). [Source:Uniprot/SWISSPROT;Acc:Q96T52]

TCTCTTAAAATAATTAATTAGATTTAAAATAAGACTGCATATGTAACCACCAAACTATATCTTTTTTAACAGTTTATTTG

GCGTTAAGATTTTCTCCCTT

In100 candidate1614 (in intron):

LOCUS ENSG00000184903

DESCRIPTION Mitochondrial inner membrane protease subunit 2 (EC 3.4.99.-)

(MP2- like protein). [Source:Uniprot/SWISSPROT;Acc:Q96T52]

TTTCCAATAGCATACTAACGTGCTGTTTGTTATCCTATATATTTTTTAAAATCTCTGTCTCTCTGCTTCTGCCTCCAGTG

ACTTCCTATTTCTCTCCTTT

In100 candidate1615 (in intron):

LOCUS ENSG00000083544

DESCRIPTION Tudor domain-containing protein 3.

[Source:Uniprot/SWISSPROT;Ac:Q9H7E2]

TTTCTTAGTTTCCTTATTGGTACTAACAAAGTTCTATCTTTGGTCCACTTTCTTGTTCTTCTGACTTTAACTGATCAGTC

TCTCTCTCTCTTTTTTTTTT

In100 candidate1616 (in intron):

LOCUS ENSG00000128512

DESCRIPTION Dedicator of cytokinesis protein 4.

[Source:Uniprot/SWISSPROT;Ac:Q8N1I0]

AAGGCAAATAAAAGCTCATCGCTTTCTAAAACACTAAAATACTCTCAATTAACTTTTGCTGTTTTTTTATGTTTCCAGAA

TCTCTTGATATTGTTTTATT

In100 candidate1617 (in intron):

LOCUS ENSG00000128512

DESCRIPTION Dedicator of cytokinesis protein 4.

[Source:Uniprot/SWISSPROT;Ac:Q8N1I0]

AGAGCTTTGAGATGATAACTAATTTTCACATATTTCTTTTTCTTTCGCAGTTCTCTGAGTCTTACCTATTACAATTCTCT

TTTGGTTCTCTTTTTCAGTA

In100 candidate1618 (in intron):

LOCUS ENSG00000128512

DESCRIPTION Dedicator of cytokinesis protein 4.

[Source:Uniprot/SWISSPROT;Ac:Q8N1I0]

CCAATATGCAATTTTTATTTTACAAAAGTATTTCACATTTCTTAACAATAAATCAAAATCATTTCCTTATTTCACCAGTT

TCTGTATCAGCCCTTTGTAT

In100 candidate1619 (in intron):

LOCUS ENSG00000066827

DESCRIPTION Zinc finger protein 406 (ZFAT zinc finger 1).

[Source:Uniprot/SISSPROT;Acc:Q9P243]

CTTTACCCTAACTTTTATGTTTTCTTTGCTTTTATCTTGTGCTGCATTCTGCGTATTTACTTCTGATCCATTTTCCAGTT

CACTATTTTTTACCTGTTAT

In100 candidate1620 (in intron):

LOCUS ENSG00000066827

DESCRIPTION Zinc finger protein 406 (ZFAT zinc finger 1).

[Source:Uniprot/SISSPROT;Acc:Q9P243]

TCCATCCCATTTTTCTGTTACCCTCTAAGCTCGAATAACTGGTGAGTTAATGGAATTTTCCTTTTCTTGTTCCCACAGAG

GCACTTTTTTGACTTCTACT

In100 candidate1621 (in intron):

LOCUS ENSG00000138688

DESCRIPTION CDNA: FLJ21404 fis, clone COL03835.

[Source:Uniprot/SPTREMBL;Ac:Q9H742]

CTGTGAAATGAAGGTTTTCCTTAAGGTTTGTTTCTGTTTAACAAACTCAAACATCTTTGACTTTTCTCTGTAAATCAGAT

CCTTTTAATGGTATGTTCCT

In100 candidate1622 (in intron):

LOCUS ENSG00000198839

DESCRIPTION Zinc finger protein 277. [Source:Uniprot/SWISSPROT;Acc:Q9NRM2]

EXON

AGTATCCATAAAATGTTTTTGATAGCCATAGCTAATAACATCCATCTTGGTTTTACTTTCGTCTGTTAATCCAGGTTATT

TCCATTGGATTTGTCCCTTT

In100 candidate1623 (in intron):

LOCUS ENSG00000134986

DESCRIPTION Neuronal protein 3.1 (Protein p311).

[Source:Uniprot/SWISSPROT;cc:Q16612]

GTTGTATACTTGATAGAAGAATATTGACCACTTAGTAACTGAGCAACACCTTTCCCTTTTTGGTAGCAGTTGAGGCTGTG

ATGCCTTTTTTTTCCATTTC

In100 candidate1624 (in intron):

LOCUS ENSG00000164603

DESCRIPTION CDNA FLJ31818 fis, clone NT2RP6000017.

[Source:Uniprot/SPTREMBLAcc:Q96MV7]

AGTCTCTGTACTTCCTCTTTAAGGCCAATAACTCTTAGATTTGCCCTTTTGAGGCTATTTTCCAGACTTGTAAGCATTCT

TCTTTCTTTTTCACTCCTTT

In100 candidate1625 (in intron):

LOCUS ENSG00000184226

DESCRIPTION Protocadherin-9 precursor. [Source:Uniprot/SWISSPROT;Acc:Q9HC56

AAGATTCCCCAAACAAAGTTTTCTTCTCAATTTAACTTTGAAAACTGCTTTTGCTTTGATAGTCTTCTGTCCAATCAGCT

TTTTTAAAATTCCTCTTGAT

In100 candidate1626 (in intron):

LOCUS ENSG00000184226

DESCRIPTION Protocadherin-9 precursor. [Source:Uniprot/SWISSPROT;Acc:Q9HC56

AGTAACTAGCTATTTGTTCTCTCCAAGGTTTTCTTTCTTATATAGAGATCATTTTTTCCCCTTCCTTAATCTACAGTGGG

ATTTCTTTCTCTACTTCATT

In100 candidate1627 (in intron):

LOCUS ENSG00000129595

DESCRIPTION Band 4.1-like protein 4A (Protein NBL4).

[Source:Uniprot/SWISSPOT;Acc:Q9HCS5]

AGACTTGGTCTCCTGCTAATAACCTTTCTCCTCTTCCCTGTCATTTCTTCAATGTTCAGGCTGGCCTTTGTGTTCCTTAC

CTCTTGTTTCATCAAAATCT

In100 candidate1628 (in intron):

LOCUS ENSG00000154415

DESCRIPTION Protein phosphatase 1 regulatory subunit 3A (Protein phosphatas 1 glycogen-associated regulatory subunit) (Protein phosphatase type-1 glycgen targeting subunit). [Source:Uniprot/SWISSPROT;Acc:Q16821]

AGTCTTGCTTTTAAAATTGGCCTGTGGCAATACATATATAAAGTCTTTTAACTGAGTATAAATTTAGCTGCCTTTCAGTT

TTTTAGTTAGCTTGTTTCTT

In100 candidate1629 (in intron):

LOCUS ENSG00000076382

DESCRIPTION Sperm-associated antigen 5 (Astrin) (Mitotic

spindleassociatedprotein

p126) (MAP126) (Deepest).

[Source:Uniprot/SWISSPROT;Acc:Q96R06]

GTTCTCTTCTGGAATTTCTTTTATTTAACTGTTAGGTATCCTGGGTTTATCTTCTAATTCTCTTAGCTTTTTCTTCAGAT

TTACATTTCTTTATATTTTT

In100 candidate1630 (in intron):

LOCUS ENSG00000128573

DESCRIPTION Forkhead box protein P2 (CAG repeat protein 44) (Trinucleotide

epeat- containing gene 10 protein). [Source:Uniprot/SWISSPROT;Acc:O15409]

AACTCTTAATAGTGGTTATGTCTGGGTAACCAAGCTAGGGGTAACTTTTTCTTGTTCTACATTTTTTAGTTTCTACAGTT

ATCATGCATTCTTTATTTTG

In100 candidate1631 (in intron):

LOCUS ENSG00000128573

DESCRIPTION Forkhead box protein P2 (CAG repeat protein 44) (Trinucleotide

epeat- containing gene 10 protein). [Source:Uniprot/SWISSPROT;Acc:O15409]

CTTGTTTTGAACTAACATGATTCATGTTATTCTTATCTCTTATTTGGACAGTTGCACGTACGTTTTTACAAGTCTTTATA

CCTCTGTCTTGTCTCACTCT

In100 candidate1632 (in intron):

LOCUS ENSG00000102974

DESCRIPTION Transcriptional repressor CTCF (CCCTC-binding factor) (CTCFL

paalog) (11-zinc finger protein). [Source:Uniprot/SWISSPROT;Acc:P49711]

TGTCTCAAAAAAAAAACAAAGTAACATTTTGTTTCTTTTGTTGTTTAAACTTCTACATGTGTATCTTTGTTTTTTCAGCC

TCTTTTATTCTTCATAGCTA

In100 candidate1633 (in intron):

LOCUS ENSG00000102974

DESCRIPTION Transcriptional repressor CTCF (CCCTC-binding factor) (CTCFL

paalog) (11-zinc finger protein). [Source:Uniprot/SWISSPROT;Acc:P49711]

GTGATAAGCATAGCTGCTTTCAAATCCTAACAACTTGTACATTTATATTCCTACTCCAGATCTTTCTTTTGAGTTAGCCT

TATATAACTGTGTATCCCTT

In100 candidate1634 (in intron):

LOCUS ENSG00000205160

DESCRIPTION

GTTCTCTTCTGGAATTTCTTTTATTTAACTGTTAGGTATCCTGGGTTTATCTTCTAATTCTCTTAGCTTTTTCTTCAGAT

TTACATTTCTTTATATTTTT

In100 candidate1635 (in intron):

LOCUS ENSG00000150361

DESCRIPTION Kelch-like protein 1. [Source:Uniprot/SWISSPROT;Acc:Q9NR64]

GGTAACGTCTCTTGTGTCATTTCTGATTGTGTTTATTTGGATCTTCTCTCTTTTCTTCTTTATTAGTCTAGCTAGCAGCC

TATCCTGTTTTTTGTTGTTG

In100 candidate1636 (in intron):

LOCUS ENSG00000150361

DESCRIPTION Kelch-like protein 1. [Source:Uniprot/SWISSPROT;Acc:Q9NR64]

GTGAATCAACTAAAGCACCAATTTCCTAAATGTTTTTAAATAATAACCATCTTTGAAAACATTATTTTACTTCAGTTTTG

ACTATTTGTTAATATCTTCC

In100 candidate1637 (in intron):

LOCUS ENSG00000150361

DESCRIPTION Kelch-like protein 1. [Source:Uniprot/SWISSPROT;Acc:Q9NR64]

CAACCTCATCCTTGGCTGCATTTTAAATCTCTTTAATTAACTGTTTTACTATCTTCTTTAGTGCCTATTTTTAATCAGAT

ATATCCATCTCTATTTTATA

In100 candidate1638 (in intron):

LOCUS ENSG00000169519

DESCRIPTION methyltransferase 5 domain containing 1

[Source:RefSeq_peptide;cc:NP_689849]

CCTCTTCAACCTCCCCTTCTGCCAACATGCGTTACTTCCTACTTGATAACCATATGAGGCACATAATTTTCTTTTCAGGT

CTCTGACTTTGCACTTGCTT

In100 candidate1639 (in intron):

LOCUS ENSG00000169519

DESCRIPTION methyltransferase 5 domain containing 1

[Source:RefSeq_peptide;cc:NP_689849]

CATGAATTTGATAGGAGAACTCCATATTGGTAACTCCTTCGGCATGTTATAACCTTTTCCAGCTCCATCTTTTTCTCCTG

AAACCTATCAAGAGTCTTTT

In100 candidate1640 (in intron):

LOCUS ENSG00000135272

DESCRIPTION MyoD family inhibitor domain containing isoform p40

[Source:Refeq_peptide;Acc:NP_951038]

TTAGGGTTTGCTAAGTAGAAACTATGAGAAGAGTTATTTTAACATGAATTTAATTTCGTTTGTGGTTTTTACTTACAGTC

TACTTCTGTTTTCAGATTCA

In100 candidate1641 (in intron):

LOCUS ENSG00000135272

DESCRIPTION MyoD family inhibitor domain containing isoform p40

[Source:Refeq_peptide;Acc:NP_951038]

AGACACTAAAACTTTTTTATTTCATTCTGCTATCAAATCTTATTAACACCTCTTCTTCTTTTTGCTAATTATCAGATTTT

TCTATCTGTCACTTAACTTC

In100 candidate1642 (in intron):

LOCUS ENSG00000165659

DESCRIPTION Dachshund homolog 1 (Dach1). [Source:Uniprot/SWISSPROT;Acc:Q9UI6]

AGAAAGTTTTAGCGATACCCATAAATGTTAACATTTTATAAAATTTTAGAATTTTTTTTATTTCAGCATAAATATTTTAT

TCTTTTTTATTTATTTTATT

In100 candidate1643 (in intron):

LOCUS ENSG00000169398

DESCRIPTION Focal adhesion kinase 1 (EC 2.7.10.2) (FADK 1) (pp125FAK)

(Protin- tyrosine kinase 2). [Source:Uniprot/SWISSPROT;Acc:Q05397]

TCTGTGTCCATAGGAAACTAACTCTGAATGATATCGTTTTCCTTTACTCCAGCTGCTTGAGCTTACTGGTTTGTTGTGTT

GTTTGTTGCTCTCACTTCTT

In100 candidate1644 (in intron):

LOCUS ENSG00000169398

DESCRIPTION Focal adhesion kinase 1 (EC 2.7.10.2) (FADK 1) (pp125FAK)

(Protin- tyrosine kinase 2). [Source:Uniprot/SWISSPROT;Acc:Q05397]

AGTCATTCTTATGTATTCTTTCAAATAACCAATTTCTGGTTTCATTTATCTTTTGCATGGTTTTTCATGTCTCAGTTCCA

TTTAGTTCAGCTCTGATTTT

In100 candidate1645 (in intron):

LOCUS ENSG00000145375

DESCRIPTION spermatogenesis associated factor SPAF

[Source:RefSeq_peptide;Ac:NP_660208]

AGTTTTGTTTTTGTTTTTGGCTGAACCTTTTAACTCCCCATCTGCGTCATGCTTTTCTTTTTTTCTTGACCACAGCTTCT

AGGATCTTAGTCTGCTTCCT

In100 candidate1646 (in intron):

LOCUS ENSG00000145375

DESCRIPTION spermatogenesis associated factor SPAF

[Source:RefSeq_peptide;Ac:NP_660208]

AGACTTCCTTTAACCAACCTGTAGTTTTGTTTTCTTTCTTAATAGCCTCAGGTCTCAGACACAACCATCTTTTATTTATT

TTTTCTTTCTTTCTGGAATC

In100 candidate1647 (in intron):

LOCUS ENSG00000145375

DESCRIPTION spermatogenesis associated factor SPAF

[Source:RefSeq_peptide;Ac:NP_660208]

AGAATTACTCAAGCCAATGATAATGCCGATTTGAGCATTTTAACATTCCACCTTATTTCTCTTCTTCCAAATTCTCAGTC

TCTCTATTTCCTCATTCTTT

In100 candidate1648 (in intron):

LOCUS ENSG00000170959

DESCRIPTION doublecortin domain containing 5

[Source:RefSeq_peptide;Acc:NP_40864]

TTGCTTAACGGCTTCTGTTTATATTTTTTCCTGCCTTCTCTTTATATTTATTTTGGCTTCAGGGTCACTGTACCATCTGA

ACTACTTTCTTTGTGTCTTC

In100 candidate1649 (in intron):

LOCUS ENSG00000151458

DESCRIPTION Ankyrin repeat domain-containing protein 50.

[Source:Uniprot/SWSSPROT;Acc:Q9ULJ7]

TATGCTTTCTTTCTACTCATAAACATAACATATCTGTGCATTTATTCAAGTTTCTCCTTAGATTTTCCAGTTATCCTTCT

ATATGTCCTACACATATCTT

In100 candidate1650 (in intron):

LOCUS ENSG00000188682

DESCRIPTION CDNA FLJ44078 fis, clone TESTI4039038.

[Source:Uniprot/SPTREMBLAcc:Q6ZU04]

TAACCTGGGCTATAATGGTCCTATGATCCATCCTATTTGTCTTTCCAGATGTTCTTGTTTTCACTCTACACTACACCTCT

CTCATTTCCTCTGATATATT

In100 candidate1651 (in intron):

LOCUS ENSG00000188682

DESCRIPTION CDNA FLJ44078 fis, clone TESTI4039038.

[Source:Uniprot/SPTREMBLAcc:Q6ZU04]

TTCCATGTCTCTTAACTCTTCTTTAATATTTTTCATCTCTTTGTATCTTTATGATGCATTTTTGCATCTTATTTTCAGAC

ATTTTTCTTCCATATTATTA

In100 candidate1652 (in exon):

LOCUS ENSG00000172795

DESCRIPTION mRNA decapping enzyme 2 (EC 3.-.-.-) (hDpc) (Nucleoside

diphospate- linked moiety X motif 20) (Nudix motif 20).

[Source:Uniprot/SWISSPROT;cc:Q8IU60]

GGGTCTCTTTCTGTATATAATTTCTCAAGCCATATTAAAGGAGAAAGTAACTTCCTTGACACTATTTATACTCTTCAGTC

ATTGTCCGTTTTTGCTGCCT

In100 candidate1653 (in intron):

LOCUS ENSG00000172795

DESCRIPTION mRNA decapping enzyme 2 (EC 3.-.-.-) (hDpc) (Nucleoside diphospate- linked moiety X motif 20) (Nudix motif 20). [Source:Uniprot/SWISSPROT;cc:Q8IU60]

TTCTTTAAGTATTTAAACTCTGTCCCCTCTCCCCCACACAAATCTTAACCATTTCTGAATTTATCATATTTTCAGTCAAA

ACAATTTATTTTCTCTCTCT

In100 candidate1654 (in intron):

LOCUS ENSG00000186688

DESCRIPTION Doublecortin domain-containing protein 1. [Source:Uniprot/SWISSROT;Acc:P59894]

TGCTTTTTTCCTATTTGCTTTCTTTGATTGTTAGTCGGTTTAACACTGGCTTTATTTGTCCTTTTCATTATATGTCAGTC

TTTCTTCTCATGTGACACTT

In100 candidate1655 (in intron):

LOCUS ENSG00000088538

DESCRIPTION Dedicator of cytokinesis protein 3 (Modifier of cell adhesion) Presenilin-binding protein) (PBP). [Source:Uniprot/SWISSPROT;Acc:Q8IZD9]

GCATATATATTTATAATTGTTATATTCTCTTGCTAACTTGATTGCTTTATTACTGTGTTGCCTTGTCTCTTTTTACAGCT

TTTAACTACAGGTCTTTTTC

In100 candidate1656 (in intron):

LOCUS ENSG00000088538

DESCRIPTION Dedicator of cytokinesis protein 3 (Modifier of cell adhesion)

Presenilin-binding protein) (PBP). [Source:Uniprot/SWISSPROT;Acc:Q8IZD9]

TATAGGCATTTTAACAATATTAATTATTCCAATCCATGAATCTGGCATACCTTTCCATTTATTTGTGTCCTTTTCAGTT

TCTTTCATTAGTGTTTTGTA

In100 candidate1657 (in intron):

LOCUS ENSG00000088538

DESCRIPTION Dedicator of cytokinesis protein 3 (Modifier of cell adhesion)

Presenilin-binding protein) (PBP). [Source:Uniprot/SWISSPROT;Acc:Q8IZD9]

GTATTCCAAAGTGACTATGTTGTAACTTGTGTATTTCCTTGGTTTTCTTCCTGCTTGGGATTCATTCAGCTTCTTGAATG

TGTACGTTTATGTCTTTTTC

In100 candidate1658 (in intron):

LOCUS ENSG00000088538

DESCRIPTION Dedicator of cytokinesis protein 3 (Modifier of cell adhesion)

Presenilin-binding protein) (PBP). [Source:Uniprot/SWISSPROT;Acc:Q8IZD9]

GGTGATAACCCCTTTATCGTTTTTTATTGCATCTATTTGATTCTTCTCTCTTTTCTTCTTTATTATTCTTGCTAGCAGTC

TATCAATTTTGTTGATCTTT

In100 candidate1659 (in intron):

LOCUS ENSG00000170946

DESCRIPTION DPH4 homolog (CSL-type zinc finger-containing protein 3).

[Soure:Uniprot/SWISSPROT;Acc:Q6P3W2]

AGTAATTCAACCACGTATCTTAACCTTTTACATTTCTTTATTTTTGTGAATTCTGTTTCTGCAGATCTATCTCTTAATTC

TCTTTTTTTTTTTTTTTTTT

In100 candidate1660 (in intron):

LOCUS ENSG00000171444

DESCRIPTION Colorectal mutant cancer protein (Protein MCC).

[Source:UniprotSWISSPROT;Acc:P23508]

AGTGTGTATTAGCTATTATTATAAATATGACTATTAATATTATTGTTAACCTCTTCTCAATTCTTTGCTGTTACAGATAT

TGTTCATGTCTCCTCCATTT

In100 candidate1661 (in intron):

LOCUS ENSG00000171444

DESCRIPTION Colorectal mutant cancer protein (Protein MCC).

[Source:UniprotSWISSPROT;Acc:P23508]

AGAATGTTAATAAGCTAACAAGTGGCAATACTAATTCCTTTTAAATTTTTATGACTTTTGCCCTAATTTTGTCCCAGGTT

TATTGTTTTCAATCCCTCTT

In100 candidate1662 (in intron):

LOCUS ENSG00000171444

DESCRIPTION Colorectal mutant cancer protein (Protein MCC).

[Source:UniprotSWISSPROT;Acc:P23508]

AGAGGGTAACAACTGCAACCTTTTAATGTCATTTCCCTACTTGAACCTTCCAGAGTCTCCCCATTATCTTTTCCTCTTTA

TTTGCTTGTTGAACAGTTCA

In100 candidate1663 (in intron):

LOCUS ENSG00000105976

DESCRIPTION Hepatocyte growth factor receptor precursor (EC 2.7.10.1) (HGF

eceptor) (Scatter factor receptor) (SF receptor) (HGF/SF receptor) (Met

proooncogene

tyrosine kinase) (c-Met). [Source:Uniprot/SWISSPROT;Acc:P08581]

EXON

GGGATGCTTAAGATAGTGGTAAATGATGAATATAACATATCTAGTCCCTGGAGTTTTTGTCACCTTCTCTTTCTCCAGTT

CTCTCTCTCTTTTTTTTTTT

In100 candidate1664 (in intron):

LOCUS ENSG00000105976

DESCRIPTION Hepatocyte growth factor receptor precursor (EC 2.7.10.1) (HGF

eceptor) (Scatter factor receptor) (SF receptor) (HGF/SF receptor) (Met

proooncogene

tyrosine kinase) (c-Met). [Source:Uniprot/SWISSPROT;Acc:P08581]

EXON

TCCCATCTTTGGATCTCCTGAAAACAACTTGTTTCATTAACATGTCATGTAGTTTTAATGTTTGTTCCAGATGCTCTGAA

ATTTGTGACCCTTTTCCCTT

In100 candidate1665 (in intron):

LOCUS ENSG00000164066

DESCRIPTION PDZ domain-containing protein 6 (Inturned planar cell polarity

ffector homolog). [Source:Uniprot/SWISSPROT;Acc:Q9ULD6]

TGTGCCACTCCTTCTATCCATAAATCCCCACCTCTAACAAGCAAGCCTGATTTATTTTTTTCCTAACTGACCTCCAGATA

CACCTTGTGTGTCCATCTTT

In100 candidate1666 (in intron):

LOCUS ENSG00000164066

DESCRIPTION PDZ domain-containing protein 6 (Inturned planar cell polarity

ffector homolog). [Source:Uniprot/SWISSPROT;Acc:Q9ULD6]

TGTGGGGTGTTAACTCTGATGATTATTTATTTTGCTATGCAGAAACTTTAGTTTAATTAAGTCCCATCTATTTATCTTTG

TTTTTGTTGCATTTGCTTTT

In100 candidate1667 (in intron):

LOCUS ENSG00000151475

DESCRIPTION solute carrier family 25 (mitochondrial carrier; adenine

nucleoide translocator), member 31 [Source:RefSeq_peptide;Acc:NP_112581]

TGACCCCTTCATATGAAATGTTAACTATCTTAAACTATCCTTACTAGTTTTTAGCTTTATCTTTTTGCTTTTCAACAGTC

CTGGAGTTTTTTTATTTGTT

In100 candidate1668 (in intron):

LOCUS ENSG00000151475

DESCRIPTION solute carrier family 25 (mitochondrial carrier; adenine

nucleoide translocator), member 31 [Source:RefSeq_peptide;Acc:NP_112581]

AGCAAAGTATTTCAAAGACCTAACTGGACTTTTTTTGTGCTGTTGTGTGTGTTTTCTATTTCCTTTATTTCTGCCAGTTA

TTTTCTTGTTTTTACTTTCT

In100 candidate1669 (in intron):

LOCUS ENSG00000164080

DESCRIPTION RAD54-like 2 [Source:RefSeq_peptide;Acc:NP_055921]

CCAATTTTCTTTGTTTTTTTAAAACAAATGCTAACATACCATAAAATACTATTTACTCCTTTTTTCACTCACCAGTAATA

GTTTATTCCTTATCATTTCT

In100 candidate1670 (in intron):

LOCUS ENSG00000004866

DESCRIPTION suppression of tumorigenicity 7 isoform a

[Source:RefSeq_peptid;Acc:NP_060882]

AGAGTTCCTACCCCTTCTTACCTCCATAACAAACTGATGTGTCTTCCACTTTTTGGTTTCTGGTGTTAACTCTTGCAGAT

TCCCCGTTTTTTTTTTTTTT

In100 candidate1671 (in intron):

LOCUS ENSG00000080709

DESCRIPTION Small conductance calcium-activated potassium channel protein

2(SK2). [Source:Uniprot/SWISSPROT;Acc:Q9H2S1]

GTACCACCTGATTTTAATGATTAACTTATGTTTTAAAATTTATGTGGCTGCTTCTCCCTTGTTACTCTTCCTTTTCAGAA

TTTTCCTGGCTATTCTACTT

In100 candidate1672 (in intron):

LOCUS ENSG00000171045

DESCRIPTION t-SNARE domain-containing protein 1.

[Source:Uniprot/SWISSPROT;cc:Q96NA8]

GTGGCCTCTATGCTCTTGTGTCTGCTTGCTCTGCTGACCGCGCTGCCTGTAACTGTGGATTTGTGTGTTTCTTTCCAGTT

CTATCAGTTTTTGCTTCACG

In100 candidate1673 (in intron):

LOCUS ENSG00000164073

DESCRIPTION major facilitator superfamily domain containing 8

[Source:RefSe_peptide;Acc:NP_689991]

TGACATTTCTCTATCCTGGCTTTCCTCTTAACTCTAATGACTTATTCTTAAATTCTTTTGTGTATTCTTCCTTATCAGTT

TATCCCTTGTTGGTTTTTCT

In100 candidate1674 (in intron):

LOCUS ENSG00000173389

DESCRIPTION IQ motif containing F1 [Source:RefSeq_peptide;Acc:NP_689610]

ACATATTGACATACTGATTCTTTGAAGAGAAATTTAGAACTCATACCTAACAACTTCTCCTCTTGCATTTCCTTACAGCT

CTTTCTCTTTAAACTTCTTT

In100 candidate1675 (in intron):

LOCUS ENSG00000154438

DESCRIPTION Ankyrin repeat, SAM and basic leucine zipper domain-containing

rotein 1 (Germ cell-specific ankyrin, SAM and basic leucine zipper

domainontaining

protein) (Ankyrin-like protein 1).

[Source:Uniprot/SWISSPROT;Acc:8WWH4]

TTTGTTTCTTAACACTCTTTTCTTCCTTTATCTTCTCCATATTCACACTGGGTATTTTCTTTTGGTCTATCTTCCAGGCC

ATTAATTCTCTTCTGTAGCT

In100 candidate1676 (in intron):

LOCUS ENSG00000154438

DESCRIPTION Ankyrin repeat, SAM and basic leucine zipper domain-containing

rotein 1 (Germ cell-specific ankyrin, SAM and basic leucine zipper

domainontaining

protein) (Ankyrin-like protein 1).

[Source:Uniprot/SWISSPROT;Acc:8WWH4]

ATCTTTTCTCCTTTGAATCCATCCCTTTCATTGCCATTCATAACAACTCTTAACTACTGATTTTCTTCTACTTCTCAGGC

TACGTTTCCTTTAGTGGCTT

In100 candidate1677 (in intron):

LOCUS ENSG00000125875

DESCRIPTION TBC1 domain family member 20.

[Source:Uniprot/SWISSPROT;Acc:Q96Z9]

GTGTGTATACACATTTAGTTTTATTGTAACAAGGCAACTCGTACACTTTTTTTTTTCCTTTTTTCTTGTATAAGCAGGTG

AATCCATGTTCTTTCTGCCT

In100 candidate1678 (in intron):

LOCUS ENSG00000001626

DESCRIPTION Cystic fibrosis transmembrane conductance regulator (CFTR)

(cAMdependent

chloride channel) (ATP-binding cassette transporter sub- familyC

member 7). [Source:Uniprot/SWISSPROT;Acc:P13569]

CATTGAATGTATTTGGGCCTGCATATGTCTAACATGAAAATTATAGTCCTTTTTTTGTTTCTTTGTTTGTATTTACAGTT

TTAAGTTCCATTTTCAACCT

In100 candidate1679 (in intron):

LOCUS ENSG00000130193

DESCRIPTION mesenchymal stem cell protein DSCD75

[Source:RefSeq_peptide;AccNP_057731]

TTTCTTACAACTTTCACAATCTTCAACATGTCTTAACTTTCTGCCTGCCTTTTACGCTATTTCTTTTTCTCAGTTTCACC

CTCTATGTCTTCCTTTGATT

In100 candidate1680 (in intron):

LOCUS ENSG00000083535

DESCRIPTION Progesterone-induced-blocking factor 1.

[Source:Uniprot/SWISSPRT;Acc:Q8WXW3]

ACAACAAATAAAAAGCTGAGCTGGAAAATCATTAACTTTTCTTTTTTTTTTTTTTTTTTTTTTTTTTTGAGACAGAG

TTTTTCACTCTTTGTTGTCC

In100 candidate1681 (in intron):

LOCUS ENSG00000128534

DESCRIPTION U6 snRNA-associated Sm-like protein LSm8.

[Source:Uniprot/SWISSROT;Acc:O95777]

GGCTACTGAACTTTCTCTATTTTCTAACCTTACCTAGTTTCTTTAAATAATTTGATCCCTTATTTTTCCATTCTTCAGTT

TTGCTTTTGTTCATTTTCTT

In100 candidate1682 (in intron):

LOCUS ENSG00000123338

DESCRIPTION Nck-associated protein 1-like (Membrane-associated protein HEM-)

(Hematopoietic protein 1). [Source:Uniprot/SWISSPROT;Acc:P55160]

CCACCTCGGCCTCCCAAAGGCTGGACTGTGCCGGCCTATTTTTAACTTTTTGAGGAACTGCTATACTGTTTTCCACAGTG

GCTGTACTCTTTTACATTCT

In100 candidate1683 (in intron):

LOCUS ENSG00000072736

DESCRIPTION Nuclear factor of activated T-cells, cytoplasmic 3 (NF-ATc3)

(NATc3) (T cell transcription factor NFAT4) (NF-AT4) (NFATx).

[Source:UniprotSWISSPROT;Acc:Q12968]

ACTGATGTCTCTTCTAACCTCTTAACTCATTCTTCTTTTTCTCCCTAATCCTAAACTTTGGAGTTCCTTGTGCCTCAGTT

GCCTACTTTCTTCTCTATTT

In100 candidate1684 (in intron):

LOCUS ENSG00000118922

DESCRIPTION Krueppel-like factor 12 (Transcriptional repressor AP-2rep).

[Surce:Uniprot/SWISSPROT;Acc:Q9Y4X4]

AATGGACTCAAATTGTGTGCACTTTTGTAACTGGTTTAATTTTATTTCACATTGCGTTTTTTTTTTTCCATGTACCAGTT

GTTTGTTCTCATTGTTTTAT

In100 candidate1685 (in exon):

LOCUS ENSG00000189184

DESCRIPTION Protocadherin-18 precursor. [Source:Uniprot/SWISSPROT;Acc:Q9HCL]

ATACTTGAACATTTTAATCTTTTGGAATATTGTCTTTCTTTGTTATAACTATTCATTTTTAGCTTTTGTCTCCAGTGCAT

GATCTCATATTTTTGCTTTT

In100 candidate1686 (in intron):

LOCUS ENSG00000092421

DESCRIPTION Semaphorin-6A precursor (Semaphorin VIA) (Sema VIA)

(Semaphorin6A-1) (SEMA6A-1). [Source:Uniprot/SWISSPROT;Acc:Q9H2E6]

GCACTCTGTTGTTGTAAATAACCCATTTATCGTCATCGACTCTTTTGGATTTTTCCCAGAACTAATTTCATTTTTGAAAT

AGTTTCTCTTCTTATTGTTT

In100 candidate1687 (in intron):

LOCUS ENSG00000106034

DESCRIPTION CDNA: FLJ21986 fis, clone HEP06248.

[Source:Uniprot/SPTREMBL;Ac:Q9H6Q5]

CATCATGCCACTTCTACATTAAAGTTGATTAATTATCTTTGATTAATTAACTATCATTGATTTCTCGTTATCATCAGCTC

CTTTGTCTCATTGAAGTCCT

In100 candidate1688 (in intron):

LOCUS ENSG00000106034

DESCRIPTION CDNA: FLJ21986 fis, clone HEP06248.

[Source:Uniprot/SPTREMBL;Ac:Q9H6Q5]

GTAACACTGGTGTTCTTTTAGTTTTGCCTTCTGATTTGCCAGATGAATATATTTCTTGTTCTCCTTTCTATTTGTTTTTA

AAACAATTGCTGCTTTATTT

In100 candidate1689 (in intron):

LOCUS ENSG00000136111

DESCRIPTION TBC1 domain family member 4 (Akt substrate of 160 kDa)

(AS160).[Source:Uniprot/SWISSPROT;Acc:O60343]

GATATAAACATAGGGCCCTTAACAATTGGTTCCCTCTTCAATATGTATTCCTTCATGTTCAGTCTAGTCTTGATGTCCTT

CACAGCTCAGTGTTTTTTTT

In100 candidate1690 (in intron):

LOCUS ENSG00000136111

DESCRIPTION TBC1 domain family member 4 (Akt substrate of 160 kDa)

(AS160).[Source:Uniprot/SWISSPROT;Acc:O60343]

TGGAGTACTAAGCTGATGTATAGCAACGACGCTTAACAAGAAAAGCCCTTGAGATGTCCTCTTCCCTATTTCTGTCAGTT

TCTCATTCTGCTTTATAAAT

In100 candidate1691 (in intron):

LOCUS ENSG00000136111

DESCRIPTION TBC1 domain family member 4 (Akt substrate of 160 kDa)

(AS160).[Source:Uniprot/SWISSPROT;Acc:O60343]

CACATGCTTTTTTTGTGGTGAACATCTAGAATCACTGCTAACAACCACTGCAAATAACTGTCCTCTGCTTTATTCCAGTT

TCTACATGTTGCTTTTATCA

In100 candidate1692 (in intron):

LOCUS ENSG00000169570

DESCRIPTION DTW domain-containing protein 2.

[Source:Uniprot/SWISSPROT;Acc:8NBA8]

CAAGTTTGAATAGTTTCTTCTTCTAACTCCTCACTTTGTCTTTTTTCTCTACTATATTGTTAGCTTCCTGTGGGTCAGCC

TCTTCGTATTTCTTACGGTA

In100 candidate1693 (in intron):

LOCUS ENSG00000160551

DESCRIPTION Serine/threonine-protein kinase TAO1 (EC 2.7.11.1) (Thousand an

one amino acid protein 1) (STE20-like kinase PSK2) (Kinase from chicken hoolog

B) (hKFC-B). [Source:Uniprot/SWISSPROT;Acc:Q7L7X3]

TGGAGTTTTTAAAAATTGTTGAATTTGAACAAGTTAAAAGAAAATAGCTAACACTTACTGTAGTGCTTATTACTCCAGAG

TATTACTCTATGCTTTCATT

In100 candidate1694 (in intron):

LOCUS ENSG00000172869

DESCRIPTION Protein DmX-like 1 (X-like 1 protein).

[Source:Uniprot/SWISSPRO;Acc:Q9Y485]

GGTAACATCTCCTTGGTCATTTCTAATTGTGTTTATTTGTATCTTCTCTCTTTTCTTTTTTATTAGTCTAGCTGGCAGTC

TGTCTGTCTTACTAATTTTT

In100 candidate1695 (in intron):

LOCUS ENSG00000186714

DESCRIPTION sarcoma antigen NY-SAR-79 [Source:RefSeq_peptide;Acc:NP_00100832]

AGATTTTCTCCCACTCTGTGGGTTGTCTGTTAACTCTGCTGATTATTTCTTTTGCTGTGCAGAATCTTTTTAGTTTAATT

AAGTCCCATCTATTCATCTT

In100 candidate1696 (in intron):

LOCUS ENSG00000014164

DESCRIPTION Zinc finger CCCH domain-containing protein 3.

[Source:Uniprot/SISSPROT;Acc:Q8IXZ2]

GTTGTTGAGAGAGGTGCTGAAGTCTCTAACTATAATTGTGGATTTGTCTGTTTCTCCTTTCATAGCTAACAGTTATTGCT

TCATGTATTTTACAGCTTTT

In100 candidate1697 (in intron):

LOCUS ENSG00000118939

DESCRIPTION Ubiquitin carboxyl-terminal hydrolase isozyme L3 (EC

3.4.19.12)(UCH- L3) (Ubiquitin thioesterase L3).

[Source:Uniprot/SWISSPROT;Acc:P15374

TGTCTTTCTAACTTTAATTATTTCTTTTTAGCCATCCCTTATTTCCTTATTTATTATTTCTATTCTTCTTGCAGTACTC

TCTTTCTGAAAATATTCTTT

In100 candidate1698 (in intron):

LOCUS ENSG00000118939

DESCRIPTION Ubiquitin carboxyl-terminal hydrolase isozyme L3 (EC

3.4.19.12)(UCH- L3) (Ubiquitin thioesterase L3).

[Source:Uniprot/SWISSPROT;Acc:P15374

AGGTGTGAGCCACTAACAAAACACTTGACTTCTTCTTAGGCAGACCCTCTGCTTTGTTTGATTTTTTTTTTTTTTTTAAG

TCTGGATCATTTCTTTTTAG

In100 candidate1699 (in intron):

LOCUS ENSG00000198720

DESCRIPTION Ankyrin repeat domain-containing protein 13B.

[Source:Uniprot/SISSPROT;Acc:Q86YJ7]

CACTGTGCCTGGCCTCCTGACATTCTTATACATTTAACATTTTTTGGCACTCTTCAATCCTTTGTACAGATTCAAGTTTC

TATCTGGGATCATTTTCTTT

In100 candidate1700 (in intron):

LOCUS ENSG00000081803

DESCRIPTION Calcium-dependent secretion activator 2 (Calcium-dependent

actiator protein for secretion 2) (CAPS-2). [Source:Uniprot/SWISSPROT;Acc:Q86UW]

AACATTGGATCTGGTATAGCAAGTTACCATCATTTAATATGTAACTCAACCTGAATTTCTCTTTTACTTTTTCAGTTTTT

ACTATTTTTTGTTTCTTTAT

In100 candidate1701 (in intron):

LOCUS ENSG00000081803

DESCRIPTION Calcium-dependent secretion activator 2 (Calcium-dependent

actiator protein for secretion 2) (CAPS-2). [Source:Uniprot/SWISSPROT;Acc:Q86UW]

TGTCTAACGTTTCGCTGCAATATATTTTGAATTTTTTTTGTATTTCATTATTACCCATGGATTTTTTTTTTCCTCCAGGT

CTATTGCTATTGTGTTTCTG

In100 candidate1702 (in intron):

LOCUS ENSG00000184838

DESCRIPTION mesenchymal stem cell protein DSC54

[Source:RefSeq_peptide;Acc:P_057728]

AGCAATCATTAGATCTGGTTAACCCCCTAGACGGTGTTTCATCTCTCATTGCTTTTTTAAATTCCTGCCATGATCCAGGT

TCTATTTGTATTTTGACCTT

In100 candidate1703 (in intron):

LOCUS ENSG00000184838

DESCRIPTION mesenchymal stem cell protein DSC54

[Source:RefSeq_peptide;Acc:P_057728]

AATATAAAAGATGTCTGGATAATGTCAAATATTAGAGATATATTAACATGTTTACAATTACTGTTTTTCTTTAATCAGTT

GCATTCTTTTGTCATGCATT

In100 candidate1704 (in intron):

LOCUS ENSG00000184838

DESCRIPTION mesenchymal stem cell protein DSC54

[Source:RefSeq_peptide;Acc:P_057728]

TAACTTTTGTATTACTTCTCACTTCCACTTTTTCTGTATTCCACTTTCAAATTTGAAATCCTTCTGGGACTTCATCAGTA

CTTTTTCCACCTTTTTATTA

In100 candidate1705 (in intron):

LOCUS ENSG00000184838

DESCRIPTION mesenchymal stem cell protein DSC54

[Source:RefSeq_peptide;Acc:P_057728]

TTATGTTTTGTAATCAATGCATAACTTGACTTACTTGTTTACCAATGCCTTTGTTTATCTATGCTTCTTGCATTCCAGTT

CTTCCTTCTGATTATTTACC

In100 candidate1706 (in intron):

LOCUS ENSG00000110422

DESCRIPTION Homeodomain-interacting protein kinase 3 (EC 2.7.11.1) (Homologof protein kinase YAK1) (Fas-interacting serine/threonine-protein kinase) (IST) (Androgen receptor-interacting nuclear protein kinase) (ANPK). [SourceUniprot/SWISSPROT;Acc:Q9H422]

CTGGTAATAAAAATGCCCACATCATTGTAGTGTTAGGAATAATAGATGCTAACAATATAATGCTGTTGTGTTTTTCAGTT

TTTTCCCCCAAGTGTTTATT

In100 candidate1707 (in intron):

LOCUS ENSG00000136153

DESCRIPTION LIM domain only protein 7 (LOMP) (F-box only protein 20). [Soure:Uniprot/SWISSPROT;Acc:Q8WWI1]

TAGGTATTTTTTTAAACTATTAACATTTTATCTGTACCCATTTTTATTGCAGACTTGGGTTTCAATGCAGGTATGGGTTC

TTTGTTCTCTTCTCTCTCCC

In100 candidate1708 (in intron):

LOCUS ENSG00000136153

DESCRIPTION LIM domain only protein 7 (LOMP) (F-box only protein 20). [Soure:Uniprot/SWISSPROT;Acc:Q8WWI1]

AAAATTCAAGTGTGTTTTGGACCACTTGAAGTCAAATAAGTCAAATTTATAACAAAGGATTTCTCTCTAGTCTGTCAGTT

ACCACTACTTTTTCTTTTTC

In100 candidate1709 (in intron):

LOCUS ENSG00000136153

DESCRIPTION LIM domain only protein 7 (LOMP) (F-box only protein 20).

[Soure:Uniprot/SWISSPROT;Acc:Q8WWI1]

GCTGATCTTGCTATTTTTCTGTTAATGGGTCCGTAACTATAACCACATTATTATCTTTCTCAGTTCCCAATTTATCACGT

ACTTTTCTGATCTGGTTCCT

In100 candidate1710 (in intron):

LOCUS ENSG00000141298

DESCRIPTION Protein phosphatase Slingshot homolog 2 (EC 3.1.3.48) (EC

3.1.316) (SSH-2L) (hSSH-2L). [Source:Uniprot/SWISSPROT;Acc:Q76I76]

CTGAAATATTTATAAGAATGAAAGCTGCAACATCTTAACTAGTGTAATTTGGTTTAATTTTTACTTTTCTCTCTTCAGGT

CTGTTTTGTTTTGTTTTTGG

In100 candidate1711 (in intron):

LOCUS ENSG00000141298

DESCRIPTION Protein phosphatase Slingshot homolog 2 (EC 3.1.3.48) (EC

3.1.316) (SSH-2L) (hSSH-2L). [Source:Uniprot/SWISSPROT;Acc:Q76I76]

CTACTTCACAAATTTTAAAAAGTAGAGTTTATATAAACCGAATCATAACACATTTCAAAACTGTATTGTTTTTTACAGAT

TTGATTGTTTTCTATAATTT

In100 candidate1712 (in intron):

LOCUS ENSG00000176927

DESCRIPTION EF-hand calcium binding domain 5 isoform 2

[Source:RefSeq_peptie;Acc:NP_001028734]

AAATACTTTTTCCCTCCTCTTCCATGATATCATACTTGTCTAACTTTCCTTCTGTTTCTCTATTTAGTCCTTCTCAGTGT

CCTTTTCTGGCTCATTCTCT

In100 candidate1713 (in intron):

LOCUS ENSG00000164675

DESCRIPTION IQ and ubiquitin-like domain-containing protein.

[Source:Unipro/SWISSPROT;Acc:Q8NA54]

TATATGTTGACATTTTTAACACTGTTTTTCATTTTATACTTTTATCTCTTATGTTTTTTTTTGCTGCTTTTGGGACAGTT

TCTTCATATGTATCTTTTTG

In100 candidate1714 (in intron):

LOCUS ENSG00000181135

DESCRIPTION Zinc finger protein 707. [Source:Uniprot/SWISSPROT;Acc:Q96C28]

EXON

AGTGTGCCTTTTCATGTTTTTATAGACCGTTGTATAACTTTGGTTGAAGTGTCTGTTCTAATATTTTGCCCAGTTTAAAT

TTTATTTCTCTTTTTTATTG

In100 candidate1715 (in intron):

LOCUS ENSG00000205302

DESCRIPTION Sorting nexin-2 (Transformation-related gene 9 protein) (TRG-9)

[Source:Uniprot/SWISSPROT;Acc:O60749]

AGTTCTAACATTTTGTTTTCTTTCTTCTTTTTGTAGCAGCTTTCTTTTTTTTGCAGCAGCTTCTGTTTCTTTGCTGAGAGC

TGTCATTTCATTCACTTCAA

In100 candidate1716 (in intron):

LOCUS ENSG00000170153

DESCRIPTION ring finger protein 150 [Source:RefSeq_peptide;Acc:NP_065775]

TAACATAGTCTTGTTTTTTTCTGCTATTCATTTTATTCCCTTTCACCTTACACCCACCATTATTTATTCTTTTCCAGACT

TTCAACTCATACACTTTTAT

In100 candidate1717 (in intron):

LOCUS ENSG00000170153

DESCRIPTION ring finger protein 150 [Source:RefSeq_peptide;Acc:NP_065775]

GTACATTAAACCTTCTCATCCCCTGTCCTCTGTATCTCTTAACCTCTTTTGCATATTTTCTATCTCCAGGTCTCTGTGCT

GCATTCTGAGTAATTTCTCC

In100 candidate1718 (in intron):

LOCUS ENSG00000170153

DESCRIPTION ring finger protein 150 [Source:RefSeq_peptide;Acc:NP_065775]

ACAATTTAGTCTAGTTATTGAGGTTATTTAATTGAATAACTGATTGAATGACTATATTCATCATTTCAGGTACAATTTGG

TTATTTGTTTTCATCTGCCT

In100 candidate1719 (in intron):

LOCUS ENSG00000135363

DESCRIPTION Rhombotin-2 (LIM domain only protein 2) (Cysteine-rich protein TG-2) (T-cell translocation protein 2). [Source:Uniprot/SWISSPROT;Acc:P2579]

TGGAAACATTTCTCTAACTGAAGATAAGAATTTTTTTTCTCTCTCTTTCTCCTTTCTACTTCTAGCTGTCTAAACCAGAG
GGTTCCTCTCATTTTCTTTC

In100 candidate1720 (in intron):

LOCUS ENSG00000168807

DESCRIPTION Beta-2-syntrophin (59 kDa dystrophin-associated protein A1 basi component 2) (Syntrophin 3) (SNT3) (Syntrophin-like) (SNTL). [Source:Uniprt/SWISSPROT;Acc:Q13425]

GTATTAACCTTTGGATTTTTGCCTTTCTTTTCCATTTAAAACAGTTTCCATAAACCCTCCCTTTCCCTTTTTTATTGCAG
TGGGAATCCATTGCTTGTTT

In100 candidate1721 (in intron):

LOCUS ENSG00000108582

DESCRIPTION Carboxypeptidase D precursor (EC 3.4.17.22) (Metallocarboxypeptdase D) (gp180). [Source:Uniprot/SWISSPROT;Acc:O75976]

TCTATCTCTGTGAATTTGCCCATTTTAGATATTTTATATAACTGGAGTTATACAATATTCTTCCTTTTGCATCAGGCTTA
TTTTTCTTAACATAATATTC

In100 candidate1722 (in intron):

LOCUS ENSG00000108582

DESCRIPTION Carboxypeptidase D precursor (EC 3.4.17.22) (Metallocarboxypeptdase D) (gp180). [Source:Uniprot/SWISSPROT;Acc:O75976]

AGTGATAACAATAGTAGGCCTTTTGCTATTGTTCTTAAAATTTTTTCTTCTTTTTTTTCAGATCTTTTTTTCTTCTTTCT
CTGTTATTCATAAGGCAATT

In100 candidate1723 (in intron):

LOCUS ENSG00000109445

DESCRIPTION Zinc finger protein 330 (Nucleolar cysteine-rich protein)

(Nuclolar autoantigen 36). [Source:Uniprot/SWISSPROT;Acc:Q9Y3S2]

GGTGCCTTAGTGGTCTGGGCGTTACGTTACCGAAATAACCACCTATTAAATCTGGCTATTTTTCAGTTGCTACCGTACTG

CTTACCACCTGTTTTGTTTT

In100 candidate1724 (in intron):

LOCUS ENSG00000064652

DESCRIPTION Sorting nexin-24. [Source:Uniprot/SWISSPROT;Acc:Q9Y343]

CTGAATTATTTAATTATCTATCATAGTAGGAAAATGTCATCTCATAGTAACTGAGAATTAATTTTAGTTTCACTCAGGTG

GGGTCTTTTTACCACTTCCT

In100 candidate1725 (in intron):

LOCUS ENSG00000064652

DESCRIPTION Sorting nexin-24. [Source:Uniprot/SWISSPROT;Acc:Q9Y343]

CACTTTCTCCCTTTTATTTTTATGTACTTTTACATAACATTTTTATGTATTTTTACATTTTTCCACATGCAGTCATCAGC

TTATCCTGTTGGTTCTACTT

In100 candidate1726 (in intron):

LOCUS ENSG00000108587

DESCRIPTION Golgi SNAP receptor complex member 1 (28 kDa Golgi SNARE protei)

(28 kDa cis-Golgi SNARE p28) (GOS-28). [Source:Uniprot/SWISSPROT;Acc:O9529]

TGATGTTAAGCCTTCCTTCAAGTGCTTATTAACCATTCACGTATCTTCTTTTTATTATTCACAACTCACAGGTTATTGT

GACATTTGTATATCTTCTTT

In100 candidate1727 (in intron):

LOCUS ENSG00000108587

DESCRIPTION Golgi SNAP receptor complex member 1 (28 kDa Golgi SNARE protei)

(28 kDa cis-Golgi SNARE p28) (GOS-28). [Source:Uniprot/SWISSPROT;Acc:O9529]

AAGTCCTATATACCCTTTACCCAACTTCTACAAAGTTTAACATACATTGATGCTCTTTACTCCTAAATATTTCAGTGTTC

GTTTCCTAAATATCTGTTTC

In100 candidate1728 (in intron):

LOCUS ENSG00000108587

DESCRIPTION Golgi SNAP receptor complex member 1 (28 kDa Golgi SNARE protei)

(28 kDa cis-Golgi SNARE p28) (GOS-28). [Source:Uniprot/SWISSPROT;Acc:O9529]

TCTTTTTGTTTAACATTCTGGGAGATTTACTGAATTTCTCCTTCCAATTCTTCTATTACATTTTTATATCAGCTCTTCAT

TTAATTTACATGAGCTCTTT

In100 candidate1729 (in intron):

LOCUS ENSG00000168944

DESCRIPTION coiled-coil domain containing 100

[Source:RefSeq_peptide;Acc:NP694955]

CTTGTTTCTTATTCCTGTTTTTGTCTGTAACTCTTTTCCCACATTTGTGGTTTTAATTTTCTCTTTCTTGCATATCAGTT

ATACTTTTTTGTTTTTTGTT

In100 candidate1730 (in intron):

LOCUS ENSG00000109452

DESCRIPTION Type II inositol-3,4-bisphosphate 4-phosphatase (EC 3.1.3.66)

(nositol polyphosphate 4-phosphatase type II).

[Source:Uniprot/SWISSPROT;AccO15327]

ATCTGTTTAATATTCCGGCCAATATTTTACACATTATATGTAACAAAATTAAAGCACATTTGGTTACTTTTCTCCCAGTT

CTTCTATGATTCACTCTGTA

In100 candidate1731 (in intron):

LOCUS ENSG00000178734

DESCRIPTION CDNA FLJ35379 fis, clone SKMUS2006481.

[Source:Uniprot/SPTREMBLAcc:Q8NAG9]

ACCAAACTCTGAACATGACTTTGCTTAACTTTTATTTTCTATTTTATTAATTTAATCACCTTGTATTTTGCCTCTCAGAT

CTCTTGTTTCATGTGTTTAC

In100 candidate1732 (in intron):

LOCUS ENSG00000128513

DESCRIPTION Protection of telomeres 1 (hPot1) (POT1-like telomere end-bindig

protein). [Source:Uniprot/SWISSPROT;Acc:Q9NUX5]

ATCTTTTTTTCTTATTGATTCTCTTTAACTTTAAATAATATATTTGTCTTTCATTTCTTATTCGTCAACTTTATTCAGTG

TTGTTGTTTACTTTCTTCTC

In100 candidate1733 (in intron):

LOCUS ENSG00000128513

DESCRIPTION Protection of telomeres 1 (hPot1) (POT1-like telomere end-bindig protein). [Source:Uniprot/SWISSPROT;Acc:Q9NUX5]

CTTCTGTAACCATACATATTTCTTCCATTCTTTGGCTATCTCTATGCTACATTTTAGTTTTCTTCATATTTGGATCAGAT
ATTCCTGTATAGCCTTTTAT

In100 candidate1734 (in intron):

LOCUS ENSG00000128513

DESCRIPTION Protection of telomeres 1 (hPot1) (POT1-like telomere end-bindig protein). [Source:Uniprot/SWISSPROT;Acc:Q9NUX5]

AACATCACAACAATTACGTATCCACCATTTAGATTTAACAAATCGTAACGTTTTGACATTATTTCAGACTTTTTTTTTTT
TTTTTTTTTTGGAGACAGT

In100 candidate1735 (in intron):

LOCUS ENSG00000197522

DESCRIPTION SUZ12P protein. [Source:Uniprot/SPTREMBL;Acc:Q86WX1]

ACTTAATGATGCATCATTTATGCATCTCATTGTTTTCCCATTGTTCTGTTAACTGGTAAAAGACTTCCTTTTTTTCAGTT
TGGCTTCCTTCTACTTTAAG

In100 candidate1736 (in intron):

LOCUS ENSG00000151292

DESCRIPTION Casein kinase I isoform gamma-3 (EC 2.7.11.1) (CKI-gamma 3). [Surce:Uniprot/SWISSPROT;Acc:Q9Y6M4]

TAAATTGCATCTTATACTTCTCTTCTCTGTGCCACCATTTGACACGTAACTAGGGACCTGCTTAGAATTTTCTTTCAGAA
TTTCTTTTTCATCTTTCTTC

In100 candidate1737 (in intron):

LOCUS ENSG00000179603

DESCRIPTION Metabotropic glutamate receptor 8 precursor (mGluR8).

[Source:Uiprot/SWISSPROT;Acc:O00222]

CTATATATAAGAATGTGCATCCATTCAAGTAACTTTGGATGTCTTGTTTATTTCATTTTCTTCTCTTCCCCAAGCCAGTG

ATTTTTCACTTCTTTTTAAA

In100 candidate1738 (in intron):

LOCUS ENSG00000179603

DESCRIPTION Metabotropic glutamate receptor 8 precursor (mGluR8).

[Source:Uiprot/SWISSPROT;Acc:O00222]

AGGGAGAACGTGTACTGGGGATGCTGTATGGGCCAAAAATAACTCGGGGCTTTTTTTAATGCTTTTTCCAACCAGATCCA

CTTTTTCTTGATTACTGTAG

In100 candidate1739 (in intron):

LOCUS ENSG00000179603

DESCRIPTION Metabotropic glutamate receptor 8 precursor (mGluR8).

[Source:Uiprot/SWISSPROT;Acc:O00222]

CTTTTTTTGTCTTCTCTGAAATTATTTTAACTGATCAAGTTTTCTCTCATTTTTTTTCATTTGATAGTTCAGGAGTTTTA

GATACTATTTTCATTTTTTT

In100 candidate1740 (in intron):

LOCUS ENSG00000176208

DESCRIPTION chromosome fragility associated gene 1

[Source:RefSeq_peptide;Ac:NP_079133]

CATTTTTAGAGTTACATTAACTAAAACTTTCTATAAAATTTCTTTTTTTTTTAATTTTTTTTTTTTTTGGAGACAGAGT

CTTGCTCTGTTTCCCATAGT

In100 candidate1741 (in intron):

LOCUS ENSG00000176208

DESCRIPTION chromosome fragility associated gene 1

[Source:RefSeq_peptide;Ac:NP_079133]

TTTTCTATATGAGTATATATATATATATAGGTATATAGTATTCTTATGTAACTATAACGTACAATCACTTTTAGCTTCAGGA

GTCTTTAAATTCTGTCTTTC

In100 candidate1742 (in intron):

LOCUS ENSG00000109458

DESCRIPTION GRB2-associated-binding protein 1 (GRB2-associated binder 1).

[ource:Uniprot/SWISSPROT;Acc:Q13480]

TTGTCCAATGTTTCTTAACCTTTTCTAACCCACTCCTCTCTCATGTATTGTTTGAGTGTCCTTTGGCTTTTAATCCAGTT

TTACTTTCTATGATTTGCAT

In100 candidate1743 (in intron):

LOCUS ENSG00000121691

DESCRIPTION Catalase (EC 1.11.1.6). [Source:Uniprot/SWISSPROT;Acc:P04040]

CACTGCTTAGAATGCCATGGGCTCTTCTTTTGAGATACTTACCTTTAACCCATTTCCTTTTTTTTTTTTTAAGTGCAGCT

TGCTGCCAGTGCTCTTTTCT

In100 candidate1744 (in intron):

LOCUS ENSG00000102908

DESCRIPTION Nuclear factor of activated T-cells 5 (T-cell transcription facor NFAT5) (NF-AT5) (Tonicity-responsive enhancer-binding protein) (TonE- biding protein) (TonEBP). [Source:Uniprot/SWISSPROT;Acc:O94916]

AACTGAGTTACCTAATAACCTGAGATGAAAATACTCTTTTTGTTCAGGTTTGCTTTTATAGTTTCTCCTTCCTTTTCCCC

AGTCTGTCTGCTAAATAGCC

In100 candidate1745 (in intron):

LOCUS ENSG00000155324

DESCRIPTION GRAM domain-containing protein 3 (HCV NS3-transactivated protei 2). [Source:Uniprot/SWISSPROT;Acc:Q96HH9]

CTCTCAACTTCCTGTTTTGATGGAAAAGAATCAACTCCCTTATACTAACTCCCTCCTTCCCTCTCTTCCTTTTAACAGGC

GTATCTTTCTAGTTCCTATT

In100 candidate1746 (in exon):

LOCUS ENSG00000155324

DESCRIPTION GRAM domain-containing protein 3 (HCV NS3-transactivated protei 2). [Source:Uniprot/SWISSPROT;Acc:Q96HH9]

CTTCAAACAAGTAACATCTACATTTTGTCTTTTTTTTTTCAGTTCTCCTGTTATGTTCTGGTTGAAATCACCTGTGTGTC

TTAATTTCTCAATTCCTTTT

In100 candidate1747 (in exon):

LOCUS ENSG00000181019

DESCRIPTION NAD(P)H dehydrogenase [quinone] 1 (EC 1.6.5.2) (Quinone reductae 1) (NAD(P)H:quinone oxidoreductase 1) (QR1) (DT-diaphorase) (DTD) (Azoredctase) (Phylloquinone reductase) (Menadione reductase).

[Source:Uniprot/SWISPROT;Acc:P15559]

AGATCAAAGCTAGAAAATGAGATTCCTTAGCCTGGATTTCCTTCTAACATGTTATCAAATCTGGGTATCTTTCCAGGCTT

CCCTGACTTGCTTTAGTTTT

In100 candidate1748 (in intron):

LOCUS ENSG00000183090

DESCRIPTION FRAS1-related extracellular matrix protein 3 precursor.

[SourceUniprot/SWISSPROT;Acc:P0C091]

TTTTCAAAGATAATATCTTTGAAAAAAATATATTTTTTGTTTTATTTAACTTTTGTATTTTTTGTTTGTTTGTTTCAGTT

TCACTTAGTTCTGCTCTGAT

In100 candidate1749 (in intron):

LOCUS ENSG00000197157

DESCRIPTION Staphylococcal nuclease domain-containing protein 1 (p100 coativator)

(100 kDa coactivator) (EBNA2 coactivator p100).

[Source:Uniprot/SWSSPROT;Acc:Q7KZF4]

ACATGTTAACCCCCCACCCCCATTTTATTCTTGCTTCAGGAATTTTTGTCTCTGTTTTCTCCTTGTAAACTCTTTTGCCA

TCCCCATTGTTGTAGAAGCC

In100 candidate1750 (in intron):

LOCUS ENSG00000197157

DESCRIPTION Staphylococcal nuclease domain-containing protein 1 (p100

coativator)

(100 kDa coactivator) (EBNA2 coactivator p100).

[Source:Uniprot/SWSSPROT;Acc:Q7KZF4]

CTCCCTCAACACTTTTGTCTTAAAAAAGAAATATAAGTTTATAGAATAACATAACAAACATCTGGGTTCCTTTTTCAGCC

TTGATATTCTTCCCTGTGTG

In100 candidate1751 (in intron):

LOCUS ENSG00000197157

DESCRIPTION Staphylococcal nuclease domain-containing protein 1 (p100

coativator)

(100 kDa coactivator) (EBNA2 coactivator p100).

[Source:Uniprot/SWSSPROT;Acc:Q7KZF4]

AGTTCTTTTGTTCTCCTTGGGATTTTGTGCTATTGGCTATTCTCTTTTTAACTAGTGTTATTTTTAATTGTCCTTCAGTC

TTCTATCCTATTATATCTGC

In100 candidate1752 (in intron):

LOCUS ENSG00000197157

DESCRIPTION Staphylococcal nuclease domain-containing protein 1 (p100

coativator)

(100 kDa coactivator) (EBNA2 coactivator p100).

[Source:Uniprot/SWSSPROT;Acc:Q7KZF4]

TTGTTAACTAATAGGTTTAGTCTATTCCCATTTTCCCATGCTGTTTTCTGTGCTGTTATGTTTACCTCTCACCAGCCCTT

TCTAGTTTCATGCCTACTAT

In100 candidate1753 (in intron):

LOCUS ENSG00000110435

DESCRIPTION Pyruvate dehydrogenase protein X component, mitochondrial precusor (Dihydrolipoamide dehydrogenase-binding protein of pyruvate dehydrogenae complex) (Lipoyl-containing pyruvate dehydrogenase complex component X) (3-binding protein) (E3BP) (proX). [Source:Uniprot/SWISSPROT;Acc:O00330]

ACCACACTTTTAATTAGAAATGTTTTATTGTATGCTTTAAAATCTGATAACAATAGCTTTGCTCTTTTTTTAAATCAGTT
TTCTTAGTTCATTCTTATTT

In100 candidate1754 (in intron):

LOCUS ENSG00000110435

DESCRIPTION Pyruvate dehydrogenase protein X component, mitochondrial precusor (Dihydrolipoamide dehydrogenase-binding protein of pyruvate dehydrogenae complex) (Lipoyl-containing pyruvate dehydrogenase complex component X) (3-binding protein) (E3BP) (proX). [Source:Uniprot/SWISSPROT;Acc:O00330]

TATTAACTTATCTAGTATTTCTGTGATACTGATCTCATTGATTTCTGTTCTTTATTTTTTTTTCTCTTTGCTTTCAGTT
TACTTTGCTCTTTTATTTCT

In100 candidate1755 (in intron):

LOCUS ENSG00000005810

DESCRIPTION Probable E3 ubiquitin-protein ligase MYCBP2 (EC 6.3.2.-) (Mycbnding protein 2) (Protein associated with Myc) (Pam/highwire/rpm-1 protein) [Source:Uniprot/SWISSPROT;Acc:O75592]

CTTCTTCATAATTCTTATTTTCACTTAATTTCATAACCTTTTTGTATTGGATATCTTTTCTGTTTTTTTCTTACCCAGAG

GATCTTCCCCTCTTTTTTAA

In100 candidate1756 (in intron):

LOCUS ENSG00000005810

DESCRIPTION Probable E3 ubiquitin-protein ligase MYCBP2 (EC 6.3.2.-) (Mycbnding protein 2) (Protein associated with Myc) (Pam/highwire/rpm-1 protein) [Source:Uniprot/SWISSPROT;Acc:O75592]

GTTTTAATTCGTATTTTCCTCCAAGTTTAGTTAAATAACTTTTCTAGTCTTTATTGACCTTTCAGATTTCTTCTTCAGAG

AATTGTCTTGACTGTCTTTT

In100 candidate1757 (in intron):

LOCUS ENSG00000198373

DESCRIPTION NEDD4-like E3 ubiquitin-protein ligase WWP2 (EC 6.3.2.-) (WW doain- containing protein 2) (Atrophin-1-interacting protein 2) (AIP2). [Soure:Uniprot/SWISSPROT;Acc:O00308]

TTGCATTCTAACTTTGTATGTTTTTAACTTTCTGTAGTCCAATCTGTCACCCTTTATGTTTTCAGCATTTTCTTTCATGC

TTTGGAAGGCATTCCCCCTT

In100 candidate1758 (in intron):

LOCUS ENSG00000145794

DESCRIPTION MEGF10 protein [Source:RefSeq_peptide;Acc:NP_115822]

AGTATTATATTGGTCTAGACATGTATTCCATATTTCTTCATAACTAGTTTGAAATACTTTGCTTTTCAGACTTTTATTAT

GTTTAATAGCTAATTTTTCC

In100 candidate1759 (in intron):

LOCUS ENSG00000064651

DESCRIPTION Solute carrier family 12 member 2 (Bumetanide-sensitive sodium-(potassium)-chloride cotransporter 1) (Basolateral Na-K-Cl symporter). [Souce:Uniprot/SWISSPROT;Acc:P55011]

GATGTTTAATGTAATCTACCACATACTTTTTGTTTAATAACAATAAAACTTTTTTTTTGTTTTTGCCAGATTTTCTTCTT

AATCTTGAAAAGTACTTCTT

In100 candidate1760 (in intron):

LOCUS ENSG00000167491

DESCRIPTION Transcriptional repressor p66 alpha (Hp66alpha) (GATA zinc fingr domain-containing protein 2A). [Source:Uniprot/SWISSPROT;Acc:Q86YP4]

AGGGTCCATGTATTATTTTAACCGAGTTCATACTTTATTCTGATTTCCTCAGTTTTTTCCAGCTGTCCTTTTTCCCTTAC

GTTATATTTAATCCTTGCGT

In100 candidate1761 (in intron):

LOCUS ENSG00000167491

DESCRIPTION Transcriptional repressor p66 alpha (Hp66alpha) (GATA zinc fingr domain-containing protein 2A). [Source:Uniprot/SWISSPROT;Acc:Q86YP4]

AGTGGTAGGACTAACTTGGGGAACTGATAACTGTGTCCTTTTTCTCTGTCACAGGATTTTTCTTTTTCTTTTTTTTTTTT

TCTTTTTTTTTTTTTTTTTT

In100 candidate1762 (in intron):

LOCUS ENSG00000164162

DESCRIPTION Anaphase-promoting complex subunit 10 (APC10) (Cyclosome subuni

10). [Source:Uniprot/SWISSPROT;Acc:Q9UM13]

AAATTTGTGACCCATGTGGTAACAATTGTATATATTTATTTCTATACACTAATCTTGGCCCTCAGTTATTTATTTTGTCA

TATTATGGCATATTTCTTTT

In100 candidate1763 (in intron):

LOCUS ENSG00000110436

DESCRIPTION Excitatory amino acid transporter 2 (Sodium-dependent

glutamateaspartate transporter 2). [Source:Uniprot/SWISSPROT;Acc:P43004]

ATTCCCTTTAAATCACTTCCTGGATATTTGCATAGCACATGTATCCTGATAACTCCCAAATGTATGTTTCTATTTCAGGC

CTTTTTTCTAGGTTCTAGAT

In100 candidate1764 (in intron):

LOCUS ENSG00000149090

DESCRIPTION regeneration associated muscle protease isoform b

[Source:RefSe_peptide;Acc:NP_001001991]

GAGTAGTGTTAGATAACAACATTATCTGTATTCTTTATAAATGTGTTTCTATTGTCTGTATTTCTCTTGATTTTCAGTCT

TGCCTTCTGTCTTAGTCAGT

In100 candidate1765 (in intron):

LOCUS ENSG00000126858

DESCRIPTION Mitochondrial Rho GTPase 1 (EC 3.6.5.-) (MIRO-1) (hMiro-1)

(Rashomolog gene family member T1) (Rac-GTP-binding protein-like protein).

[Souce:Uniprot/SWISSPROT;Acc:Q8IXI2]

AGTAGTACATTGAACTTCCCTAAATCCTTCACTTAAATTTACTAGGTAACGTTTTGCCACGTTTGCCTTATCTATCAGTT

TTTCTGTCTGTTCATACACA

In100 candidate1766 (in intron):

LOCUS ENSG00000126858

DESCRIPTION Mitochondrial Rho GTPase 1 (EC 3.6.5.-) (MIRO-1) (hMiro-1)

(Rashomolog gene family member T1) (Rac-GTP-binding protein-like protein).

[Souce:Uniprot/SWISSPROT;Acc:Q8IXI2]

TAAGTCCTTATGTGTCTGACCTTTGATCCTCTTTCTAACCCCATTTTATACCTTTTTCTCTTTACTCAGTACTCTGTAGC

CATTCTGGCATTTTCCTGTT

In100 candidate1767 (in intron):

LOCUS ENSG00000163935

DESCRIPTION Scm-like with four MBT domains protein 1 (Renal ubiquitous protin

1). [Source:Uniprot/SWISSPROT;Acc:Q9UHJ3]

TAAAATAAAATAAAATAAAATAAAATAACTTCAACCTGATATTACTAATTCTTTCTCTTTTTCCATATATATAGCCAGTT

TTATTACTTTTTAATTTATT

In100 candidate1768 (in intron):

LOCUS ENSG00000166326

DESCRIPTION Tripartite motif-containing protein 44 (Protein DIPB).

[Source:niprot/SWISSPROT;Acc:Q96DX7]

ACTTTAATGGAAAATCTTATATTTAGTTGCAACAAGTTTATACTTGGTAACAAAAATGGCATGTTTCTTTGCTTACAGTT

AACTGTTTTTCTCCCATGGT

In100 candidate1769 (in intron):

LOCUS ENSG00000170365

DESCRIPTION Mothers against decapentaplegic homolog 1 (SMAD 1) (Mothers aganst DPP homolog 1) (Mad-related protein 1) (Transforming growth factor-betsignaling protein 1) (BSP-1) (hSMAD1) (JV4-1). [Source:Uniprot/SWISSPROT;Ac:Q15797]

AGATAAATTCTGTCCCACAAAGCACCTCATATTTTGTATAATTAACTATCTTCATGGCATTTGCATTTTCTACAGTTCAC
TGATTTTACCTCTTCAGCTT

In100 candidate1770 (in intron):

LOCUS ENSG00000179241

DESCRIPTION low density lipoprotein receptor class A domain containing 3 [Surce:RefSeq_peptide;Acc:NP_777562]

CTGTAGATAACCAATCTCTTTAGTTTCTGGTTTATCCTCCTGTATTTCTTTTGCACAGATAAGCCAATACATGTATGTTT
TCTTATACCCCTTCTCTTCT

In100 candidate1771 (in intron):

LOCUS ENSG00000179241

DESCRIPTION low density lipoprotein receptor class A domain containing 3 [Surce:RefSeq_peptide;Acc:NP_777562]

TAACCTCTCGTCCATATTTTCTCTTTTTGTGTCTCTTTCTGCTGCGTTCTGAATAATTCTTCTTGAGCTATCATTCAGTT

CATTTATTCTTTTTTCATCT

In100 candidate1772 (in intron):

LOCUS ENSG00000120519

DESCRIPTION Sodium/bile acid cotransporter 7 (Na(+)/bile acid cotransporter7)

(Solute carrier family 10 member 7). [Source:Uniprot/SWISSPROT;Acc:Q0GE1]

TTTATTGCATTATTTATAAACACTAATGCTTGATAACTGATTTGAAGTAATTCTAACTCCTATTTTCTTGTTTTACAGCT

TGGTTCATCTTCTTCTGTGC

In100 candidate1773 (in intron):

LOCUS ENSG00000145808

DESCRIPTION ADAMTS-19 precursor (EC 3.4.24.-) (A disintegrin and

metalloproeinase with thrombospondin motifs 19) (ADAM-TS 19) (ADAM-TS19).

[Source:Unirot/SWISSPROT;Acc:Q8TE59]

AGAGTGAGACTCCGTCTCAAAAAAAAAAAAAAGTTAACTTCTGATATTGCTCCTCAACTATGTTCTTTTGACAGTTTTTA

CCATTTCATTATTTGCCATC

In100 candidate1774 (in intron):

LOCUS ENSG00000179832

DESCRIPTION CDNA FLJ35542 fis, clone SPLEN2002917.

[Source:Uniprot/SPTREMBLAcc:Q8NAD1]

AGAAGTTCAACATTTCATGGGATTAACCATGCTCAACTTCCCTTTGTTAATTCATCAATCACATTTATGCTTTTCAGCGT

TCACTTTGTTTTCCCCATAT

In100 candidate1775 (in intron):

LOCUS ENSG00000137473

DESCRIPTION NYD-SP14 protein [Source:RefSeq_peptide;Acc:NP_114162]

CATCATATCTCATTCTTCCCTTTAACTCCTGACATTTTATCTTGGTAATCTCTCTGCTCCATTGTTTATGTCCTCCAGGT

TCTCTCTGTAGTTCTACGTA

In100 candidate1776 (in intron):

LOCUS ENSG00000137473

DESCRIPTION NYD-SP14 protein [Source:RefSeq_peptide;Acc:NP_114162]

TTGCATTTCCCTGTTGGCCCGTAACATTGAGCATTTTTTTTCATGTGCTTCTTGGCCATTTGTATATCTTCTCGGCAGAC

GTTTTTATTCAGATTCTTTG

In100 candidate1777 (in intron):

LOCUS ENSG00000198108

DESCRIPTION Chondroitin sulfate synthase 3 (EC 2.4.1.175) (Glucuronosyl-

Ncetylgalactosaminyl-

proteoglycan 4-beta-N- acetylgalactosaminyltransferase I)

(Chondroitin synthase 2) (N- acetylgalactosaminyl-proteoglycan 3-

betaglcuronosyltransferase

II) (EC 2.4.1.226) [Source:Uniprot/SWISSPROT;Acc:Q70JA]

AAATACTTGTTATTTTGATTACCACATCTCTAATAACTTCCTTGTGTTTTTTCTTCATCTTTCAGTTTTAGTCATTTTCT

ATTGACTTAGTTAACTTTCC

In100 candidate1778 (in intron):

LOCUS ENSG00000198108

DESCRIPTION Chondroitin sulfate synthase 3 (EC 2.4.1.175) (Glucuronosyl-

Ncetylgalactosaminyl-

proteoglycan 4-beta-N- acetylgalactosaminyltransferase I)

(Chondroitin synthase 2) (N- acetylgalactosaminyl-proteoglycan 3-

betaglcuronosyltransferase

II) (EC 2.4.1.226) [Source:Uniprot/SWISSPROT;Acc:Q70JA]

TGTCTGTCATTAACTCTTTATTTCTATTTCACTTTCCTAATTTCTGCCGTTCTATCCCTTAATAATAGCTTTCCACAGTG

CCTATTTTTTTCTGATTTTA

In100 candidate1779 (in intron):

LOCUS ENSG00000197124

DESCRIPTION Zinc finger protein 682. [Source:Uniprot/SWISSPROT;Acc:O95780]

EXON

AGTTGTGTTTGGTTCTTTCAAAGGAACTAGTGAGATCCTTTTGAGGTAACGAAACACTCTGGCTTTCTGTATTGCCAGCT

TTCTTTCGCTTTCACATCTG

In100 candidate1780 (in intron):

LOCUS ENSG00000158985

DESCRIPTION CDC42 small effector 2

[Source:RefSeq_peptide;Acc:NP_001033791]EXON

AGCATTTGATGTGGTTGATAACATCATGTTTGAAATCTTCCCTTTGTTTCTGGCATATTACTGCCTGTTTTCAGTTTCTT

TTGTTGGTTCCTTTTCATTG

In100 candidate1781 (in intron):

LOCUS ENSG00000164168

DESCRIPTION transmembrane protein 34 [Source:RefSeq_peptide;Acc:NP_060711]

EXON

TTTTTATTTTTATTTTTATTTTGCTTAGTATTCTGTTATGTGGATATAACTTTTTACTAATCTCCTTTTGATATTCAGGT

TGTTCCTAATTTTTTTACCA

In100 candidate1782 (in intron):

LOCUS ENSG00000163798

DESCRIPTION Kanadaptin (Kidney anion exchanger adapter protein) (Solute

carier family 4 anion exchanger member 1 adapter protein) (Lung cancer oncogen 3

protein). [Source:Uniprot/SWISSPROT;Acc:Q9BWU0]

AGAACTGTCACCTGGGACTTAACTTTCCTATCATCCTTAAGATTTCTTTGTCCTTTCTCCAGGGTTGACTTTTCTGTTTC

TTGATTCTCATATTCTCATA

In100 candidate1783 (in intron):

LOCUS ENSG00000158987

DESCRIPTION Rap guanine nucleotide exchange factor 6 (PDZ

domaincontainingguanine

nucleotide exchange factor 2) (PDZ-GEF2) (RA-GEF-2).

[Source:Unipro/SWISSPROT;Acc:Q8TEU7]

CACCTGCTTAACTGATTTCATTGATCACTTTTATTTCATTTGCACTGTATATCCCCATTATTTCAACTCATTTCACAGTT

GTCTTTGGTACTTCTTTTAG

In100 candidate1784 (in intron):

LOCUS ENSG00000149084

DESCRIPTION Estradiol 17-beta-dehydrogenase 12 (EC 1.1.1.62) (17-beta-HSD 1)

(17- beta-hydroxysteroid dehydrogenase 12) (3-ketoacyl-CoA reductase) (EC1.3.1.-

) (KAR). [Source:Uniprot/SWISSPROT;Acc:Q53GQ0]

GTTGTCTTAACCCACTGATGCTGTCTTTAGTTTTTATTTGAGTTTCTTTTCTCTCTGTGTTTCATTTTTGGACAGTGTTT

ATTTATATGCCTTCACATTC

In100 candidate1785 (in intron):

LOCUS ENSG00000151623

DESCRIPTION Mineralocorticoid receptor (MR).

[Source:Uniprot/SWISSPROT;Acc:08235]

TCTTATTGAAAGTGGTCATCCCCTGCCTTCTAACACATCACCTTCTTTGTCTCTCTTCATAGCAGTACCTGTCAGAATGA

TCTTTCTTTACTTCATTTCT

In100 candidate1786 (in intron):

LOCUS ENSG00000151623

DESCRIPTION Mineralocorticoid receptor (MR).

[Source:Uniprot/SWISSPROT;Acc:08235]

TTAAATAACGTTTCCTTGTGGTTTTGCTGTATGTGATTTTTCCCCCTCAGCTCTGTTTCCTTCCCGTTCCTTCTAACCTC

ATCTCTGATTTCATTGTGTT

In100 candidate1787 (in intron):

LOCUS ENSG00000157445

DESCRIPTION calcium channel, voltage-dependent, alpha 2/delta 3 subunit

[Sorce:RefSeq_peptide;Acc:NP_060868]

TCTGAGCCTTTTTTGCTCATTTTTTTTTTCTTTTCCTTTAACTTGTTTTTTTTCTCCTTAAAATTTCTTTTCTGCCAGAT

ACATTTCTACTTTGCCTTTT

In100 candidate1788 (in intron):

LOCUS ENSG00000157445

DESCRIPTION calcium channel, voltage-dependent, alpha 2/delta 3 subunit

[Sorce:RefSeq_peptide;Acc:NP_060868]

CAAATGAGACTGAGATTTTCTTCACCTTTAACATTTGAGGATGTTTAGCTCTTGTTCAGCCCTCACCTATTAAAAGTTCT

ATTTGTTTTGTTGTCTGTTT

In100 candidate1789 (in intron):

LOCUS ENSG00000157445

DESCRIPTION calcium channel, voltage-dependent, alpha 2/delta 3 subunit

[Sorce:RefSeq_peptide;Acc:NP_060868]

ACTGTTGCGCATGAATAAATGCTCGATAAATGGCTATCACTCCTGTCAATAACAATGACTCTCTCTTAGTCCTATCAGCT

AAGTATTGTTATCCTCTTTT

In100 candidate1790 (in intron):

LOCUS ENSG00000157423

DESCRIPTION hydrocephalus inducing isoform b

[Source:RefSeq_peptide;Acc:NP_60028]

TATAATGATGGCAATATGGTGACACTAAGTTAATTCTGACTAACATCTATTTCATTCTATTTGCCACTTTTTATACAGTA

TTTCATTTTCTACAATTCTG

In100 candidate1791 (in intron):

LOCUS ENSG00000151348

DESCRIPTION Exostosin-2 (EC 2.4.1.224) (EC 2.4.1.225) (Glucuronosyl-

Nacetlglucosaminyl-

proteoglycan/N-acetylglucosaminyl-proteoglycan 4- alpha-

Naceylglucosaminyltransferase)

(Putative tumor suppressor protein EXT2) (Multipe

exostoses protein 2). [Source:Uniprot/SWISSPROT;Acc:Q93063]

ACTGCTTTTAACTCTAACTTTTCTCTGATACTTTCTTCCTATTTCCCCATTAATGCTGTTTTTAAATTTCTTACTCAGAA

ACCTCTTTTTACTTCAAGTT

In100 candidate1792 (in intron):

LOCUS ENSG00000158467

DESCRIPTION Putative adenosylhomocysteinase 3 (EC 3.3.1.1) (S-adenosyl-

Lhmocysteine

hydrolase 3) (AdoHcyase 3). [Source:Uniprot/SWISSPROT;Acc:Q96HN2

AACTTTGTTAATTCCCTTGTCTGCTAATTCTAACATATATGTCAATTTTGTTAGCTTCAGTTGATTGATTTTTTTCTTCT

TCATGGGTCATATTTTCTTT

In100 candidate1793 (in intron):

LOCUS ENSG00000158019

DESCRIPTION Protein BRE (Brain and reproductive organ-expressed protein)

(BCA1/BRCA2-containing complex subunit 45). [Source:Uniprot/SWISSPROT;Acc:Q9NR7]

TTTCTCTGTCTTTTTTTGGATGAGCTCTCACTTGCACTTAACAATTTCCCCCTGTGTTTTTCTAGTTCTTTCTTCCAGTT

CTCATTCATTTTTTCTTCAC

In100 candidate1794 (in intron):

LOCUS ENSG00000158019

DESCRIPTION Protein BRE (Brain and reproductive organ-expressed protein) (BCA1/BRCA2-containing complex subunit 45). [Source:Uniprot/SWISSPROT;Acc:Q9NR7]

CTGTTTCCCAAACTGGATTATTTGAAACAAATTCCATACATTTAAATTAACTGGTAAATACTTTAGCTCCTGTTCAGTT

TTTAATGCCATGCTGCCTTT

In100 candidate1795 (in intron):

LOCUS ENSG00000164398

DESCRIPTION Long-chain-fatty-acid--CoA ligase 6 (EC 6.2.1.3) (Long-chain acl-CoA synthetase 6) (LACS 6). [Source:Uniprot/SWISSPROT;Acc:Q9UKU0]

AACCCCTATGGATAACTGGTCTTTTTAGTTTTTGGCTTATCCTTCCTGTGTTTCTTTTGTACAAATCAGGAGATATCCAT

GTATTTCTTTATATCCTCTT

In100 candidate1796 (in intron):

LOCUS ENSG00000139746

DESCRIPTION RNA-binding protein 26 (RNA-binding motif protein 26) (CTCL tumr antigen se70-2). [Source:Uniprot/SWISSPROT;Acc:Q5T8P6]

GATTTATCATAGTTTTTTTTTCTTAACTGTTATTGACTATTTTTAATTTTTATTTTTTAATTCAGTGGCCTCTCAGGATT

ATTACCTGCATTTTTTTTTC

In100 candidate1797 (in intron):

LOCUS ENSG00000198589

DESCRIPTION Lipopolysaccharide-responsive and beige-like anchor protein (CD4-like protein) (Beige-like protein). [Source:Uniprot/SWISSPROT;Acc:P50851]EXON

TCTTTCTAGGATTATTGTAACACCCTTCAAAACTGTTCTTGTTCTCTGCCAGTCATTCTATACCTCCATATATTTTCCCA

CATGATATTTATACTTATCT

In100 candidate1798 (in intron):

LOCUS ENSG00000198589

DESCRIPTION Lipopolysaccharide-responsive and beige-like anchor protein (CD4-

like protein) (Beige-like protein). [Source:Uniprot/SWISSPROT;Acc:P50851]EXON

TTTATATTAATGGTAGGTTTTGAAATGGTAGTATGAGTCTTCTAACTTTGTTCTTCTTCATTTTTTTTTTTGAGACAGAT

TTTTGTTCTTGTCACCCTGG

In100 candidate1799 (in intron):

LOCUS ENSG00000198589

DESCRIPTION Lipopolysaccharide-responsive and beige-like anchor protein (CD4-

like protein) (Beige-like protein). [Source:Uniprot/SWISSPROT;Acc:P50851]EXON

AAACTGATTATATCCAAAATTTTTGACATTAAGTATTAACAATTAGTCTCATTTTTACCATTAGTTTCTATTTACAGGCA

TGCCTCATTTTACTGTGCTT

In100 candidate1800 (in intron):

LOCUS ENSG00000198589

DESCRIPTION Lipopolysaccharide-responsive and beige-like anchor protein (CD4-

like protein) (Beige-like protein). [Source:Uniprot/SWISSPROT;Acc:P50851]EXON

TCATGAACATGGTATACTTTTTTCTTTAAGTTTTAACTTTTTTTTTTTTTTTTACCATTCTTTCTGTTTGACATGCAGAA

TTCTAGCACATTTTTGTTTC

In100 candidate1801 (in intron):

LOCUS ENSG00000198589

DESCRIPTION Lipopolysaccharide-responsive and beige-like anchor protein (CD4-

like protein) (Beige-like protein). [Source:Uniprot/SWISSPROT;Acc:P50851]EXON

TAACTGTTAACTTTATCTTCTAAAGTGTATTTTTTATTTTCTATTTAATTTCTGTTCTTATCCTTATTTAACTTTCAGTG

TTCACTGGCTGTTCTTTTTC

In100 candidate1802 (in intron):

LOCUS ENSG00000198589

DESCRIPTION Lipopolysaccharide-responsive and beige-like anchor protein (CD4-

like protein) (Beige-like protein). [Source:Uniprot/SWISSPROT;Acc:P50851]EXON

ATAACCTTGTTTAAATGCTGCTGGAATTCTCAAGCTTCCATTTTCTCTCTTGCTTTTTGATTCTACCAGACTTTTTTGCT

AGAATGGTCTTTATTGTCTC

In100 candidate1803 (in intron):

LOCUS ENSG00000198589

DESCRIPTION Lipopolysaccharide-responsive and beige-like anchor protein (CD4-

like protein) (Beige-like protein). [Source:Uniprot/SWISSPROT;Acc:P50851]EXON

CCTTGCCTTTGCTTTAAAGGTTTCCTTTATTCCCACCCAAGACTAACTTCTAAAATTCCTCTTTATACAATTCAGCTTTT

CATCTCCCTAGCATAGTGTT

In100 candidate1804 (in intron):

LOCUS ENSG00000198589

DESCRIPTION Lipopolysaccharide-responsive and beige-like anchor protein (CD4-like protein) (Beige-like protein). [Source:Uniprot/SWISSPROT;Acc:P50851]EXON

TTCTCTGAGATTCTAGGCTTTGAATGTAATAACTAATTCTGCCATCTGTTTCTCACTACTCTTTGTATTGATCAGTTCTA
TTCACTGATGTTTACATTTC

In100 candidate1805 (in intron):

LOCUS ENSG00000132141

DESCRIPTION T-complex protein 1 subunit zeta-2 (TCP-1-zeta-2) (CCT-zeta-2) TCP-1- zeta-like) (CCT-zeta-like) (Testis-specific Tcp20) (Testis-specific rotein TSA303). [Source:Uniprot/SWISSPROT;Acc:Q92526]

GTTACTTTCTCTTTATAACTTTTGAATTTTTTTTTCTAAAGGTGTTTTAAAATCTCTTTTTCCTTCCAGATGTTTGGTGT
AGACTACTTCTATCTTTTTT

In100 candidate1806 (in intron):

LOCUS ENSG00000106459

DESCRIPTION Nuclear respiratory factor 1 (NRF-1) (Alpha palindromicbindingprotein)
(Alpha-pal). [Source:Uniprot/SWISSPROT;Acc:Q16656]

ATTTTTGTGCCTTGTGTATCATATTTCCTTCCTTATTAACTAGAGGGTGGATTACCCACTTTCTTGGGTTTTCAGTTAGT
ACTTTTATCCATCAGTTTTC

In100 candidate1807 (in intron):

LOCUS ENSG00000163947

DESCRIPTION Rho guanine nucleotide exchange factor 3 (Exchange factor foundin platelets and leukemic and neuronal tissues) (XPLN).
[Source:Uniprot/SWISPROT;Acc:Q9NR81]

AACACGTTATTTCCACAACTACCACACCAAAAAGCCTTTAACTTGGTTATTTTGTGCTTTATCCTGTTTTTCAGTCTTCT

TAAATATACATTTTATTCCC

In100 candidate1808 (in intron):

LOCUS ENSG00000144730

DESCRIPTION Interleukin-17 receptor D precursor (IL-17 receptor D) (IL-17RD (Interleukin-17D receptor) (IL-17D receptor) (IL17Rhom) (Interleukin- 17 rceptor-like protein) (Sef homolog) (hSef). [Source:Uniprot/SWISSPROT;Acc:Q8FM7]

CCACCCTCTAGTCTCTAGTTCTTCCTGCATATGTGGCCCTAACACTGTTTTGTTGTTATCATTCCTTTCCTGGTTCAGTT

TCCTTTAATGTTTTTTGTTT

In100 candidate1809 (in intron):

LOCUS ENSG00000144730

DESCRIPTION Interleukin-17 receptor D precursor (IL-17 receptor D) (IL-17RD (Interleukin-17D receptor) (IL-17D receptor) (IL17Rhom) (Interleukin- 17 rceptor-like protein) (Sef homolog) (hSef). [Source:Uniprot/SWISSPROT;Acc:Q8FM7]

TACTAACCTTGGCTTCTAGATTATAGTTTTCTTTTCCTCCCATGATGTTACTCTGCTTTCCTCTTCATTCTAGCCCAGTT

ATCCTTCTCCTTGTTAAGCT

In100 candidate1810 (in intron):

LOCUS ENSG00000128607

DESCRIPTION

TTCTTGTTTATAACCGGCTCTTGGATTTTTAAAACTTAGTTCCAATATTTTCTTTTTTTATTAATTTGTTTCTTTCAGTT

TTCTTTTTTTTTTTTTTTTT

In100 candidate1811 (in intron):

LOCUS ENSG00000146842

DESCRIPTION CDNA FLJ14803 fis, clone NT2RP4001442.

[Source:Uniprot/SPTREMBLAcc:Q96SK2]

AGCTCTTTCTTTAGTAATGTCTAAACTGTTATTTAACTAATTCATTGAGTTTTTTAATTTCAGCCACTTGTTCTCATTTT

ATAGTTTTATTTGGTCCTTT

In100 candidate1812 (in intron):

LOCUS ENSG00000166747

DESCRIPTION AP-1 complex subunit gamma-1 (Adapter-related protein complex

1gamma- 1 subunit) (Gamma-adaptin) (Adaptor protein complex AP-1 gamma-1 subnit)

(Golgi adaptor HA1/AP1 adaptin subunit gamma-1) (Clathrin assembly proein

complex 1 gamma-1 large chain). [Source:Uniprot/SWISSPROT;Acc:O43747]

TGTTTACTTTAAAATAAACTCTTTAAAGTTAACATATGGTAAAATTGACTCTTCTGGTTTATTGTTTTACAGTTCTGTGA

GGATTTTTTGTTTTTGTTTT

In100 candidate1813 (in intron):

LOCUS ENSG00000168387

DESCRIPTION Ankyrin repeat and SOCS box protein 14 (ASB-14).

[Source:Unipro/SWISSPROT;Acc:Q8WXK2]

AGTCATTGCCTCCTATGAAAGTTCATCCCGGATTAACTACTGCTACCATTACCCTTTCCTTTTGATTTATATCAGATAAC

AGTTTTTTCTCCTCAGTTTT

In100 candidate1814 (in intron):

LOCUS ENSG00000163669

DESCRIPTION CDNA FLJ40427 fis, clone TESTI2039113.

[Source:Uniprot/SPTREMBLAcc:Q8N7R9]

TAACTCTGCTGATTATTTCTTTTGCTGTGCAGGAGGTTTTTAGTTTAATTAAGTCCCATCTATTTATCTTTGTTTTTGTT

GCATTTGTTTGTTGTTCTTT

In100 candidate1815 (in intron):

LOCUS ENSG00000163669

DESCRIPTION CDNA FLJ40427 fis, clone TESTI2039113.

[Source:Uniprot/SPTREMBLAcc:Q8N7R9]

TGTTTTCCTCTTCTATTTTTCTGTCTTCTCTGGTTTTAACTATTTTGTATGATTCCATTTTTTCTCTTCTCTTAGCAGAT

CAATTATACTTACTTTTTTA

In100 candidate1816 (in intron):

LOCUS ENSG00000109670

DESCRIPTION F-box/WD repeat protein 7 (F-box and WD-40 domain protein 7)

(Fbox protein FBX30) (hCdc4) (Archipelago homolog) (hAgo) (SEL-10).

[Source:Uiprot/SWISSPROT;Acc:Q969H0]

TCCTGATTAATATAACTAGAATTAGTTATTTTTTTCCACTGGTTACCCACAGTATCTCTGTGGCTTTTATAGCACTTAAC

ATCCTTATTTGTATTTTTCT

In100 candidate1817 (in intron):

LOCUS ENSG00000109670

DESCRIPTION F-box/WD repeat protein 7 (F-box and WD-40 domain protein 7)

(Fbox protein FBX30) (hCdc4) (Archipelago homolog) (hAgo) (SEL-10).

[Source:Uiprot/SWISSPROT;Acc:Q969H0]

GAACTCATTCAAATCATTGATTTTCTGGTTAACTTTGTTAGTAGTTTGTTTTTTTCTGAGTCTTTTGTTTTAATCCAGAT

AGTCAGTCTTTTTCTCCTTT

In100 candidate1818 (in intron):

LOCUS ENSG00000109670

DESCRIPTION F-box/WD repeat protein 7 (F-box and WD-40 domain protein 7)

(Fbox protein FBX30) (hCdc4) (Archipelago homolog) (hAgo) (SEL-10).

[Source:Uiprot/SWISSPROT;Acc:Q969H0]

CTACAACATTAATTCTAACACCAAGTAACATTATAACCCACTTAAAACTGTTCTTTTTTCTTTCTTTTTTTAGTACAGAC

AGAACTTTTTTTTTTTTAAT

In100 candidate1819 (in intron):

LOCUS ENSG00000174844

DESCRIPTION CDNA FLJ44290 fis, clone TRACH2019473.

[Source:Uniprot/SPTREMBLAcc:Q6ZTR8]

CTGACCATTTATTTCCTTTCTAACATTCCTTTATGTTTATTCATCTGGTTCTCTATTATTTCTCTGTCATCTCCACAGGT

TCCTCTTTCTTGACTGGCTG

In100 candidate1820 (in intron):

LOCUS ENSG00000187008

DESCRIPTION Polycystic kidney disease 1-like protein 3.

[Source:Uniprot/SPTEMBL;Acc:Q7Z443]

ATTCCATTTTATATTAACTAAAGAAAGTTGACATACTGATATTATTTCTATTGTTTGTTCTTATCTTTTTACTTTCAGCC

CTTTTTATGGTTTTGCTTAG

In100 candidate1821 (in intron):

LOCUS ENSG00000187008

DESCRIPTION Polycystic kidney disease 1-like protein 3.

[Source:Uniprot/SPTEMBL;Acc:Q7Z443]

TGTAACTCCTGGAGTTCTGATTTTCTTATATTTTTGCTTCTGTTTTCCATCTTTTTCATCTAATATCTGCAGTATTTAAT

TTTTATCTTCTAAACTATTT

In100 candidate1822 (in intron):

LOCUS ENSG00000135365

DESCRIPTION PHD finger protein 21A (BRAF35-HDAC complex protein BHC80)

(BHC0a). [Source:Uniprot/SWISSPROT;Acc:Q96BD5]

GAGCTTTTGTACATGGGCTTTTTTTATAGGCCTAAGAATTCTTTAACGTTTTGATCACATTAATTTTCTCAGGTGTATGT

TTTTGCTGCTTTGCGATTTT

In100 candidate1823 (in intron):

LOCUS ENSG00000170006

DESCRIPTION transmembrane protein 154 (TMEM154), mRNA

[Source:RefSeq_dna;Ac:NM_152680]

TATGTGTTAGAGGGTAGCCCTCTAACACATCAAGTAAGGTCTTTCATCTACTTTTATTGCACCTTGTTACTTTTACAGAT

TTTTTTTTTGCATTGATTGT

In100 candidate1824 (in exon):

LOCUS ENSG00000170006

DESCRIPTION transmembrane protein 154 (TMEM154), mRNA

[Source:RefSeq_dna;Ac:NM_152680]

TATTTATGCCAAAATTGCATACAATTGTTTTCTGAATCATAACTTGTCTATTTTTCTGCCTATGTGTGCTACTTTCAGTT

TGTTTCTCATCAACATTTTG

In100 candidate1825 (in intron):

LOCUS ENSG00000164144

DESCRIPTION Arfaptin-1 (ADP-ribosylation factor-interacting protein 1).

[Sorce:Uniprot/SWISSPROT;Acc:P53367]

GAAATGTGTATTTTCTTATATCCCCCTTCATTCTTACATGAACATAACATACCATAGGGATTTTTTTGCTTTTTTCAGAC

TTTGCTTTTTTCAGTTAACA

In100 candidate1826 (in intron):

LOCUS ENSG00000158623

DESCRIPTION testis specific, 13 [Source:RefSeq_peptide;Acc:NP_443165]

AGGAACATTATCTCTACTAACACCATTCCTATTTACTTCCTTAATCTCTTTCTCTGTATCAGGTCCTTATATGTAAGTGT

CTTCTGTCTTTACCTCCACA

In100 candidate1827 (in intron):

LOCUS ENSG00000128585

DESCRIPTION Muskelin. [Source:Uniprot/SWISSPROT;Acc:Q9UL63]

TGACATAACTTCCATCATTTTTGTTTTGTTTTGTTTTTCATTTTAGTGCTTTCTTAATTTCTGGTATTACAGGATGCTCC

AGGCTCATCTTGTTTATTTT

In100 candidate1828 (in intron):

LOCUS ENSG00000128585

DESCRIPTION Muskelin. [Source:Uniprot/SWISSPROT;Acc:Q9UL63]

AGAAGTCTAACAACTTTCATGCCTTGGCATTCCCAATCTTTTATTTCAGCTAGCTATTGCTGTCGATTTTTTGTTCTGTT

CTCAGTCCTTTGGCTATTTC

In100 candidate1829 (in intron):

LOCUS ENSG00000146938

DESCRIPTION Neuroligin-4, X-linked precursor (Neuroligin X) (HNLX).

[SourceUniprot/SWISSPROT;Acc:Q8N0W4]

TTGTTTTATGAGTTATGATGAATGCACCTTAGCTGTTTCTAACCCCGCTCCCATTCCCTGTTTTTATTTGTAAGTCAGAA

CCCAGCATTTTTACATTTTT

In100 candidate1830 (in intron):

LOCUS ENSG00000146938

DESCRIPTION Neuroligin-4, X-linked precursor (Neuroligin X) (HNLX).

[SourceUniprot/SWISSPROT;Acc:Q8N0W4]

AGCTAACCTGGTTGCTAATCTCTGCAAGCTCGTACTTTTTCTGCAGCACGTGATTCTGTTCTCATTTACTCTTGTAATCC

TTCTGTTTCCTTCTGACCAG

In100 candidate1831 (in intron):

LOCUS ENSG00000121211

DESCRIPTION GAJ protein [Source:RefSeq_peptide;Acc:NP_115493]

AAGGAAAAGTAAACTCTGGGCATAACCTGAAGCTGTTCTATTTCCTCTTCAGTTGACAGTAGCAACTACTACTTCTTTAT

TTGTTCCTTTTGTTTGTTTT

In100 candidate1832 (in intron):

LOCUS ENSG00000121211

DESCRIPTION GAJ protein [Source:RefSeq_peptide;Acc:NP_115493]

ATCATGAAAAATCTGAGTTTCACGATAACCAAATCGGACCCTTCATTTACTCTTACTTGCCTTTTTTCCTGATTTCAGTT

TTGTGTGATTCTTGTACTTT

In100 candidate1833 (in intron):

LOCUS ENSG00000179399

DESCRIPTION Glypican-5 precursor. [Source:Uniprot/SWISSPROT;Acc:P78333]

TGTGCTCTTGATCTCTCTTTCTTCTTTACCTTTATAACCTTCTTGTGTTGTTTATCCCTTGTGTCATTGCATTTTCAGTT

TCTTTCATTCTTTTGGCTCT

In100 candidate1834 (in intron):

LOCUS ENSG00000179399

DESCRIPTION Glypican-5 precursor. [Source:Uniprot/SWISSPROT;Acc:P78333]

GTTTTTTGTTTTTGTTTTTTTAAATGAATATCCCTTAACAATAAGAGATATATGTTCTTCCCTGACATTTTACAGCTTTA

TTCTCTTAATCCAGTAACTT

In100 candidate1835 (in intron):

LOCUS ENSG00000179399

DESCRIPTION Glypican-5 precursor. [Source:Uniprot/SWISSPROT;Acc:P78333]

TTTTCCCCTTATGATCTAAGTAATAAATCTCTTAACTGGTGCGAACATTTAGATATCTAATCTTGTTCCCTCATTCAGTT

TTATCTTTTAATATTATTTT

In100 candidate1836 (in intron):

LOCUS ENSG00000179399

DESCRIPTION Glypican-5 precursor. [Source:Uniprot/SWISSPROT;Acc:P78333]

AGAAATCTCTTAATATTCCCAAGTTCCTAACCATTTTTTTCTCCCTGTTTTTCTATATTCATTTCACAGAGGTCCCTTCT

GTTAAATACATTGCTTTTTC

In100 candidate1837 (in intron):

LOCUS ENSG00000179399

DESCRIPTION Glypican-5 precursor. [Source:Uniprot/SWISSPROT;Acc:P78333]

CCTTCAAATTCACTCATTCTTTAGTTTTCATAGTGCTTTAACTTTGAAGTTTTCTTCCTTCGTTCTTTTCACTTTCAGCT

CTTTTTGGCTTTTTATTACT

In100 candidate1838 (in intron):

LOCUS ENSG00000179399

DESCRIPTION Glypican-5 precursor. [Source:Uniprot/SWISSPROT;Acc:P78333]

AAAAGCTCAAATTCTGAAGAATTCAAAAGATGTCATTAAAAATACGTAACTAGAAATCAAGATTTTCAAATTCTTCAGTA

TTTTTAATAGGTCCTTTTTT

In100 candidate1839 (in intron):

LOCUS ENSG00000179399

DESCRIPTION Glypican-5 precursor. [Source:Uniprot/SWISSPROT;Acc:P78333]

CTTTGGGGATAGATACAATTTAGCCAAATTATTTGCCACTTTGTAACAAAAATGGTCTTTCTTCACTCCATTTTTCAGTA

AGATTTTCCTCATTTATCTC

In100 candidate1840 (in intron):

LOCUS ENSG00000179399

DESCRIPTION Glypican-5 precursor. [Source:Uniprot/SWISSPROT;Acc:P78333]

GTGTGATGTCAAATGGATATAACTTACAAAGTGTTTTTCTTCATTTTTTTCCCTTTATAAGTGGCACAGAAAATTTGTTT

TTACTTATGTTTTTGCATCC

In100 candidate1841 (in intron):

LOCUS ENSG00000179399

DESCRIPTION Glypican-5 precursor. [Source:Uniprot/SWISSPROT;Acc:P78333]

AAGTATCTTTTCCCTGTCATCTACCCATTAATATTTTCTAGTTAACTTGGAGGTAAATCCTTGCTTTTCATGTCACAGAA

TTCTTTAATATTTTTCTTAA

In100 candidate1842 (in intron):

LOCUS ENSG00000205045

DESCRIPTION Schlafen 5 (Fragment). [Source:Uniprot/SPTREMBL;Acc:Q6IEE8]

TTCTTACTTCTGTCTAGAAAATTCTTCCCCCCCTTCTTCACCAAATTAACTCTTCACCTCATTTGCATTCTTCAGATCTT

AACTCAAATGTTACTTTTTC

In100 candidate1843 (in intron):

LOCUS ENSG00000205045

DESCRIPTION Schlafen 5 (Fragment). [Source:Uniprot/SPTREMBL;Acc:Q6IEE8]

CCTCTAGAAGCCCTGTCCGGGCGTAACAAAGGGCTCACACTTGTTTTCTGGTCACTTTTCATCATGTCCCTTCAGCTCCT

ATCTCTGTATGGCCTGGTTT

In100 candidate1844 (in intron):

LOCUS ENSG00000110497

DESCRIPTION CDNA FLJ20294 fis, clone HEP05851.

[Source:Uniprot/SPTREMBL;AccQ9NXE7]

ATATTAATATTACATCTATATGTATTACAAACCCAACAATGTGGTGTTAACTTATTGATTAATACAATCTTTTCTCAGGC

ATCCTTCTGTTTGTTGCCTG

In100 candidate1845 (in intron):

LOCUS ENSG00000183098

DESCRIPTION Glypican-6 precursor. [Source:Uniprot/SWISSPROT;Acc:Q9Y625]

TTCTTTTGCTTGGTCTAGTCTCTTGGTAAAACTTTTCACTGCATTTTGTAACTCCCTAAATGAGTCTTTCATTTCCAGAA

GTTCTGTGTTTTGTTTTGTT

In100 candidate1846 (in intron):

LOCUS ENSG00000183098

DESCRIPTION Glypican-6 precursor. [Source:Uniprot/SWISSPROT;Acc:Q9Y625]

ACCTAATTACCATGTGATTTTGGTTTGTATTCTATGGTTAGTAACTATATGAAAACTTACTTTTATATCATTTTTCAGTT

TGAACCATCCTTTAATTTCT

In100 candidate1847 (in intron):

LOCUS ENSG00000183098

DESCRIPTION Glypican-6 precursor. [Source:Uniprot/SWISSPROT;Acc:Q9Y625]

TGATGTTGCTTTGCTAACTTTTCCATTTGTTTGGAATTTTTTTCTTTGTTCTTTTTTGCTTTCTATCAGAATTAACTTCC

GAAAGCTTTCTGCTTTTCTC

In100 candidate1848 (in intron):

LOCUS ENSG00000183098

DESCRIPTION Glypican-6 precursor. [Source:Uniprot/SWISSPROT;Acc:Q9Y625]

ATGTATTATCTTAAGGTATTCATCATTAGACTAACATTTTTCTCTAATTTTCATTATTTTTTACTGTGGCTCTTACAGTC

TATTTCCTTGTTCCAATTTT

In100 candidate1849 (in intron):

LOCUS ENSG00000183098

DESCRIPTION Glypican-6 precursor. [Source:Uniprot/SWISSPROT;Acc:Q9Y625]

CCTTAGTTTTAACGACCCCCTAATTTCTTCAAGTCTAATCTTTGCTTGGTTTTTATCTCACAGTTATATCCTCATTCTTC

AGCAATCACTCTAATTCTTT

In100 candidate1850 (in intron):

LOCUS ENSG00000183098

DESCRIPTION Glypican-6 precursor. [Source:Uniprot/SWISSPROT;Acc:Q9Y625]

TCTTTACATTGTGGTCTCATTCATGATAAATGTTGCCAATAACTTATGTATAAGTTGTCTATTTTTTAGTGTTTACAGGG

ACATTTCTTTTTTCTACATG

In100 candidate1851 (in intron):

LOCUS ENSG00000140836

DESCRIPTION Alpha-fetoprotein enhancer-binding protein (AT motif-binding fator) (AT-binding transcription factor 1). [Source:Uniprot/SWISSPROT;Acc:Q1511]

TTTGCCCAAGGAAAGTACTTTGGCTTTTAACATTTAAAGGGGATTTTTTCTTTCTTTGACGTTTCTGCCTTTCAGCCTTC

ATTCATGGAGTTTCTTGCTT

In100 candidate1852 (in intron):

LOCUS ENSG00000006125

DESCRIPTION AP-2 complex subunit beta-1 (Adapter-related protein complex 2 eta-1 subunit) (Beta-adaptin) (Plasma membrane adaptor HA2/AP2 adaptin betasubunit) (Clathrin assembly protein complex 2 beta large chain) (AP105B). [ource:Uniprot/SWISSPROT;Acc:P63010]

TAACTTCTTTGCTGCTGCTCTCCCCTTCATTTTCTCTATTTCTTATCAGACGTCTTGCTATTTGGATGTTGATTCTCCTG

GACTGACTCTGTTTTCCCCT

In100 candidate1853 (in intron):

LOCUS ENSG00000175224

DESCRIPTION Uncharacterized protein KIAA0652. [Source:Uniprot/SWISSPROT;AccO75143]

AGGTGTGCACCACCGTGCCCGACCAAAAAAAAATTTAACGTGTACCCATTATTTCCTCTTTCAGCTCTCCTTTTGTTTAT

TCTTTTCCTTAAAATCTTTT

In100 candidate1854 (in intron):

LOCUS ENSG00000115295

DESCRIPTION CAP-GLY domain-containing linker protein 4 (Restin-like

protein2). [Source:Uniprot/SWISSPROT;Acc:Q8N3C7]

ATCAATTTATAGGTAAGCATTTCTATGCTTAATTAGACTTTAACTACTTTGAAAATTCTGCTTTGTTTTACCTACCAGGT

ATTGGCCGTTTTTTTTCTGT

In100 candidate1855 (in intron):

LOCUS ENSG00000130021

DESCRIPTION Haloacid dehalogenase-like hydrolase domain-containing protein A

(GS1 protein). [Source:Uniprot/SWISSPROT;Acc:Q08623]

TGTTTCAAAATATATTAATGAACTTATCATTTCTGATTACTGATTTATTTAACCTTGTAAGGACTGTCTCCATGTCAGTT

GTCACATTTTTCTTGGTGCC

In100 candidate1856 (in exon):

LOCUS ENSG00000130021

DESCRIPTION Haloacid dehalogenase-like hydrolase domain-containing protein A

(GS1 protein). [Source:Uniprot/SWISSPROT;Acc:Q08623]

AGTCTGAGTGGGAAAGTCCTGTTTTTGATGAGCACTCCTTGTTAGCTAACATTTAAATTCTTTTTGTGACCTCAGAATGT

CTCTGGATCTTTCCTCATTG

In100 candidate1857 (in intron):

LOCUS ENSG00000106554

DESCRIPTION Coiled-coil-helix-coiled-coil-helix domain-containing protein 3

[Source:Uniprot/SWISSPROT;Acc:Q9NX63]

AGGAGTGGGGTGGTTGGTGATGTTATGTTTGGAATAACATATTAAGGATTTCTATTTTTGGATTCTTATTTACAGGTAAG

TTTTTTTTTTCTTTAATTAG

In100 candidate1858 (in intron):

LOCUS ENSG00000149488

DESCRIPTION Transmembrane channel-like protein 2 (Transmembrane cochlearexressed protein 2). [Source:Uniprot/SWISSPROT;Acc:Q8TDI7]

AGTTGTTTCTATTTGGAAACTATTATGAATAACTCTACTATAAAACATTCACTTTCAGGTCTTTTTCTTTTATATCTTTA

TTTTTATATTACTAATTTCT

In100 candidate1859 (in intron):

LOCUS ENSG00000131558

DESCRIPTION Exocyst complex component 4 (Exocyst complex component Sec8).

[ource:Uniprot/SWISSPROT;Acc:Q96A65]

AATAATAGGCTTGGAAAACTTATTTCTTAACTTGTTTCTTTAGTGCTTATATTCTTAACTATCTCTTTTCCTCTTCAGTT

TGTACTTTCACTCTATTCAT

In100 candidate1860 (in intron):

LOCUS ENSG00000131558

DESCRIPTION Exocyst complex component 4 (Exocyst complex component Sec8).

[ource:Uniprot/SWISSPROT;Acc:Q96A65]

CGTCGAAAGTCCATTTTTATATATGGTATTAACTGAGGATCTAATTTTATCTTTCTTCTATATAGTCTGTTGGCTCAGGC

TGTTTATTGAATCCTTTTTC

In100 candidate1861 (in intron):

LOCUS ENSG00000131558

DESCRIPTION Exocyst complex component 4 (Exocyst complex component Sec8).

[ource:Uniprot/SWISSPROT;Acc:Q96A65]

GTATTCATTCCCTAACTATTTTGTCTTTTAAAGATTTTTTTTCTGTTTACATATGTTGGTTTTCTTTTTATCTTTCAGTT

CTTGTTTTTTTTTTTTTTCC

In100 candidate1862 (in intron):

LOCUS ENSG00000131558

DESCRIPTION Exocyst complex component 4 (Exocyst complex component Sec8).

[ource:Uniprot/SWISSPROT;Acc:Q96A65]

TTCCATTTAACTTTTCTGTTTGTGATTTTTTTCCTCTGTTCCCTCTGAGCATTTGTACTTGCCATTCCTGTACTGCAGAT

ACACCTTTGTTTTCTTCTCT

In100 candidate1863 (in intron):

LOCUS ENSG00000131558

DESCRIPTION Exocyst complex component 4 (Exocyst complex component Sec8).

[ource:Uniprot/SWISSPROT;Acc:Q96A65]

GCTACCTCTGACTCTTTTCTATAAGTTTTAGCTAACTCTAACAAGATACTTTTTCACTCTTCTCCATATTTAGTTCAGAT

CTGTCTTTGTCTGTAGATCT

In100 candidate1864 (in intron):

LOCUS ENSG00000131558

DESCRIPTION Exocyst complex component 4 (Exocyst complex component Sec8).

[ource:Uniprot/SWISSPROT;Acc:Q96A65]

CTTTCATATATTTAATAACTATTATTAGGTTATCCTTATGCTGTCATTTCTTCAGCCACAATAATCCCAGTTCCTTTAAG

CTTTATTTTTATCCTGCTTT

In100 candidate1865 (in intron):

LOCUS ENSG00000131558

DESCRIPTION Exocyst complex component 4 (Exocyst complex component Sec8).

[ource:Uniprot/SWISSPROT;Acc:Q96A65]

AGAGTTGGGAGTCTTCTCTGACAATTAACTTTGCGTCATCTCTCATCAAATTATTTGCTTTTTTCCTGTGCAGTACCTT

TTAGATCTGCCCTTTCTTAT

In100 candidate1866 (in intron):

LOCUS ENSG00000131558

DESCRIPTION Exocyst complex component 4 (Exocyst complex component Sec8).

[ource:Uniprot/SWISSPROT;Acc:Q96A65]

AAAGAAACATTAACTTATCACTCATAGTTTTGATTTTCTTTGCATTTTACTCTTGTTATTTTATTTATTCACTAACAGTA

TTATTTTGCCTCTTCATGTC

In100 candidate1867 (in intron):

LOCUS ENSG00000131558

DESCRIPTION Exocyst complex component 4 (Exocyst complex component Sec8).

[ource:Uniprot/SWISSPROT;Acc:Q96A65]

TGGTGCTCTGTCCCAAGATCATTGCCCTCCCTGGAAGGATTTATTATTAACTGCTCTCCCTATCTCTCTCTTTTTCAGGG

TTCACTGTTTCCACTATCTT

In100 candidate1868 (in intron):

LOCUS ENSG00000163686

DESCRIPTION abhydrolase domain containing 6

[Source:RefSeq_peptide;Acc:NP_05727]

TCCCACGTACATAAGCATAACAAATATATTTTCTCTCTCACTCTCTGTTTTTCAGTGTTAGGGTATTACTCTTATCCACT

GTCTTCTGTTCCATGCTTTT

In100 candidate1869 (in intron):

LOCUS ENSG00000090863

DESCRIPTION Golgi apparatus protein 1 precursor (Golgi sialoglycoprotein

MG160) (E-selectin ligand 1) (ESL-1) (Cysteine-rich fibroblast growth factor

eceptor) (CFR-1). [Source:Uniprot/SWISSPROT;Acc:Q92896]

GTCCGTCTTCCATGTCTCTTTAACTTTTTCTTTGGTACTTTTCATTTCTTTGTACTACATTTTAGGTAACTCCTCAGTTC

TCTTTCCCAGCATATAATTT

In100 candidate1870 (in intron):

LOCUS ENSG00000155530

DESCRIPTION leucine-rich repeats and guanylate kinase domain containing

[Sorce:RefSeq_peptide;Acc:NP_653249]

TTACTCCCCTCAACTCCTGGTAACCTCGAATCTACTTTCTTTTCTCTATGAGTTTGCCTACTCTCGATATTTTCACAGTT

TTATTTTTCAATTTAACCTT

In100 candidate1871 (in intron):

LOCUS ENSG00000121297

DESCRIPTION Teashirt homolog 3 (Zinc finger protein 537).

[Source:Uniprot/SISSPROT;Acc:Q63HK5]

TTTGTGGCTGGGAAGAAGGTAACTAAATAATTAGTTTCATTTATTCAGTCATTCATTCGTTCTGTCACTCATTCATTCAT

TCATTCACTCATTCCTTTAT

In100 candidate1872 (in intron):

LOCUS ENSG00000171094

DESCRIPTION ALK tyrosine kinase receptor precursor (EC 2.7.10.1) (Anaplasti

lymphoma kinase) (CD246 antigen). [Source:Uniprot/SWISSPROT;Acc:Q9UM73]

ATTAAGCAATGACTCCCCATTCCCCCACCCTAGTAACCTCTAGTCTACTTTCTGTCTCTATTTATTTGCATGTATCAGAA

TTTCATTCTTTTCTGAGGCT

In100 candidate1873 (in intron):

LOCUS ENSG00000171094

DESCRIPTION ALK tyrosine kinase receptor precursor (EC 2.7.10.1) (Anaplasti

lymphoma kinase) (CD246 antigen). [Source:Uniprot/SWISSPROT;Acc:Q9UM73]

TCCTATTTTCTATCTCTTTATCTTATAACCCTCTGTTGACTTTTCCCATTTCTACTATAATCTTTTTAATTTCACCAGTC

TTGTTTTGCTATCATAATTT

In100 candidate1874 (in intron):

LOCUS ENSG00000011201

DESCRIPTION Anosmin-1 precursor (Kallmann syndrome protein) (Adhesion molecle- like X-linked). [Source:Uniprot/SWISSPROT;Acc:P23352]

TATGGTGTCTAACATTTTAAAAATCCTATTTGACATATTTTTCATTTCTTCCTTTTTACTTCTGGAATTTCTATGCAGCT

CTGTTCTGTAGCTTTCATTC

In100 candidate1875 (in intron):

LOCUS ENSG00000151962

DESCRIPTION

CACATATGTTAAACCTTTTTACTGTAACCTGTGTATTGATTATATTTCTTCTGTATTGTCCATCCTTTTGTATGTCAGTC

TGTATATTTTCTTACCTGTC

In100 candidate1876 (in intron):

LOCUS ENSG00000173809

DESCRIPTION ES cell associated transcript 8. [Source:Uniprot/SPTREMBL;Acc:Q87J7]

CTCCCATTCTGTAGGTTTTCTGTTAACTCTGATGATAGTTTCTTCTGCTGTGCAGAAGCTCTTTAGTTTAATTCAATCCC

ATTTGTCAATTTTTACTTTT

In100 candidate1877 (in intron):

LOCUS ENSG00000185082

DESCRIPTION limb bud and heart development homolog [Source:RefSeq_peptide;Ac:NP_112177]

TATATAATATAAATTTCTATTAATAAAAATTAAATAAAAAATAACACTTTAGCCACCATTCTTATTTTTATTGTCAGTA

GAATTTTTTTTTTTTTTTT

In100 candidate1878 (in intron):

LOCUS ENSG00000172954

DESCRIPTION lysocardiolipin acyltransferase isoform 1

[Source:RefSeq_peptid;Acc:NP_872357]

AGCCATTGGTAACATTTTGATGTACTTCTATCTAGTGCTTTTTTTGTTCAGTGATTTCACTCTCTAGTTTTTGGTCCCCT

TTTGTTCTCTTGCTTTGCTT

In100 candidate1879 (in intron):

LOCUS ENSG00000113522

DESCRIPTION DNA repair protein RAD50 (EC 3.6.-.-) (hRAD50).

[Source:UniprotSWISSPROT;Acc:Q92878]

TTCTTCCTTAGTATTTTGATTCCTTCTTGAATGTGTCTTTACTCATTTTAACTATACACATTTTATATTCTCTTTCAGAT

TGTTATTTTTTAATTTCACA

In100 candidate1880 (in intron):

LOCUS ENSG00000164116

DESCRIPTION Guanylate cyclase soluble subunit alpha-3 (EC 4.6.1.2) (GCS-alpa-

3) (Soluble guanylate cyclase large subunit) (GCS-alpha-1).

[Source:Uniprt/SWISSPROT;Acc:Q02108]

AATACTGTCTTATTTAGGCAAAATATAAATACTTAATGTTAAATACTTTTAACAACATTTTAAATTCCTTTCTTTCAGAG

ATACTTCACTTTTCTTCCAT

In100 candidate1881 (in intron):

LOCUS ENSG00000131941

DESCRIPTION Rhophilin-2 (GTP-Rho-binding protein 2) (76 kDa RhoB effector

potein) (p76RBE). [Source:Uniprot/SWISSPROT;Acc:Q8IUC4]

GGAGCTACATTTCAATCCACAATAGACATTATAATGATGATAACCATGATCATGATGTTTATTCTTTTCACCTAGCAGGT

CCTCTTTAATTTTTTTAAAA

In100 candidate1882 (in intron):

LOCUS ENSG00000152749

DESCRIPTION Integral membrane protein GPR180 precursor (Intimal thicknessrlated

receptor). [Source:Uniprot/SWISSPROT;Acc:Q86V85]

AAATAGTAACATACTAAACATTTTTCTATATCTTGTTTTTTTCTCCCTATATCTTTAATGTACCAAACATTTCAACAGCT

ATTTATTAACTTATTTTTTA

In100 candidate1883 (in intron):

LOCUS ENSG00000163689

DESCRIPTION CDNA FLJ42117 fis, clone TESTI2005376.

[Source:Uniprot/SPTREMBLAcc:Q6ZVT6]

TTATTGTGAAGAATAAAATTAAAAACTATGCTTGAGCTAACCACCTAAGTTAGTTTCATTTGGTATATTTCCTTTCAGTC

TTTTTTGCCATTCACACTTT

In100 candidate1884 (in intron):

LOCUS ENSG00000163689

DESCRIPTION CDNA FLJ42117 fis, clone TESTI2005376.

[Source:Uniprot/SPTREMBLAcc:Q6ZVT6]

AGTATTATATTTTTAACTGGAAAATCTCTACTTTATTTTGAAGTTTTTTTCCTACAGTCATTCCTTTTTTTCTTTCTTT

TTTTTATTTTTTTTTATTAT

In100 candidate1885 (in intron):

LOCUS ENSG00000163689

DESCRIPTION CDNA FLJ42117 fis, clone TESTI2005376.

[Source:Uniprot/SPTREMBLAcc:Q6ZVT6]

TACTATTTACCATTGTGGCTTGGTGATATTCTGTAATCATAACATTTTATCTTCTTTGTTTGTTTACCCTACCAGTGTTT

TTTATATATTTGTGTGTTTT

In100 candidate1886 (in intron):

LOCUS ENSG00000189283

DESCRIPTION Bis(5'-adenosyl)-triphosphatase (EC 3.6.1.29) (Diadenosine

5',5''- P1,P3-triphosphate hydrolase) (Dinucleosidetriphosphatase) (AP3A

hydrolse) (AP3AASE) (Fragile histidine triad protein).

[Source:Uniprot/SWISSPROT;cc:P49789]

TACACATAAGCCATTTTCTGCCGCATTTAAGGAAACCATTAACCACTTGCTTCTGTTATTCTCTTCATGTATATTCAGCC

CCTTTCTCTGTTCTGCTTTG

In100 candidate1887 (in intron):

LOCUS ENSG00000189283

DESCRIPTION Bis(5'-adenosyl)-triphosphatase (EC 3.6.1.29) (Diadenosine 5',5''- P1,P3-triphosphate hydrolase) (Dinucleosidetriphosphatase) (AP3A hydrolse) (AP3AASE) (Fragile histidine triad protein). [Source:Uniprot/SWISSPROT;cc:P49789]

AAAAGGATTGTTATTGCTTGGTAGTGAGATTAACTGGTGATCTAAATTTTCTCCTATGTGTTTTTCTGTCTCTTCCAGAT
TTTCTATGCTGTAAGTTTTT

In100 candidate1888 (in intron):

LOCUS ENSG00000189283

DESCRIPTION Bis(5'-adenosyl)-triphosphatase (EC 3.6.1.29) (Diadenosine 5',5''- P1,P3-triphosphate hydrolase) (Dinucleosidetriphosphatase) (AP3A hydrolse) (AP3AASE) (Fragile histidine triad protein). [Source:Uniprot/SWISSPROT;cc:P49789]

AGAAAACAAAGAAGGATTCTAATCATGTATAACATAATTTTCCCATTAAACTTATGCTATTTTCTTCTTCTCAGAGCTCA
GCACTTTTAGTATTTTTTTT

In100 candidate1889 (in intron):

LOCUS ENSG00000189283

DESCRIPTION Bis(5'-adenosyl)-triphosphatase (EC 3.6.1.29) (Diadenosine 5',5''- P1,P3-triphosphate hydrolase) (Dinucleosidetriphosphatase) (AP3A hydrolse) (AP3AASE) (Fragile histidine triad protein). [Source:Uniprot/SWISSPROT;cc:P49789]

TATGAAACTGGTAACTTCTGTTTCTCATACTTTTATCCATTCAGTCAGTCACAACTTCTGTGCTTCTGCCTGTGTTGTGT
CTCGCACATTGTTCTTTTCT

In100 candidate1890 (in intron):

LOCUS ENSG00000144724

DESCRIPTION Receptor-type tyrosine-protein phosphatase gamma precursor (EC

.1.3.48) (Protein-tyrosine phosphatase gamma) (R-PTP-gamma).

[Source:Unipro/SWISSPROT;Acc:P23470]

TTTGCCCCTTAATATGTTTATATTGTTGCTGTTAACTTTATTGTTTTTGTCTGTCTTACCAGACACAAGGCAAGATTTTG

TTTCTCTCTTCACTCTCTTT

In100 candidate1891 (in intron):

LOCUS ENSG00000125257

DESCRIPTION Multidrug resistance-associated protein 4 (ATP-binding

cassettesub- family C member 4) (MRP/cMOAT-related ABC transporter)

(Multispecificorganic

anion transporter-B) (MOAT-B).

[Source:Uniprot/SWISSPROT;Acc:O15439

AATTATAGGCCATCCAAGTAAAGATTTCTAATGTAATGTCCTGTAACTTAAACCTGATCATTATCCTGTTATTTTCAGGG

ATGTTCTTGTCATGTTTTTT

In100 candidate1892 (in intron):

LOCUS ENSG00000125257

DESCRIPTION Multidrug resistance-associated protein 4 (ATP-binding

cassettesub- family C member 4) (MRP/cMOAT-related ABC transporter) (Multi

specificorganic anion transporter-B) (MOAT-B).

[Source:Uniprot/SWISSPROT;Acc:O15439

TCTAAATTTCTGTAATCTTTTAACTAAAAAGAAGGGAATTTTTTTTATCTTTCTGTATTTTACCTACAGAATACTTTTAT

TGATCATACTGTGTTTTTCT

In100 candidate1893 (in intron):

LOCUS ENSG00000125257

DESCRIPTION Multidrug resistance-associated protein 4 (ATP-binding

cassettesub- family C member 4) (MRP/cMOAT-related ABC transporter)

(Multispecificorganic

anion transporter-B) (MOAT-B).

[Source:Uniprot/SWISSPROT;Acc:O15439

CCTAGGCTAAGTTGTTTAACTTATTTGCACTTCCATCTTTATCTCTGGCTGTCAGCATCTTGATAGACTGTTTCTTGCTC

ATCTTTGAATCTCCCATTCC

In100 candidate1894 (in intron):

LOCUS ENSG00000145431

DESCRIPTION platelet-derived growth factor C precursor

[Source:RefSeq_peptie;Acc:NP_057289]

AGCTTTTTGTTTTAATTCTGAATTTTAACTTTAATTTTGATTATATCTCTTACAGCTTTTGTCTTTATTAATTTTCACCT

GATATTTTGTTTTGATTTGC

In100 candidate1895 (in intron):

LOCUS ENSG00000109738

DESCRIPTION Glycine receptor subunit beta precursor (Glycine receptor 58 kD

subunit). [Source:Uniprot/SWISSPROT;Acc:P48167]

AGCTGATTAACCACCTTCATTGGCTATTTCTTATTGTCCTTCCTGACATATTGAGTTTTCCAGATATTCATCCTTAGTTC
TTTTAGCTTCTTGCTTATCA

In100 candidate1896 (in intron):

LOCUS ENSG00000109738

DESCRIPTION Glycine receptor subunit beta precursor (Glycine receptor 58 kD subunit). [Source:Uniprot/SWISSPROT;Acc:P48167]

AGTCATCCTGTTCATAACTTTGTCTCAATTTTTTCCTTCTTCCTTGAATCAGTAACTGTTTTCAAATGTTTTCTCTGCTT
TTTTTCACATCTGTCCTATC

In100 candidate1897 (in intron):

LOCUS ENSG00000080802

DESCRIPTION CCR4-NOT transcription complex subunit 4 (EC 6.3.2.-) (E3 ubiqutin protein ligase CNOT4) (CCR4-associated factor 4) (Potential transcriptinal repressor NOT4Hp). [Source:Uniprot/SWISSPROT;Acc:O95628]

GTGAAAAATGACAAAATAGAAGAAAAGTCAACCAAGATAACTATTGGTAAAAGTTTGCTTTAATGTGTTTCCTTTCAGTC
TTTTTGTTTATTTGTTTGTT

In100 candidate1898 (in intron):

LOCUS ENSG00000120251

DESCRIPTION Glutamate receptor 2 precursor (GluR-2) (GluR-B) (GluR-K2) (Gluamate receptor ionotropic, AMPA 2) (AMPA-selective glutamate receptor 2). [ource:Uniprot/SWISSPROT;Acc:P42262]

TGGTAGCATAATATACACTGTTCTTTATCTTGATTGTTTTCTTCTTAACATAAATCGGAGTTCTGCTTTTATGTGCAGAG

ATTTTCCTCTTCTTTTTTTC

In100 candidate1899 (in intron):

LOCUS ENSG00000120251

DESCRIPTION Glutamate receptor 2 precursor (GluR-2) (GluR-B) (GluR-K2)

(Gluamate receptor ionotropic, AMPA 2) (AMPA-selective glutamate receptor 2).

[ource:Uniprot/SWISSPROT;Acc:P42262]

ACAAATATATCTTCCTTAAAAATATATTCACTGGCAATTTCATAACACAATCCTGAGTTATTCACGTATCTTTATCAGTC

ATCATTTTTCTTTGCCTTCC

In100 candidate1900 (in intron):

LOCUS ENSG00000120251

DESCRIPTION Glutamate receptor 2 precursor (GluR-2) (GluR-B) (GluR-K2)

(Gluamate receptor ionotropic, AMPA 2) (AMPA-selective glutamate receptor 2).

[ource:Uniprot/SWISSPROT;Acc:P42262]

AGAAGTGTTTAACTTTTTCCATCCTTTCATATTTATTTTTTTGTATTTCATACAGACTTTGTGATAAAGTCCCTTAATTA

TATTCTTTTTGTTATTTCTT

In100 candidate1901 (in intron):

LOCUS ENSG00000163618

DESCRIPTION Calcium-dependent secretion activator 1 (Calcium-dependent

actiator protein for secretion 1) (CAPS-1). [Source:Uniprot/SWISSPROT;Acc:Q9ULU]

GTAACATTATTTAACTGGTTGTATTGATTTCTCTTTCTTTTTTTACTACTCTATACATTCTGCAGTAAGCATCTTTGTGT

ACTTACCTTTCTACACTTCT

In100 candidate1902 (in intron):

LOCUS ENSG00000146950

DESCRIPTION Apical-like protein (Protein APXL).

[Source:Uniprot/SWISSPROT;Ac:Q13796]

AAGCAATGAACCAATATTGCTACATTATTTGTTGTTAACTAAAGCCCAAACTTTGTTCCTATTTCCTTAGTTTCCCAGTT

TCCCATTAATGTCCTTTTCT

In100 candidate1903 (in intron):

LOCUS ENSG00000134873

DESCRIPTION Claudin-10 (OSP-like protein).

[Source:Uniprot/SWISSPROT;Acc:P7369]

CTCAAATGGTTAAGATGAGAAGTGGAAATAATTTTCTAACAATAAATTATTTCCTATCTGAATTATTTTTCCTGTCAGGA

CTTTCTGTTACTTCCTCTGA

In100 candidate1904 (in intron):

LOCUS ENSG00000152910

DESCRIPTION Contactin-associated protein-like 4 precursor (Cell

recognitionmolecule Caspr4). [Source:Uniprot/SWISSPROT;Acc:Q9C0A0]

GTATATTGTTACTTTTAAAGGCTAACCACTAGTTCTATTCTCCAATGCTTTTTATTTTGATTAATTTCTTTTTGTCAGTT

AGTTGATATTTTACCTTTTT

In100 candidate1905 (in intron):

LOCUS ENSG00000171509

DESCRIPTION Relaxin receptor 1 (Relaxin family peptide receptor 1)

(Leucinerich repeat-containing G-protein coupled receptor 7).

[Source:Uniprot/SWISSROT;Acc:Q9HBX9]

TTCAAGAATTGTCCTTAGCATTCACAATTCGGCTAACTGGTGCATGAGGCCTGGTTTTTGACTTATCTCAGCTTTCAAA

ATGCCTTCCTTACTATGTTT

In100 candidate1906 (in intron):

LOCUS ENSG00000181072

DESCRIPTION Muscarinic acetylcholine receptor M2.

[Source:Uniprot/SWISSPROTAcc:P08172]

CTAATTTACCATCTGTATATGTTTTCTGGTGAGTTATCCATTAAGATAACTGTGGGTTTGTCTTTTCATTTCTCTCAGTC

TGTGGCTTCTTTGCTCATTG

In100 candidate1907 (in intron):

LOCUS ENSG00000163632

DESCRIPTION Putative uncharacterized protein C3orf49.

[Source:Uniprot/SWISSROT;Acc:Q96BT1]

ACCCAAAGTACTATATCATCTTAGAATACACATTTTTTATAGCTAACATTTACTGTATTCTTTCCATTAATACAGTAATT

GTTATTATTCTTTTACCACC

In100 candidate1908 (in intron):

LOCUS ENSG00000140873

DESCRIPTION ADAMTS-18 precursor (EC 3.4.24.-) (A disintegrin and

metalloproeinase with thrombospondin motifs 18) (ADAM-TS 18) (ADAM-TS18).

[Source:Unirot/SWISSPROT;Acc:Q8TE60]

AGCATTTTTAAAGTTTTCTGTGTGAATTTTATAACAATCTATAGTAATTTGCCTTTCTCTTTTCCAGTTTTAATAACTCT

GTCTCTCACTTGGTTTTGTT

In100 candidate1909 (in intron):

LOCUS ENSG00000126261

DESCRIPTION SUMO-activating enzyme subunit 2 (EC 6.3.2.-) (Ubiquitin-like 1 activating enzyme E1B) (Anthracycline-associated resistance ARX). [Source:niprot/SWISSPROT;Acc:Q9UBT2]

GAGAGAATCCACTTTCGGTAAAGGCTAACTTTTGCATGTGGTGTTTTGCTTTCCTTTTGTACCATTCTTCAGACAGGATT

TTCGTCTCTGTCCTGTGTCT

In100 candidate1910 (in intron):

LOCUS ENSG00000162959

DESCRIPTION Protein memo (Mediator of ErbB2-driven cell motility) (C21orf19like protein) (Hepatitis C virus NS5A-transactivated protein 7) (HCV NS5A- ransactivated protein 7). [Source:Uniprot/SWISSPROT;Acc:Q9Y316]

TCATTTTTAACTCTTTACTTAGTAGTATTTTTTCTGCATTTAATCTCCTGTTTTTCTATATTAGGTTTTCTTGACCAGTT

ACTTTGAATTCTTGGTCTTC

In100 candidate1911 (in intron):

LOCUS ENSG00000052795

DESCRIPTION CDNA FLJ12906 fis, clone NT2RP2004373.

[Source:Uniprot/SPTREMBLAcc:Q9H994]

AGCCCTACCCATGCTTCTTAACTGTATTTACTCCATTTTTTTCTTTCCAGTTACAGGGTTCTTTCTTTTTTCTTTTTCTT

TCTTTCTTTTTTTTTTTTAA

In100 candidate1912 (in intron):

LOCUS ENSG00000052795

DESCRIPTION CDNA FLJ12906 fis, clone NT2RP2004373.

[Source:Uniprot/SPTREMBLAcc:Q9H994]

AGTGGCCCCGGTGGCCCTTGAATTGTAACTAAAAGTAGTTGATGATATTTTTATCTCTTTCCAGCATTCATTTCTTTTCT

TGAATATTTAGACTTCCCTC

In100 candidate1913 (in intron):

LOCUS ENSG00000052795

DESCRIPTION CDNA FLJ12906 fis, clone NT2RP2004373.

[Source:Uniprot/SPTREMBLAcc:Q9H994]

AGTTAACTTGCAACATGTCTCTTTGTCGTTTTTTTTTTTTGCTTTTGTTTATATCTCTCTACCAGCCTCATACTTCAATC

CTGCCTTATTAGTTTTTTTT

In100 candidate1914 (in intron):

LOCUS ENSG00000141140

DESCRIPTION myosin head domain containing 1 isoform 2

[Source:RefSeq_peptid;Acc:NP_001028751]

GGGTACTTTGTGTGGGTCTTTTTAACTTGGAAACTCATGTCCTTTTGTTCAGGAAAGTATTTTTGTGTTATATCTAATAA

TTTCCTCCCCTGTTTTCTCC

In100 candidate1915 (in exon):

LOCUS ENSG00000102580

DESCRIPTION DnaJ homolog subfamily C member 3 (Interferon-induced, doublesranded RNA-activated protein kinase inhibitor) (Protein kinase inhibitor p5) (Protein kinase inhibitor of 58 kDa). [Source:Uniprot/SWISSPROT;Acc:Q1321]

TGCTATTTTTATTTCCTATAACACGAAGTCTATTATCTCCTTAATAATTTTTAATTTCCATCCTTAGAACTTCTTCAGCT

CTGCTACTTTTTCCTTAAGG

In100 candidate1916 (in intron):

LOCUS ENSG00000186153

DESCRIPTION WW domain-containing oxidoreductase isoform 2 [Source:RefSeq_petide;Acc:NP_570607]

GCCTCTGATCCATCCTAACCCGACATTCCCTTGATCTCCTGATCTGTGAGCATCCTGGCCCTTCTTTTCTTTTCAGTT

TTAATTTTTGCGCTGGCTCC

In100 candidate1917 (in intron):

LOCUS ENSG00000186153

DESCRIPTION WW domain-containing oxidoreductase isoform 2 [Source:RefSeq_petide;Acc:NP_570607]

TTTTGTTAACTTATTTTGTAGTTTCCTCAATCTTGTCGTCAGCTGTGTCTTGTATTCTGTTAAACTATATGCTGAATTCT

TCTTTACTCTTATTACTCTT

In100 candidate1918 (in intron):

LOCUS ENSG00000186153

DESCRIPTION WW domain-containing oxidoreductase isoform 2

[Source:RefSeq_petide;Acc:NP_570607]

TAACTGATTTATTTTTCCCTCTTGGAGTTGCTTATTCTTTTCTTCTTCCAGTTGCATCTCTCCCTCTACTTAGATACACA

TTTCCATTTTACTTCTATTT

In100 candidate1919 (in intron):

LOCUS ENSG00000168843

DESCRIPTION Follistatin-related protein 5 precursor (Follistatin-like 5).

[ource:Uniprot/SWISSPROT;Acc:Q8N475]

CAATATTTTCTCTATTTTCCCCTTTTCTTTCAACCTTTTTTATTAACTTTTTTCTTACCACATTTTTATCAGTCCCTAT

ATTTCTTCTCATTCCATATT

In100 candidate1920 (in intron):

LOCUS ENSG00000168843

DESCRIPTION Follistatin-related protein 5 precursor (Follistatin-like 5).

[ource:Uniprot/SWISSPROT;Acc:Q8N475]

CCAACTAGCTTCAATTGAAAATTGTTTAATGTGGAAGTTTTTAACTGATTAAAAATTTCCTATCTTTACTTTGTACAGAA

ATTTCTTCTCTTTAAACTTT

In100 candidate1921 (in intron):

LOCUS ENSG00000168843

DESCRIPTION Follistatin-related protein 5 precursor (Follistatin-like 5).

[ource:Uniprot/SWISSPROT;Acc:Q8N475]

AGCCTTGAGTACTCTGCCTAACCCTCTTGCCCCATGCCTTTGCAATTCTCATTTCTTTAATCATTCAGCTTTCTCAACTC

ATCCATGATTTCCTTTTTAC

In100 candidate1922 (in intron):

LOCUS ENSG00000168843

DESCRIPTION Follistatin-related protein 5 precursor (Follistatin-like 5).

[ource:Uniprot/SWISSPROT;Acc:Q8N475]

TTTTCTGTTTTCTCATTCTGGGATTCAAAAGAACCCTGGTTTCATTAACCGTATCTGGTTTTGCTTTTTATTCAGTAAAG

TTTCATTTAATCTCTTTTCT

In100 candidate1923 (in intron):

LOCUS ENSG00000168843

DESCRIPTION Follistatin-related protein 5 precursor (Follistatin-like 5).

[ource:Uniprot/SWISSPROT;Acc:Q8N475]

GCAAGTTCTCCATAACACAAGCTGTTCTCTGATTCCATTGTTTTATTACTACTGTTCTTTTTTTTTTTTTTGAGACAGAG

TTTCATTCTTATTGCCCTGG

In100 candidate1924 (in intron):

LOCUS ENSG00000122779

DESCRIPTION Transcription intermediary factor 1-alpha (TIF1-alpha) (Tripartte motif-containing protein 24) (RING finger protein 82).

[Source:Uniprot/SISSPROT;Acc:O15164]

AGTTGATAATAACCTCTTGGTAATTCTTTCTCTGTGTCATCTAACGTTATGAATTTTCTTTTTCATTCCCAGTGCTGATT

TTATAGGCCTTCCCTATTTC

In100 candidate1925 (in intron):

LOCUS ENSG00000021574

DESCRIPTION Spastin. [Source:Uniprot/SWISSPROT;Acc:Q9UBP0]

AATTTTAGTTAATTTATAACAAACTCTTAATAATTTCTTTTTCTTCTTCTTCTTTTTTTTTTTTTTTTTTGAGTCAGGG

TCTTGCTCTTTTGCTCAGGT

In100 candidate1926 (in intron):

LOCUS ENSG00000021574

DESCRIPTION Spastin. [Source:Uniprot/SWISSPROT;Acc:Q9UBP0]

GTTGTGGTTTTGATTTGCATTTCCCTCATAATTAACGATGTTGAGCATTTTTTCATATCTTTGTTTTTGACGGTCCAGCT

AGTTTTATTGGTTACTTTTT

In100 candidate1927 (in intron):

LOCUS ENSG00000102595

DESCRIPTION UDP-glucose:glycoprotein glucosyltransferase 2 precursor (EC 2..1.-) (UDP-glucose ceramide glucosyltransferase-like 1) (UDP-- Glc:glycoproein glucosyltransferase 2) (HUGT2). [Source:Uniprot/SWISSPROT;Acc:Q9NYU1]

GAGGAAGAGCTGGGTTGCCAAGATCTTTTGGGGACTTGCCCTACAATAACATCATATAAAATGTGTTTTTTCCCTCAGTG

TTCTTCACATTCTCAATTTT

In100 candidate1928 (in intron):

LOCUS ENSG00000102595

DESCRIPTION UDP-glucose:glycoprotein glucosyltransferase 2 precursor (EC 2..1.-) (UDP-glucose ceramide glucosyltransferase-like 1) (UDP-- Glc:glycoproein

glucosyltransferase 2) (HUGT2). [Source:Uniprot/SWISSPROT;Acc:Q9NYU1]

TTTCTTCTTTTAAAAGGGCATATATTTATGTTGGTAGTTCTAATTAACCTCTACCTTTATTTCATCTCTTTAAACCAGTT

TCCTCCTTTTCCATTATATG

In100 candidate1929 (in intron):

LOCUS ENSG00000166446

DESCRIPTION Chromodomain Y-like protein 2 (CDY-like 2).

[Source:Uniprot/SWISPROT;Acc:Q8N8U2]

GGCTTGGCTAACACCACTCCCCGTTTTCTTCCCTGTGTACCAAACTTACTGACTCTTTTTTGTTCATGCAGCTATCCTTT

GCTTCATGACCTTTGCTCAT

In100 candidate1930 (in intron):

LOCUS ENSG00000108270

DESCRIPTION Protein AATF (Apoptosis-antagonizing transcription factor) (Rbinding

protein Che-1). [Source:Uniprot/SWISSPROT;Acc:Q9NY61]

CTTTTTTTGTTTTTTGTTTTGAATCCATGGGTTTCTCTAATTAACATGGTTTGTTTCTACTTGACTTTTCAGTTTTAGGC

ATTACTTGTTGACTCCCCTT

In100 candidate1931 (in intron):

LOCUS ENSG00000151276

DESCRIPTION Membrane-associated guanylate kinase, WW and PDZ domain-containng protein 1 (BAI1-associated protein 1) (BAP-1) (Membrane-associated guanyate kinase inverted 1) (MAGI-1) (Atrophin-1-interacting protein 3) (AIP3) (W domaincontaining

protein 3) (WWP3) ([Source:Uniprot/SWISSPROT;Acc:Q96QZ]

ACTATGAGTGTAAATTTTCTCAATATAGTAGCTCTCAATAGATGGTAACTGACATTATTATTTTCCTTATTAGTACAGAT
TCTTTCACTGTTGTGTTACT

In100 candidate1932 (in intron):

LOCUS ENSG00000151276

DESCRIPTION Membrane-associated guanylate kinase, WW and PDZ domain-containng protein 1 (BAI1-associated protein 1) (BAP-1) (Membrane-associated guanyate kinase inverted 1) (MAGI-1) (Atrophin-1-interacting protein 3) (AIP3) (W domaincontaining

protein 3) (WWP3) ([Source:Uniprot/SWISSPROT;Acc:Q96QZ]

AAATAATAGTTTCTCTTTTCCCTTTCTTTCCTTTATTTTTATGACGAATTAACTTACCTGGAATCAAGTTTTTTTCAGAA
TTTGAGTTCCAACCTTTTTC

In100 candidate1933 (in intron):

LOCUS ENSG00000145416

DESCRIPTION E3 ubiquitin-protein ligase MARCH1 (EC 6.3.2.-) (Membraneassocated RING finger protein 1) (Membrane-associated RING-CH protein I) (MARCHI) (RING finger protein 171). [Source:Uniprot/SWISSPROT;Acc:Q8TCQ1]

TGTTAGTCTGAATGATATTAACTACTGTCCATCTTGTTCTCTATTTATGAAGTATGTTACTTCTCCTTTCCACTGCAGAT
CAGTTTGCTTGACTTCCTCT

In100 candidate1934 (in intron):

LOCUS ENSG00000145416

DESCRIPTION E3 ubiquitin-protein ligase MARCH1 (EC 6.3.2.-) (Membraneassocated RING finger protein 1) (Membrane-associated RING-CH protein I) (MARCHI) (RING finger protein 171). [Source:Uniprot/SWISSPROT;Acc:Q8TCQ1]

AAAAAAATTCACAAAATAATACAAATAGTAACTACACAAACAATTATATCCATCCAGGTTCCCAGCATGGATGTGGTTTT
ATCTTCTTCCTCCATCCTTT

In100 candidate1935 (in intron):

LOCUS ENSG00000152683

DESCRIPTION solute carrier family 30 (zinc transporter), member 6 [Source:RfSeq_peptide;Acc:NP_060434]

AGAATAACCCTTATATATCCTTAATTATTTTTCATGAACCTTTCCCTCATGTTCCAGTATCCTCTTAGTTGCTTCTCCTT
TGTGCTCTGGTGGTACCTTG

In100 candidate1936 (in intron):

LOCUS ENSG00000132142

DESCRIPTION Acetyl-CoA carboxylase 1 (EC 6.4.1.2) (ACC-alpha) [Includes: Bitin carboxylase (EC 6.3.4.14)]. [Source:Uniprot/SWISSPROT;Acc:Q13085]

ATAGTCTGATTTTCTGTTTAACATGTGCTGGGCTCTCTCTCTCTTTTTTTTTCCCTCAATCTATTACTAACTCTCAGAGT
AACCCTCTTCATTTCTTTCT

In100 candidate1937 (in intron):

LOCUS ENSG00000183439

DESCRIPTION Putative tripartite motif-containing protein 61.

[Source:Unipro/SWISSPROT;Acc:Q5EBN2]

TGAATCCTATTGTGATGGCGTCTTTTTCCATCTCCTGGGAAGACAATTCTAACAAACAACTCTATTATATCTATCCAGTT

TGATTTTGTCCTCTTTCTCT

In100 candidate1938 (in intron):

LOCUS ENSG00000205582

DESCRIPTION

TGAAAGCCCAATAACAAGCTGGTGGTTTGTTTTATTGCTTTTTTAACACTTTTATTTATCAGTTCATAGTAGTCATGATT

CTCTTGCTTATTTTATCTTC

In100 candidate1939 (in intron):

LOCUS ENSG00000088881

DESCRIPTION Transcription factor COE4 (Early B-cell factor 4) (EBF-4) (Olf-

/EBF- like 4) (OE-4) (O/E-4). [Source:Uniprot/SWISSPROT;Acc:Q9BQW3]

TTTTTATTATATATGAATAATTTTTTTTAACTCTGCTAAATTTTCTGCTCACCTTTTCCTTCTTGCATTTTGCAGCTTTT

TGGAGGGCCATTTTCTTTCT

In100 candidate1940 (in intron):

LOCUS ENSG00000144741

DESCRIPTION solute carrier family 25, member 26

[Source:RefSeq_peptide;Acc:P_775742]

AGTAAGGTGATAACTTTGGAATATTTTCACTTTCCTCTTGTTCAGCTTTCCCCACACCCAGCCAAAAAGTTTTTTGTTTT

TTTTTGTTTGTTTGTTTGTT

In100 candidate1941 (in intron):

LOCUS ENSG00000105948

DESCRIPTION tetratricopeptide repeat domain 26

[Source:RefSeq_peptide;Acc:N_079202]

AGTTAGCTTCATAACTGCCATCCTGTTATTAGTTCTCTATTTGCCCAGCTGGTCCATGTGGTCTGTGTTCCTTTTTCTCT

CCTTTCTTGCCTTTTTTTGG

In100 candidate1942 (in intron):

LOCUS ENSG00000105948

DESCRIPTION tetratricopeptide repeat domain 26

[Source:RefSeq_peptide;Acc:N_079202]

TTAGAGAGTGGGATTAGAAAGAGAAGTAACAATATGGAAATTTTTCTTTCATAATACTTTATTTTTTTCCAGTGAACACA

TATTACTTTTATTTTTTTTT

In100 candidate1943 (in intron):

LOCUS ENSG00000109472

DESCRIPTION Carboxypeptidase E precursor (EC 3.4.17.10) (CPE)

(Carboxypeptiase H) (CPH) (Enkephalin convertase) (Prohormone-processing

carboxypeptidas). [Source:Uniprot/SWISSPROT;Acc:P16870]

CTTGACTTTCCTAACCCCTGCTCAACCTTCTGTCTTTAGCTTAAATGTTACCTTCTCAGGGAAACCTTCCTTGTCTTTCC

AGACTGATTTAGTTTTTTTC

In100 candidate1944 (in intron):

LOCUS ENSG00000185352

DESCRIPTION Heparan-sulfate 6-O-sulfotransferase 3 (EC 2.8.2.-) (HS6ST-3).

Source:Uniprot/SWISSPROT;Acc:Q8IZP7]

GTTCCTAACTTTAAATGTATACCTATATACAACTTTCTCTCTCCCCATTTTTAGTTCAATTGCTCATATTATCAGGATTT

ATATATTTAAATTCTTTTCT

In100 candidate1945 (in intron):

LOCUS ENSG00000185352

DESCRIPTION Heparan-sulfate 6-O-sulfotransferase 3 (EC 2.8.2.-) (HS6ST-3).

Source:Uniprot/SWISSPROT;Acc:Q8IZP7]

GTTTTTTTCTTCTAACTCTTATACTTTTATTTATCTTTCCATTTCCCTTATTTATTTCCTGCCACTCCTTAATTACAGTT

TTTTGTTTGTTTGTTTTTGT

In100 candidate1946 (in intron):

LOCUS ENSG00000185352

DESCRIPTION Heparan-sulfate 6-O-sulfotransferase 3 (EC 2.8.2.-) (HS6ST-3).

Source:Uniprot/SWISSPROT;Acc:Q8IZP7]

ATAACTACTAGCTTCTGCTTCTTTCTATGTTTTAGCCCAATTTTTTTCCTTCTATAGACCAGATTTCCTTCTTCTCAGCA

TCCTTTTCCTTCTTCTGAGT

In100 candidate1947 (in intron):

LOCUS ENSG00000185352

DESCRIPTION Heparan-sulfate 6-O-sulfotransferase 3 (EC 2.8.2.-) (HS6ST-3).

Source:Uniprot/SWISSPROT;Acc:Q8IZP7]

TTGGGTCTTTATTATTTTCTTCCTTCTTATAACCTCTGGCTTACTTTTGCATTTTTGCCTAATTTCTTGCAGCCTTAGAT

TATTTTAGATCTTTCTGTTT

In100 candidate1948 (in intron):

LOCUS ENSG00000185352

DESCRIPTION Heparan-sulfate 6-O-sulfotransferase 3 (EC 2.8.2.-) (HS6ST-3).

Source:Uniprot/SWISSPROT;Acc:Q8IZP7]

TCAAATATTTGCCCATTTTGTTTTCAAAGAAATAACTTTTGACATTTTAGTTTTTTCTATTGTCTATTTGTTTTCCAGTT

CTTTATTCTCTTTGTGAATG

In100 candidate1949 (in intron):

LOCUS ENSG00000127688

DESCRIPTION Gigaxonin (Kelch-like protein 16).

[Source:Uniprot/SWISSPROT;Ac:Q9H2C0]

ATGCTTGATAACCTCTGTAGGTCCTAATTTTCCCTTCAGTTTTGCCTTGTTATCTTGTTGGCTTCTCCTCTTCTAAGAAG

ATTATTATTGTTGTTATTTT

In100 candidate1950 (in intron):

LOCUS ENSG00000146963

DESCRIPTION Putative RNA-binding protein Luc7-like 2.

[Source:Uniprot/SWISSROT;Acc:Q9Y383]

ACTTTTCATCAACATTGAAAACATTTTCTCTTAATTTTTTCTCATTAACATAATGAACCATATTCATTTCTTGGTCAGTT

GTGAGCCATTCTTTTATTCC

In100 candidate1951 (in intron):

LOCUS ENSG00000018699

DESCRIPTION tetratricopeptide repeat domain 27

[Source:RefSeq_peptide;Acc:N_060205]

TTCTGAATACCTTTTTACTCTGTAACCGCCTTTCTGAGTATTTTTTTGTTCTTATTAATGGTTTGATCTTTTCAGATCAA

GGCATCATTTTTGCCTCTTT

In100 candidate1952 (in intron):

LOCUS ENSG00000107104

DESCRIPTION Ankyrin repeat domain-containing protein 15 (Kidney ankyrin

repat- containing protein). [Source:Uniprot/SWISSPROT;Acc:Q14678]

TAATTGGTGAAGAAGTGGTGAAGAGGATAAGGATTAGTCTAACAAGTTTGTTTGTAATATTTCCCTCAGACTCTTCCCCT

CCTTGATGATGATAATATTT

In100 candidate1953 (in intron):

LOCUS ENSG00000107104

DESCRIPTION Ankyrin repeat domain-containing protein 15 (Kidney ankyrin

repat- containing protein). [Source:Uniprot/SWISSPROT;Acc:Q14678]

AGTTTTTTTTTAACTAAACTTTTGTTTTGTTGATCTTCAGTAATGTTTTAATCTCAATTTCATTTATTTTTGCTCTGATC

TTTATTATTTCCTTTCTCCT

In100 candidate1954 (in intron):

LOCUS ENSG00000172340

DESCRIPTION Succinyl-CoA ligase [GDP-forming] beta-chain, mitochondrial

preursor (EC 6.2.1.4) (Succinyl-CoA synthetase, betaG chain) (SCS-betaG)

(GTPspecific

succinyl-CoA synthetase subunit beta).

[Source:Uniprot/SWISSPROT;Ac:Q96I99]

TCTTAATTACTGTATTTATATAAATTCTGAAATTGAGTAGAATAACTCTTGTCACTTTATTCTTCTTTTCCAAAACAGTT

TTACCTGTTCTAGTTCCTAT

In100 candidate1955 (in intron):

LOCUS ENSG00000172340

DESCRIPTION Succinyl-CoA ligase [GDP-forming] beta-chain, mitochondrial

preursor (EC 6.2.1.4) (Succinyl-CoA synthetase, betaG chain) (SCS-betaG)

(GTPspecific

succinyl-CoA synthetase subunit beta).

[Source:Uniprot/SWISSPROT;Ac:Q96I99]

TCTTTTTTTTCATGACTCATCCTTCCTTAACTCATCTTTCTTATCCTCTGAAGCTTTCTTTTCAGAGAATCAAAATAGCT

TTCTTCATCTTTTTTTTCTT

In100 candidate1956 (in intron):

LOCUS ENSG00000172340

DESCRIPTION Succinyl-CoA ligase [GDP-forming] beta-chain, mitochondrial

preursor (EC 6.2.1.4) (Succinyl-CoA synthetase, betaG chain) (SCS-betaG)

(GTPspecific

succinyl-CoA synthetase subunit beta).

[Source:Uniprot/SWISSPROT;Ac:Q96I99]

GTCCTTTTAACTCCTTGTCTCCTTTTCCTATTTATAATGCCATTCAATATTTCCATCTTCAGATTTTTCTTCTCCTGAAA

ATCCTGTTAATACCTGACTT

In100 candidate1957 (in intron):

LOCUS ENSG00000196104

DESCRIPTION Testican-3 precursor (SPARC/osteonectin, CWCV, and Kazal-like dmains proteoglycan 3). [Source:Uniprot/SWISSPROT;Acc:Q9BQ16]

CACCTGATCTACCTTTTGGTTTAACTATGACATATTTTTCATCTCATTTAAGTTTCTTTTAATCAATCATTTTTCCAGTT
TCTTTATGTTTTACCACTAA

In100 candidate1958 (in intron):

LOCUS ENSG00000196104

DESCRIPTION Testican-3 precursor (SPARC/osteonectin, CWCV, and Kazal-like dmains proteoglycan 3). [Source:Uniprot/SWISSPROT;Acc:Q9BQ16]

TTTTAGTTAACATGATATTTGATATTTTTCTGCTTTTACTTTTATGTGTATTTATTTTATGCTGATTGTTTTCCCCAGGC
CATAAGTTTTTGTTTCTTCA

In100 candidate1959 (in intron):

LOCUS ENSG00000183662

DESCRIPTION Protein FAM19A1 precursor (Chemokine-like protein TAFA-1). [Souce:Uniprot/SWISSPROT;Acc:Q7Z5A9]

ATATTTTTGCATTTTTTCTCTAATATATCATAGCTAACGTTTGTCTCTGTAATTTTCCATTTTTGCTGTATTCAGCTCAT
TTTGCCACCTATGACGTTTT

In100 candidate1960 (in intron):

LOCUS ENSG00000049323

DESCRIPTION Latent-transforming growth factor beta-binding protein, isoform1L precursor (LTBP-1) (Transforming growth factor beta-1-binding protein 1)(TGFbeta1-

BP-1). [Source:Uniprot/SWISSPROT;Acc:Q14766]

GGAAATTTAACATGCTGAACACTCTTTTCATTTGCCTCTTTGTTTATTGTGTTGTTCAGTCTTGTCTCTTATCAATTTTA

GAAGCCTCATTCCTGATTTC

In100 candidate1961 (in intron):

LOCUS ENSG00000163377

DESCRIPTION Protein FAM19A4 precursor (Chemokine-like protein TAFA-4).

[Souce:Uniprot/SWISSPROT;Acc:Q96LR4]

TTGGTCCTCATTACCTAACACCTAACATTATTTCCTGTTTATCTTCTTTTCAAATTATCTCCATCCTCTAGGGTACAGGC

ATTGTTTTTCTATGTTTGCA

In100 candidate1962 (in intron):

LOCUS ENSG00000163377

DESCRIPTION Protein FAM19A4 precursor (Chemokine-like protein TAFA-4).

[Souce:Uniprot/SWISSPROT;Acc:Q96LR4]

GAACAAGGCATCCCCCCTGTACCTTTTTAGAGAAGTAAGTAAGTTTATAACAAGAGCTGCTAGTTTCTTGCATTTCAGCT

TTCATTTTTCTCCCATCCTA

In100 candidate1963 (in intron):

LOCUS ENSG00000015171

DESCRIPTION Zinc finger MYND domain-containing protein 11 (Adenovirus 5 E1A

binding protein) (BS69 protein). [Source:Uniprot/SWISSPROT;Acc:Q15326]

TCAATTCAAAAAATAACCTAATTAGATATTTAATCTTTCATTGCATATATATTTTTCCTGTTCAGCAGTAATTTCCTGAT

TTTTATCTCAGGTTTCTTTT

In100 candidate1964 (in intron):

LOCUS ENSG00000072364

DESCRIPTION AF4/FMR2 family member 4 (ALL1-fused gene from chromosome

5q31)(Major CDK9 elongation factor-associated protein).

[Source:Uniprot/SWISSPRO;Acc:Q9UHB7]

GCTTATATTTGTAACTTGATTATGTCCATCATAAATAATTTACTCTCTTCTTTGTTTACCATTTGCCTTCAGAGAGCTAG

TTTTGTTCTTTGTGTTTTTT

In100 candidate1965 (in intron):

LOCUS ENSG00000059377

DESCRIPTION Thromboxane-A synthase (EC 5.3.99.5) (TXA synthase) (TXS)

(Cytohrome P450 5A1). [Source:Uniprot/SWISSPROT;Acc:P24557]

TTCTTCTTCTTCTTACAAAATAGTTTATAGCTAACTATATTTCCTTACTCTCATTTGTTCCTTCCCTGTATTCAGTGTCT

CCCCATCAATATCTTTTATT

In100 candidate1966 (in intron):

LOCUS ENSG00000059377

DESCRIPTION Thromboxane-A synthase (EC 5.3.99.5) (TXA synthase) (TXS)

(Cytohrome P450 5A1). [Source:Uniprot/SWISSPROT;Acc:P24557]

AAAATAAAACTCTGTCAATATTTCCATGTAACATTGGGTTTTGCTTTTTTTTTTCCTCACGTAAAATCCACATATCAGGA

ATTCCTCTATTTATTCCGTC

In100 candidate1967 (in intron):

LOCUS ENSG00000086696

DESCRIPTION Estradiol 17-beta-dehydrogenase 2 (EC 1.1.1.62) (17-beta-HSD

2)(Microsomal 17-beta-hydroxysteroid dehydrogenase) (20

alphahydroxysteroiddehydrogenase)

(20-alpha-HSD) (E2DH).

[Source:Uniprot/SWISSPROT;Acc:P37059]EXON

AGAACATTGTAACTGGACCCTGACTTCTTTCTGCCCTAGTCTTTTGAGTTTCAGATCTTTGAGTCATCTTGGATTTGTTT

TTCCCCATGCCTCTCTCTTC

In100 candidate1968 (in intron):

LOCUS ENSG00000140945

DESCRIPTION Cadherin-13 precursor (Truncated-cadherin) (T-cadherin) (Tcad)(

Heart-cadherin) (H-cadherin) (P105).

[Source:Uniprot/SWISSPROT;Acc:P55290]EXON

TATTATTAATGAAAGAGTGAGTAACGCTGCCTTATTCTCCTTTTATTTCTCACCCACTCTTTTTGTGTTCAGATCACTAG

ATTTTAATTTTACGCCCTTT

In100 candidate1969 (in intron):

LOCUS ENSG00000140945

DESCRIPTION Cadherin-13 precursor (Truncated-cadherin) (T-cadherin) (Tcad)(

Heart-cadherin) (H-cadherin) (P105).

[Source:Uniprot/SWISSPROT;Acc:P55290]EXON

TTTAGGTGTTTGTGTGTGTATATTTTTTACCAAAATGAGATCGTACCATAACGTTATTTTCAAATCTGATTTTCTCAGTT

AACAATCTCTATCTTTCTAT

In100 candidate1970 (in intron):

LOCUS ENSG00000140945

DESCRIPTION Cadherin-13 precursor (Truncated-cadherin) (T-cadherin) (Tcad)(

Heart-cadherin) (H-cadherin) (P105).

[Source:Uniprot/SWISSPROT;Acc:P55290]EXON

CCATGACAATACAAATTCTTACTATTTTTTCATAGCTATGGTAGCCTTGTAACTTATTTACTGATTTCCCTTTTTCAGCC

ATCTTCCTGTTGTTTTGCAA

In100 candidate1971 (in intron):

LOCUS ENSG00000140945

DESCRIPTION Cadherin-13 precursor (Truncated-cadherin) (T-cadherin) (Tcad)(

Heart-cadherin) (H-cadherin) (P105).

[Source:Uniprot/SWISSPROT;Acc:P55290]EXON

AGTGTTCTCTTTGGTGAAATGTCTAACTATGTCTTTTGCCTATTTTTTGTTTCGTTTTGTTTTACAGGCCATTTTCAGGA

CTTTAACTTTTGCCCATTTT

In100 candidate1972 (in intron):

LOCUS ENSG00000137090

DESCRIPTION Doublesex- and mab-3-related transcription factor 1 (DM domain

xpressed in testis protein 1). [Source:Uniprot/SWISSPROT;Acc:Q9Y5R6]

TGAGAGAGTGGAGCGTGCTGATCATTATTAACCAAATTTAAAAAATACAACCTTTTTAATTTCCTTTCTCTCCAGCCATT

TTACTGAAACTCTGTTTCTT

In100 candidate1973 (in intron):

LOCUS ENSG00000137090

DESCRIPTION Doublesex- and mab-3-related transcription factor 1 (DM domain xpressed in testis protein 1). [Source:Uniprot/SWISSPROT;Acc:Q9Y5R6]

GTTGCTCTTCTTTTCCTCATTTAACTCCCTAGAATTTCATTTCATTTCATTTCTACCTCTGAATTTCAGAAAATTTCTAA
TCCATGCCATCTTTGCCTTT

In100 candidate1974 (in intron):

LOCUS ENSG00000030066

DESCRIPTION Nuclear pore complex protein Nup160 (Nucleoporin Nup160) (160 ka nucleoporin). [Source:Uniprot/SWISSPROT;Acc:Q12769]

TTGTTCATAGTGTCATACCTTAAGAATCCTTTGCCAAACCTAAGGTCATAACGATTTACTCTTATGTTTTCTTCTCAGAG
TTTTATACTTTTAGCTTTTA

In100 candidate1975 (in intron):

LOCUS ENSG00000006459

DESCRIPTION K1718_HUMAN Isoform 2 of Q6ZMT4 - Homo sapiens (Human) [Source:niprot/Varsplic;Acc:Q6ZMT4-2]

ACCTTTGCCTGAATGTACCTTGATACACACTTGGGTTGGTTTTAGGTAACTTATATGACCTCTTTATTTTACATGCAGCT
TTTTTCCTAGTACTTTTTTC

In100 candidate1976 (in intron):

LOCUS ENSG00000114541

DESCRIPTION FERM domain-containing protein 4B (GRP1-binding protein GRSP1).[Source:Uniprot/SWISSPROT;Acc:Q9Y2L6]

CCTGGTGCTTGCCAATAAATCTTTTTTTTCTTAACTCCTTTTTCATATTTCTATGTTCTCCTGACCTGTGGCTTCCAGTT
TTGTTGTTTTGTTTTAATCG

In100 candidate1977 (in intron):

LOCUS ENSG00000169933

DESCRIPTION PDZ domain containing 10 [Source:RefSeq_peptide;Acc:NP_055543]

EXON

CTTTGTGGTTTAGTTTGCATTTCCCTGATGACTAACGATGATGAGCATCTTTTCTTGTGCTTACTTACCATTCAGGTATC
TTCTTTGGTTAAGTGTCTTT

In100 candidate1978 (in intron):

LOCUS ENSG00000187098

DESCRIPTION Microphthalmia-associated transcription factor. [Source:UniprotSWISSPROT;Acc:O75030]

TGTCCCTATCCTTTCTTCACCCATCCCCTTCCTAGTTTCTGGTAACTACTGTTCTGTTTTCTACTTTTATGATGTCAGCT
TGTTTTACCCCTTAGATTTC

In100 candidate1979 (in intron):

LOCUS ENSG00000187098

DESCRIPTION Microphthalmia-associated transcription factor. [Source:UniprotSWISSPROT;Acc:O75030]

GTCTTATAAAAGGTAGTTTTAAAAATGATTTCTAACTTCTTGAGCTTCTATTTCTGCTGCAGTTTGTTTTAACAGATACT
TTAGATTTTTTTCATTCTTT

In100 candidate1980 (in intron):

LOCUS ENSG00000154447

DESCRIPTION SH3 domain containing ring finger 1

[Source:RefSeq_peptide;Acc:P_065921]

CCTCTAGGTTTTCTAGTTTATGCATGTAAAGGTGTTCATAGTAACCTCGAATGATCTTTTGTATTTCTGTGTTGTCAGTT

GTAATGTCTCTCGTTTTGTT

In100 candidate1981 (in intron):

LOCUS ENSG00000154447

DESCRIPTION SH3 domain containing ring finger 1

[Source:RefSeq_peptide;Acc:P_065921]

AGTCGTGTCCTAACACATTTGCTTCTTGTTGACTTTTTTTTTCCAGTTTTTTTTGTTCTGTTTTGTTTTGTTTTTTTTTT

TTTAAAGAGACAGCATCTCA

In100 candidate1982 (in intron):

LOCUS ENSG00000154447

DESCRIPTION SH3 domain containing ring finger 1

[Source:RefSeq_peptide;Acc:P_065921]

AGGTGTAAGCCACTGTGCCCGGCCTCTAACGTTCATTTTCTTTGTTCAAATGATTTTTCTTCCTCCTCCCAGGTGGTTTT

TTTTTTTTTTGTATTGTTTT

In100 candidate1983 (in intron):

LOCUS ENSG00000170606

DESCRIPTION Heat shock 70 kDa protein 4 (Heat shock 70-related protein APG-) (HSP70RY). [Source:Uniprot/SWISSPROT;Acc:P34932]

TAACTTTCTGAGATGTTTTCCCTCCCTCTTTTGTGTTTACTGTGTTGTTCTGCTTAGTTTGGATTTTTTAATCAGTAGTT

TTTCTTCAGTGAGTTCTTTT

In100 candidate1984 (in intron):

LOCUS ENSG00000150938

DESCRIPTION Cysteine-rich motor neuron 1 protein precursor (CRIM-1) (Cysteierich repeat-containing protein S52). [Source:Uniprot/SWISSPROT;Acc:Q9NZV1

ATATTTCTGTTGTCCATTGTACAAATTTTAATAACTTGTCTTTCCTTTTTTTAGGTCTCTGTGTTCCAGATTTTTTTTTG

TAACTATGAAATCCTTGCCT

In100 candidate1985 (in intron):

LOCUS ENSG00000137601

DESCRIPTION Serine/threonine-protein kinase Nek1 (EC 2.7.11.1) (NimA-relate protein kinase 1) (Renal carcinoma antigen NY-REN-55). [Source:Uniprot/SWISPROT;Acc:Q96PY6]

CTTTAAATTTTCCATAACTACTTTAATCTGTGATATTTTCCTTCTCTACATTCCTATCACATTTCTCATTATCACCAGTT

CTTAATTTTAATTGCCCGCT

In100 candidate1986 (in intron):

LOCUS ENSG00000137601

DESCRIPTION Serine/threonine-protein kinase Nek1 (EC 2.7.11.1) (NimA-relate protein kinase 1) (Renal carcinoma antigen NY-REN-55).

[Source:Uniprot/SWISPROT;Acc:Q96PY6]

AGTTTGTACACTACTAACCATTCACTGATGATGCTCCCTATTTTGTTCAGTCTTTTTTTTTTTCTCTGTGTGTTTCATTT

TGGGTTGTTCTATTGCTGTG

In100 candidate1987 (in intron):

LOCUS ENSG00000137601

DESCRIPTION Serine/threonine-protein kinase Nek1 (EC 2.7.11.1) (NimA-relate

protein kinase 1) (Renal carcinoma antigen NY-REN-55).

[Source:Uniprot/SWISPROT;Acc:Q96PY6]

GAATTCTTTCCTTTCTCATAACTCTTAGGACTATTTTTCTGTCCTTGACGTTCTGAACTTCACTTGAATATTTCTCAGTT

GTTTTGTTTTGTCTTACTGG

In100 candidate1988 (in intron):

LOCUS ENSG00000132670

DESCRIPTION Vacuolar protein sorting-associated protein 16 homolog (hVPS16)

[Source:Uniprot/SWISSPROT;Acc:Q9H269]

TCAAAAATAACCCTTCAATCAATTTTTTTTTTCCTGGTCAAATTGGATTTTGTGTTTCTTTTATATATTCCTTGTCAGGC

CCTACAGTTATTTTAATTTT

In100 candidate1989 (in intron):

LOCUS ENSG00000132670

DESCRIPTION Vacuolar protein sorting-associated protein 16 homolog (hVPS16)

[Source:Uniprot/SWISSPROT;Acc:Q9H269]

AAACAAACCCATAAAGTGATTACATTTCTAACTTTCGGAAGTACTCTTCAACCTCCTTTTTTTAGGTTAATTTTCAGCAC

TCTTTCCTTCTTTCTTCCTT

In100 candidate1990 (in intron):

LOCUS ENSG00000132670

DESCRIPTION Vacuolar protein sorting-associated protein 16 homolog (hVPS16)

[Source:Uniprot/SWISSPROT;Acc:Q9H269]

CCTGTTATTTCCAATAACTTACTTCCTATGGATTCTTGTAGATTTTCTTTTTTTTTTTTTAATTTTTTTATTTTCAGAT

GTTTTCTCGCTTTGTCACCA

In100 candidate1991 (in intron):

LOCUS ENSG00000198673

DESCRIPTION Protein FAM19A2 precursor (Chemokine-like protein TAFA-2).

[Souce:Uniprot/SWISSPROT;Acc:Q8N3H0]

AACACTTTATATTTTTGAATGACATAGATTCTAAGCTGATAACTGTTCCCCAATTTTCTTTTATTTCAACAGTTTTTGTC

ATTCTGCATTTCTCAGATTT

In100 candidate1992 (in intron):

LOCUS ENSG00000198673

DESCRIPTION Protein FAM19A2 precursor (Chemokine-like protein TAFA-2).

[Souce:Uniprot/SWISSPROT;Acc:Q8N3H0]

ATGAAAATTTTGGGGCACCTTTAACATTTGGTACAACATTTTTCTATTCCTTATTGTTTCTTTCCTCAGATATTTTTGTC

CCCCATAAACAGATTTTTTT

In100 candidate1993 (in intron):

LOCUS ENSG00000135655

DESCRIPTION Ubiquitin carboxyl-terminal hydrolase 15 (EC 3.1.2.15) (Ubiquitn thioesterase 15) (Ubiquitin-specific-processing protease 15) (Deubiquitinting enzyme 15) (Unph-2) (Unph4). [Source:Uniprot/SWISSPROT;Acc:Q9Y4E8]

TCTGTCCGTTTTGTTTCTCTTTTCAAAGAACTAACTTTTCATTTCATTGATCTTTTTGTATTGTGTTTTTGGTCTCAGTG
TCACTTACTTTTCCTCTGAT

In100 candidate1994 (in intron):

LOCUS ENSG00000135655

DESCRIPTION Ubiquitin carboxyl-terminal hydrolase 15 (EC 3.1.2.15) (Ubiquitn thioesterase 15) (Ubiquitin-specific-processing protease 15) (Deubiquitinting enzyme 15) (Unph-2) (Unph4). [Source:Uniprot/SWISSPROT;Acc:Q9Y4E8]

TACTTTCTTAACTCCACCCCTTATTAATTCTTACCTTTGTTGTTTTTATAGTTCCCTAACTGGTCTCCCAATCTTCAGTC
TTTTTCTCTTCCTTAAAAGT

In100 candidate1995 (in intron):

LOCUS ENSG00000135655

DESCRIPTION Ubiquitin carboxyl-terminal hydrolase 15 (EC 3.1.2.15) (Ubiquitn thioesterase 15) (Ubiquitin-specific-processing protease 15) (Deubiquitinting enzyme 15) (Unph-2) (Unph4). [Source:Uniprot/SWISSPROT;Acc:Q9Y4E8]

GGTGATAACCCCTTTATCATTTTTTATTGCGTCTTTTTGATTCTTCTCTCTTTTCTTCTTTATTAGTCTGGCTAGCAGTC

TGTCTATTTTGTTTATCTTT

In100 candidate1996 (in intron):

LOCUS ENSG00000157764

DESCRIPTION B-Raf proto-oncogene serine/threonine-protein kinase (EC 2.7.111)

(p94) (v-Raf murine sarcoma viral oncogene homolog B1).

[Source:Uniprot/WISSPROT;Acc:P15056]

GATTAACTCATTTTGTGCTTCGTGTCTTTCATATTTTCCATTCATTTATCTCTGCCTTTTGGCTAATTTTCTCAGAATCA

CCTTCTAGTCCTTTAATTCT

In100 candidate1997 (in intron):

LOCUS ENSG00000114861

DESCRIPTION Forkhead box protein P1. [Source:Uniprot/SWISSPROT;Acc:Q9H334]

EXON

CCCTAACTGCTGGGCTATGTTACTTTTACTTCCAGGTTTTTTGTTTTAAATTTTCTCACTGTTAATGTCAGAATAGCCTG

AGATCTCTTTTTTTTTTTTC

In100 candidate1998 (in intron):

LOCUS ENSG00000114861

DESCRIPTION Forkhead box protein P1. [Source:Uniprot/SWISSPROT;Acc:Q9H334]

EXON

TTTTTTTTACAAAAAAATAGCTTTCAATTTGGTAAAAGCTATTCCTAGTAACGTTTTATAAAATCATCTTCTTTTCAGGG

TGTGTTTTATTTCCTTTCCA

In100 candidate1999 (in intron):

LOCUS ENSG00000182053

DESCRIPTION similar to Tripartite motif protein 49 (RING finger protein 18)(Testis-specific ring-finger protein) (LOC642569), mRNA [Source:RefSeq_dna;cc:XR_016180]

ACTTGGAGTGAAGTCTCAATGATAACTGGGAAATGTTTTTCTTCCTCTTTATCTAACTATATTACACTTATCCATCAGGC
TTCATTGTATTAATCTATCC

In100 candidate2000 (in intron):

LOCUS ENSG00000006530

DESCRIPTION Acylglycerol kinase, mitochondrial precursor (EC 2.7.1.94) (EC .7.1.107) (hAGK) (Multiple substrate lipid kinase) (Multi- substrate lipid inase) (MuLK) (HsMuLK). [Source:Uniprot/SWISSPROT;Acc:Q53H12]

CCTCCACAACTGGCTAATTTTAACTACTTTTTGATTTATCACTTTTAGACCTTATCTTTGACCTCTTTGTTATACAGGCA
TACCTCATTTTATTACACTT

In100 candidate2001 (in intron):

LOCUS ENSG00000127359

DESCRIPTION KIAA1147 (KIAA1147), mRNA [Source:RefSeq_dna;Acc:NM_001080392]

EXON

TTTTTCTTCTTTAATAAAATATTTAACTTTTGCATTCTGTTTTTTTCTTCTCCAGGAATACCCATGATGCATATGTTTTT
CTCCCATTGTCTTCCCTGTT

In100 candidate2002 (in intron):

LOCUS ENSG00000174473

DESCRIPTION polypeptide N-acetylgalactosaminyltransferase 17

[Source:RefSeqpeptide;Acc:NP_001030017]

ATTTGTTCTACTTAAAACAATGAGGATAACTTTAGATTACCGTATTGTCTCTAGTTTTCTACCCCATTTTTTTTCAGGTA

ACCTATATTTCATCCTTACT

In100 candidate2003 (in intron):

LOCUS ENSG00000177990

DESCRIPTION dpy-19-like 2 [Source:RefSeq_peptide;Acc:NP_776173]

ATGGCTATATACCTACAAAATATGCATTATGCCAATATATGTAATAACCATAAAATGTATTTTTTTCATTTTTTTCAGAT

TTCAGTTACCCTTAGTTTCA

In100 candidate2004 (in intron):

LOCUS ENSG00000152767

DESCRIPTION FERM, RhoGEF and pleckstrin domain-containing protein 1

(Chondrcyte- derived ezrin-like protein). [Source:Uniprot/SWISSPROT;Acc:Q9Y4F1]

CTTTAATCTGTTGCATGTAACATTTTCTTTGGATGTTCATCTGTCATTTTTCTTTTTTCTTGCAGCATCTTTGTAAAGGT

TTTTTTGTTTGTTTGTTTTT

In100 candidate2005 (in intron):

LOCUS ENSG00000163412

DESCRIPTION eukaryotic translation initiation factor 4E member 3

[Source:ReSeq_peptide;Acc:NP_775495]

AGTTATTGCTGTGTTGTTCTTTAACTACTGAGGTCCCTAACTGGTATTCCACTTTTTGGCCTCTTTCAGAGTCTTCTTAT

GTTTATTTTATATAATGTTC

In100 candidate2006 (in intron):

LOCUS ENSG00000163412

DESCRIPTION eukaryotic translation initiation factor 4E member 3

[Source:ReSeq_peptide;Acc:NP_775495]

AAAGTTTTGTTTTCTTTTGGGGTAGTTCCTTCTGAATGCTCCTGAAGTAACATTTAGAATACTATTTTTCTTCCCCAGCT

TTCAGTTAAGTTTTTCTTTT

In100 candidate2007 (in intron):

LOCUS ENSG00000164104

DESCRIPTION High mobility group protein B2 (High mobility group protein 2)

HMG- 2). [Source:Uniprot/SWISSPROT;Acc:P26583]

GAAAAAGGACCCCAATGCTCCTAAAAGGCCACCGTAAGTTTAAAATAACCCAAATTGCTCCTTGGATTTTTCCTTCAGTT

TATTAAACTCTGTTGCTTCC

In100 candidate2008 (in intron):

LOCUS ENSG00000008869

DESCRIPTION HEAT repeat containing 5B

[Source:RefSeq_peptide;Acc:NP_061897]EXON

CCCAAGACGCTGTTAGGTTTTGGTTCCTCTCCCACTTCACTAACTACTACTTCTTGTTCTTTTTTAACTCTTCCTCAGTT

CAGGGCATTTTTTTTTTTTT

In100 candidate2009 (in intron):

LOCUS ENSG00000008869

DESCRIPTION HEAT repeat containing 5B

[Source:RefSeq_peptide;Acc:NP_061897]EXON

TTGACCTTTCTTTCTTTGGATTTCTTAACTGTTATTTTTCTTCCTCATTGTGTCTTGTCCTTTTACCTTAGTTCTCAGAG

ATCCTCACAGGTTTTTTCTT

In100 candidate2010 (in intron):

LOCUS ENSG00000179087

DESCRIPTION Maltase-glucoamylase, intestinal [Includes: Maltase (EC 3.2.1.2)

(Alpha-glucosidase); Glucoamylase (EC 3.2.1.3) (Glucan 1,4-alpha- glucosiase)].

[Source:Uniprot/SWISSPROT;Acc:O43451]

GTAACTCTTTCCAAAATATGTGCCTCTTTCTGGCCCACTTTAATTCTCTCTGCCTTCGTGGTTGTTATTCTTCACCAGAA

TGACTTGATCTCCTTTCTTT

In100 candidate2011 (in intron):

LOCUS ENSG00000196935

DESCRIPTION SLIT-ROBO Rho GTPase-activating protein 1 (srGAP1) (Rho

GTPaseactivating

protein 13). [Source:Uniprot/SWISSPROT;Acc:Q7Z6B7]

TGTTTGTTCCATGACATGTCGTTTCTTCCTAACTGGTCTGTAGCTTCTTTCCTTTCTATCTCTTTTAGATTGCTGCAGAT

TCACATTTATCATGTTTCTT

In100 candidate2012 (in intron):

LOCUS ENSG00000163602

DESCRIPTION RING1 and YY1-binding protein (Death effector

domainassociatedfactor)

(DED-associated factor) (YY1 and E4TF1-associated factor 1)

(Apopti-associating protein 1) (APAP-1). [Source:Uniprot/SWISSPROT;Acc:Q8N488]

CTGAAGTTTTTTCCCTCAAGTTCTGCTTTAGTTGTAACCCACAAATTTTGATATTTTTGTGTTTTCATTTTCATTCAGTT

TCAGATGCTTTCTAATTTTT

In100 candidate2013 (in intron):

LOCUS ENSG00000006837

DESCRIPTION Cyclin-dependent kinase-like 3 (EC 2.7.11.22)

(Serine/threonineprotein kinase NKIAMRE). [Source:Uniprot/SWISSPROT;Acc:Q8IVW4]

CTAACATTTCCATCACTCTCCAAAATTTCATTGTGTCCTTTTACAATTCATCTTTTCTTCCATTTCTGTCCCCAGATAAC

CACTGATTTGCTTTCTATTC

In100 candidate2014 (in intron):

LOCUS ENSG00000103194

DESCRIPTION Ubiquitin carboxyl-terminal hydrolase 10 (EC 3.1.2.15) (Ubiquitn

thioesterase 10) (Ubiquitin-specific-processing protease 10) (Deubiquitinting

enzyme 10). [Source:Uniprot/SWISSPROT;Acc:Q14694]

TTAAAAATATCTTTAGGAAATTCTTCCCATCCTTTAGATAACGAAGATATTTGTCTGTTTTCTTCTAAAAGTTTTCAGAT

TTTGTTTTCACGCTTCAGTG

In100 candidate2015 (in intron):

LOCUS ENSG00000119048

DESCRIPTION Ubiquitin-conjugating enzyme E2 B (EC 6.3.2.19) (Ubiquitin-protin ligase B) (Ubiquitin carrier protein B) (HR6B) (hHR6B) (E2-17 kDa). [Souce:Uniprot/SWISSPROT;Acc:P63146]

CCACTCTCTGTTATGGTTTGCTCCATGAAATGGTATCTGTAACATTTAAATGAATTTACTCTTTGTTTTACATAGCAGTTCACTTCTAGAAATTTTTTCT

In100 candidate2016 (in intron):

LOCUS ENSG00000102572

DESCRIPTION Serine/threonine-protein kinase 24 (EC 2.7.11.1) (STE20-like kiase MST3) (MST-3) (Mammalian STE20-like protein kinase 3). [Source:Uniprot/WISSPROT;Acc:Q9Y6E0]

TCTATTTCCCTAGGATAAACTAACTGGAAGTGGGGTTTTTGCCTTTTGCTTATTTTCATTTTCAGGTATGGAACATCTTTTCTGATCACTTTTCCATCTT

In100 candidate2017 (in intron):

LOCUS ENSG00000172986

DESCRIPTION similar to CG9996-PA [Source:RefSeq_peptide;Acc:NP_001073862]

GAAGTTGTTTTGATGATAACAATTTCACACTTTCTCTAGCTTTTTCACATTTCTCTCTCTCTTCTTGCTTATAAACAGTTTCTCTCTATATACCTTTTTC

In100 candidate2018 (in intron):

LOCUS ENSG00000163605

DESCRIPTION protein phosphatase 4, regulatory subunit 2 [Source:RefSeq_peptde;Acc:NP_777567]

AGCCAAATACTACCTTTATGAATTTTTCCACATTCTTGTAACATCTGTACAATTACTTCTTTAATGCTTTTTTTCAGGTCAGCTGAGTTTTTTTCCCTCA

In100 candidate2019 (in intron):

LOCUS ENSG00000183735

DESCRIPTION Serine/threonine-protein kinase TBK1 (EC 2.7.11.1) (TANK-bindin

kinase 1) (T2K) (NF-kappa-B-activating kinase).

[Source:Uniprot/SWISSPROT;cc:Q9UHD2]

AGCCTAACCTGTCCAACCATTCTTTTAGGTCTTAACATATTTCTCAGACCTTTATATATTTGGCCCTATCTTTTGTTTTG

TTTGTTTTGTTTTTTTGAGA

In100 candidate2020 (in intron):

LOCUS ENSG00000121440

DESCRIPTION PDZ domain-containing RING finger protein 3 (Ligand of Numbproein

X 3) (Semaphorin cytoplasmic domain-associated protein 3) (Protein

SEMAAP3). [Source:Uniprot/SWISSPROT;Acc:Q9UPQ7]

AGTTCTCATGACTGCCTTTGTAACACTTGTCTGTTTTCCATTTTTCCTTATGTCTTCACCAGCATCTGTTGTCCTGCTTT

TTGTTTTATCTTTTTCTTTC

In100 candidate2021 (in intron):

LOCUS ENSG00000121440

DESCRIPTION PDZ domain-containing RING finger protein 3 (Ligand of Numbproein

X 3) (Semaphorin cytoplasmic domain-associated protein 3) (Protein

SEMAAP3). [Source:Uniprot/SWISSPROT;Acc:Q9UPQ7]

AGCACTCTTGATAAAGTAAAGATAATTAGAAATTATAGTCAAGTAATAACCTTTTCCATTTATCTGGTTTTCTTCAGAGC

TTTATCCCTTATCTGATCTT

In100 candidate2022 (in intron):

LOCUS ENSG00000113805

DESCRIPTION Contactin-3 precursor (Brain-derived immunoglobulin

superfamilyprotein 1) (BIG-1) (Plasmacytoma-associated neuronal glycoprotein).

[SourceUniprot/SWISSPROT;Acc:Q9P232]

AACAAAGGTAAAATCATATTACCACAATGTCAAGATAACCCTCATTTTGGTCTATTTATTCTCAGTCTTATTTTTTTCCT

TACACATTTTCCCCATAATT

In100 candidate2023 (in intron):

LOCUS ENSG00000150625

DESCRIPTION Neuronal membrane glycoprotein M6-a (M6a).

[Source:Uniprot/SWISPROT;Acc:P51674]

AAAGTCTTACAAATACTATATTTTAACATTTAGTATTATCTCTGCTCCTGTAAGTATTTTTAGTTTCAGAGCTCTACTTT

CTTACTCAACTAAATATTTT

In100 candidate2024 (in intron):

LOCUS ENSG00000150625

DESCRIPTION Neuronal membrane glycoprotein M6-a (M6a).

[Source:Uniprot/SWISPROT;Acc:P51674]

AGGCGTGAGCCACTGCACACTGCCAAGTTTATTTTTTAACCATAGTTTGAACTGCTTAGGCTTATTTCTCAGAGTTTATT

TATTTATTTGTCTTGCATCT

In100 candidate2025 (in intron):

LOCUS ENSG00000150625

DESCRIPTION Neuronal membrane glycoprotein M6-a (M6a).

[Source:Uniprot/SWISPROT;Acc:P51674]

ACTCATTTATATAACTCATTTATAGTCATTTGTATAACTTTTAGTACTTCTCTGTCTCTTGTCTATTTTCTTCCCCAGAA

GCTTTTAATCCCTTAATTTT

In100 candidate2026 (in intron):

LOCUS ENSG00000080608

DESCRIPTION KIAA0020 (KIAA0020), mRNA [Source:RefSeq_dna;Acc:NM_014878]

TATCCATTAGTTAAATCTCTTAGTTTTAACTTTTTATTAACTCTCTTCCCTCTTCACTAGCTTTTAGGTTCTATGCAGTA

GTCTGCATTTCTTTTTGTCC

In100 candidate2027 (in intron):

LOCUS ENSG00000156076

DESCRIPTION Wnt inhibitory factor 1 precursor (WIF-1).

[Source:Uniprot/SWISPROT;Acc:Q9Y5W5]

TTTGTTTGGGTCTTTAACATTGAACTATTTTCCTTTCTTGCTTATTTTAGATTTTTTCTGGCCCTTCACTTACAGCGTAC

GATCTATTTTACATCTCTTT

In100 candidate2028 (in intron):

LOCUS ENSG00000150627

DESCRIPTION WD repeat protein 17. [Source:Uniprot/SWISSPROT;Acc:Q8IZU2]

GTTCTCCTTTGACATTTAACATATTATTTTCTCTATTATTTTTATTTATTGTATCTCTTTGCTTGGTTGTCTTTTCAGAC

TCCTTTTTCTTTCCTGTACT

In100 candidate2029 (in intron):

LOCUS ENSG00000174106

DESCRIPTION Inner nuclear membrane protein Man1 (LEM domain-containing protin

3). [Source:Uniprot/SWISSPROT;Acc:Q9Y2U8]

ATATAAACTATATATGTATATAAGTGAAGTCATAACTTTTTAACTGCTCTTTTTTTTCCAGTTTTTGCCTGCCTCCTCTT

ACTAAGACCTTTCTATTTAT

In100 candidate2030 (in exon):

LOCUS ENSG00000196454

DESCRIPTION

CGTCATCAATAACATATTCATATATTTCGATAGTACTATGTTTGGTTTTCTTCCCATTTCAGGGATCCTTTTGTCTTACT

ATAAAATTGTCCCCTCCATT

In100 candidate2031 (in intron):

LOCUS ENSG00000046653

DESCRIPTION Neuronal membrane glycoprotein M6-b (M6b).

[Source:Uniprot/SWISPROT;Acc:Q13491]

AAAAAAAAAAAGCACCCTCTCGTATTTCATTTTTGCTTTTTCTTAACAATACCTTTGGCCATTTTTTTCCAGTTCACTAT

GTTTGTATACTAACTTTTCT

In100 candidate2032 (in intron):

LOCUS ENSG00000174099

DESCRIPTION methionine sulfoxide reductase B3 isoform 1

[Source:RefSeq_peptde;Acc:NP_932346]

GTCCATTTCTAATAGAGGGGTGTTGAAGTCTCTAACTATAATAATGGATTTGTCTGTTTCTCTTTGAAGTTCTATCAGTT

TTTGCCTCGTGTATTTTGAT

In100 candidate2033 (in intron):

LOCUS ENSG00000174099

DESCRIPTION methionine sulfoxide reductase B3 isoform 1

[Source:RefSeq_peptde;Acc:NP_932346]

TTTCTTTTATTCTCTCTGACTGTATTTTTTAAGATAACTTGTCTTTGAGTTCATAGATTCTTTCTTCTGCTTAATCAGTT

CTTTTTACTTCTCTATGGGT

In100 candidate2034 (in intron):

LOCUS ENSG00000185736

DESCRIPTION Double-stranded RNA-specific editase B2 (EC 3.5.-.-) (dsRNA

adeosine deaminase B2) (RNA-dependent adenosine deaminase 3) (RNA-editing

deamnase 2) (RNA-editing enzyme 2). [Source:Uniprot/SWISSPROT;Acc:Q9NS39]

GTTCTCAATACTCCCTGCCCCTTTAACATCTTGACCCACACTAGCTTTTTTATCATTCTGTGCTTCCAGGCTCTCTGAGA

CCCTATCAGATTTATTCTTT

In100 candidate2035 (in intron):

LOCUS ENSG00000185736

DESCRIPTION Double-stranded RNA-specific editase B2 (EC 3.5.-.-) (dsRNA adeosine deaminase B2) (RNA-dependent adenosine deaminase 3) (RNA-editing deamnase 2) (RNA-editing enzyme 2). [Source:Uniprot/SWISSPROT;Acc:Q9NS39]

CTTATCATATCCCCTTTATTTTTAAATTCTTAAAAATATTTCTAACATCTATTTAATGTCCTTATCTGCTAATTTCAGCA
TCATTAGATCTTTTTACTTC

In100 candidate2036 (in intron):

LOCUS ENSG00000185044

DESCRIPTION Zinc finger protein 717. [Source:Uniprot/SPTREMBL;Acc:Q5XG89]

CTCTAGATAATGTTTAATATTTTCTCACATGACACATCTGCTGGTAACAAGTTTCCTCAACTTTTGTTTGTCCAGGGAAT
GTTTTATTTCTCATTTACTT

In100 candidate2037 (in intron):

LOCUS ENSG00000080298

DESCRIPTION Transcription factor RFX3. [Source:Uniprot/SWISSPROT;Acc:P48380

CTTTAACACACTTTCATCATTGTGTTTTATTTTTGTTTTCTATTTTGAGCGCTTCGCTTTCCTACACTGCAGGATGCTCC
AGATTCGCCTTTTATATTTC

In100 candidate2038 (in intron):

LOCUS ENSG00000080298

DESCRIPTION Transcription factor RFX3. [Source:Uniprot/SWISSPROT;Acc:P48380

TATCAACTATGGAAAAAAGAATGCTGAAGTCTCCAATTGTAACTGGATTTTTAAATTTTCTTCTTTCCGTTATATCAGTT
TTTGCTTCATGTCTTCTGAA

In100 candidate2039 (in intron):

LOCUS ENSG00000080298

DESCRIPTION Transcription factor RFX3. [Source:Uniprot/SWISSPROT;Acc:P48380

AAGAAAACAAAAAATCCCTATAACTTACCTAAAAATTTATTATTTCTGATGTTTCTCATTTCTTTATGTAGATCTCAGTT

TCCATCTAGTATTCTTTTTC

In100 candidate2040 (in intron):

LOCUS ENSG00000169855

DESCRIPTION Roundabout homolog 1 precursor (H-Robo-1) (Deleted in U twenty

wenty). [Source:Uniprot/SWISSPROT;Acc:Q9Y6N7]

TGAGGAAGGAGTAACTTCTTCTGGAATGAGAAGTTTCTCACTTCTTCCTCTTCTTTCCAGTCACATCATTTCTTTCTTCC

CTTAGCGGCATTTCAATTTT

In100 candidate2041 (in intron):

LOCUS ENSG00000155957

DESCRIPTION Transmembrane BAX inhibitor motif-containing protein 4 (Z-proten)

(S1R protein). [Source:Uniprot/SWISSPROT;Acc:Q9HC24]

GCAAGTTGTTTATCCTTATAGTTAATATATTTAACTCTTCCTACTTTGTCATTAGTTTTTTTAACCTTTCTATGCCAGTT

TTGTTTAATCTGTATGTTTT

In100 candidate2042 (in intron):

LOCUS ENSG00000090376

DESCRIPTION Interleukin-1 receptor-associated kinase 3 (EC 2.7.11.1) (IRAK-)

(IL- 1 receptor-associated kinase M) (IRAK-M).

[Source:Uniprot/SWISSPROT;cc:Q9Y616]

ATAACCCCCTCAAAAAGCCTATTTTTATCTTTCAGAAAAATTTTAGATATATATAATTCTTTTTCCTCAGATTTTTCCTG

TTTTCCCCCAGTGCCTCCCC

In100 candidate2043 (in intron):

LOCUS ENSG00000159763

DESCRIPTION Prolactin-inducible protein precursor (Prolactin-induced protei)

(Secretory actin-binding protein) (SABP) (Gross cystic disease fluid protin 15)

(GCDFP-15) (gp17). [Source:Uniprot/SWISSPROT;Acc:P12273]

AGGCTGTAATTAGTAGTTAGTCTTCATCTAACATGCTGATCCACATCTTGTTCATAATTTATACCTTTGTTCAGATTTCT

GCTCAGTAAACTTTACCTTC

In100 candidate2044 (in intron):

LOCUS ENSG00000175161

DESCRIPTION immunoglobulin superfamily, member 4D

[Source:RefSeq_peptide;Ac:NP_694854]

ATGTTAACTTAACTCATAATCTTTATATGGCCTTTTCTTTTCACCTCTATTGCCATAACCTTGTTTGAATCCTTGCAGTC

TTTCATCTTTACTACTGCTA

In100 candidate2045 (in intron):

LOCUS ENSG00000109716

DESCRIPTION Teneurin-3 (Ten-3) (Tenascin-M3) (Ten-m3) (Protein Odd

Oz/tenmhomolog

3). [Source:Uniprot/SWISSPROT;Acc:Q9P273]

TTTTACCTATATTACTACTTTTAGTCAAGTAATTCTAATATAAAGTTAACAAAAATAATTCAATTCTACTATCTTCAGGT

GTTTTTAATTGCTCTAACTT

In100 candidate2046 (in intron):

LOCUS ENSG00000109716

DESCRIPTION Teneurin-3 (Ten-3) (Tenascin-M3) (Ten-m3) (Protein Odd

Oz/tenmhomolog

3). [Source:Uniprot/SWISSPROT;Acc:Q9P273]

GGTCTATGTAGTATTCCCCTCTTACTGCTACTGTTTTTATAACTAGTTGTATTATTTTCTGCTGATTTGCAGATGGGTTT

CGTGCATTTTCTAATTTCCT

In100 candidate2047 (in intron):

LOCUS ENSG00000109716

DESCRIPTION Teneurin-3 (Ten-3) (Tenascin-M3) (Ten-m3) (Protein Odd

Oz/tenmhomolog

3). [Source:Uniprot/SWISSPROT;Acc:Q9P273]

CACTAACCTTAAATCTGGCTTTATCTTATGTTTCACACTTTTATTTTTCTTGTATTCTATCTTTATTTTTGGCAGTGTTG

TACTTTATCTTGTTACACTT

In100 candidate2048 (in intron):

LOCUS ENSG00000167258

DESCRIPTION Cell division cycle 2-related protein kinase 7 (EC 2.7.11.22)

(DC2- related protein kinase 7) (Cdc2-related kinase, arginine/serine-rich)

CrkRS). [Source:Uniprot/SWISSPROT;Acc:Q9NYV4]

TTACACTGCTGTTACCTACTTTTACTAACTTTTTTGGTTATTTTTGTTTTCCTTATTCTTTACATTTCCCACAGTCTTTG

CCTTCCCATTTTAATCCTTT

In100 candidate2049 (in intron):

LOCUS ENSG00000154118

DESCRIPTION Junctophilin-3 (Junctophilin type 3) (JP-3).

[Source:Uniprot/SWSSPROT;Acc:Q8WXH2]

GCAATGGAGTCTGATTCTGTACACTTCTCATGCATAACTTGAGTTAGTAATTGTCTCGCTTTTTTTCAGTTGCAATTTTC

TTTAAATCACTGGTTCTTTT

In100 candidate2050 (in intron):

LOCUS ENSG00000107249

DESCRIPTION Zinc finger protein GLIS3 (GLI-similar 3) (Zinc finger protein

15). [Source:Uniprot/SWISSPROT;Acc:Q8NEA6]

TGAAATTATATCACCCTTTCATTCCTATTATTATTAACACATGTCATCTTTCTTTTTCTTTATTAGCCTAGGCAGCAATT

TGTTAACTTTATTATTCTTC

In100 candidate2051 (in intron):

LOCUS ENSG00000107249

DESCRIPTION Zinc finger protein GLIS3 (GLI-similar 3) (Zinc finger protein

15). [Source:Uniprot/SWISSPROT;Acc:Q8NEA6]

ATGCATTTTGTAGTTTTAATTTTCCACAAATTATTCTGTAATTTAACTAAAGAAGACTTCCTTTTTCTTCAGGAAAGTCC

TTCTTGACTGTTTCTTGCTT

In100 candidate2052 (in intron):

LOCUS ENSG00000107249

DESCRIPTION Zinc finger protein GLIS3 (GLI-similar 3) (Zinc finger protein

15). [Source:Uniprot/SWISSPROT;Acc:Q8NEA6]

GTTAAGGTATTTAGGATGTCCCACAACCAAGCACAATATGTTTTTTATTTAACTATAGTCACCTTATTCTGTTACCAGAC

ATTGAATTTATTCCTTCTAT

In100 candidate2053 (in intron):

LOCUS ENSG00000106144

DESCRIPTION Caspase-2 precursor (EC 3.4.22.-) (CASP-2) (ICH-1 protease) (IC-1L/1S) [Contains: Caspase-2 subunit p18; Caspase-2 subunit p13; Caspase-2subunit p12]. [Source:Uniprot/SWISSPROT;Acc:P42575]

CTTCCTTTCTGAGAACTCTTACTCTTTTCTGTGCTTCATTAACTCTGGTGCCCTTTTTTTGGTTACTCATTCCAGTTACG

GATTTTTGATCTGTCTTTTT

In100 candidate2054 (in intron):

LOCUS ENSG00000134882

DESCRIPTION phosphoglycerate dehydrogenase like 1 [Source:RefSeq_peptide;Ac:NP_808882]

AGTGTCAACATTCCTGTGGTTTGATATTTCTTTTATAACCTGATAACATTTGATTCACATTTCACTTCCAGCACTATTAC

TTTTTGTTGCTTTGCTGTAT

In100 candidate2055 (in intron):

LOCUS ENSG00000184500

DESCRIPTION Vitamin K-dependent protein S precursor.

[Source:Uniprot/SWISSPOT;Acc:P07225]

CATGACCTCACACAAACATTAAGCAATAACCTGTGCATTTTGATTTTCTTGTTGTTTATTTGGTTTCTTTTATTCCAGGT

TGTTTCAGTGTGCCTTCCCT

In100 candidate2056 (in intron):

LOCUS ENSG00000184500

DESCRIPTION Vitamin K-dependent protein S precursor.

[Source:Uniprot/SWISSPOT;Acc:P07225]

ACACATTTTCTTTCTGTAGAATCTTCTGGTAAATGTTCTATAACGTGCTGGTGGCAATCCCTGGTTTTTCTTTCTCAGGG

CCTCTTCATTTATTTTATCC

In100 candidate2057 (in intron):

LOCUS ENSG00000166693

DESCRIPTION Seven transmembrane helix receptor.

[Source:Uniprot/SPTREMBL;Ac:Q8NH86]

TGTTTTCTTATAATTTCTTTTTTTAACATTTTTCTTTCTTTAAATATTTTTTTAAATTCCTTATTTCCTCAGAGATCTTT

GTTCTGACCTGTAATTTCTT

In100 candidate2058 (in intron):

LOCUS ENSG00000127314

DESCRIPTION Ras-related protein Rap-1b precursor (GTP-binding protein smg

p1B). [Source:Uniprot/SWISSPROT;Acc:P61224]

TTCTTCATAACCACCCCATATAAAACCATCCATTCTTATCTCTTTTTTTTTTCTCCATACTGCTTATCACAGCTGACACT

TTATACTGACTTTGTTTCCC

In100 candidate2059 (in intron):

LOCUS ENSG00000127314

DESCRIPTION Ras-related protein Rap-1b precursor (GTP-binding protein smg p1B). [Source:Uniprot/SWISSPROT;Acc:P61224]

AGCCGCCTTTAACTTCTTGAAGGTTGTTCATCTTAAAATTTCTTTCAGAGTTTTTCTGTTGATTATAGTTTTTGTTTTTC
TTCCAAAATCTCTTCACTTT

In100 candidate2060 (in intron):

LOCUS ENSG00000080224

DESCRIPTION Ephrin type-A receptor 6 precursor (EC 2.7.10.1) (Tyrosine-protin kinase receptor EHK-2) (EPH homology kinase 2).

[Source:Uniprot/SWISSPRO;Acc:Q9UF33]

GATTCTTCTAACCCATGAACATGGAATATTTTTCCATTTTTTGTTGTAATCTTCATTTTCCTTCATCAGTGTTTTATAGT
TTTCATTATAGATCTCCTTC

In100 candidate2061 (in intron):

LOCUS ENSG00000080224

DESCRIPTION Ephrin type-A receptor 6 precursor (EC 2.7.10.1) (Tyrosine-protin kinase receptor EHK-2) (EPH homology kinase 2).

[Source:Uniprot/SWISSPRO;Acc:Q9UF33]

TTTAACTTTTCAAAAAATGAGCTTTCTGTTTCATTGATCTTTTGTATTTTTTTTTCATTTCAATTTCATTTATTTCAGCT
GTGATCTTTATTGTTTCTTC

In100 candidate2062 (in intron):

LOCUS ENSG00000080224

DESCRIPTION Ephrin type-A receptor 6 precursor (EC 2.7.10.1) (Tyrosine-protin kinase receptor EHK-2) (EPH homology kinase 2).

[Source:Uniprot/SWISSPRO;Acc:Q9UF33]

ATAATTTTTAGTCTTTGGGGACATATTTCTTTCTTCTAACTTGTTTCTTTATGCATATCATTTTTTCCCTGCTACCAGAT

TCTAAGATTTCTTTGCTTTC

In100 candidate2063 (in intron):

LOCUS ENSG00000080224

DESCRIPTION Ephrin type-A receptor 6 precursor (EC 2.7.10.1) (Tyrosine-protin kinase receptor EHK-2) (EPH homology kinase 2).

[Source:Uniprot/SWISSPRO;Acc:Q9UF33]

TTACTTTCCCAATTTGTAACTATAAATATTTTCTTACATTGTTTTGTATCCTTTATTTTCTTTCCCCAGCTATTGATTGT

TTCTTCTGGGACTCTATTTC

In100 candidate2064 (in intron):

LOCUS ENSG00000080224

DESCRIPTION Ephrin type-A receptor 6 precursor (EC 2.7.10.1) (Tyrosine-protin kinase receptor EHK-2) (EPH homology kinase 2).

[Source:Uniprot/SWISSPRO;Acc:Q9UF33]

TAACCTAAAAATTAATCTCCTCTTCATTTTTATTTCATGTTTATTTCTCGACAAGCCTGTTCCACTATCCAGTATTTATG

TTTGCCAGCAATTCTTTTCT

In100 candidate2065 (in intron):

LOCUS ENSG00000198420

DESCRIPTION

AATAACTCTCCCACAATCTTTCTATTATATCTATTTGAACCTCAGATCAGATGTGTCTTAGGTCTTCTCACTATATGCTC

TATATCGTCTATCCTTTTTC

In100 candidate2066 (in intron):

LOCUS ENSG00000130234

DESCRIPTION Angiotensin-converting enzyme 2 precursor (EC 3.4.17.-) (ACEreated carboxypeptidase) (Angiotensin-converting enzyme homolog) (ACEH). [Souce:Uniprot/SWISSPROT;Acc:Q9BYF1]

GCATTAGACAAGATTTACAAGTTGTCTTTCAAATGCTTTTTTTTTTTAACCGACTTCCACATTGTCATTCTTTTCCAGAT

TTCCTTGACCTCTCTGATTG

In100 candidate2067 (in intron):

LOCUS ENSG00000125246

DESCRIPTION citrate lyase beta like [Source:RefSeq_peptide;Acc:NP_612124]

AGTTATGTATTGTAACATACTGCATCTTTTGACCCTGTCTTCTCTGTTTCAACAGAGCTTATCTTTTTCTATGTATTTCT

GCTGCTACTGCTTTTCCAAA

In100 candidate2068 (in intron):

LOCUS ENSG00000196511

DESCRIPTION Thiamin pyrophosphokinase 1 (EC 2.7.6.2) (Thiamine pyrophosphoknase 1) (hTPK1) (Placental protein 20) (PP20). [Source:Uniprot/SWISSPROT;Ac:Q9H3S4]

TCTATATCTAGTATACACCTAGGAATAAAATTGCCATATGTTTTTATTGTAACTCCATGTTTAATCATTTATTGCCAGAT
TGTTTTCTCCTCTATTCAGA

In100 candidate2069 (in intron):

LOCUS ENSG00000167635

DESCRIPTION Zinc finger protein OZF (Only zinc finger protein) (Zinc fingerprotein 146). [Source:Uniprot/SWISSPROT;Acc:Q15072]

GGGAGCCACTACACCTGGACTCATTCTTAAGTTATAATTAACTATAATATCAATTCTTATTTTGGCATCTCCAGAGTTTC
TGTCTTGCCTTACACAACTT

In100 candidate2070 (in exon):

LOCUS ENSG00000197621

DESCRIPTION Olfactory receptor 5H6. [Source:Uniprot/SWISSPROT;Acc:Q8NGV6]

ACTGTGACATTATCCCATTGTTAAAGATTTCCTGTACTGATTCCTCTATTAACTTTCTAATGGTTTTTATTTTCGCAGGT
TCTGTTCAAGTTTTTACCAT

In100 candidate2071 (in intron):

LOCUS ENSG00000174469

DESCRIPTION Contactin-associated protein-like 2 precursor (Cell recognitionmolecule Caspr2). [Source:Uniprot/SWISSPROT;Acc:Q9UHC6]

CTATGAGATTGCTTCAAAGTTACTAACTCAAGATATATTTGAGATTTTTCCATTTTGCCCCTTCTGCAGATAGCTGATGT
AATTTCCTGCATTTCTTCTT

In100 candidate2072 (in intron):

LOCUS ENSG00000174469

DESCRIPTION Contactin-associated protein-like 2 precursor (Cell recognitionmolecule Caspr2). [Source:Uniprot/SWISSPROT;Acc:Q9UHC6]

CCCTCATTTCACTGTTTCAATAAACAATACATTTACTAACTAGTTCATTGCTTCTCTGTTACCAGACTATCTCTCAACAA
TTATTTCAACCATTGCTTTT

In100 candidate2073 (in intron):

LOCUS ENSG00000174469

DESCRIPTION Contactin-associated protein-like 2 precursor (Cell recognitionmolecule Caspr2). [Source:Uniprot/SWISSPROT;Acc:Q9UHC6]

ATAATTTAATTAATTTACATTCAAAGTAATTATTGATAGGTAATAACTTACTACACTATATTGTTCATTGTTTCCCAGTT
GTTTTGAAGATTCTTTCTTC

In100 candidate2074 (in intron):

LOCUS ENSG00000174469

DESCRIPTION Contactin-associated protein-like 2 precursor (Cell recognitionmolecule Caspr2). [Source:Uniprot/SWISSPROT;Acc:Q9UHC6]

AGTATACTGTTAACCACATCAAAGTTACTTTTATTTCTGTTACAGTGTTTTTTAGCTCTAGCATTTCTCTTTAGCTCTTA
AGATTTCCATCTCTCTGCTT

In100 candidate2075 (in intron):

LOCUS ENSG00000174469

DESCRIPTION Contactin-associated protein-like 2 precursor (Cell recognitionmolecule Caspr2). [Source:Uniprot/SWISSPROT;Acc:Q9UHC6]

AGGAAATTAACATTATTTCACTAGTTCTTTCTAATCCTCAGATTCCATTTGAGTTTCACCAGTTGTCCCAAGAGTATTCT
TTTTTTTTTTTTTTTTTTTT

In100 candidate2076 (in intron):

LOCUS ENSG00000174469

DESCRIPTION Contactin-associated protein-like 2 precursor (Cell recognitionmolecule Caspr2). [Source:Uniprot/SWISSPROT;Acc:Q9UHC6]

AGACATATTTAGAATTTTTCTAACTTGTAGTGAATTTTACCTTTTTTCATTGTATACTAACCTTCCTTATCTCTCAGAGC
GCCTTTTTCCTTGTGTTGTC

In100 candidate2077 (in intron):

LOCUS ENSG00000174469

DESCRIPTION Contactin-associated protein-like 2 precursor (Cell recognitionmolecule Caspr2). [Source:Uniprot/SWISSPROT;Acc:Q9UHC6]

CTGACCTATATCATTTTTGTTTCCTCTGAATAACTTCATTTAACATTTTTTTTGCAAGTCAGATCTACTGGCAACAAATT
TCCTCTATCTTTATTTGTCT

In100 candidate2078 (in intron):

LOCUS ENSG00000174469

DESCRIPTION Contactin-associated protein-like 2 precursor (Cell recognitionmolecule Caspr2). [Source:Uniprot/SWISSPROT;Acc:Q9UHC6]

CTATTGCTTCTATAACTCCATTTACATTCATTTCACATTTCACTTTCAGCATCATCACTCTCTGTTTCTTGATGCACTTT
TATCTTAGTTAGCCAATTCT

In100 candidate2079 (in intron):

LOCUS ENSG00000094880

DESCRIPTION Cell division cycle protein 23 homolog (Anaphase-promoting compex subunit 8) (APC8) (Cyclosome subunit 8). [Source:Uniprot/SWISSPROT;Acc:QUJX2]

AGAATGCTACCTTTTTAACCCTTGAGGTACCTGTTTTTTTTTTTTGAGACAGAATCATACCTCTTTTTTTCCTTTTTTTT

TAAGATGTTGTTTTGCTCTA

In100 candidate2080 (in intron):

LOCUS ENSG00000181007

DESCRIPTION Zinc finger protein 545. [Source:Uniprot/SWISSPROT;Acc:Q8N141]

EXON

GCTTTATTACTTCTATTTCTGTATTCTAACTAATTCTGCTTTTATATCGGTTAATATTTTTCTTCTGCTTTACTTCAGCT

TAATTTTATTTTCTTTTTTT

In100 candidate2081 (in intron):

LOCUS ENSG00000088812

DESCRIPTION Attractin precursor (Mahogany homolog) (DPPT-L). [Source:Unipro/SWISSPROT;Acc:O75882]

TGTACCTAACCCTTCACCTCATTGCTGACCTGCTTCTGTTGCACTTTTACTGTTTTCTTACACCTGCAGAGTCTGCTTCA

CACTTTGTCATATCAGTCTT

In100 candidate2082 (in intron):

LOCUS ENSG00000088812

DESCRIPTION Attractin precursor (Mahogany homolog) (DPPT-L).

[Source:Unipro/SWISSPROT;Acc:O75882]

CTTTCTCTCTCCCTCTCCTGTTCTCCTTCTTAACTCTAATATCTTCTTTTCATTTTTCAGATTCCTACCACCTTTCCTTC

TGTTTTCATTCTCAGTTAAT

In100 candidate2083 (in intron):

LOCUS ENSG00000175198

DESCRIPTION Propionyl-CoA carboxylase alpha chain, mitochondrial precursor EC

6.4.1.3) (PCCase subunit alpha) (Propanoyl-CoA:carbon dioxide ligase subnit

alpha). [Source:Uniprot/SWISSPROT;Acc:P05165]

TTGGCTAACAAAGTTCCCATCCATCTTTGCTTTAGTTCAATTGCCATTAGTTTCCCAGAGTACTTTCTAATTCCTCTACC

ACAGTACTCATGCTTTATTT

In100 candidate2084 (in intron):

LOCUS ENSG00000175198

DESCRIPTION Propionyl-CoA carboxylase alpha chain, mitochondrial precursor EC

6.4.1.3) (PCCase subunit alpha) (Propanoyl-CoA:carbon dioxide ligase subnit

alpha). [Source:Uniprot/SWISSPROT;Acc:P05165]

CTTATAATAGTATTTGTAACAACTGTTTTAAGAGTCTTCATTTCTGGGTTTCTTTCTGTCAGCTATTTTCCTTCTGATAT

GAATCACATTTTGCATCTTT

In100 candidate2085 (in intron):

LOCUS ENSG00000175198

DESCRIPTION Propionyl-CoA carboxylase alpha chain, mitochondrial precursor EC

6.4.1.3) (PCCase subunit alpha) (Propanoyl-CoA:carbon dioxide ligase subnit alpha). [Source:Uniprot/SWISSPROT;Acc:P05165]

AGATGAGGTGAGGGGTAACAAGTACCATCAACTTGATCATTTCTGATTTTTCCAGGAATGCCAACTTTTGACTAAATATTTTTTTCCTTCCACTTCCCAT

In100 candidate2086 (in intron):

LOCUS ENSG00000175198

DESCRIPTION Propionyl-CoA carboxylase alpha chain, mitochondrial precursor EC 6.4.1.3) (PCCase subunit alpha) (Propanoyl-CoA:carbon dioxide ligase subnit alpha). [Source:Uniprot/SWISSPROT;Acc:P05165]

AAAGTAGGGATGAGGGACTGAACTGTTGGATATTATTTAACATTATCTGTGTGGATTATTCTTTTCCCTCCTCTTCAGATTTCTAAGTATTGTTTCTCCA

In100 candidate2087 (in intron):

LOCUS ENSG00000175198

DESCRIPTION Propionyl-CoA carboxylase alpha chain, mitochondrial precursor EC 6.4.1.3) (PCCase subunit alpha) (Propanoyl-CoA:carbon dioxide ligase subnit alpha). [Source:Uniprot/SWISSPROT;Acc:P05165]

ACATATTGACATATTAATTCTTTGAAACGAAATTTAGAACTCATATCTAACAACCCCTCCCCTTGTATTTCCTTACAGCTTTCTTTTCAAACTTTTTTTT

In100 candidate2088 (in intron):

LOCUS ENSG00000096968

DESCRIPTION Tyrosine-protein kinase JAK2 (EC 2.7.10.2) (Janus kinase 2) (JA-2). [Source:Uniprot/SWISSPROT;Acc:O60674]

CATTCTCTCTAACCACTATTTTAGCTGTATTCCCCCAAATTTTATTGTTTATTATCATTCAGGTGAAAATAATTTCTGATTTCCCTTTTAATTTCTTCTT

In100 candidate2089 (in intron):

LOCUS ENSG00000198561

DESCRIPTION Catenin delta-1 (p120 catenin) (p120(ctn)) (CadherinassociatedSrc substrate) (CAS) (p120(cas)). [Source:Uniprot/SWISSPROT;Acc:O60716]

AGGTATAGTAACTTTTGAGACCAAGACTTTTACTTTTTTTTTCTTGGTCCCAGGATAATGCTTCTGTTTTCTTTCGTTTCTTCCATTTCACCATCATGCT

In100 candidate2090 (in intron):

LOCUS ENSG00000154556

DESCRIPTION sorbin and SH3 domain containing 2 isoform 2 [Source:RefSeq_pepide;Acc:NP_066547]

ACTGGTAATATTTTAGTACTATTATGAAGAAGTTTTCATGTTAACTGCCTATCAAATTCTTCTTGACCTTCTCAGCTACAGTAATTTCGCCTTCATTTTC

In100 candidate2091 (in intron):

LOCUS ENSG00000196761

DESCRIPTION

CCTATTCTAGGATCTTGGCTAGGCTAACACATTGCATTGAGTCATTATGTTTCCCTTGGCTCCTCTTTGCTTTGACAGCTTTTCAGGCTTTCCTTGTTTG

In100 candidate2092 (in intron):

LOCUS ENSG00000144810

DESCRIPTION Collagen alpha-1(VIII) chain precursor (Endothelial collagen).

Source:Uniprot/SWISSPROT;Acc:P27658]

ATCCTTCAATCCAATCGAGTTGACACTCAATATTAACTATCACAATTCTACCTTACTTGATTTCCATTTTCCTGTCAGAA

ATGTCCTGACTTTTTTACCT

In100 candidate2093 (in intron):

LOCUS ENSG00000197863

DESCRIPTION Zinc finger protein 790. [Source:Uniprot/SWISSPROT;Acc:Q6PG37]

EXON

TCTCATTTTTCACTCATGTAACAAAAAGGTAAGTGCCATTCTTTTCTCCAGTGGCCCTTATTTTATCTATGATTTGGATC

AAGTTCCTTCTCATCTCCCT

In100 candidate2094 (in intron):

LOCUS ENSG00000167637

DESCRIPTION Zinc finger protein 345 (Zinc finger protein HZF10).

[Source:Unprot/SWISSPROT;Acc:Q14585]

CTAATATTTTTTATGTTAGTAACATCTGGTACAATCAAGCCTGCTTTTTCGTTATCTTTTACTCTTCTCACTTATCAGAT

TTCTTTTTTTTCTTTTCTTT

In100 candidate2095 (in intron):

LOCUS ENSG00000184220

DESCRIPTION Uncharacterized protein C3orf26.

[Source:Uniprot/SWISSPROT;Acc:9BQ75]

AATATACTAACCTTAAATACTTGTCTTCCTCATTTGTTCTCTTTTTATCCATCTGTGAACTTTTTTCCTTTTACTCAGCT

TTTCTTTTTTTGTACCCTTT

In100 candidate2096 (in intron):

LOCUS ENSG00000184220

DESCRIPTION Uncharacterized protein C3orf26.

[Source:Uniprot/SWISSPROT;Acc:9BQ75]

TGTTTGATATTAACATAGCTATATAATCTTTCTTTTAATTAGTAGTTACTAATGTCTTTTCTGACCTTTTACTTTCAGCT

TTACCATACCCTTATGTTCT

In100 candidate2097 (in intron):

LOCUS ENSG00000184220

DESCRIPTION Uncharacterized protein C3orf26.

[Source:Uniprot/SWISSPROT;Acc:9BQ75]

CTCTCCTTAATCTCTTATAACCTAGATTAGACACATGTTAGACTTTCTTTATCTTCACATCCCTTAACTACTCTTCAGTT

ATTCCATCTGTTTGGCTTTC

In100 candidate2098 (in intron):

LOCUS ENSG00000184220

DESCRIPTION Uncharacterized protein C3orf26.

[Source:Uniprot/SWISSPROT;Acc:9BQ75]

GTGAATATAAACATCTCTGTTACTCTCTTCCTAACATAAAATGATCTTTGCCCATTCTGCCTTTATTCCTTTGTCCAGCT

GTCAGATTCTATTTTTTTCT

In100 candidate2099 (in intron):

LOCUS ENSG00000036054

DESCRIPTION TBC1 domain family, member 23

[Source:RefSeq_peptide;Acc:NP_06079]

TTTCTTAAATTAGAAAATATAATTATTTATATTTCTCTTAGGGAAAATAACCTTAAATTTTTACTTATTTATCTCAGGA

TTGATTTTATTATTTTCCTT

In100 candidate2100 (in intron):

LOCUS ENSG00000127325

DESCRIPTION Bestrophin-3 (Vitelliform macular dystrophy 2-like protein 3).

Source:Uniprot/SWISSPROT;Acc:Q8N1M1]

CATCTCATTCCTACTTTTATAACTATTATTGTTATTATCACTTCCTGATTCTTAAGCTTCATCAGCACCAGAACACTTAA

TTCTCTTCAATTTCCTTTTT

In100 candidate2101 (in intron):

LOCUS ENSG00000197050

DESCRIPTION Zinc finger protein 420. [Source:Uniprot/SWISSPROT;Acc:Q8TAQ5]

EXON

AGTATTTGTTAGTGTTTTTTAAGAAATCGGATGCACCATTTACATATTAACAATATTGAATCCTTTTATTGTTCAGCATG

CTAAATCTGCTTTTCTTTTT

In100 candidate2102 (in intron):

LOCUS ENSG00000044115

DESCRIPTION Catenin alpha-1 (Cadherin-associated protein) (Alpha Ecatenin)(

NY- REN-13 antigen). [Source:Uniprot/SWISSPROT;Acc:P35221]

GTTTGGCTCTTATGTATGGCTTTGAAACTCATCTAGTATAACCTTATATTCCATCTCATGTTCTGTCCTATCAGGATTCC

TGCTGCATTTTTCCACATAT

In100 candidate2103 (in intron):

LOCUS ENSG00000044115

DESCRIPTION Catenin alpha-1 (Cadherin-associated protein) (Alpha Ecatenin)(

NY- REN-13 antigen). [Source:Uniprot/SWISSPROT;Acc:P35221]

GTGACTAATGGTGACTTAGTATATATGGCGACTTAGTATATAACTGAAGTGACTTTTTTTTTTTAACTTTCGTTCAGTT

TTTTTTTGTTTGTTTTTGTT

In100 candidate2104 (in intron):

LOCUS ENSG00000205111

DESCRIPTION Cyclin-dependent kinase-like 4 (EC 2.7.11.22).

[Source:Uniprot/WISSPROT;Acc:Q5MAI5]

CTCTGGTCTTGGACTTTTTTTCATTGGTAACTTTTTAATTATCATTTCAATCTCGCTGCTTCTTATTGGTCTGTTCAGAG

GTTCTATTTCTTCCTGGTTT

In100 candidate2105 (in intron):

LOCUS ENSG00000011566

DESCRIPTION Mitogen-activated protein kinase kinase kinase kinase 3 (EC

2.711.1) (MAPK/ERK kinase kinase kinase 3) (MEK kinase kinase 3) (MEKKK 3)

(Geminal center kinase-related protein kinase) (GLK).

[Source:Uniprot/SWISSPRO;Acc:Q8IVH8]

AACTTAATAAAAATAACTTTTAAAAGTTTCTTTTTATGTTGCCTTAAACAATTTTATTTTCTCTCAGTTTCCTAAATGTT

TCTCATTTTTTTTAATAGCT

In100 candidate2106 (in intron):

LOCUS ENSG00000107020

DESCRIPTION Uncharacterized protein C9orf46.

[Source:Uniprot/SWISSPROT;Acc:9HBL7]

AGGAGATTAATATTGGTATAATACTATTAACCACTGATTTTTTTTTTTTTTTGATATTTCACCAGTTTTTCCTAAAGT

CCCTTTTCTGTTCCAGGATC

In100 candidate2107 (in intron):

LOCUS ENSG00000181220

DESCRIPTION Zinc finger protein 746. [Source:Uniprot/SWISSPROT;Acc:Q6NUN9]

EXON

GGGGATGCCACCATGCCTGGCTAACTTTTTTTAAAAACTTTTTTTTTTCCCCTCTGTGGAGACAGGGTCTTCTATGTTGC

CTAGGCTGGCTTTTTTTGTT

In100 candidate2108 (in intron):

LOCUS ENSG00000154175

DESCRIPTION Target of Nesh-SH3 precursor (Tarsh) (Nesh-binding protein)

(NehBP) (ABI gene family member 3-binding protein).

[Source:Uniprot/SWISSPROT;cc:Q7Z7G0]

CAACAATAGGTAATAAAGCTTTTTTAATGAGTGAAAACAAAGTATCTTTAACACTCTCCATGTTCAACTTTGTTCAGCT

TTGGGTTTTGTTTTTTTTT

In100 candidate2109 (in intron):

LOCUS ENSG00000111596

DESCRIPTION CCR4-NOT transcription complex subunit 2 (CCR4-associated facto

2). [Source:Uniprot/SWISSPROT;Acc:Q9NZN8]

AGATCCTATTTTTTTTTTAATTTAACATTGTATTTTGCTTATTCTTTCCTTTTATATACTCATTAACTTTTTAGCAGGC

CATGTTCTCCTGTGTCGTTT

In100 candidate2110 (in intron):

LOCUS ENSG00000135643

DESCRIPTION Calcium-activated potassium channel subunit beta 4 (Calciumactvated

potassium channel, subfamily M subunit beta 4) (Maxi K channel subuni

beta 4) (BK channel subunit beta 4) (Slo-beta 4) (K(VCA)beta 4) (Charybdotxin

receptor subunit beta 4) (BKbeta4) [Source:Uniprot/SWISSPROT;Acc:Q86W47

TCTTATTTCATTTAAAAGATTTTGAAGATTTTGAGACAAAGGCGCGTGCTAACTTTGCTCCGTGATTAATCCTTCCAGAG

TTTGTTGCCATCTTGATTTT

In100 candidate2111 (in intron):

LOCUS ENSG00000120727

DESCRIPTION Polyadenylate-binding protein-interacting protein 2 (Poly(A)-

biding protein-interacting protein 2) (PABP-interacting protein 2) (PAIP-2).

Source:Uniprot/SWISSPROT;Acc:Q9BPZ3]

TTAACTTAAATTTGCTGATTCTTTTGTCTCTCTGCTCATATCTGCTGTTGAACCCCTCTAGTAAATTTTTCATTTCAGTT

GTACCTTTTTTTTTTTTTT

In100 candidate2112 (in intron):

LOCUS ENSG00000127329

DESCRIPTION Receptor-type tyrosine-protein phosphatase beta precursor (EC 31.3.48) (Protein-tyrosine phosphatase beta) (R-PTP-beta).

[Source:Uniprot/SISSPROT;Acc:P23467]

AGAGGAGGACATAATTCTTAACTTTGCTTCTCTATATGTAAGTTGTTTTTCCCCCTCTGGCTTCTTTCAGGAATTTTTCT

TTATCTTTGATTTTCTGTAG

In100 candidate2113 (in intron):

LOCUS ENSG00000081148

DESCRIPTION interphotoreceptor matrix proteoglycan 2

[Source:RefSeq_peptideAcc:NP_057331]

AGGCTTTTTGGGGGAGCATCCTTTTAGTTTTTGATCTTAGTTTGCTAACCCAAGATCACAAAGTTTTTTCTTTCTCAGTC

TTTTCCTGTAGAATTTTTAC

In100 candidate2114 (in intron):

LOCUS ENSG00000081148

DESCRIPTION interphotoreceptor matrix proteoglycan 2

[Source:RefSeq_peptideAcc:NP_057331]

AACCAAAGCTACATTGCCAAAAATGTTTGCTAACATTGCATCATATACTTTTTATCACTTTTCTTTTCCCTTTCCAGATT

TTTATTTTCTGGCTAAATTT

In100 candidate2115 (in intron):

LOCUS ENSG00000107036

DESCRIPTION KIAA1432 (KIAA1432), mRNA [Source:RefSeq_dna;Acc:NM_020829]

TTTTACCTATAAATTTCTCTCTGAGGACTTCATTCACTGTATCCCATAACGTTGCATGTTTGTTTTCATTAGTTTCAGAG

CATTTCCTGATTTCCCTTTT

In100 candidate2116 (in intron):

LOCUS ENSG00000141753

DESCRIPTION Insulin-like growth factor-binding protein 4 precursor (IGFBP-4

(IBP- 4) (IGF-binding protein 4). [Source:Uniprot/SWISSPROT;Acc:P22692]

GAGAGAGAGAGAGAGACTCTGAGAAGGCCCTTGCCCTAACCTCATTCTTACACTTTCTTTTTTTGTTTCTCAGTTTCTCC

TTAGACTCTCAGTCTCTTTT

In100 candidate2117 (in intron):

LOCUS ENSG00000138050

DESCRIPTION THUMP domain-containing protein 2.

[Source:Uniprot/SWISSPROT;Ac:Q9BTF0]

TGTTCGTGTCCTAACTCTTTCCTGTTAACTTTCTTCTGCTGTCAGGCTACTGCTTTTAATACATGCAGTCTTTAGGATTC

TGTCATTTGCCTTTTCCTTT

In100 candidate2118 (in intron):

LOCUS ENSG00000138468

DESCRIPTION Sentrin-specific protease 7 (EC 3.4.22.-) (Sentrin/SUMO-specifi

protease SENP7) (SUMO-1-specific protease 2).

[Source:Uniprot/SWISSPROT;Ac:Q9BQF6]

TTTCATTCTTTTTTAAATAAAGCATAACATTAAATTTACATAAGCATCTGTTTCCTTTCTGTTCTTCAGATTGAATATTT

CTATTGGTCTATCTATTTCC

In100 candidate2119 (in intron):

LOCUS ENSG00000138468

DESCRIPTION Sentrin-specific protease 7 (EC 3.4.22.-) (Sentrin/SUMO-specifi

protease SENP7) (SUMO-1-specific protease 2).

[Source:Uniprot/SWISSPROT;Ac:Q9BQF6]

AGTAACTGACTAAAAACCCTTTCCTGCTTCTCATGTCTATTTTCAGGTCTTGTCTTATTTTCCACTTTCACAACCATATT

TCTCAACTACTTTCATTTGC

In100 candidate2120 (in intron):

LOCUS ENSG00000102452

DESCRIPTION voltage gated channel like 1 [Source:RefSeq_peptide;Acc:NP_44309]

CTATTTATGTCACTGTTTAAACCAAACCTAATATTTAGTCTTTAACAAGAGATTTAACATTTCATCAATTTCTTTCAGGT

CTCCATTCTTTTGCTGACAT

In100 candidate2121 (in intron):

LOCUS ENSG00000153233

DESCRIPTION Receptor-type tyrosine-protein phosphatase R precursor (EC 3.1..48) (Protein-tyrosine phosphatase PCPTP1) (NC-PTPCOM1) (Ch-1PTPase). [Soure:Uniprot/SWISSPROT;Acc:Q15256]

CTTTTCAAATAACTAGCTTTTGTTTCACTCGTTTTTCTCTCTCTGTATTTTCAATGTAATGATTTCTACTTTTAACAGTT

TAATTTCTTTACATTTATTT

In100 candidate2122 (in intron):

LOCUS ENSG00000153233

DESCRIPTION Receptor-type tyrosine-protein phosphatase R precursor (EC 3.1..48) (Protein-tyrosine phosphatase PCPTP1) (NC-PTPCOM1) (Ch-1PTPase). [Soure:Uniprot/SWISSPROT;Acc:Q15256]

GTTACATATACTTGTATTTAACTATCCATTTATTTTCCATTCTTGGTCTATATTTTATTTCATTTTGTTATTTTTCAGTA

TTGAAATTGCTTTTCTTCTC

In100 candidate2123 (in intron):

LOCUS ENSG00000186660

DESCRIPTION Zinc finger protein 91 homolog (Zfp-91). [Source:Uniprot/SWISSPOT;Acc:Q96JP5]

ATCCCATTCATCTTGGCCTCTTGTCAACTTGCAACCTCAATATAACATTGACTGTTTTTACTTCCTTTACAGATGTGTCT

GTTTTATAGTATACTTTTTT

In100 candidate2124 (in intron):

LOCUS ENSG00000171130

DESCRIPTION ATPase, H+ transporting V0 subunit e2 (ATP6V0E2), mRNA [Source:efSeq_dna;Acc:NM_145230]

ACTATTAACACATGGTTTAACTTAAACCCACTTTTTTTTTTTTTTTCAAAATTCTTCCTGGACAGAGCATTCTCTGAGGT

CCTTTCTCTAATGCTATTTC

In100 candidate2125 (in intron):

LOCUS ENSG00000183023

DESCRIPTION Sodium/calcium exchanger 1 precursor (Na(+)/Ca(2+)-exchange

proein 1). [Source:Uniprot/SWISSPROT;Acc:P32418]

AGTCTCCTTGGCAACATAACGTAGAGGTTGATTTTTCTCTTTTTTATATCTATCTCTGACTAGCAGGTTTGGTTTTTCCC

TTTGTCTGAAGCTTATCTAC

In100 candidate2126 (in intron):

LOCUS ENSG00000158458

DESCRIPTION Pro-neuregulin-2, membrane-bound isoform precursor (Pro-NRG2)

[ontains: Neuregulin-2 (NRG-2) (Neural- and thymus-derived activator for ERB

kinases) (NTAK) (Divergent of neuregulin-1) (DON-1)].

[Source:Uniprot/SWISPROT;Acc:O14511]

CCTATCTAAAATTGCAACCCCATAACCCTCCCAACCCTACTCTGTTTTATTTTTCACCTCAGCTCTTATCATTATCTGAC

ATCATATTTTACCAACTTTT

In100 candidate2127 (in intron):

LOCUS ENSG00000158458

DESCRIPTION Pro-neuregulin-2, membrane-bound isoform precursor (Pro-NRG2) [ontains: Neuregulin-2 (NRG-2) (Neural- and thymus-derived activator for ERB kinases) (NTAK) (Divergent of neuregulin-1) (DON-1)]. [Source:Uniprot/SWISPROT;Acc:O14511]

AAATTTTTAAATAAATACTAGTTTATTTGAGCCTTTTTCATTTATGGTTTAACCATCCAATATGTCTGTTTACTTCAGTT
CTTTTTCTTCTTGACACTTC

In100 candidate2128 (in intron):

LOCUS ENSG00000080371

DESCRIPTION Ras-related protein Rab-21. [Source:Uniprot/SWISSPROT;Acc:Q9UL2]

ATTCTTAGCTCCTATGCTAGCATGAATGGGAGAAGACATTTATTGTCATAACATGTATTTGGTCTTCTCATTTTTCAGGT
TACTTTGCTGTTTTGGCATT

In100 candidate2129 (in intron):

LOCUS ENSG00000080371

DESCRIPTION Ras-related protein Rab-21. [Source:Uniprot/SWISSPROT;Acc:Q9UL2]

AGTAACTCTTGATACTCTTCTTTCTTTCCTATCTTCATGGTCTTAGTTCAGATCTCCTTTTTTATATTTTTCTCCAATTT
TGTAAGTGTTCTTTCTACCT

In100 candidate2130 (in intron):

LOCUS ENSG00000102466

DESCRIPTION Fibroblast growth factor 14 (FGF-14) (Fibroblast growth factor omologous factor 4) (FHF-4). [Source:Uniprot/SWISSPROT;Acc:Q92915]

GGTATATGAGTTCCTTGTGTATCTTAGATATTAACCCCTTATCAAATGCATTTACATATATCTTCTCCCATTTCACAGAC
TGTCTCTTCACTCTGTTGAT

In100 candidate2131 (in intron):

LOCUS ENSG00000102466

DESCRIPTION Fibroblast growth factor 14 (FGF-14) (Fibroblast growth factor omologous factor 4) (FHF-4). [Source:Uniprot/SWISSPROT;Acc:Q92915]

CTGTTAAATATTGTGAAGATACATTTTTAAGGTCACATAACTGCTTTTGCATAATTTTATTTCACATGTTTTTCAGTTAC
ATTCTCTTTCTTCCTACAGT

In100 candidate2132 (in exon):

LOCUS ENSG00000139287

DESCRIPTION Tryptophan 5-hydroxylase 2 (EC 1.14.16.4) (Tryptophan 5-monooxyenase 2) (Neuronal tryptophan hydroxylase). [Source:Uniprot/SWISSPROT;Acc:QIWU9]

ACCAAACTGCATCTAGTTAAAATTTGTAACAAATAGCCCTCTTATGAGTCTCATTTATGCCCTTTTCTTTTTCAGATCTA
AGCCTTTCCTCTGTGTTCAT

In100 candidate2133 (in intron):

LOCUS ENSG00000137033

DESCRIPTION Interleukin-33 precursor (IL-33) (Interleukin-1 family member 1) (IL- 1F11) (Nuclear factor from high endothelial venules) (NF-HEV). [Soure:Uniprot/SWISSPROT;Acc:O95760]

TTCTTTTTTCTGCTTCTCTGTCTGGTCTTCTTATTAACTTCGTTTCTCCTCTTTCCCCTTACATGTTAGATATTCCAGAG
TTTCATCTTCTTACAGCTTT

In100 candidate2134 (in intron):

LOCUS ENSG00000170017

DESCRIPTION CD166 antigen precursor (Activated leukocyte-cell adhesion moleule) (ALCAM). [Source:Uniprot/SWISSPROT;Acc:Q13740]

TCTTTTAAATTATACAAAATCACTTCACATGTAATGTAAGAAGGTAACAAGCATACACTTCTATTTCCCTTTCAGGTATT

TTGTGCCATTGTTGTTATTT

In100 candidate2135 (in intron):

LOCUS ENSG00000113068

DESCRIPTION Prefoldin subunit 1. [Source:Uniprot/SWISSPROT;Acc:O60925]

AGGTTCTCTAACCTGATACCCCATTTTCATTAGTATTTGCTAGGTTCCCTTTTACCCAGCTTTGTTCTTTCTGTTTCTTT

ATACTTGCCCTCTCCTGATT

In100 candidate2136 (in intron):

LOCUS ENSG00000177324

DESCRIPTION Uncharacterized protein CXorf20. [Source:Uniprot/SWISSPROT;Acc:8NDZ0]

AGTAGCTTTTCTTAAACCTAGGTTCTTATATAATAACTTCTTTGCCTACCTCCTTTATTAATCAGCTCTTTTATTCTCAT

ATTCTCTTTTTCTTGGCTTT

In100 candidate2137 (in intron):

LOCUS ENSG00000166006

DESCRIPTION Shaw-related voltage-gated potassium channel protein 2 isoform V3.2c [Source:RefSeq_peptide;Acc:NP_715624]

CCATCATTTAAGTATGATATTGAAGTCACTAACTGCAATTGTTGAATTATCTATTTCTTCCTTTAATTCTGTCAGTTTTT

GCTTCTGTATTCTGGGGTTC

In100 candidate2138 (in intron):

LOCUS ENSG00000166006

DESCRIPTION Shaw-related voltage-gated potassium channel protein 2 isoform V3.2c [Source:RefSeq_peptide;Acc:NP_715624]

ACTTTGCCACAAACTATAAAGGCAACTCTAAACACGTGACCTAACTTCTTAGGCTTTTTTTGTTTATTTGTTTTTCAGTT
ATTTTTGTTTTTTTGTTTTG

In100 candidate2139 (in intron):

LOCUS ENSG00000147854

DESCRIPTION E3 ubiquitin-protein ligase UHRF2 (EC 6.3.2.-) (Ubiquitin-like HD and RING finger domain-containing protein 2) (Ubiquitin-like-containing HD and RING finger domains protein 2) (Np95/ICBP90-like RING finger protein (Np95-like RING finger protein) (Nucle [Source:Uniprot/SWISSPROT;Acc:Q96PU]

TTATTTAATACATGAATTAATTTATGTAATGTATAACATGCTCCATCTCTGATTGTTTGTCCTACCCAGCATGGTTGGCT
CTTAGGTTTTTATTTTTTCC

In100 candidate2140 (in intron):

LOCUS ENSG00000114439

DESCRIPTION HMG box transcription factor BBX (Bobby sox homolog) (HMG boxontaining
protein 2). [Source:Uniprot/SWISSPROT;Acc:Q8WY36]

TGTGTAGACATGTGTGCCTGTATGTTCCTGTATGTAACCTTTTACTTACTTTGGGCCTTCTTTTCACTTCCACTTCAGCT

TCTTAGTTTTGTTTTGTATA

In100 candidate2141 (in intron):

LOCUS ENSG00000180881

DESCRIPTION Calcyphosin-2 (Calcyphosine-2).

[Source:Uniprot/SWISSPROT;Acc:QBXY5]

AAGCACTCTTCTCCATTTTTCTAACTCTTCCACCTCTTGTTCCTGCCCCCAATCTTCTATTGTTTTATGCAGAAATATTC

AACCCTATCCCTCTTTTTTT

In100 candidate2142 (in intron):

LOCUS ENSG00000008086

DESCRIPTION Cyclin-dependent kinase-like 5 (EC 2.7.11.22) (Serine/threonine

protein kinase 9). [Source:Uniprot/SWISSPROT;Acc:O76039]

TTATATAGGAATGTCATTTCTATTTATAACTGAATTAGAATATTTTTTCTAACAGCTTTAGCTCTCTTCCATATTTAAAC

TTAAAATTCTTTGCTGTTTT

In100 candidate2143 (in intron):

LOCUS ENSG00000173401

DESCRIPTION GLIPR1-like protein 1 precursor.

[Source:Uniprot/SWISSPROT;Acc:6UWM5]

AAACACTTGCAAAGAGAGGCACCCTAAAACTAACACGGCACCTCAAAGTGGATCATTCTTGTCTTTCCTCTTCTTCAGAT

TGTTGCCTACTTTTTTTTTT

In100 candidate2144 (in intron):

LOCUS ENSG00000173401

DESCRIPTION GLIPR1-like protein 1 precursor.

[Source:Uniprot/SWISSPROT;Acc:6UWM5]

ATTGCCTTAAAAAATTTAAATGCTTTTGTATATTAGTCTATTAACACTTTTACCAAACTCTCTTATTATTTCTGACAGTT

TATTGATTTTCTTGGATTTT

In100 candidate2145 (in intron):

LOCUS ENSG00000180481

DESCRIPTION GLI pathogenesis-related 1 like 2

[Source:RefSeq_peptide;Acc:NP689649]

TTTTATGATATATTTAACTGGCCAATTGATTTTTTAAAATTTCTAGACTTTTTTTTTAATTTGCTCATATTCCCCCAGTG

TCCTGTTTTCTCTTATGATT

In100 candidate2146 (in intron):

LOCUS ENSG00000110077

DESCRIPTION Membrane-spanning 4-domains subfamily A member 6A (Four-span

trnsmembrane protein 3) (CD20 antigen-like 3).

[Source:Uniprot/SWISSPROT;Acc:9H2W1]

TCATTTTTTCCATTATCCTATCTGTCAACCAAACTATATTTATAACCACACATGTGGCCTTTTATTTTATTACAGGGTAA

TATTTTATTTCATTCTACTT

In100 candidate2147 (in intron):

LOCUS ENSG00000086717

DESCRIPTION Serine/threonine-protein phosphatase with EF-hands 1 (EC 3.1.3.6) (PPEF-1) (Protein phosphatase with EF calcium-binding domain) (PPEF) (Seine/threonine-protein phosphatase 7) (PP7). [Source:Uniprot/SWISSPROT;Acc:O4829]

TGATTACTGTAGCTTTGTGGGAAGTTTTAACATTGGGAAGTGTGAGTTCTCCAACTTTCTTCTTCTTTTTCCAGATCGTT
TTGGCTATTCTGGGCCCCTT

In100 candidate2148 (in intron):

LOCUS ENSG00000187109

DESCRIPTION Nucleosome assembly protein 1-like 1 (NAP-1-related protein) (hRP). [Source:Uniprot/SWISSPROT;Acc:P55209]

TTTTTTTTTTTTTAAACTTTTAACCTAGTATGTCTTTTTTTTCCATCTTTTCATCTGTGTGTCTTCCTCTAGTATCAGTG
TTTGCTGCTTTTATTTTTTG

In100 candidate2149 (in intron):

LOCUS ENSG00000173848

DESCRIPTION Neuroepithelial cell-transforming gene 1 protein (p65 Net1 protoncogene) (Rho guanine nucleotide exchange factor 8). [Source:Uniprot/SWISPROT;Acc:Q7Z628]

CCAAATTCTTAGTACTAAACTTGGAGATTCAAATCACATATAACTTCATTACTAATTTCTTTCGTACTTATCCAGTTCAA
ATGTAACTCTTTTTGTTTTT

In100 candidate2150 (in intron):

LOCUS ENSG00000173848

DESCRIPTION Neuroepithelial cell-transforming gene 1 protein (p65 Net1 protoncogene)
(Rho guanine nucleotide exchange factor 8).
[Source:Uniprot/SWISPROT;Acc:Q7Z628]

CCTCTTTGCTGTAATTAACATTTTTTTCTTCCCTTGGCTTTTGCTTTGTTTCTTTCCTTCTAACTATTCCTTTTGCAGAA
TTTTCCTTCCCTTGGCTTTC

In100 candidate2151 (in intron):

LOCUS ENSG00000144821

DESCRIPTION Myosin-15 (Myosin heavy chain 15).
[Source:Uniprot/SWISSPROT;Ac:Q9Y2K3]

TCCAAGATTAGAAATAAGGTCTAAAGAAACCCTGAAGAACATAACCATTAATCTAAATTTTCACTTAGTTGTTCCCAGAC
TTTTTCTGAATGAGTTTTCT

In100 candidate2152 (in intron):

LOCUS ENSG00000152527

DESCRIPTION pleckstrin homology domain containing, family H (with MyTH4 domin) member 2 [Source:RefSeq_peptide;Acc:NP_742066]

AAGGTAAAGTTAACACAATATCATTTTCTGTCTTCTGAACAGTGATCTTCACATTATTCAACTACCCATTGAATATTTCT
TCATCTTCCTTTGTATTATT

In100 candidate2153 (in intron):

LOCUS ENSG00000110079

DESCRIPTION Membrane-spanning 4-domains subfamily A member 4A (Four-span trnsmembrane protein 1) (CD20 antigen-like 1). [Source:Uniprot/SWISSPROT;Acc:96JQ5]

AGACCTTTTAATTCTACACTTCTAATCTTTTAACCTCCCTTCTATTATGACCATTTTCTTGTTTCTCTACAGAATTCTTG

GTGATTTCTTCAAATTTTTC

In100 candidate2154 (in intron):

LOCUS ENSG00000091039

DESCRIPTION Oxysterol-binding protein-related protein 8 (OSBP-related proten 8) (ORP-8). [Source:Uniprot/SWISSPROT;Acc:Q9BZF1]

TCTCCTTTTCCCTTATATTTTTAGGTAACTTACTGTGCTTTTTCCCCTTCTCTCTGATAGTTGATATTATTAGTTCAGTT

TGTTTCTTCTGCTCCCCTGT

In100 candidate2155 (in intron):

LOCUS ENSG00000091039

DESCRIPTION Oxysterol-binding protein-related protein 8 (OSBP-related proten 8) (ORP-8). [Source:Uniprot/SWISSPROT;Acc:Q9BZF1]

AGTTCATGACTAGGTAACCTGATCTAGCACTTTCTTTCCTCATTTTTAAATTTAATTCTCACACTCCAGTATTTCTTCTT

AGCTATTCTTTATCTAAACA

In100 candidate2156 (in intron):

LOCUS ENSG00000091039

DESCRIPTION Oxysterol-binding protein-related protein 8 (OSBP-related proten 8) (ORP-8). [Source:Uniprot/SWISSPROT;Acc:Q9BZF1]

TGTGATTATCCATTAAGCTCTTATTTTAACATTTGTATCGTTTACCTTTTTACAGAATATTTATTTTCCTAATTGAATTG

TTAGCTTCTTTAATTCCCTC

In100 candidate2157 (in intron):

LOCUS ENSG00000107077

DESCRIPTION JmjC domain-containing histone demethylation protein 3C (EC 1.1.11.-) (Jumonji domain-containing protein 2C) (Gene amplified in squamous cll carcinoma 1 protein) (GASC-1 protein). [Source:Uniprot/SWISSPROT;Acc:Q9HR0]

GTATAACAAATGTAACTCTGATCTTTCCTCTCAGCTCTGCTTCTCTGGAATTTCTCCTGCATCTCATCTCATTTCATTGC
TCACTCATTTCATTATGAGT

In100 candidate2158 (in intron):

LOCUS ENSG00000107077

DESCRIPTION JmjC domain-containing histone demethylation protein 3C (EC 1.1.11.-) (Jumonji domain-containing protein 2C) (Gene amplified in squamous cll carcinoma 1 protein) (GASC-1 protein). [Source:Uniprot/SWISSPROT;Acc:Q9HR0]

TCAAGGTCTAGGTTTAATTCTAGTAGCCACTAATTTGCTGCATGTCTTAACAATTTCTGTGCCTCAATTTCTTGTCAGAG
CCATTTTTCATTTGTTTGTT

In100 candidate2159 (in intron):

LOCUS ENSG00000107077

DESCRIPTION JmjC domain-containing histone demethylation protein 3C (EC 1.1.11.-) (Jumonji domain-containing protein 2C) (Gene amplified in squamous cll carcinoma 1 protein) (GASC-1 protein). [Source:Uniprot/SWISSPROT;Acc:Q9HR0]

AAGATCTTAACCCTTCTGAGAACTACTTATATCCTTACTCTTTTCCCCTAATTTTCCTTATCTAGATCTTTACAGATTGC

TTTGTCAAAATTTCATTTTT

In100 candidate2160 (in intron):

LOCUS ENSG00000107077

DESCRIPTION JmjC domain-containing histone demethylation protein 3C (EC

1.1.11.-) (Jumonji domain-containing protein 2C) (Gene amplified in squamous cll

carcinoma 1 protein) (GASC-1 protein). [Source:Uniprot/SWISSPROT;Acc:Q9HR0]

GATTCCAATGAGGATAACAAACACGATTGTCCTCTGTAGTTCTGTGTTCTGTTGTAGCCTCCTTTCTCTCGTAGTCAGGG

TTCTTGGCTTCTCAGTGTTT

In100 candidate2161 (in intron):

LOCUS ENSG00000198919

DESCRIPTION E3 ubiquitin-protein ligase DZIP3 (EC 6.3.2.-)

(DAZinteractingprotein

3) (RNA-binding ubiquitin ligase of 138 kDa) (hRUL138).

[Source:Unirot/SWISSPROT;Acc:Q86Y13]

ACATTTGCAAGTAGTTTAACTGCTTGTTACAAATATATTTTTTCCTGTTTTTTAGCCAGTATAATATTTATAGCTTCTTG

AATCTTTAGTCTCTTTTTTT

In100 candidate2162 (in intron):

LOCUS ENSG00000173698

DESCRIPTION G-protein coupled receptor 64 precursor (Epididymis-specific

prtein 6) (He6 receptor). [Source:Uniprot/SWISSPROT;Acc:Q8IZP9]

ATACCAACTTTATTCTTTTTTGTTTGTTTTGTTTTGTTTTTAACTCCTCACTCCCACTTGGAGCTCTCTTTTCTTCAGAT

ACTTTCTTTTCCTTGGGGAT

In100 candidate2163 (in intron):

LOCUS ENSG00000067798

DESCRIPTION neuron navigator 3 [Source:RefSeq_peptide;Acc:NP_055718]

ACACTTATGACTGATGGAATTGATTATCTTTTCTGTATTTAACTGGCCATTTGGATATCTTCCGTTGTGTTCATTCAGGT

TTTCTGCTTACTTTTCTAAT

In100 candidate2164 (in intron):

LOCUS ENSG00000067798

DESCRIPTION neuron navigator 3 [Source:RefSeq_peptide;Acc:NP_055718]

AATCCTACATTTCCCTGCTCCACTCTTTCATTCTCTTAACAAATAATTCACAACTATTCTTTCTCCTCAAATTTCCAGTC

TTTCTTTCCCTTTCTCAGTT

In100 candidate2165 (in intron):

LOCUS ENSG00000133612

DESCRIPTION Centaurin-gamma 3 (ARF-GAP with GTP-binding protein-like, ankyrn repeat and pleckstrin homology domains 3) (AGAP-3) (MR1-interacting proten) (MRIP-1) (CRAM-associated GTPase) (CRAG). [Source:Uniprot/SWISSPROT;Acc:96P47]

CCTCACCGGTGCTGACCGACTTGTGCGGGGCTTGAGTATAACTTGCCAAAATCTTTATTCTTTCGATATTTGCAGATATG

TGCCACTGTTTCCAACTTTT

In100 candidate2166 (in intron):

LOCUS ENSG00000153707

DESCRIPTION Receptor-type tyrosine-protein phosphatase delta precursor (EC

.1.3.48) (Protein-tyrosine phosphatase delta) (R-PTP-delta).

[Source:Unipro/SWISSPROT;Acc:P23468]

TCCACTTCTCATAGAAACAATAATTTCTACATAGATAACCTTCAAAATAAATTTCCTCGCTTGTCTCTGGAGTTCCAGAC

GTGCATTTCTATCTGCTTGT

In100 candidate2167 (in intron):

LOCUS ENSG00000153707

DESCRIPTION Receptor-type tyrosine-protein phosphatase delta precursor (EC

.1.3.48) (Protein-tyrosine phosphatase delta) (R-PTP-delta).

[Source:Unipro/SWISSPROT;Acc:P23468]

AGGTCCTGTGATTGGATGGAGATACCATTTTAGAACCAATTCTTTAACCTAAAATTTAGTATATTCTTTTTTCTTCAGTT

TGCTTTAGCTTCACTTATTT

In100 candidate2168 (in intron):

LOCUS ENSG00000153707

DESCRIPTION Receptor-type tyrosine-protein phosphatase delta precursor (EC

.1.3.48) (Protein-tyrosine phosphatase delta) (R-PTP-delta).

[Source:Unipro/SWISSPROT;Acc:P23468]

TTGTCCATTATGGCTTTTTATAACTAAATGAAACGATTAATTTCCTTAATATTCACTTTATTCGTTCATTTCATTCAGGC

ATTCTTTTTTACAGACTTAT

In100 candidate2169 (in intron):

LOCUS ENSG00000067715

DESCRIPTION Synaptotagmin-1 (Synaptotagmin I) (SytI) (p65).

[Source:UniprotSWISSPROT;Acc:P21579]

TATTCTGATAATAACATGTTTTGAAAATGGAGGTGTTTTTTTTTTTTTTTTTTTGCCAAACTACTCTGCTCCATCAGGC

TCTTTTGTATCCTAAATATT

In100 candidate2170 (in intron):

LOCUS ENSG00000067715

DESCRIPTION Synaptotagmin-1 (Synaptotagmin I) (SytI) (p65).

[Source:UniprotSWISSPROT;Acc:P21579]

CACCAAAATCTTGTTTATTCACTAACTTTAATCGTATTTTTGTCTAAATTTTCTTCTTCCAAGTTTTTTTTTTTCAGTCA

GTAATCCTACTCTTCATTTT

In100 candidate2171 (in intron):

LOCUS ENSG00000067715

DESCRIPTION Synaptotagmin-1 (Synaptotagmin I) (SytI) (p65).

[Source:UniprotSWISSPROT;Acc:P21579]

TCACACTGATATGTCAACTGCCCATAACCATGCCCCACCTGAGAATTCTTTCTTTGCCATATCCTTGTTTTCCAGCTTTT

GTATATGCATCCCTACATTT

In100 candidate2172 (in intron):

LOCUS ENSG00000177425

DESCRIPTION PRKC apoptosis WT1 regulator protein (Prostate apoptosis respone

4 protein) (Par-4). [Source:Uniprot/SWISSPROT;Acc:Q96IZ0]

TTAGTTGCTAACTTTGCACTTGCCTCCATTCTTGATTTTTTTTTTTTCCTCTGTTTCCAACTTCATAATGCGATCAGGGT

CACTTCTTTCTCCTGTGCTT

In100 candidate2173 (in intron):

LOCUS ENSG00000138095

DESCRIPTION 130 kDa leucine-rich protein (LRP 130) (GP130) (Leucine-rich PP

motif-containing protein). [Source:Uniprot/SWISSPROT;Acc:P42704]

AGGTACCTTAACCTTCACTTAAACTGTATTTATTGTCTTCAGAATTTGCTTCTACTATGAATTCCTCTCTAATCCCATCC

TTTTTGTATTATTCAGTAGC

In100 candidate2174 (in intron):

LOCUS ENSG00000058272

DESCRIPTION Protein phosphatase 1 regulatory subunit 12A (Myosin

phosphatastargeting

subunit 1) (Myosin phosphatase target subunit 1) (Protein phospatase

myosin-binding subunit). [Source:Uniprot/SWISSPROT;Acc:O14974]

GTTTTGTATTTTAGTAGAGGACTGATAAAATTGGAGGGCCATTGCTAACTCTTCATTATCTGTTACCTTATTTCACAGAT

CTGCATTTTGAATTTTTTTG

In100 candidate2175 (in intron):

LOCUS ENSG00000101236

DESCRIPTION RING finger protein 24. [Source:Uniprot/SWISSPROT;Acc:Q9Y225]

AGTTTATCTCCTTAGCTCTCCTAATGATTATTGTTATTAAGCAAGGAATAACAACAAACAATTTTTGCTACATCTCAGTT

CCCCTCTTTTTATAAATATG

In100 candidate2176 (in intron):

LOCUS ENSG00000182346

DESCRIPTION D-amino acid oxidase activator (Protein G72).

[Source:Uniprot/SISSPROT;Acc:P59103]

TGGGTGATCTTCAATTTTTAGCCAAACTAACCCACTCTTGTCTACTCATTTGTGTTGCTTTTTGTCTCACAGATTGATTA

TCTCATCAATTCATTTATTT

In100 candidate2177 (in intron):

LOCUS ENSG00000144824

DESCRIPTION Pleckstrin homology-like domain family B member 2 (Protein LL5-

eta). [Source:Uniprot/SWISSPROT;Acc:Q86SQ0]

ACATTTTATATTATTGCATAGCTTGAATTAGTCTTGCATACTAACCTTATATCACATATAATTTTGTCCTACTTCCAGTT

TTTCCTTTTTCTTCTTAGTT

In100 candidate2178 (in intron):

LOCUS ENSG00000144824

DESCRIPTION Pleckstrin homology-like domain family B member 2 (Protein LL5-

eta). [Source:Uniprot/SWISSPROT;Acc:Q86SQ0]

CCATTTTGTATATATTTCTTTCAAAGCAAAGTCCTTCTTTCTAACTGGCATTAATTCTTGTCTATTCTAACATCTCAGAT

TTTTTTTGTAGCCACACCTA

In100 candidate2179 (in intron):

LOCUS ENSG00000138032

DESCRIPTION Protein phosphatase 2C isoform beta (EC 3.1.3.16) (PP2C-beta).

Source:Uniprot/SWISSPROT;Acc:O75688]

TTGTATTATGGGTACATGAGTGTTGAGCTGAGATAACCATTTCTGTTGTACCTTGCTGTACTTCATCAGAGCAAGACTCT

ATGCCCTTACCTTTTATTTT

In100 candidate2180 (in intron):

LOCUS ENSG00000057608

DESCRIPTION Rab GDP dissociation inhibitor beta (Rab GDI beta) (Guanosine

dphosphate dissociation inhibitor 2) (GDI-2).

[Source:Uniprot/SWISSPROT;Acc:50395]

AACAAAAACAAAACAAAACATAAAATGTATGCATCTTAAAATTTTACAATAACATTATTGTAAATACATCTCATTCAGCT

TCTTGTCTCATTCAGTAACT

In100 candidate2181 (in intron):

LOCUS ENSG00000057608

DESCRIPTION Rab GDP dissociation inhibitor beta (Rab GDI beta) (Guanosine

dphosphate dissociation inhibitor 2) (GDI-2).

[Source:Uniprot/SWISSPROT;Acc:50395]

AGCTGCTTTTAAACTGCGTATTTTAACATTTGGGCATCTGTTTCTGTTGTTTGTTTTTCTGTTTTCTTTGGATCACAGTT

TTCTGTTTCTTCCCATGTCT

In100 candidate2182 (in intron):

LOCUS ENSG00000131828

DESCRIPTION Pyruvate dehydrogenase E1 component alpha subunit, somatic form

mitochondrial precursor (EC 1.2.4.1) (PDHE1-A type I).

[Source:Uniprot/SWISPROT;Acc:P08559]

AACACATCTGTTTGATTGCTAAATATAACCATTGATTTGCTGTTTCACCTTCTCTCAGCTTTACTTCTTCCCAAATTCCT

AAATTTCCTTCACTTTTTCT

In100 candidate2183 (in intron):

LOCUS ENSG00000131828

DESCRIPTION Pyruvate dehydrogenase E1 component alpha subunit, somatic form

mitochondrial precursor (EC 1.2.4.1) (PDHE1-A type I).

[Source:Uniprot/SWISPROT;Acc:P08559]

CACTTTTTCTGAGATACATTAGTGGACTGTCTCTGCCTGTAAGTTAACTGAAACACTGATTCCTAGTATTTCAGTTGTTT

TCCTCCAGCACTGTCATTGT

In100 candidate2184 (in intron):

LOCUS ENSG00000106617

DESCRIPTION 5'-AMP-activated protein kinase subunit gamma-2 (AMPK gamma-2

cain) (AMPK gamma2) (H91620p). [Source:Uniprot/SWISSPROT;Acc:Q9UGJ0]

TTGATGGCTGTGTACTGGGGAAGGGAACGTGGCCCTTGGACCTAACGAAGGTGTCGCCTTGCTTGGCTTCAGCCTTTCTT

GGGCATTTTGGGTCACCTTT

In100 candidate2185 (in intron):

LOCUS ENSG00000204442

DESCRIPTION similar to transmembrane protein 28 (LOC728215), mRNA

[Source:RfSeq_dna;Acc:NM_001080396]

AATACTTTAACTTTGAGTTCTTACTGTATTTGACATTGCTATTTTATCATTTTATTTATATTGTTTTCTCTGTCTCAGCC

TCCTCCTCATGTGGTTTTAT

In100 candidate2186 (in intron):

LOCUS ENSG00000204442

DESCRIPTION similar to transmembrane protein 28 (LOC728215), mRNA

[Source:RfSeq_dna;Acc:NM_001080396]

TTCTCTAGCTTTTCTGGTTCTTTAACTCCCTCACTTTTTTTCCATGGCTCATTTTTCTTTTCCAGATCCATAAATCTGGA

TTCTTTCTAACATCTTTTTT

In100 candidate2187 (in intron):

LOCUS ENSG00000204442

DESCRIPTION similar to transmembrane protein 28 (LOC728215), mRNA

[Source:RfSeq_dna;Acc:NM_001080396]

AGAGTGGCCATTAACTTGAGTTAAGCTTTCTTACTCTGCTAGGTCAGTTACCCCTTCTCCATTTTCTTTTATTTCTTCAT

GTGTTGTGATTACAGGTTTT

In100 candidate2188 (in intron):

LOCUS ENSG00000204442

DESCRIPTION similar to transmembrane protein 28 (LOC728215), mRNA

[Source:RfSeq_dna;Acc:NM_001080396]

CAACCTGAAGCGCCACTCATATTTTTTTAACCGATTTGTGTTGCCAATTTTTTTTTTTAACTCAGCATTCACCTGGTTGC

TGTCAACTTTTGCCCATTTT

In100 candidate2189 (in intron):

LOCUS ENSG00000204442

DESCRIPTION similar to transmembrane protein 28 (LOC728215), mRNA

[Source:RfSeq_dna;Acc:NM_001080396]

ACGTTATTATATTCTAAATATATGTTTTAGAACTAACTATTGACATAGTTTAACTTTCTTCATACTTTCTCCATCCAGCC

TTTCTCATTATATATTTCTA

In100 candidate2190 (in intron):

LOCUS ENSG00000204442

DESCRIPTION similar to transmembrane protein 28 (LOC728215), mRNA

[Source:RfSeq_dna;Acc:NM_001080396]

AGATGCAAGTGCTTGATCAAGGCTTGCTTATTAGAAGTGCCCTAACTTTCATAGTAATTCCATGTCTTTGACACTCAGCC

ATTTTCAACTCTATTTTTAG

In100 candidate2191 (in intron):

LOCUS ENSG00000204442

DESCRIPTION similar to transmembrane protein 28 (LOC728215), mRNA

[Source:RfSeq_dna;Acc:NM_001080396]

GTGAGTTATCTGTTAACTCTGCTGATTGTTTCTTTTGCTGTGCAGAAGGCTTTTAGTTTAATTATGTCCCATCTACTTAT

CTTTGTTGCATTTGCTTTTT

In100 candidate2192 (in intron):

LOCUS ENSG00000081842

DESCRIPTION Protocadherin alpha 13 precursor (PCDH-alpha13).

[Source:Unipro/SWISSPROT;Acc:Q9Y5I0]

CCCCTTCATCTCTAAACTTCAATTATGACCATAATAACTATGGATTATTTAAATTTGCTCCTCTTTGTCTTTTAGCAGAG

TTTTGCCTTGATATTTCCAC

In100 candidate2193 (in intron):

LOCUS ENSG00000139220

DESCRIPTION Liprin-alpha-2 (Protein tyrosine phosphatase receptor type f

poypeptide-interacting protein alpha-2) (PTPRF-interacting protein alpha-2).

Source:Uniprot/SWISSPROT;Acc:O75334]

GACTGCTGCGGCTGCTCTCTAGGCTTGGAAAAGTTGTCCCCTAAATAACCATATGGCTTGCTTTCTTTACCTTTTCAGGT

CTTTACTCAAATATTTATTT

In100 candidate2194 (in intron):

LOCUS ENSG00000139220

DESCRIPTION Liprin-alpha-2 (Protein tyrosine phosphatase receptor type f

poypeptide-interacting protein alpha-2) (PTPRF-interacting protein alpha-2).

Source:Uniprot/SWISSPROT;Acc:O75334]

AAACATATGCATAATCTATCTACTTCAATCACTTCCATAGCTAACACTTAGGTTCAAGTCACCTTTATCTTTTTCCAGAT

TCTTTCAATAGTCCTCTCAT

In100 candidate2195 (in intron):

LOCUS ENSG00000139220

DESCRIPTION Liprin-alpha-2 (Protein tyrosine phosphatase receptor type f

poypeptide-interacting protein alpha-2) (PTPRF-interacting protein alpha-2).

Source:Uniprot/SWISSPROT;Acc:O75334]

CGAGCTTTCTGTGGCCTATAACATCTTCACTAATTTCTCCCCTCTGCTTTAAAAACTTGATTGTTTCAGTGCACTTACAG

GTTTATATTTTCTCTTATTT

In100 candidate2196 (in intron):

LOCUS ENSG00000178234

DESCRIPTION Polypeptide N-acetylgalactosaminyltransferase 11 (EC 2.4.1.41)

Protein-UDP acetylgalactosaminyltransferase 11) (UDP- GalNAc:polypeptide

Ncetylgalactosaminyltransferase

11) (Polypeptide GalNAc transferase 11) (GalAc-

T11) (pp-GaNTase 11). [Source:Uniprot/SWISSPROT;Acc:Q8NCW6]

AAAGCATTGTGCTCGATGCTATGGAGATACAAATAAGTCTGTAACTAAAGTAAGTATTCCTCTTTGCTAGTATTCCAGCT

CTTACCCTTCATTTCTTTTT

In100 candidate2197 (in intron):

LOCUS ENSG00000172139

DESCRIPTION sperm-specific sodium proton exchanger

[Source:RefSeq_peptide;Ac:NP_898884]

AGCTTCCTGGTTAACAACCTTGGTGCTGCCTCTAGTCTCTGTCTGTATTCTTCTGCATTTAACCTTCAGATTGTAAGCCT

TTTTGGCAAGCTTTTCTTCT

In100 candidate2198 (in intron):

LOCUS ENSG00000107165

DESCRIPTION 5,6-dihydroxyindole-2-carboxylic acid oxidase precursor (EC 1.1.18.-) (DHICA oxidase) (Tyrosinase-related protein 1) (TRP-1) (TRP1) (TRP) Catalase B) (Glycoprotein 75) (Melanoma antigen gp75). [Source:Uniprot/SWISPROT;Acc:P17643]

TCTTGTGGAAGAAGCATATATTGATTTAACAAATATCAACACCTGTATCTGTTAGTGTGATTCTTTTTTTTCCTTCAGAT ACTTTTATGTATCTCATATC

In100 candidate2199 (in intron):

LOCUS ENSG00000139826

DESCRIPTION abhydrolase domain containing 13 [Source:RefSeq_peptide;Acc:NP_16248]

ACATAATTAAGTAATTTAAATTTTGTCTCATAGAAGTAGCTGCACTTAACACACTATTACATTTTATCTCAACAGTAGTT CAATTTAATCTTTCATCCTC

In100 candidate2200 (in intron):

LOCUS ENSG00000179104

DESCRIPTION transmembrane and tetratricopeptide repeat containing 2 [SourceRefSeq_peptide;Acc:NP_689801]

GGGTTAGGGGATACTTTTTTTTCCTCCAAAAATACCTTGCTAATCTGTATAACCAAAGTAAGTTTTCTTTTGACCCAGTC ACTTTTCTAGACTTATTTTA

In100 candidate2201 (in intron):

LOCUS ENSG00000179104

DESCRIPTION transmembrane and tetratricopeptide repeat containing 2 [SourceRefSeq_peptide;Acc:NP_689801]

TTTCTTTTTCTGTCATGATTTATTTTTCATCTAGACCTAACCAAAATTATTCTGTATTCTATTGCTCAGAGGCATTTATT

GGATCTCAGTTTCTCTTCTT

In100 candidate2202 (in intron):

LOCUS ENSG00000179104

DESCRIPTION transmembrane and tetratricopeptide repeat containing 2

[SourceRefSeq_peptide;Acc:NP_689801]

AAATTTATGGAAATTAAAAAAACAATTTCTAATCTATCATTTTAACAACTACAAGCATTTTTGTGTATTTTATTCCAGTC

TTTCATCTTTGCTTCAGTAT

In100 candidate2203 (in intron):

LOCUS ENSG00000179104

DESCRIPTION transmembrane and tetratricopeptide repeat containing 2

[SourceRefSeq_peptide;Acc:NP_689801]

TATATTTTGTGTATAGTAGACACAATATTAATTTGGGGGTATATTAACCTATGAAAGAAACTATCTTCTGTTTTCAGAT

TATTTCCTCTTCTGTTCACA

In100 candidate2204 (in intron):

LOCUS ENSG00000102524

DESCRIPTION Tumor necrosis factor ligand superfamily member 13B (TNF- and

AOL- related leukocyte expressed ligand 1) (TALL-1) (B lymphocyte stimulator

(BLyS) (B cell-activating factor) (BAFF) (Dendritic cell- derived

TNFlikemolecule)

(CD257 antigen) [Contains: Tu [Source:Uniprot/SWISSPROT;Acc:Q9Y27]

ATTATATAGGGACATTTAAAGTATGATTTTAGACTAACTTCTATAGCTCTATTTTACTATTTTATCCTTATATTACAGTC

CCTCAATCTTCTTTTTATTC

In100 candidate2205 (in exon):

LOCUS ENSG00000102524

DESCRIPTION Tumor necrosis factor ligand superfamily member 13B (TNF- and

AOL- related leukocyte expressed ligand 1) (TALL-1) (B lymphocyte stimulator

(BLyS) (B cell-activating factor) (BAFF) (Dendritic cell- derived

TNFlikemolecule)

(CD257 antigen) [Contains: Tu [Source:Uniprot/SWISSPROT;Acc:Q9Y27]

GTACCATACTTAAGAATGCTGTAATACTTATCTTTTATAACATGTTTCCTTCGCTTTGCTTGTCTTTTATGTCATCAGTT

TTAACTGTTTACTTCATTTA

In100 candidate2206 (in intron):

LOCUS ENSG00000072041

DESCRIPTION Orphan sodium- and chloride-dependent neurotransmitter transporer

NTT73 (Orphan transporter v7-3) (Solute carrier family 6 member 15).

[Sorce:Uniprot/SWISSPROT;Acc:Q9H2J7]

TAACTTTTCATTTCTTTTTTGTGAACTTTCTTTACATCTGTCCTCCATGCCTCAGATATAGTTTCAGTAGTGTTTATTCT

AGTTTCTGTTGTTTCTTTTT

In100 candidate2207 (in intron):

LOCUS ENSG00000143919

DESCRIPTION CDNA: FLJ23451 fis, clone HSI06456.

[Source:Uniprot/SPTREMBL;Ac:Q9H5G8]

TTTTCCCCCCTTGGGTAATTTTTTTTAATTGTCTAAGGGATTAACTGTCACCTCATTATCTTTGTTAATCACTTTCAGAG

CTTTTCGCTATTGTTCATCA

In100 candidate2208 (in intron):

LOCUS ENSG00000161241

DESCRIPTION F-box only protein 17 (F-box only protein 26).

[Source:Uniprot/WISSPROT;Acc:Q96EF6]

TGATCGCTGGTGCCTCGTTTTTAACTTTTTCCACTTTTTATTCCTCTGGTATCTATTTCCAAATCTTTACAGCATATTTT

TGGATTGTTATTTCTTATTT

In100 candidate2209 (in intron):

LOCUS ENSG00000133640

DESCRIPTION leucine-rich repeats and IQ motif containing 1 isoform 2

[Sourc:RefSeq_peptide;Acc:NP_115541]

GATTGTTTTCTGTGCATTTGGAAAATCACTGCTGTAATTCTCTTAACAATAAATTGTATTTTTATATGTCAGTAATTATT

TTTCTACTTGTATTTATCAT

In100 candidate2210 (in intron):

LOCUS ENSG00000041515

DESCRIPTION myosin heavy chain Myr 8 [Source:RefSeq_peptide;Acc:NP_055826]

EXON

TCGGTGCCTAAAGTGATGCTTGTAACTTTTACATCATCCTTCTCCATTGCCTACTATTCATTTCCAATGTTGTTTCAGCT

CTCATTCATTCATTTGTTTG

In100 candidate2211 (in intron):

LOCUS ENSG00000041515

DESCRIPTION myosin heavy chain Myr 8 [Source:RefSeq_peptide;Acc:NP_055826]

EXON

AAAGATAGATTTCTAAATTATTTATTTTATGAATTTTTCTGATTAACACATTCTTGTTCTTGCTTCTAACTTGTCAGCA

TTCTTCTTTTTCTAAAATTA

In100 candidate2212 (in intron):

LOCUS ENSG00000041515

DESCRIPTION myosin heavy chain Myr 8 [Source:RefSeq_peptide;Acc:NP_055826]

EXON

AGAATTATTTGATTTTTATAACTTAAACATGAAAATATATCATTTTGCTTTTACCTTTTCAGCTTAACTGTGTTTTTTGT

TATTTGTTTTGTTTTGTTTT

In100 candidate2213 (in intron):

LOCUS ENSG00000180318

DESCRIPTION Cartilage homeoprotein 1 (CART-1).

[Source:Uniprot/SWISSPROT;Ac:Q15699]

CTGATGTCAAATGTGGCCTATTAAGCCCTTAACTCCATTTAAAAATGTGTCTCTTTTTCTATGTTTTTTTTTTTTCAGTT

TTCAAATTGCTTATTCTCTA

In100 candidate2214 (in intron):

LOCUS ENSG00000204894

DESCRIPTION Phosphoserine phosphatase-like protein (L-3-phosphoserinephospatase

homolog). [Source:Uniprot/SPTREMBL;Acc:O15172]

GAAGATTATTTATTTTAACCCTTCTTTGGGTTTTCTTCGTTCCCCTCTTTTTCCTGTCTCTTAACCCTTTCATTTCAGTG

TTCTTCACTTCACTTTATTA

In100 candidate2215 (in intron):

LOCUS ENSG00000134452

DESCRIPTION F-box only protein 18 (EC 3.6.1.-) (F-box DNA helicase 1).

[Souce:Uniprot/SWISSPROT;Acc:Q8NFZ0]

CGAGAGGCGTTAACTCTCCTGCAACTAATTTTCGACTTTTCCCCTCGTCTTTTTCTTTTTTCTGTTCCCGACAGTTGTCT

GTTCCCGACAGTTGTTTCCT

In100 candidate2216 (in intron):

LOCUS ENSG00000133627

DESCRIPTION actin-related protein 3-beta isoform 2

[Source:RefSeq_peptide;Ac:NP_001035225]

AACATTTTTTTCCATTAAAATTTATAACATTTACATTCTGTTCTTAATTCCCAGAAAATAAAAGTCTTTTTTTTTTTTTT

TTTTTTTTTTTTTTTGAGAC

In100 candidate2217 (in intron):

LOCUS ENSG00000068784

DESCRIPTION S1 RNA binding domain 1 [Source:RefSeq_peptide;Acc:NP_060549]

AGAACTATTTGCATATAACCACTGCAAAAGAGTTGTTTTGTTTTTCTTTGTTTACTTAGCTGCTTACAGTATACACATTT

CCACTTTATTCCCTGATGTT

In100 candidate2218 (in intron):

LOCUS ENSG00000068784

DESCRIPTION S1 RNA binding domain 1 [Source:RefSeq_peptide;Acc:NP_060549]

ACTTGTTTCCCTTATTCTAATGGCTCTTAACGATTATTCTTCCTTTTTCCGGTTTGTTTGTAGTTTTGTGTTCCTCAGAT

ATTTTTTCTTGTTGCTATCA

In100 candidate2219 (in intron):

LOCUS ENSG00000068784

DESCRIPTION S1 RNA binding domain 1 [Source:RefSeq_peptide;Acc:NP_060549]

GCTGTGGCCACCTTTTATCTTACTTTCCCTTAGACCTTCTAACCCATCACTCCTTCTTTTCATTGCTTACCCTCTCAGAA

CTATCTGCCTCTAATTCTTT

In100 candidate2220 (in intron):

LOCUS ENSG00000130226

DESCRIPTION Dipeptidyl aminopeptidase-like protein 6 (Dipeptidylpeptidase V) (Dipeptidylpeptidase 6) (Dipeptidyl peptidase IV-like protein) (Dipeptidy aminopeptidase-related protein) (DPPX). [Source:Uniprot/SWISSPROT;Acc:P4268]

CACATTAGTCAAAACTAAGAAACTGGCATTGATACACGGCTATTAACTCAACTATAGACTTTCTTTGGATTTCACCAGTT

TTCCCATGAATATTTTCTCT

In100 candidate2221 (in intron):

LOCUS ENSG00000130226

DESCRIPTION Dipeptidyl aminopeptidase-like protein 6 (Dipeptidylpeptidase V) (Dipeptidylpeptidase 6) (Dipeptidyl peptidase IV-like protein) (Dipeptidy aminopeptidase-related protein) (DPPX). [Source:Uniprot/SWISSPROT;Acc:P4268]

TTTTTAAGGTCTTGTGACATTTGGGTTATGCTGGAATTTGTTTTTAACTATTTAAAGATTTCCTATTTTGTCCCACAGTG

GTTGTCTTCTTTATCTTTAA

In100 candidate2222 (in intron):

LOCUS ENSG00000171132

DESCRIPTION Protein kinase C epsilon type (EC 2.7.11.13) (nPKC-epsilon).

[Surce:Uniprot/SWISSPROT;Acc:Q02156]

TTGATGTCTTCATGATTTGGCTTAAAAGAAAGGGTATTTCCATTCATTTAACCTAATTTAGAGTTGTCTGTCTTTCAGGA

CCTATTACTTTATTCTTTTA

In100 candidate2223 (in intron):

LOCUS ENSG00000171132

DESCRIPTION Protein kinase C epsilon type (EC 2.7.11.13) (nPKC-epsilon).

[Surce:Uniprot/SWISSPROT;Acc:Q02156]

AATATTGAAGCTTCTAATCCATGAACATGAAATAACTCTTTATTTATTTAGATCTTCCTTGATTTCTTGTGTCAGTGGTC

TATAGTTTTCTTAGTTTTCT

In100 candidate2224 (in intron):

LOCUS ENSG00000116016

DESCRIPTION Endothelial PAS domain-containing protein 1 (EPAS-1) (Member

ofPAS protein 2) (Basic-helix-loop-helix-PAS protein MOP2) (Hypoxia- inducibl

factor 2 alpha) (HIF-2 alpha) (HIF2 alpha) (HIF-1 alpha-like factor) (HLF)

[Source:Uniprot/SWISSPROT;Acc:Q99814]

TATCACCTCCCTAGGGAAGCATTTTCTCACCTCCTTTAGTTAACCTCCCTCCTGTTCTGTTTTGTTTTGTTTTAGCAGTA

CTTCCCACATGTTGTTATTT

In100 candidate2225 (in intron):

LOCUS ENSG00000198707

DESCRIPTION Centrosomal protein Cep290 (Nephrocystin-6) (Tumor antigen se2-).

[Source:Uniprot/SWISSPROT;Acc:O15078]

TTAACACATTCTCTGTTTTCTAATCTGCTTTACTCCTAATTACCTCTGTACTGTATATACTTCAGTCTGTCACTATCCAG

TTGATTTGCCTTGCTGTTTT

In100 candidate2226 (in intron):

LOCUS ENSG00000198707

DESCRIPTION Centrosomal protein Cep290 (Nephrocystin-6) (Tumor antigen se2-).

[Source:Uniprot/SWISSPROT;Acc:O15078]

TAACATACTAGACATGTACTTGTCTTTTGTTCATCTTTTTTTTTTCTTTTTTTATTAGACCATAAACTTTGATGGCAGGA

ACTTTGCCTATTTTATTTAT

In100 candidate2227 (in intron):

LOCUS ENSG00000198707

DESCRIPTION Centrosomal protein Cep290 (Nephrocystin-6) (Tumor antigen se2-).

[Source:Uniprot/SWISSPROT;Acc:O15078]

GCCTATATGTAATAACACCACAATACCCATGGTACCATTCACGTTTGTTGTTTTTCTGTTTTTCAATTGTTCTTTCAGTC

TTGGTTCCCTTAATCTTAAT

In100 candidate2228 (in intron):

LOCUS ENSG00000146910

DESCRIPTION

AGGAACAATATTAACTTATTAATAGTCTTTTTTTTTGCCACCAATAACAGGTCAGGTTTGGATTCAATCTGTTCTTTATA

CTTTGCACCCATTTTTCTTC

In100 candidate2229 (in intron):

LOCUS ENSG00000139324

DESCRIPTION transmembrane and tetratricopeptide repeat containing 3

[SourceRefSeq_peptide;Acc:NP_861448]

AGTCTTTGGCATTCCTTGGCTTGTAACTCCATCACTTCAATCTACTTTGTCTTCACATTGCCTTCTTTTCCAGTGTATCT

CTTTCTTCTCTTTTTATGAG

In100 candidate2230 (in intron):

LOCUS ENSG00000108774

DESCRIPTION Ras-related protein Rab-5C (RAB5L) (L1880).

[Source:Uniprot/SWISPROT;Acc:P51148]

AGGAATCCCATTGTCTTGCACCTTATTTGTGTTTAACATTTGGGTATCTTTGTTCTCCGTTGGTCTTTTCCAATTCAGGT

ATCATCTTTTTGATTGTCTT

In100 candidate2231 (in intron):

LOCUS ENSG00000184863

DESCRIPTION Proline-rich protein 8. [Source:Uniprot/SWISSPROT;Acc:Q9NSV0]

TTCATGACCTCTCACTGGTCTAGGACCCAAGCCACATAACCCATCGTTGTCTCTCCATTCCCCTTTGTTGTGTGACAGTT

TCTTAGTGTTTCCCTGTATT

In100 candidate2232 (in intron):

LOCUS ENSG00000119878

DESCRIPTION postsynaptic protein CRIPT [Source:RefSeq_peptide;Acc:NP_054890

GAGGAGGAGGCTGGGAGAACCTAACTGAGCTCTCCCGGGAACTTTGCTTTCTCGTTGTTTTTCTTCGCCCACAGGTCTC

AGATTCTATCCGCTGTCTTT

In100 candidate2233 (in intron):

LOCUS ENSG00000163617

DESCRIPTION KIAA1407 (KIAA1407), mRNA [Source:RefSeq_dna;Acc:NM_020817]

CTTTTTCATATCTAATGTTTTGGATACATAACCTGGACTCCTTGCCTTTGTATTTTTCTTGTCCTCTTGTGTCCACAGTT

ATTCTCCTGCGTCCTAATTT

In100 candidate2234 (in intron):

LOCUS ENSG00000105983

DESCRIPTION Limb region 1 protein homolog (Differentiation-related gene 14

rotein). [Source:Uniprot/SWISSPROT;Acc:Q8WVP7]

GAAAGATAAATTCTGGAAGCATGTATGTTTATGGATCAATAACAATAGAAAGAGCCTTCTTTTATGCTCCAGAATTTATC

TTTTTAATCATACATGTCCC

In100 candidate2235 (in intron):

LOCUS ENSG00000105983

DESCRIPTION Limb region 1 protein homolog (Differentiation-related gene 14

rotein). [Source:Uniprot/SWISSPROT;Acc:Q8WVP7]

TATCCTAAATTAACTTTACATTCTCCTGGATGACTTTATTCTGTCTTTTCTTCCCTCAGATCTTCACCTTACTCTCCACT

GGTGACCTTGTTTTCTATTT

In100 candidate2236 (in intron):

LOCUS ENSG00000171150

DESCRIPTION Suppressor of cytokine signaling 5 (SOCS-5) (CytokineinducibleSH2-containing protein 5) (Cytokine-inducible SH2 protein 6) (CIS-6). [Souce:Uniprot/SWISSPROT;Acc:O75159]

GTTTTCCTTGAGCAAAGATTTTAATTACTGATTCAAAGTCTTTAACGGCTACTGATTTTTAATTTCTTCTTGAGTCAGTT
TTGGTAATTTATGTTTTTCT

In100 candidate2237 (in intron):

LOCUS ENSG00000131504

DESCRIPTION Protein diaphanous homolog 1 (Diaphanous-related formin-1) (DRF). [Source:Uniprot/SWISSPROT;Acc:O60610]

AAAGTCTTGGATATTGATGATCATTATAATAGCTAGTTATAACTGTAGCTCTTTTGTCTTCTCTTCCAGGTATTTATGCC
CTTACACTAGTGTATCTTTC

In100 candidate2238 (in intron):

LOCUS ENSG00000068724

DESCRIPTION Tetratricopeptide repeat protein 7A (TPR repeat protein 7A). [Surce:Uniprot/SWISSPROT;Acc:Q9ULT0]

AGCATTAATTTCTAGTAAGGCTAACAATGCTATATCCCTTCTGTGTTTCTTACAGATTGTTTTATTTTTTTGAGCTTTT

TGTTTTTGGCCTTGAAGCTT

In100 candidate2239 (in intron):

LOCUS ENSG00000196243

DESCRIPTION Putative uncharacterized protein C12orf37.

[Source:Uniprot/SWISPROT;Acc:Q96LM1]

TCTTTTCAACCTTTAACTTTCATTCTTCATTATATCCATTTTCTTTCAACAGACCTCCAGATAGTGCTATTACTTTGCTT

TTATACTGTATTTTATTTTT

In100 candidate2240 (in intron):

LOCUS ENSG00000175893

DESCRIPTION Probable palmitoyltransferase ZDHHC21 (EC 2.3.1.-) (Zinc

fingerDHHC domain-containing protein 21) (DHHC-21).

[Source:Uniprot/SWISSPROT;AccQ8IVQ6]

TTGATAACTTTAACTTTGAGATCCCCTTGTGGGTCCGTTTCTGTTGACTCTTTTTTTCTTCTCAATTTCCAGATATTTGG

CCCTGTCTTCTGACATGTCT

In100 candidate2241 (in intron):

LOCUS ENSG00000134905

DESCRIPTION Probable cysteinyl-tRNA synthetase, mitochondrial precursor

(EC6.1.1.16) (Cysteine--tRNA ligase) (CysRS).

[Source:Uniprot/SWISSPROT;Acc:Q9A77]

TACACTCGCGGCATGTAATGATCTCCTTTCATTAACACAAGAGGTTCATTTGTTCTGTTTGTTTCCAGTGTGGTGGTCTC

TCTCTTCCCCTTTTCAATTT

In100 candidate2242 (in intron):

LOCUS ENSG00000177189

DESCRIPTION Ribosomal protein S6 kinase alpha-3 (EC 2.7.11.1) (S6K-alpha 3)(90 kDa ribosomal protein S6 kinase 3) (p90-RSK 3) (Ribosomal S6 kinase 2) RSK-2) (pp90RSK2) (Insulin-stimulated protein kinase 1) (ISPK-1) (MAP kinasactivated

protein kinase 1b) (MAPKAPK1 [Source:Uniprot/SWISSPROT;Acc:P5181]

AGTCTTTCAATTTTAACTTTATGTTGTTTAATTTATGCACGTTTTTTTCTTCAGGCAGATCTACCAATCTTCTTTTGTGA

TTTATTCTGACTCTCATTCT

In100 candidate2243 (in intron):

LOCUS ENSG00000139329

DESCRIPTION Lumican precursor (Keratan sulfate proteoglycan lumican) (KSPG umican). [Source:Uniprot/SWISSPROT;Acc:P51884]

AGTACATAGTGGTCATTTGAAAAGGTAACATAATAGTTTCCTTTCTTTCTTCCTTATCTTTGTACATTTAGTCAGGTATT

CTCCTTGTCTTTTTATGTAT

In100 candidate2244 (in intron):

LOCUS ENSG00000033627

DESCRIPTION Vacuolar proton translocating ATPase 116 kDa subunit a isoform (V- ATPase 116 kDa isoform a1) (Clathrin-coated vesicle/synaptic vesicle poton pump 116 kDa subunit) (Vacuolar proton pump subunit 1) (Vacuolar adenoine triphosphatase subunit Ac116). [Source:Uniprot/SWISSPROT;Acc:Q93050]

TCAAAAAAAAAAAATAATAATAAAAAATAAATAAATAGTAACATTAATGTCATTTTTATGGCATTTCACCTTCAGAGTTC

TTCAATATATTTATTTGTCT

In100 candidate2245 (in intron):

LOCUS ENSG00000095002

DESCRIPTION DNA mismatch repair protein Msh2 (MutS protein homolog 2).

[Souce:Uniprot/SWISSPROT;Acc:P43246]

AACACACAAGTTTGTTGATGCTGTTTGTCTGGGGTCCACGCTTGCCTAACTTCTGATGTGATTTATTTCTGCCAGTTTCT

TTTTTTGTTGTTGTTTTATT

In100 candidate2246 (in intron):

LOCUS ENSG00000181722

DESCRIPTION Zinc finger and BTB domain-containing protein 20 (Zinc finger

potein 288) (Dendritic-derived BTB/POZ zinc finger protein).

[Source:UniprotSWISSPROT;Acc:Q9HC78]

TATTTTTGTGGAAGCATTTTATTTCTTCTCTAATTATTTCTAACTTTTCTCTGCATTTGTCTTCCCTAGTTTGCTCAGTT

TTTCCTTTTACTACCTCTTC

In100 candidate2247 (in intron):

LOCUS ENSG00000181722

DESCRIPTION Zinc finger and BTB domain-containing protein 20 (Zinc finger

potein 288) (Dendritic-derived BTB/POZ zinc finger protein).

[Source:UniprotSWISSPROT;Acc:Q9HC78]

TAGGGCTAACATGTTTTGTTTTTCTTAATTTTCTCTGTATTTCAGATCAAATTTCACTCCTCTGTTATACTCTTATTCTT

CTAGATTTTTATGTACAATT

In100 candidate2248 (in intron):

LOCUS ENSG00000181722

DESCRIPTION Zinc finger and BTB domain-containing protein 20 (Zinc finger potein 288) (Dendritic-derived BTB/POZ zinc finger protein). [Source:UniprotSWISSPROT;Acc:Q9HC78]

TCTCTATCTCTTTATTTAACTTCTCATTTTGTTCTTGTATCTTTTTCCTAATTTTATTTAGCTATCTCTCAGTGTTCTCT
TGTAACTCACTGGGCTTCTT

In100 candidate2249 (in intron):

LOCUS ENSG00000181722

DESCRIPTION Zinc finger and BTB domain-containing protein 20 (Zinc finger potein 288) (Dendritic-derived BTB/POZ zinc finger protein). [Source:UniprotSWISSPROT;Acc:Q9HC78]

GTAACCAATTTTGGTGTGAGTTATGATTACTTATTTTCCCTTTTTTTCAATTGCACTATTATTGTTTTCTCTTAGCAGAA
TTTGTCTCACTCTTTATCAC

In100 candidate2250 (in intron):

LOCUS ENSG00000155093

DESCRIPTION Receptor-type tyrosine-protein phosphatase N2 precursor (EC 3.13.48) (R-PTP-N2) (Islet cell autoantigen-related protein) (ICAAR) (IAR) (Phgrin). [Source:Uniprot/SWISSPROT;Acc:Q92932]

GTCAAGTAGTGACAATTCTCTGGGAATGGGGCTTTGAAGGCACTCTAACCCCACCCTGTGCTTTCTGGTGGCTTCCAGGC

TGTTGGTTTTTACTGCCATT

In100 candidate2251 (in intron):

LOCUS ENSG00000155093

DESCRIPTION Receptor-type tyrosine-protein phosphatase N2 precursor (EC

3.13.48) (R-PTP-N2) (Islet cell autoantigen-related protein) (ICAAR) (IAR)

(Phgrin). [Source:Uniprot/SWISSPROT;Acc:Q92932]

CCCTCTAGCACGGGGACCCTAAAAAATTCTTGATGTTGAAGCAATATAACTTGTGTTGCAATTTTACTTCTTTGGCAGTG

TTGTCTTTTCCTTTGCGATG

In100 candidate2252 (in intron):

LOCUS ENSG00000185565

DESCRIPTION Limbic system-associated membrane protein precursor (LSAMP).

[Surce:Uniprot/SWISSPROT;Acc:Q13449]

CACTTAACATCTCTCTTCCTTAACATCTCAATTCTGTTTCTTCTTATTTTCTCTCCCATTATTACTACCCATACACAGGC

CCATATTATTTCTTGCTTAG

In100 candidate2253 (in intron):

LOCUS ENSG00000185565

DESCRIPTION Limbic system-associated membrane protein precursor (LSAMP).

[Surce:Uniprot/SWISSPROT;Acc:Q13449]

TTCTATTTTTTCCATTAGGTAAGGAGAGAATATTTTAACATTTCATTCCTTTTCTACATTTATCTTTTTCAGGTTTATTC

TTCTAGTGGAGTTTGCTTTC

In100 candidate2254 (in intron):

LOCUS ENSG00000185565

DESCRIPTION Limbic system-associated membrane protein precursor (LSAMP).

[Surce:Uniprot/SWISSPROT;Acc:Q13449]

GTTTTTGCCTCTTGCTAACATTCCTGTTTACGCTTTGTAATATTTTTTCTCACAGACTAATCATATATATTATATAACTA

ATCTTTTTCATTTTTCCTCT

In100 candidate2255 (in intron):

LOCUS ENSG00000185565

DESCRIPTION Limbic system-associated membrane protein precursor (LSAMP).

[Surce:Uniprot/SWISSPROT;Acc:Q13449]

AGAGAAGAAAGTAATGAACATAGGAGCGCCTGAGGAAACTAACATTTCCACTGAAATCTTCTTCAACTTCAGAAATGCCC

CTTTTCCTTTTTTTCAGTTC

In100 candidate2256 (in intron):

LOCUS ENSG00000185565

DESCRIPTION Limbic system-associated membrane protein precursor (LSAMP).

[Surce:Uniprot/SWISSPROT;Acc:Q13449]

AACTCGTGCTAACTATGCTCAATGTTCTTTTGAACTCAGTTAACTCAAATAGCTCTCAAGCATGTTTTATTTCTTTTGCT

TTCTAACCCCTACATTCCTT

In100 candidate2257 (in intron):

LOCUS ENSG00000155158

DESCRIPTION C9orf52 protein (Chromosome 9 open reading frame 52). [Source:Uiprot/SPTREMBL;Acc:Q8IXZ6]

TGTGGCAATTGGAGACAATCATCTCTTCCGTAACTCTGTTACGACTGACCTGTTCTGTTGTCTCTAGCTGTTTCTCAGCT
TTCTTAATTTTGGCAGTTCT

In100 candidate2258 (in intron):

LOCUS ENSG00000102189

DESCRIPTION Early endosome antigen 1 (Endosome-associated protein p162) (Zic finger FYVE domain-containing protein 2). [Source:Uniprot/SWISSPROT;Acc:Q5075]

TAACATGACTATTCATAGTGTTTCCTGCACCTTTGTTTATGCTCCTCCATTTGCTTAGAATGCTTTTCTCTCTGCCAGGT
AAACTCCTATTTATTCTTTC

In100 candidate2259 (in intron):

LOCUS ENSG00000013561

DESCRIPTION E3 ubiquitin-protein ligase RNF14 (EC 6.3.2.-) (RING finger proein 14) (Androgen receptor-associated protein 54) (Triad2 protein) (HFB30).[Source:Uniprot/SWISSPROT;Acc:Q9UBS8]

GTAGAATTCTTAATCAAATAAATGTTTATGCATATAATTAACATTTTAATTGGTCTCTTATATTCCTTCTTGGTTCAGTT
CTTGTTTGTTGCTGTACCCT

In100 candidate2260 (in intron):

LOCUS ENSG00000170802

DESCRIPTION Forkhead box protein N2 (Human T-cell leukemia virus enhancer fctor). [Source:Uniprot/SWISSPROT;Acc:P32314]

AGTACTATTAACTACATACTTTATTCCTATTTCGCTAATTTTTCCCTAATGTCTTTTTTTTGTTCCAGAATTTCACTTTC
TATGACTTCTCTTTGTTTTG

In100 candidate2261 (in intron):

LOCUS ENSG00000068781

DESCRIPTION Stonin-1 (Stoned B-like factor).

[Source:Uniprot/SWISSPROT;Acc:9Y6Q2]

AAAATGTCTGAAGAATAAAAGAAAAACCCCTAGAGATAACCACCATTAACATATTGGTGTATTTTTTTCCAGCCTTTGTG

GATGAGATTTTTTTTTTTT

In100 candidate2262 (in intron):

LOCUS ENSG00000068781

DESCRIPTION Stonin-1 (Stoned B-like factor).

[Source:Uniprot/SWISSPROT;Acc:9Y6Q2]

TTGATTCATTTGGTGTTTGTCTTGGTGTATAATGTAACATATGGTTTCAACTTTTTATTTTTTTTTTCCAGGTGGCTAC

TCAATCTTCCTAACATTTTT

In100 candidate2263 (in intron):

LOCUS ENSG00000138039

DESCRIPTION Lutropin-choriogonadotropic hormone receptor precursor (LH/CG-R

(LSH- R) (Luteinizing hormone receptor) (LHR).

[Source:Uniprot/SWISSPROT;Ac:P22888]

AAACAAGGAAAAGGTTGGAAAGATTTTGTTTAACCATATACATGGCACTATTTGCATGTTCCTCTTCAGCCTTCTGATTT

AAATTTAATTGTCATTTCTT

In100 candidate2264 (in intron):

LOCUS ENSG00000173588

DESCRIPTION Coiled-coil domain-containing protein 41 (Renal carcinoma antign NY- REN-58). [Source:Uniprot/SWISSPROT;Acc:Q9Y592]

TTTCGTTGAATTTTCTATCTGTATTCTCTTGTATCTTTCCAAGTTTCCTTAACATTGTTATTTTAATTTCTTTTCAGGC ATTTTTTAGATTTCTTTTTC

In100 candidate2265 (in intron):

LOCUS ENSG00000170820

DESCRIPTION Follicle-stimulating hormone receptor precursor (FSH-R) (Follitopin receptor). [Source:Uniprot/SWISSPROT;Acc:P23945]

CTTTTGGTATCCTCTCTCCCACATAGACTTCCCTGCCTCCAATAACCACTATTCTACTCTCTGCTTCTTTGAAGTCAGAC TTTTTTAGCTTCCACATATG

In100 candidate2266 (in intron):

LOCUS ENSG00000170820

DESCRIPTION Follicle-stimulating hormone receptor precursor (FSH-R) (Follitopin receptor). [Source:Uniprot/SWISSPROT;Acc:P23945]

ACATCCCCTTGAGTATGCTAACTCCTTATTTTGATTCTCTTCTGTTTTATTTTACAGTCCCTTTTATCATCTTTTCCCAG TCCAATCATTAAACTTTGTT

In100 candidate2267 (in intron):

LOCUS ENSG00000145819

DESCRIPTION Rho GTPase-activating protein 26 (Oligophrenin-1-like protein) GTPase regulator associated with focal adhesion kinase). [Source:Uniprot/SWSSPROT;Acc:Q9UNA1]

ACATACTGACGTACTGATTCTTTGAAAATAAATTTAAAACTTATATCTAACAATCCCTCCTCTTGCATTTCCTTACAGCT

TTCTTTTTAAACTTTTTAAC

In100 candidate2268 (in intron):

LOCUS ENSG00000145819

DESCRIPTION Rho GTPase-activating protein 26 (Oligophrenin-1-like protein)

GTPase regulator associated with focal adhesion kinase).

[Source:Uniprot/SWSSPROT;Acc:Q9UNA1]

AGCTCTGCCCTGTAACTAGGCCACGTTTCCTTGACATTTTCAGTTTGTTACTGGTGTGACCCATTTTCTGAGTGGCTTCA

TGTCCACTTTTCTCTTTCCC

In100 candidate2269 (in intron):

LOCUS ENSG00000120798

DESCRIPTION Orphan nuclear receptor TR2 (Testicular receptor 2).

[Source:Unprot/SWISSPROT;Acc:P13056]

ATAGACTAAAAGCCTGATTTAGGATGGTTAACCTTTAAAATGATTGTTAGTCCTTTTTTAACATTCAATTTTCAGCATTT

CTCTAGTTTTCTGGGGTTTT

In100 candidate2270 (in intron):

LOCUS ENSG00000179915

DESCRIPTION Neurexin-1-beta precursor (Neurexin I-beta).

[Source:Uniprot/SWSSPROT;Acc:P58400]

AGAAAATATAAGCATATTTAAATAAGACAATGTCACCGCATGTAATCATTAACATAAGTAAGGATTTGTCTTATGCAGAG

TTGCTTTTATTGTTTGTTTT

In100 candidate2271 (in intron):

LOCUS ENSG00000179915

DESCRIPTION Neurexin-1-beta precursor (Neurexin I-beta).

[Source:Uniprot/SWSSPROT;Acc:P58400]

AATTTGAAATTCAATCTGCTTGTTACCTAACCTTGTGCCTATTCCCTCATCTATATTTACCTATTAAATATTTTCCAGTA

ATATTTCCTTTGTATTCTTA

In100 candidate2272 (in intron):

LOCUS ENSG00000164989

DESCRIPTION Uncharacterized protein C9orf93.

[Source:Uniprot/SWISSPROT;Acc:6TFL3]

CCATACTTAAATTTCTTGTTTTTTTGCTTTTCAAATTTTTAACATTCTAGAGTTTCCTTATCCTAATTTGTTGCACAGAA

TTCTTTTCTTTGCCCCTTTT

In100 candidate2273 (in intron):

LOCUS ENSG00000164989

DESCRIPTION Uncharacterized protein C9orf93.

[Source:Uniprot/SWISSPROT;Acc:6TFL3]

GTGCGTATAAAATATGTGCCAAATTGTTCTCTTAAGTGGGTGTAACAATTTCAAAGAAGAGTTTCTCTTCCTTTACAGCC

TCTTGGTATTTTTCAGATTT

In100 candidate2274 (in intron):

LOCUS ENSG00000164989

DESCRIPTION Uncharacterized protein C9orf93.

[Source:Uniprot/SWISSPROT;Acc:6TFL3]

TGTAACATACTTTTATTCTTACTCTTATTCTCCATATTCTACATTTCTTATTGGGTTATTTTTTATCCCAGCCCCATTG

CTGTGGTCTTAGCTCATTTT

In100 candidate2275 (in intron):

LOCUS ENSG00000164989

DESCRIPTION Uncharacterized protein C9orf93.

[Source:Uniprot/SWISSPROT;Acc:6TFL3]

AGATAAAAAATCATGGAAAGAAAAATTTAACCTTTCAAACCTTTTTTTTCTACCCAAACTCTAATTCAGTTCTTTTTTCC

AAAGATTATTAAGTTTCTTT

In100 candidate2276 (in intron):

LOCUS ENSG00000125772

DESCRIPTION Putative glycerophosphodiester phosphodiesterase 5 (EC 3.1.-.-)

[Source:Uniprot/SWISSPROT;Acc:Q9NPB8]

AGCTCATGTGCATACAATGACTAACATTTTTCATCTTACTCTTGTTTTTATGTATATCCAGAACTGACCTTCCCTTTCCA

AGTTGTGATTTTTTTTTTTC

In100 candidate2277 (in intron):

LOCUS ENSG00000180263

DESCRIPTION FYVE, RhoGEF and PH domain-containing protein 6 (Zinc finger FYE domain-containing protein 24). [Source:Uniprot/SWISSPROT;Acc:Q6ZV73]

CCTTTTGAAGCTTGTTAACTTCATTCTTGATTATTCTGTCCCTCATTTTCTTTGCCTTCATAACTTTAAAATGATCAGAA

TCTGTCATCTTGGCTTTCCT

In100 candidate2278 (in intron):

LOCUS ENSG00000205000

DESCRIPTION

AATGCAAGATCTATGTTGAGGCCAAATTTAACTCACTTTTAAATTTTTGTATAAATCCTGGTTTTGCTTTTATTTCAGAA

TGTTACTGTTTTTTTGTTTG

In100 candidate2279 (in intron):

LOCUS ENSG00000028203

DESCRIPTION Vezatin. [Source:Uniprot/SWISSPROT;Acc:Q9HBM0]

CAATATGATTATTATAACTCTTTCCAAATATTGCTGTTTTTCAGCTATTCTCAGACACATCCCCCTTATACTCCCCTCTT

TCTTTTCTTTTCTTTTTTTT

In100 candidate2280 (in intron):

LOCUS ENSG00000082701

DESCRIPTION Glycogen synthase kinase-3 beta (EC 2.7.11.26) (GSK-3 beta). [Surce:Uniprot/SWISSPROT;Acc:P49841]

GGTTACCTGTATATAACCTTGAGCAATTTTACTTATTTTTTGAGTCTCAGTTCCCTTCTCCCTGATTTAGTACTGGTTCT

TCCTCCTTTGTAGGACTGTT

In100 candidate2281 (in intron):

LOCUS ENSG00000082701

DESCRIPTION Glycogen synthase kinase-3 beta (EC 2.7.11.26) (GSK-3 beta).

[Surce:Uniprot/SWISSPROT;Acc:P49841]

TGTCCTTCTAGTCTGTCATTTTCTCTAACTGGGACTACATTCTCATTTTAGCAGAGCTATGGGCCTTTCCCCTTTCTGTC

CTTCCAAATTTGCTATTTTT

In100 candidate2282 (in intron):

LOCUS ENSG00000082701

DESCRIPTION Glycogen synthase kinase-3 beta (EC 2.7.11.26) (GSK-3 beta).

[Surce:Uniprot/SWISSPROT;Acc:P49841]

ATGCATTTGGGAATTTAAAAAAATTCTCTCATTCTCTATAACATCTCTGACTTTCTTTCCCTCTTTTTTTTCTATCAGTC

TCTTTCTCTAGCCCTTTTTT

In100 candidate2283 (in intron):

LOCUS ENSG00000111142

DESCRIPTION Methionine aminopeptidase 2 (EC 3.4.11.18) (MetAP 2) (PeptidaseM

2) (Initiation factor 2-associated 67 kDa glycoprotein) (p67) (p67eIF2).

Source:Uniprot/SWISSPROT;Acc:P50579]

CTCATCTGTGCTCATTATTCTCTGTGATCCTGCTGTAATAACTTCCTTATAGACTATTTTCTATATTTCCTTTAACAGAA

TTCTGTCTCTCTTTGTAGAC

In100 candidate2284 (in intron):

LOCUS ENSG00000068912

DESCRIPTION XTP3-transactivated gene B protein precursor.

[Source:Uniprot/SISSPROT;Acc:Q96DZ1]

TTCTTAGAGTGGATTTTTCTTGTGTTTATGTAGAGAAAAGTGTAGGAGCTAACTTCTTAGTGATCTTTGTCTTCTCAGGT

TCTATCCTTTATTCCTTCAT

In100 candidate2285 (in intron):

LOCUS ENSG00000205549

DESCRIPTION

CTTTAAGTCCCCTGTGAGCTTCCCAATCAATTACAATTTCTCCTGCTTTTAACTACCAACACACTACATATCTTTCAGTT

GGCATTCTCTTAACTTTTTA

In100 candidate2286 (in intron):

LOCUS ENSG00000171984

DESCRIPTION Novel protein. [Source:Uniprot/SPTREMBL;Acc:Q5TGA6]

TGTGTTGGGACTAGAGAGGAGTTTGGCTACTCATACTTTTAACTTTTGTTGCTTGTGTTTCTCATTCAGAAGTGGTTACC

TTTGTTTCATTCATTTGTTT

In100 candidate2287 (in intron):

LOCUS ENSG00000173068

DESCRIPTION Zinc finger protein basonuclin-2.

[Source:Uniprot/SWISSPROT;AccQ6ZN30]

CATTCCGATTAAAAAAAAGAATTGATCCTTTAACAACAAAATAATATCCTTTTGTTTTTCTTTAATTAAGCCCATCAGTT

TTTCATTTCTCTCTGTAAAG

In100 candidate2288 (in intron):

LOCUS ENSG00000163430

DESCRIPTION Follistatin-related protein 1 precursor (Follistatin-like 1).

[ource:Uniprot/SWISSPROT;Acc:Q12841]

AGAGGAATGATTGTGTAACTAGTGGCTCCCCCCTTCTCTGTGTGCTCTTCAAACGGGCCTTTTGTTTTTTCCATTCAGTG

AGTCCTTCTGTTCTCTATTA

In100 candidate2289 (in intron):

LOCUS ENSG00000044459

DESCRIPTION Uncharacterized protein C9orf39.

[Source:Uniprot/SWISSPROT;Acc:9NXG0]

GTAGATTATTGATTTGAGATTTTTCCTGTTTTGTAACGTACACCTTTAATTTTATAAATTTTCCTCTCAGCTGCTTTAAC

TTCATCCCCCATATTTTGAT

In100 candidate2290 (in intron):

LOCUS ENSG00000044459

DESCRIPTION Uncharacterized protein C9orf39.

[Source:Uniprot/SWISSPROT;Acc:9NXG0]

TTAGATGATAATTGCTCTTTAACATTATATTTCATTTTTTCCTTACCTTCTAGGAAGCTCAGAAGTTTTGTTTCATAATT

GCAATTACTTCTTTTATCCC

In100 candidate2291 (in intron):

LOCUS ENSG00000044459

DESCRIPTION Uncharacterized protein C9orf39.

[Source:Uniprot/SWISSPROT;Acc:9NXG0]

GTTAAGGCCAATATTTGAACCCCTCCATTTAACACCTTTAATTTTTATCATATTTCAAGCTTATCTCTTTATCAGCTTCT

GAATCACATTGTCTCATTTT

In100 candidate2292 (in intron):

LOCUS ENSG00000044459

DESCRIPTION Uncharacterized protein C9orf39.

[Source:Uniprot/SWISSPROT;Acc:9NXG0]

TATGTTAGATTATTTTTTGTCCGGCCTTCATATCTATTTTCTTAACAATTCTTTAATCTTTTGTCTTCTGCATCCAGTTT

ATCTCAAGACTTTCCTTCAT

In100 candidate2293 (in intron):

LOCUS ENSG00000153767

DESCRIPTION Transcription initiation factor IIE subunit alpha (TFIIEalpha)(

General transcription factor IIE subunit 1) (General transcription factor

IE 56 kDa subunit). [Source:Uniprot/SWISSPROT;Acc:P29083]

AGATAATGAGAGGGTTGGAGTATTAACCTTTGTCTTAGCTGTGTTTCTATACTTTACCCCAGAGCATTTGCTTTTATGTT

TATATATTAATCACCTTTCC

In100 candidate2294 (in intron):

LOCUS ENSG00000145087

DESCRIPTION Syntaxin-binding protein 5-like (Tomosyn-2) (Lethal(2) giant

lavae protein homolog 4). [Source:Uniprot/SWISSPROT;Acc:Q9Y2K9]

AATTTACATCATGTGAACTTTAATTCATGTTTTATAACTTTTATGAAACTCCCTCTTATTCTTTTTTCAATGGCTCAGGT

TTTTTTGCTCCCTGTCTCTT

In100 candidate2295 (in intron):

LOCUS ENSG00000145087

DESCRIPTION Syntaxin-binding protein 5-like (Tomosyn-2) (Lethal(2) giant

lavae protein homolog 4). [Source:Uniprot/SWISSPROT;Acc:Q9Y2K9]

TTTAAAGATAACTTGTATTTTATATTTACCCATATATTTATCATTTTAAATGTTCTTTTTTCCTTTTTGTATATCCAGAT

ACTATTTGATATCATTTCCC

In100 candidate2296 (in intron):

LOCUS ENSG00000145087

DESCRIPTION Syntaxin-binding protein 5-like (Tomosyn-2) (Lethal(2) giant

lavae protein homolog 4). [Source:Uniprot/SWISSPROT;Acc:Q9Y2K9]

TCCACCTCATTTATCATGCACCTTTTTCCATAACTGATTTTTCCCTTAGCTCTCCTTTACGTTATTGATATTCTTCAGGA

TATTATGTCTTTACCCTCAT

In100 candidate2297 (in intron):

LOCUS ENSG00000145087

DESCRIPTION Syntaxin-binding protein 5-like (Tomosyn-2) (Lethal(2) giant

lavae protein homolog 4). [Source:Uniprot/SWISSPROT;Acc:Q9Y2K9]

CTATCTCATGCAAAATTATTCTCTTATCTTTACATCTATAACTCTCCTTACATCTCTTTCTGTCTCTACTATTTACAGTT

CCTTTCTATCTTGTTTCTAT

In100 candidate2298 (in intron):

LOCUS ENSG00000145087

DESCRIPTION Syntaxin-binding protein 5-like (Tomosyn-2) (Lethal(2) giant lavae protein homolog 4). [Source:Uniprot/SWISSPROT;Acc:Q9Y2K9]

TGCCTGGCTAACAATTACGTATTTTCTCTAGTCTCTTTAAATCTCCAAAACTTGCTTTTCTGTCTTCAATCTCAGCAATC
ACCATGTGTCCTCTTTTATT

In100 candidate2299 (in intron):

LOCUS ENSG00000107295

DESCRIPTION SH3-containing GRB2-like protein 2 (EC 2.3.1.-) (Endophilin-1) Endophilin-A1) (SH3 domain protein 2A) (EEN-B1). [Source:Uniprot/SWISSPROT;cc:Q99962]

TTTCCTCATCTTTAGTATTGATATTGCTTAACTTGTTTTAAATTCTTATTTTGTCTGTTTTCTCACTCATTACTTCAGTA
GTTATTTTTCCTCTATTTTT

In100 candidate2300 (in intron):

LOCUS ENSG00000091009

DESCRIPTION RNA-binding protein 27 (RNA-binding motif protein 27). [Source:niprot/SWISSPROT;Acc:Q9P2N5]

GAAGTACCAATATATGTGTATCTTTAATAAATATTAACAAATGTGAAAGTCAACTCCATTTTTACTTACTTTGTCCAGTA
TGGTCTTATCTTTTTTTTTT

In100 candidate2301 (in intron):

LOCUS ENSG00000147892

DESCRIPTION ADAMTS-like protein 1 precursor (ADAMTSL-1) (Punctin-1). [Sourc:Uniprot/SWISSPROT;Acc:Q8N6G6]

TCCAATTCATAACACATGATATCTTTCCATTAATTTGTGTTTTCTTCAATTTTTTCATCAATCTTTTATAGTTTTCAGTG

TCTTTCACTTTCTTGATTAA

In100 candidate2302 (in intron):

LOCUS ENSG00000165990

DESCRIPTION Uncharacterized protein C12orf55 (Fragment).

[Source:Uniprot/SPREMBL;Acc:Q96N23]

GTAGTCAAAACTATATTTGACTGAAAATGTCAAATGTGCCTTGATAACTTATCAAAATACATTTGACCTTTTCAGTTAGA

CATTTACTCTATTTACTTTT

In100 candidate2303 (in intron):

LOCUS ENSG00000156475

DESCRIPTION Serine/threonine-protein phosphatase 2A 55 kDa regulatory subunt

B beta isoform (PP2A, subunit B, B-beta isoform) (PP2A, subunit B, B55- bta

isoform) (PP2A, subunit B, PR55-beta isoform) (PP2A, subunit B, R2-beta soform).

[Source:Uniprot/SWISSPROT;Acc:Q00005]

CCCTTGTTTGTTCTGCTCTAGGCACACTGATCCCTTAACACATGTTCATTTCCCTCTTAGGGTCTTTCCATTGTCAGTTT

CATCTCTTTGGAACACTCTT

In100 candidate2304 (in intron):

LOCUS ENSG00000188596

DESCRIPTION Putative uncharacterized protein C12orf63.

[Source:Uniprot/SWISPROT;Acc:Q6ZTY8]

GGTGATAACCCCTTTATCATTTTTATTGTGTCTATCTGATTCTTCTCTCTTTTTTTCTTTATTAGTCTTGCTAGCAGTC

TATCAATTTTGTTGATCCTT

In100 candidate2305 (in intron):

LOCUS ENSG00000088766

DESCRIPTION Cardiolipin synthetase (EC 2.7.8.-) (Cardiolipin synthase) (CLS

(Protein GCD10 homolog). [Source:Uniprot/SWISSPROT;Acc:Q9UJA2]

TTCGAGGGCTCTGGGCTTTAGCTACTGTCACCCCATCATAACTGAGCTTCATGGATTGATTCTCTTTTTATCTTTCAGAT

TTTCTTTTAAAAATCTTTGT

In100 candidate2306 (in intron):

LOCUS ENSG00000169302

DESCRIPTION Serine/threonine-protein kinase 32A (EC 2.7.11.1) (YANK1).

[Souce:Uniprot/SWISSPROT;Acc:Q8WU08]

ACATACTGACATATTAAGTCTTTGAAAAGAAATTTAGAACTCATATCTAACAATCCCTCCCCTTGTATTTCCTTACAGCT

TTCTTTTCAAACTTTTTTTT

In100 candidate2307 (in intron):

LOCUS ENSG00000178031

DESCRIPTION Uncharacterized protein C9orf94 precursor.

[Source:Uniprot/SWISPROT;Acc:Q496M8]

GTTTAAGATTAAAAAAAAATCAATCACACATTTTTTTTCTCCTATTATAACTTTGAATATTTTGTTATATTTTTCCAGTC

TCTTTTTTTAATTATACTTG

In100 candidate2308 (in intron):

LOCUS ENSG00000176049

DESCRIPTION Janus kinase and microtubule-interacting protein 2.

[Source:Unirot/SWISSPROT;Acc:Q96AA8]

GTTATATTTTTCTGTTTTATAACTTCTATTTTGTTCTTTCCCAAATTTCCTATATAATATCACTTTTTATATTTTCAGGT

TGCCTTTCAAATTTTTCATG

In100 candidate2309 (in intron):

LOCUS ENSG00000176049

DESCRIPTION Janus kinase and microtubule-interacting protein 2.

[Source:Unirot/SWISSPROT;Acc:Q96AA8]

CCTTCTGCCTAGAATATTCTTCTTTCCCACCATAACCCTTTGCTTACTGGTTCCTTCCTTCTTATTTTACAGATCTTAGT

TACATATGTCACTTTTATTT

In100 candidate2310 (in intron):

LOCUS ENSG00000176049

DESCRIPTION Janus kinase and microtubule-interacting protein 2.

[Source:Unirot/SWISSPROT;Acc:Q96AA8]

ACACCTTACATTATGGTTTTAATTTTCCTCATTATTAAGAAGGTAGGGTAACTTTTCAAATATTTACTAGTCTTTCAGAT

ATAATTTGTCCTTAGCCTTT

In100 candidate2311 (in intron):

LOCUS ENSG00000163406

DESCRIPTION Oligopeptide transporter, kidney isoform (Peptide transporter 2

(Kidney H(+)/peptide cotransporter) (Solute carrier family 15 member 2).

[ource:Uniprot/SWISSPROT;Acc:Q16348]

AAAAAGAATCTCTGTGGTAACAAGGAAAGGCTATTCTTTTCATTATCAATTTTTCCAATGTGTTTACATCCTCTTCAGTT

GCGGTATCTTTTGTTCCTTA

In100 candidate2312 (in intron):

LOCUS ENSG00000145103

DESCRIPTION immunoglobulin-like domain containing receptor 1

[Source:RefSeqpeptide;Acc:NP_787120]

TTTTGTCTATAACACTTAAGTCTGATTTTTTTTTTTTTTACATTCGGCATCTGATTTTCCCCAAGCCAGCCCTCAGTTTT

AGGGTACATCTGTCCTTCTT

In100 candidate2313 (in intron):

LOCUS ENSG00000155875

DESCRIPTION Uncharacterized protein C9orf138.

[Source:Uniprot/SWISSPROT;AccQ8IYX7]

ATTTGCATCTCTTTGTTATATGCGTACCAAATAACCATTAAAGTTGCTTTTCTCTGACTTATACTTCAGGGGTTGGCCCT

GCTTTCTGTTATCCAATTTC

In100 candidate2314 (in intron):

LOCUS ENSG00000125845

DESCRIPTION Bone morphogenetic protein 2 precursor (BMP-2) (BMP-2A).

[Sourc:Uniprot/SWISSPROT;Acc:P12643]

CCATTCCCTTGTTGTATTTTTTTTAAAATGGAATGTTTTTAACCTTTTGTATTTGATATTTTTTTTCTCCTTGATCAGTT

GTCTGTTATTTTATTATCTG

In100 candidate2315 (in intron):

LOCUS ENSG00000133710

DESCRIPTION Serine protease inhibitor Kazal-type 5 precursor (Lymphoepitheial Kazal-type-related inhibitor) (LEKTI) [Contains: Hemofiltrate peptide H6478; Hemofiltrate peptide HF7665]. [Source:Uniprot/SWISSPROT;Acc:Q9NQ38]

ACATTTTGTGTGTCTTGTTCATAACTTGGTCAAACCTACCTTCCTTGACATTTTGCATAACTTTGGGTTTTCCTCCAGCA

TTTTTCTCTCTCTTCTATCT

In100 candidate2316 (in intron):

LOCUS ENSG00000197658

DESCRIPTION

TTTTATAAGATTAACGCTTTACAAAACCTTTTCACTTTGCTTAAACTTTCAGTTTTGTCTTATTCTCTCTTAGTTTAAGA

CAGTCTTTAAAACCCCCTCT

In100 candidate2317 (in intron):

LOCUS ENSG00000120868

DESCRIPTION Apoptotic protease-activating factor 1 (Apaf-1). [Source:Unipro/SWISSPROT;Acc:O14727]

AGGACTTTGTTAACTGTTACTTCTCTTGTTGTATTAGTCTCTGCCACAAGTCTTCTTTCTTGCTTTTCTGCATGCAGCTT

CTAAGATATTTTTTTTCAT

In100 candidate2318 (in intron):

LOCUS ENSG00000147874

DESCRIPTION Protein FAM29A. [Source:Uniprot/SWISSPROT;Acc:Q7Z4H7]

AGATTTAACATATGAAAAAACTGGATTTTGTTCTAACTTCCTCCAGTAATATTCCTATTTTCTTTATTGCCTTTTTTTTT

CTCTATTATCCCATTTTGGA

In100 candidate2319 (in intron):

LOCUS ENSG00000184999

DESCRIPTION Solute carrier family 22 member 10 (Organic anion transporter 5.

[Source:Uniprot/SWISSPROT;Acc:Q63ZE4]

AAGGGCTTAACACTGCCATTTTGTTACCGATTTTCTACTTGTTTTGTACATTTTTTTCCCCTGCTTTCACTTTTACAGTT

TTTATTTGTGGTTTTCTCTA

In100 candidate2320 (in intron):

LOCUS ENSG00000165632

DESCRIPTION Transcription initiation factor TFIID subunit 3 (TBPassociatedfactor

3) (Transcription initiation factor TFIID 140 kDa subunit) (140

kDa ATA box-binding protein-associated factor) (TAF140) (TAFII140).

[Source:Unirot/SWISSPROT;Acc:Q5VWG9]

AGTTAACACAACTTAGTTTTGCTCTTTTTATTCAGCATTATTTATGTTCTGCTTTTTTTTAACGTATCATTTGTTATGTT

TTCCTTGTTCCTGTGTATTT

In100 candidate2321 (in intron):

LOCUS ENSG00000163001

DESCRIPTION Coiled-coil domain-containing protein 104.

[Source:Uniprot/SWISPROT;Acc:Q96G28]

AGTTTCTAACCATGCTACTTGTTATTGTACCATTTCCTTTCTAACCCAGATTATCATCTCTGTTTCAAATTTCTTGCTGT

GGTAACCTCACCTTAATTTC

In100 candidate2322 (in intron):

LOCUS ENSG00000163001

DESCRIPTION Coiled-coil domain-containing protein 104.

[Source:Uniprot/SWISPROT;Acc:Q96G28]

TTTTGGCAAGTTTAAGCATTTGTTTTTAACTTTTTATACTTTTTTCTCCTTCTTTATTTTTGTCCTGGTAACTCACAGAA

CAACATTTTTTTTTTTTTT

In100 candidate2323 (in intron):

LOCUS ENSG00000163001

DESCRIPTION Coiled-coil domain-containing protein 104.

[Source:Uniprot/SWISPROT;Acc:Q96G28]

CATTACTGGTATAAAGGAAAGTGATAAACTGTTATTAATTTGTATCCTGTAACCTTGCTATATAATTATTCATTTCAGTT

TTTCTGTCAATTGTTTTGCA

In100 candidate2324 (in intron):

LOCUS ENSG00000114030

DESCRIPTION Importin alpha-1 subunit (Karyopherin alpha-1 subunit) (SRP1-bea) (RAG cohort protein 2) (Nucleoprotein interactor 1) (NPI-1). [Source:Unirot/SWISSPROT;Acc:P52294]

GTGTGTTAAAGATTGATCCATGTTGTAACATGTATTGGTACTTCATTCCCTTTTCTCACTAAATATTCCATTGTGCAGAT
ATACCACTTTTTGTTTATTG

In100 candidate2325 (in intron):

LOCUS ENSG00000114030

DESCRIPTION Importin alpha-1 subunit (Karyopherin alpha-1 subunit) (SRP1-bea) (RAG cohort protein 2) (Nucleoprotein interactor 1) (NPI-1). [Source:Unirot/SWISSPROT;Acc:P52294]

TTTTGCCTTTTTCACTGTGCCTGTATTTTATGTTTCACACTAACATTCTCCCCCCATTTTACTTTGCCCATTCTACAGAA
GTTTTTAAATCTTTTTTATT

In100 candidate2326 (in intron):

LOCUS ENSG00000182621

DESCRIPTION 1-phosphatidylinositol-4,5-bisphosphate phosphodiesterase beta (EC 3.1.4.11) (Phosphoinositide phospholipase C) (Phospholipase C- beta-1)(PLCbeta-
1) (PLC-I) (PLC-154). [Source:Uniprot/SWISSPROT;Acc:Q9NQ66]

TGATAAACCACTGTTAGATCCTCAAATGCTTCTCGTGAGGATGGATTTTTAACACTTAGAATACTCTGACTTTGTCAGCT
TTTCTCCATCAGCTCTTTCT

In100 candidate2327 (in intron):

LOCUS ENSG00000182621

DESCRIPTION 1-phosphatidylinositol-4,5-bisphosphate phosphodiesterase beta

(EC 3.1.4.11) (Phosphoinositide phospholipase C) (Phospholipase C- beta-

1)(PLCbeta-

1) (PLC-I) (PLC-154). [Source:Uniprot/SWISSPROT;Acc:Q9NQ66]

ATCATATTTCACTGTGATTAAAATGTGAATGTTCTGGACTAACAAAGTTGAGCATCTTTTCGGATGTTTATTCAGGTTTT

CACTTCTATGAAGTATCTTT

In100 candidate2328 (in intron):

LOCUS ENSG00000182621

DESCRIPTION 1-phosphatidylinositol-4,5-bisphosphate phosphodiesterase beta

(EC 3.1.4.11) (Phosphoinositide phospholipase C) (Phospholipase C- beta-

1)(PLCbeta-

1) (PLC-I) (PLC-154). [Source:Uniprot/SWISSPROT;Acc:Q9NQ66]

TTAGCAATTTACTAGGTTGGTTATAACCCAAGAGATTTTTTTCTTTTTCTTTTAAGCTATGATTTTTCCTTATTCCAGTT

TTCCATTTGCTTGTTCTTTT

In100 candidate2329 (in intron):

LOCUS ENSG00000138041

DESCRIPTION SMEK homolog 2. [Source:Uniprot/SWISSPROT;Acc:Q5MIZ7]

GATATGTAATACTAAGCATAATTAAAACTTTGTAACTTTGCTGTCATCTTTTTGATTATCAGATTGGTGCCTTTTTATAC

CAGAATTATTTCTTGTTTTT

In100 candidate2330 (in intron):

LOCUS ENSG00000164270

DESCRIPTION 5-hydroxytryptamine 4 receptor (5-HT-4) (Serotonin receptor 4) 5-

HT4). [Source:Uniprot/SWISSPROT;Acc:Q13639]

TCATGTTTTCAAAAACCTAACTTCTGGGTTCATTGATCTTTTGAATGTTTTTTTGTGTCTTGATTTCCTTCAATTCAGTT

CTGATTTTGGTTATTTCTTG

In100 candidate2331 (in intron):

LOCUS ENSG00000164270

DESCRIPTION 5-hydroxytryptamine 4 receptor (5-HT-4) (Serotonin receptor 4) 5-HT4). [Source:Uniprot/SWISSPROT;Acc:Q13639]

AGACAAATATTAAAATTTTAAGGAAGCACCATCGGTTAACACTAGAACTCAATTTTTATTAATCCCTTTATTCAGTGATT

TTTTTTTCACCTACTTAATT

In100 candidate2332 (in intron):

LOCUS ENSG00000185046

DESCRIPTION cajalin 2 isoform a [Source:RefSeq_peptide;Acc:NP_690001]

TTGATTTTATCTGTTTAATATCCCTGTCGTTATTTTTTTTTTAACTTTCTATTCCCACTGCCTTTATTCTACTTCAGGT

CCTTGTTATCTTTGATCTAG

In100 candidate2333 (in intron):

LOCUS ENSG00000185046

DESCRIPTION cajalin 2 isoform a [Source:RefSeq_peptide;Acc:NP_690001]

TTTATCTAACACTTAGGTTTATTTTACAAATCTCTTCAGGTTTTCACTTTTCAGCCTTCATTTTTCATGTTTGGGGCTTT

TCTAAAATGTCCTGTGATCC

In100 candidate2334 (in intron):

LOCUS ENSG00000185046

DESCRIPTION cajalin 2 isoform a [Source:RefSeq_peptide;Acc:NP_690001]

GCTCCCTGGGGACATTTGTTAAGAATTAATAACTGAATCCAAGAGTGTTGAATCCTATTTTTCCTTCTCCATGTCAGCTT

CCCCTCCTTTACATTGTTGT

In100 candidate2335 (in intron):

LOCUS ENSG00000185046

DESCRIPTION cajalin 2 isoform a [Source:RefSeq_peptide;Acc:NP_690001]

CTGATTGGTCCTGGACTTTTTTTTTGGTAACTTTTAAATTAGCATTTCAATCTCACTGCTTGTTATTGGTCTCTTCAGAG

TTTCTACTTCTTGGTTTAAT

In100 candidate2336 (in intron):

LOCUS ENSG00000185046

DESCRIPTION cajalin 2 isoform a [Source:RefSeq_peptide;Acc:NP_690001]

TCATATTTTTAAATATTTGTTTAGATTTAGCCTCATATTAACCATTCTCTTTATTTATCATTACTTCTTGCATCTCAGTT

CCTTTTCTGAATTATATCCT

In100 candidate2337 (in intron):

LOCUS ENSG00000185046

DESCRIPTION cajalin 2 isoform a [Source:RefSeq_peptide;Acc:NP_690001]

TCATTGAAAAAACTTTCATAGTCTAACATTCTTTTTCTTTTCTCGTTATTGTCTTCATCTTCTTTCTCTTCTCTTCAGTC

CTTCTCCATGTCTTTTTCTT

In100 candidate2338 (in intron):

LOCUS ENSG00000185046

DESCRIPTION cajalin 2 isoform a [Source:RefSeq_peptide;Acc:NP_690001]

TGATTGTTTATTTGTAGTAATTCTTTATAAATTCTTCTGTAACCCCACAAGATATGGGTCCTTTCTCAGGTGTATGCACT

CTAAATATTTTCTCCTACTC

In100 candidate2339 (in intron):

LOCUS ENSG00000185046

DESCRIPTION cajalin 2 isoform a [Source:RefSeq_peptide;Acc:NP_690001]

GAATGATCTTCCTTTCCCTCTTAACTCCTTTTTATCCTTTAGCTCTAAGCGTACTTGTCACTTTTTTTAGCTCTCAGTTC

TTTTCTCAATCTCCAAGTAT

In100 candidate2340 (in intron):

LOCUS ENSG00000173200

DESCRIPTION Poly [ADP-ribose] polymerase 15 (EC 2.4.2.30) (PARP-15) (Baggrssive

lymphoma protein 3). [Source:Uniprot/SWISSPROT;Acc:Q460N3]

ATTTCTAACTGGAAGTCTGCTGTCATTCTTATCTTTACCCTTCTGTGTGTAATTCTTTTTTCTTTAATTGCTGTTCAGAT

TTTCTCCTTATCACTGGTTT

In100 candidate2341 (in intron):

LOCUS ENSG00000111647

DESCRIPTION

ATTTTTAATTTTAATTAGAGTGGTTAAGGATAACTCTTCGCTTTATTTTTCCCACTCATTATTTTATCCACTCAGTGTTT

TTCAGAGTGTCTTTTTGCTC

In100 candidate2342 (in intron):

LOCUS ENSG00000115380

DESCRIPTION EGF-containing fibulin-like extracellular matrix protein 1

precrsor (Fibulin-3) (FIBL-3) (Fibrillin-like protein) (Extracellular protein S-

5). [Source:Uniprot/SWISSPROT;Acc:Q12805]

CCTCTGCACTCCTGGACTCACTTGCTCAACATCATTTTTCTTTTACTAACATCTTACTGATTTTGCTGTGTTTCTCAGAT

ATACCTTGTTACTCTGCTTC

In100 candidate2343 (in intron):

LOCUS ENSG00000048740

DESCRIPTION CUG triplet repeat, RNA binding protein 2 isoform 1

[Source:Refeq_peptide;Acc:NP_001020247]

AGATTTGTTAACTGGAATTTCTGTACTTCCATCTCTTTTTGTTGACAGCTTCTCTTTGTTCAGGTTCTCCATTTCCTGCA

CATTTTTAACACATTTGCAA

In100 candidate2344 (in intron):

LOCUS ENSG00000048740

DESCRIPTION CUG triplet repeat, RNA binding protein 2 isoform 1

[Source:Refeq_peptide;Acc:NP_001020247]

GTTGGTTTAACAAGCGTCCAACCCTTTTGTCTTGTTGTTTTTTGTCTTTCCTGCTCCCTGTCCCCTTGTTTCCCACAGGC

TGTGCGTTTGTCACATTTTC

In100 candidate2345 (in intron):

LOCUS ENSG00000166153

DESCRIPTION DEP domain containing 4 [Source:RefSeq_peptide;Acc:NP_689530]

CCATTTCTCCCTAACTCTACCTTCTCTGTTCTAATTCCGATCTCTTATTCAGATATTGGACCTTCTTAACTAGTCCTCTA

ATTTGTATATATTTTTCTCT

In100 candidate2346 (in intron):

LOCUS ENSG00000173210

DESCRIPTION Actin-binding LIM protein 3 (Actin-binding LIM protein family

mmber 3) (abLIM-3). [Source:Uniprot/SWISSPROT;Acc:O94929]

ATTCTTTATGTAAGTTTATTGCTAGCTACCAACTAACTTTTTTAGCCTCTTTTATTTACAGATATACTACCAACTCCTA

AATTCTGATTTTTTTCATTT

In100 candidate2347 (in intron):

LOCUS ENSG00000055813

DESCRIPTION Coiled-coil domain-containing protein 85A.

[Source:Uniprot/SWISPROT;Acc:Q96PX6]

TGTTTCTTTCTGTACCTCCTCTTTAAAGCCAATAACTCTTAGGTTTGCCCTTTTGAAGCTATTTTCCAGATGTTGTAGGA

GTGCTTCATTTTTTTTCTTT

In100 candidate2348 (in intron):

LOCUS ENSG00000012504

DESCRIPTION Bile acid receptor (Farnesoid X-activated receptor) (Farnesol

rceptor HRR-1) (Retinoid X receptor-interacting protein 14)

(RXRinteractingprotein

14). [Source:Uniprot/SWISSPROT;Acc:Q96RI1]

AGTCCATGACTTTCTTAACCTTGACATCTCTAACTTTTCCTTCAGCCTCTGTTTTCATATTTCATGAGTTACCAAAACCT

GATGTTCTCTCTGTTTCCTC

In100 candidate2349 (in intron):

LOCUS ENSG00000119866

DESCRIPTION B-cell lymphoma/leukemia 11A (B-cell CLL/lymphoma 11A) (COUP-TF interacting protein 1) (Ecotropic viral integration site 9 protein homolog (EVI-9). [Source:Uniprot/SWISSPROT;Acc:Q9H165]

AGACTGATTAACTGTTTAAGACTTGTACATTTTCTGCTTCAGTTTTATTAACTGGCAGCATCCTGGATGTTTTGTATTTT

GTGATTTTTTTTTTTTTTT

In100 candidate2350 (in intron):

LOCUS ENSG00000145882

DESCRIPTION prenylcysteine oxidase 1 like

[Source:RefSeq_peptide;Acc:NP_07633]

CTCCCTTCCCATTCTATAACTCTGCTGTTTATAGCACTTCTTCCTTATATTCTCCAGATGTCACCCAAAGCTTATTTCCT

TACCTCTCTTTCTGATATTT

In100 candidate2351 (in intron):

LOCUS ENSG00000101333

DESCRIPTION 1-phosphatidylinositol-4,5-bisphosphate phosphodiesterase beta (EC 3.1.4.11) (Phosphoinositide phospholipase C) (Phospholipase C- beta-4)(PLCbeta-

4). [Source:Uniprot/SWISSPROT;Acc:Q15147]

GAGCTCTGCCTGACACTTGCCTTCTAACTTTTTCTGGAGTAATTCTCTCCCTCATTTTTCAGCTTTGCTTTTTTGTTTTA

AGTTTGTCTAATATGCGTCT

In100 candidate2352 (in intron):

LOCUS ENSG00000173175

DESCRIPTION Adenylate cyclase type 5 (EC 4.6.1.1) (Adenylate cyclase type V (ATP pyrophosphate-lyase 5) (Adenylyl cyclase 5). [Source:Uniprot/SWISSPRO;Acc:O95622]

TTGCTTGAACCTAGCATGCCTCCCAACAAAATTCCTCATCATCCCTAACTTGACTTTATTTTCGTTATTATCAGTTCTCT CTTTGCCTAGGTGCCTGTGT

In100 candidate2353 (in intron):

LOCUS ENSG00000155886

DESCRIPTION Sodium/potassium/calcium exchanger 2 precursor (Na(+)/K(+)/Ca(2)-exchange protein 2) (Retinal cone Na-Ca+K exchanger) (Solute carrier famly 24 member 2). [Source:Uniprot/SWISSPROT;Acc:Q9UI40]

TCTAATGGATTAACTGGGTACTCATCCTTTCCTACTGGTAATACATCTTGTTTGTTTTCCCCTTTCATCTAAATCCAGAA ATGTTCTTCTCTTGTATCTG

In100 candidate2354 (in intron):

LOCUS ENSG00000148429

DESCRIPTION USP6 N-terminal-like protein (Related to the N-terminus of tre)(RN- tre). [Source:Uniprot/SWISSPROT;Acc:Q92738]

AACTTAGTCCTACTAGCAAAACACAACTGAGTTAACTGTTGGGATTTTTGTCCATTCCTCACACTTTTTTCAGTATAATC CTTTCTTTTCCCTTTTTGTA

In100 candidate2355 (in intron):

LOCUS ENSG00000171843

DESCRIPTION Protein AF-9 (ALL1 fused gene from chromosome 9 protein)

(Myelod/lymphoid or mixed-lineage leukemia translocated to chromosome 3 protein) YEATS domain-containing protein 3). [Source:Uniprot/SWISSPROT;Acc:P42568]

TTCCAATAACTCAAGTATTTATTTTTCCTTCTTTGCTTCTTACCTTTCCTCTTAACTTGAAGCTTATTCTTAATGCAGAT

GTTACTTGGACCTTTTTTGT

In100 candidate2356 (in intron):

LOCUS ENSG00000171843

DESCRIPTION Protein AF-9 (ALL1 fused gene from chromosome 9 protein) (Myelod/lymphoid or mixed-lineage leukemia translocated to chromosome 3 protein) YEATS domain-containing protein 3). [Source:Uniprot/SWISSPROT;Acc:P42568]

GATTCTTATCACATTAACTCAATTGAGGTTGGAGATTTTGAGTTGTTCTTCCTATCTCCTGTCTCTTTCCTTTCTCAGGT

TCTTCTCTACAAAACTTTCT

In100 candidate2357 (in intron):

LOCUS ENSG00000171843

DESCRIPTION Protein AF-9 (ALL1 fused gene from chromosome 9 protein) (Myelod/lymphoid or mixed-lineage leukemia translocated to chromosome 3 protein) YEATS domain-containing protein 3). [Source:Uniprot/SWISSPROT;Acc:P42568]

CTTTTTAATATCATAATTTTATATTTATTCCTATTGATGATAATAGTAACTAATATTTAGTCATCATTTATTCAGTGTTC

TGTCCTAACTGCTTTGCGTT

In100 candidate2358 (in intron):

LOCUS ENSG00000101868

DESCRIPTION DNA polymerase alpha catalytic subunit (EC 2.7.7.7).

[Source:Unprot/SWISSPROT;Acc:P09884]

AGCCATTTATGCCACTTGACTTTTATCCTTCGTAGAATAACTAACATTTACCTGTATTTTCTACATTTATCAGAAGACCC

CTTTCTCTTTTATTTCATTT

In100 candidate2359 (in intron):

LOCUS ENSG00000101868

DESCRIPTION DNA polymerase alpha catalytic subunit (EC 2.7.7.7).

[Source:Unprot/SWISSPROT;Acc:P09884]

CCTTATTTTTCCTCAATTAGTAATTTTACTCCCATAACCTTCTCTTTGCTGCCATCCTTTTGTATTTCTCAGTTAATAGT

ATTTTCTCTAGTGCCCTTCC

In100 candidate2360 (in intron):

LOCUS ENSG00000188352

DESCRIPTION KIAA1797 (KIAA1797), mRNA [Source:RefSeq_dna;Acc:NM_017794]

TTCCCCCACCTACTCTTTAACACTGAGTATTATGAATCTTATTCTTCTGTGATTTGTGTATCTCTATTCTCTGCTCAGTT

TGCTATTTTATGATCTGCTT

In100 candidate2361 (in intron):

LOCUS ENSG00000188352

DESCRIPTION KIAA1797 (KIAA1797), mRNA [Source:RefSeq_dna;Acc:NM_017794]

AGCAATAACATTGCAAATAATTTGGATAATTTTTTTTTGTTAATTTTCAGGTCAAATACCTGACTTTCTCTTCCTTCCA

TTATTTGATTTCAGCATCTT

In100 candidate2362 (in intron):

LOCUS ENSG00000188352

DESCRIPTION KIAA1797 (KIAA1797), mRNA [Source:RefSeq_dna;Acc:NM_017794]

TATAGTTTTTTTATCCTCATGCTGCATTAACCCCTTTATCATTATATCCTGACCTTATCCTTTTTACAGACTTTGTCTTG

AAATGTTTTGTCATTGCTCC

In100 candidate2363 (in intron):

LOCUS ENSG00000188352

DESCRIPTION KIAA1797 (KIAA1797), mRNA [Source:RefSeq_dna;Acc:NM_017794]

GCTATTTAACCTTCTGCTGACTTTGCCTTATTCTGGTTCCCTCTTCTTTCAGCATCTGCTGATGTGGCCTCTGCTTCTCT

TTGTTCCTGTGCTTCTCCTT

In100 candidate2364 (in intron):

LOCUS ENSG00000113716

DESCRIPTION SMF protein (Fragment). [Source:Uniprot/SWISSPROT;Acc:Q12766]

GACTCTGTCTCAAAAAAAAAAAAAAAAAAAAAAGGAGGTGACATTTAACTATCCTTGTAGATTTGTCTGTTTCTTACAGTT

CTCTTAATTTTTGTGTCGCA

In100 candidate2365 (in intron):

LOCUS ENSG00000151461

DESCRIPTION Regulator of nonsense transcripts 2 (Nonsense mRNA reducing facor

2) (Up-frameshift suppressor 2 homolog) (hUpf2).

[Source:Uniprot/SWISSPRT;Acc:Q9HAU5]

CATGTGAAAGGTGATAAAATTAGGTAACAAGATATGTAATGCCCTTTCTTAGCTAGCTGGTTTTCAACTTCTTCAGTCTT

AATTCTAACCATTCTTCCTT

In100 candidate2366 (in intron):

LOCUS ENSG00000115464

DESCRIPTION Ubiquitin carboxyl-terminal hydrolase 34 (EC 3.1.2.15) (Ubiquitn thioesterase 34) (Ubiquitin-specific-processing protease 34) (Deubiquitinting enzyme 34). [Source:Uniprot/SWISSPROT;Acc:Q70CQ2]

AATTCTTTTGTTAGGTCCATATGTATTCCTGGTAACTTGATCCTTTTTTTCGTTTTAAATGTCCTTTGTCTCTTTCAGAC

ATTTTTTGGCTTAGTCTATT

In100 candidate2367 (in intron):

LOCUS ENSG00000173163

DESCRIPTION COMM domain-containing protein 1 (Protein Murr1). [Source:Uniprt/SWISSPROT;Acc:Q8N668]

AGTAACATTTTTTCTCTTTTCTTATTTTAGTAACTTTTATCTTCCTGACTTCCATAGCCAGCATCTTATTTTCTTCTTG

GTGTCCTTTTTCCCTTATCA

In100 candidate2368 (in intron):

LOCUS ENSG00000173163

DESCRIPTION COMM domain-containing protein 1 (Protein Murr1). [Source:Uniprt/SWISSPROT;Acc:Q8N668]

TATCTCCTGACATGAGCTATTTGTTAACATATTCACATAATCTCTCTTCTCTTTTTGAATTTCCATATACCACTACAGAG

TTTATTTTTTCACTAATCAC

In100 candidate2369 (in intron):

LOCUS ENSG00000173163

DESCRIPTION COMM domain-containing protein 1 (Protein Murr1).

[Source:Uniprt/SWISSPROT;Acc:Q8N668]

CTTATATGTGTTTGCTAATCTTAAATGGCCATCACTTCTAACATTCTTGGTTTTTAACATTCTCTGTGTTCTACTCAGGG

TAGCTTTATTTTCTGCTCTT

In100 candidate2370 (in intron):

LOCUS ENSG00000136048

DESCRIPTION damage-regulated autophagy modulator

[Source:RefSeq_peptide;AccNP_060840]

AGATAATTTGTGTCATATGGTCCTGTTTCATTAACCTTGTTAGTTTCCTCCTCTTTAATTGTTAACTGATTCTGCCAGCA

CCTCATTGTTCATTGACTTT

In100 candidate2371 (in intron):

LOCUS ENSG00000169306

DESCRIPTION X-linked interleukin-1 receptor accessory protein-like 1 precuror

(IL1RAPL-1) (Oligophrenin-4) (Three immunoglobulin domain-containing ILreceptor-

related 2) (TIGIRR-2). [Source:Uniprot/SWISSPROT;Acc:Q9NZN1]

TAGCTCCAATTGATGGCTAATTGGCCCTTCAAATTTAACATACTTAAAAGGAAATTTCTGTTTATCCTCTACTCTCAGAA

TTGATTCTTCTTCAACTTCT

In100 candidate2372 (in intron):

LOCUS ENSG00000169306

DESCRIPTION X-linked interleukin-1 receptor accessory protein-like 1 precuror (IL1RAPL-1) (Oligophrenin-4) (Three immunoglobulin domain-containing ILreceptor-related 2) (TIGIRR-2). [Source:Uniprot/SWISSPROT;Acc:Q9NZN1]

GAGAAAGAGAGAACGAGAACACACCCTAAAAGGTTTTCTTATTAACTATTCTCAAGTATTATTTCTTATCAGCTATCATC

AGCTATTTCTTCTTCAGCAT

In100 candidate2373 (in intron):

LOCUS ENSG00000169306

DESCRIPTION X-linked interleukin-1 receptor accessory protein-like 1 precuror (IL1RAPL-1) (Oligophrenin-4) (Three immunoglobulin domain-containing ILreceptor-related 2) (TIGIRR-2). [Source:Uniprot/SWISSPROT;Acc:Q9NZN1]

AGAATTCAAGAACTTATTTATCATATAACCGAAAGTTTGTACCCTTTGATATTTCACTGTCTTTTTTCAGCATGTTTTTA

ATATGTTTGTACCTTTTTTT

In100 candidate2374 (in intron):

LOCUS ENSG00000169306

DESCRIPTION X-linked interleukin-1 receptor accessory protein-like 1 precuror (IL1RAPL-1) (Oligophrenin-4) (Three immunoglobulin domain-containing ILreceptor-related 2) (TIGIRR-2). [Source:Uniprot/SWISSPROT;Acc:Q9NZN1]

CTTTCCTTTAATCTTTGAGGTTGTTTCTGTAGGTGGCTTATCTAACTTTACATTGAAATTTGCATTCATTTCAGGATAAT

ACTTCCCCTGGGTTTCTTCT

In100 candidate2375 (in intron):

LOCUS ENSG00000169306

DESCRIPTION X-linked interleukin-1 receptor accessory protein-like 1 precuror

(IL1RAPL-1) (Oligophrenin-4) (Three immunoglobulin domain-containing ILreceptor-related 2) (TIGIRR-2). [Source:Uniprot/SWISSPROT;Acc:Q9NZN1]

TTATAGTATCTTGTTTACACCCCATTAACACTGATAGCACTCTATGTTACTTTATTTTCAATTTTTTTCCTTCAGAGTCT

CAGTTATTTAACCATTTTCC

In100 candidate2376 (in intron):

LOCUS ENSG00000169306

DESCRIPTION X-linked interleukin-1 receptor accessory protein-like 1 precuror (IL1RAPL-1) (Oligophrenin-4) (Three immunoglobulin domain-containing ILreceptor-related 2) (TIGIRR-2). [Source:Uniprot/SWISSPROT;Acc:Q9NZN1]

ACTTTCATGCTATCATTTTATGGCTGAATGAAAATGCCATTTTTAACCTTGAAAAAAATCTTTTACTTGTTCTAGCAGCT

TTCTTAGTTATGTTTTCTTT

In100 candidate2377 (in intron):

LOCUS ENSG00000169306

DESCRIPTION X-linked interleukin-1 receptor accessory protein-like 1 precuror (IL1RAPL-1) (Oligophrenin-4) (Three immunoglobulin domain-containing ILreceptor-related 2) (TIGIRR-2). [Source:Uniprot/SWISSPROT;Acc:Q9NZN1]

TTTGTTTATTAAAATGCAAGAATGGCCTCATACCTGTGGTTTAACTGAGCACTTAATTCCTTTATTATTTTAGTGCAGAT

GTCTCCATTTTTTTCCTTTG

In100 candidate2378 (in intron):

LOCUS ENSG00000169306

DESCRIPTION X-linked interleukin-1 receptor accessory protein-like 1 precuror (IL1RAPL-1) (Oligophrenin-4) (Three immunoglobulin domain-containing ILreceptor-related 2) (TIGIRR-2). [Source:Uniprot/SWISSPROT;Acc:Q9NZN1]

TGTGTATACTCATGTACATAAACACACTACACATTCCTAGTTTAACTATTTGCTAACCCTGTCCATTGATTTTCACAGTA

TATTTTTTTCTCCTCTCAAT

In100 candidate2379 (in intron):

LOCUS ENSG00000120860

DESCRIPTION Coiled-coil domain-containing protein 53.

[Source:Uniprot/SWISSROT;Acc:Q9Y3C0]

CATCAAGTCCTGGGGGTTTTGTTTAGTGGGAGACTTAACTACTGATTTGATCTCTCTACTTATTATTGGTCTGTTCAGAT

TACCTATTTCTTTATGATTT

In100 candidate2380 (in intron):

LOCUS ENSG00000181192

DESCRIPTION dehydrogenase E1 and transketolase domain containing protein 1

Source:RefSeq_peptide;Acc:NP_061176]

CTAACGTAAAGGTATTCATTTTTCTCTTACAGTAATTCTTTCATTTCTGCAGTAATAGAAAGTTTTGCTTTTTGTTTTGT

TTTGTTTTGTTTTGTTTTCT

In100 candidate2381 (in intron):

LOCUS ENSG00000101349

DESCRIPTION Serine/threonine-protein kinase PAK 7 (EC 2.7.11.1) (p21-activaed kinase 7) (PAK-7) (PAK-5). [Source:Uniprot/SWISSPROT;Acc:Q9P286]

TCATACTGTAGATTGTCTCTTAACTTTGTCGATTGTTTCCTTTGCTGTTCAGAAGTTTTTTAGCTTGATGTAATCCCATT

TGTCTACTTTTGGTTTTTTT

In100 candidate2382 (in intron):

LOCUS ENSG00000017427

DESCRIPTION Insulin-like growth factor IA precursor (IGF-IA) (Somatomedin C (Mechano growth factor) (MGF). [Source:Uniprot/SWISSPROT;Acc:P01343]

GTATATGAAACTGTGACTCACTTGATATAACTTTAGAGTGTATGTTTTGACACCTGCTTATTTCTTACCAGTAGCCTTT

CTGGTTTTCCAAAATCTTTC

In100 candidate2383 (in intron):

LOCUS ENSG00000160145

DESCRIPTION Huntingtin-associated protein-interacting protein (Protein Duo) [Source:Uniprot/SWISSPROT;Acc:O60229]

AGTCCCATGTTGGATTAACAATTTGCAAATATTTTCTTCCATTCTACAGGTTGTCTCTTCACTCTGTTGATTGTTTCCCT

TGCTGTGCAGAAGCTTTTTA

In100 candidate2384 (in intron):

LOCUS ENSG00000160145

DESCRIPTION Huntingtin-associated protein-interacting protein (Protein Duo) [Source:Uniprot/SWISSPROT;Acc:O60229]

AGGAGAAGCTAGGAGTCCCGGCTCCAACTCAAGCTTAACCATCAACTGTATCACCTTGTGTTTAAGTCATTTTTCCAGTC

TGATCTCAGTTTCTTTTGAT

In100 candidate2385 (in intron):

LOCUS ENSG00000160145

DESCRIPTION Huntingtin-associated protein-interacting protein (Protein Duo)

[Source:Uniprot/SWISSPROT;Acc:O60229]

AACATTTTTAACTTCTGAGAGCTCTTTCTCACTCTACAACTTTTTCCCTTTATGTAACAGCTTGTTCTTTTTTCATGGAT

ACAATATCCTCTCTTATCTT

In100 candidate2386 (in intron):

LOCUS ENSG00000160145

DESCRIPTION Huntingtin-associated protein-interacting protein (Protein Duo)

[Source:Uniprot/SWISSPROT;Acc:O60229]

AGTTTTTATAATTAACATTGTATCCTGCTTTTTTTCCACTGAATGTTATCTAAGCATTTTCCTTCAGTTTTACAAATTTA

TAATCTCTCTTTTTTAATTT

In100 candidate2387 (in intron):

LOCUS ENSG00000143951

DESCRIPTION

AGTTAACATTACTGAGTACCCCTTGTTCTTGAAATTTTTTTTTTTTTTTGGTTCTTTCAGTGTTCTCTCTATTTCTTTTT

CAGGTCCGTGTTCCTTTCCC

In100 candidate2388 (in intron):

LOCUS ENSG00000143951

DESCRIPTION

TATGGTCATTTTAACTATGTTAATTCTTTTAATCCATGAGCATGGTATTTTCTTTTCACTTGTTTGTGTCCTCTTCAGTT

TCTTTCGTCAGTGTTTTACA

In100 candidate2389 (in intron):

LOCUS ENSG00000143951

DESCRIPTION

AGTGGAAAAATCTATTCATTATTTTATTCTAACTTGGTAATATCTAATTATGTTTTTTAGCTCTTCTCACAGACAGTTTT

ATCATCTTATCATTTTTGGC

In100 candidate2390 (in intron):

LOCUS ENSG00000143951

DESCRIPTION

GTTGTGTTTCTGATTGTTTTAAAATTTTTAGCTGTATCTCATTTTGCTTTAACATTTTTATTTTGAATCCTCTTTCAGGC

ATTTTACAGATTTCTTTTTC

In100 candidate2391 (in intron):

LOCUS ENSG00000143952

DESCRIPTION Vacuolar protein sorting-associated protein 54 (Tumor antigen SP-8p) (Hepatocellular carcinoma protein 8). [Source:Uniprot/SWISSPROT;Acc:QP1Q0]

ATGGAATTTTAATTGAAAGGCTATATTAGCTAACACAATAAATTGCTTTGCATTTGTCCCAGATATTTTATTTACATACT

GGCTAATTTTATTTATTTTT

In100 candidate2392 (in intron):

LOCUS ENSG00000143952

DESCRIPTION Vacuolar protein sorting-associated protein 54 (Tumor antigen SP-8p) (Hepatocellular carcinoma protein 8). [Source:Uniprot/SWISSPROT;Acc:QP1Q0]

ATGGTATAATATTAAAGTCTAGTTGCTGTATGACTAATGAATTTAACTTTCACTGAGATTTCTGCTATTCAGACAAGTTT
GTTTATTTCAGTTTGTCTCT

In100 candidate2393 (in intron):

LOCUS ENSG00000179088

DESCRIPTION Uncharacterized protein C12orf42.

[Source:Uniprot/SWISSPROT;AccQ96LP6]

TTTTCTCTCACTCTGTGGGTTTTCTCTTAACTCTGCTGATTATTTCTTTTGCTCTGCAGAAGCTTTTTAGTTTAAGCCCC
ATGTATTTATCTTTGTTTTT

In100 candidate2394 (in intron):

LOCUS ENSG00000179088

DESCRIPTION Uncharacterized protein C12orf42.

[Source:Uniprot/SWISSPROT;AccQ96LP6]

TATAACTGTGCTATTGTGTTGACATTCACTTCTTCATTTTATTTTGTCTGCACAGTCTTGATCTGCTAATTTCATTTTTC
CTGTTGCTAAACTGTATTTT

In100 candidate2395 (in intron):

LOCUS ENSG00000119844

DESCRIPTION Aftiphilin. [Source:Uniprot/SWISSPROT;Acc:Q6ULP2]

AATAAATGTTTCTCTCTCTTAACTTTAGCTTGAATTATCATATTGTCTTTTAAACTTTGTGTGCTTTTATTCCCCCAGAT
TTTTCTTATTGCCTTTAATA

In100 candidate2396 (in intron):

LOCUS ENSG00000179833

DESCRIPTION SERTA domain-containing protein 2 (Transcriptional regulator ineracting with the PHD-bromodomain 2) (TRIP-Br2). [Source:Uniprot/SWISSPROT;cc:Q14140]

AATAGTTGTCTAATAAAACCTAGAAGGCCACCTTTGGTTTTAACTAAGATGTAATTTTATCTTTCACAGAATTGTTCTAA
AACCTTATATTTCCCTGTTT

In100 candidate2397 (in intron):

LOCUS ENSG00000163848

DESCRIPTION Zinc finger protein 148 (Zinc finger DNA-binding protein 89) (Tanscription factor ZBP-89). [Source:Uniprot/SWISSPROT;Acc:Q9UQR1]

TTTAATGTTATTGATTCTTCTAGTTCATTAACATGGAACTTTTTTTCGTTTGTTTATGTCATCTGTGATTTCCTTCAGCA
GTGTTTTTTAGTTCTTCTTA

In100 candidate2398 (in intron):

LOCUS ENSG00000163848

DESCRIPTION Zinc finger protein 148 (Zinc finger DNA-binding protein 89) (Tanscription factor ZBP-89). [Source:Uniprot/SWISSPROT;Acc:Q9UQR1]

GCTTCTGGTTTTCATGTCTTCTAATTGTTCTCACTGTAACTTTAAATAATATTCTTATACCTCAGTTTCTTAGGTTACCA
ATTTGTTATCATTTTACTTC

In100 candidate2399 (in intron):

LOCUS ENSG00000111696

DESCRIPTION 5'-nucleotidase domain containing 3 isoform 2

[Source:RefSeq_petide;Acc:NP_057659]

CTTTTTCTGTTTTTTTTTTTTTTGTAACTTCTAAAATTCTCCGGGAAATTTTTCACCTCTTCCATTTCTTCTTCAGGGACT

CCTGATCTGTATATTCTTTT

In100 candidate2400 (in intron):

LOCUS ENSG00000198947

DESCRIPTION Dystrophin. [Source:Uniprot/SWISSPROT;Acc:P11532]

TGTATCTATTTTTAGCATTAATAAAATGGATGAAGCTTTAACTCTATAATAAATTCTTCCACTTTAAATTCTATACAGCT

TGTCTTTCCCTGATTCTCTA

In100 candidate2401 (in intron):

LOCUS ENSG00000198947

DESCRIPTION Dystrophin. [Source:Uniprot/SWISSPROT;Acc:P11532]

TTTCTAACTTAGCAATCTTGTTCTAGCTTCTTTTTCTTTCTGTGACTATGTGTGTTCAGTCCCTTGAATTCCCCTTTGAA

TCAGTTTTAATTTGTTGCCT

In100 candidate2402 (in intron):

LOCUS ENSG00000198947

DESCRIPTION Dystrophin. [Source:Uniprot/SWISSPROT;Acc:P11532]

TGATTCTTCTCTCTTTTCTTCCTTATTAGTCTAACTAGAAGTCTACCTTTTTTATTTTTATTTTTTTCCAAAAACCAGCT

CCTGGATTCATTTAGTTTTT

In100 candidate2403 (in intron):

LOCUS ENSG00000198947

DESCRIPTION Dystrophin. [Source:Uniprot/SWISSPROT;Acc:P11532]

TTAGCATAACATACTATGTTTTATTTTTCTTTCAGCGTTTATCGTGATTGTTAAATCTTTTATTTGCTTTTTAAAAATTT

TCCCCGTCTATTCCCATTCT

In100 candidate2404 (in intron):

LOCUS ENSG00000198947

DESCRIPTION Dystrophin. [Source:Uniprot/SWISSPROT;Acc:P11532]

CCCTCTCATCTGCTCATTCTCTGTACTCCAACTACACTAACTTTGTTTTTTTTTTTTTTTAGATTTCTCTAACTACAGTG

CTGTAATCTCTTTTTCCTTT

In100 candidate2405 (in intron):

LOCUS ENSG00000198947

DESCRIPTION Dystrophin. [Source:Uniprot/SWISSPROT;Acc:P11532]

ATTACTAACTGTCTGGATTTTTTCTTGTTTATTGGCTGTTTACATTTTTGTCAATTTCTTTTTATGTTCTTTATCCAGTT

TTCCAGTAGATTTTTCCTTT

In100 candidate2406 (in intron):

LOCUS ENSG00000198947

DESCRIPTION Dystrophin. [Source:Uniprot/SWISSPROT;Acc:P11532]

TATGTAACATGGTGGCACCCCTTTTCATATAATTCTCTCCTCACTTCTATTTTGGGTTTTTACATGTTCTTGTTTCAGTC

TCTTCTGCTCTTTCTATCTT

In100 candidate2407 (in intron):

LOCUS ENSG00000198947

DESCRIPTION Dystrophin. [Source:Uniprot/SWISSPROT;Acc:P11532]

ACCACAAGCTGACTTGGGGGAGGAATATTTCCTCATTTAGCTGTAGTTTTAACTTTTGTTTTCACTGCATATTTTCAGTC

TATTTTATTTTCTTTCCTCT

In100 candidate2408 (in intron):

LOCUS ENSG00000198947

DESCRIPTION Dystrophin. [Source:Uniprot/SWISSPROT;Acc:P11532]

TTATTTATGTGCCAATAACTCCCCTCTGTTTTTAGCTTTTTGATCTGTTACATCTCAGATACTTTATTCTCTATTTGTAA

AACAACCACATTCCTCCTTT

In100 candidate2409 (in intron):

LOCUS ENSG00000198947

DESCRIPTION Dystrophin. [Source:Uniprot/SWISSPROT;Acc:P11532]

CTTTCCTTTGATGTGAGAACTTTTAAATACTGATGCAATCTCCTTAACTCTTTCCTTAGCTGTTACTGGTCTTTTCAGAT

TTCCAATTTTCATTATTCAG

In100 candidate2410 (in intron):

LOCUS ENSG00000183049

DESCRIPTION Calcium/calmodulin-dependent protein kinase type 1D (EC 2.7.11.7)

(CaM kinase ID) (CaM kinase I delta) (CaMKI-delta) (CaM-KI delta) (CaMKIdelta)

(Camk1D) (CamKI-like protein kinase) (CKLiK).

[Source:Uniprot/SWISSPOT;Acc:Q8IU85]

TCGTTTGGAATTTCTTCGGGAAAAAAAATCTAGTTAACTCTCTTTGATTTTCTTTTATAGATCCGTTATGTTTCCCAGTT

TGATTCTGTTACCTTCCCAT

In100 candidate2411 (in intron):

LOCUS ENSG00000183049

DESCRIPTION Calcium/calmodulin-dependent protein kinase type 1D (EC 2.7.11.7) (CaM kinase ID) (CaM kinase I delta) (CaMKI-delta) (CaM-KI delta) (CaMKIdelta) (Camk1D) (CamKI-like protein kinase) (CKLiK). [Source:Uniprot/SWISSPOT;Acc:Q8IU85]

AGAAATAACCACTGTGAGATTTTTTATGTATTTTCCAGACCTTTTCTGTGCATAGTCAACACAATTGCTTTTGTATTGTG
TGTTGTTTCTTCCCTTTTCC

In100 candidate2412 (in intron):

LOCUS ENSG00000143995

DESCRIPTION Homeobox protein Meis1. [Source:Uniprot/SWISSPROT;Acc:O00470]

TAATCATGCTTAATGAACTGAACTAAGCATAATTCTTGTTCTGTTTTTGTAACTTTGTGGATTTCTTCTAATGCTCAGTT
TCATGTGACTACTCATTCTT

In100 candidate2413 (in intron):

LOCUS ENSG00000198431

DESCRIPTION Thioredoxin reductase 1, cytoplasmic precursor (EC 1.8.1.9) (TR (TR1). [Source:Uniprot/SWISSPROT;Acc:Q16881]

TCTTTTATCCATTTTGAGGGGAGTTATAATTTAGAATCCTCACTCTTAACCACTGTGTTGGAGTTCTTCCATTCTCAGAT
TTCTTAGAGTTTTCTTATTT

In100 candidate2414 (in intron):

LOCUS ENSG00000198431

DESCRIPTION Thioredoxin reductase 1, cytoplasmic precursor (EC 1.8.1.9) (TR (TR1). [Source:Uniprot/SWISSPROT;Acc:Q16881]

ATTAGGAAAATATCAAAAAGGAAATTTTAATTCATGAAGAGATAACTGCTATTAATATTTTTGTGTATTTCCTTCCAGTT

TCTTTTTCTCTAAATATTTT

In100 candidate2415 (in intron):

LOCUS ENSG00000114544

DESCRIPTION solute carrier family 41, member 3 isoform 4 [Source:RefSeq_pepide;Acc:NP_001008487]

TTAGAGTTTATTTTTGTACATGGTGTAACATAAGCATCCAACTTCATTCTTTTGCATGTGGATATTTCGTTTTCCCAGCT

CCATTTTTTTGAAGACTCTC

In100 candidate2416 (in intron):

LOCUS ENSG00000099810

DESCRIPTION S-methyl-5-thioadenosine phosphorylase (EC 2.4.2.28) (5'-methythioadenosine phosphorylase) (MTA phosphorylase) (MTAPase). [Source:UniprotSWISSPROT;Acc:Q13126]

TGACTTGCATTTCCCTGATAATTAACAATATTGAGCATTTTTTCATGTTTGTTGGCCATTTGTATATCTTGTTTTCAGAA

TTTTCTATTCATATCCTTAG

In100 candidate2417 (in intron):

LOCUS ENSG00000196740

DESCRIPTION Uncharacterized protein C20orf61 (Fragment). [Source:Uniprot/SPREMBL;Acc:Q9NU29]

TTCTTAATGTTTTAAAGAAGTTAACTTCTGTTTGTTTTCATCATTCTGGTCTCAGTTCAGACATCATTTGTTCAGAGTGG

CCTGTCTTCTCTTTCCTTTC

In100 candidate2418 (in intron):

LOCUS ENSG00000143971

DESCRIPTION Ewing's tumor-associated antigen 1 (Ewing's tumour-associated

atigen 16). [Source:Uniprot/SWISSPROT;Acc:Q9NY74]

CTGTGACCAACAAAGCTGTGGATTATCTCTCTTTAACCTGCGTCTCCTCTTTTTTCCTTACCTACTAAGCTTCAGCTATA

CCAGCTATCTTTTAGTTTTT

In100 candidate2419 (in exon):

LOCUS ENSG00000143971

DESCRIPTION Ewing's tumor-associated antigen 1 (Ewing's tumour-associated

atigen 16). [Source:Uniprot/SWISSPROT;Acc:Q9NY74]

TTTCTAAGTTAGTAGAGACTGTTTTTGTAGTTTTACTTTCATTAACTGTTGCTTAAGGTTTTTATACACTTCCAGATTTT

AATTAATACTTTCATATTTC

In100 candidate2420 (in intron):

LOCUS ENSG00000144908

DESCRIPTION 10-formyltetrahydrofolate dehydrogenase (EC 1.5.1.6) (10-FTHFDH

(Aldehyde dehydrogenase 1 family member L1).

[Source:Uniprot/SWISSPROT;AccO75891]

CACACTTTTTTCTGCCTTTTCTGATCTTAACTGAGCATTTTATATGATTCCATTTTCTTTTCTCTCTTAGCATATCAGTT

TTACTTTTTTGTTGTTGCTT

In100 candidate2421 (in intron):

LOCUS ENSG00000115953

DESCRIPTION Calcineurin subunit B isoform 1 (Protein phosphatase 2B regulatry

subunit 1) (Protein phosphatase 3 regulatory subunit B alpha isoform

1).[Source:Uniprot/SWISSPROT;Acc:P63098]

TAACTCAAGCTTGATTGTTTAAAATTTATTCCTCATTTTTACCCTTCCAAGTAAGCCTTTTCTCCAGTAGCCCTCATCTT

TTCTTCCCTTTCTGAATTTT

In100 candidate2422 (in intron):

LOCUS ENSG00000171310

DESCRIPTION Carbohydrate sulfotransferase 11 (EC 2.8.2.5) (Chondroitin 4-

Osulfotransferase

1) (Chondroitin 4-sulfotransferase 1) (C4ST) (C4ST-1) (C4S1).

[Source:Uniprot/SWISSPROT;Acc:Q9NPF2]

AGTTAACATGAAGCCACTTCTTTCCTAAACCCTTTACGTTTTGTACCAGGTCCAGACCAGATCGTTTCTTTAAAGGCCCA

ATATTTTTCTCCTTTCTTTT

In100 candidate2423 (in intron):

LOCUS ENSG00000136052

DESCRIPTION solute carrier family 41, member 2

[Source:RefSeq_peptide;Acc:N_115524]

ATGGGATTCCAAGTTTGGATGGGCATTGCTGGCCTTCCTTTTAAGATGTAACGAAGGGAATCTTACTGTCATCTTCAGGG

TTTTCTGTTTGTTTGCCTTA

In100 candidate2424 (in intron):

LOCUS ENSG00000113140

DESCRIPTION SPARC precursor (Secreted protein acidic and rich in cysteine)

Osteonectin) (ON) (Basement-membrane protein 40) (BM-40).

[Source:Uniprot/SISSPROT;Acc:P09486]

AGGGTAACTTTTTGTTTTGTTTTGTTTTAGCTGCTATTTCACTTTGATCCTTCAACCTCAAACTATCTCAGACAAGTCCT

TCCTTGATGTTTTCTTTTCT

In100 candidate2425 (in intron):

LOCUS ENSG00000136051

DESCRIPTION KIAA1033 (KIAA1033), mRNA [Source:RefSeq_dna;Acc:NM_015275]

AGTTTTAACGTCTTATATTTAGTTTCTCTGGTTTCCTTGTAGCTTCCTCAATTGCCATCTTTTTAGCAGTTGAAGTGTTT

TTTTCCTTTCTTTTTGAGGC

In100 candidate2426 (in intron):

LOCUS ENSG00000089123

DESCRIPTION Threonine aspartase 1 (EC 3.4.25.-) (Taspase-1) [Contains:

Threnine aspartase subunit alpha; Threonine aspartase subunit beta].

[Source:Unprot/SWISSPROT;Acc:Q9H6P5]

AGACTGTAACCTTTTGGTATTTGCTTTTTTTTTTTTCCTTCCTCACTATAACTCTCTGGAGATAACATCCAGGTTGTGTT

TATCAATAGCTTGTTTTTTT

In100 candidate2427 (in intron):

LOCUS ENSG00000089123

DESCRIPTION Threonine aspartase 1 (EC 3.4.25.-) (Taspase-1) [Contains:

Threnine aspartase subunit alpha; Threonine aspartase subunit beta].

[Source:Unprot/SWISSPROT;Acc:Q9H6P5]

AGACATGCCCGTGGTCACTTATTTCTCGTATAACTTCATTTATCTTTCATTTGAATTTCACTTCCAGCATTATCACTTTT

GTTTTGTTTTGTTTTTGTTT

In100 candidate2428 (in intron):

LOCUS ENSG00000173046

DESCRIPTION

GAAATATTAAGTGTGACGTCTAATAATAACTTAGTTTGCTATTTTTATGTGCCAGCTTTGTGTTAAGTGTTTACATTGTC

TTTTTACACATTGTCTTTTT

In100 candidate2429 (in intron):

LOCUS ENSG00000197763

DESCRIPTION Thioredoxin reductase 3 (Thioredoxin reductase TR2)

(Fragment).[Source:Uniprot/SPTREMBL;Acc:Q86VQ6]

ACCATTATACACTGAAACATGAATAACTCATTTCATTCTTGCTGCCTAGGTATTCTTTTTTTAATTTTTATTTTTCAGTG

ATGACCTTTTTTCTCTGCCT

In100 candidate2430 (in intron):

LOCUS ENSG00000155511

DESCRIPTION Glutamate receptor 1 precursor (GluR-1) (GluR-A) (GluR-K1)

(Gluamate receptor ionotropic, AMPA 1) (AMPA-selective glutamate receptor 1).

[ource:Uniprot/SWISSPROT;Acc:P42261]

TTTATCTTGTCTATTGTACATTTATTTCATTAACTTCTACACTTTATTTACTTCTTCTTTTCTATGTTTAATTTGCAGGG

CTTTTTCCTGACTTCTTAAA

In100 candidate2431 (in intron):

LOCUS ENSG00000155511

DESCRIPTION Glutamate receptor 1 precursor (GluR-1) (GluR-A) (GluR-K1)

(Gluamate receptor ionotropic, AMPA 1) (AMPA-selective glutamate receptor 1).

[ource:Uniprot/SWISSPROT;Acc:P42261]

TTTGTAAAGCCTTGCATTTCTCTCTAAAGATAGACTTAACCACATCACTATTTTCTTGGCTTGTTTCAGATTTACTAAAT

TTTAAATTTTTTTGACTTTT

In100 candidate2432 (in intron):

LOCUS ENSG00000169604

DESCRIPTION Anthrax toxin receptor 1 precursor (Tumor endothelial marker 8)

[Source:Uniprot/SWISSPROT;Acc:Q9H6X2]

AGAAATTTAGCACCCCTATTTATAACTGTAATTGGGCTGCCATTGTCCTTTTTATTACTACCTTTCTTTACAGTACTTTT

GATTCTTTTTTATTTAAATT

In100 candidate2433 (in intron):

LOCUS ENSG00000159685

DESCRIPTION Coiled-coil-helix-coiled-coil-helix domain-containing protein 6

[Source:Uniprot/SWISSPROT;Acc:Q9BRQ6]

CTTTCTTAGAAATCTCCTTAGGGAAAATAGCAACCCGCTAACCCTACAAGTGAGTCTGTCTTATGGTCTTCCCAGCACAT

CTCTCTGTAATTATTTATCT

In100 candidate2434 (in intron):

LOCUS ENSG00000089048

DESCRIPTION ESF1 homolog (ABT1-associated protein).

[Source:Uniprot/SWISSPRT;Acc:Q9H501]

GTGGCTGATGCTTGTTAATTCTTTTGGCATTCCAAATAACTAGATGACCATTTTATTTTTATTGTTCACAGAACAGCTG

AGTCCTTTTTTCAATTTTCC

In100 candidate2435 (in intron):

LOCUS ENSG00000166046

DESCRIPTION t-complex 11 (mouse) like 2 [Source:RefSeq_peptide;Acc:NP_68998]

AGTTGCAAAGCTGGGTTTGGGCATGAGGCTGCCTAACTCTAAAAGGAAGTGTTTTTTCCTCAGTGCCTCACTGCCTTGTC

CTTTCTTTTTTTAATTTTTC

In100 candidate2436 (in intron):

LOCUS ENSG00000111783

DESCRIPTION regulatory factor X4 isoform c

[Source:RefSeq_peptide;Acc:NP_99759]

TCTGAGGAAAGAAAAGAATTGCCTCTAATCCTACTATGTAGAGATAACCATTCTTGATGTTTTTGTGTTTCCTTCCAGTC

TTTTTTCTTTCTAAGAATAC

In100 candidate2437 (in intron):

LOCUS ENSG00000111783

DESCRIPTION regulatory factor X4 isoform c

[Source:RefSeq_peptide;Acc:NP_99759]

AGAAGATAGTCTTGTCTTTGTCCTATATAGGTAACATGGGTTGTTTTTCTCTCTGGCTTTTTTCAGGATGTTTTTCGTTA

TCTTTGATTTTCTATAGTTT

In100 candidate2438 (in intron):

LOCUS ENSG00000111783

DESCRIPTION regulatory factor X4 isoform c

[Source:RefSeq_peptide;Acc:NP_99759]

GGAGATAAATAAAGTGGGATGTTCGCTGTAACTGCCTCTCTCCTCTTGTCTTTGGTGTCTCTCTGTCTCTCTCATCAGTC

TTTCTGTATCTCCTTTTCAT

In100 candidate2439 (in intron):

LOCUS ENSG00000115977

DESCRIPTION AP2-associated protein kinase 1 (EC 2.7.11.1) (Adaptor-associatd kinase 1). [Source:Uniprot/SWISSPROT;Acc:Q2M2I8]

GAAATTCTTCACCCTCTTCCCCCACATAACACTCTCTGTCAAACCTTCTTGCTTTATTTTTCTCCACAGCGTTTATCACA

ATCTAACGCACTTTTTTTTT

In100 candidate2440 (in exon):

LOCUS ENSG00000087338

DESCRIPTION Germ cell-less protein-like 1.

[Source:Uniprot/SWISSPROT;Acc:Q9IK5]

GTTGGTGTATTGAAAGCTTTCCTTTAACATTTTCACCTGCTCATTGTGATTCCTCCTTTTAGTCTAATATCTTTCCAGGT

CATACTTGTTTTTAATCATT

In100 candidate2441 (in intron):

LOCUS ENSG00000096872

DESCRIPTION Intraflagellar transport 74 homolog (Coiled-coil domain-containng

protein 2) (Capillary morphogenesis protein 1) (CMG-1).

[Source:Uniprot/WISSPROT;Acc:Q96LB3]

AAATAGAACGGCTCACTATTGTATCTTCTCATTTAACTGCTTCCTTTCAATTCCCTTACTTGTTACTATTTATTTCAGCT

ATTAACTTTTTTATCACTCT

In100 candidate2442 (in intron):

LOCUS ENSG00000101251

DESCRIPTION

TATTTGGTGAGTTGTAGAAGTTTTAAAATATATTCTGGATATTAACTTCTTATCTAATTTGCAAATATTTTCTCCCAGTC

TGTGGGTTGTCTTTTCACTC

In100 candidate2443 (in intron):

LOCUS ENSG00000187715

DESCRIPTION kelch domain containing 6

[Source:RefSeq_peptide;Acc:NP_997218]EXON

GGAATTTTAACATTGATTCCATATTTTTATTTATTCTACTCTTAGTCTTCTAATTTTTTCAGTTGACCTGGTAATGTTCT

TTTTGTAGCACCTTTTCTCT

In100 candidate2444 (in intron):

LOCUS ENSG00000172264

DESCRIPTION C20orf133 protein. [Source:Uniprot/SPTREMBL;Acc:Q495E0]

GCAATGGCCCTTTGAATTTTATTCTTCTGCTTGCTAACTGAAAAAATAATTTTACCTGTTTTTTTTGTCTTCCAGGTTTT

TCTAACAACTTAGTACTTCC

In100 candidate2445 (in intron):

LOCUS ENSG00000172264

DESCRIPTION C20orf133 protein. [Source:Uniprot/SPTREMBL;Acc:Q495E0]

TTAAGGTGAAACAATTTAACTTCTCCCATTCTTTTTCCTCTCACCTCTGTCTTTTTAATGATGTAGTTCGTGATTCAGAT

TAAACTTTATTTTCAATTTT

In100 candidate2446 (in intron):

LOCUS ENSG00000172264

DESCRIPTION C20orf133 protein. [Source:Uniprot/SPTREMBL;Acc:Q495E0]

CTGATGTCTTTGCATTATTTATCTGTGTTCTCTTGTATCTGAGTTCCTTTAACATCATTATTTTGAATTCCTTTTCAGGA

ATTTCATAGATTTTTTTTTC

In100 candidate2447 (in intron):

LOCUS ENSG00000172264

DESCRIPTION C20orf133 protein. [Source:Uniprot/SPTREMBL;Acc:Q495E0]

CAAAGTGAAATTAACTGGTAGTTTAATACTTCATTGCCTTATGCCTTTTTAGCTGACCATTTGACTGTTTCTCTCCAGTC

TTGCTTTTTCATCTACAAGT

In100 candidate2448 (in intron):

LOCUS ENSG00000172264

DESCRIPTION C20orf133 protein. [Source:Uniprot/SPTREMBL;Acc:Q495E0]

CTGATTTTCTCTATTAACTTTCTATTTTTAATTTCATTGATTTCTACTCTAATTTTTAGTGTCTCTTTTTTCCAGGTTAT

TTCAGATTTAATTTACTCTT

In100 candidate2449 (in intron):

LOCUS ENSG00000172264

DESCRIPTION C20orf133 protein. [Source:Uniprot/SPTREMBL;Acc:Q495E0]

AAATTAACTCTGCTGGGTTTTTTTCCTCTTTAAAAAGCCTGCTCTTCAGGTTTTTATTTGCAGACTGGTCTAGGCTTTTT

GTTTGTTTGTTTGTTTGTTT

In100 candidate2450 (in intron):

LOCUS ENSG00000184434

DESCRIPTION Leucine-rich repeat-containing protein 19 precursor.

[Source:Unprot/SWISSPROT;Acc:Q9H756]

GTAGATCATATGCTAGGTAAAAATGCACGTAATTTTGTGAAATAACAAAAGAAACTGATATTCCATCTTCTATTCCAGTT

TTCTATTTTATATTAGCTCC

In100 candidate2451 (in intron):

LOCUS ENSG00000120156

DESCRIPTION Angiopoietin-1 receptor precursor (EC 2.7.10.1) (Tyrosine-proten

kinase receptor TIE-2) (hTIE2) (Tyrosine-protein kinase receptor TEK) (p10 TEK)

(Tunica interna endothelial cell kinase) (CD202b antigen).

[Source:Uiprot/SWISSPROT;Acc:Q02763]

GATCTTTTGTAAGGTTCCATTCCTGGTTCTTTGAATTGTTCACTGTGTATAACCATTCTGTTACTCTCTCTTGGTCAGTA

CTTTATACTTTCAACTTCAT

In100 candidate2452 (in intron):

LOCUS ENSG00000170624

DESCRIPTION Delta-sarcoglycan (SG-delta) (35 kDa dystrophin-associated

glycprotein) (35DAG). [Source:Uniprot/SWISSPROT;Acc:Q92629]

AGTGGTAACATCTCCCTTGTCGTTTCTGATTGTGTTTATTTGAATCTTCTCTTTTCTTCTTTGTCTGCCTAGCAGTCTAC

CTATGTTATTAATTCTTTCA

In100 candidate2453 (in intron):

LOCUS ENSG00000116005

DESCRIPTION Prenylcysteine oxidase precursor (EC 1.8.3.5) (PCL1).

[Source:Uiprot/SWISSPROT;Acc:Q9UHG3]

AGTCTTATTTAGAATTCAACCCTCCTAACCCCATGTATTTCTAGTCTTCTTTTCAGACCATTTTCTCCTTTTTTTTTTCT

TTTGAGATGGAGTCTGTCTC

In100 candidate2454 (in intron):

LOCUS ENSG00000089177

DESCRIPTION Kinesin-like motor protein C20orf23 (Sorting nexin-23).

[SourceUniprot/SWISSPROT;Acc:Q96L93]

GAAAAAATATTTTTTGTATTTAACTTTTAAAGATACTTCTTATGTACAGATATGTAATTCAAGTTACCAATTATCTACTT

TTTCTTGACTTTTTATTTCT

In100 candidate2455 (in intron):

LOCUS ENSG00000120162

DESCRIPTION Mps one binder kinase activator-like 2B (Mob1 homolog 2b) (Protin

Mob3B). [Source:Uniprot/SWISSPROT;Acc:Q86TA1]

GAGTAACTTAAGGTGAATGTTGTGGATTTTTCTTTTCCTTTCTCTTCTTCCTCCTCTCCATTCTTTTAGGCACAGAGCAT

TCCTTTTAACATTGCCGTTT

In100 candidate2456 (in intron):

LOCUS ENSG00000120162

DESCRIPTION Mps one binder kinase activator-like 2B (Mob1 homolog 2b) (Protin

Mob3B). [Source:Uniprot/SWISSPROT;Acc:Q86TA1]

AAATATGAGAAGGAAAAATGCATCCTGGCATATTTGCATGGGAAGCCTTAACAAGAGGCCCCTTTTCATCTAGTCCAGCA

GTCTTTCTCCTTCATTTGAG

In100 candidate2457 (in intron):

LOCUS ENSG00000120071

DESCRIPTION Uncharacterized protein KIAA1267.

[Source:Uniprot/SWISSPROT;AccQ7Z3B3]

TTGCTTTTCATTTTCTGGGTCCTGTGCTTATAAACTTCTATATAGTAACTACTTGTTAAAATAATATTTTTTTTCCAGAA

ATTCTCTTAATTGTGCTCTT

In100 candidate2458 (in intron):

LOCUS ENSG00000120071

DESCRIPTION Uncharacterized protein KIAA1267.

[Source:Uniprot/SWISSPROT;AccQ7Z3B3]

TTAGATGCTTTTGTCACTGTGTTGTTCCTTTAGTCCTGGGGCCTTTAACTAGTTTGCCTTCCCTTTTCTATCTTTCAGAG

TTTTCCTTTGATTGCCTCTT

In100 candidate2459 (in intron):

LOCUS ENSG00000151474

DESCRIPTION FERM domain-containing protein 4A.

[Source:Uniprot/SWISSPROT;Ac:Q9P2Q2]

AACAACTATATATCTGTTGCCTTTTTCGTGTAACACTCATTTTACATTCTTTTGCTACTCTCTTCTCCTTCTTTGCAGAA

CTTTCACTTTCTTTGTAACT

In100 candidate2460 (in intron):

LOCUS ENSG00000176034

DESCRIPTION Uncharacterized protein CXorf59.

[Source:Uniprot/SWISSPROT;Acc:8N9S7]

TTTTGTGCTGTGAATAACTTTTCCCCACTGCTTCTATCTTCTTCTGTTCCTGACATTACTTTTTGGCTTCAGCTTACGTT

CTGGCTATGTTTATTGTCCT

In100 candidate2461 (in intron):

LOCUS ENSG00000174482

DESCRIPTION leucine rich repeat neuronal 6C

[Source:RefSeq_peptide;Acc:NP_69783]

CTAACATATGAACATGAAATATCTTTCTATCGTTTGGTGTCCTCTTCAATTTCTTTCATCAGTGTTTTATAGTTTTCATT

ATTGAGCTATTTCACTTTTT

In100 candidate2462 (in intron):

LOCUS ENSG00000174482

DESCRIPTION leucine rich repeat neuronal 6C

[Source:RefSeq_peptide;Acc:NP_69783]

ATTTTTATTTCTTCTAAAAGAAGTGTTTAAGTTTTTTTTTTTTTAATTTAACACTTTTGATACTGACACTTTTTCAGGA

CTCAATCTTTCTTCAATTAT

In100 candidate2463 (in intron):

LOCUS ENSG00000174482

DESCRIPTION leucine rich repeat neuronal 6C

[Source:RefSeq_peptide;Acc:NP_69783]

CTTTTACGTTACATAGATGGCTGATTTCCATAAACTTCATGAACTAACATCTACTAGCCTCTAATTTTTCTTATGCAGCT

TCTTCACGTCTTTCATCCTT

In100 candidate2464 (in intron):

LOCUS ENSG00000174482

DESCRIPTION leucine rich repeat neuronal 6C

[Source:RefSeq_peptide;Acc:NP_69783]

CATTGGAATAAACATTTTTTTTTCTCTAAATAACACAAAGGAACCCTAGTTTTTGTCAGACTTCAATTACTTAGTATTTA

CTTAGCACCTATTACTTTCT

In100 candidate2465 (in intron):

LOCUS ENSG00000130962

DESCRIPTION Transmembrane gamma-carboxyglutamic acid protein 1 precursor

(Poline- rich Gla protein 1) (Proline-rich gamma-carboxyglutamic acid protein1).

[Source:Uniprot/SWISSPROT;Acc:O14668]

CTTTCAAAGGTTGTTTGTGCCTAACCTTTCTTCTGTTTTTTCTGTTTGCATTCCTCATTTGCATTTCAGTTCTTCACCTT

TTAACTGAGGCTAGTTTTCT

In100 candidate2466 (in intron):

LOCUS ENSG00000130962

DESCRIPTION Transmembrane gamma-carboxyglutamic acid protein 1 precursor

(Poline- rich Gla protein 1) (Proline-rich gamma-carboxyglutamic acid protein1).

[Source:Uniprot/SWISSPROT;Acc:O14668]

CTACACTTACTACATCCTCTCTGATCTCAATAAGTAGTAACTCCATCCATCATGTTTGCTGATCTCCAGGCTTTTTGCCA

TCTCTGACTCCTCTCTTTTT

In100 candidate2467 (in intron):

LOCUS ENSG00000130962

DESCRIPTION Transmembrane gamma-carboxyglutamic acid protein 1 precursor

(Poline- rich Gla protein 1) (Proline-rich gamma-carboxyglutamic acid protein1).

[Source:Uniprot/SWISSPROT;Acc:O14668]

ACTCTTCTAACTTGGTCCTCTCACTATTTCTTTATCTGCCTGATTTGAGGATCTCTCCCCTCTCTGACTTTGTCTCAGAT

CATATATATTTTTTCTCTCC

In100 candidate2468 (in intron):

LOCUS ENSG00000073969

DESCRIPTION Vesicle-fusing ATPase (EC 3.6.4.6) (Vesicular-fusion protein NS)

(N- ethylmaleimide sensitive fusion protein) (NEM-sensitive fusion protei).

[Source:Uniprot/SWISSPROT;Acc:P46459]

CTTCTTTAAGCTGTCCATAATGGTGTAGTCGTGATTTCAAGCTAACTGTTCTTACTGCATTTTTCCCATGATTTTCAGAC

CTGCTTTTGGTCCTCTGTTA

In100 candidate2469 (in intron):

LOCUS ENSG00000065809

DESCRIPTION Protein FAM107B. [Source:Uniprot/SWISSPROT;Acc:Q9H098]

GTGGTAACAAAGATCTGTTTTCTCCCTCAAGTTTTTATTTGTTTAACAGACTATCGTAACCTTTCTGTGGCTGTTTTAAG

CTTCACAGTTTTTCCGCTTT

In100 candidate2470 (in intron):

LOCUS ENSG00000116035

DESCRIPTION Ventral anterior homeobox 2. [Source:Uniprot/SWISSPROT;Acc:Q9UI0]

AATGGAATCATATAGTGTATAACTTTTGGGAACTGTTTTTTCCCCTCAGTATAATTTCCTGTGTAAATTGTTGTGTGTTT

CAATCGTTCTTCCTTTTTTT

In100 candidate2471 (in intron):

LOCUS ENSG00000113282

DESCRIPTION Clathrin interactor 1 (Epsin-4) (Epsin-related protein) (EpsinR

(Enthoprotin) (Clathrin-interacting protein localized in the trans- Golgi egion)

(Clint). [Source:Uniprot/SWISSPROT;Acc:Q14677]

GTAAATGAACATGGAATGCAAAGGGATGGTGTTAATTAACTTGGAAGGTACATACTTACTATTTGCTTTCAGATGACGGT

TTTTATGTAGCTCTTTCTTC

In100 candidate2472 (in intron):

LOCUS ENSG00000108433

DESCRIPTION Golgi SNAP receptor complex member 2 (27 kDa Golgi SNARE protei) (Membrin). [Source:Uniprot/SWISSPROT;Acc:O14653]

ATTGTAGTCCCTTCTTTCAAAAGCTCCCAAATAACGTGGGGCTGTGTCTAGGCTGTTTCTTTCCTCTTGGGCCAGCTGTA

GGGTTTGCCTTTCCTTGTCT

In100 candidate2473 (in intron):

LOCUS ENSG00000164330

DESCRIPTION Transcription factor COE1 (OE-1) (O/E-1) (Early B-cell factor).[Source:Uniprot/SWISSPROT;Acc:Q9UH73]

AGGATGGATGAATTGCCTCGAGAGTTAAAGCAAAACCTCAAATCTCTCTAACCCAACCTTTTATTTTTTCTTCTTCAGTC

TTTTTCTCTTTCATGACTAT

In100 candidate2474 (in intron):

LOCUS ENSG00000164330

DESCRIPTION Transcription factor COE1 (OE-1) (O/E-1) (Early B-cell factor).[Source:Uniprot/SWISSPROT;Acc:Q9UH73]

TTGTCAACCTAAGAAGCCCATTGTGGGTGTAGTGGCAATTGTTAATAACTTTTGGAAGAATTCTTAACTTTTCAGCTACT

CTGTTTAGTTCCTTAACATC

In100 candidate2475 (in intron):

LOCUS ENSG00000112685

DESCRIPTION Exocyst complex component 2 (Exocyst complex component Sec5). [ource:Uniprot/SWISSPROT;Acc:Q96KP1]

AGTAACCTAACAAACATTTTGCCTCTTTAAATGCTTTCTGTGCAGTTGGTATTTCATCCTCTATTTCTTATTTTATTACT

GTTTTTGTACTTTCACTTTT

In100 candidate2476 (in intron):

LOCUS ENSG00000112685

DESCRIPTION Exocyst complex component 2 (Exocyst complex component Sec5). [ource:Uniprot/SWISSPROT;Acc:Q96KP1]

TAAATTTGCGTATAGTTCATTGCATTTTTGAATTTGAGTGCTTTATTTGTAACTGTTCCTACTTGCTTCTATGTTCAGTC

TCTCTGTTGATGTTTCTTCC

In100 candidate2477 (in intron):

LOCUS ENSG00000135083

DESCRIPTION cyclin J-like [Source:RefSeq_peptide;Acc:NP_078841]

TGTGTGTGTGCATGAATGTAGTTTAACTGGGATCGTACTCTAATCTTTTTTTGTTTTTATTCATCCCCTTAAAGACAGCT

TATTCTTTCTCTTGTTTTTG

In100 candidate2478 (in intron):

LOCUS ENSG00000112699

DESCRIPTION GDP-mannose 4,6 dehydratase (EC 4.2.1.47) (GDP-D-mannose dehydrtase) (GMD). [Source:Uniprot/SWISSPROT;Acc:O60547]

TTAATAGTTCCCATTGCCTACTGAGAACACTTTAACCCTGAACTGCTACTTCCCATGTTTCGATTTATTGCTTTCCAGTA
ATTTCATTCTCTCTTGTTTG

In100 candidate2479 (in intron):

LOCUS ENSG00000112699

DESCRIPTION GDP-mannose 4,6 dehydratase (EC 4.2.1.47) (GDP-D-mannose dehydrtase) (GMD). [Source:Uniprot/SWISSPROT;Acc:O60547]

TTTCCTTGCTACACTGTTTGTCTTTAACATTCATTTTTCTTTTTCTTCCCATTGTATTTTTAATCTCCTCAGTTGTGATT
GGTTTTTGTTTTATTTACTT

In100 candidate2480 (in intron):

LOCUS ENSG00000137074

DESCRIPTION Aprataxin (Forkhead-associated domain histidine triad-like protin) (FHA-HIT). [Source:Uniprot/SWISSPROT;Acc:Q7Z2E3]

TTAGATAACTTACTAAACTGTAGACCTTATTTGAATTTCACCCATTTTTCCTGTATTGTTCTTTTTCTACTCCAGAATCC
AATCTCACATTTCATTTATT

In100 candidate2481 (in intron):

LOCUS ENSG00000144036

DESCRIPTION Exocyst complex component Sec15B.

[Source:Uniprot/SWISSPROT;AccQ9Y2D4]

GTATTAACTTTTATAAATACATCTCCCCATTTTGTTGTCTATTTTTTCACCTAATTCATATTGCAAATTCTTATACAGAA

ACTTAACTTTTTTTTTTTG

In100 candidate2482 (in intron):

LOCUS ENSG00000144036

DESCRIPTION Exocyst complex component Sec15B.

[Source:Uniprot/SWISSPROT;AccQ9Y2D4]

TCTTTGTTGGTTTAAAGTCTGTTTTGTCTGAAACTAGGTTTGTAACCCCTGCTTTTTTGTTTTCTATTTGCTTGGCAGAT

TTTTCTCCATCCCTTTATTT

In100 candidate2483 (in intron):

LOCUS ENSG00000144036

DESCRIPTION Exocyst complex component Sec15B.

[Source:Uniprot/SWISSPROT;AccQ9Y2D4]

TTACATTTGTCAAAACTGAGAAATTAGTACTGGCACATCACTACTAACTAAAGTCTAGATTCATTTGTATTTCACCAGTT

TTTCTACTAATACTCTCTTT

In100 candidate2484 (in intron):

LOCUS ENSG00000144036

DESCRIPTION Exocyst complex component Sec15B.

[Source:Uniprot/SWISSPROT;AccQ9Y2D4]

AGGTCATGCTTTTGGTGTTGTATCTAAAAGTTGTAACTGAACCCAAGGTTACCTAGATTTTCTCTTGTTATTGTCCAGAA

GTTTTATATTTTTGTGTTTT

In100 candidate2485 (in intron):

LOCUS ENSG00000144036

DESCRIPTION Exocyst complex component Sec15B.

[Source:Uniprot/SWISSPROT;AccQ9Y2D4]

TGAGCACCTGGTGGCTCAATCAATTTGACACATAAAATTAACCATTACAAATGCTATACCCTTTCCTTTGTTTTGCAGTA

TTTTTTTCTTGTGCCCCATG

In100 candidate2486 (in intron):

LOCUS ENSG00000144036

DESCRIPTION Exocyst complex component Sec15B.

[Source:Uniprot/SWISSPROT;AccQ9Y2D4]

GATATCTTGACAATATTGTCTTCCTATCCATGAATATGAAATAACTCATTTATTTGTTATTCTTTGATATTTTATCAGAG

TTTCATAGTTTTTCTCATGT

In100 candidate2487 (in intron):

LOCUS ENSG00000172765

DESCRIPTION Transmembrane and coiled-coil domains protein 1.

[Source:Unipro/SWISSPROT;Acc:O94876]

AACAATTCTAAACAACTTTCCCTTATTGCTTGGTTTGCTAACCATTCATGTGGTCTTTGGGGCTTTTATTTTTACCAGTG

TTCTCATTTTTCACTGCTAC

In100 candidate2488 (in intron):

LOCUS ENSG00000172765

DESCRIPTION Transmembrane and coiled-coil domains protein 1.

[Source:Unipro/SWISSPROT;Acc:O94876]

AATTTTCTAATTAGAAGGTAACTATATTATGGGTATTTTGCTGCCAGTACTTCTCTCCCAATTATTTTTAAACTTTACC

CTCTTCACTCTATACTATGT

In100 candidate2489 (in intron):

LOCUS ENSG00000172765

DESCRIPTION Transmembrane and coiled-coil domains protein 1.

[Source:Unipro/SWISSPROT;Acc:O94876]

GGTATTAACTACTCATCTCTGCTACCATCTTTTTTCTTCTGTTTTATTGTATTAACCTCCTAGCTAGTCTCCCAGCATCC

GTTCTTGCTTAACACATTTT

In100 candidate2490 (in intron):

LOCUS ENSG00000172765

DESCRIPTION Transmembrane and coiled-coil domains protein 1.

[Source:Unipro/SWISSPROT;Acc:O94876]

TGGACTGTTCTGCATACTAGGCTGTTAACCTGAAATGCTTTTCCTTACCCTTTCTGTATCAGATACTAATTTCTTCAAGG

CTCATTTTTATTGTTACCTC

In100 candidate2491 (in intron):

LOCUS ENSG00000145864

DESCRIPTION Gamma-aminobutyric-acid receptor subunit beta-2 precursor (GABAA)

receptor subunit beta-2). [Source:Uniprot/SWISSPROT;Acc:P47870]

ATATTAGTCTTATTGATTTACTTAACTGTTTTTCATTTTATTGTATTTTACTCATATTTATTATTTCCTTCTTTCCAGTT

TGTGCTTAATTTGCATTTCT

In100 candidate2492 (in intron):

LOCUS ENSG00000145864

DESCRIPTION Gamma-aminobutyric-acid receptor subunit beta-2 precursor (GABAA)

receptor subunit beta-2). [Source:Uniprot/SWISSPROT;Acc:P47870]

TTTCTGGAAAAAAAAAAACAATAACAACAATCGCCTCTTGATCTTCTTTCACCAACTATTTTCTTTCCCATTTATCAGTC

TCATTTTCTTGCTGCTTCTA

In100 candidate2493 (in intron):

LOCUS ENSG00000145864

DESCRIPTION Gamma-aminobutyric-acid receptor subunit beta-2 precursor (GABAA)

receptor subunit beta-2). [Source:Uniprot/SWISSPROT;Acc:P47870]

AGAATGATAGTATTCTTAACCTTGCAAAAATATTTAATTTCTATATATATTTTCTCTCTCTCTTACCAATCTTATCAGAT

AAATCTTTTCCTTTCACAGT

In100 candidate2494 (in intron):

LOCUS ENSG00000206384

DESCRIPTION

ATGATATGTTGATGAAACCCTTAAATACTTTAACTTATAATTTAGTTTGTTTCTCAAATACTGCTATTTCACTTCCAGAT

TTCAATTGTTTTGGCCCTTC

In100 candidate2495 (in intron):

LOCUS ENSG00000196455

DESCRIPTION Phosphoinositide 3-kinase regulatory subunit 4 (EC 2.7.11.1) (P3-kinase regulatory subunit 4) (PI3-kinase p150 subunit) (Phosphoinositide3-kinase adaptor protein). [Source:Uniprot/SWISSPROT;Acc:Q99570]

AGGAGTTAACTGAATTTTTAGATCTTAGTTTGACCTTTTCAGTTTTCAGTTTTTCTTTTTTTTTTTTTATTTTTTGAGAC

GGACGGAGTCTCGCTCTGTC

In100 candidate2496 (in intron):

LOCUS ENSG00000022355

DESCRIPTION Gamma-aminobutyric-acid receptor subunit alpha-1 precursor (GAB(A) receptor subunit alpha-1). [Source:Uniprot/SWISSPROT;Acc:P14867]

AGATATACTGCACTTTCTGTTTCTTAAACATAACACATTTAACACATTTATTTTCACCTCAGATCCTTTGCCTCCTTCTT

TTTTTTAATTTTTTTCCTCT

In100 candidate2497 (in intron):

LOCUS ENSG00000113327

DESCRIPTION Gamma-aminobutyric-acid receptor subunit gamma-2 precursor (GAB(A) receptor subunit gamma-2). [Source:Uniprot/SWISSPROT;Acc:P18507]

GTTTCATGAAACATCTTCATAATTATTTCTGGCATAACCATGTAAATTATTTTCTGTTTTTGTTGTTTTTAATCCCAGTT

TTTTCTTAGATTCCATGTTC

In100 candidate2498 (in intron):

LOCUS ENSG00000113327

DESCRIPTION Gamma-aminobutyric-acid receptor subunit gamma-2 precursor (GAB(A) receptor subunit gamma-2). [Source:Uniprot/SWISSPROT;Acc:P18507]

ACTACCTCTAAAATAATAATGATAATGATAATAATGACAATATGTGCTAACATTGTGAAGCACTTTCTATACATCAGCTC

ATTCATTTTTCACAGCCATT

In100 candidate2499 (in intron):

LOCUS ENSG00000139437

DESCRIPTION trichoplein [Source:RefSeq_peptide;Acc:NP_115676]

ACTATGGGATTAAAGCGTCAACCGTCCTGGCATTTATTAACATGTTACCTTTTTCTGCATAATTCTCAGCTCGTCACTCA

AGTTGTTATTCACCTTCATT

In100 candidate2500 (in intron):

LOCUS ENSG00000116127

DESCRIPTION Alstrom syndrome protein 1. [Source:Uniprot/SWISSPROT;Acc:Q8TCU]

TTACATAACTTCTTTAAATTATTTCCTTTCTTGGGTTCTTCTGGTTTTTTTTCTACTCATCTGGCTGTTCTCTCTCAGTA

TCCTTTGCTGCTTTATCTTC

In100 candidate2501 (in intron):

LOCUS ENSG00000116127

DESCRIPTION Alstrom syndrome protein 1. [Source:Uniprot/SWISSPROT;Acc:Q8TCU]

AGGATATATAACCAAGGTTTAGTTGGGTTTTCTTTTATTGTTTCCTTCATACTTTCCTGAATTTTCTACAATGATCAGGT

TGTTTTGTTTGTTTGTTTGT

In100 candidate2502 (in intron):

LOCUS ENSG00000116127

DESCRIPTION Alstrom syndrome protein 1. [Source:Uniprot/SWISSPROT;Acc:Q8TCU]

TTTTTAAGGAGAAAATAAAAATATATTTTTTAACATCATTTTAGTCTCTAGTTTTATATCAGTGTTTTATATTTTACCAT

CTAAATCATCTGTTATTTCT

In100 candidate2503 (in intron):

LOCUS ENSG00000139436

DESCRIPTION ARF GTPase-activating protein GIT2 (G protein-coupled receptor

inase- interactor 2) (GRK-interacting protein 2) (Cool-interacting tyrosine

phosphorylated protein 2) (CAT2) (CAT-2). [Source:Uniprot/SWISSPROT;Acc:Q1161]

CCACCTGGTTGAAAACTTTTTCTGTTATCTCTGTATTGGTGTGAATATAACGTTGCCATTTCTGTCATTTTTTTTCAGTC

CTTGTTCATTTTGTTTTTGT

In100 candidate2504 (in intron):

LOCUS ENSG00000141293

DESCRIPTION Src kinase-associated phosphoprotein 1 (Src family-associated

posphoprotein 1) (Src kinase-associated phosphoprotein of 55 kDa) (pp55) (SKP-

55). [Source:Uniprot/SWISSPROT;Acc:Q86WV1]

GGTCAAATTTATTTATTTACTTTTATCACTTCTAACTTAAAATGTCCTTTCTTCTCTAGAGTTTTGTTCAACATTCAGTT

CTTTTCTGGTTTTTCTATAA

In100 candidate2505 (in intron):

LOCUS ENSG00000141293

DESCRIPTION Src kinase-associated phosphoprotein 1 (Src family-associated

posphoprotein 1) (Src kinase-associated phosphoprotein of 55 kDa) (pp55) (SKP-

55). [Source:Uniprot/SWISSPROT;Acc:Q86WV1]

AAAAACTTTAGCTTATGAAAAAGGAAAACACCATACTAAGAGTAAATTAACCACTTAAGTAACACTTTTAGTCTTCAGAC

TTTTCTCAGCATTCTTCTTT

In100 candidate2506 (in intron):

LOCUS ENSG00000196353

DESCRIPTION Copine-4 (Copine IV) (Copine-8).

[Source:Uniprot/SWISSPROT;Acc:96A23]

TTTCTCCCACTGTGGGTTGCTTGTAACTCTGCTGGTTATTTCTTTTACTGTGCAGAAGCTTTTTGGTTTAATTAAGTCCC

ATCCATTTATCTTTATTTTT

In100 candidate2507 (in intron):

LOCUS ENSG00000196353

DESCRIPTION Copine-4 (Copine IV) (Copine-8).

[Source:Uniprot/SWISSPROT;Acc:96A23]

AGGTGCCCTGTCCTGAAGATACTCCTCTTACTGAATTCTAGTAACTGCTCCCTCCCACTCTTTCTGGTCAGTTTCCATTA

ACCTTTTCCACATGTTTGTG

In100 candidate2508 (in intron):

LOCUS ENSG00000145934

DESCRIPTION Teneurin-2 (Ten-2) (Tenascin-M2) (Ten-m2) (Protein Odd

Oz/tenmhomolog

2). [Source:Uniprot/SWISSPROT;Acc:Q9NT68]

TCCTTGGGAGTAAATGTTGCAATGGGTTTAATTTCTAATGAAATAACTTAGCTTGGAATTCTAGTCCTGGTTTCTCAGCT

TCCTTAAACTACTTTTTACT

In100 candidate2509 (in intron):

LOCUS ENSG00000145934

DESCRIPTION Teneurin-2 (Ten-2) (Tenascin-M2) (Ten-m2) (Protein Odd

Oz/tenmhomolog

2). [Source:Uniprot/SWISSPROT;Acc:Q9NT68]

TGTCTGGTCCTGGACTTTTCTTTTTCGGTAACTTTTAAATACCATTTTAATCTCACTTCTTGTTATTGGTCTGTTCAGGG

TATCTAATTCTTCCTGATTT

In100 candidate2510 (in intron):

LOCUS ENSG00000145934

DESCRIPTION Teneurin-2 (Ten-2) (Tenascin-M2) (Ten-m2) (Protein Odd

Oz/tenmhomolog

2). [Source:Uniprot/SWISSPROT;Acc:Q9NT68]

AGAATTGCACCCACATCAATAAGAACTGAGTATTAGATCACATAACACTCTTTCTCCTGCTCTAATATGTCTCTCCAGGA

ATTCTACTGTTTCTCTCCTT

In100 candidate2511 (in intron):

LOCUS ENSG00000145934

DESCRIPTION Teneurin-2 (Ten-2) (Tenascin-M2) (Ten-m2) (Protein Odd

Oz/tenmhomolog

2). [Source:Uniprot/SWISSPROT;Acc:Q9NT68]

GTAAATGTTCACATCTACCTAAGGATATTGTTAATGGAAGCAAAACCTAACCCCCCTTATTTGATTTTGCCCTTCAGACT

TGAATTTTTCTCCTAACGCT

In100 candidate2512 (in intron):

LOCUS ENSG00000145934

DESCRIPTION Teneurin-2 (Ten-2) (Tenascin-M2) (Ten-m2) (Protein Odd

Oz/tenmhomolog

2). [Source:Uniprot/SWISSPROT;Acc:Q9NT68]

AGTTGATGTTGAAAACTATGGAATCTCCTTTTTAAAAAGGTAACTTTCATGATCCTCCTCTTAATGTTCTCAGTTTTTAT

ACTCTTGTTTAGTTTTTATT

In100 candidate2513 (in intron):

LOCUS ENSG00000145934

DESCRIPTION Teneurin-2 (Ten-2) (Tenascin-M2) (Ten-m2) (Protein Odd

Oz/tenmhomolog

2). [Source:Uniprot/SWISSPROT;Acc:Q9NT68]

TGTACGTGTCTGAGCTAATATTAACTAGAAATTTGTCTACTACACTTCTTTTTTTTTTTTTTTTTTTTTGAGACAGAG

TTTCTCTCTTATTTCCCAGG

In100 candidate2514 (in intron):

LOCUS ENSG00000145934

DESCRIPTION Teneurin-2 (Ten-2) (Tenascin-M2) (Ten-m2) (Protein Odd

Oz/tenmhomolog

2). [Source:Uniprot/SWISSPROT;Acc:Q9NT68]

CACAATAAAGAAGAGAAACACACTAATCTGCTACATGAGGATAACCATTTGTCAACATTTTAAATTATTTCCTTTCAGTC

TTTTATTTTCTATGTGTATG

In100 candidate2515 (in intron):

LOCUS ENSG00000014257

DESCRIPTION Prostatic acid phosphatase precursor (EC 3.1.3.2).

[Source:Unipot/SWISSPROT;Acc:P15309]

TTAACTATTCTGTAAAATTATGTCCACCCTTCTGTCATCTCTTTTTTTTTTAACTTCTCTTTTGAAAGCCATCAGTGTCT

AATTCAGTGGCCTTTCTTTC

In100 candidate2516 (in intron):

LOCUS ENSG00000076513

DESCRIPTION Ankyrin repeat domain-containing protein 13A (Protein KE03).

[Surce:Uniprot/SWISSPROT;Acc:Q8IZ07]

AAAAGTTAACATCGTAATGTATTTGGCTTTTATTTTGTACATCATCTCTAACTGTTGCCCAGGAGTTTGAGTGTTTTTTG

TTTGTTGTTTTGTTTTGTTT

In100 candidate2517 (in intron):

LOCUS ENSG00000076513

DESCRIPTION Ankyrin repeat domain-containing protein 13A (Protein KE03).

[Surce:Uniprot/SWISSPROT;Acc:Q8IZ07]

TTTCTCTGAAGGATCCTAATGATAGTTAACCATTTCTCATTTTTATTTTGCTGGATTGTTTTCTGTTTTTTGCTTCAGCA

TTCTTGCTTTTGCTGTGCTT

In100 candidate2518 (in intron):

LOCUS ENSG00000113645

DESCRIPTION WW domain-containing protein 1 (Kidney and brain protein) (KIBR)

(HBeAg-binding protein 3). [Source:Uniprot/SWISSPROT;Acc:Q8IX03]

TCTTTTGTATATGACACCCCCCACCTTATAACTATAGAAAGGTTTTGTTTGTTTTTTAATGCTTTCCAGCTTTGGGCTCA

GGAAATCCCTTCTTTTCTTC

In100 candidate2519 (in intron):

LOCUS ENSG00000122970

DESCRIPTION Intraflagellar transport 81 (Carnitine deficiency-associated

prtein expressed in ventricle 1) (CDV-1 protein).

[Source:Uniprot/SWISSPROT;Ac:Q8WYA0]

TTTTGATTGAAAAGTGGACATTGGAATCTCATAATGTAGTAACTCTGGAAATTAGGTTCTCTCTTTTCCCAGGGGTTTAC

TGTTTTTTGTTATAGTTTTT

In100 candidate2520 (in intron):

LOCUS ENSG00000122970

DESCRIPTION Intraflagellar transport 81 (Carnitine deficiency-associated

prtein expressed in ventricle 1) (CDV-1 protein).

[Source:Uniprot/SWISSPROT;Ac:Q8WYA0]

AGTCAAGTTGACACCTAATAGTAACCATCACATATATTTACTTTCATTTTTGCTTTTATAAATCAGGGCTTTTCTTTCCT

TATGTCTTCCGACTGATTTT

In100 candidate2521 (in intron):

LOCUS ENSG00000122970

DESCRIPTION Intraflagellar transport 81 (Carnitine deficiency-associated

prtein expressed in ventricle 1) (CDV-1 protein).

[Source:Uniprot/SWISSPROT;Ac:Q8WYA0]

AGTTACTGAGAGAAGTGTGTTAAAAATCTCTAACTATAATTATGGCTTTGTTGCTTTTTTTAGTTCCATCAGTTTTTGCT

TCATATTTTTTTCTTAAGTT

In100 candidate2522 (in intron):

LOCUS ENSG00000113971

DESCRIPTION Acyl-CoA dehydrogenase family member 11 (ACAD-11).

[Source:Unipot/SWISSPROT;Acc:Q709F0]

TAATATAAGAATATGTATACTCTTAACCCTTTCTACACTCTTTATCCCTCCCTTTCTTATTGTAACTTGTATCAGCTACA

GCTTATACTTTCACATTTTC

In100 candidate2523 (in intron):

LOCUS ENSG00000163170

DESCRIPTION BolA-like protein 3. [Source:Uniprot/SWISSPROT;Acc:Q53S33]

AGGGAATTAATTGGCCCATGTAACAAAATTATCTGGTTTCTGGCATATCTAGATTCCTCTCTCTCTCTGAGCTTTCAGCT

TCTTTCCCTGTGTTGGTCTC

In100 candidate2524 (in intron):

LOCUS ENSG00000184347

DESCRIPTION Slit homolog 3 protein precursor (Slit-3) (Multiple epidermal

gowth factor-like domains 5). [Source:Uniprot/SWISSPROT;Acc:O75094]

TTTGGAGTCTAAGTGCAACACTTTGCATTTATCCTAATTAACTTTTGGCAAATCATTTCATTTCATCAGCTTTTCAGGAG

CATTTTCTATTTCGATTTCC

In100 candidate2525 (in intron):

LOCUS ENSG00000180182

DESCRIPTION CRSP complex subunit 2 (Cofactor required for Sp1 transcriptionl

activation subunit 2) (Transcriptional coactivator CRSP150) (Vitamin D3

rceptorinteracting

protein complex 150 kDa component) (DRIP150) (Thyroid homone

receptor-associated protein comple [Source:Uniprot/SWISSPROT;Acc:O6024]

TAAATTAAAACTAGTCCTGTGCCTTTAAATAAGTTAACGTTTCACTGTTTTTATATCTCCAATCTCAGTCTCTCCATAA

GTCTAGTATTGTATTTTCTT

In100 candidate2526 (in intron):

LOCUS ENSG00000188687

DESCRIPTION sodium bicarbonate transporter 4 isoform c

[Source:RefSeq_peptie;Acc:NP_597812]

AAACAAACCTATGACAATCTCCTTGAAGATATTAACTATCTTTGTTTTTGTCTTTGTTTTGTAATCTTTGTACAGGGGTC

ATGCTTATCTTTTATTATTC

In100 candidate2527 (in intron):

LOCUS ENSG00000086065

DESCRIPTION Charged multivesicular body protein 5 (Chromatin-modifying protin

5) (Vacuolar protein sorting 60) (Vps60) (hVps60) (SNF7 domain-containin protein

2). [Source:Uniprot/SWISSPROT;Acc:Q9NZZ3]

AGAAGAAAATAAAATTTACCTAGATTCTTACTACTAGAAATAACCACCTTTAAAATGCTAATTTCTTCCAGTTTCTTTCC

TCTGCTTCTGCATACGTGGT

In100 candidate2528 (in intron):

LOCUS ENSG00000086065

DESCRIPTION Charged multivesicular body protein 5 (Chromatin-modifying protin 5) (Vacuolar protein sorting 60) (Vps60) (hVps60) (SNF7 domain-containin protein 2). [Source:Uniprot/SWISSPROT;Acc:Q9NZZ3]

GTTTTATAGCTTTAACTTTTTATTTTGCTGTTGTGTGTGTAGGTCTGTTTTTCTTCTGCTTTTTCAGTTGCTGCATAT
CACTTTGTCAATGTTCTTTT

In100 candidate2529 (in intron):

LOCUS ENSG00000134516

DESCRIPTION Dedicator of cytokinesis protein 2. [Source:Uniprot/SWISSPROT;Ac:Q92608]

GACGGTATAATGGCCTCTTATATTAACAAGGAGATTTGCATTCATTCATTGTCAGAGTCTGTACCACCTTTTAAAAATGT
ATCACCTTTTTTTTACTTTT

In100 candidate2530 (in intron):

LOCUS ENSG00000144868

DESCRIPTION Transmembrane protein 108 precursor. [Source:Uniprot/SWISSPROT;cc:Q6UXF1]

ATAGATCCCCTCATCTAGCCTTAATCTCTTCCACCTACTAACTTCCATAACACTTTATCTTGTTTTTTTTTAATTCAGGG
TATTTATTACTTCCTCTTTT

In100 candidate2531 (in intron):

LOCUS ENSG00000196850

DESCRIPTION T-cell activation protein phosphatase 2C [Source:RefSeq_peptideAcc:NP_644812]

ACAATCTGATCTCTCTTGCTTTTCCCCACAAATACTAATTTATAACCATCTTTGTTGTTGTTGTTGTTGTTCCAGGATCT
TGTGCTTGGCCATCATCTTT

In100 candidate2532 (in intron):

LOCUS ENSG00000163781

DESCRIPTION DNA topoisomerase 2-binding protein 1 (DNA topoisomerase IIbining protein 1) (DNA topoisomerase IIbeta-binding protein 1) (TopBP1). [Soure:Uniprot/SWISSPROT;Acc:Q92547]

ATAGGTCACTTAACCTGTCCTTATCTTCCATCTTTTTGTCTTTCTGTGATTTATTATGTGTAATTCCTTCAGATTTTTTT
CCAGATCACGAATCATCTCT

In100 candidate2533 (in intron):

LOCUS ENSG00000136436

DESCRIPTION calcium binding and coiled-coil domain 2 [Source:RefSeq_peptideAcc:NP_005822]

GCTTATGAGCTAACTTAGATTGGTTGTTTTTCTTTTCTTTTCTTTTCTTTTCTTTTCTTTTCTTTTTTTTTGAGACAGAG
TTTTTTGCTCTTGTTGCCCA

In100 candidate2534 (in intron):

LOCUS ENSG00000136436

DESCRIPTION calcium binding and coiled-coil domain 2 [Source:RefSeq_peptideAcc:NP_005822]

ACTCATAACAATAAGTGGTTTCTTCTTTTCTAAAACTTCTTTAACTCAGCTGGCATTCAGTTATTCTTTTAACTTTCCAA
TTGCTTAATTTTTGTCATCT

In100 candidate2535 (in intron):

LOCUS ENSG00000204852

DESCRIPTION TECT1_HUMAN Isoform 6 of Q2MV58 - Homo sapiens (Human) [Source:niprot/Varsplic;Acc:Q2MV58-6]

TAAGAAATCATGGCATATTAACAAAACCTTCCCCTTCACCTCACACATTTTTTTGCAGAGTGCATTATACCTTTTACGG

GGTACTATTTTTTTTTTTT

In100 candidate2536 (in intron):

LOCUS ENSG00000148481

DESCRIPTION CDNA FLJ13397 fis, clone PLACE1001351 (Novel protein) (Chromosoe 10 open reading frame 97) (Hypothetical protein DKFZp547B174) (DERP5) (Demal papilla derived protein 5). [Source:Uniprot/SPTREMBL;Acc:Q9H8M7]

GACAAATGAACTGTATTTATATTACAATTTTTATTCCGTGTAACAATCTATTTTACCTGTTCTTTCCTTTATTCCCAGCT

TTGTTCTTTTTCTTGTGTTT

In100 candidate2537 (in intron):

LOCUS ENSG00000204764

DESCRIPTION Ran-binding protein 17. [Source:Uniprot/SWISSPROT;Acc:Q9H2T7]

CCCAAAGGCCCTCTCTCTTAAAACCATCACATTGGAAATTAGATTTTAACATACGAATTTCCCGCATTTTTTCTGCAGCT

CTGTTTGTTTTTTATTCTTT

In100 candidate2538 (in intron):

LOCUS ENSG00000204764

DESCRIPTION Ran-binding protein 17. [Source:Uniprot/SWISSPROT;Acc:Q9H2T7]

TGTATCGTTTGAGTGGTTGTAGGAAATCTTATCCCTAGAGTAACTCTTCATTTTCTTTTTCTTCATGGTTTCTCTCAGTT

TTTGTCTAGATCTTGACCTT

In100 candidate2539 (in intron):

LOCUS ENSG00000204764

DESCRIPTION Ran-binding protein 17. [Source:Uniprot/SWISSPROT;Acc:Q9H2T7]

AGCCAAAAGACAAATATTTGTACTTAAGGTGGCATTTTTCACTTCACATAACACTTGCACTGATTTTTTACCTCACAGCT

TACTTTCTGCCTATCCTTTG

In100 candidate2540 (in intron):

LOCUS ENSG00000204842

DESCRIPTION Ataxin-2 (Spinocerebellar ataxia type 2 protein)

(Trinucleotiderepeat-containing gene 13 protein).

[Source:Uniprot/SWISSPROT;Acc:Q99700]

CTGTGGCTTAGGTCCGTTGTCCTAACCTGTGGCTTAGGTTCTGTTTTTGTTGCTTGTACTTGCTCCCCCTTTTTTCAGCC

ATTCCTGTTTTCTTTCTTTT

In100 candidate2541 (in intron):

LOCUS ENSG00000204842

DESCRIPTION Ataxin-2 (Spinocerebellar ataxia type 2 protein)

(Trinucleotiderepeat-containing gene 13 protein).

[Source:Uniprot/SWISSPROT;Acc:Q99700]

AGCCAATTTTCTCCATGTTTGACCTAATTGTGATTTCATAGATGTTAACTAAAACTCTTAATTTTCGTTTTCTCAGTATG

CTATTTTTTTTTTTTTTAGC

In100 candidate2542 (in intron):

LOCUS ENSG00000204842

DESCRIPTION Ataxin-2 (Spinocerebellar ataxia type 2 protein)

(Trinucleotiderepeat-containing gene 13 protein).

[Source:Uniprot/SWISSPROT;Acc:Q99700]

AAAGATGAACCCATTTCTTTGCCTGACTTGCTTTATAACTTTTGGCAAATAGTTTCTACTTCTGTACCTGGTCTTCAGAT

CTCTTTCCTGCTTTAACTAA

In100 candidate2543 (in intron):

LOCUS ENSG00000086102

DESCRIPTION Transcriptional repressor NF-X1 (EC 6.3.2.-) (Nuclear

transcripion factor, X box-binding, 1). [Source:Uniprot/SWISSPROT;Acc:Q12986]

TGCTAGCCCATTTCTGCATGTTTTTTTTCCTTTCCTGCCTTTTAATGACTAACTTCCCTCTAGTTGTTGATTTTTCAGAT

TTTGTTTCTTTACTGTCTGT

In100 candidate2544 (in intron):

LOCUS ENSG00000165983

DESCRIPTION Phosphotriesterase-related protein (Parathion

hydrolaserelatedprotein)

(HPHRP). [Source:Uniprot/SWISSPROT;Acc:Q96BW5]

ACACTAACTCATTTGTATTTCTATAATTACCGTGATTTTTTGTCTTCTCGTCTGTAGTGGCTCCCCCTTTTCTGGCAGCA

TCTCTGTAACTCCTGTTCTT

In100 candidate2545 (in intron):

LOCUS ENSG00000198740

DESCRIPTION zinc finger protein 652 [Source:RefSeq_peptide;Acc:NP_055712]

ATAACTTTCAAAACTGAGTTGGCTTTTTGCTTGTCTCCTGTCAGCTCCAGGTTCTCTAGCCACATCCACAGTTCTTTTCT

TACCATACTTCTTTTTTTTT

In100 candidate2546 (in intron):

LOCUS ENSG00000147044

DESCRIPTION Peripheral plasma membrane protein CASK (EC 2.7.11.1) (hCASK)

(alcium/calmodulin-dependent serine protein kinase) (Lin-2 homolog).

[SourceUniprot/SWISSPROT;Acc:O14936]

AAATTAGTAATCTAACTATATATTATCAATCTTTTGCTGTCTTATCTTACAGGTTTTCTTTCTGGTTTTCATACTGGATA

GTCTCTATTGACCTAGCTTC

In100 candidate2547 (in intron):

LOCUS ENSG00000147044

DESCRIPTION Peripheral plasma membrane protein CASK (EC 2.7.11.1) (hCASK)

(alcium/calmodulin-dependent serine protein kinase) (Lin-2 homolog).

[SourceUniprot/SWISSPROT;Acc:O14936]

AGTAAATCAACTAATAACTTAGTTACAATGTATTCACTTTCAGTGTTGTCTTCCTGGAATTTCCTCTTTTCTTATGTTCT

TTTTCACATTTTTTTACAGT

In100 candidate2548 (in intron):

LOCUS ENSG00000147044

DESCRIPTION Peripheral plasma membrane protein CASK (EC 2.7.11.1) (hCASK)

(alcium/calmodulin-dependent serine protein kinase) (Lin-2 homolog).

[SourceUniprot/SWISSPROT;Acc:O14936]

CTCTTTCAAGGTGTTAATGTAATGTGATAATGGATTTCCCTGAATTCATAACACATTTATTTATTCCTTTACTCTCAGCT

GTTAATTTTTTCCTCACTTT

In100 candidate2549 (in intron):

LOCUS ENSG00000148484

DESCRIPTION Ras suppressor protein 1 (Rsu-1) (RSP-1).

[Source:Uniprot/SWISSROT;Acc:Q15404]

AGATAACTGTAAAATATCTGCCATATCCGAGTGTGCTTCTTTTGCTTGATTTCTCTCCTCAGCCTTTCTTCTTTTTAGAG

TACCTTGTAATTTTGTTGTA

In100 candidate2550 (in intron):

LOCUS ENSG00000148484

DESCRIPTION Ras suppressor protein 1 (Rsu-1) (RSP-1).

[Source:Uniprot/SWISSROT;Acc:Q15404]

AGTAACAAACTCATTGACCTTGCGTTTTGCTGTTTTCAGTCTGAACCCTCTTTTGTCTAATTTTTTGCTGGTTCTTTAGC

TTTTTTCCCCTGCCTATCTA

In100 candidate2551 (in intron):

LOCUS ENSG00000148484

DESCRIPTION Ras suppressor protein 1 (Rsu-1) (RSP-1).

[Source:Uniprot/SWISSROT;Acc:Q15404]

AGAAAAGGGTAGCATTTATGATCTGTCATCCAAAAATAACCACTTTAAATTCTGAGATTTTTTTCCAGTCTTTTCTCTGT

TCATATGCATATACACTTAT

In100 candidate2552 (in intron):

LOCUS ENSG00000125821

DESCRIPTION Probable D-tyrosyl-tRNA(Tyr) deacylase 1 (EC 3.1.-.-).

[Source:niprot/SWISSPROT;Acc:Q8TEA8]

AATGCTGGATGGAATATATTTCTTTAACTCTTTTGTATTTCATTTTATTCATTTTTATTTTTAAAAATTTTCCTACAGCT

TTCATAGGCTGACTTTCTTT

In100 candidate2553 (in intron):

LOCUS ENSG00000154928

DESCRIPTION EPHB1_HUMAN Isoform 3 of P54762 - Homo sapiens (Human)

[Source:niprot/Varsplic;Acc:P54762-3]

AGACCTCTCTCTCTGTGGGTTAATATTAACAAAAATGATTGTTTCTGCTGACCAGAAGTCCTGTTTTTCTGTTGTGCTAT

CTCCTCCACTGATTTCTGAG

In100 candidate2554 (in intron):

LOCUS ENSG00000168246

DESCRIPTION Ubiquitin domain-containing protein 2 (Dendritic cell-derived

uiquitin-like protein) (DC-UbP) (Ubiquitin-like protein SB72).

[Source:Uniprt/SWISSPROT;Acc:Q8WUN7]

ACCTGGAACAATTAAAAGCTAAGCAAAGAATCTTGTAACCCTAAATTATCTATTTTTTTCTGTCATTCTAGTTTCCAGTT

ACATGATTCTTTCCTTTTGG

In100 candidate2555 (in intron):

LOCUS ENSG00000198270

DESCRIPTION Transmembrane protein 116. [Source:Uniprot/SWISSPROT;Acc:Q8NCL8

TCTGTATTCTCTCTCAACATTTAACTTTTTCTACCCACTCTACATTTTGTTCTTCCTTTTATTAGTTTTCTCTCCCAGGT

TTCTTTTTTCTTAAACTCCT

In100 candidate2556 (in intron):

LOCUS ENSG00000185052

DESCRIPTION Sodium/potassium/calcium exchanger 3 precursor (Na(+)/K(+)/Ca(2)-exchange protein 3) (Solute carrier family 24 member 3). [Source:UniprotSWISSPROT;Acc:Q9HC58]

AGGCCTATCTTTATTCTAACTTTTAGAACATTCCCAATTATTCTTCTGCTTTCCAGCTTCAAAAATTTTGTTGTTCTTAT

CAGCTCTTCTATTCCTTTTG

In100 candidate2557 (in intron):

LOCUS ENSG00000118007

DESCRIPTION Cohesin subunit SA-1 (Stromal antigen 1) (SCC3 homolog 1). [Souce:Uniprot/SWISSPROT;Acc:Q8WVM7]

AGCCACCGCATCTGGCCAAAATCTAAAGTAATATTAATTCAAATTTTTAACTCTCTCTCTTTTTACTTATGTCCTCAGAT

TTTCTAATCCTTAGTTTGCC

In100 candidate2558 (in intron):

LOCUS ENSG00000118007

DESCRIPTION Cohesin subunit SA-1 (Stromal antigen 1) (SCC3 homolog 1).

[Souce:Uniprot/SWISSPROT;Acc:Q8WVM7]

CACCTCTGCTTTTTTGAATATCTGTGCCTTAACTGGAATACCTTTTTCTGTAGCTAGATTATTTTTCTCCTTCAGGACTC

AGCTTACATGTTATCTTCTT

In100 candidate2559 (in intron):

LOCUS ENSG00000164463

DESCRIPTION adult retina protein (LOC153222), mRNA

[Source:RefSeq_dna;Acc:N_153607]

CTTTGACTCTTAAATGAATCTTAACACAACTTTGCATACTACCTTTTTGTGATATCTCATTTCTGATCTCAGCTAGTTTT

GAGTACATGTTCTTCCTTCC

In100 candidate2560 (in intron):

LOCUS ENSG00000159374

DESCRIPTION CDNA FLJ26651 fis, clone MPE05072.

[Source:Uniprot/SPTREMBL;AccQ6ZP30]

ATCTTTCGAGTCCTTGGCATTGCTTTCTTCAAAGAAAGCCTCAAATAACTGAACATTGACACTGTTTCTCCTTTTCAGAT

ATTCCTTCCACATTTAATTT

In100 candidate2561 (in intron):

LOCUS ENSG00000159374

DESCRIPTION CDNA FLJ26651 fis, clone MPE05072.

[Source:Uniprot/SPTREMBL;AccQ6ZP30]

TTTAACTCCATCTTTGGTTTTTTATTCTTTCATATACCTTTTTCATATGTTCTTTTAAAATCTGATTTCAAGTCAGCTCC

ATATTTAACTCTCTTGCCTT

In100 candidate2562 (in intron):

LOCUS ENSG00000171659

DESCRIPTION Probable G-protein coupled receptor 34.

[Source:Uniprot/SWISSPRT;Acc:Q9UPC5]

GTGAAGACACATATAACAAAGTGAAACCCTTTTCTCTATGGCTTTGTAAAGCATTCTCATCTCCAGGGTAACGTGCCTAT

TTCCTTCAACTTGCTTCTTT

In100 candidate2563 (in intron):

LOCUS ENSG00000114098

DESCRIPTION armadillo repeat containing 8 isoform 1

[Source:RefSeq_peptide;cc:NP_054873]

TATTTTTATACAATAATTCTTCCTTATAAACTATTTACTAGTGCATAACGTACTAGTTACCTCACATTTTGGTTTCAGAA

CTGATTTCTGTCACTTCTTA

In100 candidate2564 (in intron):

LOCUS ENSG00000089127

DESCRIPTION 2',5'-oligoadenylate synthetase 1 isoform 3

[Source:RefSeq_peptde;Acc:NP_001027581]

CTCCCATTCTGTGGGTTGTCTGTTAACTCTGCTAATTATTTCTTTTGCTTTGCAGAAGCTTTTTAGTTTAATTAAGTCCC

ATCTATTTATCTTTGTTTTT

In100 candidate2565 (in intron):

LOCUS ENSG00000089101

DESCRIPTION Uncharacterized protein C20orf26.

[Source:Uniprot/SWISSPROT;AccQ8NHU2]

GCACATGGCCCATCTGCTGTTTTGAGCTAATAACCTCTGTTGGCTCTTTGCTTTTTGTTTTTCAGTGATTTCTCAGCCCT

ATACTTATCACTCATTTTAT

In100 candidate2566 (in intron):

LOCUS ENSG00000176204

DESCRIPTION Leucine-rich repeat transmembrane neuronal protein 4

precursor.[Source:Uniprot/SWISSPROT;Acc:Q86VH4]

AGTGTTCCCTTTCTTTTATAACACTTTTCTTTTTCATTTTTATCTCTTTATTTTTCTTCAAGTCTTGTCAGATAGACTCA

GTCTTCAATTTTTTTGTTTT

In100 candidate2567 (in intron):

LOCUS ENSG00000176204

DESCRIPTION Leucine-rich repeat transmembrane neuronal protein 4

precursor.[Source:Uniprot/SWISSPROT;Acc:Q86VH4]

AGATATTCACTGTGATTGGTGATCGTTACTTTGTAAAATATGTTACAATAACAATGTTATTATTTACCTTTCTAACAGTG

TTTTCATTCTTCCCTTTATG

In100 candidate2568 (in intron):

LOCUS ENSG00000176204

DESCRIPTION Leucine-rich repeat transmembrane neuronal protein 4

precursor.[Source:Uniprot/SWISSPROT;Acc:Q86VH4]

AAGTTCACATATACTAACTTTAAATCTGAGCTTGATCCATTTTCGCTTACTTTCCTTTACAGATTTGTTGTTCCATCAAC

AATGGTTTATTTACTTTCCC

In100 candidate2569 (in intron):

LOCUS ENSG00000176204

DESCRIPTION Leucine-rich repeat transmembrane neuronal protein 4

precursor.[Source:Uniprot/SWISSPROT;Acc:Q86VH4]

GCAAAACTCTGTCTCAAAAAAAAAAAAAAAAAAAAAAAGGAAATGTAACGGTATTTTTTTTTGCATTCCTTTTCCCAGGA

ATCCTTTTGTATTCCTTTGT

In100 candidate2570 (in intron):

LOCUS ENSG00000176204

DESCRIPTION Leucine-rich repeat transmembrane neuronal protein 4

precursor.[Source:Uniprot/SWISSPROT;Acc:Q86VH4]

AGCTCCTGGATTCATTAACTTTTTGAAGGGTTTTTTGTGTCTCTATTTCCTTCAGTTCTGCTCTGATTTTAGTTATTTCT

TGCCTTCTGCTAGCTTTTGA

In100 candidate2571 (in intron):

LOCUS ENSG00000069535

DESCRIPTION Amine oxidase [flavin-containing] B (EC 1.4.3.4) (Monoamine

oxiase type B) (MAO-B). [Source:Uniprot/SWISSPROT;Acc:P27338]

ACAAAATCTAGTTTTTCGTGGATATACCCTTAAGCTGTATCTATAACTTGTGAAATGTCGATTTGCTTATTTCAGGACTG

TCTTTTGTTATTTTATACAT

In100 candidate2572 (in intron):

LOCUS ENSG00000175115

DESCRIPTION Phosphofurin acidic cluster sorting protein 1 (PACS-1).

[SourceUniprot/SWISSPROT;Acc:Q6VY07]

ATTACCTGGCTTGTGCTGCATCTTTATTTTTCCCTTCACCTCAAGTTAACTTTATCGTTCATATTTCTGATTTTGCAGTA

GTGTCTCTAATTTCTCCTTG

In100 candidate2573 (in intron):

LOCUS ENSG00000188624

DESCRIPTION Insulin growth factor-like family member 3 precursor.

[Source:Uiprot/SWISSPROT;Acc:Q6UXB1]

GATAATCTCCACCTTTAAACCACCCTAACCTCTGTTTGTTTGTTTGTTTGTTTTTTCCATACTTGTCACATTCTCAGCTC

CATGTTCAACCTTTTTCCTT

In100 candidate2574 (in intron):

LOCUS ENSG00000066032

DESCRIPTION Catenin alpha-2 (Alpha-catenin-related protein) (Alpha N-cateni).

[Source:Uniprot/SWISSPROT;Acc:P26232]

TAACATACGTATTTTTTCTTATTTCCTATTTCTTCAACTATTTGTGCTTTTTAAAATTCTTTTGGTTAATTTCTTCAGCT

ATCTTCTAGTTTTTTTTCTT

In100 candidate2575 (in intron):

LOCUS ENSG00000066032

DESCRIPTION Catenin alpha-2 (Alpha-catenin-related protein) (Alpha N-cateni).

[Source:Uniprot/SWISSPROT;Acc:P26232]

TAGTTTATATGTAAAAGAACACTGGAGGTTATAAAGGTAACAAAGACCTTAAACATTACATTGATTTTTTTTTCTCAGAG

CTTTTTTTCTTCTTGTTGTA

In100 candidate2576 (in intron):

LOCUS ENSG00000066032

DESCRIPTION Catenin alpha-2 (Alpha-catenin-related protein) (Alpha N-cateni).

[Source:Uniprot/SWISSPROT;Acc:P26232]

TGGATGCTCATGAGTTTCTTTTTTGTTTGCTTTTGGTGTTAACTTACTGCCTCTCTTGCGGTTTTCCAGATTGCTAAACC

TTCTCTATTGAAACTCTTCT

In100 candidate2577 (in intron):

LOCUS ENSG00000066032

DESCRIPTION Catenin alpha-2 (Alpha-catenin-related protein) (Alpha N-cateni).

[Source:Uniprot/SWISSPROT;Acc:P26232]

ATAACTTTTCTACCATACAAAGTCTTTTCCTTTCTTCTCCTGAATCACATTCTTTTTCTCAGCAATCATTTACCATTTGA

TTTGAACGTCTCTCTTTCTC

In100 candidate2578 (in intron):

LOCUS ENSG00000066032

DESCRIPTION Catenin alpha-2 (Alpha-catenin-related protein) (Alpha N-cateni).

[Source:Uniprot/SWISSPROT;Acc:P26232]

GAAAAAGCTATTAACTTGATCTCATAATGCCCAAGAGTTTTGCTTTTTAACTTTTCCTATTTTTTTTTAACTCTACAGTC

TTAAGTTTATTTTCTTTTAA

In100 candidate2579 (in intron):

LOCUS ENSG00000107341

DESCRIPTION ubiquitin-conjugating enzyme UBC3B

[Source:RefSeq_peptide;Acc:N_060281]

ATTGTCTTTGCTCCTAGTAAAATAAAATTATGTATTGTCTTTTAACATGTAAAAAATCATCTTTCTTATATTCAGTTTCT

TTGTCTTTAGCTAGAGCTTT

In100 candidate2580 (in intron):

LOCUS ENSG00000051382

DESCRIPTION Phosphatidylinositol-4,5-bisphosphate 3-kinase catalytic subuni

beta isoform (EC 2.7.1.153) (PI3-kinase p110 subunit beta) (PtdIns-3-kinas p110)

(PI3K) (PI3Kbeta). [Source:Uniprot/SWISSPROT;Acc:P42338]

GTTTAGTAATTTATAGGTATCGATATAGAATCTACGTAGTAACAAATGGGAATTTCTGCTTGTTCTTCCCATTTGCAGGT

TATCATATTTGTCTTCTGTA

In100 candidate2581 (in intron):

LOCUS ENSG00000051382

DESCRIPTION Phosphatidylinositol-4,5-bisphosphate 3-kinase catalytic subuni

beta isoform (EC 2.7.1.153) (PI3-kinase p110 subunit beta) (PtdIns-3-kinas p110)

(PI3K) (PI3Kbeta). [Source:Uniprot/SWISSPROT;Acc:P42338]

AACTTTCCTGTTCTCTCCTTTATGTCTCTTAACCTTCCATATTTTCTATCTTTTCATATCTTATCATTATAACAGTTTTA

CTCAAGTCTCTGTGTGCTCT

In100 candidate2582 (in intron):

LOCUS ENSG00000163541

DESCRIPTION Succinyl-CoA ligase [GDP-forming] subunit alpha, mitochondrial

recursor (EC 6.2.1.4) (Succinyl-CoA synthetase subunit alpha) (SCSalpha).[

Source:Uniprot/SWISSPROT;Acc:P53597]

TAGCATTCTAACTGGCCTTTCTGTTTTTAGACATACCTTTCATCATTTTTGTGTCCTATTTTCAGACTACACTTCCCCAT

AGTCTTTTAGTTGTAGTTCT

In100 candidate2583 (in intron):

LOCUS ENSG00000188559

DESCRIPTION OTTHUMP00000030392 (Fragment).

[Source:Uniprot/SPTREMBL;Acc:Q5TK3]

TGCATATTTATTATTCAAAAGGTCAAGTAACTAAGACCTCTGTTCTTTTAAACCAAATCTGTTTTCTTCTCGGTCAGCAT

TTTCTTTACCCAGAGCTCTT

In100 candidate2584 (in intron):

LOCUS ENSG00000188559

DESCRIPTION OTTHUMP00000030392 (Fragment).

[Source:Uniprot/SPTREMBL;Acc:Q5TK3]

AGAAAATGCAATAGATCTAGGATGTATTATATAATTTTATTGTAACTGATATAATTCTTTCTCCATTTTAAATATCAGAT

AATCCTGTCTTTTGGTATTT

In100 candidate2585 (in intron):

LOCUS ENSG00000188559

DESCRIPTION OTTHUMP00000030392 (Fragment).

[Source:Uniprot/SPTREMBL;Acc:Q5TK3]

GTATCTGTTTATAACCTTCACCCACTTTCATATGGGGTTGTTTGTTTGTTTGTTTTTTCTTTTTTTTACCATATACAGTT

TTATTTTTTAATTTATTTTT

In100 candidate2586 (in intron):

LOCUS ENSG00000147050

DESCRIPTION Ubiquitously transcribed X chromosome tetratricopeptide repeat

rotein (Ubiquitously transcribed TPR protein on the X chromosome).

[Source:niprot/SWISSPROT;Acc:O15550]

TGGGTGACGATTAACTGGGTTTCCTTTTTTCTTCCCTCCATCCTTTTTGTGTGTGTTAATGATTATTTCATTAGGCAGTT

GTTTTGTTGTTGTTGTTGTT

In100 candidate2587 (in intron):

LOCUS ENSG00000147050

DESCRIPTION Ubiquitously transcribed X chromosome tetratricopeptide repeat

rotein (Ubiquitously transcribed TPR protein on the X chromosome).

[Source:niprot/SWISSPROT;Acc:O15550]

AGTCTTTTTAACTTTAGCCCATCTGCCCATTCTTGTTTTTTCTTCTTTCTTGTTTTCTTTCTTTCTTTCCTCCTCAGTCT

TTTTAACTTTAGCCCGTCTA

In100 candidate2588 (in intron):

LOCUS ENSG00000147050

DESCRIPTION Ubiquitously transcribed X chromosome tetratricopeptide repeat rotein (Ubiquitously transcribed TPR protein on the X chromosome). [Source:niprot/SWISSPROT;Acc:O15550]

ACATACTGACATATTGATTCTTTGAAAAGAAATTTAGAACTCATATCTAACAACCCCTCCCCTTGTATTTCCTCACAGCT
TTCTTTTCAAACTTTTTTTA

In100 candidate2589 (in intron):

LOCUS ENSG00000147050

DESCRIPTION Ubiquitously transcribed X chromosome tetratricopeptide repeat rotein (Ubiquitously transcribed TPR protein on the X chromosome). [Source:niprot/SWISSPROT;Acc:O15550]

ATTTGAGTTACTGCTAGTTTTAAAAAAGTCTTATCCAATAGTAACTTTTAATGAAGTATTTAAATGCCTTTCCTTCAGAC
TTCCAGTATTTTTTTCTATA

In100 candidate2590 (in intron):

LOCUS ENSG00000186854

DESCRIPTION

CCGTCTCAAAAAAAAAAAAAAAAAAAAAAAAGATTCACATGAAGTCTAACCTTTTGGGAGCAATTTGCTTTTATCTCAGTC
TTTTGCTTTTTTACATAATT

In100 candidate2591 (in intron):

LOCUS ENSG00000176407

DESCRIPTION potassium channel modulatory factor 1 [Source:RefSeq_peptide;Ac:NP_064507]

AATAGAACATCTCATCCACATGTCCATATCCACTAACTGGATCTTTGTTTTGATAATCCTCTTCCCTTTCTCTGCAGGTT

TACTCCCAGTATATCCATTT

In100 candidate2592 (in intron):

LOCUS ENSG00000088930

DESCRIPTION 5'-3' exoribonuclease 2 (EC 3.1.13.-) (DHM1-like protein) (DHP

rotein). [Source:Uniprot/SWISSPROT;Acc:Q9H0D6]

TTAGGCTTTGACCACCAAGTATGATGTTAACTGTTAGGTTATTTTTTTGTTTGTTTGTTTTTGTTTTTGCAGATGCCCTT

TATGTTGAGAATTTCCCTTC

In100 candidate2593 (in intron):

LOCUS ENSG00000182404

DESCRIPTION LYR motif-containing protein 4.

[Source:Uniprot/SWISSPROT;Acc:QHD34]

CACTCATAGGAGAAGAAAGCCCTGTAACCCCAAGGGTTGTTTCCTTGTTGTTACTTTTGTTGTTTGTCATGTCAGCCCTC

TCTTCTTGTTTAAGGAATCT

In100 candidate2594 (in intron):

LOCUS ENSG00000145982

DESCRIPTION Phenylalanyl-tRNA synthetase, mitochondrial precursor (EC

6.1.120) (Phenylalanine--tRNA ligase) (PheRS).

[Source:Uniprot/SWISSPROT;Acc:O9563]

TGGGGATTTAGGATTTCTAACTTTTAAATTTCTCTTTCTGTCCTTGAGTTTCCAGCCTTTCTTTGGCACAGTTTCTTTGC

TAAACATTGGCATTTCCCTT

In100 candidate2595 (in intron):

LOCUS ENSG00000145982

DESCRIPTION Phenylalanyl-tRNA synthetase, mitochondrial precursor (EC

6.1.120) (Phenylalanine--tRNA ligase) (PheRS).

[Source:Uniprot/SWISSPROT;Acc:O9563]

TTCTGTTAACTCCTTCCTGTGTATTTTTCATCTCATTCATTTGCTTCAGTTTGGATCTTTTTAATATCATCCCTGTTTCT

ACTTAACGTGTTTAGTCTTT

In100 candidate2596 (in intron):

LOCUS ENSG00000145982

DESCRIPTION Phenylalanyl-tRNA synthetase, mitochondrial precursor (EC

6.1.120) (Phenylalanine--tRNA ligase) (PheRS).

[Source:Uniprot/SWISSPROT;Acc:O9563]

GTCTAAAATGTCAAAAGAAAACTAGATGAGGAAAAAGCAATCGAAAATAACTCTATTACAAGCTTCCTTATAGTTCAGAT

TCATCCCTTTCTTTTTGACC

In100 candidate2597 (in intron):

LOCUS ENSG00000114120

DESCRIPTION solute carrier family 25, member 36

[Source:RefSeq_peptide;Acc:P_060625]

TCTTTAATGATTTGTGCTTCTTTTGATGTCCATATAAGAAGTCTGTGCCTAACGCAAAGTTAAAAGATTTTTTTCCAGAT

GTTTTTTTCCTAGATGTTTT

In100 candidate2598 (in intron):

LOCUS ENSG00000123066

DESCRIPTION Thyroid hormone receptor-associated protein 2 (Thyroid hormone eceptor-associated protein complex 240 kDa component-like). [Source:UniprotSWISSPROT;Acc:Q71F56]

TCTGAGCACTGAGGATTCCTATTGCTACTAACTTACTCATACCTTTTAGGCCTTTTCAGTGAACAGACTTAGACGCATGT

CTTTTTTTTTTTCTTTTTTT

In100 candidate2599 (in intron):

LOCUS ENSG00000123066

DESCRIPTION Thyroid hormone receptor-associated protein 2 (Thyroid hormone eceptor-associated protein complex 240 kDa component-like). [Source:UniprotSWISSPROT;Acc:Q71F56]

AGCCTCACTCTTGTCTTTTGTGGTAACTTACACTAGTCTTTCCTTATATGTCTTCACTTTTACCACCAGCTCTAGCACTG

GACTGTTTTTTTATTTTTAT

In100 candidate2600 (in intron):

LOCUS ENSG00000123066

DESCRIPTION Thyroid hormone receptor-associated protein 2 (Thyroid hormone eceptor-associated protein complex 240 kDa component-like). [Source:UniprotSWISSPROT;Acc:Q71F56]

GTTAAATTCTTCATTATTATTCTCTAGTTTATTAACTCCTTTTCAATTTTTACTGTTTACATTTTATCTCTTACTCAGTT

TTTCATTTCCATTGCTTATT

In100 candidate2601 (in intron):

LOCUS ENSG00000065923

DESCRIPTION Sodium/hydrogen exchanger 7 (Na(+)/H(+) exchanger 7) (NHE-7)

(Slute carrier family 9 member 7). [Source:Uniprot/SWISSPROT;Acc:Q96T83]

GAATTGCATTGTCATCTTTATCGAAAATCAATTAACCATAAACACACCATTTTATTTTTGGACTCTCAGTTTTGCTCCAT

TGAGCTATGTGTCTATCCTT

In100 candidate2602 (in intron):

LOCUS ENSG00000087206

DESCRIPTION Ubiquitin interaction motif-containing protein 1 (Retinoid X

reeptor- interacting protein 110) (Receptor-associated protein 80) (Nuclear znc

finger protein RAP80). [Source:Uniprot/SWISSPROT;Acc:Q96RL1]

TAATTTAGCTACTCAAAGATGATAACCTTTAATGTATTGGCCTATTTTCTTCCAGTCTTTTTTTCTATGCATTGTTAATG

TAATTAACACTATTCTCCCT

In100 candidate2603 (in intron):

LOCUS ENSG00000069849

DESCRIPTION Sodium/potassium-transporting ATPase subunit beta-3

(Sodium/potssium- dependent ATPase beta-3 subunit) (ATPB-3) (CD298 antigen).

[Source:Uiprot/SWISSPROT;Acc:P54709]

TTTTCCTAGCTTCATAATCCTAGCCACCCGTACCAAATTTCATTAACACTCCCACCAACCTTGTCCTCATTTTATCAGGA

TCTGGTAGTGCCTTTTCTTT

In100 candidate2604 (in intron):

LOCUS ENSG00000112799

DESCRIPTION Lymphocyte antigen 86 precursor (MD-1 protein).

[Source:UniprotSWISSPROT;Acc:O95711]

AGTAACTGAACAAGTCTTTCATGGCTATGTTGTTCTGTTCTGTTTTGTCTTCTTCCTTTGATCTAAGGTCAGCCTCTAAA

ATTTTTATTTTCTACCACTG

In100 candidate2605 (in intron):

LOCUS ENSG00000114126

DESCRIPTION Transcription factor Dp-2 (E2F dimerization partner 2).

[SourceUniprot/SWISSPROT;Acc:Q14188]

AAAAAATAATTTAATAATTTAACTTTCATTTCTTATATGATGTTCCTTTTGTTTGTTTCTTTCTATTTCTTTAATCAGCT

CTTACTCATTTCTTTCTGTT

In100 candidate2606 (in intron):

LOCUS ENSG00000114126

DESCRIPTION Transcription factor Dp-2 (E2F dimerization partner 2).

[SourceUniprot/SWISSPROT;Acc:Q14188]

AAGGTCTTAAATACTTGGTAAATGAATAAATGTTGACTAGACTTTAATAACTAGGTCTGAACTATTTTTATGCTTCAGAG

GTTATTGTTTGACTCTTTCC

In100 candidate2607 (in intron):

LOCUS ENSG00000174989

DESCRIPTION F-box/WD repeat protein 8 (F-box and WD-40 domain protein 8) (Fbox only protein 29). [Source:Uniprot/SWISSPROT;Acc:Q8N3Y1]

ACTGGAATAACTTCCTTTCCTCTTTCCTGTTCTTTGCCTGCCTTTTAAAGCTCAGCTTGAGTTCTGTCTCCTGTGTTAAT
TCTTCCGTGATTTCTCCAGC

In100 candidate2608 (in intron):

LOCUS ENSG00000174989

DESCRIPTION F-box/WD repeat protein 8 (F-box and WD-40 domain protein 8) (Fbox only protein 29). [Source:Uniprot/SWISSPROT;Acc:Q8N3Y1]

ATTAACTGCATTTTTATTTTTTTATTGGCTGTTTTGTGTATGATTTACTCTTTTGTCACTGGCTCCCCCTCTCCCCAGTT
GTCTATCTTTCTCATTCCTG

In100 candidate2609 (in intron):

LOCUS ENSG00000008294

DESCRIPTION C-jun-amino-terminal kinase-interacting protein 4 (JNK-interactng protein 4) (JIP-4) (JNK-associated leucine-zipper protein) (JLP) (Spermssociated antigen 9) (Mitogen-activated protein kinase 8- interacting proten 4) (Human lung cancer protein 6) (HLC [Source:Uniprot/SWISSPROT;Acc:O6027]

CTCATTTTAGTGAATGGCTTTCTCAAATGCCATCTATTATTTTTAACCAATGGTCTTTTATGACTTCTTTCCCAGTGTCT
GTTTACTAATTTGTGTTCTT

In100 candidate2610 (in intron):

LOCUS ENSG00000008294

DESCRIPTION C-jun-amino-terminal kinase-interacting protein 4 (JNK-interactng protein 4) (JIP-4) (JNK-associated leucine-zipper protein) (JLP) (Spermssociated

antigen 9) (Mitogen-activated protein kinase 8- interacting proten 4)

(Human lung cancer protein 6) (HLC [Source:Uniprot/SWISSPROT;Acc:O6027]

CCCTACTCCATTTACTGAGGTACTTAAAAATAACTAGCATGCTGTATCTCTTTTTTTCCCAGCTTTGTCTTGAATTGGCA

TTGCCATCCTTGTTTTATCT

In100 candidate2611 (in intron):

LOCUS ENSG00000204904

DESCRIPTION Putative uncharacterized protein CXorf31.

[Source:Uniprot/SWISSROT;Acc:Q5VT33]

TCACTTTTGACCCCACTCCCCTTGTTTCCCCTTGTCTTCTATAAGTAACTTGTATTAGTTTCTTATTTGCCCTTTCAGTG

GTTCTTTTTTGGGTTTTTTT

In100 candidate2612 (in intron):

LOCUS ENSG00000175054

DESCRIPTION Serine/threonine-protein kinase ATR (EC 2.7.11.1) (Ataxia

telaniectasia and Rad3-related protein) (FRAP-related protein 1).

[Source:Unipro/SWISSPROT;Acc:Q13535]

GAAGTACTTATTTTCTGTGTGCATGTGCTACCAATTTAACATTTAGTTAGGTTCACTTGTTTCAGTTTCTTTAAAACTTT

TAAGGGTTGCTTTTTCCCTC

In100 candidate2613 (in intron):

LOCUS ENSG00000148482

DESCRIPTION solute carrier family 39 (zinc transporter), member 12

[Source:efSeq_peptide;Acc:NP_689938]

TTTTTAATTGGAAATTGTCTGTTTTCCTCTTAACTTTTAAAAGTTCCTTGTCTATTTTGGATACCATTCCTTTATCAGAT

GTTTTGCACATGTTTTCCCC

In100 candidate2614 (in intron):

LOCUS ENSG00000068654

DESCRIPTION DNA-directed RNA polymerase I largest subunit (EC 2.7.7.6)

(RNApolymerase I 194 kDa subunit) (RPA194). [Source:Uniprot/SWISSPROT;Acc:O9560]

TTTCTCAAAAAAGAAAACTGTGACCTTAACTCTACATATTATCTTGTATTCTGTTTTTTCCTCACTCAGTAATATTTTTA

TGGTTGTCCTCCCATGCCCT

In100 candidate2615 (in intron):

LOCUS ENSG00000171435

DESCRIPTION Kinase suppressor of ras-2 (hKSR2).

[Source:Uniprot/SWISSPROT;Ac:Q6VAB6]

TGTGGACATTTTAACAATATTAATTTTTCCATTCTATGAACATGGGATATCTTTCCATTTTTCTCATGTCTTCTTCAGTT

TCTTTTTTCAGTGTTTTATA

In100 candidate2616 (in intron):

LOCUS ENSG00000120756

DESCRIPTION Plastin-1 (I-plastin) (Intestine-specific plastin).

[Source:Unirot/SWISSPROT;Acc:Q14651]

AACATAACTAGAACTATGCTTTTTGGCCTTTTGACAAAATTACCTGTAACCTTTTCCCCTCTTTCTTCATTTCAGATCTG

AATCTGTTAATTATCCTCTT

In100 candidate2617 (in intron):

LOCUS ENSG00000132305

DESCRIPTION Mitochondrial inner membrane protein (Mitofilin) (p87/89)

(Prolferation-inducing gene 4 protein). [Source:Uniprot/SWISSPROT;Acc:Q16891]

AGGATATCTTGTACTATTCCTGTAACTTTGTGTGTATTCCTTTTTTTCTCACCAGCCTTAAAATTTTTTTCTTTTTTTTT

TCTTTTGAGACAGAGTCTCG

In100 candidate2618 (in intron):

LOCUS ENSG00000165995

DESCRIPTION Voltage-dependent L-type calcium channel subunit beta-2 (CAB2)

Calcium channel voltage-dependent subunit beta 2) (Lambert-Eaton

myasthenicsyndrome antigen B) (MYSB). [Source:Uniprot/SWISSPROT;Acc:Q08289]

GGTACCTTTGTTATAATTAATGAACTAATACTGATATACTAACCAACTAAAGTCCATACTTTATTCAAATTTTCTCAGTT

TTTCCTTAAGTTACTTTTAC

In100 candidate2619 (in intron):

LOCUS ENSG00000165995

DESCRIPTION Voltage-dependent L-type calcium channel subunit beta-2 (CAB2)

Calcium channel voltage-dependent subunit beta 2) (Lambert-Eaton

myasthenicsyndrome antigen B) (MYSB). [Source:Uniprot/SWISSPROT;Acc:Q08289]

ATTACCACCATTTTATAACCATGCCAATTGCTGTGTATTTTTTTAAATCTGTTTTCCTATTTGCTTCTCTGAACTCAGTT

CCTATCTTTGGAGCTTTCTA

In100 candidate2620 (in intron):

LOCUS ENSG00000165995

DESCRIPTION Voltage-dependent L-type calcium channel subunit beta-2 (CAB2) Calcium channel voltage-dependent subunit beta 2) (Lambert-Eaton myasthenicsyndrome antigen B) (MYSB). [Source:Uniprot/SWISSPROT;Acc:Q08289]

ATAAGTTAACTTCTGTTTTTTGTTTTTTAACTTTTAAGTTCCTATCACCCAGGTATTAAGACTAGTACTCACCAGTTATT
TTTTCCTGATTCTCTTCTTC

In100 candidate2621 (in intron):

LOCUS ENSG00000144935

DESCRIPTION Short transient receptor potential channel 1 (TrpC1) (TRP-1 proein). [Source:Uniprot/SWISSPROT;Acc:P48995]

CTACTGGTTTTCTATCAATTTCTGGCAAGAGAGTATTAAGATTTCTAACAATTGCTCCATTATCTTTTTCTGTGTCAGTT
TTTGCTTTGTATATTTTCAG

In100 candidate2622 (in intron):

LOCUS ENSG00000135090

DESCRIPTION Serine/threonine-protein kinase TAO3 (EC 2.7.11.1) (Thousand an one amino acid protein 3) (Jun kinase-inhibitory kinase) (JNK/SAPK- inhibiory kinase) (Dendritic cell-derived protein kinase) (Cutaneous T-cell lymphma tumor antigen HD-CL-09) (CTCL tumor [Source:Uniprot/SWISSPROT;Acc:Q9H2K8

ATTCTTTGTAGTAATACAATAGAAATGTAATTTCCTAACCCCTAATCCTTGTTTGTTTTGATCTTTCAGGGTCTTCTTTT
TACTGTACAGAGTTATTTTT

In100 candidate2623 (in intron):

LOCUS ENSG00000115548

DESCRIPTION JmjC domain-containing histone demethylation protein 2A (EC 1.1.11.-) (Jumonji domain-containing protein 1A). [Source:Uniprot/SWISSPROT;Ac:Q9Y4C1]

TTCAAGTTTGTGTTTATCGTTTGCTTATATTTACAATTTTAACATATATATTTGTATTCCTGCACAATATTCTTTCAGTT
TTTAGCCTGCTTTTGAACTT

In100 candidate2624 (in intron):

LOCUS ENSG00000154975

DESCRIPTION Carbonic anhydrase-related protein 10 (Carbonic anhydrase-relatd protein X) (CARP X) (CA-RP X) (Cerebral protein 15). [Source:Uniprot/SWISPROT;Acc:Q9NS85]

TCCCACTCTGTGGGTTGTCTATTAACTCTGCTGATTTATTTCTTTTGCTGTGCAGAAGCTTTTTAGTTTAATTAAGTCCC
ATCTGTTTATCTTTGTTTTT

In100 candidate2625 (in intron):

LOCUS ENSG00000122735

DESCRIPTION Dynein intermediate chain 1, axonemal (Axonemal dynein intermedate chain 1). [Source:Uniprot/SWISSPROT;Acc:Q9UI46]

GCCTCTCTATTTCTCTTAACAACTATGTTTCATTCTTATTCTTATTTCCCAACTGGCCAGATTTCCATGGTCTCCGCTCC
CTCATAATTTTAGTCTGTTT

In100 candidate2626 (in intron):

LOCUS ENSG00000181804

DESCRIPTION Sodium/hydrogen exchanger 9 (Na(+)/H(+) exchanger 9) (NHE-9)

(Slute carrier family 9 member 9). [Source:Uniprot/SWISSPROT;Acc:Q8IVB4]

GCCTAAGTATACTCAATTTTATAAAATTAGATATAATCATTAACTTGTCTTAATAATTTACTTTTCCATGCCCAGTATAC

TTTATTTTATATATCACTTT

In100 candidate2627 (in intron):

LOCUS ENSG00000181804

DESCRIPTION Sodium/hydrogen exchanger 9 (Na(+)/H(+) exchanger 9) (NHE-9)

(Slute carrier family 9 member 9). [Source:Uniprot/SWISSPROT;Acc:Q8IVB4]

TAAGTGTTGATTTTTTTTGGTGTTTGATTTTTTTTGGTGACCCATCCTAACTATGGCTGCTGCTTTTTTACTCCACAGGC

CTTTTTTAATGTTTTTTCAT

In100 candidate2628 (in intron):

LOCUS ENSG00000181804

DESCRIPTION Sodium/hydrogen exchanger 9 (Na(+)/H(+) exchanger 9) (NHE-9)

(Slute carrier family 9 member 9). [Source:Uniprot/SWISSPROT;Acc:Q8IVB4]

CATGCCCATTAACATTCTTTTACATTTTACCTTTCTATCTTTTTGTTTTTTAACCTGTGTTCTGGATGATTCCCTCAGAT

CCTGTTCTAATTCTCGGTTG

In100 candidate2629 (in intron):

LOCUS ENSG00000181804

DESCRIPTION Sodium/hydrogen exchanger 9 (Na(+)/H(+) exchanger 9) (NHE-9)

(Slute carrier family 9 member 9). [Source:Uniprot/SWISSPROT;Acc:Q8IVB4]

AATAAGACTCAAGGACAATTCTGTTGCCTAAAGGCAACCACTGTAACTGCTAATATTTTGACATTTTTTTCCTTCCAGTC

TTTTGTAGCTATTTCATTTA

In100 candidate2630 (in intron):

LOCUS ENSG00000181804

DESCRIPTION Sodium/hydrogen exchanger 9 (Na(+)/H(+) exchanger 9) (NHE-9)

(Slute carrier family 9 member 9). [Source:Uniprot/SWISSPROT;Acc:Q8IVB4]

TTCTCTTTTTTCTTTTGTCATTATTGGTCAAATAAATGTAACTAGATATCATTTACTTTTCTTCTCTCACAGCCTGTGGA

TTTTGATTATTTCATTCTTT

In100 candidate2631 (in intron):

LOCUS ENSG00000115561

DESCRIPTION Charged multivesicular body protein 3 (Chromatin-modifying protin

3) (Vacuolar protein sorting-associated protein 24) (hVps24) (Neuroendocine

differentiation factor). [Source:Uniprot/SWISSPROT;Acc:Q9Y3E7]

TAGAAAGTTAAAATCACCCTGAAGTTCGACCACTTAGAGATAACCACCACAAACACTTTGGTGATCATCCTTTTCCAGAT

ATATTTCTCTCTGTTTTTGT

In100 candidate2632 (in intron):

LOCUS ENSG00000153561

DESCRIPTION Protein RMD5 homolog A. [Source:Uniprot/SWISSPROT;Acc:Q9H871]

CTTTTTCCCTTAAAATTTAGTTCCACTAACCCATTTAATTCTTCATAGCTTAGTTTTTTCTTTCTAATCATTTCTCAGTT

CTTTCTCCTTTTTGAGACAG

In100 candidate2633 (in intron):

LOCUS ENSG00000105499

DESCRIPTION Cytosolic phospholipase A2 gamma precursor (EC 3.1.1.4) (cPLA2-amma) (Phospholipase A2 group IVC). [Source:Uniprot/SWISSPROT;Acc:Q9UP65]

CACCCTACTCTGTGGAGTAACGATTCTTTCACCCCTTTGCTTTCTTAACAGACTTGCTTTCGCTTTGCACTGTGGACTCA

CCCTGAATTCTTTCTTGTAT

In100 candidate2634 (in intron):

LOCUS ENSG00000108924

DESCRIPTION Hepatic leukemia factor. [Source:Uniprot/SWISSPROT;Acc:Q16534]

EXON

ATTAATATACGATTGTTGTAAAATAGGACTGTATAAAGAGTAACTTGAGTTCCCATATTCCATTTCCAGATTTAACTGTC

CACAGTTTCTGCGTACTCTT

In100 candidate2635 (in intron):

LOCUS ENSG00000114698

DESCRIPTION Phospholipid scramblase 4 (PL scramblase 4) (Ca(2+)-dependent pospholipid scramblase 4) (TRA1) (Cell growth-inhibiting gene 43 protein). [ource:Uniprot/SWISSPROT;Acc:Q9NRQ2]

TGTAGATTACTAAGAATGGAGTGTAAAAGTCCTCAACTGTAACTGTGGATTTCTCTTTATTTTTTTCAATTTTGCCAGTT

TTTCCTTCATGTATTTTGAA

In100 candidate2636 (in intron):

LOCUS ENSG00000105483

DESCRIPTION Caspase recruitment domain-containing protein 8 (Apoptotic protin NDPP1) (DACAR) (CARD-inhibitor of NF-kappa-B-activating ligand) (CARDINA)

(Tumor-up-regulated CARD-containing antagonist of CASP9) (TUCAN).

[SourceUniprot/SWISSPROT;Acc:Q9Y2G2]

GGGAGGCCCCGTCTCAAAGAAAAAAAAAGATTCTTTCTCTAACTTACTGGTTTATCTTACTTTTGTAGCTTATTTCAGGT

GATTCGTTATGTTTCTCTAT

In100 candidate2637 (in intron):

LOCUS ENSG00000204740

DESCRIPTION Novel protein (Fragment). [Source:Uniprot/SPTREMBL;Acc:Q5VYJ5]

EXON

TCATGACTCTAGTATCTCTGTTATTGTCATCATCATTGTTAACACTGTTAATTCTCTCTCAATTTCAAATTCTGTCAGCT

CTCTTTCTCAGCTGATTATA

In100 candidate2638 (in intron):

LOCUS ENSG00000120594

DESCRIPTION Plexin domain-containing protein 2 precursor (Tumor

endothelialmarker 7-related protein). [Source:Uniprot/SWISSPROT;Acc:Q6UX71]

TAACTTCTGACCTCATTTCTTTCTTGCTGATCTGCCATGCAGTCTTCTTCCTATGCAATGGCAAGACATCTGTGATTGCT

TCTCTTTTATGTCTTTCTCT

In100 candidate2639 (in intron):

LOCUS ENSG00000120594

DESCRIPTION Plexin domain-containing protein 2 precursor (Tumor endothelialmarker 7-related protein). [Source:Uniprot/SWISSPROT;Acc:Q6UX71]

AGATTTTCTCTCACTCTGTGGGTTGTCGGTTAACTCTGCTGATTATTTCTTTTGCTGTGCAGAAGCTTTTTAGTTTATTT
AAGTCCCACCTATTTATTTT

In100 candidate2640 (in intron):

LOCUS ENSG00000120594

DESCRIPTION Plexin domain-containing protein 2 precursor (Tumor endothelialmarker 7-related protein). [Source:Uniprot/SWISSPROT;Acc:Q6UX71]

AAGTTCCTTATACATTTTGGATATGAGACCCAAGGATAACTTTGACATAAGCTTTTTTTCACTTGTTTTCCCCTCCAGAT
TATTATTCATTTTTTCACCA

In100 candidate2641 (in intron):

LOCUS ENSG00000120594

DESCRIPTION Plexin domain-containing protein 2 precursor (Tumor endothelialmarker 7-related protein). [Source:Uniprot/SWISSPROT;Acc:Q6UX71]

CCTCTCCTAACCCTTGCTCTCTAGTTCATCAATCTTTCTATTTTTCTCCCAGTTTTTCAGGATCTCTGCATGTTACAATT
ATCTTTGTTTCGTTTTGTTT

In100 candidate2642 (in intron):

LOCUS ENSG00000184943

DESCRIPTION MGC4677 protein (Hypothetical protein tmp_locus_54) (Hypotheticl protein MGC4677). [Source:Uniprot/SPTREMBL;Acc:Q969Y7]

TGTGTCGTTTATATTATGGAACTATTTAACTTTTAAGTAGTTATTATTTTCCTTTTAACACCCAGGTTTTGTCTTGCTTA
GAAAGTCTGTTCCTACCCCT

In100 candidate2643 (in intron):

LOCUS ENSG00000121064

DESCRIPTION Retinoid-inducible serine carboxypeptidase precursor (EC 3.4.16-)

(Serine carboxypeptidase 1). [Source:Uniprot/SWISSPROT;Acc:Q9HB40]

AGAAATTTTGTTGTATAGAAATATAACAAGTTTCTGTTCTCACCTCCATCATTGTTTCTTTTGTACAGTCTCTGGGGTTT

GCTTAGATTCTTTCCTCATC

In100 candidate2644 (in intron):

LOCUS ENSG00000185304

DESCRIPTION RANBP2-like and GRIP domain containing 2

[Source:RefSeq_peptideAcc:NP_001071638]

CTAAAATTCTTCATCTTTCTGTCATATTTGCTAACCTCTCTCTTTTGGTTATCTCATCCTTTATTTATCCAACTTCAGAT

ATTCTTGTTCACCTCTTATG

In100 candidate2645 (in intron):

LOCUS ENSG00000078114

DESCRIPTION Nebulette (Actin-binding Z-disk protein).

[Source:Uniprot/SWISSROT;Acc:O76041]

GTAGGGTATCCTCATCCAAATTGTAACCGATTTCCCCTCCTGCTTGATTACATTCTGCATTTCTTTTGCTCTTGGCAGGA

TTATATCATCATTTTATTCT

In100 candidate2646 (in intron):

LOCUS ENSG00000078114

DESCRIPTION Nebulette (Actin-binding Z-disk protein).

[Source:Uniprot/SWISSROT;Acc:O76041]

CATGGATTTGAAAACAATAACATATGGAAATGTTGTCTCTTTCCACATTGCTTTTATTGGTTTTCTGCAGTCCTGTGCTT

TTCTCCAGCTGGGTTGCTTT

In100 candidate2647 (in intron):

LOCUS ENSG00000078114

DESCRIPTION Nebulette (Actin-binding Z-disk protein).

[Source:Uniprot/SWISSROT;Acc:O76041]

GAGTCTAGGTTTTTAACCACACACTATGCTATATTTTTCCTACCATTTCTAACCCTCTTTTTCTTGTTAAGCAAGCAGTT

TACTTCTTTCCCCAGAATTT

In100 candidate2648 (in intron):

LOCUS ENSG00000105464

DESCRIPTION Glutamate [NMDA] receptor subunit epsilon 4 precursor (N-

methylDaspartate

receptor subtype 2D) (NR2D) (NMDAR2D) (EB11).

[Source:Uniprot/WISSPROT;Acc:O15399]

AGGTGTGAGCCACCATGCCTGGTCTATTTTTCTTGAGGTTAGTAACTGTCTTTTTTCTTTTTCTTTCCTTTTTCAGTTGC

TTCATGGCTGTGTTTTCTTT

In100 candidate2649 (in intron):

LOCUS ENSG00000180891

DESCRIPTION CUE domain-containing protein 1.

[Source:Uniprot/SWISSPROT;Acc:9NWM3]

GATTATAACAAATTTTTTATTACAATTATTTTATTTTATTTTTTGGCTTCTCATGCTTATTTCCTACCTAGGCAGAATTT

TAGATTTATTTTTTAGTTTT

In100 candidate2650 (in intron):

LOCUS ENSG00000181355

DESCRIPTION MRDS1 [Source:RefSeq_peptide;Acc:NP_694548]

AGTCCTTTATTTTATGAGTGCAAAAATACAATACACCTAACAAGTATTCAAGTCCTAGTTTGTTGGTTTTCAGCCCTTTT

TTTTTTTTTTTTCAGAGAA

In100 candidate2651 (in intron):

LOCUS ENSG00000181355

DESCRIPTION MRDS1 [Source:RefSeq_peptide;Acc:NP_694548]

TACCTCTATCATTTGGTGAAAGGGGAATGTTGAAATCCTTAACTAGAATTTGTAATTTTTCGATTCTTCTTTCATCAGTA

TCAGTTTTTACTTTACTCAT

In100 candidate2652 (in intron):

LOCUS ENSG00000181355

DESCRIPTION MRDS1 [Source:RefSeq_peptide;Acc:NP_694548]

AGCGTAGAATACTGTATATCATAACATTTGATGCCCCTTTTTTTTTGCTTTTTTTCTTCCAGATGCTCCAAGATTTCTCT

GTTTATCATTTCCTTTCTGT

In100 candidate2653 (in intron):

LOCUS ENSG00000101463

DESCRIPTION UPF0338 protein C20orf39.

[Source:Uniprot/SWISSPROT;Acc:Q9H7V2]EXON

TATTGTCCATCCTTCTTCCTAACAATACAAGGAGTTTAGAATCCCCTTGCTTTCTTATGCTATTGTTCTGTATTTCAGTT

CCTTCTAACTTTTGTTCCAG

In100 candidate2654 (in intron):

LOCUS ENSG00000204683

DESCRIPTION Putative uncharacterized protein C10orf113.

[Source:Uniprot/SWISPROT;Acc:Q5VZT2]

GTAGGGTATCCTCATCCAAATTGTAACCGATTTCCCCTCCTGCTTGATTACATTCTGCATTTCTTTTGCTCTTGGCAGGA

TTATATCATCATTTTATTCT

In100 candidate2655 (in intron):

LOCUS ENSG00000204683

DESCRIPTION Putative uncharacterized protein C10orf113.

[Source:Uniprot/SWISPROT;Acc:Q5VZT2]

CATGGATTTGAAAACAATAACATATGGAAATGTTGTCTCTTTCCACATTGCTTTTATTGGTTTTCTGCAGTCCTGTGCTT

TTCTCCAGCTGGGTTGCTTT

In100 candidate2656 (in intron):

LOCUS ENSG00000101474

DESCRIPTION Adipocyte plasma membrane-associated protein (BSCv protein).

[Surce:Uniprot/SWISSPROT;Acc:Q9HDC9]

GATATGATCATGTGATTATTCTTGTTTAGATTGTTGAAGTCTCTAACTGTGATAGTGGATTCTGTTTCTCCTTGCAGTTT

CATCAGTTTTTGCCTCACAT

In100 candidate2657 (in intron):

LOCUS ENSG00000078403

DESCRIPTION Protein AF-10. [Source:Uniprot/SWISSPROT;Acc:P55197]

AGTACTCGGATTGTACCTTTTTTTTTTTTTTTTTTTTTTTTTTTACATAACACAAATAACTTTTTTATTTCTGTACAGGC

TTTTTAGCCTGTACACTTTA

In100 candidate2658 (in intron):

LOCUS ENSG00000078403

DESCRIPTION Protein AF-10. [Source:Uniprot/SWISSPROT;Acc:P55197]

GAAGACCCATTAACTGCCTAGTTGACCGTACAATTTTTCATTTTTCGACTTTCCTCAGATCCCATTAATTGGCCTGTGAG

GCCTTTAATTTTCCTCTTCT

In100 candidate2659 (in intron):

LOCUS ENSG00000078403

DESCRIPTION Protein AF-10. [Source:Uniprot/SWISSPROT;Acc:P55197]

CTTCTTCTCTTTTCTTCTCTTCCCTGTATTCCCATTTTAACTATGTCATGTGTTTTGTATATGTCCCAGCATTCATGGAT

ATTTTGTTTGCTTTTGTTTT

In100 candidate2660 (in intron):

LOCUS ENSG00000078403

DESCRIPTION Protein AF-10. [Source:Uniprot/SWISSPROT;Acc:P55197]

AGGAAGTTAACTTCAACTCTACCCTCTCATTTTCCTAGTCATCATTTTTTTAAAAAGCTTTTTTCTATTATTGATCAGTT

TAACTTCTTTTTCTTTTGTA

In100 candidate2661 (in intron):

LOCUS ENSG00000082996

DESCRIPTION RING finger protein 13. [Source:Uniprot/SWISSPROT;Acc:O43567]

ATAGAAACAAAGGAAAATGAAAAAAAATCTTCTACACTTTAACTGTGTCCCTCATATTTTGGCTTTTAGTTGTCTCAGTT

CACATATTTTTATATTACCT

In100 candidate2662 (in intron):

LOCUS ENSG00000082996

DESCRIPTION RING finger protein 13. [Source:Uniprot/SWISSPROT;Acc:O43567]

ATCATATCATAATAACTCTTTTTCTTTCTGTCTTTTTTTTTTTTTTTTTTTGGTGATTTTTTTGTTATCTCAGAAAGTA

GCCCTCTAGTTTTCCTTTCT

In100 candidate2663 (in intron):

LOCUS ENSG00000082996

DESCRIPTION RING finger protein 13. [Source:Uniprot/SWISSPROT;Acc:O43567]

TTGTTCTTGAGTTATAGGAGTTCTTTGTATATTCTAGATAACATGATTTGCAAATATTTTTTCCCATTCTGTCAGTTGTC

ACTTTCTTGATAATGTCCTT

In100 candidate2664 (in intron):

LOCUS ENSG00000111846

DESCRIPTION N-acetyllactosaminide beta-1,6-N-acetylglucosaminyltransferase(

EC 2.4.1.150) (N-acetylglucosaminyltransferase) (I-branching enzyme)

(IGNT. [Source:Uniprot/SWISSPROT;Acc:Q06430]

TGTATGGTTAGTGGGGTACACTGTTTCCTGTGTATCCCTCTAACCATTAAAGTTCCCTTGTTTTCATCTCTTCTCCAGAC

TGAATATTCTGTTTCCTCTA

In100 candidate2665 (in intron):

LOCUS ENSG00000157837

DESCRIPTION Signal peptide peptidase-like 3 (EC 3.4.23.-) (SPP-like 3 proten)

(Intramembrane protease 2) (IMP2) (Presenilin-like protein 4).

[Source:Uiprot/SWISSPROT;Acc:Q8TCT6]

TTCTGCTGCCTTTCTGAAATAACCTACTATTCTGGGTTTTCCTTCTCTGTCTCTCTTTCATCTTGGTTTTCTTTGCAGTT

CCTTCTTCTACCTGATCTTT

In100 candidate2666 (in intron):

LOCUS ENSG00000157837

DESCRIPTION Signal peptide peptidase-like 3 (EC 3.4.23.-) (SPP-like 3 proten)

(Intramembrane protease 2) (IMP2) (Presenilin-like protein 4).

[Source:Uiprot/SWISSPROT;Acc:Q8TCT6]

ACCTAATATACATTATCATAGTACCCTATACTTCTCTTTCTCTCTTTTGTAACATTCTTCATGATTATATTCATTCAGTT

ATGTTCATTTTTTTATTTCT

In100 candidate2667 (in intron):

LOCUS ENSG00000113240

DESCRIPTION Dual specificity protein kinase CLK4 (EC 2.7.12.1) (CDC-like

kiase 4). [Source:Uniprot/SWISSPROT;Acc:Q9HAZ1]

TTATTAGTCATTTGGGCAAGCTGAATGAGGTTAACTGAGTGGGTTTTACTCCATTTTAGCTTTTTTCAGCTCCTTGCTGA

ATTGTGAAATTCTTCTCTTT

In100 candidate2668 (in intron):

LOCUS ENSG00000136770

DESCRIPTION DnaJ homolog subfamily C member 1 (DnaJ protein homolog MTJ1).

Source:Uniprot/SWISSPROT;Acc:Q96KC8]

TCCTTAATTTAAAAATGTTTATTAGCCTGTCTTGTAACTGAGAATTTCCTGCTTCTATTTGGTTTCTGGTTCCAGTGCTA

TTTTTAAATTTTTTGGTTTT

In100 candidate2669 (in intron):

LOCUS ENSG00000136770

DESCRIPTION DnaJ homolog subfamily C member 1 (DnaJ protein homolog MTJ1).

Source:Uniprot/SWISSPROT;Acc:Q96KC8]

CCTTTTGATGTCTACATGCCCTGTAGTGATTTCTCCTTTTTAATATAGGTAACTTTTGTCTTCTGTTTCCTTTGTCAGAT

TCGCCAGTTGTTGATCTTTT

In100 candidate2670 (in intron):

LOCUS ENSG00000178338

DESCRIPTION zinc finger protein 354B [Source:RefSeq_peptide;Acc:NP_478137]

EXON

CTGCTCCATTCTCTTGGTCACTCTTACACACTCACCCGCCTTAACTGAGTTTGTTTTATTCATTTGTTCACTTATCAGTT

CAGCTGTCATCCATTATTTT

In100 candidate2671 (in intron):

LOCUS ENSG00000197586

DESCRIPTION Ectonucleoside triphosphate diphosphohydrolase 6 (EC 3.6.1.6)

(TPDase 6) (CD39 antigen-like 2). [Source:Uniprot/SWISSPROT;Acc:O75354]

AATTTTGCATTTTGCTAACGATTCATGATGTTGAGCCTCTTTTTGTATGCTTTTTATCTGTTATTTTTAAACAGATTGC

TATTTTTGTGGTTGACTTTT

In100 candidate2672 (in intron):

LOCUS ENSG00000178187

DESCRIPTION Zinc finger protein 454. [Source:Uniprot/SWISSPROT;Acc:Q8N9F8]

EXON

TGATAACTCTGGTATCTGTGTTCTACGGTTCTTTTTCTATTGTCTGTTTTTCCCTCTTCATTGAATTTCTTAGCACAGCT

GGCCTTTTTTATTGTATCCC

In100 candidate2673 (in intron):

LOCUS ENSG00000111837

DESCRIPTION Serine/threonine-protein kinase MAK (EC 2.7.11.22) (Male germ

cll- associated kinase). [Source:Uniprot/SWISSPROT;Acc:P20794]

GTGCTTCCTTGTGAATAGATGCTGAATATAATGCTTTGTAACATAGTTTTTCTACTTTTTTTCTTAATATATCAGATGGC

ACTTATTTCCATATCTATTC

In100 candidate2674 (in intron):

LOCUS ENSG00000147117

DESCRIPTION Zinc finger protein 157 (Zinc finger protein HZF22).

[Source:Unprot/SWISSPROT;Acc:P51786]

GAAGCAATGCTTTGTGGAATACCATGATGGTGGATTTCCTTTGTTGCATAACCCATGTATTCATTCCTTTACTTTCAGCT

ATTATTTCTTTTTTTATTTC

In100 candidate2675 (in intron):

LOCUS ENSG00000121101

DESCRIPTION Testis-expressed protein 14 (Testis-expressed sequence 14)

(Proein kinase-like protein SgK307) (Sugen kinase 307).

[Source:Uniprot/SWISSPRT;Acc:Q8IWB6]

GCCCTTTCCTTTTCAAAGCTAACAATTTAGCATCTTCTGACTTTTCTTCTGTCTTTATATCTTTTTGTGGACCTCCAGAG

TCCTTCTGTTTTTTTTCTTT

In100 candidate2676 (in intron):

LOCUS ENSG00000110931

DESCRIPTION Calcium/calmodulin-dependent protein kinase kinase 2 (EC

2.7.1117) (Calcium/calmodulin-dependent protein kinase kinase beta) (CaM-kinase

knase beta) (CaM-KK beta) (CaMKK beta). [Source:Uniprot/SWISSPROT;Acc:Q96RR4

CTGCTCCCTAAGAGTATTTGTTACTCACATTAACTATTTTTTCTCTTCTTTCTTTTGCCTGCTTTGTTTTTCTGTCAGAT

ATTTATTACTCTCATTTTAC

In100 candidate2677 (in intron):

LOCUS ENSG00000175175

DESCRIPTION protein phosphatase 1E [Source:RefSeq_peptide;Acc:NP_055721]

TATGTAATTATTATAACGTTAAATGTATTACTTTCTAATTCTTTTGCTGAAAATGGCCTTTTATCTTTTTTCTTGCAGTA

ATTGCCCTTCCTTTTTGGCT

In100 candidate2678 (in intron):

LOCUS ENSG00000135956

DESCRIPTION Transmembrane protein 127. [Source:Uniprot/SWISSPROT;Acc:O75204

TTTTTTTAAAATTGTGGTAAAATATACATAACATAAAACTTAGCATTTTAACTTTTTTTTTTTTTTTTTGAGACAGAG

TTTCTCTCTTGTTGCCCATG

In100 candidate2679 (in intron):

LOCUS ENSG00000100997

DESCRIPTION Abhydrolase domain-containing protein 12.

[Source:Uniprot/SWISSROT;Acc:Q8N2K0]

GCCAAATCATAACACGACAACTATTTTTTTATCTTCCAGTTTTTATGGTTATACTTTGTATTTTCTCTTGCCTTATTGTT

TTAACTTGCACTTCCAGAAT

In100 candidate2680 (in exon):

LOCUS ENSG00000205269

DESCRIPTION Novel protein. [Source:Uniprot/SPTREMBL;Acc:Q5T4T1]

TTTATATTCCTGATGTTTTTACAAATTAAATTTAGGCATAACTTTTCCAAGTCAATCTGATTTTTTCTTTTCTGCCAGAC

TTGAATTGTGTTCTTCTTTT

In100 candidate2681 (in intron):

LOCUS ENSG00000108406

DESCRIPTION Probable ATP-dependent RNA helicase DHX40 (EC 3.6.1.-) (DEAH bo protein 40) (ARG147) (Protein PAD). [Source:Uniprot/SWISSPROT;Acc:Q8IX18]

EXON

TGTGTACCTGCTAGTTTGCTAGAAAAGGAGAAACCAAAGTGGTTAACAATTAACAAGTCTTCTTTTATTTTCCAGAAACT

TAACTTGCTTTGATTCTTCT

In100 candidate2682 (in intron):

LOCUS ENSG00000095951

DESCRIPTION Zinc finger protein 40 (Human immunodeficiency virus type I enhncer- binding protein 1) (HIV-EP1) (Major histocompatibility complex-bindin protein 1) (MBP-1) (Positive regulatory domain II-binding factor 1) (PRDIIBF1). [Source:Uniprot/SWISSPROT;Acc:P15822]

TCCATTTTGTAAAAATTGAGTTATCTTTTTCTTAATATATTTTGAATAACTATTCATTTTATGTGCTTTATTTTTCAGAT

TTATTTTTTACTTCATTTAA

In100 candidate2683 (in intron):

LOCUS ENSG00000114982

DESCRIPTION CDNA FLJ10081 fis, clone HEMBA1002018. [Source:Uniprot/SPTREMBLAcc:Q9NWE5]

AGTTTAGAAAATAGAAGGAAAAAATAACCCCAACTCTCCCTACTCTAGCATAGTTCATCTTTTTTCATTTTCCAGTTTTC

GTCCGTAATATCTTGCATTT

In100 candidate2684 (in intron):

LOCUS ENSG00000101003

DESCRIPTION DNA replication complex GINS protein PSF1.

[Source:Uniprot/SWISPROT;Acc:Q14691]

AATAACTTTTTTGTCTCTCATTTCCTTCATTATTGCCTTCTTCAGTATTTAGTTGATTTTTTGAAACATTTAGATTTCTT

TCTCATTTCCTCATTACCCC

In100 candidate2685 (in intron):

LOCUS ENSG00000062716

DESCRIPTION transmembrane protein 49 [Source:RefSeq_peptide;Acc:NP_112200]

EXON

ACCACTATGCGGTATTGTTCTTTTTCGTAACTTTGCCTAGTGTGTCATTTCTTCCCATCTGCATGTCTACTTTTTCAGTT

TTCTTACCTTTCAATTTATG

In100 candidate2686 (in intron):

LOCUS ENSG00000101004

DESCRIPTION Ninein-like protein. [Source:Uniprot/SWISSPROT;Acc:Q9Y2I6]

GGCTTCATGGTATATGAGAAGTCTGATGTAATACTTAACTTTGCTCCTAAGGGTTTTTTTTCTTCTGGCTTCTTTCAGGT

TTCTCTTTGTTTTGGGTTCC

In100 candidate2687 (in intron):

LOCUS ENSG00000108443

DESCRIPTION Ribosomal protein S6 kinase beta-1 (EC 2.7.11.1) (Ribosomal

proein S6 kinase I) (S6K) (S6K1) (70 kDa ribosomal protein S6 kinase 1) (p70 S

kinase alpha) (p70(S6K)-alpha) (p70-S6K) (P70S6K) (p70-alpha).

[Source:Unirot/SWISSPROT;Acc:P23443]

GTGTGTGTGTGTTTTTTTCTTTTTTAGTATATTATGAACTTATATGTTTTAACACATTTGTGTTTCATTTCATCGCAGTT

ATTTTTATTTTATTTTTATT

In100 candidate2688 (in intron):

LOCUS ENSG00000112137

DESCRIPTION Phosphatase and actin regulator 1.

[Source:Uniprot/SWISSPROT;Ac:Q9C0D0]

TGTGTGTGTTTTCGTTTTAACTCGACGTTTTGCTTGATGTTTAAACCTCTTTTGCTTGTTTTTTCTTTCATTGCTCAGGT

AAGTGTTACCTTCCATTTTT

In100 candidate2689 (in intron):

LOCUS ENSG00000112137

DESCRIPTION Phosphatase and actin regulator 1.

[Source:Uniprot/SWISSPROT;Ac:Q9C0D0]

ATTCTCTGTAACTCCTGTCACCTTCACATACTGTATCATCCTCTTAATCTTTTGTTCTCTTCTTACACTCAGCATATCAC

CATGGACTTTTTCTATCCTT

In100 candidate2690 (in intron):

LOCUS ENSG00000174953

DESCRIPTION Probable ATP-dependent RNA helicase DHX36 (EC 3.6.1.-) (DEAH bo

protein 36) (MLE-like protein 1) (RNA helicase associated with AU-rich eleent

ARE). [Source:Uniprot/SWISSPROT;Acc:Q9H2U1]

CCTGACAACTGAAATTAGCCACTCATTTTCACTTCAAAAAAGGAGGTAACATATAGTTCATTTGCTTTGCTTCTTCAGGT

TTGTTGTTTTATCACTGATT

In100 candidate2691 (in intron):

LOCUS ENSG00000113269

DESCRIPTION Goliath homolog precursor (RING finger protein 130).

[Source:Unprot/SWISSPROT;Acc:Q86XS8]

GGTAATTTAGATAAACATGTAACCAAATAATAATGTTGCTTTTTAATCTTTTTATCAATCAGCAATATCAGATGCTTCTT

ACTGGGTTTTTCTTTTTTTT

In100 candidate2692 (in intron):

LOCUS ENSG00000131620

DESCRIPTION transmembrane protein 16A

[Source:RefSeq_peptide;Acc:NP_060513]EXON

TTGAGTTTTTAACTCTTTTTATGATCTTTTTTATTTCTAGGAGTTCTATTTGGTTCTTTTCCAAGTCAGCTATGTCATGT

TTTAATAGTTTCCTGTCCTT

In100 candidate2693 (in intron):

LOCUS ENSG00000158023

DESCRIPTION WD repeat domain 66 [Source:RefSeq_peptide;Acc:NP_653269]

GATTGTCTAATAACCCCAATGCTCGGATCTCCTGTGGCTTCTTTCTGCTGATTGCTTTTTCCCCTTGGTTTTCAGTCATT

TGGATGGGTCTTTTTTCTTT

In100 candidate2694 (in intron):

LOCUS ENSG00000174948

DESCRIPTION Probable G-protein coupled receptor 149 (G-protein coupled recetor PGR10). [Source:Uniprot/SWISSPROT;Acc:Q86SP6]

GATTTTTTTCCCATGCTGTGGCTTGTCTGTTAACTCTGCTGATTATTTCCTTCACTGTGCAGAAGTTTTTAGCTTACTT

AAGTCCCATCTATTTATCTT

In100 candidate2695 (in intron):

LOCUS ENSG00000174948

DESCRIPTION Probable G-protein coupled receptor 149 (G-protein coupled recetor PGR10). [Source:Uniprot/SWISSPROT;Acc:Q86SP6]

ATTGATCTGTTAAAAAAATAACTTTTTGTTTCATTAATTTCCTCTTTGTTTTTCTGTTTTCAATGTCATTGATATCAGCT

TTTATCTATTTTCCTCCTTT

In100 candidate2696 (in intron):

LOCUS ENSG00000068097

DESCRIPTION amplified in breast cancer 1 (ABC1), mRNA [Source:RefSeq_dna;Ac:NM_022070]

TGTCTTATAGAATGTGAACTCTAAGCTCTGTCACCAAAGATAACAAAATAAATCACATTCTCAATTTGTCAGATTGTTTC

TTTATGGTGTTAACTTACTC

In100 candidate2697 (in intron):

LOCUS ENSG00000170832

DESCRIPTION Ubiquitin carboxyl-terminal hydrolase 32 (EC 3.1.2.15) (Ubiquitn thioesterase 32) (Ubiquitin-specific-processing protease 32) (Deubiquitinting enzyme 32) (Renal carcinoma antigen NY-REN-60). [Source:Uniprot/SWISSPOT;Acc:Q8NFA0]

ATTTAACCAAAAAAGAAGTGTAATGCTTGCCAATTTTTCTTTTTTTTTCTTTTTATTTATTTATTTTTTTGAGACAGAGT

TTCGCTCTGTTGTCCATGCT

In100 candidate2698 (in intron):

LOCUS ENSG00000170832

DESCRIPTION Ubiquitin carboxyl-terminal hydrolase 32 (EC 3.1.2.15) (Ubiquitn thioesterase 32) (Ubiquitin-specific-processing protease 32) (Deubiquitinting enzyme 32) (Renal carcinoma antigen NY-REN-60). [Source:Uniprot/SWISSPOT;Acc:Q8NFA0]

TTTTCTTGTTTTTGAATTAAAACTTAACACAACTGCTTTTTTGTCTTTCTGTCCTATTACTATCTGTCTTACCTTCAGAC

TTTTACACATCCTGTTTTAC

In100 candidate2699 (in intron):

LOCUS ENSG00000114805

DESCRIPTION phospholipase C-like 3 [Source:RefSeq_peptide;Acc:NP_055811]

TTGCCAAACCTTTGCCTCTTTTATCCCTCCTAACTTCTCTTAGTATAATTATTTTTTCATTCTTTTTCTATACTCAGAAT

CACTCCTCCTATGTTTTGAT

In100 candidate2700 (in intron):

LOCUS ENSG00000114805

DESCRIPTION phospholipase C-like 3 [Source:RefSeq_peptide;Acc:NP_055811]

TCTTCATGAGTTTAATAACCATTGCAAGCAATTTTCACCGTCTGCTTTGCAGATGTTGCTGCCAACTCATTTTCAGCCAA

GTCCTTTGCTTTTCTGATTT

In100 candidate2701 (in intron):

LOCUS ENSG00000114805

DESCRIPTION phospholipase C-like 3 [Source:RefSeq_peptide;Acc:NP_055811]

AGATGTATTGTAACTCTATTGTATGTTATTTATTTTCTCTTGCTGCTTTTAGGATTTTTTTTTTTTTTGAGACAGAGTTT

TGTTCTTATCACCCATGCTG

In100 candidate2702 (in intron):

LOCUS ENSG00000145990

DESCRIPTION glucose-fructose oxidoreductase domain containing 1

[Source:Refeq_peptide;Acc:NP_061861]

TTTATCTGACTTGCTAACAAATCTGTGTCATTCTTTTTTTTTTTTTTCTTGCCCTCCAGTTGGCTGTTTCTCCAGGTCCA

GCTGAGTTGTTTTTCTTCTT

In100 candidate2703 (in intron):

LOCUS ENSG00000174501

DESCRIPTION KIAA1641 (KIAA1641), mRNA [Source:RefSeq_dna;Acc:NM_025190]

CTTATGCAATCACATAACTATGTTTTTTTTTTTTTTTTTTTTTTTTTTTTTACAGGACCTATCTGAGTTTATAACTGTCA

CCTTTTTATCTTGGTTCTTC

In100 candidate2704 (in intron):

LOCUS ENSG00000113300

DESCRIPTION CCR4-NOT transcription complex subunit 6 (EC 3.1.-.-) (Cytoplasic deadenylase) (Carbon catabolite repressor protein 4 homolog) (CCR4 carbo catabolite repression 4-like). [Source:Uniprot/SWISSPROT;Acc:Q9ULM6]

GTTTTCTCTGGTAACCCAAATTGCATCCCTCCTTTTTGTTTTTCAGCACTAATCGCTATTTTCTTTGGAAACTTCCTTTG

ATTCTTTACCTCCTTCACCT

In100 candidate2705 (in intron):

LOCUS ENSG00000062725

DESCRIPTION Amyloid protein-binding protein 2 (Amyloid beta precursor

protenbinding

protein 2) (APP-BP2) (Protein interacting with APP tail 1).

[Souce:Uniprot/SWISSPROT;Acc:Q92624]

GGGCAATATCAAAACCAAGAAATTGACACTGGTGTTATACCATTAACTAGACTACTGGCCTTACTTAGTTTTCACCAGTT

TTTACGTGCACTCATTTGTT

In100 candidate2706 (in intron):

LOCUS ENSG00000062725

DESCRIPTION Amyloid protein-binding protein 2 (Amyloid beta precursor

protenbinding

protein 2) (APP-BP2) (Protein interacting with APP tail 1).

[Souce:Uniprot/SWISSPROT;Acc:Q92624]

CAAATCTAGAAGTAACTCTGCTTTATTGTCCTTATTTTTTTCTGCATGTTACATTTACCTTTAGCTATTTTCATACAGGT

TTTCTCTTTGTCCCCCAATT

In100 candidate2707 (in intron):

LOCUS ENSG00000198722

DESCRIPTION Unc-13 homolog B (Munc13-2) (munc13).

[Source:Uniprot/SWISSPROTAcc:O14795]

TTTTTATACTTTTTCTCATGTTTAACTTAATATTCTTTCTCATTGAGCTTCCTTTTGATTTTTATCTGCCTCTCAGCAGT

CTTCCTCAGTCTTCTGCTTT

In100 candidate2708 (in intron):

LOCUS ENSG00000196912

DESCRIPTION Ankyrin repeat domain-containing protein 36B.

[Source:Uniprot/SISSPROT;Acc:Q8N2N9]

AAATTATCTTATGCAATCACATAACTATGTTTTTTTTTTTTTTTTTTTTACAGGACCTATCTGAGTTTATAACTGTCA

CCTTTTTATCTTGGTTCTTC

In100 candidate2709 (in intron):

LOCUS ENSG00000141376

DESCRIPTION Breast carcinoma amplified sequence 3 (GAOB1) (Maab1 protein).

Source:Uniprot/SWISSPROT;Acc:Q9H6U6]

TATCATTAACTAAAATTTTAATGTTTAATTTTTAACTGGTCTGTTTTGCCTCTCTATTCTTCTGTATTCCTCTGCCAGCA

GTTTCTGCACTTTTGTTATC

In100 candidate2710 (in intron):

LOCUS ENSG00000141376

DESCRIPTION Breast carcinoma amplified sequence 3 (GAOB1) (Maab1 protein).

Source:Uniprot/SWISSPROT;Acc:Q9H6U6]

AGATTTTCTCCCACTCTGTGGGTTGTATGTTAACTTGGCTAATTATTTCTTTTGCTGTGCAGAAGCTTTTTAGTTTAATT

AATTCCCATCTATTTATCTT

In100 candidate2711 (in intron):

LOCUS ENSG00000141376

DESCRIPTION Breast carcinoma amplified sequence 3 (GAOB1) (Maab1 protein).

Source:Uniprot/SWISSPROT;Acc:Q9H6U6]

AGGAGGAGAAGAAAAGCTTATAGTATTTAACCATGTTTTTGCTTACTATGTTTTTTCTTCCTTCCTACTGTTCCACAGTT

ACTTCTTCATTTGTTTTCCC

In100 candidate2712 (in intron):

LOCUS ENSG00000130779

DESCRIPTION CAP-GLY domain-containing linker protein 1 (Restin) (Cytoplasmi

linker protein 170 alpha-2) (CLIP-170) (Reed-Sternberg intermediate

filametassociated

protein) (Cytoplasmic linker protein 1).

[Source:Uniprot/SWISSROT;Acc:P30622]

AGAATTTAATGGTTCATTAACAATTTAATAGATATCTTCTTCACTCATTTCATATACAGCCACATTCCTTTAGTCAATTT

TTGTGTTGTTCTCATCCATT

In100 candidate2713 (in intron):

LOCUS ENSG00000162105

DESCRIPTION SH3 and multiple ankyrin repeat domains protein 2 (Shank2).

[Sorce:Uniprot/SWISSPROT;Acc:Q9UPX8]

TCCAAATTGGGCTCTGCTCCTCTTCTAACTGATCACATCTCTGCTTTGTTCTTTTGGTTCTCCTCCCAGTTGGATCACTC

ATTTATTCCTGTGCTTATTT

In100 candidate2714 (in intron):

LOCUS ENSG00000179766

DESCRIPTION

AAGGTCTCTAACACCCCCATCAAGACCCTCTATTCTATTAACCTGTTTTATTTTCCTCACAGCATTTATCACTAAATGGT

ATTTTTTTCATTTCTTGTTT

In100 candidate2715 (in intron):

LOCUS ENSG00000198853

DESCRIPTION RUN and SH3 domain-containing protein 2.

[Source:Uniprot/SWISSPOT;Acc:Q8N2Y8]

ATCTTATCTCTTAACTTCTCATTCACTTTTCTGTTCCTTTGTTTTTTTGTGCTGTTTCTATGTGATTTCTTCAGATCGAT

CTTCCAGTTCACTAATTTCT

In100 candidate2716 (in intron):

LOCUS ENSG00000198853

DESCRIPTION RUN and SH3 domain-containing protein 2.

[Source:Uniprot/SWISSPOT;Acc:Q8N2Y8]

TTTTTATTTTAATAATTATGTTTCTAACTTCTGAACATATTTCGTTCTTTTTCAGATCTGCCTGGTTATTTCTAATAATT

CATTGCCACTCGCTTACCTT

In100 candidate2717 (in intron):

LOCUS ENSG00000010017

DESCRIPTION Ran-binding protein 9 (RanBP9) (RanBP7) (Ran-binding protein

M)(RanBPM) (BPM90) (BPM-L). [Source:Uniprot/SWISSPROT;Acc:Q96S59]

TTATTTTGATTGGGAAGTTTAACATTTTTCTTCATTTTGCCCATCTGTCTTTTTCTATGCTTATTTTCTTTCTTACAGTT

TTTACTTGATTTTCAATTCA

In100 candidate2718 (in intron):

LOCUS ENSG00000172890

DESCRIPTION Glutamine-dependent NAD(+) synthetase (EC 6.3.5.1) (NAD(+) syntase [glutamine-hydrolyzing]) (NAD(+) synthetase 1). [Source:Uniprot/SWISSPRT;Acc:Q6IA69]

TCTGTGTCTGCCTGCCTTATCTTTCATCTTCTTTCTAACCATCACTGTCTTCGTTTATTTGTATTTTTTTTCTGGCAGTT

TCATTCCCCTGAATTTGTCT

In100 candidate2719 (in intron):

LOCUS ENSG00000136492

DESCRIPTION Fanconi anemia group J protein (EC 3.6.1.-) (ATP-dependent RNA elicase BRIP1) (Protein FACJ) (BRCA1-interacting protein C-terminal helicas 1) (BRCA1-interacting protein 1) (BRCA1-associated C-terminal helicase 1).[Source:Uniprot/SWISSPROT;Acc:Q9BX63]

GTTTTTAGTATTTACCTTGGGGATTATAACTATCATCTGAATCTCTGTTCCTCCCTTTTTATATTGTTATTGTCACAGAT

TCCATCTTTATACTTTGTGT

In100 candidate2720 (in intron):

LOCUS ENSG00000108510

DESCRIPTION Thyroid hormone receptor-associated protein complex 240 kDa

comonent (Trap240) (Thyroid hormone receptor-associated protein 1) (Vitamin

D3receptor-interacting protein complex component DRIP250) (DRIP 250)

(Activatrrecruited

cofactor 250 kDa component) [Source:Uniprot/SWISSPROT;Acc:Q9UHV]

TTCTACCCATTTGTTGTATAAATTGTGCATATAAACCATGTGTAACTCTTAATACATGTCTTTGCTTTTATTCAGAAGCT

CATATTGTGTCTGTTTTTCT

In100 candidate2721 (in intron):

LOCUS ENSG00000108510

DESCRIPTION Thyroid hormone receptor-associated protein complex 240 kDa

comonent (Trap240) (Thyroid hormone receptor-associated protein 1) (Vitamin

D3receptor-interacting protein complex component DRIP250) (DRIP 250)

(Activatrrecruited

cofactor 250 kDa component) [Source:Uniprot/SWISSPROT;Acc:Q9UHV]

CACTGCTTTCTTAACAAGAATGATAAGGTTTTTGTTTGTTTTTTGTTTTAGTTATATTATTTTTAGTTTTTCTGTCAGTT

ACATTAATTTTTATTTTATT

In100 candidate2722 (in intron):

LOCUS ENSG00000168658

DESCRIPTION CDNA FLJ25975 fis, clone TST07107.

[Source:Uniprot/SPTREMBL;AccQ8N770]

TCCATATGATGTATATAATGTATCTTTTTTTCTATAACTGCTTTTAAGATTTTTCCTCTTTATTCTTGTTTTCAGCTATT

GATATGCCTTCTTTTGGTTT

In100 candidate2723 (in intron):

LOCUS ENSG00000168658

DESCRIPTION CDNA FLJ25975 fis, clone TST07107.

[Source:Uniprot/SPTREMBL;AccQ8N770]

AGTGGGTCTTGCATTCTTCGTGGCTTTAACCAATCTGTTGCTGTGTTCTCCTCTTTCCAGTATATTATTTAGTCTCTCTG

CATCTTTTCTTCTCCGTCTT

In100 candidate2724 (in intron):

LOCUS ENSG00000168658

DESCRIPTION CDNA FLJ25975 fis, clone TST07107.

[Source:Uniprot/SPTREMBL;AccQ8N770]

AGACTGTTCTCATAACCATTGCATTATGCTGGTTTTCTTCAGAGACTCATTTTATCTGTCTGGTGTATCTCCTTTAACAG

TCTTCTACATCTATTATTTT

In100 candidate2725 (in intron):

LOCUS ENSG00000179133

DESCRIPTION Uncharacterized protein C10orf67.

[Source:Uniprot/SWISSPROT;AccQ8IYJ2]

ACTCCCGCTAGTAACTTTTGAGGATGTCTCTTTCCTACATATTTGCCAATTTATTGTCTTCTTTTATACTTTCAGAAACA

TTTTATAATTTTATTCTTTC

In100 candidate2726 (in intron):

LOCUS ENSG00000179133

DESCRIPTION Uncharacterized protein C10orf67.

[Source:Uniprot/SWISSPROT;AccQ8IYJ2]

TTCCACAATGGTTGAACTAATTTGCATTCCCACTAACGTGTAAAAGTGTTCCTATTTCTCTGCAGCCTCACCAGCATCTA

TTGTTTCCTGACTTTTTAAT

In100 candidate2727 (in intron):

LOCUS ENSG00000008083

DESCRIPTION Jumonji protein (Jumonji/ARID domain-containing protein 2).

[Sorce:Uniprot/SWISSPROT;Acc:Q92833]

TTTTTCTCTTTCTCTTTTAACTCCTGACTGTTTATTTTACTCCCTCTTCTATTTGATTTTCCCCAAAGTCTGTGTCAGGT

TTAAGAACCTTCTTTCTGTC

In100 candidate2728 (in intron):

LOCUS ENSG00000120549

DESCRIPTION KIAA1217 (KIAA1217), mRNA [Source:RefSeq_dna;Acc:NM_019590]

AAATAATATGCATCTGATTTTGTCTGCAAACAAATAAAGAGAAGGCTAACCCATACTTACCTTCCTTTCATGGTTCAGAC

TTTCCTGATATTTTATGCCC

In100 candidate2729 (in intron):

LOCUS ENSG00000120549

DESCRIPTION KIAA1217 (KIAA1217), mRNA [Source:RefSeq_dna;Acc:NM_019590]

AGTCTAACATCACACTTCACATCTTAATAGTCTCTCTTTCATCTTCATTTTCTTGTTTACAGTTTCAATACTTCTGTCTC

TAGTTTTGTATTTATTTTTA

In100 candidate2730 (in intron):

LOCUS ENSG00000120549

DESCRIPTION KIAA1217 (KIAA1217), mRNA [Source:RefSeq_dna;Acc:NM_019590]

AGTGGCACCATCATAACTGTTTTTACTACATATTTTGAGTTTATTCTTTCCAGTTTTCTTTTTCCTTTCCTTAATTGCTG

TAGCAGTGATTCATTTCAGT

In100 candidate2731 (in intron):

LOCUS ENSG00000120549

DESCRIPTION KIAA1217 (KIAA1217), mRNA [Source:RefSeq_dna;Acc:NM_019590]

GTGTGTGTGTGTGTGTGTGTGTGTGTGTGTGAATAGGCTTTAACTATAAATTCACTTCCTCTTATTATTGGTTAATTCAGAC

TTTGTATTGCTTCTCATGTA

In100 candidate2732 (in intron):

LOCUS ENSG00000120549

DESCRIPTION KIAA1217 (KIAA1217), mRNA [Source:RefSeq_dna;Acc:NM_019590]

AAAGTATAACAAAGTCTCCCATATCATTGTATTTTTATCTTTTTCTCCCTGCGTTCCTAAGAATTTGTTTCACAGGTTTT

TAGTTATGATCACACCTTCT

In100 candidate2733 (in intron):

LOCUS ENSG00000120549

DESCRIPTION KIAA1217 (KIAA1217), mRNA [Source:RefSeq_dna;Acc:NM_019590]

CCTCTTCATTCTGTTTAGTGTCTGTTAACTTTTTAAAAATATTATCTCTCTTGATTTACATTTGTTTGCATCTGTCAGGT

ATGCCTTATCATTCATTTTC

In100 candidate2734 (in intron):

LOCUS ENSG00000120549

DESCRIPTION KIAA1217 (KIAA1217), mRNA [Source:RefSeq_dna;Acc:NM_019590]

TTTTCTTAAACAAAGAAGGTCTTTGGTCAAGGTCTAGGAATGCTGTGTTAACCACACAAGTTTCTTTATTGCTCCAGAAC

TACTCAACTTTTCTGGCTTT

In100 candidate2735 (in intron):

LOCUS ENSG00000137522

DESCRIPTION RING finger protein 121. [Source:Uniprot/SWISSPROT;Acc:Q9H920]

EXON

ATGATTTAAAAAATACTCCACCTACTCTGTTATAACACTTATAATGAATTGTGGTTGGTTCTTTCTCCTCAGTAGACTTG

AGTTCTTCTCATTTATTCTC

In100 candidate2736 (in intron):

LOCUS ENSG00000197415

DESCRIPTION ventricular zone expressed PH domain homolog 1

[Source:RefSeq_pptide;Acc:NP_078897]

TGTCTTTCGTTTTCTTGAAATAACGACCTTATTTTTCTTCTATTTTGCTTATATTTTTCACTTAGTTTTCATCAGTGGTT

TTATATTTGGTTTCATCTTT

In100 candidate2737 (in intron):

LOCUS ENSG00000146872

DESCRIPTION Serine/threonine-protein kinase tousled-like 2 (EC 2.7.11.1)

(Tusled- like kinase 2) (PKU-alpha). [Source:Uniprot/SWISSPROT;Acc:Q86UE8]

AGCCTTCTACATCTCAATAACTGTCAACTTTTCTCCTTCTAATTGTTACAGCTAAAATCCTTGGAGTCATTCTTGTCTTT

CTCTTCTTTTTATATTATAT

In100 candidate2738 (in intron):

LOCUS ENSG00000142552

DESCRIPTION Reticulocalbin-3 precursor (EF-hand calcium-binding protein RLP9). [Source:Uniprot/SWISSPROT;Acc:Q96D15]

GCCCCTAGCGGGCAAAAAATTTAGTCAAGGAGAACGCTAACAAGGAATTTTTTTTTTTTTTTTTTTTGAGACAGTT
TTTTTCTGTTGCTCAGGCTG

In100 candidate2739 (in intron):

LOCUS ENSG00000174891

DESCRIPTION Arginine/serine-rich coiled coil protein 1. [Source:Uniprot/SWISPROT;Acc:Q96IZ7]

TTAATGATTTTTAACAATCTTCCTGGGTGAGATTTACGCGTAATTTTTCTTTCCTGTTCTCTCTTTCCCCAGATTTAGTA
TAGTAGTCTTCTCCTTATCT

In100 candidate2740 (in intron):

LOCUS ENSG00000174891

DESCRIPTION Arginine/serine-rich coiled coil protein 1. [Source:Uniprot/SWISPROT;Acc:Q96IZ7]

AACCCTCCTTCTAGGATTCTTATTATATAGATGTTAACACTTCTACTCCTGTTCTTAATTCTATCAGTTGTTTGTCTTGT
CTTTTTGCCCCCTCCCTATG

In100 candidate2741 (in intron):

LOCUS ENSG00000174891

DESCRIPTION Arginine/serine-rich coiled coil protein 1.

[Source:Uniprot/SWISPROT;Acc:Q96IZ7]

TAACATAGTACTGAATCTGGTTTTTCTACCAGCGTGACCATTCTGTCTCCTTTTCTTCTTCTTGATTTCCAAGTGTTGTT

TACAGGAACTCAGTCTTTTT

In100 candidate2742 (in intron):

LOCUS ENSG00000174891

DESCRIPTION Arginine/serine-rich coiled coil protein 1.

[Source:Uniprot/SWISPROT;Acc:Q96IZ7]

AACAAATGGGTATAAAAAAGGCTACATAACAATCTCCTATTCATCTTGTTTTTTTTTAATTTTACTTTTTAATTTCAGCT

TCTGATCTAATCCATTTCAT

In100 candidate2743 (in intron):

LOCUS ENSG00000071073

DESCRIPTION mannosyl (alpha-1,3-)-glycoprotein beta-1,4-

Nacetylglucosaminytransferase,

isoenzyme A [Source:RefSeq_peptide;Acc:NP_036346]

CGTGAGATTGACACATAAAATTAACCATCACAAGGTCCTCTAGTGAATTTTTACTTCTATTGTACTTTTCAACTCCAGAA

TTTCCATTTCGTTCTTTCTT

In100 candidate2744 (in intron):

LOCUS ENSG00000071073

DESCRIPTION mannosyl (alpha-1,3-)-glycoprotein beta-1,4-

Nacetylglucosaminytransferase,

isoenzyme A [Source:RefSeq_peptide;Acc:NP_036346]

TTGTGGAAGCCAATATCCTGTTTCTTATCATACTTTTACCTACAAACGTTAACATCCTTTGATGATTTTTGCCTGCAGCA

ATTTTTTTAAACTTTTATTT

In100 candidate2745 (in intron):

LOCUS ENSG00000071073

DESCRIPTION mannosyl (alpha-1,3-)-glycoprotein beta-1,4-

Nacetylglucosaminytransferase,

isoenzyme A [Source:RefSeq_peptide;Acc:NP_036346]

TTTTAGTTGCTCTAACAACTAAATTCCATTTTGTTTTATATTTCCTGTCTCTTCGTTTTCTCTATTCATTGAGTCAGTTT

CCTCACATGGTCCTTTAGTT

In100 candidate2746 (in intron):

LOCUS ENSG00000071073

DESCRIPTION mannosyl (alpha-1,3-)-glycoprotein beta-1,4-

Nacetylglucosaminytransferase,

isoenzyme A [Source:RefSeq_peptide;Acc:NP_036346]

GGTTGATTTCAAGATTTCTTCTTTGTCTCTAGTTTTCTAACGTTCAATTGTGGTGTTTCTTTTCATGGGTTTCTTCAGGT

TTATCCTGTTTGGGTTTTGC

In100 candidate2747 (in intron):

LOCUS ENSG00000196872

DESCRIPTION similar to 2010300C02Rik protein (MGC42367), mRNA

[Source:RefSe_dna;Acc:NM_207362]

TAACTCTTTTCTCTTGCTGCTTTCAAGTTTTTCTCCTTATCTTTAGCTTTCACATTTTCACTCTGTGTCTGTGAGCAGTT

CTCTGTGTTTATCTTACTTG

In100 candidate2748 (in intron):

LOCUS ENSG00000135951

DESCRIPTION testis specific, 10 [Source:RefSeq_peptide;Acc:NP_079520]

GACCTTCTTTTGCCTTAGGACCTTTGTTAGAGCTGTAACTTTTGCCTGCAATGTTTTTTGCCCAGATTTTCACATGATTG

ACTTCCTTTCCTCTGAAGTT

In100 candidate2749 (in intron):

LOCUS ENSG00000135951

DESCRIPTION testis specific, 10 [Source:RefSeq_peptide;Acc:NP_079520]

CTTCATCTACTACATTTCTTATAACTGTTATTTGTAACTTTTTTCCTTGTCTTTTTTCATATTGATAATATCTAGCAGTG

TCTTTTAGTTTTTTAAATTT

In100 candidate2750 (in intron):

LOCUS ENSG00000124788

DESCRIPTION Ataxin-1 (Spinocerebellar ataxia type 1 protein).

[Source:Uniprt/SWISSPROT;Acc:P54253]

AGCCTAGGATGTCCCTCACCATCATTTATAATGCTTTTAACCTTACATTCCATTTTGTCTGATATTCAGAGTGCTATCCT

GGTTTTTGTTTTTAGTTTTG

In100 candidate2751 (in intron):

LOCUS ENSG00000124788

DESCRIPTION Ataxin-1 (Spinocerebellar ataxia type 1 protein).

[Source:Uniprt/SWISSPROT;Acc:P54253]

TCTTAGTTGTGCATTGTACATTAGAAATATTAGTTTTGAAGATAACCTAAACACAAGTTTTTATTTCTTCTGTCACAGTT

GCCTCTTTTCTTAAAATCGT

In100 candidate2752 (in intron):

LOCUS ENSG00000124788

DESCRIPTION Ataxin-1 (Spinocerebellar ataxia type 1 protein).

[Source:Uniprt/SWISSPROT;Acc:P54253]

ATGCATCTCATTTAGTCATCACCCTAACCCTGTAGGGTACGTGTTATTATCTCCATTTTCTTTATTTTTTGAGACAGGGT

CTTGTTCTGTCTCCCAGGTT

In100 candidate2753 (in intron):

LOCUS ENSG00000099256

DESCRIPTION phosphoribosyl transferase domain containing 1

[Source:RefSeq_pptide;Acc:NP_064585]

TCCTGTGTTTTTCCTCTTTCTCCCTGCCTCCCACCTAACCCCTGTTGACCACTGATCTTTTTACTGTCTCCACAGTTTTG

CCTTTTCTAGAACGTCATTT

In100 candidate2754 (in intron):

LOCUS ENSG00000112186

DESCRIPTION Adenylyl cyclase-associated protein 2 (CAP 2).

[Source:Uniprot/WISSPROT;Acc:P40123]

AGAGGAATTGTCTGCTAAATCTAGTAATTGAAATAACTTAGACTTGTACTATACTTCGTATTTCAGTTTTTTTAATTTCA

TTTTTCTAGTACTGCATTTT

In100 candidate2755 (in intron):

LOCUS ENSG00000051825

DESCRIPTION M-phase phosphoprotein 9.

[Source:Uniprot/SWISSPROT;Acc:Q99550]EXON

AAGATCTAGATCCTAACTGTGATTCTGGGTGTATGTTTTCTTCAGATTTGAAGGCTTTGTTTTATTGCCTATGACATGAT

CAATTCTTTTCCTTTCTCTC

In100 candidate2756 (in intron):

LOCUS ENSG00000173826

DESCRIPTION Potassium voltage-gated channel subfamily H member 6 (Voltagegted

potassium channel subunit Kv11.2) (Ether-a-go-go-related gene

potassiumchannel 2) (Ether-a-go-go-related protein 2) (Eag-related protein 2).

[Soure:Uniprot/SWISSPROT;Acc:Q9H252]

CTCATTTGGTCTTGAAGTGTCTCATTTTCTCAACCTTATATACGCCTAACTCCTGCACATATTCTTGCTCTGCAGATTCT

GTTTTCTTAAGTATTAATCT

In100 candidate2757 (in intron):

LOCUS ENSG00000158417

DESCRIPTION Eukaryotic translation initiation factor 5B (eIF-5B) (Translatin

initiation factor IF-2). [Source:Uniprot/SWISSPROT;Acc:O60841]

TTACATATTTTGTTAGATTTAAGTATGTTACTTTCTTTGGAATAACTATAAGTAATATGATTGTTTTTTCCCCCTCAGTT

TTTCTGTTTGTTTGTTTTTT

In100 candidate2758 (in intron):

LOCUS ENSG00000151967

DESCRIPTION schwannomin interacting protein 1

[Source:RefSeq_peptide;Acc:NP055390]

ACTCTGGTTTATTTAACAAAATATTTATCACTTTAGTAAATTTCTCATATCCCCAATTTTTTGTATTTTTTTCAGAATTC

TTTTGTATCTCACTAAGCTT

In100 candidate2759 (in intron):

LOCUS ENSG00000151025

DESCRIPTION Probable G-protein coupled receptor 158 precursor.

[Source:Unipot/SWISSPROT;Acc:Q5T848]

GGTAATTCTAGCATTGGTCTTTAGGTAATCTTTTTTAACTTTTTTTTCCTTTTTCTCCTCTTTATTTTCAAACAGCCTGT

CTTTGAGCTTGCTGCTGCTT

In100 candidate2760 (in intron):

LOCUS ENSG00000068885

DESCRIPTION Tripartite motif-containing protein 59 (Tumor suppressor TSBF-1

(RING finger protein 104). [Source:Uniprot/SWISSPROT;Acc:Q8IWR1]

TGAAATTTTAAGAATTTTTTTCTAACACCTACCTTTTCTTTATCTTTTTGAAGTTGTTTCTCCAGTTTTTTGGCTTTACT

CGTGGCATGTTTTAACTTTT

In100 candidate2761 (in intron):

LOCUS ENSG00000068885

DESCRIPTION Tripartite motif-containing protein 59 (Tumor suppressor TSBF-1

(RING finger protein 104). [Source:Uniprot/SWISSPROT;Acc:Q8IWR1]

TGCTCTGAGCCTCTTTTGAAGCATTAATGTAATATTTTACTTATTGCATAACCCATTTATTCATTTCTTTACTCTCAGCT

ATTATTTCTCCTATCCATTT

In100 candidate2762 (in intron):

LOCUS ENSG00000068885

DESCRIPTION Tripartite motif-containing protein 59 (Tumor suppressor TSBF-1 (RING finger protein 104). [Source:Uniprot/SWISSPROT;Acc:Q8IWR1]

TCTTCTTAACTTTGATGCTCTCTTTTTCTCTAATCTTTTGATGCTCTCTTTTTCTCTAATCTTTTGGAAGTCACACAGTC

TATCTGTTTACTCTTAGTTA

In100 candidate2763 (in intron):

LOCUS ENSG00000095777

DESCRIPTION Myosin IIIA (EC 2.7.11.1). [Source:Uniprot/SWISSPROT;Acc:Q8NEV4

GTCTTTTAAAAAACCAAAGATATTCCCTCCTGCTATAACTAAATCTCCTCCTATTTCTTCTTATCTGTTAAACAGCCCCA

GAGATTTCTATTATTTTCTT

In100 candidate2764 (in intron):

LOCUS ENSG00000095777

DESCRIPTION Myosin IIIA (EC 2.7.11.1). [Source:Uniprot/SWISSPROT;Acc:Q8NEV4

AATGTCTTCATTGACCCATTGGTTGTTTAGTATCATATTGGTTAACCCTTTTCCTGTTTTTTTTTTAAGTACAGCTTGC

TGCTATTGCTCATTTAATTT

In100 candidate2765 (in intron):

LOCUS ENSG00000144218

DESCRIPTION AF4/FMR2 family member 3 (Protein LAF-4) (Lymphoid nuclear protin related to AF4). [Source:Uniprot/SWISSPROT;Acc:P51826]

AAGAACAATCAACCTAAGATGCTGATATAACTTTTCCTATTTTTCTCTTTCAACATACTTTCATATTTGCAGTTCACATA

TTCCATTTTTAAGCCATCTT

In100 candidate2766 (in intron):

LOCUS ENSG00000144218

DESCRIPTION AF4/FMR2 family member 3 (Protein LAF-4) (Lymphoid nuclear protin related to AF4). [Source:Uniprot/SWISSPROT;Acc:P51826]

AGATATACTGAGAGAATGTTTCACTATGAACATAACATTTAAAGCACTCAACTTTCTATCCTTAACATTTTTACAGCCTC

ATTTCATTTGAGTCATTTTT

In100 candidate2767 (in intron):

LOCUS ENSG00000188026

DESCRIPTION CDNA FLJ39378 fis, clone PERIC2000214.

[Source:Uniprot/SPTREMBLAcc:Q8N1M0]

GTGGGATCCTACAACATGTGGTCCTGCCTGGCTTCTTTAACTTAGCATGTTTTCTTGTTAATCCAGGTTCTCATATTTCA

ACTCAAGGTTTCCTGCCTTT

In100 candidate2768 (in intron):

LOCUS ENSG00000186635

DESCRIPTION Centaurin-delta 2 (Cnt-d2) (Arf-GAP, Rho-GAP, ankyrin repeat an pleckstrin homology domain-containing protein 1).

[Source:Uniprot/SWISSPRO;Acc:Q96P48]

GATTGTGTACATTTATAAAGTAACTTAGACATCATTAATCTCTTTATGTTGTCTTCTATCCAGAACTTACTGTGCTCTGC

CATTTGTCCAGGTCTTCTTT

In100 candidate2769 (in intron):

LOCUS ENSG00000163590

DESCRIPTION Protein phosphatase 2C isoform epsilon (EC 3.1.3.16) (PP2Cepsion) (Protein phosphatase 1L) (Protein phosphatase 1-like). [Source:Uniprot/WISSPROT;Acc:Q5SGD2]

GGCCAATCACCCCATCTCTCAAATTGGCGGTTTGTAACGGGTTCATTGCTGCATACTCTTTGTTTTCCCTTTTGGCAGCA
TACTTTTCTTATTCTTAACC

In100 candidate2770 (in intron):

LOCUS ENSG00000137478

DESCRIPTION FCH and double SH3 domains protein 2 (SH3 multiple domains 3) (arom). [Source:Uniprot/SWISSPROT;Acc:O94868]

AATTCCATTTAACTCTCATTTATGACTTTTTGGCTGTTAGACCTTTTGACTCTGTTCATGTCTTTTCTACTTTTTCAGCA
TTTTGTTTTCCACCCTTTTT

In100 candidate2771 (in intron):

LOCUS ENSG00000090402

DESCRIPTION Sucrase-isomaltase, intestinal [Contains: Sucrase (EC 3.2.1.48) Isomaltase (EC 3.2.1.10)]. [Source:Uniprot/SWISSPROT;Acc:P14410]

TGCTTTATTAATTTATCATCTACCATAATCCCTTCTTTATTTCTGATAACATTTTATTGATCTCCTTTTTATTTACAGAC
TTTACCCCTTTTTGTTATTC

In100 candidate2772 (in intron):

LOCUS ENSG00000071054

DESCRIPTION Mitogen-activated protein kinase kinase kinase kinase 4 (EC 2.711.1) (MAPK/ERK kinase kinase kinase 4) (MEK kinase kinase 4) (MEKKK 4)

(HP/GCK-like kinase HGK) (Nck-interacting kinase).

[Source:Uniprot/SWISSPROT;Ac:O95819]

ACCCTAACCTATTTTTCCATTCTTTTTCACTTTTAATACTAGCCTCAGAAAATAACTCATTATTCTCTAACACTTCCCTA

CTTTTGTTCATATCATTCCT

In100 candidate2773 (in intron):

LOCUS ENSG00000071054

DESCRIPTION Mitogen-activated protein kinase kinase kinase kinase 4 (EC

2.711.1) (MAPK/ERK kinase kinase kinase 4) (MEK kinase kinase 4) (MEKKK 4)

(HP/GCK-like kinase HGK) (Nck-interacting kinase).

[Source:Uniprot/SWISSPROT;Ac:O95819]

TGATGGTGGCTTAGATTCCCTGGCTGGAAGTGCTTCATGACCATGGTAACCATTCCCTCTCTTTTCTTGCTTTTGCAGGT

GTTCTTTGCCTCTGTTCGGT

In100 candidate2774 (in intron):

LOCUS ENSG00000175582

DESCRIPTION Ras-related protein Rab-6A (Rab-6).

[Source:Uniprot/SWISSPROT;Ac:P20340]

CTTAAGCCTTATATTCTGCCTCTCTCAAATTTAACCTCACCCCAATTTATCCTTTTGTGTTTCATATCCTTAACACAGTA

GGACTTTATTTTTTATTTTT

In100 candidate2775 (in intron):

LOCUS ENSG00000174776

DESCRIPTION WD repeat protein 49. [Source:Uniprot/SWISSPROT;Acc:Q8IV35]

TTGTCCTTTTAACTTGTCCAAAAATAAACTTTTTGTTTCATTAATCTTCTGTATTTTTTTCAATTTTATTTATTTCAGCT

CTGATCTTTATTATTTCTTT

In100 candidate2776 (in intron):

LOCUS ENSG00000136754

DESCRIPTION Abl interactor 1 (Abelson interactor 1) (Abi-1) (Spectrin SH3

dmain- binding protein 1) (Eps8 SH3 domain-binding protein) (Eps8-binding

prtein) (e3B1) (Nap1-binding protein) (Nap1BP) (Abl-binding protein 4) (AblBP).

[Source:Uniprot/SWISSPROT;Acc:Q8IZP0]

TGTGTTGGCAACTGTTAGTAACTGTAAACGTGTTTTAACCTGTCTGTGTACTATTACTTTTTCAGGTAGTTGTATGTTGT

AATGCTTTTTTTTTACCCCT

In100 candidate2777 (in intron):

LOCUS ENSG00000136754

DESCRIPTION Abl interactor 1 (Abelson interactor 1) (Abi-1) (Spectrin SH3

dmain- binding protein 1) (Eps8 SH3 domain-binding protein) (Eps8-binding

prtein) (e3B1) (Nap1-binding protein) (Nap1BP) (Abl-binding protein 4) (AblBP).

[Source:Uniprot/SWISSPROT;Acc:Q8IZP0]

TTCTTTAACATAAAGTTTTTATGGGTATCATTTTATCTGTAGTATCTTCTTCCCTTTCTTCTCAGCCACTTTGTTCATTT

ATGTTAATACATTTGCCTTC

In100 candidate2778 (in intron):

LOCUS ENSG00000145996

DESCRIPTION CDK5 regulatory subunit associated protein 1-like 1

[Source:Refeq_peptide;Acc:NP_060244]

AACTCCCCATTCCTCCTTTCTTCCAATCCCTGGTAACGACGATTCTACTTTCTGCCTCTCTGAATTTGACTGTTCCAGAT

TCCTTTATATCAGTCGCCTT

In100 candidate2779 (in intron):

LOCUS ENSG00000145996

DESCRIPTION CDK5 regulatory subunit associated protein 1-like 1

[Source:Refeq_peptide;Acc:NP_060244]

TCTGGTTATAACTTTCTGTCCTTTTTCTTTTCTCCTTCCTTTTTTTCCCCTTCATTTTTTTCTTTCTCCAACCCCCAGCC

ACTTCATTTCTTCTTTTCTG

In100 candidate2780 (in intron):

LOCUS ENSG00000145996

DESCRIPTION CDK5 regulatory subunit associated protein 1-like 1

[Source:Refeq_peptide;Acc:NP_060244]

ATGAAGATTGTTATTAACATTTTACCATCTTTATTATGTCATATCTTTCTTCATCCATTTCTATTCATCCATCAGTCCAA

CTTATTTTTTTAACATGTTT

In100 candidate2781 (in intron):

LOCUS ENSG00000107890

DESCRIPTION Ankyrin repeat domain-containing protein 26.

[Source:Uniprot/SWSSPROT;Acc:Q9UPS8]

CACTTGGAACATAATCTTACATTCAAATCATAACTGCATGTTTTATTAAACCTATATTCCTGTTTTTTTCTCCAGTATAC

TTCTGTCATGTTGCTGTCCT

In100 candidate2782 (in intron):

LOCUS ENSG00000107890

DESCRIPTION Ankyrin repeat domain-containing protein 26.

[Source:Uniprot/SWSSPROT;Acc:Q9UPS8]

ACCTCTGATGCCTTGGGGACAATGTTAGGAGCTAACTGCCTTTGAGACTCTTTAGTTCTTTTTGATCACCAACTCAGCTA

TCTTCTTTTCCCCTACAATT

In100 candidate2783 (in intron):

LOCUS ENSG00000165434

DESCRIPTION Phosphoglucomutase-2-like 1 (EC 5.4.2.2) (PMMLP).

[Source:Uniprt/SWISSPROT;Acc:Q6PCE3]

CAAAGACATTTTAATGTCTACCTTGTTAGAAAACCTCTTTAACGTAAGTAATGGTTTTATGTTGTGTTTCTGCTTCAGAA

TTTTTTCACGTCATTTTCTC

In100 candidate2784 (in intron):

LOCUS ENSG00000114248

DESCRIPTION Leucine-rich repeat-containing protein 31.

[Source:Uniprot/SWISPROT;Acc:Q6UY01]

AGAAAAATGTAGGGAAAATACTGAATTTCCTTTCATTATAACACAAACGATCATTTCTTTAAGATTCTTTTCAGTCTTTT

CTTTTCATATGCATGCATTT

In100 candidate2785 (in intron):

LOCUS ENSG00000115616

DESCRIPTION Sodium/hydrogen exchanger 2 (Na(+)/H(+) exchanger 2) (NHE-2)

(Slute carrier family 9 member 2). [Source:Uniprot/SWISSPROT;Acc:Q9UBY0]

AGTTTTTTAAATTGTTTTTGATATTCTGCTATTTTTTAACGTGTCTAAGTATAGATTTACTTTATTTTTCTTGCTCAGAA

CTTGTTATACTTCCATGCTT

In100 candidate2786 (in intron):

LOCUS ENSG00000115616

DESCRIPTION Sodium/hydrogen exchanger 2 (Na(+)/H(+) exchanger 2) (NHE-2)

(Slute carrier family 9 member 2). [Source:Uniprot/SWISSPROT;Acc:Q9UBY0]

GGTATGTGTTTGCTATTTCTATCTGCCTTTTCTGATAACACTGGTTAAATTATGCTTTCTTTCCTTATTGTTGTACAGCT

TCTTACTTTATATGACATTC

In100 candidate2787 (in intron):

LOCUS ENSG00000166240

DESCRIPTION

AGCACCCCATCTTTCTAACTCATCTCATTCTCTCTGTCTAGCTTTGTTTAAACATGTGTGTTTTGTCTTCTGTCTCAGTC

TGTTTCTCTGTGTTGCATGT

In100 candidate2788 (in intron):

LOCUS ENSG00000135972

DESCRIPTION 28S ribosomal protein S9, mitochondrial precursor (S9mt) (MRP-S).

[Source:Uniprot/SWISSPROT;Acc:P82933]

AGTGATTTTTTTTTCTTCTTCTTCTTTACTAACTCTTCTATTTTTTTAAACCTCTCAGGCTCCTCACCTATTTCTCTCTC

TCTTTGTTCTTTATATCTCT

In100 candidate2789 (in intron):

LOCUS ENSG00000163558

DESCRIPTION Protein kinase C iota type (EC 2.7.11.13) (nPKC-iota)

(Atypicalprotein kinase C-lambda/iota) (aPKC-lambda/iota) (PRKC-lambda/iota).

[Sourc:Uniprot/SWISSPROT;Acc:P41743]

GTTAACTCTATCTTTTTTGCTCCACTTGATTCTCTCTTATAGTTTCCAGTTAGCCATATTTCAAGTCTATGTGTTGACTT

ACTTTCTTACACTTCTCTTC

In100 candidate2790 (in intron):

LOCUS ENSG00000166435

DESCRIPTION X-ray radiation resistance associated 1 protein (Fragment).

[Sorce:Uniprot/SPTREMBL;Acc:Q7RTT7]

AGGCTTGTTTTAACTAATTAAAGGCCTTTTGAGCTTCAGGTTCTCAGGTTAGGAAATGAGTTTTAGCCACTTGAGTTTCT

TTCTTTTTTTTTTTTTTTTT

In100 candidate2791 (in intron):

LOCUS ENSG00000151948

DESCRIPTION glycosyltransferase 1 domain containing 1

[Source:RefSeq_peptid;Acc:NP_653270]

TAATAAATAAATATATTAAAAATTATATATATATATATAACATGGACTCATAGATATTTATTTTATTTCCCTTTACAGAT

TATCTGTTACTATTTTTGTT

In100 candidate2792 (in intron):

LOCUS ENSG00000151952

DESCRIPTION transmembrane protein 132D (TMEM132D), mRNA

[Source:RefSeq_dna;cc:NM_133448]

TTCCTATTTATTTCTGCATCTAATGGACTATGGGAAGTAACATTGAATCTTGATCCTTTTTGTTTCTATGATTATCAGGT

TACTTGCCTTTTATAGTTCC

In100 candidate2793 (in intron):

LOCUS ENSG00000151952

DESCRIPTION transmembrane protein 132D (TMEM132D), mRNA

[Source:RefSeq_dna;cc:NM_133448]

TTTAACTATTTTCTATTAATTTCATGTGTTCTTTGTTCTGTATCTGCCCCTTTTTCTGCCTGATTCCAGTATCTTATGTG

ATTACATTTGCTATCCTCTC

In100 candidate2794 (in intron):

LOCUS ENSG00000163581

DESCRIPTION Solute carrier family 2, facilitated glucose transporter member2

(Glucose transporter type 2, liver). [Source:Uniprot/SWISSPROT;Acc:P11168

AAACTGCTAACTCAAAGTCTGTGTTTGCTTTCTATCCAGGACTTCTGTGGACCTTATGTGTTTTTCCTCTTTGCTGGAGT

GCTCCTGGCCTTTACCCTGT

In100 candidate2795 (in intron):

LOCUS ENSG00000122707

DESCRIPTION Reversion-inducing cysteine-rich protein with Kazal motifs precrsor (hRECK) (Suppressor of tumorigenicity 15) (ST15). [Source:Uniprot/SWISPROT;Acc:O95980]

TCTTAACTACTTCTAGGTTATAGTTTTTATTTTTTTTCTTTTCTCAAAGCCTGTATCTCTTATTTTTAAACTTGACAGTC

TTATTTATCTTTTGTTCCTT

In100 candidate2796 (in intron):

LOCUS ENSG00000068308

DESCRIPTION OTU domain-containing protein 5. [Source:Uniprot/SWISSPROT;Acc:96G74]

TGGAAATCTTTAAGGAGGATCTGTGTCTCATCCTTAACTCATCCTTAATTTATTAATTTTTCCAGTTATTACGTACATCA

ATATGGTCTTTTTTTTTTTT

In100 candidate2797 (in intron):

LOCUS ENSG00000111452

DESCRIPTION Probable G-protein coupled receptor 133 precursor (G-protein copled receptor PGR25). [Source:Uniprot/SWISSPROT;Acc:Q6QNK2]

TTTCTGTCTTTACCTATTTTGGAGATTCCACATAAATGGAAATGTGTAACGTATAACCTTTTTGTCTTAATCTCTCAGAT

TTTTTTTTTGCTCTGTTAAA

In100 candidate2798 (in intron):

LOCUS ENSG00000108854

DESCRIPTION Smad ubiquitination regulatory factor 2 (EC 6.3.2.-) (Ubiquitinprotein

ligase SMURF2) (Smad-specific E3 ubiquitin ligase 2) (hSMURF2).

[ource:Uniprot/SWISSPROT;Acc:Q9HAU4]

GTTGCTGAAATATTTCATTTTTATTAAAATTTTCCTGGGTAGTGCCTAACGAGTATATATACTGTTTTCAATTTCAGGAA

CATTTATCCTATTTCCACTT

In100 candidate2799 (in intron):

LOCUS ENSG00000176809

DESCRIPTION Leucine-rich repeat-containing protein 37A.

[Source:Uniprot/SWISPROT;Acc:O60309]

GTGGCTGTTTAAATTTGTAGTGGTTGAAAAGTCTTGAGATTGTCACACCTAACAACTCTTATACTTTTCTTCTCTCAGCT

GCAGCTTGTTCTTTTCACCT

In100 candidate2800 (in intron):

LOCUS ENSG00000198382

DESCRIPTION UV radiation resistance-associated gene protein (p63).

[Source:niprot/SWISSPROT;Acc:Q9P2Y5]

CTTAACTGTTGATAGCGTCCTTCTTACATTAGCAGTTTGTCTGAGCTTTTCTGGTGAGATACTTCTTGGTTTTCCTCATA

TCTTTTAACCAGTCCTTTTC

In100 candidate2801 (in intron):

LOCUS ENSG00000159921

DESCRIPTION Bifunctional UDP-N-acetylglucosamine 2-epimerase/Nacetylmannosmine

kinase (UDP-GlcNAc-2-epimerase/ManAc kinase) [Includes: UDP-N

acetylgucosamine 2-epimerase (EC 5.1.3.14) (Uridine diphosphate-

Nacetylglucosamie-

2-epimerase) (UDP-GlcNAc-2-epimerase)

[Source:Uniprot/SWISSPROT;Acc:Q9Y22]

CGTGGATGTTCTTTAACATTTCTGCTGCTGGCTCTTTTCTGCCTTCCTCTTTGGTATTTTGATTTTGGCTTCTCTCAGAA

ACTCATTTTAATTTTTGTCT

In100 candidate2802 (in intron):

LOCUS ENSG00000112293

DESCRIPTION Phosphatidylinositol-glycan-specific phospholipase D precursor EC

3.1.4.50) (PI-G PLD) (Glycoprotein phospholipase D)

(Glycosylphosphatiylinositol-

specific phospholipase D).

[Source:Uniprot/SWISSPROT;Acc:P80108]EXON

CTGCCTGTTCTCCAAAATTGTTTGCTGGCCCTTGCCTTAGAAAATAACCATTATTAGTATTTTGCTATTTTCTTCCAGAC

TTTCAGAGTTTTTTCTTTCT

In100 candidate2803 (in intron):

LOCUS ENSG00000075420

DESCRIPTION fibronectin type III domain containing 3B

[Source:RefSeq_peptid;Acc:NP_073600]

AACTTAACATTTTCTTAAATATCTTCTAGCACTTTATCTGTACTTTTCAGAAGACTTGACTTTTTCATTGTTGTCTAATA

GTCTCCTTGTCTCCCTTATT

In100 candidate2804 (in intron):

LOCUS ENSG00000075420

DESCRIPTION fibronectin type III domain containing 3B

[Source:RefSeq_peptid;Acc:NP_073600]

TAAATTGAAAAGACATAGAAGATTTTAACTACATATGAAGTACTATTTTAAGTCATTTGAGATTTTTCTCTCCTCCAGAA

TTTTTTAATCCTGTCATACT

In100 candidate2805 (in intron):

LOCUS ENSG00000075420

DESCRIPTION fibronectin type III domain containing 3B

[Source:RefSeq_peptid;Acc:NP_073600]

GTGGGCTAGTGACAAGAATCACACTTTTATAACAAGTTTAGAAAATGTCTTTCCAACTTCATTGTCATTTTGCTTCAGGT

TTTTGTTACTATTCATACTG

In100 candidate2806 (in intron):

LOCUS ENSG00000177192

DESCRIPTION tRNA pseudouridine synthase A (EC 5.4.99.12) (tRNA-uridine

isomrase I) (tRNA pseudouridylate synthase I).

[Source:Uniprot/SWISSPROT;Acc:Q9606]

TGATAGAGTACCATCATCTAACATGTCCAAAGTAGGAAACTCTCTTTTCTGTTTTCTTTTTCTTTTCTTCAGCTTCTTTG

ATGTGGCATGCCTTTTATTT

In100 candidate2807 (in intron):

LOCUS ENSG00000179240

DESCRIPTION

AGATGATATCTTATTGTAGCAATGCACATTCTGATGTCAAGAAAACTGTAACACTTAACTGAACTTTGTTGTTTGCAGGA

TGTTTTTCTTCTCTACTAGC

In100 candidate2808 (in intron):

LOCUS ENSG00000154240

DESCRIPTION Coiled-coil domain-containing protein 46.

[Source:Uniprot/SWISSROT;Acc:Q8N8E3]

TCTTTAGGTACACTAACTAGGTAAATGCTGGATCTCCATTTTGTTTTCTTCTATGTTGGTTGTTTGCTCTTACAGTTTTT

ACTTGCTCATAATGTTATTT

In100 candidate2809 (in intron):

LOCUS ENSG00000154240

DESCRIPTION Coiled-coil domain-containing protein 46.

[Source:Uniprot/SWISSROT;Acc:Q8N8E3]

TAAAAATCCCATGGCGCGCTATAACTTGGCCCCCTTTTTTATGATCTTGTCTCAGTTCCTTTCATGATGGATTTTTTTAA

GGTATCTGCCCCTTACCCCT

In100 candidate2810 (in intron):

LOCUS ENSG00000154240

DESCRIPTION Coiled-coil domain-containing protein 46.

[Source:Uniprot/SWISSROT;Acc:Q8N8E3]

AGTTCATGTGAACTCATAACATGTTTTAATTCTCCTTTTGAAGTTTTCTCCCCTTTCAGATAAGCACATACACTGATTGC

TTTTTTTTCTTTGTATTTTC

In100 candidate2811 (in intron):

LOCUS ENSG00000154240

DESCRIPTION Coiled-coil domain-containing protein 46.

[Source:Uniprot/SWISSROT;Acc:Q8N8E3]

TCTATTCTTTGGCTTTAGCTAACTTGCTCTTTTTCCTATCATTTTCACTTTTTCCTTCTTCTATGACATTAATTGCAGCT

TCATGTCTGTAACTTCTTCC

In100 candidate2812 (in intron):

LOCUS ENSG00000154240

DESCRIPTION Coiled-coil domain-containing protein 46.

[Source:Uniprot/SWISSROT;Acc:Q8N8E3]

CTCACTAATTCTCTCCTTAGCTGTGTCTAACCTACTGTTAAACTCATTCTTAATTTTGCTTATTATATTTTCCAGAGCTA

GACTTTCTACTTAGTTCTTT

In100 candidate2813 (in intron):

LOCUS ENSG00000183495

DESCRIPTION E1A-binding protein p400 (EC 3.6.1.-) (p400 kDa SWI2/SNF2-relatd

protein) (Domino homolog) (hDomino) (CAG repeat protein 32) (Trinucleotid

repeat-containing gene 12 protein). [Source:Uniprot/SWISSPROT;Acc:Q96L91]

EXON

TATACTTTTTAACATTTTTGCTTTTTGTTTCTTCATTTGATATTTTGTATTTTTATATATCATCTTCATCAGTTATTTCC

TATCAATGGTGTTCACTTTT

In100 candidate2814 (in intron):

LOCUS ENSG00000158636

DESCRIPTION Protein EMSY. [Source:Uniprot/SWISSPROT;Acc:Q7Z589]

CTGAATTTTTAAGTCTACTTACTATAACGCTTTTAACTGCCTTTGTTGCTGGATCTTATTTTCTCAGTAATTCTTCTCTC

TTCCCTGCTTCTTGTCTAGT

In100 candidate2815 (in intron):

LOCUS ENSG00000114346

DESCRIPTION ECT2 protein (Epithelial cell-transforming sequence 2 oncogene)

[Source:Uniprot/SWISSPROT;Acc:Q9H8V3]

AGTTGAGTTAATATCTAGTTAACTAGTCTAGCTATTTGGCTTTGTTCTCAGAATGCTATAATAGTTTTCTTTTTTCTCCC

TTTTCCATTCCCCTAACTTT

In100 candidate2816 (in intron):

LOCUS ENSG00000078124

DESCRIPTION Alkaline phytoceramidase (EC 3.5.1.-) (aPHC) (Alkaline ceramidae)

(Alkaline dihydroceramidase SB89). [Source:Uniprot/SWISSPROT;Acc:Q9NUN7]EXON

ATTCTACCATTAACATTTTATTACACTTGTTTTTATCACATACCGATTCATTTTTCCATTCCTTTCTCCATCCATCAGTT

CATTTTATTTTTTGATACAT

In100 candidate2817 (in intron):

LOCUS ENSG00000078124

DESCRIPTION Alkaline phytoceramidase (EC 3.5.1.-) (aPHC) (Alkaline ceramidae)

(Alkaline dihydroceramidase SB89). [Source:Uniprot/SWISSPROT;Acc:Q9NUN7]EXON

ACAATTAGTATAGACTAACACTCATGATTGTATTTCTTCTGTTTTCTTTCTTTTCCTTTGCTTTTTCAGCATGAAGGTTG

TATTTCTTGTTTTCTTTTTT

In100 candidate2818 (in intron):

LOCUS ENSG00000078124

DESCRIPTION Alkaline phytoceramidase (EC 3.5.1.-) (aPHC) (Alkaline ceramidae)

(Alkaline dihydroceramidase SB89). [Source:Uniprot/SWISSPROT;Acc:Q9NUN7]EXON

AGCTTTTTAAAAGTAACCTTTTATTCTTTTTTCCTGACTTTACAGTCTTACCTGATTTATCAATTTTTTGCCTTTGTGGT

TAATCCTTTTCATGTCCTAC

In100 candidate2819 (in intron):

LOCUS ENSG00000144962

DESCRIPTION spermatogenesis associated 16

[Source:RefSeq_peptide;Acc:NP_11461]

GGTGTAATATAATGCAAATTAACCAACAAGCATGTTCCATGTGCTTCTTCATCTTCATTCTTGTAGTTACAGCCTTCATC

CAAATTTTGACTATTTACTT

In100 candidate2820 (in intron):

LOCUS ENSG00000144962

DESCRIPTION spermatogenesis associated 16

[Source:RefSeq_peptide;Acc:NP_11461]

ATAGCATTCCACTTCCCAACTCCATAACTTTGCCTATGCTGTTCACCATATCTGGCTCCCTGTTGTTTTTTTCTTCAGCC

TTGCTTGTGTCCCCTTTTTA

In100 candidate2821 (in intron):

LOCUS ENSG00000015568

DESCRIPTION Ran-binding protein 2-like 2 (RanBP2L2).

[Source:Uniprot/SWISSPOT;Acc:Q53T03]

CAAGTTAGATACTTTGAAGCACTCTTTAAAGATTTTCTTTAATAACTTGAAGGCACTGTTACACCTTTCCTGTATCAGAT

TTTTTTTTTTTTGGAATTGA

In100 candidate2822 (in intron):

LOCUS ENSG00000196092

DESCRIPTION Paired box protein Pax-5 (B-cell-specific transcription

factor)(BSAP). [Source:Uniprot/SWISSPROT;Acc:Q02548]

CTAACTGCGGCGTGTTTGTTATGTTTTTTCACCTATCTGTTCACACACTTCTGCATCAGTCCAAATATCTGTGAGTTTGT

CTGTGTTTTTCTCTGTTTCT

In100 candidate2823 (in intron):

LOCUS ENSG00000150054

DESCRIPTION palmitoylated membrane protein 7

[Source:RefSeq_peptide;Acc:NP_75767]

TCCTCCCAATCCCACTTGTTTTTTCCTCTTAACCATTTCTTTGCCCCCTTTTCTTGTTGGGTTATTCATCAGTCTTCTGT

TAATTTGATGAGCTCTTTTT

In100 candidate2824 (in intron):

LOCUS ENSG00000147905

DESCRIPTION Zinc finger CCHC domain-containing protein 7.

[Source:Uniprot/SISSPROT;Acc:Q8N3Z6]

TTTAAATGTTTAATAATATAGCTAAAATATAACTTCTTGTATTTTCATTTCTGCTACATTTTCAGATTTTTGTAGAGCCT

GACTTTTAATGTTCTTTTTC

In100 candidate2825 (in intron):

LOCUS ENSG00000147905

DESCRIPTION Zinc finger CCHC domain-containing protein 7.

[Source:Uniprot/SISSPROT;Acc:Q8N3Z6]

TTTTGGGTTGTGTCTTAACTTTGTTTATACAATATCAATTTTGTTTTCTTGGATCTCTCACATTTCCTTTGTTTACAGTT

GTACATTTCCTCCCTGTTTT

In100 candidate2826 (in intron):

LOCUS ENSG00000171634

DESCRIPTION bromodomain PHD finger transcription factor isoform 2

[Source:RfSeq_peptide;Acc:NP_004450]

AGTTTTGTGTCTCTATTCCATTGAGATTGTTGTAACTTATTTAAGCATGACTATTTCTTCTCAGTTTCTCTTTTTTATAT

TTTATTTTCATTTGTATGTT

In100 candidate2827 (in intron):

LOCUS ENSG00000095787

DESCRIPTION WW domain-containing adapter protein with coiled-coil.

[Source:niprot/SWISSPROT;Acc:Q9BTA9]

TTAAGTAATTTGTTAAAGTCAAGTAACTGTCTCTTCAATTTTCTGTTTTTGTGATCATTGTCTTGACAGTTTAGTTTATA

AATCGTCTCAGTTTTCTCTT

In100 candidate2828 (in intron):

LOCUS ENSG00000095787

DESCRIPTION WW domain-containing adapter protein with coiled-coil.

[Source:niprot/SWISSPROT;Acc:Q9BTA9]

TCACCTTTGTACATTTAACACTTTTTAACTCCTTTTTGAAATTCTTTCTCTGGCTTCTGTGATACTACACTCCTTCAGTT

GCCTATTTCTGTTCCTTCCT

In100 candidate2829 (in intron):

LOCUS ENSG00000184538

DESCRIPTION RANBP2-like and GRIP domain-containing protein 7 (RANBPL1 isofom

2). [Source:Uniprot/SWISSPROT;Acc:Q9H0B2]

CAAGTTAGATACTTTGAAGCACTCTTTAAAGATTTTCTTTAATAACTTGAAGGCACTGTTACACCTTTCCTGTATCAGAT

TTTTTTTTTTTGGAATTGA

In100 candidate2830 (in intron):

LOCUS ENSG00000149262

DESCRIPTION Integrator complex subunit 4 (Int4).

[Source:Uniprot/SWISSPROT;cc:Q96HW7]

GTGCTTTCTTGCCTTGACTTAACTCTTTTTGAGGACTGCTTGGTCTCCTTTACAGCTCCTGATTGTCGTTTATAACACCT

AGTCATTTTCCATTGTCTTT

In100 candidate2831 (in intron):

LOCUS ENSG00000141337

DESCRIPTION Arylsulfatase G precursor (EC 3.1.6.-) (ASG).

[Source:Uniprot/SISSPROT;Acc:Q96EG1]

TTCCAATCCATGAACATGGGATGTAACCATTTGTTTATGTCTTCTTTAATGTCTTTCAGCTATATTTTATGGTTTTCATT

ATGCAAGTCTTTCACTTTTT

In100 candidate2832 (in intron):

LOCUS ENSG00000169760

DESCRIPTION Neuroligin-1 precursor. [Source:Uniprot/SWISSPROT;Acc:Q8N2Q7]

TATTATGATACCTGCAAGGAACCAAAACAATGCTGTCTTAGGAACTGGTAACTTTGATCTCTGAACTCTTTTCTACAGAT

ATATATTTGTGTTCCTCTTA

In100 candidate2833 (in intron):

LOCUS ENSG00000169760

DESCRIPTION Neuroligin-1 precursor. [Source:Uniprot/SWISSPROT;Acc:Q8N2Q7]

CTTCTATGACTGCTTCATAATAATGTATGGGGTATGTAATATGTGCTAACCACTGTGTTGAGTGTTTTTATTCTGCAGTT

TTTTTGTTTTGCTTTTAGTT

In100 candidate2834 (in intron):

LOCUS ENSG00000169760

DESCRIPTION Neuroligin-1 precursor. [Source:Uniprot/SWISSPROT;Acc:Q8N2Q7]

CTGAGAAAAGTCATGAAGTGTAATAAATTAGCTGCATTACTGACTTTTTAACAACAAAGATATGGGTCTTTTCTTCAGCC

AGGGTCAGTTTTTTCTCTCT

In100 candidate2835 (in intron):

LOCUS ENSG00000169760

DESCRIPTION Neuroligin-1 precursor. [Source:Uniprot/SWISSPROT;Acc:Q8N2Q7]

GATATAGTTCAAAAAGGATAAGGAGCTTAATTCCTTTCTCATAAATAACTGTGCCACTGAGTATATCTCTTTACTCAGCT

ATTGCTCCTCTCTTGCTTTT

In100 candidate2836 (in intron):

LOCUS ENSG00000070601

DESCRIPTION FERM and PDZ domain containing 1

[Source:RefSeq_peptide;Acc:NP_55722]

GATAAATTAGACTGTGTCTAACTGTATTATACTGGGTTTTCTATCATTTATTTGTTATTTTATTCATTCATTCTGCAGAT

TTTTTGTACCTTGTATTTGC

In100 candidate2837 (in intron):

LOCUS ENSG00000177565

DESCRIPTION F-box-like/WD repeat protein TBL1XR1 (Transducin beta-like 1Xrlated

protein 1) (Nuclear receptor corepressor/HDAC3 complex subunit

TBLR1)(TBL1-related protein 1). [Source:Uniprot/SWISSPROT;Acc:Q9BZK7]

TAAATATTTTAAAAATATAGTTATTTGAAATTTCATGTAACCTCCATAGTAAGTTCCCATTAAATCTTTTCTTTCCAGTC

TGTTTCCTTTATGAGCTTAC

In100 candidate2838 (in intron):

LOCUS ENSG00000172965

DESCRIPTION LOC541471 protein (Hypothetical protein MGC4677).

[Source:Uniprt/SPTREMBL;Acc:Q96C48]

TGTGTCGTTTATATTATGGAACTATTTAACTTTTAAGTAGTTATTATTTTCCTTTTAACACCCAGGTTTTGTCTTGCTTA

GAAAGTCTGTTCCTACCCCT

In100 candidate2839 (in intron):

LOCUS ENSG00000108950

DESCRIPTION Protein FAM20A precursor.

[Source:Uniprot/SWISSPROT;Acc:Q96MK3]EXON

CTTCATCCTTGGACATCTGGATCTGCACTACCTCTGGTTCTGCTAACCATTCCCCTCTCTTCTGTTTATACCTGCAGAAG

AATGTCTATTTCTTCCCTCT

In100 candidate2840 (in intron):

LOCUS ENSG00000141338

DESCRIPTION ATP-binding cassette sub-family A member 8.

[Source:Uniprot/SWISPROT;Acc:O94911]

GAACATAATAAACTCTCTATTTCTGATACTGCACTTTTTCATTCTAACATTTTCATTTGTTCTTTTTTAGAGTTTCAGTT

CTTTACTATTTCTCATCTGC

In100 candidate2841 (in intron):

LOCUS ENSG00000121879

DESCRIPTION Phosphatidylinositol-4,5-bisphosphate 3-kinase catalytic subuni

alpha isoform (EC 2.7.1.153) (PI3-kinase p110 subunit alpha) (PtdIns-3- kiase

p110) (PI3K). [Source:Uniprot/SWISSPROT;Acc:P42336]

CCTTTCTTTTTATTCTGCCTTCTGGAAAATTGCCTTAACTTTAACTTCAAACATTTGTTGATTTTTTTTTTTTTTCAGTT

CTTATTCTCTGGTCTTATTG

In100 candidate2842 (in intron):

LOCUS ENSG00000121879

DESCRIPTION Phosphatidylinositol-4,5-bisphosphate 3-kinase catalytic subuni alpha isoform (EC 2.7.1.153) (PI3-kinase p110 subunit alpha) (PtdIns-3- kiase p110) (PI3K). [Source:Uniprot/SWISSPROT;Acc:P42336]

GGTTGTCTGTTAACTTTGATGATTCTTTCTTTTGTTATGCAGAGGCTTTTTAGTTTAATTAGGTCCCATTTATTTTGTTT

GTTTTTGTCGCATTTGCTTT

In100 candidate2843 (in intron):

LOCUS ENSG00000137513

DESCRIPTION Probable asparaginyl-tRNA synthetase, mitochondrial precursor (C 6.1.1.22) (Asparagine--tRNA ligase) (AsnRS).

[Source:Uniprot/SWISSPROT;Ac:Q96I59]

CCTTAATTCCCAAGGATTAACACAAATGGAGGTTTTTTTCCATGCTCTTTCCATTGAGAAATTTGTGTCCTCGCTCAGAT

TTAGGTATCTCCCATATTTC

In100 candidate2844 (in intron):

LOCUS ENSG00000137513

DESCRIPTION Probable asparaginyl-tRNA synthetase, mitochondrial precursor (C 6.1.1.22) (Asparagine--tRNA ligase) (AsnRS).

[Source:Uniprot/SWISSPROT;Ac:Q96I59]

TGACTTTTTTATTCTTATAACCCTCTACTCCCTTGACTTCTGTGACATCACATTATCCCTGTTTTTCTCCTTTCTCAGCA

TTTATTCTTTCTTAGTCTCC

In100 candidate2845 (in intron):

LOCUS ENSG00000149256

DESCRIPTION Teneurin-4 (Ten-4) (Tenascin-M4) (Ten-m4) (Protein Odd

Oz/tenmhomolog

4). [Source:Uniprot/SWISSPROT;Acc:Q6N022]

CCCTTATCCCCTTCCCATAGATAACCACTTTTTGTTTTTTTGTGTATCCTCTCAGGGTTTCTTTCTGCAAATAGAAGTCT

ATATTTTTGCCTTTTTCCCT

In100 candidate2846 (in intron):

LOCUS ENSG00000149256

DESCRIPTION Teneurin-4 (Ten-4) (Tenascin-M4) (Ten-m4) (Protein Odd

Oz/tenmhomolog

4). [Source:Uniprot/SWISSPROT;Acc:Q6N022]

GATAATTTGGCACTCCCCTGGGAAACTAACTTGGCCCCTTCTTTTGTTTTGTTTTTTTACCTTTTTCCCTTATTCAGTAG

CCATTTGCTTCATGCCTTTT

In100 candidate2847 (in intron):

LOCUS ENSG00000079691

DESCRIPTION Leucine rich repeat containing 16.

[Source:Uniprot/SPTREMBL;AccQ5VZK9]

CCACTGCACCACCCTAAATGCTTTTATTTAGTGAGAGCCATAACATCATATTGTTTCCATATTCTTTTCATCTTCCAGGA

AATTCCTAAGCTTTTTTTTT

In100 candidate2848 (in intron):

LOCUS ENSG00000198393

DESCRIPTION Zinc finger protein 26 (Zinc finger protein KOX20).

[Source:Unirot/SWISSPROT;Acc:P17031]

ACTTGTTTGCTAACATCATCTGGATCATCTGTGCATCTTTATCAATTATCTGCCTATTCCTTTTGTCAGTCATATTTTCC

TGCCTCATGTCTCAAAGTTT

In100 candidate2849 (in exon):

LOCUS ENSG00000188177

DESCRIPTION Zinc finger CCCH domain-containing protein 6.

[Source:Uniprot/SISSPROT;Acc:P61129]

TTGATCCAACTGCTTCACCATTTTGTTAGCTATTGTGTAACTGAGCAATTCTTTTCACTCTTGTGACTATCTCAGTCCTC

TGCTGTTTTGTAACTGGTTT

In100 candidate2850 (in intron):

LOCUS ENSG00000197321

DESCRIPTION Supervillin (Archvillin) (p205/p250).

[Source:Uniprot/SWISSPROTAcc:O95425]

TGCCAAATTTTATAAGATGAGGGGACTAGTACCACTAACCTTCCGCATTCCTCTCTTTTCCTGTTGTCCATCTTTCAGCT

TTTCTCAGCTATGTCTTTAT

In100 candidate2851 (in intron):

LOCUS ENSG00000197321

DESCRIPTION Supervillin (Archvillin) (p205/p250).

[Source:Uniprot/SWISSPROTAcc:O95425]

AACCACATCTGACAATAACTATCCTTACCTTTTGCTACCCCTTAAATTCAGACTTCTTTACCTTTGGCATTGGTTTGCTC

CTGATTCCTCTCCAAAATAT

In100 candidate2852 (in intron):

LOCUS ENSG00000154262

DESCRIPTION ATP-binding cassette sub-family A member 6.

[Source:Uniprot/SWISPROT;Acc:Q8N139]

GACTATTAACTTCCTGACACAATTTTCCTGTTTGTTTTTTTTCCTTTTTAATGTGACCTGTCAAATTTGTTCCTCCAGTT

TTTCTCGGGTTTATTCTTTT

In100 candidate2853 (in intron):

LOCUS ENSG00000150672

DESCRIPTION Discs large homolog 2 (Postsynaptic density protein PSD-93)

(Chnnel- associated protein of synapse-110) (Chapsyn-110).

[Source:Uniprot/SWISPROT;Acc:Q15700]

AGGTTGGAAACCATAAATAACTTGTGCTTTTTTTTTCTTACTCCATCATTCCATGACAGCCCCTGTTTCCTCTCCCGCTC

GATTTATTTTCATTTATAGT

In100 candidate2854 (in intron):

LOCUS ENSG00000150672

DESCRIPTION Discs large homolog 2 (Postsynaptic density protein PSD-93)

(Chnnel- associated protein of synapse-110) (Chapsyn-110).

[Source:Uniprot/SWISPROT;Acc:Q15700]

ATGGTAACTTGTTGCACTTGGTTTCAAGTGTCTTTTGTGCCTCCCCTTTTTTTTCTTTTTGTTTTTAATCACCAGGATTT

TTACTTTGCTAGTAATTTCC

In100 candidate2855 (in intron):

LOCUS ENSG00000150672

DESCRIPTION Discs large homolog 2 (Postsynaptic density protein PSD-93)

(Chnnel- associated protein of synapse-110) (Chapsyn-110).

[Source:Uniprot/SWISPROT;Acc:Q15700]

TTTTCCTCTAGTTTCATCTCCTGTAACTCTCTTATTAATTTCTATATTTCAGTTCTACAGGCCTATTTCCCGCCTTAGGA

CTTTTGTATTTGTCATCTTT

In100 candidate2856 (in intron):

LOCUS ENSG00000150672

DESCRIPTION Discs large homolog 2 (Postsynaptic density protein PSD-93)

(Chnnel- associated protein of synapse-110) (Chapsyn-110).

[Source:Uniprot/SWISPROT;Acc:Q15700]

CTTGTGACAAAACATACTTTTGGTTCTTTTACTTCCTCAATAACTTCTTCATATTACTTGTTTATATTACTTTTTCAGCC

TCCTCAGATTTATTTTCTTT

In100 candidate2857 (in intron):

LOCUS ENSG00000150672

DESCRIPTION Discs large homolog 2 (Postsynaptic density protein PSD-93)

(Chnnel- associated protein of synapse-110) (Chapsyn-110).

[Source:Uniprot/SWISPROT;Acc:Q15700]

CCAATCATAGCTGTGCCATATGATCTTTGGAAAGACATCTAACCTTTCTGGGTTTCAATTTTCTTACCCATACAGACATT

TCTTAAGAACTTTTATTTTT

In100 candidate2858 (in intron):

LOCUS ENSG00000150672

DESCRIPTION Discs large homolog 2 (Postsynaptic density protein PSD-93)

(Chnnel- associated protein of synapse-110) (Chapsyn-110).

[Source:Uniprot/SWISPROT;Acc:Q15700]

GCTACCAATATTTAATAACAAATTGACTTGTTTTTACATGAACCCTTGTGCCTCTCTGATCTTTGTGTCATCTGTCAGCT

GGAATTACTTTTGTTTTTCT

In100 candidate2859 (in intron):

LOCUS ENSG00000150672

DESCRIPTION Discs large homolog 2 (Postsynaptic density protein PSD-93)

(Chnnel- associated protein of synapse-110) (Chapsyn-110).

[Source:Uniprot/SWISPROT;Acc:Q15700]

AGGTATGTAGTAGTATCTTACTGGGGTTTTAATTTTCATTTCTCTAACAAGTCATGATGTTGATTATCTTTTCAGATGCT

TATTTGATACCTATATCTTC

In100 candidate2860 (in intron):

LOCUS ENSG00000150672

DESCRIPTION Discs large homolog 2 (Postsynaptic density protein PSD-93)

(Chnnel- associated protein of synapse-110) (Chapsyn-110).

[Source:Uniprot/SWISPROT;Acc:Q15700]

AGTCCCAACTCTCCCCTTTGTTCCTGACTCACGGGGTAACCTTGAACAAATTACTGTTCCTTTCACAGTCTATTTTCTCA

TTCCCCAGTGTTCTATACTT

In100 candidate2861 (in intron):

LOCUS ENSG00000150672

DESCRIPTION Discs large homolog 2 (Postsynaptic density protein PSD-93) (Chnnel- associated protein of synapse-110) (Chapsyn-110). [Source:Uniprot/SWISPROT;Acc:Q15700]

GAAAAAAAATAACAAAAACCTATCCTTTTGCACATTTTCTTTTTCTTAAAGTATGACCCTCTATTTACCCTCCAGAACAA
TCTTTTCGTATCTTAGTCTT

In100 candidate2862 (in intron):

LOCUS ENSG00000150672

DESCRIPTION Discs large homolog 2 (Postsynaptic density protein PSD-93) (Chnnel- associated protein of synapse-110) (Chapsyn-110). [Source:Uniprot/SWISPROT;Acc:Q15700]

AGTAATAAATGACAAGATTAAGACTAAAAGCTGGCCTTCTAACATAGAGTTCCTTTTTTTTTTTAATTTACTCCCAGTA
CCTTTTTTTTTTAATTATTA

In100 candidate2863 (in intron):

LOCUS ENSG00000150672

DESCRIPTION Discs large homolog 2 (Postsynaptic density protein PSD-93) (Chnnel- associated protein of synapse-110) (Chapsyn-110). [Source:Uniprot/SWISPROT;Acc:Q15700]

AACACGGTAACATGTCAACATTTTTACATTCTTTATACTTTTATAATTTTATTATTATCCTAATTTCAGAAAAAAAATTT
GCCTCTCCCCTTTTTCATTT

In100 candidate2864 (in intron):

LOCUS ENSG00000154263

DESCRIPTION ATP-binding cassette sub-family A member 10.

[Source:Uniprot/SWSSPROT;Acc:Q8WWZ4]

TCCTGGGAATGGAACCTGCCACTAAGGTTAACAAAACATGTTTGCTTGTCTCTTTGCTTCTTAAATCAGGTGAATGCTTC

TAGAGTTTTTCTTTATTTCC

In100 candidate2865 (in intron):

LOCUS ENSG00000154263

DESCRIPTION ATP-binding cassette sub-family A member 10.

[Source:Uniprot/SWSSPROT;Acc:Q8WWZ4]

TATTTATAGCTTATATTCAAATTATTCTCTTTAAAAGTAACTACCCTGTCCTCATTCTTTTAATTTTTTCTTCTTCAGTT

TTCTGTGGGTCTTACTCTTC

In100 candidate2866 (in intron):

LOCUS ENSG00000106714

DESCRIPTION Contactin-associated protein-like 3 precursor (Cell

recognitionmolecule Caspr3). [Source:Uniprot/SWISSPROT;Acc:Q9BZ76]

CTTTATGATTAGAATTTGTCCTGAAAGTTTCATAACTTTTTGTGGGACACCTGGATTTTAGTTTTTTTTTTCTAACAGAG

TTATTGAATTCATTTTCTTT

In100 candidate2867 (in intron):

LOCUS ENSG00000058056

DESCRIPTION Ubiquitin carboxyl-terminal hydrolase 13 (EC 3.1.2.15) (Ubiquitn thioesterase 13) (Ubiquitin-specific-processing protease 13) (Deubiquitinting enzyme 13) (Isopeptidase T-3) (ISOT-3). [Source:Uniprot/SWISSPROT;Acc:92995]

CTGAGAGCCACTGTGCCTGGCTACCCTTTTTATATTTTAACTTGCTGTTCACATTTGGGTCTCTCTTCACTCTCTCAGTT

GTGTTCTGTTGATCTTATTT

In100 candidate2868 (in intron):

LOCUS ENSG00000154265

DESCRIPTION ATP-binding cassette sub-family A member 5. [Source:Uniprot/SWISPROT;Acc:Q8WWZ7]

TATTAGATTTAAATTAATTATGAATTACTATTCATTTTGAAAGCTTTTTAACTCTGGAAGATAGTATTTTTTATCAGAT

TACACTTTTACCTATCACTT

In100 candidate2869 (in intron):

LOCUS ENSG00000101391

DESCRIPTION CDK5 regulatory subunit-associated protein 1 (CDK5 activatorbiding protein C42). [Source:Uniprot/SWISSPROT;Acc:Q96SZ6]

GGAAAACCTCTTTATTTTGAGGAGGAGTGTAACATGTGGATTTAAACTTATTTTCCCTTCAGGCTGATGAACACTATCAG

TTAGCCCTTCCTTTTCTTTT

In100 candidate2870 (in intron):

LOCUS ENSG00000114757

DESCRIPTION PEX5-related protein (Peroxin-5-related protein) (Pex5Rp) (PEX5like protein) (PEX2-related protein). [Source:Uniprot/SWISSPROT;Acc:Q8IYB4]EXON

GGGGATATTTATAATACCTTGCTAACTTTTAGCTTCATTAGTTGCTAAGCATTTTCTTTCTCACTGTATTCATTTCAGTT

GGTCATCATTTCCCTTTTCA

In100 candidate2871 (in intron):

LOCUS ENSG00000165757

DESCRIPTION Novel protein. [Source:Uniprot/SPTREMBL;Acc:Q5T992]

ATAACATCTGCATCACTTCAAGGTTCACTTCTACCTTTTTGCTCTCTGTTGCCAGTTCACCAGAAGCCCCTGTGATGATT

ACTATTCTTTTTTTTTTTTT

In100 candidate2872 (in intron):

LOCUS ENSG00000153822

DESCRIPTION Inward rectifier potassium channel 16 (Potassium channel, inwarly rectifying subfamily J member 16) (Inward rectifier K(+) channel Kir5.1) [Source:Uniprot/SWISSPROT;Acc:Q9NPI9]

TGCTTGTAACACTTTCTTTACTTCGTATCACATGTCACAATTTTTCCTCCTACTTCAAGCAATTCTTTTTCTCAGTCTTC

TTAGTTATAGCCTCCTCATT

In100 candidate2873 (in intron):

LOCUS ENSG00000145075

DESCRIPTION Coiled-coil domain-containing protein 39. [Source:Uniprot/SWISSROT;Acc:Q9UFE4]

ATATTGGAGGGTGGAATATTTTATCTCCTAACTGATATTATACTCTTTTGTTCCTCTTCCTGCAAAACAGTTCATTCCTG

CCTTCTCAGCATTTTCTTTT

In100 candidate2874 (in intron):

LOCUS ENSG00000078699

DESCRIPTION Protein CBFA2T2 (MTG8-like protein) (MTG8-related protein 1) (Meloid translocation-related protein 1) (ETO homologous on chromosome 20) (p5). [Source:Uniprot/SWISSPROT;Acc:O43439]

AGTGAATATTTATATCACTCCGAAAAGAAACCCCATACCCATTAACAATTACTGTCTATTTTTCCTTTCCCCAGTCCCTA
ATCTACTTTTTTATTTTTCC

In100 candidate2875 (in intron):

LOCUS ENSG00000133195

DESCRIPTION solute carrier family 39 (metal ion transporter), member 11 [Sorce:RefSeq_peptide;Acc:NP_631916]

CATACCTCATAGCTTCACTTAACAAAAACATATCTTTTTTTTCCTTGCATATGGAGTTGTTTCCCATATTATTTCCAGTT
TTTTTTTTTTTTTGGAGACA

In100 candidate2876 (in intron):

LOCUS ENSG00000133195

DESCRIPTION solute carrier family 39 (metal ion transporter), member 11 [Sorce:RefSeq_peptide;Acc:NP_631916]

GCATCACACTCTTGGGTTGGTGGGAAGAGAATGGTTAACACATGACTGTGTTCTTTAACTCCATTTCTGTTCCTTCAGCC
TTTCAGATTTATCTGTCCCT

In100 candidate2877 (in intron):

LOCUS ENSG00000133195

DESCRIPTION solute carrier family 39 (metal ion transporter), member 11 [Sorce:RefSeq_peptide;Acc:NP_631916]

ATATTGTTTTGACCTCTAACACTGTGCTGTGTGTATGTCTTTTCAGATTTTTTTTTCCACAAAAAATCTGTTCAAATTCC

CCACCCCCAACTTTTTTTTT

In100 candidate2878 (in intron):

LOCUS ENSG00000133195

DESCRIPTION solute carrier family 39 (metal ion transporter), member 11

[Sorce:RefSeq_peptide;Acc:NP_631916]

AGAGATTGCAATTAAAGTAACCAAGTATTGTCTTATTGTCACATTTTTATTAAACTCCTTCTTGACTGTGTCTTCCAGAC

CTTTCTTATAATCATTATCT

In100 candidate2879 (in intron):

LOCUS ENSG00000073921

DESCRIPTION Phosphatidylinositol-binding clathrin assembly protein (Clathri

assembly lymphoid myeloid leukemia protein).

[Source:Uniprot/SWISSPROT;AccQ13492]

CATAAGCTATCCTTGTAACATTTCCATCAACTCTCTCATTTGTTGACATCTGTTTTACCTTGTTTTGAATTATTCCAGGA

CATTTTTTTGTTGTTGTTTC

In100 candidate2880 (in intron):

LOCUS ENSG00000058063

DESCRIPTION Probable phospholipid-transporting ATPase IF (EC 3.6.3.1) (ATPae class I type 11B) (ATPase IR). [Source:Uniprot/SWISSPROT;Acc:Q9Y2G3]

CAACCCTCTTTTCCAAGATAACAATTCCACTTAAGCTTTTGATCCTTTTCTTTCATTCTGCTGTCCATACTCCATCAGTT

ATCATTAGTATGTTTCTTTT

In100 candidate2881 (in intron):

LOCUS ENSG00000058063

DESCRIPTION Probable phospholipid-transporting ATPase IF (EC 3.6.3.1) (ATPae class I type 11B) (ATPase IR). [Source:Uniprot/SWISSPROT;Acc:Q9Y2G3]

TTTAAAGACTTCTCCTCTGTTAACCTGTGAAATCCTTGAAGTCCATTTATTCTTTGTCTTTGTTCCCAGAACCTATCTCA

ATGTCTGTTTCACAGTTTCT

In100 candidate2882 (in intron):

LOCUS ENSG00000167554

DESCRIPTION Zinc finger protein 610. [Source:Uniprot/SWISSPROT;Acc:Q8N9Z0]

EXON

TATAATGAAGTTACATGATATAACTTCATTATTTGTCTTTTTGTCTTTGTATGACTGGCTTATTTCACTTACCAGAGTGT

CTTTCAGGTTCATCCATTTT

In100 candidate2883 (in intron):

LOCUS ENSG00000141219

DESCRIPTION lung cancer-related protein 8 [Source:RefSeq_peptide;Acc:NP_06011]

TAGTCACAAAGTCAAGCCCCTTCTCCGTAATAAACACATTTAATTATATTAACATTTGTGTTGCTTTGTGGTTTTCAGAG

TGCCTCCATCTGTATTTTTT

In100 candidate2884 (in intron):

LOCUS ENSG00000183621

DESCRIPTION Zinc finger protein 438. [Source:Uniprot/SWISSPROT;Acc:Q7Z4V0]

EXON

AGTGGGGCATCCTGTATATTATCTGGCAAACTTAACTTCACTTTCTGTGAATTGTCTGCCTCTGCCTTTTTGTCAGATTT

TCAGTTGCACTTTCTATCTT

In100 candidate2885 (in intron):

LOCUS ENSG00000053524

DESCRIPTION Rho family guanine-nucleotide exchange factor

[Source:RefSeq_petide;Acc:NP_055893]

TGTTTCTGAACAACAACAAAAAATTTTCCGTAACCAACCTGTTGAATATTCACACTTCCCTTCAATTTTCAGTTCATTGT

GATAGATTTTTCCTTGTTTC

In100 candidate2886 (in intron):

LOCUS ENSG00000053524

DESCRIPTION Rho family guanine-nucleotide exchange factor

[Source:RefSeq_petide;Acc:NP_055893]

GTTTAAATGGCTTTATTATATGCATTTTACATATAACCTCTTCATCTTTTCTTTTATCTTCTACACCTTCTGGAACAGTT

CATCTGATTTATTGTCTTTA

In100 candidate2887 (in intron):

LOCUS ENSG00000149201

DESCRIPTION coiled-coil domain containing 81

[Source:RefSeq_peptide;Acc:NP_68599]

GCTTGGCCTATTCATGCTCTTCTAACATGCCTGCCCTTCCCCAACTTCATCCTTTTGTTCATTTTTTTTTTCCTGCAGCC

TGAATTGTCCTTCCTTTTCT

In100 candidate2888 (in intron):

LOCUS ENSG00000148516

DESCRIPTION Transcription factor 8 (NIL-2-A zinc finger protein) (Negative

egulator of IL2). [Source:Uniprot/SWISSPROT;Acc:P37275]

TCTGTTTTATTTTATCACCTATTCCTATTCCTTGAAAATTCGTTATAAGTAACCTGTGCCTTATTTTTGTTTTTACAGAT

CTCGCTTTTTAATACTCCTT

In100 candidate2889 (in exon):

LOCUS ENSG00000148516

DESCRIPTION Transcription factor 8 (NIL-2-A zinc finger protein) (Negative

egulator of IL2). [Source:Uniprot/SWISSPROT;Acc:P37275]

AACTGTTTCCAAATGTTAGTTATTATGGACCCAATTTATTAACAACATTAGCTGATTTTTACCTATCAGTATTATTTTAT

TTCTTTTAGTTTATAGATCT

In100 candidate2890 (in intron):

LOCUS ENSG00000151376

DESCRIPTION NADP-dependent malic enzyme, mitochondrial precursor (EC 1.1.1.0)

(NADP-ME) (Malic enzyme 3). [Source:Uniprot/SWISSPROT;Acc:Q16798]

TCTGTCTGATATAACTAACCCTGTTCTTCTTTGGTTTACATTTGCGTGGAATATCTTTTTCCATCCCTCCACTTTCAGTT

TCTGGTTTTTTTTTGGTTTG

In100 candidate2891 (in intron):

LOCUS ENSG00000172578

DESCRIPTION Kelch-like protein 6. [Source:Uniprot/SWISSPROT;Acc:Q8WZ60]

CATTTACCAAACTTTAACCCTCTTGTCCTATCATCATCTTTCCCATTTTTGTTTTATTTTGTTTTGTTTTTTCTTCAGAC

AGACTTTTGTTCTTGTTGCC

In100 candidate2892 (in intron):

LOCUS ENSG00000114796

DESCRIPTION Kelch-like protein 24 (Protein DRE1).

[Source:Uniprot/SWISSPROTAcc:Q6TFL4]

AATTAAAATTTTAAAAATTGACATTCTGCAAAAATGCTGATATTATATAACTGCACTACATCCTCTGCCTTTATTCAGAG

TGCCCTTAGCATCTTTTTTT

In100 candidate2893 (in intron):

LOCUS ENSG00000163872

DESCRIPTION YEATS domain-containing protein 2.

[Source:Uniprot/SWISSPROT;Ac:Q9ULM3]

ATTTTTCCTGTCATTTTTTAGTTAACTCTTGGAGCCATCTTTGATTCTTCCCTCTTCTAAATATTTGTTTAGTTGCAGAC

CATCATTCTTTCTTCATAAT

In100 candidate2894 (in intron):

LOCUS ENSG00000166575

DESCRIPTION transmembrane protein 135 (TMEM135), mRNA

[Source:RefSeq_dna;Ac:NM_022918]

ATTTTCTTAATCACTCTTTAACATTTTCCTACTTTTTCATCTCTTTGTGCTGAATTCTGGTTATCTTTTCAGATTTGTCT

TCAAGTTAACTCATCTATTT

In100 candidate2895 (in intron):

LOCUS ENSG00000165322

DESCRIPTION Rho GTPase-activating protein 12.

[Source:Uniprot/SWISSPROT;AccQ8IWW6]

AATGTAGCTTTATAGTAAGTGTTGAAAGTAACTAATATGTGTCTTCCTACTTGGTTGTACTTCAGTATTGTGTTGGCTGT

TCTAAGTCTTTTGCTTTTCC

In100 candidate2896 (in intron):

LOCUS ENSG00000165322

DESCRIPTION Rho GTPase-activating protein 12.

[Source:Uniprot/SWISSPROT;AccQ8IWW6]

TGTATGTTAATTTAACCTTCTGATTGTTTCTTGAATCTGTCCCTGCTTCTGCATTCCTACTGTCTTGTCCTATTGCAGCT

CTCTTCATCTTCTGTCTGTA

In100 candidate2897 (in intron):

LOCUS ENSG00000165322

DESCRIPTION Rho GTPase-activating protein 12.

[Source:Uniprot/SWISSPROT;AccQ8IWW6]

CCATGTGACTTCTGTTAAGATACCTGTCTTAACTCTGATGAAATTTTACTTAATTTCTGTATTTATACTCATTCCCAGTC

TTTTCTGTTGCTGATCATGC

In100 candidate2898 (in intron):

LOCUS ENSG00000154529

DESCRIPTION Contactin associated protein-like 3B.

[Source:Uniprot/SPTREMBL;cc:Q5RGR9]

TGATTAGAATTTGTCCTGAAAGTTTCATAACTTTTTGCGGGACACCTGGATTTATTTAGTTTTTTTTTTTCTAACAGAG

TTATCGAATTCATTTTCTTT

In100 candidate2899 (in intron):

LOCUS ENSG00000158352

DESCRIPTION shroom family member 4 [Source:RefSeq_peptide;Acc:NP_065768]

AGCACTTAACATTGTTGAACATTATTGTCTTCTAAAAATTTTATCTTCCTTTGGCTCCAGAAACACTATGCTTCCCCTGG

TTTTTCTTTGCTTTCTCTTG

In100 candidate2900 (in intron):

LOCUS ENSG00000172794

DESCRIPTION Ras-related protein Rab-37. [Source:Uniprot/SWISSPROT;Acc:Q96AX]

AGCATCCCCCTACCTGGGTCCCACTGTAACATATCATCTTGTTCTGTTTTCCTCACAGTACTTATCACTTCTCACTTCTT

GAAATTATTTTTTTGTTTTC

In100 candidate2901 (in intron):

LOCUS ENSG00000175497

DESCRIPTION Inactive dipeptidyl peptidase 10 (Dipeptidyl peptidase X) (Dipetidyl peptidase-like protein 2) (DPL2) (Dipeptidyl peptidase IV-related proein 3) (DPRP-3). [Source:Uniprot/SWISSPROT;Acc:Q8N608]

CATACTGACATACTGATTCTTTGAAGAGAAACTTGGGGTTCACTATATTTAACAATCCCCCATCTTGAATTTTTTCAGTT
CTTTCTCTTCAGACTTCCTT

In100 candidate2902 (in intron):

LOCUS ENSG00000175497

DESCRIPTION Inactive dipeptidyl peptidase 10 (Dipeptidyl peptidase X) (Dipetidyl peptidase-like protein 2) (DPL2) (Dipeptidyl peptidase IV-related proein 3) (DPRP-3). [Source:Uniprot/SWISSPROT;Acc:Q8N608]

TAGCTCTCTTTTATTAGTTATTTTGGCCAATCCCTGTAGAGGATATCTTAACTGAGTATTTTATTTAATTCTTACAGCAA
ACCTCCTTTTTGTTATCCAT

In100 candidate2903 (in intron):

LOCUS ENSG00000175497

DESCRIPTION Inactive dipeptidyl peptidase 10 (Dipeptidyl peptidase X) (Dipetidyl peptidase-like protein 2) (DPL2) (Dipeptidyl peptidase IV-related proein 3) (DPRP-3). [Source:Uniprot/SWISSPROT;Acc:Q8N608]

CTCTAGCATACATGTCTTTGCCTTAACATTTGCCTTTCTTTCCTTTGGGTTCTTTCTATACACAGCATTTTCAACATGTG
TCATCTTGATCTGCCTTTTC

In100 candidate2904 (in exon):

LOCUS ENSG00000168930

DESCRIPTION Tripartite motif-containing protein 49 (RING finger protein 18)(Testis-specific ring-finger protein). [Source:Uniprot/SWISSPROT;Acc:Q9NS80

ACTTGGAGTGAAGTCTCAATGATAACTGGGAAATGTTTTTCTTCCTCTTTATCTAACTATATTACACTTATCCATCAGGT

TTCATTGTATTAATCTATCC

In100 candidate2905 (in exon):

LOCUS ENSG00000173827

DESCRIPTION Tripartite motif-containing 49.

[Source:Uniprot/SPTREMBL;Acc:A0VR7]

ACTTGGAGTGAAGTCTCAATGATAACTGGGAAATGTTTTTCTTCCTCTTTATCTAACTATATTACACTTATCCATCAGGT

TTCATTGTATTAATCTATCC

In100 candidate2906 (in intron):

LOCUS ENSG00000198346

DESCRIPTION ZNF765 protein. [Source:Uniprot/SPTREMBL;Acc:Q7L2R6]

TAAAATGGGACACATCCAAATATGGCAAACTCTTCATACTAGAAAGTAACTTTATTAAGTATTTCTATTGCATTTCAGCT

GACTTTATTTTTTATTTTTA

In100 candidate2907 (in intron):

LOCUS ENSG00000180773

DESCRIPTION solute carrier family 36 (proton/amino acid symporter), member

[Source:RefSeq_peptide;Acc:NP_689526]

CTTAAATGTCACCTGTTCTGTGAAACCTTTCTTAACTGTTCCTTCCATGTCTTCCTTTTCTTTATCTCTAGCCAGAATTT

ATTGGTTTTTGTCCTGGCTT

In100 candidate2908 (in intron):

LOCUS ENSG00000204794

DESCRIPTION

ACTATTTAGTAACCATTCTGGTTATTTTTACTACTGAATTGATCCTTTAATTATTCTATTTCCCCTTCTCCTCCCCAGTT

TTTCATCTCTTTATTTTGTT

In100 candidate2909 (in intron):

LOCUS ENSG00000184770

DESCRIPTION

ACTATTTAGTAACCATTCTGGTTATTTTTACTACTGAATTGATCCTTTAATTATTCTATTTCCCCTTCTCCTCCCCAGTT

TTTCATCTCTTTATTTTGTT

In100 candidate2910 (in intron):

LOCUS ENSG00000163075

DESCRIPTION

TCCACTATTTATCTTCCTGTGGTTTTAACCATGTATTTTATGTGATTCCATTTTCTCTCTTTTCTTTTAGCATACCAGTT

ATATTTCTTTGTTTCCTTTT

In100 candidate2911 (in intron):

LOCUS ENSG00000150076

DESCRIPTION Coiled-coil domain-containing protein 7.

[Source:Uniprot/SWISSPOT;Acc:Q96M83]

AACTCCTTCAATTCCTGCTACCTAACACAATATTAACGTCTGTACTCATTTTCTCTTTCTTTTTTCCTGTTAAGACAGAT

ATATTGTTCTTTCTTATACA

In100 candidate2912 (in intron):

LOCUS ENSG00000150076

DESCRIPTION Coiled-coil domain-containing protein 7.

[Source:Uniprot/SWISSPOT;Acc:Q96M83]

CTTATCTCGATGCATATTATTTAAAATTGTTATAACCTCTTGATGGATTGACCCCTTTATAATTTTTTTTATGCCCAGCT

AATTTTTATTTATTTATTTA

In100 candidate2913 (in intron):

LOCUS ENSG00000150076

DESCRIPTION Coiled-coil domain-containing protein 7.

[Source:Uniprot/SWISSPOT;Acc:Q96M83]

TACCATTATGTAACGGCCTTCTTTGTCTCTTTTTATCTTTGTTGGTTTAAAGTCTGTTTTGTCAGATACTAGGATTGCAA

CCCTTACCTTTTTTTGTTTT

In100 candidate2914 (in intron):

LOCUS ENSG00000088179

DESCRIPTION Tyrosine-protein phosphatase non-receptor type 4 (EC 3.1.3.48)

Protein-tyrosine phosphatase MEG1) (PTPase-MEG1) (MEG).

[Source:Uniprot/SWISPROT;Acc:P29074]

AGGAGCTTAGACTTGATTTGAGACCTTCCCTTTTTTTAATGTAACATTTAGGTTTATAAATTTTCCTCTCAGCTCTGCTT

TGGCCTTATTCCACAAGTTT

In100 candidate2915 (in intron):

LOCUS ENSG00000078747

DESCRIPTION E3 ubiquitin-protein ligase Itchy homolog (EC 6.3.2.-) (Itch)

(trophin-1-interacting protein 4) (AIP4) (NFE2-associated polypeptide 1) (NAP1).

[Source:Uniprot/SWISSPROT;Acc:Q96J02]

TAATTTTTAACTATTTTCAAAAATAATCTTATATTTACTCACATTACTATTTCTGGTTCTCCTTATTTCTTTCCTCAGAT

TCATTTTTTTTTTAAATCT

In100 candidate2916 (in intron):

LOCUS ENSG00000163882

DESCRIPTION DNA-directed RNA polymerases I, II, and III 17.1 kDa polypeptid

(EC 2.7.7.6) (RPB17) (RPB8) (RPABC3). [Source:Uniprot/SWISSPROT;Acc:P52434

AAAAGGCTGTGTGTGTGTGTGTGTGTGTGTGTGGCTACTCTGTAACCCAATCCCTAGTTCCTTGGTTTTCCTGTCCAGCT

ATTTTTTCTTTTCTTTAAAG

In100 candidate2917 (in intron):

LOCUS ENSG00000115109

DESCRIPTION Band 4.1-like protein 5. [Source:Uniprot/SWISSPROT;Acc:Q9HCM4]

EXON

AGTTTTTCTGTATGTTTAAACATTTTTATAACGATGAAAATTTTTTTTTGAATCCAGTTCTTTCATTTTCCTCTGCTCA

GTAATTTGACTGCTATACTT

In100 candidate2918 (in intron):

LOCUS ENSG00000144118

DESCRIPTION Ras-related protein Ral-B. [Source:Uniprot/SWISSPROT;Acc:P11234

TCTGTCATTGACTGAAACTTCTTTATTTGGTGCATGACTGTATAACAACTATTTTTGTTGTATATTTTCTCTCAGGTCTT

TGTTTTAGAGCTTGTGCTCT

In100 candidate2919 (in intron):

LOCUS ENSG00000177885

DESCRIPTION Growth factor receptor-bound protein 2 (Adapter protein GRB2) (H2/SH3 adapter GRB2) (Protein Ash). [Source:Uniprot/SWISSPROT;Acc:P62993]

AGTAACATAGTGAAAGTTTTTTTCTTCGTTTTAACAGTGGCTTTTATTTTCAACTAATTTATTCTTTAGTTTAATAGTGG

ACTTTTTATTCCTTTTTTTT

In100 candidate2920 (in intron):

LOCUS ENSG00000074047

DESCRIPTION Zinc finger protein GLI2 (Tax helper protein). [Source:Uniprot/WISSPROT;Acc:P10070]

TGTTTATAACTATATCCCCTTTTTCATTCTCTATGTTATTTATTTGTCCCTTCCCTGTTTCTTGGTTAGTCTTCACAGAG

TTTTATTTTTTATTTATTTT

In100 candidate2921 (in exon):

LOCUS ENSG00000166025

DESCRIPTION Angiomotin-like protein 1. [Source:Uniprot/SWISSPROT;Acc:Q8IY63

AGGATAGATTATGATCTCGGTCCCCATCCATCCTAACTTCTGAGTTCATCTCTGTCTCTGCTTTACAGTCTTTAATTTTT

TGTTGTTCCTTCATTTTTTT

In100 candidate2922 (in intron):

LOCUS ENSG00000156931

DESCRIPTION Vacuolar protein sorting-associated protein 8 homolog.

[Source:niprot/SWISSPROT;Acc:Q8N3P4]

CTAATAAATATTGTTGTTCCCCTTAACAAATGTGTTTTTCTGTGTCCATGATTTTGCTCTCACTCTTCTCTTTGCCAGGT

TTGCCTTCTCTGTTGACATT

In100 candidate2923 (in intron):

LOCUS ENSG00000074054

DESCRIPTION CLIP-associating protein 1 (Cytoplasmic linker-associated proten

1) (Multiple asters homolog 1) (hOrbit1). [Source:Uniprot/SWISSPROT;Acc:QZ460]

AGTAACATTTTCTTCATCATTTCTAATTGTGTTTATTTGGATCTTCCCTTTTTTCTTCTTTGTTAGTCTAGCTAGCAGCC

TATTTATCTTATTAATTTTT

In100 candidate2924 (in intron):

LOCUS ENSG00000074054

DESCRIPTION CLIP-associating protein 1 (Cytoplasmic linker-associated proten

1) (Multiple asters homolog 1) (hOrbit1). [Source:Uniprot/SWISSPROT;Acc:QZ460]

TAGAGTTTATTTATAAGTTTTTCCTTAAACTGAAAAGTCATTGATTCTTAACTAGTTTTATTATTTTTTTAATGCCAGCT

TTCTTTCATTGCTCTTTACC

In100 candidate2925 (in intron):

LOCUS ENSG00000074054

DESCRIPTION CLIP-associating protein 1 (Cytoplasmic linker-associated proten

1) (Multiple asters homolog 1) (hOrbit1). [Source:Uniprot/SWISSPROT;Acc:QZ460]

AGCCACATTTATTATGATTAACTAATATGTTTGAGCTTCTATCTTATTTTGTGATTTTTATTTACCAGGTTTCTGGTTAT

TTTATCCCCCTACTCCCTTT

In100 candidate2926 (in intron):

LOCUS ENSG00000073803

DESCRIPTION Mitogen-activated protein kinase kinase kinase 13 (EC 2.7.11.25 (Mixed lineage kinase) (MLK) (Leucine zipper-bearing kinase). [Source:Unipot/SWISSPROT;Acc:O43283]

TGTATTATAACTTAAATTAGGTTTGTTTTGTTTGGTCCCCTTTATTATTTCTCAGATGCAACAGGCATTTCTAATTCAAT
ATTATTTCTTTTTTTTTTTT

In100 candidate2927 (in intron):

LOCUS ENSG00000211460

DESCRIPTION Translin. [Source:Uniprot/SWISSPROT;Acc:Q15631]

AGTAACTTGCTGGTTGCTTTTTTGATCTTTCTGCTTCACTGTCAGTTTTTTTTTTTTTTTTTTTTGAGACAGAGTCC
CGCTCTGTCGCCTAGGCTGG

In100 candidate2928 (in intron):

LOCUS ENSG00000101464

DESCRIPTION GPI transamidase component PIG-U (Phosphatidylinositol-glycan bosynthesis class U protein) (Cell division cycle protein 91-like 1) (CDC91-ike 1 protein). [Source:Uniprot/SWISSPROT;Acc:Q9H490]

CTCTCTACTCTGGAAACTGATAATAGTAAGGTTTAACTTAATTTTTTTACATGTCTGTTTTTTTTCTGCAGACTCCATT

AATAATATACCTCTTTCATT

In100 candidate2929 (in intron):

LOCUS ENSG00000101464

DESCRIPTION GPI transamidase component PIG-U (Phosphatidylinositol-glycan bosynthesis class U protein) (Cell division cycle protein 91-like 1) (CDC91-ike 1 protein). [Source:Uniprot/SWISSPROT;Acc:Q9H490]

AATTTATAACTTTTGTCTTTTTTCTCTCCCTTTGTTTCTTAATTCCAGACTTTCTGTTCCAGATCTCACTCCAAACATT

GGTCTTTTCTGGTACTTCTT

In100 candidate2930 (in intron):

LOCUS ENSG00000073792

DESCRIPTION Insulin-like growth factor 2 mRNA-binding protein 2 (IGF-II mRNbinding protein 2) (IMP-2) (Hepatocellular carcinoma autoantigen p62). [Surce:Uniprot/SWISSPROT;Acc:Q9Y6M1]

TGGTCAAATCTGCTGTTGAACTCCTTTAACAAATTTTTCTGTTCAATTGTATTTTTCAGCTCCAGAATTTGTTAGGTTCT

TTCTTATAGTTTCTGTCTCT

In100 candidate2931 (in intron):

LOCUS ENSG00000155052

DESCRIPTION contactin associated protein-like 5 isoform 1 [Source:RefSeq_petide;Acc:NP_570129]

TTTTGATCTAAGAATCTTATTACTCTTTAACATGTCCTTATCCTTTTGCTTTTTCTCAGTCTGTTCAGTTGTACTTATTT

TTCATGCCAATTGTTCACCC

In100 candidate2932 (in intron):

LOCUS ENSG00000155052

DESCRIPTION contactin associated protein-like 5 isoform 1

[Source:RefSeq_petide;Acc:NP_570129]

AGAGCAACATTTAAGGAAAAGAAAACTTCTTATTTAACATCATCCTTCCCTTCTTTAAATCAGCAATCAGCATTTTTTTT

TTTTTGAGCTTTGGTTTTCC

In100 candidate2933 (in intron):

LOCUS ENSG00000155052

DESCRIPTION contactin associated protein-like 5 isoform 1

[Source:RefSeq_petide;Acc:NP_570129]

CTAGGTTCTCTAGTTTATGTGCATAAAGGTGTTCATAATAACCTTTAATAATCTTTTGTATTTTTGTGTTATCAGTAGTA

ATACCTCCTGTTTCATTTCT

In100 candidate2934 (in intron):

LOCUS ENSG00000155052

DESCRIPTION contactin associated protein-like 5 isoform 1

[Source:RefSeq_petide;Acc:NP_570129]

AACAAATTTGGTAACCTGAGTAGGATATTTAAATATTTCTATCTATCTACTCTTCCAGCAATGTATTGCCCACTCCTTTA

CTGTTTTATGTTTCACCATT

In100 candidate2935 (in intron):

LOCUS ENSG00000087053

DESCRIPTION Myotubularin-related protein 2 (EC 3.1.3.-).

[Source:Uniprot/SWSSPROT;Acc:Q13614]

GGTATTATAGATAACCTTGAAGCTAACTTGATTTTGCTTTCTTCAAAGGTAATTTTTTTTCCACCTATATTCTTACAGTT

TATATTTTAATTCTTTTAGT

In100 candidate2936 (in intron):

LOCUS ENSG00000198646

DESCRIPTION Nuclear receptor coactivator 6 (Amplified in breast cancer protin 3) (Cancer-amplified transcriptional coactivator ASC-2) (Activating signl cointegrator 2) (ASC-2) (Peroxisome proliferator-activated receptor-intercting protein) (PPAR-interacting protei [Source:Uniprot/SWISSPROT;Acc:Q1468]

TTTCATAGAACTATGAATTATACATTGTTGAATGCATAACTATGCATTGTAATGCTCTTTTGCATCTTCTCTTTTCAGAA

GTTTTCTTATTGTCCTCTTG

In100 candidate2937 (in intron):

LOCUS ENSG00000161526

DESCRIPTION SAP30-binding protein (Transcriptional regulator protein HCNGP)

[Source:Uniprot/SWISSPROT;Acc:Q9UHR5]

TGTGGACTGTCTCACCACTAAAGTAACTTGACTTTGAGCTGCTGATGCTTTTTTGTTTTTTGTTTTTTTCCCCTCCAGAA

TATCTCTTTTTCCCCTTATT

In100 candidate2938 (in intron):

LOCUS ENSG00000161526

DESCRIPTION SAP30-binding protein (Transcriptional regulator protein HCNGP)

[Source:Uniprot/SWISSPROT;Acc:Q9UHR5]

TTATCCCTACCTGATAACATTTTATGTGCTTATGTTTTTTTGTCCATGTCTGTCCCTTGTTCTAATCCCCACCTACAGAA

TTTTTCCTTCTTCTCTCAAC

In100 candidate2939 (in intron):

LOCUS ENSG00000149972

DESCRIPTION Contactin-5 precursor (Neural recognition molecule NB-2) (hNB-2.

[Source:Uniprot/SWISSPROT;Acc:O94779]

AGCTCCTGGATTCATTAACTTTTTGAAGGGTTTTTTTGGTCTCTATTTCCTTCAGTCCTGCTCTGATTTTAGTTATATCT

TGCCTTCTGCTAGCTTTTGA

In100 candidate2940 (in intron):

LOCUS ENSG00000171656

DESCRIPTION ETS translocation variant 5 (Ets-related protein ERM).

[Source:niprot/SWISSPROT;Acc:P41161]

GGCTTTTAAAAAAATTATACTTGGAATACAATTTTTCTAACCCTGGAATGCCTCTTTTCCGTTCGTTTATGGCAGTTTAT

TTCTTTTAATTTTTTAGAAT

In100 candidate2941 (in intron):

LOCUS ENSG00000110318

DESCRIPTION Uncharacterized protein KIAA1377.

[Source:Uniprot/SWISSPROT;AccQ9P2H0]

AGTTTTCTTAACATTTTCCAAAGCAACTTGTCTATGTTTTCTCTATTTTTTTTTTTTTTGCTTACTACTTCATCAGTTTA

TTTTCTTTTTATTAGATTCT

In100 candidate2942 (in intron):

LOCUS ENSG00000154330

DESCRIPTION Phosphoglucomutase-like protein 5 (Phosphoglucomutase-related

potein) (PGM-RP) (Aciculin). [Source:Uniprot/SWISSPROT;Acc:Q15124]

ACTATTTAGTAACCATTCTGGTTATTTTTACTACTGAATTGATCCTTTAATTATTCTATTTCCCCTTCTCCTCCCCAGTT

TTTCATCTCTTTATTTTGTT

In100 candidate2943 (in intron):

LOCUS ENSG00000137693

DESCRIPTION 65 kDa Yes-associated protein (YAP65).

[Source:Uniprot/SWISSPRO;Acc:P46937]

TGAAAATGAACTAATACAATCCTTAAGTGCTTTTAACAAGCAAATGCTTACTTCATTTATATTTGTCCTTATCAGTATCA

GTTTTGCTAGCTTACCTTTT

In100 candidate2944 (in intron):

LOCUS ENSG00000148498

DESCRIPTION Partitioning-defective 3 homolog (PARD-3) (PAR-3) (Atypical

PKCisotype-specific-interacting protein) (ASIP) (CTCL tumor antigen se2- 5)

(PR3-alpha). [Source:Uniprot/SWISSPROT;Acc:Q8TEW0]

CATTGAGGTGGGTAGGTGTAACCATGAGAATGGGTTGTCATCCTCATCTCCACAATTTATTTGTTATCACTTCTCCAGAT

CCCTGTTTCTTCTTTCAGTC

In100 candidate2945 (in intron):

LOCUS ENSG00000148498

DESCRIPTION Partitioning-defective 3 homolog (PARD-3) (PAR-3) (Atypical PKCisotype-specific-interacting protein) (ASIP) (CTCL tumor antigen se2- 5) (PR3-alpha). [Source:Uniprot/SWISSPROT;Acc:Q8TEW0]

AGCACATGGCAATGTGACAATTGCTCACTGCATAACCAAATGGATACTCTGAATATATTTCCTCTTCTACAGATTTTTTT
TCTCCTGGGGCAATTTTTTT

In100 candidate2946 (in intron):

LOCUS ENSG00000148498

DESCRIPTION Partitioning-defective 3 homolog (PARD-3) (PAR-3) (Atypical PKCisotype-specific-interacting protein) (ASIP) (CTCL tumor antigen se2- 5) (PR3-alpha). [Source:Uniprot/SWISSPROT;Acc:Q8TEW0]

TAAGGCTTTATTTGGACTTTGATCATAAATGTATTTACTTTGCATAGTTAACATGACTATTACTCTGTATTTTTTCAGAT
TCTTATTTTGAGTTCTTCAT

In100 candidate2947 (in intron):

LOCUS ENSG00000148498

DESCRIPTION Partitioning-defective 3 homolog (PARD-3) (PAR-3) (Atypical PKCisotype-specific-interacting protein) (ASIP) (CTCL tumor antigen se2- 5) (PR3-alpha). [Source:Uniprot/SWISSPROT;Acc:Q8TEW0]

CTTTTTTTTTTTTTGCTTTGTTTTTATTTTATTTATTAACTAAAGGAAAAGCTACTTCTTTTATAGCAGTCTGTTTAATA
GCACTTATTTTTACCTGTTT

In100 candidate2948 (in intron):

LOCUS ENSG00000107242

DESCRIPTION Phosphatidylinositol-4-phosphate 5-kinase type-1 beta (EC 2.7.168) (Phosphatidylinositol-4-phosphate 5-kinase type I beta) (PtdIns(4)P-5-inase beta) (PIP5KIbeta) (Type I phosphatidylinositol-4-phosphate 5- kinasebeta) (Protein STM-7). [Source:Uniprot/SWISSPROT;Acc:O14986]

CTGTCTCTTGCTTTTTGCACCTTTAAAAGTCTAGACCAATTTAATAACCCCACGCCCCATGGTCTCTTTGCTACTCAGCT
GATTTTTTGATCCCAACTCT

In100 candidate2949 (in intron):

LOCUS ENSG00000107242

DESCRIPTION Phosphatidylinositol-4-phosphate 5-kinase type-1 beta (EC 2.7.168) (Phosphatidylinositol-4-phosphate 5-kinase type I beta) (PtdIns(4)P-5-inase beta) (PIP5KIbeta) (Type I phosphatidylinositol-4-phosphate 5- kinasebeta) (Protein STM-7). [Source:Uniprot/SWISSPROT;Acc:O14986]

TTTTTTTAACCCACCTTTGAAATTTTAGCACTCTTTTTACTTTGGTCATCTCTGTCTCTGTGCAGCTACCAATTTTTTAA
AAATGTTCTCTTCTATTCTC

In100 candidate2950 (in intron):

LOCUS ENSG00000171402

DESCRIPTION G antigen family D member 4 (Placenta-specific gene 6 protein) Protein XAGE-3). [Source:Uniprot/SWISSPROT;Acc:Q8WTP9]

TGTGGGAAAACTTCTGAAAGTTCCCACTTATCCCTCAAAAACACTTAACTTTTAGAAACAATTATTTGGCTTTTCCAGAT
GTCTTACTGTCCTTTTCTAT

In100 candidate2951 (in intron):

LOCUS ENSG00000108094

DESCRIPTION Cullin-2 (CUL-2). [Source:Uniprot/SWISSPROT;Acc:Q13617]

CTGTTTAGTTACCCAATGGTAGAGTTCATATAGGAAAAGCAAAATAACTGTTTGTTTCTTTCCTTTTTAATAACCCAGTT

TTTTGTTTTTGTTTTGTTTT

In100 candidate2952 (in intron):

LOCUS ENSG00000182646

DESCRIPTION Transmembrane protein 29.

[Source:Uniprot/SWISSPROT;Acc:Q8NDB6]EXON

TAAGTGGGAAACTCAATCATTTTGAAATAACATTCCTTTCACTGTTTTACTTGCGTTTTCTTCCTTATTATTACTCAGCC

ACTGTTTCTCTAATAATTTT

In100 candidate2953 (in intron):

LOCUS ENSG00000136709

DESCRIPTION WD repeat protein 33 (WD repeat protein WDC146).

[Source:Unipro/SWISSPROT;Acc:Q9C0J8]

TGGGTCACATGGTATTTTGACATTTAACATTTGAGGAAATTCTTGACTATTTTCCCACCTAATTTTCAGTTTATTTTATG

CCTAACAACCACTATTCTTT

In100 candidate2954 (in intron):

LOCUS ENSG00000100991

DESCRIPTION Trpc4-associated protein (Short transient receptor potential chnnel 4-associated protein) (Trp4-associated protein) (TAP1 protein) (TNFrceptor ubiquitous scaffolding/signaling protein) (Protein TRUSS). [Source:Uiprot/SWISSPROT;Acc:Q8TEL6]

TTATCAATCGTATTGATCTTTTTAAAGAACTAACTTTTGGTTTTGTTTTCTCTCTTGATTTTTTGTTTTCAATTTCAGTG ATGTTTGCTCTCATTTTTAT

In100 candidate2955 (in intron):

LOCUS ENSG00000100991

DESCRIPTION Trpc4-associated protein (Short transient receptor potential chnnel 4-associated protein) (Trp4-associated protein) (TAP1 protein) (TNFrceptor ubiquitous scaffolding/signaling protein) (Protein TRUSS). [Source:Uiprot/SWISSPROT;Acc:Q8TEL6]

ATTTTGATAAGTTATATTTTCATTTAGTTTAAAATATTTTAACTGTAAGAGTTGATTTGCCATTTTTTTGTCTGGCAGTT CTTTTAGGCAGTTTTTGCCT

In100 candidate2956 (in intron):

LOCUS ENSG00000136715

DESCRIPTION mSin3A-associated protein 130 [Source:RefSeq_peptide;Acc:NP_07821]

GATTTTCCCCTGAAAGTAACACTTCGTATAATTCCTTTTTTTATGTGCGATTTTTTTCAAGCCAGTCTCCTTTTGATGGA TGTTTTCCTAGTATCCATTT

In100 candidate2957 (in intron):

LOCUS ENSG00000187240

DESCRIPTION dynein, cytoplasmic, heavy polypeptide 2

[Source:RefSeq_peptideAcc:NP_001073932]

TGTAACTGATTTTATTTCTTATACTCTGCCATTTAGGTTGGTATTTCCTTTTATTTTTCTTAGAATTCTCTCCTTCAGGC

TTTGAGAGTTTTCCTTGCTT

In100 candidate2958 (in intron):

LOCUS ENSG00000187240

DESCRIPTION dynein, cytoplasmic, heavy polypeptide 2

[Source:RefSeq_peptideAcc:NP_001073932]

CCTGCTGTTAACTTTGATTTCTTTTCCTCCTCTTATCTAATATTTTTCACTATGATCAGTATGTTCCATGAAATATATAT

ATTCCTTATTTTTCTCTCCT

In100 candidate2959 (in intron):

LOCUS ENSG00000187240

DESCRIPTION dynein, cytoplasmic, heavy polypeptide 2

[Source:RefSeq_peptideAcc:NP_001073932]

CGGTGGTGATACCCCCTTTAACATTTTTTATTGCATCTATTTGATTCTGCTCTCTTTTCTTATTAGTGTTGCTAGCAGTC

TATCAATTTTGTTGATCTTT

In100 candidate2960 (in intron):

LOCUS ENSG00000145012

DESCRIPTION Lipoma-preferred partner (LIM domain-containing preferred

transocation partner in lipoma). [Source:Uniprot/SWISSPROT;Acc:Q93052]

CTGGTAAACTCTTATTTAAACTTCAAGATCTGTCTCTAATATTAACTTCTTGGAGAAGTTTGACCATTCTTCCAGTTCCC

ACATATTATTAGCTTATCTT

In100 candidate2961 (in intron):

LOCUS ENSG00000145012

DESCRIPTION Lipoma-preferred partner (LIM domain-containing preferred transocation partner in lipoma). [Source:Uniprot/SWISSPROT;Acc:Q93052]

CATTTTTATGATTTTTGGTGGATTTAGCATCATTTTCTATGCTTAGTAACTGCTTGCCTATTCATATCCTTTATCCAGGT

TTTTTGTTTTGTTTTGTTTC

In100 candidate2962 (in intron):

LOCUS ENSG00000095794

DESCRIPTION cAMP-responsive element modulator. [Source:Uniprot/SWISSPROT;Ac:Q03060]

CAAAAAGAATTAAAATCATCCATGGCTCTAACCACTTAGGATACCTCCTATTATAGTCTATGTACTTCCAGTCAGCTCTC

ATTTTCTCTCACTTTTTCTT

In100 candidate2963 (in intron):

LOCUS ENSG00000136731

DESCRIPTION UDP-glucose:glycoprotein glucosyltransferase 1 precursor (EC 2..1.-) (UDP-glucose ceramide glucosyltransferase-like 1) (UDP-- Glc:glycoproein glucosyltransferase) (HUGT1). [Source:Uniprot/SWISSPROT;Acc:Q9NYU2]

AAAAAAAACACTTTCTGGTAAGAGTTTTTAACCTGTGTGTATCTTTTTTCCTTTAGTTGTCAAATATTTAATTTTCAGGG

GTTTTTTTTGTTCTTTTTAT

In100 candidate2964 (in intron):

LOCUS ENSG00000188001

DESCRIPTION Protein FAM79B. [Source:Uniprot/SWISSPROT;Acc:Q6ZUI0]

TTAAGGGATGGAGTGGTAGAGGCCTGGCATTTCCTTCCCATCTCTACGTAACAATCCCAAATTTCTGTGTTTACCAGGGT

TTCTGTTACTCCCCTGATGT

In100 candidate2965 (in intron):

LOCUS ENSG00000188001

DESCRIPTION Protein FAM79B. [Source:Uniprot/SWISSPROT;Acc:Q6ZUI0]

TAACCCTTTTTATAATTATTCACTCTCACTTGATCTAAATTCTGCTCTTGATTTAACCTCAAGTCTTTTCTTCTGCAGGT

TAAGATCACTTTTTTTCACC

In100 candidate2966 (in intron):

LOCUS ENSG00000073282

DESCRIPTION Tumor protein p73-like (p73L) (p63) (Tumor protein 63) (TP63)

(51) (p40) (Keratinocyte transcription factor KET) (Chronic ulcerative stomaitis

protein) (CUSP). [Source:Uniprot/SWISSPROT;Acc:Q9H3D4]

AGCATGAAAAGAAAAGGCCATGAAAATCCCCTAACATAGACCGTTAATTTTTCTTTCCTTTTTATTTTTAAGAAACAGAC

ACTTTCTTTAACTGGACTTT

In100 candidate2967 (in intron):

LOCUS ENSG00000204711

DESCRIPTION Uncharacterized protein C9orf135.

[Source:Uniprot/SWISSPROT;AccQ5VTT2]

TTTTTAGAAAGATAACTGAATGTTTTAATCTTTCACCTGTATTTACTTTGCATACTCAGTGTAGTTAATCTATATCTCTT

TATATTCTAATTTTATTTTT

In100 candidate2968 (in intron):

LOCUS ENSG00000204711

DESCRIPTION Uncharacterized protein C9orf135.

[Source:Uniprot/SWISSPROT;AccQ5VTT2]

AAATAATGTGGGATGTATTTTTAGGGCACATAAATCATTAACCATTGCATTATATATTTATGCTTTTAATTTTTTCAGCA

TTATTTCTAATATTTCTCAT

In100 candidate2969 (in intron):

LOCUS ENSG00000198398

DESCRIPTION SRSR846 (UNQ846), mRNA [Source:RefSeq_dna;Acc:NM_207316]

TCAAATAACAACAACACTGGTCTATTTTATTATTCCCTATTTAAATTTGTTTTCAACACCAGGGTACGTTTTTCTGATCC

AATCCAATTATTTTACTTTT

In100 candidate2970 (in intron):

LOCUS ENSG00000165072

DESCRIPTION MAM domain-containing protein 2 precursor.

[Source:Uniprot/SWISPROT;Acc:Q7Z304]

ATATATTTAACCATGTCTCCAACTCTTTTTTTCAAAAGTGCTCATCCTTCAGCTTATCTTGACTCTGGACAGTCTTCATC

CTAACCTTACTGTTCTCTTT

In100 candidate2971 (in intron):

LOCUS ENSG00000196083

DESCRIPTION Interleukin-1 receptor accessory protein precursor (IL-1 receptr accessory protein) (IL-1RAcP). [Source:Uniprot/SWISSPROT;Acc:Q9NPH3]

AACTGCATTGCTTTTCATATGCTTATTTTAATTTGTATGTTTTTGTAACCATTTTTATAGCTTTCAATTGCCTTTCAGCA

TAATTTTTCACATTTTTCAC

In100 candidate2972 (in intron):

LOCUS ENSG00000152578

DESCRIPTION Glutamate receptor 4 precursor (GluR-4) (GluR4) (GluR-D) (Glutaate receptor ionotropic, AMPA 4) (AMPA-selective glutamate receptor 4). [Sorce:Uniprot/SWISSPROT;Acc:P48058]

CGAGTATCTGCTTTCTACACCCCTCATTTATCTAACTTCTTATGGCTTCCTTAGCCTTGTTTATTATTACTTCTTCAGGA

TTTCACTTTCTTCTTAGCTT

In100 candidate2973 (in intron):

LOCUS ENSG00000198887

DESCRIPTION Structural maintenance of chromosomes protein 5 (hSMC5). [Sourc:Uniprot/SWISSPROT;Acc:Q8IY18]

AGAACTCTTCCTTGACACACATAAGTAACTTTACTTAATACTACCTCTGACTTTATTTTGCATTTCCTCAGCAATATTTT

ACACCACTCTGTTTTTCTTA

In100 candidate2974 (in intron):

LOCUS ENSG00000188729

DESCRIPTION Osteocrin precursor (Musclin).

[Source:Uniprot/SWISSPROT;Acc:P6366]

CTGGAATTTGAATTTAAATTTTAACATGGTTTTGTACACTTTGATTATTTTCCTTCTCATCCTTCACAGGTTCTTGCCAA

GTCCTTCAGCTTGGTCTGTT

In100 candidate2975 (in intron):

LOCUS ENSG00000182359

DESCRIPTION Kelch repeat and BTB domain-containing protein 3 (BTB and

kelchdomain-containing protein 3). [Source:Uniprot/SWISSPROT;Acc:Q8NAB2]

ATGAGCAAAATTCATTGGCTGAGGCATTCCTAAAACTTCTTAACATTCAAAGTGTCTTTTCATTGATTTTCAACTCAGAG

TCTCTGTGTCCATGTTTTAT

In100 candidate2976 (in intron):

LOCUS ENSG00000114279

DESCRIPTION Fibroblast growth factor 12 (FGF-12) (Fibroblast growth factor

omologous factor 1) (FHF-1) (Myocyte-activating factor).

[Source:Uniprot/SWSSPROT;Acc:P61328]

GAAACTTGGTCTTTTTGTCTTTATTTTTTAACAAGTTTGTATTTTGTGAAGTTTTCTTCTTTCTCCTTGGATCAGAGTAT

ATTGTATATATTTTCCCTTT

In100 candidate2977 (in intron):

LOCUS ENSG00000152402

DESCRIPTION Guanylate cyclase soluble subunit alpha-2 (EC 4.6.1.2) (GCS-alpa-

2). [Source:Uniprot/SWISSPROT;Acc:P33402]

CAATATTGATAGGTTATTATTAACAAAATTCCATGCTTTATTTCAATATCCTTAGTTTTTAACCTTTTCCTGTTCCAGGA

TTTCATTTCACATTACATTT

In100 candidate2978 (in intron):

LOCUS ENSG00000152402

DESCRIPTION Guanylate cyclase soluble subunit alpha-2 (EC 4.6.1.2) (GCS-alpa-

2). [Source:Uniprot/SWISSPROT;Acc:P33402]

TGTTATGAATAATGTACGCAAAACTGTTGATACTGGCTAACTTAGGGGAAAGAGCTTTTTCCATTTTTAATCTTACAGTT

TTATATCTGTTCACATTTTC

In100 candidate2979 (in intron):

LOCUS ENSG00000180611

DESCRIPTION Uncharacterized protein C3orf59.

[Source:Uniprot/SWISSPROT;Acc:8IYB1]

TCACATCCGGTATCTGAAGATAGCTTGAAGTCTGTCTTTAGTCTAACCCTTGCTATTATGATTTCATTTCCCTCACAGAA

CCAATTTTTGTCCTCTTAGT

In100 candidate2980 (in intron):

LOCUS ENSG00000152404

DESCRIPTION CWF19-like 2, cell cycle control

[Source:RefSeq_peptide;Acc:NP_89647]

CTAAAGTAAGATAATTTGTTTTAACATCCACTTTGGCATTATCTTCCATAGCCTCCTTATTTGTTCTTTGTTCTGCAGCT

GAGGTAGCTTTTTTTCTCTC

In100 candidate2981 (in intron):

LOCUS ENSG00000083067

DESCRIPTION Transient receptor potential cation channel subfamily M member

(Long transient receptor potential channel 3) (LTrpC3) (Melastatin-2) (MLS2).

[Source:Uniprot/SWISSPROT;Acc:Q9HCF6]

AACATTGGTATGTACTATTAACTGAATTACAAATTTTATTTGATTTTTTTTTTTACCAGTTTTTCTTTTTTTTTTTTTA

CTAGTTTCTTTTTCTTTTCC

In100 candidate2982 (in intron):

LOCUS ENSG00000083067

DESCRIPTION Transient receptor potential cation channel subfamily M member

(Long transient receptor potential channel 3) (LTrpC3) (Melastatin-2) (MLS2).

[Source:Uniprot/SWISSPROT;Acc:Q9HCF6]

AGATTAACTCTGTTAATTCTCTCACATCTTCTTTTCTGTTTTCTATTCTTTTTTCTCATCTATTTCAGCTTTTGATTGTT

ATTTTTTTCTGATAGCTTGT

In100 candidate2983 (in intron):

LOCUS ENSG00000083067

DESCRIPTION Transient receptor potential cation channel subfamily M member

(Long transient receptor potential channel 3) (LTrpC3) (Melastatin-2) (MLS2).

[Source:Uniprot/SWISSPROT;Acc:Q9HCF6]

TATTTTTAAAAATAACAATCTCTTGTATTTTTAAATTCTACAACATTTCTGCCCTCTAATTTTTTTTAACTTTTTCAGTT

ATTTCTTATTTTATATTGCT

In100 candidate2984 (in intron):

LOCUS ENSG00000083067

DESCRIPTION Transient receptor potential cation channel subfamily M member

(Long transient receptor potential channel 3) (LTrpC3) (Melastatin-2) (MLS2).

[Source:Uniprot/SWISSPROT;Acc:Q9HCF6]

TTTTATACAACAACCCAAGAGGAGCTGGAGAGATAACTAAGGATACAATTTCATTCATTTGAAACCTTTATCTTCCAGTA

CTTCCATTTAAGTTTTTGTT

In100 candidate2985 (in intron):

LOCUS ENSG00000083067

DESCRIPTION Transient receptor potential cation channel subfamily M member

(Long transient receptor potential channel 3) (LTrpC3) (Melastatin-2) (MLS2).

[Source:Uniprot/SWISSPROT;Acc:Q9HCF6]

AGAACTATATAACCCGAACGTTGGTCTCTTTGTACACACCTTTTCTATACTGCAGATCTTCATTCACTTTCATATGTGTA

TCATTTTTATTGTCGTGTTA

In100 candidate2986 (in intron):

LOCUS ENSG00000187527

DESCRIPTION ATPase type 13A5 [Source:RefSeq_peptide;Acc:NP_940907]

TCTATTTATGGTTAGTTTTATTGTTAAGGGGTCATTTTCATAGAGAGATAACATAATCTAATTTCTGTTTTGCTGCAGTA

TCATCTCTGTTTTCATGTTC

In100 candidate2987 (in intron):

LOCUS ENSG00000136002

DESCRIPTION Rho guanine nucleotide exchange factor 4 (APC-stimulated guanin nucleotide exchange factor) (Asef). [Source:Uniprot/SWISSPROT;Acc:Q9NR80]

EXON

GAGCCAAGAGATTTAGGAAGGGAAGGGTTTTTCTTTTTTTAACTACCATTTTAATTTCTTAAGTCTTTGTTCACTCAGAT
TTTCTCTTATGCCAATTTTA

In100 candidate2988 (in intron):

LOCUS ENSG00000127249

DESCRIPTION ATPase type 13A4 [Source:RefSeq_peptide;Acc:NP_115655]

CTGTTTTTCTCCATATAGGTAGGTATCATTACTCTTTGGGTGCTTTTAACATTTTTTTTTGTCTTTATTTTTTACAGAT
TTGACTATTATGCTTCTTTT

In100 candidate2989 (in intron):

LOCUS ENSG00000127249

DESCRIPTION ATPase type 13A4 [Source:RefSeq_peptide;Acc:NP_115655]

CTTACCGAGGACTTATGTAGCCTCATTATCTGCCCAAGTAATTTCTTCTTAACTCCTGTATCAATACTTTGGTTTCAGTT
TACCTTTCTAGCTTCATTCT

In100 candidate2990 (in intron):

LOCUS ENSG00000115762

DESCRIPTION Pleckstrin homology domain-containing family B member 2 (Evecti-2). [Source:Uniprot/SWISSPROT;Acc:Q96CS7]

AACCCTGAGCGTGGCTGGGGCTGTGGTAACTAAAGACCCATTTTCTAAATTTTGCCGGCTTTATGTTCTTCAGATTGCTC
TGAGCTACTTTTCTTTCTCA

In100 candidate2991 (in intron):

LOCUS ENSG00000166266

DESCRIPTION Cullin-5 (CUL-5) (Vasopressin-activated calcium-mobilizing recetor) (VACM-1). [Source:Uniprot/SWISSPROT;Acc:Q93034]

AGCTTTCTTCTATAACTCCATTTATGTTCTATTTTATTTTCATTTCCAGCATTATCACTTTTCATTTCCTGTTTACTTTT
ATATCTTTGCCAATTATCTT

In100 candidate2992 (in intron):

LOCUS ENSG00000112812

DESCRIPTION Thymus-specific serine protease precursor (EC 3.4.-.-) (Serine rotease 16). [Source:Uniprot/SWISSPROT;Acc:Q9NQE7]

TCCTATTAACGACCGCCACCATTTTTCCAACTTTACTTCACCTGCTTTTTGTTTCTTTCATCTGCTTTATTCTTACAGAA
CTTAGCTTTTTCTTTCCTCA

In100 candidate2993 (in intron):

LOCUS ENSG00000149311

DESCRIPTION Serine-protein kinase ATM (EC 2.7.11.1) (Ataxia telangiectasia utated) (A-T, mutated). [Source:Uniprot/SWISSPROT;Acc:Q13315]

TAGCAAAGCCTATGATGAGAACTCTTTAACAACAAATTTAAACATTTATTTCCCTGAAAACCTCTTCTTTATTTTCAGAG
TGTCTTTTCTTTTTTGCTAC

In100 candidate2994 (in intron):

LOCUS ENSG00000149311

DESCRIPTION Serine-protein kinase ATM (EC 2.7.11.1) (Ataxia telangiectasia

utated) (A-T, mutated). [Source:Uniprot/SWISSPROT;Acc:Q13315]

AGAGCACTTTAACCTGGGTGTATTTCCCTTTTCTTTTCAGTGTATTTGTCTCTCCTGGCCTTCAAGATTTTATTTTCTTC

CTAATGGACCATTCTCTCCT

In100 candidate2995 (in exon):

LOCUS ENSG00000149311

DESCRIPTION Serine-protein kinase ATM (EC 2.7.11.1) (Ataxia telangiectasia

utated) (A-T, mutated). [Source:Uniprot/SWISSPROT;Acc:Q13315]

CTTCTGTACTGTCCATGTATGTTATCTTTCTGTGATAACTTCATAGATTGCCTTCTAGTTCATGAATTCTCTTGTCAGAT

GTATATAATCTCTTTTACCC

In100 candidate2996 (in intron):

LOCUS ENSG00000198105

DESCRIPTION Zinc finger protein 248. [Source:Uniprot/SWISSPROT;Acc:Q8NDW4]

EXON

TTATTTTTCTATGAACTTAACTGTTAGTATCTTTTGCCTATTTTTCTTTTGGGTTGTCTGGTCTTTTTTCCCCCCCAGTG

TCTTTTACCCTTGTGTATTA

In100 candidate2997 (in intron):

LOCUS ENSG00000129657

DESCRIPTION SEC14-like protein 1. [Source:Uniprot/SWISSPROT;Acc:Q92503]

ATACCGTATGTAACTGCTGAGGCTTTTTTTTTTTTTTAGCTTAGCATAATTCTCCCACGATTCATCCAGTTCCTCACCTG

GATCAATAGTTCATTCTTTT

In100 candidate2998 (in intron):

LOCUS ENSG00000096654

DESCRIPTION Zinc finger protein 184. [Source:Uniprot/SWISSPROT;Acc:Q99676]

EXON

GTGTCCATTGTTTCTAACAAAAAAGTTACTGTTGTTTTTATCTTTGTTTTTTTATATTTGTGTGTCTTTTTTCCTCAGAT

TACTTGTAAGATTTTTTTCT

In100 candidate2999 (in intron):

LOCUS ENSG00000096654

DESCRIPTION Zinc finger protein 184. [Source:Uniprot/SWISSPROT;Acc:Q99676]

EXON

CCTTTTAACTCTTATAAATATGAATCTGTTTCTGGATTTTCTATTTTATTTCATTTTGTTTTATTCCAGACTAGGCCATT

TTTAAATTACTTTATCTTTT

In100 candidate3000 (in intron):

LOCUS ENSG00000183840

DESCRIPTION Probable G-protein coupled receptor 39.

[Source:Uniprot/SWISSPRT;Acc:O43194]

TGAAGATTTCCCACTCTGTGGGTTGTCTGTTAACTCTGCTGCTTATTTCTTTTGCTGAGCAGAACTTTTTAGTTTAATTA

AATTCCATCTATTTATCTTT

In100 candidate3001 (in intron):

LOCUS ENSG00000166323

DESCRIPTION Uncharacterized protein C11orf65.

[Source:Uniprot/SWISSPROT;AccQ8NCR3]

GAACAATGTAAGAAGCTCATAACATGTTAACTCACTTTAGCCTCTTCACCTTTGTCCTGTTATTTCCAGATATTTCACTT

ACACTTTTGTTTTAAGTACC

In100 candidate3002 (in intron):

LOCUS ENSG00000166323

DESCRIPTION Uncharacterized protein C11orf65.

[Source:Uniprot/SWISSPROT;AccQ8NCR3]

TCTGTGTCTTTATATTTAAAGTGGGTTGCTTGTGGATAACATATAGTTGGGTCTTGATTTTTATCCGCTCTGACAGTCT

CTGATCTGCCCTTTGTAATT

In100 candidate3003 (in intron):

LOCUS ENSG00000197755

DESCRIPTION NAP5_HUMAN Isoform 3 of O14513 - Homo sapiens (Human)

[Source:Uiprot/Varsplic;Acc:O14513-3]

TACAAACTGTTAGTTTAACATGGGATTATATCATTTTGGCCTTTTCTTGTGGCAGGAATATTCCATTTGTCCATTAAGCT

CACGTCCGTTTTCTTCACTT

In100 candidate3004 (in intron):

LOCUS ENSG00000107362

DESCRIPTION

ATGTGTTTTTTTTCTTTAGTTCATTAATGTGAATTACTTTGGTTTAACATTGGAAGATTTGTCATTCTTGTCTTTCAGCT

TGATTTTGTATTTTGTATTT

In100 candidate3005 (in intron):

LOCUS ENSG00000114331

DESCRIPTION Centaurin-beta 2 (Cnt-b2). [Source:Uniprot/SWISSPROT;Acc:Q15057

TCTTCTCTTTTCTTCCCTTTCTCTTAACATCTATCTCCCTTATTACTTATTCTCTGTTTTGCTCATTTTGCTGCTCAGTC

ATATATTTTATTTCATTATT

In100 candidate3006 (in intron):

LOCUS ENSG00000114331

DESCRIPTION Centaurin-beta 2 (Cnt-b2). [Source:Uniprot/SWISSPROT;Acc:Q15057

ACTAATAATGGGTTAAATCTACTTGGTATTCTATAACCTTCTTTTACTTGAAGTTTTGTATCTTTCTCTCAGTTTCAGAA

ATTTTCTGTTACTATCCTTT

In100 candidate3007 (in intron):

LOCUS ENSG00000114331

DESCRIPTION Centaurin-beta 2 (Cnt-b2). [Source:Uniprot/SWISSPROT;Acc:Q15057

TTATAAATGTTCTGTGGTAACGGTTGAAGTTCTTTTTCCTCTCTATTATTATTAAATTCTTTGCATGTGGCTCTCCAGCT

TGCTCTGTACATTTTCCCAT

In100 candidate3008 (in intron):

LOCUS ENSG00000114331

DESCRIPTION Centaurin-beta 2 (Cnt-b2). [Source:Uniprot/SWISSPROT;Acc:Q15057

ATGTGTGTCTGTGTGTGTATGTGTGACTTCTTTTGCTTGGAATAACATTTTTGAGATCGGTATTGTTCCCTGTTTCAGTA

GTTCTCTCTTTTTAATACTT

In100 candidate3009 (in intron):

LOCUS ENSG00000114331

DESCRIPTION Centaurin-beta 2 (Cnt-b2). [Source:Uniprot/SWISSPROT;Acc:Q15057

CTGACATTTCAAAAGCCCTAAGTATCTGATTAACTACCTGATTTCCTTCCTATTTTTTCCTCCTAAATTTTTTCCCAGTA

ACGTTTTTTCCTGCCCTTTT

In100 candidate3010 (in intron):

LOCUS ENSG00000178105

DESCRIPTION Probable ATP-dependent RNA helicase DDX10 (EC 3.6.1.-) (DEAD bo

protein 10). [Source:Uniprot/SWISSPROT;Acc:Q13206]

GTTGTCCTCTTGAAAACTTTTTTCCCTCTGACCCTAATTTCTTAACCTTCTGACCCTTATAATCTGTTTTTCTCCCAGTA

CTTGTTTTCTGGCTTTTCCC

In100 candidate3011 (in intron):

LOCUS ENSG00000178105

DESCRIPTION Probable ATP-dependent RNA helicase DDX10 (EC 3.6.1.-) (DEAD bo

protein 10). [Source:Uniprot/SWISSPROT;Acc:Q13206]

TGCTTGCTGTTTTTACTATCGACTCTGTGATCGATCTCTTAACCTTTTCCTTTTTTGTTTTGGTTTTATCAGTACCTTTA

ATGTAATTGCATTCCTTTCT

In100 candidate3012 (in intron):

LOCUS ENSG00000178105

DESCRIPTION Probable ATP-dependent RNA helicase DDX10 (EC 3.6.1.-) (DEAD bo

protein 10). [Source:Uniprot/SWISSPROT;Acc:Q13206]

ATTTATCTTTTGCTTCCAACCTGATGACATGCAAATAACTTGATTTTGTTTTGTTTGTCTTTTGTTTTTCAGATTGTCAT

TTGTTTTGGGTCATATTTTT

In100 candidate3013 (in intron):

LOCUS ENSG00000178105

DESCRIPTION Probable ATP-dependent RNA helicase DDX10 (EC 3.6.1.-) (DEAD bo

protein 10). [Source:Uniprot/SWISSPROT;Acc:Q13206]

TCTCCACCTATAACTCTTTATGTAGATCATTGCATGAGTTCCTCCTTCTTTTCTTTTAGATCTTTGCACCTTCTTCAGAA

AGGCCTTTCCTATTTTTCTA

In100 candidate3014 (in intron):

LOCUS ENSG00000178105

DESCRIPTION Probable ATP-dependent RNA helicase DDX10 (EC 3.6.1.-) (DEAD bo

protein 10). [Source:Uniprot/SWISSPROT;Acc:Q13206]

TTTCTCCCACTCTGTGGGTTGTCTGTTAACTCTGCTGATTTCTTTTGCTGTGCAGAAGCTTTTTAGCTTTAATTAAGTCC

CATCTATTTATCTTTGTTTT

In100 candidate3015 (in intron):

LOCUS ENSG00000082258

DESCRIPTION Cyclin-T2 (CycT2). [Source:Uniprot/SWISSPROT;Acc:O60583]

TTTTGGTTTGATAGGCTCACACTTGGCAAATGCCAATTAACAAATGGGCATTTTGACTTATTCCACTTCCTTTGTCAGTT

TGTTACCTCTCTTGTCTTTC

In100 candidate3016 (in intron):

LOCUS ENSG00000137714

DESCRIPTION Adrenodoxin, mitochondrial precursor (Adrenal ferredoxin)

(Ferrdoxin- 1) (Hepatoredoxin). [Source:Uniprot/SWISSPROT;Acc:P10109]

GGAGTAACTTTTTTTTTTTTCTTAAATAGCCTCTTGTTCTTCTTACAGTTTTAGGCTCTTTTTTATACCCAAATATTTA

CCTTATTTTTGCAGGTTTTT

In100 candidate3017 (in intron):

LOCUS ENSG00000115839

DESCRIPTION Rab3 GTPase-activating protein catalytic subunit (RAB3

GTPasectivating

protein 130 kDa subunit) (Rab3-GAP p130) (Rab3-GAP).

[Source:Unipot/SWISSPROT;Acc:Q15042]

AACCCATGTTTACACACTTAAATGACTATCCATAAGACACTAACATCATTCTATTCTCTTATCTCCGTGAGTTCAGTTTG

TTTGTTTTTTTAGCTCCCAC

In100 candidate3018 (in intron):

LOCUS ENSG00000115839

DESCRIPTION Rab3 GTPase-activating protein catalytic subunit (RAB3

GTPasectivating

protein 130 kDa subunit) (Rab3-GAP p130) (Rab3-GAP).

[Source:Unipot/SWISSPROT;Acc:Q15042]

AATAATAATTTGAAACATGGATTCTGTTAACTTTATTATCCATGCCTATATTCTTTATTTACCAGGCATAATTTACTAGC

TTTGGTTTTTGTTTTGTTTT

In100 candidate3019 (in intron):

LOCUS ENSG00000121988

DESCRIPTION Zinc finger Ran-binding domain-containing protein 3 (EC 3.6.1.-.

[Source:Uniprot/SWISSPROT;Acc:Q5FWF4]

TATGTTTTTTATTATTAATTAAACCCAAACTAACTTCTACTTGGGTAAATCTCTTCTCAATACTTTCAGACACATTAAGT

ATTCTATCATTTTTATCTTT

In100 candidate3020 (in intron):

LOCUS ENSG00000170145

DESCRIPTION Serine/threonine-protein kinase SNF1-like kinase 2 (EC 2.7.11.1

(Qin- induced kinase) (SIK2). [Source:Uniprot/SWISSPROT;Acc:Q9H0K1]

TTGCCTTAACCCTAATTTAGTCCCTCATTAATTCATTTTCAGCTCATGATTAGGATCCCAAGTGGTTTCTTGCCTCTTTA

TTTCCCTTATCAGTCTTTCC

In100 candidate3021 (in intron):

LOCUS ENSG00000204201

DESCRIPTION

ATAATTTAGTAACTTTAGAAATCACTTTCTCATTCTTCTCTAGACTTTGCTGTTCTTAATTACTGAAGTCTTCAGTATCC

ATTTGTTTTTATGAATTTTT

In100 candidate3022 (in intron):

LOCUS ENSG00000196693

DESCRIPTION Zinc finger protein 11B. [Source:Uniprot/SWISSPROT;Acc:Q06732]

EXON

AAAGCAAACATAATGACCCTGGCTTTTATATTTGTCTATATAGTTAACTTTATTGGAAGTCTTTATTTTTTCCTCCAGAT

TTGAGTCTAGTTTCTTTTTA

In100 candidate3023 (in intron):

LOCUS ENSG00000165091

DESCRIPTION Transmembrane channel-like protein 1 (Transmembrane cochlearexressed protein 1). [Source:Uniprot/SWISSPROT;Acc:Q8TDI8]

TCTTAGGTTTGGTCATTTAACATAATCCCAAACTTCTTGGAGGCTTTATTCATTTTTTAAAATTCTTTTACTTTCCAGTA

CATTTTGCATTTCTCTGTGT

In100 candidate3024 (in intron):

LOCUS ENSG00000086848

DESCRIPTION Alpha-1,2-mannosyltransferase ALG9 (EC 2.4.1.-) (Asparagine-lined glycosylation protein 9 homolog) (Disrupted in bipolar disorder protein). [Source:Uniprot/SWISSPROT;Acc:Q9H6U8]

TAGAAATGTAAGTAGGTTAATTTCTCAAATGTTTAAGTTCCTAAAATCTAACCCTGCTTTTTTTATGGTTTTTCTCAGAT

CATTTGTTCTTTCTTCCATT

In100 candidate3025 (in intron):

LOCUS ENSG00000165092

DESCRIPTION Retinal dehydrogenase 1 (EC 1.2.1.36) (RalDH1) (RALDH 1) (Aldehde dehydrogenase family 1 member A1) (Aldehyde dehydrogenase, cytosolic) (AHDII) (ALDH-E1). [Source:Uniprot/SWISSPROT;Acc:P00352]

GCCCTTGAAATAATGCCTGGACATGGGAAATGATTTAACGTTAGTGATCCTAGTCCTATTTTTCTGAATCCTTTCCAGTT

TTTCCAGAATATTCCCTTTT

In100 candidate3026 (in intron):

LOCUS ENSG00000072274

DESCRIPTION Transferrin receptor protein 1 (TfR1) (TR) (TfR) (Trfr) (CD71 atigen) (T9) (p90). [Source:Uniprot/SWISSPROT;Acc:P02786]

GTAGCATAACTGGTCTTGTCTGCCTTAGTCTTAATTTTGTCTTTTTGTATGTGTCACTTTCTTTTTTTTTGAGACAGAG
TTTTGCTCTTGTTTTCCAGG

In100 candidate3027 (in intron):

LOCUS ENSG00000196632

DESCRIPTION Serine/threonine-protein kinase WNK3 (EC 2.7.11.1) (Protein kinse with no lysine 3) (Protein kinase, lysine-deficient 3). [Source:Uniprot/WISSPROT;Acc:Q9BYP7]

AGAGAAATAACTTACATTCCCTATGTTCTTGGTTTATGTTCTTTCTTTCCATCTTTTTCCCAGTCTGTCTTTTTTCTAAC
TTGCTGTCTTTTACTCTTTT

In100 candidate3028 (in intron):

LOCUS ENSG00000196632

DESCRIPTION Serine/threonine-protein kinase WNK3 (EC 2.7.11.1) (Protein kinse with no lysine 3) (Protein kinase, lysine-deficient 3). [Source:Uniprot/WISSPROT;Acc:Q9BYP7]

TTGTTTAACGTAATATTTTCTGAACTGTCTCTTCTTTCATTCTTTTCAGGAATGTTTAACCTAATTAGTGGTCTTCCGAG

CTATTTTTTTTTTTTTTTTT

In100 candidate3029 (in intron):

LOCUS ENSG00000196632

DESCRIPTION Serine/threonine-protein kinase WNK3 (EC 2.7.11.1) (Protein kinse with no lysine 3) (Protein kinase, lysine-deficient 3).

[Source:Uniprot/WISSPROT;Acc:Q9BYP7]

AAGAGTCCTAACATTTTATTTCCCTTAGTGGAGAAATGTTCTTTTTTCATTTCTCTTCTTTTTCTCCCTCCCACCCAGAA

ATGTTCTTTTTTTTGTTGTT

In100 candidate3030 (in intron):

LOCUS ENSG00000144229

DESCRIPTION Thrombospondin type-1 domain-containing protein 7B precursor.

[ource:Uniprot/SWISSPROT;Acc:Q9C0I4]

CTCTTTTTTTTCCTAACCTGGCTAGAGGTTCATTAATTTTATCAGTGCTTTTAAAAACCAGCTTTTGGTTTTGTTGATTT

TTTTCTATTGATTTTTTTTC

In100 candidate3031 (in intron):

LOCUS ENSG00000144229

DESCRIPTION Thrombospondin type-1 domain-containing protein 7B precursor.

[ource:Uniprot/SWISSPROT;Acc:Q9C0I4]

TTCTCATATATCAATGATGCTATTATTGACTTTTTATAATACCTTAACTTGTGAAATGCAATAGTCTTCTCTCTTCAGTT

TTAATTTTTGTTTTTGTTTC

In100 candidate3032 (in intron):

LOCUS ENSG00000144229

DESCRIPTION Thrombospondin type-1 domain-containing protein 7B precursor.

[ource:Uniprot/SWISSPROT;Acc:Q9C0I4]

AGTATTGTGTGTTTATTTCATAAAAGTAAGATGGAGTGTTAACTCCTGGTCTTTGCTTGTTATTTTCGTTCCCAGGTTGA

CTTTCCACATGTGCTTACTT

In100 candidate3033 (in intron):

LOCUS ENSG00000144229

DESCRIPTION Thrombospondin type-1 domain-containing protein 7B precursor.

[ource:Uniprot/SWISSPROT;Acc:Q9C0I4]

TCCACTGCTTTAATATTGTTAGTTTGATGATGTTTTAACACCTAGTATGCTATTTTTTTCATTGTACAGTTATTTTCAAA

ATTTTAGATATCTTCTCTTC

In100 candidate3034 (in intron):

LOCUS ENSG00000144228

DESCRIPTION Speckle-type POZ protein-like (HIB homolog 2) (Roadkill

homolog2). [Source:Uniprot/SWISSPROT;Acc:Q6IQ16]

AAATAAGATTCACCATCTGCTTATCCTAACTTTCGCTTGTCTCTTTCCTGACTGAACTCTGTCATTCAGTGATTCTTTCC

AGGCTTCTGATGCTTTGTCT

In100 candidate3035 (in intron):

LOCUS ENSG00000163961

DESCRIPTION RING finger protein 168. [Source:Uniprot/SWISSPROT;Acc:Q8IYW5]

EXON

AGGTAACTAATGATAGTGTTCTTTTTCATGTGCTTATTGACAGTTTTGCCCTCTTCCATTGTAAAGTGTCTGTTTAATTC

TTTTTCCCCTAAATTGTTTA

In100 candidate3036 (in intron):

LOCUS ENSG00000168702

DESCRIPTION Low-density lipoprotein receptor-related protein 1B precursor

(ow- density lipoprotein receptor-related protein-deleted in tumor) (LRP- DI).

[Source:Uniprot/SWISSPROT;Acc:Q9NZR2]

CATCTATTTTGAGGAGTCCTGAAATGGTGAATCCTTTTATGTTGCTTAACTAGAATAGAAGCTGTATTCTTTCAGGCCAT

TGCATTGGTCTTATCTGTTT

In100 candidate3037 (in intron):

LOCUS ENSG00000168702

DESCRIPTION Low-density lipoprotein receptor-related protein 1B precursor

(ow- density lipoprotein receptor-related protein-deleted in tumor) (LRP- DI).

[Source:Uniprot/SWISSPROT;Acc:Q9NZR2]

ATATGAGTTTCATTTATGTTTCTACCTTTTTTATAGCCTACAACATAGATAACCGGAAAAATCTTTCTATTGTTACAGTT

TTGTATTTTTTCCTGAGCCA

In100 candidate3038 (in intron):

LOCUS ENSG00000168702

DESCRIPTION Low-density lipoprotein receptor-related protein 1B precursor

(ow- density lipoprotein receptor-related protein-deleted in tumor) (LRP- DI).

[Source:Uniprot/SWISSPROT;Acc:Q9NZR2]

AAGGATTTAAAAAATTCTTATCACACATTCTGTTTATACTTTTAACAAATCAATACATTCTAGCACTGTTTTCCTCAGCT

TGTATTTTTTGTCTTGACAT

In100 candidate3039 (in intron):

LOCUS ENSG00000168702

DESCRIPTION Low-density lipoprotein receptor-related protein 1B precursor

(ow- density lipoprotein receptor-related protein-deleted in tumor) (LRP- DI).

[Source:Uniprot/SWISSPROT;Acc:Q9NZR2]

ATCAAGCTTATGTTTCTAACTACTATACCATACTGCCTTGTTAACTCTTACTTAACTCTTTAGTTTTGTACCTCTCAGTT

TGTTTAATATCTCTTTATTG

In100 candidate3040 (in intron):

LOCUS ENSG00000168702

DESCRIPTION Low-density lipoprotein receptor-related protein 1B precursor

(ow- density lipoprotein receptor-related protein-deleted in tumor) (LRP- DI).

[Source:Uniprot/SWISSPROT;Acc:Q9NZR2]

TCTAAATGGGAATTTTAATCTTTATCTTATTGGATTCATGCATTGCATATAACCCTTTTGAACATTCTGATATCCCAGAA

GAGCTCTCATTTCTTATTTT

In100 candidate3041 (in intron):

LOCUS ENSG00000168702

DESCRIPTION Low-density lipoprotein receptor-related protein 1B precursor (ow- density lipoprotein receptor-related protein-deleted in tumor) (LRP- DI). [Source:Uniprot/SWISSPROT;Acc:Q9NZR2]

TTGCCACTCTGTCTTTCACAATGGTTGAACTAATTTACATTTCCACTAACATGTAAAAGCATTCTTACCTCTTGGCAGCC
TCTGTTGTTTCTTGACTTTT

In100 candidate3042 (in intron):

LOCUS ENSG00000168702

DESCRIPTION Low-density lipoprotein receptor-related protein 1B precursor (ow- density lipoprotein receptor-related protein-deleted in tumor) (LRP- DI). [Source:Uniprot/SWISSPROT;Acc:Q9NZR2]

GCAATATTTGTCTCTTCCCTTAACTTTCTCCTTTTCTTTTTCCTTCCTCTTAACTGCTTACAGTAGATGTACCTTCATCC
TTTTATACTGCCATTTCCTT

In100 candidate3043 (in intron):

LOCUS ENSG00000168702

DESCRIPTION Low-density lipoprotein receptor-related protein 1B precursor (ow- density lipoprotein receptor-related protein-deleted in tumor) (LRP- DI). [Source:Uniprot/SWISSPROT;Acc:Q9NZR2]

AAGTAAACAAATTTGCAAATATGAAATCTGAATAATGAGGATTAACTGTACTTGTTTGCTATTTGCATTTTTTTTCAGCT
TTCCTCTGTCTTGGTTTTTC

In100 candidate3044 (in intron):

LOCUS ENSG00000168702

DESCRIPTION Low-density lipoprotein receptor-related protein 1B precursor (ow- density lipoprotein receptor-related protein-deleted in tumor) (LRP- DI).

[Source:Uniprot/SWISSPROT;Acc:Q9NZR2]

AGCATATTTATAGTAACATTGCAAATAACATTATATAATTTTTTTATCTCAGCCTGAATCAGATTGTATTTTCACATTTT

TTTGTTCTTATTCTCTATTT

In100 candidate3045 (in intron):

LOCUS ENSG00000168702

DESCRIPTION Low-density lipoprotein receptor-related protein 1B precursor

(ow- density lipoprotein receptor-related protein-deleted in tumor) (LRP- DI).

[Source:Uniprot/SWISSPROT;Acc:Q9NZR2]

AGTTTCCATCCGGATTTTAATAGTATCTCTCTATTTTCTGTGAAATTAACCTCAAACCTTTCTGTCTGCTATTCAGAGCC

TTCTTCATTTTTGCTCATTT

In100 candidate3046 (in intron):

LOCUS ENSG00000168702

DESCRIPTION Low-density lipoprotein receptor-related protein 1B precursor

(ow- density lipoprotein receptor-related protein-deleted in tumor) (LRP- DI).

[Source:Uniprot/SWISSPROT;Acc:Q9NZR2]

AGAGACTGCCTATAACTTCCAAATTCATAGTTTGTTTTCAGGGATTATTTTTTCTTTTCTTTTTCTTTTTTTTTTTTTTT

TGGAAACAGAGTTTTGCTCT

In100 candidate3047 (in intron):

LOCUS ENSG00000168702

DESCRIPTION Low-density lipoprotein receptor-related protein 1B precursor

(ow- density lipoprotein receptor-related protein-deleted in tumor) (LRP- DI).

[Source:Uniprot/SWISSPROT;Acc:Q9NZR2]

TTAACATTTGTAGCACTTTTTTCTTTATGCTTTTTCCTCTCTTTTCCTATCTCCTCTGGTTTCAGTGGAATATTTTGTAT

AATTTCATTTTTTCCTCTTT

In100 candidate3048 (in intron):

LOCUS ENSG00000168702

DESCRIPTION Low-density lipoprotein receptor-related protein 1B precursor

(ow- density lipoprotein receptor-related protein-deleted in tumor) (LRP- DI).

[Source:Uniprot/SWISSPROT;Acc:Q9NZR2]

TAAAATTGTTTTCTTCTATGTAACATGTCATTTCCCCCTCGCTGCTATTAAGATTTGCTTCTTGTCTCTAGTTTTCAGAA

GTTTATTTTTATTTTTAATC

In100 candidate3049 (in intron):

LOCUS ENSG00000168702

DESCRIPTION Low-density lipoprotein receptor-related protein 1B precursor

(ow- density lipoprotein receptor-related protein-deleted in tumor) (LRP- DI).

[Source:Uniprot/SWISSPROT;Acc:Q9NZR2]

AACACTTATTATGAGGTTATTACTGAAATATCTGACTATAACTGGGTATTAGTCTATTTCTCTCTTCAGTTCTACCAGTT

TTCACTTATCTTTTGAAATT

In100 candidate3050 (in intron):

LOCUS ENSG00000168702

DESCRIPTION Low-density lipoprotein receptor-related protein 1B precursor

(ow- density lipoprotein receptor-related protein-deleted in tumor) (LRP- DI).

[Source:Uniprot/SWISSPROT;Acc:Q9NZR2]

ACTAGCTATTAAATAGTGTTTACTATTACTTAACTCTTACTAGTTACTATTTGTTTTACCAGATATTAACTTCATGCTGT

ATGTATTTCCCTTCAGTTTT

In100 candidate3051 (in intron):

LOCUS ENSG00000198963

DESCRIPTION Nuclear receptor ROR-beta (Retinoid-related orphan receptor-bet)

(Nuclear receptor RZR-beta). [Source:Uniprot/SWISSPROT;Acc:Q92753]

AGAGGTTTATCTTTATACATAACTGATCAAATAATCTTCTATTTCCCATCTTACATTTCACATTTTATTAAATCAGTCAG

ATTTTCTTCCTGGTCCCTTT

In100 candidate3052 (in intron):

LOCUS ENSG00000198963

DESCRIPTION Nuclear receptor ROR-beta (Retinoid-related orphan receptor-bet)

(Nuclear receptor RZR-beta). [Source:Uniprot/SWISSPROT;Acc:Q92753]

TGTTATATCACTTTAACACTTCAAGATGGTTTACTTCAATGTTTATCCTCAAATTACCTTCTCCATTTTACCTCACAGAT

ATTTTCCCTTAATATTTCTT

In100 candidate3053 (in intron):

LOCUS ENSG00000169826

DESCRIPTION Chondroitin beta-1,4-N-acetylgalactosaminyltransferase 2 (EC 2..1.174) (GalNAcT-2) (beta4GalNAcT-2). [Source:Uniprot/SWISSPROT;Acc:Q8N6G5]EXON

AGATAACGACTTAACTGCCAAATTCTTTTCTGTTATTCTTTTTCATTGCTTTTTTACCTTTGACAACCTAACCAGTATTT
CTGTCTTAAAATGTTCTTCT

In100 candidate3054 (in intron):

LOCUS ENSG00000119121

DESCRIPTION Transient receptor potential cation channel subfamily M member (EC 2.7.11.1) (Channel kinase 2) (Melastatin-related TRP cation channel 6) [Source:Uniprot/SWISSPROT;Acc:Q9BX84]

TGTCTGGCCTAACCTGTGGTTTTCTATCTTCCCTTTACTTTTCTCTCTTTTGATTTGGCATGCCCTGCCCAGTGTCTCCT
ATCAAATTTTGCCCATGTTT

In100 candidate3055 (in intron):

LOCUS ENSG00000119121

DESCRIPTION Transient receptor potential cation channel subfamily M member (EC 2.7.11.1) (Channel kinase 2) (Melastatin-related TRP cation channel 6) [Source:Uniprot/SWISSPROT;Acc:Q9BX84]

AGTGCTATCAAATTCCCTCTAACCACTGTTTTAGCTCTTTCCTACAAATTTTGATGTGTTGTTTTTATTTTCATTCAGTG
AGTCTTTTTATATTTCGTTT

In100 candidate3056 (in intron):

LOCUS ENSG00000075711

DESCRIPTION Disks large homolog 1 (Synapse-associated protein 97) (SAP-97) hDlg). [Source:Uniprot/SWISSPROT;Acc:Q12959]

TGTACTGTACATACTTTAATATAGTAGTTTGTGATGTCTCCTAACTTTTTATATTATTGATTTTTTATCTTTTCCCAGGG

CCTTTTAAATTCCTTTTGTT

In100 candidate3057 (in intron):

LOCUS ENSG00000075711

DESCRIPTION Disks large homolog 1 (Synapse-associated protein 97) (SAP-97)

hDlg). [Source:Uniprot/SWISSPROT;Acc:Q12959]

TATTTAATAATGTTTAACTTCCAAGCTAAACTTCCTTTTTTGTGCTTTTTTTCTGATTTGCTAATCCAGTATTTGTTTAG

TCTGAACCTAGTTCTTTTTC

In100 candidate3058 (in intron):

LOCUS ENSG00000156017

DESCRIPTION Uncharacterized protein C9orf41.

[Source:Uniprot/SWISSPROT;Acc:8N4J0]

TTTAAAATTAATAACTGTTTCTTGTATCTTTTTCTGTCTCTTAAATCTTTATGCCTAACTTTTTAGATTTTTCAGCCTTT

TTGTGGCAATCTTTATGTTT

In100 candidate3059 (in intron):

LOCUS ENSG00000115919

DESCRIPTION Kynureninase (EC 3.7.1.3) (L-kynurenine hydrolase).

[Source:Unirot/SWISSPROT;Acc:Q16719]

TTTCTGTAAACAATGCTTTAACCCTGAAAGAACATTCTTATTTTATTTTTCTTCTACAAGACTTTTGGTCAGTTAGTTCA

TCTCTTTGCACATCAGTTTC

In100 candidate3060 (in intron):

LOCUS ENSG00000075884

DESCRIPTION ARHGAP15 [Source:RefSeq_peptide;Acc:NP_060930]

AAATATATCAATATGCATTTGTTTCCTGATCTTGTATATTCTATATTATAACTGTTCATGAGACCTAATTATTTTCAGGG

TACTTTTTCTTTTCCCCTTA

In100 candidate3061 (in intron):

LOCUS ENSG00000075884

DESCRIPTION ARHGAP15 [Source:RefSeq_peptide;Acc:NP_060930]

AACAAAAAAGGCATTATATCATAAATATGTATAGCTGCTAACTCAATAAACTCCTATTTCTCTGACATCTATCTACAGTG

TTGCATCCTCTTATTCTTAC

In100 candidate3062 (in intron):

LOCUS ENSG00000075884

DESCRIPTION ARHGAP15 [Source:RefSeq_peptide;Acc:NP_060930]

TCACTAAAAACTATTTTTACCACAAATATTTAACATTTTCCAAAATTTTATAAATTGTTTCTTGTCTTCCAGTTTCATGT

TGTTGGAGGTTTTTTTTCTT

In100 candidate3063 (in intron):

LOCUS ENSG00000075884

DESCRIPTION ARHGAP15 [Source:RefSeq_peptide;Acc:NP_060930]

AGGGTAGAACTTCGGGTGTATTTGTATCAAAAGCCTATACTCTTAACCATTATGTTCATATCTTGTATCTCAGAATTAAA

CTTTCCTAATTATTTTCTCC

In100 candidate3064 (in intron):

LOCUS ENSG00000204409

DESCRIPTION

TTGCCCGTAAAATCCCATCCTACCTCCCCATTCCACTATTAACGTTTTGTACAATTTTTATGCATCCTTCAGTTGTGTAG

TTACTGCTCTATATTTTTCT

In100 candidate3065 (in intron):

LOCUS ENSG00000025293

DESCRIPTION PHD finger protein 20 (Hepatocellular carcinoma-associated antien

58) (Glioma-expressed antigen 2) (Transcription factor TZP) (Novel zinc inger

protein). [Source:Uniprot/SWISSPROT;Acc:Q9BVI0]

CTGAAACACAAATTTTATGAAGTAGTATTTAACCTTAATACATGCATTATACTCTGGTATTTTTTTCAGTTCTTTTTTTT

TTCGTTTGGGACAGAGTCTT

In100 candidate3066 (in intron):

LOCUS ENSG00000169554

DESCRIPTION Zinc finger homeobox protein 1b (Smad-interacting protein 1)

(SADIP1). [Source:Uniprot/SWISSPROT;Acc:O60315]

AGTACAACAACTAACATAGACTAGATGCTCACCCTGTTTTGTTTTGCCAGTTTTTTTTTTTTTTGTTTGTTTGTTTTTT

TTTGAGATGGAGTCTCGCTC

In100 candidate3067 (in intron):

LOCUS ENSG00000115947

DESCRIPTION Origin recognition complex subunit 4.

[Source:Uniprot/SWISSPROTAcc:O43929]

ACATACTGACACACTAATTCTTTGAAAAGAAATTTAGAACTCATATCTAACAATCCCTCCCCTTGTATTTCCTTACAGCT

TTCTTTTCAAACTTTTCTTA

In100 candidate3068 (in intron):

LOCUS ENSG00000141946

DESCRIPTION Zinc finger imprinted 3. [Source:Uniprot/SWISSPROT;Acc:Q96PE6]

EXON

AAAAATGGAAAATAGAAAAAAAAATATAATAACATTTTCTTTTCTTTTCTTTTTCTTTTTCTTTTTTTTCTTGAGACAGAG

TTTTGCTCTTGTTGCCCTGG

In100 candidate3069 (in intron):

LOCUS ENSG00000141564

DESCRIPTION Regulatory-associated protein of mTOR (Raptor) (P150 target of

apamycin (TOR)-scaffold protein). [Source:Uniprot/SWISSPROT;Acc:Q8N122]

TTCTTTAGTGTCTTCAAGTTCTAACTTTTAGGCTTTGTGGATTTACTGCATTATTTTATCTTTATTTTTCTATTCCAGTA

CTTTATTTTGTTTCCTTTCT

In100 candidate3070 (in intron):

LOCUS ENSG00000204527

DESCRIPTION double homeobox A [Source:RefSeq_peptide;Acc:NP_001012747]

TATCGTGCCCTAGATAACGTGGCGCTTGGTATGGTCTGTGACCTCGTCTGCTTCCCTTTTATTCTTTTTTTTCAGATGTT

CTGGCCTCCTTGCCTTATGT

In100 candidate3071 (in intron):

LOCUS ENSG00000048028

DESCRIPTION Ubiquitin carboxyl-terminal hydrolase 28 (EC 3.1.2.15) (Ubiquitn thioesterase 28) (Ubiquitin-specific-processing protease 28) (Deubiquitinting enzyme 28). [Source:Uniprot/SWISSPROT;Acc:Q96RU2]

ATTTTTTTCTTTTAACTGTTAAGATAGTGAATTTTACTGCTCCATCTAGTATTAACTCATTTTTTTTTCCTGCAGTAAAC
TCAACTTGGTCTTTTTTTTT

In100 candidate3072 (in intron):

LOCUS ENSG00000197062

DESCRIPTION zinc finger protein 187 [Source:RefSeq_peptide;Acc:NP_689949]

AACTTGCTCACACTGATTAACCCTCTTTTGTTTGTTATTAGTGCTGCTTAGAGTCTTTCTTCTATTTTCTCCATCCAGCT
TTCCTTTTACTCTCTCATTC

In100 candidate3073 (in intron):

LOCUS ENSG00000158571

DESCRIPTION 6-phosphofructo-2-kinase/fructose-2,6-biphosphatase 1 (6PF-2-K/ru- 2,6-P2ASE liver isozyme) [Includes: 6-phosphofructo-2-kinase (EC 2.7.1.05); Fructose-2,6-bisphosphatase (EC 3.1.3.46)]. [Source:Uniprot/SWISSPROT;cc:P16118]

TAAACAAATTCCTTGTCTGATATTTCCAACATCTGTGTCATAACTTACTCTGGTCCTAATGATTGCTTTTCTCTTCAGAC

ATTGTTTTTATTCCTTGCTT

In100 candidate3074 (in intron):

LOCUS ENSG00000158571

DESCRIPTION 6-phosphofructo-2-kinase/fructose-2,6-biphosphatase 1 (6PF-2-K/ru- 2,6-P2ASE liver isozyme) [Includes: 6-phosphofructo-2-kinase (EC 2.7.1.05); Fructose-2,6-bisphosphatase (EC 3.1.3.46)]. [Source:Uniprot/SWISSPROT;cc:P16118]

TTCTTCATGTCTCCTAACTTTTCCTTCCTATTTTCTATCTCTTTGTATCTTTTGCTGCATCCTGAGTAATTTCTTCAGCT

TTATCTTCTAGTCACTATTT

In100 candidate3075 (in intron):

LOCUS ENSG00000185621

DESCRIPTION leishmanolysin-like (metallopeptidase M8 family) [Source:RefSeqpeptide;Acc:NP_149018]

ATAAACTGCCTCCTAAAAACTCTTGAAAAATTTATTTAAAATGTTGTAACTTTATGCATTAGTTCTGTGTTTTGTCAGCT

CTTAAAACTTCTTTTTGCTC

In100 candidate3076 (in intron):

LOCUS ENSG00000165406

DESCRIPTION E3 ubiquitin-protein ligase MARCH8 (EC 6.3.2.-) (Membraneassocated RING finger protein 8) (Membrane-associated RING-CH protein VIII) (MARH-VIII) (RING finger protein 178) (Cellular modulator of immune recognition (c-MIR). [Source:Uniprot/SWISSPROT;Acc:Q5T0T0]

CTGTTTTTTAGTGGGTAACATATGGAGACCTTGTGGTGTTTCTCTGTCATCTTCCCTGAAATTTCTCAGAGCTTTCAAGT

TTAATTTTGGTCTTCGTTTC

In100 candidate3077 (in intron):

LOCUS ENSG00000172671

DESCRIPTION AN1-type zinc finger and ubiquitin domain-containing protein 1

AN1- type zinc finger protein 4). [Source:Uniprot/SWISSPROT;Acc:Q86XD8]

GGGTCAATTTTCACTATACACTTGAGTCTTTTTAACATAAAGATTCAAGTCTTTTATTTCAGGATTATTTCTTAGAATTT

ATATGTTTTTTTTTTTTAGT

In100 candidate3078 (in intron):

LOCUS ENSG00000166741

DESCRIPTION Nicotinamide N-methyltransferase (EC 2.1.1.1).

[Source:Uniprot/WISSPROT;Acc:P40261]

AGTTACATATAACTAATATACATGATATATCTACATATCTTTTTCTACCCTTCTGTTTTCAGATTATGTTTTCTCTTTTT

AATCCAGACTGACAATTTCT

In100 candidate3079 (in intron):

LOCUS ENSG00000158691

DESCRIPTION Zinc finger protein 96 (Zinc finger protein 305) (Zinc finger ad

SCAN domain-containing protein 12). [Source:Uniprot/SWISSPROT;Acc:O43309]EXON

CCACCTAGTCTCTTCCTCCTACTCTGAAATAGGTTGTAACTGTTACTAATTTTTCGTGTGTCCTTCCAGATATTTTTTTG

ATGCATATGTTTTCCATATT

In100 candidate3080 (in exon):

LOCUS ENSG00000158691

DESCRIPTION Zinc finger protein 96 (Zinc finger protein 305) (Zinc finger ad

SCAN domain-containing protein 12). [Source:Uniprot/SWISSPROT;Acc:O43309]EXON

AACTGTCCTTAACAAATTAAACTTTCTCTTGGTTTCATGATATTCCTTGTTTCTCAGCTTTGGATTCAAACTGATCTTCT

CTTTTTTGATTATACTTTCC

In100 candidate3081 (in intron):

LOCUS ENSG00000088367

DESCRIPTION Band 4.1-like protein 1 (Neuronal protein 4.1) (4.1N).

[Source:niprot/SWISSPROT;Acc:Q9H4G0]

TGTTTCGTCTTTGGATCATTAACCTTCCTATTACTGTGGTCTCACTTTTTCAGTTCCCCCTATTTCTTTGGCATCATCTC

ATGGCCTTTTACTTTCTTGT

In100 candidate3082 (in intron):

LOCUS ENSG00000197969

DESCRIPTION Vacuolar protein sorting-associated protein 13A (Chorein)

(Choraacanthocytosis

protein). [Source:Uniprot/SWISSPROT;Acc:Q96RL7]

TGAATTTGCATGCCTGATGTCATTAACCATAGAATATCCTTCCTGAAAGTATTTCCTTTCTTGGCTCAGTGATACTTTTC

TGTCTATATTATACACCTTC

In100 candidate3083 (in intron):

LOCUS ENSG00000197969

DESCRIPTION Vacuolar protein sorting-associated protein 13A (Chorein)

(Choraacanthocytosis

protein). [Source:Uniprot/SWISSPROT;Acc:Q96RL7]

AGGCTACTGATTTTCTGTTTACTGGTTGTGTTGAAGCCTCTGATTATAACCATTGATTTGCCTGTTTCTCCTTTCAGTTC

TTTAAGTTTTTTGCTTTGTA

In100 candidate3084 (in intron):

LOCUS ENSG00000137634

DESCRIPTION Protein FAM55D precursor.

[Source:Uniprot/SWISSPROT;Acc:Q6UWF7]EXON

GAATCTATCCTGAACTTTTTGTGTTGGCAATTTTTTAACTATCATTTCAATCTCACTGTTTGTTACTGGTCTGTTCAGAG

ATTCTATATCTTCCTGGTTT

In100 candidate3085 (in intron):

LOCUS ENSG00000204179

DESCRIPTION protein tyrosine phosphatase, non-receptor type 20A isoform 2

[ource:RefSeq_peptide;Acc:NP_001035846]

AGGAATTTATGGTAAATATTTTCTTAACGAAAGTCTATATGTGATTTCTTTTTTTATATCATTGCCAGAGGGTTTCTTTT

CTTTTCTTTCTTTATTTGGT

In100 candidate3086 (in intron):

LOCUS ENSG00000182389

DESCRIPTION Voltage-dependent L-type calcium channel subunit beta-4 (CAB4)

Calcium channel voltage-dependent subunit beta 4).

[Source:Uniprot/SWISSPRO;Acc:O00305]

AGTATAACCTAGTGAGGTCTTTTTTTCTGAGTCCAGTTCTTACTTTCTGTATGTATTTTTCTGGTTGTCTTTATTCCTAT

TCTACTGACCTCAATATTTT

In100 candidate3087 (in intron):

LOCUS ENSG00000156049

DESCRIPTION Guanine nucleotide-binding protein alpha-14 subunit (G-protein

lpha subunit 14). [Source:Uniprot/SWISSPROT;Acc:O95837]

CCTTCTAACTTAGATTTTTATATGAAATATATTTTCCTGCTTCCTTGCTCCTCTCTGTTCAGTCTTATCCCTGCTTCTAT

ATCCTCTGATTAAAGGCTTT

In100 candidate3088 (in intron):

LOCUS ENSG00000121417

DESCRIPTION Zinc finger protein 134. [Source:Uniprot/SWISSPROT;Acc:P52741]

EXON

AACTTGAATTTATACAAACTTAACTTCTTTAACTTTCCTTGTTCTTTTTTGTGCCGTGTTGTTCTGGGCCAGTGTTAGTT

GCTCACATGTCCTGTCTTTC

In100 candidate3089 (in intron):

LOCUS ENSG00000115145

DESCRIPTION Signal transducing adapter molecule 2 (STAM-2).

[Source:UniprotSWISSPROT;Acc:O75886]

TCCAAGTCATGACTTGTCTGTTCTAAGCCTTAACTCCGGTTCTTGTTTGCATATCTTTTTTCCCTTTACTCAAGCCAGGC

AATCTTTCTTGCCAGTTTTC

In100 candidate3090 (in intron):

LOCUS ENSG00000080845

DESCRIPTION Disks large-associated protein 4 (DAP-4) (SAP90/PSD-95-associatd

protein 4) (SAPAP4) (PSD-95/SAP90-binding protein 4).

[Source:Uniprot/SWISPROT;Acc:Q9Y2H0]

CCACCCTTCTCCCTTCTTTGGATTTATTTTGGTGTTTCTTTTCTAACTTTTGGAGTTGTTTATCATATTGCTTTTCAGTT

TGTTTTTTTCTTTTCTTTTT

In100 candidate3091 (in intron):

LOCUS ENSG00000156052

DESCRIPTION Guanine nucleotide-binding protein G(q) subunit alpha (Guanine

ucleotide-binding protein alpha-q). [Source:Uniprot/SWISSPROT;Acc:P50148]

AAGGCTATTCCTGAAAGATGCCTAGCATTTTAATATTTAACCTCTTTCCTCTTTCCTCATTACTTTGTTCATTTTCAGTC

ACTTGTCCCTTTTTTAAACT

In100 candidate3092 (in intron):

LOCUS ENSG00000156052

DESCRIPTION Guanine nucleotide-binding protein G(q) subunit alpha (Guanine

ucleotide-binding protein alpha-q). [Source:Uniprot/SWISSPROT;Acc:P50148]

TGGATCTGTTCATTTCTGATAGATGGATGTTGAAGTTTCTAACTGTAATAGCGGGTTTATCTGTTTTCCTTCTATCAGTT

TTTGCCTCATGTTTTGTGAT

In100 candidate3093 (in intron):

LOCUS ENSG00000157827

DESCRIPTION formin-like 2 [Source:RefSeq_peptide;Acc:NP_443137]

CTCTTCTGTTTCTTTACAAGTCCCTAACTTGGAAGTTTTTTTTTTTCTTTTTCGTTTTTAATCCCTGTCCCCAGATTA

GGCCTGTGCTTTCTCTCCTT

In100 candidate3094 (in intron):

LOCUS ENSG00000102349

DESCRIPTION Krueppel-like factor 8 (Basic krueppel-like factor 3) (Zinc finer protein 741). [Source:Uniprot/SWISSPROT;Acc:O95600]

TCCATGATACTATCTAACTGGCTCATACCTTTCTTTTTTTCCTCTCTTTTTTTAAAGCTGGGCTGCTTTCTCAGGGTTC

ATACATTTCTGTGCCACTTT

In100 candidate3095 (in intron):

LOCUS ENSG00000204694

DESCRIPTION Olfactory receptor 5V1 (Olfactory receptor OR6-26) (Hs6M1-21). Source:Uniprot/SWISSPROT;Acc:Q9UGF6]

GGTGATAACTCCTTTACCATTTTTTATTGTGTCTATTTGATTCTTCTCTCTTTTCTTCTTTACTAGTCTGGCTAGCAGTC

TATCTCTTTTGTTAATCTTC

In100 candidate3096 (in intron):

LOCUS ENSG00000204272

DESCRIPTION

TTAACATTTTATCACGTTTGCCTTATCATTCTGTTTTCTCTTCCTCTCCTCCTTCTCCTATTTGACAGTAGTTTGCAAA

TATCATTCTCCTTTACCTCT

In100 candidate3097 (in intron):

LOCUS ENSG00000198466

DESCRIPTION Zinc finger protein 587. [Source:Uniprot/SWISSPROT;Acc:Q96SQ5]

EXON

AAAATAATGAAATAACAAAGACGTATGTTGGACTTCATTCATTCTTCAGTGATGTCACTTTGCTGCAAGGAATATTTGGT

TTTCTTTTTTTTTTTTTTTT

In100 candidate3098 (in intron):

LOCUS ENSG00000123612

DESCRIPTION Activin receptor type 1C precursor (EC 2.7.11.30) (ACTR-IC)

(Acivin receptor-like kinase 7) (ALK-7).

[Source:Uniprot/SWISSPROT;Acc:Q8NER5]EXON

TGAGTTTAACTGGTCTACCTCTATCATCTCTTAACTTGTCAGTGGCTTTTTGCATGATTCCAAATGATCTCTAACTTCAC

TTTCATTTTTTAATTCCTTT

In100 candidate3099 (in intron):

LOCUS ENSG00000101082

DESCRIPTION Src-like-adapter 2 (Src-like adapter protein 2) (SLAP-2)

(Modultor of antigen receptor signaling) (MARS).

[Source:Uniprot/SWISSPROT;Acc:Q96Q3]

GCAAATCCTTTTTTCGACTCTGCTTGTCTTCCATAACTTCTATGGTTTTAATCTTGCCATACTTTTCAGAGGTCTCTCAA

ATTATATTCTTCTGTATCTT

In100 candidate3100 (in intron):

LOCUS ENSG00000153237

DESCRIPTION CDNA FLJ25376 fis, clone TST02010.

[Source:Uniprot/SPTREMBL;AccQ96LM2]

CTGCAATCACCAAAATATAGACTTTTAACCCATCTTCATCTTCTTTTAGCTTGATGCCTTCTGTATCAGGTCTGTATGAC

TCCTATCAAAATTTTCCTCT

In100 candidate3101 (in intron):

LOCUS ENSG00000131089

DESCRIPTION Rho guanine nucleotide exchange factor 9 (Rac/Cdc42 guanine

nuceotide exchange factor 9) (Collybistin) (PEM-2 homolog).

[Source:Uniprot/SWSSPROT;Acc:O43307]

TGGGAACATTGATATGATGAGTCTTCAAATCCATGAACATTTAACTGTTTATTTCGATTTTTTTGTGATTTCTCCCAGGA

TTGTTTTTTAGTTTTCATTG

In100 candidate3102 (in intron):

LOCUS ENSG00000131089

DESCRIPTION Rho guanine nucleotide exchange factor 9 (Rac/Cdc42 guanine

nuceotide exchange factor 9) (Collybistin) (PEM-2 homolog).

[Source:Uniprot/SWSSPROT;Acc:O43307]

AGATCACTGCGACTCCTGCTACTTTGCTGATACTGATATAACCAATCCCTATTCTTTGCTCTCTGCTGTTTCCAGACATC

TAGGCTATATCTGTCTTTTC

In100 candidate3103 (in intron):

LOCUS ENSG00000163331

DESCRIPTION death-associated protein-like 1 (LOC92196), mRNA

[Source:RefSeqdna;Acc:NM_001017920]

CATTTTTGACCTGAAAAAATAGCAAATGTACACACTTAGACCTAATAAATAACCCTAACTCTTCTTAACTTCTTTCAGAG

GTTCTTTCTTTCTTTTGACA

In100 candidate3104 (in intron):

LOCUS ENSG00000196781

DESCRIPTION Transducin-like enhancer protein 1 (ESG1) (E(Sp1) homolog).

[Sorce:Uniprot/SWISSPROT;Acc:Q04724]

AGGAGTGGTAACTTCTGGTGGCCCCTGGTTTATAAATTGTTCTTGTTTCCATTATCTTCAGTTCCTCTCTCCTTATTTAA

TGCTTCCCTAGTATATCTTA

In100 candidate3105 (in intron):

LOCUS ENSG00000196781

DESCRIPTION Transducin-like enhancer protein 1 (ESG1) (E(Sp1) homolog).

[Sorce:Uniprot/SWISSPROT;Acc:Q04724]

AAGGTTTCCTTTTCTCCACACCATCACTAACACTATCTTTGTCTTTTTGTTACCCACCATTTCAGCAGGTTTGAGGTGAC

ATTTCTTTCTTTCTTTCTTT

In100 candidate3106 (in intron):

LOCUS ENSG00000115183

DESCRIPTION TPR domain, ankyrin-repeat and coiled-coil-containing

[Source:RfSeq_peptide;Acc:NP_203752]

CTCTTAACTCTGATACTTTTTAGACAATTTTTTTCATTATCTTCCTATCAGAGTTTCTCCCTCGCTCCTCTTCTCTGTAG

AGTGATTTAGCAGCTTATTT

In100 candidate3107 (in intron):

LOCUS ENSG00000196151

DESCRIPTION WD repeat, SAM and U-box domain-containing protein 1.

[Source:Uiprot/SWISSPROT;Acc:Q8N9V3]

GAGTCTGTTTCTTAACTACTGTTTCTGCTATTTTGTTCTGTTTCTGTCCTTGCTCTAATATCATTCTGTCTCTCCCAGGC

TTTATATTTTGTCTATTCCT

In100 candidate3108 (in intron):

LOCUS ENSG00000123636

DESCRIPTION Bromodomain adjacent to zinc finger domain protein 2B

(hWALp4).[Source:Uniprot/SWISSPROT;Acc:Q9UIF8]

AGTTTTAACTGTTTTTCGATGCTTATTTTGTCTGTTGTTCAGACTGGATAATTTCTATTGATCTACCTTTGAGTTCATTG

GCTCTTTTTTTTTATCATCT

In100 candidate3109 (in intron):

LOCUS ENSG00000123636

DESCRIPTION Bromodomain adjacent to zinc finger domain protein 2B

(hWALp4).[Source:Uniprot/SWISSPROT;Acc:Q9UIF8]

GAAGCTCTACCATTCACATCTTTTTATTTAACTGATCTTATTGTTTTGTATTTTCTTCTGTGGTGTCAGGTTATTTATAA

CACACATTTTCCCATTGTTT

In100 candidate3110 (in intron):

LOCUS ENSG00000183675

DESCRIPTION protein tyrosine phosphatase, non-receptor type 20 isoform 8

[Surce:RefSeq_peptide;Acc:NP_001035822]

AGGAATTTATGGTAAATATTTTCTTAACGAAAGTCTATATGTGATTTCTTTTTTTATATCATTGCCAGAGGGTTTCTTTT

CTTTTCTTTCTTTATTTGGT

In100 candidate3111 (in intron):

LOCUS ENSG00000110274

DESCRIPTION centrosomal protein 164kDa [Source:RefSeq_peptide;Acc:NP_055771

GCCTAGTTAACCTTGAAGAAGCTTTCTTGGGCTCTTCCCCTCCCCCGGCCTTTTTTTTTTTTTTTTTAAGAGACCAGAG

TTTTGCTCTGTTGTCCATGC

In100 candidate3112 (in intron):

LOCUS ENSG00000177103

DESCRIPTION Down syndrome cell adhesion molecule-like protein 1 precursor

(own syndrome cell adhesion molecule 2). [Source:Uniprot/SWISSPROT;Acc:Q8TD8]

AGGGCACATGATTTTAACTACTCATGGGGCACCTTTGTATTTCACTACAGATACCAGTTATTTTTCCTCCTTTGTCTGTC

TGAAGCCTATGCATCTTTCA

In100 candidate3113 (in intron):

LOCUS ENSG00000186788

DESCRIPTION CDNA FLJ46321 fis, clone TESTI4043067.

[Source:Uniprot/SPTREMBLAcc:Q6ZQQ2]

TTCTTCATGTCCATTGTGATTTTTTTTATTTCTAACATTCCTTTTTGATTCTTAGAGTTTCCATCTCTTTATTTACAGTC

GTCATCTGTTCTTGCATGTT

In100 candidate3114 (in intron):

LOCUS ENSG00000126542

DESCRIPTION Protein tyrosine phosphatase, non-receptor type 20C

(Fragment).[Source:Uniprot/SPTREMBL;Acc:Q5SWJ0]

AGGAATTTATGGTAAATATTTTCTTAACGAAAGTCTATATGTGATTTCTTTTTTTATATCATTGCCAGAGGGTTTCTTTT

CTTTTCTTTCTTTATTTGGT

In100 candidate3115 (in intron):

LOCUS ENSG00000170324

DESCRIPTION FERM and PDZ domain containing 2 isoform 3

[Source:RefSeq_peptie;Acc:NP_001018081]

TTTGTGCCTTCATATTTAAAGGGGATTTCTTCTAGACAACATAACTGGGTTTTATTTTTTAATCTACTCTGTCAGTTTCT

GTCTTTTAACTGGCATACTT

In100 candidate3116 (in intron):

LOCUS ENSG00000165105

DESCRIPTION RAS and EF hand domain containing

[Source:RefSeq_peptide;Acc:NP689786]

GTAGTACAATTGTTACAATTAATGAACTGATTATTGATTCACTATTATTAACTAAAGTCCATATTCGGATTTCCTCAGTT

TTTCCCTAAAGTCCCTTTTT

In100 candidate3117 (in intron):

LOCUS ENSG00000165105

DESCRIPTION RAS and EF hand domain containing

[Source:RefSeq_peptide;Acc:NP689786]

GCTATGGAATAATGAAGAGTTAGTAACTTTGCTAATATCTTCTTTACTTTCTAATGATTTTATTTTCAGTATCAGGTTTC

CTAGTGTTTTTTATGTTTTT

In100 candidate3118 (in intron):

LOCUS ENSG00000147065

DESCRIPTION Moesin (Membrane-organizing extension spike protein).

[Source:Uiprot/SWISSPROT;Acc:P26038]

TTTTTTTTTAACTGTGGAAAGCAATGCCATACCTCTTCTTTCTGCTTTAGCCTATGTTCTTTCCTTCAGCTTGAAATTTC

AGGTGCCTCTTCTTCATCTC

In100 candidate3119 (in intron):

LOCUS ENSG00000147065

DESCRIPTION Moesin (Membrane-organizing extension spike protein).

[Source:Uiprot/SWISSPROT;Acc:P26038]

AGTTTCTCATCTGTACAATGCCTAACTTGTTTCTCAAATCTGGTTATCTCTCAGAAAATTTATCTCTGGCATTTATTTGT

GCATTCCATCTCTCTTTCTC

In100 candidate3120 (in intron):

LOCUS ENSG00000153250

DESCRIPTION RNA-binding motif, single-stranded-interacting protein 1

(Singlstranded

DNA-binding protein MSSP-1) (Suppressor of CDC2 with RNA- bindin motif

2). [Source:Uniprot/SWISSPROT;Acc:P29558]

CATATATGTTTATATATTTAGGCTTTAACAATTTTTTTAATTGTGTCATTCTTGATTTTCAGGTAAAATTTATTTTTGAC

TCAGTTCTCTTTACCTATTC

In100 candidate3121 (in intron):

LOCUS ENSG00000172159

DESCRIPTION FERM domain containing 3. [Source:Uniprot/SPTREMBL;Acc:Q5VZA1]

EXON

TCTCTTCTGTAACACATGATGAGATTATTTCTGAGTTTCTTCTCCACTCTGATTGTTCTTTAATAATTCTTTCTCCAGAT

TGCTACTGCTCTTATTTACA

In100 candidate3122 (in intron):

LOCUS ENSG00000136560

DESCRIPTION TRAF family member-associated NF-kappa-B activator (TRAFinterating

protein) (I-TRAF). [Source:Uniprot/SWISSPROT;Acc:Q92844]

CTATGTGGATAACCCTTTTTCATGATGCATTTTGTCCATTTGTCTTTTCTTTTTTTTTCTGATTGGATGTCACAGAACTA

ATTTCTTGGATCTATTTCTT

In100 candidate3123 (in intron):

LOCUS ENSG00000089472

DESCRIPTION Hephaestin precursor (EC 1.-.-.-).

[Source:Uniprot/SWISSPROT;Ac:Q9BQS7]

TTCTGCCTTCTGCCTGCTCAAATCTGCTGTTTAACTCTTCTAGTGAATTAAAATTTCAATTCTCCTTTTCTATTCCAGAA

TTTGTTTGGTTCCTTTTTAT

In100 candidate3124 (in intron):

LOCUS ENSG00000115233

DESCRIPTION 26S proteasome non-ATPase regulatory subunit 14 (26S

proteasomeregulatory subunit rpn11) (26S proteasome-associated PAD1 homolog 1).

[Soure:Uniprot/SWISSPROT;Acc:O00487]

CATGTGGCTTGCTTCCTCTCTCTCGAGGTAACTCTAGTTGGTATTTTTCTTTTCTGTTCCATTGGCTTGCAGTAGTACCT

TAATTTATTCATTCATTCTT

In100 candidate3125 (in intron):

LOCUS ENSG00000144290

DESCRIPTION Sodium-driven chloride bicarbonate exchanger (Solute carrier

faily 4 member 10). [Source:Uniprot/SWISSPROT;Acc:Q6U841]

GTCAATTAACTCGGTAAACATTTTACTAACTTTCTTTTGTATTCCATTTACAACTTTGTTTGCTATCAGTATTTCTCTAT

GAAGTAAGCTTTCTCTTTCT

In100 candidate3126 (in intron):

LOCUS ENSG00000144290

DESCRIPTION Sodium-driven chloride bicarbonate exchanger (Solute carrier

faily 4 member 10). [Source:Uniprot/SWISSPROT;Acc:Q6U841]

AACCTGATTTAACAAGTGTTACTGTAATATTGGATTTCCCTTAATTCCTTACTCGTTTAGTCATTCCTTTACCCTCAGCT

TCTATTCTTTTTTCTCTTCA

In100 candidate3127 (in intron):

LOCUS ENSG00000144290

DESCRIPTION Sodium-driven chloride bicarbonate exchanger (Solute carrier faily 4 member 10). [Source:Uniprot/SWISSPROT;Acc:Q6U841]

AGCTCCTGGATTCATTAACTTTTTGAAGGGTTTTTATGTCTCTATTTCCCTCAGTTCTGCTCTGATTTAGTTATTTCT

TGCCTTCTGCTAGTTTTCGA

In100 candidate3128 (in intron):

LOCUS ENSG00000197635

DESCRIPTION Dipeptidyl peptidase 4 (EC 3.4.14.5) (Dipeptidyl peptidase IV) DPP IV) (T-cell activation antigen CD26) (TP103) (Adenosine deaminase complxing protein 2) (ADABP) [Contains: Dipeptidyl peptidase 4 membrane form (Dieptidyl peptidase IV membrane form); Di [Source:Uniprot/SWISSPROT;Acc:P2748]

TGTTTATAATAGCCTCTTATGATCTTTTAACATCTGTGGAGTCTGTTGTGATATCCCATTTTTCCTTTCTGATACCAGTC

ATTTGTGCTGTTTTACTCCT

In100 candidate3129 (in intron):

LOCUS ENSG00000128805

DESCRIPTION Rho GTPase activating protein 2 [Source:RefSeq_peptide;Acc:NP_07049]

CTGTTGTTATTTATAACACTTCCTCAATTTAATTTCTGTTCTCCATTCTATATCATAAACCTCATTCAGGTGGGGCTTCA

ATCTGTCTTATTATTTCTCT

In100 candidate3130 (in intron):

LOCUS ENSG00000128815

DESCRIPTION Uncharacterized protein C10orf64.

[Source:Uniprot/SWISSPROT;AccQ6ZS81]

GACTTTGAAGGAACAATTACTGAGAGGAGTGTTGAACTCTCTAACTATAATTGTGGATTTGGCCATTTGTCCTTTCAGAT

CTATGAGTTTTTCTTTCTGA

In100 candidate3131 (in intron):

LOCUS ENSG00000128815

DESCRIPTION Uncharacterized protein C10orf64.

[Source:Uniprot/SWISSPROT;AccQ6ZS81]

ATTCATTTAATTTGCACAAATGGTCTCCTTTAACTCATGTCATTTTCTGTGTGATTGTTTCTCCCTTCGTATTCTCAGTT

TGTGTCCTGCTTTCTCCTTC

In100 candidate3132 (in intron):

LOCUS ENSG00000165113

DESCRIPTION G kinase anchoring protein 1 [Source:RefSeq_peptide;Acc:NP_07947]

AAGCCTAACATTTGCTTAGGATTCTGCTTTTTCAGTTATTGAAGGAGGCAGCCTGTCATCACTTTAGGTTTTCCTTTTTG

TTTCCTTATTTCCTTCTCCC

In100 candidate3133 (in intron):

LOCUS ENSG00000165383

DESCRIPTION Leucine-rich repeat-containing protein 18.

[Source:Uniprot/SWISPROT;Acc:Q8N456]

GGATTTAGTAAATCCTCCCTTTAACTCCTGATATGTGTAATTTGGCTTCTCTTTCTTTTTTTTTTAAATTGTCAGTCTGG

CTATCTGTTTCATTAATCTT

In100 candidate3134 (in intron):

LOCUS ENSG00000115271

DESCRIPTION Grancalcin. [Source:Uniprot/SWISSPROT;Acc:P28676]

GCTAATCTTTGTTCTTTGATTAGATGAACTTAATTGCTTCAATTTACTAACTAATAAAGGGATGTATTTTTTTGCCAGCA

AGTTCCTTTGTTTGTTTTCA

In100 candidate3135 (in intron):

LOCUS ENSG00000184611

DESCRIPTION Potassium voltage-gated channel subfamily H member 7 (Voltagegted potassium channel subunit Kv11.3) (Ether-a-go-go-related gene potassiumchannel 3) (HERG-3) (Ether-a-go-go-related protein 3) (Eag- related protein3). [Source:Uniprot/SWISSPROT;Acc:Q9NS40]

CTCTATGACCCTTTACTTGATATCCACCTGAACATATAACTTCTCACTCTATCCTCTATAATTCTTAAGCTCTTTCAGAT

TTTAAATCTTTCTGACCATC

In100 candidate3136 (in intron):

LOCUS ENSG00000184611

DESCRIPTION Potassium voltage-gated channel subfamily H member 7 (Voltagegted potassium channel subunit Kv11.3) (Ether-a-go-go-related gene potassiumchannel 3) (HERG-3) (Ether-a-go-go-related protein 3) (Eag- related protein3). [Source:Uniprot/SWISSPROT;Acc:Q9NS40]

TAGGTATTCTGTGTCTAAATATTTATTGTCTTAATTAACAAATCGCCTGTTTGCTGACATTTTTCTCAGTTGTTTTTCCT

ACAGTTAGTGTTTTGTATTT

In100 candidate3137 (in intron):

LOCUS ENSG00000184611

DESCRIPTION Potassium voltage-gated channel subfamily H member 7 (Voltagegted potassium channel subunit Kv11.3) (Ether-a-go-go-related gene potassiumchannel 3) (HERG-3) (Ether-a-go-go-related protein 3) (Eag- related protein3). [Source:Uniprot/SWISSPROT;Acc:Q9NS40]

AGGATCTGAGTCCTTCTGTCCTCATTAACCTAACACATCTTTCCATTTTGTCCAACAGTTGTGTTTTCTCTGAACCTGGC
TTTCACATTTATTGTCTTAC

In100 candidate3138 (in intron):

LOCUS ENSG00000079482

DESCRIPTION Oligophrenin 1. [Source:Uniprot/SWISSPROT;Acc:O60890]

CTTATAGGATTCCTTTTCAATAACTTGCTGAACCTTTATCTTTCTGTTTTTTACACCTTGACTGTGTGTTTCCTACAGTT
CTATCTTTGTTCTTGGTTTA

In100 candidate3139 (in intron):

LOCUS ENSG00000079482

DESCRIPTION Oligophrenin 1. [Source:Uniprot/SWISSPROT;Acc:O60890]

AGGGAGAATCAATTGGCATATGGATTTATATTAAGCTGCTAACTAGGGTAGCCTCCATCCTTTTTTCAGGTAGCACCTTC
CTCTTTTACATATATATCAT

In100 candidate3140 (in intron):

LOCUS ENSG00000079482

DESCRIPTION Oligophrenin 1. [Source:Uniprot/SWISSPROT;Acc:O60890]

AAATCGCCTTTTAATAACTGCATCCTTAGCATACAATTGTGCTCACTCTAACATCTTTCTCTTTTTGTTTCTCTACAGCT

CTGTCTCTGTCTGTCACTTT

In100 candidate3141 (in intron):

LOCUS ENSG00000115290

DESCRIPTION Growth factor receptor-bound protein 14 (GRB14 adapter protein)

[Source:Uniprot/SWISSPROT;Acc:Q14449]

TAACTTAGCCCTTCTGCTCTCCATTTAGTGTGGTCTGGTTATTCTTTCCTGAGTATTGTTCACTCCTTTCTTCCTCAGTA

TGTTTGCTCATGTTTGTCCC

In100 candidate3142 (in intron):

LOCUS ENSG00000082438

DESCRIPTION Cordon-bleu protein-like 1. [Source:Uniprot/SWISSPROT;Acc:Q53SF]

TCATTTTGCTCAAATTAACTTTCTCCTTTCTGCAAATATTACTCCCACTGTTGACTTTCATTTCTTTTCTTTCCCCAGGT

TTTTCATTGCTTAAATATTT

In100 candidate3143 (in intron):

LOCUS ENSG00000082438

DESCRIPTION Cordon-bleu protein-like 1. [Source:Uniprot/SWISSPROT;Acc:Q53SF]

CAATTAGGACATTCCTAACTTCTTTCCTTATTTTTTTTTTTTTTGTCAGAGCATGTAACACCATCTGATATTTTATATAT

TTTACTTGTTTCTTTTTTCT

In100 candidate3144 (in exon):

LOCUS ENSG00000110367

DESCRIPTION Probable ATP-dependent RNA helicase DDX6 (EC 3.6.1.-) (DEAD boxprotein 6) (ATP-dependent RNA helicase p54) (Oncogene RCK). [Source:UniprotSWISSPROT;Acc:P26196]

GGAATCAACAATAGTAACTCTTTCTTTCCGTTTTTTTTTCTTTTTCTTTTAAAAAATGTCTTCTTATTTTGGTTTCAGGT
TGAAGTCTCTTCCCTTTCTA

In100 candidate3145 (in intron):

LOCUS ENSG00000178662

DESCRIPTION TGF-beta-induced apoptosis protein 2 (TAIP-2). [Source:Uniprot/WISSPROT;Acc:Q8WYN3]

GTGGTTTTTCTGTAATGATAACATTTAATTTGTTTCTCATTTGTTTCTTTCTCATTTGTGTATCTGCTGTACCAGTACAT
TTTATACTTTTATGTATTTT

In100 candidate3146 (in intron):

LOCUS ENSG00000144285

DESCRIPTION Sodium channel protein type 1 subunit alpha (Sodium channel proein type I subunit alpha) (Voltage-gated sodium channel subunit alpha Nav1.) (Sodium channel protein, brain I subunit alpha). [Source:Uniprot/SWISSPRO;Acc:P35498]

GGAAAGTAAATAAATATCATCACTTGGAAGTAACTGCTGCCATTCACATTTTGTTTTCTGTTCAGAGTATTTCTGCACAT
ACTCTTATTGTTGTACTTTT

In100 candidate3147 (in intron):

LOCUS ENSG00000158813

DESCRIPTION Ectodysplasin-A (Ectodermal dysplasia protein) (EDA protein) [Cntains: Ectodysplasin-A, membrane form; Ectodysplasin-A, secreted form]. [Surce:Uniprot/SWISSPROT;Acc:Q92838]

CTTAACTTCCAATTCATTAATTCTGTCTTCAGCTTTGTCTAAACTCCCTTTAAACTCACTCATTGAGTTCTTAATATTAT
TTGTCATGTTTTTATTTTT

In100 candidate3148 (in intron):

LOCUS ENSG00000148053

DESCRIPTION BDNF/NT-3 growth factors receptor precursor (EC 2.7.10.1) (Neurtrophic tyrosine kinase receptor type 2) (TrkB tyrosine kinase) (GP145-TrkB (Trk-B). [Source:Uniprot/SWISSPROT;Acc:Q16620]

GCTGAGTGTTTTTATGTATATGTTTTGTTGACGCTTCTTAAGAGTTCATAACATTCTCTGGTATATCATACTTTTCAGAT
CATAAGTATACTTTTTTCTT

In100 candidate3149 (in intron):

LOCUS ENSG00000148053

DESCRIPTION BDNF/NT-3 growth factors receptor precursor (EC 2.7.10.1) (Neurtrophic tyrosine kinase receptor type 2) (TrkB tyrosine kinase) (GP145-TrkB (Trk-B). [Source:Uniprot/SWISSPROT;Acc:Q16620]

TGATTAGAGCACATTCCCCTTAGAATTGTTGAGTTTTAACTGTGCAATAACTTTGGCTTTCCTTACAAATTCCAGCTTTG
CCAAGTGTGCTTTCTACTTT

In100 candidate3150 (in intron):

LOCUS ENSG00000148053

DESCRIPTION BDNF/NT-3 growth factors receptor precursor (EC 2.7.10.1) (Neurtrophic tyrosine kinase receptor type 2) (TrkB tyrosine kinase) (GP145-TrkB (Trk-B). [Source:Uniprot/SWISSPROT;Acc:Q16620]

AGAGCACGTTTGCTTTGTCCGTTATAACTGAAGATGGATTTGCTGTTTTTATTGCCTTCAGGTGAATTTTCTCATCACT CTTGAACTTTAGTACTTCCC

In100 candidate3151 (in intron):

LOCUS ENSG00000148053

DESCRIPTION BDNF/NT-3 growth factors receptor precursor (EC 2.7.10.1) (Neurtrophic tyrosine kinase receptor type 2) (TrkB tyrosine kinase) (GP145-TrkB (Trk-B). [Source:Uniprot/SWISSPROT;Acc:Q16620]

AGGACCCAAGAATCTTCATGCTTGCATTTCTACTTAACACCTTGTCTTAACTTCCTATTTTATTTCTCTTGACTTCAGTT ATTATTACAGTTTTTGTTTT

In100 candidate3152 (in intron):

LOCUS ENSG00000089289

DESCRIPTION Immunoglobulin-binding protein 1 (CD79a-binding protein 1) (B cll signal transduction molecule alpha 4) (Alpha 4 protein) (Renal carcinomaantigen NY-REN-16). [Source:Uniprot/SWISSPROT;Acc:P78318]

AGTCTCTTTAAGTAGTTAACAACTATTATTCTCTCTTTGCTTCATGTTTGTTTCCTTCTTGGCACTTGTGTGCAGTCATT TCATGTGTCTTTCTGAGTTT

In100 candidate3153 (in intron):

LOCUS ENSG00000163092

DESCRIPTION cardiomyopathy associated 3 isoform 1

[Source:RefSeq_peptide;Ac:NP_689594]

ATTAATTTAACAAATACCGTAAGTGACTATTTTTTTCCTTTCCCTGTCCTCCTATGACTGATTTTCATACTCTATCAGGT

TCTTTTCAAAAATCTTCTCA

In100 candidate3154 (in intron):

LOCUS ENSG00000163092

DESCRIPTION cardiomyopathy associated 3 isoform 1

[Source:RefSeq_peptide;Ac:NP_689594]

ATTTTTTTAACAAGGAAGCTTCCTTATTATACCTTACCTTTTTCCTCCAGAACAAATATCATTGATTCTCTATAGTTC

TTTCATTCATTCCTGTCTTT

In100 candidate3155 (in intron):

LOCUS ENSG00000198648

DESCRIPTION STE20/SPS1-related proline-alanine-rich protein kinase (EC 2.7.1.1) (Ste-20-related kinase) (Serine/threonine-protein kinase 39) (DCHT).

[ource:Uniprot/SWISSPROT;Acc:Q9UEW8]

TATTTTAAATACAATATATAACCATAAAGCTTTTCTTTTTCTATGCTTTAGTTAATTATTCTTCCAAAAACTCTTCAGCT

TGCCTTTTTTCCCCTTCTTG

In100 candidate3156 (in intron):

LOCUS ENSG00000198648

DESCRIPTION STE20/SPS1-related proline-alanine-rich protein kinase (EC 2.7.1.1) (Ste-20-related kinase) (Serine/threonine-protein kinase 39) (DCHT).

[ource:Uniprot/SWISSPROT;Acc:Q9UEW8]

AGGTTTCGTGGTATTAAATACCTTCCACTCTTTAACTCCCAAATTTATATCTTTGATCCAGACTTTTATTTTGAATTCCA

GACTTATATTCAGCTCCCCT

In100 candidate3157 (in intron):

LOCUS ENSG00000172292

DESCRIPTION LAG1 longevity assurance homolog 6.

[Source:Uniprot/SWISSPROT;Ac:Q6ZMG9]

TTAAAAACCTAATACAAGTAGAATGTGCTTAGATGTGTTTCTTATAGATAACATATGGTGGGATTTTGTTTTATTCAGTC

CGGACACATTTGTTTTTTTT

In100 candidate3158 (in intron):

LOCUS ENSG00000135049

DESCRIPTION ATP/GTP binding protein 1

[Source:RefSeq_peptide;Acc:NP_056054]EXON

ATGTCTTTTAACCTCTTTTTTCTGTTTTCCTTTTCTTTGCCTCTCTGTGCTGCCTTCAGATTATTTCATTAGAACTATCT

GTCAGTTTCCTAATTCTCTT

In100 candidate3159 (in intron):

LOCUS ENSG00000081479

DESCRIPTION Low-density lipoprotein receptor-related protein 2 precursor (Mgalin) (Glycoprotein 330) (gp330). [Source:Uniprot/SWISSPROT;Acc:P98164]

ATTATAGAATGTTTCAAAGTGTGCACTAACCTGCCACCTTAGGTTTCTTTGTTTACTCCCCTTTAGTGTGTTTCCCAGCT

TTAATATCTTCAAGTCTTTT

In100 candidate3160 (in intron):

LOCUS ENSG00000148584

DESCRIPTION APOBEC1 complementation factor (APOBEC1-stimulating protein). [ource:Uniprot/SWISSPROT;Acc:Q9NQ94]

AATAAAAGTTAAAACCATTTCTCCGCAACTCCATTGCCCCAAAATAACCACTATGTTGATATTTTATTGTCTTTCCAGAT

TTTTCTAAACATTTTTAATC

In100 candidate3161 (in intron):

LOCUS ENSG00000154474

DESCRIPTION Kelch repeat and BTB domain-containing protein 10 (Kelch-relate protein 1) (Kel-like protein 23) (Sarcosin). [Source:Uniprot/SWISSPROT;AccO60662]

GACTATCTCGATGACTTCTAAACAACATATAAAGTATAACTTTTTTTGGTGTACTTCTAATTTTTTTCTAACAGGCTCCA

CAATCTCTCATATATGTTTT

In100 candidate3162 (in intron):

LOCUS ENSG00000101407

DESCRIPTION Uncharacterized protein KIAA0406. [Source:Uniprot/SWISSPROT;AccO43156]

CTAACATAGCTATTCTCCTGTTTTGTGTAATCTTTTTCCATTCTTTTCTGTTACAAAACTGTTTTTCCGTGGTTGCAGCT

GTGTACCCTTTTTGTGTTTA

In100 candidate3163 (in intron):

LOCUS ENSG00000138398

DESCRIPTION Peptidyl-prolyl cis-trans isomerase G (EC 5.2.1.8) (Peptidylprlyl isomerase G) (PPIase G) (Rotamase G) (Cyclophilin G) (Clk-associating Rcyclophilin)

(CARS-cyclophilin) (CARS-Cyp) (SR-cyclophilin) (SRcyp) (SR-cy)

(CASP10). [Source:Uniprot/SWISSPROT;Acc:Q13427]

AAATAGTATATTAACTATATACTATTCTTTTTCTTCTTACATGATTTTTGCTTCTTAAAAATCTCACATTTTAATCAGAA

CTCTCATTTTTGTTTTCTAG

In100 candidate3164 (in intron):

LOCUS ENSG00000185532

DESCRIPTION cGMP-dependent protein kinase 1, alpha isozyme (EC 2.7.11.12) (GK 1 alpha) (cGKI-alpha). [Source:Uniprot/SWISSPROT;Acc:Q13976]

TGAAAGAAAGAATGCTATAACTTTTAAAAAGAATTTTATCCTTGGTCAGGAAAAAGTCATTTTTCTCTTCTTCAAAATTT

TACTTCGATTCTTTGTTCTT

In100 candidate3165 (in intron):

LOCUS ENSG00000185532

DESCRIPTION cGMP-dependent protein kinase 1, alpha isozyme (EC 2.7.11.12) (GK 1 alpha) (cGKI-alpha). [Source:Uniprot/SWISSPROT;Acc:Q13976]

TTTCAAATAACAAAATTTTTGTTTCATTTATCTTTTGTATTTGTTGTTGTTTTTGTTGTTGTTCATTTATTTCAGTTTCA

TTTAATTCAGCTCTGATCTT

In100 candidate3166 (in intron):

LOCUS ENSG00000185532

DESCRIPTION cGMP-dependent protein kinase 1, alpha isozyme (EC 2.7.11.12) (GK

1 alpha) (cGKI-alpha). [Source:Uniprot/SWISSPROT;Acc:Q13976]

TGGTTGGTTGATTTTAGGAATATTGTTAGTAACCAAGCTTTAAATTTATTTAACATTCTATTTTATCAGTCTTAAAATAC

TCATTTTAGTCTTTTTACCT

In100 candidate3167 (in intron):

LOCUS ENSG00000185532

DESCRIPTION cGMP-dependent protein kinase 1, alpha isozyme (EC 2.7.11.12) (GK

1 alpha) (cGKI-alpha). [Source:Uniprot/SWISSPROT;Acc:Q13976]

AGGAACCACCTGTACAATGAAGAAAAGAAAGTTTCTGAAACTAGGAATTAACCTCACCACTTTGTATTTTGTTTCAGTGA

CTCCTGTAGTTATTTCCTTC

In100 candidate3168 (in intron):

LOCUS ENSG00000120498

DESCRIPTION testis expressed sequence 11 isoform 1

[Source:RefSeq_peptide;Ac:NP_001003811]

TATGTCTTTCTCTTCCTCATCTTTAAGGCCAATAACTATTAGCTTTGCTCTTTTGTGGCTATTTTCTAGATCCTGCAGGT

GTGCTTCATTGTTTTTTATT

In100 candidate3169 (in intron):

LOCUS ENSG00000120498

DESCRIPTION testis expressed sequence 11 isoform 1

[Source:RefSeq_peptide;Ac:NP_001003811]

TGATATATAACAAGATTTATTTCTGCGTTCTCTATTCCATTCCATTGGTCTATATGTCTATCTTTATGCCAGTTCCACAC

ATTTTCATTACTATGGCTTT

In100 candidate3170 (in intron):

LOCUS ENSG00000120498

DESCRIPTION testis expressed sequence 11 isoform 1

[Source:RefSeq_peptide;Ac:NP_001003811]

TTAACTGTCTTCCATGTTTGTAATCGCTCTGTGCTGCATTTTGGCTATTTTATTCTTTCAGCCCACTAATTTTCTCTTCA

CCTGTTTCTAATCTGCTATT

In100 candidate3171 (in intron):

LOCUS ENSG00000120498

DESCRIPTION testis expressed sequence 11 isoform 1

[Source:RefSeq_peptide;Ac:NP_001003811]

AGTTATTGAGAGAGGATATTGAAATCTTTAACTATAATTGTGGATTTGTTCATTTCTTCTTGCAGTTCTACCAGCTTTTG

CTTCTTATATTTTGAAATTT

In100 candidate3172 (in intron):

LOCUS ENSG00000120498

DESCRIPTION testis expressed sequence 11 isoform 1

[Source:RefSeq_peptide;Ac:NP_001003811]

AACTTGGTATGTATTCATTCACACATGTTTCTTCCTAACCTAAGAATCACTTTCCATATTACTTTGTCATACAGGTGTTT

GCTTCGTTTTCTTCTTCCAA

In100 candidate3173 (in intron):

LOCUS ENSG00000120498

DESCRIPTION testis expressed sequence 11 isoform 1

[Source:RefSeq_peptide;Ac:NP_001003811]

TAACTACCGTTCTTTTTTATTCTGTGTTCTTTCTCATGTACACCTCTGTGTTTTCTCAGAACTCTCTAAACTAGTAACCT

TTCTATAATCTTCTTTGCTT

In100 candidate3174 (in intron):

LOCUS ENSG00000120498

DESCRIPTION testis expressed sequence 11 isoform 1

[Source:RefSeq_peptide;Ac:NP_001003811]

ATCTTAGGGCTAAGCTTCCACCTTTGAGTATAATGTTAACTGTGGAATTTTTTTAATGCCCTTTATCAGATTGAGGCTA

TTCCCTTCCATTCCTAGTTT

In100 candidate3175 (in intron):

LOCUS ENSG00000144357

DESCRIPTION Zinc finger protein 650. [Source:Uniprot/SWISSPROT;Acc:Q6ZT12]

EXON

AAAATGTGGTTGTAGATAACTTTAAATTGTCTTTTATTTCCTCTGATATATCTATGTAACTTCTACCTTACCAGTCTTTT

TCCCTTAATCTGCCAGAGTT

In100 candidate3176 (in intron):

LOCUS ENSG00000144357

DESCRIPTION Zinc finger protein 650. [Source:Uniprot/SWISSPROT;Acc:Q6ZT12]

EXON

TATTTGGTAGCAAGAATATTTTCATTATTAGTCTCTAGTAACACTTGGATTTGTCATCTTTGACTATTACTCTATCAGTT

TTGCATCTTTTATTCATTTA

In100 candidate3177 (in intron):

LOCUS ENSG00000144357

DESCRIPTION Zinc finger protein 650. [Source:Uniprot/SWISSPROT;Acc:Q6ZT12]

EXON

TGTGTGAGATAGGGTCAATGAAATTGTATTTCTTTGGCACTTAACACGAGACTGAGTTATTTCACTGTACTTTGTCAGGT

ATCTCTGTGTTCTCCTTTGC

In100 candidate3178 (in intron):

LOCUS ENSG00000144357

DESCRIPTION Zinc finger protein 650. [Source:Uniprot/SWISSPROT;Acc:Q6ZT12]

EXON

AGGCCCTTCTGTGCTAACTCTCCTATTTATCTTGTCTATTTTCTTTTGTCAGCCTTTCACCGTGAATCCTTTTTTGTTTG

TTTGTTTTTGTTTTTGTTTT

In100 candidate3179 (in intron):

LOCUS ENSG00000071909

DESCRIPTION Myosin-IIIB (EC 2.7.11.1). [Source:Uniprot/SWISSPROT;Acc:Q8WXR4

TAACTCTAGAGGTAGATTCTGTCCCATTTGCTCAGAGATGTGATATTTTGGCTTCAAACCACCACCCTATATGTTTTCTT

TTGTCACCTTCTTTACTTTC

In100 candidate3180 (in intron):

LOCUS ENSG00000071909

DESCRIPTION Myosin-IIIB (EC 2.7.11.1). [Source:Uniprot/SWISSPROT;Acc:Q8WXR4

GAAATTTGTTCCAAAATTCGGCACAAATTAGGAAAATAACCAAACCATACCTTTCTATTTAATCTTTGCTTCTCTCAGAT

ACTTACTATTTTCTTGGCTT

In100 candidate3181 (in intron):

LOCUS ENSG00000196914

DESCRIPTION Rho guanine nucleotide exchange factor 12 (Leukemia-associated

hoGEF). [Source:Uniprot/SWISSPROT;Acc:Q9NZN5]

GTATATCTGTGAAAACTAAGAAACCAACCTTAGTACATACTGTTAACTAAACTTCATACTTCATTTGGATTTTTCCAGCT

TTTTCATTAATGGCCTCTTT

In100 candidate3182 (in intron):

LOCUS ENSG00000196914

DESCRIPTION Rho guanine nucleotide exchange factor 12 (Leukemia-associated

hoGEF). [Source:Uniprot/SWISSPROT;Acc:Q9NZN5]

CTAGACTGTGTCCTGTCTTCCAAAGATACAATAACCATGTTTGTGAAGTTCATACCTATTTTTCCTTTTCCTTTGCAGTT

TTATTTGCCCTCTTTCCTTC

In100 candidate3183 (in intron):

LOCUS ENSG00000150275

DESCRIPTION Protocadherin-15 precursor. [Source:Uniprot/SWISSPROT;Acc:Q96QU]

GCTACATAATCTGCAAATAATTTCTCCCATTCTATTGGTTGTCTGGTAACTTTGTTGATAGTTTCCTTTGCTGTGCAGAA

GCTCTTTCATTTATTTATTT

In100 candidate3184 (in intron):

LOCUS ENSG00000150275

DESCRIPTION Protocadherin-15 precursor. [Source:Uniprot/SWISSPROT;Acc:Q96QU]

TCTGGATTTCTGATGTATGCATGTCAACATCTCTAACAAAATTGAGAATATTTTCTTGAAATTTTTCCTCAGGTATGTTT

TCCAAGTTGGTTATTTTTTC

In100 candidate3185 (in intron):

LOCUS ENSG00000150275

DESCRIPTION Protocadherin-15 precursor. [Source:Uniprot/SWISSPROT;Acc:Q96QU]

GGTTTGTCTTTTTCAATGTTTTGCCTTCCAAAGTAACAAAATGGTGTGCTTTTTATACTTCATTTTTTTTGGCTTCAGTT

TTACTTTGGTCTCGTTACTG

In100 candidate3186 (in intron):

LOCUS ENSG00000150275

DESCRIPTION Protocadherin-15 precursor. [Source:Uniprot/SWISSPROT;Acc:Q96QU]

TCAAATAACTAAGATAATTCCTAAATTCATTTATCTTTTATATGGTCTTTGCATCTCAATTTCCTTCAGTTGAGCTCTGA

TTTTGGTTATTTCTTGTCTT

In100 candidate3187 (in intron):

LOCUS ENSG00000150275

DESCRIPTION Protocadherin-15 precursor. [Source:Uniprot/SWISSPROT;Acc:Q96QU]

AGTAGTTACATACTCTCTCATTCCTAACTTCCATTCCTCTCCCTATTAATTTATATTCAGCCCATTTTATTCAATTTGGT

TATGTTCCTACCTTCTCTGT

In100 candidate3188 (in intron):

LOCUS ENSG00000150275

DESCRIPTION Protocadherin-15 precursor. [Source:Uniprot/SWISSPROT;Acc:Q96QU]

AGCAAAGAAAGATTGAGCGGTTAATCTTAACTTGTATTATAATCTGTTTTTCTCCCCTCAAATATCATTCAGTTCAGTTT

ATTTTTAAGATCTTTTGCTT

In100 candidate3189 (in intron):

LOCUS ENSG00000109944

DESCRIPTION CDNA: FLJ23554 fis, clone LNG09359.

[Source:Uniprot/SPTREMBL;Ac:Q9H5D6]

AATAGCTGTGTGTTAGATTGTCTATTCTAGACTTCGTGCCTTTTTAACTGTTTTTATTTTACACCTTTTATTTCTCAGTG

CTTCTTTCTGAATAATTTCT

In100 candidate3190 (in intron):

LOCUS ENSG00000151151

DESCRIPTION Inositol polyphosphate multikinase (EC 2.7.1.151) (Inositol

1,34,6- tetrakisphosphate 5-kinase). [Source:Uniprot/SWISSPROT;Acc:Q8NFU5]

GAAAGTGAGCCAAAGCTCTATTAACTGTCTTTTTTCCTTCCTCCCTTAATTCCTTCCTTCTTTTTTGTGGGCTTACAGGT

CTTTTTAGTCCTAACTATAT

In100 candidate3191 (in intron):

LOCUS ENSG00000151151

DESCRIPTION Inositol polyphosphate multikinase (EC 2.7.1.151) (Inositol

1,34,6- tetrakisphosphate 5-kinase). [Source:Uniprot/SWISSPROT;Acc:Q8NFU5]

TTTATTCTTTAAATGCTGAGGTATTCCTCTTGGTTCCATTTTAACATCTGCCTTCCTTTTCTATTTCTTCTTAAGCAGGG

CTCTCTATTCTCATTTTAGT

In100 candidate3192 (in intron):

LOCUS ENSG00000123600

DESCRIPTION methyltransferase like 8 [Source:RefSeq_peptide;Acc:NP_079046]

EXON

AATCCTACAAACAAAGGATTTCACCACTGTTAACATTTTAGTGACCTTTTTCTTCTTTTTTTTTTTTTTTGAGACAGAC

TTTTGTTCTTGTTGCCCGGG

In100 candidate3193 (in intron):

LOCUS ENSG00000115827

DESCRIPTION CDNA FLJ13096 fis, clone NT2RP3002166.

[Source:Uniprot/SPTREMBLAcc:Q9H908]

AATTTTTAGGGAGCATATATAAAATAACTATGTCTTCATTCTTTTCTATTCACTTTTTGGCCTAAGCAGATACTAGATTA

ATTTCAGCCTCTCTTTACTT

In100 candidate3194 (in intron):

LOCUS ENSG00000071967

DESCRIPTION cytochrome b reductase 1 [Source:RefSeq_peptide;Acc:NP_079119]

EXON

TTTAAATAATTTAAAATTAGTTTAGAACTTAAAATTAAATGATAACCTTTGCACTTTTTGGTGTTTAGCTTCTTTCAGGT

TTTTCAGTCTTTCTGCTTCC

In100 candidate3195 (in intron):

LOCUS ENSG00000072401

DESCRIPTION Ubiquitin-conjugating enzyme E2 D1 (EC 6.3.2.19) (Ubiquitinproein

ligase D1) (Ubiquitin carrier protein D1) (UbcH5) (Ubiquitin- conjugatig

enzyme E2-17 kDa 1) (E2(17)KB 1). [Source:Uniprot/SWISSPROT;Acc:P51668]

GATTGTCTAGAGAAAACCAAGTGGACCTAACAAATGGTCTGGGTTCATTTTTCTTCTCTTCTCTTAAACCAGGAAATCTC

TGGCTTGTGTTTTTGCTTCT

In100 candidate3196 (in intron):

LOCUS ENSG00000115840

DESCRIPTION Calcium-binding mitochondrial carrier protein Aralar1

(Mitochonrial aspartate glutamate carrier 1) (Solute carrier family 25 member

12). [ource:Uniprot/SWISSPROT;Acc:O75746]

AGCCTCTTTTGCACTATTCCATTGAAGACATAACTCAATAGTGAATAAATTCTCTCCAGATTTTCTACATTTTTTGGAGT

TTTTGATAATTGTACCCTTC

In100 candidate3197 (in intron):

LOCUS ENSG00000128708

DESCRIPTION Histone acetyltransferase type B catalytic subunit (EC 2.3.1.48.

[Source:Uniprot/SWISSPROT;Acc:O14929]

TCTTTAAAAAATCTGAATGCCTAGTCTCTTCCCTAACAAATTATGTTGTTTTTCCCAAGTTTCTTGTCTTCTCAGTTGTT

ATATATATATATTCTCTTTT

In100 candidate3198 (in intron):

LOCUS ENSG00000122870

DESCRIPTION Protein bicaudal C homolog 1 (Bic-C).

[Source:Uniprot/SWISSPROTAcc:Q9H694]

ACCCAAGAATTCTACCACTCAAATAACAAGTGTTATTAATATTTTGGTATTTATCCTTTCAGTCTTTTTTCTAACACATT

CACTATGTATCCATATATCT

In100 candidate3199 (in intron):

LOCUS ENSG00000122870

DESCRIPTION Protein bicaudal C homolog 1 (Bic-C).

[Source:Uniprot/SWISSPROTAcc:Q9H694]

AGTGTTCCAATCCTAACCTTTGTTTTATTATTTCCCTATCCTGATTTTCCTTATGAGATTCTCTCAGTTCTTTATTTTAT

GTTTTTGTTTCTTTTGTGCT

In100 candidate3200 (in intron):

LOCUS ENSG00000122870

DESCRIPTION Protein bicaudal C homolog 1 (Bic-C).

[Source:Uniprot/SWISSPROTAcc:Q9H694]

AACTCTTCACATGTTTTTAACACATAAATTTCTATTGTTTATATTTCAGGGTTCTTTTTTATGTACTACACAGATTTAAT

AGCTGGTTTTTTTTTCCCT

In100 candidate3201 (in intron):

LOCUS ENSG00000122870

DESCRIPTION Protein bicaudal C homolog 1 (Bic-C).

[Source:Uniprot/SWISSPROTAcc:Q9H694]

AGAGAGCCACCCTGCCATCCAACGGAGCCTGGACTTGTAATAACTTTCCATACCTTTATTTTGTCCCAGGGATCCTTTTT

ACAGCTCCTCATTTAATTCA

In100 candidate3202 (in intron):

LOCUS ENSG00000152256

DESCRIPTION [Pyruvate dehydrogenase [lipoamide]] kinase isozyme 1,

mitochonrial precursor (EC 2.7.11.2) (Pyruvate dehydrogenase kinase isoform 1).

[Sorce:Uniprot/SWISSPROT;Acc:Q15118]

TTAAAAGTATATTTTGTTTGAAGTTAGTGTAACCATCCCAACTCTTTTTGTTTCTGTTTTCATAGTGTAACTTTCCAGAC

TTTTAGTTTAAACCTCTTTG

In100 candidate3203 (in intron):

LOCUS ENSG00000151150

DESCRIPTION Ankyrin-3 (ANK-3) (Ankyrin-G).

[Source:Uniprot/SWISSPROT;Acc:Q1955]

TTACCTTTCTGAGAACATTTGAGATAACTTTTCTTTTAATTTAGTTTTTCTGTTTCCCAAATGTCCCTAATTCCTCAGAT

TTCCTTTTTTTCCTTTTTTT

In100 candidate3204 (in intron):

LOCUS ENSG00000151150

DESCRIPTION Ankyrin-3 (ANK-3) (Ankyrin-G).

[Source:Uniprot/SWISSPROT;Acc:Q1955]

AAAAGGTGCCAAGACTATAACAAAATTCTCATTTTCCTAATTGTCCTCCATTCTTCTCACTTTTTGAATTCTGGACAGCC

GGAATTATTCCTTTTCTGTC

In100 candidate3205 (in intron):

LOCUS ENSG00000091436

DESCRIPTION Mitogen-activated protein kinase kinase kinase MLT (EC 2.7.11.2) (MLK-like mitogen-activated protein triple kinase) (Leucine zipper- and serile alpha motif-containing kinase) (Sterile alpha motif- and leucine zipprcontaining kinase AZK) (Mixed lineage [Source:Uniprot/SWISSPROT;Acc:Q9NYL]

TTTAATTTTTGTAACCTTGTTCACATTCTTTCCTTCACTTGTAATACCTTTGTCTTCCCTGTCTGGGCAGATCCTACCCA

TTCTTCAGCTGTAATTATTT

In100 candidate3206 (in intron):

LOCUS ENSG00000148082

DESCRIPTION SHC-transforming protein 3 (SH2 domain protein C3) (Src homolog 2 domain-containing-transforming protein C3) (Neuronal Shc) (N-Shc) (Proten Rai).

[Source:Uniprot/SWISSPROT;Acc:Q92529]

CTTATAGTTTTTTTCTTATATAAGGAATGGAGGCTAACTCTACTTTATTTCCTTTTGTTTTCTTTGGTTTTATTTCAGGC

TGATGTTCTGTTTCATTTTT

In100 candidate3207 (in intron):

LOCUS ENSG00000172845

DESCRIPTION Transcription factor Sp3 (SPR-2).

[Source:Uniprot/SWISSPROT;AccQ02447]

AGTAGTCTAACATCAAACATTTTTCAAACTTTTTTTTTTTTTTGGTCTCAGATGATTTTCTAATTTATTCCTCTCCCATT

TGGTATTTAGTGTACTTTAT

In100 candidate3208 (in intron):

LOCUS ENSG00000138430

DESCRIPTION Putative GTP-binding protein 9.

[Source:Uniprot/SWISSPROT;Acc:QNTK5]

AGTTTTTCATTCAAGTGCCAATAACTGCTTGAGGCGTTCTTTCATTCATTGATTCCTTCTTCAGTCATGTCTTCACTTGT

ATTTTTGCTCACCCCTTTTT

In100 candidate3209 (in intron):

LOCUS ENSG00000138430

DESCRIPTION Putative GTP-binding protein 9.

[Source:Uniprot/SWISSPROT;Acc:QNTK5]

TCATAGGAATGTATGCTATGATTATCTGAGAAGGAAAATTAATAATAGTAACTGCTGTTAATTCTTTACTATTTGCAGGC

TTTTCTAGGTTCCTTACAAT

In100 candidate3210 (in intron):

LOCUS ENSG00000187764

DESCRIPTION Semaphorin-4D precursor (Leukocyte activation antigen CD100)

(B18) (A8) (GR3). [Source:Uniprot/SWISSPROT;Acc:Q92854]

AGTTGTCTGTTCCCCTTTTTTTCATCTCTGCTACGTGTGTAACTTCTAGGAAACCTCTTTAGTTTACTTTCCAGCCTTTC

ATTTCTTTCACCACATTTTA

In100 candidate3211 (in intron):

LOCUS ENSG00000124177

DESCRIPTION Chromodomain-helicase-DNA-binding protein 6 (EC 3.6.1.-)

(ATPependent

helicase CHD6) (CHD-6) (Radiation-induced gene B protein).

[SourceUniprot/SWISSPROT;Acc:Q8TD26]

TTATAACGAAGTTGTCCTTTGTCTCCCATTTATTTATATCAGTATTGACTTGTGGATTTTGTTTTATTTTATAATCCATT

ACTCTATTTATTTTGTTTTC

In100 candidate3212 (in intron):

LOCUS ENSG00000150347

DESCRIPTION AT-rich interactive domain-containing protein 5B (ARID

domainontaining

protein 5B) (Mrf1-like) (Modulator recognition factor 2) (MRF-

2).[Source:Uniprot/SWISSPROT;Acc:Q14865]

CACTTTAACATATTAAGGCATCTTTTTCTTTCATTTGCGCACAATGCTGATTTTTGCAGTCCTATTTTACGCCTTAATGT

GACAACTTCCTGGTTTTTTT

In100 candidate3213 (in intron):

LOCUS ENSG00000138311

DESCRIPTION Protein ZNF365. [Source:Uniprot/SWISSPROT;Acc:Q70YC5]

CCCCAAAGCGGTTTATTTATAACATTTTCATTACTTATTGAACTTTATTTATGCTTACCTCCTTCCTAGCAGATTTAAAA

ATAGTTTTGTTTTTTTTTTT

In100 candidate3214 (in intron):

LOCUS ENSG00000138311

DESCRIPTION Protein ZNF365. [Source:Uniprot/SWISSPROT;Acc:Q70YC5]

CTTTTAATTTTTATAATTTCATTAACATGGAAGAGTTTTAACCTCTTATATACCTTCATCAGTTTCTTTCATAAACTTGG

ATTGTTCATGCATTTTTTTT

In100 candidate3215 (in intron):

LOCUS ENSG00000196090

DESCRIPTION Receptor-type tyrosine-protein phosphatase T precursor (EC

3.1..48) (R-PTP-T) (RPTP-rho). [Source:Uniprot/SWISSPROT;Acc:O14522]

AGTGGGCAAGTGCATTTTACAAGTATAACAAAACTTCCTTTTCTTCTTATTTGGACAGGTCCTACCAAATTCTCAATGAC

TTTCTTTTAATCTTTTTTTT

In100 candidate3216 (in intron):

LOCUS ENSG00000196090

DESCRIPTION Receptor-type tyrosine-protein phosphatase T precursor (EC

3.1..48) (R-PTP-T) (RPTP-rho). [Source:Uniprot/SWISSPROT;Acc:O14522]

CTGTGTGATTGGTCTTATAAACAACTTATGATGAGATGTTACATAACTAATTTTATTTTATTTTCTTCTTTCTTTCAGCC

TTTATTTTTTATTTTTATTT

In100 candidate3217 (in intron):

LOCUS ENSG00000196090

DESCRIPTION Receptor-type tyrosine-protein phosphatase T precursor (EC

3.1..48) (R-PTP-T) (RPTP-rho). [Source:Uniprot/SWISSPROT;Acc:O14522]

CATTAACCTGACCTATCTACTTTTATTTTTAACTGCCTCTATATACTCTTCCTTGGTAATGGCCTGTTTTCTTTTCAGCT

TGTTAAGTCTACTTGTTTCT

In100 candidate3218 (in intron):

LOCUS ENSG00000196090

DESCRIPTION Receptor-type tyrosine-protein phosphatase T precursor (EC

3.1..48) (R-PTP-T) (RPTP-rho). [Source:Uniprot/SWISSPROT;Acc:O14522]

CCTTGTATTCTCGATGCTAACGATTTGTGTCTTCTTTCTTTTCTTTCTCGATCAGTCTGGCTAGAGACTTGTCAGTTTTA

TTGATCTTTTCTTTTTTCTT

In100 candidate3219 (in intron):

LOCUS ENSG00000165025

DESCRIPTION Tyrosine-protein kinase SYK (EC 2.7.10.2) (Spleen tyrosine

kinae). [Source:Uniprot/SWISSPROT;Acc:P43405]

ATAACTTTAAACATGTGTTTTACTCCTTTATTTTAATTCTCTATTTCAGGGACATTAATTTTATATATTTTAGATCACCC

CTGTCTTTCTCAATTTTTCT

In100 candidate3220 (in intron):

LOCUS ENSG00000128656

DESCRIPTION N-chimaerin (NC) (N-chimerin) (Alpha chimerin) (A-chimaerin) (Ro

GTPase-activating protein 2). [Source:Uniprot/SWISSPROT;Acc:P15882]

TTTTAAAAAACTAACTCATAGTTGGTTTTTTTTCCTCTGTTGTTTTTCTAGTGTCTATTTTGTTTATTTCAGCTCTAATC

ACTGCTATTACCTTCCTTTT

In100 candidate3221 (in intron):

LOCUS ENSG00000115966

DESCRIPTION Cyclic AMP-dependent transcription factor ATF-2 (Activating trascription factor 2) (cAMP response element-binding protein CRE- BP1) (HB16) [Source:Uniprot/SWISSPROT;Acc:P15336]

TATGTCTATATTAAAATAACTGCCTATTACCTCAATATATTTTGTAGGTTTTTTCTTTTCTTTTTTAGAATTTTCAGGG

ATTTGCTCTTCCCTATTTTT

In100 candidate3222 (in intron):

LOCUS ENSG00000148090

DESCRIPTION Methylglutaconyl-CoA hydratase, mitochondrial precursor (EC 4.21.18) (AU-specific RNA-binding enoyl-CoA hydratase) (AU-binding protein/enol-CoA hydratase). [Source:Uniprot/SWISSPROT;Acc:Q13825]

TACTGTAACTTAATGTGTTTTAGTGTTAATGGATGTCCTTTTTTCTCTTTTGCTATCCCAGCCCCTTCCTTGCCTAATTG

TTACTCTCTTCTGTGTGGTT

In100 candidate3223 (in intron):

LOCUS ENSG00000183230

DESCRIPTION Catenin alpha-3 (Alpha T-catenin) (Cadherin-associated protein) [Source:Uniprot/SWISSPROT;Acc:Q9UI47]

ATTCGTTCATGTCATTGCATGTATACTTGGTTCATTCTTTCTTCTTGCATAACATGTGATGGTATTCTTGTGCCACAGAT

TGTTTATCCATTCACTTGTA

In100 candidate3224 (in intron):

LOCUS ENSG00000183230

DESCRIPTION Catenin alpha-3 (Alpha T-catenin) (Cadherin-associated protein)

[Source:Uniprot/SWISSPROT;Acc:Q9UI47]

AATTTTTTAAATTTTATCAAATTATTATGTGCTTAACCCCTCTTCACTTCCCAATTATTTTCCAGAGACAACTATTTTTA

AATTCTTGGCTCTTTCTTCT

In100 candidate3225 (in intron):

LOCUS ENSG00000183230

DESCRIPTION Catenin alpha-3 (Alpha T-catenin) (Cadherin-associated protein)

[Source:Uniprot/SWISSPROT;Acc:Q9UI47]

CTGGCATGAAGATTTTCTTTGAGATGGAATCTGAGTAACAATTATAATTTTGTATTCTTTTTCTTTTTCTTGAGACAGAG

TCTTGCTCTGTCGCTCTGTT

In100 candidate3226 (in intron):

LOCUS ENSG00000183230

DESCRIPTION Catenin alpha-3 (Alpha T-catenin) (Cadherin-associated protein)

[Source:Uniprot/SWISSPROT;Acc:Q9UI47]

CACAATAAATTCCATTCTCTTCACATAACCATCAAGTGACTTTGGGTCCTTTTATTCTTTTCTTTTTTTCACAGTAACCT

CTCTCTATTTCTCCATTTTT

In100 candidate3227 (in intron):

LOCUS ENSG00000183230

DESCRIPTION Catenin alpha-3 (Alpha T-catenin) (Cadherin-associated protein)

[Source:Uniprot/SWISSPROT;Acc:Q9UI47]

TCCTGTCAATACTCCATTCTAAAAATGGATTGTAACTTTCTACTTAGTTCCTCACCCTCTGTTAGTGTCTTAGTTCAGGT

TTTCATCATTTTTCACAGCT

In100 candidate3228 (in intron):

LOCUS ENSG00000183230

DESCRIPTION Catenin alpha-3 (Alpha T-catenin) (Cadherin-associated protein)

[Source:Uniprot/SWISSPROT;Acc:Q9UI47]

CTCTTTTAATCATTGAACATGGGATAACTCTACTTGTGTCTTCTTCAATTTCTTTTATCAATGTATCAGAGTTTTCAATC

CACAGGTCTTTTACCTCGTT

In100 candidate3229 (in intron):

LOCUS ENSG00000183230

DESCRIPTION Catenin alpha-3 (Alpha T-catenin) (Cadherin-associated protein)

[Source:Uniprot/SWISSPROT;Acc:Q9UI47]

GTCTACTTAAAGATTTGTCCATTTTGATTAACTTCCAAAAAGCCACTTTTGTTGATTTTTTTTCTATCATTTTCTCAGTC

ACTATTTCATTTATTTCTGC

In100 candidate3230 (in intron):

LOCUS ENSG00000183230

DESCRIPTION Catenin alpha-3 (Alpha T-catenin) (Cadherin-associated protein)

[Source:Uniprot/SWISSPROT;Acc:Q9UI47]

TTATTCTAAATTTAAAATGCCATGATAACTGGTAAAGAAAATTTTCATAACTATTGTTTCTTTTTTTTCTTCCAGAATAC

ACTGTTTATTCTTAACCTTT

In100 candidate3231 (in intron):

LOCUS ENSG00000183230

DESCRIPTION Catenin alpha-3 (Alpha T-catenin) (Cadherin-associated protein)

[Source:Uniprot/SWISSPROT;Acc:Q9UI47]

CAAGTAGTAACCATGTTGATACTGTGTCTTTAGACTGGCTCTCCTTTCTTTCCTGATTCGTTCTTCCCAGGCTCCCAGTT

CCGTTTACTAATATCTATTT

In100 candidate3232 (in intron):

LOCUS ENSG00000183230

DESCRIPTION Catenin alpha-3 (Alpha T-catenin) (Cadherin-associated protein)

[Source:Uniprot/SWISSPROT;Acc:Q9UI47]

TTATCTAAATCTCATCAATTATCCTTTCCCTTCATTTTAACCTCATTTTACAAATTACTTGCTTCTTCCATCTCTCAGTC

ATTTCTTTTTCTTTACATTT

In100 candidate3233 (in intron):

LOCUS ENSG00000183230

DESCRIPTION Catenin alpha-3 (Alpha T-catenin) (Cadherin-associated protein)

[Source:Uniprot/SWISSPROT;Acc:Q9UI47]

ACCTAGATTTAACTAAATATCTTTCTATTTCATAATTTTTCATCTGCAGTCATTGCCTTTTGACTCCCCATAAATTTATA

CCAATGTTTTTCAGTTCCTT

In100 candidate3234 (in intron):

LOCUS ENSG00000183230

DESCRIPTION Catenin alpha-3 (Alpha T-catenin) (Cadherin-associated protein)

[Source:Uniprot/SWISSPROT;Acc:Q9UI47]

TACACTAAGCAATTTTTTAACTATTTTTTTTGCCCATTTCTTTTATTTAAATTTTGATTTCTCCTTGACTTTTATCAGTT

ACTTCCTTTTGATCCTTTTC

In100 candidate3235 (in intron):

LOCUS ENSG00000183230

DESCRIPTION Catenin alpha-3 (Alpha T-catenin) (Cadherin-associated protein)

[Source:Uniprot/SWISSPROT;Acc:Q9UI47]

CTCCCATTCGGTGGGTTTTCTATTAACTCTGCTGATTATTTCTTTTTCTGTGCAGAAGCTTTCCATTTTAATTAAGTACC

ATCTATTTATCCTTGTTTTT

In100 candidate3236 (in intron):

LOCUS ENSG00000183230

DESCRIPTION Catenin alpha-3 (Alpha T-catenin) (Cadherin-associated protein)

[Source:Uniprot/SWISSPROT;Acc:Q9UI47]

TGCAATGTCACTTTTATAACTTCTGACTGTGCTTATTTGAATCCTCTCTCTTTTTTTCTTTGTTAATCTAGCTAGCAGTC

TACCGATCCTGTTTATTTTT

In100 candidate3237 (in intron):

LOCUS ENSG00000169071

DESCRIPTION Tyrosine-protein kinase transmembrane receptor ROR2 precursor (C

2.7.10.1) (Neurotrophic tyrosine kinase, receptor-related 2).

[Source:Unirot/SWISSPROT;Acc:Q01974]

TAGATCTTGTATCTATTAGGAGACACTGAAGTCCCTGATAACAACTGAGGATTTTTCCATTTCTCTTTTCAGATCGTTCA

GTTTTGGCTTCATGTTGTTT

In100 candidate3238 (in intron):

LOCUS ENSG00000169071

DESCRIPTION Tyrosine-protein kinase transmembrane receptor ROR2 precursor (C

2.7.10.1) (Neurotrophic tyrosine kinase, receptor-related 2).

[Source:Unirot/SWISSPROT;Acc:Q01974]

AACCCATTTATAATTATTATGATAACTCATATGTGTTATCTATTTATTCAGTCTTATTTTATGTTTTCTGTTTATGTTGC

TTTATTTTAAAATTTTCTTT

In100 candidate3239 (in intron):

LOCUS ENSG00000198739

DESCRIPTION Leucine-rich repeat transmembrane neuronal protein 3

precursor.[Source:Uniprot/SWISSPROT;Acc:Q86VH5]

TAATGATAATTAATAACTTTATTAACAATTCTCCATGTTTTCTTTTTATTTTTAAGGGCTCTCACTTCTCCCAATCAGGA

TTCTCATTTATCCTTTCTCT

In100 candidate3240 (in intron):

LOCUS ENSG00000090054

DESCRIPTION Serine palmitoyltransferase 1 (EC 2.3.1.50) (Long chain base bisynthesis protein 1) (LCB 1) (Serine-palmitoyl-CoA transferase 1) (SPT 1) (PT1). [Source:Uniprot/SWISSPROT;Acc:O15269]

ATGCAAGATCATAACTCACTCTGCCTATTCTTGCCTTTGATCTTATTTTATATTTTCTTCCTGTTTGGCTTATTCCAGAT
TTTTCTGAGATACTTTATTT

In100 candidate3241 (in intron):

LOCUS ENSG00000150455

DESCRIPTION Toll-interleukin 1 receptor domain-containing adapter protein (IR domain-containing adapter protein) (MyD88 adapter-like protein) (Adaptorprotein Wyatt). [Source:Uniprot/SWISSPROT;Acc:P58753]

CTGGATATATATTTTTAAACATTTTAACCCTCGGGAGATTGCCTTTTCACTCTCCTATTTATGTCTTTTGATGAACAGAA
GTTCTTCATTCTAATGTTTT

In100 candidate3242 (in intron):

LOCUS ENSG00000108176

DESCRIPTION DnaJ homolog subfamily C member 12 (J domain-containing protein1). [Source:Uniprot/SWISSPROT;Acc:Q9UKB3]

TACTTCTCAAGTTGCTCATGAATTGCTGAGTTTTAACCATTGTAAATTCACATTCTCCATCTTTTTAGTTTATGTCAGCT
TGCTCTCTTTTCAAGGCTTA

In100 candidate3243 (in intron):

LOCUS ENSG00000116044

DESCRIPTION Nuclear factor erythroid 2-related factor 2 (NF-E2-related factr 2) (NFE2-related factor 2) (Nuclear factor, erythroid derived 2, like 2) HEBP1). [Source:Uniprot/SWISSPROT;Acc:Q16236]

AGCTAACACATATTTACTTATCATCCTTTTCTTTCTAGCTGATTACAGGTTTCCTCTTTTCTTTTTTGTCTTCCTGGTAT

CCTTGTCTGTTCCCTCTGTT

In100 candidate3244 (in intron):

LOCUS ENSG00000116044

DESCRIPTION Nuclear factor erythroid 2-related factor 2 (NF-E2-related factr

2) (NFE2-related factor 2) (Nuclear factor, erythroid derived 2, like 2) HEBP1).

[Source:Uniprot/SWISSPROT;Acc:Q16236]

TGTCGTAATTTTAATAAGTAACTTGAATTGTTAACAATTTACTCTTGTCTTTTTCTTGGACTCAGAAATGTTATTTTATT

ATTATGTCTCTGCATCTTTC

In100 candidate3245 (in intron):

LOCUS ENSG00000018510

DESCRIPTION Alkyldihydroxyacetonephosphate synthase, peroxisomal precursor EC

2.5.1.26) (Alkyl-DHAP synthase) (Alkylglycerone-phosphate synthase)

(Agigassociated

gene 5 protein). [Source:Uniprot/SWISSPROT;Acc:O00116]

TTGTATTCTCCTTTGGGTAGTTTATAACATAGCCTTTACTTTTTACGTGCCTTTTTTTGGTTCTGTTTCTTTCCTCAGGT

TAGTCAGCTCTTGTTTCATT

In100 candidate3246 (in intron):

LOCUS ENSG00000018510

DESCRIPTION Alkyldihydroxyacetonephosphate synthase, peroxisomal precursor EC 2.5.1.26) (Alkyl-DHAP synthase) (Alkylglycerone-phosphate synthase) (Agigassociated

gene 5 protein). [Source:Uniprot/SWISSPROT;Acc:O00116]

CTTGATCTTCTGAAAAACATAGACTAACCTATTAATATAAACTTTTTTCTCTTCTTAGGTCATGCTCTTAAACCTCAGGT

TTCCTCTATTTTTACATCAT

In100 candidate3247 (in intron):

LOCUS ENSG00000128655

DESCRIPTION Dual 3',5'-cyclic-AMP and -GMP phosphodiesterase 11A (EC 3.1.4.7) (EC 3.1.4.35) (cAMP and cGMP phosphodiesterase 11A). [Source:Uniprot/SWISPROT;Acc:Q9HCR9]

CATGGAAGTTCATTCTTTTTTCCACTAACTTCCATAGTAATTTATCTGTATGACTCTTGCTATTTATTCCAGTTTTGGTT

GCTTTAATTTTGGCTCACCT

In100 candidate3248 (in intron):

LOCUS ENSG00000128655

DESCRIPTION Dual 3',5'-cyclic-AMP and -GMP phosphodiesterase 11A (EC 3.1.4.7) (EC 3.1.4.35) (cAMP and cGMP phosphodiesterase 11A). [Source:Uniprot/SWISPROT;Acc:Q9HCR9]

ATTTTTTATGAATTGGATTATTTTGTAACCATCCATTATTTTTATCTTAATTTTATTACAGAATTACCCTCTCTTTTCCC

CAAAGTGCTATTTTCTTATT

In100 candidate3249 (in intron):

LOCUS ENSG00000128655

DESCRIPTION Dual 3',5'-cyclic-AMP and -GMP phosphodiesterase 11A (EC 3.1.4.7)

(EC 3.1.4.35) (cAMP and cGMP phosphodiesterase 11A).

[Source:Uniprot/SWISPROT;Acc:Q9HCR9]

CAATAGGCCCTTCTTTAAAAAATATTTCTCTAACTTCCTGGATGTTGATTTTTTAAAATTCCCTTTCAGAATGTTTTCTG

CTGGGGTCTATTCCACTTTC

In100 candidate3250 (in intron):

LOCUS ENSG00000108187

DESCRIPTION Probable isomerase MAWBP (EC 5.1.-.-) (MAWD-binding protein)

(Uknown protein 32 from 2D-page of liver tissue).

[Source:Uniprot/SWISSPROT;Ac:P30039]

TTATGGTAACTTGATATTTTCATCCTTTTTTTTTTTCACTCTCCATCAGTGGAGTCCCAACTAGATATTTTCTTGTGTTC

CATTGCTATTTCTCCTTCTC

In100 candidate3251 (in intron):

LOCUS ENSG00000170322

DESCRIPTION Nuclear factor related to kappa-B-binding protein (DNA-binding

rotein R kappa-B). [Source:Uniprot/SWISSPROT;Acc:Q6P4R8]

TAACACGGTTGTGACCTTTTCCTCTCTGTCAGCTTCCTTGTAACAGCTGTGGGAAACCTTTTCCACTCTACATTTCCAGT

CTTTTGTGCTCCCTTTTGTT

In100 candidate3252 (in intron):

LOCUS ENSG00000155657

DESCRIPTION Titin (EC 2.7.11.1) (Connectin) (Rhabdomyosarcoma antigen MU-RM-40.14). [Source:Uniprot/SWISSPROT;Acc:Q8WZ42]

ACCTTTTGCTCATTAGTAACAAAGATAGAGATTTTTTTCTACAGCCTTCAAAGGTATCTATTTTAGACTTTTCATATCT
TGATCTTCTCAGCTCTCTTT

In100 candidate3253 (in intron):

LOCUS ENSG00000155657

DESCRIPTION Titin (EC 2.7.11.1) (Connectin) (Rhabdomyosarcoma antigen MU-RM-40.14). [Source:Uniprot/SWISSPROT;Acc:Q8WZ42]

TGTCTTATTGTCTCTTAAATCATTTGCATTCTTCTAACATGTAGCATAGTTTCTTTGATATTCATTAATTCCAGTTGCTA
ACTGTGAGCCTTTCTTCCTT

In100 candidate3254 (in intron):

LOCUS ENSG00000155657

DESCRIPTION Titin (EC 2.7.11.1) (Connectin) (Rhabdomyosarcoma antigen MU-RM-40.14). [Source:Uniprot/SWISSPROT;Acc:Q8WZ42]

AGTAAAGTAGTTTTTTACCAATCATGTATAAATAGAATGTAAATCTAACAAGATAGGAAGTAGTCTTTCTATTCCCAGTT
AGTTGTTTGCTTTCTCTGTT

In100 candidate3255 (in intron):

LOCUS ENSG00000155657

DESCRIPTION Titin (EC 2.7.11.1) (Connectin) (Rhabdomyosarcoma antigen MU-RM-40.14). [Source:Uniprot/SWISSPROT;Acc:Q8WZ42]

TAATGTAAATAAAAATAGGTCACAAAATTAACCCTGTGGTCTTTCTAAGGTTTCTAAACCTCCTTTTCTCAGGTTTCTAA
ACCTCCTTTTCTCAGAGGTT

In100 candidate3256 (in intron):

LOCUS ENSG00000138346

DESCRIPTION DNA2-like helicase (EC 3.6.1.-) (DNA replication ATP-dependent elicase-like homolog). [Source:Uniprot/SWISSPROT;Acc:P51530]

TGTGAGCCACTACACTTGGCCTCAAATGTTTTTTTATGTAAAACGTAACTTGTTTTAGTTTCATTTTTTTCTTTCCAGAA

TGAATTTTTTTTTTTTTTTT

In100 candidate3257 (in intron):

LOCUS ENSG00000188312

DESCRIPTION Centromere protein P (CENP-P). [Source:Uniprot/SWISSPROT;Acc:Q6PU0]

TTCCACTTAACTAAAAAAATTGACCTCATCTTTCTTTAGCAGTTATGAGTACATTATTATTACAATGTTTTTAAGTTGAT

TTCTCTTCCTTTCTTTTCCC

In100 candidate3258 (in intron):

LOCUS ENSG00000138336

DESCRIPTION CXXC-type zinc finger protein 6 (Leukemia-associated protein wih a CXXC domain). [Source:Uniprot/SWISSPROT;Acc:Q8NFU7]

TGGTTCCATAATAATGATAACTACCACTTATTTTCTCTCTCTCTTTTTTTTTTTTGTATTTTAGAGTTGCAAACAGGC

TTTTTTTTTTTAGCTCCTAT

In100 candidate3259 (in intron):

LOCUS ENSG00000204580

DESCRIPTION Epithelial discoidin domain-containing receptor 1 precursor (EC2.7.10.1) (Epithelial discoidin domain receptor 1) (Tyrosine kinase DDR) (Dscoidin receptor tyrosine kinase) (Tyrosine-protein kinase CAK) (Cell adheson kinase) (TRK E) (Protein-tyrosine ki [Source:Uniprot/SWISSPROT;Acc:Q0834]

TAACATGAATGCTAGATCTTTTGTTACAGTCACATAGTTATGTTCATTGTGGTCTGAGGTTACGTTCATTTTTTTCCTAT

CTATTTTCTCCCTGTTGTTC

In100 candidate3260 (in intron):

LOCUS ENSG00000196323

DESCRIPTION Zinc finger and BTB domain-containing protein 44 (BTB/POZ domaicontaining

protein 15). [Source:Uniprot/SWISSPROT;Acc:Q8NCP5]

TTTCTGAAGTAACTCCTACTATGCTGGTTATTCTGTGTATCTATCTCTTTCACAGGTTTCCCTTCTGATACCCCGCCTTC

ACTTTTTTCCTCTTTTTTTT

In100 candidate3261 (in intron):

LOCUS ENSG00000196323

DESCRIPTION Zinc finger and BTB domain-containing protein 44 (BTB/POZ domaicontaining

protein 15). [Source:Uniprot/SWISSPROT;Acc:Q8NCP5]

GTCTGCAAGGAAACCTAACTTCTGAAGCTACCCATTAGCTTCTATTTTTCTTGGCCAGATAAACATTCTCTTGACCACCG

TCTTTTACCTAAATCTTTTT

In100 candidate3262 (in intron):

LOCUS ENSG00000127080

DESCRIPTION Inositol-pentakisphosphate 2-kinase (EC 2.7.1.158) (Inositol-13,4,5,6-pentakisphosphate 2-kinase) (Ins(1,3,4,5,6)P5 2-kinase) (InsP5 2-kiase) (IPK1 homolog). [Source:Uniprot/SWISSPROT;Acc:Q9H8X2]

AACCTTATTTTATCTCCCACACATCTTAACTGCTCTTTCATATTTTTCATATCTTTGTCTCCTTGTGCTACATTCCAGGTGCTTTTCTTTTTTCAGCTCT

In100 candidate3263 (in intron):

LOCUS ENSG00000120451

DESCRIPTION Sorting nexin-19. [Source:Uniprot/SWISSPROT;Acc:Q92543]

TAGATGGCACATAGTGAACGCTCCATAAATAACACGATTCTTATTGTTACTCATCTTCAAATTCTGTTATCCCAGGCTGTTGAATTGTCTTGGTTTCCTT

In100 candidate3264 (in intron):

LOCUS ENSG00000185963

DESCRIPTION Protein bicaudal D homolog 2 (Bic-D 2). [Source:Uniprot/SWISSPRT;Acc:Q8TD16]

GGAAAGAATGAAAACCGCAAAGGTAACTGTAACCTGTTGTTGGTCGTAATCTTTTACTCTTCCTTCATCGTCCAGCTTCTGTTTCTGGATCTCAGCCTCT

In100 candidate3265 (in intron):

LOCUS ENSG00000183715

DESCRIPTION Opioid-binding protein/cell adhesion molecule precursor (OBCAM)(Opioid-binding cell adhesion molecule) (OPCML). [Source:Uniprot/SWISSPROT;cc:Q14982]

GGCTGGGGGAAATTGATTTCTTTTAAACTAACACTGAATGAATTTCTATTGTATTTCAGGCACTGGGACATTTCTATTTC

TTGTCTCATTTAAATTTCTC

In100 candidate3266 (in intron):

LOCUS ENSG00000183715

DESCRIPTION Opioid-binding protein/cell adhesion molecule precursor

(OBCAM)(Opioid-binding cell adhesion molecule) (OPCML).

[Source:Uniprot/SWISSPROT;cc:Q14982]

GCTGAATCTGGAGAAGCTAACCCCCACTCACAACTGGATTTCTTTTTCTTTTCTGATCCATGACCTATCCAGTTTGATTT

CAAGCCTTGCTATATCCTTT

In100 candidate3267 (in intron):

LOCUS ENSG00000183715

DESCRIPTION Opioid-binding protein/cell adhesion molecule precursor

(OBCAM)(Opioid-binding cell adhesion molecule) (OPCML).

[Source:Uniprot/SWISSPROT;cc:Q14982]

CATATGTGTGCAAGTTATTACTGGTACTAAGTTGAACTCCTCAATAACAACTGACAATACTCACACATTTTTTTCAGTTT

TGTCTGCTCCATCTCGTTCT

In100 candidate3268 (in intron):

LOCUS ENSG00000183715

DESCRIPTION Opioid-binding protein/cell adhesion molecule precursor

(OBCAM)(Opioid-binding cell adhesion molecule) (OPCML).

[Source:Uniprot/SWISSPROT;cc:Q14982]

AATTGTTCTCTGTCTCCTGCTTTCTTATAACCTAACAATCTCTCTTGGTTTGCTGTCCATTTGGGGCCTTTCTTTCAGGT

CTCCTTTCTGGATTTCTCTC

In100 candidate3269 (in intron):

LOCUS ENSG00000183715

DESCRIPTION Opioid-binding protein/cell adhesion molecule precursor (OBCAM)(Opioid-binding cell adhesion molecule) (OPCML). [Source:Uniprot/SWISSPROT;cc:Q14982]

ATGAGTAGTCCTTTATTTTTCAATTAGGATAATCACTGACTTTTATATTTAACCCAAATTATATTCTCTTGGTCTCAGCA
TACGTTTTGCTTCTTCACTG

In100 candidate3270 (in intron):

LOCUS ENSG00000127084

DESCRIPTION FYVE, RhoGEF and PH domain-containing protein 3 (Zinc finger FYE domain-containing protein 5). [Source:Uniprot/SWISSPROT;Acc:Q5JSP0]

ATCTTTTTGGTGACTTGACCTTTTTATAACTATATAATGTCATTCTTTGCTTCTTGTAACAGTTATTTTTTTTCCAGTTG
GCAGCAGTGATTCTTTTTTT

In100 candidate3271 (in intron):

LOCUS ENSG00000166086

DESCRIPTION Junctional adhesion molecule C precursor (JAM-C) (Junctional adesion molecule 3) (JAM-3) (JAM-2). [Source:Uniprot/SWISSPROT;Acc:Q9BX67]

GTTAACCTCCTTTGTCATTTTTTCATGTTCTTGTCTCTCTGTGCTATTCTGGGTTCTTTATTCATTTTTGTTTTACAGCT
AACTTTTTCTGCTTCCAGCT

In100 candidate3272 (in intron):

LOCUS ENSG00000166086

DESCRIPTION Junctional adhesion molecule C precursor (JAM-C) (Junctional adesion molecule 3) (JAM-3) (JAM-2). [Source:Uniprot/SWISSPROT;Acc:Q9BX67]

TGTCACTGTATTCTGAGTAACCCTGAGCATTGCCATTTTTCTTCTTTTGTTATCATGGCCTGTCTACCTCAAATGCAGGT

CATGATCTTGTCTTTCTGTT

In100 candidate3273 (in intron):

LOCUS ENSG00000166086

DESCRIPTION Junctional adhesion molecule C precursor (JAM-C) (Junctional adesion molecule 3) (JAM-3) (JAM-2). [Source:Uniprot/SWISSPROT;Acc:Q9BX67]

AGGTTTAGCGATGAGTAGTTGCTACTCTTTTGTGTCATGTTTTCCTAACTCCTTCCCCTATTTGTTGTTTTCCAGGTTCT

TCCTGCTTCTTTGAAAGTGT

In100 candidate3274 (in intron):

LOCUS ENSG00000198954

DESCRIPTION Uncharacterized protein KIAA1279.

[Source:Uniprot/SWISSPROT;AccQ96EK5]

GTTTTTAAAGAAATTAAAAAAAAAAAAGTAACATTGATACAATTCTTTTATCTACACTGCATATTTCTATTTCATCAGTT

GTTGCCATAATATTCCTTCT

In100 candidate3275 (in intron):

LOCUS ENSG00000122958

DESCRIPTION Vacuolar protein sorting-associated protein 26A (Vesicle protei sorting 26A) (hVPS26). [Source:Uniprot/SWISSPROT;Acc:O75436]

AACTGAATTGCTGTCTGCATCATATCCTAACCCTCAACAAATTTTATTCTCTTTCCTTCTAGTCTTTACCAGCTTGACAG

TTGTGTATTTTACTTCCTCT

In100 candidate3276 (in intron):

LOCUS ENSG00000187231

DESCRIPTION SEC14 and spectrin domains 1 [Source:RefSeq_peptide;Acc:NP_83524]

AAGTATTCTGTTGTTGGATGGAGTGTTCTGTAACTGTCAAGATGATTCTTAATGTTTTTCAGGTCTTCTCTATCCATGGT

AATGTTGTGTCTGCCTGTTT

In100 candidate3277 (in intron):

LOCUS ENSG00000144331

DESCRIPTION Zinc finger protein 533. [Source:Uniprot/SWISSPROT;Acc:Q569K4]

EXON

ATGTAGATATATATGTAATTTATATACACATACATAATTTAGTATAACATAGTTATGATATCGTTTTCCTTGTACCAGCC

CTATTATCTCAATTACATTT

In100 candidate3278 (in intron):

LOCUS ENSG00000115232

DESCRIPTION Integrin alpha-4 precursor (Integrin alpha-IV) (VLA-4) (CD49d atigen). [Source:Uniprot/SWISSPROT;Acc:P13612]

CTGTAACTCCTCATCTCATTCTGATCCTCTATTGGCCAGGATAATATGATACATTCCTCACTGGTTTTTCTCATGTATTT

TTAAGCCTTCCTATCTTCCT

In100 candidate3279 (in intron):

LOCUS ENSG00000147099

DESCRIPTION Histone deacetylase 8 (HD8). [Source:Uniprot/SWISSPROT;Acc:Q9BY1]

TTTTCTCCCACTCTGTGGGTTGTTAACTCTGCTGATTATTTCTTTTGCTGTGCAGAAGCATTTTAGCTTAATTATGTTTC

ATCTATTTATCTTTATTTTT

In100 candidate3280 (in intron):

LOCUS ENSG00000147099

DESCRIPTION Histone deacetylase 8 (HD8). [Source:Uniprot/SWISSPROT;Acc:Q9BY1]

AGAGTCCTTGTCTACTAACTCCATCATTTCTGTCATTTCTGGGTCTGTTTCTGTTAACTGATTTTTCTTCAGATTATAGG

TCACATTTGCCTACTTCTTT

In100 candidate3281 (in intron):

LOCUS ENSG00000150722

DESCRIPTION protein phosphatase 1, regulatory (inhibitor) subunit 1C

[Sourc:RefSeq_peptide;Acc:NP_001074014]

TCTCTTCACTTATAAAACATTTTCTAACATTTCTTTTATCCTCTCATTCTAAATCTGCTCTTTTATATGATTTTGCAGAT

TTTTATTTATTTATTTATTT

In100 candidate3282 (in intron):

LOCUS ENSG00000150722

DESCRIPTION protein phosphatase 1, regulatory (inhibitor) subunit 1C

[Sourc:RefSeq_peptide;Acc:NP_001074014]

GGTGATATCCCCTTTATAACTTTTTAATGTGTCTATTTGATTCTTCTCTCTTTTCTTCCTTATTAGTCTGGCTAGCAGTC

TATCTATTTTGTTAATCTTT

In100 candidate3283 (in intron):

LOCUS ENSG00000175787

DESCRIPTION Zinc finger protein 169. [Source:Uniprot/SWISSPROT;Acc:Q14929]

EXON

TCTTAACTATGATTATGCATTGGTCTATTTCTCTTTCTCCTATTCTGTCATTTTTCTTCCTGTATTTTCTCTCAGTCCGT

GGGTTGTCTTTTCATTATCT

In100 candidate3284 (in intron):

LOCUS ENSG00000148110

DESCRIPTION Hippocampus abundant transcript-like protein 1.

[Source:UniprotSWISSPROT;Acc:Q5SR56]

AGGCGTGAGCCACCGTGCCCGGCCTCTAACATTTAATAGTACTAATTTTTCCTCTGTCCCTTTCTTTTTCTCCTTCAGGA

TGTTTTTATATTCTTGTCCA

In100 candidate3285 (in intron):

LOCUS ENSG00000184911

DESCRIPTION Doublesex- and mab-3-related transcription factor C1.

[Source:Uiprot/SWISSPROT;Acc:Q5HYR2]

AGTAGCCTCTAACGATCCTTTGAATTTCTGCACTATCAGTTGTAATGTCTCCCTTATCATCCCTGATTTTATTTATTTGG

TTCTTTTCTTTTATTTCTCA

In100 candidate3286 (in intron):

LOCUS ENSG00000148120

DESCRIPTION Aminopeptidase O (EC 3.4.11.-) (AP-O).

[Source:Uniprot/SWISSPRO;Acc:Q8N6M6]

AACTTGGTGGAGAAAAAGAACATAATAAATTCGAGAAAATAACCATTCTTTTACACTTCTTTTTTCTCATTCAGTTTTGC

TCTTTACTTTTTGTAATACC

In100 candidate3287 (in intron):

LOCUS ENSG00000148120

DESCRIPTION Aminopeptidase O (EC 3.4.11.-) (AP-O).

[Source:Uniprot/SWISSPRO;Acc:Q8N6M6]

TCATCTTTGTAGCCCCCACTAAAAGCCCTCTCTTGAGTGTAACAATAACTTCTTCCTGACAATTTCATTTCTTCTCAGTT

TTTATCTTGCCAATCCTGTC

In100 candidate3288 (in intron):

LOCUS ENSG00000159123

DESCRIPTION Doublesex- and mab-3-related transcription factor C1.

[Source:Uiprot/SWISSPROT;Acc:Q5HYR2]

AGTAGCCTCTAACGATCCTTTGAATTTCTGCACTATCAGTTGTAATGTCTCCCTTATCATCCCTGATTTTATTTATTTGG

TTCTTTTCTTTTATTTCTCA

In100 candidate3289 (in intron):

LOCUS ENSG00000204116

DESCRIPTION Cysteine-rich hydrophobic domain 1 protein (Brain X-linked

protin). [Source:Uniprot/SWISSPROT;Acc:Q5VXU3]

AAAAATTAAATGAATTTGCCTAGGATTTTTCATAGCTAATAAGTAACTATAATTCAAGTCTCTCGATCTGTTTTTCAGTG

TTTTTAGTTTGTCTTAGTTT

In100 candidate3290 (in intron):

LOCUS ENSG00000064989

DESCRIPTION Calcitonin gene-related peptide type 1 receptor precursor

(CGRPtype 1 receptor) (Calcitonin receptor-like receptor).

[Source:Uniprot/SWISSROT;Acc:Q16602]

TGCTTTTATTTGCCTTTCACCTTTATTTACACATATATCTGTAACTACTGAACTAATTTTTGTGTTCATTGTTGCCAGAT

GTGTTTAAATCTCTCTTCTG

In100 candidate3291 (in intron):

LOCUS ENSG00000064989

DESCRIPTION Calcitonin gene-related peptide type 1 receptor precursor

(CGRPtype 1 receptor) (Calcitonin receptor-like receptor).

[Source:Uniprot/SWISSROT;Acc:Q16602]

TTTCTATCCTAGCTGTACCAAATGACCATAAATTTAGTAACTTAAAACAACATTTATTTACTATCTATCTCACAGTTCTA

TTGGTCAGTTCTGTTGCCTT

In100 candidate3292 (in intron):

LOCUS ENSG00000064989

DESCRIPTION Calcitonin gene-related peptide type 1 receptor precursor

(CGRPtype 1 receptor) (Calcitonin receptor-like receptor).

[Source:Uniprot/SWISSROT;Acc:Q16602]

TGACCAAAATTCTCATTTTTTCTTAGATCACATCTGAATATATTTAACCAATATTGTGACCTGTTATCTCATTTCCAGTT

TCTTCATATTTTTCTTCAAT

In100 candidate3293 (in intron):

LOCUS ENSG00000144366

DESCRIPTION GULP, engulfment adaptor PTB domain containing 1

[Source:RefSeqpeptide;Acc:NP_057399]

AACATCCATAGTTAACATTTATATTGTTACTTTGTTGTTTTTGAAATTGTTCCTTTTCACAGTTTGAATCCAATGGCATT

TTTCTTTTCGATCCTGTCTT

In100 candidate3294 (in intron):

LOCUS ENSG00000144366

DESCRIPTION GULP, engulfment adaptor PTB domain containing 1

[Source:RefSeqpeptide;Acc:NP_057399]

CTTCCAAAGAACTAACTTTTGGGTTTGTTCATTGTTTTTATGGATTTTTTTCTCTCAATTTCATTCAATTCCACTCAGAT

TTTAGTTATATCTTCTGCTA

In100 candidate3295 (in intron):

LOCUS ENSG00000050030

DESCRIPTION Uncharacterized protein KIAA2022.

[Source:Uniprot/SWISSPROT;AccQ5QGS0]

GTGGTACGTTTGTCACAATTAATGAACTGATATTGATATATTATAATTAACTAAAGTCTATACTTTATATTTCTTCAGTT

TTCTTCTAATGTCCTTTTTC

In100 candidate3296 (in intron):

LOCUS ENSG00000050030

DESCRIPTION Uncharacterized protein KIAA2022.

[Source:Uniprot/SWISSPROT;AccQ5QGS0]

CCTTGATCTTTAAATGATGACAAATTGTCCAAATGTATTAACTTTTCCCCTTTATTTTTCTCTTTGCTTTATTCTCAGTA

ACCTCTTTTCTCTAATTCTT

In100 candidate3297 (in intron):

LOCUS ENSG00000050030

DESCRIPTION Uncharacterized protein KIAA2022.

[Source:Uniprot/SWISSPROT;AccQ5QGS0]

TCATTATGGTTTGGTGGTTTTCCGTAGTCATAACATTTGAGTCCTTTTTTGTCCTTGTTTGTCTGTTTGCTCTACCAGTG

GTTTTATACTTTTGTGTCTT

In100 candidate3298 (in intron):

LOCUS ENSG00000050030

DESCRIPTION Uncharacterized protein KIAA2022.

[Source:Uniprot/SWISSPROT;AccQ5QGS0]

TTAACATTATTGAGTCTTCTTATTCATGAGCATGGAATGTTTTTCTTATTTGTTTGTGTCATCTTTCATTTCTTTCAGCA

GTATTTTGTCATTTTTTTGT

In100 candidate3299 (in exon):

LOCUS ENSG00000050030

DESCRIPTION Uncharacterized protein KIAA2022.

[Source:Uniprot/SWISSPROT;AccQ5QGS0]

AGAGTCCCATTTAATCTTTACTTTCCTGGAAATGGTAACCTCCCTTATTCTTCTTACTTAATCTCAGACCTTGGTCTTAG

AATTTTTCTTTTATGGTCTC

In100 candidate3300 (in intron):

LOCUS ENSG00000182150

DESCRIPTION RAD26L hypothetical protein

[Source:RefSeq_peptide;Acc:NP_00101895]

ATTATTGATATTTTTTATTTAACTTTTATCTTTTTATCTCCTATTTCTAGTTCCACTGTTTCTCTTATTCAGACATTTCT

TTAGGATTCTGTATTTGTTT

In100 candidate3301 (in intron):

LOCUS ENSG00000172731

DESCRIPTION Leucine-rich repeat-containing protein 20.

[Source:Uniprot/SWISPROT;Acc:Q8TCA0]

GGGAGTTGAGGGTGTTTGCAAGTCCTTATGTCTTAACACCTTATCATTTTGTTTTCCTCACCTTTCTTTCAGTCTCTTTT

CCTCTAGTGATCCAATTTCT

In100 candidate3302 (in intron):

LOCUS ENSG00000128699

DESCRIPTION ORM1-like protein 1 (Adoplin-1).

[Source:Uniprot/SWISSPROT;Acc:9P0S3]

AATAATCTACCTGGATAGCATGTAACCTGTATCTGGTATCTAATTTCTTTCTCCTATCTGATGTTCTGGGCCTTCCAGAA

AATAGTTCTTACCTCCTTTT

In100 candidate3303 (in intron):

LOCUS ENSG00000130956

DESCRIPTION Intracellular hyaluronan-binding protein 4 (IHABP4) (Ki-1/57 inracellular antigen). [Source:Uniprot/SWISSPROT;Acc:Q5JVS0]

TGTTATAATTTTCAACTTGTTAGGTAATTAACTTTCTTAGTGGCTTTTTCTGCCTTACATTTTGAATGCCTTTTCCAGTT

CTGAACTCTTTTTTTTTTTT

In100 candidate3304 (in intron):

LOCUS ENSG00000102383

DESCRIPTION Palmitoyltransferase ZDHHC15 (EC 2.3.1.-) (Zinc finger DHHC domin- containing protein 15) (DHHC-15).

[Source:Uniprot/SWISSPROT;Acc:Q96MV8]EXON

TATGATACATTTGTTACAATTAATGAACTAATACTGATACATTATTTTTAACTGAAGTCTATATTTAGATTTCCTCAGTT

TTTCCATAATGTCCTTTATC

In100 candidate3305 (in intron):

LOCUS ENSG00000166224

DESCRIPTION Sphingosine-1-phosphate lyase 1 (EC 4.1.2.27) (SP-lyase) (hSPL)(Sphingosine-1-phosphate aldolase). [Source:Uniprot/SWISSPROT;Acc:O95470]

AGTGCTATTAACTACACTACAAATTCTATTTCTAGTTCATCAGGGTTTTTTTTTGTTTTGCTATGTTTTGTTTTAAACTA

ATGTTCTTTTTTCTATTCTA

In100 candidate3306 (in intron):

LOCUS ENSG00000085224

DESCRIPTION Transcriptional regulator ATRX (EC 3.6.1.-) (ATP-dependent

heliase ATRX) (X-linked helicase II) (X-linked nuclear protein) (XNP) (Znf- HX)

[Source:Uniprot/SWISSPROT;Acc:P46100]

TTTTTAAATTTTCTTTTAACTTTTACAAAAACCAACATTTTGTTTTATTGATATTTTGTATTATTTTCTTTATTTCAGTT

TCGTTTGCTTCACTCTGATC

In100 candidate3307 (in intron):

LOCUS ENSG00000085224

DESCRIPTION Transcriptional regulator ATRX (EC 3.6.1.-) (ATP-dependent

heliase ATRX) (X-linked helicase II) (X-linked nuclear protein) (XNP) (Znf- HX)

[Source:Uniprot/SWISSPROT;Acc:P46100]

AGATTAGTAGTAATGTCCTCTTTTTCATTTCTGATTCTAGTAACTTGAGTCTTGTTTCTTTTTTTTCTTGGTCAGTCTAC

CCTAAAGTTTGTTCATCTTT

In100 candidate3308 (in intron):

LOCUS ENSG00000085224

DESCRIPTION Transcriptional regulator ATRX (EC 3.6.1.-) (ATP-dependent

heliase ATRX) (X-linked helicase II) (X-linked nuclear protein) (XNP) (Znf- HX)

[Source:Uniprot/SWISSPROT;Acc:P46100]

AACATGTTTAATATATGAATAACAATTTTAACTTTTTCATTGACTCAGGGTTATTAATGAGTGCATTTACTTTTATTTTT

ATTTTATTTAATTTTTTTTT

In100 candidate3309 (in intron):

LOCUS ENSG00000196312

DESCRIPTION Hippocampus abundant transcript-like protein 2.

[Source:UniprotSWISSPROT;Acc:Q5VZR4]

AGGCGTGAGCCACCGTGCCTGGCCTCTAACATTTAATAGTACTAATTTTTCCTCTGTCCCTTTCTTTTTCTCCTTCAGGA

TGTTTTTATATTCTTGTCCA

In100 candidate3310 (in intron):

LOCUS ENSG00000138378

DESCRIPTION Signal transducer and activator of transcription 4.

[Source:Unirot/SWISSPROT;Acc:Q14765]

CTCTTGCCCTGCTCACACCTCACTAGCTACCCACTGTAAGATAATTAACAAATTATCATCTTGACTCTATTTCAGAACTT

GGTTTTCTTTAATAATCCTC

In100 candidate3311 (in intron):

LOCUS ENSG00000144339

DESCRIPTION Transmembrane protein with EGF-like and two follistatin-like

doains 2 precursor (Transmembrane protein containing epidermal growth factor nd

follistatin domains) (Tomoregulin) (TR) (Hyperplastic polyposis protein).

[Source:Uniprot/SWISSPROT;Acc:Q9UIK5]

AGAAATTAACTGATGATCATTTTTGAGTTTTTCCTTTTATTTTTTATCCATTGCTCAGTTGTTTGAATTATTTTCCATTA

GTTCTGTGTTTCTTTTCTTC

In100 candidate3312 (in intron):

LOCUS ENSG00000196950

DESCRIPTION solute carrier family 39 (zinc transporter), member 10

[Source:efSeq_peptide;Acc:NP_065075]

AGTCATAACATGATATAGCCATCCGTTTATTTAGGTCTTTTATTTCTCTCAGTAATTTTCTGTCAATAATATTTCTCTCA

ATAATTTCTCTCTTTTCTAT

In100 candidate3313 (in intron):

LOCUS ENSG00000196950

DESCRIPTION solute carrier family 39 (zinc transporter), member 10

[Source:efSeq_peptide;Acc:NP_065075]

CTAGTTTGATATTAGTTTAACCACCCCTGCTCTCTTTTGGTTACTATTTTCATGGAATATCGTTTTCCATCTTTTCAGTT

TCAGCCTGTTTGTGTCTTTG

In100 candidate3314 (in intron):

LOCUS ENSG00000118997

DESCRIPTION axonemal dynein heavy chain 7

[Source:RefSeq_peptide;Acc:NP_06120]

ATGATCTGATAACCCTACCTACTCCATTTATTTTTCTTTATAATTCTTCCACTTTGAATCTTTCTCTCAGGGCCTTCCTC

ATTCTCTTTTCCATCTTTTT

In100 candidate3315 (in intron):

LOCUS ENSG00000118997

DESCRIPTION axonemal dynein heavy chain 7

[Source:RefSeq_peptide;Acc:NP_06120]

GTTCTACCTATCTCTCTCTACCTCTTCTTTAAGGCCAATAACTCTTAGATTTGCCCTTTTGAGGCTATTTTCCAGATCCT

ATAGGTGTACTTCATTCTTT

In100 candidate3316 (in intron):

LOCUS ENSG00000118997

DESCRIPTION axonemal dynein heavy chain 7

[Source:RefSeq_peptide;Acc:NP_06120]

TACATATAATATTGTGGAACTTTATATAATCATATTTCTCCTCATCTAACTATATAGAAATCCCTTTTTGTGATCCAGAT

TTGAATGTCTTTCTCTTGTT

In100 candidate3317 (in intron):

LOCUS ENSG00000118997

DESCRIPTION axonemal dynein heavy chain 7

[Source:RefSeq_peptide;Acc:NP_06120]

CCATCTGGGATATATAGCAATTGTTGCTTTCTCTCAATTTTTCTTTAACACAAGGGCTAATATTTTTCCAATTCCCAGTT

AATTTTTTCTATTCCTTTGT

In100 candidate3318 (in intron):

LOCUS ENSG00000138411

DESCRIPTION E3 ubiquitin-protein ligase HECW2 (EC 6.3.2.-) (HECT, C2 and WWdomain-containing protein 2) (NEDD4-like ubiquitin-protein ligase 2). [Soure:Uniprot/SWISSPROT;Acc:Q9P2P5]

GGAACTTTTTCAAAACATGAGTTTTAACCACTGCTTATTTTTCTTAATGTTTATTTTTCATGTCATTCGGTTTTCCAGTG TTGGATTTGCCTTTCTCTAT

In100 candidate3319 (in intron):

LOCUS ENSG00000138411

DESCRIPTION E3 ubiquitin-protein ligase HECW2 (EC 6.3.2.-) (HECT, C2 and WWdomain-containing protein 2) (NEDD4-like ubiquitin-protein ligase 2). [Soure:Uniprot/SWISSPROT;Acc:Q9P2P5]

TCACACACTTTTCTCATGCAATACTAACCTTGCAATGCCCCTGGGTTTGCCTTTTCTGTCTTCCTTGTTCAGGAATATTT CCTCATAGCACTTTTTGTTT

In100 candidate3320 (in intron):

LOCUS ENSG00000144395

DESCRIPTION CDNA FLJ39660 fis, clone SMINT2006801. [Source:Uniprot/SPTREMBLAcc:Q8N8C6]

TGTATCCCTGTCATTACATAACTCATTACTGTATTTGTGTCATCCTCAATTTGTTTCATCAGCCTTTTAGAGTTTTTATT GTACAGATCTTTCACCTCCT

In100 candidate3321 (in intron):

LOCUS ENSG00000138303

DESCRIPTION Activating signal cointegrator 1 complex subunit 1 (ASC-1 complx subunit p50) (Trip4 complex subunit p50). [Source:Uniprot/SWISSPROT;Acc:QN9N2]

GAGAGAGGAGTGTTGAAATCTCCACCTGTAACTGTGGATTCATCTATTTCTCTTTTCAGTTCTACCAGTTTTTTCCACAC

CTTCTTGATAAGATCCTTTT

In100 candidate3322 (in intron):

LOCUS ENSG00000065413

DESCRIPTION Ankyrin repeat domain-containing protein 44.

[Source:Uniprot/SWSSPROT;Acc:Q8N8A2]

TTAAACTAAATAAATAGAAAGCTAACGGGTTTCTAATTCCCTTTTGAATTTCTTTTTTTCAGAAATGATTTATCCATTCC

ATGTTTTATTTCACATTCTT

In100 candidate3323 (in intron):

LOCUS ENSG00000078589

DESCRIPTION Putative P2Y purinoceptor 10 (P2Y10) (P2Y-like receptor).

[Soure:Uniprot/SWISSPROT;Acc:O00398]

TATGATGGAGTTTTAGTCATAACTTTGTGCGTGCCATTTTTATTATCAGGAACATCTTTAGAGCCATACTTTGATTTTTG

TTTTTTTGCCCATATTTTCT

In100 candidate3324 (in intron):

LOCUS ENSG00000204234

DESCRIPTION

AGCTTTTCATTAACTGAGCTTAGAGTTGCCTCCTTTTATTGCTTTTTACATGTTTACAGTATCTCATTCTTTCCTTGATT

CTGGATTTTCACACCATATG

In100 candidate3325 (in intron):

LOCUS ENSG00000166321

DESCRIPTION Nucleoside diphosphate-linked moiety X motif 13 (EC 3.-.-.-)

(Ndix motif 13) (Protein KiSS-16). [Source:Uniprot/SWISSPROT;Acc:Q86X67]

AAGGAAAAATAGGTATTATTCATAATTTATTCTCAATGTTAACCACTCTTAACATTTTAGTATTTGTACTTCCAGGATTT

ATTTAATGTATCTTATCTTT

In100 candidate3326 (in intron):

LOCUS ENSG00000163535

DESCRIPTION Shugoshin-like 2 (Tripin). [Source:Uniprot/SWISSPROT;Acc:Q562F6

AGTTTTATTCCCCCTAACCTCCTAATATATAGCTTTTCTTTTATTTGTTTGTTTGTTTGTTTTGTCCAGGTTTTGGCTTT

TATTTTTTCTTACCTTGTTG

In100 candidate3327 (in intron):

LOCUS ENSG00000138286

DESCRIPTION

CTACTTTTTTGATATAAGCATTTATTGCTATAACTTTCCTCTTAATACTGCTTTTGCTGTATCCTACAGGTTTTAATAGA

TTGTATTTTTATTTTCATTT

In100 candidate3328 (in intron):

LOCUS ENSG00000136928

DESCRIPTION Gamma-aminobutyric acid type B receptor, subunit 2 precursor

(GBA-B receptor 2) (GABA-B-R2) (Gb2) (GABABR2) (G-protein coupled receptor 51

(HG20). [Source:Uniprot/SWISSPROT;Acc:O75899]

AAGTACTCCATGGCTTTATAAATAACCCACTTATATCTCCTTTTGGTTACAGTAATGGTTTGATTTTTAAATATTTAACT

CTTTTTTTTATTGTTTTCTT

In100 candidate3329 (in intron):

LOCUS ENSG00000136928

DESCRIPTION Gamma-aminobutyric acid type B receptor, subunit 2 precursor (GBA-B receptor 2) (GABA-B-R2) (Gb2) (GABABR2) (G-protein coupled receptor 51 (HG20). [Source:Uniprot/SWISSPROT;Acc:O75899]

CATTTCTTAGGAATCCTAGGACAACCTTGGGGTAACTGTATTGTGATTATGTATTTTGTTCTGGGTCTTTCCTTCCAGAT
ATTTTCATCTCTTTGTTCCC

In100 candidate3330 (in intron):

LOCUS ENSG00000156042

DESCRIPTION tetratricopeptide repeat domain 18 [Source:RefSeq_peptide;Acc:N_660153]

TAACAATTATCAACATTCTGCCAAAGGCCTTTTGACTCCCCTGCTTTTGTTTTGACTTTATTCTATTTTTCCCGTCAGAA
CACCCAGTTGTCTTTTTATT

In100 candidate3331 (in intron):

LOCUS ENSG00000183035

DESCRIPTION Cylicin-1 (Cylicin I) (Multiple-band polypeptide I). [Source:Unprot/SWISSPROT;Acc:P35663]

CTTAATTCCTAGTAATTTTACTATTAGGTGATCAATCTCATATCTAACATTTTAATTATTTGACTTTCTCTTCTCCAGTT
ATTTTGTCTTACATCCTACT

In100 candidate3332 (in intron):

LOCUS ENSG00000072133

DESCRIPTION Ribosomal protein S6 kinase alpha-6 (EC 2.7.11.1) (S6K-alpha 6)(90 kDa ribosomal protein S6 kinase 6) (p90-RSK 6) (Ribosomal S6 kinase 4) RSK-4) (pp90RSK4). [Source:Uniprot/SWISSPROT;Acc:Q9UK32]

AGATGATGGGATTTTCATTGATGGGAGACTTTTTGTAACTGATTTAATCCTGTTACTTTCTATTGGTCTGTTCAGATTTT
CTATTTTGTCCATTCAATTT

In100 candidate3333 (in intron):

LOCUS ENSG00000107758

DESCRIPTION Serine/threonine-protein phosphatase 2B catalytic subunit beta soform (EC 3.1.3.16) (Calmodulin-dependent calcineurin A subunit beta isofom) (CAM-PRP catalytic subunit). [Source:Uniprot/SWISSPROT;Acc:P16298]

TCTAACTTGTCTCTAGATTCTATTACCTTTCTGCTTTTTCAGGAAACTTGCTTTATCGTTTATGTCCTCTATATCTTCAC
ATTCTCAGTACTAGTTCTTT

In100 candidate3334 (in intron):

LOCUS ENSG00000136874

DESCRIPTION Syntaxin-17 (KAT01814). [Source:Uniprot/SWISSPROT;Acc:P56962]

AGACCTTAAGGTTCATAGATTTAACATTAACATGTTTGTTGTTTTTGTTTTGCCTTTCAGATTATTTATCTCCTGGTCAT
CAAAGATTTCCTTTTTTTAT

In100 candidate3335 (in intron):

LOCUS ENSG00000136874

DESCRIPTION Syntaxin-17 (KAT01814). [Source:Uniprot/SWISSPROT;Acc:P56962]

AACTATATCTTCTGTATTTCTTAACTTTTCTTCTATATTTTTTACTTTCCTTGTTTCTTTTTTACATAGTTTCTTCAGAT

CTGTTTTCCAATTTATATCT

In100 candidate3336 (in intron):

LOCUS ENSG00000136874

DESCRIPTION Syntaxin-17 (KAT01814). [Source:Uniprot/SWISSPROT;Acc:P56962]

AGATTTTTAAACCTATCCTAGCTTTGTTTTATAACTACTGCCCTTGTTTTCTTTTTTTGTTTCAGTGTTTCTCTTTTGTT

AGTAACTTGTCTCATCTTGT

In100 candidate3337 (in intron):

LOCUS ENSG00000188419

DESCRIPTION Rab proteins geranylgeranyltransferase component A 1 (Rab escor

protein 1) (REP-1) (Choroideraemia protein) (TCD protein).

[Source:UniprotSWISSPROT;Acc:P24386]

TTTTGATTTCAATAAGGGTTTTAACTTAAAAACTGCATTTAGCTATTTAAAACTTTCTCCTATTTCCCTCTACTCCAGAA

ATTCTTATTTTGTTTACTTG

In100 candidate3338 (in intron):

LOCUS ENSG00000188419

DESCRIPTION Rab proteins geranylgeranyltransferase component A 1 (Rab escor

protein 1) (REP-1) (Choroideraemia protein) (TCD protein).

[Source:UniprotSWISSPROT;Acc:P24386]

TCTTTTATTCCTCATAACATCTCTTTCCCATTTCTTGTCTTTTTCTTTTCGATGCTGTATTCTGAGTAATTTCCTCAGCT

TTATCTTCTGGTTTTGAGTT

In100 candidate3339 (in intron):

LOCUS ENSG00000023318

DESCRIPTION Thioredoxin domain-containing protein 4 precursor (Endoplasmic

eticulum resident protein ERp44). [Source:Uniprot/SWISSPROT;Acc:Q9BS26]

GTAGGGAAGAACTTCCTAACTGGGTGGCTGATATTATTGATCTCTTCAGCCTTGCCCCATTTGCCTCTGTTGGGAGTAGC

CTTTGTTGTTTTGATTCTCT

In100 candidate3340 (in intron):

LOCUS ENSG00000023318

DESCRIPTION Thioredoxin domain-containing protein 4 precursor (Endoplasmic

eticulum resident protein ERp44). [Source:Uniprot/SWISSPROT;Acc:Q9BS26]

GAGCATTTCCTACTGTTAACTACCTCATTCTTCCCTGTTAGGCTATTTTCTCTGGTTCTTTTATGCTCCAGTAGTTCCAT

CTGTCTGTCCGTCTGTCTTT

In100 candidate3341 (in intron):

LOCUS ENSG00000023318

DESCRIPTION Thioredoxin domain-containing protein 4 precursor (Endoplasmic

eticulum resident protein ERp44). [Source:Uniprot/SWISSPROT;Acc:Q9BS26]

GTAGTAATAACATTTTCACAATATTGATTCTTCCAATCCATAAACATAGAATTTCTTTCCTTTTTTTGTCCCTTTCAGTT

TCTTCCATCGATGTTTTATA

In100 candidate3342 (in intron):

LOCUS ENSG00000119509

DESCRIPTION Inversin (Inversion of embryo turning homolog) (Nephrocystin-2)

[Source:Uniprot/SWISSPROT;Acc:Q9Y283]

AACATATTAGACTATCCATTTTATAACCCCTGCTCACCTCTCTGATCTTACTTTTCACTTCCTCTTACAGTGCATTTCTT

TTCTTTTTTCTTTTTTTTTA

In100 candidate3343 (in intron):

LOCUS ENSG00000126733

DESCRIPTION Dachshund homolog 2 (Dach2). [Source:Uniprot/SWISSPROT;Acc:Q96N9]

GTGAATTACTGATAGATGGCTGCTGAAATGTACAACTATAACAATGGGTTCATTTTTTTACCCTTGAAATTTTATCAGTT

TTGCCTCATGTATCTTGTGC

In100 candidate3344 (in intron):

LOCUS ENSG00000126733

DESCRIPTION Dachshund homolog 2 (Dach2). [Source:Uniprot/SWISSPROT;Acc:Q96N9]

AAGTATTCTCATTAATACTAGAAATTTTCCTCTAACCATTGTTTTACTTTGTTCCCTTTTCATTTACAATGATTTCAGAA

CTAACTTTCTATTTGACTTC

In100 candidate3345 (in intron):

LOCUS ENSG00000126733

DESCRIPTION Dachshund homolog 2 (Dach2). [Source:Uniprot/SWISSPROT;Acc:Q96N9]

AGATTTCTCTTTCATGTGATTGAGCTTCTCTAAAATTAACATTTTGAATATTTTATCTGTCATTCAGTAATTTCATTTTG

ATTCATTTCTTTTCCTGAAC

In100 candidate3346 (in intron):

LOCUS ENSG00000082146

DESCRIPTION Pseudokinase ALS2CR2 (Amyotrophic lateral sclerosis 2 chromosoml region candidate gene 2 protein) (STRAD beta) (ILP-interacting protein) (ALS-21). [Source:Uniprot/SWISSPROT;Acc:Q9C0K7]

GAGTACAATGAATGCTCTAACTTTGCCTTCTGTAATACTATTCACTCCTACTTCTCTTCTATTTCTTTTTCTTTACAGTG
TCCTTTACTATTTCTTCCTC

In100 candidate3347 (in intron):

LOCUS ENSG00000155754

DESCRIPTION Amyotrophic lateral sclerosis 2 chromosomal region candidate gee 11 protein. [Source:Uniprot/SWISSPROT;Acc:Q53TS8]

TTCTTACACTATCTTCCATGTCTTTTAACCTATAAAAAATATTTTTCATCACTTTGTCTCTCTGCCCAGCATTCCAAGTA
ATTCTTCTGATCTTTATTTT

In100 candidate3348 (in intron):

LOCUS ENSG00000102290

DESCRIPTION Protocadherin-11 X-linked precursor (Protocadherin-11) (Protocaherin on the X chromosome) (PCDH-X) (Protocadherin-S). [Source:Uniprot/SWISPROT;Acc:Q9BZA7]

AACAATATTCATTCTTCCAATTCATTAACATGGAATGTCTTTCCATTTTTTTTGTCCTCTACAGTTTTTTCCAGCACTGT
TTTATAATGTTCATTGTCTT

In100 candidate3349 (in intron):

LOCUS ENSG00000102290

DESCRIPTION Protocadherin-11 X-linked precursor (Protocadherin-11)

(Protocaherin on the X chromosome) (PCDH-X) (Protocadherin-S).

[Source:Uniprot/SWISPROT;Acc:Q9BZA7]

AGTAAGTTTACAAGTAACTTAAATGTGAGACTTTTTACTTTATTCTTCTTTTCAAGATTGTTTTCACTATTGCAGGTCTC

TTGAAATTCCATATGTTTTT

In100 candidate3350 (in intron):

LOCUS ENSG00000102290

DESCRIPTION Protocadherin-11 X-linked precursor (Protocadherin-11)

(Protocaherin on the X chromosome) (PCDH-X) (Protocadherin-S).

[Source:Uniprot/SWISPROT;Acc:Q9BZA7]

GATGTATTTTAACCTAAATTTTATCTATCTATCTATCATCTATCTATCTATCTATTTATCTATCTTCCTATTTAACAGAT

TTTCTATTTGTACATTCATG

In100 candidate3351 (in intron):

LOCUS ENSG00000102290

DESCRIPTION Protocadherin-11 X-linked precursor (Protocadherin-11)

(Protocaherin on the X chromosome) (PCDH-X) (Protocadherin-S).

[Source:Uniprot/SWISPROT;Acc:Q9BZA7]

ATATTTTCTCCCATTTTGTGAGTTGTCTTTTAACTTTGTTTATTATTTTCTTCATCCTGCAGAAGCTTTTTAACTTTACG

TGATCCCATTTGTCCATTTT

In100 candidate3352 (in intron):

LOCUS ENSG00000102290

DESCRIPTION Protocadherin-11 X-linked precursor (Protocadherin-11)

(Protocaherin on the X chromosome) (PCDH-X) (Protocadherin-S).

[Source:Uniprot/SWISPROT;Acc:Q9BZA7]

TTTGTCTTGATACCTATCTTGTCTTATATAACTGTTCTGCTCTTTTTTTTGATTTTCATTGGCAGGTATATTTTTGGCAG

ATATATTTTTCTATCCCCTT

In100 candidate3353 (in exon):

LOCUS ENSG00000102290

DESCRIPTION Protocadherin-11 X-linked precursor (Protocadherin-11)

(Protocaherin on the X chromosome) (PCDH-X) (Protocadherin-S).

[Source:Uniprot/SWISPROT;Acc:Q9BZA7]

ATAACGTTAAAAGGTATACGTACTCTAGCCTTTTTTTGGGCTTTCTTTTTGATTTTTGTTTGTTGTTTTCAGTTTTTTTG

TTGTTGTTAGTGAGTCTCCC

In100 candidate3354 (in intron):

LOCUS ENSG00000182329

DESCRIPTION

AGGGATATCTAGTCTTCTAACTTAGCCTGTTACTCCTTTCCATTTATTCAGGGAATTTTTTTTTTTTTTTTTTTTTTTTT

TTCCTGTACCAGGCCAAATT

In100 candidate3355 (in intron):

LOCUS ENSG00000116030

DESCRIPTION Small ubiquitin-related modifier 1 precursor (SUMO-1)

(Sentrin)(Ubiquitin-like protein SMT3C) (SMT3 homolog 3) (Ubiquitin-homology

domain rotein PIC1) (Ubiquitin-like protein UBL1) (GAP-modifying protein 1)

(GMP1) [Source:Uniprot/SWISSPROT;Acc:P63165]

GTGAAGTTGTTTTAACTAATTTATTCTGTTAACCTCATTTTCATTTCAGTATCTCTTTATTGGAAATTGTTTTGTAACTG

TTTTTGTGATCTTTTTTTTT

In100 candidate3356 (in intron):

LOCUS ENSG00000204217

DESCRIPTION Bone morphogenetic protein receptor type-2 precursor (EC

2.7.1130) (Bone morphogenetic protein receptor type II) (BMP type II receptor)

(BPR-II). [Source:Uniprot/SWISSPROT;Acc:Q13873]

CCTTTTGGCGGGGGTGGAATTTAACTTTAGACTGTTATTTCTCTCTCTTCTTCAGTTTTTCACTGTTTCTTTCATAATTC

ACTCATCGAATGCCTTGATT

In100 candidate3357 (in intron):

LOCUS ENSG00000148123

DESCRIPTION plasticity related gene 3

[Source:RefSeq_peptide;Acc:NP_060223]EXON

GAGTCTCTTTCTAGGGAAGTTGTCCTGACCTCTTAACTCTAAGGTTGGCTTCTCTTTCTATGTGTTTCAGTAGTCCCCAA

CTCTGTATTTTTTTGTGCCT

In100 candidate3358 (in intron):

LOCUS ENSG00000148123

DESCRIPTION plasticity related gene 3

[Source:RefSeq_peptide;Acc:NP_060223]EXON

TAACCCTAGACTCCATTTGTTCTACTCTGCAGCCTGAGCTTTCTCCCAGAGTCTGAGCTCTCTGCTTGCTGTTTAGGTGC

CTTTTCTGTTCTGTGCTTTT

In100 candidate3359 (in intron):

LOCUS ENSG00000138442

DESCRIPTION WD repeat protein 12 (YTM1 homolog).

[Source:Uniprot/SWISSPROT;cc:Q9GZL7]

TACCTGTCATTTGCTTGATATATTAACATACAATAGTCTTTTCCTCCTTAATTCTCAAAATTGCTTTTTTCCCTCCAGTA

CTTCTGTTCCACTTAGTTTG

In100 candidate3360 (in intron):

LOCUS ENSG00000147202

DESCRIPTION Protein diaphanous homolog 2 (Diaphanous-related formin-2) (DRF).

[Source:Uniprot/SWISSPROT;Acc:O60879]

ATCTTTTCAAAAAAAAAAAACCTAACTTTTTGTTTTGTTGCTCTTTTGTATTATATTTTTAATCTCTGTCTGGCTCAGTT

CTGCTCTGATCTTTATTATT

In100 candidate3361 (in intron):

LOCUS ENSG00000147202

DESCRIPTION Protein diaphanous homolog 2 (Diaphanous-related formin-2) (DRF).

[Source:Uniprot/SWISSPROT;Acc:O60879]

ATGCACCTTAACGTTTTATTCATTTTGCTATTCTTTCAGTGATGGTTTCCCTATGTCTGCGGTTTTTCGTTTTTTACTGT

GTGTGTATCCTTATATTTTT

In100 candidate3362 (in intron):

LOCUS ENSG00000147202

DESCRIPTION Protein diaphanous homolog 2 (Diaphanous-related formin-2) (DRF).

[Source:Uniprot/SWISSPROT;Acc:O60879]

TTATGCCTTTATTGCTCCCATAGCTGGTGTGACATGGAGAGTTATAACCACTGAGTTCACTCTTACGTTAATTCTCAGGG

ATGTTCTTCCTTCTATCTTA

In100 candidate3363 (in intron):

LOCUS ENSG00000147202

DESCRIPTION Protein diaphanous homolog 2 (Diaphanous-related formin-2) (DRF).

[Source:Uniprot/SWISSPROT;Acc:O60879]

TTTCCTTAACAATGAATGAACTCTCTGTTCTCCTTTCTATATCAGGGTATCTGCTTATTTATGGTTTCTACTGCCATGTG

GCTTTTAACATCCTCTCTCT

In100 candidate3364 (in intron):

LOCUS ENSG00000147202

DESCRIPTION Protein diaphanous homolog 2 (Diaphanous-related formin-2) (DRF).

[Source:Uniprot/SWISSPROT;Acc:O60879]

TATCTTAGATAGGCTGTATAGTAGAGACTTTACTGTAACGAGAAAGATCCTGAATTGTTACCCTACTTGTCTTCCCAGAT

GTTTTCTTTGTTAGGCTTTT

In100 candidate3365 (in intron):

LOCUS ENSG00000163597

DESCRIPTION Amyotrophic lateral sclerosis 2 chromosomal region candidate gee 16 protein. [Source:Uniprot/SWISSPROT;Acc:Q6ZP36]

AAAGGAACACTGTGTGTTCCTTGAGTATGTATTTCCCATTCAAATTTGTAACTTAACATTTTGCCATATTTGCTTCAGTT
CTTTCTTTTCCCTGTGAAAT

In100 candidate3366 (in intron):

LOCUS ENSG00000144426

DESCRIPTION Neurobeachin-like 1 (Amyotrophic lateral sclerosis 2 chromosoma region candidate gene 17 protein). [Source:Uniprot/SWISSPROT;Acc:Q6ZS30]

AATTAACTGCAAAATGTTTTGCCTACTCCTTAGTTTTGTCTACTCCTTAGTTTTGTCTATATTTCCGTTAACTGCCAGAA
TTCATGGTTTTCTTTTTTTA

In100 candidate3367 (in intron):

LOCUS ENSG00000165152

DESCRIPTION Uncharacterized protein C9orf125.
[Source:Uniprot/SWISSPROT;AccQ9BRR3]

AGAAGTTGCACTAAAATATAACTATTGTTGTAGCTCCATTTATTCATCTAAGCCCTAGTTCATGTTCATTTCTCAGAGTC
TGTGTCCTTTACTGTCTTCC

In100 candidate3368 (in intron):

LOCUS ENSG00000156110

DESCRIPTION Adenosine kinase (EC 2.7.1.20) (AK) (Adenosine 5'-phosphotransfrase). [Source:Uniprot/SWISSPROT;Acc:P55263]

AAGAAAAATCCTGCATCACTGATATGAAATGTACCAAATTAAGTAACATCATCTTATTTCTTCTGATTTTGTTTTCAGTT
TGTTTATATTCCTTTCCATA

In100 candidate3369 (in intron):

LOCUS ENSG00000156110

DESCRIPTION Adenosine kinase (EC 2.7.1.20) (AK) (Adenosine 5'-

phosphotransfrase). [Source:Uniprot/SWISSPROT;Acc:P55263]

AGTGTTGACCTTACCATAACTCTGAAACTTTTCCTGTCTTGCTTAGCTTATGTGATACCTTTTCCCTCAGGTTTTTCTTC

TTTTCTGGCTCCTCTTCTTC

In100 candidate3370 (in intron):

LOCUS ENSG00000156110

DESCRIPTION Adenosine kinase (EC 2.7.1.20) (AK) (Adenosine 5'-

phosphotransfrase). [Source:Uniprot/SWISSPROT;Acc:P55263]

AGCACTTAACAAGGGTGCTGATCTCTCCCCATCTGAGGTCTTCTTTCTTCTTTTCATCTAATTTTAAGCACTGATCAGTG

AATTCTTTCTCATTATTTTA

In100 candidate3371 (in intron):

LOCUS ENSG00000155827

DESCRIPTION E3 ubiquitin-protein ligase BRE1A (EC 6.3.2.-) (BRE1-A)

(hBRE1)(RING finger protein 20). [Source:Uniprot/SWISSPROT;Acc:Q5VTR2]

AAGTAGACTACAAAAGTTTCCCCATTCTCATTTCTCAAAAGATAACCATTCTAAACAATGTGTTTTATGTCCTCTCAGAC

TTTTGTACTTTTGTATTAAT

In100 candidate3372 (in intron):

LOCUS ENSG00000156650

DESCRIPTION Histone acetyltransferase MYST4 (EC 2.3.1.48) (EC 2.3.1.-) (MYS protein 4) (MOZ, YBF2/SAS3, SAS2 and TIP60 protein 4) (Histone acetyltranserase MOZ2) (Monocytic leukemia zinc finger protein- related factor) (Histoe acetyltransferase MORF). [Source:Uniprot/SWISSPROT;Acc:Q8WYB5]

AGATTTGGAGATGATACATTATCACTGCAAATTTGACTATAAAGATAACTACTGTTGAAAGTGTGTATTTTCTTCCAGTC
TTTTTTCCTTTTTTCCTACA

In100 candidate3373 (in intron):

LOCUS ENSG00000165194

DESCRIPTION Protocadherin-19 precursor. [Source:Uniprot/SWISSPROT;Acc:Q8TAB]

TGTGTGCATTGGTAACACTGTTAACTGCTCTGTTCCCTTTCTTTTCCAGTAGAATTTCTTTTGTTTGGGATGACTGTTAC
TTTTCCTTTTGGTCCTCTTT

In100 candidate3374 (in intron):

LOCUS ENSG00000198785

DESCRIPTION Glutamate [NMDA] receptor subunit 3A precursor (N-methyl-Daspatate receptor subtype NR3A) (NMDAR-L). [Source:Uniprot/SWISSPROT;Acc:Q8TCU5

TCTTGAAAAAGTGTTTTTCTCTTACCTATAACAAGACTTTTCATAACATCTTTGACTTCTCCCTTTTCTTGTTACCAGGT
TCTGTTGCTTTCCTTCATAT

In100 candidate3375 (in intron):

LOCUS ENSG00000116117

DESCRIPTION Partitioning-defective 3 homolog B (PAR3-beta) (Partitioningdeective 3-like protein) (PAR3-L protein) (Amyotrophic lateral sclerosis 2

chomosome region candidate gene 19 protein).

[Source:Uniprot/SWISSPROT;Acc:Q8EW8]

AAAAATTAAGAGGTATTAGTAAGAAATGTGTGGATTCTTCCTTCTCTTAACATTACTGTAGATCCTGTTTTATTTCAGCT

GTTTGTTCCCTTATCCTTAC

In100 candidate3376 (in intron):

LOCUS ENSG00000116117

DESCRIPTION Partitioning-defective 3 homolog B (PAR3-beta) (Partitioningdeective

3-like protein) (PAR3-L protein) (Amyotrophic lateral sclerosis 2

chomosome region candidate gene 19 protein).

[Source:Uniprot/SWISSPROT;Acc:Q8EW8]

AGTCAATAATTATTTGCTGAATGAATGCATAAACAAATCAAATTAACAAATTAAACTCTAGATTTTCATCTTCTTCAGAA

TTTTTTTTTTCAGTGTTCCA

In100 candidate3377 (in intron):

LOCUS ENSG00000116117

DESCRIPTION Partitioning-defective 3 homolog B (PAR3-beta) (Partitioningdeective

3-like protein) (PAR3-L protein) (Amyotrophic lateral sclerosis 2

chomosome region candidate gene 19 protein).

[Source:Uniprot/SWISSPROT;Acc:Q8EW8]

GCATACAACCGCATTAAAGAGATAGTGTTTTAACTGCACATATAACTTAACTATTTCTGTATTTATCTTCAGTTTTCAGT

GTTTAAACCTGCCTGTTTCT

In100 candidate3378 (in intron):

LOCUS ENSG00000116117

DESCRIPTION Partitioning-defective 3 homolog B (PAR3-beta) (Partitioningdeective 3-like protein) (PAR3-L protein) (Amyotrophic lateral sclerosis 2 chomosome region candidate gene 19 protein). [Source:Uniprot/SWISSPROT;Acc:Q8EW8]

TAGCTATTTTGATCTTAGTAAAATTTTCCCACTTTTCTTCTATGTATAACCAAAATATTTTTCTTTTATCTCACACAGTT

TATTTTCTGTTTTTTGTTTT

In100 candidate3379 (in intron):

LOCUS ENSG00000116117

DESCRIPTION Partitioning-defective 3 homolog B (PAR3-beta) (Partitioningdeective 3-like protein) (PAR3-L protein) (Amyotrophic lateral sclerosis 2 chomosome region candidate gene 19 protein). [Source:Uniprot/SWISSPROT;Acc:Q8EW8]

GAAAACAAGATTAATTTAACGGAAAATTGCTTTCCACAATCTTTTCTGGAATCTCTTTTGTCTGTTACCCAGGCAATATC

TATATTATTACTTTGTCCTC

In100 candidate3380 (in intron):

LOCUS ENSG00000116117

DESCRIPTION Partitioning-defective 3 homolog B (PAR3-beta) (Partitioningdeective 3-like protein) (PAR3-L protein) (Amyotrophic lateral sclerosis 2 chomosome region candidate gene 19 protein). [Source:Uniprot/SWISSPROT;Acc:Q8EW8]

TTACTGTTTATATGGTAAGCATTTATTTTAACTGTTGCTTTTTCTCTTTGTTCCTTTCTGAATATCTGTGTTTCCCAGAA

TACTTTTCCTTTTTTCCTTA

In100 candidate3381 (in intron):

LOCUS ENSG00000116117

DESCRIPTION Partitioning-defective 3 homolog B (PAR3-beta) (Partitioningdeective

3-like protein) (PAR3-L protein) (Amyotrophic lateral sclerosis 2

chomosome region candidate gene 19 protein).

[Source:Uniprot/SWISSPROT;Acc:Q8EW8]

CTTTCTTTATTCTCCTGAAAATTACTTCTTTTATTTTATATTTAACAAGTTTTTATGTTTTTATTATTTTTCTTACAGTT

CTTTTTTTTTTTTTTTACTT

In100 candidate3382 (in intron):

LOCUS ENSG00000101811

DESCRIPTION Cleavage stimulation factor 64 kDa subunit (CSTF 64 kDa subunit

(CF-1 64 kDa subunit) (CstF-64). [Source:Uniprot/SWISSPROT;Acc:P33240]

TCAAGCCATTAGTAGTACTAACTGTGGCATTAGTCATTCCTGTCACTCTTTTCAGGAATTTTTGAAGTTAGCGTATTTTT

CTCTTTTAACCTTTTGTTTC

In100 candidate3383 (in intron):

LOCUS ENSG00000158296

DESCRIPTION Solute carrier family 13 member 3 (Sodium-dependent high-affiniy dicarboxylate transporter 2) (Na(+)/dicarboxylate cotransporter 3) (NaDC-) (hNaDC3). [Source:Uniprot/SWISSPROT;Acc:Q8WWT9]

ATAAAATAAAACATAAATTGATAATTCTATGTACGCTGTTTTGTAACCTGCTATTTCACATTTTTTTCTTCATTTCAGTC
TGTTTCCAAATACCTATTTT

In100 candidate3384 (in intron):

LOCUS ENSG00000165028

DESCRIPTION Protein NipSnap3B (SNAP1). [Source:Uniprot/SWISSPROT;Acc:Q9BS92

ACTGCTAAGCTACATTATTGTAGTTTAACTTTTGTGTCTTCCTCTTTCTTGTTTCTCAGTTTGAAAAGATTTGTCTTCTT
CCTGATTTCACTTCTTGCTC

In100 candidate3385 (in intron):

LOCUS ENSG00000114933

DESCRIPTION CDNA FLJ20309 fis, clone HEP07296.
[Source:Uniprot/SPTREMBL;AccQ9NXD5]

AGCATCCAAGTGCGTCTTTCCACACTTAACTTGAGAAGCACTGATTTAGTCCTTCCATTCTGCAGTAATAGCATCTTTTT
TATTTTTTCAGTTTTCCATA

In100 candidate3386 (in intron):

LOCUS ENSG00000114933

DESCRIPTION CDNA FLJ20309 fis, clone HEP07296.
[Source:Uniprot/SPTREMBL;AccQ9NXD5]

AACATTTATAGATTTTTAACTACAAAAGTAGTATTTTATGTTTATTTACATGTGTTATTTTAGTTTCTTTTCAGTGTTGG
TTTTCATTTTTTTGTTTTTA

In100 candidate3387 (in intron):

LOCUS ENSG00000114948

DESCRIPTION ADAM 23 precursor (A disintegrin and metalloproteinase domain 2) (Metalloproteinase-like, disintegrin-like, and cysteine-rich protein 3) (DC-3). [Source:Uniprot/SWISSPROT;Acc:O75077]

CTGAAGTTAGATTTAGAGAAGATTTAAGCTCTAACAAGAGAAGCCAAGGTTCATTCTTTCCAAATTTTCTCTCCACAGTG

TTTCTGTGATTTTTTTTTTA

In100 candidate3388 (in intron):

LOCUS ENSG00000102384

DESCRIPTION Centromere protein I (CENP-I) (Interphase centromere complex prtein 19) (Follicle-stimulating hormone primary response protein) (FSH primay response protein 1) (Leucine-rich primary response protein 1). [Source:Unprot/SWISSPROT;Acc:Q92674]

TGTCCTTTCCTCTTCCATCTTTTACCATCTTCTGCCTTGAGATAACCATTTCTATTGCTTTTTGTTTTTTCCATCCAGTG

TTTTCCTTTCTTTAAACTTA

In100 candidate3389 (in intron):

LOCUS ENSG00000102385

DESCRIPTION Dystrophin-related protein 2. [Source:Uniprot/SWISSPROT;Acc:Q1374]

GGCCCTGCTGGGATCAACTTTGTGAGGGAAATGATTGCCAAGATGTTAACCCTTTACATACATTCATATTCTCAGTCTCT

TCTTTTTAAACCTAGTGGTC

In100 candidate3390 (in intron):

LOCUS ENSG00000148655

DESCRIPTION Uncharacterized protein C10orf11.

[Source:Uniprot/SWISSPROT;AccQ9H2I8]

TGCTTTTTATCATCAACTTGACTTCAACATAACTGTCCTCCTAGTTATTTCTGTCTCGTTTTAATTGTTTTGTGCAGAA

CTTTAGTTTCTTTGTCTTTT

In100 candidate3391 (in intron):

LOCUS ENSG00000148655

DESCRIPTION Uncharacterized protein C10orf11.

[Source:Uniprot/SWISSPROT;AccQ9H2I8]

AGATAGAGACATATTATATCTAACTTAATTGGATTTGTTTATTTCTGTTTATTTATTTCTCCTTGCAGTCCTAGTACTTT

TTTGCCTCATGTATTTTGAT

In100 candidate3392 (in intron):

LOCUS ENSG00000156113

DESCRIPTION Calcium-activated potassium channel subunit alpha 1 (Calciumacivated potassium channel, subfamily M subunit alpha 1) (Maxi K channel) (MaiK) (BK channel) (K(VCA)alpha) (BKCA alpha) (KCa1.1) (Slowpoke homolog) (Sl homolog) (Slo-alpha) (Slo1) (hSlo). [Source:Uniprot/SWISSPROT;Acc:Q12791]

EXON

CCAAAGACAAATTCTTCCAAGTTGGGTTGGGGGAAAAAAATCACCATTCTAACTTTGCTTCTGACTTTCAATCTTCAGAT

TGCTTTTGCTTTCTAATCCT

In100 candidate3393 (in intron):

LOCUS ENSG00000124151

DESCRIPTION Nuclear receptor coactivator 3 (EC 2.3.1.48) (NCoA-3) (Thyroid ormone receptor activator molecule 1) (TRAM-1) (ACTR) (Receptor-associated oactivator 3) (RAC-3) (Amplified in breast cancer-1 protein) (AIB-1) (Sterod receptor coactivator protein 3) (SRC- [Source:Uniprot/SWISSPROT;Acc:Q9Y6Q]

TTCAAAAAGTATTTTCTTAACTGAGACCATTTGCAACTCTCTTTAAGTCAACATTTTCTTTTTTTTTTCTAGTATCAGAA

GATTTTATTTTTTTACTTTT

In100 candidate3394 (in intron):

LOCUS ENSG00000124151

DESCRIPTION Nuclear receptor coactivator 3 (EC 2.3.1.48) (NCoA-3) (Thyroid ormone receptor activator molecule 1) (TRAM-1) (ACTR) (Receptor-associated oactivator 3) (RAC-3) (Amplified in breast cancer-1 protein) (AIB-1) (Sterod receptor coactivator protein 3) (SRC- [Source:Uniprot/SWISSPROT;Acc:Q9Y6Q]

TGGTTTCTCACTGTTAACCAAGGTCTTTTCTTTACCTTTGGAAGCTTCTTTACTGCGTGTGTTCTCTGCTCCTGTCAGAT

TGATTTTTGTTTAGTCCTTG

In100 candidate3395 (in intron):

LOCUS ENSG00000124151

DESCRIPTION Nuclear receptor coactivator 3 (EC 2.3.1.48) (NCoA-3) (Thyroid ormone receptor activator molecule 1) (TRAM-1) (ACTR) (Receptor-associated oactivator 3) (RAC-3) (Amplified in breast cancer-1 protein) (AIB-1) (Sterod receptor coactivator protein 3) (SRC- [Source:Uniprot/SWISSPROT;Acc:Q9Y6Q]

TAAAGGTAGATAAGGTTTTTTTGAAAAAAATCCTTACTGCTAATAACCAAATACTTAAACCTTCTTGATTTCAGCATTT
TTAATGGAACTCTTCTTTCT

In100 candidate3396 (in intron):

LOCUS ENSG00000148143

DESCRIPTION Zinc finger protein 462. [Source:Uniprot/SWISSPROT;Acc:Q96JM2]

EXON

GCCTCTTATAACTTTAATATATTTATTACTTCTGCAATGATATTATTTTTGTCATCATTTTCTTTCTTCAACTGCCAGCC
TCTCTTTCATCTTGTGCTGA

In100 candidate3397 (in intron):

LOCUS ENSG00000144407

DESCRIPTION Parathyroid hormone receptor precursor (PTH2 receptor). [SourceUniprot/SWISSPROT;Acc:P49190]

TGATAATAAATGTTAAATTAGAAAACAATGTAGCAACTTGATTATTAACTAATTTTGATGTTTTTCTTTGTTTCCCAGTT
TCATATTCTTCTTGTTTTCT

In100 candidate3398 (in intron):

LOCUS ENSG00000078018

DESCRIPTION Microtubule-associated protein 2 (MAP 2) (MAP-2). [Source:Uniprt/SWISSPROT;Acc:P11137]

TGGGGATGGAGAAAGCTCCCAACTACCATTCATTGTCTAACCTCATACCCTGCTTTATCTTCTTCATAGCAGTCATCATT

CTCTTAAGTATTTGTGTTCC

In100 candidate3399 (in exon):

LOCUS ENSG00000126947

DESCRIPTION Armadillo repeat-containing X-linked protein 1 (Protein ALEX1)

ARM protein lost in epithelial cancers on chromosome X 1).

[Source:Uniprot/WISSPROT;Acc:Q9P291]

AGATGGCTGGGCTAAGACTGTTAACCAACATGACTGTGACTAATCATTACCAACATTTGCTTTCCTATTCTTTTCCAGAC

TTTTTTGCTTTGTTATTCCT

In100 candidate3400 (in intron):

LOCUS ENSG00000144406

DESCRIPTION CDNA FLJ14677 fis, clone NT2RP2004095.

[Source:Uniprot/SPTREMBLAcc:Q96SS0]

TAGTTAGCAATTCCTCTAACCTTTTATCAAGGTTCTTAGCTTCTTTTCATTCCATTATAACATGCTCCTTTAGCTCAGTG

TGGTTTTCTATTACCCATCT

In100 candidate3401 (in intron):

LOCUS ENSG00000144406

DESCRIPTION CDNA FLJ14677 fis, clone NT2RP2004095.

[Source:Uniprot/SPTREMBLAcc:Q96SS0]

AGTGGTGTCAAATTTTGTTTAGATGTTCACCTGTAACTGTGCCTCTGATACTCTGTGGTTCTTTTCAGCTATGCGGCATT

CTTCTGATTCATTTTTCTCC

In100 candidate3402 (in intron):

LOCUS ENSG00000197713

DESCRIPTION Ribulose-phosphate 3-epimerase (EC 5.1.3.1) (Ribulose-5-phosphae-

3- epimerase). [Source:Uniprot/SWISSPROT;Acc:Q96AT9]

ACTAGCCAAAGTTGGTCAATACCACTCTCCACGTCTTTGACTGTAACTTGCTTATTTCTGCTTTTGCTTCATTTTCAGCC

TTTGTTTCTTTTCCCATCTT

In100 candidate3403 (in intron):

LOCUS ENSG00000144445

DESCRIPTION CDNA FLJ32349 fis, clone PROST2007237.

[Source:Uniprot/SPTREMBLAcc:Q96MI0]

TAGTGTTACAATAATACTAGCTGTTATAATTATGCATGTATTTAACTTCATTGAGATTTTTCTTTCATCATACAGCTTTA

AGTTACTATCTACGTCTTTC

In100 candidate3404 (in intron):

LOCUS ENSG00000115365

DESCRIPTION LanC-like protein 1 (40 kDa erythrocyte membrane protein) (p40)

[Source:Uniprot/SWISSPROT;Acc:O43813]

AGTAGACACCATGCCAAAAATAATAAAATAACTGCCTTTCCTCATCTTTACATTGTTTCCTTTTTTTTTTTTCAGGTATT

GCTGTGCTTTACTTACATCT

In100 candidate3405 (in intron):

LOCUS ENSG00000115365

DESCRIPTION LanC-like protein 1 (40 kDa erythrocyte membrane protein) (p40)

[Source:Uniprot/SWISSPROT;Acc:O43813]

AGAACAAAGATTTAGTGTCGAAAGCTACCTGATAACATTTGTTAGGGCTAATGTCGTCTCATTACTACTTTCTTCCAGAG

TTTCATTGGCTCTTTGTGCT

In100 candidate3406 (in intron):

LOCUS ENSG00000021826

DESCRIPTION Carbamoyl-phosphate synthase [ammonia], mitochondrial

precursor(EC 6.3.4.16) (Carbamoyl-phosphate synthetase I) (CPSase I).

[Source:Unipro/SWISSPROT;Acc:P31327]

GATTCACAATGCCTACACCTTCTATTTTCTAACAAACTCTTGAATTTAATTGCGCCTTTTCCTCTTTCCCAGTGTCTTTA

AGTTCACAATCCTTTCACTT

In100 candidate3407 (in intron):

LOCUS ENSG00000178568

DESCRIPTION Receptor tyrosine-protein kinase erbB-4 precursor (EC

2.7.10.1)(p180erbB4) (Tyrosine kinase-type cell surface receptor HER4).

[Source:Unipot/SWISSPROT;Acc:Q15303]

TTCCTTTGTTCATGCTTTTCTTCCTTCTTTGAGATTAACGAAAATTTTTTTTTCTATTTCTCTATAGGCTTTTTCAGGA

TCTCTTTTTGTATTATTTTT

In100 candidate3408 (in intron):

LOCUS ENSG00000178568

DESCRIPTION Receptor tyrosine-protein kinase erbB-4 precursor (EC 2.7.10.1)(p180erbB4) (Tyrosine kinase-type cell surface receptor HER4). [Source:Unipot/SWISSPROT;Acc:Q15303]

AACTGTTTCTCATTATTAGCTGTAAGTAATAACACTAATCCCTTTCTTTTCTTTTCAGTCTTCTTTAATGAATATGTGTT

TAACTTCACTCTCATCTTCC

In100 candidate3409 (in intron):

LOCUS ENSG00000178568

DESCRIPTION Receptor tyrosine-protein kinase erbB-4 precursor (EC 2.7.10.1)(p180erbB4) (Tyrosine kinase-type cell surface receptor HER4). [Source:Unipot/SWISSPROT;Acc:Q15303]

TTTCTTTTCTTTTTTAAAACTCCTTTTCTCTCTGAGAAAAAATAACTGAACTTCCTTTCCCTCTTATGATATTTTCAGTT

CCATATAAATATTTCTTCCT

In100 candidate3410 (in intron):

LOCUS ENSG00000165424

DESCRIPTION Zinc finger CCHC domain-containing protein C10orf56. [Source:Unprot/SWISSPROT;Acc:Q8N2G6]

ACCACTTTTTGTTCTACATTAATAAATAACAATGGCATTAATATTCCTGTTTTGTCTTCTGTGTGCCAGGCACTGTTCTA

AATGTTTTGCATTTACTCCT

In100 candidate3411 (in intron):

LOCUS ENSG00000144451

DESCRIPTION Sperm-associated antigen 16 protein (PF20 protein homolog). [Sorce:Uniprot/SWISSPROT;Acc:Q8N0X2]

AATTCAACTATTCTGACAATATATTAATACTAACCCCAAATCTTCTGGTCATTCTTCAGCAAGTGTCCAGTTTGTAGGAA

TTTTTTTTTTTTTTTTTTTT

In100 candidate3412 (in intron):

LOCUS ENSG00000144451

DESCRIPTION Sperm-associated antigen 16 protein (PF20 protein homolog).

[Sorce:Uniprot/SWISSPROT;Acc:Q8N0X2]

AACCTACCCTTAAGGGTAACTGTTGCACTATCTTTAGTCTTTCTGATTCCAGGTCTCTTCCCATTTTTCTCTGACTTGTG

TGAAATATATACACTTTCCT

In100 candidate3413 (in intron):

LOCUS ENSG00000144451

DESCRIPTION Sperm-associated antigen 16 protein (PF20 protein homolog).

[Sorce:Uniprot/SWISSPROT;Acc:Q8N0X2]

ATGTCTGAAATAACTTTCTTCTGCCTGTTCTGACCTTTGTTGTCTCACTTTAACTCATCATCCAGCTGCTGCCTGAGTAT

TTTGCTTTAATCTATTCTCT

In100 candidate3414 (in intron):

LOCUS ENSG00000144451

DESCRIPTION Sperm-associated antigen 16 protein (PF20 protein homolog).

[Sorce:Uniprot/SWISSPROT;Acc:Q8N0X2]

GAGGGAAGGGAGGAAAAAAAAGAATCTGTCTTACCATGGTGTTTCTTCTAACTCAAGAACTTGTGTCATGTTCTTCAGAT

ATACCCTATTTCACTTTTAT

In100 candidate3415 (in intron):

LOCUS ENSG00000144451

DESCRIPTION Sperm-associated antigen 16 protein (PF20 protein homolog).

[Sorce:Uniprot/SWISSPROT;Acc:Q8N0X2]

ATAATGTGATTTCCCTTAGTTCCCAAATCCCAAAATTCCTACTATTACTAACCCTGCCTGACAATTTCTTCTCTTCAGAC

TTATTATCTTCTAGTACATT

In100 candidate3416 (in intron):

LOCUS ENSG00000144451

DESCRIPTION Sperm-associated antigen 16 protein (PF20 protein homolog).

[Sorce:Uniprot/SWISSPROT;Acc:Q8N0X2]

ACTGTTGGTTTAATCTCTCTTATGTAACATAAGAAAAGCTACTCCTGTTCACTTTTGTTTTTCTTTTGTATGATACAGCT

TTTTCTACCACTTTACTTTA

In100 candidate3417 (in intron):

LOCUS ENSG00000185554

DESCRIPTION Nuclear RNA export factor 2 (TAP-like protein 2) (TAPL-2).

[Souce:Uniprot/SWISSPROT;Acc:Q9GZY0]

GGTTTGTCTCATTTGTTTAACTTTTCACAAAATCAACTTTCTGTTTCAGTGATTGTTCAGTGTATTGTTTTCTTCAGTTC

AATTTCATTTATTTCTGCTC

In100 candidate3418 (in intron):

LOCUS ENSG00000144452

DESCRIPTION ATP-binding cassette sub-family A member 12 (ATP-binding cassete

transporter 12) (ATP-binding cassette 12). [Source:Uniprot/SWISSPROT;Acc:86UK0]

TTAATAAAGATAAAGTCTAACTCTAGCTGCATTACCTTACTTGTGAGTTTACAACTCTTCCCTTGATTTCTCTCCAGGTC

CTGAATACATGTTTTCCTCT

In100 candidate3419 (in intron):

LOCUS ENSG00000144452

DESCRIPTION ATP-binding cassette sub-family A member 12 (ATP-binding cassete

transporter 12) (ATP-binding cassette 12). [Source:Uniprot/SWISSPROT;Acc:86UK0]

TATACACGCTTGCACTAGGAGAGAGGAAGGGGAGAGAGAGAGAATGAATTAACTTTATTTTTTCCTTTGGCTTACCAGGC

ATTAATGGCTTTATTTTCCC

In100 candidate3420 (in intron):

LOCUS ENSG00000106771

DESCRIPTION Uncharacterized protein C9orf5 (Protein CG-2).

[Source:Uniprot/WISSPROT;Acc:Q9H330]

AGATTATTTGTATTGATAGATTTGACCAAAATAACGTAGGAGATAGTTTTTTTCTGTCTTTTTTAGTAATCCCATCAGTT

CTTTTTTCTTCATGCTCTGA

In100 candidate3421 (in intron):

LOCUS ENSG00000185945

DESCRIPTION Nuclear RNA export factor 2 (TAP-like protein 2) (TAPL-2).

[Souce:Uniprot/SWISSPROT;Acc:Q9GZY0]

GGTTTGTCTCATTTGTTTAACTTTTCACAAAATCAACTTTCTGTTTCAGTGATTGTTCAGTGTATTGTTTTCTTCAGTTC

AATTTCATTTATTTCTGCTC

In100 candidate3422 (in intron):

LOCUS ENSG00000157654

DESCRIPTION Paralemmin-2. [Source:Uniprot/SWISSPROT;Acc:Q8IXS6]

ACTATAAATTCTCTCTAGTCGTTAGTTTAGCTGCCTCTCATAATTGTAACACACTGTGTTATCATTTTCATTCAGTTTTA

AATATTTTCCAAATTCCTTT

In100 candidate3423 (in intron):

LOCUS ENSG00000165124

DESCRIPTION polydom [Source:RefSeq_peptide;Acc:NP_699197]

ACAACTAAGAAAATGTTTGTAGTATCTGAAATCCTCAAAGGAACCTAACTAACTGTTTTTTCCTTTTTTCTCCATCAGCT

TTTTCTTTGTTAACTGTCTT

In100 candidate3424 (in intron):

LOCUS ENSG00000177410

DESCRIPTION C20orf199 protein (Fragment). [Source:Uniprot/SPTREMBL;Acc:Q8N53]

AGTTAAAACTTTGAAGAGAAATCCATTGTATCTAACGATTTCCCCCTTTTGATTTTTATAATTATTTCTCTTCAGACTTG

TTTTAGCGTGTCTTGATTTT

In100 candidate3425 (in intron):

LOCUS ENSG00000158470

DESCRIPTION Beta-1,4-galactosyltransferase 5 (EC 2.4.1.-) (Beta-1,4-GalTase5)

(Beta4Gal-T5) (b4Gal-T5) (UDP-galactose:beta-N-acetylglucosamine beta- 14-

galactosyltransferase 5) (UDP-Gal:beta-GlcNAc beta-1,4- galactosyltransfease 5) (Beta-1,4-GalT II). [Source:Uniprot/SWISSPROT;Acc:O43286]

TAATCCCCATTTTGATTCTATTTTATTCCCTGGAAGTAACCATTTCTTTCCGTACTTTTCTTTTCTTTTTTGAGACAGAT
TTTCTTTCTGTTACCCAAGC

In100 candidate3426 (in intron):

LOCUS ENSG00000198121

DESCRIPTION Lysophosphatidic acid receptor Edg-2 (LPA receptor 1) (LPA-1). Source:Uniprot/SWISSPROT;Acc:Q92633]

TGATCTTTTTCTCTTTGTTTTGTGGATGAGTAAATTAACATTCCCTCCCATTTCTTCCTCTCTCTCTTAATGGCTCAGAC
TGAATTCAATTTCTTTCTTT

In100 candidate3427 (in exon):

LOCUS ENSG00000079246

DESCRIPTION ATP-dependent DNA helicase 2 subunit 2 (EC 3.6.1.-) (ATP-dependnt DNA helicase II 80 kDa subunit) (Lupus Ku autoantigen protein p86) (Ku86 (Ku80) (86 kDa subunit of Ku antigen) (Thyroid-lupus autoantigen) (TLAA) (TC boxbinding factor 85 kDa subunit) ([Source:Uniprot/SWISSPROT;Acc:P1301]

TCCTTTACTAAATAAGAAAATAACAAAGCCCTTATTCTCTTTTTTTCTTGTCCTCATTCTTGCCTTGAGTTCCAGTTCCT
CTTTGGTGTACAGACTTCTT

In100 candidate3428 (in intron):

LOCUS ENSG00000185737

DESCRIPTION Pro-neuregulin-3, membrane-bound isoform precursor (Pro-NRG3)

[ontains: Neuregulin-3 (NRG-3)]. [Source:Uniprot/SWISSPROT;Acc:P56975]

TTTTCTTTAGCTGAGGAAAGTTATTTTTCATTTTTTTCTATGGTAACTGCACTTGTTCCATTTTGCTGTCTTTTTCAGAT

TTCTTTCTTATGTGAATTTG

In100 candidate3429 (in intron):

LOCUS ENSG00000185737

DESCRIPTION Pro-neuregulin-3, membrane-bound isoform precursor (Pro-NRG3)

[ontains: Neuregulin-3 (NRG-3)]. [Source:Uniprot/SWISSPROT;Acc:P56975]

TCTCTTAATAATAAACAAAATGACTATTAATTTAACTCTGAATATGACTTTGAATATTTTCTCTTTGTGCAGCCTAAGAT

TGCTGCATTTGTCTTCTTTC

In100 candidate3430 (in intron):

LOCUS ENSG00000185737

DESCRIPTION Pro-neuregulin-3, membrane-bound isoform precursor (Pro-NRG3)

[ontains: Neuregulin-3 (NRG-3)]. [Source:Uniprot/SWISSPROT;Acc:P56975]

ACTGTGGCTTTAAGACTCAATAGAAGAATCAAATTAACATCTGAAAAATATTTCTTACCAAGCTTTCATTTCCAGTATTA

CCCTGGTAATTTGTTTTTCT

In100 candidate3431 (in intron):

LOCUS ENSG00000185737

DESCRIPTION Pro-neuregulin-3, membrane-bound isoform precursor (Pro-NRG3)

[ontains: Neuregulin-3 (NRG-3)]. [Source:Uniprot/SWISSPROT;Acc:P56975]

TTATATTATCTGTTATATTTACCTATGTACTTTAACTAGTGCTTTTATTTCTTCTTGTGGATTTGACTTCCTGTCCAGTA

TTCCCCCTTTTTATTTAGGT

In100 candidate3432 (in intron):

LOCUS ENSG00000185737

DESCRIPTION Pro-neuregulin-3, membrane-bound isoform precursor (Pro-NRG3)

[ontains: Neuregulin-3 (NRG-3)]. [Source:Uniprot/SWISSPROT;Acc:P56975]

TAATACCTACCTTGATCTTTTATTAACCTCATAAGCATCTTTAGTCCTTTGATCCCTCTTTTTTTAATACCATACCAGCA

ACCTTAACATTTTCCTTTCT

In100 candidate3433 (in intron):

LOCUS ENSG00000185737

DESCRIPTION Pro-neuregulin-3, membrane-bound isoform precursor (Pro-NRG3)

[ontains: Neuregulin-3 (NRG-3)]. [Source:Uniprot/SWISSPROT;Acc:P56975]

TTTTAAATCCATGGTAACAAAATAACTCACTGCTTTGTTTTTCTGTTCCTTTTCTGGTCTTCAAATCAGAAGCTGCTGTT

ATAGTTTTCCTTTATATTTT

In100 candidate3434 (in intron):

LOCUS ENSG00000185737

DESCRIPTION Pro-neuregulin-3, membrane-bound isoform precursor (Pro-NRG3)

[ontains: Neuregulin-3 (NRG-3)]. [Source:Uniprot/SWISSPROT;Acc:P56975]

TTTAATCGTTTTTGTGGTTTCTCATTAGAGAATAGGTAAATAATGAAATAACTAGAGTCTTCCCTTGCTTTCATTCAGTT

TTGTGTTGTTCTTTCCTTCC

In100 candidate3435 (in intron):

LOCUS ENSG00000136813

DESCRIPTION Proteasome-associated protein ECM29 homolog (Ecm29).

[Source:Unprot/SWISSPROT;Acc:Q5VYK3]

GGCCTTGGAGAGTAATCTTATTAACATTTATGTATGTCTGACTCATCTTTGTCAAATTTTTACATTTTCTGCTATCAGCT

TCAGGTCTTGTATTTTATTT

In100 candidate3436 (in intron):

LOCUS ENSG00000136813

DESCRIPTION Proteasome-associated protein ECM29 homolog (Ecm29).

[Source:Unprot/SWISSPROT;Acc:Q5VYK3]

ATGCTGCTGTAAATTTATATTCACTAACCTCAATTGGGTCCTTTACACTTTCTTCACATTTGTAGTTCATTTTACCAGTC

AATTTTCTTCTCTTCTTCAC

In100 candidate3437 (in intron):

LOCUS ENSG00000107771

DESCRIPTION granule cell antiserum positive 14

[Source:RefSeq_peptide;Acc:N_061872]

TTAGTGATCTTCTCTATCTCTAACTATGCTTTTTGTTTTCACACTCCTTTTTTTCCAAATATTAATATACCTTACCAGTT

TTATTTATATATTTTCTCTT

In100 candidate3438 (in intron):

LOCUS ENSG00000182771

DESCRIPTION Glutamate receptor delta-1 subunit precursor (GluR delta-1).

[Surce:Uniprot/SWISSPROT;Acc:Q9ULK0]

CTGTCTTCTGTCTGTTCAAATCTGCTCTTTAACCCCCTAATGAATTTTTCATTTTGTTATTGTACTTTTTAGCCCCAGAA

TTTCTGTTTGCTTCTTTTAA

In100 candidate3439 (in intron):

LOCUS ENSG00000062650

DESCRIPTION Wings apart-like protein homolog (Friend of EBNA2 protein).

[Sorce:Uniprot/SWISSPROT;Acc:Q7Z5K2]

AGTGGGGATAACATGTCATTTCCTCTTAGATTTTCAGGAAGGAATGCTTTCTTTCCTCTGTTTTTGTTGTTGTTGTTTT

GTTTTTTTTGTTTGTTTTTG

In100 candidate3440 (in intron):

LOCUS ENSG00000196396

DESCRIPTION Tyrosine-protein phosphatase non-receptor type 1 (EC 3.1.3.48)

Protein-tyrosine phosphatase 1B) (PTP-1B). [Source:Uniprot/SWISSPROT;Acc:P1031]

GAATTTGTACTCACTGCTGAATGCTTCCAAATGATACGTAGAATAACAAATTTAAGACTTAGATTTTTACTTTTTCAGGT

CTTTTTTTTTTTTTCTGTGC

In100 candidate3441 (in intron):

LOCUS ENSG00000189108

DESCRIPTION X-linked interleukin-1 receptor accessory protein-like 2 precuror

(IL1RAPL-2-related protein) (Interleukin-1 receptor 9) (IL-1R9) (IL-1 reeptor

accessory protein-like 2) (Three immunoglobulin domain- containing IL1 receptorrelated

1) (TIGIRR-1). [Source:Uniprot/SWISSPROT;Acc:Q9NP60]

CCTTAGCAAATCTAGCCAATTCTTTTTCAATTCTATGACTTAACATTACCTTTTCTTTTTGTTTTTATTAGCGTTCAGGT

TTTAGACTATTTGTTTCCAC

In100 candidate3442 (in intron):

LOCUS ENSG00000189108

DESCRIPTION X-linked interleukin-1 receptor accessory protein-like 2 precuror (IL1RAPL-2-related protein) (Interleukin-1 receptor 9) (IL-1R9) (IL-1 reeptor accessory protein-like 2) (Three immunoglobulin domain- containing IL1 receptorrelated

1) (TIGIRR-1). [Source:Uniprot/SWISSPROT;Acc:Q9NP60]

GGAATCATGCTTTTTATTGAAATTGTTGGTAATTTTATTTGTATGTATTAACTGAACATTGATTATTTTGCTACCCAGAA

TCTATTCACCTTTCTTTTTG

In100 candidate3443 (in intron):

LOCUS ENSG00000189108

DESCRIPTION X-linked interleukin-1 receptor accessory protein-like 2 precuror (IL1RAPL-2-related protein) (Interleukin-1 receptor 9) (IL-1R9) (IL-1 reeptor accessory protein-like 2) (Three immunoglobulin domain- containing IL1 receptorrelated

1) (TIGIRR-1). [Source:Uniprot/SWISSPROT;Acc:Q9NP60]

AATTGCTTTGGGCAATGTGACCATTTTAACAATGTTGATTCTTCTTATCCATCAGCATGGAATGCTTTTCCATTTGTTTG

TGTCATCTCTGATTTCTTCC

In100 candidate3444 (in intron):

LOCUS ENSG00000189108

DESCRIPTION X-linked interleukin-1 receptor accessory protein-like 2 precuror (IL1RAPL-2-related protein) (Interleukin-1 receptor 9) (IL-1R9) (IL-1 reeptor accessory protein-like 2) (Three immunoglobulin domain- containing IL1 receptorrelated

1) (TIGIRR-1). [Source:Uniprot/SWISSPROT;Acc:Q9NP60]

TCACTTATTATGTTAACCATATAGTCTTCTATTTTTTCATAATACTTATCAGAATTTTAATTTTGTATTTTGATATTTAT

TTGTTTAGTGTTTGTCTTTC

In100 candidate3445 (in intron):

LOCUS ENSG00000189108

DESCRIPTION X-linked interleukin-1 receptor accessory protein-like 2 precuror (IL1RAPL-2-related protein) (Interleukin-1 receptor 9) (IL-1R9) (IL-1 reeptor accessory protein-like 2) (Three immunoglobulin domain- containing IL1 receptorrelated

1) (TIGIRR-1). [Source:Uniprot/SWISSPROT;Acc:Q9NP60]

TGATACATAGAAAAAAAATTACCATAAACTCTTAACCCTAATGTGAGCATTATAACTGTTTGATTATTTTCCTTCCAGCC

TTTTAAGGCCTATTTTATTT

In100 candidate3446 (in intron):

LOCUS ENSG00000189108

DESCRIPTION X-linked interleukin-1 receptor accessory protein-like 2 precuror (IL1RAPL-2-related protein) (Interleukin-1 receptor 9) (IL-1R9) (IL-1 reeptor accessory protein-like 2) (Three immunoglobulin domain- containing IL1 receptorrelated

1) (TIGIRR-1). [Source:Uniprot/SWISSPROT;Acc:Q9NP60]

AGAGTGATAACTTCTCAATTACTATTTCTGTGGTGTTTTTTTGTTTCATTTTGTTTTTGCAGATTTCACTGCTTATTTTT

TCCCCTGAATGCATAATCTC

In100 candidate3447 (in intron):

LOCUS ENSG00000135913

DESCRIPTION Ubiquitin carboxyl-terminal hydrolase 37 (EC 3.1.2.15) (Ubiquitn thioesterase 37) (Ubiquitin-specific-processing protease 37) (Deubiquitinting enzyme 37). [Source:Uniprot/SWISSPROT;Acc:Q86T82]

CTGAGCCATCCTGCCTTGTACTGTGGGTGTTTTGAAATTTTTAGGTAGATAACTTTTACTATCTTTTCCTATGACCAGTT

CAAATTTTTCTCTCTTGATA

In100 candidate3448 (in intron):

LOCUS ENSG00000107779

DESCRIPTION Bone morphogenetic protein receptor type IA precursor (EC 2.7.1.30) (Serine/threonine-protein kinase receptor R5) (SKR5) (Activin receptorlike kinase 3) (ALK-3) (CD292 antigen). [Source:Uniprot/SWISSPROT;Acc:P3689]

AGTAACTTGATTTTATACTTGGCTTTCTATATTGTGGCTCCAATTTTTCTTCCCAGTTTATACTCTTATTTTTCCTTTCT

TTTTCTTTTCTTTTCTTTTT

In100 candidate3449 (in intron):

LOCUS ENSG00000157502

DESCRIPTION MUM1-like protein 1 (Mutated melanoma-associated antigen 1-likeprotein 1). [Source:Uniprot/SWISSPROT;Acc:Q5H9M0]

AGCTTCTGCTTTTAACTGGGTACATATTCTTAACTATATCATTATCTCCAGATTGTAGCATTTTGTTTTCTACTTCTTTC

TTCCTCCCCATTTCCTTCCT

In100 candidate3450 (in intron):

LOCUS ENSG00000106868

DESCRIPTION Sushi domain-containing protein 1 precursor.

[Source:Uniprot/SWSSPROT;Acc:Q6UWL2]

ATTCTTTAACATCTTTAAAATTGTATTTACTAATCCCTAAATCCCCATTTTCTTTTTTCCTTTTTTCAGGTTTCTTATAT

TAAAATTCTAGTGTTTTCTT

In100 candidate3451 (in intron):

LOCUS ENSG00000106868

DESCRIPTION Sushi domain-containing protein 1 precursor.

[Source:Uniprot/SWSSPROT;Acc:Q6UWL2]

CAAGTTAATCTAGAGAGAATTAGGATTTATGTTGAGCCATCCTAACTGGGAGCAAGGTATGTTTCCACATTTACTCAGTG

TCTACTCAGGTCTTCTGTTT

In100 candidate3452 (in intron):

LOCUS ENSG00000106868

DESCRIPTION Sushi domain-containing protein 1 precursor.

[Source:Uniprot/SWSSPROT;Acc:Q6UWL2]

CTTTGCCTATATTCCACTTCATTCACTTTATTTTTGACCCTTTTAACTTCTTTTTGATCTCATTTTTATTATTTTCAGTT

GTTTTCCTATATTTCTTCAA

In100 candidate3453 (in intron):

LOCUS ENSG00000101096

DESCRIPTION Nuclear factor of activated T-cells, cytoplasmic 2 (T cell trancription factor NFAT1) (NFAT pre-existing subunit) (NF-ATp). [Source:Unipro/SWISSPROT;Acc:Q13469]

TGAGATTTGGGTGTGTTTTGGTGTGTGCTGCCTTCAAGTAACATTCTGGACTCTCTTTTTGCTCTGAACCAGTTCAACTT
TGTTTTAGCACCAATTTTCC

In100 candidate3454 (in intron):

LOCUS ENSG00000119314

DESCRIPTION Regulator of differentiation 1 (Rod1). [Source:Uniprot/SWISSPRO;Acc:O95758]

CCCATATAAGAGTTTTTGTCACCCCACATTATTACTAACATGATATTGTCCATCTTTTTAATTTTACAGTATTTCATTGT
GGTTTTAACTTGCATTTCCC

In100 candidate3455 (in intron):

LOCUS ENSG00000115649

DESCRIPTION Uncharacterized protein C2orf24. [Source:Uniprot/SWISSPROT;Acc:9BV87]

CCCTGGCCCATCTTTAACCGTCCTTTAGTCCTTTGTTCTCTCTCAGTCTTCTGTTGGTTCGTCATTTCTCTTCCTGTCCT
CCTCCTGCCTTTGTGTTAGT

In100 candidate3456 (in intron):

LOCUS ENSG00000020256

DESCRIPTION Zinc finger protein 64, isoforms 3 and 4 (Zinc finger protein 38). [Source:Uniprot/SWISSPROT;Acc:Q9NTW7]

AATAACATTCTAAGTCTTATTTTGTATTTTTTTTTCATTTGCCTGGTTCATATCTGCCCATTCTGTTATTCAGTATCTTT

CTTTATCAGCCTCTCCTTGT

In100 candidate3457 (in intron):

LOCUS ENSG00000089682

DESCRIPTION RNA-binding protein 41 (RNA-binding motif protein 41).

[Source:niprot/SWISSPROT;Acc:Q96IZ5]

GCCTCTCTCATCTAACCCTTTCTTTCCATTTTCACTATTACCATTTCAGTCTAGACCTTCATCACCTTTTACTTCAGTAG

TTTCATAACTAGTTCTTCTT

In100 candidate3458 (in intron):

LOCUS ENSG00000089682

DESCRIPTION RNA-binding protein 41 (RNA-binding motif protein 41).

[Source:niprot/SWISSPROT;Acc:Q96IZ5]

TTCATCTGTCTAAACCATCACTTGCACTGTGTCACTTAACTGTTTGATAAATTACTACTTCTTATTATCCTTCAGTATAT

ATTACTATCCCTTTGTACTC

In100 candidate3459 (in intron):

LOCUS ENSG00000165185

DESCRIPTION Uncharacterized protein KIAA1958.

[Source:Uniprot/SWISSPROT;AccQ8N8K9]

ATTCTTGGTTTTCTTCAAAACTGATACTGCTTTAACCTTTTCTGTCCTTTTTTAATTGCAAATATTCTGTCCCTTCAGTT

TTTTCTTATATTTTCCTCTG

In100 candidate3460 (in intron):

LOCUS ENSG00000138138

DESCRIPTION ATPase family AAA domain-containing protein 1.

[Source:Uniprot/WISSPROT;Acc:Q8NBU5]

CCTCCTAAAGTGCTGGGATTATAACGCCTAGCTGATTTACTTCTGCTATATTATTTTTTGCTTTTAATTTACCAGTCTAG

TAGACTTTTTTGCTTTATTC

In100 candidate3461 (in intron):

LOCUS ENSG00000138138

DESCRIPTION ATPase family AAA domain-containing protein 1.

[Source:Uniprot/WISSPROT;Acc:Q8NBU5]

ATTATGCATTTTGAGTAAAAATCTGATGTAACTAGAACTACATTAGCTTTCTTTTTATTAATCGTTGATGTCTTTCAGTC

TTTTACTTCCTGCTTTTCTT

In100 candidate3462 (in intron):

LOCUS ENSG00000198088

DESCRIPTION Nucleoporin-62 C-terminal-like protein.

[Source:Uniprot/SWISSPRT;Acc:Q9H1M0]

TTGTCATGCTTTTCTCCCTTATTCTTCACATAACTAAGTATGTACCTGTATTTATTTTTCTGTTCACTTACTAGCCAGTT

CATTCTTCTTTTTACATATT

In100 candidate3463 (in intron):

LOCUS ENSG00000171862

DESCRIPTION Phosphatidylinositol-3,4,5-trisphosphate 3-phosphatase and dual

specificity protein phosphatase PTEN (EC 3.1.3.67) (EC 3.1.3.16) (EC 3.1.348) (Phosphatase and tensin homolog) (Mutated in multiple advanced cancers). [Source:Uniprot/SWISSPROT;Acc:P60484]

TATTAAACTTGTATGTTTTAAAAGCTTATTTACTTGTTGAAATGGTTAACTTAATTAGTTTTTTCTTTGAAGTTTCAGCC
TAAATATTTTCTGTTTTTTT

In100 candidate3464 (in intron):

LOCUS ENSG00000171862

DESCRIPTION Phosphatidylinositol-3,4,5-trisphosphate 3-phosphatase and dual specificity protein phosphatase PTEN (EC 3.1.3.67) (EC 3.1.3.16) (EC 3.1.348) (Phosphatase and tensin homolog) (Mutated in multiple advanced cancers). [Source:Uniprot/SWISSPROT;Acc:P60484]

ATCATTTACAATTAAAGTAATTATTGATAAGGATTTAACTGCTGCCATTTTGCTGTTTATTTTCTATATGCCTTACAGCT
TTTTTGTCCCTCATTTCCTG

In100 candidate3465 (in intron):

LOCUS ENSG00000184719

DESCRIPTION Renalase precursor (EC 1.4.-.-). [Source:Uniprot/SWISSPROT;Acc:5VYX0]

ATAAGGTTTTGTAATAGAAATGCAATTGCAATATAAGTAATTAACTGATATGTCTCTTAAATCTCTTTTATTCTACAGGT
TTTTTTTCTCATTTTTCCCC

In100 candidate3466 (in intron):

LOCUS ENSG00000184719

DESCRIPTION Renalase precursor (EC 1.4.-.-).

[Source:Uniprot/SWISSPROT;Acc:5VYX0]

CATTCTTTGAAATGTGCTTTCCCTGCTCCTACCCCATTTTTAACTAGACCCTCTCTTTTCTGTATCTTATTGCTCCAGTC

CTATTCAATTTGGATTCTCT

In100 candidate3467 (in intron):

LOCUS ENSG00000136866

DESCRIPTION Zinc finger protein 37 homolog (Zfp-37).

[Source:Uniprot/SWISSPOT;Acc:Q9Y6Q3]

ATTCACCATGAGTAGTATAAAGTGGATAACCTTTCCTATTTATTCTCTTAGCCACTTATCAGCTCATCAGTACTTCTTGG

TGGACTCACTTTATTTTTCT

In100 candidate3468 (in intron):

LOCUS ENSG00000157514

DESCRIPTION TSC22 domain family protein 3 (Glucocorticoid-induced leucine

zpper protein) (Delta sleep-inducing peptide immunoreactor) (DSIP- immunoreative

peptide) (Protein DIP) (hDIP) (TSC-22-like protein) (TSC-22R).

[SourceUniprot/SWISSPROT;Acc:Q99576]

TTCAACCATCCCATTAACACTGTGTTAACGCTCTAGTGCTCAATATCCTTTTTGTATTTCCTTTTCCAGCTCCTAACCCA

GTGTTTGCTTTGTGGCTTCT

In100 candidate3469 (in intron):

LOCUS ENSG00000101134

DESCRIPTION Docking protein 5 (Downstream of tyrosine kinase 5) (IRS6)

(Proein dok-5). [Source:Uniprot/SWISSPROT;Acc:Q9P104]

AGTTGTTAACGATCTTATTGCCTGTCCCCTTTGCTTTTCTCTTCTCTATTTGTCAGGTTTTTTTTTTTTTTTTTTTTA

CTTTCATGTTATTCAATACC

In100 candidate3470 (in exon):

LOCUS ENSG00000148229

DESCRIPTION DNA polymerase epsilon subunit 3 (EC 2.7.7.7) (DNA polymerase I subunit 3) (DNA polymerase epsilon subunit p17) (Chromatin accessibility cmplex 17) (HuCHRAC17) (CHRAC-17) (Arsenic- transactivated protein) (AsTP). Source:Uniprot/SWISSPROT;Acc:Q9NRF9]

CTCCCTAAACCATCCTCTTTAACTGCTTCTAAAATATCTCTGTTGGCCTTTCTTAGCCTTTTTCTGTTTCCATTCAGTGC

TCCAAGCGCTTTTTGTTTCT

In100 candidate3471 (in intron):

LOCUS ENSG00000087586

DESCRIPTION Serine/threonine-protein kinase 6 (EC 2.7.11.1) (Aurora kinase) (Aurora-A) (Serine/threonine kinase 15) (Aurora/IPL1-related kinase 1) (Arorarelated

kinase 1) (hARK1) (Breast-tumor-amplified kinase).

[Source:Unirot/SWISSPROT;Acc:O14965]

TCCATCTCAAAAATAATAATAATAATACATTTTAGTAGTAACTTTGTGAAGTGTCTACATTTGTTTCCTCTTTGTCAGTT

TTTTGCTCAATTCCATTTTG

In100 candidate3472 (in intron):

LOCUS ENSG00000087586

DESCRIPTION Serine/threonine-protein kinase 6 (EC 2.7.11.1) (Aurora kinase)

(Aurora-A) (Serine/threonine kinase 15) (Aurora/IPL1-related kinase 1)

(Arorarelated

kinase 1) (hARK1) (Breast-tumor-amplified kinase).

[Source:Unirot/SWISSPROT;Acc:O14965]

ATTAATATGGCACATTTGTCACATTCAATGAACCAATATTGATATATAACTATTAACTCAAGTCCACATTTTATTCAGAT

TTTCTTAGTTTTTCTCATGA

In100 candidate3473 (in intron):

LOCUS ENSG00000101844

DESCRIPTION Cysteine protease ATG4A (EC 3.4.22.-) (Autophagy-related protei 4

homolog A) (hAPG4A) (Autophagin-2) (Autophagy-related cysteine endopeptiase 2)

(AUT-like 2 cysteine endopeptidase). [Source:Uniprot/SWISSPROT;Acc:QWYN0]

GCATCTTTTTATATGCTTTTTGGCCATTTGTATATCATCTTTAGATAACTATTCAATTCTTTGCCCATTTTTAATCAGGT

TATTTGATTTTTTGTTGTTC

In100 candidate3474 (in intron):

LOCUS ENSG00000157657

DESCRIPTION zinc finger protein 618 [Source:RefSeq_peptide;Acc:NP_588615]

TTTCTTCTTTAATATCTAACCTCTTCTTTTTTTTTTTCTAACCTATTCTTAATACTACCCAGTGTATTTTTTTAATCCCA

GGCATTGTATTTTTCATCTC

In100 candidate3475 (in intron):

LOCUS ENSG00000157657

DESCRIPTION zinc finger protein 618 [Source:RefSeq_peptide;Acc:NP_588615]

TTTCTATTTCTTTAACTTCTGTTTATTTTTATTCATTTTCTGCTTTCTTTTGGCTTATTTTGATTTTATTCTTCTCAGTT

CATTTCTTTTTTGAGTGACT

In100 candidate3476 (in intron):

LOCUS ENSG00000197565

DESCRIPTION Collagen alpha-6(IV) chain precursor.

[Source:Uniprot/SWISSPROTAcc:Q14031]

TATTAACACCACCTGTGTGTTTTTTTTCATTTCAGATATTATATTTTTCTTTTCTAGAAATTCCTTTTGGATTTTAAATT

TTACAAACTTCCATTCTTCT

In100 candidate3477 (in intron):

LOCUS ENSG00000152782

DESCRIPTION Pantothenate kinase 1 (EC 2.7.1.33) (Pantothenic acid kinase

1)(hPanK1) (hPanK). [Source:Uniprot/SWISSPROT;Acc:Q8TE04]

CCTGTCCTGGAAGTAACAATGTATCTATCACCTTTTTCAACCCTAAAGTTTTCCTTCAACATTGTTTTTCAGTTCCTATC

CAGTAAGATACTCTTTTTTT

In100 candidate3478 (in intron):

LOCUS ENSG00000188153

DESCRIPTION Collagen alpha-5(IV) chain precursor.

[Source:Uniprot/SWISSPROTAcc:P29400]

GAAGTGTATATAATAAAAGGTGCAAGCTTCCTGAGAGGTAACTGTTATTTGCAATTTCGTATATCTTTTCAGATATTTTT

CTGGTATCTATCTTTCAAGT

In100 candidate3479 (in intron):

LOCUS ENSG00000188153

DESCRIPTION Collagen alpha-5(IV) chain precursor.

[Source:Uniprot/SWISSPROTAcc:P29400]

AACATGCCAAATTATTCCATAACACTTGTATCATTCTCTTATAATTTATCCCAGTTCCTCTCATTTCCCTCTGTCTTTAA

TCTTATCTATTTTAAAATTA

In100 candidate3480 (in intron):

LOCUS ENSG00000188153

DESCRIPTION Collagen alpha-5(IV) chain precursor.

[Source:Uniprot/SWISSPROTAcc:P29400]

CCAACAAAGGCACTTAAATGCATCAAATTTTGGTAACAAATATTAATTTTCACTTCATTCATACTGTTTCACCAGTCAAC

TTTTGTTTGAGTATTTTCTT

In100 candidate3481 (in intron):

LOCUS ENSG00000148688

DESCRIPTION Ribonuclease P protein subunit p30 (EC 3.1.26.5) (RNaseP protei

p30) (RNase P subunit 2). [Source:Uniprot/SWISSPROT;Acc:P78346]

ATAACTGGTATTTGTGGTTTTTTTGTTTTGTTTTGTTTTTTTCATGCTGCTAACCTTTGATAGGCTCAGTTTGAAAGAGC

CCGTTTTCTTCTGTTTCTTT

In100 candidate3482 (in intron):

LOCUS ENSG00000116120

DESCRIPTION Phenylalanyl-tRNA synthetase beta chain (EC 6.1.1.20)

(Phenylalnine-- tRNA ligase beta chain) (PheRS).

[Source:Uniprot/SWISSPROT;Acc:Q9NSD]

GGAAGTAAATAAAAGTTGGAAAAAATTATCCTTGCCCAAACATAACCTCTGTTAACATTTAGGTATATTTCTTTTCAGTC

TTTTTCTCTGTTCATGGGGT

In100 candidate3483 (in intron):

LOCUS ENSG00000095397

DESCRIPTION Whirlin (Autosomal recessive deafness type 31 protein).

[SourceUniprot/SWISSPROT;Acc:Q9P202]

CTTCTCATATATTTAATGGCCATTTATAATGTCTTCTTTGTAACTTGTTCATGTCTTTTATTATCCTTTTGCTCTCAGGA

TTTTGGATCTTTTTTCCTCT

In100 candidate3484 (in intron):

LOCUS ENSG00000123983

DESCRIPTION Long-chain-fatty-acid--CoA ligase 3 (EC 6.2.1.3) (Long-chain acl-

CoA synthetase 3) (LACS 3). [Source:Uniprot/SWISSPROT;Acc:O95573]

CCCTGACACTACTCTGTCGTGATTCTTAGTGATTTCAATATCGTTCTAACACTGTGGTCTTATTATTTCATACTCCAGCC

TGCCTTTGCTATTCCTTTTC

In100 candidate3485 (in intron):

LOCUS ENSG00000152056

DESCRIPTION AP-1 complex subunit sigma-3 (Adapter-related protein complex 1sigma- 1C subunit) (Sigma-adaptin 1C) (Adaptor protein complex AP-1 sigma-1 subunit) (Golgi adaptor HA1/AP1 adaptin sigma-1C subunit) (Clathrin assemby protein complex 1 sigma-1C small chai [Source:Uniprot/SWISSPROT;Acc:Q96PC]

ACATTGCTTATAACTGCAAAATTTTTCATATGCTTCCAGGATGCCTACAGACACTCCTTTTCCTTCCACATTATCTTGGA

ACCTTGGACATTTTTTTTTT

In100 candidate3486 (in intron):

LOCUS ENSG00000124019

DESCRIPTION family with sequence similarity 124B (FAM124B), mRNA [Source:ReSeq_dna;Acc:NM_024785]

TTAGGTTGAGGCATGTTATTACATATTTTAATATTAAGTTAACTTTTCCTCCGATAATTTTTTCAAGCTTTTCAGTTTGC

TTCACGTTCCCGTTCATGTT

In100 candidate3487 (in intron):

LOCUS ENSG00000144460

DESCRIPTION CDNA FLJ30664 fis, clone FCBBF1000604, weakly similar to Myosinheavy chain Myr 8b. [Source:Uniprot/SPTREMBL;Acc:Q96NL2]

AACATTTTCAACAACAAATGTTTAATAATATTTCTGCATATTTATATATAACCTATTTCCCTATCAATTATTTTTCAGTG

ATTCTAACTTATTGCTTTTT

In100 candidate3488 (in intron):

LOCUS ENSG00000144468

DESCRIPTION Rhomboid domain-containing protein 1 (EC 3.4.21.-). [Source:Unirot/SWISSPROT;Acc:Q8TEB9]

ACCTTTTATTTTAACAAAATCATAGGTTTTTTTCTTCCCTTTTTTTCCTTCCTCCTACACATGTATTTTCAGAATTTAAA

ACAGTCTCTTCTTTCCATTC

In100 candidate3489 (in intron):

LOCUS ENSG00000081052

DESCRIPTION Collagen alpha-4(IV) chain precursor.

[Source:Uniprot/SWISSPROTAcc:P53420]

CATGGTCTTAAGAGTTCAAGGGTGATTTGGAAAATCTGTATAATTAGTATAACAAAAGCATCTTTCCTGTTTGTTCAGTT

TTTCATTTATTTATACCTTG

In100 candidate3490 (in intron):

LOCUS ENSG00000081052

DESCRIPTION Collagen alpha-4(IV) chain precursor.

[Source:Uniprot/SWISSPROTAcc:P53420]

AGCTATTGCTTCTATAACTCCACTTATGTTTTATTTGAATTTCATTTCCAGCATTACCATTTTCATTTCTTTGCTTCATT

TTCATCATTATTGGCCGATT

In100 candidate3491 (in intron):

LOCUS ENSG00000081052

DESCRIPTION Collagen alpha-4(IV) chain precursor.

[Source:Uniprot/SWISSPROTAcc:P53420]

TCAAGGAAACGGCTTTTTAACTAGCTTTTTATTCATTAATTTTTCATATTTTTTTGGTTTGCATTTCATTTGGTTCAGTC

TGATCTTTGTTATTTCTTCT

In100 candidate3492 (in intron):

LOCUS ENSG00000198060

DESCRIPTION E3 ubiquitin-protein ligase MARCH5 (EC 6.3.2.-) (Membraneassocated RING finger protein 5) (Membrane-associated RING-CH protein V) (MARCHV) (RING finger protein 153). [Source:Uniprot/SWISSPROT;Acc:Q9NX47]

GTTTTTCTTCAAATACCTTTTCTTTCCACTCTAACTTGAATTCTCATCTGTAGTTATTTCCTTTTCTTGAAACCTCAGCT
GTTTCATCCTGTTTCTTGGT

In100 candidate3493 (in intron):

LOCUS ENSG00000101938

DESCRIPTION Chordin-like protein 1 precursor (Neuralin-1) (Ventroptin) (Neuogenesin-1). [Source:Uniprot/SWISSPROT;Acc:Q9BU40]

GAAATGCTCAACAAATATAACTTGTTGTTTTTGTTATTGTTATCACCGTTCTCATCTGGCTCTATATTTTTCTTGCAGTT
GGTTCCCTGGCTGCTTTTTA

In100 candidate3494 (in intron):

LOCUS ENSG00000124237

DESCRIPTION Uncharacterized protein C20orf85.
[Source:Uniprot/SWISSPROT;AccQ9H1P6]

AGATTTAGATATAAGTAAGTTGTAGTAACAATAAAACATACTTGTTTACCTTCTAAACTTCAGTTATCCTATTTACCCTC
TCTATAATTTTTACCATTTC

In100 candidate3495 (in intron):

LOCUS ENSG00000182752

DESCRIPTION Pappalysin-1 precursor (EC 3.4.24.79) (Pregnancy-associated plama protein-A) (PAPP-A) (Insulin-like growth factor-dependent IGF-binding prtein 4

protease) (IGF-dependent IGFBP-4 protease) (IGFBP-4ase).

[Source:Unirot/SWISSPROT;Acc:Q13219]

AGCATTGCATTTTGTTCATGGGTTTGTTTGCATAACTAGAATATGCTCGTGTTTTCCCATTTTTCAGAGGAGCATTTTCT

GTCTTCCTCCTTTCTCCTTT

In100 candidate3496 (in intron):

LOCUS ENSG00000077264

DESCRIPTION Serine/threonine-protein kinase PAK 3 (EC 2.7.11.1) (p21-activaed

kinase 3) (PAK-3) (Beta-PAK) (Oligophrenin-3).

[Source:Uniprot/SWISSPROTAcc:O75914]

CACTTGAAACAAGAAGATCCCTTAAACTTTATAAAAATTTTGCCTAATAACTCTTCCCTTTTTTATTAATTTTTCCAGCT

TTGACATTTTAATGTTCTCT

In100 candidate3497 (in intron):

LOCUS ENSG00000148219

DESCRIPTION astrotactin 2 isoform a [Source:RefSeq_peptide;Acc:NP_054729]

AAGGTCACCTTCATATTTAACCCTGGCTCCCTCCATTCTGCCTCTTTTGTTTCTGTTATTGGTAATGCCCAGACTGGAAG

CAAGTCTTTTTTTTTTTTTT

In100 candidate3498 (in intron):

LOCUS ENSG00000148219

DESCRIPTION astrotactin 2 isoform a [Source:RefSeq_peptide;Acc:NP_054729]

ACCTGTGTCCCTCTATAACTCTCTGTCTCTCTGTCTGTCCATCTATACCTTTTAGATCCCTTTATCTGTTTCCAGGTGGC

TATATGTGTTTCTGCCTCTC

In100 candidate3499 (in intron):

LOCUS ENSG00000138190

DESCRIPTION Exocyst complex component 6 (Exocyst complex component Sec15A)

Sec15- like 1). [Source:Uniprot/SWISSPROT;Acc:Q8TAG9]

TCTGCCTATTCTGTTATTGAAAGTAGAAGTATTGGAGTCTTCAACTATAACTGTTGAATTGTCCATTTTTTCTTTCAGTT

TTGCCAGTTTTTTATTCATG

In100 candidate3500 (in intron):

LOCUS ENSG00000168955

DESCRIPTION Transmembrane 4 L6 family member 20.

[Source:Uniprot/SWISSPROT;cc:Q53R12]

CATCTTGGGAGGAAGGGAAAGGGTTGACCAAGGCTTGTCCCCTATTTTAACTGTTCTTTGCTTCTTCCCTTTTTCCAGTT

CTTATTTTTGCTTCTGAATA

In100 candidate3501 (in intron):

LOCUS ENSG00000173744

DESCRIPTION Nucleoporin-like protein RIP (HIV-1 Rev-binding protein)

(Revnteracting

protein) (Rev/Rex activation domain-binding protein).

[Source:Unprot/SWISSPROT;Acc:P52594]

ATAGAAGAAGCTAGATGTTGTTTGTAACTACTCTGAGAGTTTACTTAACTTTTTTTACATTACATATTCCAGGTTACAGT

GTATTTCTTTCTAGCTGTTT

In100 candidate3502 (in intron):

LOCUS ENSG00000138119

DESCRIPTION Myoferlin (Fer-1-like protein 3).

[Source:Uniprot/SWISSPROT;AccQ9NZM1]

AGAAATGGCAATTATATTTAACTTTATGGTTTGCATATTTGTTTCCTCTTTAGCCAGATTCAATTCTCCTTTTTTTATCC

TATTTTATGAATCACAACTT

In100 candidate3503 (in intron):

LOCUS ENSG00000153823

DESCRIPTION PTB-containing, cubilin and LRP1-interacting protein (P-CLI1)

(rotein NYGGF4). [Source:Uniprot/SWISSPROT;Acc:Q7Z2X4]

ATTGTTTTTAAGCTTGGCTTCCAATTTCACAAGCACTCTAACACCTTCTAGAAGTTCTTATTTTCTCAGAAGATCTTTTT

GCACCCTGTTATGATCTTTC

In100 candidate3504 (in intron):

LOCUS ENSG00000153827

DESCRIPTION Thyroid receptor-interacting protein 12 (TRIP-12).

[Source:Unipot/SWISSPROT;Acc:Q14669]

TGTTGCATTATTAGTTCTTCATTAATGTGCTTTATATTTACTTAACCATAATTGTTAAAATCTTTCCCTTTTTGTCAGAT

TAAAATTTTTTTTTTTTTTT

In100 candidate3505 (in intron):

LOCUS ENSG00000153827

DESCRIPTION Thyroid receptor-interacting protein 12 (TRIP-12).

[Source:Unipot/SWISSPROT;Acc:Q14669]

GGACCTTTGCTCTAACCATTTGGTATGTTTTTAGTCTCTTTAAACGTACATTCCACTTTCTTCAGCATATTTTTCTCTTG

CCAGGAAATATCTTTCCTCT

In100 candidate3506 (in intron):

LOCUS ENSG00000078725

DESCRIPTION Deleted in bladder cancer protein 1 precursor (Protein FAM5A).

Source:Uniprot/SWISSPROT;Acc:O60477]

ACTGGAAGGAGCTTTCTTAAATCTCCTTAACAATCTGCGTTTTGCTTTCTCTCTTTATCTGTCAGCATCATGAATATCTT

CCACTCTTCTAGTGCTTTCT

In100 candidate3507 (in intron):

LOCUS ENSG00000078725

DESCRIPTION Deleted in bladder cancer protein 1 precursor (Protein FAM5A).

Source:Uniprot/SWISSPROT;Acc:O60477]

GCACCTGACCTAACTGAAACACTTTCTTTACTCAATTTCTCTCTCGTTCATCTTTGTTCACTAGGATAGCCTTCTCAGTG

TCATTGCTGGATTCTTCTCT

In100 candidate3508 (in intron):

LOCUS ENSG00000078725

DESCRIPTION Deleted in bladder cancer protein 1 precursor (Protein FAM5A).

Source:Uniprot/SWISSPROT;Acc:O60477]

AGCTCCTGGATTCATTAACTTTTTGATGGGTTTTTTGTGTCTCTATTTCCTTCAGTTCTGCTCTGATTTTAGTTATTTCT

TGCCTTCTGCTAGCTTTTGA

In100 candidate3509 (in intron):

LOCUS ENSG00000153832

DESCRIPTION F-box only protein 36. [Source:Uniprot/SWISSPROT;Acc:Q8NEA4]

GCTGCTTTACCTACATAGACAAGTTTTGATAACTTGCGTCTTTATTCTCATTCATTTTCATTCAGTTAAAACATTTTCTA

ATTTCCATTTTGATTTCTTC

In100 candidate3510 (in intron):

LOCUS ENSG00000072315

DESCRIPTION Short transient receptor potential channel 5 (TrpC5) (Htrp-5)

(trp5). [Source:Uniprot/SWISSPROT;Acc:Q9UL62]

GGATTAAAGACAATCCAAGACTAGTAATGAAATGCAAGACTTTTAACGAATTACGCTGCTTATTATTTGCTGTTTCAGTG

TGTTTCCATTCTGCTTTTAC

In100 candidate3511 (in intron):

LOCUS ENSG00000072315

DESCRIPTION Short transient receptor potential channel 5 (TrpC5) (Htrp-5)

(trp5). [Source:Uniprot/SWISSPROT;Acc:Q9UL62]

TCTACATTAACTGCCCTCCAATCCTCATTAACCTTCTCATTATTTTTCCTCTTTCTTTTAGTCCTTTTGCTGTCCAGAA

ATTTTGTCCCTTGTGTTCTT

In100 candidate3512 (in intron):

LOCUS ENSG00000072315

DESCRIPTION Short transient receptor potential channel 5 (TrpC5) (Htrp-5) (trp5). [Source:Uniprot/SWISSPROT;Acc:Q9UL62]

TGACCTCTTTCTGCCTCATTTGACTTCTCTGTAAAATAGGGAAGATAACTAGGCTTTTCATTTGCTGCTTACTCTCAGGT
CTATTCCCTGCCTGTTTCTT

In100 candidate3513 (in intron):

LOCUS ENSG00000126016

DESCRIPTION Angiomotin. [Source:Uniprot/SWISSPROT;Acc:Q4VCS5]

TCCCTCCCACATTTGCAAAGTACCTAACCCTTGAATGATCTTTTTTTTTTTTTCCCCAGAAGTCCATGTTTAGTTTCATC
CTGGGTTCTGTTCTGTTCCT

In100 candidate3514 (in intron):

LOCUS ENSG00000079263

DESCRIPTION Nuclear body protein SP140 (Nuclear autoantigen Sp-140) (Speckld 140 kDa) (LYSp100 protein) (Lymphoid-restricted homolog of Sp100). [Sourc:Uniprot/SWISSPROT;Acc:Q13342]

TAAGTTTCTCGAATGTTTTCCTGTTTCAATTTTTTTAACTTCCACTTTAATTTTAATTTTTTTTCCTGCTTGCATCAGGC
TTAAATTGCTCCTTTTTTCT

In100 candidate3515 (in intron):

LOCUS ENSG00000079263

DESCRIPTION Nuclear body protein SP140 (Nuclear autoantigen Sp-140) (Speckld 140 kDa) (LYSp100 protein) (Lymphoid-restricted homolog of Sp100). [Sourc:Uniprot/SWISSPROT;Acc:Q13342]

TTGCTTTACTTTAAATATTTTGAGGCTATTTATGATTTGTAACTTTTTAATGGATGTTTTTCTTTTATCATTATACAGCA

TTCTGCTTTATCCCTGTTAA

In100 candidate3516 (in intron):

LOCUS ENSG00000147246

DESCRIPTION 5-hydroxytryptamine 2C receptor (5-HT-2C) (Serotonin receptor 2) (5- HT2C) (5-HTR2C) (5HT-1C). [Source:Uniprot/SWISSPROT;Acc:P28335]

TAAAACTAACACTACATTATTTTGATATTTGACTGTTTTTCATCTGCCTCTATACCCTTCAAGATGTTTCTTTTTCAGTA
TCATTTTCTTCTCTCATTTT

In100 candidate3517 (in intron):

LOCUS ENSG00000185404

DESCRIPTION CDNA FLJ13054 fis, clone NT2RP3001527, highly similar to Human p140 protein (Sp140) mRNA. [Source:Uniprot/SPTREMBL;Acc:Q9H930]

CTTTAAATATGTTGAGACCATTAATGACTTTATAACCTCTTAGTGGATTTCTTTTCTTTTATCATTATACAGCATTCTGC
TTTATCCATATTGGTGCTTT

In100 candidate3518 (in intron):

LOCUS ENSG00000067066

DESCRIPTION Nuclear autoantigen Sp-100 (Speckled 100 kDa) (Nuclear dotassoiated Sp100 protein) (Lysp100b). [Source:Uniprot/SWISSPROT;Acc:P23497]

CCTAACCCTTCTCATATTTTTTGTTATTATACTATTTCTATATTGTCAGAGCATATGCATTTCATATTCTCATCTGTCAC
CCTACTGCTCACTTTTATTT

In100 candidate3519 (in intron):

LOCUS ENSG00000106780

DESCRIPTION Multiple epidermal growth factor-like domains 9 precursor (EGFike domain-containing protein 5) (Multiple EGF-like domain protein 5). [Souce:Uniprot/SWISSPROT;Acc:Q9H1U4]

GCTTTTTAACATGTAGGTTGATGACATTTTCATTCTTTTGTTTTTTTCAAGTTTTGCTTCAGTTGTGAGACCATTTTATT

TCACAGCCTTATTCTCTCTC

In100 candidate3520 (in intron):

LOCUS ENSG00000138193

DESCRIPTION 1-phosphatidylinositol-4,5-bisphosphate phosphodiesterase epsiln 1 (EC 3.1.4.11) (Phospholipase C-epsilon-1) (PLC-epsilon-1)

(Phosphoinosiidespecific

phospholipase C epsilon-1) (Pancreas- enriched phospholipase C. [Source:Uniprot/SWISSPROT;Acc:Q9P212]

AACTTTCAATTTATAAGGTGATACTTAAGCGACAAAGTAACAAGCTATATTGGCTTTCTTTGTTGTTTGTTTTACAGATC

TCCCTTTACATTTAAATGCT

In100 candidate3521 (in intron):

LOCUS ENSG00000204128

DESCRIPTION

AAGTAGGTATTTGCTGGGGACAACTTTCTCCTTTTCCTTCCTAGATAACTCACTCACAACCTTTCTCTTCTGATTCAGTG

ATTTCTTCTCCTGTGTTCCA

In100 candidate3522 (in intron):

LOCUS ENSG00000173692

DESCRIPTION 26S proteasome non-ATPase regulatory subunit 1 (26S proteasome egulatory subunit RPN2) (26S proteasome regulatory subunit S1) (26S proteasme subunit p112). [Source:Uniprot/SWISSPROT;Acc:Q99460]

TTGAACATACTGACATATTCTTTGAAAACAAATTTAGAACTCATATCTAACAATCCCTCCCCTTGTATTTCCTTACAGCT
TTCTTTTCAAACTTTTTTAC

In100 candidate3523 (in intron):

LOCUS ENSG00000102024

DESCRIPTION Plastin-3 (T-plastin). [Source:Uniprot/SWISSPROT;Acc:P13797]

TCATAATAACTAATGATGTTTAACATTTTTTATGCACTTATTTCCCATCTGTTTGCCCTCTTCAGTGAAATGTCTGTTCA
TGTCTTTTACCTATTTTCTT

In100 candidate3524 (in exon):

LOCUS ENSG00000087916

DESCRIPTION Sodium- and chloride-dependent neutral and basic amino acid trasporter B(0+) (Amino acid transporter ATB0+) (Solute carrier family 6 membe 14). [Source:Uniprot/SWISSPROT;Acc:Q9UN76]

ATGAAGAAAATGAGGGTTCCCATAACTTTGGGCTGCTGCTTGGTTTTGTTTCTCCTTGGTCTCGTCTGTGTGACTCAGGT
ATACTACAGCATTTTTTTTC

In100 candidate3525 (in intron):

LOCUS ENSG00000003096

DESCRIPTION Kelch-like protein 13 (BTB and kelch domain-containing protein).

[Source:Uniprot/SWISSPROT;Acc:Q9P2N7]

AGTAACTTTGGAAGCTATTCTTGCTTCTTGTATCATTTGTGTTCCAGGCTACGTTGGCTCTGTTTCTTTTTATGTGAGTC

CTTTACACCTCTGTCTTTCT

In100 candidate3526 (in intron):

LOCUS ENSG00000003096

DESCRIPTION Kelch-like protein 13 (BTB and kelch domain-containing protein).

[Source:Uniprot/SWISSPROT;Acc:Q9P2N7]

TCTGAAAATCCTCAAGCAAAACGTCTTGATCATCCCAAAATAACTGTTGTTGTGACTTTTGCTTGTGACCAGTCTGCTTT

TGTTTTGACTGGGTTACTTC

In100 candidate3527 (in intron):

LOCUS ENSG00000003096

DESCRIPTION Kelch-like protein 13 (BTB and kelch domain-containing protein).

[Source:Uniprot/SWISSPROT;Acc:Q9P2N7]

ATGTATGGTTTTCGGTGGTGCTGTTTTAACATGGAAAATTGATGCACTTTTTTCATACTGTCCATCTTTATCTCACAGCT

TTTCTTTTTCAAGCTTATAT

In100 candidate3528 (in intron):

LOCUS ENSG00000119969

DESCRIPTION Lymphoid-specific helicase (EC 3.6.1.-) (SWI/SNF2-related

matriassociated

actin-dependent regulator of chromatin subfamily A member 6)

(roliferation-associated SNF2-like protein).

[Source:Uniprot/SWISSPROT;Acc:QNRZ9]

TTAGTCATTTATTCCCTGCTCTTTAACCTGTTAATTTAATATATCCTTCACTTTATTCCACCTAACTTTTTATACCAGGG

TTTTGATTTTTTTCCCCTCT

In100 candidate3529 (in intron):

LOCUS ENSG00000119969

DESCRIPTION Lymphoid-specific helicase (EC 3.6.1.-) (SWI/SNF2-related

matriassociated

actin-dependent regulator of chromatin subfamily A member 6)

(roliferation-associated SNF2-like protein).

[Source:Uniprot/SWISSPROT;Acc:QNRZ9]

AGAGGTGGGATGTTATTATCATGTAACTGAAGGAGACCCTTAAGTTTTGTTTAACTTTCCAGGTTTTTTATTGTACACTA

TAAACCTACGTATTTTCTTT

In100 candidate3530 (in intron):

LOCUS ENSG00000144535

DESCRIPTION DIS3 mitotic control homolog (S. cerevisiae)-like 2

[Source:Refeq_peptide;Acc:NP_689596]

AGGTAACTGCACATACCTTATTCACATTCTACCATTTTCACTTGTACTCCGTATTCAGATTTTACCAGTTTTGCTTTTAC

TCATTTGTGTGTGTGTTCTG

In100 candidate3531 (in intron):

LOCUS ENSG00000144535

DESCRIPTION DIS3 mitotic control homolog (S. cerevisiae)-like 2

[Source:Refeq_peptide;Acc:NP_689596]

AGTCATCTGAAGGCTACATTTGTTTCCTTATATGTAACTGCTCCTAATTTTTCTGTTTCTATTGTCTGAGTTCAGGACCT

TGCAATTATTTTTGACCCTT

In100 candidate3532 (in intron):

LOCUS ENSG00000144535

DESCRIPTION DIS3 mitotic control homolog (S. cerevisiae)-like 2

[Source:Refeq_peptide;Acc:NP_689596]

TTTTTTCCTCTATTGTTGATGTTTTGTGTATCTTTGATATTTGCCCTTAACTTTTTAATTTAATTTCTTATTTTCCAGGG

ATGTTGCTTTTTTGCCCTTT

In100 candidate3533 (in intron):

LOCUS ENSG00000144535

DESCRIPTION DIS3 mitotic control homolog (S. cerevisiae)-like 2

[Source:Refeq_peptide;Acc:NP_689596]

ACCTTCTTGAAAATATTATGATGTTAGAGGACCTTAGATAACCATCAACCTCCTCTTATACTCATTAAATCCTTTCAGCT

TCCTCTTTGCTTTCTTCATA

In100 candidate3534 (in intron):

LOCUS ENSG00000144535

DESCRIPTION DIS3 mitotic control homolog (S. cerevisiae)-like 2

[Source:Refeq_peptide;Acc:NP_689596]

TTGTTGTTTTAAAACTTTGAGCTAGTTTTACTTAACTGGATTTGTCCTTTTCAAACTTTTTTCTTTTCTTGGCTTCAGTC

CTGCCTCTTCTCTTTTTTTT

In100 candidate3535 (in intron):

LOCUS ENSG00000144535

DESCRIPTION DIS3 mitotic control homolog (S. cerevisiae)-like 2

[Source:Refeq_peptide;Acc:NP_689596]

GAGTCCCCTTTCTTAAAGCTCTTTTCTCCTGCTTTTCCTAACATCGGTCACTCCTGGTTTTTGTCCTGTCTCTCTCAGCT

CTCTTGTTTTTTCTACTGTT

In100 candidate3536 (in intron):

LOCUS ENSG00000131725

DESCRIPTION WD repeat protein 44 (Rabphilin-11).

[Source:Uniprot/SWISSPROT;cc:Q5JSH3]

TTACTAAATGCCTGTTTAACTGCTGTCTGTTTTCTTTTTCTTTCCTTCTCAGAATCACATCCAGCTTCTGTCTTCTAACT

TAAGTCATTTACGTTTTTTC

In100 candidate3537 (in intron):

LOCUS ENSG00000108242

DESCRIPTION Cytochrome P450 2C18 (EC 1.14.14.1) (CYPIIC18) (P450-6B/29C).

[ource:Uniprot/SWISSPROT;Acc:P33260]

AGTATGTTGAAAACTAGTTGAGAAGTTTTTGCTCCTGTAACAATTTACAAAACACGTTCATTTTGGTCTCTTTTGCAGTT

TTCTTATTGTATTCATTTTT

In100 candidate3538 (in intron):

LOCUS ENSG00000165841

DESCRIPTION Cytochrome P450 2C19 (EC 1.14.13.80) ((R)-limonene 6-monooxygense) (EC 1.14.13.48) ((S)-limonene 6-monooxygenase) (EC 1.14.13.49) ((S)- lionene 7-monooxygenase) (CYPIIC19) (P450-11A) (Mephenytoin 4-hydroxylase) (YPIIC17) (P450-254C). [Source:Uniprot/SWISSPROT;Acc:P33261]

AGCTCCTGGATTCATTAACTTTTTGAAGGGTTTTCTGTGTCTATCTCCTCCAGTTCTGCTCTGATCTTAGTTATTTCTTG
TCTTCTGCTAGCTTTTGAAT

In100 candidate3539 (in intron):

LOCUS ENSG00000119397

DESCRIPTION centrosomal protein 110kDa [Source:RefSeq_peptide;Acc:NP_008949

AGTTTAACTCTCCAAGTGACGGTATCTGTTTGCTTCTTCTTTTAGCCCTTGTCTTGGTTTCTTGCAGGCCCTTTTCTGCT
GATTCTTTATTCTTCCTTAA

In100 candidate3540 (in intron):

LOCUS ENSG00000119397

DESCRIPTION centrosomal protein 110kDa [Source:RefSeq_peptide;Acc:NP_008949

TTTTAGAGGGTCTGTGTGCATTTACAAGTTTAACTGTAGTAGTCACTCACATTCTTTTGCAGTTTTTCTTGCACATTGTA
ATTTGTAACAATTTCTTTCT

In100 candidate3541 (in intron):

LOCUS ENSG00000138109

DESCRIPTION Cytochrome P450 2C9 (EC 1.14.13.80) ((R)-limonene 6-monooxygenae) (EC 1.14.13.48) ((S)-limonene 6-monooxygenase) (EC 1.14.13.49) ((S)- limnene 7-monooxygenase) (CYPIIC9) (P450 PB-1) (P450 MP-4/MP-8) (S- mephenytoi 4-hydroxylase) (P-450MP). [Source:Uniprot/SWISSPROT;Acc:P11712]

TATGGTTTTTGTTTTGAAATCTACTTTGTCTGTTTTAAATATAGTAACTCATGCTCTTTTTTTCATTTCCATTGGCAGGT

ACTGTCTCATTCAATTCCTT

In100 candidate3542 (in intron):

LOCUS ENSG00000147251

DESCRIPTION dedicator of cytokinesis 11 [Source:RefSeq_peptide;Acc:NP_65325]

GTGCCATGGTAAATTTGGGTACTGAATGCCTGAGACTATTGTTCTGCTAACTTCCTGCTGCTATCTTTTCTTTATCAGTT

CACATCTTTATTATTTATTT

In100 candidate3543 (in intron):

LOCUS ENSG00000135930

DESCRIPTION Eukaryotic translation initiation factor 4E type 2 (eIF4E type)

(eIF-4E type 2) (mRNA cap-binding protein type 3) (Eukaryotic

translationinitiation factor 4E-like 3) (Eukaryotic translation initiation

factor 4E hmologous protein) (mRNA cap-binding pro

[Source:Uniprot/SWISSPROT;Acc:O6057]

AGAATTCCTTGTTGCCTATATTAACCTCTAATTAATTTTTTCTGTTTTTAGAAACTCCAGTCATGACACTTCTTACATTT

AACTTGTTCTTTCTTTACCT

In100 candidate3544 (in intron):

LOCUS ENSG00000115468

DESCRIPTION EF-hand domain-containing protein 1 (Swiprosin-2).

[Source:Unipot/SWISSPROT;Acc:Q9BUP0]

AGTTTGAAACTAACTATGCACATTTTATATTTTTCCCTTCCAGAATTCTACCTCTTAAGACTTCTTCTTTTTTTTTTTTT

TTTTTTGAGATTGAGTCTCA

In100 candidate3545 (in intron):

LOCUS ENSG00000204120

DESCRIPTION PERQ amino acid-rich with GYF domain-containing protein 2 (Grb1-interacting GYF protein 2) (Trinucleotide repeat-containing protein 15). Source:Uniprot/SWISSPROT;Acc:Q6Y7W6]

TCTAAAATTCCTTACTTAGAGCCTTTCTGTATAGACTGTAGCTAACAAAATTCATCATTCTCATTTGGGCTTTTTCAGGA

CTGTTGTTACTTCTTACCTA

In100 candidate3546 (in intron):

LOCUS ENSG00000204120

DESCRIPTION PERQ amino acid-rich with GYF domain-containing protein 2 (Grb1-interacting GYF protein 2) (Trinucleotide repeat-containing protein 15). Source:Uniprot/SWISSPROT;Acc:Q6Y7W6]

CTGAAGTCAACTTTATCAAACATGAATATAACTTACTTTAAGTTAATATTTGCATGATATATCTTTTTCTCTCCTCAGTC

TGCCTACTTTGTTATATTTT

In100 candidate3547 (in intron):

LOCUS ENSG00000204120

DESCRIPTION PERQ amino acid-rich with GYF domain-containing protein 2 (Grb1-interacting GYF protein 2) (Trinucleotide repeat-containing protein 15). Source:Uniprot/SWISSPROT;Acc:Q6Y7W6]

CCTTATATACCATTATAAAATTCTGTAACATTTCCTTTGACCTTTAGATACTTTTACATTTTTCTGTGTTATATCCAGTT

TCTCTAATTTCTACATTATC

In100 candidate3548 (in intron):

LOCUS ENSG00000204120

DESCRIPTION PERQ amino acid-rich with GYF domain-containing protein 2 (Grb1-interacting GYF protein 2) (Trinucleotide repeat-containing protein 15). Source:Uniprot/SWISSPROT;Acc:Q6Y7W6]

AATGAGAATATGTCTGTTTGCTAAAAGCCTAGACTGATAATTAACATATTAATCAACTTTTTTTGGCCACAGACTTTTCC

CTTCCTGTCTTTGCATGAGT

In100 candidate3549 (in intron):

LOCUS ENSG00000066248

DESCRIPTION Ephexin-1 (Eph-interacting exchange protein) (Neuronal guanine ucleotide exchange factor). [Source:Uniprot/SWISSPROT;Acc:Q8N5V2]

CCACCACGTCTGGCCACATTTACAATTTTTAACATGTCCATGTACTGCCTGTCTTATTAATATTTTCTACAGATTGCCTT

TAAATTAAATTTCTTACTTC

In100 candidate3550 (in intron):

LOCUS ENSG00000131724

DESCRIPTION Interleukin-13 receptor alpha-1 chain precursor (IL-13R-alpha-1 (IL- 13RA-1) (CD213a1 antigen). [Source:Uniprot/SWISSPROT;Acc:P78552]

AAGGAGCTTTTTAAACATGGGGATAACATCCAAATACTGCTGATTGTTATTTTTACTTCAGTGTGTATCTTTTTTTTTT

CCTTTTTGAGATGGAGTCTC

In100 candidate3551 (in intron):

LOCUS ENSG00000087495

DESCRIPTION Phosphatase and actin regulator 3 (Scapinin) (Scaffold-associatd PP1- inhibiting protein). [Source:Uniprot/SWISSPROT;Acc:Q96KR7]

GCATGGTCTTGGCACATGTAACATGCCCCGTTTCTCTCTTTCTTCTGGGGTTCAGGCATATTTCTGGTCCATGATAAGAT
CTTTGCCTCCTTCCTGTCTT

In100 candidate3552 (in intron):

LOCUS ENSG00000077044

DESCRIPTION Diacylglycerol kinase delta (EC 2.7.1.107) (Diglyceride kinase elta) (DGK-delta) (DAG kinase delta) (130 kDa diacylglycerol kinase). [Soure:Uniprot/SWISSPROT;Acc:Q16760]

AAAGCAAGTTATTGAATAATTTGTACATCATGTAACTCCCCTTTATGTCTTTTAGTTTAATTTTTTTTTTTACACAGGG
TTTTTTTTGTTTTTGTTTGT

In100 candidate3553 (in intron):

LOCUS ENSG00000077044

DESCRIPTION Diacylglycerol kinase delta (EC 2.7.1.107) (Diglyceride kinase elta) (DGK-delta) (DAG kinase delta) (130 kDa diacylglycerol kinase). [Soure:Uniprot/SWISSPROT;Acc:Q16760]

ATTCCATTCCTCATTTAATTACCTTTGCTGCCTTTTTTCAAAAGTCAATTAACCTTTTATTTTTGAGTCTCTTTCCAGAT
TTTCTATTTTGTTTTATTGA

In100 candidate3554 (in intron):

LOCUS ENSG00000085982

DESCRIPTION Ubiquitin carboxyl-terminal hydrolase 40 (EC 3.1.2.15) (Ubiquitn

thioesterase 40) (Ubiquitin-specific-processing protease 40) (Deubiquitinting enzyme 40). [Source:Uniprot/SWISSPROT;Acc:Q9NVE5]

ATTTTTTAATCACATCTAATTTTTGTTTAAATAATTAACTTAGCATTTTTTTTTTTGTTTCTGTGTAGTTCTTACAGCTC

CATTCTCAGATATTTCTACT

In100 candidate3555 (in intron):

LOCUS ENSG00000119977

DESCRIPTION Tectonic-3 precursor. [Source:Uniprot/SWISSPROT;Acc:Q6NUS6]

TCCTAAAAAAGATTCTCAACTTTATCTTTTAACTTTTTTACTGAATTTTTTATTTTGGCCATCAGATTTTTAATACCTAA

GGTCTGTCTTATTCTGTTTC

In100 candidate3556 (in intron):

LOCUS ENSG00000119977

DESCRIPTION Tectonic-3 precursor. [Source:Uniprot/SWISSPROT;Acc:Q6NUS6]

CATAATATCTTTTTTCATGTATCTCTGAAGATACTAACAACTTTGCATCTTCATCTTTCATTGTCTGTGTTTCCTCAGAG

TTGTTTATTCTGTTCATTTG

In100 candidate3557 (in intron):

LOCUS ENSG00000167165

DESCRIPTION UDP-glucuronosyltransferase 1-8 precursor (EC 2.4.1.17)

(UDPgucuronosyltransferase

1A8) (UDPGT) (UGT1*8) (UGT1-08) (UGT1.8) (UGT- 1H)

(UT1H). [Source:Uniprot/SWISSPROT;Acc:Q9HAW9]

GGGACAAATATCCAAGCTATAACACTGAACTGTGATTTGACATTTTCATTTGTCTCATTTGGAATTTTTTTCCAGTTTCA

TGAATCCTTTTGTGCCACTT

In100 candidate3558 (in intron):

LOCUS ENSG00000167165

DESCRIPTION UDP-glucuronosyltransferase 1-8 precursor (EC 2.4.1.17)

(UDPgucuronosyltransferase

1A8) (UDPGT) (UGT1*8) (UGT1-08) (UGT1.8) (UGT- 1H)

(UT1H). [Source:Uniprot/SWISSPROT;Acc:Q9HAW9]

GATTGGTCTTCTCAAAGAAATAACTGTGGAATTTGTATATTCTTTTCACTGTTTCATTTTCCGTTTCGTTAATTGCAGCT

TATTTTTGCCTTAATGATTT

In100 candidate3559 (in intron):

LOCUS ENSG00000077713

DESCRIPTION mitochondrial solute carrier protein

[Source:RefSeq_peptide;AccNP_660348]

TTTTCTATTCGTTGGTAAGAAGTCTTTATATATTAAAGATATTAACCCTTTGTCATACATTTCACACAATTTTTCCAGTT

TGACATTTGGTGTTTTTTTT

In100 candidate3560 (in intron):

LOCUS ENSG00000124215

DESCRIPTION Cadherin-like protein 26 precursor (Cadherin-like protein VR20)

[Source:Uniprot/SWISSPROT;Acc:Q8IXH8]

TTGGTGTGTTGTTGGTCTTTGATAACTATGTGCTTGAACATTTTGCTATGTTTCTTCACCATTTCCATGTTCTATCAGTT

GCTTTTTCCTTTCTTTTCTT

In100 candidate3561 (in intron):

LOCUS ENSG00000173088

DESCRIPTION Novel protein. [Source:Uniprot/SPTREMBL;Acc:Q5VUS1]

TATATTCTATTGACCTGTCTTAACCATTCACTTATATAATTTTCTGCCATATTTTATCTCCAGTTAAATCCACCCAATGA

GTTCTTTCCCTTTATTTCTT

In100 candidate3562 (in intron):

LOCUS ENSG00000175764

DESCRIPTION Tubulin--tyrosine ligase-like protein 11.

[Source:Uniprot/SWISSROT;Acc:Q8NHH1]

CTTCCGATATTCTCATAACATATATTTTGTACTTTTTGTCATTGTCCCACTGTTCCTGGATAGTCTCCTTTTTTTCAGTA

TTTTTTTTCTTTAATTCATT

In100 candidate3563 (in intron):

LOCUS ENSG00000106852

DESCRIPTION LIM/homeobox protein Lhx6.1 (Lhx6).

[Source:Uniprot/SWISSPROT;Ac:Q9UPM6]

AAGACGGTAAGCAAAAAGAAGAAAATAACGATTGCTCCAAATGCTGCCTTCTTCATATTTTAATGTGTATCTTCTCAGAT

TTTTTTCCATGTATGTTACA

In100 candidate3564 (in intron):

LOCUS ENSG00000064999

DESCRIPTION Ankyrin repeat and SAM domain-containing protein 1A (Odin).

[Sorce:Uniprot/SWISSPROT;Acc:Q92625]

CTCTATAACTGGCCTTCTCTTTCATCTCATACCCTATTTTTCCAGCTCAGTTAATTCCTATACTCACTGTTTTCCCCTAC

ACGTTGGTTTATACTATTCT

In100 candidate3565 (in intron):

LOCUS ENSG00000130699

DESCRIPTION Transcription initiation factor TFIID subunit 4 (TBPassociatedfactor

4) (Transcription initiation factor TFIID 135 kDa subunit)

(TAF(II)15) (TAFII-135) (TAFII135) (TAFII-130) (TAFII130).

[Source:Uniprot/SWISSPROTAcc:O00268]

AGTTGAAGTTTCCATTTTGTTAACTTATTTTGCAATTCTAAGATTTCCATCTAGTTCTTTTTTACAGTTTATATTTCCCC

TAATTCTTTGAGCTTATTAT

In100 candidate3566 (in intron):

LOCUS ENSG00000112033

DESCRIPTION Peroxisome proliferator-activated receptor delta (PPAR-delta)

(PAR- beta) (Nuclear hormone receptor 1) (NUC1) (NUCI).

[Source:Uniprot/SWISPROT;Acc:Q03181]

TATTTTTTCTGTCTTTCGAAATGGGATGCTGTTTAACATTTAGTTTTGTGACTTGCCTTTCCCATTTTTCAGGTGGTTTT

GTGGTGTCTGCTTGTCCTCC

In100 candidate3567 (in intron):

LOCUS ENSG00000157985

DESCRIPTION Centaurin-gamma 2 (ARF-GAP with GTP-binding protein-like, ankyrn repeat and pleckstrin homology domains 1) (AGAP-1) (GTP-binding and GTPasactivating
protein 1) (GGAP1). [Source:Uniprot/SWISSPROT;Acc:Q9UPQ3]

AAATAGCTGTCTACTCTTACATCTCATGAAAAAAAAATGGCTGTGAATTTAACCTACGGATTTTGTTTTTTCTTGCAGTT
TCTGACTTGTACCTTTTCAT

In100 candidate3568 (in intron):

LOCUS ENSG00000196233

DESCRIPTION Ligand-dependent corepressor (LCoR) (Mblk1-related protein 2).
Source:Uniprot/SWISSPROT;Acc:Q96JN0]

TTGAATAAGCCTGCTTAACTGCTTTTTCATTTTTAGCCTATTTAGTTTACATTTGCTCCTAGCTTTTAATATCCTCAGGT
GTTTCTTTGTGTCTCTGCAG

In100 candidate3569 (in intron):

LOCUS ENSG00000187122

DESCRIPTION Slit homolog 1 protein precursor (Slit-1) (Multiple epidermal gowth factor-like domains 4). [Source:Uniprot/SWISSPROT;Acc:O75093]

TCTTGCTATGTTCCTGCTTTATCATTTTTCCTAAAATTCTTCTTAACTATTACTAGTTCTGTCCTCTACTGTTATCAGTC
CATCTCTTTCAGTCTGGTTT

In100 candidate3570 (in intron):

LOCUS ENSG00000187122

DESCRIPTION Slit homolog 1 protein precursor (Slit-1) (Multiple epidermal gowth factor-like domains 4). [Source:Uniprot/SWISSPROT;Acc:O75093]

TTTTGGGTGTTCCTTTAACACGATTATTTTTCCCAAATGAATTTTTTTCTTCCTCTTGAGTGTTCTCTCTTTCCAGCT

TCCTGCTCTTTGAAACTGTT

In100 candidate3571 (in intron):

LOCUS ENSG00000186130

DESCRIPTION Zinc finger and BTB domain-containing protein 6 (Zinc finger prtein 482) (Zinc finger protein with interaction domain). [Source:Uniprot/SWSSPROT;Acc:Q15916]

AAGTTCTCGTTTTGAGATGGTGGCATAAAGGTTCATAACACCTACATTAGACTTTGAGTCTTCTTCTTCCTAATTCAGGT

TTGGGGTTTTTCGTTTGTTT

In100 candidate3572 (in intron):

LOCUS ENSG00000124831

DESCRIPTION leucine rich repeat (in FLII) interacting protein 1 [Source:Refeq_peptide;Acc:NP_004726]

TGAGCTGTTGAGAAAGTTGTTGTTTTAACGGTCGGTCCTTTGTAGGGACCTTTTTTTTTCTTCTCTAGTTTCTTTCAGGT

TCTGTTTGTCTTTGATTTTC

In100 candidate3573 (in intron):

LOCUS ENSG00000011454

DESCRIPTION RAB GTPase activating protein 1 [Source:RefSeq_peptide;Acc:NP_06329]

TTCTAGCCTTAGCTGTGCCATTAACTGGTTAAATGACCTTACTTCTCTTGGGGTTGCCATTTCTTTATCTAACAGTATTG

ATTTGTAAGTTTTTTTTTC

In100 candidate3574 (in intron):

LOCUS ENSG00000132329

DESCRIPTION Receptor activity-modifying protein 1 precursor (CRLR

activitymodifying

protein 1) (Calcitonin-receptor-like receptor activitymodifyingprotein

1). [Source:Uniprot/SWISSPROT;Acc:O60894]

GCTTTAAGAAGTATGCTTGGAGCTCATCTAACATCTTATTTTCCATTTTCTGTTTCTTAATACTTACAGAGGCTTCCGAC

TTTTTCTCCAGGTCTGTATT

In100 candidate3575 (in intron):

LOCUS ENSG00000132329

DESCRIPTION Receptor activity-modifying protein 1 precursor (CRLR

activitymodifying

protein 1) (Calcitonin-receptor-like receptor activitymodifyingprotein

1). [Source:Uniprot/SWISSPROT;Acc:O60894]

GCCTTTCAACTACTTGAAGTTTACTGGTAACTCTTTTTAGTCTTCCTATTTTGTTTTAATGACCTCTCTTGCCCACAGCT

TATTGCTGGATTTCATTTTT

In100 candidate3576 (in intron):

LOCUS ENSG00000005893

DESCRIPTION Lysosome-associated membrane glycoprotein 2 precursor (LAMP-2)

CD107b antigen). [Source:Uniprot/SWISSPROT;Acc:P13473]

TTCTTCTGTGGTTATTGATTCTTATAACTAGCTTGACTTCTGTTTTTTGGTTTGTTTGTTTGTTTTTTTAGGGTTCAGAA

TTTTTTTTTTTTAGGTTTGA

In100 candidate3577 (in intron):

LOCUS ENSG00000005893

DESCRIPTION Lysosome-associated membrane glycoprotein 2 precursor (LAMP-2)

CD107b antigen). [Source:Uniprot/SWISSPROT;Acc:P13473]

ATGTCTTACGCTTTTGGAATTCAATCTCTTAACTTGTAGGAGAACTTGTACCATTCAGCATCTTCTGTCAATTGCTAATG

GCTGTTTCCCTCATCCTATT

In100 candidate3578 (in intron):

LOCUS ENSG00000165209

DESCRIPTION Spermatid perinuclear RNA-binding protein.

[Source:Uniprot/SWISPROT;Acc:Q96SI9]

CAATAGCTCTCTGGGATAACTTTTTTTTTTTTTTTTTTTTTTTTTTTTTTTGCTGAACTTGTCTCTTCATCCAGATTCT

ACTTAGATTTTCAGTTGTCT

In100 candidate3579 (in intron):

LOCUS ENSG00000165209

DESCRIPTION Spermatid perinuclear RNA-binding protein.

[Source:Uniprot/SWISPROT;Acc:Q96SI9]

TAAAAAAGTATGTCTGTATCTCTCTCCATAAATGATATGTAACATTATCTTATGTTTTTTCAAGCTTATATTCAGGGCAT

CACTTTGTTCATGCTATTCT

In100 candidate3580 (in intron):

LOCUS ENSG00000065802

DESCRIPTION Ankyrin repeat and SOCS box protein 1 (ASB-1).

[Source:Uniprot/WISSPROT;Acc:Q9Y576]

TGTAACTGTTTCCATTTGATATCTTTTCTTCTTATATGTTGTTTTTTTCTTTCCTCTTCCATTATACTAGGTTTACAGTC

TTATTTTATTTTATTTTTTT

In100 candidate3581 (in intron):

LOCUS ENSG00000065802

DESCRIPTION Ankyrin repeat and SOCS box protein 1 (ASB-1).

[Source:Uniprot/WISSPROT;Acc:Q9Y576]

TTAATCATACTAACGGTTGCCTTTTTTTTTTTCTTTTTCAACCAGATTGTTTTTATTTTTCATGTTTCTAGTGACTTCTT

TCCAGCAAAGATGCTTTTTT

In100 candidate3582 (in intron):

LOCUS ENSG00000068024

DESCRIPTION Histone deacetylase 4 (HD4). [Source:Uniprot/SWISSPROT;Acc:P5654]

GGAATGGAATATTAAAATCCCCCTGATGTAACTGTGGATTGGCATTGTTTCTCTTTTTAGCTCTGTCAGGTTTAGCCTTT

GTTTATTTTGAAGCTATTTT

In100 candidate3583 (in intron):

LOCUS ENSG00000112062

DESCRIPTION Mitogen-activated protein kinase 14 (EC 2.7.11.24) (Mitogenactvated

protein kinase p38 alpha) (MAP kinase p38 alpha) (Cytokine suppressiv

anti-inflammatory drug-binding protein) (CSAID-binding protein) (CSBP) (MAinteracting

protein 2) (MAP kinase MXI [Source:Uniprot/SWISSPROT;Acc:Q1653]

CACAATGTATACATCTTAACATTTATTGTGCTCTTTTTTTTTTTTTAAACACCACCTTAAAGTACTCTGCTATTCAGAT

TTTTCCTTGTGTTTCTCTAA

In100 candidate3584 (in intron):

LOCUS ENSG00000119522

DESCRIPTION DENN domain-containing protein 1A.

[Source:Uniprot/SWISSPROT;Ac:Q8TEH3]

CATATTACGTTAATATAAATTCATGTATTAGAATTTGGATTTTTTAACCAAAAATACCTTTATTTTAATCTTCAGAGAAA

AAACTCTTTTCTTCTTTTTC

In100 candidate3585 (in intron):

LOCUS ENSG00000119522

DESCRIPTION DENN domain-containing protein 1A.

[Source:Uniprot/SWISSPROT;Ac:Q8TEH3]

AGAAATTATGAAAGATATAGGAATTTAGGAAAGTAACACCTAAGCTTGCCCTGTTTTCTTTCCAGTTTCAATTTCAGCTC

CTCCTCTTTGTAGCTGTGTG

In100 candidate3586 (in intron):

LOCUS ENSG00000119522

DESCRIPTION DENN domain-containing protein 1A.

[Source:Uniprot/SWISSPROT;Ac:Q8TEH3]

ACTTTTCTTGAATTAACTTTGGTTCTGATTATTACTTTTTCTTCTTCAGGCACTCCAGTTAGCCGTATATTCAATCTTCT

TTGCCTATTTTCAATATTTT

In100 candidate3587 (in intron):

LOCUS ENSG00000148200

DESCRIPTION Orphan nuclear receptor NR6A1 (Germ cell nuclear factor) (GCNF)(Retinoid receptor-related testis-specific receptor) (RTR). [Source:UniprotSWISSPROT;Acc:Q15406]

CAAAGTATGCCTTTTTGAGATAACCTTTCCTTTCATTCCATTCCTGTTGACTCAGTTCTACCCCTGCTAACTTGTGCAAG
AAAGTTCTCTTTTTTCTCTT

In100 candidate3588 (in intron):

LOCUS ENSG00000148200

DESCRIPTION Orphan nuclear receptor NR6A1 (Germ cell nuclear factor) (GCNF)(Retinoid receptor-related testis-specific receptor) (RTR). [Source:UniprotSWISSPROT;Acc:Q15406]

ATTCCTGGCCTCTTCTTTTCAAAGTTCCTAACACATACTTTCTTTTCCTTCTTTTCTTTTTTTTTTTTTTGAGACAGAT
TTTCACTCTTTTTGCCCAGG

In100 candidate3589 (in intron):

LOCUS ENSG00000112081

DESCRIPTION Splicing factor, arginine/serine-rich 3 (Pre-mRNA-splicing factr SRP20). [Source:Uniprot/SWISSPROT;Acc:P84103]

AGTAACTTCTATCTTGGATCTGTCTTCTAGTTCATCTTTCTAGTTGCATCTTTCCAAATCTTCCCTTATACTTCAGTTTA
GGCCTTTTTCCATTTCTTGG

In100 candidate3590 (in intron):

LOCUS ENSG00000136935

DESCRIPTION Golgin subfamily A member 1 (Golgin-97).

[Source:Uniprot/SWISSPOT;Acc:Q92805]

AGGCTAATTGGGAAAAATTGTCCATTTTATAATATTAATTATTCCTTCTAACAATGTAAATATATTCCATTTATTCAGAT

TATTTTCTGTGTGTTTTATT

In100 candidate3591 (in intron):

LOCUS ENSG00000115685

DESCRIPTION Protein phosphatase 1 regulatory subunit 7 (Protein phosphatase1

regulatory subunit 22). [Source:Uniprot/SWISSPROT;Acc:Q15435]

GTCTAACCCTGGCGCCTCTGTTTTCAACCACTTTGTCCTTTTGTTCCCTCTTGATACTTTCTGCTAGCTTTAGTCCAGTG

CATTTTCCCTTTTATTATTA

In100 candidate3592 (in intron):

LOCUS ENSG00000173611

DESCRIPTION Uncharacterized protein C9orf126.

[Source:Uniprot/SWISSPROT;AccQ8N9R8]

TGAGTATCTTTTCATGTGCTAATTTGCCATTCATATAACTTAGTGAAGTATCCAAATCTTTTGCTCACTTTTTGTCAGTT

GTTTGTCTTCTTACATAGTT

In100 candidate3593 (in intron):

LOCUS ENSG00000173611

DESCRIPTION Uncharacterized protein C9orf126.

[Source:Uniprot/SWISSPROT;AccQ8N9R8]

AAAAATCTGAGCTCATAACAAAATGATCCCTTTCTTAGCATGTGATTTCCATAAATGCTCTCCTTTCTTTGTCAGAATAA

ATTTTTTTGTTTTTTAATTT

In100 candidate3594 (in intron):

LOCUS ENSG00000125676

DESCRIPTION THO complex subunit 2 (Tho2).

[Source:Uniprot/SWISSPROT;Acc:Q8N27]

GAACAAAAACTTGACTTAGTTTAACCAAAAAAACTAACCATATTTCTTTTTAATTCTTGTACCTTTGGTTTCTAGCAGCC

AGTTCCTTTTCTTTCAACTT

In100 candidate3595 (in intron):

LOCUS ENSG00000115677

DESCRIPTION Vigilin (High density lipoprotein-binding protein)

(HDLbindingprotein).

[Source:Uniprot/SWISSPROT;Acc:Q00341]

CTGTGTTAATGATAATGGTTTCATGTTTAATGTCTAGGACCACTGTTAACACCTCCACCCTGCACTATTCTTTTTCAGGG

TTTTCTTGTTCATTTTTGTT

In100 candidate3596 (in intron):

LOCUS ENSG00000101972

DESCRIPTION Cohesin subunit SA-2 (Stromal antigen 2) (SCC3 homolog 2).

[Souce:Uniprot/SWISSPROT;Acc:Q8N3U4]

TTTCCTGAGGGCCACCTAAGTTAAAACAAAACTCTAAGATAACGACATCTGAGTTTGTTTTCCGTATGTATTATTCAGGT

TCCCTTTACCATTTTCTTAG

In100 candidate3597 (in intron):

LOCUS ENSG00000101972

DESCRIPTION Cohesin subunit SA-2 (Stromal antigen 2) (SCC3 homolog 2).

[Souce:Uniprot/SWISSPROT;Acc:Q8N3U4]

GGCGTGAGCCACTGCGCCTGACCGAATATGGAAATTCTAACTGAGCTAGGAATTCATGTGTTCCCATTCTCTTGGCAGAT

TGATTTCCTTCCTGTTTTTC

In100 candidate3598 (in intron):

LOCUS ENSG00000119487

DESCRIPTION Stress-activated map kinase-interacting protein 1 (SAPKinteracing

protein 1) (Putative Ras inhibitor JC310).

[Source:Uniprot/SWISSPROT;Ac:Q9BPZ7]

AGTTATGTCACCTTTTGCTTTCATTTGTAACCATACTGTCCATTGTAGACATGTACTTCTTATACTTCTCTCCTTCAGTT

TCCTTCTCTCTCTTTTCTGT

In100 candidate3599 (in intron):

LOCUS ENSG00000119487

DESCRIPTION Stress-activated map kinase-interacting protein 1 (SAPKinteracing

protein 1) (Putative Ras inhibitor JC310).

[Source:Uniprot/SWISSPROT;Ac:Q9BPZ7]

AAGAGATGACATTGGCATTAAAATTGAGAAGAGATGGCTTAGATTGTAACAACTGGTAGTGTTTTTTTACACACACAGAC

TTCTTGTTGATTTTCAGTAC

In100 candidate3600 (in intron):

LOCUS ENSG00000009694

DESCRIPTION Teneurin-1 (Ten-1) (Tenascin-M1) (Ten-m1) (Protein Odd

Oz/tenmhomolog

1). [Source:Uniprot/SWISSPROT;Acc:Q9UKZ4]

AGTTGGATTATTTCCTTATATAATTTTTAACTTTAGGTTGTGATCTTATTTGCATTTGCCTCTGCCAGGCTCTCTAATCC

TTTGTATTAATGTTAATTTC

In100 candidate3601 (in intron):

LOCUS ENSG00000009694

DESCRIPTION Teneurin-1 (Ten-1) (Tenascin-M1) (Ten-m1) (Protein Odd

Oz/tenmhomolog

1). [Source:Uniprot/SWISSPROT;Acc:Q9UKZ4]

GGTCTTGGGTTTCTCATCTGTAAAATGGCGAGTGGAGAGAGGCAAGCTTAACTAGGTGATCCCTATGATTTCTTCCAGCC

TTATAGTTTTATACTTTTAT

In100 candidate3602 (in intron):

LOCUS ENSG00000196814

DESCRIPTION Protein FAM125B. [Source:Uniprot/SWISSPROT;Acc:Q9H7P6]

TATATAAATTTTATAACGTGCTTATTATTGCATCATATTTTCTTATTGTATTTTTGCAGTATTTTCTTAGATCATCGGAT

ATTCTTCTATAGTCCTTCTT

In100 candidate3603 (in intron):

LOCUS ENSG00000165886

DESCRIPTION Ubiquitin domain-containing protein 1.

[Source:Uniprot/SWISSPRO;Acc:Q9HAC8]

TTGTTTAGTCTAAATTAACTTAGAATTGGTATAGATGGCCTTTTTTTCTCTCTGCTGGTCTTTACTTGCCTCTGCCAGCT

GCTTATGCTGCTGTTATTTT

In100 candidate3604 (in intron):

LOCUS ENSG00000136828

DESCRIPTION Ral GEF with PH domain and SH3 binding motif 1

[Source:RefSeq_pptide;Acc:NP_055451]

AGGTTTGATATTTAGCTTTTTTTAGTTTGATGGTTTGTTTTTTAACATGCCAATTTTGTCATTGATTTCTTTCTCCAGTT

TTCCCACTATTTTCTTAGCT

In100 candidate3605 (in intron):

LOCUS ENSG00000122126

DESCRIPTION Inositol polyphosphate 5-phosphatase OCRL-1 (EC 3.1.3.36)

(Loweoculocerebrorenal syndrome protein). [Source:Uniprot/SWISSPROT;Acc:Q01968]

EXON

CTATATTTATTTTCTTTGTAATATCTTTTATTATAAGATAACCACTGACTATCCACTTGTTTCTCATTTCCTTTCAGGAA

CTTTTGACCTCTTTCCATAT

In100 candidate3606 (in intron):

LOCUS ENSG00000136895

DESCRIPTION GTPase activating Rap/RanGAP domain-like 3

[Source:RefSeq_peptie;Acc:NP_115669]

AACATTAGTTCCTTCCTCTGCTATTCTGTAACTCAACATCCTATTTGTTTTCTTAGCAGTGCTTATCACAATCTATATTC

ATTTCATTTACCACTTACTT

In100 candidate3607 (in intron):

LOCUS ENSG00000136895

DESCRIPTION GTPase activating Rap/RanGAP domain-like 3

[Source:RefSeq_peptie;Acc:NP_115669]

CTTAAAGGACTTTATAACATGCATCCTCATCTTATTCTCTTGTTTTCAGTTTATTTACCTACACTTGATTTTTCCTTGTT

CTGAGTGTGCTCAGTATCTC

In100 candidate3608 (in intron):

LOCUS ENSG00000095713

DESCRIPTION Cartilage acidic protein 1 precursor (Chondrocyte expressed

proein 68 kDa) (CEP-68) (ASPIC). [Source:Uniprot/SWISSPROT;Acc:Q9NQ79]

AGTTTCTTAACTGTTCTCACATTATTTCATCTTCTGTCTCTCTGTGCTGCTGCATTCTGGATAATTTTCCAGTTATATCT

TCCAGTTCACTAATTATTTC

In100 candidate3609 (in intron):

LOCUS ENSG00000156639

DESCRIPTION AN1-type zinc finger protein 3 (Testis-expressed sequence 27).

Source:Uniprot/SWISSPROT;Acc:Q9H8U3]

TGCTGAACTAAAATTAGGTCCTTAGAATTAACCCACCACCACCGTCTTTCCTACTACTTCTCCTCGTTATATTTTCAGGT

TGATCTTTTCCTTTTTAAAG

In100 candidate3610 (in intron):

LOCUS ENSG00000183826

DESCRIPTION BTB/POZ domain-containing protein 9.

[Source:Uniprot/SWISSPROT;cc:Q96Q07]

CGATTCATGTGCCAATACTACATTATTTATTTATAGAGGCTTTATAACATGTTTTAATATCTGCTCTTCTTTTTCAGGG

TTTTCTTGGCTATTCTTGCA

In100 candidate3611 (in intron):

LOCUS ENSG00000183826

DESCRIPTION BTB/POZ domain-containing protein 9.

[Source:Uniprot/SWISSPROT;cc:Q96Q07]

TAATGTCTGTTTTTTCTGACATTATTGTAACTTTTGCATCTACACTTGTTATGTCTTTTTCCATACCTTTAGTTTCAGGC

TTCCTTTGCCCTTATATTTT

In100 candidate3612 (in intron):

LOCUS ENSG00000183826

DESCRIPTION BTB/POZ domain-containing protein 9.

[Source:Uniprot/SWISSPROT;cc:Q96Q07]

TCATGCTGAGAGAATTGTGATATGTAACCTAATTTCACCATTTTCTTAATTTCTTCCCATTACTCCCAGTAATATGCCTC

TCAAACCATTTTATTTTCTT

In100 candidate3613 (in intron):

LOCUS ENSG00000166024

DESCRIPTION Growth inhibition and differentiation-related protein 88 (Putatve

mitochondrial space protein 32.1). [Source:Uniprot/SWISSPROT;Acc:Q7Z5L2]EXON

TAATCTCACAATCTAAACATAACATAATGAGGCCCACATTTTTCATGTTTTTTTTTCCCAGTCTTCTTTTTCTAGGCAT

TTACTAAAGCCCATCTACTT

In100 candidate3614 (in intron):

LOCUS ENSG00000124721

DESCRIPTION Ciliary dynein heavy chain 8 (Axonemal beta dynein heavy chain).

[Source:Uniprot/SWISSPROT;Acc:Q96JB1]

AGCCACTGTTTGTCATTTTCTTGTAATCATCATAACTTTTGTACATCTACTCTTTGTTCTTCTTTTCCAGATTTGTCTTA

ACAATTCCTATGTTTTCATC

In100 candidate3615 (in intron):

LOCUS ENSG00000124721

DESCRIPTION Ciliary dynein heavy chain 8 (Axonemal beta dynein heavy chain).

[Source:Uniprot/SWISSPROT;Acc:Q96JB1]

AGGCCTGCTATGCTAACTCTATTTTTTTTTTTTTTTCTGGTGCAGCATTCTTATCAAGTCACTTTGACTATTTGATTTAT

TTTACTTTTTTTTTTTTTGA

In100 candidate3616 (in intron):

LOCUS ENSG00000124721

DESCRIPTION Ciliary dynein heavy chain 8 (Axonemal beta dynein heavy chain).

[Source:Uniprot/SWISSPROT;Acc:Q96JB1]

CTGAGTTTTGTTGCTGCTGATGAGAAGTCTGCAAGAGTCTAACTGAATATTCTCTTTTAATCCTTAATGTTTCTGCAGTT

TTCTTTACCCCGTGTGGATT

In100 candidate3617 (in intron):

LOCUS ENSG00000124721

DESCRIPTION Ciliary dynein heavy chain 8 (Axonemal beta dynein heavy chain).

[Source:Uniprot/SWISSPROT;Acc:Q96JB1]

AAATTTAAATTTCTGAAAATGTATATAGTAACATGTGGATCCTTGACTTTTGTTGACTTCATTTTGTTGTCTCATCAGCT

ATTTTCATTGAACTTTCTTC

In100 candidate3618 (in intron):

LOCUS ENSG00000124721

DESCRIPTION Ciliary dynein heavy chain 8 (Axonemal beta dynein heavy chain).

[Source:Uniprot/SWISSPROT;Acc:Q96JB1]

TGTGTAGTTTGGAGATTGATAAGCAAGTAACACTTAACATTGCTTCCTTCTTGTTTTTAAAGCAGATTGCAACAAGGCTT

CAACATCTCTTCTTTTTTTC

In100 candidate3619 (in intron):

LOCUS ENSG00000196152

DESCRIPTION Zinc finger protein 79 (ZNFpT7).

[Source:Uniprot/SWISSPROT;Acc:15937]

GCTGACTGTCAATGACTGGGTTATTTTAACATCTCTCAAAACCCTAATATTCTAAATTTCTCTTTGCATTTCCTGCAGCT

TTTTCTTTACGTATTATATA

In100 candidate3620 (in intron):

LOCUS ENSG00000134594

DESCRIPTION Ras-related protein Rab-33A (Small GTP-binding protein S10).

[Surce:Uniprot/SWISSPROT;Acc:Q14088]

AGATAACGCGGCCTTTTTCTGTTTCTGTTCTGGTGTCAGCCCTAACCCTGAAATTCTTTCATTGTATCTTTTTTCATCCT

CTTTCATAGTCTTGTAGGCC

In100 candidate3621 (in intron):

LOCUS ENSG00000172987

DESCRIPTION Heparanase-2 (EC 3.2.-.-) (Hpa2).

[Source:Uniprot/SWISSPROT;AccQ8WWQ2]

CCACTTATTTGGTAGCTAATATGCTAATAACCTCTCTCCCACTTCTAGTTGTTGCTTAACTTCTTCATACTTCTACAGAT

TTATGCTTTTCATTCATTTT

In100 candidate3622 (in intron):

LOCUS ENSG00000172987

DESCRIPTION Heparanase-2 (EC 3.2.-.-) (Hpa2).

[Source:Uniprot/SWISSPROT;AccQ8WWQ2]

AGTTATTACTTGATATCATTGCCTTTTAACCTGAAGCACTTCCTTTAATATTTCTTGTGTTGCAGGTCTGCTAGCAACAA

ATTCTCTCAGTCTTTTTTTT

In100 candidate3623 (in intron):

LOCUS ENSG00000172987

DESCRIPTION Heparanase-2 (EC 3.2.-.-) (Hpa2).

[Source:Uniprot/SWISSPROT;AccQ8WWQ2]

TCAAGCACACTTGATGGGATTTATAGTGTAATTTTGAAGTAACACACATATATATATATATTTAACCTCTCTTTTCAGTT

TTAGTTTTGTTTATTCATTA

In100 candidate3624 (in intron):

LOCUS ENSG00000142609

DESCRIPTION KIAA1751 (KIAA1751), mRNA [Source:RefSeq_dna;Acc:NM_001080484]

EXON

CTGAATTAAGGCCGTGCTTTCACTCTGTAACAATGTTTCCATTTGCTTCCTTGCAGACTCCACTTACCTGTGTTTTGGAT

CCTCCTTGCTGGTCTTTGGT

In100 candidate3625 (in intron):

LOCUS ENSG00000067606

DESCRIPTION Protein kinase C zeta type (EC 2.7.11.13) (nPKC-zeta).

[Source:niprot/SWISSPROT;Acc:Q05513]

GGTTGGTGATAACGTCTCCTTTTGGTGTTTTCTTTGGCCTCGTACTCAGCTGTTTTCACCGAGAGTCCCTTTTTGTGTTT

GTCCTGGCCTTTGCGGCTTC

In100 candidate3626 (in intron):

LOCUS ENSG00000119929

DESCRIPTION Copper homeostasis protein cutC homolog.

[Source:Uniprot/SWISSPOT;Acc:Q9NTM9]

TTTCTAAATCGTTCTATTTAACCCTTTGCTATTACTTACCTTCTACCATATATACATTTTTCTTCTCAGTGTCTGTCCTA

CCTTTTAGAGCCATCTTTGT

In100 candidate3627 (in intron):

LOCUS ENSG00000101210

DESCRIPTION Elongation factor 1-alpha 2 (EF-1-alpha-2) (Elongation factor 1A-

2) (eEF1A-2) (Statin S1). [Source:Uniprot/SWISSPROT;Acc:Q05639]

AAACGCTTTTGTTAACTTACTGTCTGAGACATCTCTGTCATACTTTTATTCTCTACAGTCTTCATTGCCCCTCTTTGCTG

GCCTCTTCATATGATTTATT

In100 candidate3628 (in intron):

LOCUS ENSG00000164627

DESCRIPTION Kinesin-like protein KIF6. [Source:Uniprot/SWISSPROT;Acc:Q6ZMV9

TGCCTATTTCTGTAACTCATATTAAACATACATTTTTCTTTTCATTCAGTGTTATCCCTTAGACTTTGAAAAATATATTT

TCTTTCTTTTTATTGCTCTT

In100 candidate3629 (in intron):

LOCUS ENSG00000165675

DESCRIPTION Tumor-associated hydroquinone oxidase (tNOX) (Cytosolic

ovariancarcinoma antigen 1) (APK1 antigen) [Includes: Hydroquinone [NADH]

oxidase EC 1.-.-.-); Protein disulfide-thiol oxidoreductase (EC 1.-.-.-)].

[Source:niprot/SWISSPROT;Acc:Q16206]

TTATTGATAAGTAAGAATTAACTACTGCCATTTTGTTCATTGTCTTCTGGTTGTTTTGCAGATCCGTTATTCCTTTCTTC

CTCTCTTGCCATCTTCTTTT

In100 candidate3630 (in intron):

LOCUS ENSG00000165675

DESCRIPTION Tumor-associated hydroquinone oxidase (tNOX) (Cytosolic ovariancarcinoma antigen 1) (APK1 antigen) [Includes: Hydroquinone [NADH] oxidase EC 1.-.-.-); Protein disulfide-thiol oxidoreductase (EC 1.-.-.-)]. [Souce:niprot/SWISSPROT;Acc:Q16206]

TTTCATTTGATGCTATTAACAATGCCCCCTTCTTTGTATTTTCTCTACTTTTGCTTTCCTTGGTAATACACTTTGCAGAT

TCTTCCTTTACTGTCTCCTT

In100 candidate3631 (in intron):

LOCUS ENSG00000107566

DESCRIPTION SPFH domain-containing protein 1 precursor (Protein KE04). [Souce:Uniprot/SWISSPROT;Acc:O75477]

CTAAATGTAACTATATTCTGAGTCTCTTCTTGGTTCTCTCTTTTTCCATTACATTTCCTTTCTTTTATTAGGCAGCCTCT

TCAACTGTATAGCTTTACTT

In100 candidate3632 (in intron):

LOCUS ENSG00000142606

DESCRIPTION Membrane metallo-endopeptidase-like 1 (EC 3.4.24.11) (Membrane etallo-endopeptidase-like 2) (Neprilysin-2) (Neprilysin II) (NL2) (NEPII) (EP2(m)) [Contains: Membrane metallo-endopeptidase-like 1, soluble form (Nepilysin-2 secreted) (NEP2(s))]. [Source:Uniprot/SWISSPROT;Acc:Q495T6]

TTTTGGATCAACCTATCATTATTTCCTCTATTTTCCCTCAACTAACTTGGAAGTTTTGTATTTCTATCTTTTCGACAGTT

ATCTTAGATTTTTTTGTTTT

In100 candidate3633 (in exon):

LOCUS ENSG00000076770

DESCRIPTION Muscleblind-like X-linked protein (Muscleblind-like protein 3)

Cys3His CCG1-required protein) (Protein HCHCR).

[Source:Uniprot/SWISSPROT;Ac:Q9NUK0]

AATTGTATATATAAAATTCTAATCACTTATGATTCTTAGTTAACATTTTATAAGCTTTGTATTTTACAGGGGCCTCTATT

TATCCAAATTTATTGTTTTT

In100 candidate3634 (in intron):

LOCUS ENSG00000171004

DESCRIPTION Heparan-sulfate 6-O-sulfotransferase 2 (EC 2.8.2.-) (HS6ST-2).

Source:Uniprot/SWISSPROT;Acc:Q96MM7]

CATTTTAACAATATTAATTCTTTCAATCTATGAGCATGTGATGTCTTTCCTTTTTTGTGTCTTCTTCAATTTCTTCAGAG

TTTTATTGTTTTTATTTTAC

In100 candidate3635 (in intron):

LOCUS ENSG00000171004

DESCRIPTION Heparan-sulfate 6-O-sulfotransferase 2 (EC 2.8.2.-) (HS6ST-2).

Source:Uniprot/SWISSPROT;Acc:Q96MM7]

AGAGAGGAGGTCGAAGTTTCTAACTATAACTGTGGATTTGTCTATTTCTCCTTTTAGTTCCATCAGTTTTCACTTTATAT

ATTTTGCATTTCTGTTGTTT

In100 candidate3636 (in intron):

LOCUS ENSG00000171004

DESCRIPTION Heparan-sulfate 6-O-sulfotransferase 2 (EC 2.8.2.-) (HS6ST-2).

Source:Uniprot/SWISSPROT;Acc:Q96MM7]

ATAATATACCATTTTAGATGGTATATTATTCATGTGAAAACCTATAACAATATATTTTAATTTTCCTCTTCTCAGCCTTT

GAACTACCTTTCTCATCATT

In100 candidate3637 (in intron):

LOCUS ENSG00000171004

DESCRIPTION Heparan-sulfate 6-O-sulfotransferase 2 (EC 2.8.2.-) (HS6ST-2).

Source:Uniprot/SWISSPROT;Acc:Q96MM7]

TCTTCGAATATTTTGGTACTTCATTGAACCCTGTGGTGTTTCCCATAGCTAACTGATGCTCTGTTCTGTTTGTCTCAGTC

TTTTTTCTCTATGTATTTCA

In100 candidate3638 (in intron):

LOCUS ENSG00000171004

DESCRIPTION Heparan-sulfate 6-O-sulfotransferase 2 (EC 2.8.2.-) (HS6ST-2).

Source:Uniprot/SWISSPROT;Acc:Q96MM7]

CAAACACCACCCATTCCTCAATCCTACTTAAGGGATTAACTATGCCCTTTCTTCTCTTTTTTCTTAGTCTTTTGTCAGCT

TTGCTGCTCATTAGCTCTTA

In100 candidate3639 (in exon):

LOCUS ENSG00000171004

DESCRIPTION Heparan-sulfate 6-O-sulfotransferase 2 (EC 2.8.2.-) (HS6ST-2).

Source:Uniprot/SWISSPROT;Acc:Q96MM7]

TAGAATTTAACCTAAAATGTGAACTCTTACTTACTTTTTGTACATCTCTATTGTAATGTTTCCAGTTGGGCCAACTATTT

CTAGCCCTTTAGTTCTTTTT

In100 candidate3640 (in intron):

LOCUS ENSG00000076716

DESCRIPTION Glypican-4 precursor (K-glypican).

[Source:Uniprot/SWISSPROT;Ac:O75487]

TTTTTCCTAACTTTGATATTTTATATGTCCTTTCTCTTTCCATTTTTCTTATAACTTCCTCATCTCTCTTTTCAGTTCTG

GTATTATTTAGTGTTTCCCT

In100 candidate3641 (in intron):

LOCUS ENSG00000147257

DESCRIPTION Glypican-3 precursor (Intestinal protein OCI-5) (GTR2-2) (MXR7)

[Source:Uniprot/SWISSPROT;Acc:P51654]

ATAACATATGATATTGAATATCTTTTTCTAGTTTTTTTTCCCCATCTGTATATCTTCTTTGGTGAGATGTCTGTTCAGAT

CTTCTGCCCATTTTTTAATT

In100 candidate3642 (in intron):

LOCUS ENSG00000186376

DESCRIPTION Zinc finger protein 75. [Source:Uniprot/SWISSPROT;Acc:P51815]

TATAGCTCAAGTGTCGACGTAATTCTTACATTCGCATTAATTTTAACTCAATTCTATGTTGCTGTTTTGCAGAGCTGCCT

TCTTTTCACCTCTGAGTTTT

In100 candidate3643 (in intron):

LOCUS ENSG00000186376

DESCRIPTION Zinc finger protein 75. [Source:Uniprot/SWISSPROT;Acc:P51815]

AGTAGGTCTCTTTGGTAAATCTATCCATAACAAAATTCATTATTTTACATCTGTATTTCTTCATTCTACAACCTACAGGC

TGCTATAATTTGTTTCCTCA

In100 candidate3644 (in intron):

LOCUS ENSG00000186376

DESCRIPTION Zinc finger protein 75. [Source:Uniprot/SWISSPROT;Acc:P51815]

GGGAAGGTAACCCTTTTCTTCATTCTCATTTATATCCTATCTTTTTCTAAGTTGGTTTCCAGTTTTCCATATTTCTCGAA

AATTGATCTACATTTTTCCT

In100 candidate3645 (in intron):

LOCUS ENSG00000173275

DESCRIPTION Zinc finger protein 449 (Zinc finger and SCAN

domaincontainingprotein

19). [Source:Uniprot/SWISSPROT;Acc:Q6P9G9]

TGTTCCTGCCACTGGTTTAACATAACTCTTCATTTTCCTTCACTTTCTTTTCTCCATGGAACACAATATCATCTGCAGTT

TCTATTTCTTCTCTCAGGTT

In100 candidate3646 (in intron):

LOCUS ENSG00000107829

DESCRIPTION F-box/WD repeat protein 4 (F-box and WD-40 domain protein 4)

(Dctylin). [Source:Uniprot/SWISSPROT;Acc:P57775]

AATCACTTTTAACATTTTAGTTTTCTTCCACTCTTTCCTTTTCTCTGCATAGGCTCTTTTTTTTAGTAGTTGTAACAGTG

CTGTTTGTACCCTGCTTTTT

In100 candidate3647 (in intron):

LOCUS ENSG00000156920

DESCRIPTION Probable G-protein coupled receptor 112.

[Source:Uniprot/SWISSPOT;Acc:Q8IZF6]

AGACATTTCCTTTCCCCTCATCTCTGCTTTTCCCATTACCTAACTCATCAACCTGTCAATTTCTTTCAGAGTACTGTATG

TAATTTGTGTTTTTTCCTTT

In100 candidate3648 (in intron):

LOCUS ENSG00000120029

DESCRIPTION Uncharacterized protein C10orf76.

[Source:Uniprot/SPTREMBL;Acc:5T2E6]

TGAGCCCCTAGGCCTGGGCTCAAGGTGGATTTTTTGTTTTTTAACCTTTTGGTTTGTTTAGGTTGCCTTTATTTTCAGTT

ACTCTTTTTGTCTTTTTTTT

In100 candidate3649 (in intron):

LOCUS ENSG00000112655

DESCRIPTION Tyrosine-protein kinase-like 7 precursor (Colon carcinoma kinas

4) (CCK-4). [Source:Uniprot/SWISSPROT;Acc:Q13308]

AGTTGGTGGCACATTGTTATCTAACTTCTGTCTTGTTTTCCAAATTTGTTCCAGAAAGCTGCTTTAACTTTTTTTTTTTT

TTTTTTTGAGACAGTCTCGC

In100 candidate3650 (in intron):

LOCUS ENSG00000112655

DESCRIPTION Tyrosine-protein kinase-like 7 precursor (Colon carcinoma kinas 4) (CCK-4). [Source:Uniprot/SWISSPROT;Acc:Q13308]

TAGACTTTGTCTCAAAAAAAAAAAAAACTTTACAAAAAAATAACTTTACAAGGAGTTGTTTCTGTCCTTTATTCATCAGTT

TGTTTTTTTCATCTATTGTT

In100 candidate3651 (in intron):

LOCUS ENSG00000129682

DESCRIPTION Fibroblast growth factor 13 (FGF-13) (Fibroblast growth factor omologous factor 2) (FHF-2). [Source:Uniprot/SWISSPROT;Acc:Q92913]

GTTATATGGAATAGAATGTATATATTGATTTTGGCTGTGTCCTTTAACAAGGATAAATTTTCTTACTTCTGACAGTTTTT

GTAATCAAAACTCTTTTTTT

In100 candidate3652 (in intron):

LOCUS ENSG00000162408

DESCRIPTION Nucleolar protein 9. [Source:Uniprot/SWISSPROT;Acc:Q5SY16]

GTCTCTAATGTTTTCTTGTAAGAGCTTTATAGTTTTTAACTTTTTAAATTTTTTTATTTTTAAATTTTTCAGCCTGAATT

TGTTTGTTCAGTTTTTACTC

In100 candidate3653 (in intron):

LOCUS ENSG00000101974

DESCRIPTION Probable phospholipid-transporting ATPase IG (EC 3.6.3.1) (ATPae class I type 11C) (ATPase IG) (ATPase IQ) (ATPase class VI type 11C). [Sorce:Uniprot/SWISSPROT;Acc:Q8NB49]

AGTGTTCTTTTAGTAACCTTTCCCACTATGAAGTTTCATATTTACTCCTACTTCTCTTTCTCTCATTTCCCCAGCGTCTT

CCTTCCTGTTTTCTTCAGAA

In100 candidate3654 (in intron):

LOCUS ENSG00000101974

DESCRIPTION Probable phospholipid-transporting ATPase IG (EC 3.6.3.1) (ATPae class I type 11C) (ATPase IG) (ATPase IQ) (ATPase class VI type 11C). [Sorce:Uniprot/SWISSPROT;Acc:Q8NB49]

TGCTCTGAGACTCTTGAGTTGGTAACATAATATTTAATTTTCCTCAATATCCCATTTATTCATTTCTTTAATTTCAGCTA

GTATTTCTCCTTCTCTCCGT

In100 candidate3655 (in intron):

LOCUS ENSG00000101974

DESCRIPTION Probable phospholipid-transporting ATPase IG (EC 3.6.3.1) (ATPae class I type 11C) (ATPase IG) (ATPase IQ) (ATPase class VI type 11C). [Sorce:Uniprot/SWISSPROT;Acc:Q8NB49]

TGTTTTTAAAAGATTAACAATAAGAACTATATTTTATATTTACCCACATTTTTTCATTTCCCTATGCAGATTTAAATTTT

CATATAGTATCTTATTTTTT

In100 candidate3656 (in intron):

LOCUS ENSG00000101974

DESCRIPTION Probable phospholipid-transporting ATPase IG (EC 3.6.3.1) (ATPae class I type 11C) (ATPase IG) (ATPase IQ) (ATPase class VI type 11C). [Sorce:Uniprot/SWISSPROT;Acc:Q8NB49]

AGGAAATCTTCATCTCTGATAACATGGTTTTCATTTCTGACTTTACATTTTCATTTTTTTGTAGTATCCCTCTGCCAGCA

TTCTTCGTTCACTGTTACAT

In100 candidate3657 (in intron):

LOCUS ENSG00000101974

DESCRIPTION Probable phospholipid-transporting ATPase IG (EC 3.6.3.1) (ATPae class I type 11C) (ATPase IG) (ATPase IQ) (ATPase class VI type 11C). [Sorce:Uniprot/SWISSPROT;Acc:Q8NB49]

AGTGCTATCTGTTGATTTAAGATGTTTAAATATCTTAACATATTTCTGTTGTGCTTTATTTTTCTCAGTATATTCTAGGT

TTTTTACTTTGCTCATTTCT

In100 candidate3658 (in intron):

LOCUS ENSG00000101974

DESCRIPTION Probable phospholipid-transporting ATPase IG (EC 3.6.3.1) (ATPae class I type 11C) (ATPase IG) (ATPase IQ) (ATPase class VI type 11C). [Sorce:Uniprot/SWISSPROT;Acc:Q8NB49]

TACATTTTTTCAAATAACCATCTCCTGGATTTCTTGGTCTTTTGTATGGTTTTTTTGCATCTCAGTTTCCCTCAGTTCAG

CTCTGATTTGGGTTATTTCT

In100 candidate3659 (in intron):

LOCUS ENSG00000171735

DESCRIPTION Calmodulin-binding transcription activator 1. [Source:Uniprot/SISSPROT;Acc:Q9Y6Y1]

GAACAATTCCTTTCTAAATATGTTTTAGCGTATGTTCCATGTGTTTTAACATGTAGTAGTTTGATTATCCTTCAGTTCGC

AATGTTTTTTAACCTCCATT

In100 candidate3660 (in intron):

LOCUS ENSG00000171735

DESCRIPTION Calmodulin-binding transcription activator 1.

[Source:Uniprot/SISSPROT;Acc:Q9Y6Y1]

AGGATAATAAAAACCACCTATAATCCCATCACCCAAAAATAACCTCTGTTTACATTTTGTTATATTTCCTGCCAGTTTGT

TGTTGTTGTTGTTGTTGTTT

In100 candidate3661 (in intron):

LOCUS ENSG00000116288

DESCRIPTION Protein DJ-1 (Oncogene DJ1) (Parkinson disease protein 7).

[Souce:Uniprot/SWISSPROT;Acc:Q99497]

AGATGACATAAGAATAAATACCTGTTGATCCACTGCTCACATTTAACGCTTGTTAATGTCTTGCCATATTTCCTTCAGAC

CCATTTCTCTTTTGTTTTGA

In100 candidate3662 (in intron):

LOCUS ENSG00000116288

DESCRIPTION Protein DJ-1 (Oncogene DJ1) (Parkinson disease protein 7).

[Souce:Uniprot/SWISSPROT;Acc:Q99497]

CTTCTCTTTGTACCTTAGTAATATTTTATTTTCTCATAACCATTTTAAGATCCATTCTTGTTGCTTTCCTATCAGATGAT

GTTCTGCCATTTTGGTTTTT

In100 candidate3663 (in intron):

LOCUS ENSG00000142599

DESCRIPTION Arginine-glutamic acid dipeptide repeats protein (Atrophin-1-lie protein) (Atrophin-1-related protein). [Source:Uniprot/SWISSPROT;Acc:Q9P26]

AAAGTTTGGTATTTAACACTTAAATTTGTTTTCTGCTGCCAGTAATGGAGATAATCTATTATCACTTTTTTTAGGGGTTT

TTTTTTTTTTTTTTTTTTTT

In100 candidate3664 (in intron):

LOCUS ENSG00000142599

DESCRIPTION Arginine-glutamic acid dipeptide repeats protein (Atrophin-1-lie protein) (Atrophin-1-related protein). [Source:Uniprot/SWISSPROT;Acc:Q9P26]

CTTATTTTCATTCCCTCTGCCCCTTCTTAGATAAATAATAACATAATATACATCCTTTTGCTACCTTTCTTTTAACAGTA

TATTCTTGTGTTCTCCTCAT

In100 candidate3665 (in intron):

LOCUS ENSG00000176988

DESCRIPTION fragile X mental retardation 1 neighbor [Source:RefSeq_peptide;cc:NP_689791]

TGCCATCTTCCTAACATCATCTGCTTTTTTTTTTTTTTTATCCAGCCTGGATCCCATTGTCTAGTACTTTAATCCATTCT

TGTTTCCAGTCCCCTTGTTT

In100 candidate3666 (in intron):

LOCUS ENSG00000148843

DESCRIPTION RRP5 protein homolog (Programmed cell death protein 11). [Sourc:Uniprot/SWISSPROT;Acc:Q14690]

AGAACTAACCTCTCCTGTCTTCTGTTTTCACTTATACCCAGTTCTGGTTCTCATGGTTTCTCTGCTGTACTGCATTTGAA

TCCTTTTTTTTTTTTTTTTT

In100 candidate3667 (in intron):

LOCUS ENSG00000107954

DESCRIPTION Neuralized-like protein 1 (h-neuralized 1) (h-neu) (RING fingerprotein 67). [Source:Uniprot/SWISSPROT;Acc:O76050]

GCTTATATCATTGAGATCATAATGTGAGAACATTTTAACCCCTGCTTTTCTGCTTTTAAAATTTTCTTTTATTTTCAGAC

TTTTTTTTTAAATTTTTTGA

In100 candidate3668 (in intron):

LOCUS ENSG00000130939

DESCRIPTION Ubiquitin conjugation factor E4 B (Ubiquitin fusion degradationprotein 2) (Homozygously deleted in neuroblastoma 1). [Source:Uniprot/SWISSROT;Acc:O95155]

GTGACTACTTTAAATACCCCCTCATGGTAAAACTTTGTTCTTTTTCTTTAACTCATTCAATAGATGTTTTTCTTTCAGAT

TATTTTGACATATACTTTTC

In100 candidate3669 (in intron):

LOCUS ENSG00000065621

DESCRIPTION Glutathione transferase omega-2 (EC 2.5.1.18) (GSTO-2). [SourceUniprot/SWISSPROT;Acc:Q9H4Y5]

GTGCCATACTTTTCTCTTTGGGAAGACCTGTACCAACAATAACCTCTGCTAAACACTTTCGCTTTTTTAAATTTGCAGGT

ACTTTTTTGGGGCTGTCTTT

In100 candidate3670 (in intron):

LOCUS ENSG00000120051

DESCRIPTION Leucine-rich repeat-containing protein C10orf80.

[Source:Unipro/SWISSPROT;Acc:Q5T655]

GTCTTCCCACTAACCATTACTACCTATTCCCTTTTTACAGTTTTTCTTTTACTCAACAGTTACCACCACTCAGTATAATA

TTCTCTCTCTGTCTCTGATT

In100 candidate3671 (in intron):

LOCUS ENSG00000156395

DESCRIPTION VPS10 domain-containing receptor SorCS3 precursor.

[Source:Unipot/SWISSPROT;Acc:Q9UPU3]

GTTTTGTTTTGTTCTTCATAGTGATAACATTTGAATCTTTTATCTTCCTTTTTTGTTTGTTTGTTCTACCAGTGGCTTTT

ATATTTTCATGTGTCTGCCT

In100 candidate3672 (in intron):

LOCUS ENSG00000108018

DESCRIPTION VPS10 domain-containing receptor SorCS1 precursor (hSorCS).

[Sorce:Uniprot/SWISSPROT;Acc:Q8WY21]

AAAAGAAACTAATGATCTTACCATAATAAATTATTTACCATAACGAATAATTACCCATGTATTTGTTCTTTCCCACAGTC

CCCTTTTGATTATTTGAATT

In100 candidate3673 (in intron):

LOCUS ENSG00000108018

DESCRIPTION VPS10 domain-containing receptor SorCS1 precursor (hSorCS).

[Sorce:Uniprot/SWISSPROT;Acc:Q8WY21]

AACTAATTTCACTTAAGAAATTTTAACCTATCATTGTAATTCCTTTCAATGATGTGTTCTATCTTCCTTCTCGTTCAGAG

GTTCTTAGTGATTTTCTTCT

In100 candidate3674 (in intron):

LOCUS ENSG00000108039

DESCRIPTION Xaa-Pro aminopeptidase 1 (EC 3.4.11.9) (X-Pro aminopeptidase 1)(X- prolyl aminopeptidase 1, soluble) (Cytosolic aminopeptidase P) (Solubleaminopeptidase P) (sAmp) (Aminoacylproline aminopeptidase). [Source:UniprotSWISSPROT;Acc:Q9NQW7]

GCCTGATATTTCACTGATGTGCTTCTTTTTTCTTGCCCTTTTAACATTTTCCCTTCTTGTTTTCCTTAGCAGTAGGTGTT

TATTTATATCCTGCCTCTCT

In100 candidate3675 (in intron):

LOCUS ENSG00000011677

DESCRIPTION Gamma-aminobutyric-acid receptor subunit alpha-3 precursor (GAB(A) receptor subunit alpha-3). [Source:Uniprot/SWISSPROT;Acc:P34903]

CTTCTTCAAACACCTCCTAGTTATAGGGCCCTGTAACATTTGCAACATTTGCTTCTTTTTCCACATAGTGGCTTTCAGAT

ATACTCCATGCTTTCTTGTC

In100 candidate3676 (in intron):

LOCUS ENSG00000148700

DESCRIPTION Gamma-adducin (Adducin-like protein 70).

[Source:Uniprot/SWISSPOT;Acc:Q9UEY8]

ACACTAATAAAATATTAATAGTCTTATTATTGATAACTGAGTTTTGTCTGGTGCTGTTTTTTTTTTTTTTTCCATCAGTT

TTTTTACTTCTTAGGCTTGG

In100 candidate3677 (in intron):

LOCUS ENSG00000148700

DESCRIPTION Gamma-adducin (Adducin-like protein 70).

[Source:Uniprot/SWISSPOT;Acc:Q9UEY8]

CAATTTTATGGCATTCACTTTGTATCTTAACCTCATTCCTGGCTCTGTCTACCTTCCTTATGCTTATTTTCTCTTCAGCT

TTATTTTTCTTACCTATATT

In100 candidate3678 (in intron):

LOCUS ENSG00000187239

DESCRIPTION Formin-binding protein 1 (Formin-binding protein 17) (hFBP17).

Source:Uniprot/SWISSPROT;Acc:Q96RU3]

TAAAAGCTCTTCTAGGTAGTTTTGATGTGGCTTAGGTAGCACTGTTTAACATTTTGATTCCTTCTCATGCATTCTCAGTT

TTTGCTCATTTTTATTCTTT

In100 candidate3679 (in intron):

LOCUS ENSG00000203869

DESCRIPTION Novel protein (Fragment). [Source:Uniprot/SPTREMBL;Acc:Q5T481]

EXON

TCACCCCTAACTTTGGCGTCCATATTTGTAAAGTGTCTTTTCCTTCCTCTTTGACTGCCTCAGTTTCCCCCAGAATACCT

CTTGTGATGCTTCTTTTGTT

In100 candidate3680 (in intron):

LOCUS ENSG00000203869

DESCRIPTION Novel protein (Fragment). [Source:Uniprot/SPTREMBL;Acc:Q5T481]

EXON

TTTCCAACGTGGCTACACCATTTTACACCAATGCCACTAACAATGAATGAGGATTCCAATTTTTCCACATTCTTGCAGCA

CTTATTATCATCTTTTTGAT

In100 candidate3681 (in intron):

LOCUS ENSG00000203869

DESCRIPTION Novel protein (Fragment). [Source:Uniprot/SPTREMBL;Acc:Q5T481]

EXON

AATGGCACATGCAAATGTGATGAAGATGGGCCTGTGTATCTAACCGTTGTGAGGTTGTCTTTTAGCTCTACTCTGCAGTA

ACTTTGTTTTTTTTTTTGTT

In100 candidate3682 (in intron):

LOCUS ENSG00000097007

DESCRIPTION Proto-oncogene tyrosine-protein kinase ABL1 (EC 2.7.10.2) (p150

(c- ABL) (Abelson murine leukemia viral oncogene homolog 1).

[Source:Uniprt/SWISSPROT;Acc:P00519]

TAGTACTGGTAACATTTATTAACTTTTTTTTTTTTCCTGTTCCCTAATTTGCTACCCAGCTTTTACATCAAAGCTCCTAT

CACAACCCTTCTTGTTTTTT

In100 candidate3683 (in intron):

LOCUS ENSG00000196284

DESCRIPTION Transcription initiation protein SPT3 homolog (SPT3-like protei).

[Source:Uniprot/SWISSPROT;Acc:O75486]

TTGCCTCTTCCATAACTGCTGCCAAATTCTTCTGTTAGTTTCTTTGTAATATCTCTTCAACTTGCCCATATTTTTCAGTC

CAGTCTCCGTTTTTTTTTAA

In100 candidate3684 (in intron):

LOCUS ENSG00000196284

DESCRIPTION Transcription initiation protein SPT3 homolog (SPT3-like protei).

[Source:Uniprot/SWISSPROT;Acc:O75486]

AGGAAAAACAAAAATTTGCATAACAAACCAAATTGTGCACCTTTTTTTTTTTAATTCAGAATTTTCATTATTTTTTTTTGA

CTTTTTTTTTTTTAAATTTA

In100 candidate3685 (in intron):

LOCUS ENSG00000196284

DESCRIPTION Transcription initiation protein SPT3 homolog (SPT3-like protei).

[Source:Uniprot/SWISSPROT;Acc:O75486]

TTTATTTATTTATTTCGTTCTGTTACTCATTTTTAACATGTTTTTCTTTTTCTTAATTTCATGATTTTCCCACAGTTTAC

TCTGTGCAATTTGATTTTCT

In100 candidate3686 (in intron):

LOCUS ENSG00000196284

DESCRIPTION Transcription initiation protein SPT3 homolog (SPT3-like protei).

[Source:Uniprot/SWISSPROT;Acc:O75486]

GATTTTGTTATCTAGATATAACTACTCCTTAGTATTTTTTTTTTTTTGAGACTGAGTTTCGCTCTTGTTGCCCAGGCTGG

TTAATTAATTCTGTTTTCCT

In100 candidate3687 (in intron):

LOCUS ENSG00000124813

DESCRIPTION Runt-related transcription factor 2 (Core-binding factor, alpha1 subunit) (CBF-alpha 1) (Acute myeloid leukemia 3 protein) (Oncogene AML-3 (Polyomavirus enhancer-binding protein 2 alpha A subunit) (PEBP2-alpha A) PEA2-alpha A) (SL3-3 enhancer factor 1 [Source:Uniprot/SWISSPROT;Acc:Q13950

GTGACAATTTTGATAAACTATACATTTATTCCGTGTCACCAATGTGTTAACTGGCAAATACATTATGATCTCCTTCAGTT
TCTAATCCTTTTTGCCAAGT

In100 candidate3688 (in intron):

LOCUS ENSG00000172348

DESCRIPTION Calcipressin-2 (Thyroid hormone-responsive protein ZAKI-4) (Dow syndrome candidate region 1-like 1) (Myocyte-enriched calcineurin- interacing protein 2) (MCIP2). [Source:Uniprot/SWISSPROT;Acc:Q14206]

ATTACTCATTTTCCCAAATAACAAGCACTGTTTTTATGTCTTCTGTATTTGTCTATGTACTTCTACATGCATCTCAGAC
TCTCGATGGTGTTTTTCCTG

In100 candidate3689 (in intron):

LOCUS ENSG00000172348

DESCRIPTION Calcipressin-2 (Thyroid hormone-responsive protein ZAKI-4) (Dow syndrome candidate region 1-like 1) (Myocyte-enriched calcineurin- interacing protein 2) (MCIP2). [Source:Uniprot/SWISSPROT;Acc:Q14206]

ATGTCTATTACTAATTAAGATTTTTTAAAACTAGTAACTTTACTAGAAAATTTTTTCTGACCTCATTCATTTTACCAGTT TTGCTTTCATGCTCATTTCT

In100 candidate3690 (in intron):

LOCUS ENSG00000172348

DESCRIPTION Calcipressin-2 (Thyroid hormone-responsive protein ZAKI-4) (Dow syndrome candidate region 1-like 1) (Myocyte-enriched calcineurin- interacing protein 2) (MCIP2). [Source:Uniprot/SWISSPROT;Acc:Q14206]

AACCTCTCAATTTGCAAACTTGCTTGGGTTTCTTCCATCCTAACAACTGATGCTCTTTTGATGCCATGTTTCCTTCAGTC TTTTTTCTTCTCTTAAGTGT

In100 candidate3691 (in intron):

LOCUS ENSG00000146233

DESCRIPTION Cytochrome P450 39A1 (EC 1.14.13.99) (24-hydroxycholesterol 7-apha- hydroxylase) (Oxysterol 7-alpha-hydroxylase) (hCYP39A1). [Source:Uniprt/SWISSPROT;Acc:Q9NYL5]

TTTGTTGAAAACTGGACATTTTAAATAATAGAATGTAACAACTTTGGAAATTAGATTCTTCCCTTTCCTCAGGGTTTGTT GTTGTTGCTGTTTATTGTTC

In100 candidate3692 (in intron):

LOCUS ENSG00000153291

DESCRIPTION Mitochondrial uncoupling protein 4 (UCP 4) (Solute carrier famiy 25 member 27). [Source:Uniprot/SWISSPROT;Acc:O95847]

AATTTAACCACTCCAAGAGTATTACTGACTCGGCTTTTGCATGTACTGTTCTATCTTCTTGTAACATTCTTCCTTCAGGT

CTTCATATACATGTGTCCTT

In100 candidate3693 (in intron):

LOCUS ENSG00000180113

DESCRIPTION Tudor domain-containing protein 6 (Antigen NY-CO-45).

[Source:Uiprot/SWISSPROT;Acc:O60522]

TCCCTTTTAGAGAACATTACATTTGTCATGGTAACACCCATGGAAATAAATTTTTTTGTTTGTATGTTTCTTTCTCAGTT

TGAAGTTTTTCTCTTTTTGT

In100 candidate3694 (in intron):

LOCUS ENSG00000112818

DESCRIPTION Meprin A subunit alpha precursor (EC 3.4.24.18) (Endopeptidase-)

(N- benzoyl-L-tyrosyl-P-amino-benzoic acid hydrolase subunit alpha) (PABApeptide

hydrolase) (PPH alpha). [Source:Uniprot/SWISSPROT;Acc:Q16819]

TGGGTTTGGATTTGGATTGTTCTTGTTTCTCCAATTCTGTGTGGTGTAACCTTAGATTGTCTATTTGTGCTCTTTCAGAC

TTTTTGGTGTCATTTAATTC

In100 candidate3695 (in intron):

LOCUS ENSG00000112818

DESCRIPTION Meprin A subunit alpha precursor (EC 3.4.24.18) (Endopeptidase-)

(N- benzoyl-L-tyrosyl-P-amino-benzoic acid hydrolase subunit alpha) (PABApeptide

hydrolase) (PPH alpha). [Source:Uniprot/SWISSPROT;Acc:Q16819]

GTTGGACTTTACCTTTCTCTGGTGCCTCCTTGATTGGCTTAATAACTGACCTTCAAAATTCTTTTTCTGGCAATTCAGAG

ATTTTGTCTTCATTTGGCTC

In100 candidate3696 (in intron):

LOCUS ENSG00000069122

DESCRIPTION Probable G-protein coupled receptor 116 precursor.

[Source:Unipot/SWISSPROT;Acc:Q8IZF2]

TTTCATTTTGCAAAACTCAAACTCTATACCCATTTGTCAATAACTCCTCATTTCCTTCTCTACTGACAGCTGCTGGTTAC

TACTATTCTAATTTCTGTCT

In100 candidate3697 (in intron):

LOCUS ENSG00000198087

DESCRIPTION CD2-associated protein (Cas ligand with multiple SH3 domains)

(dapter protein CMS). [Source:Uniprot/SWISSPROT;Acc:Q9Y5K6]

ATATTGGGAATTATTGTTAACCATCATATTTGTTCTTTTTTAAGTACTTTTTTTTTAAAATTAACCTCAACTTCCCAGGT

TTTTATTCTTACTTCTCTTC

In100 candidate3698 (in intron):

LOCUS ENSG00000153294

DESCRIPTION Probable G-protein coupled receptor 115 (G-protein coupled

recetor PGR18). [Source:Uniprot/SWISSPROT;Acc:Q8IZF3]

TTGGTAATAACTAAGAGTATGTTTTAACATCATGATTCTTTTGTAAATGGCTCTTCTTCCCTCTTCTGTGTTTCCCAGGG

TTTCTTCATCCTAGTGTTTG

In100 candidate3699 (in intron):

LOCUS ENSG00000198924

DESCRIPTION DNA cross-link repair 1A protein (hSNM1) (hSNM1A).

[Source:Unipot/SWISSPROT;Acc:Q6PJP8]

AGGCCAAGCTATTACATTAGGTTGCCTTTAAGTTTTTGTCTGCTAACTTTGCTACTGTTTTTTTTTTTTAATTGCCAGTT

TGAATTCTGATTTTTTTTTT

In100 candidate3700 (in intron):

LOCUS ENSG00000107290

DESCRIPTION Probable helicase senataxin (EC 3.6.1.-) (SEN1 homolog).

[Sourc:Uniprot/SWISSPROT;Acc:Q7Z333]

TTGTCACCTAGATTCTAGCTTCTGGATTTTACTGTTGCTTAACTTTGGAAAATGTTACTATTTAGCATCACTTTCCAGAA

TGTTGCTTTCTTAATTTGCT

In100 candidate3701 (in intron):

LOCUS ENSG00000096006

DESCRIPTION Cysteine-rich secretory protein 3 precursor (CRISP-3) (SGP28

prtein). [Source:Uniprot/SWISSPROT;Acc:P54108]

GGTTTTTTTTTATTTTTAATTATAAGTTACATTTAACATTAAATGTTATTTTTCATATTGCAACCTTCTGTGCTTCAGGA

ACTTTTCTTATCTCTCTAGG

In100 candidate3702 (in intron):

LOCUS ENSG00000116708

DESCRIPTION PRAME family member 4. [Source:Uniprot/SWISSPROT;Acc:O60810]

TATTCCTGTCTGTGTTTTTGTTCCCATTAACCATCCCCTTCTTATTCTCTATTTCCCAGTACCCTTCCTAGCTTCTGATA

ACCGTCATTTTACTATTTTT

In100 candidate3703 (in intron):

LOCUS ENSG00000008197

DESCRIPTION transcription factor AP-2 beta-like 1

[Source:RefSeq_peptide;Ac:NP_758438]

TTTCATTGATATTAACTGTTCTCTTTTGTATATTTTGTATATCTATAAACTTTCCCTCCCTTTTCTTCATATTATCAGCT

ATTTCATTTTTTCCTGGTAC

In100 candidate3704 (in intron):

LOCUS ENSG00000204501

DESCRIPTION PRAME family member 9. [Source:Uniprot/SWISSPROT;Acc:Q5VWM5]

TATTCCTGTCTGTGTTTTTGTTCCCATTAACCATCCCCTTCTTATTCTCTATTTCCCAGTACCCTTCCTAGCTTCTGATA

ACCGTCATTTTACTATTTTT

In100 candidate3705 (in intron):

LOCUS ENSG00000107518

DESCRIPTION attractin-like 1 [Source:RefSeq_peptide;Acc:NP_997186]

CATCATTTATATGTAAGAGAGTTGCTTAACTGTTATCAAATTGTTCCTGTTCCTTTTCTATTCCAGAATGTCCTGCTCAA

TCCTATTGCTCGATTCTTTT

In100 candidate3706 (in intron):

LOCUS ENSG00000107518

DESCRIPTION attractin-like 1 [Source:RefSeq_peptide;Acc:NP_997186]

TGTTTTTTCTTAGTTTGTCTGGCTAAAGGTTTTTCTATTTTGTTTAACCTTTCAAAAATCAACCTTTTGTTTCATCAGTC

TTTTTTTTATTGTTTTCTTC

In100 candidate3707 (in intron):

LOCUS ENSG00000107518

DESCRIPTION attractin-like 1 [Source:RefSeq_peptide;Acc:NP_997186]

GATCGTCAAAAATTGTAACTTAAAAATGAATTTCCATTTGTGTCATCTCTGATTTTTTTGTGCAGTGGTTTTTAGTTTTC

CTTGTAGAGATCTTTCTCTT

In100 candidate3708 (in intron):

LOCUS ENSG00000107518

DESCRIPTION attractin-like 1 [Source:RefSeq_peptide;Acc:NP_997186]

TTTTTCTCAATTCTTTAATTTTTACTATTAATATTTTATAGTTATAACTGTACACATATTTCACATTTTTGTCAGATTTA

TCCCTGTATATTTTAAATTT

In100 candidate3709 (in intron):

LOCUS ENSG00000107518

DESCRIPTION attractin-like 1 [Source:RefSeq_peptide;Acc:NP_997186]

CCATATATAACAATTTCAATTTCTCATTTCCATTTACAGCCCTTCAGATTTGTAAAGTATTTTCTACGGTTTACACATTC

CTTGTTCATTTTTCTACTTC

In100 candidate3710 (in intron):

LOCUS ENSG00000107518

DESCRIPTION attractin-like 1 [Source:RefSeq_peptide;Acc:NP_997186]

AAGTACTTGGTATAACATAAAGCATTATATTAATTGTTGCAATTCTTACTATTTTATTTTTCACTCAGTTCTCTTAATA

ACTAGCATGCCTCTATTTCT

In100 candidate3711 (in intron):

LOCUS ENSG00000096093

DESCRIPTION EF-hand domain-containing protein 1.

[Source:Uniprot/SWISSPROT;cc:Q5JVL4]

TATGTAACGACATTCTTTGTCTCTTTTGATCTTTCTTGGTTTAAAGTCTGTTTTATCAGAGACTAGGATTGCAACCCCTG

CTTTTTTTTGTTTTCCATTT

In100 candidate3712 (in intron):

LOCUS ENSG00000182645

DESCRIPTION

TTCATTGGGTTTTGAATAATCTCCATTTTTAACTTCATAGCTATTTTTATATTTATACTATTTTCTGCTACTCAGTGTTT

TTTTCCCACTGCCAGATATT

In100 candidate3713 (in intron):

LOCUS ENSG00000182645

DESCRIPTION

GTGTTTATGCCTTGAAGAACCATTTGTAAATTGTTTTTTTAACTACCTATCTATGACTTTTTCCCATTTTTCTATCAGGC

TTTTGGTCTTTTTTGTCCCT

In100 candidate3714 (in intron):

LOCUS ENSG00000203837

DESCRIPTION pancreatic lipase-related protein 3

[Source:RefSeq_peptide;Acc:P_001011709]

ATATCCTTTATTATTGGCCACTTAAGTTATTGACCCAATTTCAATGTATTAACTTTATAGCAAACATTATTTTTACAGAA

CTATTAATCTTTCAATCTTT

In100 candidate3715 (in intron):

LOCUS ENSG00000189337

DESCRIPTION kazrin isoform B [Source:RefSeq_peptide;Acc:NP_001018000]

GAGACTCCGTCTCAAGAAAAAAAAAAAACAACAAAAAAAACCACTAACTATTATGGATTTGTCTATTTCTCCCTGCAGTT

CTCAGCTTTCGTTTACTGTA

In100 candidate3716 (in intron):

LOCUS ENSG00000165650

DESCRIPTION PDZ domain-containing protein 8 (Sarcoma antigen NY-SAR-84/NY-SR-

104). [Source:Uniprot/SWISSPROT;Acc:Q8NEN9]

TTAATGATAACCTTCAAATTGAGTTTTACCTTTTTAAATTTTTCTTATTTTTCTTTTTTTATGTCACAAATGTCTCAGCA

TCTTTTTTTTAACTTATTAA

In100 candidate3717 (in intron):

LOCUS ENSG00000165650

DESCRIPTION PDZ domain-containing protein 8 (Sarcoma antigen NY-SAR-84/NY-SR-104). [Source:Uniprot/SWISSPROT;Acc:Q8NEN9]

TTAACCAAAAGACAACTGCCTTTATTTTCTCTCTTTCAAAATTTTTTCAACCCATTTGTCTCAACTTATATAGTCCAGAC

TTTTCATTTATTAGCTGTGC

In100 candidate3718 (in intron):

LOCUS ENSG00000107560

DESCRIPTION Rab11 family-interacting protein 2 (Rab11-FIP2) (NRip11). [Soure:Uniprot/SWISSPROT;Acc:Q7L804]

TGACTATAAACACGAAGTATGCAACGTGATTAGTTCCCTTAACACTAACTAGAACTTTTATGTTTAACTCCCCAGATACT

ACTTGAAACTTTTTTACCCT

In100 candidate3719 (in intron):

LOCUS ENSG00000165669

DESCRIPTION Uncharacterized protein C10orf84. [Source:Uniprot/SWISSPROT;AccQ9H8W3]

CATCCCTGAACCTTTTCTTAACCCTTGGTAAAATGCTTTCTTACTGTTTGCTCAGCACATCCCCCTCAGCCCTAAAGAAT

TTTGGGTCTTTTTTCTTTTT

In100 candidate3720 (in intron):

LOCUS ENSG00000151893

DESCRIPTION Uncharacterized protein C10orf46. [Source:Uniprot/SWISSPROT;AccQ86Y37]

GATTTAATTCTCTTATACCTCTAACATACCTTACCTTTCTAATTTTTTTACTATCACAGTTTTGTTTGTTTTTTTTTTTT

TTGAGATGGAATCTCACACT

In100 candidate3721 (in intron):

LOCUS ENSG00000137269

DESCRIPTION Leucine-rich repeat-containing protein 1 (LAP and no PDZ protei) (LANO adapter protein). [Source:Uniprot/SWISSPROT;Acc:Q9BTT6]

CCCACTAATTAGCATTCTGTGAATCGCCTTTGATTGTTCAACACGTTTTAACCTCCCCGCCCCCATTTTATTCTTCAGCC

CATGTTTCTCCATTGTGTTC

In100 candidate3722 (in intron):

LOCUS ENSG00000137251

DESCRIPTION Tubulointerstitial nephritis antigen (TIN-Ag). [Source:Uniprot/WISSPROT;Acc:Q9UJW2]

CCAACTTTGGCATTTGCTGTTTAAATAACTTTGGGCAATTTTCTTAACCTTTCAGAATCTTCAAGTTGTTGTATTATTGT

TTCAAGTGTTTTTTTTTTCT

In100 candidate3723 (in intron):

LOCUS ENSG00000198873

DESCRIPTION G protein-coupled receptor kinase 5 (EC 2.7.11.16) (G proteincupled receptor kinase GRK5). [Source:Uniprot/SWISSPROT;Acc:P34947]

ATTAATATCTGATTTGTGATTCTGAATCTTTAACATATTTAACCGTCTGCTTTTTTTTTTTTTTTTTTTAATTCACAGAT

GACATGGTCTTTTTTTTTTT

In100 candidate3724 (in intron):

LOCUS ENSG00000198873

DESCRIPTION G protein-coupled receptor kinase 5 (EC 2.7.11.16) (G proteincupled receptor kinase GRK5). [Source:Uniprot/SWISSPROT;Acc:P34947]

AAAAAACAAAAAACAACTCTGTCCCCTTCTCCCTGTGGCCGTCACCTCTAACCTCTACGGCTGTTATTCTGGTTCAGTCT
CATTTCTTCTCTCGGACTTT

In100 candidate3725 (in intron):

LOCUS ENSG00000168143

DESCRIPTION family with sequence similarity 83, member B (FAM83B), mRNA [Sorce:RefSeq_dna;Acc:NM_001010872]

GGAGAAGGACTTACTATTGTCATTTTTTTTCCATATGTCTTGTAACTTCTTTGTTCTTTTTTCCCTCTTTTACTGCAGTC
TTTTGTGTTTTGTTTTGTTT

In100 candidate3726 (in intron):

LOCUS ENSG00000137252

DESCRIPTION Orexin receptor type 2 (Ox2r) (Hypocretin receptor type 2). [Sorce:Uniprot/SWISSPROT;Acc:O43614]

TGTCTTTTATTGGGATTTACTTTTCCTTTTTAACATTCCAACTTTTTTTTGCTGTATTTTTCTCTGTATCATTTTCAGTT
TTTTCCAATTTTCCAAATTA

In100 candidate3727 (in intron):

LOCUS ENSG00000124749

DESCRIPTION collagen, type XXI, alpha 1 precursor [Source:RefSeq_peptide;Ac:NP_110447]

AAATGATGACTAACTTTTTTGCAAACATATTCCCTTTGTTCCTTCTACTAATCAGTGTCAGTTATTTGCTTGGATTCCCC
CAGGCTATTTTCTCTGCTTC

In100 candidate3728 (in intron):

LOCUS ENSG00000198825

DESCRIPTION inositol polyphosphate-5-phosphatase F

[Source:RefSeq_peptide;Ac:NP_055752]

TAATTGGGGGAGACTTGAAATCTTTACAATATTGAATCTTGCTGCCTAGTAACATGGTATATGTTTCTGTTAATTCAGGT

TTTCTATCATATGCTTTATG

In100 candidate3729 (in intron):

LOCUS ENSG00000107651

DESCRIPTION SEC23-interacting protein (p125).

[Source:Uniprot/SWISSPROT;AccQ9Y6Y8]

ACTTACATTTCTATTATTAGTGAAGTTGAGCTCTTTGGGTGTATCTTAACTGGCTGATTATATTTCTTTTGTCAGTTGCC

TGTTCATGACCTTTGTGTTT

In100 candidate3730 (in intron):

LOCUS ENSG00000107651

DESCRIPTION SEC23-interacting protein (p125).

[Source:Uniprot/SWISSPROT;AccQ9Y6Y8]

CTTTTCTTTCTTATGGATTTATAAATATGATACTTAAGGATATTAACCTTTGTCATTATAAGTATTTTTTCCCAGTTTGA

AATTAGCCTTTTATTTTTCC

In100 candidate3731 (in intron):

LOCUS ENSG00000151914

DESCRIPTION Bullous pemphigoid antigen 1, isoforms 1/2/3/4/5/8 (230 kDa bulous pemphigoid antigen) (BPA) (Hemidesmosomal plaque protein) (Dystonia musulorum protein) (Dystonin) (Fragment). [Source:Uniprot/SWISSPROT;Acc:Q03001

TTTTTTTTTTTTTTACTTTTAATTGTATTTATTTTAACGCTGAATTTACTCCTGTGCTGTAAGTTTTTGTTTCTTCAGTT

TCTTCTGGGATATCTTTTTC

In100 candidate3732 (in intron):

LOCUS ENSG00000151914

DESCRIPTION Bullous pemphigoid antigen 1, isoforms 1/2/3/4/5/8 (230 kDa bulous pemphigoid antigen) (BPA) (Hemidesmosomal plaque protein) (Dystonia musulorum protein) (Dystonin) (Fragment). [Source:Uniprot/SWISSPROT;Acc:Q03001

CGTCCTTTCTTCTCCCTCCATATTATTTCTACACAACTCTAACACTTAATGTTTACCTTTTTTTTTTTTGAGATACAGCC

TTTTTCAAAGTTTTTCTCCA

In100 candidate3733 (in intron):

LOCUS ENSG00000151914

DESCRIPTION Bullous pemphigoid antigen 1, isoforms 1/2/3/4/5/8 (230 kDa bulous pemphigoid antigen) (BPA) (Hemidesmosomal plaque protein) (Dystonia musulorum protein) (Dystonin) (Fragment). [Source:Uniprot/SWISSPROT;Acc:Q03001

CTCCCATTCTGTAGGTTAACTGTTTACTCTGTTGATTCTATCTTTTGTTGTGCAGAAGCTTTTTTATTTAATTAAGTCCC

ATTTGTCTATATTTGTTTTT

In100 candidate3734 (in intron):

LOCUS ENSG00000203805

DESCRIPTION phosphatidic acid phosphatase type 2 domain containing 1A

[Soure:RefSeq_peptide;Acc:NP_001025230]

CATAACACCGAAATCTCTCTTATGTCCATGTCCAGTCAATCTTCATCCCCATGAGGCAGCCACTGTTCAGGGTTTTTTTT

TGTTTGTTTTTTGTTTTTTT

In100 candidate3735 (in intron):

LOCUS ENSG00000037637

DESCRIPTION F-box only protein 42. [Source:Uniprot/SWISSPROT;Acc:Q6P3S6]

GTTTTAGAAAAATGCACTCGAAGATAGAGATGTGTAACCTAGATGCCTGTTCTTGGCCTTGCTACTTCTCAGTTATTTGC

CTCTGAATTTAATCATTTTT

In100 candidate3736 (in intron):

LOCUS ENSG00000112200

DESCRIPTION Zinc finger protein 451 (Coactivator for steroid receptors).

[Surce:Uniprot/SWISSPROT;Acc:Q9Y4E5]

GGTTTAATGGTTCACTGCAAGAATAAATTTCTGATAACCTTATTTGTCCTTACTCCTTCCCTTTAAATATTACTGCAGCT

TTTTTTTTTTGTTGTTGTTG

In100 candidate3737 (in intron):

LOCUS ENSG00000146143

DESCRIPTION DNA primase large subunit (EC 2.7.7.-) (DNA primase 58 kDa

subuit) (p58). [Source:Uniprot/SWISSPROT;Acc:P49643]

AGCATGCCTGGCCTTCACTTTCATATCATTTATGTGTTAACTGGATTAGCACTTTGAATTTCCTTCAGGAACTTTTCCTT

TGCATTTACGACTTTGCTCA

In100 candidate3738 (in intron):

LOCUS ENSG00000112232

DESCRIPTION KH domain-containing, RNA-binding, signal transduction-associatd

protein 2 [Source:RefSeq_peptide;Acc:NP_689901]

GTCATATGATACATATTTGCTGATTAACTGATTTATAAAAGTTTTCCCTTGTTTTTTAAATTTTGTATTCTTCCAGAA

ATTATTTACCCTTCTTCCTT

In100 candidate3739 (in intron):

LOCUS ENSG00000112232

DESCRIPTION KH domain-containing, RNA-binding, signal transduction-associatd

protein 2 [Source:RefSeq_peptide;Acc:NP_689901]

TGTTATTTCTATCTACATAAATGAGTCAATTGTAACTTTCCAAAATCTGTTTCCTTTTATATTGCTACTATTTCTCAGAT

GTCTTTCATTCCCTTTCTTC

In100 candidate3740 (in intron):

LOCUS ENSG00000112232

DESCRIPTION KH domain-containing, RNA-binding, signal transduction-associatd

protein 2 [Source:RefSeq_peptide;Acc:NP_689901]

CATTAACTGAACCAACTCTAACTTTCTCATCTTGGATCATCTTTCTTTTCCTCCTGTATGTCATTTTCATAATACCAGAA

TACTTTTTCCATTCTAATTC

In100 candidate3741 (in intron):

LOCUS ENSG00000138162

DESCRIPTION Transforming acidic coiled-coil-containing protein 2 (Anti Zuai1)

(AZU-1). [Source:Uniprot/SWISSPROT;Acc:O95359]

TTTAACTTTATTTTTTCTTCTTTGTGCTTTTCTTAGTTTACTTTGTTTTTCCTTTCCAGTTTTTTGAGTTGGGAATTTAA

TTCATTATTTTCCTTCTTTC

In100 candidate3742 (in intron):

LOCUS ENSG00000117118

DESCRIPTION Succinate dehydrogenase [ubiquinone] iron-sulfur protein, mitocondrial precursor (EC 1.3.5.1) (Ip) (Iron-sulfur subunit of complex II). [Surce:Uniprot/SWISSPROT;Acc:P21912]

ATTAGTCAATTAACCTTCCGAGTTTTTACTTTTCTATATCAGTTTATTTTAAAAATTAGCAGTCCTGGAGTATAGTCTTT

TCTTTCTTTTTTTCTTTTTT

In100 candidate3743 (in intron):

LOCUS ENSG00000118482

DESCRIPTION PHD finger protein 3. [Source:Uniprot/SWISSPROT;Acc:Q92576]

TTTCTATCTAAATGTACATATGCATGCATTTTATTTGTGTGTTTGTTTCTAACAAGGATCACACTGTTTTCTGGTCAGCA

TTTTCTTCATAATCCATTCT

In100 candidate3744 (in intron):

LOCUS ENSG00000118482

DESCRIPTION PHD finger protein 3. [Source:Uniprot/SWISSPROT;Acc:Q92576]

ATTTTTCTTTAACGTGCTTAAATTTTCTCATATTTATCCTCCAACTTTTCAGTCAAGTTTTGTACTTCTGATTCTTAAAT

GTTCTTTTTGTTGTTGTTTT

In100 candidate3745 (in intron):

LOCUS ENSG00000188107

DESCRIPTION EGF-like-domain, multiple 11 [Source:RefSeq_peptide;Acc:NP_93804]

AAAAAACACAAAATTTAAAACTAATCTACTTTAACTTCACCATCTGCTTTATTTCTGTCTTCTATTTCTATCCTCCAGTT

AAATCAGTTTTGCTTTTTTT

In100 candidate3746 (in intron):

LOCUS ENSG00000135298

DESCRIPTION Brain-specific angiogenesis inhibitor 3 precursor.

[Source:Unipot/SWISSPROT;Acc:O60242]

ATCTAACATTTTTATTTGCTTTTCCAACTTTAATTACACTTCCCTTTCTCATGTTATTTTTCCAGTGGTACCTTTCTTGT

TAATAGATTGCCCTCTTTTT

In100 candidate3747 (in intron):

LOCUS ENSG00000135298

DESCRIPTION Brain-specific angiogenesis inhibitor 3 precursor.

[Source:Unipot/SWISSPROT;Acc:O60242]

TAATACCGAATAATATTGTTTCCTATCCTTTTCATCTCATAACCTCTCTTCCATAGTTCTACTTTTTTCCCCTCTCAGTT

ATCTATTTTAACTTACTTTC

In100 candidate3748 (in intron):

LOCUS ENSG00000168216

DESCRIPTION LMBR1 domain-containing protein 1 (Nuclear export signalinterating

protein) (HDAg-L interacting protein NESI).

[Source:Uniprot/SWISSPROT;cc:Q9NUN5]

TGACTGCTTAGACATTTATAATGTTTCCCCATTATAAATAACACTTTGATGAAAATTCTTGTCTGTGCATCTTTTCAGAT

TTGACCCTATCAGTCTTTTA

In100 candidate3749 (in intron):

LOCUS ENSG00000082293

DESCRIPTION Collagen alpha-1(XIX) chain precursor (Collagen alpha-1(Y) chai).

[Source:Uniprot/SWISSPROT;Acc:Q14993]

TAGTCCTACGTTCTCTTTTATTATTAACCCCAAATATGTTTCTTAGACATTCTCATTTATTATTCCCAGTTCTGCCTTCT

GTAAAATAAGTCTCTTTCCT

In100 candidate3750 (in intron):

LOCUS ENSG00000112280

DESCRIPTION Collagen alpha-1(IX) chain precursor.

[Source:Uniprot/SWISSPROTAcc:P20849]

AGCTATTTGTGTAATAACTGTAAATAATAGGCTTTAATTTTATGCTTTGGATTCCTTTTCTCTCTTTAGTTTACTCAGTC

TTTTTTTACATATTTTTTAA

In100 candidate3751 (in intron):

LOCUS ENSG00000112305

DESCRIPTION Stromal membrane-associated protein 1.

[Source:Uniprot/SWISSPRO;Acc:Q8IYB5]

AGATTTCAACTTTTCATTACCTGGTATGATTTATGTTAGGCTTTTGGATTAACTATATATTTGCTGTTGTTACCTCAGTC

ATTCTTATTTAATTTTTCAA

In100 candidate3752 (in intron):

LOCUS ENSG00000079841

DESCRIPTION Regulating synaptic membrane exocytosis protein 1 (Rab3-

interacing molecule 1) (RIM 1). [Source:Uniprot/SWISSPROT;Acc:Q86UR5]

GTATTGGTAGAAAGAGATGAAAACAAAAGTAAAGTAACCAAATGGCTTTTCCTTTTATATTTCACACAGCTTAGCAGATT

TATCTTCCTTGGCTATCTCT

In100 candidate3753 (in intron):

LOCUS ENSG00000079841

DESCRIPTION Regulating synaptic membrane exocytosis protein 1 (Rab3-

interacing molecule 1) (RIM 1). [Source:Uniprot/SWISSPROT;Acc:Q86UR5]

CTATCAATAACTACTAACTTCTCTTAGTATATCTTTCTCTTATATACTTTCATACCTATTTTCAGTTTTTCTCTTTGTTG

AAGTACTTTTTGTCAAACTT

In100 candidate3754 (in intron):

LOCUS ENSG00000079841

DESCRIPTION Regulating synaptic membrane exocytosis protein 1 (Rab3-

interacing molecule 1) (RIM 1). [Source:Uniprot/SWISSPROT;Acc:Q86UR5]

ATCATCACCACTAAACTATTTTAAGAAACAAAACAATACTATCACCTAACTTACTATTTCATTTCTATTATCTACCAGGA

TCATATATTCCCTTATTTTT

In100 candidate3755 (in intron):

LOCUS ENSG00000079841

DESCRIPTION Regulating synaptic membrane exocytosis protein 1 (Rab3-interacing molecule 1) (RIM 1). [Source:Uniprot/SWISSPROT;Acc:Q86UR5]

TAGGCCAAGCCCTCTCTTTTACTTCTATTAAAATAACTCTATTCTGGCTTTCCTGTTTTTCTGCCCTAATATTGCCAGTG

ACATCACTTGTTTTCTCTGT

In100 candidate3756 (in intron):

LOCUS ENSG00000185760

DESCRIPTION Potassium voltage-gated channel subfamily KQT member 5 (Voltagegated potassium channel subunit Kv7.5) (Potassium channel subunit alpha KvLT5) (KQT-like 5). [Source:Uniprot/SWISSPROT;Acc:Q9NR82]

TTTTCTGAAAATAGTAACCTTCGAAATGTGATTTTTTTAACATTGTTGCTCTCCAACCCTCATATTTATATTTTCAGTT

TAGTACATTTTTTCTTTATA

In100 candidate3757 (in intron):

LOCUS ENSG00000185760

DESCRIPTION Potassium voltage-gated channel subfamily KQT member 5 (Voltagegated potassium channel subunit Kv7.5) (Potassium channel subunit alpha KvLT5) (KQT-like 5). [Source:Uniprot/SWISSPROT;Acc:Q9NR82]

AATCATCTCATCTGAAATAACCTACCCACCTATTCTCTATCCCATTATTCATTTTTGTTTTCTTCATGTCACTATCAGAA

ATTTTTACTCACTTTTTTAT

In100 candidate3758 (in intron):

LOCUS ENSG00000185760

DESCRIPTION Potassium voltage-gated channel subfamily KQT member 5 (Voltagegated potassium channel subunit Kv7.5) (Potassium channel subunit alpha KvLT5) (KQT-like 5). [Source:Uniprot/SWISSPROT;Acc:Q9NR82]

ACCTTTTGGAAATATTGTTTTGGTAAATAAAAATTCCTGGTCGTGCTAACTGTGGCTATTTCGACCTGTTTCTTTCAGAT

ACCATTGTTCTTATCGCTTC

In100 candidate3759 (in intron):

LOCUS ENSG00000185760

DESCRIPTION Potassium voltage-gated channel subfamily KQT member 5 (Voltagegated potassium channel subunit Kv7.5) (Potassium channel subunit alpha KvLT5) (KQT-like 5). [Source:Uniprot/SWISSPROT;Acc:Q9NR82]

ATTGGAAACAACTATAGATTTCCAATAGTTGAAAATCGTTTAAAAATAACAAAGATCTATTCTTCATTTGTGTTTCAGTT

GTTTTTATTGTTTCTTTTTT

In100 candidate3760 (in intron):

LOCUS ENSG00000138161

DESCRIPTION CUB and zona pellucida-like domain-containing protein 1 precursr (Transmembrane protein UO-44). [Source:Uniprot/SWISSPROT;Acc:Q86UP6]

AAGACTGGAAATTTGCAATCAAAATGGATTTTACTGTAACTGAAAAAAATTGTCTGATTCTATCTTCAGGTCTTTCTAAT

ACAAGCTGTATTTCTCATCT

In100 candidate3761 (in intron):

LOCUS ENSG00000165775

DESCRIPTION FUN14 domain containing 2

[Source:RefSeq_peptide;Acc:NP_076423]EXON

CTTTTCTCTAGCTTCTTGAACATATGGAATATAATTGTAATAACTTTTAATGCTTTTGTCTACTAATTTTTTCATCAGTA

TCATTTCTGGGTTTCTTTTA

In100 candidate3762 (in intron):

LOCUS ENSG00000135314

DESCRIPTION Novel protein similar to chromosome 6 open reading frame 148.

[ource:Uniprot/SPTREMBL;Acc:Q5JSQ8]

TGGAGTCATATCTGAGAAATCATTGCCAAATCTAACATCATGAAGCTTTTGCCCTGTGTTTTCTTCTAACAGTTTTACAT

TTAGGTCTTTGATCCACTTT

In100 candidate3763 (in intron):

LOCUS ENSG00000164430

DESCRIPTION Uncharacterized protein C6orf150.

[Source:Uniprot/SWISSPROT;AccQ8N884]

CTAACTATATTAAACTGTGACTTCCTTTTTTTTTCGGTTAGGCCCATTTTTTGTCTCAGTGTACTTATTTTTATACATAA

TTATTTATCTGTTCACTTCT

In100 candidate3764 (in intron):

LOCUS ENSG00000185515

DESCRIPTION BRCA1/BRCA2-containing complex subunit 3.

[Source:Uniprot/SWISSROT;Acc:P46736]

ATGTATTATAGTTCTTGTTAGCATTTGTTTGCCTATTATAACTTTTTCCACTCTTTTATTTTTGACCTTCCTCAGTTATT

TTGTTTTAGGTATGCCTCTT

In100 candidate3765 (in intron):

LOCUS ENSG00000185515

DESCRIPTION BRCA1/BRCA2-containing complex subunit 3.

[Source:Uniprot/SWISSROT;Acc:P46736]

TGAGTTCAACCTGATAACTCCAATTCTATTTCCATACTTTAAGGTTCATCCTAGCATTCTCCTTTTTCATATTCACAGTT

CTTTTTATTAAGCTTTATTG

In100 candidate3766 (in intron):

LOCUS ENSG00000155959

DESCRIPTION Prefoldin subunit 3 (Von Hippel-Lindau-binding protein 1) (VHLinding

protein 1) (VBP-1) (HIBBJ46). [Source:Uniprot/SWISSPROT;Acc:P61758]

EXON

CACTCCCCAATTGTGCCACACCCTTTGCATTTCTACACTTTGTTCCTCCTAACTGGAATGACTTTCCCATATTTACAGGA

TGTGCTCTTTTTTTACCCTT

In100 candidate3767 (in intron):

LOCUS ENSG00000185973

DESCRIPTION Trimethyllysine dioxygenase, mitochondrial precursor (EC

1.14.1.8) (Epsilon-trimethyllysine 2-oxoglutarate dioxygenase) (TML-

alphaketogltarate

dioxygenase) (TML hydroxylase) (TML dioxygenase) (TMLD).

[Source:Unirot/SWISSPROT;Acc:Q9NVH6]

CCATTAACCACTACTAAATTTCTTAAATATTCCTTCTTTCCTAGTTTTCTTCCTATATATCTGACCATTTTTCCTCAGCC

TCCTTTGCTGGCTTCTTTTG

In100 candidate3768 (in intron):

LOCUS ENSG00000185973

DESCRIPTION Trimethyllysine dioxygenase, mitochondrial precursor (EC

1.14.1.8) (Epsilon-trimethyllysine 2-oxoglutarate dioxygenase) (TML-

alphaketogltarate

dioxygenase) (TML hydroxylase) (TML dioxygenase) (TMLD).

[Source:Unirot/SWISSPROT;Acc:Q9NVH6]

CTCTCTTGGGAAATACTTGTCTGTCAACTAAGAAGAAGGATAACTTTGTTAAATCTTCCTAGCTTTTCTTGTCAGATAAA

TGCCTGCTTTATTTGCTTTC

In100 candidate3769 (in intron):

LOCUS ENSG00000156535

DESCRIPTION CD109 antigen precursor (p180) (150 KDa TGF-beta-1-binding

protin) (r150) (Platelet-specific Gov antigen).

[Source:Uniprot/SWISSPROT;Acc:QYHK3]

GGCTGAATATTTTTCTGCTTATTAATAACTTATATTTCTACTTTTGTAAATTGCCTCTTCAGTTTTTTTTTTTTTTTGTA

AATTTAGGTCACTGACATTT

In100 candidate3770 (in intron):

LOCUS ENSG00000077549

DESCRIPTION F-actin capping protein subunit beta (CapZ beta).

[Source:Uniprt/SWISSPROT;Acc:P47756]

GGTTATTGCTTCTTTGTTAACACTGGGCGCTTTTCTCCCTTAATTTCTAATTTAGTCAGCAGTACATCGTCCCTCATTTT

TCACTTGTATGTCTTCACTT

In100 candidate3771 (in intron):

LOCUS ENSG00000065154

DESCRIPTION Ornithine aminotransferase, mitochondrial precursor (EC 2.6.1.1)

(Ornithine--oxo-acid aminotransferase) [Contains: Ornithine aminotransferse,

hepatic form; Ornithine aminotransferase, renal form].

[Source:Uniprot/WISSPROT;Acc:P04181]

AACACTCATACAAGTAACGGATTGTAGTTTGTAGTTTGACTCTCTCTAGTTTCTCAGTTACCTTTTGCTAACAATAGCTT

TTTCGTTTGTTTGTTTGTTT

In100 candidate3772 (in intron):

LOCUS ENSG00000065154

DESCRIPTION Ornithine aminotransferase, mitochondrial precursor (EC 2.6.1.1)

(Ornithine--oxo-acid aminotransferase) [Contains: Ornithine aminotransferse,

hepatic form; Ornithine aminotransferase, renal form].

[Source:Uniprot/WISSPROT;Acc:P04181]

TTTTATTTTGGCATAGTTTTAGACTTAAGAAAAGTTGTAACAAATAGTACTGACATTTTCCTTTTATACTTCTCCCAGAT

TTTCCAAGTGCTAACTTTTT

In100 candidate3773 (in intron):

LOCUS ENSG00000111799

DESCRIPTION Collagen alpha-1(XII) chain precursor.

[Source:Uniprot/SWISSPRO;Acc:Q99715]

TTTTTCACTTATTTTTTTGTAACAAGGTGTCCCATCTTCTATCTTTGCATCCATTCCTTTTATGATACTTTCCATCAGCC

ACTGTCATTAATTTATCTTC

In100 candidate3774 (in intron):

LOCUS ENSG00000019995

DESCRIPTION Zinc finger Ran-binding domain-containing protein 1 (Protein

TRBID). [Source:Uniprot/SWISSPROT;Acc:Q9UGI0]

GAATGATTACTAAGTTTAAACTTTGTGTGGAACAATAGCTAAGCTTTAACTATACCTTAGCTGTATGTTTTCCCTCAGTA

CCAATTTCTTTTATTTATCC

In100 candidate3775 (in intron):

LOCUS ENSG00000175029

DESCRIPTION C-terminal-binding protein 2 (CtBP2).

[Source:Uniprot/SWISSPROTAcc:P56545]

CCAATTATCAATACATTAATATTAACTGAAGTCTACATTGTATTCTGATGATCTGATATCCTTTTTCCATCCCAGAATCC

TGTCCAGTTTTTCACCTTGT

In100 candidate3776 (in intron):

LOCUS ENSG00000203780

DESCRIPTION Fibronectin type 3 and ankyrin repeat domains protein 1.

[Sourc:Uniprot/SWISSPROT;Acc:Q8TC84]

CTCTAACTTACTATTTTTGTTGTTGTTTTTACCTTTCCAGGGACTTTGTTTCAAGGCCTTTCTTTCTCTGCCCCGCCTAA

GCTGTCTTATTGTTCCTTTT

In100 candidate3777 (in intron):

LOCUS ENSG00000075151

DESCRIPTION Eukaryotic translation initiation factor 4 gamma 3 (eIF-4-gamma3) (eIF-4G 3) (eIF4G 3) (eIF-4-gamma II) (eIF4GII). [Source:Uniprot/SWISSPRT;Acc:O43432]

GATGCGGAAAAACACTAGACCTAACTCTCAAGTGTTTCTTTTGGTTGCCATCTCTCAGTGGCCCATCATTCTTACATATT

TCATCGCCATTGTTATTCTT

In100 candidate3778 (in intron):

LOCUS ENSG00000112701

DESCRIPTION Sentrin-specific protease 6 (EC 3.4.22.-) (Sentrin/SUMO-specifi protease SENP6) (SUMO-1-specific protease 1). [Source:Uniprot/SWISSPROT;Ac:Q9GZR1]

TCCTGAAGTAACTTTTTTCTGCCTTCCTTTCTAATATACATATACTACTACTTTAGTTTGTTTTCCTTTTCATTTCAGCC

TGTCTTCCTTTATGGACCTT

In100 candidate3779 (in intron):

LOCUS ENSG00000112701

DESCRIPTION Sentrin-specific protease 6 (EC 3.4.22.-) (Sentrin/SUMO-specifi protease SENP6) (SUMO-1-specific protease 1). [Source:Uniprot/SWISSPROT;Ac:Q9GZR1]

AGAATAACAACCGTTAAGATCTCATTTTTCTCTCCACCTACTCTCTCATTTGTTTCTTAAACTCCCTTTTTTTTCAGCCT

GTTTTTTGTATCCTTGTTAG

In100 candidate3780 (in intron):

LOCUS ENSG00000162551

DESCRIPTION Alkaline phosphatase, tissue-nonspecific isozyme precursor (EC .1.3.1) (AP-TNAP) (TNSALP) (Alkaline phosphatase liver/bone/kidney isozyme) [Source:Uniprot/SWISSPROT;Acc:P05186]

TTTTAACCATTCCTCTTTCTGTTTTGCTAACATCTTTGCTCATTTCTTTTGCATGTTTCTCACAGTTTATGGAGACTTTT
TGTTTGCTTCATTTTGTTTC

In100 candidate3781 (in intron):

LOCUS ENSG00000187609

DESCRIPTION HBE269. [Source:Uniprot/SPTREMBL;Acc:Q8NHD1]

ATACTAGTTATGGGTAATCTTACCCATATAGTTTTTAATAACATTTATTCCCTCTTTTTTTCCTTCAGTAAGGAGTGAA
TTTTTTTTTTTTTTTTTTTT

In100 candidate3782 (in intron):

LOCUS ENSG00000187609

DESCRIPTION HBE269. [Source:Uniprot/SPTREMBL;Acc:Q8NHD1]

CTCCTGTGTAACTAGTCTGTTTCTTCCCTTTGACCCGTTTCCTTTGGTTTTTTCCCGTATATTTACTTTATTTACCAGTT
GTGTTTGTTTTTACTTTTGT

In100 candidate3783 (in intron):

LOCUS ENSG00000146247

DESCRIPTION pleckstrin homology domain interacting protein

[Source:RefSeq_pptide;Acc:NP_060404]

TTTCCATTGGAAATTTATACCTTTGCAAAAGATAATGGAAATCATGTAACATATAATTTAGTATTTTGTATTCATCAGCA

TTTTACTGCATTTTTTGTGT

In100 candidate3784 (in intron):

LOCUS ENSG00000165752

DESCRIPTION Serine/threonine-protein kinase 32C (EC 2.7.11.1) (PKE) (YANK3)

[Source:Uniprot/SWISSPROT;Acc:Q86UX6]

AGTATCTCTTTTCACAAATAAAACATTTACTAACCATTTGAGCGTATTCCCTCTTGCTTGTTATTGTTGCCTCAGGTTTT

TGTTTCACATTTTTCATGCT

In100 candidate3785 (in intron):

LOCUS ENSG00000118418

DESCRIPTION High mobility group nucleosome-binding domain-containing protei 3

(Thyroid receptor-interacting protein 7) (TRIP-7).

[Source:Uniprot/SWISSROT;Acc:Q15651]

ACATCCCCTTGGTTCCTTTTTCCTTATCATCCCCCCTAAAGATAACCATCATCACGAATTTGTTGTGTATTCTTCCAGTT

CTTGCTTTTATACCTTTATG

In100 candidate3786 (in intron):

LOCUS ENSG00000148408

DESCRIPTION Voltage-dependent N-type calcium channel subunit alpha-1B

(Voltge- gated calcium channel subunit alpha Cav2.2) (Calcium channel, L type,

apha-1 polypeptide isoform 5) (Brain calcium channel III) (BIII).

[Source:Unprot/SWISSPROT;Acc:Q00975]

TCTACTTTTTGTTGCTATCACTGGTTTTGGTTTTTAACTTGCATTTGCCTGGTACCTTTCTTTTCCTTTCATTTCCAGCT

CTTCTTTATCATTTTAAGTG

In100 candidate3787 (in intron):

LOCUS ENSG00000184677

DESCRIPTION Zinc finger and BTB domain-containing protein 40.

[Source:Uniprt/SWISSPROT;Acc:Q9NUA8]

TCTGAAAATATTGAATATTTCTATCTAACAACATGGGATATCTCTCCATTTTTAAAGTTCTTTGATATCTTTCATCAGAG

TTTTGTTGTTTTCTTCATAT

In100 candidate3788 (in intron):

LOCUS ENSG00000118420

DESCRIPTION UBCH10 binding protein with a hect-like domain

[Source:RefSeq_pptide;Acc:NP_944602]

TTCCAAAAACCTATCAACAACATTAACTGGGGACTTACTGTACTTTATCTCCAATTCTCCAGTCTTTTCATATCAATTAG

AATTCACCTTTCTGCACCTT

In100 candidate3789 (in intron):

LOCUS ENSG00000083097

DESCRIPTION dopey family member 1 [Source:RefSeq_peptide;Acc:NP_055833]

CTTCCCACTATGGCTTCTCCATCTCTTAACCATTTTTCATATTTCTAACTTTTTGTCCTTATGTGCTTCATTCTCAGGA

ATTTCTTCTGACCTAGTTTC

In100 candidate3790 (in intron):

LOCUS ENSG00000169641

DESCRIPTION Leucine zipper protein 1.

[Source:Uniprot/SWISSPROT;Acc:Q86V48]EXON

TAAAATGGATTGGTCACTTCTGGTCTTGACTCTGGTTAAATCTATAACAAGTAAAAAGTCTTATGTTGTTTTCAGAGTGG

ACTTTCTTAATTTTTATTTT

In100 candidate3791 (in intron):

LOCUS ENSG00000125944

DESCRIPTION Heterogeneous nuclear ribonucleoprotein R (hnRNP R).

[Source:Unprot/SWISSPROT;Acc:O43390]

TGTTTCTTTTCTCTTGATAAAAAGGTTATAACTTGATAAAAAGTTGTTATAACTGCTTTCCTCCTTTTGAATATTCAGTT

ACCTCAGTTTCTTTATTTAT

In100 candidate3792 (in intron):

LOCUS ENSG00000203877

DESCRIPTION OTTHUMP00000016800 (Chromosome 6 open reading frame 159).

[Soure:Uniprot/SPTREMBL;Acc:Q5TAB7]

GCTGTTCATAATATTCTCTTATCTTTTTAACATCCATAAGATCTGTAGTGGTCTCCCCTCTTTAATTTCTGTTATCAGTA

ATTTGTGTCCTCTTTTTTTT

In100 candidate3793 (in intron):

LOCUS ENSG00000203877

DESCRIPTION OTTHUMP00000016800 (Chromosome 6 open reading frame 159).

[Soure:Uniprot/SPTREMBL;Acc:Q5TAB7]

CACTTTCCACAATGGTTATGCTAATTAACACTCCCACCAATGTGTATAAGTGTTTCCTTTTCTCCTTGACCTTGCCAGCC

TCTGTTATTTTTTTAGTTTT

In100 candidate3794 (in intron):

LOCUS ENSG00000203877

DESCRIPTION OTTHUMP00000016800 (Chromosome 6 open reading frame 159).

[Soure:Uniprot/SPTREMBL;Acc:Q5TAB7]

GCTCTGGAAGCATTTATCTTTTCATTTAAGACCATTTATAACTAGAAACTAAGCTTTAGTGCTTAGTTCCTTCAGCTGAC

TTTAGTCTTCCATGATTTCT

In100 candidate3795 (in intron):

LOCUS ENSG00000065615

DESCRIPTION cytochrome b5 reductase 4

[Source:RefSeq_peptide;Acc:NP_057314]EXON

TGGTCTGTTTTACTTTCTACATATTGAACTTCCTTAACTGTTCTTCTAATTATCTTATCTTTTCTCTCTTATTTTCAGTT

TTGTCCTTTTGTTCTACTTT

In100 candidate3796 (in intron):

LOCUS ENSG00000135317

DESCRIPTION Sorting nexin-14. [Source:Uniprot/SWISSPROT;Acc:Q9Y5W7]

AGATTCTGTACGTTTTTCCTATTTGGTGTCTAACTCATGTCTTCCTCTTCATTCCCTTTACTGTTTCCTTAGTTCAGTTT

TCATTGCCTCTTATTTGAAC

In100 candidate3797 (in intron):

LOCUS ENSG00000188822

DESCRIPTION Cannabinoid receptor 2 (CB2) (CB-2) (CX5).

[Source:Uniprot/SWISPROT;Acc:P34972]

CCTTCATAACTCTTAACTTCTCTTTCTTGTTTTCCATCACTTTATCTCTTTGTTCTGCATTGTGAGAAATTTCTTCAGAG

CTACATTCTTTTTTTTTTTT

In100 candidate3798 (in intron):

LOCUS ENSG00000168830

DESCRIPTION 5-hydroxytryptamine 1E receptor (5-HT-1E) (Serotonin receptor 1)

(5- HT1E) (S31). [Source:Uniprot/SWISSPROT;Acc:P28566]

ACATTTTATTATTATAATTTCTACTTTAACAAGTAACTATACCTTTCACTGTGTTTTATTTAGCACCTTTCACAGTGTTG

TTGTCTTTTTTAATATACCT

In100 candidate3799 (in intron):

LOCUS ENSG00000164414

DESCRIPTION Uncharacterized protein C6orf165.

[Source:Uniprot/SWISSPROT;AccQ8IYR0]

TATCTTCCACAATGGTTGAACTAATTTACACTCCCACTAACTATAAAAGCATTGCTATTTCTTCACAGCCTCACCAGCAT

CTGTTGTTTCTTCACTTTTT

In100 candidate3800 (in intron):

LOCUS ENSG00000164414

DESCRIPTION Uncharacterized protein C6orf165.

[Source:Uniprot/SWISSPROT;AccQ8IYR0]

TCTATTATATAGCTACCATATTTGAATTCAATTATAACCTCCTTAAGCCTTTTGGTATTTGCTTCCTCACAGCCTTTTTA

TTTAATTAAGTTCATACTTT

In100 candidate3801 (in intron):

LOCUS ENSG00000146282

DESCRIPTION Probable arginyl-tRNA synthetase, mitochondrial precursor (EC 61.1.19) (Arginine--tRNA ligase) (ArgRS) (Arginyl-tRNA synthetase-like).

[Sorce:Uniprot/SWISSPROT;Acc:Q5T160]

CTGACTGTAACATTTTTATTTTATAAACTTTTCATTTTTTAAACTTTTTTCTTCTATTTATTTTTCTTTATATCTCAGCT

TGTTTAACTTTTCAGCTGTT

In100 candidate3802 (in intron):

LOCUS ENSG00000146282

DESCRIPTION Probable arginyl-tRNA synthetase, mitochondrial precursor (EC 61.1.19) (Arginine--tRNA ligase) (ArgRS) (Arginyl-tRNA synthetase-like).

[Sorce:Uniprot/SWISSPROT;Acc:Q5T160]

AATAATTTCCACCTTATAAACATTAAATAACTGTATTTTTACTAGTTATCTGCTCTCAGTGGCTTAGTTTTCAAAATTCT

TAATCCACATCTCCTTTTTC

In100 candidate3803 (in intron):

LOCUS ENSG00000111880

DESCRIPTION mRNA capping enzyme (HCE) (HCAP1) [Includes: Polynucleotide 5'-triphosphatase (EC 3.1.3.33) (mRNA 5'-triphosphatase) (TPase); mRNA guanylytransferase (EC 2.7.7.50) (GTP--RNA guanylyltransferase) (GTase)]. [Source:niprot/SWISSPROT;Acc:O60942]

AGCCTAAGTCCTTAACAACATGATGGGTTGTGTGTCTTCCATGCCATTTTTGCAGATGATGTCATCATTGCTTTTTATTT

GATCTTCCCTTTTGTTGATT

In100 candidate3804 (in intron):

LOCUS ENSG00000111880

DESCRIPTION mRNA capping enzyme (HCE) (HCAP1) [Includes: Polynucleotide 5'-triphosphatase (EC 3.1.3.33) (mRNA 5'-triphosphatase) (TPase); mRNA guanylytransferase (EC 2.7.7.50) (GTP--RNA guanylyltransferase) (GTase)]. [Source:niprot/SWISSPROT;Acc:O60942]

CTCTTTGTTAACCTGAACTGTCTATTAACTTTTCATTATATTATTGGTTACCTTTCCTTTCCTTATATTCATAATCAGAT

ATATTTCTCTGTTATTACTT

In100 candidate3805 (in intron):

LOCUS ENSG00000111880

DESCRIPTION mRNA capping enzyme (HCE) (HCAP1) [Includes: Polynucleotide 5'-triphosphatase (EC 3.1.3.33) (mRNA 5'-triphosphatase) (TPase); mRNA guanylytransferase (EC 2.7.7.50) (GTP--RNA guanylyltransferase) (GTase)]. [Source:niprot/SWISSPROT;Acc:O60942]

GAAATTACTTTTTTTTTAACTGACCTGTTTCTTGTTTTTTTTGTATTATTTTTGTTTTTATTCACCTTCTACATACAGTA

ATTCACTCCTTTTGGTGTTT

In100 candidate3806 (in intron):

LOCUS ENSG00000111880

DESCRIPTION mRNA capping enzyme (HCE) (HCAP1) [Includes: Polynucleotide 5'-triphosphatase (EC 3.1.3.33) (mRNA 5'-triphosphatase) (TPase); mRNA guanylytransferase (EC 2.7.7.50) (GTP--RNA guanylyltransferase) (GTase)]. [Source:niprot/SWISSPROT;Acc:O60942]

AGATTTTAAAAAGCAATGTTTCATTGCCTAATTTTATATTTGGTTAACAATTTTACTGATTGTTCTATGTCCCAGGCACT

TTCAATTATTATTCTTTCAG

In100 candidate3807 (in intron):

LOCUS ENSG00000117616

DESCRIPTION C1orf63 protein. [Source:Uniprot/SPTREMBL;Acc:Q49AA4]

AAATGGGCATTTCTAACTCCAAGTTTGCTAAGACTTTTGTTTCTAAAATATTATTTTCCCTCTGCCTGTTCCCTTCAGAC

CTATTTGAGATTCTTTTTCA

In100 candidate3808 (in intron):

LOCUS ENSG00000135299

DESCRIPTION Ankyrin repeat domain-containing protein 6. [Source:Uniprot/SWISPROT;Acc:Q9Y2G4]

AGCTATTTCTTCTATATGTAACTCTGTTTACATTCTTTTCATATTTTACTTCCAGTGTTAATCACTTTTCATTTCTTTGC

TACACTTTCATCTTTGTTGG

In100 candidate3809 (in intron):

LOCUS ENSG00000135299

DESCRIPTION Ankyrin repeat domain-containing protein 6.

[Source:Uniprot/SWISPROT;Acc:Q9Y2G4]

AGCCTCCCATATTCTTATTGTTAACATTAAATTACACTTGCCTTAAATCTCTTTTTATTCAGTTTCTTCATTGGTTGTTA

GATGTTTGTAACTTTTTTCC

In100 candidate3810 (in exon):

LOCUS ENSG00000112182

DESCRIPTION Transcription regulator protein BACH2 (BTB and CNC homolog 2).

Source:Uniprot/SWISSPROT;Acc:Q9BYV9]

CATTCCTCTTCTTTTGTCTCTGATGCTAGAACTAGTTTGGTAACTGTTAACATGGTCATTTTTCTTGCTTCACAGTTCAA

TTTTCAATTCGTACTTATTT

In100 candidate3811 (in intron):

LOCUS ENSG00000135341

DESCRIPTION Mitogen-activated protein kinase kinase kinase 7 (EC

2.7.11.25)(Transforming growth factor-beta-activated kinase 1) (TGF-

betaactivated

knase 1). [Source:Uniprot/SWISSPROT;Acc:O43318]

TGAGGCTATTATCTTAGTTACCTGCCTTAACTAATGAAAAAATCACTTAGCCTTTTTTTCTTCATTGTGAATATTCAGTC

ATCCTTTTTCCACTATATTA

In100 candidate3812 (in intron):

LOCUS ENSG00000135333

DESCRIPTION Ephrin type-A receptor 7 precursor (EC 2.7.10.1) (Tyrosine-protin

kinase receptor EHK-3) (EPH homology kinase 3) (Receptor protein- tyrosie kinase HEK11). [Source:Uniprot/SWISSPROT;Acc:Q15375]

GTAGCTTTTCTGGGAGTAGTTGGTGAGCTCGTCTAACTTTCCTGTATGTATGTATCTCTCAGATATGCCACTTTACCCTG

CTTTATCTCATTGAGTATTT

In100 candidate3813 (in intron):

LOCUS ENSG00000112214

DESCRIPTION Four and a half LIM domains protein 5 (FHL-5) (Activator of

cAMresponsive

element modulator in testis) (Activator of CREM in testis).

[Surce:Uniprot/SWISSPROT;Acc:Q5TD97]

TTTATTTTTGTGTAACTGCATGTTAGTCGAGGTCTTTGCTCCTTATTCTTTCTTTTTGTCTTCAGTTTTCCTAAACCTTC

CGTTAGAGAGTTTCCTTTTT

In100 candidate3814 (in intron):

LOCUS ENSG00000112214

DESCRIPTION Four and a half LIM domains protein 5 (FHL-5) (Activator of

cAMresponsive

element modulator in testis) (Activator of CREM in testis).

[Surce:Uniprot/SWISSPROT;Acc:Q5TD97]

TTAACCTTTCTAGTTTCCTGCTTTCTTTTTAGTTTTAGCCTCTGAGATTTTCCCTTACTTTCTTGCCAGTTCGGCAACAC

ATTTTATTTTGTTCGTTTTT

In100 candidate3815 (in intron):

LOCUS ENSG00000186231

DESCRIPTION Kelch-like protein 32 (BTB and kelch domain-containing protein).

[Source:Uniprot/SWISSPROT;Acc:Q96NJ5]

ATACTTTGTATTTCTGAATTTTATGAAATGAGCATTTATTACTTTTTATAACTAGAAATGGCACTCTTTCTATAACAGCA

GTAATTTTTGTTTCCCATCC

In100 candidate3816 (in intron):

LOCUS ENSG00000186231

DESCRIPTION Kelch-like protein 32 (BTB and kelch domain-containing protein).

[Source:Uniprot/SWISSPROT;Acc:Q96NJ5]

AGTAGCTAACTCTACTGACTGATTATTTTCCTGATGCCTTTCTGCCTTATTTCATTATTTGTGTTACTTTAATCAGTAAC

TTCCCTCTTCATTATTGCTT

In100 candidate3817 (in intron):

LOCUS ENSG00000186231

DESCRIPTION Kelch-like protein 32 (BTB and kelch domain-containing protein).

[Source:Uniprot/SWISSPROT;Acc:Q96NJ5]

TGGGTTTTTCTAGTATTAATTAAAATAATGTAAAATTTTATTAACATTTAAAAATATAATTGCCTTATCTTTTTGCAGAT

ATTGTTTTACTTACATCTTG

In100 candidate3818 (in intron):

LOCUS ENSG00000130695

DESCRIPTION Coiled-coil domain-containing protein 21.

[Source:Uniprot/SWISSROT;Acc:Q6P2H3]

CCTCCATTCACTCCCTTTCTAAGCTTTATAACTGGTGTGTTACTTCTTGTACATTCACTTCTCTCATTTTTATCCCAGGA

AATTTCTGTCTCCTTCAGTT

In100 candidate3819 (in intron):

LOCUS ENSG00000112249

DESCRIPTION Activating signal cointegrator 1 complex subunit 3 (EC 3.6.1.-)(ASC-1 complex subunit p200) (Trip4 complex subunit p200) (Helicase, ATP biding 1). [Source:Uniprot/SWISSPROT;Acc:Q8N3C0]

AAGTCTGTTATTTTGGCTTTCCCCCTCATAGTTTACCTCTTGTAATAACCTTGTCTAGGTCCTTTAAGTCTCAGCTCAAG
CTCAGCTTCCTTCTTTATAT

In100 candidate3820 (in intron):

LOCUS ENSG00000112249

DESCRIPTION Activating signal cointegrator 1 complex subunit 3 (EC 3.6.1.-)(ASC-1 complex subunit p200) (Trip4 complex subunit p200) (Helicase, ATP biding 1). [Source:Uniprot/SWISSPROT;Acc:Q8N3C0]

TTCTCAAACCTGTTTTTTTTTTAACTTTTGATTCTACTCATTTTTGATGCTTAATACTACTCATTGTTTCCTCAGTCTTG
AAACTCTTTGCTCTTTGACT

In100 candidate3821 (in intron):

LOCUS ENSG00000112249

DESCRIPTION Activating signal cointegrator 1 complex subunit 3 (EC 3.6.1.-)(ASC-1 complex subunit p200) (Trip4 complex subunit p200) (Helicase, ATP biding 1). [Source:Uniprot/SWISSPROT;Acc:Q8N3C0]

TTAAATCTGTAGATCAATTTGGGAGAATTAAGGTATAACCCTTCATTTAGTTGGATCCTCTTTATTTTTTCCTAGCAGTG

TTTTCTTTGTTTTGTTTTCA

In100 candidate3822 (in intron):

LOCUS ENSG00000117713

DESCRIPTION AT-rich interactive domain-containing protein 1A (ARID

domainontaining

protein 1A) (SWI/SNF-related, matrix-associated, actindependentregulator

of chromatin subfamily F member 1) (SWI-SNF complex protein

p270)(B120) (SWI-like protein) (Osa homolog [Source:Uniprot/SWISSPROT;Acc:O14497

CTATGTCTGATTAACAAGTTCCTAAATGGTCTCTACCTCTTCTCATTTCCCACTTTATTGTCTTTGGTCTGGAGACAGAT

TCCTTCATCTGTTTTTGGGG

In100 candidate3823 (in intron):

LOCUS ENSG00000164418

DESCRIPTION Glutamate receptor, ionotropic kainate 2 precursor (Glutamate

rceptor 6) (GluR-6) (GluR6) (Excitatory amino acid receptor 4) (EAA4).

[Soure:Uniprot/SWISSPROT;Acc:Q13002]

AGGAGATTCAACTTGCAATAAAATTCATAACAAGTGCTATGAATCTTGCTCTATCCAAATTTTTTTCTTCAGAATTTTT

TTTATTTTTTTTATTATAC

In100 candidate3824 (in intron):

LOCUS ENSG00000164418

DESCRIPTION Glutamate receptor, ionotropic kainate 2 precursor (Glutamate

rceptor 6) (GluR-6) (GluR6) (Excitatory amino acid receptor 4) (EAA4).

[Soure:Uniprot/SWISSPROT;Acc:Q13002]

TAGTCTAATATTTGAAAATAATAAAAACTGATTATAATAAACATGTTCATAACATGCACTGTTAATTATTCTTTCCAGAT

TTCTACTGACTTTAAATTTT

In100 candidate3825 (in intron):

LOCUS ENSG00000164418

DESCRIPTION Glutamate receptor, ionotropic kainate 2 precursor (Glutamate

rceptor 6) (GluR-6) (GluR6) (Excitatory amino acid receptor 4) (EAA4).

[Soure:Uniprot/SWISSPROT;Acc:Q13002]

ATTAAGCAATATATAATATTAGAGATTCTTGGTAAAGTAATAACACAAGGATAAATATTTTCTGGATTTTCTCAGAATAT

TTTGTTCAATTTTCAATCTT

In100 candidate3826 (in intron):

LOCUS ENSG00000142784

DESCRIPTION WD and tetratricopeptide repeats protein 1.

[Source:Uniprot/SWISPROT;Acc:Q8N5D0]

TTGCTCATCTTACTCTGTCCCCTTCATCACAATGCTCTAACCACATTGGCTTTCATTCTGTTTCTTTGGCATTATCAGGC

CTGTTATTTTTATACTCATT

In100 candidate3827 (in intron):

LOCUS ENSG00000057663

DESCRIPTION Autophagy protein 5 (APG5-like) (Apoptosis-specific protein).

[ource:Uniprot/SWISSPROT;Acc:Q9H1Y0]

TGAGGTTAGAGAAGAATTTCTGTTTTTAATATGTAACCAAGACTAGTTGGCCTTTCCTGTTTAGCATTTTTTTTCAGGT

AATTTTTAATCTTTTAGTTT

In100 candidate3828 (in intron):

LOCUS ENSG00000130348

DESCRIPTION glutaminyl-tRNA synthase (glutamine-hydrolyzing)-like 1

[SourceRefSeq_peptide;Acc:NP_060762]

TGACGGAGTGAGACTCTGTCTCCAAAAAAAAAAAAAAACATATTTATAGTAACTGTTTTAATGTTCTTATCTACTCAGTA

TTCCATCATTTTTGCATCTG

In100 candidate3829 (in intron):

LOCUS ENSG00000164494

DESCRIPTION Decaprenyl-diphosphate synthase subunit 2 (EC 2.5.1.-) (Decapreyl

pyrophosphate synthetase subunit 2) (Candidate tumor suppressor protein)

[Source:Uniprot/SWISSPROT;Acc:Q86YH6]

CTTTTCTTTGTTATCTGTTCCTTGTATTATTCAATTTAACCATACTTTGAATCTACTATTATAGCTCTTTTCTTACAGCT

CGTATTTCTTTGTATACCCT

In100 candidate3830 (in intron):

LOCUS ENSG00000164494

DESCRIPTION Decaprenyl-diphosphate synthase subunit 2 (EC 2.5.1.-) (Decapreyl

pyrophosphate synthetase subunit 2) (Candidate tumor suppressor protein)

[Source:Uniprot/SWISSPROT;Acc:Q86YH6]

AGCAAATAACTTGGTCTCTGGCTTTTTTGGAGTCTGTCTCAGGTCTTTTCTCCCCAGTTAAGGATTTTTTTTCCTTCACT

GCCTCTTCTTTGATTTTTGA

In100 candidate3831 (in intron):

LOCUS ENSG00000080546

DESCRIPTION Sestrin-1 (p53-regulated protein PA26).

[Source:Uniprot/SWISSPRT;Acc:Q9Y6P5]

GGTGTGAGCCACCATACCTGGCCACTTCTTTGTTAACAATAAGTTGACCATATTTTTATCAGTCTATTTCTGGGCTCTCT

ATTCTGTTCCATTGATATGT

In100 candidate3832 (in intron):

LOCUS ENSG00000080546

DESCRIPTION Sestrin-1 (p53-regulated protein PA26).

[Source:Uniprot/SWISSPRT;Acc:Q9Y6P5]

TCATCTTAACATGTCTTTTATTTTCTCTCTCATAAATTTCATTTCTTTGCTGTATGTGCTATATTCTGGACTCTTCAGAT

TTACTTTCTAGTTTACTAAT

In100 candidate3833 (in intron):

LOCUS ENSG00000080546

DESCRIPTION Sestrin-1 (p53-regulated protein PA26).

[Source:Uniprot/SWISSPRT;Acc:Q9Y6P5]

ACTGAAACTTATTTTCATTTATGAGTCCTGTTCTGTGGCCATAACTACTGCTATAAATTCCACTTGTTTCCTTGTCAGAA

TGTTCCAACTTTTTTTGTTT

In100 candidate3834 (in intron):

LOCUS ENSG00000158161

DESCRIPTION Eyes absent homolog 3 (EC 3.1.3.48).

[Source:Uniprot/SWISSPROT;cc:Q99504]

TATTTTGGTAACTCCACATCTTTTTGCATTTTCTTTCTCACAAACATTAGCCTTTCTTTTGTTAGTTTTCATATACAGCC

TGAGTCTCTTTATCTTTTCC

In100 candidate3835 (in intron):

LOCUS ENSG00000112365

DESCRIPTION Zinc finger and BTB domain-containing protein 24 (Zinc finger

potein 450). [Source:Uniprot/SWISSPROT;Acc:O43167]

CTCTCTAACTTTATTTTCTCTTATATTTTCCATCATTTTTGGCCCCACCCTCTGGGGGATTTCCACTTTGTTTTTCAGCC

CTTTGAGGTTTTCTGTTTCA

In100 candidate3836 (in intron):

LOCUS ENSG00000112367

DESCRIPTION SAC domain-containing protein 3.

[Source:Uniprot/SWISSPROT;Acc:92562]

GTTAGGTGCTTTGTGAACGAACATTTTCTCGCTTATTCTTGTGCCTGTAACAAACATACCATCTTCTTCCCTTTTCAGTT

CTTTGCAATTGCTATTTCCT

In100 candidate3837 (in intron):

LOCUS ENSG00000168438

DESCRIPTION Pre-mRNA-processing factor 17 (PRP17 homolog) (hPRP17) (Cell

diision cycle 40 homolog) (EH-binding protein 3) (Ehb3).

[Source:Uniprot/SWISPROT;Acc:O60508]

TCTTTTTTTGTATAGTTTTATTTAATAACCCTAGGCTTTCTCACCCCTTAATCTTTCTTTATTCTGTAATTACCACAGAT

AAGTCTCTTTCTCCTATTTC

In100 candidate3838 (in intron):

LOCUS ENSG00000159023

DESCRIPTION Protein 4.1 (Band 4.1) (P4.1) (EPB4.1) (4.1R).

[Source:Uniprot/WISSPROT;Acc:P11171]

TCTACGCACTAACTGCTTATAACTGCTTAGTTGATCTTCTTCCCTCTAGTCTTTTTCCTTTTGCCATATTCATTCCAGTT

TATCTTGCCATACTCATCTT

In100 candidate3839 (in intron):

LOCUS ENSG00000009413

DESCRIPTION DNA polymerase zeta catalytic subunit (EC 2.7.7.7) (hREV3).

[Sorce:Uniprot/SWISSPROT;Acc:O60673]

TAGTTGTTTAGCTTAGTGCCATTCTAAATTGAATCTCTTTATTTGTTGTAACATTAACCTTTCTTCTCTTCGGACAGTTT

TGGCCTTGGCTTAACTTCCT

In100 candidate3840 (in intron):

LOCUS ENSG00000009413

DESCRIPTION DNA polymerase zeta catalytic subunit (EC 2.7.7.7) (hREV3).

[Sorce:Uniprot/SWISSPROT;Acc:O60673]

TATCCTATGCTTTGTCTAACCCCCATACCTTTGCTTAAACTGTTCCCTCTGTTTTTTTGTCTGCTTTACCTTTTCCAGTC

TCCTTTTCCTATGTCTGTTA

In100 candidate3841 (in intron):

LOCUS ENSG00000009413

DESCRIPTION DNA polymerase zeta catalytic subunit (EC 2.7.7.7) (hREV3).

[Sorce:Uniprot/SWISSPROT;Acc:O60673]

TTAACATCTATGGTTGTTTCTTAGAATTCTGCCTTAGACTTTCATCTTTACTCTGTATATTTTCTGTTCCCTCTACAGAC

TTATTTACTCCCATGGGTTT

In100 candidate3842 (in intron):

LOCUS ENSG00000009413

DESCRIPTION DNA polymerase zeta catalytic subunit (EC 2.7.7.7) (hREV3).

[Sorce:Uniprot/SWISSPROT;Acc:O60673]

AGTCCTAATATCCTTTCTAACTGCCATAATTTTTTTTCTTTTCCATCACTGTCAACCTTCTTGAAGCAGAGGTTAGCATT

TTTTGTCTCTTAGTTCACTT

In100 candidate3843 (in intron):

LOCUS ENSG00000112769

DESCRIPTION Laminin subunit alpha-4 precursor.

[Source:Uniprot/SWISSPROT;Ac:Q16363]

TATTAATAAATATTTGTAACATTTTGTTTCTTTTGTCCCTTTTTTAAAAAATGTTTTCTTGTCTGCCTTCCCCAGATTTT

GCTATCTGAGGCCATTTTCT

In100 candidate3844 (in intron):

LOCUS ENSG00000111816

DESCRIPTION Tyrosine-protein kinase FRK (EC 2.7.10.2) (FYN-related kinase)

Nuclear tyrosine protein kinase RAK). [Source:Uniprot/SWISSPROT;Acc:P42685]EXON

TTTTTCCATTCAATTCTACTTTACTCTTTGCAATAACCCTGTGAAGAATGTACTATTATTACCCCCTTTTTAACTCAGAT

GAGTTTCCTCATCTCAGTTT

In100 candidate3845 (in intron):

LOCUS ENSG00000111816

DESCRIPTION Tyrosine-protein kinase FRK (EC 2.7.10.2) (FYN-related kinase)

Nuclear tyrosine protein kinase RAK). [Source:Uniprot/SWISSPROT;Acc:P42685]EXON

CTTTGTCAATTACAACCCCACACTATCCCAAATTTTATGATAACTGCTTTCCCATTACTTTATGTTTACCTCTACCAGAA

CCTTCTTGTCTTCCTTCTTG

In100 candidate3846 (in intron):

LOCUS ENSG00000178425

DESCRIPTION 5'-nucleotidase domain-containing protein 1.

[Source:Uniprot/SWSSPROT;Acc:Q5TFE4]

TACCTTTGTCACATCTAAGAAACCAACATTGGCACATTCCTATTAACTAACCACAAGACTTCATTTAGCTTCTACCAGTT

TTTATACTAATGTCCTTTTC

In100 candidate3847 (in intron):

LOCUS ENSG00000160062

DESCRIPTION Zinc finger and BTB domain-containing protein 8.

[Source:Unipro/SWISSPROT;Acc:Q8NAP8]

GTATGCAAATTATCAATTTATCATCACTTAACTTCAAACTTAAGCTTTTTTTGCCCTGCTTTGTGCAACCAGCTGGACTC

TATAAACATTTCTTCTTTCC

In100 candidate3848 (in intron):

LOCUS ENSG00000160062

DESCRIPTION Zinc finger and BTB domain-containing protein 8.

[Source:Unipro/SWISSPROT;Acc:Q8NAP8]

GGAAAAGACGCAACTTGCTATTTTTACTAATTTTATATTTCTCTGGACTTAACTAAGAATTATGTTCTCTTATCTCAGCC

TCTCTGGGTTTTCCTATTCA

In100 candidate3849 (in intron):

LOCUS ENSG00000162521

DESCRIPTION Histone-binding protein RBBP4 (Retinoblastoma-binding protein 4

(RBBP-4) (Retinoblastoma-binding protein p48) (Chromatin assembly factor

1subunit C) (CAF-1 subunit C) (Chromatin assembly factor I p48 subunit) (CAFI 48

kDa subunit) (CAF-I p48) (Nucleoso [Source:Uniprot/SWISSPROT;Acc:Q0902]

AAAACCACGGTTGTAGTTCTGTAACACTGTCCTCTCCTTCATTCTTTTTCTGGTTCTTGTCTAACCTTCTACATACAGGT

AATTCCAATTTTCTGTTCAT

In100 candidate3850 (in intron):

LOCUS ENSG00000047936

DESCRIPTION Proto-oncogene tyrosine-protein kinase ROS precursor (EC 2.7.101)

(c- ros-1). [Source:Uniprot/SWISSPROT;Acc:P08922]

CACTTATATTCACACATATATGTATATATTTCATGCAAACTAGATAACTACCTGTAATTTTTCTTTCATTTCTTACAGTT

TTATGGTCCTTTACTCACCT

In100 candidate3851 (in intron):

LOCUS ENSG00000116514

DESCRIPTION IBR domain-containing protein 3.

[Source:Uniprot/SWISSPROT;Acc:6ZMZ0]

AAGTAACACATTGAGCCATTTCTCTGTGCCAGGCTTGTGCCACGTATCATCTTATTTTGATTTAATTGTCAATGGTTTTG

TTTTGTTTTGTTTTTACTTT

In100 candidate3852 (in intron):

LOCUS ENSG00000196376

DESCRIPTION solute carrier family 35, member F1

[Source:RefSeq_peptide;Acc:P_001025029]

GAATAACAATCTTTAATATTTTGCTTCCACTGTCTAAACTCCCTCTGGAGTTTGTTTCTTTGTACTTTCCCTCAGAATAT

GGCTCTTCTGCTCATGTTTT

In100 candidate3853 (in intron):

LOCUS ENSG00000196376

DESCRIPTION solute carrier family 35, member F1

[Source:RefSeq_peptide;Acc:P_001025029]

AATAGAGAGCACCTGTGAATCTTGTAAATTATTGTTTTAACTTAAGAACTCATCTTATTTCTGTCTTATTTCCAGACATT

TGTTTCTCAGAGTCCCTATT

In100 candidate3854 (in intron):

LOCUS ENSG00000196376

DESCRIPTION solute carrier family 35, member F1

[Source:RefSeq_peptide;Acc:P_001025029]

AGCCAAGAAAATTGTAGTAACCTCTCAATATTATCTTTTCTAACTATACTTTCAGTTTCTTTAACTTTTCTTCCTGGGTT

CTGTTTTCAAATATTAGCTA

In100 candidate3855 (in intron):

LOCUS ENSG00000111860

DESCRIPTION C6orf204 protein. [Source:Uniprot/SPTREMBL;Acc:Q2TAM2]

ATGGAAGCTTAGGTTGAGAGCTTTTTCCCTTTTTCTAATATAACTGCTTAGTGCTGTAAATTTCCTTCTCAGTACTGCTT

TACCTGCTTCCTTAAAATTT

In100 candidate3856 (in intron):

LOCUS ENSG00000111860

DESCRIPTION C6orf204 protein. [Source:Uniprot/SPTREMBL;Acc:Q2TAM2]

TTGGTTATCTTTAACATTTATTTTCCATTTACATCTTTAAATAATCATTTTCTCTAAATCCTTTCACCCTGCTTCCAGTT

TCTTCATTCTGTTCTCCATG

In100 candidate3857 (in intron):

LOCUS ENSG00000111879

DESCRIPTION Uncharacterized protein C6orf60.

[Source:Uniprot/SWISSPROT;Acc:8NB25]

GTAATTAGTTCAATAACATCCCCTCCTTTTTTTTTTTACTTTTCCATCTTCCTTCTCTCAGTAAGCTTACTCCTTCATT

CCTACTTTCTGGAGTCATTT

In100 candidate3858 (in intron):

LOCUS ENSG00000111885

DESCRIPTION Mannosyl-oligosaccharide 1,2-alpha-mannosidase IA (EC 3.2.1.113 (Processing alpha-1,2-mannosidase IA) (Alpha-1,2-mannosidase IA) (Mannosidse alpha class 1A member 1) (Man(9)-alpha-mannosidase) (Man9-mannosidase). Source:Uniprot/SWISSPROT;Acc:P33908]

AGTGCAAACTGCTGGGCTGTGGGCTAACTGTGGCTCACGGATTTTTTTTTTTAACCCTTAGCCAGCGTAGTTTTGATTTT
TTTTTTTTTTTAAATAGCT

In100 candidate3859 (in intron):

LOCUS ENSG00000111885

DESCRIPTION Mannosyl-oligosaccharide 1,2-alpha-mannosidase IA (EC 3.2.1.113 (Processing alpha-1,2-mannosidase IA) (Alpha-1,2-mannosidase IA) (Mannosidse alpha class 1A member 1) (Man(9)-alpha-mannosidase) (Man9-mannosidase). Source:Uniprot/SWISSPROT;Acc:P33908]

AGCCTCCTCTTAACTTTTTTCTCCTTGCCTTTCTTGTCTTCTTTCTCATGGCTTTTACTGCTTCACAGCCCTTTCTCCTG
TGTATTTGTACTTCTCTGTT

In100 candidate3860 (in intron):

LOCUS ENSG00000111885

DESCRIPTION Mannosyl-oligosaccharide 1,2-alpha-mannosidase IA (EC 3.2.1.113 (Processing alpha-1,2-mannosidase IA) (Alpha-1,2-mannosidase IA) (Mannosidse

alpha class 1A member 1) (Man(9)-alpha-mannosidase) (Man9-mannosidase).

Source:Uniprot/SWISSPROT;Acc:P33908]

AAGTTACATCTTTCTAAGTATTTTAAGTTAACCTTCTTTATGGTTTCTTTTTCTTCCCCCAGTTGGATTTTTTTTAAATA

TTGGGTTCCCCACTTCCCTC

In100 candidate3861 (in intron):

LOCUS ENSG00000121904

DESCRIPTION CUB and sushi domain-containing protein 2 (CUB and sushi multipe

domains protein 2). [Source:Uniprot/SWISSPROT;Acc:Q7Z408]

AAAGTAGCTCATTATTGCGTTCCTTTAATTATAACTGAAACTAAGCATCTTTTCATGTTTCCCAGTTGTCTGTATCTCTT

CTCCTGAAAACTATCTGTTT

In100 candidate3862 (in intron):

LOCUS ENSG00000121904

DESCRIPTION CUB and sushi domain-containing protein 2 (CUB and sushi multipe

domains protein 2). [Source:Uniprot/SWISSPROT;Acc:Q7Z408]

GTTCTTCTAGTTTTCCTTTTTCATAGACACTAATAGTAACATCTAACTGCTCAATTATTTCTTGCATATTTTTCTCAGTT

TGAGTCTTGTCACCTTTTCC

In100 candidate3863 (in intron):

LOCUS ENSG00000146350

DESCRIPTION Uncharacterized protein C6orf170.

[Source:Uniprot/SWISSPROT;AccQ96NH3]

GTTACTATAGCTTTGTAATAAGTTAAAATAACTGATAGAGCAATTCTTCTTGCTTTGGTCTTTTCAAAAATATCTCAGCT

ATTTTTAGTCCTTTTCTTTC

In100 candidate3864 (in intron):

LOCUS ENSG00000146350

DESCRIPTION Uncharacterized protein C6orf170.

[Source:Uniprot/SWISSPROT;AccQ96NH3]

CCCCCAACCCATAATTCTCTAACATCTTATCCTACTTTAGTTTTTCTCACAGTTTTTATCACCATCCGAAATTATTTTAT

ATGTGTTTTTGTCTGTCTTC

In100 candidate3865 (in intron):

LOCUS ENSG00000163867

DESCRIPTION Zinc finger MYM-type protein 6 (Zinc finger protein 258).

[Soure:Uniprot/SWISSPROT;Acc:O95789]

ATTAACCATTGTTAGATTTGGTTTATTTGCTCAGTTTCTTTCCCCTACCTTTTTCTTCTTTTTTAAAAAATTAACCACTA

CTACCAGTTAGTCTCCCTTT

In100 candidate3866 (in intron):

LOCUS ENSG00000186439

DESCRIPTION Triadin. [Source:Uniprot/SWISSPROT;Acc:Q13061]

ATCATAGGCTGAACAAATATAAGATGTGTCATCCCAAGTTCCTGTAACAACCCTAAACTACTTTTACTGCCTTGTCAGTT

CTATATTTGTGTTTCCCTTC

In100 candidate3867 (in intron):

LOCUS ENSG00000186439

DESCRIPTION Triadin. [Source:Uniprot/SWISSPROT;Acc:Q13061]

GTGTGTGTGTGTGTTTTGGGTATGTATACCCTTCATCTATAACTTATTTTCAAATATTTTTCTGAATTTGTTTCACAGTT

AATATTATGCTTTCTGTCTT

In100 candidate3868 (in intron):

LOCUS ENSG00000186439

DESCRIPTION Triadin. [Source:Uniprot/SWISSPROT;Acc:Q13061]

GATGAATTCTATCTTCTATGTCTGTTAACCTCTTGTATATTTTCCATCTTTTTGTTTCTCTGGGATGAATTCCAGGCTAT

TTCTTCAGATATGTATTTCT

In100 candidate3869 (in intron):

LOCUS ENSG00000188580

DESCRIPTION T-cell lymphoma breakpoint associated target protein 1

(ProteinFAM77B). [Source:Uniprot/SWISSPROT;Acc:Q5VXU1]

GTCTGATACTGTTGGCTAAAATTTCTAGAAAATTATAATAACAATTAGAAATGTGTTTTTCTTTTGGTCCAGATTTTATT

AGAAATCTATCTTTTGTTTT

In100 candidate3870 (in intron):

LOCUS ENSG00000188580

DESCRIPTION T-cell lymphoma breakpoint associated target protein 1

(ProteinFAM77B). [Source:Uniprot/SWISSPROT;Acc:Q5VXU1]

TAAAAGTGTTAATGTGTTTAAATGTAGACATATAACATTTATTTACATTTTTAAACCTCTGTTTGGCATTTTTTTCAGTT

TTTTTATGTTTTTCAGGTGT

In100 candidate3871 (in intron):

LOCUS ENSG00000188580

DESCRIPTION T-cell lymphoma breakpoint associated target protein 1

(ProteinFAM77B). [Source:Uniprot/SWISSPROT;Acc:Q5VXU1]

ATCGAGGTTTTAACGTTTGGGGACTTTTTTTTTTTTTTAATTTCTCCCTTTCATTTTCCTTTCATTTTGCATCACCAGTG

CTTTCATTAGATTTTCTTTT

In100 candidate3872 (in intron):

LOCUS ENSG00000188580

DESCRIPTION T-cell lymphoma breakpoint associated target protein 1

(ProteinFAM77B). [Source:Uniprot/SWISSPROT;Acc:Q5VXU1]

AGTTCCAAGAGTTTCCCACCCTTCTTATAATCACAATAACTCTGAGTTTTTTCCTTTTCTTTCCTTTCCTCAGCCGTGGT

ATATCTTTACTGCTTCTCTC

In100 candidate3873 (in intron):

LOCUS ENSG00000188580

DESCRIPTION T-cell lymphoma breakpoint associated target protein 1

(ProteinFAM77B). [Source:Uniprot/SWISSPROT;Acc:Q5VXU1]

TTTAACCCATGAACGTGGAACATCTTTCATTCTTTTGTGTCCTCTTCAATTTCCTTCATCAATATTTTACAGTTTTCATT

ATAGGCATCTTTTACTACTT

In100 candidate3874 (in intron):

LOCUS ENSG00000188580

DESCRIPTION T-cell lymphoma breakpoint associated target protein 1 (ProteinFAM77B). [Source:Uniprot/SWISSPROT;Acc:Q5VXU1]

GGGCATTCATAGCTAACAATACCATATTTTATTTTATCTTCCTGAGAGTTATTGACTTATCTTTATTTCTTACCCCAGCT
TCTTCCTTTTTTCTCATTCT

In100 candidate3875 (in intron):

LOCUS ENSG00000111907

DESCRIPTION Tumor protein D53 (hD53) (Tumor protein D52-like 1). [Source:Unprot/SWISSPROT;Acc:Q16890]

TTTCTCTGTCCTTTTTTTTCCCACTAGACTAAATAATAGATATGTATAACCCTTTTTATATAGCTTATTTTCTGCAGTT
TACTTTTGTGTTCTAAGTCT

In100 candidate3876 (in intron):

LOCUS ENSG00000146463

DESCRIPTION zinc finger protein 262 [Source:RefSeq_peptide;Acc:NP_005086]

GTGTCATATCTAACTTCTGTGCCTTTTAGCATTTTATTTTTTTCTGTCTCCAGATGTCTTTCTACCTGCTCTTCCTTCAA
GAACCAGCTTTTTCTTTCTT

In100 candidate3877 (in intron):

LOCUS ENSG00000142687

DESCRIPTION polycystic kidney disease 1-like isoform a [Source:RefSeq_peptie;Acc:NP_079150]

AATTGCTTACCATTACTTAGTTTCTTAAAATAACATGTAATGCTTTTTTCCATCTGGTCTTTGCCTCTATCTTTCCAGCT
CATCTTCTATTACTTTTGCC

In100 candidate3878 (in intron):

LOCUS ENSG00000066651

DESCRIPTION Putative RNA methylase C6orf75 (EC 2.1.1.-).

[Source:Uniprot/SWSSPROT;Acc:Q7Z4G4]

AATGCTTCTTGAAAAACTAGTATAACTTCTTGTCTCCTCTTCTTCATCTTTCACTCTCTTCTGGCCATTATTTTCCAGCT

TCTGTGCCTATCATTCTGTT

In100 candidate3879 (in intron):

LOCUS ENSG00000093144

DESCRIPTION Enoyl-CoA hydratase domain-containing protein 1.

[Source:Unipro/SWISSPROT;Acc:Q9NTX5]

AAAATCAAAGCTTAACATAACTTTTCCCTTCTTTCCCTTCTTCTCTTATATGTTTATCATCTTAAGGATAACAGATGGGT

ATATCTTATGCCTTTCCCCT

In100 candidate3880 (in intron):

LOCUS ENSG00000126070

DESCRIPTION Eukaryotic translation initiation factor 2C 3 (eIF2C 3) (eIF-2C3)

(Argonaute-3). [Source:Uniprot/SWISSPROT;Acc:Q9H9G7]

GAAACAAGTTCATCCATAATCCTACCACTCTAATGTAACCACTGTTAACATTTTGGTATACTATACTTCTTTCAGTCTTC

ATCTGGTGTACATTTTACTT

In100 candidate3881 (in intron):

LOCUS ENSG00000172673

DESCRIPTION Uncharacterized protein C6orf190.

[Source:Uniprot/SWISSPROT;AccQ8N1K5]

ACATGTAACATTTTTTGAGACCATTTTCATTTTCAACAGTTGTTATGGTTTCCTTGGAAGTTTATTCTTTTATTCCTTTC

TTCATTTACAGTTAACTTGC

In100 candidate3882 (in intron):

LOCUS ENSG00000152894

DESCRIPTION Receptor-type tyrosine-protein phosphatase kappa precursor (EC

.1.3.48) (Protein-tyrosine phosphatase kappa) (R-PTP-kappa).

[Source:Unipro/SWISSPROT;Acc:Q15262]

AGGGTTATCAATTTCAATTGGGTCCAAATATAACTGGAAATTCTACTACTATATTTTTCTGTTCAGTTATCTTTGTTCTT

CTACTCTGTATTTCAACTCC

In100 candidate3883 (in intron):

LOCUS ENSG00000152894

DESCRIPTION Receptor-type tyrosine-protein phosphatase kappa precursor (EC

.1.3.48) (Protein-tyrosine phosphatase kappa) (R-PTP-kappa).

[Source:Unipro/SWISSPROT;Acc:Q15262]

AGAGTTTACCAAATACCTAAGAGCTAACCCCAAGTGATGCTTATTAGTTTCAGTTTCTCTTGACCTCTTTCTTATGTTAG

ATATTATTTGCCTTTACCTA

In100 candidate3884 (in intron):

LOCUS ENSG00000152894

DESCRIPTION Receptor-type tyrosine-protein phosphatase kappa precursor (EC

.1.3.48) (Protein-tyrosine phosphatase kappa) (R-PTP-kappa).

[Source:Unipro/SWISSPROT;Acc:Q15262]

TGTAATCTTAACTATTAAAATTGGATTTTTTTTCTGGCTGTGTACATATTTCTGTCATTTTGCTTTCTCATCAACCAGTT

AAAATATTTTTTTTTCTGTT

In100 candidate3885 (in intron):

LOCUS ENSG00000196569

DESCRIPTION Laminin subunit alpha-2 precursor (Laminin M chain) (Merosin hevy chain). [Source:Uniprot/SWISSPROT;Acc:P24043]

ATGACGCTAACACCAAAATCATGAAGTAAATGAATTTTTTCTTTTTCTCCTCTTCCTCTCAGTCCCTGCCCCTTTTCAGT

TATGTGTGAATATTCTCATT

In100 candidate3886 (in intron):

LOCUS ENSG00000196569

DESCRIPTION Laminin subunit alpha-2 precursor (Laminin M chain) (Merosin hevy chain). [Source:Uniprot/SWISSPROT;Acc:P24043]

ATTTTCATTTCATATCATTTTTCTTCTTCCATTAACTACTTCCTAATCTTTATCATTTTATTCCTGTTGTTTCAGGTCTG

CTGACAATAATTTTTTCCTT

In100 candidate3887 (in intron):

LOCUS ENSG00000196569

DESCRIPTION Laminin subunit alpha-2 precursor (Laminin M chain) (Merosin hevy chain). [Source:Uniprot/SWISSPROT;Acc:P24043]

ATCTGTAAAGCAAGGTCCTTTATTTCTCTTCTCTTCTAATTAACTAGATGCTCTTACATATTCTTCCAGCTGACACTTTT

ATGCACTATTTTAATCTCTT

In100 candidate3888 (in intron):

LOCUS ENSG00000196569

DESCRIPTION Laminin subunit alpha-2 precursor (Laminin M chain) (Merosin hevy chain). [Source:Uniprot/SWISSPROT;Acc:P24043]

TCAAATGCAAGGATCGCATTCCCACAAGATAACATTCAATGTTGCATTCTTGCTGCCATGTTTATTTTTTGTCAGGTGGT

TTTTTTAAATTATTATTTTC

In100 candidate3889 (in intron):

LOCUS ENSG00000196569

DESCRIPTION Laminin subunit alpha-2 precursor (Laminin M chain) (Merosin hevy chain). [Source:Uniprot/SWISSPROT;Acc:P24043]

TTAGCTCATATACATGAGCTAATAATATTGAAATATTACCATTAACCCAAGACTGTATTCATATTTTTTCAGTGTTTACT

GAATGTTTTTTTACTGTTTC

In100 candidate3890 (in intron):

LOCUS ENSG00000146376

DESCRIPTION Rho GTPase-activating protein 18 (MacGAP). [Source:Uniprot/SWISPROT;Acc:Q8N392]

AGCCTGGTTACCTCCTGAGTCATCTTACATTTCATAGCTAACTGACCCATCTCTTTATTTCCTAATTTCAGTTTTTTGTT

TTTTGTTTTTTTTTATTTGA

In100 candidate3891 (in intron):

LOCUS ENSG00000146376

DESCRIPTION Rho GTPase-activating protein 18 (MacGAP).

[Source:Uniprot/SWISPROT;Acc:Q8N392]

GAGGAGGCAAAAGTCACTTATATTATTAATATAATACTACTCTAGAGCTAACCACAATTGAATTTTATTTTGCCTCAGTA

TTTTTTTCCCATGCACATTC

In100 candidate3892 (in intron):

LOCUS ENSG00000146376

DESCRIPTION Rho GTPase-activating protein 18 (MacGAP).

[Source:Uniprot/SWISPROT;Acc:Q8N392]

TACTTTATAAATATCTTCTCCATTTATATTTTCTAACATAAACACAAATCTCCTTTCACTTTTAATATTTTCATCCAGTG

TTTGTCTGTGTTTTTTCTTT

In100 candidate3893 (in intron):

LOCUS ENSG00000146376

DESCRIPTION Rho GTPase-activating protein 18 (MacGAP).

[Source:Uniprot/SWISPROT;Acc:Q8N392]

TAGTAACTCTGGCTTTTCTCTTTGGCTCTCTACAGAAGTAAGTGACTGAATTATCTCTTGTAACACACTTAATTCCTTTT

CTCTGCTTTCTTTCCCTTCC

In100 candidate3894 (in intron):

LOCUS ENSG00000146376

DESCRIPTION Rho GTPase-activating protein 18 (MacGAP).

[Source:Uniprot/SWISPROT;Acc:Q8N392]

AATTCACATTAGTAATTTAAAAAATGGAATGAATTAACTTTGACCATGTTTTATATTTTACCCTTTTGTAATTATCAGTG

AGTCTTAACCATCTCTTTTT

In100 candidate3895 (in intron):

LOCUS ENSG00000198945

DESCRIPTION Lethal(3)malignant brain tumor-like 3 protein (L(3)mbt-like 3

potein) (H-l(3)mbt-like protein). [Source:Uniprot/SWISSPROT;Acc:Q96JM7]

GCATGTGCTGATGCTACTCTTTGCCCCATAACAAAAAAGACACCCTTTCTCTGATCTGTTTTCTCTAAGGGCAGATTCCT

GGGTCATCTCTGATCTGTCT

In100 candidate3896 (in intron):

LOCUS ENSG00000198945

DESCRIPTION Lethal(3)malignant brain tumor-like 3 protein (L(3)mbt-like 3

potein) (H-l(3)mbt-like protein). [Source:Uniprot/SWISSPROT;Acc:Q96JM7]

AGCAAGTTCTAACCATTTAAAATTTTTCTGTCTTCTTTTATGACATCATTCTCTAAATAGCCCAGCTATTTCTTTTTTTA

ATTTTTATTTTTTTGAGACA

In100 candidate3897 (in intron):

LOCUS ENSG00000198945

DESCRIPTION Lethal(3)malignant brain tumor-like 3 protein (L(3)mbt-like 3

potein) (H-l(3)mbt-like protein). [Source:Uniprot/SWISSPROT;Acc:Q96JM7]

ATTGGAATCTTCTCTGCTTTCAAAATCCTCTCTCCAAAAAGAGAAATAACTTTTAAGTAACTTTTCTTGCTCCAGTTCTG

TCCCTTGAAATCTTCTCTGT

In100 candidate3898 (in intron):

LOCUS ENSG00000198945

DESCRIPTION Lethal(3)malignant brain tumor-like 3 protein (L(3)mbt-like 3 potein) (H-l(3)mbt-like protein). [Source:Uniprot/SWISSPROT;Acc:Q96JM7]

AGCCTATTTTTTGTTTTATAGAGTTTATTTCCCCTTGTGCATTGCTAACGTCATGAAACTCTTCTGTCTATAACTCAGTG
CTCTTCTTCTGATGTTTTCT

In100 candidate3899 (in intron):

LOCUS ENSG00000079819

DESCRIPTION Band 4.1-like protein 2 (Generally expressed protein 4.1) (4.1G. [Source:Uniprot/SWISSPROT;Acc:O43491]

TTTTGGTTTTATTTTCTGGTGTTTTGTCTCTAAATAACATGATTTTCTTGTTTCTTGATTTTTTTTTAAAGTTTCAGAA
TATTTTTATGTTTTATTATT

In100 candidate3900 (in intron):

LOCUS ENSG00000204084

DESCRIPTION Type II inositol-1,4,5-trisphosphate 5-phosphatase precursor (E 3.1.3.36) (Phosphoinositide 5-phosphatase) (5PTase) (75 kDa inositol polyposphate-5-phosphatase). [Source:Uniprot/SWISSPROT;Acc:P32019]

GCCTGGCCTCACTGTTCTTAACATCTTTTTTCTTTTCTTTTTTTTTCTTCCTAACCACTAGATGACCAGGGATGCTGTTA
ACTTCCTTTTGCAGCTTTTT

In100 candidate3901 (in intron):

LOCUS ENSG00000112282

DESCRIPTION CRSP complex subunit 3 (Cofactor required for Sp1 transcriptionl activation subunit 3) (Transcriptional coactivator CRSP130) (Vitamin D3 rceptorinteracting

protein complex 130 kDa component) (DRIP130) (Activator-ecruited cofactor 130 kDa component) (A [Source:Uniprot/SWISSPROT;Acc:Q9ULK]

AACCATTTCTGTACACTTTGGTAACAAACATTCCCCAAATGTTTTTTTCTTCTCCTGACTTTGATGTCTATTTCCAGCAG

AATTTTTTTTTCTTTTTTT

In100 candidate3902 (in intron):

LOCUS ENSG00000183431

DESCRIPTION Splicing factor 3A subunit 3 (Spliceosome-associated protein 61 (SAP 61) (SF3a60). [Source:Uniprot/SWISSPROT;Acc:Q12874]

GACATGTATGTAAGGAATGGTACTGACTCACTCCAAGTCCCATAGTAACTGACAATAGCCTCCCTTTTTTTGAGTCAGTG

GTTTTCAGTATTTTTTTTCT

In100 candidate3903 (in intron):

LOCUS ENSG00000127603

DESCRIPTION Microtubule-actin cross-linking factor 1, isoforms 1/2/3/5 (Actn cross-linking family protein 7) (Macrophin-1) (Trabeculin-alpha) (620 kDaactinbinding

protein) (ABP620). [Source:Uniprot/SWISSPROT;Acc:Q9UPN3]

GGGTAACCACTGTTCAAGCTTTGGTTTGTATCCTTACAGGTTTTTTCTTGCCAGGCTTGCTTTGGAACTGCACCTTTACA

TCCTTTATATTCCTTTAGGT

In100 candidate3904 (in intron):

LOCUS ENSG00000127603

DESCRIPTION Microtubule-actin cross-linking factor 1, isoforms 1/2/3/5 (Actn cross-linking family protein 7) (Macrophin-1) (Trabeculin-alpha) (620 kDaactinbinding protein) (ABP620). [Source:Uniprot/SWISSPROT;Acc:Q9UPN3]

AGACATCTGTTAACTCTTTTCTCTTCATCAACCCCATTTTCAATCTGTCATTGAGTTCTTTTGTTCTTCAGAGTTTCTCT
TAAATTTGTCTTTTTCTCCC

In100 candidate3905 (in intron):

LOCUS ENSG00000127603

DESCRIPTION Microtubule-actin cross-linking factor 1, isoforms 1/2/3/5 (Actn cross-linking family protein 7) (Macrophin-1) (Trabeculin-alpha) (620 kDaactinbinding protein) (ABP620). [Source:Uniprot/SWISSPROT;Acc:Q9UPN3]

AGTTCTCTTTACCTGGAATGCCCTTGTTCTTTATACTTAACTGAAATCTGTCTATTCCTTCAAGTTTCACTTCAGGTCTT
TCTTCTGATGAAGACTTCTC

In100 candidate3906 (in intron):

LOCUS ENSG00000127603

DESCRIPTION Microtubule-actin cross-linking factor 1, isoforms 1/2/3/5 (Actn cross-linking family protein 7) (Macrophin-1) (Trabeculin-alpha) (620 kDaactinbinding protein) (ABP620). [Source:Uniprot/SWISSPROT;Acc:Q9UPN3]

AGTCCCTTAACTACTATTTCTGTTTCACCTTATCTCAGTTTGTTTACATCCACTTTCTACGTGGAATTCCTTTCTTCCTT
GGCTTTCACCTGTTCTGTAA

In100 candidate3907 (in intron):

LOCUS ENSG00000112319

DESCRIPTION Eyes absent homolog 4 (EC 3.1.3.48).

[Source:Uniprot/SWISSPROT;cc:O95677]

GTGCCAATGTGTGACTGTATGCTTGTATATGTTATACACAAAAGTTGTTAACTTCTGCAAAACTTTGACCATTTTCAGTT

TACATTGTTAATTTTCATTT

In100 candidate3908 (in intron):

LOCUS ENSG00000028839

DESCRIPTION TATA box-binding protein-like protein 1 (TBP-like protein 1) (TTA

box-binding protein-related factor 2) (TBP-related factor 2) (STUD proten) (21

kDa TBP-like protein). [Source:Uniprot/SWISSPROT;Acc:P62380]

TCATTGCTCCTGAAGTTTCTCTTTCATTATCTCGTTTCCTTTATAACATAGATCACATTCTCTTGATTATTTTGTCAGCC

TATTTGTTGTCTTTTTCTTC

In100 candidate3909 (in intron):

LOCUS ENSG00000118515

DESCRIPTION Serine/threonine-protein kinase Sgk1 (EC 2.7.11.1)

(Serum/glucoorticoid-regulated kinase 1). [Source:Uniprot/SWISSPROT;Acc:O00141]

GGAACAAAATTTAAGGGATAAGCAAAGTTTAACTGCATGCCAATTCATTTTTTTTGCAGTTATTTCTCTTTTTCCTCAAA

GTTTATCATTATACATATAT

In100 candidate3910 (in intron):

LOCUS ENSG00000112339

DESCRIPTION HBS1-like protein (ERFS).

[Source:Uniprot/SWISSPROT;Acc:Q9Y450]EXON

TGTAATAAAAGGGAAAAAGGTCACTCATGATTTTCTTACCTTGAGATAACATAATTTATCTTTGCCTTATTTTTTCAGTT

TTTGTCTATGCGTTTCTATT

In100 candidate3911 (in intron):

LOCUS ENSG00000135541

DESCRIPTION Jouberin (Abelson helper integration site 1 protein homolog) (AI-

1). [Source:Uniprot/SWISSPROT;Acc:Q8N157]

TCTTACATTGTTTGGATTTTTCTACATATGCAATCATGTAATACGTTAATAACGATAGTTTTATCGCTTCTTTTTCAGTC

CTTATGCCTTATCATTTTGC

In100 candidate3912 (in intron):

LOCUS ENSG00000084073

DESCRIPTION CAAX prenyl protease 1 homolog (EC 3.4.24.84) (Prenyl proteinsecific

endoprotease 1) (Farnesylated proteins-converting enzyme 1) (FACE-

1)(Zinc metalloproteinase Ste24 homolog). [Source:Uniprot/SWISSPROT;Acc:O7584]

AGCTTGGGCAACAAGAGTGAAACTCCATCTAAAATAAATAATAATAACTACTGTTAACATTTGTATATACTTTCAGTCTA

CTTTTTATATTTGTATATTT

In100 candidate3913 (in intron):

LOCUS ENSG00000171408

DESCRIPTION cAMP-specific 3',5'-cyclic phosphodiesterase 7B (EC 3.1.4.17).

Source:Uniprot/SWISSPROT;Acc:Q9NP56]

AATATTTTCAAAAAACTAACTCCTGGAATCACTGATCTTTTGAATGGTTTTTCATTCCTTGACTACTTTCAGCTCAGCTC

TGATTTCGATTACTTGTCTT

In100 candidate3914 (in intron):

LOCUS ENSG00000197442

DESCRIPTION Mitogen-activated protein kinase kinase kinase 5 (EC

2.7.11.25)(MAPK/ERK kinase kinase 5) (MEK kinase 5) (MEKK 5) (Apoptosis

signalregulting

kinase 1) (ASK-1). [Source:Uniprot/SWISSPROT;Acc:Q99683]

GCAAGTTAAAGGTTTTCACTGATTTTTTCTTGATCTGTTTACTATTTAACTATATTTTAATTTCTTCTTTGACTACAGTT

GTCATTTGCTTTTTCATTTT

In100 candidate3915 (in intron):

LOCUS ENSG00000135540

DESCRIPTION NHS-like 1. [Source:Uniprot/SPTREMBL;Acc:Q5SYE7]

TTAACACTGATATGTTGGTCCCAAGGAGTTTCTGGATTTTTTTTTCTTTCTCCTTTCCTTCTTTTAACCAGAGTTTTTA

GATTTCTATCAAGTTTCTTT

In100 candidate3916 (in intron):

LOCUS ENSG00000203735

DESCRIPTION OTTHUMP00000017300 (Fragment).

[Source:Uniprot/SPTREMBL;Acc:Q5JK3]

TTATAGGTTGGAAAGATTCACCACGTCTCATAACTTTCATATTCTTTTATACACATTTTCCATTTCTGTGACCTTCAGTA

TTTCAGTCTGGATTTTCTTT

In100 candidate3917 (in intron):

LOCUS ENSG00000135597

DESCRIPTION RalBP1-associated Eps domain-containing protein 1 (RalBP1-

intercting protein 1). [Source:Uniprot/SWISSPROT;Acc:Q96D71]

TCTTCTTGTTCATCGTGGAGCTGAAATACTCTGTCTTTAACGTCCTGATTGTCTTCCTACTGTCTTCTTTGGAGTCAGCT

TTGACTCCTCTTTTCCATCT

In100 candidate3918 (in intron):

LOCUS ENSG00000010803

DESCRIPTION Polycomb protein SCMH1 (Sex comb on midleg homolog 1).

[Source:niprot/SWISSPROT;Acc:Q96GD3]

TTTCTCTTTTTATGAGTGAAGTTTAACATCTTTTCATATGTCTAGGGTGCCATTTTATCTTTTTTTTTTCTTTTACAGGT

TGTCATTTCTTTTGCTCATT

In100 candidate3919 (in intron):

LOCUS ENSG00000127124

DESCRIPTION human immunodeficiency virus type I enhancer binding protein 3

Source:RefSeq_peptide;Acc:NP_078779]

TCCTTTGTTCCTTTCTTCCTTCTTAACTTCTTTTTTTACTTCTTTCTTATTTTCTGGCACTACAAGTTTCTCCAGGCTGG

TCTTGTATTTTCTGAGCCCT

In100 candidate3920 (in intron):

LOCUS ENSG00000198815

DESCRIPTION Forkhead box protein J3. [Source:Uniprot/SWISSPROT;Acc:Q9UPW0]

EXON

AAACCTGTTCCTTGACTTGTTTTTAAAAAAAGTAACTCCCTGGTACTGCCTAATTTAATTTTTCTCTCACTCATCAGTG

TCCTTGACTTTGCATTCCTT

In100 candidate3921 (in intron):

LOCUS ENSG00000198815

DESCRIPTION Forkhead box protein J3. [Source:Uniprot/SWISSPROT;Acc:Q9UPW0]

EXON

TTGCAATTTGGTGGTTTTCTGTAGTGGTAACATTTGAGTTTTTTCTCTTCTTCATTTGTATGTTCGTTCTACCAGTGAGT

TTTTATACTTCTCCGTGTTT

In100 candidate3922 (in intron):

LOCUS ENSG00000009844

DESCRIPTION Uncharacterized protein C6orf55 (Dopamine-responsive protein DR-

1). [Source:Uniprot/SWISSPROT;Acc:Q9NP79]

AATGATAGTCATCCTTCATGCCCTGATTTCTACTGTTCCTATTATGTAACACACTACTACTTTACTTTTATTTGTCAGCC

TTATTTTACCCAAATTTTTT

In100 candidate3923 (in intron):

LOCUS ENSG00000009844

DESCRIPTION Uncharacterized protein C6orf55 (Dopamine-responsive protein DR-

1). [Source:Uniprot/SWISSPROT;Acc:Q9NP79]

CTAGAAACTTCCTATTTATTTTAACCTTTTTCTCTGTTTCCTTTTTCTTATTCCAGTATTTTTCCCACTTTACCCTTTCT

ACAAATTGCCTATTCGAATT

In100 candidate3924 (in intron):

LOCUS ENSG00000112414

DESCRIPTION Probable G-protein coupled receptor 126 precursor.

[Source:Unipot/SWISSPROT;Acc:Q86SQ4]

CCTAACTTTTCGAAATTATCCTTCACTGTATTTATATTTAAATCCTCCACTATCTTCAGCAATTTGTATTGTCCATATAT

CCTCCTGTGTACCTTGTTTT

In100 candidate3925 (in intron):

LOCUS ENSG00000164010

DESCRIPTION Erythroid membrane-associated protein precursor (hERMAP) (Sciana

blood group antigen) (Radin blood group antigen).

[Source:Uniprot/SWISSPRT;Acc:Q96PL5]

GAGTGAACTGAGTAAGTTTCCCATGTTCTTGTAACTTCCGTACCAACTTATCTCCTGTTGCCCTAATATTTGCAGTGATA

GCTCATTCCCCTTGTTTCTT

In100 candidate3926 (in intron):

LOCUS ENSG00000112419

DESCRIPTION Phosphatase and actin regulator 2.

[Source:Uniprot/SWISSPROT;Ac:O75167]

TGTTATTAGTAATGATTATATTTGACTTATTAACCTGTATCTTCTGTTATTTTCCATGTATCCTCTACATCTACCAGTCT

GTTCCACGTCTGTTCTGTAT

In100 candidate3927 (in intron):

LOCUS ENSG00000112419

DESCRIPTION Phosphatase and actin regulator 2.

[Source:Uniprot/SWISSPROT;Ac:O75167]

TGAAATGTACAATCAATAAAGCATGAATAGGCAAACTAAACTAACCTGCTTTCTGCTTTCTCTCATGTAATTTTGCAGTA

CTGTTCTCTTTTCATTTCTC

In100 candidate3928 (in intron):

LOCUS ENSG00000152818

DESCRIPTION Utrophin (Dystrophin-related protein 1) (DRP1) (DRP).

[Source:Uiprot/SWISSPROT;Acc:P46939]

TCCTCCGTGGAGAGAGGATTAGTTTCTGAATTCCTTTGCTTCCCTCTCTAACTTCAACATTACCTCCTGTAGTTTCAGCT

AATTTGTTCTGTAATTCTCT

In100 candidate3929 (in intron):

LOCUS ENSG00000152818

DESCRIPTION Utrophin (Dystrophin-related protein 1) (DRP1) (DRP).

[Source:Uiprot/SWISSPROT;Acc:P46939]

CCTCTTTTCTTTTAGGTAACCATTCTAGAAAACACTTTCCTCCATGATTTTTATCTCCAATGCCTTTTTTTCTTCCAGTT

CCTTTAAATTTTTTCCATTA

In100 candidate3930 (in intron):

LOCUS ENSG00000146414

DESCRIPTION SNF2 histone linker PHD RING helicase isoform a

[Source:RefSeq_eptide;Acc:NP_001036148]

AGTTTTATGCATGTTTGACTCCTTAACATTGTCTAGTAGGTTTCTGATGTTCTATTTATTGTTTTTTCAGTCTTTGTTTA

TCTCTACTTCATTTTTGACA

In100 candidate3931 (in intron):

LOCUS ENSG00000152822

DESCRIPTION Metabotropic glutamate receptor 1 precursor (mGluR1).

[Source:Uiprot/SWISSPROT;Acc:Q13255]

TTCTTTTATTTCATGCTTAGTTTTAAATATTTCCTATGATAATTCATTAACGGATAACTCATTTAACCTCTTTTCCAGCT

CATTTCTTTATCTTAACATG

In100 candidate3932 (in intron):

LOCUS ENSG00000152822

DESCRIPTION Metabotropic glutamate receptor 1 precursor (mGluR1).

[Source:Uiprot/SWISSPROT;Acc:Q13255]

AACTGCTTATGGGGCATATTTGAAAGGGAAGACAAAGTGAAAGGTTAACTCTATTGTCCATTATTCTACCATTTCAGCTC

TGTTTTTTTCTTTCTTGAAA

In100 candidate3933 (in intron):

LOCUS ENSG00000152822

DESCRIPTION Metabotropic glutamate receptor 1 precursor (mGluR1).

[Source:Uiprot/SWISSPROT;Acc:Q13255]

AGCTCCTACTAACGCTGCTTGAAACTCTATGCATTCTTTTCATTTTATTTTGCATAATTATCATCACAGTGTGCATACAA

TTCTCTATCTTATTTCACTC

In100 candidate3934 (in intron):

LOCUS ENSG00000118492

DESCRIPTION Uncharacterized protein C6orf103.

[Source:Uniprot/SWISSPROT;AccQ8N7X0]

GGCCTGTGAGGTTCCTACTAAGAAATTTGCTGAGAAATTATAACTCCTTATTTGCTTCATTTCTCTTGCTGCTTTCAGAA

TCCTCTCTTTGTCTTTGATT

In100 candidate3935 (in intron):

LOCUS ENSG00000203727

DESCRIPTION SAM domain containing 1 (LOC389432), mRNA

[Source:RefSeq_dna;Ac:NM_001030060]

TTACCTAACCCTGTTTATTTAATGAAGCTCCTTCACATTTTTAACATTTTTGGATTTCTTTCCCATTCTCTCTTCAGGAG

TTCCTAATCTCTTTATCCAT

In100 candidate3936 (in intron):

LOCUS ENSG00000111962

DESCRIPTION Uronyl 2-sulfotransferase (EC 2.8.2.-).

[Source:Uniprot/SWISSPRT;Acc:Q9Y2C2]

TAGCCTTATTATCTACACCTCCTTCCATGAAACTCTGCTTCTGATTTCCTAACTCTTCTGGGGGTTTATACCCTTCAGCT

TTTTCCACGAGCTGCTATTT

In100 candidate3937 (in intron):

LOCUS ENSG00000111962

DESCRIPTION Uronyl 2-sulfotransferase (EC 2.8.2.-).

[Source:Uniprot/SWISSPRT;Acc:Q9Y2C2]

CATCTCTATTCTTGTCCTTATGCTGATAACCTAAGTTGAAAGGCCTTTTTTGTCTTTTTCTTCTTTTTTTGAGACAGGAT

CTTCCTCTGTTGCCTAGGTT

In100 candidate3938 (in intron):

LOCUS ENSG00000055208

DESCRIPTION Mitogen-activated protein kinase kinase kinase 7-interacting

prtein 2 (TAK1-binding protein 2). [Source:Uniprot/SWISSPROT;Acc:Q9NYJ8]

ATCTCATATCAAACTGCCTGTGGACATTTTAACTTGGTTATTCATCTTTCTTAAGTCTAAAACTCTCCTTTCTGTCAGGT

TCTTAATCTTCCAGGCTTAT

In100 candidate3939 (in intron):

LOCUS ENSG00000055208

DESCRIPTION Mitogen-activated protein kinase kinase kinase 7-interacting

prtein 2 (TAK1-binding protein 2). [Source:Uniprot/SWISSPROT;Acc:Q9NYJ8]

TATCTGTGATTAACTACAACCATTTTTGTTTAGTTTCAGCAGTTTATAAATGTTATATGCTATTATTTTTTCCACTTACA

ACTTAATTCTCCTCTTCTCC

In100 candidate3940 (in intron):

LOCUS ENSG00000055208

DESCRIPTION Mitogen-activated protein kinase kinase kinase 7-interacting

prtein 2 (TAK1-binding protein 2). [Source:Uniprot/SWISSPROT;Acc:Q9NYJ8]

AACTGTTGTGATCTCTGCTTGAACATGAGAGTAGGCCATCTAACATAAGTGTAATATCTCTATCTAGTTTTCTTCAGATG

TTCTTAACTCTTGTTTTTCT

In100 candidate3941 (in intron):

LOCUS ENSG00000131013

DESCRIPTION Peptidyl-prolyl cis-trans isomerase-like 4 (EC 5.2.1.8) (PPIase (Rotamase) (Cyclophilin-like protein PPIL4). [Source:Uniprot/SWISSPROT;AccQ8WUA2]

AACTACTCTTAACTTGAAACATACTCCTAACTACTTTTGCTCGTCTTCTTACCTGGGGTATATTCTCATCAGTTTTCTTT

CCTAAACTCTAATGTTATTT

In100 candidate3942 (in intron):

LOCUS ENSG00000120265

DESCRIPTION Protein-L-isoaspartate(D-aspartate) O-methyltransferase (EC 2.11.77) (Protein-beta-aspartate methyltransferase) (PIMT) (Protein Lisoaspatyl/ D-aspartyl methyltransferase) (L-isoaspartyl protein carboxyl methyltrasferase). [Source:Uniprot/SWISSPROT;Acc:P22061]

AAACTTACGAGTAACATACCTGGTGTCTGATTGGTTGTCATACTTTTCATTTTCCCTCCTTTCAGATCCAATCCATTCTA

CACATTATACTGATTTTTTC

In100 candidate3943 (in intron):

LOCUS ENSG00000009765

DESCRIPTION Iodotyrosine dehalogenase 1 precursor (EC 1.-.-.-) (IYD-1). [Sorce:Uniprot/SWISSPROT;Acc:Q6PHW0]

TTAATTTTTCTGTAGCTAGTGTAACCTGCTAGTCAACCATCCTTTTAAGTTCTTAATTCTATTATTTTTCAATTCCAGAA

GTTCTCTATTTTTTCAGATT

In100 candidate3944 (in intron):

LOCUS ENSG00000120278

DESCRIPTION Pleckstrin homology domain-containing family G member 1.

[Sourc:Uniprot/SWISSPROT;Acc:Q9ULL1]

AGCAAACGTGTCCCCATCTTAATTAACACTGGTCCCTGGGTCCTCATTCTTCCTGTATTTTCAGGGATGCTTTGGCTTAC

TCTTCCTTTTTCAGTATTTT

In100 candidate3945 (in intron):

LOCUS ENSG00000120254

DESCRIPTION methylenetetrahydrofolate dehydrogenase (NADP+ dependent) 1-lik

[Source:RefSeq_peptide;Acc:NP_056255]

TAGTAACTGACACTTTATTTATTTATTTATTTATTTATTTATTTTTTCTTTTTTTTTTTTTTTTTTTTGAGACAGAGTCT

TGCTCTGGTCTTGAACTCTT

In100 candidate3946 (in intron):

LOCUS ENSG00000120254

DESCRIPTION methylenetetrahydrofolate dehydrogenase (NADP+ dependent) 1-lik

[Source:RefSeq_peptide;Acc:NP_056255]

GCTACAAATACTGCGCTGGGATCGACTTCCTTTTGATCATTACACAAATAACCATGGCTGCCTCTTATGTTTATTCAGCT

TGTTTTTTTTCCATAATTTG

In100 candidate3947 (in intron):

LOCUS ENSG00000120262

DESCRIPTION Coiled coil-containing protein C6orf97.

[Source:Uniprot/SWISSPRT;Acc:Q8IYT3]

AGTTCCAAGACTTAACCTTGGTTCTTTTTCTTATATTTATTTCTTTGCTGACTTTCTTATTCAGATCATGATTTTTTCTT

ATTTCATTGAATTATCTCTT

In100 candidate3948 (in intron):

LOCUS ENSG00000091831

DESCRIPTION Estrogen receptor (ER) (Estradiol receptor) (ER-alpha).

[SourceUniprot/SWISSPROT;Acc:P03372]

CTGAAGTTCTTGAAAATAATCTCTTCTAACCTGGGTAAATATTCCTCCTTTTCAGAAATGTTTGCAATGTTGCTTTTTTT

TAAATTAATCTCTTTTTCTT

In100 candidate3949 (in intron):

LOCUS ENSG00000091831

DESCRIPTION Estrogen receptor (ER) (Estradiol receptor) (ER-alpha).

[SourceUniprot/SWISSPROT;Acc:P03372]

TTAGTTCTACTAGTTTTCTGCTTTCTAACTCATTAAGTTATGCCTTTAGCTTTATTAAATCCTTCATTCTGCTATCAGTT

AATGTTTTAGTTCATTTTCT

In100 candidate3950 (in intron):

LOCUS ENSG00000131018

DESCRIPTION Nesprin-1 (Nuclear envelope spectrin repeat protein 1) (Synapti nuclear envelope protein 1) (Syne-1) (Myocyte nuclear envelope protein 1) Myne-1) (Enaptin). [Source:Uniprot/SWISSPROT;Acc:Q8NF91]

TAACTGTTTTCTTAAAACTGTAGCCTTCTTTTCAATTTTTTTTCTTCTTAATAGAACCTTCTGACTCCTGTTTTCAGGGC

CACTTGCTCTTCCATTTTTT

In100 candidate3951 (in intron):

LOCUS ENSG00000131018

DESCRIPTION Nesprin-1 (Nuclear envelope spectrin repeat protein 1) (Synapti nuclear envelope protein 1) (Syne-1) (Myocyte nuclear envelope protein 1) Myne-1) (Enaptin). [Source:Uniprot/SWISSPROT;Acc:Q8NF91]

CTTTAACCTGTTGATGATTTTCTCTTATACATTCTCTGCTCTTTGTATATTTTATGGTGTTTCAGTTTTCTAAAGATGCC

TCTTGATTTATTTTGCCTTT

In100 candidate3952 (in intron):

LOCUS ENSG00000159592

DESCRIPTION GC-rich promoter binding protein 1-like 1 [Source:RefSeq_peptid;Acc:NP_067652]

CCATAATTCCACCATTCTAATAAAACCATTATTAACATTTTGGGATTTTTCCTTTTAGTCTTTTACTTCCAGAACAGAAA

TCTCTTTTATCTGTCTTCTT

In100 candidate3953 (in intron):

LOCUS ENSG00000197429

DESCRIPTION Actin-binding protein IPP (MIPP protein) (Kelch-like protein 27. [Source:Uniprot/SWISSPROT;Acc:Q9Y573]

AGGTGATCCTTTCTACCTTAACTGATGGTCTTGTAAAGACTTTCCCTTTTAATGATTTTGTTCCTCAACTTTTCTCAGTT

CTGCTTTTCATTCTTGATTC

In100 candidate3954 (in intron):

LOCUS ENSG00000086015

DESCRIPTION Microtubule-associated serine/threonine-protein kinase 2 (EC 2..11.1). [Source:Uniprot/SWISSPROT;Acc:Q6P0Q8]

AGTCCTTAACGCTGCTTACCAATTCTACTTTTTGTTCAGCGTATTTTCTTGTTCTCTGTATTATCTCTCAATTTTTTTTT

TTTTTTACTCATCTCAAATT

In100 candidate3955 (in intron):

LOCUS ENSG00000086015

DESCRIPTION Microtubule-associated serine/threonine-protein kinase 2 (EC 2..11.1). [Source:Uniprot/SWISSPROT;Acc:Q6P0Q8]

TTTTTATTGATCATGCTTTTGGTATCTAAGAACTTACTTGCCTAACCCAAGCTGTTTTTTTTTTCCATTTTCTTCCAGAA

GTTTTACATTTTCTACTCCT

In100 candidate3956 (in intron):

LOCUS ENSG00000086015

DESCRIPTION Microtubule-associated serine/threonine-protein kinase 2 (EC 2..11.1). [Source:Uniprot/SWISSPROT;Acc:Q6P0Q8]

GGTGATAACCCCTTTATCATTTTTTATTGTGTCTATTTGATTCTTCTCTCTTTTCTTCTTTATTAGTCTTGCTAGCAGTC

TATCAATTTTGTTGATCTTT

In100 candidate3957 (in intron):

LOCUS ENSG00000159658

DESCRIPTION Uncharacterized calcium-binding protein KIAA0494. [Source:Uniprt/SWISSPROT;Acc:O75071]

ACCGTAGAGTTTGTGAAGGTAACGTGTCCTCTACAAATTCTCATTTGTTTTCATTGTTTGTTACTCTTTTTTGTTCAGAT

TTCTCTGTTGACTTCTGCAG

In100 candidate3958 (in intron):

LOCUS ENSG00000091844

DESCRIPTION Regulator of G-protein signaling 17 (RGS17).

[Source:Uniprot/SWSSPROT;Acc:Q9UGC6]

TTCTCTCTTTTTTTCTTAGTCTAGCTAAAGGTTTTCCTATTTTGTTTAACTTTTCAAGAAACCAAATTTTTGTTTCAGTG

ATCTTTTTTTATTGTTTTCC

In100 candidate3959 (in intron):

LOCUS ENSG00000154198

DESCRIPTION CDNA FLJ40054 fis, clone TBAES2000315, weakly similar to

CYTOCHOME P450 4A1 (EC 1.14.15.3). [Source:Uniprot/SPTREMBL;Acc:Q8N1L4]

TAATCTTCTCAAAGAAATAACTTTTTGTTTGTTAATTTTTTTCTATTGTTTTTCCTGTTTTTATTTTATTTACCTCAGCT

CTAATCTTTATCATTTCCTT

In100 candidate3960 (in intron):

LOCUS ENSG00000154198

DESCRIPTION CDNA FLJ40054 fis, clone TBAES2000315, weakly similar to

CYTOCHOME P450 4A1 (EC 1.14.15.3). [Source:Uniprot/SPTREMBL;Acc:Q8N1L4]

TTGTGGTTAGGTTGTATTCTGTAGTGGTAACATTTGAGCTTTTTCTCTGTCTTGTTTGTGTGTTAGTTTTACCAGTGATC

TTTATATTTTCTAGTATTTT

In100 candidate3961 (in intron):

LOCUS ENSG00000186160

DESCRIPTION Cytochrome P450 4Z1 (EC 1.14.14.1) (CYPIVZ1).

[Source:Uniprot/SISSPROT;Acc:Q86W10]

TAATCTTCTCAAAGAAATAACTTTTTGTTTGTTAATTTTTTTCTATTGTTTTTCCTGCTTTTATTTTATTTACCTCAGCT

CTAATCTTTATCATTTCCTT

In100 candidate3962 (in intron):

LOCUS ENSG00000146426

DESCRIPTION Putative RNA-binding protein 16 (RNA-binding motif protein

16).[Source:Uniprot/SWISSPROT;Acc:Q9UPN6]

AGTGTTAACACTTTAGCTCTTATCCTCATCGCTTCTCCTTTGGTTTGTTGCAGTCAGTTTTTAATTGGCTCCTAATTTAC

TATGCTCTTTTTCTCTAGTT

In100 candidate3963 (in intron):

LOCUS ENSG00000146426

DESCRIPTION Putative RNA-binding protein 16 (RNA-binding motif protein

16).[Source:Uniprot/SWISSPROT;Acc:Q9UPN6]

AGAATCACCTGGAGGGCTTGCTAACGCATAAATCTCTGGGTCCTGTCCTCTGTTTCTGACTCAGAAGATCTTTTTTTTTT

TTTTTTTTTTTTTTTTTTGA

In100 candidate3964 (in intron):

LOCUS ENSG00000049618

DESCRIPTION AT-rich interactive domain-containing protein 1B (ARID

domainontaining

protein 1B) (Osa homolog 2) (hOsa2) (p250R) (BRG1-binding

proteinhELD/OSA1) (BRG1-associated factor 250b) (BAF250B).

[Source:Uniprot/SWISSPRT;Acc:Q8NFD5]

AGGCATGAGCCACCATGTCCTGCTATAAATAACTTTTAATAGGCTCTTTTTCTGGCTTTTTCTTTTAGTAACTCAGCCCA

TTTTCTTCTTCTCAATCTGG

In100 candidate3965 (in intron):

LOCUS ENSG00000049618

DESCRIPTION AT-rich interactive domain-containing protein 1B (ARID

domainontaining

protein 1B) (Osa homolog 2) (hOsa2) (p250R) (BRG1-binding

proteinhELD/OSA1) (BRG1-associated factor 250b) (BAF250B).

[Source:Uniprot/SWISSPRT;Acc:Q8NFD5]

AGATCATTAATTGGTTAACTAGACAATAATCATTAGTTTACCCATCTTATTTTTATGCCTTTGATTTTCCTTTCCAGTAA

CATAATCTTTTTTTGTTTCT

In100 candidate3966 (in intron):

LOCUS ENSG00000132122

DESCRIPTION Spermatogenesis-associated protein 6 precursor.

[Source:UniprotSWISSPROT;Acc:Q9NWH7]

TGAAAACTTGATAGTATAGCTTAGAATCATTACCTGATTTTAGTAACAAAAATAAAACTTATTTTTTGCCTACAGATTTC

ATTCTGATTGGTGTTCACCT

In100 candidate3967 (in intron):

LOCUS ENSG00000186094

DESCRIPTION ATP/GTP binding protein-like 4.

[Source:Uniprot/SPTREMBL;Acc:Q5U57]

TCTTAACCACTAAATACTTTGGATCCTTCTACTTCTCAGCATCTCCACTGCTTTTCTTCTCTTCTAGATTATCAACATCA

GACTCCTGGACTTTTATTAT

In100 candidate3968 (in intron):

LOCUS ENSG00000186094

DESCRIPTION ATP/GTP binding protein-like 4.

[Source:Uniprot/SPTREMBL;Acc:Q5U57]

AGTAGGGGTTATTAACAAAGACCACCTCTTTTTTTTCTGAGCAGTTCATTTATCATCCTTACCCTACTTAAATCATTTCT

TTGTTGTTGTTGTTGTTGTT

In100 candidate3969 (in intron):

LOCUS ENSG00000186094

DESCRIPTION ATP/GTP binding protein-like 4.

[Source:Uniprot/SPTREMBL;Acc:Q5U57]

CGTCAAATTTACTATTTTTCTGTTTTCTAACTTTTTTTATTTACATTTTTACTACTTCTTTCCTACTGCTTTCCACAGGT

TTCTTTTTGGTTCTTTTTCA

In100 candidate3970 (in intron):

LOCUS ENSG00000162373

DESCRIPTION Coiled-coil domain-containg protein C1orf165.

[Source:Uniprot/SISSPROT;Acc:Q7L4P6]

AGTAGGGGTTATTAACAAAGACCACCTCTTTTTTTTCTGAGCAGTTCATTTATCATCCTTACCCTACTTAAATCATTTCT

TTGTTGTTGTTGTTGTTGTT

In100 candidate3971 (in intron):

LOCUS ENSG00000122339

DESCRIPTION CDNA FLJ10029 fis, clone HEMBA1000817 (Chromosome 6 open readin frame 35). [Source:Uniprot/SPTREMBL;Acc:Q9NWH2]

CTAATGTGCTATATATTTTTCCACTATGTAAATTGTGCTGAACATAACTTATCATGTAACTTTTTCTTGGTGACTCAGAT

CTATCATTTATTGTTTGCTT

In100 candidate3972 (in intron):

LOCUS ENSG00000162374

DESCRIPTION ELAV-like protein 4 (Paraneoplastic encephalomyelitis antigen HD) (Hu-antigen D). [Source:Uniprot/SWISSPROT;Acc:P26378]

GCTGGATTTGCCTTAGGGTAACAAGACTATGGGTCCTTAAAAACCCGCTTCATCTTATTTTCCTTTGTGCAGCTTCATAC

AGTAGTGGTTTTCTCTTCTC

In100 candidate3973 (in intron):

LOCUS ENSG00000162374

DESCRIPTION ELAV-like protein 4 (Paraneoplastic encephalomyelitis antigen HD) (Hu-antigen D). [Source:Uniprot/SWISSPROT;Acc:P26378]

CTTAATTGATATGGTCCCATCTTTGCTGTCTGTATATTGTAACTTGCAAATTATTTGACTTTTTCCCAGAAACCAACTTA

CTTCAGATCTATTTTTTTTC

In100 candidate3974 (in intron):

LOCUS ENSG00000130340

DESCRIPTION Sorting nexin-9 (SH3 and PX domain-containing protein 1) (Proten SDP1) (SH3 and PX domain-containing protein 3A).

[Source:Uniprot/SWISSPRO;Acc:Q9Y5X1]

AGAGAGGTAGATACTTAAGAATGGGGCCTTAGCTTTTGCATTCTAACTCCCATATGAACTTTTTTTATTGCCAGGGTTTT

GTGAATTTTTTTTCCTTCCT

In100 candidate3975 (in intron):

LOCUS ENSG00000185104

DESCRIPTION FAS-associated factor 1 (Protein FAF1) (hFAF1).

[Source:UniprotSWISSPROT;Acc:Q9UNN5]

GTAACACACAAATGTGCTTTTGTTTTTCACCCCTAGTCTTTTCAGCTAGTAATACTTTTTGCTGCCTAAATTGCTTGATG

AATTTTCTTTTCTTTCTTTT

In100 candidate3976 (in intron):

LOCUS ENSG00000185104

DESCRIPTION FAS-associated factor 1 (Protein FAF1) (hFAF1).

[Source:UniprotSWISSPROT;Acc:Q9UNN5]

TTGCATTAATTGAGTTGTAACCTCGGATATGTCTGTATTTTATATTTCCTGCACATATCACTTGGACCTCTTCTCCAGTG

TTTTTGTGCACATTTTTGCT

In100 candidate3977 (in intron):

LOCUS ENSG00000185104

DESCRIPTION FAS-associated factor 1 (Protein FAF1) (hFAF1).

[Source:UniprotSWISSPROT;Acc:Q9UNN5]

AGTTTTATTTATTCTTTCAAGGAACTAACTCTTGACCTTATTGATCTTTTATATGCTGTTTTCATGTCTCAGTTTCCTTC

ATTTCAGCTTTGATTTTGGT

In100 candidate3978 (in intron):

LOCUS ENSG00000185104

DESCRIPTION FAS-associated factor 1 (Protein FAF1) (hFAF1).

[Source:UniprotSWISSPROT;Acc:Q9UNN5]

AAAGGAAATTGTTGGCTTTGTTGTTTGCTACAAGTGTGTAACTTTTTCCCCTAAGCTTTGTCTTAGTCTGTTTTCAGCAG

TGCATTCCTTTTCCTTTCCT

In100 candidate3979 (in intron):

LOCUS ENSG00000130338

DESCRIPTION Tubby-related protein 4 (Tubby-like protein 4) (Tubby superfamiy

protein). [Source:Uniprot/SWISSPROT;Acc:Q9NRJ4]

AATTCATTTAAAAAATACAAATAGATTTTCATGCAAAGGTCATGATTATAACTTCAAAAGAGCCTTTATTTTATGCAGCT

TTTATTTTGTTATAATGTCT

In100 candidate3980 (in intron):

LOCUS ENSG00000130338

DESCRIPTION Tubby-related protein 4 (Tubby-like protein 4) (Tubby superfamiy protein). [Source:Uniprot/SWISSPROT;Acc:Q9NRJ4]

AGGCACTTCATATATGTGTACCTTTACGTAAAAAATAATCATACATAACGCTTATTGAATCTTTCTGTGTGCCAGACAGT
TTTTGTTTTATTTCTCACAA

In100 candidate3981 (in intron):

LOCUS ENSG00000146433

DESCRIPTION Transmembrane protein 181. [Source:Uniprot/SWISSPROT;Acc:Q9P2C4

AATAGTTTGGGTCCCTTTCCCCTTTTCATAACATGATCATAGCACCTTTCATTTCCTTTTCTATCACAGCTGTAATTATT
TATTTATTTATTTATTTATT

In100 candidate3982 (in intron):

LOCUS ENSG00000164674

DESCRIPTION synaptotagmin-like 3 [Source:RefSeq_peptide;Acc:NP_001009991]

GATGCATGTAACCTAGAACCACCTCCACTTGCAATCTTGTGTCTTTCAATGTTCTTTCTGCTCAGATACTTTTTTCTCTG
TTTATCTTAAAATTCAAGTC

In100 candidate3983 (in exon):

LOCUS ENSG00000085832

DESCRIPTION Epidermal growth factor receptor substrate 15 (Protein Eps15) (F-1p protein). [Source:Uniprot/SWISSPROT;Acc:P42566]

CACCCCTTTATCTTCTCCTAGATTTACTTGTTAACCATTTTGTCACATTTGCTTTCTCCCTGTATCCATATTCTTCAGAA
TTTTTTCCTAGTTTTAAACT

In100 candidate3984 (in intron):

LOCUS ENSG00000085832

DESCRIPTION Epidermal growth factor receptor substrate 15 (Protein Eps15) (F-

1p protein). [Source:Uniprot/SWISSPROT;Acc:P42566]

TGTAACATAAATTAATCCTGCCCATCAACTTTATTATCCCTATTTTGTAATTCAGAAAGTTAAGGTTTCGTTTTATATTT

CTTTTCTGTTAGTTTCTTTT

In100 candidate3985 (in intron):

LOCUS ENSG00000117859

DESCRIPTION Oxysterol-binding protein-related protein 9 (OSBP-related proten

9) (ORP-9). [Source:Uniprot/SWISSPROT;Acc:Q96SU4]

GCCTTAGCTTAACACTCTCTATTCACTTGAGCTTTTCTTCTGTTCCTTTGAAGTTCTTCAAACTTGTTCTTGCCTCAGGG

CCTCTGAATGTTTTCTGTCT

In100 candidate3986 (in intron):

LOCUS ENSG00000164694

DESCRIPTION fibronectin type III domain containing 1

[Source:RefSeq_peptideAcc:NP_115921]

GGAGCCTCACACACATGAAAATTGGACAATTAACTTGCTCTGGTCATCTTCCTTTATTCCATCAGTTTATTCAAGTTTCT

CCTTCATTTTGTGATAACCT

In100 candidate3987 (in intron):

LOCUS ENSG00000146457

DESCRIPTION Wilms' tumor 1-associating protein (WT1-associated protein)

(Puative pre-mRNA-splicing regulator female-lethal(2D) homolog).

[Source:Uniprt/SWISSPROT;Acc:Q15007]

TAGGTGTGAGCCATCGCACCTGGCCCATTATCATTTTCTCTACCTTCTTAACACATTTTGTCCTTTTGTTTCTATCAGTT

TTGTTGTAATTCTTACCACC

In100 candidate3988 (in intron):

LOCUS ENSG00000157077

DESCRIPTION Zinc finger FYVE domain-containing protein 9 (Mothers against

dcapentaplegic homolog-interacting protein) (Madh-interacting protein)

(Smadanchor for receptor activation) (Receptor activation anchor) (hSARA)

(Novelserine protease) (NSP). [Source:Uniprot/SWISSPROT;Acc:O95405]

ACATTTTTAGAAGGTGGCTTATTAATTAACCTCTCCTGAACTTTGTCCTAGGCTTTTAACTGGTCTCCTTGCCTTCAGTC

TTTCTTTCCTTCATTTTGTC

In100 candidate3989 (in intron):

LOCUS ENSG00000134748

DESCRIPTION PRP38 pre-mRNA processing factor 38 (yeast) domain containing

Aisoform 2 [Source:RefSeq_peptide;Acc:NP_116253]

AGGCCTAGCACATAGATGTCCTGATATGAAGTCTTAACACTTCTTTTACTTTTTGGCATCTAAATCTTTTATTTCCAGGT

TGTTTGCTCTAATGACTTTT

In100 candidate3990 (in intron):

LOCUS ENSG00000162378

DESCRIPTION zyg-11 homolog B [Source:RefSeq_peptide;Acc:NP_078922]

TGTCCCAAAAAAATTAAAAAAAAGGAATTAGTAATAGTAGTGATACCTAACTATACCTACATGTTTATGTTTCTTCAGTT

TTTTTTCCTGAATTGTGTAT

In100 candidate3991 (in intron):

LOCUS ENSG00000162378

DESCRIPTION zyg-11 homolog B [Source:RefSeq_peptide;Acc:NP_078922]

ACATGCACTATTCCTGGCCCTCTGTGAGCACTATGCACTTTTCTCTAACTCTTTAGATGGTTATTTTCCTGCCCTCAGCT

TCCTTGTGTTCATTTTATTA

In100 candidate3992 (in intron):

LOCUS ENSG00000203995

DESCRIPTION Zyg-11 homolog A. [Source:Uniprot/SPTREMBL;Acc:Q6WRX3]

ACCCATATCTTTCCATCCAAAACTATCTCTGTTAACATTTTGTTGCATATGACTTACTTCGGTCCTCTTTCTCATCAGCT

TTTTTTTTTAAAACTATTT

In100 candidate3993 (in intron):

LOCUS ENSG00000116171

DESCRIPTION Nonspecific lipid-transfer protein (EC 2.3.1.176) (Propanoyl-Co

C- acyltransferase) (NSL-TP) (Sterol carrier protein 2) (SCP-2) (Sterol carier

protein X) (SCP-X) (SCP-chi) (SCPX). [Source:Uniprot/SWISSPROT;Acc:P2207]

AGAATTCCCTTCCCAACTACAAAAGGGAGCCATGATGGATCTGCTTAACTGGTTCTTGTTTGTTTGTTTGCCAGTAATAA

ACCTCTTGTTTTTTTTTTCT

In100 candidate3994 (in intron):

LOCUS ENSG00000116171

DESCRIPTION Nonspecific lipid-transfer protein (EC 2.3.1.176) (Propanoyl-Co

C- acyltransferase) (NSL-TP) (Sterol carrier protein 2) (SCP-2) (Sterol carier

protein X) (SCP-X) (SCP-chi) (SCPX). [Source:Uniprot/SWISSPROT;Acc:P2207]

TTGCTAGCTAGAGATAACTAGGAGAAAGCCCTGAATTTCTGTTTCTATTTCTTTTCTTTTTTTATTTTTGAAACCAGGAT

TTCGCTCTGTTGCTCTGGCT

In100 candidate3995 (in intron):

LOCUS ENSG00000157184

DESCRIPTION Carnitine O-palmitoyltransferase 2, mitochondrial precursor

(EC2.3.1.21) (Carnitine palmitoyltransferase II) (CPT II).

[Source:Uniprot/SWISPROT;Acc:P23786]

GCCACAAAATTACTCCCTAACGCGGTTATACTAATTTTATTCTCACTCTTTTCTCATATCATTTTTTAAATTCGGCAGAT

TTTTTTCTGTTTTTATTCTA

In100 candidate3996 (in intron):

LOCUS ENSG00000122194

DESCRIPTION Plasminogen precursor (EC 3.4.21.7) [Contains: Plasmin heavy chin

A; Activation peptide; Angiostatin; Plasmin heavy chain A, short form; Pasmin

light chain B]. [Source:Uniprot/SWISSPROT;Acc:P00747]

TGCTAACATCCCCTTTGTCATTTCTAATTGTGTTTATTTTGGTCTTATCTTCCTTTTCTTCATTAGCCTAGCTAGCAGCC

TACCTATCTTATTACTGTTT

In100 candidate3997 (in intron):

LOCUS ENSG00000058804

DESCRIPTION Nucleoporin NDC1 (hNDC1) (Transmembrane protein 48).

[Source:Unprot/SWISSPROT;Acc:Q9BTX1]

ACATAGATGAGTAAGTGATACCTAAAAAGTCTAATTTTAACTCTGTTTCCATTCTTTTAGATGTTTGCTATTTTCCAGAT

ATTTTGTTTAGATTTTGCCT

In100 candidate3998 (in intron):

LOCUS ENSG00000085511

DESCRIPTION Mitogen-activated protein kinase kinase kinase 4 (EC

2.7.11.25)(MAPK/ERK kinase kinase 4) (MEK kinase 4) (MEKK 4) (MAP three kinase

1). [Surce:Uniprot/SWISSPROT;Acc:Q9Y6R4]

AGTCCTGTAATTAGAACTTGCCAAATAAAACATTCTATATAGCTAACTCCTGTACTTTTTATTCATTTTCCTTTTCAGAA

TTCTGCTCTGTTTCCATTTG

In100 candidate3999 (in intron):

LOCUS ENSG00000185345

DESCRIPTION Parkin (EC 6.3.2.-) (Ubiquitin E3 ligase PRKN) (Parkinson juvenle

disease protein 2) (Parkinson disease protein 2).

[Source:Uniprot/SWISSPOT;Acc:O60260]

CATTAGAAAGCATCCTTTATATGTATTAGAAATTTAACTTAGAATTTAATTAACTTTTCTATATTCACTCTACAGCTACT

TTTCTTAAAAGCTATCTTTC

In100 candidate4000 (in intron):

LOCUS ENSG00000185345

DESCRIPTION Parkin (EC 6.3.2.-) (Ubiquitin E3 ligase PRKN) (Parkinson juvenle disease protein 2) (Parkinson disease protein 2).

[Source:Uniprot/SWISSPOT;Acc:O60260]

GAGTCTTCTAACCTGTAACATAACATACTTCATTTACTTGAGGCTACTTTATTTCCTCTCAGTGGTGTTTTGTTTTGGGT
TTTCAATGTCTCTTCTGTTT

In100 candidate4001 (in intron):

LOCUS ENSG00000185345

DESCRIPTION Parkin (EC 6.3.2.-) (Ubiquitin E3 ligase PRKN) (Parkinson juvenle disease protein 2) (Parkinson disease protein 2).

[Source:Uniprot/SWISSPOT;Acc:O60260]

ATATACATACCTATATTTACACTGTATTCATATAACTGATAAGTTTATATTTGTTTATAACATCTCTTTTCTACAGTTCC
TCTTCTATTACCTTTTCCTT

In100 candidate4002 (in intron):

LOCUS ENSG00000185345

DESCRIPTION Parkin (EC 6.3.2.-) (Ubiquitin E3 ligase PRKN) (Parkinson juvenle disease protein 2) (Parkinson disease protein 2).

[Source:Uniprot/SWISSPOT;Acc:O60260]

AGCCACTCTTGATAACTGAGCATTCTCTTTTCCTGCTCTGTGCTTTCACAGCTCCATTCTTTTCTCTTGACCTCAGGTGT
TGTCTGCATCTTTATGCACT

In100 candidate4003 (in intron):

LOCUS ENSG00000185345

DESCRIPTION Parkin (EC 6.3.2.-) (Ubiquitin E3 ligase PRKN) (Parkinson juvenle

disease protein 2) (Parkinson disease protein 2).

[Source:Uniprot/SWISSPOT;Acc:O60260]

AGAATGAATGCTTAATGTGACCAAGCCCGTATGTTAAGTAGATAACTCACGGTGATCTCTCTCTTTCATTTACAGGTCTT

TTCTCTCTCTTATAATTAAC

In100 candidate4004 (in intron):

LOCUS ENSG00000112530

DESCRIPTION Parkin coregulated gene protein (PARK2 coregulated) (Molecular

haperone/chaperonin-binding protein). [Source:Uniprot/SWISSPROT;Acc:Q96M98]EXON

TAGGAAGGACCTCAATGTATAACTATTGATATGACTGTCATCCTTAGTTCAGTTCTGAGATTTCCATTCCTAACTTATCA

TTGCATCTACATTCTATTTC

In100 candidate4005 (in intron):

LOCUS ENSG00000112530

DESCRIPTION Parkin coregulated gene protein (PARK2 coregulated) (Molecular

haperone/chaperonin-binding protein). [Source:Uniprot/SWISSPROT;Acc:Q96M98]EXON

CTATAACATCATATTTGCTGATTTCATTCTGAATCTTTCCAGGTCATGGTTTTGCCTGCTTTTATGTAATATTCATTTGT

GTTTTATTTTTATCCATGCC

In100 candidate4006 (in intron):

LOCUS ENSG00000112531

DESCRIPTION Quaking protein (Hqk). [Source:Uniprot/SWISSPROT;Acc:Q96PU8]

CTGAAAATACATTTCCAATTTTATTTTTCTAAATTATAACCGCTAGTCATGTCATTTTATTTGTTGTTCAGTCATCCCTT

CCCAGTATTTCTGTTTAATT

In100 candidate4007 (in intron):

LOCUS ENSG00000112531

DESCRIPTION Quaking protein (Hqk). [Source:Uniprot/SWISSPROT;Acc:Q96PU8]

TCTATTTCATTTCATATGATAGTAACTTTTTCCTTCTTTTAATTTTGAGGTCTTTATTTTTTTCTCCCCAAATACCAGTT

ATTTCATGCAGCATCTTTTC

In100 candidate4008 (in intron):

LOCUS ENSG00000112531

DESCRIPTION Quaking protein (Hqk). [Source:Uniprot/SWISSPROT;Acc:Q96PU8]

TTTTCAATTTCCTTCATTGACTGCTACTAACTATTAGTTGTTAGTTGTATTTTATTTCTATTTTTTCTTGTTAACCAGTT

TTTTTTTTTTTTTTGTGGGA

In100 candidate4009 (in intron):

LOCUS ENSG00000116209

DESCRIPTION Transmembrane protein 59 precursor (Liver membrane-bound protei).

[Source:Uniprot/SWISSPROT;Acc:Q9BXS4]

TTGTCTGTATTTTTAAATTAACTTCCAAACTGTTTTTCTAATTTAACCCTGTCTAACTCCATCCTTTGCTGTGTCCAGAT

TTTTTTTTCTAAATTTCATC

In100 candidate4010 (in intron):

LOCUS ENSG00000112541

DESCRIPTION cAMP and cAMP-inhibited cGMP 3',5'-cyclic phosphodiesterase

10A(EC 3.1.4.17). [Source:Uniprot/SWISSPROT;Acc:Q9Y233]

CCCAATGGGTTTAACACTTCACTTGGATTCTTATTACTTTTTCTTTTTTTTTTTTAATTGAATGCCTCCTACACTCAGCA

TTCCTTACTTCTATTTGATC

In100 candidate4011 (in intron):

LOCUS ENSG00000112541

DESCRIPTION cAMP and cAMP-inhibited cGMP 3',5'-cyclic phosphodiesterase

10A(EC 3.1.4.17). [Source:Uniprot/SWISSPROT;Acc:Q9Y233]

TGTGTCTTTTATGTCTACTGTAGGAAATCATTGCCTAACTCAATATATTCTCCTGTTTTCTTATACACATTTTACAGTTT

TCACCCTTATATGTGCATCT

In100 candidate4012 (in intron):

LOCUS ENSG00000112541

DESCRIPTION cAMP and cAMP-inhibited cGMP 3',5'-cyclic phosphodiesterase

10A(EC 3.1.4.17). [Source:Uniprot/SWISSPROT;Acc:Q9Y233]

AGCCTGTTTCTCCAAACTTAACTACGTTCCATGTTTCACTTCATTCACTGTGTCGCAGCCATAATGGCATTTCTTTTTGT

TCCTCAAACCTGCCTTGCTT

In100 candidate4013 (in intron):

LOCUS ENSG00000071242

DESCRIPTION Ribosomal protein S6 kinase alpha-2 (EC 2.7.11.1) (S6K-alpha

2)(90 kDa ribosomal protein S6 kinase 2) (p90-RSK 2) (Ribosomal S6 kinase 3)

RSK-3) (pp90RSK3) (MAP kinase-activated protein kinase 1c) (MAPKAPK1C).

[Sorce:Uniprot/SWISSPROT;Acc:Q15349]

CTTCCACCCTGTGGGTTGTCTGTTAACTCTGCTGATCATTTCTTTTGCTGTGCAGAAGCTTTTTAATTTAATTAAGTCCC

ATCTATTTATCTTTGTTTTT

In100 candidate4014 (in intron):

LOCUS ENSG00000071242

DESCRIPTION Ribosomal protein S6 kinase alpha-2 (EC 2.7.11.1) (S6K-alpha

2)(90 kDa ribosomal protein S6 kinase 2) (p90-RSK 2) (Ribosomal S6 kinase 3)

RSK-3) (pp90RSK3) (MAP kinase-activated protein kinase 1c) (MAPKAPK1C).

[Sorce:Uniprot/SWISSPROT;Acc:Q15349]

CTTCTGCTGCTTTTCACCGTGTAACAAATCAAGGTTTTTTTCACTCTTGCCTTTTACTCCAGTGTCTTCTGGTCATCAAG

GTTTGATTCTTTTACCTCCC

In100 candidate4015 (in intron):

LOCUS ENSG00000071242

DESCRIPTION Ribosomal protein S6 kinase alpha-2 (EC 2.7.11.1) (S6K-alpha

2)(90 kDa ribosomal protein S6 kinase 2) (p90-RSK 2) (Ribosomal S6 kinase 3)

RSK-3) (pp90RSK3) (MAP kinase-activated protein kinase 1c) (MAPKAPK1C).

[Sorce:Uniprot/SWISSPROT;Acc:Q15349]

CCATATTTCCTAACCCCCCTTTCATATTTCCTGTATTTCTGTCTTTCTCTCAATTATCATCAGATCCAACGTCTCTTTTA

CCAACTCTTTGTTGGACTTT

In100 candidate4016 (in intron):

LOCUS ENSG00000071242

DESCRIPTION Ribosomal protein S6 kinase alpha-2 (EC 2.7.11.1) (S6K-alpha

2)(90 kDa ribosomal protein S6 kinase 2) (p90-RSK 2) (Ribosomal S6 kinase 3) RSK-3) (pp90RSK3) (MAP kinase-activated protein kinase 1c) (MAPKAPK1C). [Sorce:Uniprot/SWISSPROT;Acc:Q15349]

TCTGCCCTGGAGGTAATGCTGAAATTTGACTTTGTGTATAACTGATGTGTGTTACCTTTGTCATGTCTTCAGAATAAACT
GTGGTCATTCTTTTTCTTTC

In100 candidate4017 (in intron):

LOCUS ENSG00000071242

DESCRIPTION Ribosomal protein S6 kinase alpha-2 (EC 2.7.11.1) (S6K-alpha 2)(90 kDa ribosomal protein S6 kinase 2) (p90-RSK 2) (Ribosomal S6 kinase 3) RSK-3) (pp90RSK3) (MAP kinase-activated protein kinase 1c) (MAPKAPK1C). [Sorce:Uniprot/SWISSPROT;Acc:Q15349]

AAAGGGAATAATTATACGGGACATGCTTGTGTCTGTACCCATTAACTTAGAGTAAACTTGCATTTTTTTACTTACAGACT
TGTTTTTTTCCCAATACCCT

In100 candidate4018 (in intron):

LOCUS ENSG00000157216

DESCRIPTION Single-stranded DNA-binding protein 3 (Sequence-specific single stranded-DNA-binding protein). [Source:Uniprot/SWISSPROT;Acc:Q9BWW4]

AGGACTAACTTTGTGCTAACTTTTCGAGTATTTCTTTTCAGGCTGTTTTCCTACTTTTACTTTACTTTTTTCACATCATC
TTCTTCTGGTCAGAAAATTC

In100 candidate4019 (in intron):

LOCUS ENSG00000162402

DESCRIPTION Ubiquitin carboxyl-terminal hydrolase 24 (EC 3.1.2.15) (Ubiquitn thioesterase 24) (Ubiquitin-specific-processing protease 24) (Deubiquitinting enzyme 24). [Source:Uniprot/SWISSPROT;Acc:Q9UPU5]

GAATAATTTGAAGTATAGGAGACCTAACATTTGAGAAGGCCATTTACCTTTTCAGTTTATCATTTTATAAAATATAATTT
TTATTTATTTTTCATTTTCT

In100 candidate4020 (in intron):

LOCUS ENSG00000130396

DESCRIPTION Afadin (Protein AF-6). [Source:Uniprot/SWISSPROT;Acc:P55196]

TTTTGATAACTTTTTGTTCATTTATTGCCATTCTCTCTTGTAGCTCGTTCTACAGGTGAATTTCAGATACAGCTTTGGTT
CATCCTTTTTTGTCCTCCTT

In100 candidate4021 (in intron):

LOCUS ENSG00000173406

DESCRIPTION Disabled homolog 1. [Source:Uniprot/SWISSPROT;Acc:O75553]

TAACAATGCCCACTTGCCTTTTTTGTTTCTCTCACATTATTTCAAACTACTGTATCACATAGTTACTTTACTTCCAGGTG
TCTCACTTCTTCTATACTCT

In100 candidate4022 (in intron):

LOCUS ENSG00000173406

DESCRIPTION Disabled homolog 1. [Source:Uniprot/SWISSPROT;Acc:O75553]

TCCCTCCCTCTCTGCTTTATTTAACTTTCATTTTCTTTCATTCTTCTTCAATTTGTTCATACCATGCCACTTCAGCTCCC
TTACTGGTCTACACTTTTAT

In100 candidate4023 (in intron):

LOCUS ENSG00000173406

DESCRIPTION Disabled homolog 1. [Source:Uniprot/SWISSPROT;Acc:O75553]

AGTGTGCTTATGATGCCCATTTAACATAAAATTGAATTGATTTGCTTCAGTCTGCTTTTAGTTCTACGATCCCCCTTACC

ATCCCTATTAATTTAATTTT

In100 candidate4024 (in intron):

LOCUS ENSG00000112562

DESCRIPTION SPARC-related modular calcium-binding protein 2 precursor

(Secrted modular calcium-binding protein 2) (SMOC-2) (Smooth muscle-associated

potein 2) (SMAP-2). [Source:Uniprot/SWISSPROT;Acc:Q9H3U7]

TAACAACAAAATGAATCTCTTTGTTTTTGTTTCTATATTTTTCACTCAGTGGAATAGGCCTCCATCCTGCCGCTGCTTTC

TGTGGTCTTTTCTTTCTTTT

In100 candidate4025 (in intron):

LOCUS ENSG00000112562

DESCRIPTION SPARC-related modular calcium-binding protein 2 precursor

(Secrted modular calcium-binding protein 2) (SMOC-2) (Smooth muscle-associated

potein 2) (SMAP-2). [Source:Uniprot/SWISSPROT;Acc:Q9H3U7]

AGGGTCTGTGCTCTCAAGCACTAACGGTGACCTGCACTACATTCCTTTTTTTTTTTTTTTTTGTGAGACAGAGTTTTGC

TTTTGTTGCCCAGGCTTCTG

In100 candidate4026 (in intron):

LOCUS ENSG00000172456

DESCRIPTION Novel protein. [Source:Uniprot/SPTREMBL;Acc:Q5VSS0]

TTGTAACTATTTTATATTTGTAACATTTATTCTTTGTTTACCTTTTTTTCCTCTTGGCCTTTCACTGGTATTCATCAGGC

ATTTTATGTGATTTCATTTT

In100 candidate4027 (in intron):

LOCUS ENSG00000130023

DESCRIPTION CDNA FLJ11152 fis, clone PLACE1006901. (Fragment).

[Source:Unipot/SPTREMBL;Acc:Q9NUT5]

GTTAATGGGGAACTGAAGGGAGTATGGGTAATTTGGGGCCTAACCCAAAATCTTGTTTTCATTTGTTTTTTTGGTCAGTT

TTTCTTCATTTTATTTGGCA

In100 candidate4028 (in intron):

LOCUS ENSG00000162598

DESCRIPTION Uncharacterized protein C1orf87.

[Source:Uniprot/SPTREMBL;Acc:QN0U7]

TGGCTTGTTATTGTGTAACTCCAATCTCTGCCTTTGTTTTCACAAGGCCTTCTTCTTTCTGTCTTCTATATATCTCAGTA

TCCAAGTGTTCCTCTTCTTT

In100 candidate4029 (in intron):

LOCUS ENSG00000162598

DESCRIPTION Uncharacterized protein C1orf87.

[Source:Uniprot/SPTREMBL;Acc:QN0U7]

AGAGACTCTAACACCATCAAGACTTCCTATATCTTTTTTCTTCTCTCGTTGTCAGCTTCATATTCTCTCTCTTTCTCAGT

AGACCAGCTTTTTCTCATTT

In100 candidate4030 (in intron):

LOCUS ENSG00000162599

DESCRIPTION Nuclear factor 1 A-type (Nuclear factor 1/A) (NF1-A) (NFI-A) (NI/A) (CCAAT-box-binding transcription factor) (CTF) (TGGCA-binding protein. [Source:Uniprot/SWISSPROT;Acc:Q12857]

TGTATGCTATTATTTAACTAAAACCAAGCAAGCATTTTCTCCTTCTCTTCAGTTATAGGTTAACATGAGATTTTCATTTT
TTTTTTCTTTTGGCTTTTCT

In100 candidate4031 (in intron):

LOCUS ENSG00000162599

DESCRIPTION Nuclear factor 1 A-type (Nuclear factor 1/A) (NF1-A) (NFI-A) (NI/A) (CCAAT-box-binding transcription factor) (CTF) (TGGCA-binding protein. [Source:Uniprot/SWISSPROT;Acc:Q12857]

TCTGGCTTCTGTATCTACCTTTCTAACCCCACTTTTTTTTTTTTTTTTTTTTTAACCGTGACTCCTTAGATGCTCCAGCC
ATACTGGTTTCCTTTCAGTT

In100 candidate4032 (in intron):

LOCUS ENSG00000125703

DESCRIPTION Cysteine protease ATG4C (EC 3.4.22.-) (Autophagy-related protei 4 homolog C) (Autophagin-3) (Autophagy-related cysteine endopeptidase 3) (UT-like 3 cysteine endopeptidase). [Source:Uniprot/SWISSPROT;Acc:Q96DT6]

ACAATTTTGTCTTTATTTTTAGTTAGCTCTAAGATTTTAACCTGATATGCTTTCTCTGTTTAATTTTTTCAGTTGCAGCT

CTCATTCAGTTTATCATTTC

In100 candidate4033 (in intron):

LOCUS ENSG00000158966

DESCRIPTION cache domain containing 1

[Source:RefSeq_peptide;Acc:NP_065976]EXON

CTATCATTTGCTAACTCACATGAAACTTCATGGGATTTTTTTTTCCCCACTTGGATTACTCCGTGTGCCTCTGCCAGAA

TACTGGCCTTCTTTGTTCTA

In100 candidate4034 (in intron):

LOCUS ENSG00000158966

DESCRIPTION cache domain containing 1

[Source:RefSeq_peptide;Acc:NP_065976]EXON

TACTAAAGATTAGTTAGAACTAGACAATGTTTTTATAAGTAATTTTAACTAATGGCTAATTTTTACTTTTTGTCTCAGGC

CTAACTTTTCATTTTTAATG

In100 candidate4035 (in intron):

LOCUS ENSG00000162437

DESCRIPTION Ribonucleoprotein PTB-binding 2 (Protein raver-2).

[Source:Unipot/SWISSPROT;Acc:Q9HCJ3]

ATTGGCATAACATTGTTCATAGTATTCCCTTATTATTCTTTAATCTCTGCAGGATCTATAGTGATGGCCTCTTTCACATT

CCTGATATTTTTGTCTTCTC

In100 candidate4036 (in intron):

LOCUS ENSG00000116675

DESCRIPTION Putative tyrosine-protein phosphatase auxilin (EC 3.1.3.48) (DnJ

homolog subfamily C member 6). [Source:Uniprot/SWISSPROT;Acc:O75061]

AAGCTAGCTCCTGAATGCTTCTAACACACTCAAGTAGTCTTTGATGCTTTCCTTGCTTTCTGGTATCACCAGATGTTCCA

GGCTCATCTTGTATGTTTTC

In100 candidate4037 (in intron):

LOCUS ENSG00000116675

DESCRIPTION Putative tyrosine-protein phosphatase auxilin (EC 3.1.3.48) (DnJ

homolog subfamily C member 6). [Source:Uniprot/SWISSPROT;Acc:O75061]

TAACAATATTATTTCTTCTAATCCATGAGCATGGAATACCTTTCCTTTTTTTGTGTCCTCTTCTATTTCTTTCATCAGAG

TTTTACAGTTCTTCTTGTGT

In100 candidate4038 (in intron):

LOCUS ENSG00000116678

DESCRIPTION Leptin receptor precursor (LEP-R) (OB receptor) (OB-R)

(HuB219)(CD295 antigen). [Source:Uniprot/SWISSPROT;Acc:P48357]

AGAAAATTGCCGCAAATATCTTCTAAAGATAACCACAAATTGCACTTTATGTGTTTCCATTCAGTTTTTCTTCTTTTTTT

GTAGCTATAATCTTTTAGAC

In100 candidate4039 (in intron):

LOCUS ENSG00000184588

DESCRIPTION cAMP-specific 3',5'-cyclic phosphodiesterase 4B (EC 3.1.4.17)

(PDE4) (PDE32). [Source:Uniprot/SWISSPROT;Acc:Q07343]

AGCTGTAGATTTCCCTGAACATGCTGCTTTAGCTGCATCCTACAAATTTTAACATATAATGTTTTCGTTGTCATTCAGTT

CAAAATACTTTTCTCTTTTC

In100 candidate4040 (in exon):

LOCUS ENSG00000184588

DESCRIPTION cAMP-specific 3',5'-cyclic phosphodiesterase 4B (EC 3.1.4.17)

(PDE4) (PDE32). [Source:Uniprot/SWISSPROT;Acc:Q07343]

GATGAACTCACACTGAGATCACCTAATGCCTGACACCTAACGATGCCTCACATGCTTTTCTATTTCCAGCAGCGCTTCAT

ACTTAAAATCTTTCTCTCTT

In100 candidate4041 (in intron):

LOCUS ENSG00000118473

DESCRIPTION SH3-containing GRB2-like protein 3-interacting protein 1

(Endopilin- 3-interacting protein). [Source:Uniprot/SWISSPROT;Acc:Q9BQI5]

CTTCTGAGACTCCAAATGATAACTATTTTGTTATAGTCTCACATGTCCTTAAGGCTCTGTTCATTTTTTTTTCTCAGTC

TATTTTCTCTATTTTCTGAT

In100 candidate4042 (in intron):

LOCUS ENSG00000118473

DESCRIPTION SH3-containing GRB2-like protein 3-interacting protein 1

(Endopilin- 3-interacting protein). [Source:Uniprot/SWISSPROT;Acc:Q9BQI5]

TTCTCCTTGGATCTTCTGAAAGATTCATTAAGATATGAATAACTTACTTTCATAACTTCATTTGCTTTAGTCTTTCAGTT

TTCTTCATATTTTTTGTAAA

In100 candidate4043 (in intron):

LOCUS ENSG00000116704

DESCRIPTION UDP-glucuronic acid/UDP-N-acetylgalactosamine transporter (UDPGlcA/ UDP-GalNAc transporter) (Solute carrier family 35 member D1) (UDP-galatose transporter-related protein 7) (UGTrel7). [Source:Uniprot/SWISSPROT;Ac:Q9NTN3]

TCATAATTACATCTTGTAACTGACCATGACTTTATCTTATTTTCCTACCCATATTTTCTCTTTAGTCAGATGACTTTAAC
TGCTTAGTTTTTAGTCTTTT

In100 candidate4044 (in intron):

LOCUS ENSG00000142864

DESCRIPTION Plasminogen activator inhibitor 1 RNA-binding protein (PAI1 RNA binding protein 1) (PAI-RBP1) (SERPINE1 mRNA-binding protein 1). [Source:Uiprot/SWISSPROT;Acc:Q8NC51]

GGCTTCCTGTTTGGCTTTAAAAACTCATACAAGCCCTAACTATATTATTTCTTCTCCTAAATTCATTTTCTCTATCAGCT
TCCTTTAACCTTTTGACATT

In100 candidate4045 (in intron):

LOCUS ENSG00000116729

DESCRIPTION Integral membrane protein GPR177 precursor (Protein wntless homlog) (Putative NFkB-activating protein 373). [Source:Uniprot/SWISSPROT;Acc:5T9L3]

TGGTTTTCTTAGCTCTGGTAACATTGTTATCAATTCTTCATTTATATTCCATTTTCCAGAAGTTTGTTGCTGGTATCTCA

TCTCATATTTTGCCCATTCT

In100 candidate4046 (in intron):

LOCUS ENSG00000024526

DESCRIPTION DEP domain containing 1 [Source:RefSeq_peptide;Acc:NP_060249]

AACAATATATACTATAGTAATGCTTTATTTTTAACTTTACTATTGTTTTTGTTTTATTTTTGTCCACTGTCATACAGTT

GCATCTTAATTTTTGTTTCA

In100 candidate4047 (in intron):

LOCUS ENSG00000033122

DESCRIPTION Leucine-rich repeat-containing protein 7 (Protein LAP1)

(Densin180). [Source:Uniprot/SWISSPROT;Acc:Q96NW7]

CTGTGAATGTTTTCTCCATTACATTCCTAACATTGTTTCACACCATTTATCTTTTAGTCTTTTTAGCAACTTTTCCAGAG

GTTTTTCTACTTTATTAATC

In100 candidate4048 (in intron):

LOCUS ENSG00000033122

DESCRIPTION Leucine-rich repeat-containing protein 7 (Protein LAP1)

(Densin180). [Source:Uniprot/SWISSPROT;Acc:Q96NW7]

CAAATAAAGATACTGCCACTTTGGTTAACCATTGTTTAAATGTTTACTTTCCTTGTCCTGTATTATTATATCTCTCAGGA

TTCTTTTGTTCATCCAGCTA

In100 candidate4049 (in intron):

LOCUS ENSG00000116754

DESCRIPTION Splicing factor arginine/serine-rich 11 (Arginine-rich 54 kDa

nclear protein) (p54). [Source:Uniprot/SWISSPROT;Acc:Q05519]

AATTAGCATTTGTTTTAACTGCTATTCTGTTTCTCTTTTATGTTATTTGTTTATTTTTTAGTGATTCGCCTTTGCCAGTC

TCATCTCGTGTCTGCTTTGT

In100 candidate4050 (in intron):

LOCUS ENSG00000050628

DESCRIPTION Prostaglandin E2 receptor, EP3 subtype (Prostanoid EP3 receptor

(PGE receptor, EP3 subtype) (PGE2-R). [Source:Uniprot/SWISSPROT;Acc:P43115

AACCATGCTGAACTTTTTAACTCCCTGTGAAAAGACCATGCATTTTTGTAATTTCCTGCTATTCCCTTTTCCCAGCTTAC

TTCCCATTCGTTTCATCTTG

In100 candidate4051 (in intron):

LOCUS ENSG00000132485

DESCRIPTION Zinc finger Ran-binding domain-containing protein 2 (Zinc finge

protein 265) (Zinc finger, splicing). [Source:Uniprot/SWISSPROT;Acc:O95218

GCAAATTTTATTTGATAACTTCTCACTTTTCTTATTGTCATTTTTCAGTCATTCTGGTGTCTTGAACCTTTCTCACTTCC

CTTGCTACAAGTATTTGTCT

In100 candidate4052 (in intron):

LOCUS ENSG00000132485

DESCRIPTION Zinc finger Ran-binding domain-containing protein 2 (Zinc finge

protein 265) (Zinc finger, splicing). [Source:Uniprot/SWISSPROT;Acc:O95218

TTAACTACTAGAGTTATAGTTCACTTTCTAATTTTACTATATTTTTGAGCTTTCCATATTTGTCATATTACTTTTCAGTT

TTTTCCTATGTAAATTATTT

In100 candidate4053 (in intron):

LOCUS ENSG00000172260

DESCRIPTION Neuronal growth regulator 1 precursor.

[Source:Uniprot/SWISSPRO;Acc:Q7Z3B1]

TAATAACTGCTTAATACATGTGCTTTCTCATCCTAACATTTTATTTCAGAGTCAATTTTGATTGATATATTTTCCTTCAT

TACGGTTTTTATTGCTTTTT

In100 candidate4054 (in intron):

LOCUS ENSG00000172260

DESCRIPTION Neuronal growth regulator 1 precursor.

[Source:Uniprot/SWISSPRO;Acc:Q7Z3B1]

TTCTTTCGAAATTTCTGTAGATAACTCCATATGAAACTACATATTTTTTTCTAATTTTTCCTATGGTAGTCTCTTCAGGA

TCTTTCATTTTTCATATTGA

In100 candidate4055 (in intron):

LOCUS ENSG00000172260

DESCRIPTION Neuronal growth regulator 1 precursor.

[Source:Uniprot/SWISSPRO;Acc:Q7Z3B1]

TTTTGCTTGTTCCAAACTTCTTTCTGTTAAAAACAAATATAACAACGACAAAAAAATCTTTCTCTTACATATCTGCAGGC

TCATGTCTTTACCATTTTTG

In100 candidate4056 (in exon):

LOCUS ENSG00000172260

DESCRIPTION Neuronal growth regulator 1 precursor.

[Source:Uniprot/SWISSPRO;Acc:Q7Z3B1]

TTTATCTTTTAATCTATAACAATTTGTGTTAGCTGTTCATTTCAGGATTATATTTTCTACAAGTTCCACTTGTGGGACTC

CTTTTGTTGCCCCTATTTTT

In100 candidate4057 (in intron):

LOCUS ENSG00000162620

DESCRIPTION LRRC44 protein. [Source:Uniprot/SPTREMBL;Acc:Q6P5P7]

TCCTCACTTGTTCCTTTCCCAACACTCTTTCTTAACATAGATATGAGTATCTGACCTATATCATTTCTCTTCTCTCAGAA

CTTCTTTTAACATTTCTTAC

In100 candidate4058 (in intron):

LOCUS ENSG00000162620

DESCRIPTION LRRC44 protein. [Source:Uniprot/SPTREMBL;Acc:Q6P5P7]

CCATAACCAATCAAGCTGTTTCTATATTTCTTCCATTTTCTATCTATAAATACTCCCTGCTCATTTATTACGCAGAGTTT

TCTAAATTTTCACTGGTTCT

In100 candidate4059 (in intron):

LOCUS ENSG00000116783

DESCRIPTION Serine/threonine-protein kinase TNNI3K (EC 2.7.11.1) (TNNI3-ineracting kinase) (Cardiac ankyrin repeat kinase).

[Source:Uniprot/SWISSPROTAcc:Q59H18]

GACTACTTGTGATAACTAATTACCTAAGGCTGGAGTAATTTTTCATTCTTTCCCTTTCCTGCTTCTACTTACACTCAGTC
ACCTTTTCTGGATGTCTCTT

In100 candidate4060 (in intron):

LOCUS ENSG00000137968

DESCRIPTION Choline transporter-like protein 5 (Solute carrier family 44 meber 5). [Source:Uniprot/SWISSPROT;Acc:Q8NCS7]

AAATATAGCTAGAAAGAAATGTTAGAAAACCCAAGTTGATAACCTTCAAGCTCAATTCATGTTCTTGTTTGCTTCCAGAC
TTTCTTTTTAAATCACTATT

In100 candidate4061 (in intron):

LOCUS ENSG00000057468

DESCRIPTION MutS protein homolog 4. [Source:Uniprot/SWISSPROT;Acc:O15457]

CCTTTAGGGAAACTTAAGCAATATATTTAACTTTATGCATGATTCATGGCCTTCATTATTTCTTATAATGTTTCTCAGTT
ATCATTTTTTTAACCATCTG

In100 candidate4062 (in intron):

LOCUS ENSG00000184005

DESCRIPTION Alpha-N-acetylgalactosaminide alpha-2,6-sialyltransferase 3 (EC2.4.99.-) (GalNAc alpha-2,6-sialyltransferase III) (ST6GalNAc III) (Sialyltansferase 7C) (STY). [Source:Uniprot/SWISSPROT;Acc:Q8NDV1]

TTACTTTTCACTCTTTAAATTACTGGCTTTCTGTAACTTCCTTCTGTGTTTTCTGACATCTACAGTTTCACAATTATTAT
TCTTCAAGATTTGTCTTCCT

In100 candidate4063 (in intron):

LOCUS ENSG00000117069

DESCRIPTION Alpha-N-acetylgalactosaminide alpha-2,6-sialyltransferase 5

(EC2.4.99.-) (GalNAc alpha-2,6-sialyltransferase V) (ST6GalNAc V) (GD1 alpha snthase) (Sialyltransferase 7E). [Source:Uniprot/SWISSPROT;Acc:Q9BVH7]

TTTTTATTATTTACTAACCCATTCATGTAGGTTTTCTTTTTTCTTCTTTAAGACTCCCCAATCCTCTTTTCTTTCCAGTC
CTTTCACTGATTTTGTTTTG

In100 candidate4064 (in intron):

LOCUS ENSG00000142892

DESCRIPTION GPI-anchor transamidase precursor (EC 3.-.-.-) (GPI transamidas) (Phosphatidylinositol-glycan biosynthesis class K protein) (PIG-K) (hGPI8. [Source:Uniprot/SWISSPROT;Acc:Q92643]

CTTTTCTGAGTCCATCTAACACTGTTTTTTTTCTTATTCTGTATTTCAGTCTTTTGTGAGATTAATCTTCCTAATTTAAA
ACTTTTATTCTTTATTTCCT

In100 candidate4065 (in intron):

LOCUS ENSG00000154027

DESCRIPTION Adenylate kinase isoenzyme 5 (EC 2.7.4.3) (ATP-AMP transphosphoylase). [Source:Uniprot/SWISSPROT;Acc:Q9Y6K8]

AGACTTCCTTAACTTCACCTTCCATCTGTTCTATTGAGTTTATTTCAGCTATCATAGTTTTAATTTTCAAGAGCTCTTTC
TTTCTTTAACTGTTCCTTTT

In100 candidate4066 (in intron):

LOCUS ENSG00000154027

DESCRIPTION Adenylate kinase isoenzyme 5 (EC 2.7.4.3) (ATP-AMP

transphosphoylase). [Source:Uniprot/SWISSPROT;Acc:Q9Y6K8]

AGCTTTTAAGAGTTAACTCTCATGCTTGCTTTTCTGTCCTGAGCCTCTTTCATTTCTGCTTTTGGCAGTTTTCTTTTTTC

TATACCACATTATATATGAT

In100 candidate4067 (in intron):

LOCUS ENSG00000154027

DESCRIPTION Adenylate kinase isoenzyme 5 (EC 2.7.4.3) (ATP-AMP

transphosphoylase). [Source:Uniprot/SWISSPROT;Acc:Q9Y6K8]

CTTCCATAGATGGAATCACACTATATAACTGTATATATTATTTTCTAGCTGTCTTCTTTCAGCACATCATTTTAATGACT

GCATCCTATTCCTTTATTTT

In100 candidate4068 (in intron):

LOCUS ENSG00000154027

DESCRIPTION Adenylate kinase isoenzyme 5 (EC 2.7.4.3) (ATP-AMP

transphosphoylase). [Source:Uniprot/SWISSPROT;Acc:Q9Y6K8]

TTCCCTGACCACCCTATAGAAAGTAACACCTCTAGCATTCTGTACCTCTTTGTCCTGCTTTAATTTTGTACCTATCAGTT

ATCTTCGCTGACTCTTCATT

In100 candidate4069 (in intron):

LOCUS ENSG00000036549

DESCRIPTION zinc finger, ZZ domain containing 3

[Source:RefSeq_peptide;Acc:P_056349]

GGTGTAATATTTGATATTCTGTCTAGAATGTCTTAACCTCTTTTTGCCTTTCCTTTTGAAATCCCATCTATTTTTCAGGA

TTCAACTTGCTTTTTACTTC

In100 candidate4070 (in intron):

LOCUS ENSG00000137960

DESCRIPTION PDZ domain-containing protein GIPC2.

[Source:Uniprot/SWISSPROT;cc:Q8TF65]

ATCAAAGCCATTCTATTAGCATAACTCAAGAAATCTATTTTTTTCTGTATTGATCTCCACTACATAATCTGTTTCCAGAG

TAAATTTTTCTGTTTTTTTT

In100 candidate4071 (in intron):

LOCUS ENSG00000137959

DESCRIPTION histocompatibility 28 [Source:RefSeq_peptide;Acc:NP_006811]

TAACTAATTTTACTGTATCCTTTAATCCCTTTTGCCCTTTTCTCTTTTTATTCTTTTTTTGTCTCAATACTTCAGTTGGT

AAATTTTCTTCTAATTCTTT

In100 candidate4072 (in intron):

LOCUS ENSG00000117114

DESCRIPTION Latrophilin-2 precursor (Calcium-independent alpha-latrotoxin

rceptor 2) (Latrophilin homolog 1) (Lectomedin-1).

[Source:Uniprot/SWISSPROTAcc:O95490]

AGGTAAAGGAACTAAACATATGGATTTTCTACCCATTGAGGTACTCTAACCACATGCTAGAGGCATTTTGTTTCTCAGCT

CTTAGTCATTTCATTTTTTC

In100 candidate4073 (in intron):

LOCUS ENSG00000117114

DESCRIPTION Latrophilin-2 precursor (Calcium-independent alpha-latrotoxin rceptor 2) (Latrophilin homolog 1) (Lectomedin-1). [Source:Uniprot/SWISSPROTAcc:O95490]

GTGTGTGGTAAGGTAACAATTGAATTGGTTTTATTCGCTAGCCTCCATAATGCTTTCTCTTGCAGTGTTTTTTGTATTAT
GCACCAATACATCCTTCTTC

In100 candidate4074 (in intron):

LOCUS ENSG00000117114

DESCRIPTION Latrophilin-2 precursor (Calcium-independent alpha-latrotoxin rceptor 2) (Latrophilin homolog 1) (Lectomedin-1). [Source:Uniprot/SWISSPROTAcc:O95490]

AGTTTAACCTGTGATATCCTGTGTTTTTTCTGCAGTCATTCTACCTTTTTCTCATTCTGGCTTCTCCTTCATTGACTATA
GATATCCTCTCAGATTCTGT

In100 candidate4075 (in intron):

LOCUS ENSG00000117114

DESCRIPTION Latrophilin-2 precursor (Calcium-independent alpha-latrotoxin rceptor 2) (Latrophilin homolog 1) (Lectomedin-1). [Source:Uniprot/SWISSPROTAcc:O95490]

AGCTTTTTGTTTCATTTAACTTTGGTATTTTTTGTTTGTTTGTTTCAGTTTCATTTAATTCTGCTCTGATCTCGGTTATT
TCCTTTCTTATGCTGGCTTT

In100 candidate4076 (in intron):

LOCUS ENSG00000117114

DESCRIPTION Latrophilin-2 precursor (Calcium-independent alpha-latrotoxin rceptor 2) (Latrophilin homolog 1) (Lectomedin-1). [Source:Uniprot/SWISSPROTAcc:O95490]

TTACTAAATCACCAAATAGAGAAACCTAGATAACTGTGATGACCCTGCCTAATATTTAGCTCTCTCTATTCTCTGCAGAA
CTTTTTTTCTTCATGCCCTA

In100 candidate4077 (in intron):

LOCUS ENSG00000142875

DESCRIPTION cAMP-dependent protein kinase, beta-catalytic subunit (EC 2.7.1.11) (PKA C-beta). [Source:Uniprot/SWISSPROT;Acc:P22694]

AGTTGTAAAATATTTTTCTTTTCTGATTTTGTTAACTTGGATCTTCTCTCTTTTTTTCTTGGTTAGTCTAGCTAGCAGTT
TATCAATTTTGGTTATTTTT

In100 candidate4078 (in intron):

LOCUS ENSG00000117133

DESCRIPTION Ribosome production factor 1 (Ribosome biogenesis protein RPF1)(Brix domain-containing protein 5). [Source:Uniprot/SWISSPROT;Acc:Q9H9Y2]

ATGTGTTTCCCCCTAGTAGATAACTATCTTGATCTCTATTTCTTCTCCTTGTTACTTCTGCACAGTTTTAAGCCTTTTCA
TTCAGTGTTGGCCATTCTTC

In100 candidate4079 (in intron):

LOCUS ENSG00000153898

DESCRIPTION Mucolipin-2. [Source:Uniprot/SWISSPROT;Acc:Q8IZK6]

AGCATCTCTGGCTTTTATTTAACCCACTGTATGGGTGGCTTTCTATTCTATTTCAGATCTTATTTTTATGCCTTCAAATA

CTATGCTTTTTTTCTTTGAT

In100 candidate4080 (in exon):

LOCUS ENSG00000142867

DESCRIPTION B-cell lymphoma/leukemia 10 (B-cell CLL/lymphoma 10) (Bcl-10) (ED- 3/ICH-1 prodomain homologous E10-like regulator) (CIPER) (CARD- containg molecule enhancing NF-kappa-B) (Cellular homolog of vCARMEN) (cCARMEN) (ammalian CARD-containing adapter molecu [Source:Uniprot/SWISSPROT;Acc:O9599]

TCTAGGATAGCTAACTGTAAATCATGTTGATCATGTACTTTTTAGTAATTTCTTTTTTTCCTTTTTAAGGTCTTTCAGTA

CTTTTTTAAATATTTTCTAT

In100 candidate4081 (in exon):

LOCUS ENSG00000153904

DESCRIPTION NG,NG-dimethylarginine dimethylaminohydrolase 1 (EC 3.5.3.18) (imethylargininase-1) (Dimethylarginine dimethylaminohydrolase 1) (DDAHI) (DAH- 1). [Source:Uniprot/SWISSPROT;Acc:O94760]

GGAGAAACCTCTGAGAAAATGAAAACATCCTTAACCACTATCTTTCCCTTTTATTTGATTATTTTATGTCAGAAATTTGC

AAAAGTTTTTTTCTCCTCCT

In100 candidate4082 (in intron):

LOCUS ENSG00000117174

DESCRIPTION CDNA FLJ20760 fis, clone HEP01082 (Chromosome 1 open reading frme 181) (Novel protein). [Source:Uniprot/SPTREMBL;Acc:Q9NWK9]

AAGAGGTGTTTAAGTTCCACATGTCTGGTAGTAGTTAACTGTATCGTGTATTACTTTTTTTGTTTATTTGTTTAACAGCT

TCTTTTGACCAAATTCATTT

In100 candidate4083 (in intron):

LOCUS ENSG00000117174

DESCRIPTION CDNA FLJ20760 fis, clone HEP01082 (Chromosome 1 open reading frme 181) (Novel protein). [Source:Uniprot/SPTREMBL;Acc:Q9NWK9]

TGGGCAACAAAGCGAGACTCCATATCAAAAAAAGAAAAAGAAAATTAGTAACTTGTTTTAGAGCTTTCCTAATGTCAGAA

TATTTGATTTTTTTTTATTT

In100 candidate4084 (in intron):

LOCUS ENSG00000171502

DESCRIPTION collagen, type XXIV, alpha 1 [Source:RefSeq_peptide;Acc:NP_69080]

TACATTTGCCTTTTGAAATTTTTTTGGCATTAACTTTTGGATAGGTTATTCTCACTCAGTAAAGGCTATTTTAATGTTTT

TCTTTTTTATTATTACTTTC

In100 candidate4085 (in intron):

LOCUS ENSG00000171502

DESCRIPTION collagen, type XXIV, alpha 1 [Source:RefSeq_peptide;Acc:NP_69080]

AACAAGCATATCAAACTAACATGTCCAAAACTTAGCTTCTTATTTACCCTCCAAACCTGTTTTCAGTCTTCTTCATTTCA

TTTGGTCTGCTACTCTTACA

In100 candidate4086 (in intron):

LOCUS ENSG00000171502

DESCRIPTION collagen, type XXIV, alpha 1 [Source:RefSeq_peptide;Acc:NP_69080]

CCATTTCATCATGTCAACATGTAACCATACCTGAGATGCTGCCTTCTCTGTAAAGTCTTTTCTCATGTTTTCAACCAGAT

ATAATCTCTTCTGCCTTTGT

In100 candidate4087 (in intron):

LOCUS ENSG00000183291

DESCRIPTION 15 kDa selenoprotein precursor.

[Source:Uniprot/SWISSPROT;Acc:O0613]

GTCTCTTTTTTCATTTATATTTTCACGTGTTTGTGATTTTGAAAATAACATAATTCAAACATTTTTCTTTAACCTCAGAA

CATATACTTCTTTGTTATCC

In100 candidate4088 (in intron):

LOCUS ENSG00000153936

DESCRIPTION Heparan sulfate 2-O-sulfotransferase 1 (EC 2.8.2.-) (2-

Osulforansferase)

(2OST). [Source:Uniprot/SWISSPROT;Acc:Q7LGA3]

AATAAAATTCTCTTTTTTTTAACTGTGTACCTTCATGATCTCATTTACTCCTTTTACATCAGCTGATGCCTTTGTGTTGT

GACTCTTAATTTCTATCTTT

In100 candidate4089 (in intron):

LOCUS ENSG00000162645

DESCRIPTION Interferon-induced guanylate-binding protein 2 (GTP-binding

proein 2) (Guanine nucleotide-binding protein 2) (GBP-2) (HuGBP-2).

[Source:Unprot/SWISSPROT;Acc:P32456]

TTCTTTTTTAGTCTAACTAGAAGTATATCCATCTTATGTATTTTTCCAGAGAACCATTGGTTTCATTGATCTTTTGTATG

GTTTTTCTTGTCTCCACTTT

In100 candidate4090 (in intron):

LOCUS ENSG00000171488

DESCRIPTION Leucine-rich repeat-containing protein 8C (Protein AD158).

[Souce:Uniprot/SWISSPROT;Acc:Q8TDW0]

CAAAGGTCTAATCTGATTCTTAGCATCTTTAACTTTTGAATCACATCCTTTTTTGTTGTCTTGAGTCAGAAACATGTACT

CATTCTATAGTTTTTCCTTT

In100 candidate4091 (in intron):

LOCUS ENSG00000171492

DESCRIPTION Leucine-rich repeat-containing protein 8D.

[Source:Uniprot/SWISPROT;Acc:Q7L1W4]

TAATCAAATTATTAACTTCCCTGGTAATCTAACCTTTTTTTTTGGTTTGGCCCACCTTTCAGCCTGTTAAGGTGTTTTCA

TTGCTAACTCTTTCTATCCT

In100 candidate4092 (in intron):

LOCUS ENSG00000162669

DESCRIPTION CDNA FLJ36760 fis, clone UTERU2018867.

[Source:Uniprot/SPTREMBLAcc:Q8N9Q0]

TACTGCCATCTCTTTTTGTACTGCTTCTTTAAGGTCAATAACTCTTAGATTTGCCCTTTTGAGGCTATTTTCCAGATCTT

ATAGGTGTACTTCATTTTTT

In100 candidate4093 (in intron):

LOCUS ENSG00000097046

DESCRIPTION Cell division cycle 7-related protein kinase (EC 2.7.11.1)

(CDCrelated

kinase) (HsCdc7) (huCdc7). [Source:Uniprot/SWISSPROT;Acc:O00311]

EXON

TTGACTTCCATGCTAATAACTCTTCCTTCAAAATTCCTTTCTTATTTGGCTCTCTAACTTGGCTTCTTCCAAGCTCAGTT

TTCTTTTCTCGCTATTAATT

In100 candidate4094 (in intron):

LOCUS ENSG00000069702

DESCRIPTION TGF-beta receptor type III precursor (TGFR-3) (Transforming groth

factor beta receptor III) (Betaglycan). [Source:Uniprot/SWISSPROT;Acc:Q0167]

AGATAATAAACATTAATTAACATGTGTTTCGGTTTTTCTTTTTGCCTCTGTTTAGTGTCAGCTAAATTTTTTTGGTTTGT

TCACTCATGTATTCGTTCTT

In100 candidate4095 (in intron):

LOCUS ENSG00000137948

DESCRIPTION Bromodomain testis-specific protein (RING3-like protein).

[Soure:Uniprot/SWISSPROT;Acc:Q58F21]

AGAGAGTTGGACCCTTGACATACTTCCTAGGCTTTTATGACACCTCTCTTAACAATCCCATCTGTCTGTTCCTTACAGAT

ATCTCTTTATTTGCCTCCCT

In100 candidate4096 (in intron):

LOCUS ENSG00000203910

DESCRIPTION

CATTCCACACTTGTGTTTAACCTGCTCTTTAACCTATCCATTAAATTGTTTCCAATTTTGATATTTTTTCATTTCCAGAA

TTTCCATTTTATTTACTTTT

In100 candidate4097 (in intron):

LOCUS ENSG00000067208

DESCRIPTION Ecotropic viral integration site 5 protein homolog (EVI-5)

(Neuoblastoma stage 4S gene). [Source:Uniprot/SWISSPROT;Acc:O60447]

ACCCACCTTTGGAAAAAACATCTTTGGAGTCACTTGGTAGCTTTTGTATAACAACTTTAATGATTTTTGTTTTACCAGAA

TTTTTTTTCATTGCAAGTTT

In100 candidate4098 (in intron):

LOCUS ENSG00000067208

DESCRIPTION Ecotropic viral integration site 5 protein homolog (EVI-5)

(Neuoblastoma stage 4S gene). [Source:Uniprot/SWISSPROT;Acc:O60447]

AACCTTCGTGATAAGTTCTAATTCTTTAACATGGCATAAGATTCTTCATAGTTTAGCCCCAGCTTTCTTTCAAGTTTCAT

CTCTCACCTCTCATTTTGAG

In100 candidate4099 (in intron):

LOCUS ENSG00000154511

DESCRIPTION Novel protein possible ortholog of mouse RIKEN cDNA 2900024C23

ene. [Source:Uniprot/SPTREMBL;Acc:Q5T7M9]

TATTTTGATTATAACAATTTTATTTTTTAATGTTTTCTTATGCTCTGTAATTGTCTTCATTCCTTTCAGAGATTTTATTT

TCTATATGTTTATCTTAGCT

In100 candidate4100 (in intron):

LOCUS ENSG00000154511

DESCRIPTION Novel protein possible ortholog of mouse RIKEN cDNA 2900024C23

ene. [Source:Uniprot/SPTREMBL;Acc:Q5T7M9]

ATTATCCATAATCCCACCACCTCTTGACATTTTGAGATAACAACCCTTAATGTTTTGTTTATTTCCTTGCAGTTTCTAAA

GTTTTTTGGTGATTTCTCTT

In100 candidate4101 (in intron):

LOCUS ENSG00000137962

DESCRIPTION PTPL1-associated RhoGAP 1

[Source:RefSeq_peptide;Acc:NP_004806]EXON

ATCAATTACTGAGAGAAGAGTGCTGAAATCTTAACTGTCATTGGGGATTTGTCTATTTTTTCCATACATTTCTCTCAGTT

TTTTTCTCATACTCTGGACC

In100 candidate4102 (in intron):

LOCUS ENSG00000117528

DESCRIPTION ATP-binding cassette sub-family D member 3 (70 kDa peroxisomal

embrane protein) (PMP70). [Source:Uniprot/SWISSPROT;Acc:P28288]

AATTTCTTTCATCTTTGTATCCTGAATTAAATAACCTGTTTCTGGATTTTTTGTTTTTCTCTTCCTCAGTTTACTTTCTC

GTTTTGGCAGATGCACTCTT

In100 candidate4103 (in intron):

LOCUS ENSG00000172339

DESCRIPTION UDP-N-acetylglucosamine transferase subunit ALG14 homolog.

[Souce:Uniprot/SWISSPROT;Acc:Q96F25]

CAAGGGACCTTGTGGTTTTCTTCTTCTTTGTCTAACCATTCTTAAAGCATTTTCCTGACTTTGCTTCCTGTTCAGCTTTA

CAGCCAAGCTTCTTCTCTTT

In100 candidate4104 (in intron):

LOCUS ENSG00000188641

DESCRIPTION Dihydropyrimidine dehydrogenase [NADP+] precursor (EC 1.3.1.2)

DPD) (DHPDHase) (Dihydrouracil dehydrogenase) (Dihydrothymine dehydrogenase.

[Source:Uniprot/SWISSPROT;Acc:Q12882]

AGCATTCCTTATTACTCCTAACCACATTTTATTTATTACTCCTGACTACATTTTATTTTCTCTACAGAATTTATTATCAT

CTCTATCTTTACTGTTTTGC

In100 candidate4105 (in intron):

LOCUS ENSG00000188641

DESCRIPTION Dihydropyrimidine dehydrogenase [NADP+] precursor (EC 1.3.1.2)

DPD) (DHPDHase) (Dihydrouracil dehydrogenase) (Dihydrothymine dehydrogenase.

[Source:Uniprot/SWISSPROT;Acc:Q12882]

ATTATTGATATTGAAGAGCACTTTATATAACAAATTATATAATTTTTACCTTTATGTGTTGCAAATATCTTCCCTCAGTT

TGTGGCATATCTTTTCACTC

In100 candidate4106 (in intron):

LOCUS ENSG00000188641

DESCRIPTION Dihydropyrimidine dehydrogenase [NADP+] precursor (EC 1.3.1.2)

DPD) (DHPDHase) (Dihydrouracil dehydrogenase) (Dihydrothymine dehydrogenase.

[Source:Uniprot/SWISSPROT;Acc:Q12882]

TTAAGCTTCATAGGTATGTTGAAGACATAATACCATTTGCTTAAGTTTGTAACACTTTTATTTTTCATTTATTTTCAGTT

TGTATGTTACATATTCCTTC

In100 candidate4107 (in intron):

LOCUS ENSG00000188641

DESCRIPTION Dihydropyrimidine dehydrogenase [NADP+] precursor (EC 1.3.1.2)

DPD) (DHPDHase) (Dihydrouracil dehydrogenase) (Dihydrothymine dehydrogenase.

[Source:Uniprot/SWISSPROT;Acc:Q12882]

AGTCAAAAGCCTTTAATTATAAGCAAACTAGCTAACCTAAAGATATTTTTCACATTTCATATTTTTCTTTAATTTCAGTT

TTATTTAATATCACCTTCTA

In100 candidate4108 (in intron):

LOCUS ENSG00000188641

DESCRIPTION Dihydropyrimidine dehydrogenase [NADP+] precursor (EC 1.3.1.2)

DPD) (DHPDHase) (Dihydrouracil dehydrogenase) (Dihydrothymine dehydrogenase.

[Source:Uniprot/SWISSPROT;Acc:Q12882]

CTACATTCCCATAAGTTGCCAATAACTGTGAGTGGTATATTCTATTACTTACATTTCAGTCTTCATTCTACCTAATTTCT

CTGCAGTATTTTACCTCAAT

In100 candidate4109 (in intron):

LOCUS ENSG00000162627

DESCRIPTION Sorting nexin-7. [Source:Uniprot/SWISSPROT;Acc:Q9UNH6]

GTGTAGAGCCATCTTGTTTGACCTCCATGTAACTGATTTGTGTCTAGTTTCTTTCCTTCTTCTTTAAAACACCAGAAGGC

CCTTCTCTTTTATGTCCTGT

In100 candidate4110 (in intron):

LOCUS ENSG00000162627

DESCRIPTION Sorting nexin-7. [Source:Uniprot/SWISSPROT;Acc:Q9UNH6]

AGCATCCTAACATTCCCTGTCTACCGTTCTGCTTCATTTTTCTCTTTAGCATTTATTACCAGCTGACAGTTCCCATTTTT

TATTTACCTTGCTTGTAGTT

In100 candidate4111 (in intron):

LOCUS ENSG00000117620

DESCRIPTION UDP-N-acetylglucosamine transporter (Golgi UDP-GlcNAc transportr)

(Solute carrier family 35 member A3). [Source:Uniprot/SWISSPROT;Acc:Q9Y22]

GTTTCAAGGGAATGACTTGTAAACTTATATTTCTGAGGCTAACTTTCCTCCCAACTGTTGATTCTATCTCTTCAGCATCT

CAAATTTAGTTCTCCCCTTT

In100 candidate4112 (in intron):

LOCUS ENSG00000156876

DESCRIPTION Spindle assembly abnormal protein 6 homolog (HsSAS-6).

[Source:niprot/SWISSPROT;Acc:Q6UVJ0]

GAGGGCCTGATACAATTAGCCTTTTAACACCATTTGGATCACTCACCTAGTTGAAGCATTTATCTTTTTCTTTTCCAGTT

TTCTCCATGATTATTGGTCT

In100 candidate4113 (in intron):

LOCUS ENSG00000137992

DESCRIPTION Lipoamide acyltransferase component of branched-chain alpha-ket acid dehydrogenase complex, mitochondrial precursor (EC 2.3.1.168) (Dihydrlipoyllysine-residue (2-methylpropanoyl)transferase) (E2) (Dihydrolipoamidebranched chain transacylase) (BCKAD E2 [Source:Uniprot/SWISSPROT;Acc:P11182

AGCAACACTTAACACATATTGGTCTCTCTTCTTTCTAGAGCTTTCCATTCTTGTTTTCTGTGACACCAGTTTCCCAATAT
TATTCTTTCTTCATCTTTGG

In100 candidate4114 (in intron):

LOCUS ENSG00000137992

DESCRIPTION Lipoamide acyltransferase component of branched-chain alpha-ket acid dehydrogenase complex, mitochondrial precursor (EC 2.3.1.168) (Dihydrlipoyllysine-residue (2-methylpropanoyl)transferase) (E2) (Dihydrolipoamidebranched chain transacylase) (BCKAD E2 [Source:Uniprot/SWISSPROT;Acc:P11182

TTTTTACAAGATATAGAATTATTTATATTTTCTTTAACTTGCTTTTTTCTTTTTCTTTTTTTTTTTTTTTGATACAGAG
TCTTGCTCTGTTGCCTATCT

In100 candidate4115 (in intron):

LOCUS ENSG00000079335

DESCRIPTION Dual specificity protein phosphatase CDC14A (EC 3.1.3.48) (EC 31.3.16) (CDC14 cell division cycle 14 homolog A). [Source:Uniprot/SWISSPROTAcc:Q9UNH5]

CTTTACTTCTCTGAGTAATTTCGCACTGTGTTAACTAGTAACTTCGTCTTAGGATTCTTCTCTTGTGTGATTTCCCAGTT

CTTTTAGGCACATTTCATTT

In100 candidate4116 (in intron):

LOCUS ENSG00000162695

DESCRIPTION zinc transporter like 2 [Source:RefSeq_peptide;Acc:NP_598003]

TTACTCTCTGTCCTTAACTGCTCCCCCTTTCTCCCTGGCTTGGCTTCTTTCTTTAGCAGGCACACACTCTTTCTTTCACA

TGCTCTTGTTCTGTTTAGAT

In100 candidate4117 (in intron):

LOCUS ENSG00000118733

DESCRIPTION Noelin-3 precursor (Olfactomedin-3) (Optimedin).

[Source:Unipro/SWISSPROT;Acc:Q96PB7]

ATGTTTTAACTGAAAATTGTGTTGTGTCCCTTTCCATTTCTCTTGATTTTGCCTTCCAAATATCTTTTGTCTTTCCAGCT

TTTTCCCATTTTTGCCATCC

In100 candidate4118 (in intron):

LOCUS ENSG00000118733

DESCRIPTION Noelin-3 precursor (Olfactomedin-3) (Optimedin).

[Source:Unipro/SWISSPROT;Acc:Q96PB7]

AACTCTTTCATATATTTTCTTTACTTTATTTAGCTTTCCAATCCACTAACTCTACCATTTTTTTTCTACTTTATCAGCTC

CTTCCTAGATTCACTTCTTT

In100 candidate4119 (in intron):

LOCUS ENSG00000162699

DESCRIPTION HEJ1. [Source:Uniprot/SPTREMBL;Acc:Q96RF1]

AGTAAGATTTGAATTTTTTCAAGTAGATTGTATGGTAACATTTACATTCATTCATTTATTCATTCTAATCAACCTCAGTT

CCTGAATCTTTGATGTCTCT

In100 candidate4120 (in intron):

LOCUS ENSG00000162631

DESCRIPTION Netrin-G1 precursor (Laminet-1).

[Source:Uniprot/SWISSPROT;Acc:9Y2I2]

GATACTTTGAACTGAATTCATGCTCTTTGTCCTCATAACTCATTATTCTGGTATTCTCTGTACTTCTCTGGCCAGAAACC

CCCTTTCTTAATCTTTTAGT

In100 candidate4121 (in intron):

LOCUS ENSG00000162631

DESCRIPTION Netrin-G1 precursor (Laminet-1).

[Source:Uniprot/SWISSPROT;Acc:9Y2I2]

GCTTTTTGTTGTTGTTTTTAAATATCTCTTCTAAGGAGAAAAGGAACTAACATTTAAGAAGTTCTTACTGTTTGCCAGAT

GTTTCATATATATCTCATTT

In100 candidate4122 (in intron):

LOCUS ENSG00000134215

DESCRIPTION Protein vav-3. [Source:Uniprot/SWISSPROT;Acc:Q9UKW4]

CTTCAATTTTAAAGAACACCGGCATAACTCATTTTTCTTTTTTTCCCCTGTGTTCTTTTTGACCTTCTTAGCAGCTCCTG

ACTTTCTCCCTTGGGGATTT

In100 candidate4123 (in intron):

LOCUS ENSG00000134215

DESCRIPTION Protein vav-3. [Source:Uniprot/SWISSPROT;Acc:Q9UKW4]

AGATGATTTATAACTTCTTGGCACAATTTGTTATTATTTTAACATTCTTATTTTTCTTTATAGCCTACAGTTTACATTTT

TATTGTTGTATATTTTTATT

In100 candidate4124 (in intron):

LOCUS ENSG00000134215

DESCRIPTION Protein vav-3. [Source:Uniprot/SWISSPROT;Acc:Q9UKW4]

AGAATGTACTTTATTTCTTATTTTTAACATTAATATACCTGCTTTGTCTTTGTTCATTTTCTATTTGACCATTAGCAGCT

ACAAGTTTTCTTTCTTTGTT

In100 candidate4125 (in intron):

LOCUS ENSG00000134215

DESCRIPTION Protein vav-3. [Source:Uniprot/SWISSPROT;Acc:Q9UKW4]

AGGTTCCTAACCATCCACTTAACTTAGTTTCTCCCTTGCCCTTTTAATTCAGCATTCCCATATTCACCTGCCTTTATTTT

CTGATTTTATTGTATCCTCA

In100 candidate4126 (in intron):

LOCUS ENSG00000085491

DESCRIPTION solute carrier family 25 member 24 isoform 2

[Source:RefSeq_pepide;Acc:NP_998816]

AAATAACCTGTCTTTGAGTTCATAGTTTCTTTCTTCTTCTTGATCACTTCTGCAGTTGATGCTCCCATATTGCATTTTAA

TTTTGTTCATTGTATTTTTC

In100 candidate4127 (in intron):

LOCUS ENSG00000085491

DESCRIPTION solute carrier family 25 member 24 isoform 2

[Source:RefSeq_pepide;Acc:NP_998816]

CTCTGAAGTGCCCTGGAGGCATTTTCCCCATTGTCTTAGTGATTAACATTTCACTCCTTGTTTCTTATGCAGATTTCTGC

AGCTGGCTTGAATTTTTTCC

In100 candidate4128 (in intron):

LOCUS ENSG00000116266

DESCRIPTION Syntaxin-binding protein 3 (Unc-18 homolog 3) (Unc-18C) (Unc-183)

(Platelet Sec1 protein) (PSP). [Source:Uniprot/SWISSPROT;Acc:O00186]

GCATTGCAACTGAATGTTCTCTATTAACCTGACTTTGAGTCCCCTACTTCTGTCTTCTGTTGTATTCAGTCTGCTATTAA

ACCCATTCATTGTATCCTTT

In100 candidate4129 (in intron):

LOCUS ENSG00000065135

DESCRIPTION Guanine nucleotide-binding protein G(k) subunit alpha (G(i) alpa-

3). [Source:Uniprot/SWISSPROT;Acc:P08754]

GCCTTTAATATCATTTCCATTTTCTTTGTTGTTGTTAACTTCCAAGCCTATCCTCCATTGTCTTCATTCTGTGTACAGAT

TCTTACGGTCCTTATTTGCC

In100 candidate4130 (in intron):

LOCUS ENSG00000143119

DESCRIPTION Leukocyte surface antigen CD53 (Cell surface glycoprotein

CD53)(Tetraspanin-25) (Tspan-25). [Source:Uniprot/SWISSPROT;Acc:P19397]

TACTGATTCCATCAACTATTGAGAGAAGGATATTGAACTATTCAAGTATAACTTATACTTGTCTATATCTTCTTTCAGTT

CTGTTCATTTCTGTCTCATA

In100 candidate4131 (in intron):

LOCUS ENSG00000121933

DESCRIPTION Adenosine A3 receptor. [Source:Uniprot/SWISSPROT;Acc:P33765]

GCCATTAATAACTACATTTAAAGTCTCGGTAGGTCACTTTTTCCTTACATGGTGTTTCTGCTGTCCCTTACTCAGGGAAT

TTTGTTTCCTTGTGTATTTT

In100 candidate4132 (in intron):

LOCUS ENSG00000116473

DESCRIPTION Ras-related protein Rap-1A precursor (GTP-binding protein smgp1A)

(Ras-related protein Krev-1) (C21KG) (G-22K).

[Source:Uniprot/SWISSPROTAcc:P62834]

AAGGGAGCATTTCTCTTATGTGCAAACTAACCATAGTGGACATGTTTGTCTACATCCAGACCAGTTCCTAGATTCAAAAC

TTGTCTTTGTTTTTGTTTTT

In100 candidate4133 (in intron):

LOCUS ENSG00000116473

DESCRIPTION Ras-related protein Rap-1A precursor (GTP-binding protein smgp1A)

(Ras-related protein Krev-1) (C21KG) (G-22K).

[Source:Uniprot/SWISSPROTAcc:P62834]

CTGGCATGGAAGGAGACCCTTGACCTGAAGGGTGATCATCTTTAACCCTGAATTCTGTTAACTGTTTCTCAGAAACTCTC

TCTGAATCTATTTAACTTTT

In100 candidate4134 (in intron):

LOCUS ENSG00000116473

DESCRIPTION Ras-related protein Rap-1A precursor (GTP-binding protein smgp1A)

(Ras-related protein Krev-1) (C21KG) (G-22K).

[Source:Uniprot/SWISSPROTAcc:P62834]

AAAAAAGGTTTGCTTCCTTGCCCTCCTAACTGATCTTCCTTCTCTAGTTTTTCATTCCTCTGTTGCAATCTACAGAGTAC

TCATAGAGCTTTCTTTTTTC

In100 candidate4135 (in intron):

LOCUS ENSG00000143079

DESCRIPTION CTTNBP2 N-terminal-like protein.

[Source:Uniprot/SWISSPROT;Acc:9P2B4]

TACTGTAAGTAAATTCTTAAATAACTAAGTAAATTCTTTTTAATGTCTCTTTTGTCAGTCTCTTCCATTCTTATTGCTAC

CATCCTAGTTCATACATTTT

In100 candidate4136 (in intron):

LOCUS ENSG00000116489

DESCRIPTION F-actin capping protein subunit alpha-1 (CapZ alpha-1).

[SourceUniprot/SWISSPROT;Acc:P52907]

AGTTGCAAATCCTAACATTTCTTGTCCCTTTTCTTTGCCTTGTTTTTTCTCCTTAGCATTTATCACTTGCCAGGTAGTAT

TTTACTTAATCTTCTCTGTT

In100 candidate4137 (in intron):

LOCUS ENSG00000116793

DESCRIPTION Putative homeodomain transcription factor 1.

[Source:Uniprot/SWSSPROT;Acc:Q9UMS5]

TTTTTAACTTTCTAACTGCTTTCTCTTGCTTCCTATCTCTCTTTACCCTCCATCTGTTCTTTATAGCAGAGCCAGGTTGT

TGTTTTTTGGTTTCTGTTTT

In100 candidate4138 (in intron):

LOCUS ENSG00000198765

DESCRIPTION Synaptonemal complex protein 1 (SCP-1).

[Source:Uniprot/SWISSPRT;Acc:Q15431]

ACCCGAAACCAAAATAAAAACCTTCATTAATTTTCTAACTCTTCTTTGACTCTTAATCTCTTTTTTCTATTTCAACAGTT

TTGCCTTTCTATACCTTATC

In100 candidate4139 (in intron):

LOCUS ENSG00000198765

DESCRIPTION Synaptonemal complex protein 1 (SCP-1).

[Source:Uniprot/SWISSPRT;Acc:Q15431]

TTTTAGTTACTAAGATGTTTATTTTAAGATGTTATTCTTCTTTTTCTAACCTGCCATTCTACTTTATCTAGTTTTCAGTT

TCTTCTTATTTTCGAGGTTG

In100 candidate4140 (in intron):

LOCUS ENSG00000198765

DESCRIPTION Synaptonemal complex protein 1 (SCP-1).

[Source:Uniprot/SWISSPRT;Acc:Q15431]

AAAATATAACTAACTTAGAAGTTCCTCTTAACTATCATTTGAATCTTTTCCTTTTCTTGATTTCCACAGATCTAGAACAG

TTCAATTCCTCATTTTCTCT

In100 candidate4141 (in intron):

LOCUS ENSG00000116815

DESCRIPTION Lymphocyte function-associated antigen 3 precursor (Ag3) (Surfae

glycoprotein LFA-3) (CD58 antigen). [Source:Uniprot/SWISSPROT;Acc:P19256]EXON

TTTATTGCCAACTATCTGCATAACTAGATTTTAAGTTCCTTGTATGCAGAAGTCATGTATTATTTTTTAAAATTTTCCTT

ATATTGATCTGTTTTTTTTT

In100 candidate4142 (in intron):

LOCUS ENSG00000134258

DESCRIPTION V-set domain containing T cell activation inhibitor 1

[Source:RfSeq_peptide;Acc:NP_078902]

AAATACAAAAAAGCACAAAGGAAAAAAATATCTATCGGTCCTAACCTAGAGATAACCACTATTTATATGTCCTTCCAGTC

TTTTTTTTCCGTCTCTCTCT

In100 candidate4143 (in intron):

LOCUS ENSG00000198162

DESCRIPTION Mannosyl-oligosaccharide 1,2-alpha-mannosidase IB (EC 3.2.1.113

(Processing alpha-1,2-mannosidase IB) (Alpha-1,2-mannosidase IB) (Mannosidse

alpha class 1A member 2). [Source:Uniprot/SWISSPROT;Acc:O60476]

TGTGCCATTAAAAGTGAGCTCACTTCATGTTCTTGTTGAATTCTAACTGAAGCTCATTTCTCCCCTTTTTATTTTCAGAT

ATTTGTATCTGCTGTTCTCC

In100 candidate4144 (in intron):

LOCUS ENSG00000196505

DESCRIPTION ganglioside induced differentiation associated protein 2

[Sourc:RefSeq_peptide;Acc:NP_060156]

ATTTTCCAAATTGTGTTTATGTTTCTAACTATGGCAACTAGCTTAAATTATTTTTTCTCTTCTATTAATCAGGTGTCAAC

ATGGTTTTTTACCACCTTTT

In100 candidate4145 (in intron):

LOCUS ENSG00000155761

DESCRIPTION projection protein PF6 [Source:RefSeq_peptide;Acc:NP_996879]

AATCGTTTGTTGCTAATACACACCTAAAGAAAAGCCCTTAACTTGGTATGTCATTCTTCCATACGTTATCACATTCAGCT

TCATGTGCCTTTATACATTT

In100 candidate4146 (in intron):

LOCUS ENSG00000155761

DESCRIPTION projection protein PF6 [Source:RefSeq_peptide;Acc:NP_996879]

AGTCTTACTAATATTTTTAAGAAATCTGTTCTTGCTTTCTAACTCTTTCTCCTTTTATCTCATTTAATTTTGCCAGCATC

TTGTTTATTTCTCCTCTTTA

In100 candidate4147 (in intron):

LOCUS ENSG00000092607

DESCRIPTION T-box transcription factor TBX15 (T-box protein 15).

[Source:Unprot/SWISSPROT;Acc:Q96SF7]

TTAAAGGCTTCCTAGGGGGAGAAGATGGGCTGGATAACTGTGTTCAATATCATCTCTTTTTCGTTGCTTGGGTCTCAGTG

TACTTCTGTTCCACATTTTG

In100 candidate4148 (in intron):

LOCUS ENSG00000116874

DESCRIPTION Tryptophanyl-tRNA synthetase, mitochondrial precursor (EC 6.1.12)

(Tryptophan--tRNA ligase) (TrpRS) ((Mt)TrpRS).

[Source:Uniprot/SWISSPROTAcc:Q9UGM6]

CCCATCACCTGAGTTCTGTTAGGTTTCTACTCATTAACCAAAATTCATCTGTAGCTTTCTTTTTTTCTTGCCACACAGTC

TTCTGTTTTTTTTCTAGAGC

In100 candidate4149 (in intron):

LOCUS ENSG00000134250

DESCRIPTION Neurogenic locus notch homolog protein 2 precursor (Notch 2) (h2)

[Contains: Notch 2 extracellular truncation; Notch 2 intracellular domai].

[Source:Uniprot/SWISSPROT;Acc:Q04721]

CATTTTTTGTTTTGTGTTCTGTGAGAGCTCTCTTAAGGTTCTTCTTTATAACACTGACTTATATTTCTTCATTGTCAGGT

CAGTCCTTTATAGCTTCTTC

In100 candidate4150 (in intron):

LOCUS ENSG00000178104

DESCRIPTION phosphodiesterase 4D interacting protein isoform 3

[Source:RefSq_peptide;Acc:NP_071754]

AATCCAAGCCTCAAGGCATTGCTCTTTCTATCATATCCACCAATCTAACTGTAATGTATTGTTATTCTGTTTTATCAGAT

TATCTTCCCCTTAGTAGTAT

In100 candidate4151 (in intron):

LOCUS ENSG00000178104

DESCRIPTION phosphodiesterase 4D interacting protein isoform 3

[Source:RefSq_peptide;Acc:NP_071754]

CTGTGTGTTACCACAAAGCCTAACTGGCTTTACTTTGACATTCTTGCATTTTCTCATCTGCCTTAGTTTCTCTGCCAGAA

TCACATTTTTCTTTTGGTGC

In100 candidate4152 (in intron):

LOCUS ENSG00000178104

DESCRIPTION phosphodiesterase 4D interacting protein isoform 3

[Source:RefSq_peptide;Acc:NP_071754]

TTTCTCTGATCAAACAATTTAGGGTAATTTAACCACCCACTCTGTATTATGCTTTCCTGTTTTGCCGCACAGGGCTTGTC

CATAATATTTTTTGCATTTC

In100 candidate4153 (in intron):

LOCUS ENSG00000121848

DESCRIPTION Zinc finger protein 364 (Rabring 7) (RING finger protein 115).

Source:Uniprot/SWISSPROT;Acc:Q9Y4L5]

CTAACATACCTAATGTCTGCACTGTTTGTTCTCAGCTGGAGCAGTTCCCTCTCCAGGGATATTGCGATGTCTGCCACTTC

GTCTTTTTCTTTCTCTTTTT

In100 candidate4154 (in intron):

LOCUS ENSG00000178669

DESCRIPTION CDNA FLJ39739 fis, clone SMINT2016440.

[Source:Uniprot/SPTREMBLAcc:Q8N8A9]

CTATCATGAAGTCATTTTCTTCCTAATGAAAAATTTAACACTTTTAGATAGTCTTGGCATTCTTCTTTGGTTCTCCAGAT

TTTCACATCATTTATATTAT

In100 candidate4155 (in intron):

LOCUS ENSG00000163125

DESCRIPTION Uncharacterized protein KIAA0460.

[Source:Uniprot/SWISSPROT;AccQ5VT52]

AGTTTAGGTTATTCCCTAAGTCTTAGGCTAAAATAGCTCTTAACTGTTGTCCTGAGTTGAGTCCTCTATTTTCCAGATTC

CATGTCTTTTTTTCTTGGTT

In100 candidate4156 (in exon):

LOCUS ENSG00000163125

DESCRIPTION Uncharacterized protein KIAA0460.

[Source:Uniprot/SWISSPROT;AccQ5VT52]

TGGTGGTTTTCAACAAAGGTTAACTATTAAAGAAAGAAATATTTGTCTTTATCTTCCTTTTCCCCTTGCCTCAGTCGTGT

TATTCACCCCTATTCTTGAT

In100 candidate4157 (in intron):

LOCUS ENSG00000143374

DESCRIPTION Threonyl-tRNA synthetase, mitochondrial precursor (EC 6.1.1.3) Threonine--tRNA ligase) (ThrRS) (Threonyl-tRNA synthetase-like 1). [Source:niprot/SWISSPROT;Acc:Q9BW92]

ATTCAACTTTTAACTTCATGCATAGAGCTTTTTCTCCAGCTGTAATATCTGTTACCTGATATCCTATTTATATTTTCTCT
GAGTTTGTCTGTCTTTGCCT

In100 candidate4158 (in intron):

LOCUS ENSG00000143398

DESCRIPTION Phosphatidylinositol-4-phosphate 5-kinase type-1 alpha (EC 2.7..68) (Phosphatidylinositol-4-phosphate 5-kinase type I alpha) (PtdIns(4)P- -kinase alpha) (PIP5KIalpha) (68 kDa type I phosphatidylinositol-4- phosphae 5-kinase alpha). [Source:Uniprot/SWISSPROT;Acc:Q99755]

GACTACCTTTTCTCTCCCTTGAAATTTTCCTGTAACTTAATGTTCATTTCCTTTTGTTTCAGTAAACCAGTCTCAACTCT
TTTCCTTTTCTCTTTCTGTT

In100 candidate4159 (in intron):

LOCUS ENSG00000143398

DESCRIPTION Phosphatidylinositol-4-phosphate 5-kinase type-1 alpha (EC 2.7..68) (Phosphatidylinositol-4-phosphate 5-kinase type I alpha) (PtdIns(4)P- -

kinase alpha) (PIP5KIalpha) (68 kDa type I phosphatidylinositol-4- phosphae 5-kinase alpha). [Source:Uniprot/SWISSPROT;Acc:Q99755]

AGTGGTATTGTCACATCTATAAAATTAATAATTGTTTAACTTTGCTTCTCAAGGCTCTTTTCTCAGAAATGTCTTTTTAT

GGTTTCTTTTTTTCTAATCA

In100 candidate4160 (in intron):

LOCUS ENSG00000159352

DESCRIPTION 26S proteasome non-ATPase regulatory subunit 4 (26S proteasome egulatory subunit S5A) (Rpn10) (Multiubiquitin chain-binding protein) (Antiecretory factor 1) (AF) (ASF). [Source:Uniprot/SWISSPROT;Acc:P55036]

GCATTTCGGATCTCAAGAGTTTCTGATTAAGTGTACTTAACCTATATATTCCCTTTTCTGTTAATACTTGTTTTCCAGTA

CCTTCTTAATTACTTCTCTT

In100 candidate4161 (in intron):

LOCUS ENSG00000143376

DESCRIPTION Sorting nexin-27. [Source:Uniprot/SWISSPROT;Acc:Q96L92]

AATTTATGGATAATGCTTTTTTGTGTTCTAAGAAATCTACCTAACCCAAGGACACCAATCTTTTATGTTTTCTTACAGTT

TTATAGTTTCAGCTATCTTT

In100 candidate4162 (in intron):

LOCUS ENSG00000159445

DESCRIPTION thioesterase superfamily member 4 [Source:RefSeq_peptide;Acc:NP444283]

AGGACAACAAAATATAATCGTTGTCCCTAACATTTCTACTTCATTTATAATCTATGCTTTGTCTTTACTTCAGGTATCTC

TTAGTTTTCACCTCCTTGAA

In100 candidate4163 (in intron):

LOCUS ENSG00000163216

DESCRIPTION Small proline-rich protein 2B (SPR-2B).

[Source:Uniprot/SWISSPRT;Acc:P35325]

CTCCCATTCTTTAGGTTGTCTGTTAACTTTCTTGATAGTTTCCTTTGCTATGCAGAAGTTCTTTAGTTTAATTAGATCTC

ATTTGTCAATTTTCTTTTCT

In100 candidate4164 (in intron):

LOCUS ENSG00000163216

DESCRIPTION Small proline-rich protein 2B (SPR-2B).

[Source:Uniprot/SWISSPRT;Acc:P35325]

AGATGTTAATGCTTGTGATTGCATTATGACATAACAAAAAATGAATTTTGTAGTGTTTTTCAGCTTTTTCAGGTCAGTTA

TGTTCTTTTCTTTACTGGCT

In100 candidate4165 (in intron):

LOCUS ENSG00000163218

DESCRIPTION Peptidoglycan recognition protein I-beta precursor (Peptidoglycn recognition protein intermediate beta) (PGRP-I-beta) (PGLYRPIbeta) (Peptioglycan recognition protein 4). [Source:Uniprot/SWISSPROT;Acc:Q96LB8]

TCTGATAGAGGCATTTGATGCTGTAACATTCCATCTTATCACTGTTTTTTGTTGTATCCCAGAGGCTTTGGTATGTTGTG

TCTCTATTTTCATTTATTTC

In100 candidate4166 (in intron):

LOCUS ENSG00000143552

DESCRIPTION Nuclear pore membrane glycoprotein 210-like precursor (Nucleopoin 210 kDa-like). [Source:Uniprot/SWISSPROT;Acc:Q5VU65]

TTTTCAAATAACCTGTCCTCAAGCTCACTGATTCTTTCTTTTGCTTCATCAGTTCTGCTGTTGAGAGACTGATGCATTCT

TTAGTTTTTTTTTTTTTTTT

In100 candidate4167 (in intron):

LOCUS ENSG00000143549

DESCRIPTION Tropomyosin alpha-3 chain (Tropomyosin-3) (Tropomyosin gamma) (TM5). [Source:Uniprot/SWISSPROT;Acc:P06753]

AGTAGTATTCTTGGCTTTGTTTTCTTTAGAGATTATTTGACTTAACTGTGAGCGCCCTTTTATTTATCCCATCAGTTATT

ACTTTGGCCTCTACTTTTTC

In100 candidate4168 (in intron):

LOCUS ENSG00000163239

DESCRIPTION Tudor domain-containing protein 10. [Source:Uniprot/SWISSPROT;Ac:Q5VZ19]

AAATAGTTGTGCTAGATGTTACATTATTACATTATATACACATAACTTATAGCCTACTCTTGTTGGTTTTATCAGTTTGA

GTACATCTATTTACTTCCCT

In100 candidate4169 (in intron):

LOCUS ENSG00000116580

DESCRIPTION GON-4-like protein (GON-4 homolog). [Source:Uniprot/SWISSPROT;Ac:Q3T8J9]

AACCGAATGTCACTTTTGTTGCCATCTTGGTTGTAACTGGTTTTGGCTGCTTTCTTTGCTGCATCCTGTTTTGATCAGGG

TTTTGTTCTTGTTTTGTTCA

In100 candidate4170 (in intron):

LOCUS ENSG00000132680

DESCRIPTION KIAA0907 (KIAA0907), mRNA [Source:RefSeq_dna;Acc:NM_014949]

AAAGAAGATACTAACCACTAACATTTAATGTATATACTCTTTTTTCTTCTTTCTGTTCCATATGATCTGCAGAAATAATG

TATATACTTTTTTTTTTTTT

In100 candidate4171 (in intron):

LOCUS ENSG00000163554

DESCRIPTION Spectrin alpha chain, erythrocyte (Erythroid alpha-spectrin).

[ource:Uniprot/SWISSPROT;Acc:P02549]

AATGAATGAGTGCTGGAGTAGGAAAAATAACCTATGTTTTCCTATTTTTCTTTCAGGCCATATTTCCAGTAGTTTCTAAA

TTTTCTACCTCCATCACTTT

In100 candidate4172 (in intron):

LOCUS ENSG00000163554

DESCRIPTION Spectrin alpha chain, erythrocyte (Erythroid alpha-spectrin).

[ource:Uniprot/SWISSPROT;Acc:P02549]

CAATATCTTAATATAATTTTCTCTTCCTTGGTAACATCTAGATATCTAAGTACTGTCTACTCTTTTCCTTTACAGCATAT

TTTTCCTTTATAGTATATCT

In100 candidate4173 (in intron):

LOCUS ENSG00000163554

DESCRIPTION Spectrin alpha chain, erythrocyte (Erythroid alpha-spectrin).

[ource:Uniprot/SWISSPROT;Acc:P02549]

TTCTTGTTAGCATCGTTCGTTAGATTTATAGTGAGAAGTAACCTCTTATTCTCAAAATTTCTGTCCCTCATTCTTCAGGT

AATGGTTTTTTTTTTTTTT

In100 candidate4174 (in intron):

LOCUS ENSG00000163554

DESCRIPTION Spectrin alpha chain, erythrocyte (Erythroid alpha-spectrin).

[ource:Uniprot/SWISSPROT;Acc:P02549]

TAGTGTTCCCACCCCTGAGCAACATTAACATCTTATTTTACTCCATATGATACTTTTGCTTTCAGTTTTGTTTTTTAATT

GCTATTAGTGCCTATGCTCT

In100 candidate4175 (in intron):

LOCUS ENSG00000162738

DESCRIPTION Vang-like protein 2 (Van Gogh-like protein 2) (Strabismus 1)

(Lop- tail protein 1 homolog). [Source:Uniprot/SWISSPROT;Acc:Q9ULK5]

AGTAACTACTTCGTATATCTTTTAGCCTTTCAGTCCTCTTGTGAATTTTCTGTTCACCTCCTTTGCCCTTTTTTCTCTAG

GTTTCCTGTCTTTTTCTTCT

In100 candidate4176 (in intron):

LOCUS ENSG00000162739

DESCRIPTION SLAM family member 6 precursor (NK-T-B-antigen) (NTB-A)

(Activaing NK receptor). [Source:Uniprot/SWISSPROT;Acc:Q96DU3]

TATCCTCTTTTAACATTTTTTATTGTGTCCATTTGATTCTTCTCTCTTTTCTTCTTTGTTAGTCTAGCTAACTAGCAGTC

TATCTATATTATTAGTTTTT

In100 candidate4177 (in intron):

LOCUS ENSG00000118217

DESCRIPTION Cyclic AMP-dependent transcription factor ATF-6 alpha (Activatig transcription factor 6 alpha) (ATF6-alpha). [Source:Uniprot/SWISSPROT;AccP18850]

AGATTGTATGTTGGCTGATTTTCTCTCTCCTTTAACTCCACCCAATCCTTGTCCTGGTATTTCTCCTATCAGACTTTTTT
TTTTTTTTTCCAAAGAATAT

In100 candidate4178 (in intron):

LOCUS ENSG00000198929

DESCRIPTION Carboxyl-terminal PDZ ligand of neuronal nitric oxide synthase rotein (C--terminal PDZ ligand of neuronal nitric oxide synthase protein) (itric oxide synthase 1 adaptor protein). [Source:Uniprot/SWISSPROT;Acc:O7502]

CATCATGGAATCTGAAGAGGTTAACAACACTATGGTGGACATCTAAACCAATTCCTCCTATTTTTTTTTTTTCAGTTTAT
CCTTTATTACAGTATACCTC

In100 candidate4179 (in intron):

LOCUS ENSG00000143228

DESCRIPTION Kinetochore protein Nuf2 (hNuf2) (hNuf2R) (hsNuf2) (Cell divisin cycle-associated protein 1). [Source:Uniprot/SWISSPROT;Acc:Q9BZD4]

TTGGGGTAGGTAATAACATCTTCTCTATCATTTCTGTCATTAATAATTTGTTTATTCTTTTTTTTTCCTAACCAGTCTGG
TTATCAGCTTATCACTTTTC

In100 candidate4180 (in intron):

LOCUS ENSG00000185630

DESCRIPTION Pre-B-cell leukemia transcription factor 1 (Homeobox protein PB1) (Homeobox protein PRL). [Source:Uniprot/SWISSPROT;Acc:P40424]

CCATATTCCACTAACTTCTACATGTGTGGATCTCCTTTCAGCAGGCTCTATCTTTACCTCCAAAGTGCTTCCGCATCTCT
TGTCCCATTTTCTTTTTTTT

In100 candidate4181 (in intron):

LOCUS ENSG00000143183

DESCRIPTION Transmembrane and coiled-coil domain-containing protein 1 (Xenoeneic cross-immune protein PCIA3). [Source:Uniprot/SWISSPROT;Acc:Q9UM00]

TTCTTTCAAATTTTTTCATTTTTTTGATAATTTGTGTGAGATTTAACATCTTTTCATATACTTACTATGTTTTTCCAGTT
TTAAGTTCCTAGTTCATTCC

In100 candidate4182 (in intron):

LOCUS ENSG00000143179

DESCRIPTION Uridine-cytidine kinase 2 (EC 2.7.1.48) (UCK 2) (Uridine monophsphokinase 2) (Cytidine monophosphokinase 2). [Source:Uniprot/SWISSPROT;AccQ9BZX2]

CTTTGCCAACTCGCTATTGGCAATACTCTTTCCATAACATGGGTTTCTTTCTTTCTTTTTTTGTTTTTTTTAAAGCAGTT
GTTTTTACTTGGTTTCTCCC

In100 candidate4183 (in intron):

LOCUS ENSG00000143190

DESCRIPTION POU domain, class 2, transcription factor 1 (Octamer-binding trnscription factor 1) (Oct-1) (OTF-1) (NF-A1). [Source:Uniprot/SWISSPROT;AccP14859]

GTCTTCACTGTCACTGGTGCTAACCTTGGATTCACCTCTAATTTATTCCCTGTCACATTTTTCATTGCATATTTCCAGTC

TTCTCTATTTCTGTTTTGCT

In100 candidate4184 (in intron):

LOCUS ENSG00000143199

DESCRIPTION soluble adenylyl cyclase [Source:RefSeq_peptide;Acc:NP_060887]

EXON

TGGTTCACTTCTTGGTGTTCCACAAGTCCCTTAGGCTCTGTTAACTTTTCTTCAATCTTTTTTCTTTCTGTTCATCAGAC

TCAATAATTTCCATTGTCTT

In100 candidate4185 (in intron):

LOCUS ENSG00000143164

DESCRIPTION IQ motif and WD repeats 1 isoform a [Source:RefSeq_peptide;Acc:P_060912]

TTTCTTTTGGCCTTAACATGCTCTTATTATCTCCTTTCTTTAAAATTTTCTCTTAATTATCTCACATTCCCCTTCAGCT

ATTGCCTTATTTCTCTGCTG

In100 candidate4186 (in intron):

LOCUS ENSG00000143147

DESCRIPTION Probable G-protein coupled receptor 161 (G-protein coupled

recetor RE2). [Source:Uniprot/SWISSPROT;Acc:Q8N6U8]

TAGTGAACTGGGCTTTGTAACTTCTTACCTGATTATTTGCTGCCCTGTTTGTCCTTCCTTCTTTCCCTTTTTAGCAGTT

CTACTCTGTGCTTCAACTTT

In100 candidate4187 (in intron):

LOCUS ENSG00000143155

DESCRIPTION TIP41, TOR signalling pathway regulator-like isoform 1

[Source:efSeq_peptide;Acc:NP_690866]

GTTTTCATAGGCTTTACTTCTTTGCTCACTTATAACTCTAAATTCACTTTTTTTTGTTTAGATTTATTCTCTCTTCAGTT

CACCTAGTCTTGTATCTTTC

In100 candidate4188 (in intron):

LOCUS ENSG00000143153

DESCRIPTION Sodium/potassium-transporting ATPase subunit beta-1

(Sodium/potssium- dependent ATPase beta-1 subunit).

[Source:Uniprot/SWISSPROT;Acc:P0506]

AGTAACTGATAGCCACCCTCATTCCCATCTATGTGTTGAGTCTTTTTTTTTTTTTTTTTTTGCTTTTGCAGCTATTTCAG

CCTCCATCCTGTTGTCTTGT

In100 candidate4189 (in intron):

LOCUS ENSG00000143153

DESCRIPTION Sodium/potassium-transporting ATPase subunit beta-1

(Sodium/potssium- dependent ATPase beta-1 subunit).

[Source:Uniprot/SWISSPROT;Acc:P0506]

AACTCTGTTTACCATTGCTGTCTCAAGCATTAACTATCTGTGGAAGCTTCTGCTCACCTTTATTTTTGCAGGTATCTCTT

CTCTTTACAATACTGCTGTT

In100 candidate4190 (in intron):

LOCUS ENSG00000143156

DESCRIPTION Nucleoside diphosphate kinase 7 (EC 2.7.4.6) (NDK 7) (NDP kinas

7) (nm23-H7). [Source:Uniprot/SWISSPROT;Acc:Q9Y5B8]

ACTATAGCTGCACTGGCTTTCTTTTAGTTAACATTCGCATGGTATACCTTTTCCATTCTTTTTACTTTAGATTTTCAGTA

TTCTCTTCTATGTGTGCCTT

In100 candidate4191 (in intron):

LOCUS ENSG00000143156

DESCRIPTION Nucleoside diphosphate kinase 7 (EC 2.7.4.6) (NDK 7) (NDP kinas

7) (nm23-H7). [Source:Uniprot/SWISSPROT;Acc:Q9Y5B8]

AACTGCTCTTTGGCTGCATTCACTATTTTTCTTTTTTTTTAACAAGTGATACTGTTTTCTTTAAGATCTCAGCTTAACAT

TTTACCTCTTTTATTTCTTT

In100 candidate4192 (in exon):

LOCUS ENSG00000117479

DESCRIPTION Thiamine transporter 1 (ThTr-1) (ThTr1) (Thiamine carrier 1) (T1)

(Solute carrier family 19 member 2). [Source:Uniprot/SWISSPROT;Acc:O6077]

CTAGTTAACCTTTTGTTATCTTTTTAATTATTCCATTGCTTTGTGATTTTTACCTTTGACTCTTCCCCCTTATTTCAGAT

GTTTTCCCCCTTTTTTTTGG

In100 candidate4193 (in intron):

LOCUS ENSG00000117501

DESCRIPTION CDNA: FLJ23550 fis, clone LNG08970.

[Source:Uniprot/SPTREMBL;Ac:Q9H5D7]

AAAGGTATGTCACTTTTGTTTAACTTTTAAAAAAAGCCAACTTTTTGTTTCACTTATCATTTGTATTGTTGTTTTCAGTT

CAATTCCATTTATTTCTGCT

In100 candidate4194 (in intron):

LOCUS ENSG00000117523

DESCRIPTION HBxAg transactivated protein 2

[Source:RefSeq_peptide;Acc:NP_05987]

TGAGTACCAAAGGAATTTGAGAGACCATAGCTAATCTCTTTAACAAATGTGGAAACCACTTAGACTTTTTTCTTCCAGAT

TTTCTCTTCTTTTTAATTGT

In100 candidate4195 (in intron):

LOCUS ENSG00000197959

DESCRIPTION Dynamin-3 (EC 3.6.5.5) (Dynamin, testicular) (T-dynamin).

[Soure:Uniprot/SWISSPROT;Acc:Q9UQ16]

CATAGAATAAGTTAGTAACTCTCCCTCTGCTTCTCTTTTACTTTCTTCTATTAGCTTTTGCAATTCTTAAAAGCCCAGGG

ATGCATTTTAGTCTCTCTTT

In100 candidate4196 (in intron):

LOCUS ENSG00000197959

DESCRIPTION Dynamin-3 (EC 3.6.5.5) (Dynamin, testicular) (T-dynamin).

[Soure:Uniprot/SWISSPROT;Acc:Q9UQ16]

ATACATTTATATTGTCTATTATATTTAACTATGTAGTTAACTTTACTGGTGTTCTTCAGTTTTTCCTGGGTATTTGAGTT

ACTGTCCAGTGTCCTTTTTT

In100 candidate4197 (in intron):

LOCUS ENSG00000197959

DESCRIPTION Dynamin-3 (EC 3.6.5.5) (Dynamin, testicular) (T-dynamin).

[Soure:Uniprot/SWISSPROT;Acc:Q9UQ16]

GCAATATTGAGTCTTTCTACTTGTTAACATGTAAATAGCTCTTCATTTATTTAGTTCTTCTTTAATATCTTTCATCAGAG

TTTTGTAGTTTTCCTCATAC

In100 candidate4198 (in intron):

LOCUS ENSG00000197959

DESCRIPTION Dynamin-3 (EC 3.6.5.5) (Dynamin, testicular) (T-dynamin).

[Soure:Uniprot/SWISSPROT;Acc:Q9UQ16]

TTGCTGGATCTATCCATTTCTGATAGAGGAGTGTTAAATTCTGTAACTGTAATAGTGGATCCATCCGTTTCTTTGCAGTT

CTTTCTGTTTCTCCTCAGGT

In100 candidate4199 (in intron):

LOCUS ENSG00000197959

DESCRIPTION Dynamin-3 (EC 3.6.5.5) (Dynamin, testicular) (T-dynamin).

[Soure:Uniprot/SWISSPROT;Acc:Q9UQ16]

TAAGATAATTTAATCTTTTGTCTGTTAACTATATGTCGTCTCATTTCTAATTTTGTTTTCTCATCTTTTATATTACAGAA

TTCTGTTTTATTGTCTGATT

In100 candidate4200 (in intron):

LOCUS ENSG00000135845

DESCRIPTION Phosphatidylinositol N-acetylglucosaminyltransferase subunit C EC

2.4.1.198) (Phosphatidylinositol-glycan biosynthesis class C protein) (PG-C).

[Source:Uniprot/SWISSPROT;Acc:Q92535]

TAACCCCATAAGGAAAGAATTGGAAGTTTCATTTATTTTTTTCCTACCTTTAGTTCTCTTTTTTCCATCTTTCAGATCTT

TAGATGTTCTGAGTTTTTCT

In100 candidate4201 (in intron):

LOCUS ENSG00000180999

DESCRIPTION Uncharacterized protein C1orf105.

[Source:Uniprot/SWISSPROT;AccO95561]

TTTTAACTACCAATGCCCAATTCATTCTCCCTTATTTCTTGATGATTTTAGCTCCTGTTTCAATGTCACTCCCTCCAGTG

ATACTCCTGTTTTAATTCTT

In100 candidate4202 (in intron):

LOCUS ENSG00000152061

DESCRIPTION RAB GTPase activating protein 1-like.

[Source:Uniprot/SPTREMBL;cc:Q5VTT7]

AGTATGGACATTTCAACAATATTTTTCCAATCCATGAACATGAGATAACTTTCCATCTATTTGTGTCATTTTCAGTTTCC

TTCATCAATGTCTTACAGTT

In100 candidate4203 (in intron):

LOCUS ENSG00000152061

DESCRIPTION RAB GTPase activating protein 1-like.

[Source:Uniprot/SPTREMBL;cc:Q5VTT7]

TGTTTGGAAATCTATGGGTCTCATAACTTTGACTGTCCTCTTCTTTTCCTAAATTTGGGATCTTTTCAGCCATTATTTCT

TTATATAGATTTTTCAGTCT

In100 candidate4204 (in intron):

LOCUS ENSG00000152061

DESCRIPTION RAB GTPase activating protein 1-like.

[Source:Uniprot/SPTREMBL;cc:Q5VTT7]

AGTTGCGCATAGTAACCACTGATGATCCTTTGAATTTCTGCCATCTCAGTGATAATGTCTCTGTTTTCCTTTCTGATCTT

ATTTATCTGTATCTTTTTGC

In100 candidate4205 (in intron):

LOCUS ENSG00000152061

DESCRIPTION RAB GTPase activating protein 1-like.

[Source:Uniprot/SPTREMBL;cc:Q5VTT7]

TGTTTGTCAATTTTGTTTAACTTTTAAAAAAACAATTTTTTGTTTCATTGATCTTTTATTCTGTTTTCTTCATTTCAGTT

TCATTTATTTCTGCTCTGAT

In100 candidate4206 (in intron):

LOCUS ENSG00000152061

DESCRIPTION RAB GTPase activating protein 1-like.

[Source:Uniprot/SPTREMBL;cc:Q5VTT7]

TCTTTGCGGGGTTGGAGTTTTCATATTGTGACTTTTGGGCTAACATCTCTCTAGCTCTTCTTATTAGTTAATTTTCAGCA

GATTTTTTTTTTCTATATTG

In100 candidate4207 (in intron):

LOCUS ENSG00000152061

DESCRIPTION RAB GTPase activating protein 1-like.

[Source:Uniprot/SPTREMBL;cc:Q5VTT7]

AGATTGGCTAACTGTTCACCAAATCTATTTTCTTTTCTTTCTGCTGACACAGCTGGACTATATTTCCCAGCCTCCTCTGG

CTTCTAAAATTATTTTCTTT

In100 candidate4208 (in intron):

LOCUS ENSG00000116147

DESCRIPTION Tenascin-R precursor (TN-R) (Restrictin) (Janusin).

[Source:Unirot/SWISSPROT;Acc:Q92752]

TCCTACTTTATTTTTATTTAACCAATTTCTCCAAATTTCATGTATTTTATTTTGCTCTATAGTTTATCTTTTTATCAGCA

CTTTGAGTTCTTTTATTATC

In100 candidate4209 (in intron):

LOCUS ENSG00000143207

DESCRIPTION E3 ubiquitin-protein ligase RFWD2 (EC 6.3.2.-) (RING finger andWD

repeat domain protein 2) (Constitutive photomorphogenesis protein 1 homoog)

(hCOP1) (RING finger protein 200). [Source:Uniprot/SWISSPROT;Acc:Q8NHY2

CGTTTTTCTCTCTAACTGCTTTTAAGATTTTCTTTCTGACATTGGTTTTCAGCAATTTGACTATGTTATTCCTACATTTT

ATTATTTTTATATTTATTCT

In100 candidate4210 (in intron):

LOCUS ENSG00000143207

DESCRIPTION E3 ubiquitin-protein ligase RFWD2 (EC 6.3.2.-) (RING finger andWD repeat domain protein 2) (Constitutive photomorphogenesis protein 1 homoog) (hCOP1) (RING finger protein 200). [Source:Uniprot/SWISSPROT;Acc:Q8NHY2

AGATTGTCTAAGCACTTAACATTTTATCACATTTGTTTTATCATTCTCATTCAGCCTTTCTTTTACCTATATATGCGTAT

AATTTCTTTTCTGGATTGTT

In100 candidate4211 (in intron):

LOCUS ENSG00000116183

DESCRIPTION Pappalysin-2 precursor (EC 3.4.24.-) (Pregnancy-associated plasa protein-A2) (PAPP-A2) (Pregnancy-associated plasma protein-E1) (PAPPE).[Source:Uniprot/SWISSPROT;Acc:Q9BXP8]

GTCTCATGATTGCTTTTAGAGAGGAGGGGGAGAAATGGAACGTATAACCTTTCTCCGTTTCTTTTTTGTTTCTCTCAGTT

TTCTTCAGTCTCTTTCTTCA

In100 candidate4212 (in intron):

LOCUS ENSG00000116183

DESCRIPTION Pappalysin-2 precursor (EC 3.4.24.-) (Pregnancy-associated plasa protein-A2) (PAPP-A2) (Pregnancy-associated plasma protein-E1) (PAPPE).[Source:Uniprot/SWISSPROT;Acc:Q9BXP8]

TTTTCTTTTTTAACTCTAGCTAATGGCTTGTCCATTTTGTATATCTTTTCAGAAAACCAAGTTTTTGTTTCATTGAACTT
TCTTAGTCTCTCTTTTGTTT

In100 candidate4213 (in intron):

LOCUS ENSG00000116183

DESCRIPTION Pappalysin-2 precursor (EC 3.4.24.-) (Pregnancy-associated plasa protein-A2) (PAPP-A2) (Pregnancy-associated plasma protein-E1) (PAPPE).[Source:Uniprot/SWISSPROT;Acc:Q9BXP8]

GAGTTTCATTCTTTCCCCTTCTCTCATTTTCCCCCCTCACACCCCAATAACCAACTCTCTTTTATCTTTCTCTTCCAGCT
CTCCTCTTCCTGTTAGTTCT

In100 candidate4214 (in intron):

LOCUS ENSG00000075391

DESCRIPTION Ras GTPase-activating protein nGAP (RAS protein activator-like). [Source:Uniprot/SWISSPROT;Acc:Q9UJF2]

TTCTAAGGTTTGGAGGATGTGTACCATAACTGATTTGTATGTCATTTTTGTTGTTTTGATTCACCTTTTACAATTCAGAA
TTTTTTTTTTTTTTTTTTTT

In100 candidate4215 (in intron):

LOCUS ENSG00000075391

DESCRIPTION Ras GTPase-activating protein nGAP (RAS protein activator-like). [Source:Uniprot/SWISSPROT;Acc:Q9UJF2]

AGTTAACTATATAAATGTGTGTCTTTTTCCAGTCTCTCTATTCTGTTTCATTGCTTTTTCTGTTCTTTGTAGATACTATG

CTCTCTTGATTACTGTAGCT

In100 candidate4216 (in intron):

LOCUS ENSG00000116191

DESCRIPTION Ral GEF with PH domain and SH3 binding motif 2 isoform 2

[Sourc:RefSeq_peptide;Acc:NP_689876]

ATTCTTTCCTGCTTGTCTCTTAACCTTACATATTTTCTCTCTGTTTTTGTCCTCCATTCTGAGTGATTTCCTCAGATCTA

CATTTCAGTTCACTGATTCT

In100 candidate4217 (in intron):

LOCUS ENSG00000057252

DESCRIPTION Sterol O-acyltransferase 1 (EC 2.3.1.26) (Cholesterol

acyltranserase 1) (Acyl coenzyme A:cholesterol acyltransferase 1) (ACAT-1).

[Source:niprot/SWISSPROT;Acc:P35610]

AACATATTGATCATTCTGATTTTTTTGTCCTAAAAGTAACAATTCCATTATCTTTAGTTTTTCAGATCTGCATCTCTTGG

CTTTTGTTTCTGCTGTTTCC

In100 candidate4218 (in intron):

LOCUS ENSG00000057252

DESCRIPTION Sterol O-acyltransferase 1 (EC 2.3.1.26) (Cholesterol

acyltranserase 1) (Acyl coenzyme A:cholesterol acyltransferase 1) (ACAT-1).

[Source:niprot/SWISSPROT;Acc:P35610]

GTTTTTCCTTTTGTTTAGTTTTCTTCACATTTAACTCTCCTAGCTTTTGGTTTTCTTCTTTTACTGCCTTTTCTTCAGCC

CCTTCTAATTTTTTTTTTTT

In100 candidate4219 (in intron):

LOCUS ENSG00000162779

DESCRIPTION CDNA FLJ32940 fis, clone TESTI2007750.

[Source:Uniprot/SPTREMBLAcc:Q96M01]

CTCCCTCTCTGTGGGTTGTCTGTTTAACCTGCTGATTATTTCTTTTGTTGTGCAGAAGTTCTTTAGTTTAATTAGGTCCC

ATCTATTTATTTTTGTTTTT

In100 candidate4220 (in intron):

LOCUS ENSG00000143340

DESCRIPTION

TGAGTTCCTGCGTTGGCCATTCATAACTGATTTACATTTTATTATTCTTCCTTTCTCTGCCTATAGATCCTATGACAGGA

GTCTTTCTCTATTATTATTA

In100 candidate4221 (in intron):

LOCUS ENSG00000135847

DESCRIPTION Acyl-CoA-binding domain-containing protein 6.

[Source:Uniprot/SISSPROT;Acc:Q9BR61]

ACTGTTGCCCCATAGTGGCTGTACTAATTTACATTCTCACTAACCATGTATAAGAGTTTTTCTCTGCATCCTTGCCAGCA

TCTGTTAATTTTTAATCTTT

In100 candidate4222 (in intron):

LOCUS ENSG00000135847

DESCRIPTION Acyl-CoA-binding domain-containing protein 6.

[Source:Uniprot/SISSPROT;Acc:Q9BR61]

AGTATTCTTCTAACTCATGAGCATGGCATGTCTTTCTATTTTTTTGTGAATTTCTTTTGTCAGTTTTGTAGTTTTCCTTA

TAGAGATCTTTCACCTCCTT

In100 candidate4223 (in intron):

LOCUS ENSG00000135847

DESCRIPTION Acyl-CoA-binding domain-containing protein 6.

[Source:Uniprot/SISSPROT;Acc:Q9BR61]

TGTAGTTTAGTGGTATTCTGTAGTGGTAACATTTGAGGTTTTTCTCTTTCTTGATTTGTGTGTTTGCTCCACCAGTGGTT

TTTATATTTTCACGTGTCTT

In100 candidate4224 (in intron):

LOCUS ENSG00000135847

DESCRIPTION Acyl-CoA-binding domain-containing protein 6.

[Source:Uniprot/SISSPROT;Acc:Q9BR61]

CATAGCTTCAATTTTAAGCATGCTTGAATTTTTTTACATTTTAACTCTATTATGTAATTTTGTTTATTTTTAATTCAGTT

GGGTATTTTTATTTTACCCT

In100 candidate4225 (in intron):

LOCUS ENSG00000143324

DESCRIPTION xenotropic and polytropic retrovirus receptor

[Source:RefSeq_petide;Acc:NP_004727]

GTATTATATAACATTTACTACTCCCTTCTTTCTTCCTTTTTTCATTTTCTCTTTTTGTAAATTTTCCCTTCTTCCCAGTT

TTTTTTTTAAATTTTCATGT

In100 candidate4226 (in intron):

LOCUS ENSG00000143324

DESCRIPTION xenotropic and polytropic retrovirus receptor

[Source:RefSeq_petide;Acc:NP_004727]

AGCATCCATGCTGTTTTCTAACTATAGTTATTCCTGCTTTTTTTCCTCCTTTACTTCCTTTCTTAGTTATCCATTCAGCT

CATTCCTTTCACTGCTAATT

In100 candidate4227 (in intron):

LOCUS ENSG00000198216

DESCRIPTION Voltage-dependent R-type calcium channel subunit alpha-1E

(Voltge- gated calcium channel subunit alpha Cav2.3) (Calcium channel, L type,

apha-1 polypeptide, isoform 6) (Brain calcium channel II) (BII).

[Source:Unirot/SWISSPROT;Acc:Q15878]

AAACAAACTTCCTTTAATTCCTTGAATTCATTCTTAACATCTCTGTTCTTCCTAATGCTTGTTTCTCAGCTCTACTTCCA

GGATTGGTTCATCTATTTTT

In100 candidate4228 (in intron):

LOCUS ENSG00000121446

DESCRIPTION Regulator of G protein signaling protein (Regulator of G-protei

signalling like 1). [Source:Uniprot/SPTREMBL;Acc:Q86UV0]

AGTCATGAGCATGGAGTAACCTTTCATTTGTGTATATCTTCTTTTTTGTTTCTTTCATCAGCGTTTTGTAATTTTTCTTG

TATAGATCTTTCACTTCTTT

In100 candidate4229 (in intron):

LOCUS ENSG00000135862

DESCRIPTION Laminin subunit gamma-1 precursor (Laminin B2 chain).

[Source:Uiprot/SWISSPROT;Acc:P11047]

AACCCATAAACACTTTCTACATAACCCACTTTTGCACACTTTGCTCTCTTTTCTAAGATATTATCAGCTGTGTATACTCT

TTTCTTCCTTTTACTTTGAC

In100 candidate4230 (in intron):

LOCUS ENSG00000058085

DESCRIPTION Laminin subunit gamma-2 precursor (Laminin 5 gamma 2 subunit)

(alinin/nicein/epiligrin 100 kDa subunit) (Laminin B2t chain) (Cell- scatterng

factor 140 kDa subunit) (CSF 140 kDa subunit) (Large adhesive scatter fator 140

kDa subunit) (Ladsin 140 kDa su [Source:Uniprot/SWISSPROT;Acc:Q1375]

CAAATGTAGAGCAAACTCAACTTGTTTTTCAAACTGATCTATTTAACAAGCCTTTTACGCACTTCCTTTGTGTCAGGCAC

TGTTCTCTTGTCTCTAGCTT

In100 candidate4231 (in intron):

LOCUS ENSG00000116698

DESCRIPTION Protein SMG7 (SMG-7 homolog) (EST1-like protein C).

[Source:Unirot/SWISSPROT;Acc:Q92540]

AGAATCCCTTCCTTTAACTGTACTCTTTAATGTTTACCATCCTTCAGGATCTTTTTTCACTTGTGTCGCTCTCTTCTTTT

AAATTATTCCCTTTACCGAT

In100 candidate4232 (in intron):

LOCUS ENSG00000116698

DESCRIPTION Protein SMG7 (SMG-7 homolog) (EST1-like protein C).

[Source:Unirot/SWISSPROT;Acc:Q92540]

GCCAAGCGTTTCAAGTTTGCCCTAACCTATGTTTTTCCTTTAGTTACCATCTACTTTTCCTTAATCCCTTGCTATCAGTC

CATTTTTTTCTGAGTACATT

In100 candidate4233 (in intron):

LOCUS ENSG00000116698

DESCRIPTION Protein SMG7 (SMG-7 homolog) (EST1-like protein C).

[Source:Unirot/SWISSPROT;Acc:Q92540]

CTGTTATAACTGCTATATTGCATTAATATTCTCATCTGTTTCCCAAAACTCTTTGGGTTCTTTTCTCAGATCGCAGAAGT

TGAGTTCTTTTTCTTTCCTT

In100 candidate4234 (in intron):

LOCUS ENSG00000143344

DESCRIPTION Ral guanine nucleotide dissociation stimulator-like 1 (RalGDS-lke

1). [Source:Uniprot/SWISSPROT;Acc:Q9NZL6]

AAAGTTTAACTGCCAAAGGCACTTTCTTTCCTATAGTTCTGACAACTTCTCCTTTTGTTATATACCACTGATATCCAGGT

TCAGTTCATCATCTATTTCA

In100 candidate4235 (in intron):

LOCUS ENSG00000198756

DESCRIPTION glycosyltransferase 25 domain containing 2

[Source:RefSeq_peptie;Acc:NP_055916]

TTCCCCATTTGTACCATCTTTAACTGAAAGTGGTATGTTAATATCTCTTATTATTGTTATTTCTGTTTTTCTTTACAGTT

TCTATACTTTCTACTATATT

In100 candidate4236 (in intron):

LOCUS ENSG00000116667

DESCRIPTION Uncharacterized protein C1orf21 (Proliferation-inducing gene 13protein). [Source:Uniprot/SWISSPROT;Acc:Q9H246]

GTTACCTTGTTTCTCCCGTTTCTTATTTAACTATGCTATATCTTTTACTTCTCTTTCTCCATTAACTAATTCCCACAGCC

CTTTTCTTAAACTTTTCCTA

In100 candidate4237 (in intron):

LOCUS ENSG00000116667

DESCRIPTION Uncharacterized protein C1orf21 (Proliferation-inducing gene 13protein). [Source:Uniprot/SWISSPROT;Acc:Q9H246]

TGGCTTTGGTGTTAACTTCTGGAAAATCGACTGTCTTTCATTTGTTCCTTTCTTCTTAGAGACTCCCATTGCTTGCAGGT

TACATCTCTACACTCTATTT

In100 candidate4238 (in intron):

LOCUS ENSG00000116406

DESCRIPTION ER degradation-enhancing alpha-mannosidase-like 3. [Source:Unipot/SWISSPROT;Acc:Q9BZQ6]

TAAAATTTGTTGATAACCTCATTTATGTTCAATTCAAATTTCACTTCGTGTTGTCACCTTTTATTTCTTTGCCAGACTCT

CATGTTTGTTGGCTAATTCT

In100 candidate4239 (in intron):

LOCUS ENSG00000135842

DESCRIPTION Niban protein. [Source:Uniprot/SWISSPROT;Acc:Q9BZQ8]

AAGCCATAAGATATGTCTCCTGACCCATGACAACTTTAGTGACTAACCCACTTACTCACACATTTCATGTTTCAGCATTT

CCTCCCTTCGGTATCATTAT

In100 candidate4240 (in intron):

LOCUS ENSG00000135842

DESCRIPTION Niban protein. [Source:Uniprot/SWISSPROT;Acc:Q9BZQ8]

TTTCACTGCATTTAACACTAACTGAGGTTTCATTTTGAATCAAATGTTGGTGTTTTTCCTCTTTTCACTTATTTCAGCTT

GCTCTCTCAGCTTGCAGTTT

In100 candidate4241 (in intron):

LOCUS ENSG00000135842

DESCRIPTION Niban protein. [Source:Uniprot/SWISSPROT;Acc:Q9BZQ8]

AGCCTACTTGTTAACTTTTAATTTTATATTTTCTATCTTTTTTCTCTCTTTTTTTTTCCCTGAACAGTTTCTGTGCTCTG

TCATTCAGTTCTCTACTTCT

In100 candidate4242 (in intron):

LOCUS ENSG00000121486

DESCRIPTION N2,N2-dimethylguanosine tRNA methyltransferase-like

[Source:Refeq_peptide;Acc:NP_112196]

AGACTACATTTAATATCTTCCATATCTGATCCTAACCTGCTTGTCCAAATTTATTTCTCAGTATTCTTCACCATACACCG

TTTTTCTTTACTCTTTTGAG

In100 candidate4243 (in intron):

LOCUS ENSG00000143341

DESCRIPTION Hemicentin-1 precursor (Fibulin-6) (FIBL-6).

[Source:Uniprot/SWSSPROT;Acc:Q96RW7]

GTTTTCTTCTCTTGTTCTTTTCTAACTTTAAAATTTAAATGTTTCTTTTTACCTTTCTAATGCAGATATTTGAAACCTTT

TAACTTCGTCATTTATTTTC

In100 candidate4244 (in intron):

LOCUS ENSG00000143341

DESCRIPTION Hemicentin-1 precursor (Fibulin-6) (FIBL-6).

[Source:Uniprot/SWSSPROT;Acc:Q96RW7]

AGAGAGGATCTCTCACTCAATAACTGGACAAGGTTTATCATTCCTTCTATTCCTAATATGTGCCTTCAGATATTTCTTAG

CTGTCCTCATGTTTGGCCCA

In100 candidate4245 (in intron):

LOCUS ENSG00000162670

DESCRIPTION Protein FAM5C precursor (DBCCR1-like protein 1).

[Source:Unipro/SWISSPROT;Acc:Q76B58]

AGTTGTTCACAATAGTCTCTAACGATTCTTTGTGCTTCTGGGTTCTCAGTTGTTATGTCTCCTTTTTCATTTCTGATTTT

ATTTAATTAGGTCTTTTTTT

In100 candidate4246 (in intron):

LOCUS ENSG00000116747

DESCRIPTION 60 kDa SS-A/Ro ribonucleoprotein (60 kDa Ro protein) (60 kDa rionucleoprotein Ro) (RoRNP) (Ro 60 kDa autoantigen) (TROVE domain family memer 2) (Sjoegren syndrome type A antigen) (SS-A) (Sjoegren syndrome antigen 2). [Source:Uniprot/SWISSPROT;Acc:P10155]

AGCACCTAACATGTAGTAGAAGTACACTTTGGTTCTCTATTTCTCCCTTCCACACATATTTTATATACAGAATCTCTTC
AGTAATTCCTGACTTATTTT

In100 candidate4247 (in intron):

LOCUS ENSG00000162687

DESCRIPTION potassium channel, subfamily T, member 2 [Source:RefSeq_peptideAcc:NP_940905]

ATTACATTTTAACATTATTCTGTGTTTTCTGTGTACTTACTATTTCCAGTGAGTTTTATACCTTCAGATGATCTTTTCTT
GCTTATTAACATCCTTTCCT

In100 candidate4248 (in intron):

LOCUS ENSG00000162687

DESCRIPTION potassium channel, subfamily T, member 2 [Source:RefSeq_peptideAcc:NP_940905]

AAATGTCTCACCATATTTATTTATGTTGCTAACACTACTCTGAACGTGTTTTATTCCTTAATGTTCCACCAGCGTTTTCT
GTAGTTGACTTGTATCCTTC

In100 candidate4249 (in intron):

LOCUS ENSG00000162687

DESCRIPTION potassium channel, subfamily T, member 2

[Source:RefSeq_peptideAcc:NP_940905]

GTAACAAAGCCTGTTTTGCAATATTAATTTATGATTCATTTTCCATTTTATTTCCATATCTCTTTTCGCCAGATGCTAAA

CTTGTATTTGCTTTTCTGTT

In100 candidate4250 (in intron):

LOCUS ENSG00000066279

DESCRIPTION Abnormal spindle-like microcephaly-associated protein

(Abnormalspindle protein homolog) (Asp homolog).

[Source:Uniprot/SWISSPROT;Acc:Q8IZT]

AGTTAGGAAACTACCACCACTGAGATACAAAATATTTCTGTAACACAAAGTTTCCTTATGCCCCTTTGTTGTCAGTTCTC

TTTCTTTATTCCCTGGATTT

In100 candidate4251 (in intron):

LOCUS ENSG00000134376

DESCRIPTION Crumbs homolog 1 precursor. [Source:Uniprot/SWISSPROT;Acc:P8227]

ACTACTTCCTAAGGTCTAACCTCTGAAAAAGGTTGGACCTTTCTCTTGAAATTCTCATAGGTTCTTTTCTTCTCTCAGAA

ATAAATCTCTACTCTTTCTT

In100 candidate4252 (in intron):

LOCUS ENSG00000162701

DESCRIPTION DENN/MADD domain containing 1B

[Source:RefSeq_peptide;Acc:NP_65414]

AGAAAGATTTATTTTGCTTACTGTAACGAAACTAAACTTATTTCTTGAGATCTCCTCAGACATTAAGTTCTATGATTTTG

TGTCTTGGTCCTTCTACCTC

In100 candidate4253 (in intron):

LOCUS ENSG00000151414

DESCRIPTION Serine/threonine-protein kinase Nek7 (EC 2.7.11.1) (NimA-relate

protein kinase 7). [Source:Uniprot/SWISSPROT;Acc:Q8TDX7]

GACAAAATTACTCTGCTTTATGAAGAGAACTATGGGGTTAACATTAAAGTAGGATTAGGTCTTCTAATTCTTTTCCAGTT

CTGTGGTTTTATTATTTTAT

In100 candidate4254 (in intron):

LOCUS ENSG00000151418

DESCRIPTION Vacuolar ATP synthase subunit G 3 (EC 3.6.3.14) (V-ATPase G

subnit 3) (Vacuolar proton pump G subunit 3) (V-ATPase 13 kDa subunit 3).

[Souce:Uniprot/SWISSPROT;Acc:Q96LB4]

ACCTGGTCGTGCTACTATCTTTCTAATACCATCTCCTAACATTCATGATTGAATATCCTGTTCTCTTTGCTCCAGCCATG

ATGGTGTTTTTGCTCTTCCT

In100 candidate4255 (in intron):

LOCUS ENSG00000081237

DESCRIPTION Leukocyte common antigen precursor (EC 3.1.3.48) (L-CA) (T200)

CD45 antigen). [Source:Uniprot/SWISSPROT;Acc:P08575]

TTTTTTTTGTAACCTCCCTTAAAGATTCTTTGATGCTTTGCTCTATTACTGTGGGCCTGGTCTTTTTTTCCCCCCCAGTT

TTTTCTTCTTTAAACTCTGG

In100 candidate4256 (in intron):

LOCUS ENSG00000118200

DESCRIPTION calmodulin regulated spectrin-associated protein 1-like 1

[Soure:RefSeq_peptide;Acc:NP_982284]

AACTCTGTCTCAAAAAAAAAAAAAAAAGCTCATTTATGTACTTGAATAACTTCTTGGTAAATTTCTGCATATCAGATATT

TTCGTTTATTTGATAGCTTT

In100 candidate4257 (in intron):

LOCUS ENSG00000118200

DESCRIPTION calmodulin regulated spectrin-associated protein 1-like 1

[Soure:RefSeq_peptide;Acc:NP_982284]

CTAAATGAAGTGCCTCTATCAACCCCTTGTTTTCTATACCCTAACACTCTACTTTTTTTCTTTCATAGCACTTTTCAGAC

TAATTACTTTTCTTAATCAT

In100 candidate4258 (in intron):

LOCUS ENSG00000118200

DESCRIPTION calmodulin regulated spectrin-associated protein 1-like 1

[Soure:RefSeq_peptide;Acc:NP_982284]

ATTAATGCTGATGCTTTTCTGAGAGATAGCATTGAAAAGAAATCTAACTTCACTACCAAATGTTGTGTTCTCTTTCAGTA

AATTTTTATTTTTCTATTTT

In100 candidate4259 (in intron):

LOCUS ENSG00000118200

DESCRIPTION calmodulin regulated spectrin-associated protein 1-like 1

[Soure:RefSeq_peptide;Acc:NP_982284]

AATTTGAAGTCTTATGATGTGGAACTTTTAGCTTTAACTGTCCATTTCTTTTCTTTGCATCTTTTATCGTTATCCCAGCT

TTTATTCCCTTTTAGATTCT

In100 candidate4260 (in exon):

LOCUS ENSG00000118200

DESCRIPTION calmodulin regulated spectrin-associated protein 1-like 1

[Soure:RefSeq_peptide;Acc:NP_982284]

AAATATGGTTAAGATTACAAATTATGTGTTTTATTTGTTGCTTTTTTTTAACCTTTTAATGTATATTCTTGTCTTCAGAT

GGTTTGCTATTTTTCTCTCC

In100 candidate4261 (in intron):

LOCUS ENSG00000116852

DESCRIPTION Kinesin family member 21B. [Source:Uniprot/SWISSPROT;Acc:O75037

AGTTCCTTGGGTCTTTTCCTTTGGACCTGTTTGTAACCGTTCTTACATTCTATCTTCATTATGCTGCTCTTCAGCCCATT

CATTCCATTTTTTATTCATT

In100 candidate4262 (in intron):

LOCUS ENSG00000134369

DESCRIPTION neuron navigator 1 [Source:RefSeq_peptide;Acc:NP_065176]

AAATATTAGTTAATACTGATGTCTGCAACGCTAACCTAATAGCACGTGGTTCATTCTAAACTTTCTTCCTTTATGCAGCC

TTTTTTTTTTTTTTTTTAGA

In100 candidate4263 (in intron):

LOCUS ENSG00000134369

DESCRIPTION neuron navigator 1 [Source:RefSeq_peptide;Acc:NP_065176]

AGGGTGGAAAGCCTTTGTCTCTGCTTGTTAACATCACTGCACCTCATTTTCCTTCCAAATCTTATACAGCTTCTGCAGCC

AGTAGTTTGTATTTCTCTTC

In100 candidate4264 (in intron):

LOCUS ENSG00000077157

DESCRIPTION Protein phosphatase 1 regulatory subunit 12B (Myosin

phosphatastargeting

subunit 2) (Myosin phosphatase target subunit 2).

[Source:Uniprt/SWISSPROT;Acc:O60237]

AGAGTCAAATCTCTCATATCCTGTAACTTTGCTGCATGCTCCTTAGTTTTTTCTTTATCTACACATATATCTGGTCAGTT

TGATACATTTTTTTCTTTTT

In100 candidate4265 (in intron):

LOCUS ENSG00000077157

DESCRIPTION Protein phosphatase 1 regulatory subunit 12B (Myosin

phosphatastargeting

subunit 2) (Myosin phosphatase target subunit 2).

[Source:Uniprt/SWISSPROT;Acc:O60237]

TTCCTGCCTCTCTCCCTCATGCTGATAACTTCCAAATTCTTTCACCTTGATCTGCTTATACTCACTTGTCAGATTGAGTC

ACTTTCATCATTCATGTCTT

In100 candidate4266 (in intron):

LOCUS ENSG00000143858

DESCRIPTION Synaptotagmin-2 (Synaptotagmin II) (SytII).

[Source:Uniprot/SWISPROT;Acc:Q8N9I0]

CCTCTTTTACCCTTTATTCTGCTTCTGCCAAGTTTTAACCACTGTGTTCTGGATTATTTCTTCTGATCTGCTTTCCAGTT

CATTAATGTCTTCTTCAGCT

In100 candidate4267 (in intron):

LOCUS ENSG00000117139

DESCRIPTION Jumonji, AT rich interactive domain 1B (RBP2-like)

[Source:RefSq_peptide;Acc:NP_006609]

GGAGTCGGTTGGTGGGTGGAGGTTAGTCTCTGCTAACATCACTTCATTTCTTTATCTGTTCATTTTTGTATTGGCCAGAC

TTTATCTGAACTTTCTTTTC

In100 candidate4268 (in exon):

LOCUS ENSG00000117153

DESCRIPTION Kelch-like protein 12 (CUL3-interacting protein 1).

[Source:Unirot/SWISSPROT;Acc:Q53G59]

TGGGAATGTGATAACCTAAACAACCTTTGCTCTGAAATTCCATTTTTCCCTCTTTCCCTGAGTTGTATTGACCTACAGAG

TTAATTTCCTTTGTATTTTT

In100 candidate4269 (in intron):

LOCUS ENSG00000163444

DESCRIPTION Transmembrane protein 183A. [Source:Uniprot/SWISSPROT;Acc:Q8IXX]

AGGCACTTAACCATAATTCATATTTTACTTCCTTATTCTACTTTACCTCCATTAGGTTTCTCCATTCCATCAGACTGGTC

TTTTTTTTTTTTTTTCTTTT

In100 candidate4270 (in intron):

LOCUS ENSG00000117222

DESCRIPTION Retinoblastoma-binding protein 5 (RBBP-5) (Retinoblastoma-bindig protein RBQ-3). [Source:Uniprot/SWISSPROT;Acc:Q15291]

AACTCTATTGAACTTTCTGTTGCATTTTTCTATTATTTATTTGAGATAACATTTTTCTTTTGCTTTTCTTACTTTCAGCT
CAGTTTCTGTTTTACATCTT

In100 candidate4271 (in intron):

LOCUS ENSG00000186007

DESCRIPTION LEM domain containing 1 [Source:RefSeq_peptide;Acc:NP_001001552

CCATGGCAACCACTAACCTGAATTTTCTACTTATCCTTCTTTCCCTTTTCTTTATAGCATCCACAAACTTTAGTTCAGTT
TTTCATGATTTTGTATTTCA

In100 candidate4272 (in intron):

LOCUS ENSG00000203710

DESCRIPTION Complement receptor type 1 precursor (C3b/C4b receptor) (CD35 atigen). [Source:Uniprot/SWISSPROT;Acc:P17927]

AGATAATGTGAAGCCTTAACAATTTGCTTTCTTTCTCTCTTTCTTTTGTTTCTTTTTTCTCTTCCTTCCTTTCTTCAGTT
CTTTCTTCCCTTCTCTTTCT

In100 candidate4273 (in intron):

LOCUS ENSG00000203710

DESCRIPTION Complement receptor type 1 precursor (C3b/C4b receptor) (CD35 atigen). [Source:Uniprot/SWISSPROT;Acc:P17927]

AGATAATGTGAAGCCTTAACAATTTGCTTTCTTTCTCTCTTTCTTTTGTTTCTTTTTTCTCTTCCTTCCTTTCTTCAGTT

CTTTCTTCCCTTCTCTTTCT

In100 candidate4274 (in intron):

LOCUS ENSG00000203710

DESCRIPTION Complement receptor type 1 precursor (C3b/C4b receptor) (CD35 atigen). [Source:Uniprot/SWISSPROT;Acc:P17927]

CTGCTGCATGGGCTAAGTAACCAAAAAGACTCTCTTTGTCTTGACTTAAAGTTTGTCCACTTGTCATCTTGCCTCCAGGC

TGTCTTAATCTTTGTACTTG

In100 candidate4275 (in intron):

LOCUS ENSG00000076356

DESCRIPTION Plexin-A2 precursor (Semaphorin receptor OCT). [Source:Uniprot/WISSPROT;Acc:O75051]

AATCTCCCTCTTTCACTACCATGGAGTCCCCCACAACTAACACGTTGCCCATTTTTCATCCTTAAGTCATCTGCCAGTTG

CATCTATTTCTAGCCTCTGT

In100 candidate4276 (in intron):

LOCUS ENSG00000076356

DESCRIPTION Plexin-A2 precursor (Semaphorin receptor OCT). [Source:Uniprot/WISSPROT;Acc:O75051]

TCACCCCCTCTCTGATTAATCCCACCCCACTCTAATCACCACTTAACTCCACATGCCCTTGTCTGGCTCCAGCTTAATTT

TCACATCTCAGTATCTGCCT

In100 candidate4277 (in intron):

LOCUS ENSG00000143469

DESCRIPTION Synaptotagmin-14 (Synaptotagmin XIV) (SytXIV).

[Source:Uniprot/WISSPROT;Acc:Q8NB59]

CTATCTACTTTCATTTTTCTTTTACTTAGATTTATACTTTAACTTCTTGAGCTGGATGCTTACTTCATTGTTTTTCAGTC

TTTTTTGGTTTCTAACATGT

In100 candidate4278 (in intron):

LOCUS ENSG00000054392

DESCRIPTION Protein-cysteine N-palmitoyltransferase HHAT (EC 2.3.1.-)

(Hedghog acyltransferase) (Skinny hedgehog protein 1) (Melanoma antigen

recognizd by T-cells 2) (MART-2). [Source:Uniprot/SWISSPROT;Acc:Q5VTY9]

TTCATAATTTTACTCCCCGATATTAACATTTTATTGTATTTCCTTCTCATTTTTTGTTCCATGCATATTTTTGCACAGGT

TATTTCTTGTTTCATGCTCT

In100 candidate4279 (in intron):

LOCUS ENSG00000143473

DESCRIPTION Potassium voltage-gated channel subfamily H member 1 (Voltagegted

potassium channel subunit Kv10.1) (Ether-a-go-go potassium channel 1)

(EAG1) (h-eag). [Source:Uniprot/SWISSPROT;Acc:O95259]

AACTAGTCATATTTATGTGTGTAGAGTTGTTTGTAACATTACCTTATTTTTATTTTTTTTTATGTCTCCAGGGTTTATAG

TGATATTCTCTTTTCCATTC

In100 candidate4280 (in intron):

LOCUS ENSG00000143473

DESCRIPTION Potassium voltage-gated channel subfamily H member 1 (Voltagegted potassium channel subunit Kv10.1) (Ether-a-go-go potassium channel 1) (EAG1) (h-eag). [Source:Uniprot/SWISSPROT;Acc:O95259]

CTCCCACTCTGTGGGTTGTCTATTAACTCTGCTGCTTATTTCTTTTGCTGTGCAGAAGCTTTTTAGTTTAATTAAGTCCC

ACCTATTTATCTTTGTTTTT

In100 candidate4281 (in intron):

LOCUS ENSG00000117625

DESCRIPTION REST corepressor 3. [Source:Uniprot/SWISSPROT;Acc:Q9P2K3]

CAAGCTTCTAACATTCTCAAAGTTTACATTGTTCCTTTTTGTCTTTGAAGTCATTTCCATGCATTTTCTCCACAGAATTT

TATCATATATAATTTTTTTT

In100 candidate4282 (in intron):

LOCUS ENSG00000143493

DESCRIPTION Integrator complex subunit 7 (Int7). [Source:Uniprot/SWISSPROT;cc:Q9NVH2]

ATCATTATCATTATGATTAACTGCTATTTTGGCTAGTCTTTATTTTCATTCAGACCTCGGTATTTCACCTCTCTACATTT

CTGTACCTGTTTTACTTCTT

In100 candidate4283 (in intron):

LOCUS ENSG00000143493

DESCRIPTION Integrator complex subunit 7 (Int7).

[Source:Uniprot/SWISSPROT;cc:Q9NVH2]

TGATAACCCTTCTCCCGTTAATCCCTCATGTTCCTTATCTTGTTTTCTATTTTCTATCTCTTTGTGCTAAGTTCTCAGTA

ATTTCTTCTTATATATGTTT

In100 candidate4284 (in intron):

LOCUS ENSG00000065600

DESCRIPTION Transmembrane protein C1orf75.

[Source:Uniprot/SWISSPROT;Acc:Q9813]

ATAATTTCTCTCATTCTGTAGGTTGTCTCTTAACTTTGTTGATTGTTTCCTTTGCTGTACAGAAGCTTTTTAGCTTAATG

TAATCCCATTTTTCTGTTTT

In100 candidate4285 (in intron):

LOCUS ENSG00000185523

DESCRIPTION HSD-44 (Hypothetical LOC149643).

[Source:Uniprot/SPTREMBL;Acc:Q37H7]

ATTTTGAGAACTTCATGGTGAACTTTTAACTAAGACATCTATGTTTTATTTGCAGTTCACCTATAAAATGGGCTTTTATT

TTATTTTTATTTTATTTTTT

In100 candidate4286 (in intron):

LOCUS ENSG00000136643

DESCRIPTION Ribosomal protein S6 kinase delta-1 (EC 2.7.11.1) (52 kDa ribosmal protein S6 kinase) (Ribosomal S6 kinase-like protein with two PSK domais 118 kDa protein) (SPHK1-binding protein). [Source:Uniprot/SWISSPROT;Acc:Q6S38]

TTGATTCCCTTTTAGTAACTTCTCATCCATCTACCTTTTTTTTTTTAAATTATATTCCATGGCAGCTTTTATATTCTAAA
ATTTTGTTGCTGTTACCTTC

In100 candidate4287 (in intron):

LOCUS ENSG00000117724

DESCRIPTION centromere protein F (350/400kD) [Source:RefSeq_peptide;Acc:NP_57427]

CAATTCATTGTTGAGAGAGAATGTTGGAGTCCCCACCTATAACTGTGATTTGTCTGTTTCTCCTTTGATTTCTTCCAGTT
TTTGCTTCACATATTTTGCA

In100 candidate4288 (in intron):

LOCUS ENSG00000136636

DESCRIPTION BTB/POZ domain-containing protein KCTD3 (Renal carcinoma antige NY- REN-45). [Source:Uniprot/SWISSPROT;Acc:Q9Y597]

TAGATCACTTTATATTAACCTGGGGTTGTGGAGCCCTGATTTTTTATTTCATTTTTTTCTCCATTCTTCAGTTTAGATA
TCTTTTATTGATCTACCTTT

In100 candidate4289 (in intron):

LOCUS ENSG00000136636

DESCRIPTION BTB/POZ domain-containing protein KCTD3 (Renal carcinoma antige NY- REN-45). [Source:Uniprot/SWISSPROT;Acc:Q9Y597]

GTATAACATGTTTGCTTTCTTTCTTTCTGTCTTTTTAGTTTCATAGCTTCTTTTTAAATAATTGCTCATTCCCATCAGGG

CTCTTTCTGCTTTTCTTTTG

In100 candidate4290 (in intron):

LOCUS ENSG00000042781

DESCRIPTION Usherin precursor (Usher syndrome type-2A protein) (Usher syndrme type IIa protein). [Source:Uniprot/SWISSPROT;Acc:O75445]

AATTTCGGGAACCTGACTACACTTAACCATTAATACTTATCTCTAGTTTTCCCTAGTCTTCACCTTATATTTAATCAGGT

TGATCGTCTTACTAATTTCC

In100 candidate4291 (in intron):

LOCUS ENSG00000042781

DESCRIPTION Usherin precursor (Usher syndrome type-2A protein) (Usher syndrme type IIa protein). [Source:Uniprot/SWISSPROT;Acc:O75445]

ATCAATTTCACTGTTTTCTCTAATTACATATTAACCCTGTGTTGGATTCCTGGATTTTCCAGGACAATTATATTATTCCC

TCTTTTCCCCATATCTAAGT

In100 candidate4292 (in intron):

LOCUS ENSG00000042781

DESCRIPTION Usherin precursor (Usher syndrome type-2A protein) (Usher syndrme type IIa protein). [Source:Uniprot/SWISSPROT;Acc:O75445]

GTTGAATTAACTGTCACAAATATCACTTAATTTCATTCTCAGCTTATTTGCCATGTATTCATCTTAGTCTAAGCCCTTCT

TTCAATAACTGTCTGCTCTT

In100 candidate4293 (in intron):

LOCUS ENSG00000196482

DESCRIPTION Estrogen-related receptor gamma (Estrogen receptor-related protin 3) (ERR gamma-2). [Source:Uniprot/SWISSPROT;Acc:P62508]

ATAGAAAATTAAAAAGAATTAATTCTAATGTAACCATCTTTTTACTTGACTTTTTTTTTTTTTTGCTCTGCTCACAGGT
CTAATTGTTGTTATTCTCAC

In100 candidate4294 (in intron):

LOCUS ENSG00000196482

DESCRIPTION Estrogen-related receptor gamma (Estrogen receptor-related protin 3) (ERR gamma-2). [Source:Uniprot/SWISSPROT;Acc:P62508]

GAAGAAAGAGCGTTTCATTGCTTTATAAGTAACCTCAAACCCGCGCCTTTCCTTAGCCATTCCTTTCTTTTTGGTCAGCT
GCGTTTTTATTTTTTGGACC

In100 candidate4295 (in intron):

LOCUS ENSG00000143353

DESCRIPTION Lysophospholipase-like protein 1 (EC 3.1.2.-). [Source:Uniprot/WISSPROT;Acc:Q5VWZ2]

ATAAGTTATTAAACATTTATGAAAATAAATTTAACATTCACTTAAATATCTATTGCTGTTTCTTTTGGGTTCTTCCAGAA
TAATTTTTTCTTATTCTTTA

In100 candidate4296 (in intron):

LOCUS ENSG00000154309

DESCRIPTION dispatched A [Source:RefSeq_peptide;Acc:NP_116279]

ATGACTGTATAATTTTTATAACTTACATTTCCTTTCATTTCTTTATTCATTTTCTAATTTCTGACTCATTTTTCTCAGAG
GTTTTCTTTTTGTTTCCCCT

In100 candidate4297 (in intron):

LOCUS ENSG00000143786

DESCRIPTION Cornichon homolog 3. [Source:Uniprot/SWISSPROT;Acc:Q8TBE1]

GTTCATCACCTTGTCAATACTTTATACTTGGTGTCACCTCTGTACCTGTAACATGCCATTATTTATTTATTTGCAGTTC

TACTTTTTACCAGTCCATAT

In100 candidate4298 (in intron):

LOCUS ENSG00000154380

DESCRIPTION Protein enabled homolog. [Source:Uniprot/SWISSPROT;Acc:Q8N8S7]

EXON

AGTAACTGTTGTTACTAGCTGTTTCTAGCTCTGTCTCAGTCAGTACATTTGTGATTTTTCTTTTTGTGATTTTTTTTGTG

ATTTTTTCATAATTATATCT

In100 candidate4299 (in intron):

LOCUS ENSG00000183814

DESCRIPTION Lin-9 homolog (huLin-9) (hLin-9) (Beta-subunit associated

regultor of apoptosis) (Type I interferon receptor beta chain-associated protein

(TUDOR gene similar protein) (pRB-associated protein).

[Source:Uniprot/SWISPROT;Acc:Q5TKA1]

GTTAACTTCTAATTGTCTTTTAGATCCCATTTTATGCTTCTTCAGAGAGCCATTTCCCATCCTCCCTGACTAAATTATGT

CTTCTTTAAATCTGTTTTTT

In100 candidate4300 (in intron):

LOCUS ENSG00000183814

DESCRIPTION Lin-9 homolog (huLin-9) (hLin-9) (Beta-subunit associated regultor of apoptosis) (Type I interferon receptor beta chain-associated protein (TUDOR gene similar protein) (pRB-associated protein). [Source:Uniprot/SWISPROT;Acc:Q5TKA1]

AGCCTCTTCATGCCACCATAACTAGTAGTCCTTTTCTATGCTTATATTTGTTTACTCAGCTTCATTATTTACATCTTCTG

TATCCATGTTTACTTTTCCC

In100 candidate4301 (in intron):

LOCUS ENSG00000143772

DESCRIPTION Inositol-trisphosphate 3-kinase B (EC 2.7.1.127) (Inositol 1,4,-trisphosphate 3-kinase B) (IP3K B) (IP3 3-kinase B) (IP3K-B). [Source:Unirot/SWISSPROT;Acc:P27987]

GGGAGAGTGGCCTGAATGTGGGGAGCCTTTGAGTAACTGACCTATAACTTTTCTTAGGCCCTTCTGGTTTTATCCCAGTT

ACTGATGCTGTTCTTATCTC

In100 candidate4302 (in intron):

LOCUS ENSG00000143776

DESCRIPTION Serine/threonine-protein kinase MRCK alpha (EC 2.7.11.1) (CDC42 binding protein kinase alpha) (Myotonic dystrophy kinase-related CDC42-bining kinase alpha) (Myotonic dystrophy protein kinase-like alpha) (MRCK alph) (DMPKlike

alpha). [Source:Uniprot/SWISSPROT;Acc:Q5VT25]

AACACATGATAATCGTTCTTTTTGGCATTAACACAAGTGACTTTTTGCCCTCTCATTGCATTTCAGTTTTTAATGTACCT

TTCATTGCTTTAATGTTTTC

In100 candidate4303 (in intron):

LOCUS ENSG00000181450

DESCRIPTION Zinc finger protein 678. [Source:Uniprot/SWISSPROT;Acc:Q5SXM1]

EXON

CTTTTCTTTTTTTGTTTTGATGAGCTCTCACTTCCACTTAACAATTTCTCCCTGTCTCTTTCTAGTTCTTTCTTCCAGTT

CTCATTCATTTTTTTCTTCA

In100 candidate4304 (in intron):

LOCUS ENSG00000135776

DESCRIPTION ATP-binding cassette sub-family B member 10, mitochondrial

precrsor (ATP-binding cassette transporter 10) (ABC transporter 10 protein)

(Miochondrial ATP-binding cassette 2) (M-ABC2).

[Source:Uniprot/SWISSPROT;Acc:9NRK6]

CTCCCCTTTCGTAGGTAATGAGAATGTCTTGCCACCCAATGTAACAAACACCTCTGAATTTTTTTTGTTTTTGACAGTT

TTTTGTCTCACCTAATCTGG

In100 candidate4305 (in intron):

LOCUS ENSG00000143641

DESCRIPTION Polypeptide N-acetylgalactosaminyltransferase 2 (EC 2.4.1.41) (rotein-UDP acetylgalactosaminyltransferase 2) (UDP- GalNAc:polypeptide Nactylgalactosaminyltransferase

2) (Polypeptide GalNAc transferase 2) (GalNAc-2)

(pp-GaNTase 2) [Contains: Polypeptid [Source:Uniprot/SWISSPROT;Acc:Q1047]

TACATTCTTGCTCTAAAACTCTTTGATTCTGTTAACTGTGTCTTACCATTTCCCTTTAATCTCTATTCTCAGATTGGAAT

CACCAATTTTATTTTCTTTT

In100 candidate4306 (in intron):

LOCUS ENSG00000143641

DESCRIPTION Polypeptide N-acetylgalactosaminyltransferase 2 (EC 2.4.1.41) (rotein-UDP acetylgalactosaminyltransferase 2) (UDP- GalNAc:polypeptide Nactylgalactosaminyltransferase

2) (Polypeptide GalNAc transferase 2) (GalNAc-2)

(pp-GaNTase 2) [Contains: Polypeptid [Source:Uniprot/SWISSPROT;Acc:Q1047]

GTTTCTTCTCTATTTGTACCATGTGCATTTTGCTTTTTGTAACTGTGTTCCTGCTTTCTTGCCTTCTTTTGGATTCAGTG

ATTTTTTTAGTATTCCATTT

In100 candidate4307 (in intron):

LOCUS ENSG00000135775

DESCRIPTION Conserved oligomeric Golgi complex component 2 (Low density lipprotein receptor defect C-complementing protein).

[Source:Uniprot/SWISSPROTAcc:Q14746]

AGAAGAAAGCATTTTAATAACATTTTAAAGTCCTATACGTTTTTGTCTCCATATCAGGTATTCCACACTTTATATCTTGT

TCCCTCTTTATTGTTACTGG

In100 candidate4308 (in intron):

LOCUS ENSG00000116906

DESCRIPTION Dihydroxyacetone phosphate acyltransferase (EC 2.3.1.42) (DHAP-T) (DAP-AT) (Glycerone-phosphate O-acyltransferase) (Acyl-CoA:dihydroxyaceonephosphateacyltransferase). [Source:Uniprot/SWISSPROT;Acc:O15228]

TTTTTTTCTTCTAGGAAAGCTCTTAACTTCACTTGCCTTCCTCCTCATCCCTTTTAATATTTTGTTTTAATTCAGTCATT
TACTTTCCTGTCAAATATTT

In100 candidate4309 (in intron):

LOCUS ENSG00000010072

DESCRIPTION DDDL1880 (Chromosome 1 open reading frame 124). [Source:UniprotSPTREMBL;Acc:Q6UWW6]

TCATGATCTCTTAACTTTGTTTCCTTATTATTGTCTTTTTTAGTCCATCCTGTATCCTGCTACCATTGCAGATGTCATTC
CCTCATCAGAATTCTTCTTT

In100 candidate4310 (in intron):

LOCUS ENSG00000162946

DESCRIPTION Disrupted in schizophrenia 1 protein. [Source:Uniprot/SWISSPROTAcc:Q9NRI5]

AAAAATGGCTGTATGATATGTAACAATAAGTGTGGCTGATTCCAATTTTCTCCATTTGGTTGTTTCCAGTTATTTCTCAT
GCTGCAATGATCATCCTTTT

In100 candidate4311 (in intron):

LOCUS ENSG00000116991

DESCRIPTION Signal-induced proliferation-associated 1-like protein 2.

[Soure:Uniprot/SWISSPROT;Acc:Q9P2F8]

TACCTGGTCCAACTAACTGGTCAACTGCCCTCTCTTTTTTTGATAAGAATATGTATTCTTCCCTTTGAACCTTCTCAGTT

CTCTAGGTTTTTTGGTTTTG

In100 candidate4312 (in intron):

LOCUS ENSG00000135749

DESCRIPTION pecanex-like 2 [Source:RefSeq_peptide;Acc:NP_079214]

AAAATCACAAATCTGACTTTGTTTGTTTTAAATAACTTTTTATTTGTAACTCATTATTCCTCATCTTATTTTTGCCAGTT

TCCATTATCTAGTTTGCTTT

In100 candidate4313 (in intron):

LOCUS ENSG00000183780

DESCRIPTION solute carrier family 35, member F3

[Source:RefSeq_peptide;Acc:P_775779]

ATCCCAATAACTTTTCCTATACTTTCTTTTCATTTTGCTTGATTTTTCATTTCTAACCTCTGTCATTTATGATGTCAGCA

GTGGTTATTCTTCCTTCTGT

In100 candidate4314 (in intron):

LOCUS ENSG00000183780

DESCRIPTION solute carrier family 35, member F3

[Source:RefSeq_peptide;Acc:P_775779]

CTGTTGCTTACTTCTAAAATTATATGGACTTCTCTGTCTCTTATAACTTTTTCTCCCATTTTCTCTGTTCTTGTGCAGTT

TTGTCCTCGTTATCCTTTAC

In100 candidate4315 (in intron):

LOCUS ENSG00000183780

DESCRIPTION solute carrier family 35, member F3

[Source:RefSeq_peptide;Acc:P_775779]

TTTTCTTTTCTTTTTTGTCATGATTTTTTTAATGATACCTTAACTAGTTGTAGGATTCTCTGTTGACAGGTTTTTTTAAA

TATGTTATCACACTGCCCTT

In100 candidate4316 (in intron):

LOCUS ENSG00000059588

DESCRIPTION Probable methyltransferase TARBP1 (EC 2.1.1.-) (TAR

RNAbindingprotein

1) (TAR RNA-binding protein of 185 kDa) (TRP-185).

[Source:Uniprot/WISSPROT;Acc:Q13395]

TAATCACTCTTTGGTATGAATATATCTTTTTAACTGGTTTCCATTCTTATTCTCTTAGTATATCTTTTCCAGTGTTTCTT

AAGTTTTCAGTTATTGTTCT

In100 candidate4317 (in intron):

LOCUS ENSG00000054267

DESCRIPTION AT rich interactive domain 4B isoform 2

[Source:RefSeq_peptide;cc:NP_112739]

CCTGTTAGTTCAACAATGAGTATCATATTTGCATACTTTTAACATTTTAAGTATTCTCTTCATATTTTCTTTTTTCAGTG

AATTTTATTTTTTATCTTTG

In100 candidate4318 (in intron):

LOCUS ENSG00000116962

DESCRIPTION Nidogen-1 precursor (Entactin).

[Source:Uniprot/SWISSPROT;Acc:P4543]

ACTTATTAATTTTAGGGGATGTTATTAACTTCTAAACTGTTTTTGATTTTCTTTTAAATCCCATCACCCCAGGGGTGGTG

TGTCTTTGCTTCTTTGTTTC

In100 candidate4319 (in intron):

LOCUS ENSG00000077585

DESCRIPTION Integral membrane protein GPR137B (Transmembrane 7 superfamily

ember 1). [Source:Uniprot/SWISSPROT;Acc:O60478]

CTTTTAAAATTCTTTCCTTTGTCTTGACTTTAGATAACCTGACTGTGCCTGATCATCTTTTTGCAATTATTTTCCCAGGT

GTTCTTTGAGCTTCTTGTAT

In100 candidate4320 (in intron):

LOCUS ENSG00000086619

DESCRIPTION ERO1-like protein beta precursor (EC 1.8.4.-) (ERO1-Lbeta)

(Oxioreductin-1-Lbeta) (Endoplasmic oxidoreductin-1-like protein B).

[Source:Unprot/SWISSPROT;Acc:Q86YB8]

TGCTACATTTTAGACTTTGATTTTTGTTATCTAACATAATTAATTATATTCCTCTTCTTCAACAGTAATTAGTTGTAAGC

TTTGTTTTCCTTTCACTTTC

In100 candidate4321 (in intron):

LOCUS ENSG00000186197

DESCRIPTION Ectodysplasin A receptor-associated adapter protein (EDARassocated

death domain protein) (Crinkled homolog).

[Source:Uniprot/SWISSPROT;Ac:Q8WWZ3]

CCTTGCTTTTAACTGTCCTGTGTGTGTCCTCAATGATGCTTTTTTTGTGGCTCTGCCTTTCCCAGCGATCTACTGCATTT

TCCTCTCCTTCCTTTCTTTT

In100 candidate4322 (in intron):

LOCUS ENSG00000198626

DESCRIPTION Ryanodine receptor 2 (Cardiac muscle-type ryanodine receptor)

(yR2) (RYR-2) (Cardiac muscle ryanodine receptor-calcium release channel) (hYR-

2). [Source:Uniprot/SWISSPROT;Acc:Q92736]

AACCCCTATTAACTATGAAAAGATTTTCTGATTCCCAGTCTCATTTTTTCTATTTTCTTTCTTCTTGCCTTGCTCTCTTT

TTTCTAACTTTATAAGATGA

In100 candidate4323 (in intron):

LOCUS ENSG00000198626

DESCRIPTION Ryanodine receptor 2 (Cardiac muscle-type ryanodine receptor)

(yR2) (RYR-2) (Cardiac muscle ryanodine receptor-calcium release channel) (hYR-

2). [Source:Uniprot/SWISSPROT;Acc:Q92736]

AAAGCAAAAAAAAAGAATATTAACTTTGAATTCACATTATCATTTCTTTTTGGTTACTCCTATACTGTCATTGCCCAGAT

TTCATCAGTTTCCCTTTGAA

In100 candidate4324 (in intron):

LOCUS ENSG00000155816

DESCRIPTION Formin-2. [Source:Uniprot/SWISSPROT;Acc:Q9NZ56]

TCTTCTAAATAAGATGAATGTTTTAGAACATTCTAACTAACAATTGAACTCCTTCTACTTTCTTTTCAGTTTTTTTCCAG

ACTTTGGGTACTGTCATTTT

In100 candidate4325 (in intron):

LOCUS ENSG00000180875

DESCRIPTION Gremlin-2 precursor (Cysteine knot superfamily 1, BMP antagonis

2) (Protein related to DAN and cerberus) (DAN domain family member 3).

[Sorce:Uniprot/SWISSPROT;Acc:Q9H772]

AGTAGCCTCCTAACTAGTCTCCTGCATTTTAGCTTTTCCTCATTTCAGTTCATCTTCCATAGTATTGCATTTTTTTTTTT

TTTTTTTTTTGAGATGGAG

In100 candidate4326 (in intron):

LOCUS ENSG00000182901

DESCRIPTION Regulator of G-protein signaling 7 (RGS7).

[Source:Uniprot/SWISPROT;Acc:P49802]

TTTGAACAAGTCCATTATAACTTTCTTTGATATTACCTTTTTAATTTTATTCCTAGCAGTTATTACAGCCTGAAATTTTC

TTTTGTTTGTTCATTTGTTT

In100 candidate4327 (in intron):

LOCUS ENSG00000182901

DESCRIPTION Regulator of G-protein signaling 7 (RGS7).

[Source:Uniprot/SWISPROT;Acc:P49802]

GCTTATTTGGTCTGTCAATTAATGAAAAAGTGGTGTTGAAGTTTGTAACTGTAATAATGAGTTTATTTTTCCTTTCAGTT

CTCTCTAGTTTTGCCTTATG

In100 candidate4328 (in intron):

LOCUS ENSG00000117009

DESCRIPTION Kynurenine 3-monooxygenase (EC 1.14.13.9) (Kynurenine 3-hydroxyase). [Source:Uniprot/SWISSPROT;Acc:O15229]

TCCAAAGGATATATCTAACCTTTTTTAGGTTTTACTATTATCTTTTTGGTTACAGAGGCCACTTTATAGCGCTTGACTTG
TTTATTCTTATTTATTCTTC

In100 candidate4329 (in intron):

LOCUS ENSG00000174371

DESCRIPTION Exonuclease 1 (EC 3.1.-.-) (hExo1) (Exonuclease I) (hExoI). [Sorce:Uniprot/SWISSPROT;Acc:Q9UQ84]

AGACCCTGTCTCCAAAAAATAAAATGATTACTTTTGTAACTTGTAGGATTCTTTTTTCCTCTGGCTGTGTAGCAGTATTT
CTTTTCTCCAAATTTTATAA

In100 candidate4330 (in intron):

LOCUS ENSG00000054282

DESCRIPTION Serologically defined colon cancer antigen 8 (Centrosomal coloncancer autoantigen protein) (hCCCAP) (Antigen NY-CO-8). [Source:Uniprot/SWISPROT;Acc:Q86SQ7]

TATGCTGTGATATGTTTTCCTGCATTTTGGGTAACTGTTTAAAGATAATTTTTCTTTGGTAATTCTTTTATCTACCAGCT
TTTATACTTTTTAGATTTTT

In100 candidate4331 (in intron):

LOCUS ENSG00000054282

DESCRIPTION Serologically defined colon cancer antigen 8 (Centrosomal coloncancer autoantigen protein) (hCCCAP) (Antigen NY-CO-8). [Source:Uniprot/SWISPROT;Acc:Q86SQ7]

GGTCAAAAAGCCAAACTCATTATCTGTTTATTTGAAGAGGATATTATAACATTGATGTATATTCTTGCTATTTTACAGTG

AACTTTATCTTTTTTTTCCT

In100 candidate4332 (in intron):

LOCUS ENSG00000117020

DESCRIPTION RAC-gamma serine/threonine-protein kinase (EC 2.7.11.1) (RACPKgamma) (Protein kinase Akt-3) (Protein kinase B, gamma) (PKB gamma) (STK-2) [Source:Uniprot/SWISSPROT;Acc:Q9Y243]

TCTGGTTCTTCTTAGTGGAAAATAGTATTAACTTTCCTTTATTTTTAATTCTTTTATTTACCTTTGTTCTTCATGCAGTA

TTTTTCTTTTCCTTATCCTG

In100 candidate4333 (in intron):

LOCUS ENSG00000117020

DESCRIPTION RAC-gamma serine/threonine-protein kinase (EC 2.7.11.1) (RACPKgamma) (Protein kinase Akt-3) (Protein kinase B, gamma) (PKB gamma) (STK-2) [Source:Uniprot/SWISSPROT;Acc:Q9Y243]

ACCCTTGTTGAATTCTGGTGTAGTATCAAAGAATGATATCCATAACTATCTGAATATTTTTCCCTTTTTCAGCTTCATAT

CTCTTTGAGCCAGATTTTTT

In100 candidate4334 (in intron):

LOCUS ENSG00000117020

DESCRIPTION RAC-gamma serine/threonine-protein kinase (EC 2.7.11.1) (RACPKgamma) (Protein kinase Akt-3) (Protein kinase B, gamma) (PKB gamma) (STK-2) [Source:Uniprot/SWISSPROT;Acc:Q9Y243]

AAAATTCATGCTCTTGTCATGTTAGTAAAGATCCTAACCAATCTGATCTCATCTTTTTTTTCCAGAAGTCCTATTATTTC
TCTGATTATATATTATTTTT

In100 candidate4335 (in intron):

LOCUS ENSG00000035687

DESCRIPTION Adenylosuccinate synthetase isozyme 2 (EC 6.3.4.4) (Adenylosuccnate synthetase, acidic isozyme) (IMP--aspartate ligase 2) (AdSS 2) (AMPSas 2). [Source:Uniprot/SWISSPROT;Acc:P30520]

TATTGATTTTGTGGGTTTTTGATTTTTTTTTCCCCAAAAAATAACCATTTTCTCTATTTTCTGTTTCACTGGGTTCAGCT
CTTTCTCTTCTTTCTACTTT

In100 candidate4336 (in intron):

LOCUS ENSG00000203666

DESCRIPTION EF-hand calcium-binding domain-containing protein 2. [Source:Unprot/SWISSPROT;Acc:Q5VUJ9]

CCACTAACCACATTTTCTCATTTTTTTTCTCCATCCTTGAATTCTTTGATTTATTTTTGTGTGTTGATTCTCTTGTCAGTT
TTTTTTTTTTTTTCAGGAAG

In100 candidate4337 (in intron):

LOCUS ENSG00000203666

DESCRIPTION EF-hand calcium-binding domain-containing protein 2.

[Source:Unprot/SWISSPROT;Acc:Q5VUJ9]

GTGACTCCACATCACCTGGTAATCTAACATGCTGGTGATTCTACTCACCTGCTTTTATTTCTTCTTTTCCAGATTCTTCC

TATTTTGCTCCCGACTGATT

In100 candidate4338 (in intron):

LOCUS ENSG00000185420

DESCRIPTION SET and MYND domain-containing protein 3 (EC 2.1.1.43) (Zinc

figer MYND domain-containing protein 1). [Source:Uniprot/SWISSPROT;Acc:Q9H7B4

AGTATATTTCTTTATCATTAGATTCTTTCATTTTCTTATTTTCTGTGTAACTACTTTTACTTTGTTTTCTCTTTTCAGTA

GTCTTTTTTTATTATTTTAT

In100 candidate4339 (in intron):

LOCUS ENSG00000185420

DESCRIPTION SET and MYND domain-containing protein 3 (EC 2.1.1.43) (Zinc

figer MYND domain-containing protein 1). [Source:Uniprot/SWISSPROT;Acc:Q9H7B4

TTCTCTGCATTAGTAACGTACTGGTTAAATCTACACATGTGGTAAACTTTTTTTTTTTTTTTCATTCTCACTTACCAGGA

AATTCTATTTTCTTTTACCC

In100 candidate4340 (in intron):

LOCUS ENSG00000206368

DESCRIPTION Baculoviral IAP repeat-containing 1 variant (Fragment).

[SourceUniprot/SPTREMBL;Acc:Q59GI6]

TTTTAAAGATGTTACACTTTGCTGTGAGAGATGTAGCTGACATAACTAAATTTTTATTTTCCCTTTCATAGTTGCCAGTT

TGCCAAATTTTATTTCTCTG

In100 candidate4341 (in intron):

LOCUS ENSG00000206374

DESCRIPTION coiled-coil domain containing 125

[Source:RefSeq_peptide;Acc:NP789786]

GATGCCTGAATTTATGTGGTGCATTGAAAAATATTCTTAGCTAACTCTACAATTGCTTACTCTTTCCTTTTACAGCTTTT

CCCCCTTATATTGACCAATT

In100 candidate4342 (in intron):

LOCUS ENSG00000206374

DESCRIPTION coiled-coil domain containing 125

[Source:RefSeq_peptide;Acc:NP789786]

ACCATGTCCAAACTGATGTTAACTTTTTACATATTTTATTTATTTATTTGTTTATTTATTTATTTATTTTCACACAGGGT

CTTGCTCTGTTGCCCAGTTT

total exon length=65661947

total intron length=1068751168

exonlocation = 65; intronlocation = 4277
